# Transcriptomic analysis reveals mitochondrial pathways associated with distinct adolescent behavioral phenotypes and stress response

**DOI:** 10.1038/s41398-023-02648-3

**Published:** 2023-11-17

**Authors:** Thamyris Santos-Silva, Doğukan Hazar Ülgen, Caio Fábio Baeta Lopes, Francisco S. Guimarães, Luciane Carla Alberici, Carmen Sandi, Felipe V. Gomes

**Affiliations:** 1 Department of Pharmacology, Ribeirão Preto Medical School, University of São Paulo, Ribeirão Preto, Brazil; 2 Brain Mind Institute, École Polytechnique Fédérale de Lausanne, Lausanne, Switzerland; 3 Ribeirão Preto Pharmaceutical Sciences School, University of São Paulo, Ribeirão Preto, Brazil

**Keywords:** Molecular neuroscience, Genomics

## Abstract

Adolescent individuals exhibit great variability in cortical dynamics and behavioral outcomes. The developing adolescent brain is highly sensitive to social experiences and environmental insults, influencing how personality traits emerge. A distinct pattern of mitochondrial gene expression in the prefrontal cortex (PFC) during adolescence underscores the essential role of mitochondria in brain maturation and the development of mental illnesses. Mitochondrial features in certain brain regions account for behavioral differences in adulthood. However, it remains unclear whether distinct adolescent behavioral phenotypes and the behavioral consequences of early adolescent stress exposure in rats are accompanied by changes in PFC mitochondria-related genes and mitochondria respiratory chain capacity. We performed a behavioral characterization during late adolescence (postnatal day, PND 47–50), including naïve animals and a group exposed to stress from PND 31–40 (10 days of footshock and 3 restraint sessions) by z-normalized data from three behavioral domains: anxiety (light–dark box tests), sociability (social interaction test) and cognition (novel-object recognition test). Employing principal component analysis, we identified three clusters: naïve with higher-behavioral z-score (HBZ), naïve with lower-behavioral z-score (LBZ), and stressed animals. Genome-wide transcriptional profiling unveiled differences in the expression of mitochondria-related genes in both naïve LBZ and stressed animals compared to naïve HBZ. Genes encoding subunits of oxidative phosphorylation complexes were significantly down-regulated in both naïve LBZ and stressed animals and positively correlated with behavioral z-score of phenotypes. Our network topology analysis of mitochondria-associated genes found *Ndufa10* and *Cox6a1* genes as central identifiers for naïve LBZ and stressed animals, respectively. Through high-resolution respirometry analysis, we found that both naïve LBZ and stressed animals exhibited a reduced prefrontal phosphorylation capacity and redox dysregulation. Our findings identify an association between mitochondrial features and distinct adolescent behavioral phenotypes while also underscoring the detrimental functional consequences of adolescent stress on the PFC.

## Introduction

Adolescence is a period of learning, social adaptation, and dynamic neurobiological maturation, which begins with puberty and ends in adulthood [1]. Evident structural and functional alterations occur in the brain during adolescence, particularly within cognitive [2], social [3], and emotional processes [4]. These changes are particularly pronounced in cortical regions: the latest brain areas to mature [5]. Throughout adolescence, functional connectivity is strengthened between cortical regions and limbic circuits [6]. This leads to better stabilization and efficiency of prefrontal cortex (PFC) activity [7], a crucial component for higher-order cognitive processes [8]. PFC maturation also reduces inter-individual variability in cognitive task performance and enhances overall brain function, refining complex behaviors [9].

Social and neurobiological factors collectively shape brain development and personality traits [10]. Social experiences influence behavioral outcomes and predict vulnerability and resilience to stress [11]. Adolescence represents a critical period for experience-dependent plasticity in social behaviors [12, 13]. Adolescent animals display significant variability in cortical dynamics [14], which affects behavioral phenotypes related to motivation [15], learning [16], and decision-making [16, 17].

Susceptibility to adverse socio-environmental factors, such as trauma, psychosocial insults, and maltreatment, is also increased in adolescence. Animal studies show the potential of stress during the peripubertal period to cause changes in the brain and behavior in adulthood [17–23]. Furthermore, the timing of stress seems critical for the behavioral outcomes [24–26].

Cortical gene signature during adolescence highlighted increased expression of genes associated with energy metabolism and oxidative phosphorylation (OXPHOS) [27], supporting the role of mitochondria in normal cortical development and behavior [28, 29]. Mitochondria are emerging as a critical regulator of social behaviors and trait anxiety [30–33]. Changes in mitochondrial function and gene expression are implicated in the modulation of behaviors in response to stress [34]. Therefore, changes in mitochondrial gene expression during adolescence may influence mitochondrial function and redox regulation, potentially leading to behavioral alterations.

Here, we explored the behavioral consequences of stress during late adolescence in rats and linked these outcomes to cortical transcriptomic changes. We first investigated individual phenotype variations within three behavioral domains: anxiety, sociability, and cognition. Then, we performed genome-wide bulk RNA sequencing in the PFC and examined the relationship between mitochondria-related genes and behavioral phenotypes in both naïve and stressed animals. Lastly, we investigated PFC mitochondria respiratory function and reactive oxygen species (ROS) production. Altogether, our findings underscore that differences in mitochondrial gene expression and respiratory function in PFC contribute to individual behavioral variability in naïve animals and highlight the detrimental functional consequences of stress on prefrontal redox regulation.

## Materials and methods

### Animals

Male and female Sprague-Dawley rats (postnatal day, PND70), an outbred laboratory rat population, were obtained from the Central Animal Facility of the University of São Paulo, Ribeirão Preto, and allowed to acclimatize for one week in the local animal facility before breeding. Mating was confirmed by spermatozoa presence in the vaginal smear, and birthday was defined as PND0. Pups were weaned on PND21. We use only the male offspring, as female adolescent rats do not show the behavioral and electrophysiological changes induced by this stress protocol applied from PND31 to 40 [35, 36]. Animals were randomly assigned to experimental groups, each cage devoted to a specific experimental procedure. Rats were housed (2–3 animals per cage) at temperature- (22 °C) and humidity-controlled (47%) room. The Ribeirão Preto Medical School Ethics Committee (CEUA-FMRP 248/2019) approved the procedures following Brazilian and international regulations.

### Adolescent stress protocol

Animals were exposed to inescapable foot shock (FS; from PND31 to 40) daily and three restraint stress (RS) sessions (PND31, 32, and 40), as previously described [24, 26, 37]. Briefly, rats were exposed to one session of FS per day for 10 consecutive days. In each session, animals were placed in a Plexiglas chamber with a grid floor of 0.48 cm stainless steel rods spaced 1.6 cm apart (EP107R, Insight Equipment, Brazil). 25 FS (1.0 mA, 2 s) was delivered pseudo-randomly (5 cycles of 30, 60, 40, 60, and 90 s). Immediately after receiving FS, rats were submitted to RS for 1 h in Plexiglas cylindrical restraint tubes (20.3 × 5.1 cm) ventilated by holes (1 cm diameter) on the first, second, and last day of FS exposure. Naïve animals were left undisturbed in their home cages (*n* = 18), while stressed animals (*n* = 14) were subjected to the stress protocol.

### Behavioral characterization

One week after stress, rats were tested in the following behavioral tests: light-dark box (LDB) on PND47 to assess anxiety-like behavior; social interaction test (SIT) on PND48 to measure sociability; and novel-object recognition test (NOR) on PND49 to evaluate cognitive function.

#### LDB

The LDB apparatus has two compartments (240 cm × 210 cm) connected by a door. The light and the dark compartments have a grid floor. Two hours after the EPM, animals were placed in the dark compartment and allowed to explore the apparatus freely for 5 min after the first entry into the light zone. The ANY-maze Software analyzed the time spent in the light zone.

#### SIT

Animals were placed in the center of a circular arena (D60 cm × H65 cm) and allowed to explore for 5 min. Then, an unfamiliar male Sprague-Dawley rat (PND50–54) was placed in the arena as a social target for 10 min. The social interaction time was measured when the tested animal was sniffing the unfamiliar rat’s anogenital region, head, or body and when they were following, crawling over and under each other. A blind experimenter quantified the social interaction time.

#### NOR

Two hours after the SIT, each animal was habituated in a circular arena (D60 cm × H60 cm) for 10 min. The NOR test was conducted in the same circular arena 24 h later. Animals were subjected to two trials separated by 1 h. During the first trial (acquisition trial, T1), rats were placed in the arena containing two identical objects for 5 min. For the second trial (retention trial, T2), one of the objects presented in T1 was replaced by an unknown (novel) object. Animals were then placed back in the arena for 5 min. Object exploration was defined as when the animal faced the object at 2 cm of distance or less while watching, licking, sniffing, or touching it with the forepaws while sniffing. A blind experimenter quantified object exploration. Recognition memory was assessed using the discrimination index:

$$\left[\left({{t}}_{{\rm{novel}}\; {\rm{object}}}{{\mbox{-}}}{{t}}_{{\rm{familiar}}\; {\rm{object}}}\right)/\left({{t}}_{{\rm{novel}}\; {\rm{object}}}+{{t}}_{{\rm{familiar}}\; {\rm{object}}}\right)\right]$$

#### Behavioral *z*-score index

The integrated behavioral *z*-score method allows the normalization of individual data observed in different behavioral parameters to decrease the intrinsic variability of single tests and enhance the sensitivity and reliability of the individual phenotyping [38]. We calculated the integrated behavioral *z*-score by averaging the *z*-normalized data [(*x*−mean of naïve group)/(standard deviation of naïve group)] for time spent in light zone (LBD), social interaction time (SIT) and novel object discrimination index (NOR).

### Gene expression profiling from the rat PFC

#### RNA isolation

Following the behavioral tests on PND 51, animals were anesthetized (urethane 25%, 1 mL/100 g/rat) and perfused with cold 0.01 M phosphate-buffered saline (PBS, pH = 7.4). PFC from both hemispheres was collected and snapped frozen in liquid nitrogen until use for RNA extraction (*n* = 8/group). To this end, we used RNAqueous-Micro Total RNA Isolation Kit (Thermo Fisher Scientific; #AM1931), according to the manufacturer’s instructions.

#### Bulk RNA-sequencing

The extracted RNA was used for performing the transcriptomic analysis from the PFC of naïve and adolescent-stressed rats using bulk RNA barcoding and sequencing, as previously described [39, 40]. Briefly, the RNA samples were reverse transcribed with individual barcoded oligo-dT primers. Then, all samples were pooled together, and the second strand synthesis generated the double-stranded cDNA via the nick translation method. Illumina-compatible libraries were prepared by tag-mentation of 5 ng of full-length double-stranded cDNA. Then, the library was amplified, profiled, and sequenced using the Illumina NextSeq 500 platform.

#### Transcriptomic analysis

Following a quality assessment with FastQC [41], gene reads were mapped with HISAT2 onto Rnor_6.0/rn6 genome assembly for *Rattus norvegicus* [42]. Mapped reads were counted for each gene locus using the featureCounts function of the subread (2.0.2) package [43]. Read counts are available at 10.6084/m9.figshare.24125793.v1. We normalized count data by size factor and applied a variance stabilizing transformation for visualization purposes [44]. Low-abundance genes were removed before data normalization, keeping only genes with at least ten reads in all samples. Subsequently, we performed a generalized linear model to assess differentially expressed genes (DEGs) using the DESeq2 package [45]. *p-*values were corrected for multiple testing using the Benjamini–Hochberg method [46]. Transcriptomic analysis was performed in R (R Core Team, 2014).

#### Functional gene set enrichment analysis

Only DEGs with a padj < 0.1 were explored for enriched gene sets and function. Gene set enrichment analyses were derived from bioinformatics resource systems (web servers) for functional annotation and enrichment analyses of gene lists. *Database for Annotation, Visualization and Integrated Discovery* (DAVID) [47] was used for gene ontology enrichments, and the *Kyoto Encyclopedia of Genes and Genomes* (KEGG) database [48] was assayed to examine gene functions, linking genomic information with higher-order functional processes and diseases.

#### Network visualization and analysis

The transcriptomic data of DEGs were mapped onto unbiased interaction networks using Cytoscape [49, 50]. The STRING app [51] was used to retrieve information about gene–gene associations and subcellular compartments where gene products are located (COMPARTMENTS database) [52]. The STRING database systematically collects and integrates physical and functional protein–protein interactions from various sources, including automated text mining of scientific literature, computational interaction predictions derived from co-expression and conserved genomic context, databases of interaction experiments, and known complexes/pathways from curated sources [51].

We constructed interactive networks, featuring nodes corresponding to DEGs and edges representing predicted functional and/or physical interactions with a confidence score cut-off of 0.5. Then, to create mitochondria-associated gene networks [52], nodes were filtered based on the likelihood of the gene’s association with the mitochondrial compartment with a confidence score ranging from 4.0 to 5.0. To better visualize up and down-regulated genes, nodes were colored according to the quantitative expression data (Log_2_FoldChange). Nodes with the highest betweenness and closeness centrality for each comparison were identified using the CytoNCA plugin.

### Biomolecular analyses

#### High-resolution respirometry

2 mg of fresh PFC samples (*n* = 5 naïve HBZ; 5 naïve LBZ; 6 stressed), finely cut into pieces, were permeabilized in BIOPS solution (2.7 mM EGTA, 20 mM imidazole, 20 mM taurine, 50 mM acid 2-(N-morpholino) ethanesulfonic potassium, 0.5 mM dithiothreitol, 6.5 mM MgCl_2_, 15 mM phosphocreatine, 0.57 mM ATP, pH 7.1) containing 0.01% saponin for 5 min at 4 °C, then carefully transferred to the chambers of a Oxygraph-2k respirometer (Oroboros Instruments, Austria), containing 2 mL of air-saturated respiration medium MIR05 (0.5 mM EGTA, 3 mM MgCl_2_, 60 mM K-lactobionate, 20 mM taurine, 10 mM KH_2_PO_4_, 20 mM HEPES, 110 mM sucrose, 1 g/L albumin, pH 7.1). First, 9 mM glutamate, 5 mM malate, and 10 mM pyruvate were added to the chambers to determine LEAK respiration. Then, OXPHOS capacity linked to complex I activity (CI) was determined after adding 1 mM ADP to the chambers, followed by adding 10 mM. Maximal capacity rates of the electron transfer system (ETS) were defined after 1 pulse of the mitochondrial uncoupler carbonyl cyanide m-chlorophenylhydrazone (CCCP, 1 μM). Finally, residual oxygen consumption (Rox) rates were determined by adding 1 mM NaCN. Rox rates were subtracted from all other measurements. The experiments were made in duplicate. The O_2_ flux obtained in each step of the protocol was normalized by the wet weight of the tissue sample used for the analysis.

#### Amplex™Red assay

Naïve and stressed animals (*n* = 5 naïve HBZ; 5 naïve LBZ; 6 stressed) were anesthetized (urethane 25%, 1 mL/100 g), and decapitated. PFC tissues were immediately collected and cut into ~1 mm^3^ cubes. Samples were carefully transferred to cuvette chambers (Hitachi 4500 Fluorescence Spectrophotometer, Japan) with 2 mL of air-saturated respiration medium containing MIR05, 50 μM Amplex™Red reagent, 0.1 U/mL horseradish peroxidase, substrates (9 mM glutamate, 5 mM malate, and 10 mM pyruvate) and 1 mM ADP to determine hydrogen peroxide production by OXPHOS linked to CI. The fluorescence of each step was measured for 100 s. Then, hydrogen peroxide levels were determined as the angular coefficient of the linear regression lines made for the spectrophotometric data. The total protein amount of each sample was measured by Bradford protein assay and used to normalize data.

### Statistical analyses

Data were presented as mean ± SEM. The Bartlett’s and Shapiro–Wilk tests were applied to check for variance and normal distribution before the parametric analysis (two-sided Student’s *t*-test or one-way ANOVA followed by *posthoc* Turkey’s test). Significant differences were indicated by *p* < 0.05. In RNA-seq analysis, *p*-values were corrected using the Benjamini–Hochberg method for multiple testing. A Pearson correlation analysis tested the correlation between integrated behavioral *z*-score and normalized read counts. Sample size was based on previous works of the research group.

#### Behavioral data analysis

Each animal’s behavioral *z*-scores (LDB, SIT, and NOR) were calculated, and their dimensionality was reduced by principal component analysis (PCA) (code available: https://uc-r.github.io/pca). We performed an unsupervised clustering method (*k*-means) that separated the animals into three clusters in two PC dimensions. We performed the Elbow, Silhouette and Gap statistic methods to confirm the optimal number of clusters (code available: https://uc-r.github.io/kmeans_clustering). Behavioral data analysis was performed in R (R Core Team, 2014).

## Results

### Adolescent behavioral phenotypes are affected by stress exposure

**Fig. 1:**
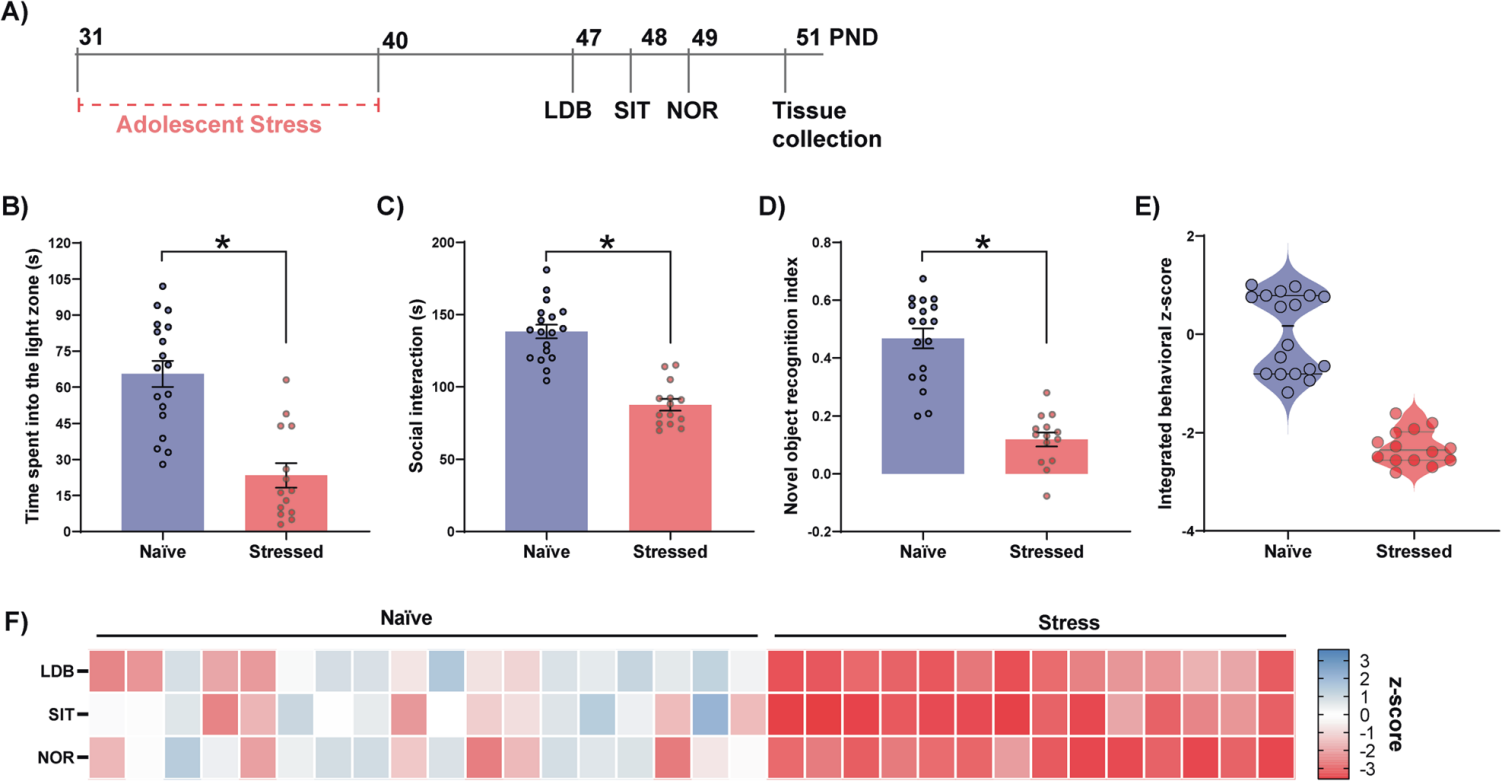
Effects of stress on adolescent behavior. **A** Experimental timeline: adolescent rats were stressed via inescapable foot shocks from PND 31 to PND 40. From PND 47 to PND 49, animals were tested in the LDB, SIT, and NOR (*n* = 18 naïve and 14 stressed). Brain tissue was collected on PND 51. Adolescent stress decreased **B** time spent in the light zone of the LDB (*t*_30_ = 5.53, *p* < 0.0001), **C** social interaction time (*t*_30_ = 7.86, *p* < 0.0001), and **D** the discrimination index in the NOR test (*t*_30_ = 7.83, *p* < 0.0001). **E** Integrated *z*-score computed from LDB, SIT, and NOR *z*-scores and **F** a heatmap of each behavioral *z*-score indicates the impact of adolescent stress on behavior. Data are shown as mean ± SEM. *p < 0.05; unpaired *t*-test.

First, we aimed to characterize the adolescent behavioral phenotypes and the impact of stress. Adolescent rats were exposed to the stress protocol (PND31–40) and subsequent anxiety, sociability, and cognitive function behavioral assessments (PND47–49), followed by PFC collection (PND51) (Fig. 1A). Stressed animals exhibited reduced time in the light zone of the LDB, indicating an anxiogenic response (Fig. 1B), and social interaction deficits (Fig. 1C). Moreover, stressed animals showed a decreased novel object recognition index in NOR test, suggesting cognitive impairments (Fig. 1D).

Next, we applied a *z*-normalization for all behavioral parameters to combine data from all tests into a single index. Notably, the integrated behavioral *z*-scores (Fig. 1E) and the heatmap displaying *z*-normalized behavioral data (Fig. 1F) indicate a high intra-group variability among naïve animals. Conversely, stressed animals are narrowly distributed, indicating a homogenous detrimental impact of stress.

### Revealing individual behavioral variability

**Fig. 2:**
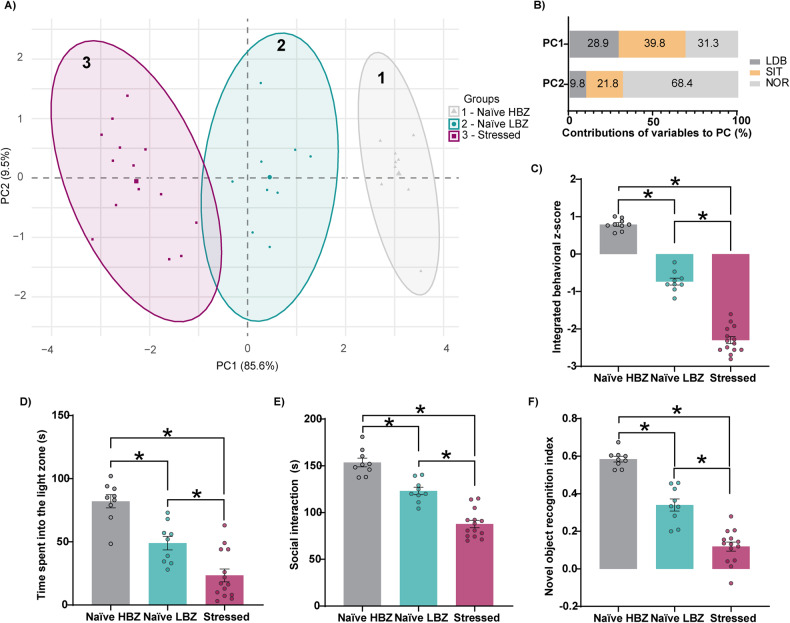
Animals cluster into three groups based on behavioral phenotypes. **A** Expression of significant PCs after behavioral analyses. Dots represent integrated *z*-scores from the four behavioral paradigms for each rat. Three groups of individuals (gray, blue, and purple dots) were separated by unsupervised *k*-meaning clustering (*n* = 9 naïve HBZ, *n* = 9 naïve LBZ, *n* = 14 stressed). **B** Representative contributions of each behavioral paradigm to the two PCA components. **C** Intergroup comparisons of the integrated behavioral *z*-score: naïve HBZ, naïve LBZ, and stressed animals (*F*_2,29_ = 317.6, *p* < 0.0001). **D** Naïve LBZ and stressed animals spent less time in the light zone of the LDB test (*F*_2,29_ = 31.32, *p* < 0.0001), and **E** presented reduced sociability (*F*_2,29_ = 62.71, *p* < 0.0001). Also, **F** both groups displayed a decreased discrimination index in the NOR test (*F*_2,29_ = 87.83, *p* < 0.0001). Data are shown as mean ± SEM. *p < 0.05; one-way ANOVA, followed by Tukey’s multiple comparison test.

To gain deeper insight into behavioral variability in naïve and stressed animals, we performed a principal component analysis (PCA) on the *z*-normalized behavioral scores. The clustering analysis, based on the first and second principal components (PC), categorized animals into three distinct groups (Fig. 2A). The highest contributor to the PC1, which accounted for 85.6% of the explained variance, was social interaction time (39.8%), while for PC2, explaining 9.5% of the variance, was predominantly composed by the discrimination index in the NOR test (60.9%) (Fig. 2B). Clusters 1 and 2 comprised naïve animals, while cluster 3 exclusively consisted of stressed animals, being all separated by the contribution of PC1. We refer to these three groups as “naïve with higher-behavioral *z*-score” (naïve HBZ; cluster 1), “naïve with lower-behavioral *z*-score” (naïve LBZ; cluster 2), and “stressed animals” (cluster 3), according to their distinct behavioral phenotypes, as revealed by the integrated behavioral *z*-score) (Fig. 2C). Our findings were validated by the Silhouette, Elbow, and GAP statistical methods (Supplementary Fig. 1A–C), which indicate *k* = 3 as the optimal number of clusters and supported our PCA and unsupervised *k*-mean clustering analysis (Supplementary Fig. 1D).

Compared to naïve HBZ, naïve LBZ showed decreased time spent in the light zone of the LDB (Fig. 2D), reduced sociability (Fig. 2E), and deficits in the NOR test (Fig. 2F). Stressed animals showed marked impairments in all behavioral tests, distinguishing them from both naïve groups (Fig. 2D–F). These data confirm that adolescent naïve animals cluster into two distinct behavioral phenotypes, while adolescent stress exposure impacts all animals, forming a third cluster.

### Transcriptomic analysis of the PFC reveals changes in mitochondrial pathways

**Fig. 3:**
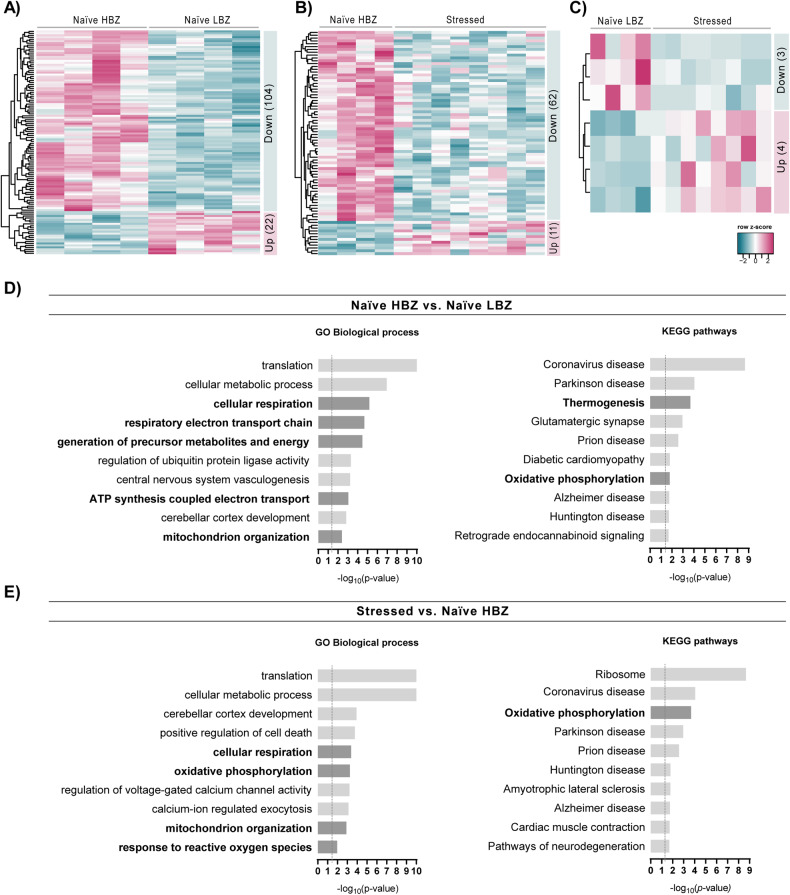
Gene expression changes in the PFC of naïve LBZ and stressed animals. Heatmap of normalized mRNA expression levels of differentially expressed genes (DEGs, *p*_adj_ < 0.1) in the PFC comparing **A** naïve LBZ vs. naïve HBZ, **B** naïve HBZ vs. stressed animals, and **C** naïve LBZ vs. stressed animals. The overall numbers of DEGs up and down-regulated are described in their respective heatmaps. The dendrograms represent the hierarchical clustering of genes according to gene expression (Euclidean distance method). **D** Gene set enrichment analyses of DEGs in the PFC of naïve LBZ and **E** stressed animals compared to naïve HBZ. Mitochondria-associated pathways are highlighted in black font.

Considering the variable behavioral phenotypes among naïve animals and their modulation by stress, we postulated that these variations would be reflected in prefrontal gene expression profiles. Employing a hypothesis-free transcriptomic analysis of the PFC, we performed a series of comparisons, contrasting two groups at a time (naïve LBZ vs. naïve HBZ; stressed vs. naïve HBZ; stressed vs. naïve LBZ) (Supplementary Table 1). These analyses revealed 126 DEGs in naïve LBZ (Fig. 3A), 73 DEGs in stressed animals compared to naïve HBZ (Fig. 3B), and 7 DEGs in stressed animals compared to naïve LBZ (Fig. 3C).

Next, we performed a gene set enrichment analysis to identify the most significantly affected pathways when comparing naïve LBZ and stressed animals to HBZ rats (Fig. 3D, E). At the transcriptomic level, some of the top 10 genes affected common pathways in these groups were associated with mitochondrial function, including “oxidative phosphorylation” and “mitochondrion organization”. The cellular component analysis also revealed “mitochondrion” as an enriched set in both naïve LBZ and stressed animals (Supplementary Fig. 2A, B). Notably, naïve LBZ animals displayed enrichment of gene sets related to “Thermogenesis”. In contrast, stressed animals showed enrichment in “Response to reactive oxygen species” (Fig. 3E). These findings reveal transcriptional disparities in mitochondrial and metabolic genes among behaviorally distinct subgroups.

### Mitochondrial-related gene signatures differ between naïve LBZ and stressed animals

**Fig. 4:**
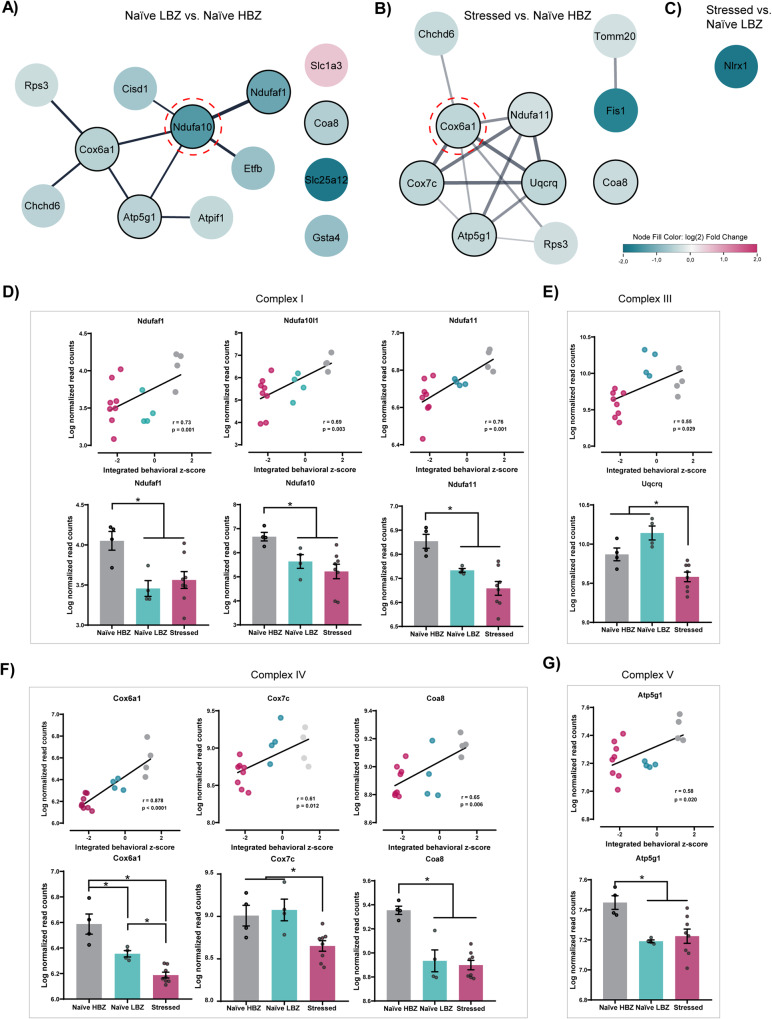
Expression profiles of mitochondria-related genes in the PFC in different adolescent behavioral phenotypes. Summary of DEGs maintained by Cytoscape mitochondrion-node filter from **A** naïve LBZ vs. naïve HBZ, **B** stressed animals vs. naïve HBZ, and **C** stressed animals vs. naïve LBZ. The node’s colors represent the corresponding genes’ relative expression (log_2_(Fold Change)). Nodes rounded with red lines correspond to central identifiers in the network topology analysis. Nodes rounded with black lines correspond to genes from different OXPHOS complexes and were used to correlate the normalized gene expression levels with the integrated behavioral *z*-score and distinguish gene expression profiles among intergroups. Lines (edges) represent predicted gene–gene interaction. **D–G** A positive *Pearson* correlation was observed between the expression levels of OXPHOS genes and behavioral phenotype. *r* and *p* values are described in the graphs. Levels of gene expression from different OXPHOS complexes were significantly different among groups: **D** Complex I = *Ndufaf1* (*F*_2,13_ = 6.16, *p* = 0.01), *Ndufa10* (*F*_2,13_ = 5.63, *p* = 0.02), and *Ndufa11* (*F*_2,13_ = 11.80, *p* = 0.001). **E** Complex III = *Uqcrq* (*F*_2,13_ = 14.70, *p* = 0.0005). **F** Complex IV = *Cox6a1* (*F*_2,13_ = 25, *p* < 0.0001), *Cox7c* (*F*_2,13_ = 11, *p* = 0.001), and *Coa8* (*F*_2,13_ = 19.4, *p* = 0.0001). **G** Complex V = *Atp5g1* (*F*_2,13_ = 7.21, *p* = 0.008). Data are shown as mean ± SEM. *p < 0.05; One-way ANOVA, followed by Tukey’s multiple comparison test.

Variability in mitochondrial phenotypes within brain regions associated with anxiety, sociability, and cognition has been described in adult outbred [30, 31, 53, 54] and inbred rodents [55]. Therefore, prefrontal DEGs were filtered by their probability to be present in the mitochondrion compartment with a high confidence score (Fig. 4A–C). We identified 15 mitochondria-related genes comparing naïve LBZ and naïve HBZ, with most of them being down-regulated in naïve LBZ, except for *Slc1a3* (Fig. 4A). Comparing stressed and naïve HBZ animals, we found 10 mitochondria-related genes, all down-regulated (Fig. 4B). Only one gene (down-regulated *Nlrx1*) emerged when comparing stressed with naïve LBZ animals (Fig. 4C).

Next, we sought to enable the inference of molecular function associations through an interaction prediction pipeline [51], thereby supporting a more comprehensive interpretation of mitochondria-related gene variation among behavioral clusters [56, 57]. To achieve this, we constructed interactive networks, where nodes represented DEGs with a padj of <0.1, and edges represented predicted functional and/or physical gene–gene interactions (Supplementary Fig. 3A, B). In the network topology analysis of mitochondrial-related genes (Fig. 4A), *Ndufa10* was identified as having the highest betweenness and closeness centrality for naïve LBZ. *Ndufa10* was associated with the following genes: (1) *Ndufa1, Cox6a1*, and *Atp5g1*—subunits of OXPHOS; (2) *Cisd1*—a member of the CDGSH domain-containing family; and (3) *Etfb*—an enzyme located in mitochondrial matrix space.

The mitochondrial-related gene with the highest betweenness and closeness centrality for stressed animals was *Cox6a1* (Fig. 4B), which was predicted to link with genes encoding (1) subunits of OXPHOS—*Ndufa11, Uqcrq, Cox7c*, and *Atp5g1*; (2) a component of MICOS complex—*Chchd6*; and (3) a ribosomal protein—*Rps3*. Additionally, stressed animals displayed a second network formed by the interaction of *Tomm20* and *Fis1* genes. These findings point to distinct molecular functions associated with changes in OXPHOS according to behavioral subgroups.

### Expression profiles of mitochondria-related genes in the PFC are associated with behavioral phenotypes

Since “oxidative phosphorylation” was a commonly enriched pathway among subgroups, and most of the mitochondria-related DEGs were directly linked to OXPHOS, we performed an in-depth analysis of gene reads from each electron transport chain complex. Positive correlations were observed for genes of complex I (*Ndufaf1, Ndufa10l1* and *Ndufa11*; Fig. 4D), complex III (*Uqcrq*; Fig. 4E), complex IV (*Cox6a1, Cox7c*, and *Coa8*; Fig. 4F), and complex V (*Atp5g1*; Fig. 4G) with the behavioral *z*-scores. Additionally, we re-evaluated gene expression levels based on behavioral phenotypes (Fig. 4D–G). DEGs were significantly down-regulated in both naïve LBZ and stressed animals compared to naïve HBZ, except for *Uqcrq and Cox7c* (Fig. 4F), which were reduced only in stressed animals. Re-evaluating gene expression levels based on behavioral phenotypes revealed differences in *Cox6a1* among groups, with stressed animals being the most affected. Our results support the notion that distinct behavioral phenotypes are correlated with prefrontal mitochondria-related gene expression.

### Changes in mitochondrial respiratory function and ROS production rate account for individual behavioral variations and long-lasting stress response

**Fig. 5:**
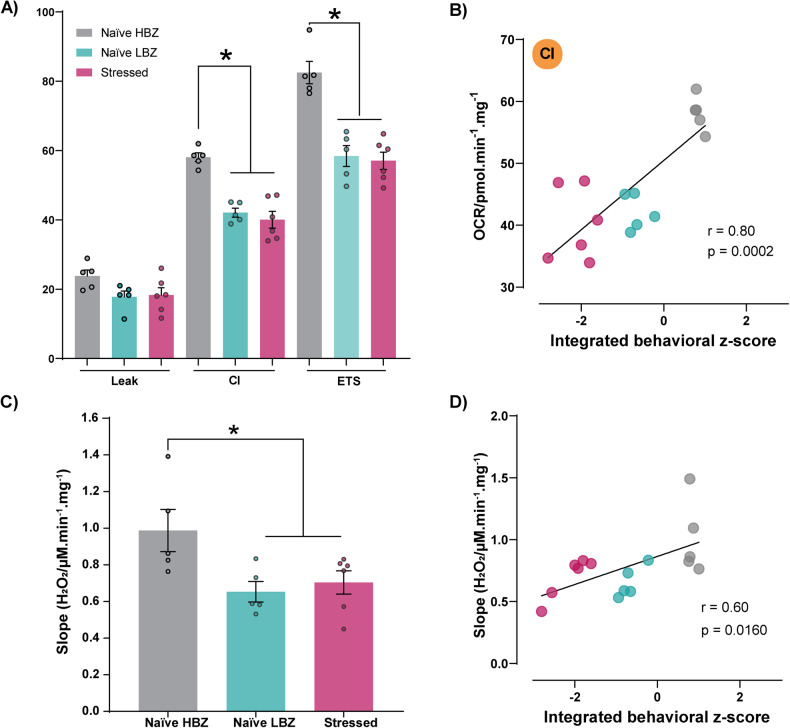
Profile of mitochondrial respiratory capacity and ROS production rate according to behavioral phenotypes. Mitochondrial respiratory capacity and hydrogen peroxide production in the PFC of naïve HBZ, naïve LBZ, and stressed animals was evaluated on PND 51 (*n* = 5 naïve HBZ, 5 naïve LBZ, and 6 stressed) after behavioral tests. **A** Naïve LBZ and adolescent stressed animals showed reduced complex I activity (CI) (*F*_2,13_ = 27.82, *p* < 0.0001) and maximal electron transport system capacity (ETS) (*F*_2,13_ = 33.95, *p* < 0.0001), without changing Leak. **B** A positive Pearson correlation was observed between behavioral phenotypes and OXPHOS respiration linked to CI. **C** Naïve LBZ and adolescent stress also showed reduced hydrogen peroxide (H_2_O_2_) released by CI on PND 51 (*F*_2,13_ = 4.43, *p* = 0.0341). **D** Positive Pearson correlation with behavioral phenotypes and H_2_O_2_ production rate. Data are shown as mean ± SEM. *p < 0.05. One-way ANOVA, followed by Tukey’s multiple comparison test. *r* and *p* values are described in the graphs.

Our transcriptomic analysis alluded to a potential alteration in mitochondrial respiratory function in the PFC of naïve LBZ and stressed animals due to the down-regulation of OXPHOS genes. To explore this possibility, we performed high-resolution respirometry of PFC samples on PND51. Both naïve LBZ and stressed animals exhibited reduced CI and ETS, but not in leak, suggesting a diminished mitochondrial OXPHOS capacity primarily linked to complex I (Fig. 5A). Notably, the integrated behavioral *z*-score positively correlated with CI (Fig. 5B). Mitochondrial electron transport is the primary source of ROS, and its dysregulation can lead to changes in ROS levels. Accordingly, we found a decrease in hydrogen peroxide production rate in naïve LBZ and stressed animals after stimulating CI (Fig. 5C), which was also associated with behavioral performance (Fig. 5D).

### Mitochondrial respiratory capacity and hydrogen peroxide production rate are enhanced one day after adolescent stress

**Fig. 6:**
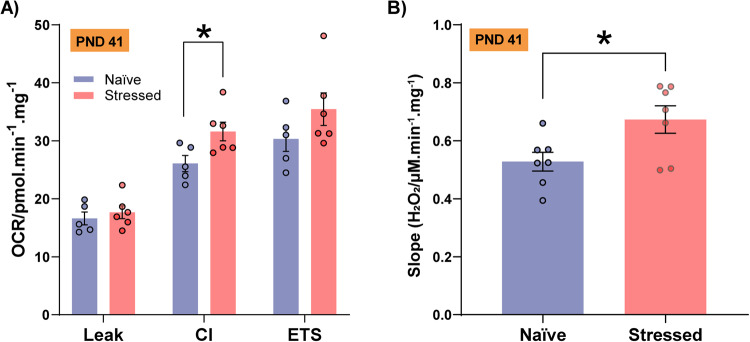
PFC mitochondrial respiration and hydrogen peroxide levels one day after adolescent stress. **A** Adolescent stress increased OXPHOS capacity due to complex I activity (CI) (*n* = 5 naïve and 6 stressed; *t*_9_ = 2.36, *p* = 0.03) (**B**) and enhanced hydrogen peroxide (H_2_O_2_) levels (*n* = 7/group; *t*_12_ = 2.53, *p* = 0.03) on PND 41. Data are shown as mean ± SEM. *p < 0.05; unpaired *t*-test.

Our results pointed to long-lasting changes in prefrontal mitochondrial features in stressed animals, characterized by the down-regulation of OXPHOS genes, enriched pathways related to “response to oxidative stress” and lower complex I respiratory activity 10 days after stress exposure. The temporal changes in mitochondrial respiratory function and ROS abnormal levels after 10 days of stress could represent responses to the “allostatic load” induced by the chronic stress protocol [58, 59]. Then, we postulated that differences in mitochondrial adaptive response under stressful conditions may explain these findings. In another cohort, one day after the adolescent stress (PND41), animals showed higher oxygen consumption at the OXPHOS state linked to complex I activity (Fig. 6A) compared to naïve animals. This aligns with the increased hydrogen peroxide production rate (Fig. 6B).

## Discussion

Given the significance of successful adolescent development for adequate sociability and cognitive performance in adulthood, the identification of critical genes moderating distinct behavioral phenotypes and stress responses can shed light on the mechanisms underpinning the development of psychiatric disorders. Here, using adolescent rats, we showed that: (1) naïve animals exhibited considerable behavioral variability, resulting in two distinct behavioral phenotypes and the exposure to adolescent stress led to marked behavioral changes, such as anxiety, reduced sociability, and cognitive impairment; (2) differences in mitochondria-related genes in the PFC correlated with these behavioral phenotypes, with most of the DEGs being down-regulated in both naïve LBZ and stressed animals compared to naïve HBZ; (3) both naïve LBZ and stressed animals showed reduced mitochondrial respiratory capacity and redox dysregulation in the PFC. These findings unveil new molecular-based insights and strengthen the view that brain mitochondria-related genes and mitochondrial respiratory capacity relate to differences in anxiety, sociability, and cognition functions [39, 60–63].

While individual differences in anxiety traits have been observed in both inbred and outbred adult rodents [30, 31, 53, 54, 64–67], few studies have investigated this variability in adolescent naïve animals, as in the outbred Sprague Dawley population. Most reports have focused on individual variability in risk-taking, learning, and other cognitive processes during adolescence [8]. In our study, the time spent in the light zone of LDB identified two distinct behavioral profiles in naïve animals and the drastic impact of stress during adolescence. Animals exposed to stress also presented marked deficits in sociability and novel object recognition memory. Inter-individual differences in naïve rats were also observed for these domains. Adolescence is an adaptive period that shapes social and behavioral phenotypes based on individual differences, some insensitive to social contexts [68]. Social behaviors acquired during childhood and adolescence, such as playing and dominance, are crucial for social organization, which impact subsequent interactions with conspecifics and can predict behavioral phenotypes and cognitive skills development [69, 70].

In agreement with reports showing inter-individual variation in sociability among adolescent animals [70, 71], our PCA results defined a PC1 with a significant contribution from the “social interaction time” variable. At the same time, PC2 was influenced by the “novel object recognition index” variable. It is worth noting that when these variables were projected onto a coordinate system, PC1 signs were all negative for cluster 3 (stressed animals), positive for cluster 1 (naïve HBZ), and intermediate to cluster 2 (naïve LBZ). These findings indicate that variability among naïve and stressed animals appears more prominently associated with sociability, even though subgroups also differ in anxiety-like behavior and cognitive function. Indeed, individual differences in anxiety traits are proposed to influence social dominance [30, 66], motivation [31, 66], spatial learning/memory [67], and depressed-like behaviors [31].

Consistent with the idea that mitochondrial features are linked to variability in social behaviors [30–32] and are modulated by stress [72], we observed that animals with lower behavioral scores (naïve LBZ and stressed animals) exhibit, except for complex II, down-regulated expression of genes directly linked to OXPHOS, which were positively associated with behavioral performance. Previous studies identified down-regulated genes for several complex I subunits in the dorsal lateral PFC and anterior cingulate cortex in SCZ and autism subjects [73–75]. Reduced expression of the *Uqcrq* gene, which encodes a complex III subunit, was observed only in stressed animals. Genes for complex III subunits are less expressed in cortical regions of patients with neurodevelopmental disorders [75–77]. Expression of the gene for complex IV subunit, *Cox6a1*, was impacted in naïve LBZ and stressed animals compared to naïve HBZ. Moreover, lowered expression levels of complex IV subunits were linked to cognitive impairments in SCZ [78–80].

The most down-regulated mitochondrial-related gene in Naïve LBZ was *Slc25a12*, which encodes the Aralar/slc25a12/AGC1 protein, involved in the transport of aspartate from mitochondria to cytosol in exchange for glutamate. Therefore, it is also a component of the malate-aspartate shuttle, essential to maintain glycolytic pyruvate supply to neuronal mitochondria [81]. A recent study has identified Aralar/slc25a12/AGC1 as the GABA sequesters mitochondrial transporter upon increased mitochondrial activity in Drosophila mutants of the human homolog CYFIP1, leading to social behavioral deficits [82]. Particularly, the only mitochondria-related gene up-regulated in LBZ animals was *Slc1a3*, which encoded a sodium-dependent, high-affinity amino acid transporter that mediates the uptake of l-glutamate, l-aspartate, and d-aspartate. The transporter, also called glutamate aspartate transporter 1 (GLAST-1), is highly expressed in astrocytes and found in the mitochondrial inner membrane as part of the malate-aspartate shuttle [83]. Metabolite levels for glutamate, glutamine, and GABA in some brain regions, such as the nucleus accumbens, predict cognitive performance and anxiety in healthy humans [84, 85]. However, the connection between transcriptomic changes presented in this study and the energy metabolism still needs to be further consolidated.

The mitochondria-related gene network of naïve LBZ revealed that *Ndufa10* is predicted to be associated with genes encoding OXPHOS subunits (*Ndufa11*, Cox6a1, and *Atp5g1*) and genes that regulate mitochondrial respiratory capacity, such as *Cisd1* and *Etfb*. The *Cisd1* gene, which encodes CDGSH iron–sulfur domain protein 1 in the mitochondrial outer membrane, regulates oxidative capacity and metabolism, possibly serving as a redox and pH sensor for mitochondrial function [86]. Notably, presynaptic mitochondrial proteome comparing PND7 and PND42 revealed increased Cisd1 levels at adolescence [87]. The electron-transfer flavoprotein b, a protein encoded by the *Etfb* gene, mediates the transfer of electrons from a series of mitochondrial dehydrogenases to the respiratory chain and is required for normal mitochondrial fatty acid oxidation and amino acid metabolism [88].

In stressed animals, our gene association network analysis revealed that the *Cox6a1* gene is predicted to interact with other DEGs for complex I, III, and V subunits. Also, it is associated with the *Chchd6* (Coiled-coil-helix-coiled-coil-helix domain-containing protein 6) gene, which is a component of the MICOS complex, a large protein complex of the mitochondrial inner membrane that maintains crista junctions and the formation of contact sites to the outer membrane [89]. Although no stress-induced psychiatry disorder has so far been associated with changes in *Chchd6* gene expression, dysfunction of MICOS and OXPHOS impairment were found in neuronal cells lacking *Disc1* (disrupted in Schizophrenia-1) gene [90]. Also, *Cox6a1* was predicted to interact with a ribosomal protein gene (*Rps3*), which reduces cellular reactive oxygen species (ROS) levels and mitochondrial DNA damage when located in the mitochondrion. Furthermore, adolescent stress led to the down-regulation of *Fis1* and *Tomm20* genes, both key mediators of mitochondrial fission and mitochondrial translocase system, respectively. We also found a down-regulation of the *Nlrx1* gene regulated in stressed animals compared to naïve LBZ. *Nlrx1* is involved in oxidative stress and mitochondrial dynamics and detects mitochondrial protein-importing stress [91].

At the functional level, the behavioral variability among naïve animals and their mitochondrial gene signature was associated with reduced PFC OXPHOS capacity and changes in hydrogen peroxide production rate. Indeed, high-anxious outbred naïve rats exhibit reduced mitochondrial complex I and II proteins and respiratory capacity in the nucleus accumbens [30, 31]. Also, significant individual differences in mitochondrial content and respiratory chain activities across brain areas were described in inbred naïve mice not exposed to stressors [55].

Emerging evidence indicates that stress can impact mitochondrial function in different brain regions [39]. One day after stress (PND 41), the OXPHOS capacity of PFC mitochondria increased in stressed animals, accompanied by elevated hydrogen peroxide production. However, 10 days after stress exposure (PND 51), we found a decrease in OXPHOS capacity and reduced production of hydrogen peroxide linked to complex I activity. These findings suggest that adaptive changes in cortical energy metabolism, such as increased OXPHOS capacity after stress, may precede constitutive defects in mitochondrial function. Stress during critical neurodevelopmental periods, such as adolescence, can lead to a glutamatergic overdrive onto fast-spiking GABAergic interneurons expressing parvalbumin (PV), potentially changing the balance of excitation and inhibition (E/I) in cortical circuits that support cognitive processes [92–94]. PV interneurons are highly vulnerable to stress, particularly during neurodevelopment, and its reduction in cortical regions of animal models to study SCZ is accompanied by oxidative stress [95]. Although our adolescent stress protocol also negatively impacts PV-positive cells [24], this study lacks cell type specificity. In the future, it will also be essential to perform similar studies in female animals, especially under stressful conditions, as females are affected when the same stress protocol is applied later in adolescence [36].

Overall, our results provide evidence that distinct behavioral phenotypes in adolescent naïve animals and stressed animals are associated with diversity in mitochondrial features. While behavioral individualities, as characterized by LBZ vs. HBZ, may be related to inherited variations in mitochondrial gene expression and respiration, stress-associated changes are likely adaptative. Although further causal experiments are required, our study provides new insights into potential mechanisms of this variability, focusing on PFC mitochondrial-associated processes.

### Supplementary information

Supplemental Figures

Supplemental Tables

## Data Availability

The data that support the findings of this study and the R script analyses are available from the corresponding author upon reasonable request. Read counts of PFC RNA-sequencing are available on 10.6084/m9.figshare.24125793.v1.
