# Supplementary material for: Transcriptomic analysis reveals mitochondrial pathways associated with distinct adolescent behavioral phenotypes and stress response
Source: Transl Psychiatry. 2023 Nov 17;13:351. doi: 10.1038/s41398-023-02648-3 (PMC10656500; doi:10.1038/s41398-023-02648-3)
Supplement: Supplementary file 2 — Supplemental Tables [file 41398_2023_2648_MOESM2_ESM.pdf]

gene ID	gene name	baseMean	log2FoldChange	lfcSE
ENSRNOG00000001420	Fis1	446.7826351	-1.614954097	0.221477704
ENSRNOG00000049075	Fabp5	920.2745424	-0.54988949	0.083702505
ENSRNOG00000019578	Rps16	2284.752148	-0.480196986	0.095195143
ENSRNOG00000011705	Stmn2	5857.141333	-0.256622837	0.05355413
ENSRNOG00000057823	Ubc	7284.900444	-0.220135054	0.045921434
ENSRNOG00000022877	Poldip3	331.5915967	-0.502856781	0.113761149
ENSRNOG00000023919	Nipsnap2	372.6837776	-0.316733076	0.072859063
ENSRNOG00000010812	Osbpl6	193.5223516	0.469502947	0.107358216
ENSRNOG00000048470	Pomp	374.6192538	-0.343850407	0.082091012
ENSRNOG00000047151	Rps25	686.1213721	-0.357066852	0.085797754
ENSRNOG00000048258	Cisd2	346.5416293	-0.438555686	0.105680002
ENSRNOG00000013292	Rps19l2	746.4679784	-0.250553611	0.06066076
ENSRNOG00000030278	AABR07021008.1	228.9501179	-0.360491011	0.091636957
ENSRNOG00000029670	RGD1564606	860.8776555	-0.249893216	0.064338787
ENSRNOG00000054294	Rprd2	814.2057693	-0.312384132	0.081601632
ENSRNOG00000010189	Rps24	1367.63053	-0.223522178	0.059417875
ENSRNOG00000017359	Itm2c	5833.332045	-0.205554655	0.055318027
ENSRNOG00000047287	Nucks1	584.3340908	-0.282020346	0.075750994
ENSRNOG00000020833	Cspg5	291.9319946	0.524137783	0.141847578
ENSRNOG00000007235	Atp5mc1	1312.250869	-0.302114342	0.083728709
ENSRNOG00000011542	Coa8	509.2625146	-0.258754957	0.072817864
ENSRNOG00000001170	Cox6a1	533.4705119	-0.354998159	0.09836052
ENSRNOG00000004159	Flii	211.5203644	0.563077124	0.158057517
ENSRNOG00000047365	Fau	1777.175085	-0.25441895	0.071593525
ENSRNOG00000007532	RGD1305178	240.8789989	-0.32491794	0.091298479
ENSRNOG00000011817	Rpl22l1	1353.280228	-0.198072206	0.055736506
ENSRNOG00000032825	Rpl30	787.2874821	-0.269122524	0.075506373
ENSRNOG00000017418	Rps3	1751.670091	-0.222240745	0.061892849
ENSRNOG00000004214	Rpl26	2039.074475	-0.203111218	0.057318124
ENSRNOG00000061058	Csde1	263.6012365	-0.51165853	0.146729004
ENSRNOG00000058920	Coa8	274.6807164	-0.274100053	0.079012574
ENSRNOG00000006030	Ptprz1	421.199668	0.319339248	0.092133024
ENSRNOG00000039249	Rpl31l4	1074.159499	-0.260958507	0.075038691
ENSRNOG00000019980	Tomm20	1692.343163	-0.191808379	0.055215403
ENSRNOG00000059018	Tmem196	758.1443262	-0.219061208	0.063404617
ENSRNOG00000051711	Kpna1	394.9625605	-0.292941718	0.084994618
ENSRNOG00000033146	Rps19l2	763.9249442	-0.22941189	0.067507463
ENSRNOG00000060248	Chchd6	881.0774272	-0.292536955	0.086697947
ENSRNOG00000040273	RGD1562402	559.8726242	-0.269992536	0.08006536
ENSRNOG00000004805	Stac2	322.025935	0.337750123	0.10045211
ENSRNOG00000018533	Iffo1	8881.518245	-0.133987632	0.039948136
ENSRNOG00000026260	Rpl11	1938.370834	-0.206659414	0.06218959
ENSRNOG00000015691	Inka2	999.0484795	-0.334878144	0.101004927
ENSRNOG00000018217	Syt5	2143.387685	-0.202397982	0.061187087
ENSRNOG00000019973	Arl3	1588.26982	-0.260892413	0.079060976
ENSRNOG00000039473	Ccdc152	2247.830072	0.276657313	0.084573034
ENSRNOG00000030237	Cox7c	443.5097092	-0.356980098	0.109569287
ENSRNOG00000023385	Rpl37a	482.6490158	-0.303432107	0.092545692
ENSRNOG00000000491	Spdef	314.283543	0.421914285	0.129252031

ENSRNOG00000036816	Wls	241.1607926	0.310411057	0.095149573
ENSRNOG00000040429	Mir674	457.7914968	-0.332494127	0.102869694
ENSRNOG00000051286	Chchd2	736.5518613	-0.325017679	0.101454213
ENSRNOG00000009350	Sez6	207.1464922	0.577615598	0.180888729
ENSRNOG00000023182	AABR07030085.1	621.7370023	-0.286055569	0.091973338
ENSRNOG00000052707	Cacna1a	410.4044466	0.311374373	0.099716043
ENSRNOG00000007221	Dut	869.6829265	-0.218574502	0.070645805
ENSRNOG00000020495	Eif3k	365.2584375	-0.336461718	0.107564817
ENSRNOG00000047321	Hba-a3	196.7617682	-2.14320919	0.693606943
ENSRNOG00000048109	LOC100365839	1810.219009	-0.18350936	0.05892068
ENSRNOG00000046533	LOC100364335	309.1492794	-0.368282363	0.11825149
ENSRNOG00000058105	Hbb	230.4454194	-2.035942569	0.656027328
ENSRNOG00000048320	Ndufa11	753.5837508	-0.203617745	0.065655101
ENSRNOG00000003951	Psmc1	904.020216	-0.18276697	0.058855122
ENSRNOG00000019106	Rps17	558.89126	-0.254488246	0.081572231
ENSRNOG00000024578	Ttyh2	729.5800513	-0.211572511	0.068449603
ENSRNOG00000005630	AABR07060778.1	1606.555541	-0.208599628	0.068078704
ENSRNOG00000031703	AABR07071294.1	580.8888982	-0.217719718	0.071485595
ENSRNOG00000061299	Hbb-bs	237.1085437	-2.027329459	0.663678164
ENSRNOG00000010237	Map7d1	1291.860505	-0.189742839	0.062363619
ENSRNOG00000061213	Mrps36	355.8830297	-0.221578837	0.072527953
ENSRNOG00000010479	Mycbp2	573.8734563	0.236414304	0.077507646
ENSRNOG00000013386	Psmb5	1629.423865	-0.207568431	0.068049208
ENSRNOG00000016387	Rpl34	1005.897544	-0.238303005	0.077649985
ENSRNOG00000008629	Secisbp2l	547.7578906	0.230629225	0.076198955
ENSRNOG00000048342	Tbca	669.9082193	-0.215187145	0.071155379
ENSRNOG00000006542	Atp6v0c	729.1067321	-0.232409362	0.077034667
ENSRNOG00000030614	NA	735.9023292	-0.213133678	0.070854871
ENSRNOG00000015987	Rap1gds1	1208.957294	-0.259763268	0.086804183
ENSRNOG00000037540	Tmem256	196.8460308	-0.283722562	0.095022159
ENSRNOG00000004252	Eif3h	1330.777781	-0.188132242	0.063258016
ENSRNOG00000016896	Rpl3	1461.505209	-0.235959851	0.079268941
ENSRNOG00000051180	Chchd2	659.5441852	-0.401797455	0.135422202
ENSRNOG00000014104	Myo5b	286.8156045	-0.238147621	0.080634665
ENSRNOG00000019992	Cnbd2	354.9946048	-0.47095666	0.15973828
ENSRNOG00000000920	Phkg1	546.1904771	-0.185728934	0.063189396
ENSRNOG00000042499	NA	5562.169654	-0.18599518	0.063639983
ENSRNOG00000001991	Atp5po	2385.41122	-0.13913351	0.047801098
ENSRNOG00000001142	Prkab1	268.8799968	0.233137496	0.080117993
ENSRNOG00000020263	Atp1a3	5798.529934	-0.173839115	0.060049965
ENSRNOG00000004196	Rps29	2492.272813	-0.264916256	0.09152356
ENSRNOG00000028884	Atp5mg	1468.378372	-0.226146518	0.078283875
ENSRNOG00000005457	Lamp5	844.4971586	0.284357821	0.098891382
ENSRNOG00000008551	NA	1473.920166	-0.198819575	0.069089847
ENSRNOG00000005116	NA	1312.83775	-0.257542255	0.089823713
ENSRNOG00000024309	Cox6b1	1599.893307	-0.140579494	0.049160093
ENSRNOG00000037897	Rps19	3133.85337	-0.142341051	0.049801228
ENSRNOG00000014010	Gfra2	383.2774554	0.231670297	0.081164658
ENSRNOG00000013742	Large1	834.1219967	0.18580487	0.065272594
ENSRNOG00000016580	Rps23	2167.765544	-0.158306257	0.055697104

ENSRNOG00000020455	Cst6	284.1603167	-0.355054224	0.12578636
ENSRNOG00000043098	Mt2A	436.5143262	-0.327394741	0.116929196
ENSRNOG00000009722	Chd3	1203.778573	-0.166909452	0.060084123
ENSRNOG00000028273	Tmem150b	1165.218562	-0.181402761	0.065278656
ENSRNOG00000059539	NA	3317.209424	-0.148096451	0.05355283
ENSRNOG00000059061	Uqcr10	1196.278861	-0.161221107	0.058279131
ENSRNOG00000052802	Aldoa	3165.599082	-0.735842327	0.266802376
ENSRNOG00000005109	Rprm	255.2740056	-0.38873617	0.14100794
ENSRNOG00000016411	Rps12	683.4640228	-0.181013028	0.065759059
ENSRNOG00000051483	Selenow	1534.052054	-0.204157097	0.074414664
ENSRNOG00000020075	Eef1g	1691.252196	-0.16380643	0.06002971
ENSRNOG00000010915	Ermp1	198.3331808	0.316852222	0.115962944
ENSRNOG00000002151	NA	3301.752626	-0.147200493	0.053921981
ENSRNOG00000028449	Rpl9	911.2039109	-0.217441722	0.079814765
ENSRNOG00000007219	NA	2790.597541	-0.157005492	0.057803013
ENSRNOG00000001971	Bbx	892.5269651	-0.176741413	0.065142665
ENSRNOG00000013110	Nudt2	273.3928628	-0.248861352	0.092012029
ENSRNOG00000005512	Ndufa4	1711.795835	-0.162201223	0.060246094
ENSRNOG00000042458	AABR07046707.1	679.4798329	-0.154400039	0.057412717
ENSRNOG00000061441	AABR07071935.1	338.9073086	-0.319988446	0.119121251
ENSRNOG00000002934	Atp1b1	316.873128	-0.220920351	0.082435474
ENSRNOG00000013290	Nrip3	682.154102	0.261654457	0.097621003
ENSRNOG00000017940	Rere	210.778062	0.266117691	0.099438718
ENSRNOG00000050206	Shank2	403.3918718	0.22505039	0.084129728
ENSRNOG00000011202	Chrna4	228.0949713	-0.246718316	0.092498782
ENSRNOG00000010353	Dnajb6	271.5211585	-0.258063946	0.096658456
ENSRNOG00000056487	Oxr1	643.5778603	0.261083831	0.097997495
ENSRNOG00000004107	Rpl23	469.3969907	-0.208667524	0.078313947
ENSRNOG00000017086	AABR07054367.1	9257.51427	-0.28558994	0.107507101
ENSRNOG00000047628	Khsrp	276.0483012	-0.401572807	0.151236724
ENSRNOG00000003401	AABR07029195.1	265.1351454	-0.219646879	0.082985674
ENSRNOG00000013461	Ralbp1	390.6398857	-0.238039232	0.089859263
ENSRNOG00000029995	Herpud2	223.9159531	0.26105513	0.098922606
ENSRNOG00000002456	NA	770.6084312	-0.502396265	0.190212241
ENSRNOG00000026455	Gpd1l	287.5466122	0.237248762	0.090093762
ENSRNOG00000016163	Slc1a3	2118.64574	0.250528494	0.095088101
ENSRNOG00000017467	Rab4a	350.2488299	0.218934287	0.083282905
ENSRNOG00000043348	Rpl39	671.8423266	-0.227328163	0.086560263
ENSRNOG00000020527	Drap1	431.9978472	-0.232039529	0.088523127
ENSRNOG00000062143	NA	4257.269501	-0.179679803	0.068604738
ENSRNOG00000002005	Tmprss11f	4876.759901	-0.201996797	0.077028318
ENSRNOG00000019704	Resp18	1043.454941	-0.176015943	0.067380472
ENSRNOG00000003833	Nenf	542.0600823	-0.22015873	0.084401147
ENSRNOG00000018651	Agtpbp1	718.4245757	0.151743958	0.058534107
ENSRNOG00000012207	Dst	475.651409	0.254489776	0.097998827
ENSRNOG00000004216	Lrr1	313.10517	-0.22809783	0.088087699
ENSRNOG00000015231	Mrpl44	485.5393246	-0.241913361	0.093280491
ENSRNOG00000019328	Phgdh	213.821602	0.320554291	0.123729739
ENSRNOG00000058909	Rps9	3477.956427	-0.23693355	0.091540304
ENSRNOG00000018645	Rpsa	1659.37249	-0.163870071	0.063121077

ENSRNOG00000017029	Snx1	233.3895766	-0.239528983	0.091994618
ENSRNOG00000023387	NA	844.3293433	-0.157275382	0.060839074
ENSRNOG00000019240	Ampd2	233.8166021	0.229817306	0.089070758
ENSRNOG00000049394	NA	847.0646299	-0.161522374	0.062787205
ENSRNOG00000026646	Ndufs5	1604.793807	-0.134358455	0.052220728
ENSRNOG00000029209	Rnf13	313.5659095	0.241978846	0.093909114
ENSRNOG00000019262	Trak1	480.9658946	0.18826878	0.073106633
ENSRNOG00000030174	AABR07042903.1	898.4058321	-0.226850016	0.088343338
ENSRNOG00000010434	Dync1li1	458.2278316	-0.338708796	0.132291298
ENSRNOG00000051615	Hmgn2	623.5973488	-0.239675493	0.093570082
ENSRNOG00000031315	Rpl36a	262.5446413	-0.261670533	0.10215974
ENSRNOG00000011276	Tmem254	207.5725758	0.298910793	0.116725034
ENSRNOG00000018320	Rps15a	2743.252271	-0.196938645	0.077093975
ENSRNOG00000013171	Grm2	308.5782103	-0.322845946	0.127045938
ENSRNOG00000007900	Tspan33	284.7694385	-0.220758176	0.087141182
ENSRNOG00000020027	Arhgef2	354.7438356	0.195317113	0.077641318
ENSRNOG00000020698	Rnd2	833.4747246	-0.192423572	0.076615541
ENSRNOG00000019491	Stard10	370.7495819	-0.216770674	0.086381987
ENSRNOG00000028543	AABR07052523.1	1424.775236	-0.13546738	0.054320442
ENSRNOG00000004781	Crmp1	1206.419329	-0.16036426	0.064272189
ENSRNOG00000043103	Frrs1l	967.0290398	0.330666717	0.132334691
ENSRNOG00000008103	Mdh1	4858.066218	-0.139939579	0.055978229
ENSRNOG00000049269	NA	1686.194519	-0.148827934	0.059588202
ENSRNOG00000001979	Rcan1	281.7789464	0.24518501	0.098200813
ENSRNOG00000014368	Eif4g3	477.9447413	0.221610974	0.089192831
ENSRNOG00000040354	Mir673	874.7109572	0.310015885	0.125141002
ENSRNOG00000011237	Zc3h13	226.1623803	-0.313730992	0.126650322
ENSRNOG00000030238	Fndc5	583.5950298	0.242481844	0.098007396
ENSRNOG00000000648	Jmjd1c	218.0809784	0.271235687	0.109821734
ENSRNOG00000007793	Pnrc1	624.6564999	-0.168203993	0.06807826
ENSRNOG00000011057	Mfn1	413.6759429	0.210563155	0.085372911
ENSRNOG00000002312	Atp10d	387.8919928	-0.210891435	0.085675638
ENSRNOG00000016196	Dlgap1	790.3488641	0.146181727	0.059507049
ENSRNOG00000001488	Psmb1	1122.071647	-0.137203832	0.055819807
ENSRNOG00000004155	Samd14	841.6002663	0.207319721	0.08438562
ENSRNOG00000012393	NA	467.221749	-0.283392827	0.115952453
ENSRNOG00000015320	Atp5mc2	522.4209296	-0.167160794	0.068525273
ENSRNOG00000034237	Rpl39	545.4438359	-0.225257922	0.092375176
ENSRNOG00000029644	Clec2l	849.8123835	0.189261827	0.077731692
ENSRNOG00000049330	Mrpl30	490.7511997	-0.268391689	0.110466528
ENSRNOG00000031022	NA	616.5599563	-0.172992436	0.071189058
ENSRNOG00000004621	Rtn4	459.7076053	-0.178173742	0.073297411
ENSRNOG00000001525	Igsf11	254.6119318	-0.244799177	0.100951662
ENSRNOG00000016795	Rabl6	308.683427	-0.268645537	0.110705619
ENSRNOG00000007948	Nf2	332.1760986	0.201006758	0.082992175
ENSRNOG00000008919	Arpp21	1899.161177	-0.203034096	0.084289766
ENSRNOG00000036876	Faap20	234.6993161	-0.213407221	0.088548318
ENSRNOG00000003280	Grin2c	241.2301442	0.24878168	0.10345862
ENSRNOG00000000622	NA	2879.100488	-0.15542371	0.06444513
ENSRNOG00000034025	Ptprj	212.3530155	0.280390684	0.116468224

ENSRNOG00000056535	U6	233.2173613	0.22524156	0.093754534
ENSRNOG00000002343	Uchl1	11481.82356	-0.155070139	0.064543441
ENSRNOG00000043419	Hist3h2ba	348.3823365	-0.235777028	0.098352652
ENSRNOG00000009439	Eef1a1	10579.72845	-0.126009126	0.052800348
ENSRNOG00000006867	Etv1	568.1224628	0.201603265	0.084675863
ENSRNOG00000020867	Numbl	608.0468475	0.187600415	0.07876874
ENSRNOG00000019926	Ramp1	600.2436867	-0.183673965	0.076878998
ENSRNOG00000000524	RGD735065	458.9958362	-0.172393962	0.072250763
ENSRNOG00000001238	Rsrc2	298.0042192	0.189517754	0.079452004
ENSRNOG00000014524	S1pr3	523.3089248	0.216217162	0.090761109
ENSRNOG00000010420	Sem1	225.7042382	-0.304750961	0.12798725
ENSRNOG00000054374	Smim29	771.8645971	0.228983673	0.096227683
ENSRNOG00000045558	Cd34	194.9069021	-0.443127846	0.186518706
ENSRNOG00000007605	AABR07052430.1	375.1696349	-0.217190768	0.091724344
ENSRNOG00000001448	Hip1	232.7053435	0.267614242	0.113013366
ENSRNOG00000050910	NA	452.2597351	-0.24063364	0.101615737
ENSRNOG00000024065	Zfp575	198.4345929	-0.27817866	0.117588756
ENSRNOG00000018279	Sfxn1	496.5637938	-0.189631979	0.080231437
ENSRNOG00000016638	Gnb1	1738.723913	-0.473574534	0.20076074
ENSRNOG00000026528	Mrps33	259.1799455	-0.225502101	0.095732499
ENSRNOG00000049229	Pomp	770.7063403	-0.211916002	0.089929175
ENSRNOG00000008516	AABR07049516.1	419.2098017	-0.245116468	0.104237004
ENSRNOG00000038951	NA	282.3604084	-0.20141837	0.085703686
ENSRNOG00000003099	Ubqln2	924.8574914	-0.151443622	0.064436923
ENSRNOG00000004258	Chst7	194.9322671	0.236036096	0.100595589
ENSRNOG00000007629	Sf3a3	330.3892343	-0.631867146	0.269476383
ENSRNOG00000012136	Mrps30	277.6994011	-0.215668513	0.09210317
ENSRNOG00000055331	Ints10	247.8888195	0.258804287	0.110629025
ENSRNOG00000025715	Dynlrb1	1306.27757	-0.143482639	0.061466915
ENSRNOG00000018797	Myrip	458.1519448	0.198377551	0.0852856
ENSRNOG00000019352	Emc6	253.6327105	-0.270723795	0.116818056
ENSRNOG00000013718	Herc2	269.2265386	0.295545607	0.127690203
ENSRNOG00000011879	Nfat5	206.0189708	0.272471994	0.117648152
ENSRNOG00000004813	Rtcb	328.9210276	-0.170185651	0.073727103
ENSRNOG00000003743	Dars1	523.1856937	-0.144398469	0.062892791
ENSRNOG00000022868	Ell3	193.0798608	0.311500361	0.135608042
ENSRNOG00000009630	Hsd17b12	1039.195259	-0.136028294	0.059169953
ENSRNOG00000049912	NA	2339.00991	-0.210158221	0.091232958
ENSRNOG00000029512	NA	265.4858833	-0.203334097	0.088539091
ENSRNOG00000018273	Ncl	376.0273351	-0.182649776	0.07944005
ENSRNOG00000007989	Chst1	1281.974164	0.119925006	0.052407644
ENSRNOG00000017876	Dnajc21	293.2893935	0.244300936	0.106648395
ENSRNOG00000059714	Hsp90aa1	6511.461051	-0.116030286	0.050731969
ENSRNOG00000002864	Nacc1	194.5454793	0.289974387	0.126842584
ENSRNOG00000054058	Osbpl1a	472.9759153	-0.229002253	0.099978216
ENSRNOG00000030345	Rps20	227.8596341	-0.28139158	0.122705646
ENSRNOG00000048396	NA	1623.049832	-0.184596496	0.081080866
ENSRNOG00000060728	NA	15748.6198	-0.106063837	0.046644964
ENSRNOG00000015733	NA	2491.098694	-0.108313479	0.047696372
ENSRNOG00000019154	Zmynd8	267.70925	0.202295911	0.089097615

ENSRNOG00000021223	Ptpra	988.2117587	-0.138688347	0.061126984
ENSRNOG00000031287	Cacna2d3	193.7429366	0.252816122	0.111725281
ENSRNOG00000053756	NA	469.8783826	-0.16530471	0.073071599
ENSRNOG00000002632	Naca	1907.731247	-0.115185957	0.051090875
ENSRNOG00000021688	AABR07056686.1	360.8022333	-0.179472797	0.080523374
ENSRNOG00000001710	Abcf3	323.5747488	-0.197095737	0.08764519
ENSRNOG00000009826	Bche	433.0497075	-0.332566194	0.14915626
ENSRNOG00000034161	Cox6b1-ps1	507.6260689	-0.152732034	0.068478701
ENSRNOG00000010320	Efnb3	502.9836012	-0.162112693	0.072647192
ENSRNOG00000011015	Hivep2	503.8513031	0.185846064	0.083274393
ENSRNOG00000014201	Manf	348.0719264	0.177091508	0.078800221
ENSRNOG00000020093	NA	1241.10718	-0.176226748	0.078482154
ENSRNOG00000051964	NA	358.8880827	-0.214533638	0.09623055
ENSRNOG00000029366	Prrt2	1029.919106	-0.141411988	0.063378678
ENSRNOG00000004741	Rpl19	2078.080674	-0.152183713	0.068262026
ENSRNOG00000018039	Sncb	4710.372827	-0.112859121	0.050536903
ENSRNOG00000004534	Spdya	246.9437128	-0.23494224	0.105392668
ENSRNOG00000049020	Tceal3	586.9837554	-0.198680129	0.088806899
ENSRNOG00000019961	Tcte1	951.5814007	0.144504115	0.064829385
ENSRNOG00000057290	Tmem168	3544.912197	-0.126581695	0.056832346
ENSRNOG00000043026	AC128207.2	277.1245798	-0.268278691	0.120532397
ENSRNOG00000043212	Dip2a	276.2985902	0.300700437	0.135190546
ENSRNOG00000053811	Arg2	257.3043405	-0.239147425	0.107733799
ENSRNOG00000006505	Borcs5	191.5264127	-0.23338279	0.105169576
ENSRNOG00000020552	Fosl1	1022.143124	-0.14341891	0.064566907
ENSRNOG00000016550	Dclk2	201.8248011	0.230353104	0.10409643
ENSRNOG00000017309	Rsrp1	499.8733081	0.207068436	0.093631949
ENSRNOG00000000747	NA	570.1655035	0.240851847	0.109358573
ENSRNOG00000009085	Prkag2	405.2199297	-0.163503529	0.074243971
ENSRNOG00000015655	Ptgfrn	205.6345832	0.211369166	0.096049328
ENSRNOG00000010176	Map2k1	288.1231462	-0.317026576	0.144277615
ENSRNOG00000013493	Psma4	356.6565949	-0.181641277	0.082673281
ENSRNOG00000005482	Sap30bp	226.602962	-0.268132643	0.12211611
ENSRNOG00000016511	Pitrm1	1069.118779	-0.141785041	0.064688897
ENSRNOG00000052764	AABR07016779.1	333.0657831	-0.180835445	0.082642592
ENSRNOG00000053288	Ank3	263.6807129	0.22533552	0.103431245
ENSRNOG00000018937	Gstm2	1040.974051	-0.164211233	0.075343677
ENSRNOG00000023764	Kctd2	443.2184904	-0.157066334	0.072092422
ENSRNOG00000016280	Btrc	220.6640794	0.222955422	0.102785863
ENSRNOG00000030871	Calm-ps2	2182.849205	-0.110556277	0.05086518
ENSRNOG00000021269	Chgb	1928.852706	-0.137883902	0.063606772
ENSRNOG00000049949	Hapln4	684.2619227	0.166877708	0.077134513
ENSRNOG00000002365	Itm2a	453.8338945	0.225020921	0.103431121
ENSRNOG00000011841	Map2	2283.307749	0.130331482	0.060266423
ENSRNOG00000000579	Marcks	1360.230565	-0.135564388	0.062668701
ENSRNOG00000021177	NA	2907.816927	-0.126705371	0.058378357
ENSRNOG00000008289	NA	4872.54914	-0.166490539	0.076924833
ENSRNOG00000001837	Sst	1632.396226	-0.170469862	0.078638753
ENSRNOG00000021230	Ubox5	276.6065124	0.198094044	0.091412731
ENSRNOG00000003050	Tbc1d9b	621.6309885	-0.190217644	0.088086957

ENSRNOG00000011156	Clstn3	1921.259696	-0.103942646	0.048317352
ENSRNOG00000028236	RGD1309104	284.3963512	-0.20787262	0.096650377
ENSRNOG00000039017	Naa50	281.5793765	-0.217529332	0.10126251
ENSRNOG00000009078	Mrpl37	832.6677738	0.131905359	0.061598946
ENSRNOG00000056637	Abhd16a	388.6480896	0.268745766	0.12591026
ENSRNOG00000059510	Amph	1365.250046	-0.131778827	0.061707897
ENSRNOG00000054352	Atp6ap1	520.4486592	0.287942241	0.134717594
ENSRNOG00000034198	Tceal9	707.3873875	-0.135301569	0.063404459
ENSRNOG00000015221	Eif3f	1759.686239	-0.135135606	0.063436543
ENSRNOG00000013683	S1pr1	592.4468264	-0.168163247	0.07898583
ENSRNOG00000011498	Psip1	1114.300163	-0.147418044	0.069620602
ENSRNOG00000021262	Slc23a2	208.0849135	0.212407794	0.100667388
ENSRNOG00000031092	Rock1	339.413495	0.214749665	0.101877589
ENSRNOG00000029004	NA	214.1584529	0.220149385	0.104528772
ENSRNOG00000010912	Mrps25	659.6905088	-0.157668274	0.074920753
ENSRNOG00000020233	Rabac1	1248.234385	-0.14660543	0.069717703
ENSRNOG00000000127	Kpna6	215.8822136	-0.280300967	0.133431906
ENSRNOG00000020386	Ash1l	248.7161029	-0.290100283	0.138322822
ENSRNOG00000022582	Ccnq	224.7244807	-0.269673899	0.128910766
ENSRNOG00000045636	Fasn	628.3228167	0.164771976	0.078765548
ENSRNOG00000020354	Mrpl23	340.2915577	-0.218602598	0.104160522
ENSRNOG00000008305	NA	574.7418044	0.222278945	0.106174911
ENSRNOG00000018075	Spcs1	317.199092	-0.186626869	0.089208371
ENSRNOG00000010550	Trappc3	425.6612604	-0.194018667	0.092645411
ENSRNOG00000006589	Mif	2847.661794	-0.120917162	0.057851102
ENSRNOG00000016930	Ube2s	487.7716922	-0.167469146	0.080297122
ENSRNOG00000014635	Clta	218.7530946	-0.215484008	0.103488528
ENSRNOG00000003177	Mat2b	858.0433024	-0.146616904	0.070464599
ENSRNOG00000015055	Scg2	728.1133209	-0.142316277	0.068599041
ENSRNOG00000055770	NA	243.0996274	-0.208939772	0.100901344
ENSRNOG00000023456	Rnf41	441.1270476	-0.177358963	0.085654592
ENSRNOG00000005667	Astn1	692.7623085	0.145866444	0.070922842
ENSRNOG00000013492	Cap1	405.9813038	0.18096562	0.087642097
ENSRNOG00000036802	NA	565.0457727	0.248934325	0.120707323
ENSRNOG00000046168	NA	273.1051992	0.239479409	0.116377517
ENSRNOG00000017106	NA	609.063586	-0.162410175	0.078983593
ENSRNOG00000018939	Rexo2	716.3357511	-0.170704209	0.083019334
ENSRNOG00000031889	Rpl6-ps1	1568.3731	-0.135814304	0.066035263
ENSRNOG00000008555	Rps20	444.4429657	-0.177297515	0.085781478
ENSRNOG00000016388	Sphkap	294.160544	0.203175078	0.098565025
ENSRNOG00000009778	Tmem59	794.9611276	-0.122476175	0.059427473
ENSRNOG00000004612	Ppp1cb	1145.039363	-0.127104892	0.061945314
ENSRNOG00000022922	Slc25a12	296.6706929	-0.849169933	0.413745191
ENSRNOG00000020185	Wdr6	234.9001041	-0.232784696	0.113728197
ENSRNOG00000026050	Epb41l4a	239.2853296	-0.218293725	0.106812829
ENSRNOG00000001781	Lmln	407.0791426	-0.179335695	0.087689535
ENSRNOG00000039464	NA	267.7923837	0.230439616	0.112820765
ENSRNOG00000053972	NA	378.6378887	-0.168157652	0.082346371
ENSRNOG00000011969	Dock9	285.6526552	0.204634639	0.100355617
ENSRNOG00000002314	NA	375.9827961	0.170212027	0.083515848

ENSRNOG00000013516	Sf3b1	484.7445975	0.177047159	0.086906041
ENSRNOG00000003550	Acbd6	308.4034356	-0.194963591	0.095780837
ENSRNOG00000056248	Marf1	378.6540198	0.173647652	0.085346387
ENSRNOG00000010967	Cdc37l1	464.6114198	-0.144406653	0.071095924
ENSRNOG00000020828	Pde4a	1208.848175	-0.195239524	0.096170592
ENSRNOG00000026978	Oscp1	1282.086938	-0.148537816	0.073239367
ENSRNOG00000033603	AABR07034315.1	207.7813767	-0.180231577	0.089231084
ENSRNOG00000029341	AABR07036375.1	194.3550147	-0.242541333	0.120110905
ENSRNOG00000016660	Cox5b	2447.759346	-0.128136307	0.063472348
ENSRNOG00000013981	Ptpn5	570.4880711	0.173115193	0.085666127
ENSRNOG00000049056	AABR07051450.1	1311.494784	-0.532473971	0.265004177
ENSRNOG00000031052	NA	362.4942105	-0.170399648	0.084800999
ENSRNOG00000001709	Ap2m1	1685.025427	-0.473512576	0.236265108
ENSRNOG00000015292	Cul2	251.6309891	-0.172362276	0.086139741
ENSRNOG00000002618	Ivns1abp	203.8192044	0.196302785	0.098060115
ENSRNOG00000032835	NA	358.4200005	-0.224132023	0.112011593
ENSRNOG00000020427	Borcs8	262.258129	-0.204779077	0.102738216
ENSRNOG00000018816	Cox5a	2501.639573	-0.110381825	0.055386259
ENSRNOG00000031517	AABR07039133.2	209.4511042	-0.177715216	0.089422683
ENSRNOG00000005185	Nxph3	357.5078705	-0.29726208	0.149677946
ENSRNOG00000019326	Cfdp1	713.2520981	-0.109925337	0.055492606
ENSRNOG00000010649	Ctnnd2	781.2083562	0.142052629	0.071651389
ENSRNOG00000003209	Pcp4l1	302.6983846	-0.2032516	0.102584087
ENSRNOG00000015002	Abhd15	340.8420506	0.182227519	0.092445756
ENSRNOG00000001596	Atp5mc3	4835.464151	-0.126530065	0.064139218
ENSRNOG00000010252	Hexa	290.3558966	0.196174525	0.099519905
ENSRNOG00000015134	Map3k12	601.8756754	-0.123715513	0.062741379
ENSRNOG00000052279	NA	307.4311357	-0.162570951	0.082477004
ENSRNOG00000057102	Pafah1b2	589.1321372	-0.128210505	0.065055843
ENSRNOG00000047088	Rab10	608.8797742	0.15456224	0.078566633
ENSRNOG00000012121	Sdf2	306.1618215	-0.184410422	0.09373593
ENSRNOG00000007663	Rps6	1592.097333	-0.117388875	0.059775528
ENSRNOG00000000467	Ring1	251.4701324	-0.180082261	0.091863674
ENSRNOG00000021232	Ddrgk1	332.4069693	-0.163627384	0.083713326
ENSRNOG00000024689	Hopx	535.0060456	0.218969472	0.11205673
ENSRNOG00000050044	Ptp4a2	433.3028258	-0.162719829	0.083413896
ENSRNOG00000023538	Aldh5a1	347.0608826	0.142265557	0.073014342
ENSRNOG00000014928	Apba1	255.6823196	-0.316288819	0.162569418
ENSRNOG00000005668	Ndufa8	720.7640141	-0.162260947	0.083372902
ENSRNOG00000047708	Gstz1	233.4387099	0.178756407	0.092046055
ENSRNOG00000033855	AABR07062466.1	541.622301	-0.146245282	0.075521824
ENSRNOG00000055068	NA	7588.063439	-0.101158846	0.052222841
ENSRNOG00000005711	Ptprd	470.1441415	0.171144777	0.088376891
ENSRNOG00000008704	Tmem97	201.6101995	-0.221625481	0.114756251
ENSRNOG00000048326	NA	1800.270354	-0.099350797	0.051540257
ENSRNOG00000046390	Tceal3	577.4201393	-0.181315722	0.094232419
ENSRNOG00000029173	AABR07053716.1	358.8723863	-0.179090269	0.093279915
ENSRNOG00000013254	Dctn6	578.9061556	-0.127047994	0.066359332
ENSRNOG00000058908	Hmgb1	663.3546148	-0.130972952	0.068316371
ENSRNOG00000059081	NA	258.7369556	0.198409144	0.103609508

ENSRNOG00000001517	Pdk1	209.0398966	-0.252260297	0.131605674
ENSRNOG00000013874	Rplp1	7870.582781	-0.112384769	0.058715971
ENSRNOG00000053122	Scn1a	232.1591274	0.255009312	0.132859001
ENSRNOG00000017602	Il34	385.1604035	0.150216457	0.078696328
ENSRNOG00000015568	NA	268.2305432	0.188327351	0.098725496
ENSRNOG00000058580	Paip1	331.3181423	-0.146111261	0.076578955
ENSRNOG00000055471	Ywhah	12684.44621	-0.110057663	0.057627555
ENSRNOG00000013657	Stmn3	5603.003567	-0.105844713	0.055546371
ENSRNOG00000001440	Mdh2	1937.446692	-0.103301896	0.054252718
ENSRNOG00000010379	Celf1	543.1675326	0.188258248	0.09920008
ENSRNOG00000047574	Nrxn3	342.3980517	0.180769764	0.095203614
ENSRNOG00000006247	Copa	478.7446781	-0.122435607	0.064632925
ENSRNOG00000029339	Ndufs5	714.3780989	-0.128008047	0.067616692
ENSRNOG00000028158	Tex22	418.1858926	0.141859316	0.074929577
ENSRNOG00000010975	Adnp	501.3294146	-0.138814814	0.073487792
ENSRNOG00000061335	NA	286.2602785	-0.181586436	0.096052142
ENSRNOG00000031368	NA	271.9381996	0.195222305	0.103371044
ENSRNOG00000020088	Klhl26	315.1938131	-0.166339533	0.088220765
ENSRNOG00000057493	NA	523.5606173	-0.143965126	0.076368051
ENSRNOG00000006375	Vdac1	271.5609711	-0.447254433	0.237762438
ENSRNOG00000025418	Armc9	216.7031113	0.186237291	0.099443883
ENSRNOG00000008098	Lrrc4	355.3110574	-0.160129327	0.085566384
ENSRNOG00000018676	NA	589.7733493	-0.124466564	0.06649479
ENSRNOG00000001746	Ncbp2	383.1400592	-0.144600283	0.077253968
ENSRNOG00000006784	Sema4f	313.392782	-0.232309074	0.123917488
ENSRNOG00000002908	Slc35a4	192.7327255	0.182011255	0.097492595
ENSRNOG00000017397	Upf3a	233.5309804	-0.182120789	0.097555536
ENSRNOG00000031560	AABR07030162.1	1392.522228	-0.112789524	0.060685281
ENSRNOG00000049959	Igsf21	582.6955609	-0.132421028	0.071217336
ENSRNOG00000001120	Med13l	226.6867274	0.197154344	0.106027246
ENSRNOG00000023781	Plec	375.0459445	0.238016577	0.128209223
ENSRNOG00000021010	Arl2	214.8253382	-0.213855194	0.115471565
ENSRNOG00000006137	Arid1a	473.4357099	0.170033085	0.091895872
ENSRNOG00000008615	Mal2	1193.427939	-0.126993201	0.068675075
ENSRNOG00000001309	Camkk2	721.2881366	-0.192242893	0.104057374
ENSRNOG00000013211	Chchd3	375.638181	-0.14273242	0.077428479
ENSRNOG00000001789	Zfp148	246.4402956	0.157853248	0.085642063
ENSRNOG00000031816	Nckipsd	522.1763707	-0.126046965	0.068472308
ENSRNOG00000008609	Capn3	517.6437839	0.15366513	0.083652886
ENSRNOG00000013393	Eif2a	195.0007462	-0.189402791	0.103223565
ENSRNOG00000014819	Hap1	664.9626055	-0.178485365	0.097334274
ENSRNOG00000057497	NA	522.6086134	-0.129651004	0.070669252
ENSRNOG00000008065	Wwc1	203.7343292	0.247501282	0.134973738
ENSRNOG00000053468	Tuba1b	9330.232811	-0.112989839	0.06169696
ENSRNOG00000000024	Hebp1	408.7151984	-0.131010542	0.071708769
ENSRNOG00000050767	Nrn1	1815.90586	-0.105899181	0.057989953
ENSRNOG00000001769	Tnk2	604.8540867	0.131233463	0.071900809
ENSRNOG00000001958	Ift57	256.9860762	-0.17974423	0.098779253
ENSRNOG00000009094	Nudt4	623.114834	0.161191952	0.088630995
ENSRNOG00000042781	Ropn1l	650.2462609	0.111066893	0.060911524

ENSRNOG00000052207	Rps15al4	436.1131154	-0.129534368	0.071227888
ENSRNOG00000020020	Wbp1l	327.4835886	0.138824646	0.076253655
ENSRNOG00000010945	Ywhab	4128.948759	-0.076497491	0.042002633
ENSRNOG00000012733	Ankrd12	362.5253356	-0.207624141	0.115211672
ENSRNOG00000013346	Asb14	205.1218576	0.173536393	0.095731409
ENSRNOG00000009308	Bcl2l10	703.3249512	-0.124063203	0.0684519
ENSRNOG00000001173	Cabp1	928.2947031	0.113225812	0.062783214
ENSRNOG00000017939	Chn1	4974.292024	-0.086636649	0.047964062
ENSRNOG00000043201	Coq8a	331.7072356	0.213689312	0.118488244
ENSRNOG00000061845	Cttnbp2	237.5806332	0.18091313	0.101044885
ENSRNOG00000006557	Cyfip2	3690.959485	0.093523574	0.052027343
ENSRNOG00000003078	Dcaf6	231.9782484	-0.186712588	0.104112267
ENSRNOG00000026589	Dpy19l1	367.1079931	0.1569964	0.086685572
ENSRNOG00000004395	Fbxw11	313.4306942	0.170493607	0.094405916
ENSRNOG00000053362	Gabarapl1	2945.321085	-0.110405544	0.061654732
ENSRNOG00000019638	Lmna	394.6373672	-0.150982176	0.08366396
ENSRNOG00000002340	NA	249.5260148	-0.221491399	0.122649818
ENSRNOG00000050660	NA	547.2949414	-0.157172036	0.087094883
ENSRNOG00000058695	NA	242.0842325	-0.190738395	0.106179601
ENSRNOG00000055748	NA	231.7122843	0.214833906	0.119729377
ENSRNOG00000021179	Naa40	338.4286888	-0.166760133	0.09264568
ENSRNOG00000042503	Ndufv2	1717.941847	-0.106153674	0.058643139
ENSRNOG00000007256	Necab1	401.6579563	0.139574714	0.077474235
ENSRNOG00000010784	Scg3	2031.404145	-0.101276825	0.056387346
ENSRNOG00000020646	Sdhaf2	311.4740661	-0.161489312	0.090196551
ENSRNOG00000000170	Slc30a4	230.62761	0.200790414	0.111085334
ENSRNOG00000019048	Sod2	521.5276186	-0.12673209	0.070286265
ENSRNOG00000003769	Tmem163	404.2582574	0.175188179	0.097072716
ENSRNOG00000009449	Trim35	710.7281186	-0.114647143	0.063868189
ENSRNOG00000005614	Txn2	468.291973	-0.1415269	0.078039558
ENSRNOG00000016684	Wnk2	486.2047817	0.123869261	0.06892504
ENSRNOG00000008195	Ywhaz	7230.297524	-0.106777952	0.059580769
ENSRNOG00000013916	Nefm	523.1362286	-0.379265058	0.212025867
ENSRNOG00000054272	Dhx57	194.7590143	0.17232721	0.096678877
ENSRNOG00000011523	Macroh2a1	582.5260787	-0.173456513	0.097274816
ENSRNOG00000038074	NA	501.654468	-0.153809224	0.086174415
ENSRNOG00000013498	Pex14	283.8516006	-0.169616878	0.095064363
ENSRNOG00000002305	Slc15a2	199.8791383	-0.199422575	0.11167238
ENSRNOG00000006961	Snrpb	255.2645104	0.200921173	0.112537753
ENSRNOG00000023150	Gpi	3409.433611	-0.087159392	0.049038691
ENSRNOG00000013061	Tusc3	1542.861454	-0.120547937	0.06786999
ENSRNOG00000056940	Cited2	505.2574359	-0.185012605	0.104356985
ENSRNOG00000006051	NA	256.6260796	0.20423047	0.115095186
ENSRNOG00000005108	Wfs1	1395.425239	-0.154833939	0.087334689
ENSRNOG00000021256	Adra1d	261.4562754	0.197086026	0.111337733
ENSRNOG00000010133	Bpgm	523.0923104	0.168986583	0.095633386
ENSRNOG00000011914	Dtnb	394.0461501	-0.136019786	0.076974738
ENSRNOG00000006859	Insig1	465.6049196	0.167530235	0.094811209
ENSRNOG00000050899	AABR07054362.1	591.3930553	-0.182638308	0.103464076
ENSRNOG00000011463	Psmb2	420.351497	-0.136909254	0.077784429

ENSRNOG00000016831	Serpinh1	286.0214554	0.231477129	0.13146819
ENSRNOG00000020138	Slc4a3	406.5514755	0.141947476	0.080572569
ENSRNOG00000004774	Ccdc175	290.8974273	-0.164105453	0.093384876
ENSRNOG00000015630	Cct7	983.725591	0.105287837	0.060034849
ENSRNOG00000015303	Fbll1	457.6499889	-0.122438062	0.069794024
ENSRNOG00000014060	Mras	749.0209735	0.128540354	0.073304198
ENSRNOG00000052019	NA	901.6010407	-0.146434972	0.083471844
ENSRNOG00000043466	NA	253.0776114	-0.190648963	0.108777349
ENSRNOG00000023338	Tspan2	344.7589756	0.200742342	0.114360286
ENSRNOG00000020744	Adar	317.2641889	0.134560687	0.076828333
ENSRNOG00000025936	Rpl6	3022.989692	-0.112613172	0.064406583
ENSRNOG00000003990	Grb2	1104.547165	-0.116750532	0.066846266
ENSRNOG00000007104	Itpr1	909.1155814	0.122631035	0.070307687
ENSRNOG00000002983	Nfix	776.5017765	-0.465899875	0.267261725
ENSRNOG00000005828	Skp1	1452.579846	-0.148310184	0.085163474
ENSRNOG00000024142	Arglu1	550.001092	-0.132363569	0.076253275
ENSRNOG00000052174	NA	256.141299	-0.157952801	0.090979467
ENSRNOG00000003117	Psmd12	781.0486966	-0.100002233	0.057620493
ENSRNOG00000046345	Rpn1	1696.316614	-0.089185513	0.051406183
ENSRNOG00000060821	Rnf114	305.6591437	-0.180124814	0.104054535
ENSRNOG00000023365	AABR07013086.1	3538.835858	-0.084599881	0.04899453
ENSRNOG00000025011	Chd8	288.9931941	-0.178942607	0.103969896
ENSRNOG00000012260	Ddx25	357.5407104	-0.146871893	0.085348643
ENSRNOG00000000940	Flt1	610.8664392	0.125546591	0.072792251
ENSRNOG00000053479	NA	304.5881868	0.223531383	0.129635837
ENSRNOG00000051382	NA	362.9829959	-0.127953781	0.074210673
ENSRNOG00000019955	Ogdhl	291.4512614	0.140833852	0.081590255
ENSRNOG00000004820	Sar1b	438.2589239	-0.124401616	0.072282205
ENSRNOG00000023107	Yeats2	314.0137776	0.145761973	0.084579342
ENSRNOG00000012185	Zfyve21	284.7067188	0.162670688	0.094291602
ENSRNOG00000011550	Kcnab2	592.1428319	0.135276028	0.078707363
ENSRNOG00000018687	Fbxw2	509.2299529	-0.172274421	0.100493614
ENSRNOG00000013971	Psat1	605.2853962	0.163547322	0.095350902
ENSRNOG00000047635	Tmem178b	228.4429628	0.161190602	0.09405608
ENSRNOG00000013714	Snrpd1	247.5712952	-0.170812682	0.099759299
ENSRNOG00000013300	Atp5if1	2589.230519	-0.165820934	0.097260213
ENSRNOG00000005195	Cst3	18037.60835	-0.12096568	0.071130883
ENSRNOG00000052226	NA	472.7739496	-0.172871111	0.101621252
ENSRNOG00000000979	NA	300.3848738	-0.498163088	0.292974667
ENSRNOG00000011949	Ndufb5	492.8184591	-0.119214397	0.069948984
ENSRNOG00000007184	RGD1305298	261.8662111	0.154643346	0.090536457
ENSRNOG00000018774	Rps14	2070.697089	-0.122463557	0.071934977
ENSRNOG00000033152	Rps18l1	321.8462153	-0.230508124	0.135031688
ENSRNOG00000003809	Sat1	649.9488108	0.144663492	0.085092448
ENSRNOG00000020893	Snx27	393.0734077	0.132180095	0.077763574
ENSRNOG00000024907	Tnrc6b	366.583165	0.16121758	0.094815107
ENSRNOG00000011955	AABR07026997.1	7387.597328	-0.106091287	0.062482614
ENSRNOG00000028198	Sh2b3	250.1949932	0.141795794	0.083509926
ENSRNOG00000031244	NA	2147.047195	-0.087299842	0.051483968
ENSRNOG00000008451	Fut8	285.8642088	-0.187173662	0.110491892

ENSRNOG00000020167	Reep5	3021.308692	-0.110137256	0.065078335
ENSRNOG00000001559	Mtx2	215.3829649	0.174966502	0.103672131
ENSRNOG00000062084	NA	755.4114936	-0.111845919	0.066432523
ENSRNOG00000042271	NA	20013.64041	0.149321414	0.088750571
ENSRNOG00000018237	Gstp1	394.130218	-0.145252108	0.086518855
ENSRNOG00000017383	Hikeshi	199.365495	-0.166934547	0.099451046
ENSRNOG00000004616	Npm1	623.5996547	-0.134773639	0.080309815
ENSRNOG00000018020	Apbb1	250.6580855	-0.260972442	0.155674015
ENSRNOG00000032803	NA	1382.778092	-0.093362496	0.055751374
ENSRNOG00000005203	Sec61g	300.7111563	-0.128600246	0.076960499
ENSRNOG00000006759	Ankrd24	333.9243624	-0.158177163	0.094821671
ENSRNOG00000001227	Adarb1	443.5721043	0.130447783	0.078766672
ENSRNOG00000008364	Cat	357.7169946	-0.148207654	0.089072022
ENSRNOG00000007283	Erlec1	453.8410053	-0.123922289	0.074373776
ENSRNOG00000018200	Gad2	498.211358	0.131517291	0.079396119
ENSRNOG00000021685	NA	208.4185796	-0.200605427	0.120648303
ENSRNOG00000013895	NA	1668.790815	-0.094031687	0.056719891
ENSRNOG00000006751	Psmd6	871.3019398	-0.094767053	0.057082203
ENSRNOG00000014097	Psmd7	509.8714988	-0.155875104	0.093653598
ENSRNOG00000004181	Sdccag8	481.3045262	-0.118654969	0.071446744
ENSRNOG00000008090	Txndc12	310.0066219	-0.147899303	0.088759082
ENSRNOG00000025735	Wdr86	333.7438946	-0.144756953	0.087015324
ENSRNOG00000013288	Xpo7	255.9784195	0.154017524	0.092938373
ENSRNOG00000005290	Ywhae	4761.686953	-0.078941012	0.047547667
ENSRNOG00000008194	Znfx1	381.2030923	0.14196832	0.085637178
ENSRNOG00000025705	Armcx2	442.2703938	-0.125109343	0.075725529
ENSRNOG00000008830	Nfe2l1	956.3083254	-0.111351035	0.067420091
ENSRNOG00000005730	Pcmtd1	263.6826729	0.169833117	0.102882881
ENSRNOG00000014621	Rab8a	244.5979584	-0.150447783	0.091175545
ENSRNOG00000014083	Iqsec3	840.8353847	0.147629981	0.089793005
ENSRNOG00000036572	Sfxn4	193.7216459	-0.161941871	0.098462254
ENSRNOG00000010232	Enpp5	259.1345217	-0.147961181	0.090187018
ENSRNOG00000048523	NA	607.127725	-0.12698923	0.077407464
ENSRNOG00000014371	Cdh13	1076.94642	-0.169117247	0.103211807
ENSRNOG00000006143	Ngly1	204.1988841	-0.153460028	0.093692615
ENSRNOG00000002013	Sidt1	412.6151671	0.143412461	0.087535583
ENSRNOG00000021729	Iqub	486.1002737	-0.143840602	0.087942196
ENSRNOG00000006824	Hdac11	1309.674352	-0.096095721	0.058918831
ENSRNOG00000026672	MGC94199	232.6636513	-0.142858129	0.087488639
ENSRNOG00000045999	NA	416.7821343	-0.119711076	0.073284819
ENSRNOG00000002906	Nr1i2	376.9307518	0.159811473	0.097931592
ENSRNOG00000018903	Pik3r1	541.8824821	-0.151534165	0.092931389
ENSRNOG00000012302	Gucy1a1	281.7914982	0.17473926	0.107388218
ENSRNOG00000032348	Rpl35a	354.2130913	-0.16283136	0.100083837
ENSRNOG00000019482	Gnao1	3272.867744	-0.085734871	0.052748554
ENSRNOG00000001082	Abcb9	196.8156035	0.160334883	0.098707357
ENSRNOG00000060793	Eif2s3x	455.4717479	-0.139510156	0.086120882
ENSRNOG00000046271	Gspt1	675.692509	-0.150911453	0.093147675
ENSRNOG00000000938	Ran	1648.943866	-0.105151585	0.064967004
ENSRNOG00000012942	Satb1	214.9283673	0.159858658	0.098762326

ENSRNOG00000052725	Shisal1	247.691106	0.191270589	0.118109358
ENSRNOG00000010830	Slc25a4	5636.963629	-0.072334555	0.044567297
ENSRNOG00000020762	Zfp260	319.5268975	-0.127182733	0.078415916
ENSRNOG00000003365	Cadm3	1280.406315	-0.106678055	0.066004015
ENSRNOG00000002258	Tmem150c	326.6241177	-0.146999535	0.091118058
ENSRNOG00000054271	U6	842.5158027	-0.096879218	0.060030272
ENSRNOG00000003071	Vamp4	660.7412824	-0.115705352	0.071613721
ENSRNOG00000019802	Zfp428	863.78031	-0.110489964	0.068460912
ENSRNOG00000013213	Epha4	470.4170872	0.137682344	0.085434028
ENSRNOG00000050052	Cox19	207.9303934	-0.15662962	0.097281617
ENSRNOG00000013961	Ghitm	677.9449871	-0.10519762	0.065347153
ENSRNOG00000033426	Cdc37	1090.036177	-0.097513339	0.060647012
ENSRNOG00000016596	Hspa4	369.0914532	-0.172392557	0.107280378
ENSRNOG00000057125	Ddr1	204.9959229	0.182319083	0.113658472
ENSRNOG00000050404	Pmepa1	540.9020881	0.125197831	0.078010133
ENSRNOG00000019572	Lsm4	290.4299422	-0.145164642	0.090704277
ENSRNOG00000004574	Ofd1	2469.095626	0.090629641	0.056609251
ENSRNOG00000014382	C2cd5	199.7177433	0.165594589	0.103593993
ENSRNOG00000052141	NA	530.7897305	-0.15002412	0.093938116
ENSRNOG00000048915	Twf2	302.0654086	-0.155179219	0.097252316
ENSRNOG00000010117	Eif3a	407.3515371	-0.130973995	0.082167737
ENSRNOG00000050747	NA	196.3915224	-0.216039165	0.135534057
ENSRNOG00000007967	Sdhb	424.5243979	-0.10699466	0.067159965
ENSRNOG00000010633	Acsl1	205.8197555	0.164906754	0.104108294
ENSRNOG00000047466	Bdnf	266.5669121	-0.157298682	0.099621065
ENSRNOG00000047436	Cabyr	1141.051668	0.137626	0.087907647
ENSRNOG00000007636	Cadps2	206.8932418	-0.154658222	0.097942095
ENSRNOG00000030541	Ccdc154	272.3089423	0.143368506	0.091323091
ENSRNOG00000000304	Cd164	309.1030314	0.14637521	0.092510972
ENSRNOG00000020778	Chrnb2	358.6598869	0.146411943	0.093432038
ENSRNOG00000012263	Cnot7	463.7232506	-0.128420031	0.081351747
ENSRNOG00000000312	Fig4	232.0278552	-0.14878408	0.094799326
ENSRNOG00000027434	Fitm2	213.6493909	-0.171546538	0.109518898
ENSRNOG00000009726	Grip2	207.742982	0.172216239	0.109933819
ENSRNOG00000048193	Hnrnph3	632.4938935	-0.098524707	0.062495426
ENSRNOG00000011910	Hnrnpr	313.8795542	-0.143609805	0.091082324
ENSRNOG00000039278	Mcart1	484.2918357	0.119263594	0.074984948
ENSRNOG00000049287	NA	233.8216441	-0.138505025	0.08735518
ENSRNOG00000048456	NA	539.8945131	-0.128777605	0.081264358
ENSRNOG00000028974	NA	2079.110484	-0.081530918	0.051569262
ENSRNOG00000013548	NA	544.0400154	-0.125610219	0.079791528
ENSRNOG00000053258	NA	5356.554464	-0.117684125	0.075071962
ENSRNOG00000049848	NA	672.1569297	-0.125641486	0.080218101
ENSRNOG00000051885	NA	14956.07448	-0.111483491	0.071186094
ENSRNOG00000021890	Nob1	230.165039	-0.163321113	0.103499267
ENSRNOG00000014761	Rasd2	514.5922146	-0.148555604	0.094590865
ENSRNOG00000028939	Rps29	418.2226399	-0.237537186	0.1511671
ENSRNOG00000027513	Rtel1	208.9839549	0.155159581	0.098178375
ENSRNOG00000018229	Slc45a1	423.7040694	0.11372444	0.072006763
ENSRNOG00000060919	Snord72	401.4268131	-0.139822365	0.088756959

ENSRNOG00000058003	Spon1	366.8218346	-0.161663435	0.102071973
ENSRNOG00000002999	Timmdc1	198.081748	0.160269075	0.101894767
ENSRNOG00000002254	Tmem33	312.407569	-0.152040343	0.096124703
ENSRNOG00000020791	Ube2q1	558.7473697	-0.121006689	0.077152658
ENSRNOG00000006989	Vamp2	8222.735804	-0.077439741	0.049452625
ENSRNOG00000013506	Zfand6	211.4130292	0.151235872	0.095117062
ENSRNOG00000015517	Zfp444	229.2594441	-0.160593271	0.101956062
ENSRNOG00000018590	Drg1	310.7075582	-0.186233806	0.119167429
ENSRNOG00000017689	Itih3	760.9627384	-0.113452809	0.072595732
ENSRNOG00000024737	Tnrc6a	224.1523439	-0.225761038	0.144527925
ENSRNOG00000048686	AABR07066379.1	445.0834703	0.158786565	0.101873808
ENSRNOG00000020542	Krtcap2	319.9871613	-0.13841377	0.088794532
ENSRNOG00000008937	Csrp1	1017.082587	0.112547968	0.072285957
ENSRNOG00000060185	Kansl2	347.7008231	-0.13974579	0.089791184
ENSRNOG00000046858	MGC109340	461.5648405	-0.13626413	0.087599123
ENSRNOG00000027368	Ubald1	531.5614367	-0.138564826	0.089212622
ENSRNOG00000002378	Bpnt1	418.4603185	-0.133623618	0.086084582
ENSRNOG00000020644	Nsg2	3914.562272	-0.088274874	0.056934286
ENSRNOG00000031974	Pcdh10	258.8593901	0.186505431	0.120440343
ENSRNOG00000009740	Slco1c1	313.678127	0.148516096	0.095878505
ENSRNOG00000059605	Ddn	2214.057947	-0.122556781	0.07918444
ENSRNOG00000013017	Arnt2	692.1764734	0.112836433	0.072949944
ENSRNOG00000002141	Cd200	593.899115	-0.115979276	0.075050314
ENSRNOG00000012438	Larp6	210.6038131	-0.167629687	0.10868267
ENSRNOG00000014044	Pank4	557.270418	0.173128039	0.11225602
ENSRNOG00000001098	Pds5b	457.4379159	-0.229639062	0.148932793
ENSRNOG00000059741	Tmc4	905.7046316	-0.099871687	0.064706002
ENSRNOG00000056135	Tsc22d3	501.9008902	-0.154598405	0.100287225
ENSRNOG00000045771	Chl1	202.609471	0.153155799	0.099544372
ENSRNOG00000003844	Dhx15	263.042564	0.148782795	0.096632934
ENSRNOG00000006206	Tmem106b	434.7679965	-0.109977636	0.071450688
ENSRNOG00000018387	Wdr7	404.6368604	0.124067245	0.08071465
ENSRNOG00000019875	Matr3	1292.156105	-0.086234007	0.056158151
ENSRNOG00000003223	Degs1	310.2871167	-0.143959357	0.093994014
ENSRNOG00000004117	Kcnv1	588.8499151	-0.127964456	0.083593426
ENSRNOG00000050061	NA	221.307613	0.153686775	0.100309313
ENSRNOG00000030442	NA	316.9979014	-0.126735281	0.082828021
ENSRNOG00000016907	Ppp5c	817.6561684	-0.134662892	0.087890838
ENSRNOG00000021125	Prdx5	944.3842811	-0.132892987	0.086824213
ENSRNOG00000027271	RGD1359290	362.8632708	0.45916204	0.299526186
ENSRNOG00000027906	Ankrd11	777.8031696	-0.100794542	0.065993504
ENSRNOG00000056855	NA	14973.07686	-0.108926212	0.071278785
ENSRNOG00000018356	Trim3	688.5141852	-0.092281968	0.060436125
ENSRNOG00000019271	Ulk4	1109.091045	-0.080869277	0.052890711
ENSRNOG00000021702	Mcmdc2	419.7546523	0.110459253	0.072441823
ENSRNOG00000007151	Cdk14	301.7611856	0.13225993	0.086933292
ENSRNOG00000017941	Optn	322.4033686	-0.131489916	0.086441299
ENSRNOG00000019551	Psmb6	423.2848174	-0.111115063	0.073021433
ENSRNOG00000007542	Scg5	2602.511921	-0.07750483	0.050964446
ENSRNOG00000010646	Tmem229b	199.6137447	0.155697728	0.102322443

ENSRNOG00000031381	AC129049.1	708.5371011	-0.100868285	0.066465178
ENSRNOG00000001992	Atp6v1a	1972.521027	-0.091565581	0.060297364
ENSRNOG00000046313	NA	2771.680909	-0.107739358	0.070986699
ENSRNOG00000009850	St3gal4	329.1780474	-0.142951681	0.094145693
ENSRNOG00000024808	Stk39	350.6293996	-0.124470496	0.08212153
ENSRNOG00000053919	NA	519.5767889	-0.119863751	0.079213245
ENSRNOG00000019598	Vegfa	255.5186735	0.159345117	0.105274934
ENSRNOG00000001239	NA	530.6227351	-0.116953116	0.077383853
ENSRNOG00000000885	Auts2l1	191.5742755	-0.267953796	0.177771861
ENSRNOG00000000168	Gatm	303.4356178	0.170789952	0.113314391
ENSRNOG00000011946	Ptn	2563.458824	0.107529715	0.071316373
ENSRNOG00000009402	Rabgap1	531.2818437	0.106618359	0.070662459
ENSRNOG00000002749	Spag9	595.9370046	0.121675281	0.080766212
ENSRNOG00000009700	Dync1i1	810.9449339	-0.101142615	0.06727726
ENSRNOG00000002671	Nme2	2953.083419	-0.10425356	0.069400776
ENSRNOG00000037871	Sfxn5	946.256085	0.125780272	0.083729422
ENSRNOG00000023861	Snap91	1797.590497	0.082789785	0.055077422
ENSRNOG00000019476	Agap1	369.6219401	0.138640353	0.092796569
ENSRNOG00000002045	Anxa3	389.9825501	-0.149349411	0.100150211
ENSRNOG00000013536	Cdc42	1044.063243	-0.08785378	0.058842494
ENSRNOG00000000551	Fam241b	492.655483	-0.110520869	0.073897878
ENSRNOG00000003385	Glrx2	488.1503335	-0.099712254	0.066786969
ENSRNOG00000016999	Grp	450.0135909	-0.184085355	0.122848037
ENSRNOG00000060956	NA	1547.617608	-0.123167271	0.08219097
ENSRNOG00000038150	NA	396.3358399	-0.214775476	0.143890023
ENSRNOG00000012046	Prmt5	299.1282676	-0.148855676	0.099855579
ENSRNOG00000015508	Relch	224.9565589	0.15264554	0.102354108
ENSRNOG00000004794	Rtn1	8926.856904	-0.075985086	0.050976726
ENSRNOG00000020475	Sart1	466.194512	-0.200050664	0.133674788
ENSRNOG00000015741	Slc2a13	389.744509	0.122743067	0.081904445
ENSRNOG00000013391	Sorbs2	441.0956755	0.135513682	0.090657018
ENSRNOG00000012199	Sox2	223.6189063	0.17392718	0.116284249
ENSRNOG00000010752	Tmem41b	242.3171795	0.139716403	0.09367428
ENSRNOG00000008001	Rab3b	519.2970917	-0.12091592	0.081174806
ENSRNOG00000017097	Lhpp	291.4438562	-0.12234218	0.082209293
ENSRNOG00000018712	Camk2a	306.8717617	0.172186522	0.115801353
ENSRNOG00000024967	Uqcrb	581.4132282	-0.107529172	0.072382323
ENSRNOG00000011814	Cbx3	939.4326948	-0.085926571	0.057904812
ENSRNOG00000034246	Rps27a-ps1	788.4333968	-0.16854969	0.113604635
ENSRNOG00000014575	Rtca	254.5424089	-0.129125574	0.087048331
ENSRNOG00000018584	Ptma	2466.608838	-0.12282685	0.082895792
ENSRNOG00000021469	Ccdc14	319.5574418	-0.122389585	0.082700457
ENSRNOG00000006652	Ddx1	484.7670088	-0.095894543	0.064933167
ENSRNOG00000006075	Fez1	1634.782593	-0.094257256	0.063856354
ENSRNOG00000051548	Lmod1	1250.501511	0.123475696	0.083613376
ENSRNOG00000046051	NA	703.5292604	-0.118681124	0.080381378
ENSRNOG00000046799	Phb	223.3691685	-0.137559056	0.093134171
ENSRNOG00000009882	Ppp3ca	2393.738203	0.098808234	0.067011865
ENSRNOG00000048853	Slc25a41	2060.344059	-0.112810178	0.076472637
ENSRNOG00000057501	Fam81a	1278.859828	-0.090597217	0.061478852

ENSRNOG00000059240	RGD1359449	372.5251153	-0.123197455	0.083637209
ENSRNOG00000030747	Rpl38	517.6229046	-0.197135666	0.133897726
ENSRNOG00000001171	Coq5	333.0745863	-0.161613967	0.109903756
ENSRNOG00000057676	Myzap	850.0192277	0.101123898	0.068778668
ENSRNOG00000028062	Arpc5	436.1221974	-0.102337534	0.069706431
ENSRNOG00000028956	Mbd3	821.129914	-0.12552044	0.08551739
ENSRNOG00000013602	Usp48	206.4561206	0.177960875	0.121189152
ENSRNOG00000005533	1700067K01Rik	255.2039131	-0.141241041	0.096382143
ENSRNOG00000020950	Syvn1	204.5609761	0.170530904	0.116385413
ENSRNOG00000009161	Clasp2	539.7260379	0.130005479	0.089201911
ENSRNOG00000016343	Dkk3	499.9905196	0.11797547	0.080792446
ENSRNOG00000037476	Galnt9	563.4177039	-0.118735436	0.081588618
ENSRNOG00000007754	NA	207.4858108	0.170006429	0.116241292
ENSRNOG00000019655	NA	397.9060491	0.731556674	0.500740198
ENSRNOG00000032733	NA	204.6669971	-0.147429993	0.10101516
ENSRNOG00000054489	NA	369.3137111	-0.127306116	0.087440067
ENSRNOG00000005064	NA	225.233711	0.145769669	0.100163379
ENSRNOG00000004067	Nrcam	803.4990579	0.100855062	0.069327637
ENSRNOG00000012686	Pomc	506.4818938	0.10795686	0.073892662
ENSRNOG00000020723	Pten	224.5164848	-0.161107634	0.110042631
ENSRNOG00000042916	Rwdd1	192.347113	-0.154806623	0.106305653
ENSRNOG00000022274	Slc2a8	460.7750178	0.105271285	0.072323771
ENSRNOG00000007006	Slc3a1	1328.398331	-0.075543966	0.051768746
ENSRNOG00000008301	Tagln2	203.5899161	0.166584532	0.114057114
ENSRNOG00000017193	Lingo1	1720.567784	-0.112854862	0.077619864
ENSRNOG00000015191	Phc1	538.0894502	-0.095749887	0.065880591
ENSRNOG00000015670	Stx7	549.9801861	-0.100862961	0.069460862
ENSRNOG00000046333	AABR07007032.1	241.5201006	0.132983537	0.091792476
ENSRNOG00000004589	Galnt16	577.1576571	-0.142643804	0.098440203
ENSRNOG00000053537	NA	890.8916102	-0.091680362	0.063236032
ENSRNOG00000058589	NA	54556.34156	-0.121737804	0.084047192
ENSRNOG00000031269	RGD1304694	303.6457261	0.132514986	0.091331455
ENSRNOG00000040136	Stpg4	6395.606854	-0.064592169	0.044607665
ENSRNOG00000013141	Eno2	7805.070842	-0.088905743	0.061488638
ENSRNOG00000046120	NA	352.9150711	-0.197352624	0.136618605
ENSRNOG00000049025	Rps6	610.9712127	-0.107216408	0.074209375
ENSRNOG00000006545	Septin7	828.419433	-0.101015682	0.069840499
ENSRNOG00000029134	Adgrl1	1177.545535	0.086507808	0.060153651
ENSRNOG00000028743	Gal3st3	318.5959832	-0.134120635	0.093167098
ENSRNOG00000014525	Hspd1	347.6094735	0.179398077	0.124499233
ENSRNOG00000058914	Rbx1	194.0097627	-0.173560729	0.120642812
ENSRNOG00000017445	Tubb2b	1304.39553	-0.081912585	0.056939966
ENSRNOG00000009729	Ubp1	224.4565166	0.156828528	0.109015285
ENSRNOG00000001211	Gatd3a	587.8434966	-0.104879771	0.07316512
ENSRNOG00000033517	NA	867.944258	-0.366965773	0.256089453
ENSRNOG00000001783	Tra2b	293.0857139	-0.141852136	0.099051829
ENSRNOG00000010326	Col20a1	374.4694593	-0.122641829	0.08571736
ENSRNOG00000018761	Eif3c	626.7941116	0.18682398	0.130635084
ENSRNOG00000061943	NA	491.5447584	-0.145992526	0.102210991
ENSRNOG00000013579	NA	642.5758282	-0.117159616	0.082036992

ENSRNOG00000007808	Nap1l5	1773.540869	-0.077402851	0.054191368
ENSRNOG00000034254	Actb	2592.688374	-0.077835744	0.054636595
ENSRNOG00000012019	B3galnt1	309.9721819	-0.181239541	0.127360741
ENSRNOG00000017817	Cox4i1	3470.658018	-0.083067289	0.058377891
ENSRNOG00000015128	Sae1	521.8769958	-0.147343718	0.103533481
ENSRNOG00000015332	Thoc1	272.6887766	-0.12903985	0.090623079
ENSRNOG00000005736	NA	388.0197768	-0.118689734	0.083497039
ENSRNOG00000045665	Amigo1	280.9556527	-0.129955001	0.091885472
ENSRNOG00000016047	Macf1	362.8224902	0.123236115	0.087213247
ENSRNOG00000008163	NA	905.1760992	-0.087333371	0.061699732
ENSRNOG00000046950	NA	296.6540363	0.110035776	0.07778489
ENSRNOG00000018322	Picalm	416.4535303	0.113428741	0.080249207
ENSRNOG00000050401	NA	368.6256503	-0.129265972	0.091642563
ENSRNOG00000039091	Pnpla8	479.3796259	0.101471794	0.07194427
ENSRNOG00000029178	Abcc5	306.4822431	0.158356507	0.112358335
ENSRNOG00000006001	Luc7l2	640.0980224	0.112433575	0.080063115
ENSRNOG00000002563	Mcts1	266.8387291	-0.128959713	0.091745489
ENSRNOG00000000527	Mtch1	951.7356128	-0.146374823	0.104356477
ENSRNOG00000057292	NA	390.0717034	-0.120809329	0.08577053
ENSRNOG00000018653	Pfdn1	425.2554392	0.111077969	0.079194524
ENSRNOG00000015912	Ptdss2	304.7090452	-0.11828706	0.083999843
ENSRNOG00000013035	Rab33b	195.6749893	-0.169561829	0.120653507
ENSRNOG00000038436	RGD1307100	287.2618291	0.114896936	0.081785968
ENSRNOG00000038228	Serp2	1160.049593	-0.085068851	0.060559467
ENSRNOG00000018960	Syne1	623.9553009	0.100679918	0.071583483
ENSRNOG00000034075	Ube2ql1	2035.224465	-0.10354178	0.073825381
ENSRNOG00000047796	NA	595.4339011	-0.094736703	0.067612653
ENSRNOG00000055583	Ttyh3	843.1335092	0.087710895	0.062608605
ENSRNOG00000030089	NA	204.9254768	-0.141883113	0.101401171
ENSRNOG00000006992	NA	3693.731529	-0.061446681	0.043922049
ENSRNOG00000011421	Smap2	952.9331225	-0.089514086	0.063959648
ENSRNOG00000018691	Aurkaip1	324.0802119	-0.14561414	0.104264044
ENSRNOG00000020675	Ccdc97	268.5864903	-0.122549088	0.087705581
ENSRNOG00000061215	Crym	711.2235384	-0.247044202	0.17752039
ENSRNOG00000036798	Dusp3	310.8105258	0.144678248	0.103751091
ENSRNOG00000027690	Eif3el1	541.5757936	-0.101227646	0.072736283
ENSRNOG00000019428	Higd1a	495.8562956	-0.10522046	0.075546745
ENSRNOG00000059069	NA	864.4501881	-0.184203567	0.132040232
ENSRNOG00000057193	NA	371.2650385	0.134996763	0.096835693
ENSRNOG00000029592	Srsf11	239.6155215	-0.15092126	0.108365288
ENSRNOG00000019318	Syt3	315.0183043	-0.120303281	0.086452873
ENSRNOG00000009990	Zranb2	358.7266279	0.114038591	0.081728066
ENSRNOG00000015192	Mrps5	214.6115773	-0.122126018	0.087817781
ENSRNOG00000033625	AABR07027015.1	352.9326069	-0.143934051	0.103635734
ENSRNOG00000055777	AABR07064980.1	217.1556386	-0.130618429	0.094132057
ENSRNOG00000003988	Dnajc27	460.5928056	0.115015224	0.082874403
ENSRNOG00000023643	Mmp17	195.7092715	0.191622972	0.138145237
ENSRNOG00000004464	Sel1l	402.6116265	0.133314078	0.09598518
ENSRNOG00000018454	Apoe	531.5744568	-0.102107635	0.073814552
ENSRNOG00000004364	Babam2	249.4706588	-0.133205016	0.096262587

ENSRNOG00000004077	Kcnc2	264.7073547	0.146052612	0.105512293
ENSRNOG00000012415	Mpc1	955.6822076	-0.078479743	0.056743018
ENSRNOG00000032439	Rsl1d1	311.8236555	-0.129528102	0.093477181
ENSRNOG00000019207	Shank1	1020.925328	-0.176282095	0.127477997
ENSRNOG00000052506	Ak3	317.1562594	-0.122914273	0.088979525
ENSRNOG00000045931	Sik3	397.6887731	-0.102600516	0.074262048
ENSRNOG00000032585	AABR07048992.1	10576.70078	-0.221380989	0.160696793
ENSRNOG00000010551	Lhx2	372.5162622	0.15802761	0.114612569
ENSRNOG00000031383	NA	5121.190398	-0.09160104	0.066534052
ENSRNOG00000018289	Park7	588.7807084	-0.121081653	0.087764869
ENSRNOG00000030124	Ptpn11	551.9892537	-0.100607647	0.073069009
ENSRNOG00000017091	Slc25a46	363.324284	-0.109883806	0.079700482
ENSRNOG00000003139	Smc1a	256.9124112	-0.14534977	0.10556471
ENSRNOG00000020851	Aplp1	6502.93404	-0.068029601	0.049463229
ENSRNOG00000037984	NA	708.0680544	-0.107726951	0.078352019
ENSRNOG00000013059	Aste1	557.3263475	-0.098002013	0.071403725
ENSRNOG00000012749	C1qb	258.848875	-0.156078996	0.114115041
ENSRNOG00000013654	Cbln2	285.742685	-0.128285311	0.093554492
ENSRNOG00000017895	Eno1	7085.02298	-0.093671512	0.068352359
ENSRNOG00000019272	Gkap1	234.8168156	-0.139809795	0.102149205
ENSRNOG00000002339	Mark1	367.6023247	0.118824647	0.08682277
ENSRNOG00000001136	Pebp1	3058.218528	-0.077178065	0.056415022
ENSRNOG00000008465	Tmem176b	501.2868201	0.176200523	0.128629989
ENSRNOG00000005623	Ankmy2	438.7130024	-0.119329219	0.087360257
ENSRNOG00000001689	Arl6	338.9952998	-0.122046307	0.089666546
ENSRNOG00000048726	Clec2d	194.1268939	0.182491463	0.134006648
ENSRNOG00000022727	NA	387.1739418	0.113228785	0.083063498
ENSRNOG00000046109	NA	232.1008966	-0.124127103	0.091065943
ENSRNOG00000024953	NA	204.1931837	-0.153315034	0.112741448
ENSRNOG00000008810	Nsmf	3683.268721	0.100691281	0.073762537
ENSRNOG00000001245	Pcbp3	342.1062428	-0.139060753	0.102242126
ENSRNOG00000019560	Pde2a	1349.853799	0.084991984	0.062303772
ENSRNOG00000008943	Penk	695.4497281	-0.14191411	0.104123568
ENSRNOG00000021042	Psmd4	349.7520419	-0.117334489	0.086085661
ENSRNOG00000010488	Zmiz1	485.4076542	-0.148835244	0.109440197
ENSRNOG00000011384	Aimp1	277.0326954	-0.132438394	0.097521342
ENSRNOG00000011621	Hnrnpc	533.1544001	-0.10183243	0.075041076
ENSRNOG00000031273	AABR07034639.1	394.6527329	-0.115043221	0.084977697
ENSRNOG00000008374	Nipsnap1	367.0878796	-0.126664515	0.093488169
ENSRNOG00000000954	Polr1d	494.339373	-0.110224151	0.081406493
ENSRNOG00000049382	RGD1563905	238.5577253	-0.135454027	0.100079554
ENSRNOG00000000463	Col11a2	253.7824891	0.152773251	0.113017956
ENSRNOG00000046280	Tceal5	297.1295428	-0.153171612	0.113412625
ENSRNOG00000018183	Ubr4	270.9008459	0.139510079	0.103274052
ENSRNOG00000046349	NA	1718.204884	-0.08334366	0.061750253
ENSRNOG00000018823	Nisch	217.8658911	0.127020482	0.094165723
ENSRNOG00000004322	Sh3kbp1	218.9575585	0.131226478	0.097402207
ENSRNOG00000027882	NA	893.0343468	-0.12112669	0.089948294
ENSRNOG00000008411	Sec23b	1650.939108	-0.071293884	0.052981606
ENSRNOG00000047276	NA	4822.519244	-0.085791908	0.063839812

ENSRNOG00000015461	Serpine2	682.0886542	0.094121062	0.070029813
ENSRNOG00000017510	Mfge8	1020.670874	-0.105715625	0.078824
ENSRNOG00000047185	Eif3i	712.8996517	-0.08197032	0.061199022
ENSRNOG00000003147	Sqstm1	285.1852091	0.135928177	0.101494145
ENSRNOG00000055226	Dab2ip	379.3365255	0.116734009	0.087210484
ENSRNOG00000002229	Adcy5	240.0100659	0.122623611	0.091772014
ENSRNOG00000042492	Cop1	302.4296391	0.130026242	0.097277656
ENSRNOG00000011560	Mtmr9	410.6173388	-0.110978317	0.083013921
ENSRNOG00000054603	Pacsin1	884.587528	0.13807456	0.103382591
ENSRNOG00000012550	Uqcrh	1090.164429	-0.095796978	0.071788257
ENSRNOG00000027787	Cdc6	253.6001975	0.117086923	0.087924251
ENSRNOG00000013596	Dimt1	427.9062954	0.109240289	0.082061749
ENSRNOG00000002079	Mapk10	582.7242954	-0.117163845	0.088065961
ENSRNOG00000004382	Arel1	327.913576	0.115505344	0.087102874
ENSRNOG00000010737	Mbnl2	928.4999113	0.089956424	0.067883478
ENSRNOG00000008431	Gabbr2	2687.944823	-0.123795941	0.093567659
ENSRNOG00000002896	NA	936.6876274	-0.091596783	0.069232409
ENSRNOG00000012349	Tlnrd1	204.5166345	0.145744451	0.110097206
ENSRNOG00000016180	Pdp1	256.2553665	-0.148362066	0.112216249
ENSRNOG00000008056	Ankrd9	289.5197741	0.121553373	0.092035304
ENSRNOG00000017108	Syngr1	850.46035	-0.260761693	0.197438093
ENSRNOG00000018042	Mrpl48	200.4063652	0.152678559	0.11567785
ENSRNOG00000016727	Akr1a1	2889.984167	-0.087285878	0.066193909
ENSRNOG00000003205	Ldb2	576.6055271	-0.104864817	0.079616795
ENSRNOG00000050437	Bod1l1	221.8486485	-0.15703774	0.11948645
ENSRNOG00000004996	Zcrb1	436.5949605	-0.097893076	0.074464773
ENSRNOG00000005561	Brinp1	657.8872734	-0.11552424	0.088101052
ENSRNOG00000054140	Myl6	490.7202935	-0.114573627	0.087358698
ENSRNOG00000057470	Pla2g12a	236.238217	-0.125438795	0.095621426
ENSRNOG00000052429	Stard3nl	470.092809	-0.094148821	0.071724966
ENSRNOG00000033843	AABR07007121.1	982.8580344	-0.073470835	0.05609879
ENSRNOG00000011020	Eif3l	526.4908983	-0.098466262	0.075209924
ENSRNOG00000040153	NA	1054.484771	-0.098628603	0.075437386
ENSRNOG00000018337	Caly	2270.30546	-0.087762672	0.067201845
ENSRNOG00000014051	Pgrmc2	239.9962797	0.121911426	0.093375601
ENSRNOG00000003116	Dph1	242.4523189	-0.346660732	0.265971554
ENSRNOG00000056183	AABR07030781.1	2716.791687	-0.086283111	0.066350574
ENSRNOG00000010753	Aig1	559.4681908	-0.106828226	0.082138741
ENSRNOG00000030963	NA	14258.73441	-0.067355872	0.051805821
ENSRNOG00000019645	Osbp2	299.6797387	0.142206501	0.109292282
ENSRNOG00000050794	Pdlim4	328.1359331	-0.166922367	0.128425442
ENSRNOG00000013162	Zcchc12	860.5303327	-0.118371617	0.091037835
ENSRNOG00000038909	Pou3f3	290.9323739	0.119778216	0.092262697
ENSRNOG00000000054	Abhd8	2614.106271	0.108454156	0.084479064
ENSRNOG00000016610	Arhgap1	488.2419439	-0.082351997	0.0634907
ENSRNOG00000006818	Arl6ip5	479.5845648	-0.112933155	0.087482095
ENSRNOG00000011150	Arsb	545.3652769	0.095215118	0.074196943
ENSRNOG00000017737	Dgkz	534.3097733	0.398963235	0.310363613
ENSRNOG00000018526	Dlg4	1050.113884	0.128538507	0.099957213
ENSRNOG00000025791	Dync1li2	572.6563536	0.093465601	0.072243461

ENSRNOG00000057817	Epb41l1	595.2530656	-0.126810067	0.098745437
ENSRNOG00000024886	Ext1	206.7563315	-0.17415236	0.134367778
ENSRNOG00000003890	Nap1l1	546.2621281	-0.102397605	0.079492076
ENSRNOG00000001182	Ndufv3	415.9731592	-0.121518794	0.093972566
ENSRNOG00000061176	Nudt3	2512.627931	-0.102615353	0.079945102
ENSRNOG00000055790	Nup210l	537.0831404	-0.097516188	0.07525788
ENSRNOG00000028648	Olig1	600.5400077	0.111022055	0.086499136
ENSRNOG00000009965	Pih1d2	387.5135411	0.094707055	0.073190346
ENSRNOG00000013368	Plcl2	303.2076235	-0.132266847	0.102965624
ENSRNOG00000007114	Psma6	253.2142151	-0.124849849	0.097161996
ENSRNOG00000002944	RGD1304587	3808.990828	-0.058115838	0.045141892
ENSRNOG00000011952	Samm50	478.5330061	-0.090923488	0.070221333
ENSRNOG00000020412	Sf3b2	383.3994187	-0.153263098	0.119035746
ENSRNOG00000006871	Slc25a14	198.599871	-0.128071334	0.099236994
ENSRNOG00000011078	Srm	320.8311394	-0.117341384	0.090966126
ENSRNOG00000012594	Sugt1	437.6666397	-0.095100086	0.074050576
ENSRNOG00000003229	Tspan7	7742.00855	-0.061921247	0.047989717
ENSRNOG00000018395	Usp40	284.5550608	0.119326341	0.092153604
ENSRNOG00000001004	Ncor2	385.6177073	0.111835986	0.08725713
ENSRNOG00000050624	Lysmd4	287.3306799	-0.126193153	0.098507701
ENSRNOG00000018448	NA	2106.476583	-0.113840905	0.08890304
ENSRNOG00000014698	Chrnb1	508.04114	0.104087217	0.08135068
ENSRNOG00000018671	Commd4	424.3762859	0.100016417	0.078338583
ENSRNOG00000027498	Engase	852.2790523	-0.097655101	0.076498377
ENSRNOG00000016611	Hras	1796.64843	-0.080617682	0.063084552
ENSRNOG00000055246	Ncor1	483.7313039	0.118858098	0.092998673
ENSRNOG00000011785	Xrn2	220.0530923	0.114857962	0.08999101
ENSRNOG00000018164	Spcs2	501.6034398	-0.099731374	0.078275501
ENSRNOG00000016936	Nadk	1559.775955	-0.081384745	0.063938062
ENSRNOG00000018958	Mt3	4963.276072	-0.092243531	0.072571418
ENSRNOG00000020140	Pigq	420.8190088	0.112081621	0.088223171
ENSRNOG00000057116	Pex19	296.2962594	-0.107816028	0.08492542
ENSRNOG00000061004	Asphd2	442.5944017	0.093220536	0.073666512
ENSRNOG00000008921	Dynll2	1410.268233	-0.100956763	0.079744452
ENSRNOG00000005285	Fbxo33	231.0916512	-0.128682992	0.101578748
ENSRNOG00000002449	Maged2	200.3471793	-0.134123949	0.10598844
ENSRNOG00000014508	Mgll	209.3141151	-0.151026429	0.119246806
ENSRNOG00000051650	Ywhaq	2322.057207	-0.063282826	0.050035423
ENSRNOG00000002553	NA	370.8160284	-0.099063582	0.078428086
ENSRNOG00000010472	Ccdc32	529.4841228	-0.130688676	0.103538035
ENSRNOG00000011684	Auh	297.0929908	-0.142894974	0.113543648
ENSRNOG00000028946	Cdc42ep4	277.2312827	0.125179194	0.099425504
ENSRNOG00000009773	Elovl4	314.6463305	0.132903867	0.105845542
ENSRNOG00000032866	AABR07035366.1	264.844302	-0.115661181	0.092165555
ENSRNOG00000022116	Gjb6	224.3565356	-0.124632034	0.09953692
ENSRNOG00000042983	Nudt21	367.2850542	-0.265128144	0.211601237
ENSRNOG00000015072	Ptgr1	197.0357911	-0.127286886	0.101590866
ENSRNOG00000012806	Rbbp6	404.0740496	-0.105415425	0.084188633
ENSRNOG00000006077	Snx5	516.6509711	-0.092441393	0.073862333
ENSRNOG00000003741	Nptx1	688.7166852	0.131235087	0.104927566

ENSRNOG00000003261	Usp9x	468.9613897	0.104512353	0.083635076
ENSRNOG00000001479	Gtf2i	768.9975243	0.09592398	0.076840152
ENSRNOG00000050104	Clip3	479.8996288	0.366885408	0.294217369
ENSRNOG00000013589	Cxcl12	691.6516864	0.102993438	0.082894498
ENSRNOG00000031802	NA	229.8506604	-0.11892226	0.095727987
ENSRNOG00000029280	Gpaa1	299.8810194	-0.107718358	0.086753794
ENSRNOG00000029813	AABR07035218.1	1407.622151	-0.095999727	0.077442494
ENSRNOG00000010474	Hmces	771.1261336	-0.112998479	0.091273317
ENSRNOG00000010427	Ipo7	199.1919397	-0.160072173	0.129358579
ENSRNOG00000025179	NA	264.4836755	-0.152295355	0.123057322
ENSRNOG00000019862	Pinlyp	249.4140823	0.133389677	0.107772607
ENSRNOG00000055917	Trim27	322.3977199	-0.110424361	0.089039358
ENSRNOG00000058136	Vom1r90	236.298819	0.125991847	0.101925146
ENSRNOG00000021263	NA	198.8358165	-0.121995155	0.098795504
ENSRNOG00000015425	Ssx2ip	257.4727828	-0.129407966	0.104829873
ENSRNOG00000015692	Taok1	461.2915466	-0.475062971	0.385466148
ENSRNOG00000029049	Zdhhc14	253.3168603	0.154200088	0.125156977
ENSRNOG00000011216	Tbl1xr1	194.2585905	0.132320045	0.107553302
ENSRNOG00000003626	Atp5pd	1006.858041	-0.072836656	0.059357524
ENSRNOG00000032922	Dclk1	1401.070526	0.088235246	0.071958944
ENSRNOG00000030596	NA	204.4060228	-0.139771959	0.113935185
ENSRNOG00000011334	Tmem63c	194.2979048	0.14884988	0.121417894
ENSRNOG00000005256	Zc3h15	723.2013271	-0.078325765	0.063792945
ENSRNOG00000015420	Stxbp1	323.6575744	0.57159815	0.467385123
ENSRNOG00000025244	Trrap	251.1673087	0.137073796	0.112156241
ENSRNOG00000010668	Anxa6	353.629239	0.141807007	0.116174225
ENSRNOG00000019253	Bcar1	248.6692043	-0.118725657	0.097369503
ENSRNOG00000018366	Cracdl	655.6613416	-0.118896303	0.097503131
ENSRNOG00000031730	Eloc	255.6356878	0.114962017	0.094278788
ENSRNOG00000001989	Alcam	214.8810178	0.142883986	0.11759283
ENSRNOG00000014289	Arpc2	1412.6372	-0.064320173	0.053209612
ENSRNOG00000001304	Bcr	235.2335069	-0.512024699	0.422697159
ENSRNOG00000015465	Cep72	1359.760528	0.078706413	0.064919238
ENSRNOG00000033064	Edil3	360.1185377	-0.105384563	0.087256207
ENSRNOG00000033765	Eif1	5248.727947	-0.055168201	0.045487001
ENSRNOG00000049221	Kdm4b	198.0992499	-0.125375224	0.10393103
ENSRNOG00000047450	Lmo3	455.3772494	-0.093490471	0.077332002
ENSRNOG00000022490	NA	451.0783928	-0.086312526	0.071246885
ENSRNOG00000036689	P4hb	581.1857221	0.081083025	0.067026031
ENSRNOG00000009570	Pdxp	1932.00787	-0.090496735	0.074580451
ENSRNOG00000011282	Ppp2r1a	3017.894085	-0.070974742	0.058475472
ENSRNOG00000001273	Psmg3	327.79381	0.196256753	0.16179843
ENSRNOG00000006180	Pum2	745.4726708	0.088762895	0.073494991
ENSRNOG00000011302	Rab11a	769.9699878	-0.083454741	0.069242601
ENSRNOG00000016220	Rpl12	1556.54155	-0.117736069	0.097047895
ENSRNOG00000026716	Rpl9	2146.121439	-0.080083729	0.06585369
ENSRNOG00000013236	Samd8	509.2077379	-0.08567792	0.070501302
ENSRNOG00000018412	Sfi1	197.3265455	0.14911368	0.123577204
ENSRNOG00000014287	Stk11	1685.047919	-0.098344168	0.081602143
ENSRNOG00000030625	Tf	1002.826073	-0.152156257	0.126215456

ENSRNOG00000028498	Wdr1	962.8300529	-0.073701704	0.060933949
ENSRNOG00000061963	NA	299.2592164	-0.12538024	0.104201931
ENSRNOG00000012366	Mesd	370.5048581	-0.093860862	0.078212998
ENSRNOG00000002387	NA	466.1588381	-0.101241797	0.084225278
ENSRNOG00000057601	NA	254.5408085	0.122760223	0.102318103
ENSRNOG00000023095	Npepps	919.4226124	0.069661791	0.058049177
ENSRNOG00000021206	Plaat3	198.0926458	-0.121983469	0.101608278
ENSRNOG00000007040	Timm17a	459.95255	-0.094099905	0.078412531
ENSRNOG00000033057	AABR07025010.1	10107.94689	-0.062571553	0.052521723
ENSRNOG00000038610	AABR07034445.1	5958.687103	-0.060923104	0.05114193
ENSRNOG00000048273	Apod	448.012205	-0.132256869	0.110512719
ENSRNOG00000061304	Atp2b3	289.3910894	-0.125365564	0.105044568
ENSRNOG00000004206	Glrx5	362.625811	-0.100399581	0.083992769
ENSRNOG00000009539	Khdrbs3	1903.517352	-0.07067988	0.059207944
ENSRNOG00000037220	NA	435.0613852	-0.096256242	0.080417553
ENSRNOG00000054031	NA	205.3300824	0.135432843	0.113626479
ENSRNOG00000010060	Panx1	270.6579756	-0.107093287	0.089716028
ENSRNOG00000051490	Plcg1	368.7049817	0.09609329	0.080150977
ENSRNOG00000006858	Pyurf	268.9050514	0.142588116	0.118976763
ENSRNOG00000031789	Rangap1	1050.208697	-0.11222475	0.094084661
ENSRNOG00000000520	Srsf3	375.9646642	-0.090054306	0.075323537
ENSRNOG00000019383	Tef	851.6481047	-0.092295012	0.0773789
ENSRNOG00000018281	Uqcrfs1	1201.269556	0.074526087	0.062360253
ENSRNOG00000019400	Dag1	432.3615552	0.088357283	0.07424606
ENSRNOG00000046005	Scd2	4079.387937	0.082421532	0.069317452
ENSRNOG00000001351	Trafd1	489.1452213	0.106475231	0.089559307
ENSRNOG00000009271	Smarca4	563.8950261	0.095158187	0.080146357
ENSRNOG00000047933	Phykpl	360.6478847	-0.121809482	0.102761707
ENSRNOG00000002125	Ccni	1731.040025	0.067309675	0.056929078
ENSRNOG00000005061	Dlst	215.1880041	0.15265449	0.128982834
ENSRNOG00000010966	Itgb1	291.7108066	0.121456907	0.10260648
ENSRNOG00000000395	Kifbp	726.2606926	-0.077048727	0.065066503
ENSRNOG00000000546	Nt5dc1	1073.51202	-0.082578714	0.069862361
ENSRNOG00000012148	Trio	289.9894912	-0.148237671	0.12533973
ENSRNOG00000014375	Adgrb2	1301.005396	0.067604423	0.057222712
ENSRNOG00000010330	Mark3	354.7262498	0.098086092	0.083107459
ENSRNOG00000003228	NA	273.9821	0.096524324	0.082015913
ENSRNOG00000003863	Trir	401.1756192	-0.088946259	0.075633528
ENSRNOG00000004448	Acss3	226.5505814	-0.116935722	0.100016795
ENSRNOG00000015514	Bcat1	4996.738981	-0.063606569	0.054436829
ENSRNOG00000027623	D5Ertd579e	213.1466143	0.126748291	0.108104848
ENSRNOG00000038970	Fgd1	257.2370632	0.113202514	0.096904705
ENSRNOG00000059381	Mrps6	355.0146179	-0.110363179	0.094403877
ENSRNOG00000017446	Ndufs8	222.2335666	-0.494808586	0.421792933
ENSRNOG00000006235	Nell2	5027.838994	-0.078007155	0.066595513
ENSRNOG00000010068	Plpp7	191.9483558	-0.129931367	0.111246683
ENSRNOG00000061795	Rbm4b	807.7921958	0.097298679	0.083343416
ENSRNOG00000001064	Sbno1	292.672771	0.095904544	0.08200872
ENSRNOG00000010248	Serpini1	1015.28795	0.074349636	0.063546595
ENSRNOG00000016423	Tacc1	652.1881229	0.085818179	0.073517298

ENSRNOG00000012477	NA	1578.516359	-0.065560125	0.056210014
ENSRNOG00000036687	Alyref	287.2401752	-0.106334412	0.091258962
ENSRNOG00000022234	mrpl24	424.7261933	-0.119455923	0.102698407
ENSRNOG00000005355	Ncstn	370.7321675	-0.098297891	0.084489002
ENSRNOG00000019018	Plat	221.5089732	0.18709497	0.160719577
ENSRNOG00000009112	Pon2	484.8335766	0.09516689	0.081924881
ENSRNOG00000042320	Slc41a1	277.8070503	0.105378086	0.090694379
ENSRNOG00000001436	Ywhag	5200.103654	0.06183239	0.053212232
ENSRNOG00000003887	NA	206.7042809	-0.161663999	0.139318028
ENSRNOG00000048932	NA	790.5659688	0.082167312	0.070882102
ENSRNOG00000046955	Cbx6	1579.704478	0.057702782	0.049898676
ENSRNOG00000011394	Kif3c	333.6502492	-0.097254379	0.0841181
ENSRNOG00000005464	Lgalsl	375.6000943	0.097067063	0.083984958
ENSRNOG00000056882	U6	250.4845153	0.10069566	0.087077771
ENSRNOG00000033979	Gm5471	669.1714969	-0.077668833	0.067268632
ENSRNOG00000005299	Kif5a	2660.09499	0.083545015	0.07238833
ENSRNOG00000012255	Ktn1	221.7613625	-0.131992409	0.114410045
ENSRNOG00000004026	Atp2b1	1021.466531	-0.073097387	0.063520934
ENSRNOG00000007478	Cry2	527.4997506	0.077331877	0.06709056
ENSRNOG00000015097	Kcmf1	623.5487077	0.084905613	0.073838286
ENSRNOG00000009715	Me1	451.4832318	0.095402289	0.082929313
ENSRNOG00000016390	NA	348.5276071	-0.111908686	0.097142258
ENSRNOG00000003148	NA	746.724564	-0.079426769	0.069069789
ENSRNOG00000052664	Tnks2	488.9873344	0.083926437	0.072859007
ENSRNOG00000048982	NA	222.9747528	0.105040051	0.091437171
ENSRNOG00000048704	AABR07032392.1	2506.061129	-0.059567535	0.051948805
ENSRNOG00000019223	Taf3	2293.097797	-0.059056455	0.051463637
ENSRNOG00000009088	Txnrd1	218.0265297	-0.10772973	0.093936792
ENSRNOG00000018343	Isca1	1542.426164	0.076637357	0.066873149
ENSRNOG00000010642	Lysmd2	730.8954797	-0.085762361	0.075050766
ENSRNOG00000029778	Maob	249.255274	-0.117590107	0.102912768
ENSRNOG00000056028	NA	219.7038212	-0.108718867	0.095115743
ENSRNOG00000008656	Snca	4087.93151	-0.100148546	0.087559199
ENSRNOG00000013816	Cmas	457.9155728	-0.092969437	0.081443305
ENSRNOG00000007887	Elk4	243.8636097	0.104668165	0.091950965
ENSRNOG00000018007	Gtf2h5	228.0608525	-0.118881716	0.104261192
ENSRNOG00000002544	Kifap3	1396.268392	-0.067685668	0.059423101
ENSRNOG00000004925	Ppp1r12a	495.511336	0.093484865	0.082160137
ENSRNOG00000011771	Ptp4a1	618.5084876	0.089021808	0.078150926
ENSRNOG00000009523	Rab11fip2	216.6339369	-0.134545451	0.118160187
ENSRNOG00000031173	Snd1	254.9469191	-0.111397653	0.097786116
ENSRNOG00000010816	Kcnk15	403.0556507	0.095792749	0.084263179
ENSRNOG00000031997	Ttll7	324.508699	0.112965524	0.099462389
ENSRNOG00000016670	AABR07028769.1	400.5121084	-0.131776545	0.116159765
ENSRNOG00000004430	Cep131	218.2763303	-0.117859895	0.103883041
ENSRNOG00000006204	Slc30a3	878.1399141	0.07930584	0.069931454
ENSRNOG00000062272	NA	1043.815735	-0.066133704	0.058393338
ENSRNOG00000022066	Fam117b	318.666183	-0.090470238	0.079948917
ENSRNOG00000005438	Pcsk2	1371.15823	0.06723936	0.059494809
ENSRNOG00000052273	RGD1305713	200.4302714	-0.108959694	0.096409352

ENSRNOG00000039871	RGD1306063	252.1522632	-0.102209137	0.090354488
ENSRNOG00000010691	Cmtm3	463.1938126	0.092333109	0.08185395
ENSRNOG00000007410	Dab1	270.6524256	-0.113102183	0.100177473
ENSRNOG00000018781	Map1s	417.0576519	-0.08490424	0.07523403
ENSRNOG00000050240	NA	193.7057867	0.116414198	0.103217857
ENSRNOG00000016847	Bace1	312.1168448	0.095737261	0.085007346
ENSRNOG00000015894	Dock8	344.0294395	-0.111170212	0.098705515
ENSRNOG00000059579	Gpt2	337.5782923	-0.099221212	0.088043258
ENSRNOG00000022482	Trappc11	203.0576249	0.105202733	0.093457194
ENSRNOG00000001044	Aimp2	260.1539765	-0.10219705	0.091362382
ENSRNOG00000028247	Ankrd13d	1351.203424	0.064917767	0.057769748
ENSRNOG00000046918	Apoo	304.7120472	0.118713498	0.10598064
ENSRNOG00000015205	Cyb5a	375.0107593	-0.083858138	0.074545278
ENSRNOG00000007570	Dera	198.0115725	-0.117231182	0.104365483
ENSRNOG00000027860	Ggnbp2	256.2910246	-0.12623842	0.112374291
ENSRNOG00000017283	Kcnt1	200.5093285	0.111447777	0.099557272
ENSRNOG00000010545	Mrap2	193.9408341	-0.110391741	0.09838481
ENSRNOG00000010540	Mrpl45	301.7270405	-0.096469656	0.086124814
ENSRNOG00000004068	Ncoa1	559.4532339	0.074416051	0.066557533
ENSRNOG00000019459	Oaz1	1033.205006	-0.074253441	0.066276962
ENSRNOG00000014485	Pdcd6	375.922884	0.099527024	0.088767609
ENSRNOG00000052787	Rsl24d1	196.8002347	-0.106522782	0.095230805
ENSRNOG00000002722	Sec14l1	743.5719213	0.066157816	0.059196482
ENSRNOG00000045860	U2af1	289.4477227	-0.163582267	0.146158996
ENSRNOG00000001706	Kalrn	231.3859761	0.13852268	0.124183141
ENSRNOG00000013728	Polg2	1209.291008	-0.077013477	0.069005338
ENSRNOG00000004477	Ublcp1	267.5689859	0.10158165	0.091072466
ENSRNOG00000009550	Sqle	220.8631988	0.105657899	0.094826822
ENSRNOG00000009184	Foxp1	249.4459241	-0.115753835	0.104019577
ENSRNOG00000007739	Rars1	224.4625987	-0.102370397	0.092009025
ENSRNOG00000021438	Tuba1c	1055.858243	-0.081481301	0.073413851
ENSRNOG00000046214	Cyp27b1	278.9622987	0.103448151	0.093393933
ENSRNOG00000000981	Scarb1	295.0514865	0.125103927	0.11290896
ENSRNOG00000042879	Cep192	264.6802823	-0.095561211	0.086478196
ENSRNOG00000011663	Rnf7	424.7553484	-0.086863033	0.078717493
ENSRNOG00000004959	Actr2	350.850484	0.145669194	0.132094328
ENSRNOG00000005326	Cnrip1	909.4818587	-0.067378636	0.061366192
ENSRNOG00000002660	Magee1	409.7328302	0.08660854	0.078847088
ENSRNOG00000006403	Nectin1	215.575656	0.114503625	0.104253473
ENSRNOG00000007735	Pigm	351.2648338	0.101284657	0.092097471
ENSRNOG00000030888	Rela	340.7736459	-0.116159121	0.105625799
ENSRNOG00000021117	Rps6ka4	300.2963472	0.096462197	0.087615017
ENSRNOG00000002106	Usp46	618.1103034	0.071513041	0.065082984
ENSRNOG00000014520	Ing1	268.3297579	0.115196507	0.104981592
ENSRNOG00000011323	Lgi3	326.7476245	0.119236478	0.108791155
ENSRNOG00000004730	Meis2	420.1583986	-0.096504963	0.088102828
ENSRNOG00000016623	Tmeff2	619.4938577	-0.107263129	0.09788053
ENSRNOG00000018529	Csnk1g2	782.4454213	-0.069214751	0.063396435
ENSRNOG00000010452	Cycs	2170.578587	-0.079835865	0.07317337
ENSRNOG00000059865	Dnah12	3483.20953	-0.073749073	0.067578107

ENSRNOG00000046177	NA	420.7979458	0.352588951	0.322681105
ENSRNOG00000043645	NA	1655.758259	-0.05421255	0.049688452
ENSRNOG00000017612	Vps35	632.195159	0.082831191	0.075972166
ENSRNOG00000018434	Stab1	1292.64815	0.064787154	0.059464474
ENSRNOG00000004602	Rnf145	364.6464727	-0.092285014	0.084847304
ENSRNOG00000009530	Uchl3	380.9679462	-0.084351573	0.077630678
ENSRNOG00000015852	Arhgap35	539.9818773	0.083687018	0.077122019
ENSRNOG00000050630	AABR07070043.1	9975.10043	-0.057007156	0.052733214
ENSRNOG00000047179	Aplp2	1246.832105	0.060701592	0.05619321
ENSRNOG00000010239	Cnbp	1855.594416	-0.071549498	0.066185261
ENSRNOG00000009090	Dad1	759.5109825	-0.072716119	0.067138003
ENSRNOG00000009054	Elmod1	1809.676947	-0.065832545	0.060899747
ENSRNOG00000055597	Hdac7	214.1966343	0.120926707	0.111683533
ENSRNOG00000029418	NA	235.5591148	-0.107650477	0.099615971
ENSRNOG00000013195	Ruvbl1	226.348745	-0.103767294	0.095909431
ENSRNOG00000011294	Usp33	335.9465005	0.087338167	0.080586164
ENSRNOG00000033100	AABR07054189.1	1658.816056	-0.080136565	0.074875135
ENSRNOG00000018317	Aak1	842.1480984	0.084326057	0.080194842
ENSRNOG00000029370	Abhd3	706.2005948	0.078345968	0.074046775
ENSRNOG00000055942	AC120310.1	804.1908245	-0.07912538	0.075260056
ENSRNOG00000020205	Agrn	280.5494477	0.09812102	0.093179745
ENSRNOG00000013969	Ahi1	195.7802218	0.128227968	0.119295126
ENSRNOG00000018118	Atad3a	335.7736882	-0.094498374	0.088951583
ENSRNOG00000003031	Atp2b4	977.4413147	0.084067965	0.079844082
ENSRNOG00000007049	Bcl11a	224.2152675	-0.110787236	0.103693517
ENSRNOG00000005776	Bcl11b	293.3626017	-0.112190754	0.104223543
ENSRNOG00000015447	Calcoco1	224.5520666	0.094613277	0.089717172
ENSRNOG00000009687	Ccdc120	618.890033	-0.072785565	0.067514456
ENSRNOG00000018999	Cdc14b	870.5405074	-0.071109957	0.067024057
ENSRNOG00000002152	Dcun1d4	195.7387897	0.118281508	0.110955783
ENSRNOG00000030547	Dpp6	723.6680119	0.067572844	0.064057585
ENSRNOG00000001126	Fbxw8	324.0659538	-0.11362931	0.106123314
ENSRNOG00000005850	Gdap1	232.2581248	-0.104370473	0.097004001
ENSRNOG00000029038	Gemin8	223.8879203	-0.123272241	0.117089042
ENSRNOG00000008897	Gga1	377.4142021	0.085511987	0.080526875
ENSRNOG00000010731	Gpm6a	6512.900119	-0.063241611	0.059263263
ENSRNOG00000011293	Hpf1	395.1992802	-0.114618687	0.10817623
ENSRNOG00000019960	Ino80e	650.750092	-0.074191364	0.07024939
ENSRNOG00000018784	Jph3	1100.858017	-0.064927416	0.061061713
ENSRNOG00000006756	Maged1	2045.914293	-0.058964116	0.055792883
ENSRNOG00000014980	Mob4	444.3896024	-0.083962168	0.079571665
ENSRNOG00000018700	Mobp	241.4363997	-0.174398735	0.16560843
ENSRNOG00000017500	Mtss2	227.4198548	0.1050872	0.098674801
ENSRNOG00000012307	Mybpc3	436.3946059	0.090955462	0.086527928
ENSRNOG00000062154	NA	533.2705303	0.092128943	0.085443093
ENSRNOG00000061168	NA	374.6101378	0.090292188	0.083793392
ENSRNOG00000045653	NA	442.9688294	0.327479057	0.304494793
ENSRNOG00000021403	NA	1060.566214	-0.073349776	0.068762922
ENSRNOG00000009255	NA	471.8096052	-0.083101811	0.078030374
ENSRNOG00000042233	NA	1083.497285	-0.253733945	0.238443783

ENSRNOG00000053207	NA	474.0411859	-0.14485818	0.136638336
ENSRNOG00000026616	Ndufb2	800.8095286	-0.067805029	0.06380996
ENSRNOG00000058898	Nedd4	1193.117885	-0.060277598	0.056415334
ENSRNOG00000021118	Nomo1	453.6605419	-0.08270717	0.077256188
ENSRNOG00000018839	Ntrk2	1966.955878	0.083255494	0.077285679
ENSRNOG00000001456	Nup153	571.9728484	-0.129382892	0.122600161
ENSRNOG00000004904	Pa2g4	301.2927228	-0.105913643	0.098513323
ENSRNOG00000017052	Pianp	1252.551318	-0.071517128	0.0673855
ENSRNOG00000018457	Ptpa	972.9887327	-0.05742145	0.053590171
ENSRNOG00000002652	Rap1gap2	956.1563428	0.066832043	0.06352423
ENSRNOG00000017641	Rnf44	789.945007	0.060033633	0.056065238
ENSRNOG00000029410	Rpl21	1353.033582	-0.067728908	0.063828723
ENSRNOG00000016751	Slc25a28	282.7763808	0.089720511	0.084478802
ENSRNOG00000004351	Slc25a29	215.7705419	-0.096693367	0.090853435
ENSRNOG00000009459	Slc35f6	216.2027224	-0.103032717	0.097310411
ENSRNOG00000003762	Smarca1	395.5396725	-0.080362244	0.076198331
ENSRNOG00000007033	Sorcs2	309.5388647	0.084226467	0.079263047
ENSRNOG00000002607	Sox9	325.4072238	-0.083239778	0.078095582
ENSRNOG00000007400	Srebf2	404.6672474	-0.386191249	0.365365233
ENSRNOG00000000033	Tmcc2	1224.011305	0.081279057	0.075891056
ENSRNOG00000014058	Tomm22	234.9320764	-0.105430875	0.09825059
ENSRNOG00000012081	Txn1	624.5227869	-0.079999172	0.074234582
ENSRNOG00000000921	Ubl3	1285.283598	0.062112261	0.058533677
ENSRNOG00000017780	Akr7a2	246.1301957	-0.118551379	0.113279603
ENSRNOG00000009050	Amn	270.6222187	0.10692525	0.102176173
ENSRNOG00000018048	Dctn5	405.7665331	-0.081778867	0.078003218
ENSRNOG00000057078	Ddit4	307.223817	-0.170651047	0.16338726
ENSRNOG00000013011	Dnajb4	275.722974	-0.114042239	0.109024471
ENSRNOG00000026328	Gpr137c	267.2313554	-0.102269184	0.097678126
ENSRNOG00000045968	NA	372.4608984	-0.512385901	0.488698135
ENSRNOG00000061320	NA	671.6874833	-0.084334421	0.080627679
ENSRNOG00000053405	Rpap3	193.5753195	0.111359189	0.106496629
ENSRNOG00000023226	S100a10	253.0516104	-0.118869375	0.113717151
ENSRNOG00000000879	Slc9a6	303.299525	-0.100256332	0.095962841
ENSRNOG00000003496	Tbc1d9	228.0707051	0.090298274	0.086465879
ENSRNOG00000027126	Dpy30	264.364924	-0.113578782	0.108924432
ENSRNOG00000000246	Amz2	493.5019628	0.072388006	0.069527874
ENSRNOG00000027797	Nit2	831.8622548	-0.08064787	0.07745389
ENSRNOG00000021259	Prnp	4000.157823	-0.05121655	0.049193193
ENSRNOG00000042333	Dnal1	2711.956084	-0.052629638	0.050665653
ENSRNOG00000016541	Enc1	2994.01955	0.07152433	0.068900717
ENSRNOG00000048623	NA	199.0716468	0.105212199	0.101210699
ENSRNOG00000009248	Pnrc2	362.7923877	0.080285931	0.077211622
ENSRNOG00000052307	Spats2	192.8761692	-0.11693058	0.112643295
ENSRNOG00000010263	Cldn11	910.7638713	0.104592629	0.10088073
ENSRNOG00000022664	Kat7	325.0340267	-0.092240892	0.089108141
ENSRNOG00000033134	Mef2c	2298.938027	-0.055312874	0.053412169
ENSRNOG00000018593	Txndc9	286.8214474	-0.090785728	0.08767951
ENSRNOG00000019306	Syt12	271.0842255	-0.104402712	0.101207636
ENSRNOG00000011194	Tpp2	250.01121	-0.112026925	0.108561086

ENSRNOG00000012062	Npc2	460.6406079	-0.10054578	0.097515549
ENSRNOG00000008016	Ckap4	268.9045669	-0.099385541	0.096650524
ENSRNOG00000007775	Cmpk1	685.0387519	-0.07832558	0.076043667
ENSRNOG00000020119	Pcdhac2	517.4139124	0.079788267	0.07751119
ENSRNOG00000016872	Plppr4	751.8287508	0.076149551	0.074006522
ENSRNOG00000054118	Tpgs2	456.3811025	-0.090232203	0.087719607
ENSRNOG00000021614	Kmt2e	220.6992928	0.107386058	0.10463244
ENSRNOG00000057367	Glud1	2249.175095	-0.06389704	0.062324893
ENSRNOG00000056040	NA	233.4079804	0.109644142	0.106905212
ENSRNOG00000009640	Psmf1	319.6726355	-0.101668768	0.099229183
ENSRNOG00000028129	Fktn	219.4539763	0.100493712	0.098177688
ENSRNOG00000049071	NA	2698.454511	-0.049690936	0.048529471
ENSRNOG00000033844	Bex1	1144.51361	0.101200219	0.098915836
ENSRNOG00000036688	Arhgdia	2064.953865	-0.056608244	0.055459691
ENSRNOG00000014908	Sf3b5	317.5663195	-0.079895051	0.078279675
ENSRNOG00000007688	Sms	411.7887286	-0.082937558	0.08118247
ENSRNOG00000020551	Bles03	265.9877367	-0.121746338	0.119396301
ENSRNOG00000013250	NA	563.0435597	-0.065500975	0.064244534
ENSRNOG00000009920	NA	5592.639344	-0.062634569	0.061549174
ENSRNOG00000025286	Pank2	233.74704	-0.090744091	0.089192028
ENSRNOG00000031852	Lsamp	223.7515152	-0.117764056	0.115835229
ENSRNOG00000016923	Clptm1l	837.8522939	-0.060448537	0.059545481
ENSRNOG00000018566	Ctsl	350.7171539	0.089516218	0.088275026
ENSRNOG00000031506	Ftl1	571.2707493	-0.087988889	0.086904409
ENSRNOG00000000048	Gak	281.1729082	0.106703352	0.105334398
ENSRNOG00000009421	Ivd	494.5058649	-0.101902235	0.100783881
ENSRNOG00000009381	Mapk6	258.7746564	-0.097447691	0.096165633
ENSRNOG00000048897	NA	306.9565679	-0.081155156	0.080253835
ENSRNOG00000017227	Ppme1	769.3316113	-0.067913233	0.067114016
ENSRNOG00000007628	Ptp4a3	475.0675493	-0.084735754	0.083837217
ENSRNOG00000016877	Shisa7	355.1677398	0.091308473	0.090329614
ENSRNOG00000013938	Tshz3	217.9514157	0.09281196	0.09156625
ENSRNOG00000017777	Ahcy	238.7558152	-0.093187349	0.09243144
ENSRNOG00000004526	Cox7a2l	784.5597202	0.089465944	0.088917905
ENSRNOG00000060048	Eif2s3y	192.5908356	0.10483776	0.104073808
ENSRNOG00000001893	NA	783.5098375	-0.073082423	0.072416077
ENSRNOG00000036432	NA	862.5965764	0.076339402	0.075856824
ENSRNOG00000014050	Ptges2	350.8592647	-0.085748784	0.085205184
ENSRNOG00000011912	Tmem38a	614.8520868	-0.071371895	0.070929087
ENSRNOG00000018107	Zfand5	1015.339138	-0.071880053	0.071332874
ENSRNOG00000031367	Hprt1	750.6403058	-0.066031372	0.065660113
ENSRNOG00000050946	Fam110a	880.3622792	-0.106796896	0.106396845
ENSRNOG00000000321	Cd24	476.4635128	0.087967391	0.087900831
ENSRNOG00000030408	Dnajb12	223.4818744	-0.101132048	0.101006277
ENSRNOG00000014775	Nol4	241.4957695	0.091676878	0.091637137
ENSRNOG00000060817	Stam	313.7780273	-0.083932992	0.083862272
ENSRNOG00000048682	Zwint	16594.92862	-0.045250951	0.045188113
ENSRNOG00000032828	Mapk8ip2	1419.835511	-0.057911201	0.057966885
ENSRNOG00000020133	NA	241.9917475	-0.118293395	0.118428753
ENSRNOG00000033722	Rnf207	386.7840573	0.105623311	0.105718908

ENSRNOG00000015895	B4galt6	595.9307082	-0.07260639	0.07277467
ENSRNOG00000046053	Nudt10	269.1015309	-0.083508466	0.083751959
ENSRNOG00000011142	Cyb5b	243.0499767	-0.091414544	0.091895339
ENSRNOG00000002940	Ankrd17	377.596209	0.109692129	0.11152491
ENSRNOG00000052080	Camk2b	1511.241945	-0.087798124	0.088635636
ENSRNOG00000006985	Ccnk	379.6346103	-0.115591749	0.117468561
ENSRNOG00000012788	Clns1a	460.7297352	0.077968205	0.079080716
ENSRNOG00000006499	Cops5	520.6191788	-0.075095451	0.076357315
ENSRNOG00000050015	Cox7a1	1138.289685	0.111652136	0.11309998
ENSRNOG00000052758	Cyria	737.0641033	0.084813838	0.08589668
ENSRNOG00000052887	Dnajc6	1030.490147	-0.069662985	0.070217105
ENSRNOG00000051948	Hcfc1	212.3950273	0.121906675	0.123027504
ENSRNOG00000000902	Hsph1	739.70388	0.08089065	0.082312958
ENSRNOG00000014571	NA	676.1179666	-0.058868407	0.059712492
ENSRNOG00000031579	NA	318.0337274	-0.093932355	0.095572238
ENSRNOG00000015603	Prkce	414.3760067	-0.103089563	0.104803532
ENSRNOG00000005538	Psmd11	273.4881521	-0.090178893	0.091067587
ENSRNOG00000021295	Rae1	352.9012193	-0.075798504	0.076742924
ENSRNOG00000006833	Rb1cc1	264.5705696	-0.11003243	0.111028316
ENSRNOG00000002773	Rgs4	1499.871588	0.073321349	0.074495136
ENSRNOG00000024603	Rps15	4789.063993	-0.056557727	0.05725413
ENSRNOG00000055019	Snord89	328.4260468	-0.082909732	0.083986124
ENSRNOG00000000547	Tspyl4	477.0587846	0.113060111	0.114067318
ENSRNOG00000025252	Yars2	519.4579135	0.072403327	0.073656229
ENSRNOG00000010589	Zfp622	346.4788575	-0.095721504	0.097048224
ENSRNOG00000019933	Ubqln4	404.7618018	0.083278929	0.084921683
ENSRNOG00000047187	NA	198.8275029	0.103509255	0.105662629
ENSRNOG00000013239	Tle4	243.0976918	-0.136980644	0.139816699
ENSRNOG00000042245	Dcaf7	317.3238162	0.092729444	0.094738524
ENSRNOG00000013866	Usp49	554.1111128	-0.073556331	0.075275498
ENSRNOG00000056825	AABR07072539.4	801.3130925	-0.074037128	0.075847814
ENSRNOG00000001247	Clip1	347.7866017	0.080445946	0.082387716
ENSRNOG00000009276	Schip1	1021.327531	-0.063746498	0.065334361
ENSRNOG00000053044	NA	231.7385468	-0.103180716	0.105816164
ENSRNOG00000018285	Kcna2	458.9674817	0.093527783	0.096420345
ENSRNOG00000027955	Zfp133	278.8483089	0.111254213	0.114695606
ENSRNOG00000011798	Mapre1	255.9714516	0.091430514	0.094386711
ENSRNOG00000009983	Lrrc42	216.3020772	0.09629806	0.099489913
ENSRNOG00000055446	Amfr	2483.906289	0.050756802	0.052658017
ENSRNOG00000016358	Apba2	891.15275	0.055045289	0.057103777
ENSRNOG00000016412	Fxyd6	882.2878757	-0.10472803	0.108866739
ENSRNOG00000019570	Gng3	3109.432062	-0.058983796	0.061276799
ENSRNOG00000000913	Gusb	296.1530341	-0.087226275	0.09070677
ENSRNOG00000012601	Leprotl1	417.6533734	-0.082883982	0.086093392
ENSRNOG00000010180	NA	227.1052304	-0.091992052	0.095259746
ENSRNOG00000020615	Nap1l4	653.5657148	-0.105696901	0.109731335
ENSRNOG00000004784	Ndufaf5	255.7937061	0.088813157	0.092280989
ENSRNOG00000022323	Tcea1	377.1011842	-0.08569268	0.088863176
ENSRNOG00000008709	Arhgap32	346.5762908	0.135899361	0.141465159
ENSRNOG00000013577	Kdelr3	581.7346177	0.066206812	0.068895981

ENSRNOG00000003206	Actr3	1006.501157	-0.063569677	0.06646051
ENSRNOG00000042419	Acyp2	376.8198793	-0.080796067	0.085466624
ENSRNOG00000043465	Arc	637.8972993	-0.470576172	0.494576355
ENSRNOG00000004560	Cacna1b	261.830174	0.095567646	0.099793468
ENSRNOG00000013481	Cdh11	256.4418252	0.087922977	0.091827119
ENSRNOG00000004695	Celf5	1398.077546	0.06724499	0.070937125
ENSRNOG00000000105	Cplx2	458.4150349	-0.204405275	0.215962088
ENSRNOG00000005796	Ctnna1	299.2033193	-0.081704888	0.085703077
ENSRNOG00000014599	Dhx36	338.6118495	-0.094904205	0.100275399
ENSRNOG00000014856	Etnk1	375.2202828	0.081372737	0.086063184
ENSRNOG00000010019	Fam166a	1084.238805	-0.060005733	0.062641451
ENSRNOG00000042274	Fbxo31	437.6933706	-0.06899673	0.072304138
ENSRNOG00000036660	Fn3krp	211.2304868	-0.104664887	0.110210453
ENSRNOG00000032401	H3f3c	991.4611041	0.056378463	0.059597671
ENSRNOG00000019568	Jund	1174.776057	-0.106861142	0.112222137
ENSRNOG00000061038	Myh15	236.079497	-0.083794197	0.088402306
ENSRNOG00000049536	NA	352.0259153	-0.10427476	0.108909321
ENSRNOG00000020830	NA	232.0571874	0.105484889	0.110432526
ENSRNOG00000005811	NA	5453.202748	-0.060357525	0.063767806
ENSRNOG00000048136	NA	246.4287472	-0.099146665	0.104866623
ENSRNOG00000014078	Ndufb8	632.0210102	0.070918852	0.074910331
ENSRNOG00000017610	Nedd4l	196.9773504	0.095795337	0.101194253
ENSRNOG00000000411	Nus1	392.7973782	0.071723145	0.07552992
ENSRNOG00000018586	Retreg2	1450.098761	-0.054675557	0.057507889
ENSRNOG00000003121	Rtn4rl1	343.9603296	0.098682449	0.104087327
ENSRNOG00000061582	Scamp1	749.4642101	-0.056503322	0.059295
ENSRNOG00000005018	Scn2a	1166.403798	0.072216755	0.076122527
ENSRNOG00000011488	Sergef	212.1990504	-0.094026309	0.098076817
ENSRNOG00000012957	Sh3glb1	370.6458573	0.072059117	0.075590511
ENSRNOG00000011160	Sv2b	1645.536862	-0.064326058	0.067857279
ENSRNOG00000061782	Tspyl2	429.1660739	-0.087393261	0.092191056
ENSRNOG00000015734	Ube3a	432.3802238	-0.078969537	0.082952831
ENSRNOG00000020110	B4gat1	383.6141009	0.075507742	0.079990489
ENSRNOG00000048506	Inpp5b	259.6507094	-0.127716646	0.13529649
ENSRNOG00000059659	Zfand3	401.0779636	-0.07621583	0.080838456
ENSRNOG00000007738	Hm13	684.2980463	0.056602596	0.060123413
ENSRNOG00000025079	Fam126b	261.4867475	-0.09377609	0.09966074
ENSRNOG00000000894	Fry	532.6554607	0.074917477	0.079838525
ENSRNOG00000047374	Gnas	4322.309965	-0.053217207	0.056672531
ENSRNOG00000020255	Sars1	713.511823	-0.066792365	0.071172919
ENSRNOG00000018475	Eif4enif1	248.9300734	-0.079069389	0.084333679
ENSRNOG00000027204	Map6	410.6755223	-0.079673844	0.085168508
ENSRNOG00000003276	Myo1d	1828.90836	-0.078825837	0.08433696
ENSRNOG00000019688	Diaph1	3223.324846	-0.051095587	0.054710136
ENSRNOG00000026745	Acsl6	625.788807	0.079617705	0.085402369
ENSRNOG00000009629	Car2	409.9843107	-0.078277453	0.083949781
ENSRNOG00000000314	Sec63	205.1585544	-0.090095629	0.09657765
ENSRNOG00000016866	Fhl2	279.592965	0.093726881	0.100757208
ENSRNOG00000023935	Nrsn2	711.968388	-0.077993817	0.083734023
ENSRNOG00000019007	Rpl14	1498.0304	-0.053436244	0.05741077

ENSRNOG00000007892	Tram1	322.5174614	-0.08835768	0.095019984
ENSRNOG00000005480	Ybx3	216.1907039	-0.090936837	0.097779247
ENSRNOG00000019750	NA	241.6085076	0.116829929	0.125758151
ENSRNOG00000001827	Masp1	223.9855491	-0.119911151	0.129232049
ENSRNOG00000058476	Mast2	428.5433766	0.0713725	0.076890727
ENSRNOG00000043350	Cap2	345.4064947	-0.078734806	0.085286072
ENSRNOG00000021031	Grn	324.6388033	-0.100432164	0.108435591
ENSRNOG00000004132	Lasp1	1025.204443	0.059860075	0.064613385
ENSRNOG00000017428	Map1b	4149.712239	0.076217057	0.082652321
ENSRNOG00000024954	Mgat5b	422.4873055	-0.079415349	0.085991145
ENSRNOG00000020659	Mrpl4	391.5525392	-0.080718246	0.087231989
ENSRNOG00000051077	NA	194.2187345	-0.087786694	0.095099579
ENSRNOG00000006335	NA	255.8194605	0.092057411	0.099798944
ENSRNOG00000017127	Rpl28	1680.258451	-0.060672132	0.065619852
ENSRNOG00000022472	Snap47	324.7766261	-0.089836148	0.097428759
ENSRNOG00000010284	St3gal5	947.1956138	-0.063431285	0.068551172
ENSRNOG00000001201	Cstb	413.4182642	-0.079447891	0.086291833
ENSRNOG00000032913	Rasl2-9	531.3349478	0.234731054	0.254998682
ENSRNOG00000030520	Sarnp	298.1000902	-0.087382553	0.09496574
ENSRNOG00000002240	Slc49a4	465.5381986	-0.078647562	0.085431078
ENSRNOG00000007390	Nfkbia	253.7724704	0.127496504	0.138758983
ENSRNOG00000008744	Cops2	414.0606301	-0.08119419	0.088415602
ENSRNOG00000033030	AABR07072440.1	650.6231334	-0.058496932	0.06478955
ENSRNOG00000011929	Abcd3	685.0469798	-0.085706886	0.094574289
ENSRNOG00000009244	Abhd4	307.7974092	-0.129295237	0.14130506
ENSRNOG00000014035	Arhgef4	544.1298962	0.075338978	0.083474716
ENSRNOG00000013591	Ascl3	274.6904463	-0.089353436	0.097958905
ENSRNOG00000020202	Asrgl1	405.3583331	-0.073995488	0.082036021
ENSRNOG00000017123	B2m	1298.604046	-0.071457425	0.078658209
ENSRNOG00000003198	Bmerb1	1215.000019	-0.048212156	0.053139113
ENSRNOG00000016912	Btf3	650.1609409	-0.069618607	0.076966481
ENSRNOG00000020643	Bub3	202.6324205	-0.102086459	0.113015964
ENSRNOG00000036835	Copz1	475.7808734	-0.064126003	0.070888867
ENSRNOG00000010807	Cox6c	3132.604185	-0.083279695	0.091433452
ENSRNOG00000018198	Dapk1	317.9524405	0.080214196	0.08802686
ENSRNOG00000003611	Dynlt3	553.8712654	-0.070235543	0.076765919
ENSRNOG00000004220	Ebag9	211.0296174	0.099315475	0.109692169
ENSRNOG00000002327	Gabrb1	296.307235	0.127331103	0.140807899
ENSRNOG00000003847	Gid4	203.0435473	0.104466149	0.114879658
ENSRNOG00000033824	Gpd2	339.5102936	0.100822656	0.111119065
ENSRNOG00000002627	Kctd3	195.199183	0.092857913	0.101343261
ENSRNOG00000017087	Man1c1	222.6427591	-0.102776898	0.112484505
ENSRNOG00000052007	NA	619.1491183	-0.29703378	0.328759833
ENSRNOG00000037673	NA	207.3416358	0.130217043	0.14431498
ENSRNOG00000016708	Necab3	619.3633907	-0.069303205	0.076391766
ENSRNOG00000006228	Pdia4	242.5327676	0.103668978	0.114336387
ENSRNOG00000018044	Phyh	465.2224349	-0.070061655	0.077538099
ENSRNOG00000002097	Rasl11b	631.1319952	-0.070149173	0.077187141
ENSRNOG00000050298	Rfng	404.986518	0.078235905	0.085693657
ENSRNOG00000001185	RGD1311899	692.2380426	0.309997979	0.338684153

ENSRNOG00000045637	Rnf14	2032.474252	0.054898801	0.060533947
ENSRNOG00000020425	Stim1	264.7236806	-0.09581898	0.105711832
ENSRNOG00000023708	Tmem176a	394.3067042	-0.197332368	0.217446469
ENSRNOG00000027300	Ttc7b	1624.416041	-0.079602227	0.088105884
ENSRNOG00000006392	Xrcc6	699.6580499	-0.066618852	0.073221626
ENSRNOG00000017568	Rit2	713.4978428	0.057558035	0.063877376
ENSRNOG00000000967	Aacs	205.3779877	0.118237734	0.131330905
ENSRNOG00000001285	Atp2a2	2336.951648	0.045542954	0.050611114
ENSRNOG00000015544	NA	1066.736359	-0.067731997	0.075294203
ENSRNOG00000011704	Fbxo34	419.7176158	-0.086560565	0.096275159
ENSRNOG00000004049	Baiap2	2240.223618	0.052097368	0.058013539
ENSRNOG00000001190	Git2	231.5419867	0.092282154	0.102804935
ENSRNOG00000052998	NA	1677.66635	-0.445664855	0.496110708
ENSRNOG00000022428	Flrt1	203.8112239	-0.120494981	0.134400458
ENSRNOG00000001300	P2rx4	413.6949861	0.112915603	0.125996727
ENSRNOG00000020763	Snrnp70	580.0577867	0.12485363	0.139266589
ENSRNOG00000005902	Ccdc34	207.8538091	-0.120078489	0.13440638
ENSRNOG00000001253	Eif3b	366.5662498	-0.076480245	0.085758953
ENSRNOG00000050983	Hpcal4	2916.191278	-0.059589648	0.066732782
ENSRNOG00000052486	Kcna6	375.4912851	0.064906516	0.072897431
ENSRNOG00000002886	Myh10	810.1656206	0.079922995	0.089449477
ENSRNOG00000055305	Parvb	322.9007546	0.079642847	0.089257303
ENSRNOG00000006800	Pgap4	548.2858387	-0.066034147	0.074128253
ENSRNOG00000012164	Plppr2	1503.819878	-0.052278171	0.058659329
ENSRNOG00000030920	Rtn4r	819.156461	0.074084437	0.082963966
ENSRNOG00000010626	Sphk1	455.7552988	0.065100502	0.072969988
ENSRNOG00000019965	Tgfb1i1	209.0764977	0.09036741	0.101417635
ENSRNOG00000023065	NA	673.9560752	-0.058799151	0.06615105
ENSRNOG00000006305	Slc38a2	352.7904494	0.090795688	0.102345164
ENSRNOG00000034182	NA	659.9323886	-0.064900698	0.073219641
ENSRNOG00000020117	Brms1	431.0065458	0.067239298	0.075927723
ENSRNOG00000011411	Adgrg6	334.2265803	-0.066330361	0.074975114
ENSRNOG00000008157	Syn2	4077.786284	-0.044004825	0.049785408
ENSRNOG00000046975	Sult4a1	2743.412785	-0.037531429	0.0424923
ENSRNOG00000010103	NA	701.9564271	-0.078889092	0.08956128
ENSRNOG00000009512	Esd	383.1702233	-0.081154092	0.092263202
ENSRNOG00000011199	Parp6	1117.871095	-0.054373403	0.061869842
ENSRNOG00000010628	Sec13	242.6337875	0.087764385	0.099865177
ENSRNOG00000006785	Dcaf8	258.7119508	-0.078782409	0.089858335
ENSRNOG00000011140	Prxl2a	568.6815672	-0.067409688	0.076903197
ENSRNOG00000017679	Cckbr	211.8276832	0.124398566	0.142118583
ENSRNOG00000046211	Fbxw4	209.5914604	0.110420046	0.126290671
ENSRNOG00000053147	NA	224.3702593	0.091322324	0.104358903
ENSRNOG00000010146	Ndn	2486.542583	-0.062313335	0.071261058
ENSRNOG00000020937	Ubtf	429.7534487	0.072163888	0.082750834
ENSRNOG00000009716	Casc3	491.7803893	0.058892294	0.06795518
ENSRNOG00000007682	Gria3	542.5551014	0.071110558	0.082041789
ENSRNOG00000015380	Jup	457.2128731	0.090167937	0.103715089
ENSRNOG00000021579	Mpp6	210.207323	-0.099054343	0.114334024
ENSRNOG00000060262	NA	311.9809462	-0.073396671	0.084348663

ENSRNOG00000047364	NA	1006.353381	0.101136424	0.116613229
ENSRNOG00000018129	Ndufab1	1933.346994	-0.058830077	0.067832368
ENSRNOG00000010477	Pomt1	747.2117407	0.066380806	0.076625604
ENSRNOG00000052113	Ppp1r9b	2063.704345	0.047410596	0.054492261
ENSRNOG00000011400	Ralyl	308.62607	0.084912191	0.097803739
ENSRNOG00000020457	Tacc2	328.0661801	0.083216271	0.09591456
ENSRNOG00000010881	Trak2	328.7336086	0.080500143	0.092870011
ENSRNOG00000011825	Ndufb3	680.0439566	-0.054542815	0.063127251
ENSRNOG00000016137	Rad23b	619.0052627	-0.073282782	0.0847451
ENSRNOG00000014025	Rasgrf1	1666.378145	0.057246184	0.066232609
ENSRNOG00000006604	Thy1	1380.82235	-0.065457242	0.075650359
ENSRNOG00000059545	AC114446.1	976.4216312	-0.062833051	0.072899377
ENSRNOG00000002032	Ifngr2	367.5893247	-0.087569765	0.101565392
ENSRNOG00000054371	Prkcg	628.6962113	-0.328724197	0.381441169
ENSRNOG00000042449	Cdc42se2	637.2798989	0.070211239	0.081608256
ENSRNOG00000018648	Mppe1	296.0549731	-0.079833349	0.092781178
ENSRNOG00000043182	Septin6	372.6694448	0.079567308	0.092717081
ENSRNOG00000000549	Tspyl1	658.3448203	-0.052831211	0.06166347
ENSRNOG00000024437	Blcap	656.9501153	0.065927094	0.077535829
ENSRNOG00000012795	Cables1	200.4547577	-0.09130196	0.107354602
ENSRNOG00000034078	Mxi1	512.853164	0.065592858	0.076873977
ENSRNOG00000056765	NA	1813.420932	0.350588975	0.411767899
ENSRNOG00000011329	Pkm	3428.476653	-0.039507604	0.046315681
ENSRNOG00000027871	Rapgefl1	969.9867299	0.065226558	0.076716492
ENSRNOG00000005017	RGD1566369	317.4726597	-0.070341298	0.082757675
ENSRNOG00000010358	Sh3rf1	209.6913076	0.089436397	0.105020725
ENSRNOG00000010601	Srpk2	589.0439251	-0.074864814	0.088067571
ENSRNOG00000003794	Nmral1	340.2453765	-0.075417194	0.088815743
ENSRNOG00000013751	Plpbp	192.6927059	-0.08429834	0.099411401
ENSRNOG00000026742	Ypel5	652.9360867	-0.050757687	0.059899284
ENSRNOG00000010892	Ythdf2	205.6318917	-0.100508123	0.118684515
ENSRNOG00000021051	Cyth2	368.1285273	-0.072787189	0.086238464
ENSRNOG00000004201	Rims2	441.0261203	-0.058048688	0.068756975
ENSRNOG00000014986	Ube4b	274.289572	-0.088745234	0.105298904
ENSRNOG00000006895	Vps53	300.3467465	-0.073990913	0.087856912
ENSRNOG00000019772	Dnpep	433.0951871	0.077335916	0.091931225
ENSRNOG00000060098	Ptms	671.3060184	-0.200759373	0.238623184
ENSRNOG00000004099	R3hdm1	406.3917135	0.077175758	0.091789801
ENSRNOG00000010171	Elk1	234.7365841	0.102174893	0.121611024
ENSRNOG00000007427	Entpd6	354.3060793	-0.063330131	0.075408638
ENSRNOG00000047931	NA	6664.44553	-0.033041601	0.039361519
ENSRNOG00000036841	Cbx5	218.5615009	-0.084901722	0.101329427
ENSRNOG00000052343	Eif4e	380.4690866	-0.062781468	0.074962415
ENSRNOG00000020533	Htra1	732.5567441	-0.052164631	0.062295683
ENSRNOG00000058461	Sfpq	212.5301814	0.10023062	0.119725097
ENSRNOG00000022741	Ilf3	205.644237	0.103080253	0.123260855
ENSRNOG00000014648	NA	214.3755123	-0.092678097	0.110792459
ENSRNOG00000057623	Copb1	274.4121842	-0.077998153	0.093406878
ENSRNOG00000004245	Mgst3	859.0797694	-0.064603493	0.077384682
ENSRNOG00000054908	NA	198.0541185	0.084479323	0.101342192

ENSRNOG00000055323	NA	994.191787	-0.405844129	0.487175936
ENSRNOG00000016414	Slc22a17	4750.658012	-0.07242332	0.086908973
ENSRNOG00000017333	Syt4	1126.177192	-0.048837553	0.058609608
ENSRNOG00000037327	NA	262.3676423	-0.239903616	0.288173165
ENSRNOG00000025075	Relt	434.3258601	0.068624267	0.082488903
ENSRNOG00000016778	Cops7a	889.3791399	-0.091668385	0.110255998
ENSRNOG00000016271	Itm2b	5647.438676	-0.03795633	0.045787167
ENSRNOG00000007515	Kif3a	203.2576763	-0.089337274	0.107782399
ENSRNOG00000042189	Rab31	387.4746096	0.062756121	0.075678976
ENSRNOG00000019987	Scand1	292.9244097	-0.076817694	0.092607036
ENSRNOG00000007901	Tmed10	241.6328479	0.077579014	0.093423428
ENSRNOG00000011014	Copg2	837.6204902	0.093660628	0.113373455
ENSRNOG00000003969	Fam20a	2037.442092	-0.050068789	0.060715388
ENSRNOG00000019497	Mrpl17	461.3616661	-0.069346877	0.084053944
ENSRNOG00000009552	Serinc3	1114.841747	0.068588179	0.083044294
ENSRNOG00000049983	Shd	505.6636691	-0.065876964	0.079867862
ENSRNOG00000050562	Zfp449	16509.05799	-0.122887192	0.149138942
ENSRNOG00000008249	Brms1l	195.0253423	-0.077754855	0.094420899
ENSRNOG00000023993	Kif1a	846.5878218	0.089035344	0.108178527
ENSRNOG00000007304	Herc3	437.3696616	0.070334668	0.085619513
ENSRNOG00000016827	Slc38a3	456.9639001	0.077962384	0.095044057
ENSRNOG00000005890	Serbp1	767.1201217	-0.063926072	0.078040058
ENSRNOG00000003009	AABR07039336.1	283.0569944	-0.093992581	0.115237914
ENSRNOG00000022307	Acap3	480.3543084	0.081288588	0.099595975
ENSRNOG00000012934	Arhgef7	743.8627873	0.045767733	0.056093767
ENSRNOG00000009643	Msl1	422.4634831	-0.062982724	0.07709815
ENSRNOG00000011849	Ndufs1	225.8383982	0.080422825	0.098499468
ENSRNOG00000023312	Lonrf2	524.7414155	0.068597616	0.084234555
ENSRNOG00000018824	Slc7a5	234.2078381	0.075396609	0.092617998
ENSRNOG00000024139	Miip	234.2429889	-0.079887481	0.098223869
ENSRNOG00000015550	Ptgds	3604.824873	0.267084848	0.328788818
ENSRNOG00000002697	Mtmr1	196.4535826	-0.09044243	0.111442563
ENSRNOG00000000412	Slc35f1	219.9708716	-0.113905499	0.14038505
ENSRNOG00000016937	Mtfr1l	319.8858013	-0.082241909	0.101428448
ENSRNOG00000021714	Cibar2	765.480979	0.044964867	0.055491681
ENSRNOG00000052731	NA	249.6554616	0.076101804	0.094034157
ENSRNOG00000001829	Ube2v2	400.9059786	-0.064471282	0.079671368
ENSRNOG00000001316	Anapc5	755.0571248	-0.062989907	0.077984038
ENSRNOG00000006966	Nfia	241.2151171	0.097302345	0.120478865
ENSRNOG00000012764	Agap3	1516.196533	-0.061080227	0.075731213
ENSRNOG00000047028	Mmp24	839.7937022	-0.055390845	0.068918229
ENSRNOG00000017155	Nck2	200.2566717	-0.073017648	0.090816052
ENSRNOG00000014650	Sema4g	221.4557304	0.210615047	0.262133149
ENSRNOG00000049127	Smap1	224.8273053	-0.117280553	0.145932833
ENSRNOG00000031536	Syndig1	255.7559837	-0.087106675	0.108315101
ENSRNOG00000004755	Commd9	440.8817888	-0.093371042	0.116567642
ENSRNOG00000009341	Hivep3	298.3783149	0.09457169	0.117954224
ENSRNOG00000002364	Rnf112	763.2427284	0.059625241	0.074470707
ENSRNOG00000028185	Tstd3	208.7086158	-0.088754484	0.110778341
ENSRNOG00000052087	Ubap2	326.0108266	-0.080329682	0.100353287

ENSRNOG00000010041	Ube2g1	358.071563	-0.069602472	0.08676896
ENSRNOG00000011804	Stx12	678.3162822	-0.059884452	0.074927318
ENSRNOG00000016532	Ino80c	193.4662143	-0.094812599	0.118826029
ENSRNOG00000015162	Lonp2	254.2200698	0.091044714	0.114011796
ENSRNOG00000025731	Ptma	458.8606842	-0.069630896	0.087317214
ENSRNOG00000005823	Utp20	348.6740038	0.067372847	0.084489707
ENSRNOG00000001192	Gltp	220.3930455	0.079720849	0.100159846
ENSRNOG00000011398	Trmt61a	304.0386676	-0.080177676	0.100781727
ENSRNOG00000008673	Arpc3	1144.905414	-0.063334289	0.079745565
ENSRNOG00000047280	NA	318.4167586	0.070558784	0.08883853
ENSRNOG00000013479	Stard7	502.3867509	0.057279232	0.072158322
ENSRNOG00000011227	Atp1b2	931.2916143	0.055462217	0.070082258
ENSRNOG00000000697	Coro1c	353.6986492	-0.067715735	0.085506186
ENSRNOG00000014302	Dlgap3	792.1182756	0.070505135	0.089113997
ENSRNOG00000056172	NA	788.2270373	-0.376223305	0.474431624
ENSRNOG00000037214	NA	284.7531186	0.073500373	0.092947533
ENSRNOG00000002642	Ptges3	1160.456643	-0.053699728	0.067819784
ENSRNOG00000047853	Ss18l2	371.5716827	-0.059533236	0.075294322
ENSRNOG00000008746	Dtd1	695.1758843	-0.047046651	0.059628251
ENSRNOG00000059406	Ier5	643.6777042	-0.164624982	0.208681006
ENSRNOG00000017850	Dctpp1	253.0506726	-0.094882717	0.120363411
ENSRNOG00000010350	Rcan2	774.3852196	-0.054288758	0.068970381
ENSRNOG00000003211	Srp9	354.1560081	-0.069440767	0.088286
ENSRNOG00000048088	Mest	246.9731312	0.073912497	0.09408391
ENSRNOG00000032729	Bex2	1983.529948	-0.046424774	0.059187443
ENSRNOG00000011111	Cipc	346.089753	0.066274523	0.084648389
ENSRNOG00000016034	Cnot9	249.8845844	0.102245265	0.130808373
ENSRNOG00000009217	Fbxo6	575.872829	0.054946134	0.070279454
ENSRNOG00000055911	NA	1702.333643	-0.049763848	0.063560608
ENSRNOG00000007238	NA	606.8576057	-0.061345577	0.078457149
ENSRNOG00000049219	Rnf187	3834.732168	-0.046961694	0.060073303
ENSRNOG00000020501	Tjp3	617.8305018	-0.589610519	0.752613809
ENSRNOG00000009513	Akr1b1	408.8345772	0.066058016	0.08584578
ENSRNOG00000017913	Atg16l1	209.3688612	0.078333153	0.101514232
ENSRNOG00000053075	B4galnt4	349.462878	-0.056883245	0.073942753
ENSRNOG00000011778	Blvra	215.0827676	0.090010125	0.115443571
ENSRNOG00000005592	Brinp2	598.9093363	-0.075035577	0.096594675
ENSRNOG00000017158	Eif4g2	1759.330224	0.059646405	0.077282164
ENSRNOG00000002024	Epha5	296.8830159	-0.065106408	0.084474278
ENSRNOG00000061733	Galr2	270.4755759	-0.078842545	0.102180835
ENSRNOG00000025959	Gp1ba	477.4123419	0.060276338	0.07816993
ENSRNOG00000010819	Hspa4l	251.8458068	0.073829848	0.095487585
ENSRNOG00000061230	L1cam	641.4088675	-0.048920722	0.063095094
ENSRNOG00000006865	Laptm4a	1019.806805	-0.046480544	0.060345191
ENSRNOG00000012178	Lmbrd1	559.3845142	0.067052184	0.087082395
ENSRNOG00000011592	Lrp10	305.3049945	0.066256181	0.085151116
ENSRNOG00000000513	Mapk14	314.5282406	0.085843837	0.110439221
ENSRNOG00000003336	Mybph	271.8543714	0.084898359	0.108891229
ENSRNOG00000027929	NA	595.270337	0.063758504	0.081874287
ENSRNOG00000030228	NA	4520.48825	-0.046487539	0.060351368

ENSRNOG00000048036	Ncan	359.6481439	-0.279727283	0.359011423
ENSRNOG00000049054	Ntsr2	675.2682082	0.055806132	0.072316411
ENSRNOG00000017741	Nudt5	288.1048111	-0.068935461	0.088717301
ENSRNOG00000021492	Rbbp4	246.5484717	-0.109410075	0.140983145
ENSRNOG00000001483	Rcc1l	386.6891277	0.065319138	0.084870398
ENSRNOG00000012840	Sparc	3072.293471	-0.058420099	0.075687942
ENSRNOG00000042501	Tmem250	247.6654273	0.074972527	0.097395239
ENSRNOG00000053061	Ubl4a	298.3154932	0.080035405	0.1039353
ENSRNOG00000023896	Dusp6	197.333051	-0.203793403	0.265318328
ENSRNOG00000007862	Acat1	501.9805504	-0.059285338	0.077283051
ENSRNOG00000017444	Nrsn1	3352.950338	-0.040609022	0.052965706
ENSRNOG00000014268	Abca2	460.3341696	0.061907227	0.080911391
ENSRNOG00000013620	Cdk13	257.9910659	0.094350497	0.123431746
ENSRNOG00000027711	Usp32	534.1502754	0.062179774	0.081387916
ENSRNOG00000024647	Ppp4r2	282.6440521	0.073706226	0.096835998
ENSRNOG00000022980	Sdhd	505.2043169	-0.052118245	0.068487684
ENSRNOG00000014331	Zfp810	214.1602779	-0.073259345	0.096165299
ENSRNOG00000002840	Atp5f1b	10633.4538	-0.039655141	0.052365741
ENSRNOG00000020753	Bnip1	232.9506931	-0.079745826	0.105168394
ENSRNOG00000000522	Cpne5	996.9656932	-0.068422135	0.09002931
ENSRNOG00000003248	Mpzl1	205.9063005	-0.073706379	0.097351385
ENSRNOG00000059660	NA	986.1801798	0.196585837	0.259590373
ENSRNOG00000029115	RGD1564883	279.2495846	-0.296771632	0.391861517
ENSRNOG00000017036	Sec11c	998.2947639	-0.058443472	0.076930843
ENSRNOG00000018644	Slc6a7	412.9169473	0.070335792	0.092859716
ENSRNOG00000001657	Cldnd1	433.4780312	0.059314569	0.078409104
ENSRNOG00000053452	Actg1	9507.752465	-0.03160066	0.041866345
ENSRNOG00000017731	Agpat4	360.3369792	0.064370755	0.085405716
ENSRNOG00000004284	Btg1	256.8307805	-0.067646032	0.089641833
ENSRNOG00000015939	Ccdc25	210.9287416	-0.074008227	0.098198035
ENSRNOG00000018704	Nolc1	366.2229536	-0.056201785	0.074507729
ENSRNOG00000013180	Ercc3	200.0632402	-0.079202412	0.105345386
ENSRNOG00000018808	Vip	385.4516501	0.058744344	0.078124505
ENSRNOG00000019891	Sgta	1221.005364	-0.048691363	0.064842842
ENSRNOG00000014064	Ctsh	283.6671198	0.064478045	0.085980887
ENSRNOG00000048242	Txlna	296.8696858	0.066962586	0.089248147
ENSRNOG00000006148	RGD1310352	617.9009019	-0.094722342	0.12639401
ENSRNOG00000004814	Elob	643.6664324	0.055698535	0.074494495
ENSRNOG00000014297	Sdc4	310.6223841	-0.066685497	0.089389798
ENSRNOG00000020279	Syt11	4610.265827	-0.115515612	0.15476386
ENSRNOG00000027569	Trappc9	272.812227	-0.063834281	0.085672563
ENSRNOG00000001205	Agpat3	847.1995114	0.050542605	0.067982972
ENSRNOG00000023467	Fam168b	1393.090259	-0.050582018	0.068070941
ENSRNOG00000056701	Gtf2a2	250.8879417	-0.069412181	0.093266132
ENSRNOG00000010218	NEWGENE_619861	670.4817535	0.057018917	0.076684458
ENSRNOG00000012061	Prkcb	940.4047179	0.071508899	0.096365265
ENSRNOG00000007733	Arhgef9	1229.478709	-0.055445863	0.074870379
ENSRNOG00000000130	Dnajb5	440.7067902	-0.08622513	0.116659974
ENSRNOG00000030864	NA	357.6271974	0.072238759	0.097697063
ENSRNOG00000027171	NA	845.7167968	0.047838735	0.06484416

ENSRNOG00000031656	Ccnh	261.5890397	0.06719681	0.091137472
ENSRNOG00000008519	Dipk2a	195.6912421	0.094861814	0.128789151
ENSRNOG00000016722	Xpo6	287.4365685	-0.060631669	0.082413948
ENSRNOG00000020894	Lsm12	991.4815146	0.04490954	0.061095902
ENSRNOG00000006283	NA	1804.203749	-0.054292941	0.074056819
ENSRNOG00000049876	Prkar1a	606.2853399	-0.05145039	0.070154338
ENSRNOG00000050994	Cttn	316.6699921	0.064791183	0.088443422
ENSRNOG00000016635	Katnip	344.5113052	-0.071803854	0.098096117
ENSRNOG00000018184	Tpm1	1074.806166	-0.048653937	0.066514238
ENSRNOG00000001528	Gap43	2467.798614	-0.056026946	0.076733853
ENSRNOG00000019181	Myoz3	691.9833876	0.061450922	0.08413192
ENSRNOG00000019277	Vdac3	946.0445074	-0.046835017	0.064156326
ENSRNOG00000060869	Irak1	237.9714467	0.07154513	0.098072054
ENSRNOG00000000585	Amd1	742.8925442	-0.048898048	0.067189494
ENSRNOG00000000557	Sar1a	694.3990869	-0.046767146	0.064363028
ENSRNOG00000052376	Bend6	493.8749724	-0.067255204	0.09274494
ENSRNOG00000019113	Hnrnpk	1641.014785	-0.046682652	0.064352168
ENSRNOG00000030019	Atp1a1	3721.744779	0.056097205	0.077591644
ENSRNOG00000000158	Cdo1	257.9620382	-0.077260513	0.106900988
ENSRNOG00000000275	Fam13c	14777.3705	-0.114372807	0.158629651
ENSRNOG00000023360	Fus	437.6102114	-0.109946312	0.152363982
ENSRNOG00000018668	Glg1	766.1562506	-0.053539205	0.074509907
ENSRNOG00000034066	Hspa8	4535.25228	-0.039911255	0.05533524
ENSRNOG00000020694	Icam5	844.485246	-0.048852766	0.06765609
ENSRNOG00000061050	Mboat2	211.4056339	-0.071884429	0.099982034
ENSRNOG00000006901	Ndufa7	297.9624399	-0.071517035	0.098985508
ENSRNOG00000020696	Pmvk	241.1038852	0.065776576	0.091377062
ENSRNOG00000005257	Prkaca	227.2345012	-0.259189796	0.360645799
ENSRNOG00000005963	Rab2a	204.0584326	-0.089458134	0.12370085
ENSRNOG00000001124	Rnft2	613.4705804	-0.052996099	0.073427819
ENSRNOG00000055579	Tmbim6	1304.228081	-0.044537057	0.061978421
ENSRNOG00000022748	Tmem126a	243.2720965	0.066039063	0.091776235
ENSRNOG00000023383	Ddx3x	388.082146	-0.059268469	0.082648297
ENSRNOG00000003928	Tmco1	198.5804691	-0.075273594	0.104948127
ENSRNOG00000024852	Tmx4	678.6300566	-0.057987369	0.080855121
ENSRNOG00000017250	Gmpr	403.4904669	0.054492528	0.076197365
ENSRNOG00000007492	Rpn2	547.6156891	-0.16947464	0.236866123
ENSRNOG00000016257	Cotl1	422.6993732	-0.057744538	0.080815198
ENSRNOG00000061774	RGD1560986	532.9786559	-0.079572933	0.111803188
ENSRNOG00000002342	Aldh3a2	199.5040485	-0.076501581	0.108103244
ENSRNOG00000010861	Atad1	476.8131812	0.049484843	0.069919415
ENSRNOG00000050419	Avil	270.1107798	-0.069385642	0.097907383
ENSRNOG00000004060	Calm1	13918.41381	0.058789344	0.083255019
ENSRNOG00000011865	Ciao2b	222.0210113	0.091452452	0.129495805
ENSRNOG00000015633	Cul3	489.6150067	-0.050424442	0.071214957
ENSRNOG00000029726	Gstm1	577.4418392	-0.073212691	0.10383528
ENSRNOG00000006532	H3f3a	701.7272471	0.093953957	0.132980222
ENSRNOG00000058975	Iqsec2	591.2380044	0.075465341	0.10625626
ENSRNOG00000012832	Mfsd5	197.7789552	-0.081868286	0.11609889
ENSRNOG00000056559	NA	228.0010746	-0.081775398	0.115460774

ENSRNOG00000017079	Pgm2l1	621.6271682	0.062724586	0.088879146
ENSRNOG00000053766	Ramp3	213.9830258	0.079897341	0.11311814
ENSRNOG00000019776	Sh3gl3	282.4365773	0.081861308	0.115818574
ENSRNOG00000016474	Tspan3	2353.065659	-0.038699376	0.05465138
ENSRNOG00000003186	Anks3	225.3490904	-0.54734716	0.778235236
ENSRNOG00000001964	Cd47	593.434693	0.055360097	0.078729218
ENSRNOG00000011383	Ndufs4	526.3661727	-0.044644363	0.063533953
ENSRNOG00000002292	Hnrnpd	478.2817684	-0.047669794	0.067935376
ENSRNOG00000017342	Zdhhc7	260.5246184	0.063532913	0.090556686
ENSRNOG00000015335	Rpl13	3993.078303	-0.046447834	0.066286036
ENSRNOG00000016177	Scara3	206.669354	-0.072548479	0.103553897
ENSRNOG00000013000	Ldhb	4094.760034	-0.041723714	0.059692084
ENSRNOG00000030106	NA	228.3482135	-0.062504604	0.089615746
ENSRNOG00000005346	Tmem128	203.749336	0.079355001	0.113758594
ENSRNOG00000009266	Anp32b	398.0291074	-0.060414562	0.086767788
ENSRNOG00000019556	Cd9	282.3882227	0.058489449	0.084367039
ENSRNOG00000009275	Kpnb1	496.6523026	0.050681254	0.073080937
ENSRNOG00000012015	Lrrc49	378.1863967	0.060977232	0.087688198
ENSRNOG00000007334	Ly6h	3033.978138	-0.054684438	0.078874618
ENSRNOG00000017552	NA	219.9896052	-0.068475244	0.098840801
ENSRNOG00000007407	Ndufa12	618.3864265	-0.044496774	0.064113896
ENSRNOG00000008902	Pon1	285.9281754	0.072972387	0.10527274
ENSRNOG00000014816	Slc1a1	407.4083306	0.059279629	0.085467245
ENSRNOG00000008597	Tada3	216.0352041	-0.082026994	0.117791037
ENSRNOG00000002763	Ulk2	265.2123093	0.065732904	0.09469566
ENSRNOG00000002506	Cdkl2	326.9143048	0.073463962	0.106122053
ENSRNOG00000008167	Abhd6	309.0243623	0.056231923	0.081439614
ENSRNOG00000000157	Atg12	388.4850536	0.059930197	0.086946315
ENSRNOG00000013869	Kcnj4	394.4580645	-0.071075495	0.10281088
ENSRNOG00000017284	Nop16	193.0877052	0.072834139	0.105623424
ENSRNOG00000020452	Nsmce4a	253.5637242	-0.060741851	0.088016605
ENSRNOG00000001223	Pttg1ip	447.3243067	-0.057750961	0.08355576
ENSRNOG00000014604	Sigmar1	275.1800184	-0.06703835	0.09713874
ENSRNOG00000042821	Cd59	445.789523	-0.047448488	0.069176624
ENSRNOG00000055319	NA	1761.936295	-0.03051718	0.044462715
ENSRNOG00000038948	Stab2	250.2172368	0.092019516	0.133997888
ENSRNOG00000010303	Trim32	443.0106472	-0.056188909	0.081871217
ENSRNOG00000016592	Gnai2	1916.35165	-0.058977065	0.086105903
ENSRNOG00000021175	Otub1	390.8182909	-0.184980841	0.270100467
ENSRNOG00000027015	Ythdf1	312.5485829	-0.061222988	0.089436934
ENSRNOG00000012204	AU040320	226.1690959	0.073576376	0.107775474
ENSRNOG00000007782	Ghrh	222.2074149	-0.256955922	0.376178459
ENSRNOG00000016394	Wdr77	297.4586268	0.061637539	0.090180833
ENSRNOG00000016043	Aqp4	1001.088586	0.063370471	0.093477454
ENSRNOG00000015602	Cdh2	400.5788507	-0.052645906	0.077416078
ENSRNOG00000013442	Ciz1	731.1043209	-0.046754584	0.068963843
ENSRNOG00000016326	Cx3cl1	2801.444154	-0.030665927	0.045189263
ENSRNOG00000009170	Dmxl2	367.6025802	0.067965559	0.099718688
ENSRNOG00000002667	Lamc2	375.3691264	0.062511254	0.092213016
ENSRNOG00000009205	Lmo4	3467.163904	-0.048060462	0.070778345

ENSRNOG00000009280	Mkrn1	530.2851568	-0.049913254	0.073628751
ENSRNOG00000051377	NA	240.9783232	-0.067966723	0.100004604
ENSRNOG00000001057	NA	467.3818036	0.563492009	0.83094215
ENSRNOG00000004719	Pp2d1	232.4346244	-0.073638487	0.107972015
ENSRNOG00000027295	Aph1b	557.2108206	0.064238677	0.094874857
ENSRNOG00000019267	Dkk4	212.1143867	0.065947594	0.097491505
ENSRNOG00000053334	Stmn4	899.9017273	0.084220516	0.124545637
ENSRNOG00000003506	Gosr2	256.0071744	-0.072791278	0.108164379
ENSRNOG00000016322	NA	6692.793412	0.071195393	0.105713525
ENSRNOG00000054685	NA	730.8138221	0.414983478	0.61626245
ENSRNOG00000012053	S100a16	595.1042914	-0.066486087	0.098801164
ENSRNOG00000015080	Wdfy1	289.7518793	-0.057613059	0.0855766
ENSRNOG00000021691	Ccdc92	644.7079962	-0.045136236	0.067139161
ENSRNOG00000014480	Dbndd2	245.7536109	0.066133516	0.09863747
ENSRNOG00000002262	Enoph1	396.2073116	0.050561008	0.075408358
ENSRNOG00000054197	NA	927.7257688	0.397244233	0.592410271
ENSRNOG00000007254	Ttc9	235.2020999	-0.066779024	0.099439877
ENSRNOG00000017852	Nars1	391.2979519	0.049528263	0.073945998
ENSRNOG00000020407	Atcay	205.8775985	-0.085689643	0.128298732
ENSRNOG00000011422	Atmin	208.1485405	-0.06933805	0.10375615
ENSRNOG00000037983	Car15	336.3184229	0.059369723	0.088783294
ENSRNOG00000053350	NA	740.7345898	0.412967045	0.618535452
ENSRNOG00000029735	Pid1	357.3944863	0.050502117	0.075719057
ENSRNOG00000020213	Tubg1	266.6728314	-0.057946909	0.086863337
ENSRNOG00000000956	Rasl11a	222.3220606	-0.179609821	0.270179935
ENSRNOG00000011891	Atp6v1b2	2583.82182	0.033923292	0.051119722
ENSRNOG00000011052	Gars	776.7542625	-0.050183876	0.075620134
ENSRNOG00000011501	Atp1b3	699.6422226	0.050651848	0.076466549
ENSRNOG00000009079	Prkar2b	320.568139	-0.060671458	0.091546602
ENSRNOG00000020897	Prpf19	449.5527607	0.057000213	0.086146945
ENSRNOG00000046871	2310039H08Rik	253.2220652	0.062726156	0.095763548
ENSRNOG00000019180	Acsl4	412.6105756	-0.045554037	0.06960768
ENSRNOG00000048981	Ahsa1	431.9521335	0.052142994	0.07924474
ENSRNOG00000008786	Ap1b1	524.2095301	0.05325791	0.080930885
ENSRNOG00000007546	Asns	219.0172571	0.070772388	0.107755257
ENSRNOG00000004742	Chp1	950.9998381	-0.046918002	0.071441151
ENSRNOG00000048472	Comp	459.7576942	0.054946232	0.083559436
ENSRNOG00000000111	Coprs	371.8548787	-0.057192903	0.087435052
ENSRNOG00000011933	Csnk2a2	412.9053784	-0.069503288	0.105340564
ENSRNOG00000030771	Dgkb	225.6638826	0.070815235	0.108182797
ENSRNOG00000027099	Fbxl2	315.3511101	0.054739638	0.083557591
ENSRNOG00000018077	Gpat4	231.6053551	0.067591638	0.102853758
ENSRNOG00000012156	Ostf1	206.8264325	0.063236488	0.096023427
ENSRNOG00000013454	Rala	374.3624205	-0.076276321	0.116626722
ENSRNOG00000018841	Sox8	199.7392906	-0.064819367	0.098928063
ENSRNOG00000009351	Srp68	285.3902559	0.055659056	0.084364521
ENSRNOG00000007439	Unc13d	1643.103352	-0.052009799	0.079326895
ENSRNOG00000017040	Wrnip1	279.9297469	-0.07094521	0.107904257
ENSRNOG00000024924	Cep19	385.6916669	0.051037346	0.078104387
ENSRNOG00000029571	Coq10a	1322.461491	0.04165401	0.063949112

ENSRNOG00000004735	Gzf1	2084.643065	-0.042600282	0.065372145
ENSRNOG00000009691	Lrrn2	789.1189409	-0.041034786	0.063070946
ENSRNOG00000009281	NA	441.8908159	-0.051916387	0.079615235
ENSRNOG00000028993	Rpl12	680.9956386	-0.141161564	0.217253403
ENSRNOG00000017832	Snx2	394.9602914	-0.054306125	0.083206869
ENSRNOG00000020269	Sugp2	215.7850655	0.068638637	0.105639972
ENSRNOG00000012379	Wdr18	310.7836717	-0.062532676	0.09612156
ENSRNOG00000049743	Gstm3	482.444866	-0.058371081	0.090132786
ENSRNOG00000013313	Nceh1	352.6940966	-0.057713277	0.08907569
ENSRNOG00000014272	Rpl35	196.2879241	-0.664431644	1.025079581
ENSRNOG00000010538	Commd7	202.5961945	0.06166917	0.095346007
ENSRNOG00000042523	NA	445.5466449	-0.04746922	0.073424109
ENSRNOG00000006839	Arl5a	399.9545927	0.045964351	0.071322384
ENSRNOG00000014637	Atxn10	1175.758687	-0.039912971	0.061928205
ENSRNOG00000018214	Bok	225.1384715	0.063367427	0.098415007
ENSRNOG00000010834	Mpped1	1794.701477	0.038054753	0.058987863
ENSRNOG00000022860	Swi5	398.2846613	-0.083608971	0.129875269
ENSRNOG00000004143	Adipor1	612.1990094	-0.047659701	0.074631818
ENSRNOG00000004481	Adss	230.7411291	-0.059840296	0.093914313
ENSRNOG00000021866	Bola3	317.5192405	0.059398527	0.092741446
ENSRNOG00000002863	Cacna1e	641.8422694	-0.054894393	0.086500198
ENSRNOG00000003262	Cacng4	280.2708089	0.064067297	0.100760204
ENSRNOG00000000397	Ccar1	196.848127	0.077462749	0.121689896
ENSRNOG00000018932	Ccdc124	380.5711385	-0.052336765	0.082133568
ENSRNOG00000008214	Fbxo9	931.0784951	0.039142862	0.061449952
ENSRNOG00000027230	Fhod3	240.4225967	0.079151334	0.123926473
ENSRNOG00000047891	Foxg1	688.1813147	0.037165731	0.05843631
ENSRNOG00000011728	Fto	688.0035492	-0.058589898	0.092405214
ENSRNOG00000019425	Gabarapl2	1896.725089	-0.034551081	0.053861094
ENSRNOG00000016717	Gas2	292.2416795	-0.066312976	0.104402027
ENSRNOG00000014634	Gtpbp1	210.4824543	-0.080685509	0.126629739
ENSRNOG00000013781	Kcnq5	318.5857436	0.053597333	0.084588579
ENSRNOG00000016749	Klhl10	491.5855496	0.076371842	0.11996813
ENSRNOG00000026180	Letm2	379.3369573	0.051568258	0.080470426
ENSRNOG00000004810	Plcb1	202.1272065	-0.076140007	0.118920393
ENSRNOG00000005015	Rabep1	197.1617654	-0.083889062	0.131081898
ENSRNOG00000014352	Smim12	299.0629584	-0.062082716	0.09771552
ENSRNOG00000009370	Tbkbp1	215.8933447	0.068484326	0.10769668
ENSRNOG00000022615	Tp53i13	2219.649047	-0.046465031	0.073285882
ENSRNOG00000003723	Wdr26	325.645623	-0.050525039	0.079416419
ENSRNOG00000020463	Eif1ad	215.890113	-0.072670843	0.114926717
ENSRNOG00000030515	Nfasc	426.7916456	0.066180005	0.104724965
ENSRNOG00000010944	Hyou1	249.5908318	-0.056905327	0.090522811
ENSRNOG00000017730	Psmd1	641.45625	-0.046134829	0.073373524
ENSRNOG00000029095	Trabd	236.1523902	-0.059750833	0.09507403
ENSRNOG00000009285	Eipr1	220.3838094	0.057449722	0.091638646
ENSRNOG00000042309	Kiaa0408L	296.4784992	0.064311671	0.102684834
ENSRNOG00000026055	Neurod6	640.6277846	-0.05649607	0.09014786
ENSRNOG00000001803	Dnajb11	333.0277618	-0.061890493	0.099048598
ENSRNOG00000012362	Cacng3	975.4696138	-0.045164834	0.073107889

ENSRNOG00000019374	Csk	318.3441665	-0.053547306	0.086344613
ENSRNOG00000005428	Ctbp1	1582.266355	0.041725631	0.067363244
ENSRNOG00000000841	Ddx39b	785.6812117	-0.047988305	0.07759222
ENSRNOG00000016161	Lman2	220.8563683	-0.062897997	0.101899702
ENSRNOG00000011751	Ncdn	3002.724081	0.041935494	0.067940022
ENSRNOG00000010688	Nrgn	2863.25961	-0.291585146	0.472445364
ENSRNOG00000002546	Rchy1	251.2622877	-0.064521728	0.103951773
ENSRNOG00000020771	Sap18	303.0517748	0.053319948	0.086264323
ENSRNOG00000015393	Slc32a1	372.220295	-0.045503546	0.073460773
ENSRNOG00000027468	Slc6a15	320.1942646	-0.060743867	0.098064132
ENSRNOG00000004200	Sybu	446.9046057	-0.047605604	0.076752507
ENSRNOG00000012405	Tcf4	822.2949307	-0.055646498	0.089803342
ENSRNOG00000061099	AABR07033324.1	5939.463238	-0.037096408	0.060162957
ENSRNOG00000004496	Rock2	312.0489535	-0.069040974	0.112028799
ENSRNOG00000012364	Prickle2	472.1732613	-0.046807646	0.076219612
ENSRNOG00000001030	Tsc22d1	2645.93951	0.049891331	0.081262292
ENSRNOG00000049629	Reep2	1299.781285	-0.039568286	0.064544672
ENSRNOG00000018271	Scamp4	261.9876582	0.062394158	0.101732191
ENSRNOG00000014615	Grk6	324.1956274	-0.062535935	0.10268332
ENSRNOG00000016957	Igfbp2	243.8428518	0.156867773	0.257429162
ENSRNOG00000020526	Mau2	238.8263355	0.072937313	0.119383679
ENSRNOG00000017074	Rab28	447.0411893	0.043210603	0.071020566
ENSRNOG00000017151	Rnf214	502.0571811	0.054704484	0.089568572
ENSRNOG00000005007	Scn3a	208.9428301	-0.081151763	0.1331951
ENSRNOG00000018487	Slc3a2	1260.868409	-0.047945649	0.078813803
ENSRNOG00000001439	Srrm3	226.0080584	0.065409995	0.106957744
ENSRNOG00000000262	Zfp821	263.5249254	0.061314275	0.10047234
ENSRNOG00000026941	Tril	301.4675885	0.066894964	0.110073984
ENSRNOG00000010553	Pnma1	286.133335	-0.062007848	0.102367458
ENSRNOG00000005271	Rapgef5	314.1284391	0.052314783	0.08628133
ENSRNOG00000008392	Sbf1	334.1343683	0.088089175	0.145353151
ENSRNOG00000011540	Vta1	251.0972601	-0.059601972	0.098241944
ENSRNOG00000048812	Gpx1	233.9186314	0.073559826	0.121539224
ENSRNOG00000003782	Acot9	252.9004882	0.050586353	0.085029924
ENSRNOG00000014454	Ap1m1	588.2753723	0.062805229	0.105046159
ENSRNOG00000061543	Ap2b1	1078.009976	-0.037255834	0.062290755
ENSRNOG00000015732	Bcl2l2	633.8711485	0.03463946	0.058518196
ENSRNOG00000056207	Capza2	835.105014	0.038927275	0.065620703
ENSRNOG00000014208	Dctn3	275.4819422	-0.256316311	0.426293758
ENSRNOG00000018848	Eif1b	1076.260661	-0.033285829	0.055761927
ENSRNOG00000014303	Lrp11	2368.0847	-0.033955962	0.056561238
ENSRNOG00000014230	Map1a	791.4263753	-0.075027917	0.125328365
ENSRNOG00000006705	Mkks	351.1983403	0.052654466	0.088566027
ENSRNOG00000049248	NA	316.1057567	-0.051693182	0.086664657
ENSRNOG00000007419	Pank3	248.353627	0.072756374	0.122780545
ENSRNOG00000014690	Pigt	598.6920772	-0.049653473	0.083875385
ENSRNOG00000008116	Plpp3	273.2680365	-0.089757325	0.149874278
ENSRNOG00000048297	Qk	843.6575334	-0.081947384	0.136153369
ENSRNOG00000007648	Rab11b	1667.698776	-0.029758081	0.049664393
ENSRNOG00000006763	Rbm18	306.2373108	-0.051112706	0.086141029

ENSRNOG00000028436	Rprml	426.9030739	0.049017435	0.082927399
ENSRNOG00000051850	Scarna2	271.7764727	0.051676428	0.087309158
ENSRNOG00000015143	Siah1	310.2014626	-0.054964685	0.091789317
ENSRNOG00000007284	Slc2a1	372.948688	-0.064943224	0.10833012
ENSRNOG00000018746	Slc30a5	194.0986944	0.072785133	0.123039794
ENSRNOG00000017838	Snrnp27	308.5706432	-0.053447025	0.089525608
ENSRNOG00000046984	St6galnac6	786.4191193	0.039835944	0.066635871
ENSRNOG00000013351	Stxbp5	432.4656577	0.047349003	0.079767966
ENSRNOG00000030776	Sytl2	254.023929	0.072045722	0.121285217
ENSRNOG00000028585	Tceal8	404.1748848	-0.048845172	0.081027646
ENSRNOG00000013238	Tmem14a	803.0354057	0.040975844	0.068118558
ENSRNOG00000050317	Uhrf1bp1l	262.4191846	-0.049818768	0.084256813
ENSRNOG00000008038	Wbp2nl	1976.680587	-0.027181659	0.045455366
ENSRNOG00000020330	Wdr47	437.4091341	0.050545161	0.085352968
ENSRNOG00000034093	AABR07011951.1	4364.906066	-0.038397513	0.065168183
ENSRNOG00000025679	Stk40	265.5061862	-0.065575729	0.111446421
ENSRNOG00000019968	Trim8	517.83679	0.044900759	0.076270126
ENSRNOG00000012803	Wdr61	795.7365797	0.037952055	0.064470875
ENSRNOG00000000996	Arpc1a	1306.877645	-0.034567536	0.058916228
ENSRNOG00000021318	Epas1	449.0916243	0.053647322	0.091765896
ENSRNOG00000016244	Mical2	1324.234624	0.033916154	0.057787066
ENSRNOG00000050514	NA	197.5515738	0.065668362	0.112219516
ENSRNOG00000008565	Nkiras1	519.5889724	-0.040123414	0.068708537
ENSRNOG00000010409	Nol6	246.2120082	0.067908455	0.115698945
ENSRNOG00000050220	Nrxn1	792.3109636	0.040767873	0.069583081
ENSRNOG00000042195	Pabpn1	1410.169963	0.035878697	0.061253889
ENSRNOG00000017843	Polr3k	305.816261	-0.050377503	0.086213615
ENSRNOG00000025324	Spire1	590.3717474	0.041109615	0.070298319
ENSRNOG00000046159	Gns	296.9649017	-0.048371821	0.083059863
ENSRNOG00000017194	Prdx1	224.9998447	0.089844252	0.154271111
ENSRNOG00000017659	Hs3st2	309.2170165	-0.053822936	0.092639563
ENSRNOG00000011158	Ppp2r2a	493.552214	-0.046894179	0.080773259
ENSRNOG00000045605	Uxs1	214.6774611	-0.056503351	0.097452005
ENSRNOG00000007237	Id2	892.3573833	-0.062173246	0.10745534
ENSRNOG00000009987	Akap11	656.0930381	0.036275528	0.062853918
ENSRNOG00000058050	Bclaf1	317.6909486	0.050488871	0.087504653
ENSRNOG00000014516	Hs6st1	219.2419386	0.061180716	0.106312531
ENSRNOG00000014153	Lhfpl6	497.6636209	-0.040796026	0.070721499
ENSRNOG00000058658	Mtch2	421.505171	0.043336922	0.075219181
ENSRNOG00000028856	Pknox2	263.6990392	0.053079797	0.092309445
ENSRNOG00000006695	Rbck1	297.0520759	-0.052291787	0.090517224
ENSRNOG00000003201	Rps4x	327.5644744	-0.075530647	0.131304298
ENSRNOG00000000708	Sgsm1	349.7431645	0.047129738	0.081989457
ENSRNOG00000057186	Kctd5	383.4953609	0.056794839	0.098874755
ENSRNOG00000029798	Lrrc4c	398.3139915	-0.045111292	0.078808659
ENSRNOG00000003178	NA	230.7308449	-0.063102754	0.110303756
ENSRNOG00000017003	Plxna1	303.3444549	0.05111413	0.089321892
ENSRNOG00000000957	Rpl21	3169.772679	-0.123742311	0.216189632
ENSRNOG00000022273	Rfk	332.3253657	-0.080868804	0.1416182
ENSRNOG00000008000	Syt13	758.6819493	-0.041502627	0.072718744

ENSRNOG00000019727	NA	252.3517655	-0.050341783	0.088355328
ENSRNOG00000053659	RGD1311703	606.3148538	-0.042791874	0.075388327
ENSRNOG00000007462	Septin8	1670.896416	0.033596234	0.05919037
ENSRNOG00000004936	Sdc2	199.7682237	0.056945683	0.10040377
ENSRNOG00000004998	Jakmip1	550.7574892	0.048909531	0.086559146
ENSRNOG00000004726	Mapkapk2	268.9662153	0.053480355	0.094607886
ENSRNOG00000061685	NA	332.5248472	0.324303216	0.573304306
ENSRNOG00000002807	Septin9	416.1277158	-0.041077686	0.072583104
ENSRNOG00000010676	Smarce1	325.9301568	-0.049850844	0.088087503
ENSRNOG00000001647	Ets2	369.45778	-0.054752657	0.097060902
ENSRNOG00000020339	Neurl1	1642.585083	0.045284153	0.080274099
ENSRNOG00000033748	AABR07060610.1	597.001133	-0.145636835	0.260827448
ENSRNOG00000006997	App	3701.217201	-0.037678145	0.067557319
ENSRNOG00000007100	Ccdc136	405.6177812	0.040703216	0.073056808
ENSRNOG00000018207	Dynlt1	303.4306252	0.060253596	0.108019967
ENSRNOG00000010800	Hadhb	466.5952234	-0.041219695	0.073974525
ENSRNOG00000023176	Kidins220	495.9395028	0.047204553	0.084313727
ENSRNOG00000019804	NA	220.0861346	-0.054278959	0.096795309
ENSRNOG00000032885	NA	222.7398981	-0.060011543	0.107149312
ENSRNOG00000008939	Nxph1	192.6395526	0.058300004	0.103493435
ENSRNOG00000043094	Oxct1	1036.808416	0.031924788	0.057274246
ENSRNOG00000017708	Pcbp1	831.5969818	-0.035156761	0.062833374
ENSRNOG00000005726	Pclo	238.6131864	-0.090402634	0.161056271
ENSRNOG00000039551	Rdh14	196.4859368	0.060784074	0.108639059
ENSRNOG00000014338	Slc25a25	206.2667493	0.071623779	0.127574756
ENSRNOG00000001139	Suds3	615.1865449	-0.045879388	0.082322574
ENSRNOG00000007527	Tm2d1	516.5663694	0.044683272	0.079374575
ENSRNOG00000005610	Yipf4	302.4852034	0.054364657	0.096602582
ENSRNOG00000017297	Cab39	740.9252499	0.032361009	0.058550966
ENSRNOG00000009538	Etfdh	210.142438	-0.050699382	0.091829508
ENSRNOG00000048194	NA	391.9018586	0.044686661	0.081027145
ENSRNOG00000003033	Plcd3	279.2429584	-0.046469429	0.084235204
ENSRNOG00000018494	Ppp1r3c	232.752392	-0.057689206	0.104563111
ENSRNOG00000048824	Rnf165	224.0899019	0.064815383	0.117571483
ENSRNOG00000002575	Sap30l	200.3125846	-0.061135435	0.11084902
ENSRNOG00000005550	Lrfn5	281.3050021	0.049580833	0.090024495
ENSRNOG00000007091	Ly6e	1321.649632	-0.035923628	0.065335728
ENSRNOG00000020373	Dap3	224.2727796	-0.05005195	0.091216703
ENSRNOG00000005397	Maea	613.2935689	0.043556959	0.079381412
ENSRNOG00000022429	Minar2	400.6077617	-0.040736074	0.074424284
ENSRNOG00000022417	Prickle3	5225.824084	-0.034869265	0.063715496
ENSRNOG00000043141	Ap3s2	254.1016181	-0.04841955	0.090023114
ENSRNOG00000004452	Aqp12a	2006.956683	-0.039844064	0.074947163
ENSRNOG00000010838	Araf	558.9541768	-0.041591045	0.077398959
ENSRNOG00000025730	Armcx3	321.5285575	-0.044996916	0.084264953
ENSRNOG00000030862	Atp6v1h	1042.20032	-0.03961976	0.073007718
ENSRNOG00000047296	Bptf	242.3735638	0.057010711	0.107062128
ENSRNOG00000007456	Calb1	460.9049422	-0.06273268	0.117919082
ENSRNOG00000008672	Chmp5	762.8252778	0.029117032	0.053552649
ENSRNOG00000046889	Dbi	1259.646036	-0.036180705	0.067996485

ENSRNOG00000058790	Fam120b	401.4686583	0.041649381	0.077316089
ENSRNOG00000010803	Gabra5	490.626992	-0.055578844	0.104763306
ENSRNOG00000051482	Hmg1l1	306.2692874	-0.052076881	0.097793107
ENSRNOG00000013809	Kbtbd2	207.6886613	-0.052281006	0.096775732
ENSRNOG00000055401	Kcnc1	263.6215174	0.047297135	0.088553787
ENSRNOG00000020746	Lin7b	495.1550467	0.040448622	0.074877225
ENSRNOG00000011555	Lrrc40	343.8804467	0.055991422	0.104511557
ENSRNOG00000040384	Mir770	1934.897144	0.12521986	0.229354893
ENSRNOG00000007120	Mtmr3	294.6878768	0.051017408	0.093634479
ENSRNOG00000047067	NA	887.0038405	-0.038864921	0.072063191
ENSRNOG00000061438	NA	2331.884017	-0.036106641	0.067870532
ENSRNOG00000045877	NA	567.7531744	-0.038214232	0.072085043
ENSRNOG00000015675	Nell1	210.2895266	-0.048767305	0.091547353
ENSRNOG00000046912	Nr1d2	339.9306134	-0.048107639	0.090267726
ENSRNOG00000009756	Pacsin2	192.188343	0.059629698	0.110192056
ENSRNOG00000001747	Pak2	269.4852855	-0.047992967	0.088975224
ENSRNOG00000001628	Pcp4	820.6880995	-0.060272996	0.111551596
ENSRNOG00000006655	Plrg1	196.6304933	0.055760436	0.103586354
ENSRNOG00000047686	Pou3f1	351.1262779	0.061194942	0.113082737
ENSRNOG00000017692	Ppp1r37	321.6124065	0.048860335	0.089405525
ENSRNOG00000021581	Rapgef2	446.5625663	0.04199507	0.079055521
ENSRNOG00000012153	Rbl2	206.3943704	0.053008109	0.099392013
ENSRNOG00000018767	Rbm17	223.0509739	-0.050036846	0.094244476
ENSRNOG00000030568	Rgs12	326.8539246	-0.050517125	0.094648958
ENSRNOG00000018567	Slc20a1	219.3290318	-0.057279355	0.10539347
ENSRNOG00000016507	Snrpg	249.5147705	-0.04648684	0.085921195
ENSRNOG00000015093	Sparcl1	1048.516514	-0.039980913	0.073781186
ENSRNOG00000003562	Susd4	518.7041707	-0.037385903	0.069591509
ENSRNOG00000010895	Tmem30a	210.9150651	0.055631358	0.104705609
ENSRNOG00000000900	Tpst1	192.4458326	0.061973017	0.1152177
ENSRNOG00000027088	Ube2k	721.1908229	-0.037064347	0.069171962
ENSRNOG00000014765	Vapa	1750.304623	-0.036083093	0.066244711
ENSRNOG00000025462	Wdr41	209.678947	-0.049753097	0.092926272
ENSRNOG00000039850	Pigp	197.9877467	0.054589236	0.103228566
ENSRNOG00000053804	Prmt8	388.857288	-0.04401786	0.083209949
ENSRNOG00000007147	Cyp46a1	1928.946844	-0.036478468	0.069248719
ENSRNOG00000020431	Matk	332.0248162	0.124602187	0.237374941
ENSRNOG00000026238	NA	284.8665942	0.079780208	0.151241872
ENSRNOG00000050450	NA	633.3206864	0.05031588	0.095477631
ENSRNOG00000004973	Ppp2r5c	898.7621178	-0.033479477	0.063792371
ENSRNOG00000050223	Rin1	272.2828655	-0.050890765	0.096647522
ENSRNOG00000014311	Slc7a8	267.489763	-0.05800578	0.11021604
ENSRNOG00000047505	Tubb4a	399.8758938	-0.116312896	0.221443965
ENSRNOG00000008108	Ubr7	196.7472702	0.059734314	0.113711422
ENSRNOG00000021168	Anp32e	400.3847678	-0.038793218	0.074095336
ENSRNOG00000006444	Fkbp4	973.3754641	-0.038404589	0.073508939
ENSRNOG00000045844	Impact	1176.66062	-0.03120095	0.05968416
ENSRNOG00000010164	Mrps2	281.4029885	-0.042337001	0.081041174
ENSRNOG00000008569	Ndufa6	347.5925346	-0.051516913	0.098736501
ENSRNOG00000042592	Rgs10	581.5885202	-0.038657092	0.074063874

ENSRNOG00000024336	Senp6	230.4125877	0.053177073	0.102037012
ENSRNOG00000055089	Slc44a1	336.0646503	0.047054745	0.090302699
ENSRNOG00000011517	Tnfrsf21	264.6525358	0.062140517	0.119582307
ENSRNOG00000018569	Ahcyl1	727.9414207	0.031901832	0.061524839
ENSRNOG00000009820	Bnip3l	975.7395685	0.032226146	0.062703541
ENSRNOG00000042179	Cfap20	232.6676731	-0.055665488	0.108273135
ENSRNOG00000052305	Cnot10	205.1122589	0.063494126	0.123537062
ENSRNOG00000005927	Cpsf6	331.3912668	0.052615536	0.101982041
ENSRNOG00000009414	Creld1	278.3625632	-0.05283405	0.102334182
ENSRNOG00000016156	Nptxr	5013.409112	-0.026356582	0.051298059
ENSRNOG00000007673	Ppig	259.0623922	-0.052156846	0.101047203
ENSRNOG00000019435	Psd	1211.661311	0.258493387	0.501827241
ENSRNOG00000001061	Rilpl2	575.546466	0.057700917	0.11215509
ENSRNOG00000014093	Tom1	240.0569683	0.057497595	0.111884505
ENSRNOG00000017502	Sike1	199.3268198	0.051069488	0.099489366
ENSRNOG00000025235	Tmem130	3417.677647	-0.044895393	0.087574115
ENSRNOG00000011541	Cygb	304.9812555	-0.042458603	0.083410139
ENSRNOG00000021298	Dstyk	242.8747644	-0.048848491	0.095820155
ENSRNOG00000009681	Flot2	373.6251951	0.039717049	0.077707265
ENSRNOG00000018018	Kcnip2	633.9272732	0.048652772	0.095578379
ENSRNOG00000016000	NA	2120.022246	-0.029552825	0.057904041
ENSRNOG00000000934	Piwil1	381.756807	0.044184223	0.086712057
ENSRNOG00000007671	Smu1	539.5249168	0.04626382	0.090403734
ENSRNOG00000005686	Suclg2	241.4199392	0.061627961	0.120682416
ENSRNOG00000047476	Wasf1	1449.49693	-0.037026317	0.072329167
ENSRNOG00000016133	Sumo1	557.0725488	-0.038238026	0.07518875
ENSRNOG00000036318	NA	644.5976626	0.041352039	0.081655837
ENSRNOG00000013234	Ccdc115	250.0882329	-0.05070272	0.100564654
ENSRNOG00000000007	Gad1	892.143468	-0.035496948	0.070383947
ENSRNOG00000012962	Nudt16	193.7807337	-0.059480086	0.117952828
ENSRNOG00000017136	Syt17	803.3945877	-0.044313874	0.087628791
ENSRNOG00000005377	Tmem11	227.5562371	-0.048643219	0.096344019
ENSRNOG00000053456	NA	293.301966	-0.041339614	0.082112749
ENSRNOG00000026799	Zbtb9	855.9254697	0.034893199	0.069328373
ENSRNOG00000061246	Cyrib	499.8518245	0.035066101	0.070013615
ENSRNOG00000037658	Gprasp2	425.6476949	-0.039292341	0.0784528
ENSRNOG00000000164	Lamp2	220.2085878	-0.046674188	0.093251667
ENSRNOG00000003812	Nlgn3	358.2435469	0.042803932	0.085427628
ENSRNOG00000049181	Sh2b1	399.6414718	0.040900151	0.08148126
ENSRNOG00000020777	Zfp382	280.9779693	-0.128596822	0.257289361
ENSRNOG00000020460	Banf1	219.4470216	-0.047039442	0.094471899
ENSRNOG00000026048	Gbf1	224.8120873	0.043022199	0.086401295
ENSRNOG00000016122	Hmgcr	323.3343293	0.057502637	0.115217096
ENSRNOG00000028366	NA	269.8185107	0.042356588	0.084941422
ENSRNOG00000016939	Chmp7	319.5367733	0.0470175	0.094610595
ENSRNOG00000027259	Faxdc2	573.1236863	0.038549841	0.077609976
ENSRNOG00000036814	Atp6v0a1	1364.129088	-0.027729753	0.055996865
ENSRNOG00000001682	Ttc3	1194.003786	-0.047281436	0.095597899
ENSRNOG00000027341	Tenm2	457.9141419	0.049609866	0.100395038
ENSRNOG00000015686	Cert1	365.5265177	0.046228802	0.093737459

ENSRNOG00000011060	Unc119	219.6688737	-0.056758774	0.115071384
ENSRNOG00000008223	Cnr1	699.1561892	0.031775367	0.064553102
ENSRNOG00000016863	Pnma8b	250.2366587	-0.188200038	0.382146531
ENSRNOG00000018994	Psmc4	382.8748069	-0.043792371	0.089022347
ENSRNOG00000006853	Elavl2	192.1862438	0.051443053	0.104775788
ENSRNOG00000031590	NA	1546.404923	-0.031885338	0.064986525
ENSRNOG00000005096	Bzw2	312.7944151	-0.048929079	0.100359375
ENSRNOG00000009934	Emc3	399.723604	0.038768626	0.079584508
ENSRNOG00000020248	Ina	1930.38261	-0.031786571	0.065279935
ENSRNOG00000054467	NA	264.0367574	0.045609147	0.093610528
ENSRNOG00000023841	NA	313.5702786	0.051461474	0.105758147
ENSRNOG00000042855	Ahdc1	221.4929911	-0.054710509	0.112534904
ENSRNOG00000015445	Mal	316.5295301	-0.079842375	0.164602363
ENSRNOG00000004863	Mpped2	302.0914608	-0.039511628	0.081441484
ENSRNOG00000052909	NA	211.8414406	0.04930386	0.101723533
ENSRNOG00000007646	Sipa1l1	317.6003438	0.049769382	0.102766784
ENSRNOG00000009768	NA	373.8395956	-0.04441216	0.091920206
ENSRNOG00000045949	Faah	224.5732897	-0.052414497	0.108742472
ENSRNOG00000002336	Gabra4	384.9517257	-0.044688149	0.092882108
ENSRNOG00000059866	NA	385.5520062	-0.064254833	0.133276182
ENSRNOG00000012138	NA	273.4577676	-0.045788523	0.095155435
ENSRNOG00000003853	Scoc	1381.740752	-0.033948189	0.070504689
ENSRNOG00000014340	Pdcd7	197.6343951	0.049388622	0.102763237
ENSRNOG00000016315	Cnpy3	410.2235543	0.036933643	0.0771192
ENSRNOG00000013484	Gsta1	368.2606067	-0.055018689	0.114771945
ENSRNOG00000048308	NA	195.1023539	-0.049445659	0.103181039
ENSRNOG00000052256	Dact3	1691.978222	0.036761864	0.07695826
ENSRNOG00000058561	Srrm2	310.0918914	0.058359273	0.122125771
ENSRNOG00000002926	Uap1	242.4310831	0.051560802	0.107835217
ENSRNOG00000001222	Ube2g2	289.3155671	0.040479867	0.084821511
ENSRNOG00000038218	NA	519.5860791	0.043411583	0.091188728
ENSRNOG00000007882	Ablim2	241.401011	0.043454453	0.092243329
ENSRNOG00000005063	Atl1	887.9534244	-0.033092667	0.070507991
ENSRNOG00000018778	Cadm1	322.3186313	0.048929507	0.104338499
ENSRNOG00000007834	Cand1	232.4883324	-0.045493471	0.096595829
ENSRNOG00000016227	Glrx3	624.1447681	-0.029756675	0.063062826
ENSRNOG00000015173	Mbtps1	343.7141106	0.040354553	0.085750251
ENSRNOG00000046299	NA	2120.351164	-0.027718705	0.058477524
ENSRNOG00000055154	NA	275.4783534	-0.068369235	0.144273347
ENSRNOG00000015084	Necab2	654.7006798	-0.039477186	0.083388351
ENSRNOG00000014806	Pnkd	418.6850894	0.038733529	0.082690253
ENSRNOG00000001733	Ppp1r2	753.645414	-0.03162042	0.067313224
ENSRNOG00000000237	RGD1310429	250.8775904	-0.058111593	0.123175345
ENSRNOG00000029598	Robo2	297.361894	0.044741172	0.09537939
ENSRNOG00000061579	Rpl12-ps1	307.3962347	-0.218074273	0.459850863
ENSRNOG00000018920	Slc25a17	661.9935709	0.040834198	0.087158956
ENSRNOG00000015019	Spg21	361.264451	-0.040467446	0.085967194
ENSRNOG00000010007	Srsf4	331.8896828	-0.043755132	0.09263612
ENSRNOG00000021026	Zfp687	251.3649265	0.051261948	0.109280333
ENSRNOG00000011627	Zfr	874.4032546	0.030444019	0.064760319

ENSRNOG00000016460	Clu	8627.36303	-0.034682901	0.074233586
ENSRNOG00000019023	Tars1	214.6259183	0.045916967	0.098219861
ENSRNOG00000031864	AABR07021871.1	418.8048631	-0.040258035	0.087139617
ENSRNOG00000017893	Baiap3	564.2372196	-0.034453264	0.074589049
ENSRNOG00000003772	Csrp2	296.7535393	0.048548058	0.104857515
ENSRNOG00000028711	Dgat1	258.1326105	-0.044526918	0.095450347
ENSRNOG00000003424	Elac2	809.8542638	0.027444036	0.059135683
ENSRNOG00000009409	Fbxo2	511.7683754	0.031364515	0.068089206
ENSRNOG00000004169	Fzr1	200.4600401	0.052949695	0.114227567
ENSRNOG00000046295	NA	215.3893275	-0.062453624	0.135346368
ENSRNOG00000062261	NA	407.1174219	-0.053593708	0.116285014
ENSRNOG00000055562	NA	392.2349967	-0.04670934	0.101486965
ENSRNOG00000018674	Ntrk3	261.5415419	0.048580085	0.105293147
ENSRNOG00000001312	Pdgfa	590.4165	0.03806179	0.082553543
ENSRNOG00000050585	Pgam1	339.1602733	0.04059785	0.087773553
ENSRNOG00000042289	Plcxd2	279.8602118	-0.063521348	0.136508749
ENSRNOG00000020674	Rpl27	262.0814037	-0.042398499	0.091682632
ENSRNOG00000000490	Rps10	2462.977997	-0.03225767	0.069757247
ENSRNOG00000011029	Sec11a	256.5692696	0.041819221	0.08992935
ENSRNOG00000062247	AABR07031521.1	2556.415561	-0.029774187	0.067098
ENSRNOG00000012683	Abhd17c	233.830097	-0.038481277	0.087045979
ENSRNOG00000027260	Adprh	485.9564349	0.034187506	0.076173176
ENSRNOG00000008659	Arhgap21	443.1835515	0.037704514	0.085125708
ENSRNOG00000010034	Asah1	236.3948407	0.043523139	0.097525499
ENSRNOG00000002094	Atg3	287.2161137	0.042145043	0.093952269
ENSRNOG00000021240	Atrn	390.5799062	0.042053773	0.09289483
ENSRNOG00000004697	Baalc	956.4470593	-0.031187762	0.070189568
ENSRNOG00000015997	Ccdc106	300.2840779	0.036546029	0.080971325
ENSRNOG00000009675	Cdc42bpb	454.6564987	0.036882178	0.081597356
ENSRNOG00000027282	Cep68	401.2755298	-0.034224016	0.075227463
ENSRNOG00000009781	Dync1i2	590.9904368	0.034074569	0.07437149
ENSRNOG00000004883	Erh	293.5330801	0.03681607	0.083404372
ENSRNOG00000002039	Evi5	252.0862325	-0.041219156	0.093757368
ENSRNOG00000020518	Fam189b	383.8464593	0.04189983	0.093790031
ENSRNOG00000015794	Fam83d	455.6564229	0.035122474	0.078493191
ENSRNOG00000004577	Fez2	235.5909866	-0.046884257	0.10262638
ENSRNOG00000000805	Gja1	1025.825296	-0.037434725	0.085068863
ENSRNOG00000019205	Gnpat	496.6897497	-0.029674161	0.066807288
ENSRNOG00000008423	Gpr22	211.2739304	0.056703528	0.128634291
ENSRNOG00000012236	Hddc3	646.2664514	-0.028844377	0.064100945
ENSRNOG00000021160	Hormad1	883.6770913	-0.05011947	0.110379768
ENSRNOG00000010482	Impa1	221.3271875	0.04836159	0.107894619
ENSRNOG00000020032	Impdh1	275.8108094	0.045554495	0.101843264
ENSRNOG00000021748	Las1l	206.0465391	0.040246783	0.091496735
ENSRNOG00000050849	Leprot	228.1651681	0.044775702	0.099666158
ENSRNOG00000021023	Mag	328.781974	0.06631646	0.149738935
ENSRNOG00000014013	Map4k4	233.6128382	0.047859461	0.106190553
ENSRNOG00000008049	Max	254.5514569	-0.060209229	0.131196032
ENSRNOG00000045655	NA	769.8500861	-0.02775275	0.060987505
ENSRNOG00000045583	NA	418.5129346	0.033797882	0.074331821

ENSRNOG00000007112	NA	346.5786943	-0.036478356	0.082028338
ENSRNOG00000018148	NA	314.4969481	0.04730847	0.107718015
ENSRNOG00000008663	Nat10	354.2604878	-0.037917392	0.084707585
ENSRNOG00000013562	Ndfip1	5516.158709	-0.033618349	0.073440503
ENSRNOG00000046449	Npy	375.5370717	-0.042252633	0.092215148
ENSRNOG00000005111	Nutm1	210.6719149	-0.042877406	0.097146996
ENSRNOG00000013825	Rap1gap	720.8040161	0.03284535	0.074147692
ENSRNOG00000019235	Rbm22	246.4146435	0.044040643	0.099011953
ENSRNOG00000005387	Rbm3	297.9015282	-0.050275248	0.114480835
ENSRNOG00000005218	Sf3a1	281.6859949	-0.040359378	0.091590598
ENSRNOG00000016949	Slc25a33	329.113351	0.04389658	0.099337906
ENSRNOG00000004510	Slc35b1	576.4110281	-0.035013048	0.078471458
ENSRNOG00000004168	Slc35e4	312.470637	0.059551376	0.132372341
ENSRNOG00000018194	Srrm1	218.9376802	0.041892446	0.093303529
ENSRNOG00000003402	Stx6	328.4469089	-0.039812114	0.089537159
ENSRNOG00000020916	Tmub2	790.0859939	0.03192923	0.069980559
ENSRNOG00000008296	Tpgs1	484.6508757	-0.039383921	0.087947235
ENSRNOG00000010124	Trim2	1246.300926	0.037324435	0.08190974
ENSRNOG00000022507	Twf1	283.6739988	-0.046109599	0.103954927
ENSRNOG00000050207	Utf1	242.0252842	-0.050349266	0.110346198
ENSRNOG00000012524	Zfp91	326.7210308	-0.044649893	0.100415541
ENSRNOG00000020239	Capn15	300.1915619	0.042809547	0.097607895
ENSRNOG00000014264	Phactr1	829.5551653	0.028864708	0.065914104
ENSRNOG00000013557	Lancl1	1087.374215	-0.02449302	0.056016242
ENSRNOG00000032027	Tmem42	293.8604607	0.046604702	0.107078463
ENSRNOG00000000060	NA	2224.334529	-0.018984784	0.043682602
ENSRNOG00000001719	Psmd2	595.727394	0.028095018	0.064680735
ENSRNOG00000012820	Add3	384.5025551	-0.031722511	0.074663142
ENSRNOG00000009719	C2cd2l	1376.499219	-0.030176404	0.070957635
ENSRNOG00000000610	Cisd1	1264.986115	-0.030603144	0.072029398
ENSRNOG00000026577	Cpne4	356.8534357	-0.047978019	0.111974798
ENSRNOG00000013451	Drosha	281.3733923	0.037730225	0.088663518
ENSRNOG00000006462	Ero1a	267.8289716	0.053092383	0.124369291
ENSRNOG00000013186	G3bp1	532.7783538	0.027118847	0.063607018
ENSRNOG00000008790	Gdap1l1	429.1149083	-0.034508142	0.08046716
ENSRNOG00000000808	Hsf2	3788.791096	0.031164559	0.072999353
ENSRNOG00000019937	Kcnk1	809.9502258	-0.030010253	0.070628918
ENSRNOG00000033694	Klf16	579.0478821	-0.030152153	0.070735665
ENSRNOG00000003977	NA	370.5687226	0.184582117	0.432017402
ENSRNOG00000002721	Ndufb4	1511.749601	-0.023659408	0.055603815
ENSRNOG00000005504	Pkp4	674.7432579	-0.028823159	0.067548818
ENSRNOG00000008140	Rpl15	2339.07835	-0.020513055	0.047488281
ENSRNOG00000010188	Satb2	505.1647506	0.036056266	0.084215742
ENSRNOG00000020781	Tbcb	768.306483	-0.026572116	0.061781063
ENSRNOG00000009156	Tra2a	200.6920097	0.04806361	0.11184593
ENSRNOG00000003786	Tspan6	207.6333678	0.042085405	0.098116817
ENSRNOG00000011678	Wbp4	302.4072739	-0.04215409	0.099290272
ENSRNOG00000018798	Bcan	1777.172545	0.039220294	0.092488154
ENSRNOG00000017983	Ubac1	373.4926048	-0.035768635	0.084552996
ENSRNOG00000043114	Tomm7	407.3259255	-0.03801277	0.090044129

ENSRNOG00000020246	Myl9	252.496966	0.185040441	0.438877967
ENSRNOG00000008996	Dpysl5	303.6040906	-0.035289649	0.08397228
ENSRNOG00000000640	Egr2	241.7157339	-0.122290665	0.29108139
ENSRNOG00000024022	Ndfip2	925.9787459	-0.031437667	0.074872326
ENSRNOG00000060863	NA	572.4400163	0.038957961	0.092876977
ENSRNOG00000026319	Akap9	216.165414	-0.047539082	0.114235726
ENSRNOG00000007445	Asph	342.8685722	-0.034295935	0.082181732
ENSRNOG00000025164	Bhlha15	341.9601241	0.034607313	0.084898444
ENSRNOG00000003256	Ccng1	1226.084585	0.041824406	0.102282361
ENSRNOG00000025768	Clk1	962.8053928	-0.031773171	0.077273483
ENSRNOG00000001069	Elavl1	274.113939	-0.041797407	0.1021679
ENSRNOG00000030127	Eml2	203.2788432	-0.061187431	0.149565742
ENSRNOG00000011203	Farp1	490.9168145	0.032254378	0.077213137
ENSRNOG00000033790	Hnrnpu	945.7936424	-0.025610773	0.061816569
ENSRNOG00000019525	Hspa9	557.2684244	0.035397251	0.086047325
ENSRNOG00000037659	Mto1	603.0460335	-0.028186561	0.069063072
ENSRNOG00000049931	NA	274.6002529	-0.041129525	0.099976921
ENSRNOG00000024763	NA	514.8157699	-0.033306128	0.081660949
ENSRNOG00000009015	Nfx1	219.4401239	0.039831619	0.09767718
ENSRNOG00000016807	Oat	633.5152377	-0.042626128	0.102513776
ENSRNOG00000002827	Rbfox1	1416.692773	0.027174295	0.065978588
ENSRNOG00000003386	Rbfox3	712.4039998	0.033213748	0.080924205
ENSRNOG00000019193	Stx1b	202.6178423	-0.134622003	0.324287666
ENSRNOG00000018367	Taldo1	439.3687619	-0.219893346	0.529368894
ENSRNOG00000003048	Tiprl	238.8735711	0.040546177	0.096942996
ENSRNOG00000001817	Tm7sf3	231.3858802	0.051457419	0.123814759
ENSRNOG00000003691	Tmed7	391.0733245	0.041118272	0.098497919
ENSRNOG00000022432	Tmem59l	2253.128045	-0.02456791	0.059420894
ENSRNOG00000019164	Uba1	243.2522512	-0.043220312	0.106112397
ENSRNOG00000013741	Ube2d3	373.6484873	-0.032624517	0.07990132
ENSRNOG00000010727	Ube2r2	204.9848608	0.039416933	0.09603558
ENSRNOG00000003625	Ubxn4	367.143981	0.031892115	0.077334491
ENSRNOG00000015644	Ugcg	367.1870379	-0.04174826	0.100892229
ENSRNOG00000030055	Vamp3	770.9305479	-0.034880722	0.083942217
ENSRNOG00000015750	Wnt7b	273.0013353	-0.04642191	0.113282818
ENSRNOG00000004773	Yaf2	949.7489398	-0.023076916	0.056608718
ENSRNOG00000059827	6330403K07Rik	731.3790674	-0.035615447	0.087578386
ENSRNOG00000024879	Polrmt	528.9695845	0.03152635	0.077591247
ENSRNOG00000042359	Pigk	211.7349289	-0.046731776	0.115560894
ENSRNOG00000028822	Bex3	2125.523891	-0.024120678	0.060144594
ENSRNOG00000008741	Camsap2	375.6327809	0.031015897	0.077015016
ENSRNOG00000015753	Epn1	814.0211104	0.026337168	0.065431205
ENSRNOG00000018836	RGD1559896	688.1277914	0.030082365	0.075055419
ENSRNOG00000011307	Slmap	223.0561974	-0.039326695	0.097512338
ENSRNOG00000054446	Sltm	303.3333918	-0.03479435	0.086705805
ENSRNOG00000004018	Tdrd5	1267.255301	0.265416754	0.660404466
ENSRNOG00000039985	Ube2a	312.7486147	-0.039035011	0.097035322
ENSRNOG00000016834	Wdr37	304.2627444	-0.034981285	0.086927627
ENSRNOG00000017328	Pter	1212.711811	-0.021508927	0.053719992
ENSRNOG00000003428	Rufy3	903.8861622	0.026838643	0.067133992

ENSRNOG00000001344	NA	358.2903989	-0.043021868	0.107814591
ENSRNOG00000033531	Cacna2d1	515.644176	0.045552022	0.115279182
ENSRNOG00000008063	Hibadh	354.5279791	-0.037685242	0.095015098
ENSRNOG00000018468	Ldb1	375.0944839	-0.035539288	0.089685631
ENSRNOG00000006050	Med19	221.3022085	0.039811551	0.100629841
ENSRNOG00000016955	Mindy3	271.6897535	-0.042869918	0.107655841
ENSRNOG00000055038	NA	529.317471	-0.033854763	0.085263279
ENSRNOG00000058793	NA	314.3104896	0.036363838	0.091875258
ENSRNOG00000018972	Rab18	274.2391872	0.042869966	0.107939609
ENSRNOG00000049780	Sri	345.7659853	0.035391842	0.089326416
ENSRNOG00000005528	Tbk1	299.6480478	0.03957253	0.100182155
ENSRNOG00000017872	NA	288.6989321	0.101135196	0.258041264
ENSRNOG00000038375	AABR07026311.1	373.3798104	0.033251654	0.085380334
ENSRNOG00000007433	Cyb561	612.7121204	0.035183442	0.090589559
ENSRNOG00000009102	Fermt2	258.5957611	0.04274271	0.109686784
ENSRNOG00000003453	Lypd1	235.5029361	-0.049129701	0.125851686
ENSRNOG00000017150	Med10	257.1532305	-0.048842878	0.12564054
ENSRNOG00000021144	NA	560.8970997	-0.030219599	0.077554766
ENSRNOG00000021170	Plekho1	523.4298376	0.031574737	0.081037706
ENSRNOG00000001203	Rrp1	242.2801072	-0.036686993	0.094339406
ENSRNOG00000016064	Tkt	949.9676894	-0.021450396	0.054798257
ENSRNOG00000022144	AABR07072356.1	265.2934713	-0.035861221	0.092626719
ENSRNOG00000032878	Cxxc5	847.801865	0.024804023	0.064012853
ENSRNOG00000009894	Vps50	213.2725344	-0.036702106	0.094908959
ENSRNOG00000036701	Actg1	443.7546861	-0.029537677	0.076586849
ENSRNOG00000052730	Psmb3	892.8529031	-0.020023143	0.051943883
ENSRNOG00000025040	NA	1231.567923	0.021461449	0.056050253
ENSRNOG00000056756	Actn1	573.1970063	0.032410934	0.085622014
ENSRNOG00000000461	Brd2	564.23703	-0.027080486	0.071063101
ENSRNOG00000010706	Ccdc117	439.3936355	-0.033235462	0.088035063
ENSRNOG00000012815	Chst10	386.1178816	-0.028254809	0.074747636
ENSRNOG00000021611	Clip2	444.5718933	0.027207856	0.073065072
ENSRNOG00000013076	Csnk1e	518.2157579	0.023884029	0.063558525
ENSRNOG00000020206	Ctsd	245.3655647	-0.036467022	0.096597476
ENSRNOG00000014811	Cyhr1	465.3803059	-0.027668126	0.072881858
ENSRNOG00000020383	Dapk3	723.2897352	-0.029913982	0.078818976
ENSRNOG00000004006	Dnajb9	200.2036049	-0.038204231	0.101537775
ENSRNOG00000058882	Emd	219.154005	0.054073693	0.14289055
ENSRNOG00000027707	Exd2	314.9523161	0.03137553	0.083921442
ENSRNOG00000000798	Gnl1	939.7864213	-0.027542215	0.073517127
ENSRNOG00000011782	Got2	1147.010456	0.023452182	0.062019919
ENSRNOG00000012360	Gtf2h1	251.3725323	0.033708433	0.089808264
ENSRNOG00000020812	Gys1	361.5650855	-0.039736338	0.106531568
ENSRNOG00000054458	Kcnmb4	1761.555584	-0.025005298	0.06598825
ENSRNOG00000020005	Map2k2	814.4940134	0.025726652	0.068241059
ENSRNOG00000017311	Me3	197.2585194	0.04272998	0.11467662
ENSRNOG00000024616	NA	515.2129229	-0.02892823	0.077464252
ENSRNOG00000008761	NA	2297.495915	-0.019424689	0.052058164
ENSRNOG00000048186	NA	453.1791997	-0.026274413	0.070420309
ENSRNOG00000002693	Nme1	270.465703	-0.214053026	0.574716017

ENSRNOG00000000123	Rnf19b	217.83972	0.03450198	0.091961701
ENSRNOG00000011927	Sdc3	704.2671439	0.029359112	0.078358402
ENSRNOG00000022686	Zdhhc2	277.6514264	-0.031771151	0.083945094
ENSRNOG00000004281	Cobl	353.7525237	0.030372177	0.081779445
ENSRNOG00000002301	Uso1	461.2582556	0.032818812	0.08842387
ENSRNOG00000016724	Epb41l3	361.7199987	0.038972671	0.105115407
ENSRNOG00000050578	Rheb	1195.944678	-0.022503553	0.06080289
ENSRNOG00000023786	Ybx1	407.4845983	0.03770008	0.101907629
ENSRNOG00000018991	Gsn	293.3377003	0.096087895	0.260320671
ENSRNOG00000042387	Slc25a6	398.4824416	0.037102015	0.100501539
ENSRNOG00000000918	Zbed5	758.7546109	-0.022815511	0.062060635
ENSRNOG00000057996	Phactr3	948.8017927	-0.022926144	0.062611818
ENSRNOG00000019868	Psma5	236.1995064	-0.038916973	0.106205428
ENSRNOG00000016789	Actr1b	1348.89725	0.025055144	0.069414092
ENSRNOG00000061108	Arcn1	427.8427268	-0.0298694	0.083393357
ENSRNOG00000058841	Bag5	220.2482334	0.038737585	0.107247761
ENSRNOG00000032705	Bcl7b	301.3287798	-0.03090141	0.086064307
ENSRNOG00000038002	Ccdc62	332.0141706	0.027466432	0.076812889
ENSRNOG00000004148	Cdk17	619.6074761	-0.028949898	0.080641761
ENSRNOG00000006787	Dhcr24	444.5060947	0.026733923	0.073526605
ENSRNOG00000007600	Igsf1	210.0982322	0.086429514	0.239688821
ENSRNOG00000009097	Immt	612.1738921	0.025603752	0.071190203
ENSRNOG00000056954	Inpp5k	973.5175862	-0.023338155	0.064598637
ENSRNOG00000020044	Mob2	207.9841031	0.037000062	0.102525782
ENSRNOG00000042720	Mrpl28	216.1783264	0.039554254	0.108664753
ENSRNOG00000001156	Msi1	233.4744772	0.034466086	0.096086962
ENSRNOG00000010407	NA	239.0276724	-0.038003456	0.104771912
ENSRNOG00000017824	Ncoa5	1352.696195	0.026293918	0.072400911
ENSRNOG00000013653	Pdlim7	485.005465	-0.031235855	0.086867745
ENSRNOG00000049203	Pex5	241.3365418	-0.037536817	0.104373146
ENSRNOG00000012247	Rab7a	1089.540773	0.025229997	0.070064898
ENSRNOG00000049784	Tsnax	485.0312479	-0.025709497	0.071902415
ENSRNOG00000027317	Ylpm1	216.7613247	0.039245062	0.107671033
ENSRNOG00000010635	Igfbp4	201.4058566	-0.046991783	0.131601915
ENSRNOG00000014363	Arhgef3	257.0042758	-0.046809134	0.131275272
ENSRNOG00000047082	NA	427.4487993	-0.031901622	0.089602154
ENSRNOG00000005519	Grm3	267.8802015	-0.029492487	0.082933246
ENSRNOG00000051190	AABR07015507.1	196.8142311	-0.039564919	0.113894496
ENSRNOG00000017914	Cavin3	317.9877132	-0.033666213	0.095381401
ENSRNOG00000015591	Cndp2	301.4269107	-0.029292349	0.084194748
ENSRNOG00000020225	Dlgap4	712.1178544	-0.03413055	0.09874129
ENSRNOG00000004259	Dohh	385.3982011	-0.028902686	0.083445053
ENSRNOG00000028674	Fbxw5	331.0635018	0.031301073	0.089396334
ENSRNOG00000043171	Hdhd2	460.3412469	0.02556686	0.073884516
ENSRNOG00000014562	Hnrnpf	484.7428545	0.033658695	0.096728543
ENSRNOG00000014565	Lcmt1	525.0068023	0.025283968	0.071721209
ENSRNOG00000005133	Mapt	2154.809447	0.026215717	0.074576084
ENSRNOG00000015059	Mcfd2	251.6564873	0.031933978	0.092455376
ENSRNOG00000009309	Meaf6	282.4469468	0.030657711	0.086349748
ENSRNOG00000001113	Mmd2	965.6841735	0.030179899	0.087021

ENSRNOG00000047557	NA	365.4144258	-0.040406238	0.115060915
ENSRNOG00000018914	Napg	665.3755297	-0.024198117	0.068692884
ENSRNOG00000049402	Nbl1	1158.70688	-0.023994055	0.068651063
ENSRNOG00000004676	Pak3	273.9728606	-0.033150024	0.095707194
ENSRNOG00000002579	Parm1	225.2201114	-0.037247843	0.106882777
ENSRNOG00000060571	Peds1	292.6017071	0.033341094	0.096508151
ENSRNOG00000014791	Peg3	685.1759688	0.027608166	0.080187132
ENSRNOG00000057626	Pgd	1952.530488	0.018471688	0.05339236
ENSRNOG00000015528	Pja2	2522.807984	0.018729011	0.054359585
ENSRNOG00000019620	Pmf1	239.7571104	-0.034787413	0.09823978
ENSRNOG00000000433	Prrt1	898.1858675	0.028407984	0.080508341
ENSRNOG00000001374	Rasal1	302.9556166	0.032837409	0.092983602
ENSRNOG00000008851	Slc35b4	233.9413491	0.0327691	0.093301719
ENSRNOG00000010712	Terf2ip	269.5142204	0.032519648	0.092597909
ENSRNOG00000009300	Tlk1	415.0115079	-0.024881734	0.071269966
ENSRNOG00000017558	Tubb2a	226.9556364	-0.032440022	0.094045824
ENSRNOG00000000604	Hdac2	605.6158629	0.024865083	0.072365502
ENSRNOG00000021110	NA	3388.658263	0.026794589	0.078041516
ENSRNOG00000046257	Ctdspl	200.5346449	-0.040354672	0.118310002
ENSRNOG00000018825	Dcaf11	835.9802481	0.131194475	0.386989778
ENSRNOG00000009766	Gpr180	208.763924	0.035933606	0.105179373
ENSRNOG00000026953	Gpr88	425.3845827	0.027359664	0.081020022
ENSRNOG00000004474	Klhdc2	558.4612965	0.025052112	0.074106229
ENSRNOG00000056817	Muc6	1816.924919	0.021282969	0.062953978
ENSRNOG00000008639	Pabpc1	975.3202948	-0.023827306	0.069977095
ENSRNOG00000019977	Ptprf	545.8740035	-0.028365774	0.083909795
ENSRNOG00000004093	Rnf135	299.7501236	0.029251727	0.086385613
ENSRNOG00000001425	Sh2b2	203.5183747	-0.037084601	0.109374597
ENSRNOG00000046705	Snx3	2726.457605	-0.018407827	0.053958303
ENSRNOG00000034242	Vcp	1339.861849	-0.020266433	0.059801683
ENSRNOG00000005345	Vsnl1	6875.466257	-0.017980742	0.052535263
ENSRNOG00000007869	Wscd1	459.4847242	0.034794268	0.10192276
ENSRNOG00000057221	Scn3b	1298.079365	-0.019704683	0.058483276
ENSRNOG00000006380	Srsf6	210.231481	-0.041951453	0.12464564
ENSRNOG00000014718	Acsl3	592.885629	-0.028344393	0.084556942
ENSRNOG00000025624	Arhgap20	286.9294494	-0.034392479	0.103190666
ENSRNOG00000003239	Cant1	244.5085127	-0.034099743	0.103903392
ENSRNOG00000043387	Cpe	7295.663465	-0.012993912	0.039053411
ENSRNOG00000015215	Cript	286.2678111	-0.034690335	0.105805328
ENSRNOG00000002767	Dlg3	566.4099321	0.024619718	0.074055861
ENSRNOG00000004637	Fbxo7	341.359038	-0.027391166	0.083442023
ENSRNOG00000007351	Ggh	247.7265138	-0.039552273	0.120014389
ENSRNOG00000016516	Mbp	10573.47153	0.041804393	0.125532479
ENSRNOG00000037402	NA	567.5864671	-0.02269799	0.068411955
ENSRNOG00000048365	NA	633.9218014	-0.034572658	0.104581081
ENSRNOG00000008604	Nsfl1c	435.5821051	-0.025225543	0.075921101
ENSRNOG00000015346	Obsl1	415.9315842	-0.031156711	0.094919647
ENSRNOG00000009889	Pgm1	488.8919187	-0.027823316	0.08366622
ENSRNOG00000007324	Plxna2	260.8874014	0.032704763	0.09901213
ENSRNOG00000012026	Psmc2	552.7055681	0.028946904	0.087724602

ENSRNOG00000005587	Suclg1	641.9479716	-0.022963153	0.069791194
ENSRNOG00000018243	Ubfd1	192.6089272	-0.090490398	0.272859719
ENSRNOG00000001418	Znhit1	358.7120251	-0.02474765	0.074947692
ENSRNOG00000007069	Adhfe1	221.3594471	0.033708656	0.103293009
ENSRNOG00000006118	Klf10	231.3785237	-0.093038257	0.285258004
ENSRNOG00000032134	Uqcrc1	392.5948462	0.024695652	0.076055315
ENSRNOG00000014613	Ddah1	355.548059	0.033981336	0.104839577
ENSRNOG00000011268	Chd5	2592.500654	-0.017023855	0.052850423
ENSRNOG00000015051	Golga7b	937.3843382	-0.022570288	0.070125859
ENSRNOG00000012918	Mtmr6	349.3961305	0.029983016	0.093097445
ENSRNOG00000009206	Fezf2	355.0124834	-0.028408081	0.088609866
ENSRNOG00000017905	Map1lc3b	259.3726919	-0.027186424	0.084799904
ENSRNOG00000016963	Trip12	303.2990438	0.038556181	0.120166085
ENSRNOG00000008924	Arhgef12	352.1928393	-0.074358362	0.233544716
ENSRNOG00000009152	Caprin1	198.8399527	-0.033345235	0.104348731
ENSRNOG00000022702	Fbxo22	657.8852187	0.024101253	0.075439652
ENSRNOG00000021133	Lsm14a	279.9795725	0.033097177	0.103773092
ENSRNOG00000011979	Mrps24	456.5291874	-0.024360515	0.076555462
ENSRNOG00000057756	Osbpl2	223.8002673	-0.031197377	0.097770901
ENSRNOG00000028826	AC128901.1	252.6013543	-0.02788391	0.088267487
ENSRNOG00000026610	Akirin1	368.1372763	-0.027834001	0.087980383
ENSRNOG00000010199	Glrb	680.1609762	0.025075935	0.079290469
ENSRNOG00000030800	Igdcc3	279.1506479	0.029536121	0.093165017
ENSRNOG00000013320	NA	501.9339238	0.023917799	0.075581078
ENSRNOG00000046021	Pcsk1n	413.6792355	0.039724303	0.126115652
ENSRNOG00000009278	Ift88	516.4883684	0.02555073	0.081517291
ENSRNOG00000038735	AABR07064702.1	371.5501867	0.024052729	0.077209273
ENSRNOG00000018345	Abce1	500.1252104	0.023342406	0.077452818
ENSRNOG00000007946	Bcl2l1	486.4966926	0.023405097	0.075619521
ENSRNOG00000008414	Bsg	289.1166824	0.181835855	0.600408775
ENSRNOG00000007859	Cep83	210.509248	-0.033268904	0.10659389
ENSRNOG00000005733	Cpsf2	228.3499943	-0.033516249	0.111188889
ENSRNOG00000015295	Ctdsp1	194.7130188	0.03429178	0.111534566
ENSRNOG00000043503	Ehd1	338.134845	0.027358423	0.090427597
ENSRNOG00000030391	Ei24	692.2494842	0.024943837	0.081803715
ENSRNOG00000003653	Fh	515.7907223	-0.023224026	0.076014132
ENSRNOG00000008822	Fkbp1a	5946.660773	-0.02215758	0.071331131
ENSRNOG00000020878	Keap1	429.1828224	-0.022938286	0.075757269
ENSRNOG00000006766	Laptm4b	337.964063	-0.024867731	0.082567821
ENSRNOG00000020363	Med25	399.4592338	0.02216835	0.072766737
ENSRNOG00000059663	NA	242.3752282	0.030947427	0.09962694
ENSRNOG00000045747	NA	398.1573015	0.025064874	0.081717589
ENSRNOG00000026519	NA	204.5759756	0.03152989	0.103911066
ENSRNOG00000010309	Nfyb	218.6489505	-0.032903299	0.108644217
ENSRNOG00000016680	Nsd1	468.1582674	0.021170963	0.069732282
ENSRNOG00000005424	Odc1	426.0368698	-0.02315882	0.075638561
ENSRNOG00000055530	Panx2	520.3266679	-0.022473243	0.074158574
ENSRNOG00000019268	Pelp1	215.3639712	-0.028210743	0.093049393
ENSRNOG00000018851	Ppp2r2b	1191.722162	-0.020259892	0.06609866
ENSRNOG00000060449	Rps21	313.6800543	-0.023835583	0.077408226

ENSRNOG00000019737	Sema4a	293.0146304	0.028599797	0.094255032
ENSRNOG00000038001	Slc25a1	233.5076524	0.031113194	0.103199356
ENSRNOG00000008725	Slc37a3	253.8650248	0.027858258	0.090059251
ENSRNOG00000006037	Snap25	13927.68058	-0.015811732	0.052433311
ENSRNOG00000005046	Tspan13	940.5706547	0.023299105	0.076634976
ENSRNOG00000000638	Zfp365	1432.675393	0.015484069	0.051175203
ENSRNOG00000011104	Rpl22	633.0034359	-0.022989631	0.076505507
ENSRNOG00000018935	NA	1278.547286	-0.020480528	0.068424633
ENSRNOG00000012933	Prcc	304.7108451	-0.027972562	0.093456019
ENSRNOG00000008697	Ccn3	1218.583202	-0.026586032	0.089299067
ENSRNOG00000021224	Mrps26	311.2946547	0.025899703	0.087275991
ENSRNOG00000058571	NA	315.1506669	-0.026979494	0.091013618
ENSRNOG00000012953	Arsa	237.0369763	-0.026117159	0.08856485
ENSRNOG00000005589	Dhrs7	234.7284173	0.03055624	0.103426059
ENSRNOG00000001178	Sppl3	530.3036357	0.02293916	0.077558364
ENSRNOG00000018122	Tspan17	874.9847804	-0.021415127	0.072557159
ENSRNOG00000004846	Atp6v1c1	992.2188247	-0.024388948	0.085358236
ENSRNOG00000003563	Blmh	245.5857524	-0.027629255	0.094266979
ENSRNOG00000010535	Cdh18	225.9900134	-0.036584452	0.127875611
ENSRNOG00000007821	Dyrk2	286.4373403	-0.031288157	0.107321455
ENSRNOG00000053607	Fam217b	228.935651	0.028891149	0.100342009
ENSRNOG00000005378	Gna15	204.0945996	-0.030629497	0.106688642
ENSRNOG00000000701	Iscu	1213.503839	-0.037388164	0.129498215
ENSRNOG00000015401	Mapk4	267.3381055	-0.027198381	0.094854323
ENSRNOG00000058412	Morf4l1	344.9542883	-0.03221779	0.110516891
ENSRNOG00000047984	Mrps22	231.9525286	-0.035054863	0.119573783
ENSRNOG00000017172	Mvb12b	696.3683855	0.025018445	0.087609277
ENSRNOG00000003160	NA	309.7161178	0.026354273	0.0923038
ENSRNOG00000005883	Nek10	313.3779488	-0.028898288	0.099199231
ENSRNOG00000008970	Pcdh17	342.5353366	-0.026096292	0.089138877
ENSRNOG00000015385	Pink1	352.2200307	0.021594257	0.075673634
ENSRNOG00000022772	Prickle1	288.4563205	-0.038286682	0.132503002
ENSRNOG00000006345	Sec61b	210.6012102	0.028093772	0.096714542
ENSRNOG00000055257	Selenof	1896.18453	-0.023117443	0.081003756
ENSRNOG00000039980	Slc25a5	2708.184958	0.018111867	0.062136594
ENSRNOG00000020204	Srp19	387.5057546	-0.022655259	0.078749709
ENSRNOG00000037275	Tlcd2	460.0353865	-0.033324481	0.11650917
ENSRNOG00000032699	Ttyh1	235.6308051	0.141657663	0.492060851
ENSRNOG00000010702	Ube3c	429.4860723	0.023036555	0.079705975
ENSRNOG00000055925	Zc3h7b	927.3703085	-0.016896115	0.05763021
ENSRNOG00000021157	Ctss	196.5324543	0.037668249	0.132711358
ENSRNOG00000005985	Kcnma1	257.9834902	0.03385998	0.119249551
ENSRNOG00000000596	Fyn	467.1567155	0.020332662	0.071934286
ENSRNOG00000025504	Ankrd46	725.9863284	-0.018119223	0.064680854
ENSRNOG00000008566	Mrpl15	429.1089921	-0.02103073	0.074892907
ENSRNOG00000007607	Nr4a1	824.1003758	-0.115489034	0.412046896
ENSRNOG00000023812	Raver2	360.5592477	0.029076353	0.103922193
ENSRNOG00000050323	Srsf1	400.8244948	0.020761412	0.073950659
ENSRNOG00000028103	Psmd3	830.9630224	0.019681158	0.07044471
ENSRNOG00000047546	Gas2l2	1433.824402	0.026354336	0.09507593

ENSRNOG00000049067	NA	308.0629403	0.036598888	0.132135134
ENSRNOG00000000098	Rnf115	365.1569936	-0.025405027	0.091825926
ENSRNOG00000052572	Aff2	541.3587578	-0.026183974	0.095107112
ENSRNOG00000003300	Btg2	262.3461789	0.120109973	0.437003979
ENSRNOG00000015442	Sfxn3	392.3400627	0.034782835	0.126806842
ENSRNOG00000018685	AABR07028237.1	401.5706538	-0.01968802	0.072801575
ENSRNOG00000019087	Fam174a	403.1988427	0.021754876	0.07996057
ENSRNOG00000056728	NA	314.2700401	0.025853198	0.095172072
ENSRNOG00000037711	NA	280.6037715	-0.026729057	0.098868904
ENSRNOG00000000165	Pfkfb1	252.7390498	-0.026655902	0.09819121
ENSRNOG00000036827	Ppp1r1a	733.953469	-0.01987701	0.073339896
ENSRNOG00000007907	Tmem178a	633.6408931	-0.023639199	0.087278974
ENSRNOG00000019091	Wtap	445.5941467	-0.024424774	0.090236442
ENSRNOG00000004556	Dcaf5	243.7905648	-0.02747205	0.101826364
ENSRNOG00000016321	Entpd4	279.2138615	0.023175431	0.086060696
ENSRNOG00000014107	Omg	1167.142469	0.019484281	0.072404448
ENSRNOG00000018153	Rbm5	461.4701338	0.021939634	0.081738947
ENSRNOG00000053631	Tdrd9	2257.682718	-0.018671973	0.069690118
ENSRNOG00000010682	Clcn3	813.0155096	-0.027531037	0.103659328
ENSRNOG00000058386	Golt1b	198.2801342	-0.027665221	0.103989109
ENSRNOG00000005176	Map7d2	385.299159	-0.02300168	0.086705592
ENSRNOG00000043377	Rusc1	1187.681294	0.017231685	0.065090444
ENSRNOG00000019940	Ssr2	362.007335	-0.022243823	0.08399554
ENSRNOG00000012084	Xpnpep1	236.7989531	0.024280019	0.092227396
ENSRNOG00000023427	Ano7	357.0510115	0.02803116	0.107078993
ENSRNOG00000006870	Mtdh	395.9572827	-0.027385764	0.104955549
ENSRNOG00000050800	NA	485.4708921	-0.019293796	0.074046105
ENSRNOG00000010886	Nfe2l3	1571.558862	-0.017742258	0.068047353
ENSRNOG00000004992	Rab1a	564.1531485	0.01717021	0.065858096
ENSRNOG00000006663	Usp2	368.2104043	-0.084854554	0.326220127
ENSRNOG00000029529	Eef1akmt4	1038.179366	0.023442354	0.091177043
ENSRNOG00000031038	Gtdc1	191.6959588	0.02861267	0.111870002
ENSRNOG00000048226	Hmgn1	282.5786064	0.022333323	0.086186012
ENSRNOG00000006226	NA	440.4927813	-0.028256343	0.109223143
ENSRNOG00000040005	NA	365.8392041	0.020007068	0.078229938
ENSRNOG00000046621	NA	282.4215469	0.026802535	0.104869296
ENSRNOG00000025254	Qrich2	561.1339819	0.021011873	0.081616978
ENSRNOG00000004420	Rad21	276.739917	0.021356171	0.083392955
ENSRNOG00000003767	Rps6kc1	250.9768216	0.022966094	0.089103977
ENSRNOG00000002358	Scpep1	192.8471628	0.027230263	0.106040423
ENSRNOG00000006426	Syt1	3827.834185	0.016239337	0.063557484
ENSRNOG00000025463	AI593442	775.2850248	-0.018549852	0.072879494
ENSRNOG00000000541	Glo1	556.1142143	0.019663577	0.07731681
ENSRNOG00000018745	Sgsm3	267.2524825	0.024152204	0.095179279
ENSRNOG00000014496	Coro6	537.334743	0.01757661	0.069397123
ENSRNOG00000013255	Dnajc8	214.489894	0.026794082	0.1061478
ENSRNOG00000024144	Cdipt	797.1870659	-0.017458541	0.069329494
ENSRNOG00000013911	Nagk	212.6647247	-0.027213846	0.108450638
ENSRNOG00000059744	Atxn7l3b	895.9452979	-0.019301083	0.077382374
ENSRNOG00000003260	Nr1i3	257.1201622	0.020804837	0.083426016

ENSRNOG00000059852	NA	4109.017814	-0.075707677	0.306840083
ENSRNOG00000020658	Aarsd1	345.7785086	-0.021417618	0.087652554
ENSRNOG00000055860	Arl8b	512.491845	-0.020092503	0.082196802
ENSRNOG00000012760	Chtop	708.2260305	-0.013596541	0.055466364
ENSRNOG00000003549	Cnpy2	309.8557152	0.025996969	0.105772794
ENSRNOG00000033835	Dnm1	2532.391893	-0.061048251	0.249761355
ENSRNOG00000005158	Slc24a5	349.6895941	0.023504328	0.095872893
ENSRNOG00000016587	Ninj1	335.3386897	-0.024372773	0.100068331
ENSRNOG00000001295	S100b	507.6893042	0.031963881	0.131446123
ENSRNOG00000017917	Cdhr2	978.6207841	0.014916411	0.061704722
ENSRNOG00000030091	Dusp14	519.9458177	0.021597989	0.089374534
ENSRNOG00000000583	Cdk19	248.98487	-0.022925103	0.095348635
ENSRNOG00000011984	Cxcl14	823.9191822	0.018990095	0.079274103
ENSRNOG00000004763	Sirpa	1817.513803	-0.010971071	0.04581657
ENSRNOG00000050090	Slc6a17	1046.724864	0.015644546	0.065193833
ENSRNOG00000012710	Ubac2	359.8724288	-0.023777123	0.099328039
ENSRNOG00000004650	Begain	243.3599479	0.031679546	0.132976146
ENSRNOG00000018242	Camkk1	641.4084871	0.016317322	0.068576302
ENSRNOG00000013804	Exoc5	211.7256384	0.023214255	0.097254196
ENSRNOG00000002928	Guk1	225.6561985	-0.066961332	0.281569879
ENSRNOG00000043295	1810010H24Rik	204.238473	0.02365361	0.099899234
ENSRNOG00000060141	Epdr1	534.7601367	-0.019838944	0.083846665
ENSRNOG00000024539	Ndufb6	242.105942	-0.027719363	0.116966051
ENSRNOG00000010567	Angel1	257.2389685	0.026355334	0.111887837
ENSRNOG00000009364	NA	465.4910914	-0.019968749	0.084706038
ENSRNOG00000007838	Ddx47	196.1959911	-0.023971426	0.102731434
ENSRNOG00000019143	Galnt2	510.7789629	0.028109019	0.12107813
ENSRNOG00000014415	Hsbp1	2086.531716	-0.014832037	0.063695494
ENSRNOG00000029441	Klhl2	1033.95773	-0.013028684	0.056195888
ENSRNOG00000010058	Spry2	305.751494	-0.021424773	0.092290634
ENSRNOG00000000327	Hace1	218.3379697	0.023017205	0.099574344
ENSRNOG00000001785	Etv5	512.6679394	0.020687753	0.090057563
ENSRNOG00000018441	Ggt7	585.0019917	-0.017963586	0.078104597
ENSRNOG00000050535	NA	306.7569724	0.021391595	0.093140809
ENSRNOG00000019321	Cck	7086.045964	-0.014830943	0.064723108
ENSRNOG00000017032	Atp5f1a	2544.848122	0.013631227	0.060950792
ENSRNOG00000004310	Caskin2	191.698952	0.088537798	0.394123019
ENSRNOG00000004014	Chmp6	294.6948964	0.018287605	0.081440716
ENSRNOG00000054962	Dao	942.0369438	0.01938983	0.086328918
ENSRNOG00000016573	Dgat2	275.3724624	0.029767708	0.13174563
ENSRNOG00000023601	Elavl4	686.0423013	0.016786706	0.074817318
ENSRNOG00000004333	Flywch1	439.6743251	-0.024687573	0.110107966
ENSRNOG00000049985	Gprasp1	698.0525804	-0.014977475	0.065703531
ENSRNOG00000002368	Iars2	270.7994477	-0.027033892	0.120193206
ENSRNOG00000030180	Lrrc10b	1575.905318	0.017910645	0.080019113
ENSRNOG00000031208	NA	194.0201944	0.023460591	0.104661926
ENSRNOG00000010389	Ndrg2	3883.856687	0.015854418	0.070803642
ENSRNOG00000011815	Sgk1	239.6812143	-0.026910144	0.120244725
ENSRNOG00000008061	Nuak1	315.1738369	-0.021043943	0.094342545
ENSRNOG00000014445	Hp1bp3	244.9352671	0.021521709	0.097432344

ENSRNOG00000011287	Minpp1	255.7733484	0.022802388	0.103258598
ENSRNOG00000029910	NA	268.5723547	0.020197573	0.091405265
ENSRNOG00000009649	Slitrk5	264.9875972	-0.019895591	0.089853379
ENSRNOG00000013970	Cdt1	223.5974362	0.019928122	0.090809279
ENSRNOG00000012074	Ifngr1	233.3622093	0.025409447	0.115788678
ENSRNOG00000026942	RGD1311595	261.919279	0.019161825	0.087079906
ENSRNOG00000007726	Mcam	307.6026822	-0.01843373	0.084359943
ENSRNOG00000005775	Phf14	215.9874012	-0.024988081	0.114663596
ENSRNOG00000006369	Alg2	799.2619826	0.018561537	0.085594416
ENSRNOG00000015913	Tspan5	837.5151	-0.014232792	0.065750147
ENSRNOG00000012514	Abhd17b	299.1143245	0.020614931	0.096098615
ENSRNOG00000018783	Bcas2	249.5310613	-0.018288017	0.086275653
ENSRNOG00000012439	Bid	227.4450497	-0.02132796	0.100495262
ENSRNOG00000033688	Cep170b	1455.45658	0.014248015	0.067017555
ENSRNOG00000002389	Morf4l2	781.0188417	0.015715422	0.073671284
ENSRNOG00000019062	NA	328.943577	0.01984642	0.093418398
ENSRNOG00000061684	Ndufa9	454.2578348	-0.017193968	0.079784048
ENSRNOG00000017904	Nol7	355.6269817	0.019375196	0.091488187
ENSRNOG00000021057	Osbp	207.0782175	-0.020223741	0.095458948
ENSRNOG00000018220	Pde4dip	511.4357396	0.017552156	0.082770884
ENSRNOG00000046996	Pea15	323.4829425	-0.0178376	0.08302851
ENSRNOG00000019850	Speg	396.8187309	0.015990718	0.074273554
ENSRNOG00000020660	Cfl1	195.0159757	-0.019470814	0.09346695
ENSRNOG00000013178	Cmip	1015.969475	-0.014587012	0.070102795
ENSRNOG00000022921	Dact2	412.8121027	-0.027496788	0.131784401
ENSRNOG00000052219	Gm2a	192.747805	-0.021333861	0.102674332
ENSRNOG00000010267	Klhdc10	228.1518372	0.019760263	0.094498239
ENSRNOG00000029087	Nap1l3	295.3678679	0.01864121	0.088885439
ENSRNOG00000056371	Pik3ca	260.8151627	-0.019370386	0.093002332
ENSRNOG00000008530	Stoml1	224.429044	-0.02238837	0.107672046
ENSRNOG00000015977	Zfp609	1261.024191	-0.015810764	0.075719687
ENSRNOG00000012906	Bcas1	234.4185216	0.021772334	0.107397664
ENSRNOG00000026787	Disp2	668.8543984	-0.017173294	0.085226638
ENSRNOG00000001454	Eif4h	1438.318594	0.012192473	0.060129312
ENSRNOG00000015301	Elp2	282.2841982	0.0221728	0.107161757
ENSRNOG00000012982	Map9	418.0617795	0.016130812	0.0804742
ENSRNOG00000018860	Mtg1	807.540822	-0.015404529	0.075381426
ENSRNOG00000058677	NA	266.1860217	-0.020341574	0.100275698
ENSRNOG00000060572	NA	421.2728762	-0.014172258	0.070062746
ENSRNOG00000057845	NA	611.6911158	-0.012469891	0.06189902
ENSRNOG00000048606	NA	557.3933432	-0.017226233	0.086109185
ENSRNOG00000019643	Pcyox1l	242.6898894	-0.02180754	0.108959733
ENSRNOG00000002419	Plp1	640.081607	0.025988595	0.129143774
ENSRNOG00000012874	Rgma	240.7872166	0.023490034	0.114071835
ENSRNOG00000011763	Serp1	364.8888084	0.018178428	0.089884303
ENSRNOG00000004103	Trappc6b	206.4292906	-0.027887495	0.136208316
ENSRNOG00000000186	Tst	556.258792	-0.018629251	0.091550846
ENSRNOG00000008794	Ube2e1	387.3151173	0.015594258	0.075530034
ENSRNOG00000007710	Usp20	555.2582513	0.015294301	0.074319033
ENSRNOG00000025923	Zmiz2	1130.410304	0.01265957	0.063190713

ENSRNOG00000014670	Zmynd11	553.386859	0.015885582	0.07706075
ENSRNOG00000011853	Mbd2	195.6009684	-0.022010907	0.110303672
ENSRNOG00000052925	NA	194.2796077	0.051685424	0.260079922
ENSRNOG00000012098	Adcyap1r1	347.7207097	0.017150388	0.087048723
ENSRNOG00000006649	Thrb	485.9891428	0.017612142	0.089281962
ENSRNOG00000059883	NA	726.1800171	0.015686716	0.079891564
ENSRNOG00000048589	NA	193.8900033	-0.018587533	0.094899116
ENSRNOG00000003150	Mpc2	991.1043684	0.01469993	0.075379859
ENSRNOG00000011292	Col1a2	195.7681087	0.049589462	0.256859953
ENSRNOG00000015822	Klf13	335.1334621	-0.019106519	0.098992452
ENSRNOG00000051132	NA	396.91178	0.017497124	0.09054152
ENSRNOG00000009198	Rab6b	2607.96563	-0.00959474	0.04942737
ENSRNOG00000051135	Tug1	224.0476953	0.020817617	0.107280716
ENSRNOG00000010872	Ckb	11396.47292	-0.011764913	0.061213592
ENSRNOG00000006697	Pip4p2	357.260341	-0.016365152	0.084981193
ENSRNOG00000001134	Rfc5	2098.196166	0.010961945	0.057163086
ENSRNOG00000007504	Actr10	500.6626974	0.015059005	0.078783258
ENSRNOG00000055564	RGD1564664	294.2677157	0.0413234	0.217413454
ENSRNOG00000046022	NA	308.3161351	0.016342502	0.086995164
ENSRNOG00000053782	NA	2090.720313	-0.043207148	0.230472136
ENSRNOG00000017622	Npm3	378.7907121	0.018517356	0.098432037
ENSRNOG00000002182	Septin11	317.1569161	0.021375776	0.113350474
ENSRNOG00000014453	Anxa5	422.2787203	-0.017968762	0.096233162
ENSRNOG00000018053	Fech	228.6618193	-0.018912598	0.10154539
ENSRNOG00000005826	NA	649.4243759	-0.046407641	0.249791799
ENSRNOG00000003686	Tbl1x	212.4304923	0.019568373	0.105365549
ENSRNOG00000003980	Ttc1	262.1657394	-0.018715683	0.101130541
ENSRNOG00000005505	Wbp11	264.6083582	-0.017206608	0.092909265
ENSRNOG00000016818	Fgfr3	490.8845891	-0.016646288	0.090405009
ENSRNOG00000043436	Micu1	628.2706637	0.012973339	0.070916491
ENSRNOG00000011709	Pum1	270.7377698	-0.015325174	0.08418134
ENSRNOG00000010141	Ttll1	215.4744437	-0.02830128	0.155390982
ENSRNOG00000021824	Dnajb1	269.6569453	0.014957529	0.082394494
ENSRNOG00000005366	AABR07053472.1	446.7409802	-0.017971821	0.10086636
ENSRNOG00000018655	Adsl	337.7394955	-0.014739966	0.082747764
ENSRNOG00000019020	Bbs2	243.047105	-0.017373973	0.097669127
ENSRNOG00000056870	Gdi1	981.0157319	-0.090045874	0.503745371
ENSRNOG00000046883	Mydgf	292.9770139	0.019705466	0.110973412
ENSRNOG00000060312	NA	328.9988454	-0.016157413	0.089899889
ENSRNOG00000007851	Psma3	507.8511566	0.011096947	0.061385736
ENSRNOG00000013743	Sec61a1	277.6814997	0.017852828	0.100652904
ENSRNOG00000008376	Slc2a3	568.2201797	0.015499893	0.086250122
ENSRNOG00000012751	Tm9sf2	536.289128	0.012810982	0.07190003
ENSRNOG00000052549	Chga	726.3090541	-0.011820069	0.067458886
ENSRNOG00000043006	Epm2aip1	361.5237998	-0.019480871	0.11091701
ENSRNOG00000014758	Lgi1	459.92216	0.013109834	0.074905855
ENSRNOG00000057732	NA	417.5528423	-0.014421072	0.082085567
ENSRNOG00000007393	Ndrg1	199.5305659	0.018893586	0.107693788
ENSRNOG00000001049	Tpt1	3038.47303	-0.010764173	0.061680017
ENSRNOG00000049437	Gpc1	599.6231049	0.013444316	0.077274915

ENSRNOG00000018673	Sec22b	332.4543824	-0.016549977	0.09530566
ENSRNOG00000009495	Src	419.1031439	-0.018362291	0.106058916
ENSRNOG00000010580	Acot7	301.5942825	-0.017471905	0.101692686
ENSRNOG00000011096	Hmgb3	335.8192938	-0.019793095	0.115289048
ENSRNOG00000056703	Atrx	428.7225003	-0.015605021	0.091590796
ENSRNOG00000021153	Fkbp2	510.0475875	-0.037994607	0.223240572
ENSRNOG00000017956	Golga7	595.2229333	0.010381876	0.061441907
ENSRNOG00000013009	Ldha	3396.587023	-0.013711841	0.080885179
ENSRNOG00000029784	Pak1	973.9505418	0.01542595	0.09121234
ENSRNOG00000052025	Tle5	532.121783	0.017045242	0.100692239
ENSRNOG00000048205	Ube2h	338.0911586	0.014124875	0.082508564
ENSRNOG00000010609	Abcf2	368.051908	0.013541324	0.080529843
ENSRNOG00000046548	NA	858.7881122	0.009268204	0.055126122
ENSRNOG00000016770	Calm1	9898.430344	-0.008070022	0.04825325
ENSRNOG00000048617	Ensa	2214.823018	-0.01007808	0.060342376
ENSRNOG00000008393	Tax1bp1	432.5355833	-0.012494037	0.074944685
ENSRNOG00000060266	Exoc2	304.4361938	0.018294285	0.110259606
ENSRNOG00000003253	Qdpr	476.5198094	-0.011166758	0.067255179
ENSRNOG00000006623	Cd302	233.4724573	0.01570463	0.095793726
ENSRNOG00000003958	Commd10	208.6665899	-0.019232568	0.117576529
ENSRNOG00000018212	Abhd17a	770.8836681	-0.011147534	0.0685608
ENSRNOG00000032398	Macir	362.1332231	-0.01337117	0.082391109
ENSRNOG00000011407	Prag1	236.626383	0.01942831	0.119867328
ENSRNOG00000016356	Got1	279.6149082	0.017647065	0.109372446
ENSRNOG00000004518	Cacnb1	795.3238771	0.012177471	0.076239859
ENSRNOG00000049349	Tafa5	868.1724095	0.012661007	0.079184192
ENSRNOG00000003583	Smyd2	818.2463784	-0.011264754	0.070733802
ENSRNOG00000031532	AABR07018167.1	229.5158722	-0.016792773	0.10658446
ENSRNOG00000042037	Smim7	284.0297513	0.015186207	0.096237565
ENSRNOG00000031814	Sort1	258.8172406	-0.015726063	0.099565638
ENSRNOG00000060349	Fam3c	254.942996	-0.016341333	0.104281535
ENSRNOG00000001811	Fgfr1op2	428.5201251	0.011227391	0.071775609
ENSRNOG00000001006	Nptx2	282.7488347	-0.015190225	0.097344583
ENSRNOG00000037298	RGD1309036	1053.571304	0.01477541	0.095067958
ENSRNOG00000021247	Spef1	365.5531427	-0.017401853	0.112697775
ENSRNOG00000025695	Tns3	244.4230497	-0.014494122	0.093740581
ENSRNOG00000042753	Fgf13	1171.449862	0.010694823	0.069515793
ENSRNOG00000009155	Ndufs3	299.4433792	-0.013253417	0.087104678
ENSRNOG00000019222	Tfdp1	258.4649748	-0.014706938	0.09658238
ENSRNOG00000006646	Vopp1	531.3235406	0.012893357	0.084757822
ENSRNOG00000003858	Atp6ap2	2120.389013	-0.011364547	0.0763597
ENSRNOG00000042556	Bmyc	367.4058635	0.010575018	0.071164296
ENSRNOG00000014743	Hagh	602.7179002	-0.010296766	0.069094607
ENSRNOG00000026293	Jun	293.8855902	0.024892134	0.169165691
ENSRNOG00000000390	NA	214.6762106	0.015588192	0.1049695
ENSRNOG00000014096	Nr3c1	204.0216947	-0.017047913	0.116039682
ENSRNOG00000020497	Plekha1	206.5058404	-0.013676343	0.090779946
ENSRNOG00000021085	Sf1	469.2386015	0.010895273	0.072550553
ENSRNOG00000010150	Strbp	347.9875129	-0.015240983	0.101313018
ENSRNOG00000038489	Sumo3	199.7114175	-0.020114719	0.13649101

ENSRNOG00000030245	Tango2	553.8071414	-0.011545111	0.07861549
ENSRNOG00000019861	Tollip	485.176921	0.012668041	0.085384012
ENSRNOG00000009049	Usp11	516.5303318	0.010545095	0.071466097
ENSRNOG00000017243	Bnip3	947.208056	0.008962676	0.062122789
ENSRNOG00000010184	Brk1	1062.460976	-0.00909384	0.063202961
ENSRNOG00000005024	Cdkn2aipnl	267.7175209	-0.014444216	0.102951132
ENSRNOG00000052990	Cers5	360.4669079	0.014138728	0.098130567
ENSRNOG00000006803	Dnajc10	347.4742999	0.013055665	0.089633723
ENSRNOG00000048980	Gng2	761.8126209	-0.009792536	0.067293433
ENSRNOG00000002509	Gnl3l	482.6162735	-0.011452647	0.080437421
ENSRNOG00000006957	Gria4	294.1194292	-0.012905658	0.091377796
ENSRNOG00000005492	Hpcal1	581.2718306	-0.015290569	0.105830739
ENSRNOG00000017466	Kif5b	254.7440494	-0.015676584	0.10970478
ENSRNOG00000023322	Ostc	201.6120726	-0.018292855	0.128358002
ENSRNOG00000004019	Phlda1	211.1680643	0.016959	0.121143027
ENSRNOG00000016528	Ppp1r21	216.6902439	0.016307714	0.112423454
ENSRNOG00000009836	Rbm26	192.6413762	0.01520914	0.107602768
ENSRNOG00000026866	Syn3	279.8121221	0.013655179	0.097606473
ENSRNOG00000009069	Tnfaip1	400.1168221	-0.011073982	0.078104915
ENSRNOG00000019713	Wdr24	4228.082405	0.008473096	0.059901962
ENSRNOG00000020416	Zfr2	203.7871655	-0.033331829	0.23827988
ENSRNOG00000060576	AABR07071223.2	238.7100536	-0.017677577	0.126868469
ENSRNOG00000029028	Mknk2	287.0021526	0.011853359	0.085661555
ENSRNOG00000020717	Bod1	466.2805801	-0.011240065	0.082018409
ENSRNOG00000018425	Dym	343.926848	0.012665347	0.092618641
ENSRNOG00000008292	Hif1a	264.653796	-0.012760458	0.092572178
ENSRNOG00000002214	Klhl8	247.2567289	-0.012447781	0.090918086
ENSRNOG00000061153	Hrh3	1254.754387	-0.008642731	0.063785495
ENSRNOG00000039300	Ahcyl2	245.4478358	-0.013517585	0.101344079
ENSRNOG00000005333	Azin1	441.650724	0.009889428	0.076258598
ENSRNOG00000012852	Bin1	1475.824582	-0.008634873	0.066310904
ENSRNOG00000033261	Fam107a	3626.924792	-0.010256906	0.077457192
ENSRNOG00000005347	Fjx1	425.9135259	0.012635166	0.097597246
ENSRNOG00000055408	Gcat	407.7791442	-0.009424613	0.072584624
ENSRNOG00000025940	Mettl9	797.4118037	0.009766882	0.075089034
ENSRNOG00000023407	Mrpl36	241.7682195	0.012956717	0.096650138
ENSRNOG00000049238	NA	270.5002905	-0.011830522	0.089011302
ENSRNOG00000052192	NA	502.0256311	-0.010970189	0.084601444
ENSRNOG00000005698	NA	203.8804644	-0.016980292	0.131400028
ENSRNOG00000036664	Narf	273.0055419	0.012397056	0.09295773
ENSRNOG00000005003	Ptprn2	975.9350265	-0.008688588	0.065798666
ENSRNOG00000008472	Rbm10	243.1864405	0.015187979	0.113119652
ENSRNOG00000016810	Stmn1	2119.662452	-0.01019145	0.07753844
ENSRNOG00000049530	AABR07012054.1	624.0166601	-0.0082731	0.065348508
ENSRNOG00000003220	H3f3a	350.7323633	-0.011194271	0.087485591
ENSRNOG00000015035	Myo7b	7410.166624	0.005684286	0.044564374
ENSRNOG00000030204	NA	521.4069642	0.010296278	0.081543961
ENSRNOG00000052424	RGD1306556	259.7304002	0.012192944	0.096040018
ENSRNOG00000002972	Spata46	274.4573627	-0.011946307	0.094676357
ENSRNOG00000007988	Timm22	1825.144735	0.007014033	0.055529782

ENSRNOG00000010258	Vhl	195.4430917	-0.013901533	0.110689406
ENSRNOG00000014165	Ssr1	421.2630078	-0.009288769	0.074243624
ENSRNOG00000054775	Fkbp11	4643.50273	0.005644189	0.046496328
ENSRNOG00000008782	Pnisr	240.6868851	0.016642168	0.137112744
ENSRNOG00000021984	Rgs7	466.5255009	0.010335179	0.086155364
ENSRNOG00000051858	NA	215.1523727	-0.011763359	0.09902111
ENSRNOG00000020086	Aar2	1138.206859	-0.0069832	0.063736202
ENSRNOG00000018262	Ampd3	269.0958265	-0.01500648	0.143833177
ENSRNOG00000025619	Ap1g2	2050.408091	-0.005618459	0.055852472
ENSRNOG00000003829	Ap3s1	754.9132757	0.006505961	0.057962498
ENSRNOG00000016544	Arhgef28	226.7483251	0.011219858	0.098082512
ENSRNOG00000037604	Ascc3	286.7473727	0.010252378	0.096195852
ENSRNOG00000013118	Atox1	361.433567	-0.010935087	0.104032602
ENSRNOG00000006770	Brd4	365.6150101	0.007132213	0.075327252
ENSRNOG00000017883	Camsap1	353.2680968	-0.008856307	0.085562067
ENSRNOG00000013538	Capza1	196.762308	0.010592275	0.111780645
ENSRNOG00000017201	Cd2bp2	401.1081908	-0.013184682	0.121041829
ENSRNOG00000022586	Cdk9	756.4434869	-0.006222064	0.066387462
ENSRNOG00000006792	Cep57	214.0336655	-0.012993043	0.11467945
ENSRNOG00000005551	Derl1	358.5516383	-0.010258314	0.093386788
ENSRNOG00000009063	Dnajc15	229.5799197	-0.011028002	0.109560053
ENSRNOG00000016640	Dner	654.0893513	-0.007917321	0.078565135
ENSRNOG00000025025	Dnttip2	270.8368542	-0.009980971	0.090816023
ENSRNOG00000000070	Dr1	194.8720643	-0.00942592	0.101495187
ENSRNOG00000011518	Dusp26	729.5768625	-0.006891917	0.070574923
ENSRNOG00000024025	Edc4	253.1795773	-0.011038342	0.094307226
ENSRNOG00000029941	Grina	3513.940173	-0.005339335	0.05124594
ENSRNOG00000016046	Hecw1	653.6904776	-0.007009384	0.074178784
ENSRNOG00000026235	Hk3	449.0296986	0.007948835	0.078941302
ENSRNOG00000008984	Irak1bp1	231.7056196	-0.010584723	0.094843713
ENSRNOG00000015843	Itfg1	1268.26979	0.007949625	0.072050909
ENSRNOG00000015417	Kansl3	332.5830466	-0.008440256	0.090187949
ENSRNOG00000061499	Kmt2d	601.1910773	-0.009549278	0.088159658
ENSRNOG00000002835	Luc7l3	297.6933794	-0.011774744	0.103227603
ENSRNOG00000001376	Mettl7a	273.5960838	-0.019371452	0.166781982
ENSRNOG00000015428	Mff	1532.831572	-0.005501645	0.058426338
ENSRNOG00000015546	MGC116121	325.053133	-0.008623317	0.079673792
ENSRNOG00000002436	Mmd	2559.437288	-0.006048611	0.061505187
ENSRNOG00000056325	Mocs2	290.8248757	-0.008330615	0.087794785
ENSRNOG00000032297	Msmo1	884.961797	0.007639434	0.074793632
ENSRNOG00000052188	NA	291.9391992	0.011457028	0.097295815
ENSRNOG00000052785	NA	269.6893318	0.011692604	0.103766726
ENSRNOG00000023720	NA	832.7191927	-0.008361469	0.077729758
ENSRNOG00000049593	NA	209.5998852	-0.010948336	0.103324412
ENSRNOG00000015848	NA	279.7732888	-0.009973927	0.096454999
ENSRNOG00000051831	NA	1674.12849	-0.022513224	0.231818674
ENSRNOG00000059394	NA	1706.25324	-0.025918443	0.280130605
ENSRNOG00000016470	Ndufa10l1	899.7042663	0.007443031	0.066318745
ENSRNOG00000049951	NEWGENE_1310680	1788.415674	0.006587961	0.06218653
ENSRNOG00000014795	Nr2f1	609.9074748	-0.007452535	0.077720046

ENSRNOG00000002205	Ociad1	1407.15731	-0.006114142	0.054206759
ENSRNOG00000018506	Olr46	255.3487886	0.009549099	0.096395946
ENSRNOG00000011311	Papss1	418.4745162	-0.011904967	0.127849617
ENSRNOG00000025991	Pex1	393.395073	0.008661273	0.074190546
ENSRNOG00000012091	Ppa2	292.3832327	0.011937572	0.11283487
ENSRNOG00000030667	Ppm1b	363.4012208	-0.009157837	0.082727521
ENSRNOG00000001269	Ppp1cc	1004.342812	-0.005753862	0.052380673
ENSRNOG00000005045	Ppp2r5e	384.6245067	0.007701157	0.077463375
ENSRNOG00000015780	Rcn2	813.9431444	0.008602428	0.091265229
ENSRNOG00000015772	RGD1560341	221.7667078	-0.010077986	0.096961194
ENSRNOG00000015616	Rgs14	234.1645069	-0.011040711	0.105014722
ENSRNOG00000059857	Rnd1	348.7616808	-0.010195102	0.092288039
ENSRNOG00000024322	Shroom2	337.3111704	0.008800957	0.086412745
ENSRNOG00000024930	Smim19	387.5242099	0.006991127	0.072485574
ENSRNOG00000015095	Spryd7	223.9973309	0.011374763	0.09643793
ENSRNOG00000018293	Strip1	367.860447	0.007881601	0.083716969
ENSRNOG00000021808	Tecr	262.4209599	0.030484632	0.309092623
ENSRNOG00000030418	Tmem47	366.1356966	0.008113503	0.086943409
ENSRNOG00000012124	Trappc13	847.1410368	-0.007212732	0.07140192
ENSRNOG00000013381	Tsg101	676.2915773	-0.008037352	0.069362398
ENSRNOG00000019282	Ubqln1	698.9390365	0.006402973	0.061583773
ENSRNOG00000012184	Urgcp	250.3542375	0.009664149	0.101485597
ENSRNOG00000000901	Vkorc1l1	222.08042	-0.011393393	0.099529147
ENSRNOG00000014903	Zfyve27	229.7530035	-0.01111027	0.100287501
ENSRNOG00000011589	Camk2d	352.0975061	-0.009101522	0.099723668
ENSRNOG00000019532	Emc10	583.515273	0.008523155	0.094032559
ENSRNOG00000009549	Fbxo3	379.7190778	-0.008109009	0.089439783
ENSRNOG00000009076	Ttpal	380.160881	0.007957854	0.088061984
ENSRNOG00000057428	U6	329.9789273	-0.00763813	0.0848868
ENSRNOG00000001204	Ankrd13a	793.5015818	0.005409271	0.065986188
ENSRNOG00000014828	Avpi1	406.6732926	0.005807307	0.070418248
ENSRNOG00000008390	Dcun1d5	408.7663793	-0.00597226	0.074519611
ENSRNOG00000018549	Fbh1	286.0852942	0.007066517	0.087170557
ENSRNOG00000036663	Foxk2	363.592857	0.007330431	0.08612709
ENSRNOG00000000774	Gabbr1	2746.758675	-0.004329487	0.052350883
ENSRNOG00000003512	Gabra1	1101.741689	-0.006746883	0.079577807
ENSRNOG00000016385	Gabrd	392.1067555	-0.008780875	0.099761764
ENSRNOG00000018282	Gda	423.741734	-0.011445483	0.139265863
ENSRNOG00000003661	Jpt1	403.5385415	-0.012811751	0.148198655
ENSRNOG00000050200	Kdm3b	358.1759413	0.007056674	0.088936133
ENSRNOG00000028021	NA	233.6991994	-0.008858211	0.10165912
ENSRNOG00000027079	NA	423.9584828	-0.006346332	0.079003067
ENSRNOG00000047384	NA	432.2956123	-0.007000987	0.087806942
ENSRNOG00000019047	Ola1	820.4607848	0.006555246	0.074643983
ENSRNOG00000015642	Pabpc4	247.1485788	0.008003304	0.095633273
ENSRNOG00000033280	Pam	962.8603324	0.005428469	0.067620144
ENSRNOG00000029574	Rps4x	912.0398024	0.006278538	0.074050408
ENSRNOG00000016603	Rtn2	579.7406922	-0.005942814	0.075132221
ENSRNOG00000009057	Sec62	219.56207	-0.01027055	0.12372676
ENSRNOG00000011977	Sema5a	212.7735603	-0.012612211	0.152518299

ENSRNOG00000014369	Slc27a4	391.7533857	0.006351783	0.07700529
ENSRNOG00000009209	Slitrk1	509.5546007	-0.005536443	0.06920738
ENSRNOG00000007346	Tamalin	328.9357122	0.010080395	0.127038022
ENSRNOG00000002828	Tob1	294.7629369	-0.007633203	0.091619243
ENSRNOG00000000611	Ube2d1	284.4492535	-0.007875782	0.091831585
ENSRNOG00000008377	Wdtc1	355.3626999	0.009620186	0.115698931
ENSRNOG00000014564	Yipf5	226.9027568	0.008941585	0.100429176
ENSRNOG00000017695	AABR07012795.1	192.5962077	0.008297848	0.136792922
ENSRNOG00000028958	AABR07024795.1	197.3934226	0.00820423	0.107139857
ENSRNOG00000000799	Abcf1	327.77091	-0.005602132	0.081993478
ENSRNOG00000004731	Ano3	329.2958163	-0.005035326	0.095368207
ENSRNOG00000001551	Atp5pf	329.4057343	0.023838201	0.413306242
ENSRNOG00000008283	B4galt5	238.2290654	-0.007834054	0.144553243
ENSRNOG00000024410	Blvrb	259.1280612	0.007481144	0.105630849
ENSRNOG00000046647	Bpnt2	421.771203	0.004652444	0.078852386
ENSRNOG00000052963	Brox	207.3402843	-0.006225226	0.110966341
ENSRNOG00000021317	Cct2	675.9857104	-0.006757178	0.091439253
ENSRNOG00000009642	Cct4	792.3470247	0.00622362	0.080556547
ENSRNOG00000059485	Cdkl3	642.7867519	0.00633811	0.08966974
ENSRNOG00000045892	Cfl2	658.6598996	-0.005116919	0.072399945
ENSRNOG00000016313	Ctnnbip1	440.4437599	-0.005856553	0.08660466
ENSRNOG00000023458	Dcaf12l1	490.1522514	-0.00518899	0.077656375
ENSRNOG00000010048	Dctn1	1502.083942	-0.003016154	0.047956845
ENSRNOG00000019422	Egr1	1052.59849	-0.015068667	0.304064912
ENSRNOG00000015719	Egr4	730.8944997	0.015838529	0.248518079
ENSRNOG00000055760	Eif3i-ps1	218.8812672	0.005397452	0.098795381
ENSRNOG00000008666	Etl4	314.7959567	-0.008257879	0.119118915
ENSRNOG00000012183	Glrx	362.0475373	-0.007039677	0.095553164
ENSRNOG00000049560	Glul	2676.142701	0.003829147	0.077747734
ENSRNOG00000016357	Golm2	670.6859575	0.003634377	0.060220615
ENSRNOG00000021145	Gpr137	196.8199157	0.006443901	0.133996608
ENSRNOG00000054204	Gria2	2285.725365	-0.003504364	0.056707031
ENSRNOG00000016218	Gtf3c1	317.5651549	0.005762817	0.096320546
ENSRNOG00000012060	Gucy1b1	605.6201618	-0.00424643	0.082579282
ENSRNOG00000007034	Hipk2	193.1984341	-0.007280411	0.105119496
ENSRNOG00000027761	Htatsf1	201.0187953	-0.009544119	0.136619993
ENSRNOG00000014616	Iars1	268.3822297	0.004488264	0.087570954
ENSRNOG00000030004	Ier3ip1	291.2443028	0.005850472	0.090125008
ENSRNOG00000007604	Igsf8	327.7566406	-0.005854514	0.094719757
ENSRNOG00000014252	Irf2bp1	285.4756653	-0.004339232	0.088919287
ENSRNOG00000004516	Itgbl1	195.3486638	0.005875482	0.104555061
ENSRNOG00000016467	Kctd1	558.1818305	-0.00581755	0.093923143
ENSRNOG00000046794	Khdrbs1	494.3154591	-0.005777514	0.085741263
ENSRNOG00000004680	Kif5c	1483.811433	-0.0032815	0.065006407
ENSRNOG00000011572	Klc1	3096.170001	-0.003377694	0.048213982
ENSRNOG00000007981	Klhl23	319.9442518	-0.004432456	0.083314759
ENSRNOG00000019036	Ldhd	822.2440265	-0.005144633	0.074997649
ENSRNOG00000012988	Lix1	481.6076916	0.0041094	0.080190559
ENSRNOG00000005825	Lyz2	638.275541	-0.007596096	0.101117513
ENSRNOG00000007769	Marchf2	897.2428391	-0.003789606	0.077407542

ENSRNOG00000007632	Mss51	1905.442726	-0.00407129	0.060910131
ENSRNOG00000059998	NA	252.1483503	0.007537296	0.102340365
ENSRNOG00000042696	NA	390.934948	-0.006137578	0.094434598
ENSRNOG00000055853	NA	578.1115445	-0.00474288	0.075999299
ENSRNOG00000049814	NA	344.5360326	0.007199236	0.124788217
ENSRNOG00000017251	NA	242.6162664	0.004777223	0.090482762
ENSRNOG00000023605	Ncbp1	199.5964077	0.007684596	0.10684552
ENSRNOG00000013658	Nefl	2327.311762	0.002810957	0.058354758
ENSRNOG00000028417	Neurod2	331.763911	-0.008079063	0.116378463
ENSRNOG00000045686	Nfs1	192.0174131	0.007094137	0.114000728
ENSRNOG00000014162	Plekhb2	1273.446353	-0.00412238	0.062897826
ENSRNOG00000011951	Plk2	825.0008934	-0.005014721	0.094023743
ENSRNOG00000027520	Pls3	273.0433608	-0.004750538	0.093466859
ENSRNOG00000058317	Pnck	455.3464297	0.005603228	0.080173835
ENSRNOG00000002724	Prpsap2	330.0390239	-0.007372543	0.121485583
ENSRNOG00000042960	Rgcc	448.8204924	0.00403367	0.083365236
ENSRNOG00000002545	RGD1309748	286.4085829	0.008330519	0.117341567
ENSRNOG00000057774	RGD1560212	246.8024924	0.005667698	0.09946297
ENSRNOG00000013389	Rgs7bp	256.2275184	-0.007560722	0.148232479
ENSRNOG00000014691	Ric3	625.2164242	-0.004936525	0.071966015
ENSRNOG00000023376	Riok3	312.078048	0.005531304	0.098206451
ENSRNOG00000045535	Rtl8a	313.9883286	-0.005573076	0.082443028
ENSRNOG00000007099	S100pbp	474.9392575	0.008003198	0.102858928
ENSRNOG00000012329	Saraf	1465.332542	-0.005201127	0.073537118
ENSRNOG00000013331	Sdha	576.4502326	-0.006282659	0.098347872
ENSRNOG00000038933	Spcs3	333.7855497	-0.005475043	0.092127613
ENSRNOG00000010882	Sptlc1	212.7698753	0.006033129	0.113395913
ENSRNOG00000056153	Tent5b	867.5942243	0.005263055	0.076460394
ENSRNOG00000015460	Tm4sf20	1298.931387	0.003361205	0.062530854
ENSRNOG00000012860	Tmem184c	208.4229522	-0.005309734	0.090264319
ENSRNOG00000010204	Tmem9	391.8976955	-0.005071093	0.072934048
ENSRNOG00000004544	Ube2e3	943.1795408	0.004977159	0.092289118
ENSRNOG00000013505	Vdac2	199.8316092	-0.006345264	0.112460962
ENSRNOG00000019915	Xrcc1	289.95935	-0.005918591	0.089231061
ENSRNOG00000040205	Zcchc24	289.7914179	0.006905452	0.102803066
ENSRNOG00000004581	Zdhhc9	196.7369826	0.005306969	0.104280523
ENSRNOG00000033176	Zrsr2	221.8260642	0.005414808	0.099066784
ENSRNOG00000053084	Fuz	466.9461704	-0.003693818	0.077781915
ENSRNOG00000053790	NA	307.7610737	0.004501339	0.095642619
ENSRNOG00000001283	Anapc7	239.7836012	-0.004954186	0.117632769
ENSRNOG00000019249	Ap3b2	928.7867913	0.002751337	0.06636471
ENSRNOG00000000396	Ddx50	226.4826978	-0.004551552	0.103757459
ENSRNOG00000018796	Herpud1	231.4798538	0.004431438	0.102616683
ENSRNOG00000059402	Itgb1bp1	263.0266347	0.003741845	0.08970355
ENSRNOG00000004090	NA	229.3336743	-0.004693522	0.112915089
ENSRNOG00000004139	Ndel1	365.6270552	-0.003431602	0.082210622
ENSRNOG00000008747	Plekha5	254.1548442	-0.004549915	0.1086084
ENSRNOG00000012747	Spock1	204.9822106	0.004070549	0.095883194
ENSRNOG00000009535	Stoml2	211.4553119	0.00429327	0.094218841
ENSRNOG00000004307	Tor3a	192.2602689	0.004429423	0.097838275

ENSRNOG00000048699	Vps37d	213.597231	0.004058602	0.096483007
ENSRNOG00000059593	Cetn2	213.536211	0.004471696	0.12008524
ENSRNOG00000007014	Cnksr2	555.6716416	-0.002615465	0.068373801
ENSRNOG00000023356	Eif5b	290.1366901	0.004067193	0.110116853
ENSRNOG00000015233	Etfa	232.2199303	0.004659124	0.134756648
ENSRNOG00000048738	Hdac6	285.269937	0.003947537	0.112089288
ENSRNOG00000004084	Lratd1	231.2505463	-0.005371068	0.135131219
ENSRNOG00000006093	LRRTM1	334.3294014	-0.003520421	0.09401097
ENSRNOG00000017583	Mapkap1	210.5247294	0.003445834	0.097748032
ENSRNOG00000010735	Nfyc	329.6886223	-0.002906668	0.085005505
ENSRNOG00000008981	Pdcd6ip	357.8726127	0.003268183	0.093134108
ENSRNOG00000002821	Phlpp1	257.7788484	-0.003495897	0.100216533
ENSRNOG00000012722	Ppdpf	338.8661959	-0.003505691	0.098438404
ENSRNOG00000046535	Ppm1m	366.501576	-0.003015788	0.09027503
ENSRNOG00000013360	Prkcsh	954.4906434	-0.002872344	0.077411154
ENSRNOG00000032463	Rap1a	323.3023072	-0.002754944	0.081691319
ENSRNOG00000017604	Ripor1	663.5613679	0.00243242	0.06485448
ENSRNOG00000054626	Rps8	207.8547072	0.021340356	0.625639447
ENSRNOG00000002319	Tsn	332.8995918	-0.002589942	0.075505632
ENSRNOG00000017456	Vstm2b	422.3106366	-0.003665176	0.093771856
ENSRNOG00000002028	Tmem50b	724.3386833	0.002729086	0.082907238
ENSRNOG00000001314	Fam20c	338.8568192	-0.002534513	0.079508625
ENSRNOG00000021102	Scn1b	1434.344545	0.001739151	0.054703553
ENSRNOG00000008456	Thoc5	394.5629054	-0.002708957	0.086244895
ENSRNOG00000009243	Oaf	383.7313511	0.003400746	0.115776475
ENSRNOG00000001492	Slc8a2	724.4172658	-0.001934427	0.066550648
ENSRNOG00000009114	Fam110b	228.5999866	-0.002584733	0.091634821
ENSRNOG00000014291	Elp3	634.3666958	0.002072693	0.076298245
ENSRNOG00000012383	Ndufc2	1077.869251	-0.001538221	0.056399741
ENSRNOG00000016530	Nsa2	252.4637843	-0.002535757	0.098206423
ENSRNOG00000037647	Ostm1	263.0796262	-0.003086825	0.116155767
ENSRNOG00000028504	Socs5	373.9464708	-0.002188926	0.083824891
ENSRNOG00000060561	Gdpd1	265.5120383	-0.002814802	0.110787011
ENSRNOG00000020837	Cd300lg	550.5387091	0.002253139	0.092705618
ENSRNOG00000007442	Ubl7	612.9646746	-0.001761022	0.071931706
ENSRNOG00000054061	AABR07052327.1	591.3506619	-0.002210976	0.103302961
ENSRNOG00000027654	Appbp2	221.8255064	0.002190454	0.10243155
ENSRNOG00000015213	Fxn	222.3002396	0.002995481	0.137853034
ENSRNOG00000010855	NA	208.6120155	-0.002113279	0.101143361
ENSRNOG00000014215	Klf9	667.5107008	0.001776543	0.091803589
ENSRNOG00000007172	Map4k3	283.8133413	0.001683673	0.087852683
ENSRNOG00000049681	AABR07031491.2	1136.626978	-0.001336226	0.08586167
ENSRNOG00000055795	AABR07035832.1	448.0206661	0.001096047	0.070039846
ENSRNOG00000030269	Atp2b2	1340.37676	0.001134489	0.06660175
ENSRNOG00000012206	Dennd5a	1054.415736	-0.000895894	0.057469167
ENSRNOG00000033653	Mpp3	226.731992	0.001887573	0.114019618
ENSRNOG00000003226	Mprip	830.6220381	-0.001156296	0.072062568
ENSRNOG00000051120	NA	253.5587249	0.001364015	0.089308522
ENSRNOG00000019848	Rbm39	344.7332842	-0.002076138	0.126224068
ENSRNOG00000058586	Tyro3	1410.333978	0.000956666	0.054353881

ENSRNOG00000025580	Ube2v1	1218.732796	-0.00100097	0.064352717
ENSRNOG00000012186	Golph3	317.3566425	0.001181324	0.085249961
ENSRNOG00000021663	Vxn	1542.853539	-0.001171709	0.08360665
ENSRNOG00000009862	Olfm1	12467.26972	0.000736854	0.054729292
ENSRNOG00000006975	Wasl	524.8065094	0.000951653	0.074406637
ENSRNOG00000026466	Lrrtm3	229.9543046	0.001354074	0.115332527
ENSRNOG00000038025	NA	431.4194529	0.000900242	0.075871059
ENSRNOG00000058896	NA	617.0657068	-0.001572878	0.140117362
ENSRNOG00000014714	Trpv6	294.5501319	0.000916075	0.08574637
ENSRNOG00000020230	Pias4	527.5025308	-0.00072558	0.071502177
ENSRNOG00000017426	Rragc	398.3181835	-0.000734175	0.089079719
ENSRNOG00000001931	Fgf12	309.698233	0.000671422	0.09243258
ENSRNOG00000004239	NA	203.5620358	0.000887791	0.120881856
ENSRNOG00000060107	Dnajb14	634.7694872	0.000266841	0.066164549
ENSRNOG00000013002	Gpbp1	274.438847	-0.000428001	0.108334487
ENSRNOG00000017023	Tcf25	603.6159094	0.000391758	0.094328657
ENSRNOG00000010512	Yipf1	276.5116043	0.000336651	0.096869657
ENSRNOG00000011211	Pex5l	439.0038043	0.00029402	0.111324396
ENSRNOG00000018818	Txnl1	477.9918761	-0.00026232	0.107075235
ENSRNOG00000059919	Mtg2	200.3813963	6.39581E-05	0.098968389
ENSRNOG00000005183	Rtf1	452.140924	-1.17838E-05	0.08015071
ENSRNOG00000002191	0610040J01Rik	63.77873771	0.386025076	0.178973822
ENSRNOG00000034107	1110038F14Rik	27.37393045	0.233959745	0.243292109
ENSRNOG00000060879	1500009L16Rik	105.8853632	0.072466316	0.130295405
ENSRNOG00000014966	1600014C10Rik	10.65429032	-0.505922518	0.434627848
ENSRNOG00000036888	1700001P01Rik	99.74287668	-0.117578906	0.152117953
ENSRNOG00000038738	1700019D03Rik	30.11698951	-0.481187131	0.279956215
ENSRNOG00000047876	1700028K03Rik	34.3829842	0.025106984	0.240722517
ENSRNOG00000045541	1700030J22Rik	12.8058283	-0.422645754	0.360741581
ENSRNOG00000046155	1700102P08Rik	15.8676272	-0.013818027	0.340726201
ENSRNOG00000009135	1700109H08Rik	128.3072274	0.060447781	0.129956268
ENSRNOG00000008244	2700049A03Rik	27.71444767	0.271678023	0.2925258
ENSRNOG00000023431	2810021J22Rik	38.03246087	-0.028948306	0.202336038
ENSRNOG00000017133	4833439L19Rik	49.5434346	-0.126318701	0.197532652
ENSRNOG00000047310	4930444P10Rik	8.672781452	0.291040207	0.435029779
ENSRNOG00000028677	4930503L19Rik	30.23169233	0.634084094	0.266318
ENSRNOG00000049537	4930523C07Rik	7.978137637	0.856049914	0.497546647
ENSRNOG00000058198	5S_rRNA	8.156246534	-0.074452997	0.414148007
ENSRNOG00000028896	A2m	19.06518782	0.415276132	0.302903939
ENSRNOG00000013445	Aaas	25.97114967	0.084222298	0.267420844
ENSRNOG00000014431	AABR07000222.1	118.540652	0.183360453	0.134077595
ENSRNOG00000047746	AABR07000398.1	121.8728372	-0.16625916	0.179567458
ENSRNOG00000017678	AABR07001064.1	32.48741768	-0.141384606	0.225842722
ENSRNOG00000042547	AABR07001902.1	69.02804326	-1.009446227	0.722249975
ENSRNOG00000060429	AABR07002065.2	60.55562455	0.145116861	0.189416189
ENSRNOG00000048028	AABR07002546.1	30.12603672	0.039284947	0.225699522
ENSRNOG00000060587	AABR07002627.1	13.81825341	0.145629643	0.39356003
ENSRNOG00000054609	AABR07002683.1	151.2609801	-0.215494969	0.112992885
ENSRNOG00000018928	AABR07002774.1	9.290272002	0.224523803	0.457356766
ENSRNOG00000053366	AABR07002784.1	32.29168287	-0.541562085	0.221299046

ENSRNOG00000050453	AABR07002848.1	62.13692352	-0.068501649	0.172964132
ENSRNOG00000055519	AABR07002868.1	11.09828635	0.486306279	0.402354479
ENSRNOG00000056062	AABR07002870.2	34.68544273	-0.997271793	0.522987047
ENSRNOG00000014380	AABR07003176.1	67.58362861	-0.084881361	0.157745648
ENSRNOG00000050257	AABR07003273.1	49.46025349	-0.124074539	0.177025851
ENSRNOG00000021484	AABR07003537.1	55.43907814	0.204281329	0.199427914
ENSRNOG00000016498	AABR07004112.1	50.23507956	-0.43169004	0.224322898
ENSRNOG00000051102	AABR07004269.3	44.86196083	-0.134414691	0.191947667
ENSRNOG00000018518	AABR07005779.1	38.90574743	0.090426826	0.208091504
ENSRNOG00000053539	AABR07005886.1	12.16999639	0.082766288	0.365534386
ENSRNOG00000027374	AABR07005985.1	15.93716149	-0.047424034	0.333775465
ENSRNOG00000056396	AABR07006120.1	154.3839893	-0.094857797	0.117443906
ENSRNOG00000017884	AABR07006160.1	89.18552348	-0.290304943	0.161631452
ENSRNOG00000017438	AABR07007068.1	45.79959924	0.232354981	0.206795783
ENSRNOG00000047236	AABR07008066.2	27.87606172	-0.175616914	0.241318025
ENSRNOG00000031378	AABR07009715.1	21.07109097	0.443976198	0.28682949
ENSRNOG00000055556	AABR07010601.1	10.69411244	0.617910919	0.504107111
ENSRNOG00000032715	AABR07010705.1	10.17112131	-0.44473648	0.431091397
ENSRNOG00000000889	AABR07011057.1	8.778170479	-0.219385701	0.44065754
ENSRNOG00000002223	AABR07012587.1	103.8971107	-0.097164317	0.138303197
ENSRNOG00000053898	AABR07013208.1	15.92236092	-0.583205958	0.476084709
ENSRNOG00000003606	AABR07014550.1	45.14333467	-0.233972887	0.796712943
ENSRNOG00000033207	AABR07014676.1	156.7936844	0.016462638	0.131772904
ENSRNOG00000024829	AABR07014804.1	22.73762765	-0.107959499	0.287391258
ENSRNOG00000027791	AABR07015346.1	148.8708695	-0.193049833	0.114925527
ENSRNOG00000058474	AABR07015941.1	15.32493736	-0.375720109	0.332230881
ENSRNOG00000049099	AABR07017250.1	137.8432198	-0.064169644	0.132433837
ENSRNOG00000049583	AABR07017733.1	24.91142538	-0.203855871	0.295511125
ENSRNOG00000048927	AABR07018533.1	43.53398075	-0.00592075	0.197393062
ENSRNOG00000057291	AABR07019088.1	11.39522238	-0.222215871	0.37734537
ENSRNOG00000058057	AABR07019403.1	38.04399418	0.277863216	0.213947028
ENSRNOG00000026917	AABR07021573.2	98.30692145	-0.071140728	0.128093989
ENSRNOG00000052574	AABR07021955.1	8.59572888	0.702141858	0.472316344
ENSRNOG00000046460	AABR07022144.1	16.8405844	0.492240317	0.331291994
ENSRNOG00000031098	AABR07022157.1	104.245662	0.532815712	0.433177985
ENSRNOG00000046132	AABR07024439.1	23.54994483	0.643081595	0.335769491
ENSRNOG00000046720	AABR07024442.1	15.44195993	0.263885252	0.329664756
ENSRNOG00000030299	AABR07024742.1	32.66085744	0.155851694	0.274233873
ENSRNOG00000038744	AABR07025819.1	11.18966363	0.007297132	0.444623721
ENSRNOG00000038458	AABR07026240.1	147.6572224	0.027132763	0.114949282
ENSRNOG00000014275	AABR07026291.1	141.8681727	-0.008733845	0.112368072
ENSRNOG00000049442	AABR07026317.1	84.59503552	-0.139099779	0.140327078
ENSRNOG00000027309	AABR07026805.1	15.94952413	0.237977533	0.425853799
ENSRNOG00000058895	AABR07027069.1	11.127451	-0.316618211	0.49133147
ENSRNOG00000055463	AABR07027150.1	60.08436639	-0.098634676	0.177854745
ENSRNOG00000060680	AABR07027309.1	24.29904542	0.087879523	0.294797057
ENSRNOG00000057759	AABR07027854.1	30.29044651	-0.281891273	0.258626382
ENSRNOG00000013237	AABR07027925.1	8.069525572	0.372947196	0.51180917
ENSRNOG00000021292	AABR07028027.1	87.76448129	-0.174175704	0.134649219
ENSRNOG00000049614	AABR07028488.1	9.059144931	-0.57133548	0.409810204

ENSRNOG00000040024	AABR07028805.1	44.99340595	0.008083449	0.194729935
ENSRNOG00000009861	AABR07028859.1	10.4797384	-0.388190539	0.374685631
ENSRNOG00000058094	AABR07028902.1	29.28997145	-0.113620428	0.234030512
ENSRNOG00000057978	AABR07028997.1	17.94486369	-0.114674134	0.353250052
ENSRNOG00000046330	AABR07029581.1	166.7032684	-0.175061101	0.115796993
ENSRNOG00000009104	AABR07029605.1	164.8018501	-0.32315171	0.136657095
ENSRNOG00000038478	AABR07029613.1	19.77238295	-0.041969499	0.279365085
ENSRNOG00000046598	AABR07029742.2	9.986931712	0.057007498	0.422188414
ENSRNOG00000045669	AABR07030335.1	16.13455638	0.521724087	0.366438815
ENSRNOG00000056057	AABR07030425.1	27.2390247	0.06562621	0.248469451
ENSRNOG00000030224	AABR07030729.1	130.2138332	0.02852642	0.134301233
ENSRNOG00000053469	AABR07030861.1	164.1878673	0.21290327	0.116392312
ENSRNOG00000032442	AABR07030911.1	14.78810821	-0.640070715	0.321717941
ENSRNOG00000015645	AABR07031480.1	39.33292565	-0.014790951	0.214921437
ENSRNOG00000030364	AABR07031666.1	13.20099962	-1.118575763	0.442413814
ENSRNOG00000056958	AABR07031669.1	12.21668535	0.534177468	0.430265287
ENSRNOG00000060281	AABR07031691.1	11.91855863	0.517280541	0.468596465
ENSRNOG00000019744	AABR07032097.1	85.64732935	-0.317330405	0.171501583
ENSRNOG00000061507	AABR07032328.1	26.54510491	-0.769295697	0.319358409
ENSRNOG00000024426	AABR07032520.1	9.42734072	0.247194667	0.455603324
ENSRNOG00000038211	AABR07032856.1	16.24779331	-0.196311704	0.327065091
ENSRNOG00000055484	AABR07033465.1	19.60557313	0.149498881	0.310044317
ENSRNOG00000023917	AABR07034362.1	52.08938725	0.034968123	0.192757878
ENSRNOG00000046969	AABR07034438.1	13.66222341	0.509934596	0.3674178
ENSRNOG00000038370	AABR07034632.1	35.30184992	-0.00167161	0.227023576
ENSRNOG00000033115	AABR07034641.1	27.99775224	-0.242829281	0.236888809
ENSRNOG00000039674	AABR07034657.1	9.137500297	-0.247990353	0.409067953
ENSRNOG00000001071	AABR07035091.1	40.92722047	0.102144786	0.220707683
ENSRNOG00000039819	AABR07035338.1	58.42816017	-0.016706801	0.185505254
ENSRNOG00000050264	AABR07035357.2	16.32253265	0.166026408	0.340027777
ENSRNOG00000056768	AABR07035428.2	107.5835394	-0.037371268	0.12693441
ENSRNOG00000061371	AABR07035541.3	70.07820638	-0.172417971	0.155061407
ENSRNOG00000048277	AABR07035787.1	63.28223277	-0.126384935	0.153299213
ENSRNOG00000055283	AABR07036463.1	50.4655259	-0.057630691	0.189617156
ENSRNOG00000056398	AABR07036855.1	75.55431967	-0.126898929	0.149788992
ENSRNOG00000023163	AABR07037058.1	18.78110321	-0.237653862	0.317787846
ENSRNOG00000003158	AABR07037343.1	151.9991361	-0.180619829	0.110636378
ENSRNOG00000060137	AABR07037489.1	35.01881824	-0.167873764	0.249969512
ENSRNOG00000047491	AABR07037520.1	46.83514144	0.059534249	0.184092382
ENSRNOG00000004319	AABR07038019.1	48.03502034	-0.358371919	0.182185589
ENSRNOG00000051159	AABR07038578.1	13.81382026	-0.224189162	0.338533601
ENSRNOG00000051341	AABR07038849.1	98.70466781	-0.138741817	0.128823331
ENSRNOG00000051544	AABR07038871.1	10.24545016	0.091629655	0.434848897
ENSRNOG00000048213	AABR07039304.1	8.259289418	0.263958472	0.435556205
ENSRNOG00000046832	AABR07039307.1	29.57399697	-0.076960548	0.230289615
ENSRNOG00000022286	AABR07039316.1	86.2995347	0.332773317	0.191865797
ENSRNOG00000051300	AABR07039328.2	104.1371584	0.058252768	0.145509271
ENSRNOG00000018301	AABR07039356.1	159.964722	0.026788224	0.12545424
ENSRNOG00000002789	AABR07039648.1	135.053124	-0.425989092	0.116463359
ENSRNOG00000037770	AABR07039936.1	10.28458935	0.621025166	0.454222533

ENSRNOG00000051669	AABR07040855.1	77.98313846	-0.076418981	0.156596119
ENSRNOG00000050960	AABR07040864.1	10.39947238	0.245233216	0.394632329
ENSRNOG00000005402	AABR07040938.1	20.98673949	0.334571672	0.307597445
ENSRNOG00000005430	AABR07040944.1	12.21428732	-0.290022469	0.34956534
ENSRNOG00000018923	AABR07041089.1	11.19445268	0.71177905	0.503807173
ENSRNOG00000006459	AABR07041140.1	33.04468113	0.19785785	0.224860631
ENSRNOG00000003734	AABR07041600.1	53.41170132	-0.182866535	0.194208883
ENSRNOG00000033451	AABR07042892.1	35.81260677	0.102216915	0.206101453
ENSRNOG00000032666	AABR07042915.1	12.82495387	-0.045185613	0.374012668
ENSRNOG00000046163	AABR07042936.1	43.26920634	0.029421975	0.203350346
ENSRNOG00000052109	AABR07043389.1	107.5819638	-0.129197419	0.136846416
ENSRNOG00000001226	AABR07044593.1	35.13927555	-0.225440089	0.20862376
ENSRNOG00000062276	AABR07044631.2	13.50919029	-0.300504835	0.403353213
ENSRNOG00000056298	AABR07044959.1	8.665194978	0.142187152	0.432431491
ENSRNOG00000033652	AABR07045321.1	177.8620482	0.002635397	0.101580533
ENSRNOG00000016336	AABR07045373.1	15.62413853	-0.267701532	0.364637536
ENSRNOG00000043033	AABR07045431.1	62.66220066	0.122267437	0.176603849
ENSRNOG00000000320	AABR07045462.1	23.3177268	-0.098086062	0.298607714
ENSRNOG00000049150	AABR07045621.1	21.84607425	0.312345232	0.278580846
ENSRNOG00000048698	AABR07048031.1	131.3485373	-0.084906298	0.123687265
ENSRNOG00000033271	AABR07048271.1	8.735178377	-0.125688015	0.453644363
ENSRNOG00000061149	AABR07048401.1	39.48915727	0.022034641	0.226505678
ENSRNOG00000047928	AABR07048653.1	28.63447437	0.359442173	0.302157454
ENSRNOG00000047278	AABR07049043.1	19.63158188	-0.109291002	0.302788465
ENSRNOG00000033445	AABR07049060.1	21.83594527	0.403510865	0.286324068
ENSRNOG00000030107	AABR07049329.1	147.3501759	-0.060764451	0.107885878
ENSRNOG00000059243	AABR07051787.1	38.29411941	0.388408212	0.237354619
ENSRNOG00000046568	AABR07051791.1	119.3245315	-0.171569519	0.138719127
ENSRNOG00000015854	AABR07051831.1	26.82670391	-0.242842808	0.229632701
ENSRNOG00000009382	AABR07052486.1	23.51297778	0.154571889	0.286039706
ENSRNOG00000029423	AABR07052519.1	12.35954602	-0.373852897	0.344146727
ENSRNOG00000052197	AABR07052859.1	16.07799359	0.102137673	0.343416046
ENSRNOG00000004381	AABR07053065.1	14.33193079	0.041166869	0.375385804
ENSRNOG00000029262	AABR07053166.1	61.55463952	0.385184468	0.26893799
ENSRNOG00000004787	AABR07053185.1	19.19536351	-0.026027911	0.282379483
ENSRNOG00000031694	AABR07053188.1	145.5258991	-0.134594271	0.13285403
ENSRNOG00000050552	AABR07053516.1	9.629276794	0.294614206	0.453269372
ENSRNOG00000033913	AABR07053734.2	161.5335864	-0.230588109	0.123319763
ENSRNOG00000053270	AABR07053741.1	14.55864668	0.585891765	0.370434804
ENSRNOG00000053070	AABR07053749.2	130.3671977	0.084142896	0.115096457
ENSRNOG00000054840	AABR07053837.1	47.99962581	0.067509374	0.216040361
ENSRNOG00000050784	AABR07054000.1	22.36418511	0.244695378	0.302318849
ENSRNOG00000005920	AABR07054266.1	9.722980347	0.011764847	0.393010942
ENSRNOG00000031275	AABR07054361.1	48.97557531	-0.245416897	0.209771195
ENSRNOG00000029996	AABR07054368.1	30.31469484	-0.05898402	0.229988395
ENSRNOG00000008245	AABR07054614.1	24.82765454	0.326790284	0.283407184
ENSRNOG00000000958	AABR07055943.1	9.48797684	0.264230891	0.457353984
ENSRNOG00000009072	AABR07056013.1	16.04900377	0.437981291	0.348161087
ENSRNOG00000032424	AABR07056556.1	58.42140712	-0.999756237	2.502273377
ENSRNOG00000005546	AABR07057196.1	10.04945452	0.266722211	0.380518056

ENSRNOG00000032777	AABR07058519.1	186.9156364	-0.203921336	0.100751695
ENSRNOG00000029243	AABR07058578.1	60.10971995	0.117947896	0.157929107
ENSRNOG00000004457	AABR07058615.1	13.56710564	0.461031065	0.360497671
ENSRNOG00000016100	AABR07058934.1	9.815979586	1.286388576	0.535926075
ENSRNOG00000061046	AABR07059632.3	42.81286785	-0.015468592	0.20155123
ENSRNOG00000051922	AABR07059663.1	24.08453929	0.201225241	0.286455365
ENSRNOG00000032847	AABR07060145.1	9.160394369	-0.35902603	0.430354759
ENSRNOG00000053654	AABR07061614.3	23.28593709	-0.098007629	0.277500831
ENSRNOG00000011606	AABR07061825.1	102.0861251	0.022336995	0.14596065
ENSRNOG00000059964	AABR07061825.2	124.7271623	-0.079826472	0.134952819
ENSRNOG00000055966	AABR07062138.2	12.07036122	-0.25172648	0.390115665
ENSRNOG00000053414	AABR07062323.1	32.87879682	-0.114841187	0.236487704
ENSRNOG00000004199	AABR07062753.1	10.16504522	0.686913068	0.44622442
ENSRNOG00000054705	AABR07062833.3	10.54616562	0.07227837	0.419027812
ENSRNOG00000031342	AABR07063082.1	10.97626955	0.537080379	0.399018362
ENSRNOG00000057813	AABR07063281.1	8.314594251	0.21564032	0.493913552
ENSRNOG00000029594	AABR07064000.1	12.86848361	0.366822764	0.39988753
ENSRNOG00000056128	AABR07064719.2	91.55859372	0.014568536	0.140268676
ENSRNOG00000005568	AABR07064804.1	75.48583904	-0.107845863	0.182726596
ENSRNOG00000033058	AABR07064810.1	188.2662307	-0.136804411	0.119142005
ENSRNOG00000048462	AABR07066529.1	16.0236078	0.080123042	0.350626496
ENSRNOG00000047581	AABR07066700.1	50.40383638	-0.124178657	0.201759234
ENSRNOG00000061680	AABR07066800.1	39.00924405	-0.426586764	0.284839131
ENSRNOG00000058900	AABR07067076.1	34.41369223	-0.619803354	0.259497968
ENSRNOG00000011124	AABR07067267.1	24.5703639	0.062101639	0.252533643
ENSRNOG00000045975	AABR07067388.1	78.95513679	-0.163366146	0.166514914
ENSRNOG00000032289	AABR07067814.1	74.06373356	-0.399964739	0.166685773
ENSRNOG00000053030	AABR07068274.1	9.333765434	-0.470327372	0.42129469
ENSRNOG00000032765	AABR07068536.1	21.08913951	-0.311395463	0.29999431
ENSRNOG00000057499	AABR07068587.1	7.888416228	0.563786037	0.505125916
ENSRNOG00000059163	AABR07069454.1	14.99356843	0.246618248	0.329555828
ENSRNOG00000026979	AABR07069466.1	14.87152394	0.235082117	0.32733276
ENSRNOG00000015333	AABR07070225.1	21.75702895	-0.025396441	0.287633592
ENSRNOG00000022382	AABR07070238.1	17.48091968	0.27189507	0.339610771
ENSRNOG00000053753	AABR07070307.1	17.9445883	0.19043778	0.326349264
ENSRNOG00000029843	AABR07070957.1	72.44897805	-0.1216416	0.160280501
ENSRNOG00000051416	AABR07071000.1	149.0520716	0.019807342	0.120525822
ENSRNOG00000034052	AABR07071482.2	49.11579378	1.214915057	0.936550926
ENSRNOG00000047457	AABR07071765.1	91.34703549	-0.346565641	0.161339939
ENSRNOG00000042579	AABR07072078.2	8.413228334	0.555768529	0.565641348
ENSRNOG00000006943	AABR07072108.1	17.41231351	-0.001276034	0.341739423
ENSRNOG00000053569	AABR07072127.1	9.966801361	-0.365259913	0.401515201
ENSRNOG00000032877	AABR07072261.1	60.35416385	-0.003030468	0.188790111
ENSRNOG00000011861	Aadat	40.91851426	0.392517831	0.214164169
ENSRNOG00000008424	Aagab	12.89383564	0.358365882	0.381460523
ENSRNOG00000012584	Aamdc	46.5336327	0.103218565	0.214393303
ENSRNOG00000004708	Aard	20.56861277	0.248703545	0.324999967
ENSRNOG00000018404	Aars1	104.0431031	0.649313203	0.329454201
ENSRNOG00000002137	Aasdh	10.08314721	-0.147557832	0.372950598
ENSRNOG00000005795	Aasdhppt	172.8593318	0.04790573	0.127989706

ENSRNOG00000002778	Aatf	67.29463484	-0.181774153	0.167092239
ENSRNOG00000004392	Aatk	48.66637305	0.234076898	0.199496008
ENSRNOG00000033256	AB124611	13.97658191	-0.098648191	0.348178722
ENSRNOG00000002636	Abat	94.18720795	-0.001467727	0.132161395
ENSRNOG00000018126	Abca1	46.99855277	0.088998597	0.19783122
ENSRNOG00000025645	Abca13	12.5253653	0.184130371	0.370444046
ENSRNOG00000039656	Abca17	15.72329825	-0.008145384	0.342735983
ENSRNOG00000050057	Abca3	40.81502028	0.258549566	0.196713288
ENSRNOG00000012939	Abca7	124.5169666	-0.049996707	0.153903849
ENSRNOG00000004040	Abca8	14.69634281	0.152666354	0.348308015
ENSRNOG00000004147	Abca8a	26.41839825	-0.478935141	0.283679675
ENSRNOG00000017993	Abcb10	114.4033973	0.156169068	0.140282672
ENSRNOG00000008012	Abcb1b	104.9134427	0.205052898	0.148415278
ENSRNOG00000022305	Abcc1	38.28463678	0.12129443	0.20530782
ENSRNOG00000018863	Abcc10	10.07748799	0.790984825	0.442340741
ENSRNOG00000046727	Abcc2	8.593109161	0.442531003	0.458455574
ENSRNOG00000010064	Abcc4	23.30692335	0.207394996	0.285751264
ENSRNOG00000021130	Abcc8	38.1472206	0.429093268	0.221092864
ENSRNOG00000036960	Abcc9	41.33519555	0.473937155	0.248803417
ENSRNOG00000056524	Abcd1	50.62448219	-0.174204576	0.200902129
ENSRNOG00000015538	Abcd2	141.5062459	0.326628566	0.138205766
ENSRNOG00000011964	Abcd4	29.60399516	0.0175225	0.283691212
ENSRNOG00000001158	Abcg1	178.2616475	-0.054634184	0.117347544
ENSRNOG00000007041	Abcg2	41.8443699	-0.061383868	0.290146814
ENSRNOG00000008862	Abcg4	29.22090523	0.643178559	0.282619496
ENSRNOG00000025689	Abhd1	10.77887611	-0.084329979	0.441438334
ENSRNOG00000054334	Abhd10	35.22092369	-0.105539963	0.257443522
ENSRNOG00000018910	Abhd11	77.38714524	-0.092258774	0.167510645
ENSRNOG00000036934	Abhd12	15.87204646	-0.344786237	0.327228198
ENSRNOG00000014598	Abhd13	105.6045598	-0.072481365	0.145634153
ENSRNOG00000011936	Abhd14a	24.56902975	-0.164106552	0.293563088
ENSRNOG00000013692	Abhd18	19.35825334	0.028290941	0.324955583
ENSRNOG00000017120	Abhd2	46.86025673	0.570214614	0.335151742
ENSRNOG00000000221	Abhd5	52.70712723	0.072665805	0.199770856
ENSRNOG00000031325	Abi1	22.14281689	0.45589595	0.287662636
ENSRNOG00000017707	Abi2	52.36183229	0.402530202	0.229129101
ENSRNOG00000001627	Abi3bp	9.529078227	0.052708412	0.393639404
ENSRNOG00000047356	Abl1	134.9760451	-0.036194279	0.113002312
ENSRNOG00000004305	Abl2	38.27131974	-0.174018135	0.235166513
ENSRNOG00000019365	Ablim3	23.96997384	0.371569073	0.271391435
ENSRNOG00000046958	Abo	19.28859751	-0.520617201	0.287058453
ENSRNOG00000056837	Abr	63.0789052	-0.012969601	0.200634102
ENSRNOG00000007999	Abra	11.77087384	-0.243396872	0.375732392
ENSRNOG00000051450	Abracl	169.0558865	0.024389165	0.118932848
ENSRNOG00000002165	Abraxas1	8.541567803	-0.395310742	0.50556245
ENSRNOG00000017222	Abraxas2	62.9983582	0.155420253	0.16075804
ENSRNOG00000017585	Abt1	100.5561003	-0.006434428	0.132081924
ENSRNOG00000015762	Abtb1	77.64223796	-0.11043962	0.161670925
ENSRNOG00000027960	AC094055.1	9.272763995	0.599922711	0.48335113
ENSRNOG00000055222	AC094212.1	115.5719919	-0.388346078	0.113283282

ENSRNOG00000021048	AC095693.1	71.78084495	0.419057712	0.164725886
ENSRNOG00000033695	AC095825.1	120.236984	0.088814097	0.131232076
ENSRNOG00000052304	AC096201.1	47.66575565	-0.059268062	0.185259344
ENSRNOG00000033186	AC097037.2	90.06690686	-0.170494863	0.147079581
ENSRNOG00000036856	AC097183.1	12.27043827	-0.388122669	0.353820563
ENSRNOG00000056374	AC097784.1	15.8240321	0.135375438	0.338459552
ENSRNOG00000055683	AC098125.3	171.0563352	-0.111664291	0.103324292
ENSRNOG00000021123	AC098622.1	16.19617832	-0.310077204	0.303950261
ENSRNOG00000029315	AC099294.1	174.8418704	-0.076789268	0.18509212
ENSRNOG00000053437	AC103090.1	91.17145048	-0.181366798	0.148684644
ENSRNOG00000053814	AC105531.1	78.45574048	-0.101583873	0.139702878
ENSRNOG00000033015	AC105604.1	33.24678366	-0.031795469	0.259375808
ENSRNOG00000030447	AC105662.1	15.71427796	-0.579859982	0.346287843
ENSRNOG00000060851	AC105662.3	32.75599202	-0.630724496	0.729153929
ENSRNOG00000051643	AC108261.1	14.79661809	-0.519302924	0.311287604
ENSRNOG00000018801	AC109048.1	187.9234545	-0.061768452	0.099005976
ENSRNOG00000014312	AC109737.1	17.14586235	0.859495576	0.408273399
ENSRNOG00000029416	AC109942.1	22.95762233	0.495780229	0.296247805
ENSRNOG00000059820	AC110837.2	8.135786226	0.441310777	0.505144022
ENSRNOG00000026991	AC110981.1	26.87778188	-0.261128339	0.259237327
ENSRNOG00000033274	AC113910.1	25.66425631	-0.181498048	0.279718797
ENSRNOG00000056279	AC115369.1	56.45993695	-0.077624305	0.183014086
ENSRNOG00000052874	AC115378.1	39.16724175	-0.083278145	0.218753262
ENSRNOG00000055844	AC115401.1	27.01208244	-0.138970641	0.253903392
ENSRNOG00000031439	AC117869.1	60.95188292	-0.201105474	0.1812401
ENSRNOG00000032625	AC118165.1	15.83187169	-0.335726802	0.359316821
ENSRNOG00000010101	AC118490.1	41.9953908	0.042841633	0.198708527
ENSRNOG00000026579	AC119332.1	9.015982546	0.337928848	0.401680291
ENSRNOG00000054155	AC120594.1	44.31345211	0.278470073	0.232028487
ENSRNOG00000028991	AC125757.1	10.92161803	0.259419699	0.425313675
ENSRNOG00000061945	AC126482.2	61.09063073	-0.222906464	0.170233143
ENSRNOG00000051957	AC126897.1	30.97576113	-0.037934942	0.232166546
ENSRNOG00000008664	AC127920.1	113.4780262	-0.068333023	0.146940665
ENSRNOG00000028837	AC128207.1	9.178454117	-0.11496734	0.444798959
ENSRNOG00000054355	AC128212.1	24.15022091	-0.016208101	0.260579389
ENSRNOG00000054663	AC128303.1	10.91158677	0.328851354	0.454967941
ENSRNOG00000025764	AC128848.1	49.91534003	-0.365463216	0.191197239
ENSRNOG00000039470	AC131360.1	12.31656034	0.229875245	0.387104802
ENSRNOG00000058218	AC131806.3	95.47162628	0.098092553	0.14083157
ENSRNOG00000017757	AC132020.1	15.16906539	-0.254869406	0.324761334
ENSRNOG00000054780	AC132740.1	13.71448631	0.302344782	0.348437405
ENSRNOG00000031651	AC133270.1	122.2158306	0.131293477	0.130586622
ENSRNOG00000048439	AC134087.1	8.776517359	0.700649647	0.440695368
ENSRNOG00000059750	AC141102.1	25.39539982	0.067429173	0.260463123
ENSRNOG00000062115	AC242953.1	17.00728555	0.349503905	0.32147709
ENSRNOG00000032908	Acaa1a	42.3414401	0.114033075	0.222032295
ENSRNOG00000013766	Acaa2	60.04760401	0.46487697	0.227037365
ENSRNOG00000034013	Acaca	30.87233016	0.392266859	0.285877856
ENSRNOG00000000658	Acacb	10.99409904	0.730152578	0.450539589
ENSRNOG00000037815	Acad10	36.21987253	0.406757745	0.23549417

ENSRNOG00000010940	Acad11	41.40779458	0.313516138	0.20686159
ENSRNOG00000048164	Acad8	10.72928469	-0.11187988	0.395507571
ENSRNOG00000014178	Acad9	158.203331	-0.21515103	0.108486791
ENSRNOG00000012966	Acadl	132.4422349	-0.039879237	0.117041947
ENSRNOG00000009845	Acadm	117.0156361	0.038731609	0.130625024
ENSRNOG00000001177	Acads	63.98429841	-0.08388293	0.171855107
ENSRNOG00000020624	Acadsb	15.43510518	0.373555161	0.341090385
ENSRNOG00000018114	Acadvl	31.99909811	-0.280086493	0.298797326
ENSRNOG00000001730	Acap2	108.3512334	0.146631898	0.131877879
ENSRNOG00000019189	Acat2	16.62446949	-0.205267384	0.363503055
ENSRNOG00000013975	Acat2l1	99.03265438	-0.0494502	0.139554074
ENSRNOG00000003185	Acbd3	155.4552061	0.01890502	0.109281202
ENSRNOG00000003108	Acbd4	49.14995908	0.016369245	0.204278363
ENSRNOG00000017642	Acbd5	10.79450457	0.189262533	0.384300088
ENSRNOG00000009199	Accs	14.33144089	-0.141647139	0.365873506
ENSRNOG00000007637	Acer2	176.4165412	-0.409568577	0.458137646
ENSRNOG00000036866	Acer3	75.54935465	0.232250812	0.152066362
ENSRNOG00000013533	Acin1	40.20145923	-0.070133613	0.229482617
ENSRNOG00000019622	Ackr3	59.87726336	0.155656717	0.200233436
ENSRNOG00000016924	Acly	75.95587307	-0.139451376	0.618761056
ENSRNOG00000005849	Aco1	170.937762	0.051283948	0.104050299
ENSRNOG00000024128	Aco2	124.1649041	-0.200902029	0.144646189
ENSRNOG00000055221	Acot1	21.87121886	-0.214956716	0.329851224
ENSRNOG00000018415	Acot13	59.46605768	0.072183633	0.183511704
ENSRNOG00000010134	Acot2	96.70878174	0.049660135	0.161850694
ENSRNOG00000053460	Acot3	36.81492454	-0.135474841	0.240560614
ENSRNOG00000046864	Acot4	89.76989711	-0.243850664	0.191691981
ENSRNOG00000032508	Acot5	13.43600172	0.073771965	0.351397482
ENSRNOG00000029637	Acot6	17.16265078	-0.127996402	0.924541806
ENSRNOG00000008755	Acox1	35.15978836	0.218472496	0.231508175
ENSRNOG00000046261	Acp5	12.41469561	-0.390976858	0.344337651
ENSRNOG00000017494	Acp6	42.34910616	0.31680875	0.212034139
ENSRNOG00000011381	Acsbg1	53.58743023	0.0872444	0.196708432
ENSRNOG00000003330	Acsf2	163.6592046	0.030460709	0.119933107
ENSRNOG00000015077	Acsf3	68.21126844	-0.07985038	0.155136094
ENSRNOG00000016265	Acsl5	18.34553879	-0.270679515	0.306505611
ENSRNOG00000032246	Acsm3	31.2070624	0.592434005	0.282518629
ENSRNOG00000014726	Acsm4	14.61970335	-0.277710999	0.439269576
ENSRNOG00000007102	Acss1	110.8952982	0.218554633	0.123082976
ENSRNOG00000018755	Acss2	11.57928931	-0.238542925	0.383078515
ENSRNOG00000017786	Acta1	153.1782853	0.055127747	0.120001653
ENSRNOG00000029401	Actg2	28.61755903	0.145560798	0.248616991
ENSRNOG00000011553	Actl6a	55.57890611	-0.092270662	0.165881462
ENSRNOG00000001408	Actl6b	31.30173549	0.035416386	0.281362296
ENSRNOG00000016641	Actl7a	122.0831826	0.022908779	0.125165081
ENSRNOG00000017833	Actn2	93.08576533	0.004313386	0.15073651
ENSRNOG00000020433	Actn4	71.02467806	-0.004481147	0.173507745
ENSRNOG00000019725	Actr1a	33.43626959	-0.292521724	0.236062642
ENSRNOG00000031855	Actr3b	96.92099394	0.217080095	0.145004289
ENSRNOG00000023989	Actr5	46.36068012	-0.004672722	0.199986883

ENSRNOG00000007875	Actr6	71.72971398	0.309458187	0.157253618
ENSRNOG00000015280	Actr8	73.03971909	0.037272184	0.164181466
ENSRNOG00000005033	Acvr1	154.7343195	0.113303422	0.120687084
ENSRNOG00000006934	Acvr1b	18.62868617	0.458582877	0.311490465
ENSRNOG00000004828	Acvr1c	33.23596131	0.259191038	0.226080478
ENSRNOG00000005334	Acvr2a	90.32843406	0.261435511	0.162669717
ENSRNOG00000014477	Acvr2b	67.04236429	0.151388089	0.188100835
ENSRNOG00000028713	Acvrl1	52.85595987	-0.085490192	0.180195857
ENSRNOG00000017901	Acy3	48.946847	-0.255730754	0.173766434
ENSRNOG00000017246	Adad1	68.31546593	0.227778275	0.163747431
ENSRNOG00000012166	Adal	16.0650591	0.082365703	0.325787789
ENSRNOG00000054257	Adam10	31.49157644	-0.096906546	0.223163324
ENSRNOG00000002753	Adam11	12.63124765	-0.176153304	0.399317923
ENSRNOG00000020590	Adam15	11.7025827	1.833607063	0.865994456
ENSRNOG00000042980	Adam19	66.65653377	-0.262323582	0.170463917
ENSRNOG00000001347	Adam1a	21.45626015	0.400672905	0.330622217
ENSRNOG00000042478	Adam22	53.5700364	0.757806463	0.306305505
ENSRNOG00000012424	Adam23	26.43573942	-0.120117694	0.302846672
ENSRNOG00000025728	Adam32	126.1352364	-0.05398515	0.123595069
ENSRNOG00000017897	Adam8	140.8811356	-0.040290852	0.136956333
ENSRNOG00000017231	Adam9	16.0266719	0.524305057	0.371319466
ENSRNOG00000001607	Adamts1	14.24034134	-0.107720878	0.425381272
ENSRNOG00000008857	Adamts10	26.36535661	-0.10349772	0.267088352
ENSRNOG00000009892	Adamts15	13.32737055	-0.367377513	0.376422475
ENSRNOG00000037080	Adamts17	8.667647096	-0.132098018	0.440213966
ENSRNOG00000061484	Adamts2	10.65771308	0.005203155	0.379786629
ENSRNOG00000003538	Adamts4	24.42623162	0.204653261	0.290936122
ENSRNOG00000012655	Adamts6	9.020390614	-0.070418663	0.449568256
ENSRNOG00000005574	Adamts8	9.560648891	-0.226835236	0.474167458
ENSRNOG00000023257	Adamts9	18.95888721	0.26730759	0.300221454
ENSRNOG00000006956	Adamtsl1	19.57648456	-0.1698516	0.329084
ENSRNOG00000010840	Adamtsl3	11.10426805	-0.013900065	0.478884968
ENSRNOG00000049385	Adamtsl4	13.96044321	0.056968187	0.46290861
ENSRNOG00000054033	Adap1	30.82705722	0.173250363	0.236332047
ENSRNOG00000019434	Adat1	21.45553236	0.100880451	0.328290452
ENSRNOG00000012685	Adck1	24.63134341	-0.15993844	0.304939498
ENSRNOG00000030334	Adck5	10.30456316	0.618492601	0.484852594
ENSRNOG00000059479	Adcy1	26.64505797	0.450285803	0.276557355
ENSRNOG00000053410	Adcy10	11.31504783	0.185306312	0.402485288
ENSRNOG00000032150	Adcy2	41.44560528	0.366800746	0.204849316
ENSRNOG00000003999	Adcy3	8.18146772	-0.131684919	0.473873288
ENSRNOG00000054757	Adcy6	79.79409108	-0.056000103	0.150497178
ENSRNOG00000004890	Adcy8	26.42258685	-0.121791261	0.270654042
ENSRNOG00000049768	Adcy9	162.9241871	0.06040541	0.135377595
ENSRNOG00000049882	Adcyap1	164.0472789	0.027448865	0.122430323
ENSRNOG00000013039	Add1	86.39273978	-0.020033149	0.155629447
ENSRNOG00000015903	Add2	35.8659938	0.024151529	0.264796179
ENSRNOG00000054955	Adgra1	22.27757365	0.596066693	0.323673181
ENSRNOG00000004279	Adgra3	32.65339917	-0.375357331	0.246161195
ENSRNOG00000029450	Adgrb1	42.41267697	-0.037694772	0.20323612

ENSRNOG00000012045	Adgrb3	75.25134468	0.300620992	0.163735029
ENSRNOG00000046254	Adgre1	20.66393233	0.592130646	0.345236341
ENSRNOG00000011154	Adgrf5	76.21159825	0.176057175	0.174355768
ENSRNOG00000014963	Adgrg1	31.02385912	-0.109083994	0.247816367
ENSRNOG00000032472	Adgrg2	44.28671137	0.041387764	0.218885742
ENSRNOG00000030149	Adgrl3	70.7064071	0.157165036	0.196973283
ENSRNOG00000033940	Adgrl4	40.78012775	0.283498799	0.258943208
ENSRNOG00000016306	Adgrv1	54.9816692	0.188039344	0.176616205
ENSRNOG00000046357	Adh5	92.14320205	-0.13410243	0.161733567
ENSRNOG00000008950	Adi1	124.443505	-0.226157922	0.131955252
ENSRNOG00000007990	Adipor2	23.45454924	-0.160619706	0.257963334
ENSRNOG00000012325	Adk	50.6263714	0.384185552	0.286652105
ENSRNOG00000053370	Adnp2	19.55292971	-0.522611873	0.310815645
ENSRNOG00000001302	Adora2a	17.37526939	0.083978951	0.312701993
ENSRNOG00000015788	Adora3	8.764111198	1.034438219	0.5289365
ENSRNOG00000026178	Adpgk	45.11853436	0.194629605	0.20388405
ENSRNOG00000019504	Adprhl1	31.58232501	0.056463968	0.232460015
ENSRNOG00000003397	Adprm	17.39112185	-0.08027903	0.30134831
ENSRNOG00000060087	Adra1b	126.6880726	0.052270505	0.13971737
ENSRNOG00000047545	Adra2a	90.50310199	-0.248114243	0.157071374
ENSRNOG00000009299	Adra2c	123.3563123	-0.178697551	0.135443404
ENSRNOG00000017002	Adrb1	8.326026708	0.107648022	0.463442272
ENSRNOG00000019217	Adrb2	12.26409211	0.056066964	0.366074598
ENSRNOG00000055984	Adrm1	23.44914338	0.337793566	0.260681249
ENSRNOG00000046763	Adssl1	129.0953138	0.015234832	0.158170857
ENSRNOG00000014481	Adtrp	16.85996879	0.042261351	0.308977372
ENSRNOG00000008929	Aebp2	20.1123238	0.127371488	0.295162137
ENSRNOG00000018421	Aen	37.01364818	0.058957324	0.245711931
ENSRNOG00000060665	Afap1	67.22536516	0.186621232	0.188172483
ENSRNOG00000023753	Afdn	142.5761795	-0.07058614	0.141132309
ENSRNOG00000018830	Aff3	44.95648879	0.198157017	0.208918567
ENSRNOG00000006965	Aff4	76.53639176	0.039596705	0.17512169
ENSRNOG00000026994	Afg3l1	11.85383085	-0.081808865	0.374025101
ENSRNOG00000017965	Afg3l2	49.0345142	0.054497396	0.211748144
ENSRNOG00000050205	Afmid	44.17027787	0.196650456	0.221414226
ENSRNOG00000005411	Aftph	60.69140073	-0.00935354	0.181960708
ENSRNOG00000000108	Aga	88.56608414	-0.12848815	0.137005628
ENSRNOG00000025584	Agap2	152.5320372	0.134499649	0.307054605
ENSRNOG00000010397	Agbl3	21.51885203	0.109285013	0.291238282
ENSRNOG00000008612	Agbl5	39.17277606	0.066621522	0.200504263
ENSRNOG00000015619	Agfg1	60.57216684	-0.195854503	0.163356459
ENSRNOG00000001404	Agfg2	68.67341031	0.315585039	0.210290534
ENSRNOG00000029304	Aggf1	92.60388034	0.168408216	0.151959629
ENSRNOG00000011509	Agk	188.9712832	0.14439569	0.119498356
ENSRNOG00000016214	Agl	96.86005755	-0.145259602	0.141610678
ENSRNOG00000012315	Agmat	187.855415	0.116944391	0.105921084
ENSRNOG00000023116	Agmo	9.91951393	-0.38069642	0.392907632
ENSRNOG00000055915	Ago1	42.09520584	0.262000354	0.24033322
ENSRNOG00000008533	Ago2	31.41313973	-0.665496636	0.265772729
ENSRNOG00000034269	Ago3	27.22085738	-0.066332627	0.237901863

ENSRNOG00000025596	Ago4	17.51864545	-0.050226426	0.309471461
ENSRNOG00000000437	Agpat1	14.43090611	0.70896957	0.350756327
ENSRNOG00000019466	Agpat2	39.29858193	0.066279079	0.248569915
ENSRNOG00000028623	Agpat5	191.1290688	0.00051253	0.116410072
ENSRNOG00000018445	Agt	70.41375217	0.179495916	0.17610727
ENSRNOG00000008619	Agtrap	11.78250712	-0.045599588	0.397103071
ENSRNOG00000023541	Ahctf1	23.76675802	0.466313948	0.280761927
ENSRNOG00000057569	Ahnak	61.52519769	0.340960888	0.16397036
ENSRNOG00000004342	Ahr	25.14544449	0.697541331	0.273636609
ENSRNOG00000052142	Ahsa2	27.41790165	-0.228705008	0.259768794
ENSRNOG00000058805	Aida	189.2677335	0.108628806	0.110513136
ENSRNOG00000000853	Aif1	113.2415779	-0.03315923	0.1397379
ENSRNOG00000009951	Aif1l	30.99927991	0.346700935	0.286162548
ENSRNOG00000006067	Aifm1	73.39903763	0.27087741	0.19185879
ENSRNOG00000037957	Aifm3	8.35939315	0.577329295	0.488282019
ENSRNOG00000022289	Aip	14.11108879	-0.867914577	0.420160193
ENSRNOG00000050137	Ajap1	188.7347613	-0.167254163	0.163968736
ENSRNOG00000012791	Ajuba	15.19037943	-0.354904551	0.324675224
ENSRNOG00000000122	Ak2	39.20271798	0.057643461	0.255800395
ENSRNOG00000046947	Ak5	93.57630074	0.051796101	0.146483015
ENSRNOG00000026813	Ak6	11.69727746	-0.393629516	0.353891749
ENSRNOG00000002373	Akap1	123.4062985	0.117868226	0.143481894
ENSRNOG00000002899	Akap10	35.33667539	0.443373744	0.217610254
ENSRNOG00000019549	Akap12	84.07693713	-0.036841789	0.179827833
ENSRNOG00000010964	Akap13	55.4455185	0.014340384	0.162542595
ENSRNOG00000006899	Akap14	93.66515271	-0.112953418	0.143845554
ENSRNOG00000028211	Akap17a	17.81562706	0.382137579	0.742321056
ENSRNOG00000009746	Akap17b	35.81019666	0.070115718	0.262836542
ENSRNOG00000011504	Akap2	51.04110863	0.181087587	0.266836876
ENSRNOG00000006410	Akap5	23.78701894	-0.056716122	0.257837887
ENSRNOG00000004841	Akap6	71.6398157	0.190972679	0.168881619
ENSRNOG00000006559	Akap8	10.95909243	-0.120305393	0.459388063
ENSRNOG00000006355	Akap8l	14.71257174	0.498626743	0.334378438
ENSRNOG00000008288	Akirin2	124.3480965	-0.041636577	0.120252434
ENSRNOG00000009734	Akr1b10	19.25235906	0.16602063	0.284575868
ENSRNOG00000027433	Akr1b8	13.74088192	0.456502498	0.34612294
ENSRNOG00000017672	Akr1c14	13.00482753	-0.629045161	0.387467341
ENSRNOG00000021735	Akr1c15	27.1785372	0.100432531	0.24849477
ENSRNOG00000038319	Akr1c19	11.31328753	-0.253126313	0.423713435
ENSRNOG00000017165	Akr1e2	106.7905352	0.041027407	0.163454868
ENSRNOG00000017899	Akr7a3	79.87784565	0.028231691	0.143051457
ENSRNOG00000028629	Akt1	18.80187704	0.049662907	0.299240579
ENSRNOG00000018677	Akt2	33.69524648	0.410868413	0.243788674
ENSRNOG00000021497	Akt3	84.2313136	-0.025277005	0.146917745
ENSRNOG00000015206	Alad	13.38962434	0.014262471	0.37096447
ENSRNOG00000056596	Alas1	187.8490624	-0.015637651	0.118761878
ENSRNOG00000002911	Alb	60.91249807	-0.289828792	0.377078718
ENSRNOG00000020623	Aldh16a1	66.37681914	-0.116444989	0.154458433
ENSRNOG00000017619	Aldh1a1	75.69825201	0.08231135	0.168862442
ENSRNOG00000055049	Aldh1a2	63.67050348	-0.16049904	0.206952085

ENSRNOG00000011497	Aldh1b1	45.6465707	0.411771656	0.192656076
ENSRNOG00000047023	Aldh1l1	56.52217748	-0.056996578	0.174133379
ENSRNOG00000008586	Aldh1l2	12.60228496	0.248736668	0.385523659
ENSRNOG00000017512	Aldh3b1	53.47809611	-0.208764171	0.452154004
ENSRNOG00000061876	Aldh4a1	177.8363484	0.073912101	0.098954072
ENSRNOG00000014645	Aldh7a1	181.7414599	-0.195703613	0.104578799
ENSRNOG00000014907	Aldh8a1	19.23954067	0.209950776	0.282784693
ENSRNOG00000004027	Aldh9a1	43.05302064	-0.229256529	0.186170356
ENSRNOG00000002883	Alg1	13.40563661	-0.218600679	0.333327997
ENSRNOG00000012841	Alg11	24.55469017	0.189138366	0.273559435
ENSRNOG00000004591	Alg12	53.06298367	0.145191517	0.226448128
ENSRNOG00000005753	Alg13	165.9324472	0.206122422	0.111461733
ENSRNOG00000011528	Alg14	119.0098924	0.133393283	0.14023518
ENSRNOG00000001712	Alg3	83.85108501	-0.029188976	0.142951672
ENSRNOG00000058694	Alg5	82.21698783	0.137703378	0.146006754
ENSRNOG00000012292	Alg8	26.49009946	-0.043967705	0.250359073
ENSRNOG00000010877	Alg9	95.50378142	0.208694261	0.146570781
ENSRNOG00000008683	Alk	27.5346085	-0.08593373	0.250042553
ENSRNOG00000012264	Alkbh1	56.04461302	0.136857092	0.182669655
ENSRNOG00000021678	Alkbh3	121.3472256	-0.072226305	0.14284095
ENSRNOG00000028341	Alkbh5	84.03298739	-0.200710646	0.153582016
ENSRNOG00000024525	Alkbh8	15.70472267	0.041837373	0.314519821
ENSRNOG00000022343	Alms1	42.36990385	0.161373149	0.240949093
ENSRNOG00000019183	Alox15	9.379384442	-0.281588436	0.417223236
ENSRNOG00000000907	Alox5ap	24.93398324	-0.05048212	0.267630964
ENSRNOG00000007454	Aloxe3	32.33183189	-0.066058245	0.248310809
ENSRNOG00000022636	Alpk1	29.63340877	0.153694869	0.2623446
ENSRNOG00000011659	Alpk3	10.53105484	0.150286279	0.468096128
ENSRNOG00000013954	Alpl	91.46588849	-0.060657194	0.139332224
ENSRNOG00000023280	Als2	8.214529156	-0.343544059	0.444381167
ENSRNOG00000018662	Amacr	75.45392421	-0.037283746	0.156200804
ENSRNOG00000006889	Ambp	12.09604305	-0.226357413	0.402065625
ENSRNOG00000017422	Ambra1	22.65758816	0.534647241	0.278251893
ENSRNOG00000006460	Amdhd2	74.3556438	-0.206449002	0.165516128
ENSRNOG00000013623	Amer2	41.49303829	0.33804929	0.258592061
ENSRNOG00000007032	Amigo2	58.30729685	-0.067232811	0.167850705
ENSRNOG00000016250	Ammecr1l	85.1144734	-0.082796195	0.168718643
ENSRNOG00000036917	Amn1	68.02367568	-0.1650004	0.17390797
ENSRNOG00000007345	Amot	53.41922917	0.039460289	0.164563853
ENSRNOG00000008487	Amotl2	63.33800037	0.168653251	0.199759869
ENSRNOG00000024264	Amz1	64.06110434	-0.004877955	0.209325197
ENSRNOG00000016965	Anapc1	87.16558713	0.146511482	0.145075305
ENSRNOG00000018296	Anapc10	37.47475959	0.080965491	0.214722869
ENSRNOG00000019936	Anapc15	8.48036942	-0.623099541	0.420654536
ENSRNOG00000000576	Anapc16	75.86155778	-0.06349057	0.14146496
ENSRNOG00000011295	Anapc2	10.66170752	-0.502748327	0.457427406
ENSRNOG00000004130	Anapc4	142.7202855	-0.046471505	0.118336648
ENSRNOG00000005008	Angpt4	118.6591874	0.022521124	0.142610618
ENSRNOG00000004712	Angptl1	8.214354673	0.005933453	0.518102577
ENSRNOG00000007545	Angptl4	45.99489802	0.161130302	0.21009589

ENSRNOG00000020603	Angptl6	31.03759352	-0.37739075	0.233718945
ENSRNOG00000018241	Ank1	10.64722976	0.615109642	0.429677348
ENSRNOG00000011076	Ank2	187.4965852	0.069876393	0.153203229
ENSRNOG00000003991	Ankar	10.45233789	0.299860697	0.456162911
ENSRNOG00000016212	Ankfy1	161.1603841	0.064325496	0.106267983
ENSRNOG00000010960	Ankh	76.52427612	0.052753611	0.149906749
ENSRNOG00000005502	Ankib1	123.7876331	0.105344645	0.123209332
ENSRNOG00000060144	Ankle2	70.04947129	-0.042556261	0.15896807
ENSRNOG00000048567	Ankmy1	13.01546289	0.972452154	0.369951196
ENSRNOG00000016622	Ankra2	126.5299891	-0.074612228	0.15264983
ENSRNOG00000048118	Ankra2	126.9054525	-0.072120985	0.156503715
ENSRNOG00000014742	Ankrd13b	16.89266359	0.485370803	0.330950559
ENSRNOG00000011340	Ankrd13c	53.28454594	-0.002994136	0.211084714
ENSRNOG00000028168	Ankrd16	32.74473134	-0.071541888	0.224114724
ENSRNOG00000006717	Ankrd26	60.43168271	0.243050355	0.208854412
ENSRNOG00000052814	Ankrd27	27.36287322	-0.047056488	0.311956024
ENSRNOG00000019697	Ankrd28	29.02875974	0.086854429	0.260664217
ENSRNOG00000046744	Ankrd29	63.71782784	-0.048701694	0.19342018
ENSRNOG00000010888	Ankrd33b	161.7517324	-0.1822785	0.148046782
ENSRNOG00000040166	Ankrd34b	37.05803987	-0.031181094	0.226242778
ENSRNOG00000025108	Ankrd35	18.91499285	-0.269313146	0.317612377
ENSRNOG00000002935	Ankrd40	20.61679038	-0.090087569	0.275358734
ENSRNOG00000009664	Ankrd42	65.35328171	-0.315847899	0.154037254
ENSRNOG00000021384	Ankrd44	8.720370031	0.478740315	0.524530765
ENSRNOG00000009233	Ankrd49	57.22345522	0.071529489	0.17113595
ENSRNOG00000010534	Ankrd50	78.81785822	0.165919655	0.181931028
ENSRNOG00000021402	Ankrd53	104.6834694	-0.095991514	0.147946933
ENSRNOG00000010821	Ankrd54	113.1953848	-0.02271695	0.11847489
ENSRNOG00000013555	Ankrd55	21.02354643	0.132425749	0.311827571
ENSRNOG00000007110	Ankrd6	19.84763418	0.076928417	0.321383519
ENSRNOG00000000498	Anks1a	79.91105727	0.173392022	0.153666553
ENSRNOG00000024870	Anks1b	134.4794834	0.134529422	0.133087128
ENSRNOG00000046181	Anks4b	22.84728359	0.327806055	0.280541168
ENSRNOG00000023309	Anks6	28.49459117	0.110855532	0.223922483
ENSRNOG00000019052	Ankzf1	33.62600124	-0.210633289	0.221206716
ENSRNOG00000020865	Ano1	9.052975446	0.206435043	0.453595726
ENSRNOG00000000219	Ano10	87.46466993	-0.056339473	0.154344033
ENSRNOG00000006841	Ano4	71.41254721	0.164013366	0.221068703
ENSRNOG00000006995	Ano6	30.04028502	0.04452938	0.248637939
ENSRNOG00000014846	Anp32a	189.498987	-0.007186252	0.1470254
ENSRNOG00000014610	Anpep	14.02305814	0.172360887	0.426865369
ENSRNOG00000017469	Anxa1	9.924010076	0.16223361	0.420922447
ENSRNOG00000010984	Anxa11	12.6461439	-0.142248213	0.390549777
ENSRNOG00000010362	Anxa2	98.53677033	0.268444064	0.165607894
ENSRNOG00000018159	Anxa4	41.7038935	0.013665572	0.21063525
ENSRNOG00000007136	Anxa7	84.2106565	-0.293322323	0.587419794
ENSRNOG00000017505	Aopep	15.05718765	0.646824662	0.349532472
ENSRNOG00000015354	Aox1	20.85864405	0.690592471	0.299786445
ENSRNOG00000001415	Ap1s1	55.37965571	0.154021176	0.179331296
ENSRNOG00000038686	Ap1s2	8.70937795	-0.195200778	0.414322939

ENSRNOG00000049873	Ap1s3	8.124315148	-0.267342903	0.415489389
ENSRNOG00000026243	Ap2a1	67.54643544	-0.450383392	0.449824863
ENSRNOG00000019534	Ap2a2	25.69444184	0.030239663	0.245994798
ENSRNOG00000015865	Ap2s1	146.7017926	0.068010597	0.109139841
ENSRNOG00000010624	Ap3b1	57.37744169	-0.13275057	0.187386888
ENSRNOG00000018977	Ap3d1	139.123331	0.090649531	0.136590291
ENSRNOG00000018650	Ap3m2	59.5231067	0.013374102	0.185773304
ENSRNOG00000019455	Ap4b1	44.93125953	-0.092960949	0.217987087
ENSRNOG00000022938	Ap4e1	9.511319822	0.119844572	0.415590784
ENSRNOG00000005765	Ap4s1	124.4740111	-0.023627695	0.127509672
ENSRNOG00000026114	Ap5b1	89.64614337	-0.08186508	0.138246717
ENSRNOG00000014148	Ap5m1	28.65435922	-0.341171739	0.231312764
ENSRNOG00000021249	Ap5s1	32.32406596	-0.265872486	0.241661112
ENSRNOG00000008022	Apaf1	29.313528	0.07411794	0.255534308
ENSRNOG00000017803	Apbb1ip	36.25565619	-0.028980253	0.211853306
ENSRNOG00000020423	Apc	155.9985108	-0.006245581	0.183039539
ENSRNOG00000033791	Apc2	28.81502875	0.271152379	0.256345504
ENSRNOG00000043304	Apcdd1	106.2220118	0.089277684	0.125575116
ENSRNOG00000028440	Apcdd1l	99.60492511	0.049698325	0.15440612
ENSRNOG00000029572	Apeh	34.2452181	0.261112331	0.60471176
ENSRNOG00000026046	Apex2l1	28.48858972	-0.084044865	0.244001847
ENSRNOG00000009689	Api5	12.79757668	0.348595515	0.365597317
ENSRNOG00000007446	Apip	59.62623506	-0.12446081	0.160551125
ENSRNOG00000043059	Aplf	38.05860234	0.223552403	0.207071072
ENSRNOG00000009227	Aplnr	13.30319704	0.612059893	0.348099913
ENSRNOG00000015411	Apobec1	13.07462046	-0.218625839	0.372080918
ENSRNOG00000012303	Apobec2	14.65960114	0.05903665	0.33356658
ENSRNOG00000018405	Apoc4	11.26327744	-1.175952317	0.575741475
ENSRNOG00000042771	Apol3	13.88379065	0.562704938	0.450187238
ENSRNOG00000007830	Apold1	56.48265689	0.687061707	0.623135842
ENSRNOG00000004512	Apool	16.21903613	-0.057847843	0.363041459
ENSRNOG00000013574	Appl1	83.2750016	-0.174774028	0.145503834
ENSRNOG00000008174	Appl2	164.131249	0.099995538	0.120473272
ENSRNOG00000006582	Aptx	13.87914443	0.580399106	0.352991526
ENSRNOG00000011648	Aqp1	12.40943347	0.148786272	0.464114853
ENSRNOG00000013358	Aqp11	79.47447434	0.117919742	0.149543664
ENSRNOG00000061883	Aqp9	25.54720188	-0.213932172	0.261851754
ENSRNOG00000008912	Aqr	129.8647192	0.031711407	0.143678198
ENSRNOG00000005639	Ar	7.552417072	0.755252903	0.493772405
ENSRNOG00000019555	Arap1	53.25021383	0.015882199	0.194078692
ENSRNOG00000056826	Arap2	122.7368315	0.136652116	0.136942318
ENSRNOG00000060229	Arf1	50.90853541	-0.0634362	0.181880821
ENSRNOG00000004807	Arf2	101.7830412	0.0030611	0.142892533
ENSRNOG00000012623	Arf4	21.04075052	-0.215239493	0.276968245
ENSRNOG00000043150	Arfgap1	102.1228322	-0.072770794	0.147895506
ENSRNOG00000014429	Arfgap2	14.27435548	0.641975657	0.356473123
ENSRNOG00000046472	Arfgap3	129.488031	0.213829634	0.123798039
ENSRNOG00000005703	Arfgef1	77.75529498	0.045601993	0.174622702
ENSRNOG00000007485	Arfgef2	17.59044497	0.053648108	0.371521842
ENSRNOG00000011460	Arfgef3	24.56985157	-0.16492268	0.346714366

ENSRNOG00000010533	Arfip1	12.6209632	-0.039752186	0.366951416
ENSRNOG00000018440	Arfip2	60.0312499	0.123764371	0.24494896
ENSRNOG00000013992	Arfrp1	8.461234327	0.071745447	0.466612349
ENSRNOG00000013152	Arhgap10	19.45702154	-0.384926309	0.321185266
ENSRNOG00000017791	Arhgap12	91.95856398	0.136443512	0.135097124
ENSRNOG00000031168	Arhgap15	51.95036082	-0.469564471	0.228569892
ENSRNOG00000013836	Arhgap17	38.00729297	0.251723144	0.204975176
ENSRNOG00000011245	Arhgap18	21.08706382	0.08484729	0.289720545
ENSRNOG00000048166	Arhgap19	13.21949766	-0.678111594	0.488821698
ENSRNOG00000024728	Arhgap22	54.15203189	0.052193563	0.214441945
ENSRNOG00000022771	Arhgap23	63.49171662	0.574036359	0.383539982
ENSRNOG00000056944	Arhgap24	14.8982795	0.182715335	0.313463132
ENSRNOG00000009347	Arhgap25	116.2617307	0.112972644	0.146224608
ENSRNOG00000013920	Arhgap26	74.88994948	-0.376132878	0.165499796
ENSRNOG00000012563	Arhgap29	34.66446001	0.078843482	0.230260841
ENSRNOG00000003088	Arhgap31	8.496032429	0.151117083	0.484286889
ENSRNOG00000024677	Arhgap33	9.769182864	0.20699105	0.388906983
ENSRNOG00000016566	Arhgap39	77.41735907	-0.005492336	0.187994187
ENSRNOG00000058545	Arhgap4	19.54805617	0.124335123	0.323221922
ENSRNOG00000003603	Arhgap44	37.84980898	0.130850994	0.217728432
ENSRNOG00000004696	Arhgap5	114.3014597	0.020941279	0.1560615
ENSRNOG00000020130	Arhgef1	100.986815	0.049858585	0.143150552
ENSRNOG00000012561	Arhgef10	56.57864198	0.146866556	0.171639571
ENSRNOG00000050636	Arhgef10l	53.95431592	0.21932098	0.178619722
ENSRNOG00000015026	Arhgef11	17.19710521	-0.144650698	0.388290565
ENSRNOG00000004566	Arhgef15	45.13719817	-0.158982962	0.18000028
ENSRNOG00000053502	Arhgef17	26.0531219	-0.191862272	0.275125773
ENSRNOG00000028090	Arhgef18	17.58704174	0.081206462	0.310054474
ENSRNOG00000047967	Arhgef19	31.96775358	-0.046241661	0.259332655
ENSRNOG00000005034	Arhgef25	12.88598719	-0.335268127	0.352370627
ENSRNOG00000014549	Arhgef26	13.15161598	0.160650551	0.358654128
ENSRNOG00000007061	Arhgef33	28.51870317	-0.265542714	0.262973255
ENSRNOG00000025502	Arhgef37	21.78586607	0.321767874	0.288440344
ENSRNOG00000021433	Arhgef39	176.8408976	-0.202602453	0.102931407
ENSRNOG00000052354	Arhgef40	10.70915359	0.389185249	0.442598727
ENSRNOG00000017030	Arid1b	189.5988641	0.128551092	0.117734795
ENSRNOG00000004831	Arid2	27.03610373	0.009836199	0.259893771
ENSRNOG00000026435	Arid3a	25.06949441	0.149805423	0.249373338
ENSRNOG00000019677	Arid3b	39.04458563	0.47091082	0.281461392
ENSRNOG00000026239	Arid4a	142.9041441	-0.208865145	0.119628162
ENSRNOG00000016391	Arid4b	148.4419685	-0.209948779	0.124108653
ENSRNOG00000015382	Arid5a	26.64532968	-0.189134424	0.265831242
ENSRNOG00000000635	Arid5b	125.2164522	0.041769088	0.142217276
ENSRNOG00000009887	Arih1	181.952487	0.000477968	0.115096569
ENSRNOG00000031827	Arih2	86.10066334	-0.133184375	0.142277467
ENSRNOG00000014653	Arl11	9.834908172	0.373846413	0.4282067
ENSRNOG00000055454	Arl13a	73.14543462	0.178550801	0.149790789
ENSRNOG00000047194	Arl13b	37.36203422	-0.151475055	0.217087343
ENSRNOG00000004891	Arl14ep	78.00515113	-0.303197703	0.19276868
ENSRNOG00000016991	Arl2bp	165.3223	-0.05762116	0.419283934

ENSRNOG00000020770	Arl4d	72.78851642	0.316495414	0.379691843
ENSRNOG00000036880	Arl5c	8.232003046	-0.208610958	0.462076974
ENSRNOG00000017751	Arl6ip1	139.2493453	-0.03276922	0.110756496
ENSRNOG00000001080	Arl6ip4	15.60789524	0.160812439	0.401024993
ENSRNOG00000005074	Arl6ip6	20.56693008	0.157175055	0.29316539
ENSRNOG00000005988	Arl8a	25.28499502	0.285347242	0.26242484
ENSRNOG00000013253	Armc1	49.23958247	-0.06217125	0.190758333
ENSRNOG00000012785	Armc10	76.01985292	-0.021564188	0.167615216
ENSRNOG00000026217	Armc2	8.883976963	0.090680364	0.461762999
ENSRNOG00000020280	Armc6	145.3089268	0.009326141	0.117293193
ENSRNOG00000014521	Armc8	145.3851562	0.129546653	0.131882626
ENSRNOG00000046862	Armcx1	122.9590199	0.015234572	0.129656742
ENSRNOG00000043128	Armcx4	21.41563669	-0.244089342	0.260690974
ENSRNOG00000003187	Armcx5	31.53703392	-0.077023432	0.234714775
ENSRNOG00000025523	Armh3	15.24386829	0.375853778	0.325313221
ENSRNOG00000031849	Armh4	26.5418377	-0.032194059	0.244205308
ENSRNOG00000019489	Armt1	107.2376402	0.212081417	0.164716271
ENSRNOG00000031174	Arnt	37.20909937	0.385657246	0.260614449
ENSRNOG00000008994	Arpc4	108.2362545	-0.302506504	0.216942896
ENSRNOG00000014317	Arpc5l	37.71090625	-0.188238568	0.228662056
ENSRNOG00000014589	Arpin	40.77683349	-0.076582087	0.239589028
ENSRNOG00000023086	Arpp19	74.94752757	-0.234522441	0.14397756
ENSRNOG00000002904	Arr3	12.44819476	-0.402189988	0.346984063
ENSRNOG00000030404	Arrb1	31.87686193	-0.175135225	0.232892482
ENSRNOG00000019308	Arrb2	150.678128	-0.107486489	0.124823644
ENSRNOG00000007622	Arrdc1	60.01657286	0.046987838	0.172532052
ENSRNOG00000019009	Arrdc2	80.68719046	-0.279274715	0.173200583
ENSRNOG00000010775	Arrdc4	19.60409097	0.140856507	0.328182767
ENSRNOG00000028350	Arse	53.06089901	0.310540303	0.182296946
ENSRNOG00000026937	Arsk	33.68539754	-0.030151148	0.247361481
ENSRNOG00000002256	Art3	9.383039335	-0.103951566	0.43891712
ENSRNOG00000019842	Artn	161.801148	-0.106599908	0.128239862
ENSRNOG00000018909	Arv1	96.97790251	0.213745909	0.14914328
ENSRNOG00000053562	Arx	146.1992756	0.174064832	0.116222408
ENSRNOG00000020081	As3mt	136.6212403	0.038645379	0.124343975
ENSRNOG00000012196	Asah2	45.51407997	0.121987256	0.20222809
ENSRNOG00000058733	Asap1	102.6835614	0.170832531	0.187310093
ENSRNOG00000061519	Asap2	32.63065576	0.047829376	0.231993637
ENSRNOG00000020322	Asb1	16.69090057	-0.083458099	0.313068508
ENSRNOG00000003452	Asb11	15.07487497	-0.01233394	0.369772313
ENSRNOG00000004197	Asb12	8.559078289	0.038045264	0.488209121
ENSRNOG00000017967	Asb13	23.56278532	-0.154455867	0.272392691
ENSRNOG00000006365	Asb15	52.33941291	0.361771041	0.213682322
ENSRNOG00000051619	Asb2	15.10737517	-0.183113667	0.350657443
ENSRNOG00000032471	Asb3	47.41158323	0.099485309	0.23586223
ENSRNOG00000013795	Asb7	82.24558654	0.024943144	0.152359488
ENSRNOG00000010099	Asb8	81.59933949	0.008658265	0.17675325
ENSRNOG00000056647	Ascc1	105.8187552	0.039273769	0.125193347
ENSRNOG00000007349	Ascc2	12.72254448	0.776586494	0.416870481
ENSRNOG00000020434	Ascl2	8.113947617	0.614493755	0.520739724

ENSRNOG00000000415	Asf1a	18.85490875	-0.064804629	0.337421338
ENSRNOG00000014875	Ash2l	30.77209517	-0.064063828	0.284209319
ENSRNOG00000059765	Asic1	28.52473317	0.411216415	0.265102896
ENSRNOG00000058308	Asic2	14.26316859	-0.39659675	0.33688068
ENSRNOG00000008380	Asic3	27.51842883	0.10559385	0.249242614
ENSRNOG00000000903	Asl	30.01463744	0.178551758	0.35236692
ENSRNOG00000003942	Asnsd1	52.33380066	-0.072832449	0.190805176
ENSRNOG00000019659	Aspa	65.22579823	0.055061685	0.193458517
ENSRNOG00000012318	Aspm	37.21623992	-0.02298359	0.199769304
ENSRNOG00000015410	Aspn	12.96845233	0.597127794	0.369666202
ENSRNOG00000036678	Aspscr1	87.4529115	-0.000166073	0.136909609
ENSRNOG00000008837	Ass1	107.453324	-0.058625116	0.187175089
ENSRNOG00000060105	Astn2	9.935479907	0.59947787	0.454078729
ENSRNOG00000061603	Asxl1	50.08124268	0.099943292	0.232746736
ENSRNOG00000011908	Asxl2	12.47348327	0.145616302	0.363253101
ENSRNOG00000058973	Atad2b	26.7727937	-0.101581241	0.28296381
ENSRNOG00000021903	Atad5	15.16692033	0.320822245	0.347703494
ENSRNOG00000000809	Atat1	13.32209647	0.075947654	0.366189471
ENSRNOG00000024414	Ate1	177.6921636	0.09078864	0.115009257
ENSRNOG00000061088	Atf1	90.28909632	-0.066060692	0.136499471
ENSRNOG00000001597	Atf2	24.27239283	-0.424762853	0.271968744
ENSRNOG00000017801	Atf4	24.58340196	-0.260451224	0.304624642
ENSRNOG00000020060	Atf5	29.47326699	0.434411946	0.239746988
ENSRNOG00000024632	Atf6	50.75784056	-0.029968721	0.170925619
ENSRNOG00000000431	Atf6b	23.04518414	-0.003180294	0.253005512
ENSRNOG00000015269	Atf7	24.70175886	0.505724196	0.316189953
ENSRNOG00000008870	Atf7ip	42.79047604	0.186720972	0.269186261
ENSRNOG00000016342	Atg10	29.31790977	-0.186886195	0.272476417
ENSRNOG00000007756	Atg101	9.567452317	0.291920812	0.407949148
ENSRNOG00000016984	Atg13	24.71155989	0.090795467	0.271247006
ENSRNOG00000011873	Atg14	58.87091962	0.247570785	0.188461679
ENSRNOG00000019413	Atg16l2	28.04588356	0.182770093	0.246912467
ENSRNOG00000060707	Atg2a	11.27126808	-0.29903902	0.382955818
ENSRNOG00000004519	Atg2b	179.5893643	0.042458179	0.101695834
ENSRNOG00000018403	Atg4b	13.33838433	0.817744098	0.407438223
ENSRNOG00000047625	Atg4d	123.509447	0.081938963	0.125300423
ENSRNOG00000000322	Atg5	110.3549741	-0.026829222	0.128367676
ENSRNOG00000007486	Atg7	127.2103775	0.048643075	0.182100148
ENSRNOG00000015511	Atic	188.4055086	0.26025179	0.127326534
ENSRNOG00000006523	Atl2	137.2168043	0.067499296	0.121099899
ENSRNOG00000029773	Atm	16.23626807	0.201465817	0.317770472
ENSRNOG00000060594	Atn1	26.20782002	-0.223873505	0.266659522
ENSRNOG00000010716	Atoh8	23.17421081	-0.50071681	0.364296686
ENSRNOG00000056228	Atp10a	21.81759677	-0.434027495	0.265471675
ENSRNOG00000017154	Atp11a	46.05248578	0.347094368	0.216166364
ENSRNOG00000052116	Atp11b	29.90739466	0.214549045	0.253671678
ENSRNOG00000010776	Atp13a1	9.554385367	-0.383594906	0.427191772
ENSRNOG00000008052	Atp13a2	64.22563291	0.411196893	0.23192351
ENSRNOG00000001714	Atp13a4	22.28987668	-0.43450987	0.321282978
ENSRNOG00000007290	Atp1a2	114.9522433	0.178408405	0.126707454

ENSRNOG00000045629	Atp23	48.40812102	0.049337583	0.205017462
ENSRNOG00000017912	Atp2a3	12.13758948	-0.06427768	0.35123039
ENSRNOG00000013305	Atp2c1	24.67889559	-0.057122393	0.270880584
ENSRNOG00000049334	Atp2c2	41.6003225	-0.021164923	0.213265704
ENSRNOG00000020985	Atp4a	10.03457999	-0.074568875	0.465091445
ENSRNOG00000014625	Atp5f1d	73.96852245	-0.092404532	0.144757672
ENSRNOG00000000064	Atp5me	28.72076961	-0.19544121	0.256182308
ENSRNOG00000027049	Atp5mf	31.35001048	-0.333726324	0.244009605
ENSRNOG00000040201	Atp6ap1l	81.58767793	-0.037743757	0.3422605
ENSRNOG00000013428	Atp6v0a4	44.24754805	0.202992535	0.238835717
ENSRNOG00000017235	Atp6v0d1	60.38010853	-0.293258195	0.267631547
ENSRNOG00000008218	Atp6v0e2	121.4253201	-0.127149848	0.12820673
ENSRNOG00000013573	Atp6v1b1	28.3175394	0.051169186	0.239177952
ENSRNOG00000050553	Atp6v1c2	69.19108955	-0.057852445	0.153052097
ENSRNOG00000009080	Atp6v1d	183.0760422	-0.035781282	0.12111996
ENSRNOG00000011905	Atp6v1e1	88.69946716	0.16648143	0.15706059
ENSRNOG00000000840	Atp6v1g2	170.5120059	-0.209522015	0.18582287
ENSRNOG00000034200	Atp8a1	29.6391942	-0.117625717	0.290197122
ENSRNOG00000008053	Atp8a2	13.94301662	-0.379680275	0.331479498
ENSRNOG00000020822	Atp8b2	122.573519	0.193680839	0.127301694
ENSRNOG00000024975	Atp8b3	10.99701524	-0.298393917	0.428021568
ENSRNOG00000049484	Atp9a	64.62559957	-0.405817894	0.203873804
ENSRNOG00000032039	Atp9b	115.7959089	0.11924231	0.125316986
ENSRNOG00000010169	Atpaf1	183.8166093	-0.147041567	0.117334681
ENSRNOG00000003719	Atpaf2	183.4433675	-0.142791359	0.107615527
ENSRNOG00000022347	Atpsckmt	29.15890998	0.762147593	0.282487721
ENSRNOG00000010027	Atr	14.90225363	-0.108937839	0.341167185
ENSRNOG00000020670	Atrip	7.907378687	0.250946283	0.451454946
ENSRNOG00000017406	Atrnl1	19.03714467	0.193772017	0.333312791
ENSRNOG00000016998	Atxn1	20.25360161	-0.098558684	0.296649672
ENSRNOG00000001256	Atxn2	49.93951984	0.192466728	0.182786242
ENSRNOG00000018686	Atxn2l	34.82291478	-0.434680271	0.223683821
ENSRNOG00000005470	Atxn3	20.97319724	-0.229823638	0.293130573
ENSRNOG00000010415	Atxn7l1	115.2633812	0.07975847	0.149280297
ENSRNOG00000020930	Atxn7l3	10.56936177	-0.126327904	0.386321695
ENSRNOG00000022030	Aunip	10.35239143	0.528109006	0.405277265
ENSRNOG00000004479	Aurka	117.9697364	0.028950916	0.140534891
ENSRNOG00000005659	Aurkb	16.39890744	0.332818871	0.332232454
ENSRNOG00000015825	Aurkc	71.06576841	0.147047871	0.162153771
ENSRNOG00000006419	Aven	21.8394899	0.787603362	0.375569847
ENSRNOG00000055010	Axin2	77.50433936	0.180190882	0.169394379
ENSRNOG00000020716	Axl	10.27690252	0.605325318	0.444927827
ENSRNOG00000010096	Azi2	30.70204308	0.265433107	0.239470453
ENSRNOG00000051507	Azin2	55.18203689	0.431983705	0.176577789
ENSRNOG00000016855	B3galnt2	9.12725272	0.142953638	0.47010257
ENSRNOG00000007625	B3galt1	10.72308281	0.088626043	0.417043646
ENSRNOG00000003251	B3galt2	190.1745044	-0.235659795	0.155703788
ENSRNOG00000030983	B3galt5	20.05017944	0.335780645	0.315401392
ENSRNOG00000019979	B3galt6	10.83899262	-0.329959682	0.368047102
ENSRNOG00000007142	B3gat1	19.80703042	-0.043419395	0.280598092

ENSRNOG00000061346	B3glct	23.3940987	0.003872019	0.268529257
ENSRNOG00000009267	B3gnt2	130.622817	0.104115736	0.134951887
ENSRNOG00000018267	B3gnt7	35.34156102	0.072836099	0.228938966
ENSRNOG00000053895	B3gnt9	158.9383855	0.158081827	0.116042967
ENSRNOG00000004839	B4galnt1	10.44507471	0.046745394	0.385115571
ENSRNOG00000019609	B4galt2	32.65245577	0.548821672	0.297719128
ENSRNOG00000003114	B4galt4	35.92316193	0.274644123	0.202092884
ENSRNOG00000021886	B4galt7	141.156827	-0.092329615	0.113668094
ENSRNOG00000002462	B9d1	179.7436388	-0.130382572	0.15229515
ENSRNOG00000028753	B9d2	9.395626633	0.018126575	0.42402422
ENSRNOG00000017071	Babam1	21.15721463	0.20550636	0.292626933
ENSRNOG00000001953	Bace2	41.7057546	0.194724898	0.21965328
ENSRNOG00000006170	Bach2	8.892149662	-0.469647044	0.437172835
ENSRNOG00000021147	Bad	30.44834613	-0.096102817	0.226316594
ENSRNOG00000008277	Bag1	58.92645339	0.35730871	0.180406319
ENSRNOG00000012709	Bag2	91.35603867	0.066523367	0.148615087
ENSRNOG00000020298	Bag3	32.74193692	-0.004961659	0.222051957
ENSRNOG00000000851	Bag6	25.2087419	0.127196713	0.301200039
ENSRNOG00000000485	Bak1	64.77999942	0.045000515	0.210369149
ENSRNOG00000016066	Bambi	25.15283653	0.435241767	0.282789493
ENSRNOG00000019140	Banp	139.3452367	0.085003983	0.132033497
ENSRNOG00000019097	Bap1	8.482941763	0.534263524	0.513063549
ENSRNOG00000008592	Barx2	17.92585035	0.50701763	0.330965311
ENSRNOG00000020876	Bax	36.43682722	-0.108547481	0.20254693
ENSRNOG00000006828	Baz1a	10.70072003	-0.438250697	0.38931329
ENSRNOG00000001453	Baz1b	57.23740549	-0.209257763	0.174156299
ENSRNOG00000053881	Baz2a	63.45562449	-0.077972982	0.186351444
ENSRNOG00000056984	Baz2b	97.64481605	0.163997289	0.153931528
ENSRNOG00000011376	Bbof1	65.01561598	0.023285508	0.170039503
ENSRNOG00000059519	Bbox1	11.42913207	-0.184834358	0.401319738
ENSRNOG00000026913	Bbs10	190.9600403	-0.003987142	0.118002328
ENSRNOG00000026171	Bbs4	52.98902169	-0.236678268	0.198292063
ENSRNOG00000007127	Bbs5	26.59748017	0.184499629	0.261477827
ENSRNOG00000015189	Bbs9	67.2330978	-0.300853595	0.161796647
ENSRNOG00000018456	BC005537	30.92497531	0.346495057	0.243425778
ENSRNOG00000013713	BC051019	21.19429667	-0.738777388	0.45689001
ENSRNOG00000059276	BC055324	34.54564645	0.175419061	0.235323769
ENSRNOG00000029399	Bcam	105.4134071	0.285684844	0.147493455
ENSRNOG00000007884	Bcap29	97.96764835	0.061433944	0.145111428
ENSRNOG00000055756	Bcap31	55.63277479	0.087288142	0.177872399
ENSRNOG00000013737	Bcar3	86.05285103	-0.032281684	0.137629961
ENSRNOG00000020956	Bcat2	62.93729236	-0.135365512	0.170629106
ENSRNOG00000058430	Bcdin3d	63.56067147	-0.302056917	0.153830716
ENSRNOG00000020607	Bckdha	9.627956444	-0.465855005	0.43808323
ENSRNOG00000009928	Bckdhb	177.4049207	0.046088812	0.099119136
ENSRNOG00000042389	Bcl10	10.69189096	0.448422035	0.437359532
ENSRNOG00000047606	Bcl2a1	14.22925527	-0.26351818	0.330827201
ENSRNOG00000016551	Bcl2l11	21.97153896	-0.208111677	0.277760983
ENSRNOG00000012394	Bcl2l13	12.08982277	-0.056724424	0.44413821
ENSRNOG00000001843	Bcl6	66.14525981	0.036709359	0.170924709

ENSRNOG00000018916	Bcl7c	158.7186441	-0.163535522	0.118722517
ENSRNOG00000017516	Bcl9	124.3372465	0.196204174	0.126547759
ENSRNOG00000012420	Bcl9l	12.45800707	-0.220039509	0.412846009
ENSRNOG00000005135	Bclaf3	12.67186351	0.354715701	0.377419849
ENSRNOG00000034240	Bcor	54.2826773	-0.006316762	0.264757968
ENSRNOG00000005076	Bcorl1	44.90064101	0.19563017	0.203876763
ENSRNOG00000001736	Bdh1	17.48712952	0.374251275	0.359043705
ENSRNOG00000014490	Bdh2	11.08950869	0.446246303	0.402602464
ENSRNOG00000020513	Becn1	40.36661079	-0.168374385	0.219978245
ENSRNOG00000053303	Bend4	10.26043616	-0.478459538	0.494629901
ENSRNOG00000008025	Bend5	115.004509	-0.071929714	0.129287437
ENSRNOG00000022712	Bend7	9.918827722	0.347974403	0.453340961
ENSRNOG00000011008	Bet1	72.22731895	-0.154387058	0.149509359
ENSRNOG00000003151	Bfar	95.61380994	-0.039393595	0.134812023
ENSRNOG00000005707	Bfsp1	24.03884904	-0.431439003	0.25617079
ENSRNOG00000010899	Bfsp2	114.2955724	0.239376003	0.122353666
ENSRNOG00000055962	Bgn	137.2463844	0.134093198	0.170806507
ENSRNOG00000003191	Bhlhb9	114.2638898	0.084893548	0.133647109
ENSRNOG00000021745	Bhlhe22	115.1148571	-0.040738384	0.134811267
ENSRNOG00000007152	Bhlhe40	126.3311816	-0.319012648	0.203466826
ENSRNOG00000011200	Bhmt	26.31482577	-0.283554025	0.290662926
ENSRNOG00000000614	Bicc1	16.81004075	0.270900497	0.380574644
ENSRNOG00000036911	Bicd1	42.19374431	0.402413325	0.219103693
ENSRNOG00000016031	Bicd2	149.6564384	0.076208295	0.118985146
ENSRNOG00000056955	Bicdl1	132.845856	0.166719674	0.135330019
ENSRNOG00000011262	Bicra	10.93677845	-0.535512632	0.454485717
ENSRNOG00000016229	Bicral	32.67517806	0.200064492	0.225564363
ENSRNOG00000031930	Bin2	34.6212659	0.15916155	0.227314142
ENSRNOG00000018023	Bin3	8.342098842	0.649402624	0.73308156
ENSRNOG00000010602	Birc2	94.95215518	-0.061027119	0.145186789
ENSRNOG00000027191	Birc6	67.32351422	-0.097743613	0.216653364
ENSRNOG00000022894	Bivm	15.23031218	-0.162617423	0.313460213
ENSRNOG00000011213	Blm	19.17704945	-0.181806477	0.293433378
ENSRNOG00000013967	Blnk	51.82799068	-0.094858923	0.187310804
ENSRNOG00000007784	Bloc1s1	12.30315128	-0.104133345	0.466818195
ENSRNOG00000012684	Bloc1s2	24.01765376	-0.109622215	0.26269098
ENSRNOG00000017463	Bloc1s3	74.56304302	-0.057768917	0.151406263
ENSRNOG00000054224	Bloc1s4	113.5463302	0.191050859	0.14090109
ENSRNOG00000016497	Bloc1s5	104.5003732	0.032968395	0.137260833
ENSRNOG00000037160	Bloc1s6	83.46314307	0.061069457	0.168296324
ENSRNOG00000002884	Blzf1	18.20046828	0.382747101	0.312469935
ENSRNOG00000010890	Bmp1	120.4183682	0.082525529	0.127419259
ENSRNOG00000002984	Bmp15	8.24678655	0.07085515	0.453743946
ENSRNOG00000002040	Bmp2k	30.5514601	-0.149382523	0.292930693
ENSRNOG00000002381	Bmp3	25.35660564	0.170624353	0.268184457
ENSRNOG00000009694	Bmp4	25.10306877	0.41257296	0.357752596
ENSRNOG00000053384	Bmp7	17.10297445	-0.398270967	0.372366472
ENSRNOG00000030155	Bmp8a	10.09025886	0.338216358	0.47494005
ENSRNOG00000015357	Bmper	86.84877418	0.051663501	0.140533154
ENSRNOG00000052469	Bmpr1a	26.28681365	0.425441178	0.260114901

ENSRNOG00000022196	Bmpr2	63.20375024	0.284888136	0.236584509
ENSRNOG00000006576	Bms1	73.07793717	0.180666478	0.154565547
ENSRNOG00000056024	Bnip2	11.07553013	-0.186488943	0.434736654
ENSRNOG00000002041	Boc	13.44591549	0.176839812	0.346697396
ENSRNOG00000021185	Bola1	19.93400393	-0.27794432	0.284965736
ENSRNOG00000024798	Bora	35.30926428	0.608419426	0.221968217
ENSRNOG00000006513	Borcs6	156.2120818	0.045987239	0.113462929
ENSRNOG00000020076	Borcs7	126.3763088	0.188009587	0.125529478
ENSRNOG00000017577	Bphl	31.00000564	0.102974598	0.275735054
ENSRNOG00000025238	Bpifb5	8.825786921	-0.433152547	0.45111733
ENSRNOG00000010957	Braf	54.71055853	0.508057607	0.204753456
ENSRNOG00000046497	Brap	12.8480589	-0.184831622	0.420931736
ENSRNOG00000001236	Brat1	68.14542557	-0.117795248	0.174269691
ENSRNOG00000001111	Brca2	19.83682558	-0.009485513	0.285128232
ENSRNOG00000048061	Brcc3	112.1108172	-0.147163687	0.135023447
ENSRNOG00000004538	Brd1	165.9254324	-0.027564712	0.103692323
ENSRNOG00000007681	Brd3	123.6166433	0.203630271	0.121663708
ENSRNOG00000014419	Brd7	101.9679691	-0.010211224	0.164003996
ENSRNOG00000020340	Brd8	36.46629387	0.24892472	0.210051444
ENSRNOG00000015676	Brd9	20.69068617	0.597551419	0.336947367
ENSRNOG00000002073	Brdt	10.059256	0.145693373	0.447393706
ENSRNOG00000014595	Brf1	176.363986	-0.046054323	0.105277136
ENSRNOG00000012739	Brf2	58.40536818	0.023724059	0.197939425
ENSRNOG00000001009	Bri3	112.5488474	0.322850581	0.355215033
ENSRNOG00000002811	Brinp3	96.58945575	-0.095894169	0.186915425
ENSRNOG00000059997	Brip1	39.51856097	-0.091025825	0.243968088
ENSRNOG00000018021	Brix1	28.2903472	-0.013738874	0.258688458
ENSRNOG00000028641	Brpf3	147.9171793	0.070225646	0.110732185
ENSRNOG00000017673	Brsk1	59.63461808	-0.047630722	0.186529266
ENSRNOG00000020021	Brsk2	70.22119327	-0.647921441	0.407933494
ENSRNOG00000001632	Brwd1	186.2004003	-0.087325336	0.109849148
ENSRNOG00000002291	Brwd3	9.805716397	-0.267032313	0.40831546
ENSRNOG00000052393	Bscl2	20.98916679	0.275823971	0.30109235
ENSRNOG00000008338	Bsdc1	10.81405817	0.341190468	0.39691431
ENSRNOG00000030714	Bsn	87.70121088	0.671182124	0.575491112
ENSRNOG00000059900	Bst2	17.65480321	0.1130107	0.390366994
ENSRNOG00000017938	Btaf1	114.635004	0.013739214	0.148073541
ENSRNOG00000019529	Btbd1	101.5673506	0.216873529	0.196768886
ENSRNOG00000014341	Btbd10	9.889448687	-0.198398597	0.399819575
ENSRNOG00000005758	Btbd11	8.116788353	-0.313802688	0.485778796
ENSRNOG00000042163	Btbd19	90.31978724	0.034454581	0.155714573
ENSRNOG00000018788	Btbd2	11.68108823	0.021839224	0.368785989
ENSRNOG00000008088	Btbd3	15.77849245	0.263105985	0.343874323
ENSRNOG00000008598	Btbd7	111.2778035	-0.145161971	0.123799759
ENSRNOG00000023446	Btbd8	170.6185655	-0.063535176	0.118369748
ENSRNOG00000000540	Btbd9	99.55850281	-0.159795406	0.167327735
ENSRNOG00000019656	Btd	69.22109304	0.163946913	0.189585095
ENSRNOG00000008512	Btf3l4	60.21090702	0.036343142	0.179182821
ENSRNOG00000001555	Btg3	23.02536933	3.436670422	0.915216739
ENSRNOG00000018665	Bud13	16.26855174	-0.03776987	0.300880263

ENSRNOG00000013977	Bzw1	101.0799641	0.041713273	0.134840358
ENSRNOG00000039379	C17h6orf52	14.03172481	0.208460148	0.329989753
ENSRNOG00000005982	C1d	187.028747	-0.060524766	0.102604038
ENSRNOG00000007804	C1galt1	41.22022285	0.233188732	0.20972884
ENSRNOG00000012807	C1qa	17.99073799	-0.067244397	0.317340575
ENSRNOG00000006949	C1qbp	83.81893257	0.197878626	0.152291926
ENSRNOG00000002963	C1ql1	171.9854329	-0.146112093	0.116604145
ENSRNOG00000046863	C1ql2	25.47899295	-0.384532287	0.242733497
ENSRNOG00000017459	C1ql3	84.33608905	-0.176162966	0.183118345
ENSRNOG00000003259	C1qtnf1	31.18506856	-0.265885404	0.243138903
ENSRNOG00000007300	C1qtnf6	8.080212226	-0.219577768	0.48358293
ENSRNOG00000011796	C1r	31.13707936	0.191140854	0.24485515
ENSRNOG00000011971	C1s	12.6222566	0.162291606	0.36916878
ENSRNOG00000001621	C2cd2	36.25787182	0.079746272	0.221251997
ENSRNOG00000017608	C2cd3	12.01568224	-0.765467671	0.424641334
ENSRNOG00000046834	C3	15.39318667	-0.280749592	0.349095656
ENSRNOG00000013738	C330018D20Rik	53.15328138	0.034198151	0.18968419
ENSRNOG00000018899	C5	11.80732953	0.561464061	0.396753111
ENSRNOG00000028701	C8g	15.43734679	-0.024827562	0.319018073
ENSRNOG00000021017	Ca11	21.28614319	0.255115896	0.296708405
ENSRNOG00000017766	Ca12	11.37502037	0.18490603	0.419495467
ENSRNOG00000023162	Ca14	33.54467433	-0.024601018	0.220645867
ENSRNOG00000010079	Ca3	108.3362599	-2.144106523	0.793344352
ENSRNOG00000029330	Ca5b	13.48173814	-0.381166679	0.383756202
ENSRNOG00000021355	Ca6	129.2334418	0.116178271	0.138049512
ENSRNOG00000005669	Ca8	85.1501573	0.190689733	0.169978425
ENSRNOG00000017073	Ca9	13.66878019	0.008752527	0.421491561
ENSRNOG00000007299	Caap1	40.78774465	-0.229263781	0.225534673
ENSRNOG00000011603	Cab39l	66.48569631	0.121243503	0.16571139
ENSRNOG00000000634	Cabcoco1	36.66867971	0.119227658	0.234464043
ENSRNOG00000001237	Cabin1	186.4136098	-0.08721107	0.128549879
ENSRNOG00000007090	Cacna1c	60.14424858	0.327565315	0.203299203
ENSRNOG00000013147	Cacna1d	77.68518391	0.285769643	0.189807381
ENSRNOG00000060528	Cacna1g	146.7146578	0.187457597	0.137919817
ENSRNOG00000060407	Cacna1i	19.12312239	0.269270555	0.310451152
ENSRNOG00000015835	Cacna2d2	17.03449608	-0.03145494	0.300976799
ENSRNOG00000008031	Cacna2d4	167.556569	-0.045787415	0.118032272
ENSRNOG00000018378	Cacnb2	10.05211275	-0.07327082	0.418781247
ENSRNOG00000054274	Cacnb3	24.65752819	0.23438199	0.288852036
ENSRNOG00000007666	Cacnb4	161.6915266	0.17472577	0.126644317
ENSRNOG00000003288	Cacng5	13.53358381	-0.176367146	0.345799084
ENSRNOG00000056257	Cacng7	54.37359485	0.257234067	0.186702846
ENSRNOG00000057848	Cacng8	8.747066659	0.214959909	0.42712836
ENSRNOG00000009954	Cacul1	100.9252692	0.184546488	0.173871905
ENSRNOG00000002572	Cacybp	36.61474416	-0.083049638	0.21304724
ENSRNOG00000026474	Cad	18.3142681	0.40569304	0.37029461
ENSRNOG00000030840	Cadm2	23.70368312	-0.000482178	0.275186424
ENSRNOG00000024243	Cadm4	11.88168348	-0.05902774	0.404307666
ENSRNOG00000008570	Cadps	64.15437517	0.035547646	0.181751732
ENSRNOG00000024111	Cage1	11.06924574	-0.167255946	0.390208102

ENSRNOG00000016977	Calb2	125.3060001	-0.161525157	0.124694392
ENSRNOG00000011130	Calca	76.24575328	-0.189649087	0.198293296
ENSRNOG00000054695	Calcrl	46.77209973	0.065761231	0.247212889
ENSRNOG00000010233	Cald1	77.40001515	0.127811991	0.176333467
ENSRNOG00000038202	Calml4	135.3825342	-0.041164956	0.146632418
ENSRNOG00000000886	Caln1	21.14065272	-0.022378371	0.314851458
ENSRNOG00000003029	Calr	136.9136915	-0.009206352	0.334491513
ENSRNOG00000021781	Camk1	35.82311824	0.509520751	0.227939066
ENSRNOG00000017882	Camk1d	25.4033819	-0.165241063	0.26890861
ENSRNOG00000009783	Camk2g	77.6788676	0.16538434	0.174915639
ENSRNOG00000020478	Camk4	132.5243386	-0.029132931	0.131563448
ENSRNOG00000030629	Camkmt	42.76430074	0.089135869	0.192471827
ENSRNOG00000058938	Camkv	23.95708263	-0.021697816	0.271120234
ENSRNOG00000021911	Camlg	20.6818201	-0.07848509	0.287034611
ENSRNOG00000018602	Camta1	46.03842161	0.070030864	0.219716923
ENSRNOG00000004283	Camta2	84.94027803	0.558858834	0.468673603
ENSRNOG00000003343	Canx	46.77017574	0.069134174	0.184563158
ENSRNOG00000013668	Capg	8.550750889	-0.499536954	0.465184446
ENSRNOG00000045623	Capn10	48.7317848	0.019406373	0.235703248
ENSRNOG00000026025	Capn11	28.46410867	-0.130711408	0.244807733
ENSRNOG00000020389	Capn12	33.61912372	0.372924567	0.280861308
ENSRNOG00000034015	Capn2	145.1044546	-0.014593744	0.108734774
ENSRNOG00000019273	Capn7	13.09764389	0.214453408	0.381672634
ENSRNOG00000047319	Caprin2	13.76247423	1.075144295	0.403348096
ENSRNOG00000007330	Capzb	72.73114963	0.051227523	0.169004057
ENSRNOG00000002626	Car10	24.02250679	-0.165033009	0.275498563
ENSRNOG00000012371	Car7	84.96055409	0.188576964	0.141726223
ENSRNOG00000047367	Card14	13.90009598	0.277853278	0.364732858
ENSRNOG00000016560	Card19	24.39683364	0.352610243	0.246630001
ENSRNOG00000051470	Card9	47.01897156	0.177677733	0.214540091
ENSRNOG00000017491	Carf	16.68883253	0.455759583	0.342622035
ENSRNOG00000002610	Carhsp1	16.4576571	-0.486755766	0.307656312
ENSRNOG00000031129	Carm1	40.91215627	0.117019111	0.209462548
ENSRNOG00000016576	Carmil1	95.26554841	-0.128844672	0.140640893
ENSRNOG00000024452	Carmil2	23.45820219	0.085157048	0.268367878
ENSRNOG00000025518	Carmil3	51.47830791	-0.194046421	0.209111188
ENSRNOG00000012872	Carnmt1	26.30276741	0.047760358	0.26175021
ENSRNOG00000020651	Cars	174.6822443	-0.015432523	0.12658373
ENSRNOG00000014526	Cars2	72.92721304	0.287631139	0.157923107
ENSRNOG00000060946	Cask	63.47870127	0.031878383	0.179277066
ENSRNOG00000003195	Caskin1	62.2730576	0.042334713	0.186947821
ENSRNOG00000016707	Casp2	40.568672	0.042782147	0.19436205
ENSRNOG00000010475	Casp3	68.96058556	-0.357298298	0.15345797
ENSRNOG00000033697	Casp4	8.977256516	0.612533557	0.418881691
ENSRNOG00000052613	Casp6	12.06148876	0.47933949	0.381374101
ENSRNOG00000056216	Casp7	29.18306437	-0.478176076	0.221286913
ENSRNOG00000006487	Casp8ap2	19.88253892	0.047033404	0.300444004
ENSRNOG00000012944	Casp9	14.78261184	0.411369359	0.381235188
ENSRNOG00000010286	Cast	50.98552345	0.02765806	0.184546554
ENSRNOG00000006740	Castor1	111.3797302	-0.077907759	0.144013224

ENSRNOG00000013474	Casz1	16.32369815	-0.094393196	0.294378387
ENSRNOG00000037835	Catip	10.11249887	-0.028887541	0.387739892
ENSRNOG00000023064	Catsper2	11.30384774	-0.068658104	0.377532622
ENSRNOG00000011374	Catsper3	45.57700237	-0.418065708	0.711641876
ENSRNOG00000048818	Catsperd	8.28280404	-0.479585046	0.432221331
ENSRNOG00000060693	Catsperg	36.39592556	0.6310344	0.232915834
ENSRNOG00000056836	Cav1	136.302758	0.261955144	0.144504186
ENSRNOG00000057713	Cav2	48.65289748	0.078259295	0.210306767
ENSRNOG00000025895	Cavin2	53.03739227	0.025920158	0.216024896
ENSRNOG00000024349	Cbarp	19.93150501	0.083274317	0.300501592
ENSRNOG00000016352	Cbfa2t2	35.12427686	-0.032249931	0.239743953
ENSRNOG00000014723	Cbfa2t3	108.806582	0.039032356	0.161838628
ENSRNOG00000014647	Cbfb	71.87735303	-0.046371658	0.174284039
ENSRNOG00000008444	Cbl	19.64556309	-0.653876968	0.415076228
ENSRNOG00000001982	Cblb	79.85488485	-0.18545543	0.166776763
ENSRNOG00000007253	Cbll1	110.1105966	0.093030989	0.139691276
ENSRNOG00000000010	Cbln1	85.61658049	-0.237969536	0.158099358
ENSRNOG00000004372	Cbln4	28.91872133	-0.039189642	0.276960599
ENSRNOG00000049693	Cbr1	101.7257744	0.241245791	0.153823405
ENSRNOG00000047848	Cbr1	102.9999655	0.214058993	0.153844155
ENSRNOG00000049911	Cbr1	105.1360582	0.164002719	0.1547821
ENSRNOG00000001701	Cbr3	112.441595	-0.155457979	0.148002321
ENSRNOG00000024411	Cbr4	71.77885956	0.202785429	0.22899992
ENSRNOG00000029528	Cbs	29.30653411	-0.590813547	0.27414689
ENSRNOG00000015516	Cbwd1	32.73285167	0.073742234	0.238671206
ENSRNOG00000008689	Cbx1	54.12824581	-0.681704513	0.220554027
ENSRNOG00000049215	Cbx2	9.084213769	-0.346047682	0.453623018
ENSRNOG00000016875	Cbx7	34.62853151	-0.398733139	0.232649683
ENSRNOG00000048113	Cbx8	19.49042704	-0.071510455	0.307856475
ENSRNOG00000006747	Cc2d1a	28.29303675	-0.106284324	0.263245654
ENSRNOG00000008634	Cc2d1b	183.5356651	0.003470436	0.116824798
ENSRNOG00000059715	Cc2d2a	60.08122665	0.418242792	0.220768032
ENSRNOG00000018295	Ccar2	12.0725742	-0.234867492	0.45279986
ENSRNOG00000025843	Ccdc102a	23.3783031	-0.151938682	0.279531846
ENSRNOG00000003559	Ccdc112	41.86236274	-0.367319643	0.242350489
ENSRNOG00000020946	Ccdc12	64.55901051	-0.240198478	0.181571448
ENSRNOG00000027410	Ccdc125	44.48341253	0.084758897	0.207808927
ENSRNOG00000009322	Ccdc126	56.05475443	-0.271608224	0.18975718
ENSRNOG00000025643	Ccdc13	13.51429121	0.273164327	0.457605232
ENSRNOG00000008319	Ccdc130	11.68467372	-0.268842264	0.413075074
ENSRNOG00000007262	Ccdc134	11.51242483	0.250049321	0.410649567
ENSRNOG00000048712	Ccdc137	48.00268392	0.217285994	0.414272985
ENSRNOG00000028581	Ccdc138	12.26321786	0.892257833	0.46023886
ENSRNOG00000012580	Ccdc141	39.09788122	0.233329158	0.219442611
ENSRNOG00000057740	Ccdc148	13.90185143	0.252764069	0.392561381
ENSRNOG00000024454	Ccdc149	13.51099213	-0.350997097	0.356707488
ENSRNOG00000022953	Ccdc163	12.86410469	-0.289144575	0.331385651
ENSRNOG00000047521	Ccdc166	59.96285406	-0.01016994	0.183993277
ENSRNOG00000023216	Ccdc17	37.30932683	0.168310489	0.222807086
ENSRNOG00000010090	Ccdc174	64.93719334	-0.093734988	0.186845058

ENSRNOG00000024545	Ccdc18	11.30951958	-0.236775114	0.361412834
ENSRNOG00000010412	Ccdc180	17.11082881	-0.161734074	0.332555063
ENSRNOG00000002860	Ccdc181	57.2493513	-0.059482796	0.181921402
ENSRNOG00000017044	Ccdc183	18.14462085	-0.205258304	0.313932893
ENSRNOG00000021945	Ccdc184	48.34649557	-0.005695058	0.199620585
ENSRNOG00000051680	Ccdc186	40.68922773	0.012738666	0.228284698
ENSRNOG00000042711	Ccdc188	16.24451732	0.442546405	0.329612489
ENSRNOG00000057815	Ccdc191	28.22355015	-0.189882954	0.260114212
ENSRNOG00000053347	Ccdc28a	11.96977285	-0.270024022	0.372490671
ENSRNOG00000047578	Ccdc28b	65.50406775	-0.157962453	0.189558977
ENSRNOG00000008560	Ccdc30	39.63126467	0.327513671	0.226666971
ENSRNOG00000021892	Ccdc38	12.67398323	-0.35578964	0.39469951
ENSRNOG00000011440	Ccdc39	185.1136388	0.026044067	0.107737796
ENSRNOG00000004075	Ccdc42	17.36031685	0.20538294	0.342183701
ENSRNOG00000002748	Ccdc43	22.69341655	-0.252267533	0.282655149
ENSRNOG00000009006	Ccdc47	27.5520525	0.065044359	0.259517257
ENSRNOG00000001930	Ccdc50	29.58402232	-0.139069334	0.243207691
ENSRNOG00000020688	Ccdc51	69.80444571	-0.067162127	0.167802735
ENSRNOG00000047531	Ccdc57	29.38725549	0.921648341	0.265082543
ENSRNOG00000004183	Ccdc59	56.89249741	0.125023192	0.177166488
ENSRNOG00000024019	Ccdc6	8.846905863	0.535135109	0.530576036
ENSRNOG00000013767	Ccdc61	14.41337172	-0.970275627	0.798667182
ENSRNOG00000034266	Ccdc63	43.51422533	0.56754598	0.230919243
ENSRNOG00000058916	Ccdc65	100.0304839	-0.344597105	0.144074523
ENSRNOG00000028380	Ccdc66	95.40933322	0.039697826	0.157234114
ENSRNOG00000048718	Ccdc71	46.35402863	-0.233477532	0.207487845
ENSRNOG00000037206	Ccdc77	29.98480614	-0.106925118	0.524209389
ENSRNOG00000027936	Ccdc8	13.96503864	0.151398604	0.366992764
ENSRNOG00000002052	Ccdc80	33.63569426	0.055702109	0.264175382
ENSRNOG00000033733	Ccdc81	12.48772947	-0.575518813	0.336525054
ENSRNOG00000003374	Ccdc85a	8.254821013	0.084391628	0.514204557
ENSRNOG00000004057	Ccdc88a	179.1291285	0.006460711	0.156438842
ENSRNOG00000004482	Ccdc88c	33.93367253	0.323558318	0.267550472
ENSRNOG00000048848	Ccdc9	15.00112292	0.066453541	0.352671481
ENSRNOG00000009462	Ccdc90b	16.71124813	-0.073736146	0.335807098
ENSRNOG00000001847	Ccdc91	100.847473	0.114688919	0.139783231
ENSRNOG00000002514	Ccdc93	28.03209788	-0.065362288	0.23913737
ENSRNOG00000039530	Ccl27	12.80597516	0.866187668	0.930363537
ENSRNOG00000060825	Ccm2	18.86032282	0.055299074	0.286227401
ENSRNOG00000009191	Ccm2l	26.87540107	0.131160458	0.348926309
ENSRNOG00000014350	Ccn1	25.02554786	0.982460504	0.877022232
ENSRNOG00000015036	Ccn2	100.8226103	-0.230265932	0.151543544
ENSRNOG00000010666	Ccn5	12.34036028	0.413274784	0.436630325
ENSRNOG00000014052	Ccna1	16.33240796	0.041567813	0.31535467
ENSRNOG00000007719	Ccnc	160.685645	-0.144975015	0.115651011
ENSRNOG00000020918	Ccnd1	138.6867991	0.195005005	0.155239797
ENSRNOG00000050258	Ccnd3	46.84782297	0.035094038	0.212573269
ENSRNOG00000011379	Ccndbp1	176.6638131	0.151951475	0.103481607
ENSRNOG00000014786	Ccne1	88.15650699	-0.076058408	0.155320786
ENSRNOG00000008055	Ccne2	16.21337171	0.438267919	0.33098584

ENSRNOG00000002089	Ccng2	172.1315356	0.025321295	0.118604217
ENSRNOG00000003893	Ccnjl	13.03350502	-0.04299483	0.356215998
ENSRNOG00000011586	Ccnl1	106.5949705	0.252014767	0.175604027
ENSRNOG00000053054	Ccnt1	30.81201331	0.299139132	0.242014802
ENSRNOG00000017113	Ccnt2	89.74026754	0.28932972	0.14570662
ENSRNOG00000018123	Ccny	142.8205689	-0.006762044	0.118205519
ENSRNOG00000023807	Ccnyl1	81.98422679	0.066193008	0.171457846
ENSRNOG00000027405	Ccp110	15.24978297	0.417365301	0.336633635
ENSRNOG00000053428	Ccpg1	25.48391288	-0.199081571	0.27379131
ENSRNOG00000049115	Ccr5	21.369688	0.670077601	0.292605822
ENSRNOG00000047816	Ccs	16.61806269	0.07396905	0.33368121
ENSRNOG00000017690	Ccsap	26.07192537	0.064902324	0.246628939
ENSRNOG00000022781	Ccser2	113.2273342	-0.235759896	0.147952962
ENSRNOG00000019090	Cct3	18.18863725	-0.282984063	0.277460877
ENSRNOG00000011632	Cct5	33.66721527	0.383544953	0.597949264
ENSRNOG00000007523	Cct6b	21.28644456	-0.090308332	0.292291573
ENSRNOG00000001592	Cct8	13.97655863	-0.078487831	0.379153647
ENSRNOG00000001032	Ccz1b	29.36404688	0.165092921	0.245496334
ENSRNOG00000010253	Cd163	10.06488833	-0.452881692	0.385136725
ENSRNOG00000009505	Cd164l2	17.45547892	-0.428069676	0.340501142
ENSRNOG00000010266	Cd180	13.55878997	0.096480198	0.328276179
ENSRNOG00000039048	Cd200r1	10.06112917	0.248381625	0.404560349
ENSRNOG00000003298	Cd247	19.13852176	0.03490827	0.311929804
ENSRNOG00000020197	Cd248	86.82377992	-0.003288195	0.165372468
ENSRNOG00000033608	Cd276	76.7993284	0.104766811	0.144835305
ENSRNOG00000006094	Cd44	91.87321479	0.004124359	0.144120893
ENSRNOG00000004737	Cd48	101.9427565	0.08661099	0.143194847
ENSRNOG00000017874	Cd53	41.13658181	0.281964645	0.198119472
ENSRNOG00000003927	Cd55	14.41171121	0.112917638	0.402823516
ENSRNOG00000034230	Cd5l	7.956771182	0.105916583	0.478894104
ENSRNOG00000020884	Cd6	10.03449382	0.013512235	0.373731667
ENSRNOG00000007650	Cd63	11.04427188	0.334713418	0.395808989
ENSRNOG00000018735	Cd74	66.9016111	-0.1236597	0.166978323
ENSRNOG00000020125	Cd79a	25.80800268	2.49792948	2.6532445
ENSRNOG00000020451	Cd81	27.70041428	-0.562506679	0.266685155
ENSRNOG00000000047	Cd82	56.43846891	-0.128600478	0.2041451
ENSRNOG00000018092	Cd83	160.703487	0.050809438	0.105561793
ENSRNOG00000007129	Cd8b	15.60981903	-0.168715122	0.695160084
ENSRNOG00000024799	Cd93	18.17630315	0.930353268	0.400591226
ENSRNOG00000012209	Cdadc1	69.78803136	0.003420396	0.157209763
ENSRNOG00000047427	Cdan1	170.9805866	0.251378635	0.117693403
ENSRNOG00000017770	Cdc123	126.3698737	-0.193618826	0.114586728
ENSRNOG00000014515	Cdc14a	21.14509396	0.370444668	0.294789889
ENSRNOG00000017536	Cdc16	42.30669715	0.287615932	0.225652583
ENSRNOG00000020737	Cdc25a	38.0821636	0.067986862	0.214305818
ENSRNOG00000021248	Cdc25b	39.27251388	0.103760245	0.219852417
ENSRNOG00000024008	Cdc25c	11.95661302	0.763649474	0.45056949
ENSRNOG00000005904	Cdc27	45.97980031	-0.066209407	0.227264239
ENSRNOG00000060530	Cdc34	24.01293452	1.875778712	0.815946578
ENSRNOG00000000581	Cdc40	112.6273427	0.122201858	0.152666846

ENSRNOG00000002841	Cdc42bpa	90.99949442	-0.176011975	0.136252778
ENSRNOG00000027456	Cdc42bpg	16.50083577	-0.044516973	0.313222074
ENSRNOG00000008517	Cdc42ep1	10.08029105	-0.167143877	0.407766056
ENSRNOG00000020904	Cdc42ep2	12.18855949	0.168724936	1.240225255
ENSRNOG00000032136	Cdc42ep3	173.6131432	-0.010477898	0.124677527
ENSRNOG00000019975	Cdc5l	188.7814449	-0.101590704	0.105894692
ENSRNOG00000002105	Cdc7	27.84608778	0.292716064	0.283128368
ENSRNOG00000003258	Cdc73	57.61549719	0.026687009	0.187989733
ENSRNOG00000031431	Cdca8	48.00087723	0.193197649	0.265034949
ENSRNOG00000009771	Cdh10	128.915837	0.179155076	0.140221678
ENSRNOG00000026392	Cdh12	26.9401967	-0.183489579	0.243203729
ENSRNOG00000027954	Cdh15	29.57166793	-0.048832999	0.236579413
ENSRNOG00000014556	Cdh20	12.08383093	-0.339226271	0.345643957
ENSRNOG00000018557	Cdh22	13.55447587	0.55484832	0.390720594
ENSRNOG00000033087	Cdh23	31.94376529	0.596431269	0.253730557
ENSRNOG00000052405	Cdh4	32.39066019	-0.369544478	0.250839459
ENSRNOG00000013324	Cdh5	57.81854434	0.258813329	0.167511027
ENSRNOG00000032490	Cdh7	23.48554808	0.263706728	0.310645466
ENSRNOG00000056643	Cdh8	91.68559624	0.042380715	0.137644526
ENSRNOG00000033837	Cdh9	51.93713575	-0.095437898	0.2329491
ENSRNOG00000004571	Cdin1	15.86850659	-0.093586595	0.317774673
ENSRNOG00000003328	Cdip1	75.97388005	-0.031016746	0.244745819
ENSRNOG00000016088	Cdk10	32.2580466	0.307739812	0.220259289
ENSRNOG00000017213	Cdk11b	178.4984211	0.193078534	0.252550309
ENSRNOG00000006000	Cdk12	50.82084329	-0.158815295	0.201853207
ENSRNOG00000008578	Cdk16	119.2036573	0.220693347	0.134316702
ENSRNOG00000008137	Cdk18	167.0397786	0.135340532	0.132466062
ENSRNOG00000017991	Cdk20	62.23744036	-0.202986848	0.167590369
ENSRNOG00000037793	Cdk5r2	84.03457939	-0.112542174	0.186946313
ENSRNOG00000015696	Cdk5rap1	72.5779656	0.092428412	0.163377568
ENSRNOG00000005788	Cdk5rap2	33.93303993	0.145016279	0.266494877
ENSRNOG00000048747	Cdk5rap3	167.1206706	-6.53922E-05	0.116591482
ENSRNOG00000018510	Cdk7	42.59663685	-0.154725816	0.219961927
ENSRNOG00000038720	Cdkl1	9.159212537	-0.053235093	0.442123174
ENSRNOG00000003742	Cdkl5	21.09319704	0.031328611	0.276725581
ENSRNOG00000000521	Cdkn1a	55.89672312	-0.215260494	0.219728302
ENSRNOG00000007249	Cdkn1b	93.54784739	0.054263732	0.138224045
ENSRNOG00000059500	Cdkn1c	58.02350734	-0.272068211	0.18865337
ENSRNOG00000032846	Cdpf1	62.56441706	-0.032204293	0.158732325
ENSRNOG00000017260	Cdr2	32.18861008	0.24616066	0.233208844
ENSRNOG00000025815	Cdr2l	82.23650852	-0.018085173	0.134481437
ENSRNOG00000002142	Cds1	49.9819249	-0.030601357	0.189632369
ENSRNOG00000021265	Cds2	46.84655953	-0.312997653	0.212430315
ENSRNOG00000010186	Cdv3	76.70930473	0.129760424	0.166080118
ENSRNOG00000042888	Cdyl2	12.59887532	0.282720895	0.413208776
ENSRNOG00000020578	Ceacam1	8.187696343	0.415197501	0.457106397
ENSRNOG00000057347	Cebpb	95.26489047	-0.109468119	0.136923436
ENSRNOG00000005087	Cebpz	138.5664533	0.01017567	0.125315446
ENSRNOG00000013628	Cela2a	74.15065523	0.146474919	0.160953067
ENSRNOG00000021619	Cela3b	8.925806852	-0.466964797	0.393983085

ENSRNOG00000023661	Celf2	101.2073255	0.398949533	0.151053788
ENSRNOG00000020875	Celf3	116.607434	-0.020320475	0.153999898
ENSRNOG00000014503	Celf4	45.54042202	0.133171004	0.220463668
ENSRNOG00000052224	Celf6	39.53064017	-0.104500835	0.215381972
ENSRNOG00000021285	Celsr1	17.5803477	0.100878601	0.311705071
ENSRNOG00000020058	Celsr2	37.43848772	0.273598193	0.236950785
ENSRNOG00000012442	Cemip	47.6246371	0.11664809	0.244578631
ENSRNOG00000012782	Cemip2	45.34903432	0.154318963	0.189580888
ENSRNOG00000012137	Cenatac	127.8678516	0.09803699	0.126520201
ENSRNOG00000021776	Cenpc	52.23973757	0.098975775	0.187961102
ENSRNOG00000018615	Cenph	16.72495678	0.093590066	0.322661198
ENSRNOG00000038789	Cenpl	17.96178248	0.023658247	0.320170455
ENSRNOG00000023262	Cenpm	16.23151122	0.120806661	0.342056179
ENSRNOG00000011296	Cenpn	26.89590805	0.009624425	0.285602146
ENSRNOG00000003086	Cenpv	167.3199616	-0.196030742	0.119451692
ENSRNOG00000025000	Cep104	58.71592805	0.164908003	0.1770572
ENSRNOG00000024557	Cep112	10.63718183	0.268335644	0.429743742
ENSRNOG00000017035	Cep120	96.08492407	0.033335309	0.144372693
ENSRNOG00000021839	Cep126	25.48962983	-0.042038996	0.264796458
ENSRNOG00000003908	Cep128	11.65919334	0.519320531	0.436290523
ENSRNOG00000002153	Cep135	13.27834903	-0.073564305	0.345387963
ENSRNOG00000010608	Cep162	17.5276666	0.201705207	0.300344378
ENSRNOG00000029826	Cep164	25.18720539	0.17501485	0.268542017
ENSRNOG00000004127	Cep170	88.1460825	-0.267701482	0.139532983
ENSRNOG00000053230	Cep20	179.6012715	-0.344244011	0.271092813
ENSRNOG00000019340	Cep250	11.36432581	0.027118226	0.383864256
ENSRNOG00000056458	Cep290	48.40923698	-0.06761374	0.191827879
ENSRNOG00000010999	Cep295	78.30260863	0.118940199	0.185390754
ENSRNOG00000003882	Cep350	27.81984553	-0.06469036	0.308305418
ENSRNOG00000010950	Cep41	52.22567729	0.021189121	0.189061142
ENSRNOG00000055093	Cep43	40.64043902	0.226480359	0.253290429
ENSRNOG00000010566	Cep44	28.33588522	-0.1418036	0.2283891
ENSRNOG00000000303	Cep57l1	36.38150764	-0.37809998	0.227132361
ENSRNOG00000008410	Cep63	37.78449777	0.597231239	0.212128155
ENSRNOG00000022845	Cep70	27.23524806	0.139855907	0.278282479
ENSRNOG00000021918	Cep76	21.72181798	0.49997017	0.300242262
ENSRNOG00000014041	Cep78	30.66948081	0.218164911	0.225159511
ENSRNOG00000016249	Cep85	9.642914752	-0.219166006	0.408348199
ENSRNOG00000000414	Cep85l	12.63594091	0.152852077	0.374404081
ENSRNOG00000012140	Cep89	65.40517737	0.179768706	0.174794954
ENSRNOG00000014354	Cep95	19.63627311	0.023584311	0.277446246
ENSRNOG00000001609	Cep97	20.26831904	0.166996598	0.27229035
ENSRNOG00000026604	Cercam	14.46354634	-0.067086417	0.36358058
ENSRNOG00000017022	Cerk	14.05542413	-0.463814294	0.322458071
ENSRNOG00000001072	Cers4	10.7587979	-0.343872331	0.374053215
ENSRNOG00000024595	Cers6	11.16599153	0.889627781	0.412173123
ENSRNOG00000050877	Cetn3	126.2846865	0.10451066	0.11988496
ENSRNOG00000017959	Cfap100	56.0651614	0.007654152	0.180357902
ENSRNOG00000034221	Cfap126	20.43083473	0.587339867	0.323817624
ENSRNOG00000039315	Cfap157	73.65992208	-0.176834557	0.159577986

ENSRNOG00000007206	Cfap20dc	149.1678856	0.144606644	0.146722982
ENSRNOG00000021399	Cfap298	11.28897934	-0.020597374	0.410030713
ENSRNOG00000043410	Cfap300	14.50381636	0.826116458	0.441241352
ENSRNOG00000003901	Cfap36	41.53646511	0.072453636	0.232762474
ENSRNOG00000001215	Cfap410	17.79056471	0.092343356	0.373268623
ENSRNOG00000028077	Cfap44	61.99372394	0.195930143	0.204534333
ENSRNOG00000008492	Cfap45	10.22671143	-0.186898003	0.37835922
ENSRNOG00000037718	Cfap52	59.45902345	0.025231643	0.398127669
ENSRNOG00000014585	Cfap53	10.45524958	-0.062523391	0.47638987
ENSRNOG00000007037	Cfap57	8.466166147	-0.036354242	0.475543022
ENSRNOG00000011028	Cfap61	26.06764144	0.126398028	0.240332831
ENSRNOG00000006114	Cfap69	44.17228427	0.281931761	0.20424031
ENSRNOG00000007046	Cfap70	169.757557	-0.108509814	0.111001274
ENSRNOG00000056407	Cfap73	44.28077466	0.010057905	0.234486642
ENSRNOG00000023627	Cfap74	30.46417152	0.420971497	0.248608327
ENSRNOG00000028805	Cfap77	21.93141861	-0.02988585	0.298713479
ENSRNOG00000002976	Cfap91	29.85180797	0.247380782	0.33616058
ENSRNOG00000032192	Cfap97	69.60195312	0.068393974	0.204827808
ENSRNOG00000058434	Cfap99	26.56882874	0.198431218	0.321383867
ENSRNOG00000030715	Cfh	90.45326029	0.083081258	0.13425108
ENSRNOG00000012473	Cflar	168.6527329	-0.174481768	0.121026285
ENSRNOG00000025811	Cfp	43.61103099	0.381090874	0.189765441
ENSRNOG00000000718	Cggbp1	162.9185584	0.021308617	0.116275505
ENSRNOG00000054080	Cgnl1	30.88378146	0.142902692	0.280617394
ENSRNOG00000010178	Cgrrf1	138.5812274	-0.091592747	0.133317343
ENSRNOG00000019141	Ch25h	26.8211376	0.064725591	0.262882651
ENSRNOG00000014387	Chac1	110.9876614	-0.272756666	0.133373854
ENSRNOG00000024631	Chadl	9.418572998	0.033191608	0.485228938
ENSRNOG00000000112	Champ1	112.9797718	-0.070798727	0.138651027
ENSRNOG00000009297	Chchd1	11.9749423	0.097871385	0.593718829
ENSRNOG00000028356	Chchd10	116.4146301	-0.060118528	0.811015224
ENSRNOG00000022466	Chchd4	137.1504064	-0.09145686	0.124841942
ENSRNOG00000018491	Chchd5	102.0216805	0.10339116	0.172630942
ENSRNOG00000031769	Chchd7	68.53636935	-0.340887791	0.164197717
ENSRNOG00000014434	Chd1	24.42790283	0.142923513	0.26848337
ENSRNOG00000012716	Chd2	147.6383704	0.283891395	0.134278095
ENSRNOG00000018309	Chd4	57.45268451	-0.389197698	0.20830568
ENSRNOG00000016744	Chd6	139.2317407	0.24383649	0.113989618
ENSRNOG00000006689	Chd7	54.16118665	0.069006516	0.184565096
ENSRNOG00000049302	Chd9	39.26608983	0.420309591	0.243764725
ENSRNOG00000015859	Chdh	76.10860066	0.42397406	0.171251192
ENSRNOG00000012323	Cherp	186.0837298	-0.129161824	0.095961508
ENSRNOG00000037430	Chfr	43.84467317	0.127278161	0.221790775
ENSRNOG00000053272	Chi3l1	10.80327543	-0.286505239	0.396687116
ENSRNOG00000002267	Chic2	87.42780207	-0.047284072	0.140581885
ENSRNOG00000028072	Chit1	11.93543573	-0.369425257	0.360913983
ENSRNOG00000016791	Chka	33.27268349	0.594605324	0.245553571
ENSRNOG00000016001	Chmp1a	37.58150255	0.059578661	0.207143334
ENSRNOG00000040257	Chmp2b	8.170671592	-0.430758188	0.46652364
ENSRNOG00000007356	Chmp3	155.8199524	-0.111000679	0.109371699

ENSRNOG00000046585	Chmp4b	33.73303517	-0.115559275	0.261790289
ENSRNOG00000034071	Chmp4bl1	130.2139374	-0.070753115	0.48425964
ENSRNOG00000009411	Chn2	135.7968803	0.249954295	0.13544233
ENSRNOG00000026643	Chordc1	167.103337	-0.149180378	0.125924573
ENSRNOG00000058271	Chpt1	15.54801725	0.04059173	0.32507267
ENSRNOG00000009103	Chrac1	184.8395166	0.173554388	0.096085254
ENSRNOG00000004330	Chrdl1	81.07985432	0.005784239	0.138482542
ENSRNOG00000018385	Chrm1	23.75869127	-0.333082668	0.290667247
ENSRNOG00000049410	Chrm3	55.34870855	0.045155884	0.203265731
ENSRNOG00000017424	Chrna2	8.913711448	0.493060058	0.439003235
ENSRNOG00000013829	Chrna3	44.81851323	-0.036348897	0.244199036
ENSRNOG00000001252	Chst12	67.5666361	0.266558614	0.255533026
ENSRNOG00000045997	Chst14	12.37321051	-0.466242559	0.367881308
ENSRNOG00000016267	Chst15	9.848238106	-0.16356949	0.423028292
ENSRNOG00000047734	Chst2	90.46060772	-0.175686413	0.185294309
ENSRNOG00000000572	Chst3	14.89051457	0.079217914	0.401877845
ENSRNOG00000021521	Chst5	8.183726482	0.333929487	0.456945465
ENSRNOG00000012698	Chsy1	72.90260961	0.031963203	0.149845807
ENSRNOG00000019174	Chtf18	10.0589222	-0.136985071	0.422155521
ENSRNOG00000047246	Chtf8	9.202656151	1.271661978	0.559949189
ENSRNOG00000058457	Chtopl1	27.07444686	0.141936595	0.248046534
ENSRNOG00000022485	Chuk	21.16361167	-0.295016689	0.311332876
ENSRNOG00000007660	Churc1	94.20240303	0.010790832	0.192079489
ENSRNOG00000012638	Ciao1	79.37767354	0.176158185	0.165061868
ENSRNOG00000017119	Ciao2a	105.8342583	-0.199469449	0.157511575
ENSRNOG00000019522	Ciao3	14.19310571	-0.274521003	0.363351736
ENSRNOG00000016234	Ciapin1	121.5634567	-0.096251966	0.1193523
ENSRNOG00000042717	Ciart	113.329658	-0.227013276	0.158554416
ENSRNOG00000016338	Cibar1	160.4947749	0.121145532	0.104078692
ENSRNOG00000056118	Cic	13.39230406	0.534704141	0.364550579
ENSRNOG00000008691	Cilk1	68.64107361	-0.044693602	0.164030092
ENSRNOG00000008035	Cinp	69.64819305	0.030486644	0.170194864
ENSRNOG00000018719	Cir1	142.4836766	-0.096525777	0.118297526
ENSRNOG00000036894	Cisd3	143.3484896	0.002713948	0.123621492
ENSRNOG00000029543	Cish	17.12061735	0.051565266	0.37636566
ENSRNOG00000001143	Cit	170.5486044	0.358685598	0.126300944
ENSRNOG00000003189	Cited1	17.37703857	-0.326596162	0.352321045
ENSRNOG00000016067	Ckap5	47.30948689	0.448411486	0.216779846
ENSRNOG00000012752	Cklf	11.39629806	-0.061438996	0.377967549
ENSRNOG00000014573	Ckmt1	22.94585054	-0.321802026	0.269631204
ENSRNOG00000042561	Cks1b	30.69488493	-0.455931768	0.236458206
ENSRNOG00000002376	Clasp1	188.3029158	0.082574421	0.100673543
ENSRNOG00000046000	Clasrp	25.55644875	0.135876331	0.26667006
ENSRNOG00000020360	Clcc1	29.53704221	-0.046795104	0.32001561
ENSRNOG00000001742	Clcn2	24.27449353	0.421655455	0.261628656
ENSRNOG00000003533	Clcn4	127.2662875	-0.025481345	0.137175738
ENSRNOG00000008345	Clcn6	25.23727649	-0.115829313	0.260674327
ENSRNOG00000016976	Clcn7	20.43579142	-0.635226256	0.382752308
ENSRNOG00000010085	Cldn10	68.53169452	0.146762499	0.163719465
ENSRNOG00000039862	Cldn12	104.1508452	0.125509341	0.153432609

ENSRNOG00000001476	Cldn4	8.442518282	-0.862082107	0.452395625
ENSRNOG00000018715	Clec10a	8.329184795	0.030182349	0.535771441
ENSRNOG00000019138	Clec11a	129.1860082	-0.013809817	0.116926172
ENSRNOG00000022697	Clec14a	26.24025193	0.236940996	0.290186978
ENSRNOG00000057378	Clec16a	17.61849803	0.368073614	0.317689422
ENSRNOG00000059538	Clec2g	156.7994272	0.159101207	0.157594022
ENSRNOG00000004540	Clec3b	20.4645733	0.484633622	0.382918851
ENSRNOG00000003755	Clgn	12.74209306	-0.405247827	0.380854868
ENSRNOG00000000728	Clic2	9.460264478	0.307540106	0.449553206
ENSRNOG00000047218	Clic5	44.42697886	0.254083184	0.22224519
ENSRNOG00000005406	Clint1	190.9230592	-0.223553234	0.092917695
ENSRNOG00000008806	Clip4	20.17419362	-0.257642161	0.306577719
ENSRNOG00000020500	Clk2	143.3488177	0.126776897	0.114913527
ENSRNOG00000030126	Clk3	108.7668755	0.119861455	0.135959904
ENSRNOG00000003714	Clk4	26.11781116	0.142733218	0.25631529
ENSRNOG00000011044	Clmn	39.35424734	-0.230428845	0.307406973
ENSRNOG00000053239	Clmp	54.91736641	0.0723022	0.182082631
ENSRNOG00000019103	Cln3	15.14156948	0.282088881	0.333791517
ENSRNOG00000007164	Cln6	117.2221039	0.105114605	0.133965357
ENSRNOG00000012565	Cln8	64.29263251	0.116693412	0.183557879
ENSRNOG00000051169	Clnk	16.60651098	-0.073576761	0.353814484
ENSRNOG00000002175	Clock	16.28988965	-0.707540667	0.341779571
ENSRNOG00000007416	Clp1	29.26295333	-0.138331816	0.24825729
ENSRNOG00000019693	Clpb	123.4813881	0.057080967	0.135761998
ENSRNOG00000047052	Clpp	8.940845557	0.806175835	0.442127623
ENSRNOG00000018255	Clptm1	74.67614919	-0.112393425	0.165853797
ENSRNOG00000030225	Clpx	122.4231533	-0.040960724	0.13377214
ENSRNOG00000016398	Clstn1	162.8529799	0.126155759	0.552806286
ENSRNOG00000043085	Clstn2	42.12083082	0.116106033	0.256671381
ENSRNOG00000017506	Cltb	173.3565525	-0.020120127	0.118595334
ENSRNOG00000004291	Cltc	73.21699046	-0.115179808	0.170198248
ENSRNOG00000007117	Cluap1	109.2400875	-0.047815602	0.132091166
ENSRNOG00000002669	Cluh	16.41948471	0.36687808	0.324086786
ENSRNOG00000012122	Clvs2	44.00022434	-0.19800832	0.184617101
ENSRNOG00000014075	Clybl	30.2484306	0.088689309	0.281211442
ENSRNOG00000011260	Cmbl	76.29806225	-0.078818545	0.167289694
ENSRNOG00000007690	Cmpk2	130.8892985	-0.040220194	0.122298713
ENSRNOG00000027888	Cmss1	86.81104846	-0.045901971	0.188142408
ENSRNOG00000016828	Cmtm5	15.98559199	0.01902189	0.33559801
ENSRNOG00000010951	Cmtm6	35.71949823	-0.098647927	0.201639843
ENSRNOG00000011201	Cmtm8	21.69859688	0.047039588	0.330190145
ENSRNOG00000000532	Cmtr1	55.37088884	0.298039024	0.193772038
ENSRNOG00000023803	Cmya5	72.66688034	-0.13341251	0.15165304
ENSRNOG00000027739	Cndp1	11.03518014	-0.081895709	0.463992036
ENSRNOG00000015534	Cnep1r1	55.32694056	-0.131830266	0.172921093
ENSRNOG00000030119	Cnga2	62.26252273	-0.103630515	0.162168688
ENSRNOG00000017609	Cnga4	44.77936189	-0.205755878	0.18462343
ENSRNOG00000009811	Cnih1	11.94098952	0.507192791	0.417295095
ENSRNOG00000020247	Cnih2	38.84020808	-0.165403482	0.236006053
ENSRNOG00000022724	Cnih3	28.50187855	-0.032936188	0.255800797

ENSRNOG00000003717	Cnih4	26.02416433	0.054201859	0.256986021
ENSRNOG00000018052	Cnksr3	26.01190602	0.009550299	0.308733698
ENSRNOG00000012821	Cnmd	11.39441705	0.143997809	0.370413098
ENSRNOG00000016302	Cnnm1	18.40611789	0.129987451	0.335503979
ENSRNOG00000020113	Cnnm2	73.75106639	0.046251635	0.149451773
ENSRNOG00000016032	Cnnm3	14.43046907	0.657017174	0.377111571
ENSRNOG00000012271	Cnot1	27.04500724	0.007096964	0.248365625
ENSRNOG00000023220	Cnot11	37.33066578	-0.075876105	0.278496797
ENSRNOG00000004909	Cnot2	18.69186609	0.051198953	0.307772774
ENSRNOG00000010795	Cnot4	66.68600995	0.040295145	0.168198796
ENSRNOG00000054891	Cnot6	26.51089663	-0.014436658	0.285963813
ENSRNOG00000002075	Cnot6l	27.4866184	-0.167371779	0.264646065
ENSRNOG00000002630	Cnot8	44.35517721	0.487638109	0.213685841
ENSRNOG00000017496	Cnp	17.14533476	-0.095251051	0.341672173
ENSRNOG00000018325	Cnppd1	151.9552777	0.204288255	0.306431321
ENSRNOG00000002710	Cnst	11.49018776	-0.315829241	0.397972131
ENSRNOG00000012460	Cntf	15.09585436	0.443826594	0.382135055
ENSRNOG00000047307	Cntfr	149.8304425	0.171479081	0.122297427
ENSRNOG00000004438	Cntn1	29.73878542	0.004996713	0.236819716
ENSRNOG00000009033	Cntn2	32.58214434	0.048514767	0.214734574
ENSRNOG00000006144	Cntn3	24.41888747	0.248974649	0.266002131
ENSRNOG00000005652	Cntn4	113.3207615	0.248855432	0.127688725
ENSRNOG00000007038	Cntn5	11.85386696	0.251793653	0.359513861
ENSRNOG00000032517	Cntn6	32.89670743	-0.040584381	0.272662782
ENSRNOG00000020277	Cntnap1	15.24811882	0.174659578	0.408106529
ENSRNOG00000006617	Cntnap2	39.14250238	-0.049961693	0.21037671
ENSRNOG00000011231	Cntnap4	23.6828182	0.250347278	0.3031937
ENSRNOG00000022015	Cntrl	16.19141697	-0.229402956	0.293298716
ENSRNOG00000020487	Coa3	12.60700011	1.167266337	0.864144431
ENSRNOG00000010636	Coa7	28.83360787	0.000936885	0.224978326
ENSRNOG00000027016	Cobll1	44.30814255	0.187221937	0.186373502
ENSRNOG00000002795	Cog1	8.251025969	0.077347679	0.42969309
ENSRNOG00000018228	Cog2	17.84054744	-0.582896115	0.276763239
ENSRNOG00000000155	Cog3	24.97426074	0.058144304	0.270098141
ENSRNOG00000017745	Cog4	35.77688157	0.316656658	0.268776699
ENSRNOG00000056610	Cog5	22.89320153	0.002712308	0.25871243
ENSRNOG00000013660	Cog6	164.336761	0.158381415	0.103030571
ENSRNOG00000060008	Cog7	140.1931676	-0.150588358	0.10908515
ENSRNOG00000020379	Cog8	12.80758967	-0.416220524	0.359660004
ENSRNOG00000000244	Coil	112.3010524	-0.200424234	0.127024012
ENSRNOG00000023148	Col11a1	8.140328828	0.354998006	0.435203876
ENSRNOG00000058470	Col12a1	18.38490258	0.545839468	0.328635377
ENSRNOG00000026415	Col14a1	26.64187075	0.125717126	0.329929135
ENSRNOG00000060381	Col15a1	11.58992392	0.197585132	0.44064099
ENSRNOG00000031475	Col16a1	48.77571108	0.411586612	0.197184668
ENSRNOG00000012110	Col17a1	179.1658587	-0.024007253	0.144283089
ENSRNOG00000012759	Col19a1	11.65406585	0.467714754	0.46705597
ENSRNOG00000003897	Col1a1	114.2721717	0.193323234	0.229311558
ENSRNOG00000024824	Col22a1	27.30917457	0.250810921	0.239557945
ENSRNOG00000050706	Col25a1	8.821556364	0.423632658	0.543754651

ENSRNOG00000007657	Col27a1	10.69512642	-0.345855854	0.369661681
ENSRNOG00000003357	Col3a1	86.99811816	-0.100506205	0.172710249
ENSRNOG00000016281	Col4a1	103.8394302	0.201634291	0.15045958
ENSRNOG00000015365	Col4a3	14.20927589	-0.359055733	0.416069459
ENSRNOG00000014851	Col4a4	8.736766295	0.246660004	0.467438474
ENSRNOG00000018951	Col4a5	23.55512711	0.358595842	0.327697174
ENSRNOG00000056772	Col4a6	28.26569026	-0.192009708	0.25897958
ENSRNOG00000008749	Col5a1	8.069257519	0.631690564	0.472571373
ENSRNOG00000003736	Col5a2	22.35493709	0.098602914	0.313408689
ENSRNOG00000020525	Col5a3	39.12206235	0.112925327	0.230024235
ENSRNOG00000001249	Col6a1	11.16527888	0.04141178	0.411467984
ENSRNOG00000001254	Col6a2	33.00944314	0.371811265	0.259179388
ENSRNOG00000019648	Col6a3	13.51112413	0.373640543	0.365784053
ENSRNOG00000039668	Col8a1	9.158914979	0.927392838	0.47654171
ENSRNOG00000012920	Col9a1	20.06245039	0.553606508	0.293357797
ENSRNOG00000011502	Col9a2	101.8283008	-0.102836111	0.146115692
ENSRNOG00000009531	Col9a3	22.34638226	-0.00883443	0.304114369
ENSRNOG00000008373	Colec11	13.98534959	0.162825531	0.392973383
ENSRNOG00000016366	Colec12	36.73431832	0.032558955	0.236111217
ENSRNOG00000043386	Commd2	87.52195792	0.212815777	0.135169968
ENSRNOG00000016383	Commd3	78.57256969	0.157702416	0.141407499
ENSRNOG00000038012	Commd6	144.5668903	-0.018815951	0.115326632
ENSRNOG00000002320	Commd8	157.1157887	0.079949865	0.119772991
ENSRNOG00000020178	Cope	62.10292149	-0.212211191	0.172601266
ENSRNOG00000053943	Cops3	168.7215476	-0.094006993	0.108408686
ENSRNOG00000023650	Cops4	129.8599012	0.154672727	0.110421459
ENSRNOG00000018723	Cops7b	118.099343	0.078623341	0.137296686
ENSRNOG00000019635	Cops8	12.21438288	-0.018590533	0.356961888
ENSRNOG00000009225	Copz2	16.6537463	-0.210916476	0.317959865
ENSRNOG00000014456	Coq10b	46.92572022	-0.18550538	0.199262291
ENSRNOG00000002194	Coq2	163.5348978	0.181500159	0.129006269
ENSRNOG00000009974	Coq3	103.2908772	-0.0349181	0.138257834
ENSRNOG00000026691	Coq4	62.52004942	-0.159579164	0.164730883
ENSRNOG00000017012	Coq7	123.1556926	0.088083361	0.130799142
ENSRNOG00000020848	Coq8b	51.66308476	-0.27520704	0.177982771
ENSRNOG00000002302	Corin	8.062012122	0.459094622	0.455188959
ENSRNOG00000019430	Coro1a	92.3471844	0.208594815	0.375176415
ENSRNOG00000008901	Coro2a	13.85900907	0.164725279	0.396919322
ENSRNOG00000015257	Coro2b	30.3827996	0.413051841	0.237470803
ENSRNOG00000004146	Coro7	14.03444716	-0.190481033	0.363247718
ENSRNOG00000024972	Cox10	78.32373942	-0.050337354	0.161563387
ENSRNOG00000052096	Cox11	16.77642313	-0.269122363	0.326253592
ENSRNOG00000017230	Cox15	55.13447113	0.024236877	0.207879431
ENSRNOG00000003052	Cox18	85.27334162	0.184031685	0.140245124
ENSRNOG00000007827	Cox4i2	8.286794698	0.327071397	0.472786097
ENSRNOG00000042903	Cox7a2l2	9.720225771	0.079528807	0.408920089
ENSRNOG00000011913	Cp	7.914901043	-0.794253804	0.443815105
ENSRNOG00000003873	Cpd	78.71319369	-0.014338408	0.190010335
ENSRNOG00000019161	Cpeb1	11.64283137	-0.313646481	0.351230684
ENSRNOG00000005043	Cpeb2	19.57201423	0.097522926	0.293577159

ENSRNOG00000020689	Cpeb3	37.77919084	0.215397916	0.229882517
ENSRNOG00000033169	Cpeb4	21.39005313	0.80218506	0.28437035
ENSRNOG00000042118	Cplane1	62.74421713	0.217209128	0.183766261
ENSRNOG00000023346	Cplane2	21.5613112	-0.129853586	0.293527764
ENSRNOG00000019317	Cplx3	88.72020893	-0.090070363	0.143285545
ENSRNOG00000019723	Cpne1	43.56407403	-0.159364688	0.206369769
ENSRNOG00000043286	Cpne2	22.70522546	-0.049623687	0.274807522
ENSRNOG00000006298	Cpne3	13.03171019	-0.070156625	0.38448215
ENSRNOG00000018399	Cpne6	27.19062165	-0.020326248	0.272890385
ENSRNOG00000015397	Cpne7	171.3805037	0.002352537	0.104153112
ENSRNOG00000026128	Cpne8	127.7373602	0.080420806	0.148025506
ENSRNOG00000023077	Cpne9	16.30265988	-0.343713332	0.319229894
ENSRNOG00000001654	Cpox	151.8989673	-0.171785342	0.129149748
ENSRNOG00000015118	Cpped1	37.40235043	-0.06915102	0.225140599
ENSRNOG00000005931	Cpq	158.4314808	0.017819805	0.12857234
ENSRNOG00000013704	Cps1	9.526128598	0.221697688	0.42306671
ENSRNOG00000052418	Cpsf3	40.45090178	0.210624203	0.212510626
ENSRNOG00000000985	Cpsf4	20.87839028	-0.023304693	0.33132486
ENSRNOG00000020668	Cpsf7	187.9963527	0.216696495	0.110026595
ENSRNOG00000026163	Cpt1c	105.2474113	-0.044968421	0.166371183
ENSRNOG00000012443	Cpt2	65.84586124	0.079084275	0.174963269
ENSRNOG00000021220	Cpxm1	43.19805293	-0.091324511	0.205311029
ENSRNOG00000015902	Cpxm2	10.27428785	-0.166516988	0.389962563
ENSRNOG00000008947	Cpz	11.69886635	0.832902684	0.416011545
ENSRNOG00000008193	Cr1l	115.3811514	-0.1668952	0.147765216
ENSRNOG00000023633	Crabp1	34.18432826	-0.001240116	0.234732232
ENSRNOG00000022101	Crabp2	18.67289328	0.208155685	0.30044582
ENSRNOG00000002139	Cracd	24.0366026	0.541360136	0.427919393
ENSRNOG00000024635	Cramp1	23.36561227	-0.109962672	0.36670287
ENSRNOG00000018145	Crat	19.55592204	-0.097874892	0.340119778
ENSRNOG00000006534	Crbn	10.74936865	0.184831075	0.405264284
ENSRNOG00000013412	Creb1	8.162018031	0.396871542	0.458950002
ENSRNOG00000005413	Creb3l1	31.77423387	-0.213396763	0.290128409
ENSRNOG00000012826	Creb3l2	13.80362908	0.707888385	0.386278269
ENSRNOG00000008622	Creb5	9.507606667	-0.189320465	0.458144373
ENSRNOG00000005330	Crebbp	188.8063033	-0.029822019	0.139883136
ENSRNOG00000020769	Crebrf	64.40893325	-0.091646642	0.156240964
ENSRNOG00000003291	Creg1	133.8614965	0.049679397	0.151846739
ENSRNOG00000013991	Creg2	8.79757877	-0.348512601	0.481635485
ENSRNOG00000004659	Creld2	118.6829955	0.082896547	0.148204718
ENSRNOG00000014900	Crem	129.6175759	-0.195462934	0.135295449
ENSRNOG00000017890	Crhbp	61.53031964	-0.009099414	0.192981133
ENSRNOG00000004208	Crim1	52.35073381	0.182309848	0.188442648
ENSRNOG00000005041	Crip2	39.16059897	1.409467625	0.591343204
ENSRNOG00000016752	Crispld2	26.07237319	0.045175364	0.304741987
ENSRNOG00000025792	Crk	20.90576997	-0.176692386	0.322364012
ENSRNOG00000001868	Crkl	164.3148211	-0.024080601	0.116676434
ENSRNOG00000049828	Crlf2	60.24153092	0.180437877	0.167854321
ENSRNOG00000050657	Crlf3	9.658989341	0.087761324	0.431055547
ENSRNOG00000010587	Crnkl1	16.51623364	0.535388532	0.383779075

ENSRNOG00000040045	Crnkl1	21.39964924	-0.141151622	0.31757081
ENSRNOG00000006779	Crot	78.73394795	-0.232110963	0.183237495
ENSRNOG00000006199	Crppa	25.72797989	-0.314225999	0.241667825
ENSRNOG00000015220	Crtac1	66.12517349	0.137223288	0.179078731
ENSRNOG00000009878	Crtapl1	57.28241127	0.207897076	0.182331741
ENSRNOG00000022421	Crtc1	35.0093983	0.324384406	0.232673936
ENSRNOG00000011975	Crtc3	51.34216968	0.357625045	0.185290278
ENSRNOG00000006622	Cry1	19.11726414	0.5781699	0.442843942
ENSRNOG00000010524	Cryab	150.9658587	0.214064216	0.128168539
ENSRNOG00000008700	Cryba1	129.6589905	0.165809879	0.139841899
ENSRNOG00000047653	Crybb1	9.909290581	0.025108381	0.394493587
ENSRNOG00000014790	Cryga	86.07862434	-0.249178928	0.157380715
ENSRNOG00000046343	Cryga	9.184922551	0.192784021	0.422237597
ENSRNOG00000008989	Cryl1	183.0310855	-0.114974375	0.111149283
ENSRNOG00000028319	Cryz	44.75140875	0.221575189	0.208508416
ENSRNOG00000028014	Cryzl1	47.30170147	0.044288547	0.187129174
ENSRNOG00000023520	Cs	17.62444429	0.836729598	0.329963149
ENSRNOG00000011573	Csad	178.4494519	0.019249644	0.100305963
ENSRNOG00000005332	Csdc2	18.70461654	-0.002962722	0.317015112
ENSRNOG00000007665	Cse1l	16.97978409	-0.117268755	0.347455966
ENSRNOG00000018659	Csf1	54.18883837	0.032176031	0.182988558
ENSRNOG00000018414	Csf1r	137.5171692	-0.004189636	0.125837013
ENSRNOG00000049782	Csf2ra	178.6898109	0.056450457	0.100451282
ENSRNOG00000013024	Csgalnact1	172.8638995	-0.227743084	0.124339883
ENSRNOG00000014713	Csgalnact2	29.04320464	0.243616718	0.367395241
ENSRNOG00000030719	Csmd1	56.83973429	0.179610731	0.207667903
ENSRNOG00000007057	Csmd2	43.03282499	0.309273526	0.271761477
ENSRNOG00000004937	Csmd3	48.78171508	0.407945628	0.21429502
ENSRNOG00000036676	Csnk1d	57.68590024	0.082517914	0.17862636
ENSRNOG00000016620	Csnk1g1	23.75899891	0.400198373	0.331882823
ENSRNOG00000016677	Csnk1g3	12.82650305	0.626484366	0.393521777
ENSRNOG00000005276	Csnk2a1	86.96968205	-0.391050862	0.173336612
ENSRNOG00000017208	Cspg4	70.95859491	0.142317637	0.190526244
ENSRNOG00000021718	Cspp1	36.55211622	0.043819366	0.207812528
ENSRNOG00000005359	Csrnp3	62.46971656	0.107844521	0.156759867
ENSRNOG00000004775	Cstf1	107.777264	0.133098904	0.131560494
ENSRNOG00000048025	Cstf2	14.11325304	-0.028609659	0.367350575
ENSRNOG00000012089	Cstf3	116.3841593	0.046701921	0.131588961
ENSRNOG00000017326	Ctbp2	156.4951412	-0.028766863	0.112953871
ENSRNOG00000017674	Ctcf	103.6656565	-0.05719487	0.159796456
ENSRNOG00000017352	Ctdnep1	11.24350053	0.002312956	0.388476576
ENSRNOG00000061474	Ctdp1	69.24910507	0.237712625	0.156101456
ENSRNOG00000022141	Ctdspl2	65.0603468	-0.17250493	0.157517681
ENSRNOG00000030907	Ctf2	8.864442076	0.947285401	0.493518113
ENSRNOG00000010658	Cth	43.82562478	-0.179544258	0.184075125
ENSRNOG00000004578	Cthrc1	9.052513945	0.544355662	0.431715857
ENSRNOG00000018342	Ctif	168.7239121	-0.110299444	0.105827237
ENSRNOG00000006003	Ctnna2	8.815401593	0.135626642	0.477757051
ENSRNOG00000010593	Ctnnal1	99.71294372	-0.017890331	0.143251788
ENSRNOG00000054172	Ctnnb1	181.7226551	-0.442796634	0.136425333

ENSRNOG00000012021	Ctnnbl1	130.6344685	0.198502801	0.121891825
ENSRNOG00000030790	Ctnnd1	84.13217493	0.116125374	0.152294859
ENSRNOG00000009963	Ctps1	34.70321023	0.022627935	0.295095172
ENSRNOG00000004257	Ctps2	152.3834345	0.09924731	0.126650784
ENSRNOG00000017195	Ctr9	146.59158	0.298993837	0.118491802
ENSRNOG00000019068	Ctrb1	8.102654839	-0.322644712	0.496155352
ENSRNOG00000013745	Ctrc	30.64920131	-0.025494702	0.240742153
ENSRNOG00000010331	Ctsb	57.72047065	-0.017933908	0.185591371
ENSRNOG00000016496	Ctsc	21.65475622	0.202721933	0.300692284
ENSRNOG00000021155	Ctsk	105.9110783	0.108534984	0.15622987
ENSRNOG00000050697	Ctsz	156.335132	-0.29399494	0.118029371
ENSRNOG00000014479	Cttnbp2nl	84.45030982	-0.048167806	0.166025415
ENSRNOG00000018334	Ctu1	75.40453273	0.210939103	0.162096992
ENSRNOG00000037146	Ctxn2	12.96335045	-0.451746606	0.356486247
ENSRNOG00000022957	Ctxn3	46.41113551	0.43171689	0.253727127
ENSRNOG00000010257	Cuedc1	113.7661685	0.234869401	0.127987704
ENSRNOG00000005310	Cul1	54.1475349	-0.103259809	0.185306003
ENSRNOG00000019649	Cul4a	55.38116705	0.439728053	0.184946544
ENSRNOG00000002585	Cul4b	43.58648393	0.028483249	0.195596409
ENSRNOG00000008039	Cul5	45.73092682	0.032199868	0.211940353
ENSRNOG00000017857	Cul7	105.5265756	-0.041040586	0.130558278
ENSRNOG00000018372	Cul9	26.42012	-0.321100887	0.250048955
ENSRNOG00000017298	Cutc	25.98414662	0.370022864	0.271010813
ENSRNOG00000001424	Cux1	8.095309922	1.036028916	0.514494993
ENSRNOG00000001259	Cux2	85.51414989	0.095349051	0.151567216
ENSRNOG00000008490	Cwc15	189.4304296	-0.111919286	0.110106565
ENSRNOG00000012651	Cwc22	25.78587485	0.107214154	0.25553218
ENSRNOG00000004091	Cwc25	89.95841831	-0.281441467	0.317375721
ENSRNOG00000013252	Cwc27	37.62482419	0.007469186	0.272372246
ENSRNOG00000024372	Cwf19l2	72.75321651	0.059820295	0.157766813
ENSRNOG00000018509	Cx3cr1	15.4660408	0.390645028	0.356044132
ENSRNOG00000001557	Cxadr	16.88880943	-0.452936958	0.302213277
ENSRNOG00000029029	Cxadrl1	97.37502826	-0.064552183	0.128534196
ENSRNOG00000037814	Cxcl17	15.64927077	0.370874035	0.339879537
ENSRNOG00000003866	Cxcr4	8.850468344	0.115582768	0.480752037
ENSRNOG00000012430	Cxcr5	60.63534009	0.133252597	0.173242468
ENSRNOG00000014614	Cxxc1	115.2827593	-0.027025454	0.147766119
ENSRNOG00000045764	Cxxc4	135.5806298	0.061538696	0.126353371
ENSRNOG00000052232	Cyb561d1	47.46597499	0.180654191	0.187721494
ENSRNOG00000024553	Cyb5d2	22.07086136	0.453122312	0.276321889
ENSRNOG00000003973	Cyb5r1	21.03261735	-0.508226541	0.268915418
ENSRNOG00000010024	Cyb5r4	134.0358864	-0.020340229	0.128475479
ENSRNOG00000009148	Cyb5rl	10.43557318	0.096954586	0.417858939
ENSRNOG00000013014	Cyba	104.2386556	-0.04239432	0.127888568
ENSRNOG00000003622	Cybb	12.42802947	0.158882938	0.379595989
ENSRNOG00000009620	Cybrd1	8.795697939	-0.736550326	0.448063139
ENSRNOG00000012457	Cyc1	29.74114168	-0.106329542	0.21972607
ENSRNOG00000011945	Cyfip1	168.052356	-0.056807195	0.122538595
ENSRNOG00000014048	Cyld	29.98139366	0.303563698	0.27031136
ENSRNOG00000008861	Cyld-ps1	9.692888175	0.160293578	0.391878026

ENSRNOG00000030111	Cyp11b2	45.83324337	0.349702567	0.195783071
ENSRNOG00000000196	Cyp19a1	13.87731246	-0.305712622	0.462815952
ENSRNOG00000040287	Cyp1b1	8.082677322	-0.113657149	0.448847745
ENSRNOG00000017631	Cyp20a1	89.12244733	-0.023845413	0.181781564
ENSRNOG00000016750	Cyp26a1	13.50052073	-0.030431962	0.356826729
ENSRNOG00000015076	Cyp26b1	158.5719619	-0.25770409	0.191949861
ENSRNOG00000030698	Cyp26c1	101.3212623	-0.212635759	0.166549936
ENSRNOG00000013241	Cyp2c24	105.6642687	0.201352844	0.122106467
ENSRNOG00000029179	Cyp2d3	13.95520662	0.196779379	0.463984365
ENSRNOG00000032261	Cyp2d4	48.75707686	0.330085319	0.235573891
ENSRNOG00000029128	Cyp2d5	8.951114249	-0.541292832	0.400582454
ENSRNOG00000042224	Cyp2j10	31.80542852	-0.380704136	0.228565318
ENSRNOG00000011053	Cyp2u1	55.19013591	-0.248096131	0.188798346
ENSRNOG00000051770	Cyp2w1	10.5239234	-0.295486122	0.44080202
ENSRNOG00000001379	Cyp3a62	14.25677909	-0.021762917	0.344521674
ENSRNOG00000004786	Cyp4f1	26.09045171	0.279633481	0.258696603
ENSRNOG00000029478	Cyp4f39	78.55980027	-0.107156874	0.198627058
ENSRNOG00000032895	Cyp4f4	26.87424163	0.251079836	0.269429844
ENSRNOG00000042496	Cyp4f5	16.07929985	-0.554953532	0.397057359
ENSRNOG00000043233	Cyp4f6	30.26085188	0.125059382	0.27272206
ENSRNOG00000042426	Cyp4v3	37.45683022	0.136858115	0.220221817
ENSRNOG00000043513	Cyp4x1	8.028265106	0.592976236	0.499637425
ENSRNOG00000007234	Cyp51	26.2962969	-0.222623405	0.244931246
ENSRNOG00000009730	Cyp7b1	68.26123298	-0.022028704	0.183211821
ENSRNOG00000026958	Cyren	35.78242238	0.193035529	0.222120007
ENSRNOG00000010600	Cysrt1	22.55207473	0.151359132	0.273046461
ENSRNOG00000018775	Cystm1	99.0596547	-0.122923858	0.130456439
ENSRNOG00000043381	Cyth1	181.3690843	0.009231324	0.118574431
ENSRNOG00000001065	Cyth3	8.593279178	-2.321595889	1.115801987
ENSRNOG00000007679	Cyth4	19.2795124	0.616592515	0.316922148
ENSRNOG00000001544	Cyyr1	49.8278396	0.07097955	0.222742753
ENSRNOG00000012724	Czib	97.77915071	-0.065322715	0.14052319
ENSRNOG00000025644	D030056L22Rik	188.8880009	0.16150001	0.114540548
ENSRNOG00000019012	D2hgdh	63.37762965	0.167220753	0.17872082
ENSRNOG00000011132	D430041D05Rik	45.22630762	0.214772705	0.278090451
ENSRNOG00000004345	Daam1	179.5455807	0.005356715	0.100711505
ENSRNOG00000053945	Daam2	13.18114974	0.134938489	0.34866996
ENSRNOG00000028930	Dab2	9.221352266	0.025778035	0.42640372
ENSRNOG00000027264	Dagla	66.49053708	-0.290675804	0.182699959
ENSRNOG00000001079	Daglb	25.33682605	0.064311818	0.30415727
ENSRNOG00000010747	Dap	136.6423879	-0.178370113	0.132551437
ENSRNOG00000017332	Dapk2	29.31756613	0.132672314	0.272815454
ENSRNOG00000010575	Dapp1	8.950459882	0.527119416	0.45857269
ENSRNOG00000002813	Dars2	64.3547851	0.009422122	0.155355511
ENSRNOG00000031387	Dazap1	132.0185901	0.058443152	0.116856338
ENSRNOG00000006641	Dbh	9.270165559	-0.708748377	0.440209476
ENSRNOG00000014170	Dbn1	21.59784718	0.227714674	0.306859563
ENSRNOG00000026974	Dbndd1	35.37022044	0.306707261	0.222253381
ENSRNOG00000012378	Dbnl	25.32873237	0.036583061	0.245916872
ENSRNOG00000021027	Dbp	9.61994958	-0.099262887	0.441024953

ENSRNOG00000014588	Dbr1	34.41715424	-0.454674446	0.263755512
ENSRNOG00000015029	Dbt	88.73143587	-0.003935196	0.143189682
ENSRNOG00000013841	Dcaf1	159.6982633	-0.073262147	0.123248352
ENSRNOG00000011655	Dcaf10	40.21429223	-0.038030708	0.21853233
ENSRNOG00000050790	Dcaf12	148.2802418	0.039746488	0.114704684
ENSRNOG00000004301	Dcaf13	25.50795048	-0.21963612	0.252231366
ENSRNOG00000009403	Dcaf17	9.072858082	0.817604894	0.51966313
ENSRNOG00000008399	Dcaf4	9.478391805	-0.220396885	0.421651034
ENSRNOG00000021682	Dcakd	159.7935095	-0.025540357	0.134987009
ENSRNOG00000033099	Dcc	12.85381015	0.775838664	0.386520312
ENSRNOG00000017511	Dcdc2	9.796568382	-0.098623114	0.456171549
ENSRNOG00000003296	Dck	160.7588389	-0.158886807	0.111883398
ENSRNOG00000033026	Dclk3	130.0926622	-0.061130107	0.133512826
ENSRNOG00000026204	Dclre1a	8.957540074	0.15400162	0.445022715
ENSRNOG00000019367	Dclre1b	36.30653564	-0.190208668	0.198405285
ENSRNOG00000015980	Dclre1c	15.79957802	0.144599092	0.39311017
ENSRNOG00000004554	Dcn	66.95839269	0.158105629	0.233271133
ENSRNOG00000016015	Dcp1a	66.72480558	0.186813355	0.160094079
ENSRNOG00000007340	Dcp1b	85.97371601	-0.029286328	0.151249737
ENSRNOG00000045895	Dcp2	12.83238165	-0.467656298	0.347803969
ENSRNOG00000058618	Dcst2	35.63083523	-0.068294693	0.227630125
ENSRNOG00000013215	Dctd	24.08210625	-0.159219805	0.24303575
ENSRNOG00000025481	Dctn2	60.90758123	-0.309380801	0.187892552
ENSRNOG00000019298	Dctn4	63.03464337	-0.456688936	0.227121592
ENSRNOG00000012734	Dcun1d1	112.4083408	-0.098349231	0.120953408
ENSRNOG00000019473	Dcun1d2	11.28325638	0.073407322	0.401083009
ENSRNOG00000047712	Dcx	45.95121574	-0.023028936	0.224615302
ENSRNOG00000039417	Dda1	101.4495749	0.176103492	0.143420031
ENSRNOG00000020715	Ddb1	26.11922673	0.679123766	0.278197742
ENSRNOG00000014071	Ddb2	72.3677662	0.067718838	0.162307637
ENSRNOG00000004327	Ddc	76.24835536	-0.053710457	0.173354433
ENSRNOG00000009481	Ddhd1	190.7625754	-0.01600847	0.136622285
ENSRNOG00000012274	Ddi2	172.7188616	-0.05937475	0.114847049
ENSRNOG00000006789	Ddit3	19.84847176	0.008492508	0.28465958
ENSRNOG00000010047	Ddit4l	8.856375287	-0.15309058	0.421722635
ENSRNOG00000000582	Ddo	37.78966264	-0.578538487	0.226916061
ENSRNOG00000015079	Ddost	109.9523955	0.155264953	0.150135134
ENSRNOG00000002881	Ddr2	12.65525531	0.620638117	0.465576053
ENSRNOG00000012500	Ddx10	124.133734	-0.130440662	0.136243418
ENSRNOG00000051170	Ddx17	58.89710885	0.445457702	0.441361198
ENSRNOG00000025430	Ddx18	56.20182242	0.193934757	0.173587585
ENSRNOG00000018033	Ddx19a	96.04320758	0.07425785	0.151317149
ENSRNOG00000015274	Ddx20	8.442451304	0.022489608	0.413391933
ENSRNOG00000043099	Ddx21	16.42754527	0.246880904	0.318946291
ENSRNOG00000060154	Ddx23	12.46955276	-0.003410132	0.360597414
ENSRNOG00000009166	Ddx24	82.18391397	0.229674132	0.172122746
ENSRNOG00000008081	Ddx27	24.70109293	0.230903202	0.273548571
ENSRNOG00000019817	Ddx28	58.83489078	-0.200119754	0.169042195
ENSRNOG00000057231	Ddx3	166.9602075	-0.040499229	0.114087717
ENSRNOG00000013040	Ddx31	18.82277802	-0.060375193	0.272790944

ENSRNOG00000004373	Ddx39a	29.10265778	0.713518104	0.266395356
ENSRNOG00000009474	Ddx42	33.41341877	0.116244797	0.239191288
ENSRNOG00000050868	Ddx43	7.990176064	1.342341094	0.762541934
ENSRNOG00000021637	Ddx46	89.55111523	-0.154303236	0.133830598
ENSRNOG00000022368	Ddx49	38.13231491	0.020362443	0.204344836
ENSRNOG00000030680	Ddx5	75.87746686	-0.140326787	0.856903766
ENSRNOG00000037480	Ddx51	55.07415661	0.592536771	0.190114113
ENSRNOG00000002612	Ddx52	13.33324356	0.037540168	0.373845109
ENSRNOG00000001043	Ddx55	10.28776164	0.049748076	0.383200503
ENSRNOG00000004670	Ddx56	157.6002721	-0.001078459	0.114133701
ENSRNOG00000042451	Ddx59	36.18537823	-0.072065421	0.201474623
ENSRNOG00000053932	Ddx6	56.47201023	-0.114189739	0.198685325
ENSRNOG00000017960	Deaf1	32.32841351	-0.032721696	0.224331111
ENSRNOG00000008236	Decr1	32.91580264	0.03863852	0.256402592
ENSRNOG00000003779	Dedd	74.4059552	-0.211605512	0.215960312
ENSRNOG00000046112	Dedd2	84.96450246	-0.062999651	0.152947267
ENSRNOG00000000502	Def6	18.75651896	0.061114969	0.306988415
ENSRNOG00000000264	Def8	115.453006	0.163182353	0.136728136
ENSRNOG00000016152	Dek	60.77115896	-0.083748272	0.211345048
ENSRNOG00000019276	Dele1	46.31032093	-0.186979542	0.188698883
ENSRNOG00000010230	Dennd10	10.60655857	-0.339186459	0.383655799
ENSRNOG00000011854	Dennd11	20.92422143	-0.065701408	0.270393834
ENSRNOG00000010526	Dennd1a	28.31123233	-0.12511546	0.258985258
ENSRNOG00000026748	Dennd2a	87.54962419	-0.095112236	0.169643793
ENSRNOG00000013934	Dennd2b	10.16366962	0.758043487	0.407833564
ENSRNOG00000010794	Dennd3	57.10004458	-0.005011228	0.201869916
ENSRNOG00000011739	Dennd4a	36.19138763	0.693405666	0.242379231
ENSRNOG00000022373	Dennd4b	21.84675625	-0.095845014	0.261583057
ENSRNOG00000008036	Dennd4c	45.71418537	0.037353658	0.196954958
ENSRNOG00000049378	Dennd5b	42.19789836	-0.200389781	0.227918397
ENSRNOG00000011636	Dennd6a	88.54318388	0.085161135	0.135158889
ENSRNOG00000029866	Dennd6b	164.0542199	-0.045261087	0.122974212
ENSRNOG00000001106	Denr	26.13369049	-0.006054174	0.237700393
ENSRNOG00000018144	Depdc5	127.334852	0.120413667	0.123358829
ENSRNOG00000012262	Depdc7	13.56565603	-0.163914362	0.40151622
ENSRNOG00000055466	Derl2	69.2392339	-0.073400075	0.175857193
ENSRNOG00000019810	Des	32.36329966	0.18944316	0.243623885
ENSRNOG00000005577	Desi1	136.1798845	0.015810021	0.114381893
ENSRNOG00000004524	Desi2	116.6928441	0.173133393	0.144737012
ENSRNOG00000018515	Det1	17.7567354	0.327015229	0.301923287
ENSRNOG00000013603	Dffa	11.57951109	0.036800392	0.381637118
ENSRNOG00000061814	Dgcr2	11.33058421	-0.015178651	0.376736885
ENSRNOG00000001880	Dgcr6	16.09112956	-0.245849548	0.339919576
ENSRNOG00000022943	Dgka	25.51863661	-0.531128127	0.242112041
ENSRNOG00000023238	Dgkd	17.79545068	0.051186476	0.316771641
ENSRNOG00000002338	Dgke	92.5524489	0.294167245	0.143458861
ENSRNOG00000001796	Dgkg	163.7459894	-0.075643956	0.13982153
ENSRNOG00000010065	Dgkh	7.85401971	0.318979787	0.455271246
ENSRNOG00000026705	Dgki	56.71865303	0.1748073	0.203174379
ENSRNOG00000024112	Dgkq	166.1894743	-0.074166276	0.113951114

ENSRNOG00000004442	Dglucy	12.18420156	-0.191955261	0.340435749
ENSRNOG00000011617	Dguok	111.9953751	0.266971415	0.139138094
ENSRNOG00000020776	Dhcr7	96.07907824	-0.026695679	0.163229264
ENSRNOG00000014665	Dhdds	54.75300547	0.259342028	0.217819046
ENSRNOG00000020883	Dhdh	20.76349717	0.708962576	0.297248916
ENSRNOG00000013521	Dhfr	26.39494662	0.036690498	0.235385942
ENSRNOG00000053675	Dhh	16.60629343	0.247591635	0.31728412
ENSRNOG00000015063	Dhodh	24.73109776	-0.469101915	0.288997427
ENSRNOG00000020264	Dhrs1	102.8856733	0.081824698	0.192369309
ENSRNOG00000015736	Dhrs3	127.8927537	-0.111598839	0.119223477
ENSRNOG00000018239	Dhrs4	8.908371859	0.37310745	0.463372648
ENSRNOG00000005360	Dhrs7b	186.8285392	-0.139885446	0.101545396
ENSRNOG00000028323	Dhrsx	8.095208785	-0.294898531	0.405330002
ENSRNOG00000010369	Dhx29	60.29298636	-0.012929086	0.21469966
ENSRNOG00000029194	Dhx30	22.85830014	0.296596009	0.263425668
ENSRNOG00000018119	Dhx32	14.59835991	0.259140424	0.452751014
ENSRNOG00000007054	Dhx33	33.35019581	-0.035196321	0.216077494
ENSRNOG00000047575	Dhx34	16.8617166	0.665812393	0.343191517
ENSRNOG00000015928	Dhx35	60.64529155	0.470676118	0.188229043
ENSRNOG00000022171	Dhx37	30.02211236	0.579572699	0.271577949
ENSRNOG00000014619	Dhx38	74.3627023	0.324101689	0.150096258
ENSRNOG00000004549	Dhx40	140.1377229	0.050308831	0.114404689
ENSRNOG00000018247	Dhx58	14.01882657	-0.205884407	0.368115773
ENSRNOG00000020772	Dhx8	12.81175492	-0.484327915	0.388857551
ENSRNOG00000002735	Dhx9	15.93516736	-0.066327325	0.329795624
ENSRNOG00000029197	Diablo	13.85057097	-0.353110439	0.328395257
ENSRNOG00000010711	Dicer1	72.49040923	0.216139051	0.156839055
ENSRNOG00000009936	Dido1	73.66749852	0.119477175	0.15382625
ENSRNOG00000052017	Dio3	23.02537807	-0.373927605	0.288950385
ENSRNOG00000056106	Dip2b	29.77054621	0.159683335	0.250588345
ENSRNOG00000015288	Dip2c	42.12458528	0.240471979	0.231113773
ENSRNOG00000023533	Dipk1a	28.71657005	0.43899679	0.242128018
ENSRNOG00000004532	Dipk1b	75.09639596	0.13134447	0.183831896
ENSRNOG00000029738	Diras1	26.02321667	0.056273952	0.233064224
ENSRNOG00000009125	Dis3	9.24276163	-0.153034985	0.409653295
ENSRNOG00000010537	Dis3l	34.44572011	-0.164169491	0.25794223
ENSRNOG00000018931	Dis3l2	54.62606651	0.16263355	0.178729591
ENSRNOG00000003635	Disp1	21.17110939	0.056808904	0.266777055
ENSRNOG00000026447	Disp3	10.31433272	-0.457155777	0.397415948
ENSRNOG00000010260	Dixdc1	61.59162575	-0.089882311	0.172864765
ENSRNOG00000009994	Dlat	46.85532003	-0.116665209	0.206376862
ENSRNOG00000010780	Dlc1	118.6179154	0.100135566	0.190407452
ENSRNOG00000006364	Dld	24.77930732	-0.406476702	0.252500459
ENSRNOG00000032085	Dlec1	8.78401629	-0.176367133	0.530552273
ENSRNOG00000009181	Dleu7	95.71509665	-0.200511743	0.158274562
ENSRNOG00000038597	Dlg1	100.7272359	0.107062281	0.145491021
ENSRNOG00000022635	Dlg2	44.99187521	0.446270275	0.20015259
ENSRNOG00000012573	Dlgap2	22.32941925	0.494690453	0.340612858
ENSRNOG00000019584	Dlk1	9.079468827	0.71625421	0.586912143
ENSRNOG00000059984	Dll1	15.33923669	0.311136472	0.389619121

ENSRNOG00000019338	Dll3	156.0267965	-0.248258771	0.142583231
ENSRNOG00000014011	Dll4	15.94227647	0.109540372	0.45187288
ENSRNOG00000001520	Dlx1	18.98115992	-0.339020451	0.312083437
ENSRNOG00000010905	Dlx5	31.03118726	-0.107805228	0.230910876
ENSRNOG00000023946	Dmac1	84.13924478	0.260451869	0.148219375
ENSRNOG00000004893	Dmac2l	78.54380368	0.263214989	0.171233856
ENSRNOG00000013807	Dmc1	9.488946772	0.775336057	0.427751443
ENSRNOG00000046366	Dmd	134.9505959	-0.000885577	0.136595949
ENSRNOG00000048295	Dmrtc1a	136.1452118	-0.118962958	0.120257318
ENSRNOG00000005908	Dmtf1	15.15184411	-0.204306285	0.341415251
ENSRNOG00000012273	Dmtn	27.99907418	0.339338872	0.295940958
ENSRNOG00000014984	Dmwd	26.92987074	0.186500605	0.292162937
ENSRNOG00000024671	Dmxl1	55.07183011	0.340159931	0.222976566
ENSRNOG00000000387	Dna2	109.5421731	-0.25497821	0.158083628
ENSRNOG00000005233	Dnaaf11	10.66365166	0.681747515	0.453094152
ENSRNOG00000021312	Dnaaf5	44.38800249	-0.153465083	0.227098114
ENSRNOG00000021237	Dnaaf9	114.7646307	0.100070529	0.16253706
ENSRNOG00000003028	Dnah17	13.50482835	-0.279782627	0.35276061
ENSRNOG00000052688	Dnah2	20.38105047	-0.020603146	0.273765584
ENSRNOG00000050426	Dnah3	7.536695665	-1.070056542	0.688324032
ENSRNOG00000015581	Dnah6	9.625347091	-0.427261066	0.375018611
ENSRNOG00000000542	Dnah8	11.43858099	0.037262434	0.404212448
ENSRNOG00000004171	Dnah9	50.10207812	-0.235537198	0.17908641
ENSRNOG00000006999	Dnai4	102.1583752	0.159403524	0.134922533
ENSRNOG00000027630	Dnai7	22.90426261	0.055096077	0.256896974
ENSRNOG00000007029	Dnaja1	169.7294286	0.108273175	0.109681481
ENSRNOG00000016251	Dnaja2	26.62680898	0.274992428	0.28603526
ENSRNOG00000003855	Dnaja3	172.8795895	-0.173148731	0.115098542
ENSRNOG00000012106	Dnaja4	12.7179284	0.018863447	0.339260319
ENSRNOG00000017975	Dnajb13	28.75933494	0.244552057	0.25210949
ENSRNOG00000019506	Dnajb2	17.16643532	0.480593828	0.350122016
ENSRNOG00000000150	Dnajc1	88.52543237	-0.186447241	0.142373423
ENSRNOG00000008802	Dnajc11	46.12718903	-0.298008154	0.210154707
ENSRNOG00000051960	Dnajc12	136.5112497	-0.146859514	0.12766139
ENSRNOG00000011491	Dnajc13	53.48120152	0.328917459	0.20379342
ENSRNOG00000006844	Dnajc14	24.49862213	0.319236862	0.257600619
ENSRNOG00000012503	Dnajc16	9.850040836	-0.318708736	0.378490559
ENSRNOG00000019995	Dnajc18	90.92593656	0.064757034	0.142783967
ENSRNOG00000049819	Dnajc19	122.8172702	0.055586316	0.125136108
ENSRNOG00000012392	Dnajc2	43.99302243	-0.252949459	0.203844805
ENSRNOG00000004842	Dnajc24	17.32581831	0.298640454	0.337861315
ENSRNOG00000015340	Dnajc25	10.55478158	-0.018147441	0.522452851
ENSRNOG00000002026	Dnajc28	10.04837695	-0.047310617	0.401080854
ENSRNOG00000010352	Dnajc3	60.66971726	-0.141148976	0.208746739
ENSRNOG00000031721	Dnajc30	109.5811185	-0.000449235	0.120262046
ENSRNOG00000015202	Dnajc5	161.1140442	0.198988096	0.118510612
ENSRNOG00000017781	Dnajc7	20.55251817	-0.351579607	0.298013573
ENSRNOG00000006619	Dnajc9	34.65087119	-0.069632263	0.227924963
ENSRNOG00000015583	Dnal4	183.6355274	-0.140579377	0.125459059
ENSRNOG00000023830	Dnase2	8.303960711	-0.666508571	0.459245086

ENSRNOG00000051291	Dnhd1	25.42793907	0.183553421	0.283786517
ENSRNOG00000001813	Dnm1l	40.9125785	0.361365238	0.217965887
ENSRNOG00000007649	Dnm2	36.37233391	0.143319455	0.244293735
ENSRNOG00000026490	Dnm3	68.75330539	-0.192713111	0.188328578
ENSRNOG00000050742	Dnmbp	24.81112982	-0.165727663	0.25497289
ENSRNOG00000039859	Dnmt1	96.27147599	0.088260152	0.143806272
ENSRNOG00000026649	Dnmt3a	72.70034667	-0.129833734	0.203713186
ENSRNOG00000014933	Dnttip1	29.28309375	-0.200207586	0.295048788
ENSRNOG00000018683	Dock1	72.20199231	-0.166677193	0.167472803
ENSRNOG00000053200	Dock10	60.15322645	0.097764601	0.204694515
ENSRNOG00000013321	Dock11	20.37027995	0.00209897	0.264299802
ENSRNOG00000014576	Dock3	81.92737203	0.082930624	0.185350376
ENSRNOG00000004823	Dock4	40.62353027	0.385243888	0.262485668
ENSRNOG00000008298	Dock7	23.27649283	0.038495662	0.295595658
ENSRNOG00000013922	Dok2	9.99255608	0.44250131	0.430546899
ENSRNOG00000015679	Dok4	11.0167064	-0.342695629	0.44700557
ENSRNOG00000013196	Dok5	154.662678	0.066443908	0.129208257
ENSRNOG00000038190	Dok6	9.568120308	0.521482037	0.426177632
ENSRNOG00000022419	Dok7	179.6822119	0.017204165	0.105099096
ENSRNOG00000017663	Dolpp1	33.82306694	-1.084262945	1.135469374
ENSRNOG00000022847	Dop1a	79.8914335	-0.089383695	0.153315454
ENSRNOG00000051204	Dop1b	142.1521001	0.06893954	0.144811968
ENSRNOG00000032546	Dot1l	18.00751453	-0.373537407	0.365879937
ENSRNOG00000017241	Dpcd	24.5351185	-0.22537083	0.259714031
ENSRNOG00000020687	Dpf1	39.21112478	0.257334025	0.233426254
ENSRNOG00000020892	Dpf2	10.8394302	0.140144155	0.433070737
ENSRNOG00000019735	Dph2	9.928719699	-0.466674349	0.371466743
ENSRNOG00000013719	Dph5	39.24894645	0.001041592	0.204819099
ENSRNOG00000037356	Dph6	14.68337666	-0.277741938	0.360604455
ENSRNOG00000008102	Dph7	74.98012983	-0.114897747	0.166725759
ENSRNOG00000010993	Dpm1	75.01362844	0.167088778	0.164596779
ENSRNOG00000029077	Dpm3	10.73061771	-0.468942155	0.416778583
ENSRNOG00000002595	Dpp10	32.42240428	-0.102859789	0.238216086
ENSRNOG00000031485	Dpp3	21.63752367	-0.500073953	0.331861147
ENSRNOG00000030763	Dpp4	11.3049577	-0.268046324	0.354926534
ENSRNOG00000012640	Dpp7	10.11657801	0.039709993	0.440433275
ENSRNOG00000019105	Dpp8	56.79788992	0.257091619	0.167522055
ENSRNOG00000050748	Dpp9	17.03149062	0.218576745	0.313042756
ENSRNOG00000021573	Dpy19l3	186.9622212	0.189363434	0.119524224
ENSRNOG00000025416	Dpy19l4	15.22124356	0.076603229	0.341917157
ENSRNOG00000017105	Dpyd	11.25878444	0.107884336	0.368384076
ENSRNOG00000009625	Dpysl2	185.2980882	-0.026585802	0.177462181
ENSRNOG00000018992	Dpysl3	34.35763446	-0.071103791	0.259136606
ENSRNOG00000027582	Dpysl4	8.762880615	-0.245224973	0.416680661
ENSRNOG00000017744	Dram2	32.84806134	0.298540717	0.221865949
ENSRNOG00000021303	Drc3	70.76257567	-0.165779395	0.14938969
ENSRNOG00000008428	Drd2	49.05326499	-0.478343564	0.185052586
ENSRNOG00000055309	Drg2	34.84068345	0.150674481	0.228756744
ENSRNOG00000026704	Drp2	37.46842397	0.014961908	0.233771151
ENSRNOG00000027992	Dscam	67.06689141	0.112623082	0.172185177

ENSRNOG00000016502	Dscaml1	60.24556421	0.061696448	0.222697892
ENSRNOG00000000824	Dse	16.15228373	0.106630588	0.318058687
ENSRNOG00000032307	Dsel	52.32590792	-0.008049306	0.202460295
ENSRNOG00000006236	Dsn1	68.42106579	0.476959202	0.16313701
ENSRNOG00000005924	Dstn	75.12517083	0.269098515	0.161099521
ENSRNOG00000006727	Dtd2	44.41766707	-0.445971468	0.191899731
ENSRNOG00000004195	Dtl	21.04961344	-0.274325767	0.289191917
ENSRNOG00000016671	Dtna	28.73968441	0.360166639	0.278196993
ENSRNOG00000048719	Dtnbp1	123.2935643	0.10307897	0.129820745
ENSRNOG00000009593	Dtwd1	28.88890797	0.282309893	0.253865568
ENSRNOG00000050848	Dtx1	57.52599361	-0.114778699	0.185972121
ENSRNOG00000001432	Dtx2	40.45975046	0.151013741	0.212607548
ENSRNOG00000005129	Dtx3	20.12734961	0.053455555	0.388646425
ENSRNOG00000023400	Dtx3l	14.11047598	0.562883225	0.34407462
ENSRNOG00000021086	Dtx4	139.7925701	-0.006977505	0.125025528
ENSRNOG00000018904	Dtymk	152.9393493	0.033253904	0.118171739
ENSRNOG00000047905	Dus1l	24.76957055	0.036144998	0.436781949
ENSRNOG00000019819	Dus2	71.90043114	0.055602896	0.162707999
ENSRNOG00000008155	Dus4l	18.53699056	0.094759633	0.322399015
ENSRNOG00000004003	Dusp10	32.99303552	-0.330557551	0.220368065
ENSRNOG00000045539	Dusp11	38.27953382	-0.340561051	0.208973122
ENSRNOG00000003100	Dusp12	191.3991961	-0.18145401	0.108703719
ENSRNOG00000046368	Dusp14l1	36.68296261	-0.010047167	0.222064998
ENSRNOG00000008534	Dusp15	130.6992282	-0.15352683	0.12601517
ENSRNOG00000006628	Dusp16	13.75455481	0.070414554	0.392572692
ENSRNOG00000024945	Dusp18	137.497317	0.050783717	0.137179087
ENSRNOG00000008868	Dusp19	17.47097834	0.304262549	0.377675123
ENSRNOG00000056376	Dusp22	94.07202978	0.145626083	0.148154304
ENSRNOG00000011921	Dusp4	30.00882498	-0.301485876	0.287231123
ENSRNOG00000014061	Dusp5	31.67519335	0.029591348	0.333134318
ENSRNOG00000010789	Dusp7	88.24642864	-0.540656028	0.204580613
ENSRNOG00000029394	Dusp8	15.18040039	0.069314668	0.32981057
ENSRNOG00000055185	Dusp9	10.16993794	-0.236200921	0.439386128
ENSRNOG00000017915	Dvl2	11.77823228	-0.298677845	0.368924061
ENSRNOG00000001708	Dvl3	58.42113611	0.462329757	0.269395547
ENSRNOG00000006178	Dync1h1	99.30661805	-0.289716679	0.209766465
ENSRNOG00000032070	Dync2h1	66.35830282	0.067588637	0.170860476
ENSRNOG00000004520	Dync2i1	49.46370624	-0.163109254	0.225268561
ENSRNOG00000015636	Dync2i2	9.468934565	-0.127850683	0.398981599
ENSRNOG00000005151	Dync2li1	43.3664365	-0.042169212	0.219631508
ENSRNOG00000011222	Dynll1	70.77636677	-0.157990253	0.1513202
ENSRNOG00000001758	Dynlt2b	38.50273799	-0.093558865	0.222617683
ENSRNOG00000023100	Dynlt5	11.39889971	0.367204254	0.413414766
ENSRNOG00000001662	Dyrk1a	67.11127358	0.295660516	0.172508429
ENSRNOG00000019254	Dyrk1b	88.26745962	-0.047285034	0.143951763
ENSRNOG00000004870	Dyrk3	88.11107403	-0.08161953	0.177321751
ENSRNOG00000007440	Dzank1	30.09013363	-0.170421829	0.256335386
ENSRNOG00000010311	Dzip1	110.1698432	0.100006274	0.153541827
ENSRNOG00000014746	Dzip1l	58.35697131	-0.094341439	0.164701522
ENSRNOG00000001956	Dzip3	119.0744711	-0.170091438	0.13649393

ENSRNOG00000024567	E130309D02Rik	53.13157211	0.078034202	0.174181776
ENSRNOG00000047741	E2f2	9.956826245	0.465044655	0.480863109
ENSRNOG00000029273	E2f3	55.46101639	-0.452698231	0.237925953
ENSRNOG00000015708	E2f4	20.80626378	-0.11241327	0.350046663
ENSRNOG00000010760	E2f5	8.778860836	0.017136977	0.575328304
ENSRNOG00000004449	E2f6	127.7160334	0.284729558	0.125312935
ENSRNOG00000009224	E4f1	19.98191258	0.183645411	0.33496099
ENSRNOG00000019559	Eaf1	51.13083416	-0.139409654	0.208341556
ENSRNOG00000002350	Eaf2	10.00078671	-0.505431139	0.389014457
ENSRNOG00000004509	Eapp	86.29595882	0.190844919	0.226197253
ENSRNOG00000025353	Ears2	17.31803658	-0.367684035	0.305463346
ENSRNOG00000028845	Ebf1	11.59616718	0.196793426	0.419756377
ENSRNOG00000007408	Ebf4	13.4384917	-0.118154638	0.356358438
ENSRNOG00000045760	Ebna1bp2	135.2884162	-0.00647619	0.118632454
ENSRNOG00000004903	Ebp	34.23725881	0.315898039	0.225033004
ENSRNOG00000014659	Ebpl	165.4101591	-0.111107887	0.119947078
ENSRNOG00000042253	Ecd	19.44824384	0.441278346	0.321397371
ENSRNOG00000014241	Ece1	144.7103016	0.06934237	0.114970145
ENSRNOG00000001715	Ece2	11.96633776	0.542299404	0.410714593
ENSRNOG00000019447	Ecel1	13.49680525	-0.672111453	0.384141931
ENSRNOG00000020308	Ech1	51.4774458	0.051024551	0.191009105
ENSRNOG00000008843	Eci1	12.80500672	0.283653328	0.349533987
ENSRNOG00000029549	Eci3	133.8834417	0.10178307	0.108410617
ENSRNOG00000021166	Ecm1	29.5984078	0.143870724	0.241803252
ENSRNOG00000031716	Ecm2	13.83065793	-0.292939276	0.359849773
ENSRNOG00000025076	Ecpas	181.6326163	0.046520927	0.124479389
ENSRNOG00000023576	Ecrg4	24.19311154	0.304560421	0.368232702
ENSRNOG00000014128	Ecsit	28.04437759	-0.224138292	0.24681265
ENSRNOG00000055365	Edem1	17.08410028	0.403312576	0.300245026
ENSRNOG00000019299	Edem2	101.6910474	-0.117520718	0.128421285
ENSRNOG00000028358	Edem3	105.1789582	-0.039997203	0.142102647
ENSRNOG00000016272	Edf1	40.33481219	0.091775781	0.211885377
ENSRNOG00000012721	Ednra	14.44815574	-0.034020413	0.370344688
ENSRNOG00000010997	Ednrb	49.49342965	0.2212252	0.198567307
ENSRNOG00000017728	Edrf1	11.82248078	-0.208280566	0.367003729
ENSRNOG00000021681	Eea1	119.6548848	-0.141694192	0.128527805
ENSRNOG00000017509	Eed	70.91528207	0.065379064	0.156984662
ENSRNOG00000011624	Eef1a2	29.22040675	0.074829332	0.241401058
ENSRNOG00000009849	Eef1akmt1	179.3888969	-0.10214613	0.104133988
ENSRNOG00000017137	Eef1akmt2	50.47134606	-0.061011611	0.214231998
ENSRNOG00000003042	Eef1aknmt	58.79588135	-0.227358616	0.158215673
ENSRNOG00000020266	Eef2	29.06798916	0.421989437	0.316795829
ENSRNOG00000016448	Eef2k	119.0497097	-0.07731807	0.141866206
ENSRNOG00000012954	Eefsec	172.8625625	-0.024419996	0.119458633
ENSRNOG00000006931	Eepd1	92.96209333	0.206943077	0.142496751
ENSRNOG00000010730	Efcab10	11.15973013	-0.22903827	0.402387075
ENSRNOG00000026176	Efcab12	28.2654099	0.258688743	0.248865776
ENSRNOG00000010091	Efcab14	62.77664069	0.13061967	0.182374933
ENSRNOG00000003266	Efcab15	9.379702481	-0.278271742	0.397442532
ENSRNOG00000042201	Efcab2	21.21119752	-0.084314476	0.313483004

ENSRNOG00000011094	Efcab6	18.96714153	0.982177116	0.33728115
ENSRNOG00000009782	Efcab7	10.21217391	-0.523314289	0.385642737
ENSRNOG00000003553	Efemp1	51.59086147	-0.099196981	0.184467965
ENSRNOG00000020587	Efemp2	33.26733219	0.542543896	0.249728708
ENSRNOG00000042729	Efhc1	9.21635865	0.127970526	0.478438428
ENSRNOG00000015596	Efhd1	134.5566727	0.219132836	0.13172562
ENSRNOG00000013783	Efhd2	54.33451258	0.006560927	0.177382412
ENSRNOG00000032723	Efl1	29.56094836	-0.449441901	0.282512205
ENSRNOG00000016203	Efna2	16.09934464	0.121659399	0.336747261
ENSRNOG00000057322	Efna3	21.39047924	-0.283565115	0.297308898
ENSRNOG00000006877	Efnb1	70.46676761	0.069896868	0.200508803
ENSRNOG00000025528	Efr3a	28.34264187	0.079504361	0.245336941
ENSRNOG00000012950	Efr3b	54.27674483	0.303513573	0.211101769
ENSRNOG00000002818	Eftud2	16.26654435	-0.018421836	0.310772739
ENSRNOG00000004275	Egfl6	12.86348801	0.047943064	0.410100898
ENSRNOG00000012058	Egflam	33.31084214	0.197826145	0.237898918
ENSRNOG00000020947	Egln2	7.922037029	0.458272983	0.505612178
ENSRNOG00000005053	Egln3	177.8321507	0.042484377	0.113111973
ENSRNOG00000008917	Ehbp1	161.7243234	-0.001397963	0.11620667
ENSRNOG00000056038	Ehbp1l1	29.59383008	0.05736249	0.254639846
ENSRNOG00000007744	Ehd3	125.5778327	-0.130651852	0.119168381
ENSRNOG00000007584	Ehd4	55.67848681	0.102390387	0.206073851
ENSRNOG00000007242	Ehmt1	159.0497372	-0.045193597	0.139099561
ENSRNOG00000030630	Ehmt2	35.99651056	0.088420563	0.260786171
ENSRNOG00000008452	Eid1	118.1608393	-0.26152527	0.196607114
ENSRNOG00000031421	Eif1a	84.82496459	-0.114153673	0.155041525
ENSRNOG00000048315	Eif2ak2	40.65353422	0.011476343	0.211642089
ENSRNOG00000006069	Eif2ak3	47.5639744	0.096561394	0.206007438
ENSRNOG00000006027	Eif2ak4	27.81153253	-0.336244082	0.266111156
ENSRNOG00000006467	Eif2b2	191.0173724	-0.026446738	0.101459007
ENSRNOG00000018375	Eif2b3	81.94175967	-0.029153644	0.143805859
ENSRNOG00000005301	Eif2b4	139.4357113	0.038585782	0.128052828
ENSRNOG00000038160	Eif2b5	148.281942	0.019281577	0.117412006
ENSRNOG00000004910	Eif2d	47.31318216	0.050056208	0.191420957
ENSRNOG00000009432	Eif2s1	16.61365577	-0.137431546	0.353185217
ENSRNOG00000017447	Eif2s2	123.9413617	-0.180296017	0.132864458
ENSRNOG00000005804	Eif3d	73.829287	0.447664879	0.262232423
ENSRNOG00000020619	Eif3g	18.76674858	0.287495935	0.319496303
ENSRNOG00000016459	Eif3j	79.33950509	0.300320423	0.172331092
ENSRNOG00000012738	Eif3m	119.2081419	0.085877966	0.124540562
ENSRNOG00000001815	Eif4a2	158.4315355	-0.700192659	1.024629994
ENSRNOG00000045791	Eif4a3	24.92272515	-0.2418141	0.269382276
ENSRNOG00000019634	Eif4e2	67.83146379	0.189702937	0.160645053
ENSRNOG00000010301	Eif4e3	86.31069671	0.091560884	0.189523187
ENSRNOG00000012582	Eif4ebp1	120.3802266	-0.008010755	0.16019689
ENSRNOG00000055391	Eif4ebp2	68.29715417	0.092083991	0.178195483
ENSRNOG00000030247	Eif4ebp3	177.6939672	0.043680675	0.103088253
ENSRNOG00000001738	Eif4g1	24.35513588	0.304099487	0.3265264
ENSRNOG00000011859	Eif5a2	182.5619988	0.090631903	0.133540756
ENSRNOG00000049497	Eif6	43.10918671	0.229671318	0.223395919

ENSRNOG00000000113	Elac1	13.68980665	-0.357756376	0.342953518
ENSRNOG00000020272	Elapor1	41.1878652	0.206870643	0.206314922
ENSRNOG00000005818	Elapor2	25.81960821	0.670641506	0.262798121
ENSRNOG00000013752	Elavl3	16.10622527	0.326673402	0.339096044
ENSRNOG00000011762	Elf1	10.61948753	0.743717731	0.457895678
ENSRNOG00000010815	Elf2	13.42475709	-0.348656099	0.335553049
ENSRNOG00000022767	Elfn1	130.8334705	0.066854499	0.118508801
ENSRNOG00000007934	Elfn2	31.64211676	-0.062233541	0.22831585
ENSRNOG00000004367	Elk3	53.07900956	0.160888402	0.171147238
ENSRNOG00000027089	Ell2	40.78819198	-0.062446405	0.227057566
ENSRNOG00000018747	Elmo2	15.11084463	0.481235458	0.344786392
ENSRNOG00000028176	Elmod2	8.338964198	0.052319917	0.455376543
ENSRNOG00000001469	Eln	86.59830492	0.341752497	0.205660303
ENSRNOG00000010902	Eloa	48.88539375	0.086173885	0.195155541
ENSRNOG00000014284	Elof1	12.74071407	-0.181370501	0.347000096
ENSRNOG00000014702	Elovl2	113.627343	0.093393803	0.154227833
ENSRNOG00000048949	Elovl6	102.336408	0.086847735	0.134118907
ENSRNOG00000016725	Elp1	76.75913656	-0.262293551	0.149312816
ENSRNOG00000027628	Elp5	10.76773745	0.581085501	0.415792754
ENSRNOG00000020847	Elp6	70.93972049	0.057420759	0.164055475
ENSRNOG00000060329	Emb	21.15646615	-0.137445717	0.282173831
ENSRNOG00000018097	Emc1	41.97805206	-0.152101637	0.20125024
ENSRNOG00000005123	Emc2	81.96067845	-0.092233492	0.155395269
ENSRNOG00000005884	Emc7	19.86657506	-0.217735105	0.320394016
ENSRNOG00000017654	Emc8	87.27206064	0.077279508	0.159085304
ENSRNOG00000019162	Emc9	65.70216082	-0.078042127	0.177734359
ENSRNOG00000022910	Emcn	27.19548597	0.289186848	0.268725171
ENSRNOG00000012828	Emg1	14.65921655	0.66968434	0.366156278
ENSRNOG00000033575	Emid1	15.05345685	0.900525659	0.393127038
ENSRNOG00000043143	Eml1	11.15868847	0.883626953	0.479729315
ENSRNOG00000030294	Eml4	138.8306796	0.179072687	0.112903672
ENSRNOG00000004207	Eml5	53.50059707	0.020439909	0.177399916
ENSRNOG00000008676	Emp1	23.99352422	0.132400494	0.277220353
ENSRNOG00000002664	Emp2	16.7586796	-0.159745598	0.311786179
ENSRNOG00000015560	Emsy	57.19860553	0.210071851	0.174987057
ENSRNOG00000015493	Emx1	164.8708786	-0.067282632	0.140393128
ENSRNOG00000009482	Emx2	27.89914254	0.494787237	0.321906924
ENSRNOG00000031934	Enah	60.60486964	-0.041258594	0.171939227
ENSRNOG00000024757	Endod1	14.61890941	-0.236557482	0.31355338
ENSRNOG00000003699	Endov	47.97845436	-0.218247289	0.199800025
ENSRNOG00000050190	Eng	8.748203903	0.025932939	0.456768994
ENSRNOG00000018631	Enkur	83.64323305	-0.198893829	0.154307076
ENSRNOG00000004078	Eno3	8.991299812	-0.163911371	0.431381224
ENSRNOG00000018310	Eno4	159.1272284	-0.00257636	0.108385684
ENSRNOG00000047817	Enox1	117.7764568	0.13024606	0.132764429
ENSRNOG00000030622	Enox2	9.965932383	-0.077698037	0.481233851
ENSRNOG00000051854	Enpep	9.138389108	1.079715403	0.443232753
ENSRNOG00000013994	Enpp1	15.28096264	0.16638229	0.362981368
ENSRNOG00000004089	Enpp2	164.0162115	0.003792104	0.116303029
ENSRNOG00000010174	Enpp4	81.09033201	0.094153133	0.1518949

ENSRNOG00000009660	Enpp6	14.6117118	0.336697106	0.352257257
ENSRNOG00000025344	Enthd1	14.51065136	-0.393999399	0.435935923
ENSRNOG00000014574	Entpd1	69.05424275	0.248332137	0.184661851
ENSRNOG00000018982	Entpd3	55.41517618	0.344327624	0.17562735
ENSRNOG00000033206	Entpd5	11.7867554	-0.070274703	0.385730657
ENSRNOG00000016883	Entpd7	17.62943463	-0.294398703	0.32638431
ENSRNOG00000004681	Eny2	66.73359266	-0.051895443	0.159033786
ENSRNOG00000024533	Eogt	161.1055159	0.016636399	0.137968551
ENSRNOG00000000190	Ep300	19.9193055	0.08704838	0.316783577
ENSRNOG00000037483	Ep400	149.718973	0.207954011	0.11510719
ENSRNOG00000010037	Epb41	35.56644493	0.062667142	0.232996854
ENSRNOG00000012346	Epb41l2	50.4781106	0.260875856	0.193359316
ENSRNOG00000056550	Epb41l4b	18.09209623	0.241279385	0.324787061
ENSRNOG00000014834	Epc1	12.1034428	0.721501234	0.379217721
ENSRNOG00000029447	Epc2	139.6578177	0.157307983	0.122027124
ENSRNOG00000052894	Epg5	14.10675958	-0.129142419	0.384113816
ENSRNOG00000037340	Epha10	13.20155822	0.167916056	0.438304057
ENSRNOG00000029184	Epha6	8.379164322	0.446583192	0.481328589
ENSRNOG00000007030	Epha7	98.58854899	-0.203098262	0.135596921
ENSRNOG00000007865	Ephb1	127.69681	0.094685401	0.144966908
ENSRNOG00000012531	Ephb2	44.48573041	0.246086422	0.236023789
ENSRNOG00000031801	Ephb3	101.941722	0.182306303	0.167777365
ENSRNOG00000014367	Ephb6	15.34491256	-0.316379859	0.333396557
ENSRNOG00000003515	Ephx1	72.25726444	-0.091443181	0.151705617
ENSRNOG00000017286	Ephx2	51.98219277	-0.113536761	0.211712095
ENSRNOG00000023389	Ephx4	129.912351	0.128736019	0.142070642
ENSRNOG00000060550	Epn2	54.71254429	-0.017416896	0.20515166
ENSRNOG00000001412	Epo	25.22057842	0.451603064	0.305126299
ENSRNOG00000048187	Epop	14.80576632	0.204326527	0.364318177
ENSRNOG00000028908	Eppin	60.12718	-0.101093961	0.172385489
ENSRNOG00000002393	Eprs	179.0619628	0.14395239	0.109712198
ENSRNOG00000010299	Eps15	40.54520432	0.126766313	0.199696997
ENSRNOG00000013502	Eps15l1	158.1503947	0.102205215	0.121210785
ENSRNOG00000007047	Eps8	188.6044413	-0.024358935	0.116766269
ENSRNOG00000018336	Eps8l2	34.99392081	0.267688058	0.219882116
ENSRNOG00000009471	Epsti1	16.27314574	0.126902508	0.314953309
ENSRNOG00000009997	Erap1	15.71470937	0.031540028	0.318173997
ENSRNOG00000042470	Eras	161.3278004	0.096561756	0.12036834
ENSRNOG00000047137	Erbin	115.8231508	0.251898759	0.142857474
ENSRNOG00000009264	Erc1	127.3178659	0.299932615	0.146147729
ENSRNOG00000015148	Erc2	53.03599328	-0.06349312	0.216562182
ENSRNOG00000017839	Ercc1	37.25486929	-0.097402878	0.217700291
ENSRNOG00000017753	Ercc2	18.78024894	0.085605359	0.302057012
ENSRNOG00000022812	Ercc5	92.32016755	0.156966304	0.141104557
ENSRNOG00000030017	Ercc6	59.7433671	-0.101250102	0.174926693
ENSRNOG00000019175	Ercc6l2	54.35200523	-0.14011985	0.179192142
ENSRNOG00000009968	Ercc8	151.4347872	-0.021047901	0.105070525
ENSRNOG00000020426	Erf	169.5565994	-0.040228587	0.184717641
ENSRNOG00000001652	Erg	15.64352988	0.297994637	0.334806326
ENSRNOG00000003508	Ergic1	145.5492656	-0.301671704	0.131915683

ENSRNOG00000001852	Ergic2	77.73434668	-0.048996537	0.141742983
ENSRNOG00000031085	Ergic3	34.7645834	-0.085759766	0.27497226
ENSRNOG00000011448	Eri1	41.71321458	0.105892148	0.207674966
ENSRNOG00000019381	Eri3	72.46826426	-0.228731841	0.152313117
ENSRNOG00000042114	Erich1	61.54026764	0.052078583	0.177704597
ENSRNOG00000037798	Erich4	22.61888997	-0.019336487	0.289881308
ENSRNOG00000005956	Erich5	50.75504379	0.072046983	0.185494258
ENSRNOG00000012911	Erlin1	95.28172731	-0.109772958	0.151392903
ENSRNOG00000015230	Ermard	59.98372504	0.330407209	0.195267986
ENSRNOG00000021472	Ermn	17.75204276	0.24152616	0.338683315
ENSRNOG00000012864	Ern1	37.97006959	-0.106738081	0.218751069
ENSRNOG00000002609	Ero1b	43.80971046	-0.124362071	0.221383664
ENSRNOG00000001348	Erp29	61.96813885	0.162751617	0.209534223
ENSRNOG00000005841	Erp44	143.0893563	0.062704769	0.117597792
ENSRNOG00000058186	Errfi1	136.8834413	-0.068329015	0.129364672
ENSRNOG00000033217	Esam	40.80793345	0.262428	0.211812546
ENSRNOG00000014031	Esco1	67.48022553	-0.176068499	0.16902798
ENSRNOG00000004777	Esf1	84.06641352	-0.2954268	0.158886824
ENSRNOG00000010270	Espn	20.39952608	0.553109332	0.401648142
ENSRNOG00000002593	Esrrg	51.65226021	0.03548446	0.20695003
ENSRNOG00000060753	Esyt1	111.1179559	-0.161992234	0.193589958
ENSRNOG00000032391	Esyt2	59.27111219	-0.022867209	0.178346861
ENSRNOG00000022704	Esyt3	23.23650223	-0.018657427	0.299967295
ENSRNOG00000006185	Etaa1l1	22.45075136	-0.079396118	0.271188177
ENSRNOG00000019450	Etf1	131.5212496	-0.062484657	0.11873371
ENSRNOG00000017851	Etfb	189.9796588	-0.191514194	0.103622146
ENSRNOG00000036918	Etfbkmt	10.011869	0.014114535	0.444567204
ENSRNOG00000019982	Ethe1	23.27801103	-0.289410687	0.265641429
ENSRNOG00000045743	Etnppl	32.91806446	0.020162995	0.293517695
ENSRNOG00000008941	Ets1	34.4980466	0.477449305	0.223461287
ENSRNOG00000043095	Etv3	34.61694955	-0.463894107	0.22317898
ENSRNOG00000020792	Etv4	29.29924159	0.135146609	0.239487153
ENSRNOG00000005984	Etv6	22.13766793	-1.065229269	0.585415914
ENSRNOG00000007118	Eva1a	45.59740277	0.383260057	0.216237169
ENSRNOG00000002072	Eva1c	11.66999274	0.051622739	0.410693459
ENSRNOG00000007564	Evc	16.08022567	0.133927814	0.346290274
ENSRNOG00000022764	Evi2a	87.76246597	-0.055190496	0.156182843
ENSRNOG00000001034	Evi5l	43.30578577	0.453124595	0.234454712
ENSRNOG00000014476	Evl	116.0292844	-0.083473161	0.129015256
ENSRNOG00000009343	Evpl	9.872564256	-0.66544877	0.426454783
ENSRNOG00000009437	Ewsr1	59.05710145	0.085282768	0.194558004
ENSRNOG00000037432	Exo5	26.44255417	0.08273193	0.247555288
ENSRNOG00000002144	Exoc1	188.9490337	0.118593977	0.115425363
ENSRNOG00000039776	Exoc3	31.02164662	-0.090966205	0.219917109
ENSRNOG00000017448	Exoc3l2	34.02751848	-0.144260195	0.261016204
ENSRNOG00000046023	Exoc4	22.24309904	0.180524503	0.290874221
ENSRNOG00000036601	Exoc6	16.87930895	0.150545871	0.301051724
ENSRNOG00000059615	Exoc6b	191.2701358	0.042231851	0.093864041
ENSRNOG00000019766	Exoc8	8.316625536	0.019612995	0.460123859
ENSRNOG00000014801	Exog	43.1345171	-0.420404468	0.203736378

ENSRNOG00000048708	Exosc1	32.98956302	0.116372771	0.281423144
ENSRNOG00000010719	Exosc10	154.5401871	0.022493411	0.123390098
ENSRNOG00000009245	Exosc2	26.04258374	-0.019861872	0.243115491
ENSRNOG00000012348	Exosc4	9.402430417	-0.49262545	0.45398146
ENSRNOG00000020635	Exosc5	86.7570355	-0.405555794	0.134204756
ENSRNOG00000060337	Exosc7	69.58338008	0.093887158	0.190401439
ENSRNOG00000014674	Exosc9	9.30153914	0.138162015	0.433229165
ENSRNOG00000025115	Exph5	7.943096652	-0.371107463	0.492178028
ENSRNOG00000008944	Ext2	145.1863136	0.075198736	0.11870504
ENSRNOG00000016776	Extl1	14.36624291	0.046525816	0.324829942
ENSRNOG00000014323	Extl2	79.75176004	-0.161148647	0.160557831
ENSRNOG00000013581	Extl3	12.38720838	0.156707537	0.374480086
ENSRNOG00000007590	Eya1	8.991463808	0.093364948	0.437161643
ENSRNOG00000010396	Eya3	27.5948524	0.101020288	0.26746818
ENSRNOG00000020336	Ezh1	57.52511621	-0.063154439	0.188297995
ENSRNOG00000006048	Ezh2	32.48161242	0.250776694	0.228567167
ENSRNOG00000004414	F11r	53.82798264	0.015886782	0.186720281
ENSRNOG00000048043	F2r	124.9308543	-0.161977994	0.119530607
ENSRNOG00000011800	F3	113.8176733	0.158849517	0.138194223
ENSRNOG00000031197	F8	27.47826635	0.279354891	0.273752097
ENSRNOG00000018950	Fa2h	121.3666718	-0.19143651	0.161108221
ENSRNOG00000012879	Fabp3	15.90084396	0.064164091	0.321183288
ENSRNOG00000000814	Fabp7	101.5167068	0.032370435	0.124652631
ENSRNOG00000047035	Fadd	12.15876202	0.495369124	0.368863689
ENSRNOG00000020480	Fads1	16.90828703	0.208578093	0.344005998
ENSRNOG00000020440	Fads2	66.73982099	-0.031629182	0.157325928
ENSRNOG00000020385	Fads3	188.5997409	-0.055516662	0.108041884
ENSRNOG00000008523	Faf1	143.0907746	0.078134942	0.119621595
ENSRNOG00000017607	Faf2	171.7045042	0.005175972	0.108511031
ENSRNOG00000013223	Fah	129.1358577	-0.068464592	0.121704534
ENSRNOG00000014727	Fahd1	84.41152274	-0.092291726	0.14707428
ENSRNOG00000030069	Faim	66.51912234	0.059853823	0.187770008
ENSRNOG00000045554	Faim2	90.97867841	-0.044751198	0.143540306
ENSRNOG00000050834	Fam102a	23.48905026	1.074191083	0.584486176
ENSRNOG00000002851	Fam104a	35.86989217	0.476326344	0.552525655
ENSRNOG00000014886	Fam107b	52.18345559	0.017110703	0.182812767
ENSRNOG00000012067	Fam111a	49.11507435	1.348035053	1.089739449
ENSRNOG00000011543	Fam118b	184.5605064	-0.028850482	0.099628437
ENSRNOG00000016779	Fam120a	45.39972009	-0.050127151	0.210602794
ENSRNOG00000062231	FAM120C	59.2088637	-0.072335079	0.190751471
ENSRNOG00000002285	Fam122b	12.79150459	0.120594006	0.393052196
ENSRNOG00000009802	Fam124a	141.5939529	0.100431367	0.121150683
ENSRNOG00000017149	Fam131b	12.62920201	-0.507312713	0.334500416
ENSRNOG00000009163	Fam133b	44.03293359	0.133135091	0.209707145
ENSRNOG00000013587	Fam135a	38.09803798	0.345389631	0.211632928
ENSRNOG00000005159	Fam135b	11.22577999	0.033576149	0.504343645
ENSRNOG00000007947	Fam13a	16.89290305	0.28246699	0.317361659
ENSRNOG00000020384	Fam13b	19.90821303	0.380368902	0.302072015
ENSRNOG00000021693	Fam149a	29.74879334	0.264058142	0.223426346
ENSRNOG00000006554	Fam149b1	41.28605395	0.291846116	0.212457538

ENSRNOG00000007799	Fam151a	21.14954938	0.003337421	0.309336748
ENSRNOG00000024136	Fam151b	113.7956503	0.081629782	0.135189541
ENSRNOG00000038066	Fam155b	51.89123396	0.187688098	0.184359055
ENSRNOG00000009881	Fam161a	11.94989294	-0.289971896	0.359369986
ENSRNOG00000011112	Fam161b	16.03435095	-0.126539626	0.33432226
ENSRNOG00000002255	Fam162a	51.47015818	-0.052568639	0.181978726
ENSRNOG00000024825	Fam163a	131.0359029	0.352862938	0.131396023
ENSRNOG00000018873	Fam168a	48.79634225	-0.146903277	0.193598581
ENSRNOG00000016505	Fam169a	50.69960889	-0.023707744	0.173147005
ENSRNOG00000022822	Fam169b	12.37298342	0.661369172	0.428952975
ENSRNOG00000021041	Fam171a2	14.24435522	-0.523526905	0.334565929
ENSRNOG00000004783	Fam171b	135.4471731	0.146064739	0.135502244
ENSRNOG00000011750	Fam180a	10.50452534	0.479217962	0.506653231
ENSRNOG00000010763	Fam181b	42.50740113	0.001501994	0.187635629
ENSRNOG00000002873	Fam183a	16.89582469	0.627782658	0.352117977
ENSRNOG00000026407	Fam184a	23.02060849	-0.017680424	0.278072833
ENSRNOG00000003704	Fam184b	8.017068633	1.042009018	0.496667192
ENSRNOG00000023496	Fam189a1	20.91536876	-0.109609675	0.294007347
ENSRNOG00000061147	Fam189a2	12.35440438	-0.843569897	0.334148986
ENSRNOG00000013832	Fam193a	127.4498638	-0.061142023	0.130189296
ENSRNOG00000039807	Fam193b	57.17513115	-0.022962731	0.193418709
ENSRNOG00000009830	Fam204a	52.41095175	0.145256332	0.201275647
ENSRNOG00000001225	Fam207a	163.9890266	-0.080543129	0.111013683
ENSRNOG00000004337	Fam20b	35.32933715	0.41030598	0.262242572
ENSRNOG00000058522	Fam214a	89.7489065	-0.041835678	0.137120274
ENSRNOG00000031731	Fam216a	68.41567408	0.05375669	0.160688893
ENSRNOG00000016672	Fam217a	33.11757602	-0.158538511	0.231315071
ENSRNOG00000039559	Fam219a	12.07829872	0.007506377	0.372921061
ENSRNOG00000054468	Fam219b	89.29847128	0.073434265	0.135869047
ENSRNOG00000024605	Fam220a	153.3821386	-0.040074608	0.112803043
ENSRNOG00000009447	Fam221a	10.15571817	0.016820184	0.397405728
ENSRNOG00000029570	Fam222b	8.359577662	0.173002699	0.488415782
ENSRNOG00000021795	Fam227a	31.30490817	0.246741583	0.260980091
ENSRNOG00000024918	Fam229b	41.82858503	0.000243894	0.22212191
ENSRNOG00000008443	Fam234b	14.69750189	-0.331438514	0.344994036
ENSRNOG00000010875	Fam241a	13.31582868	-0.308818423	0.409773694
ENSRNOG00000060623	Fam3a	74.30854541	-0.122454741	0.149843142
ENSRNOG00000001728	Fam43a	97.3907883	-0.202127982	0.184894236
ENSRNOG00000058439	Fam50a	149.840863	-0.1927119	0.133045576
ENSRNOG00000017548	Fam53a	57.46869645	-0.151022247	0.201505596
ENSRNOG00000061348	Fam53b	65.81984412	0.052586722	0.176345995
ENSRNOG00000020261	Fam53c	16.16928886	0.405216423	0.33853128
ENSRNOG00000042747	Fam72a	15.43043977	-0.326838733	0.373468672
ENSRNOG00000011254	Fam76a	40.9450543	-0.033718917	0.262842753
ENSRNOG00000024863	Fam76b	48.91489317	0.220715507	0.20201827
ENSRNOG00000026845	Fam81b	19.98779038	-0.03358386	0.354379815
ENSRNOG00000019022	Fam89a	46.0167769	-0.132563869	0.21579816
ENSRNOG00000026120	Fam8a1	165.0981833	-0.056104844	0.396302135
ENSRNOG00000008271	Fam91a1	167.6164963	0.10448296	0.116220472
ENSRNOG00000030328	Fam98a	88.3187864	0.017288597	0.148880464

ENSRNOG00000024036	Fam98c	163.589686	0.027447559	0.140026117
ENSRNOG00000023985	Fan1	20.626995	0.154772118	0.29770102
ENSRNOG00000016706	Fanca	12.92354259	0.100185481	0.354840407
ENSRNOG00000016889	Fancc	22.07222043	0.384290455	0.274912581
ENSRNOG00000000504	Fance	21.94113515	-0.116945153	0.279390071
ENSRNOG00000057945	Fancg	19.51952492	-0.007621456	0.359585696
ENSRNOG00000016689	Fanci	86.30057529	0.028916482	0.164631956
ENSRNOG00000056721	Fancm	24.01265694	0.29628078	0.296474289
ENSRNOG00000018327	Fank1	19.20915328	-0.294204265	0.305420262
ENSRNOG00000013176	Far1	90.65795197	0.037184064	0.150075854
ENSRNOG00000001851	Far2	84.24501302	0.071967056	0.149213823
ENSRNOG00000018051	Farp2	19.74024099	0.026526769	0.292517251
ENSRNOG00000016135	Fars2	89.13489874	0.221704991	0.162487341
ENSRNOG00000003149	Farsa	28.00229917	0.097296764	0.261674001
ENSRNOG00000014119	Farsb	64.57140378	-0.047333238	0.16299012
ENSRNOG00000024335	Fastkd1	28.08544108	0.029458393	0.244308867
ENSRNOG00000023923	Fastkd2	44.66295207	0.286638334	0.209151644
ENSRNOG00000027422	Fastkd3	59.21310758	0.109476782	0.189275918
ENSRNOG00000030954	Fat1	73.39314955	0.387665218	0.161413812
ENSRNOG00000011585	Fat3	8.144914173	0.246787123	0.480746841
ENSRNOG00000028335	Fat4	11.18026264	0.627160527	0.390459085
ENSRNOG00000010106	Faxc	11.86288089	0.08397949	0.480069089
ENSRNOG00000008577	Fbf1	48.74736718	-0.364670876	0.224914642
ENSRNOG00000014137	Fbln1	128.2570245	-0.232838113	0.121054076
ENSRNOG00000007338	Fbln2	42.33182618	0.041842135	0.199601183
ENSRNOG00000050539	Fbln5	17.48234274	0.258208825	0.329461636
ENSRNOG00000007302	Fbn1	14.65451605	-0.194080827	0.313155618
ENSRNOG00000030721	Fbrsl1	73.02888689	0.443720775	0.154523598
ENSRNOG00000022248	Fbxl16	62.37339848	0.135913151	0.185884487
ENSRNOG00000013875	Fbxl17	18.40697915	-0.186914648	0.304657726
ENSRNOG00000001117	Fbxl18	75.86222273	-1.475920769	1.327466224
ENSRNOG00000005081	Fbxl20	122.0773077	0.258508663	0.150905776
ENSRNOG00000012168	Fbxl21	25.53472353	-0.367943635	0.26932285
ENSRNOG00000017684	Fbxl22	16.24121532	0.244190572	0.416952184
ENSRNOG00000059334	Fbxl3	125.7949999	-0.067557186	0.145876748
ENSRNOG00000005641	Fbxl4	88.00817273	-0.084229137	0.149421539
ENSRNOG00000005261	Fbxl5	25.64169756	-0.087546827	0.295132014
ENSRNOG00000025497	Fbxl6	80.17918964	0.081415822	0.153043122
ENSRNOG00000015242	Fbxl8	13.53120811	0.394288612	0.344653704
ENSRNOG00000016396	Fbxo11	31.89753944	0.339739768	0.287776548
ENSRNOG00000038225	Fbxo15	18.56629678	-0.153132693	0.30370907
ENSRNOG00000014003	Fbxo16	51.04990579	-0.307987861	0.181275975
ENSRNOG00000001129	Fbxo21	90.94527301	-0.415918589	0.132086268
ENSRNOG00000042464	Fbxo25	20.21431989	-0.263329695	0.34224784
ENSRNOG00000000066	Fbxo28	43.96671464	0.211489543	0.193665455
ENSRNOG00000014852	Fbxo30	14.68479189	-0.106155882	0.344738261
ENSRNOG00000019063	Fbxo38	106.9302372	-0.01344417	0.134896463
ENSRNOG00000015622	Fbxo4	20.81688502	0.127613914	0.312443796
ENSRNOG00000033202	Fbxo41	19.8752329	0.505143074	0.350062282
ENSRNOG00000014305	Fbxo42	162.9201894	0.348660281	0.114868098

ENSRNOG00000009298	Fbxo44	28.64039	-0.432762028	0.266205946
ENSRNOG00000008815	Fbxo46	45.08139093	0.091154934	0.189844451
ENSRNOG00000024077	Fbxo5	13.1304266	0.724527782	0.373329357
ENSRNOG00000010502	Fbxo8	106.0266573	0.14774382	0.135329599
ENSRNOG00000030027	Fbxw17	28.33198745	-0.161349164	0.234947939
ENSRNOG00000010889	Fbxw7	27.09652504	-0.112029527	0.279690591
ENSRNOG00000001005	Fcer2	183.7343552	-0.061350502	0.099044437
ENSRNOG00000004723	Fcf1	117.9367551	-0.151563298	0.156810772
ENSRNOG00000019129	Fcgbp	13.04634497	0.529284062	0.394265097
ENSRNOG00000021199	Fcgr1a	13.91031932	0.040511697	0.34557658
ENSRNOG00000049422	Fcgr2a	51.699001	-0.066238579	0.202115276
ENSRNOG00000046663	Fcgr2a	124.50975	-0.03496959	0.170163218
ENSRNOG00000046452	Fcgr2b	26.24376624	-0.119902318	0.240136318
ENSRNOG00000024382	Fcgr3a	11.67133928	-0.015268708	0.409941324
ENSRNOG00000033912	Fcho1	10.10378164	0.298360083	0.419321115
ENSRNOG00000015334	Fcho2	141.4812151	-0.064922065	0.11948256
ENSRNOG00000039415	Fchsd1	31.08104277	0.478647615	0.252912704
ENSRNOG00000019319	Fchsd2	139.892784	0.150838857	0.124536619
ENSRNOG00000059453	Fcsk	32.72241383	-0.048327301	0.235149645
ENSRNOG00000021314	Fdft1	93.55169629	0.345756881	0.196716946
ENSRNOG00000012123	Fdx1	99.64687215	-0.164678344	0.130639083
ENSRNOG00000023020	Fdx2	14.89401788	-0.320328634	0.305998466
ENSRNOG00000007077	Fem1b	67.10662409	0.006843604	0.161559837
ENSRNOG00000003578	Fem1c	76.68057135	0.212348561	0.14880328
ENSRNOG00000015898	Fer	22.97253408	-0.015058293	0.274584015
ENSRNOG00000024201	Fer1l5	13.62875605	-0.012819496	0.368340686
ENSRNOG00000000528	Fgd2	17.42977794	0.14122181	0.302836088
ENSRNOG00000016225	Fgd3	29.77077918	0.618498048	0.259179533
ENSRNOG00000059491	Fgd4	22.89670587	0.131798679	0.317106654
ENSRNOG00000010213	Fgd5	31.92031623	-0.067716206	0.259864511
ENSRNOG00000013867	Fgf1	17.84571219	0.003834192	0.290160552
ENSRNOG00000009288	Fgf14	12.57546655	0.181748939	0.365656687
ENSRNOG00000008960	Fgf22	10.61612668	0.269116773	0.407988393
ENSRNOG00000011471	Fgf9	68.92596488	-0.008372562	0.163306227
ENSRNOG00000022796	Fgfbp3	91.46892361	-0.368249735	0.162254633
ENSRNOG00000016374	Fgfr2	168.4888418	0.036934846	0.110831374
ENSRNOG00000024207	Fgfrl1	61.14740181	-0.00926737	0.166750131
ENSRNOG00000008907	Fggy	28.95659949	-0.169576822	0.245421007
ENSRNOG00000024594	Fhdc1	21.37759289	-0.302967164	0.290520635
ENSRNOG00000017225	Fhip2a	74.87426152	0.342552941	0.170972
ENSRNOG00000012014	Fhip2b	54.30572582	0.038303833	0.194381267
ENSRNOG00000000875	Fhl1	17.93794428	-0.139088003	0.29735632
ENSRNOG00000007541	Fhl3	27.4557954	-0.02909354	0.242025521
ENSRNOG00000004699	Fibin	22.95568298	-0.51676955	0.322134862
ENSRNOG00000020567	Fibp	18.19819569	-0.278219809	0.302371231
ENSRNOG00000011521	Filip1	19.00157413	-0.138198537	0.293157141
ENSRNOG00000001645	Filip1l	17.26868308	0.702780795	0.316075222
ENSRNOG00000002275	Fip1l1	51.59493441	-0.167299205	0.176887542
ENSRNOG00000016574	Fiz1	126.935503	-0.052177118	0.126294565
ENSRNOG00000015941	Fkbp10	26.94886885	-0.337934628	0.289605168

ENSRNOG00000024663	Fkbp15	17.1090697	0.065131551	0.340763109
ENSRNOG00000047143	Fkbp1b	91.51539881	0.011533747	0.152330354
ENSRNOG00000022523	Fkbp5	143.5082032	-0.081400264	0.131503493
ENSRNOG00000011758	Fkbp7	9.027958271	0.218950252	0.448111525
ENSRNOG00000058359	Fkbp8	174.0964166	-0.110898605	0.099733585
ENSRNOG00000000432	Fkbpl	8.537108703	-0.614797017	0.978655894
ENSRNOG00000016055	Fkrp	179.9766246	0.072560766	0.101607833
ENSRNOG00000020642	Flad1	9.12762543	0.705100654	0.451433695
ENSRNOG00000003302	Flcn	180.3445447	0.058953798	0.101006414
ENSRNOG00000008904	Fli1	24.23748026	-0.223882287	0.288726621
ENSRNOG00000054890	Flna	68.28197925	0.432419703	0.169639839
ENSRNOG00000009470	Flnb	62.19143778	0.03315324	0.163709126
ENSRNOG00000007281	Flnc	10.63592639	0.170977798	0.420646403
ENSRNOG00000000826	Flot1	24.86637019	0.149494819	0.253214525
ENSRNOG00000003732	Flrt2	23.55803974	-0.375726402	0.272544534
ENSRNOG00000004874	Flrt3	150.6816982	-0.126415204	0.110375091
ENSRNOG00000054764	Flt3	15.52226428	-0.001804824	0.302812456
ENSRNOG00000002511	Flt4	8.522371069	0.344961209	0.452765796
ENSRNOG00000003207	Fmnl1	95.14371614	0.355381433	0.145662254
ENSRNOG00000055567	Fmnl2	49.62532814	0.056689316	0.235835944
ENSRNOG00000034191	Fmo1	23.39740012	-0.496857361	0.355149198
ENSRNOG00000003183	Fmod	24.88020883	0.002687586	0.625441582
ENSRNOG00000057464	Fmr1	119.9990816	0.140391938	0.148517212
ENSRNOG00000014288	Fn1	90.07479979	-0.093581164	0.241633927
ENSRNOG00000036659	Fn3k	45.53549624	-0.150640211	0.203636343
ENSRNOG00000008258	Fnbp1	97.5382153	0.077249941	0.154750011
ENSRNOG00000013798	Fnbp1l	61.12662942	-0.321711995	0.19889964
ENSRNOG00000007241	Fnbp4	41.87602324	0.151296626	0.203098899
ENSRNOG00000013243	Fndc11	11.93843771	0.926689436	0.400732742
ENSRNOG00000014478	Fndc3a	26.57676507	0.412983783	0.269859193
ENSRNOG00000027833	Fnip2	9.221398031	0.981287092	0.489536056
ENSRNOG00000014462	Fnta	55.8484908	-0.011931667	0.185965254
ENSRNOG00000024402	Focad	36.04371951	0.595101164	0.226885784
ENSRNOG00000013770	Folh1	17.34231583	-0.315859754	0.389281751
ENSRNOG00000008015	Fos	190.3321068	0.195570047	0.490691654
ENSRNOG00000046667	Fosb	61.3249491	0.139837602	0.373744907
ENSRNOG00000043332	Foxd1	8.872603491	0.452117496	0.510966573
ENSRNOG00000049906	Foxf1	24.2336954	0.037901425	0.258549721
ENSRNOG00000009000	Foxj2	104.0200643	-0.251495953	0.623239623
ENSRNOG00000061851	Foxj3	9.337184936	0.833154559	0.472256968
ENSRNOG00000040110	Foxn2	56.95839606	-0.214120169	0.174310539
ENSRNOG00000004709	Foxn3	113.4026275	0.122975855	0.13369203
ENSRNOG00000013397	Foxo1	17.64095929	0.356342276	0.340297629
ENSRNOG00000054508	Foxp2	75.27690053	-0.327306306	0.195905406
ENSRNOG00000022804	Foxp4	131.913061	-0.064006543	0.161352528
ENSRNOG00000021752	Foxq1	12.74321209	0.269269573	0.358658541
ENSRNOG00000029089	Foxr1	9.277998763	0.316141161	0.480104572
ENSRNOG00000005749	Foxred2	87.21711401	-0.199219333	0.158128598
ENSRNOG00000008529	Foxs1	15.35718807	-0.271888349	0.3210713
ENSRNOG00000050780	Fpgs	43.92080575	0.372158329	0.214954124

ENSRNOG00000009548	Fpgt	71.39814358	0.146029524	0.157712441
ENSRNOG00000015235	Fra10ac1	26.73315366	-0.164376679	0.236179525
ENSRNOG00000002053	Fras1	16.99757348	0.101255687	0.369476533
ENSRNOG00000009846	Frg1	104.0726654	-0.062803534	0.148938518
ENSRNOG00000000964	Frg1l1	18.04431074	-0.22145103	0.345655915
ENSRNOG00000006503	Frmd3	29.52021303	0.002440924	0.305695923
ENSRNOG00000018500	Frmd4a	41.96860912	-0.169168378	0.257180267
ENSRNOG00000007764	Frmd4b	14.16015797	0.536028182	0.415253637
ENSRNOG00000016189	Frmd5	68.55232117	0.512216839	0.159295105
ENSRNOG00000007329	Frmd6	100.8376519	-0.180639458	0.17817844
ENSRNOG00000020881	Frmd8	97.89283496	0.027478299	0.190991936
ENSRNOG00000012546	Frmpd1	73.3146027	-0.112191705	0.258612147
ENSRNOG00000004118	Frmpd4	17.61653	-0.035888233	0.342609934
ENSRNOG00000005642	Frs2	124.3730749	-0.080487783	0.14125566
ENSRNOG00000014328	Frs3	72.05095585	0.009262153	0.161606898
ENSRNOG00000002248	Fryl	55.03392214	-0.017928921	0.196125474
ENSRNOG00000007765	Frzb	21.4237529	0.151807854	0.327192983
ENSRNOG00000056585	Fscn1	70.19843931	0.052513787	0.21652397
ENSRNOG00000050318	Fsd1	21.183115	0.20298248	0.282879344
ENSRNOG00000059876	Fsd1l	35.32725516	0.075514081	0.227793385
ENSRNOG00000019278	Fsd2	106.4096747	-0.181099815	0.131443838
ENSRNOG00000011631	Fst	7.884785546	0.219111107	0.477240672
ENSRNOG00000002746	Fstl1	142.3969948	0.074148939	0.161510889
ENSRNOG00000006565	Fstl4	10.36957354	-0.014791894	0.416211496
ENSRNOG00000001261	Ftcd	20.85981393	-0.922854848	0.366545698
ENSRNOG00000004776	Ftsj1	40.08179956	0.396814178	0.219042689
ENSRNOG00000043370	Fubp1	81.0724911	0.075717414	0.162925671
ENSRNOG00000009139	Fubp3	63.53787618	-0.068111522	0.175372087
ENSRNOG00000009325	Fuca1	163.7786475	0.139758997	0.101876912
ENSRNOG00000015551	Fuca2	120.7744965	-0.219024746	0.136058984
ENSRNOG00000003470	Fundc1	173.1061576	-0.213321576	0.112343775
ENSRNOG00000024066	Fundc2	31.0090971	0.09329986	0.244973797
ENSRNOG00000050857	Fut10	18.3277988	0.325729668	0.29467288
ENSRNOG00000021011	Fut2	27.97029559	-0.059377726	0.236721482
ENSRNOG00000051480	Fxr1	142.149771	0.005757124	0.121816794
ENSRNOG00000011876	Fxr2	37.46051102	0.226920522	0.241890036
ENSRNOG00000021079	Fxyd1	7.91688838	0.216182925	0.466417204
ENSRNOG00000014578	Fxyd4	19.43075073	-0.189788027	0.318163594
ENSRNOG00000021062	Fxyd5	99.92438268	-0.036856475	0.159355126
ENSRNOG00000021067	Fxyd7	26.83750346	-0.338238165	0.29937463
ENSRNOG00000013886	Fyb1	12.57827815	0.196480439	0.379291647
ENSRNOG00000006336	Fyco1	24.34198789	0.114709196	0.260810942
ENSRNOG00000034233	Fyttd1	30.05794882	-0.108670152	0.249179925
ENSRNOG00000022784	Fzd10	18.91746808	-0.197395808	0.339738406
ENSRNOG00000021962	Fzd2	44.16158863	-0.081207948	0.196084561
ENSRNOG00000047211	Fzd3	24.05125424	-0.107590852	0.248608531
ENSRNOG00000006019	G0s2	56.37646916	0.031969981	0.182117097
ENSRNOG00000002433	G3bp2	9.232233765	-0.307502544	0.396916535
ENSRNOG00000020902	G6pc3	58.13121327	0.208658088	0.167354418
ENSRNOG00000047656	Gaa	29.94650252	0.215301455	0.269020625

ENSRNOG00000011882	Gab2	89.34058742	-0.023897888	0.153293248
ENSRNOG00000053205	Gabpa	49.18708826	0.143082165	0.194001517
ENSRNOG00000010659	Gabpb1	73.49155867	0.047791282	0.158757702
ENSRNOG00000002349	Gabra2	29.18131346	-0.326931291	0.277786245
ENSRNOG00000060599	Gabrb3	34.02274577	-0.062898478	0.288874693
ENSRNOG00000002360	Gabrg1	11.79680885	-0.09483219	0.382285306
ENSRNOG00000003241	Gabrg2	50.50525917	0.020350787	0.232367428
ENSRNOG00000007603	Gabrr1	20.27180569	-0.248475806	0.266413532
ENSRNOG00000007490	Gabrr2	20.49481679	0.024225352	0.315062972
ENSRNOG00000001679	Gabrr3	8.737186927	-0.172556732	0.433337786
ENSRNOG00000005615	Gadd45a	29.58410845	0.097957265	0.236021356
ENSRNOG00000019822	Gadd45b	74.49833831	0.29308048	0.24429356
ENSRNOG00000013090	Gadd45g	155.058581	-0.2587239	0.314717528
ENSRNOG00000003011	Gadd45gip1	22.34305353	0.281025024	0.274170867
ENSRNOG00000042041	Gal3st1	46.99799479	0.202510075	0.195100215
ENSRNOG00000006359	Galk1	120.703612	-0.264728014	0.293238421
ENSRNOG00000009289	Galk2	21.5385737	0.031600508	0.285937008
ENSRNOG00000007023	Galm	100.8311154	-0.132210992	0.147753748
ENSRNOG00000014461	Galns	33.55374817	0.455813946	0.543817301
ENSRNOG00000016207	Galnt1	12.72358966	-0.324429086	0.396233822
ENSRNOG00000008117	Galnt11	159.3542294	0.042452388	0.113284723
ENSRNOG00000005335	Galnt13	28.03526558	-0.031334324	0.249542578
ENSRNOG00000007951	Galnt14	83.99740946	-0.139751931	0.150164295
ENSRNOG00000024435	Galnt17	8.312273206	0.446587779	0.481088018
ENSRNOG00000017021	Galnt18	156.0292712	-0.028484263	0.140646265
ENSRNOG00000004645	Galnt5	98.1522133	0.199495958	0.223369768
ENSRNOG00000012037	Galnt7	51.7671166	-0.086087993	0.193320257
ENSRNOG00000014766	Galt	13.63616032	0.220283791	0.3267572
ENSRNOG00000024577	Gamt	126.0205718	0.032246407	0.12386614
ENSRNOG00000019724	Ganab	8.339590889	-0.173761747	0.475981014
ENSRNOG00000024563	Ganc	27.56330793	0.046017751	0.266064059
ENSRNOG00000018630	Gapdh	135.0663761	-0.203173883	0.133462517
ENSRNOG00000021009	Gapdhs	16.6999449	0.337127162	0.320108515
ENSRNOG00000017925	Gapvd1	90.97450507	-0.173877557	0.146197381
ENSRNOG00000057044	Garnl3	98.70259472	0.176276826	0.199188783
ENSRNOG00000021129	Garre1	70.33035386	0.041762207	0.154090343
ENSRNOG00000028292	Gart	158.8184291	-0.029559623	0.107237737
ENSRNOG00000009040	Gas2l1	80.0782281	0.259037006	0.15536875
ENSRNOG00000018233	Gas6	106.2070224	-0.435995546	0.19035349
ENSRNOG00000049361	Gas7	149.3332109	0.063493911	0.130008607
ENSRNOG00000026964	Gas8	18.31641537	0.189042714	0.318826392
ENSRNOG00000025676	Gask1a	79.63745331	-0.015530985	0.144464633
ENSRNOG00000010183	Gask1b	34.12663025	0.39370373	0.316944734
ENSRNOG00000012347	Gata2	26.9944888	0.292253634	0.245378183
ENSRNOG00000008613	Gatad1	11.66061907	-0.0045747	0.400335085
ENSRNOG00000022173	Gatad2a	158.402602	-0.087398256	0.139613885
ENSRNOG00000015553	Gatad2b	171.6654293	-0.491597788	0.1380195
ENSRNOG00000037655	Gatb	12.52766815	0.099184212	0.377047802
ENSRNOG00000045621	Gatc	9.925473589	0.259725483	0.453599156
ENSRNOG00000045795	Gatd1	56.65720075	0.125316694	0.221633267

ENSRNOG00000016364	Gba2	93.45977588	0.137726469	0.139617715
ENSRNOG00000051232	Gbe1	94.828171	0.201828073	0.137660431
ENSRNOG00000031743	Gbp2	11.42790937	-0.039437959	0.444681016
ENSRNOG00000007359	Gca	174.3135841	-0.054019628	0.121039683
ENSRNOG00000000823	Gcc2	143.2711662	0.071754563	0.125785571
ENSRNOG00000003307	Gcdh	27.83607946	-0.624047328	0.303085889
ENSRNOG00000006888	Gcfc2	34.44385712	0.147248566	0.256942559
ENSRNOG00000006302	Gclc	175.7882557	0.020185034	0.112806798
ENSRNOG00000013409	Gclm	69.11799168	-0.01141153	0.159400383
ENSRNOG00000021871	Gcn1	158.0505443	0.133394005	0.129406421
ENSRNOG00000023778	Gcnt2	30.22628687	0.162534251	0.251883911
ENSRNOG00000011535	Gcsh	12.91218582	0.153100629	0.366187338
ENSRNOG00000019754	Gdap2	125.9360821	0.07780962	0.114393433
ENSRNOG00000050445	Gde1	100.022752	0.082419404	0.152029027
ENSRNOG00000051993	Gdf10	32.86667075	-0.213550108	0.230500849
ENSRNOG00000007610	Gdf11	144.9728542	0.093319344	0.113282592
ENSRNOG00000018091	Gdi2	66.00358662	-0.12564528	0.431107497
ENSRNOG00000002800	Gdpd2	17.06326761	-0.082341194	0.326864418
ENSRNOG00000036859	Gdpd5	110.9617254	-0.173956418	0.133543858
ENSRNOG00000015160	Gem	17.08565673	0.053481745	0.303140327
ENSRNOG00000004360	Gemin2	55.10242657	0.234435818	0.176009882
ENSRNOG00000055236	Gemin4	64.89556838	-0.038511969	0.158761912
ENSRNOG00000002645	Gemin5	48.32500591	0.309707495	0.22493671
ENSRNOG00000027332	Gemin6	55.10034528	-0.060480809	0.179335758
ENSRNOG00000049772	Gemin7	10.64267999	0.803130858	0.434924478
ENSRNOG00000034168	Gemin7l1	15.57213378	0.474265748	0.360562173
ENSRNOG00000003747	Get3	31.71195706	0.054932405	0.22249372
ENSRNOG00000001293	Get4	144.0040802	0.346607813	0.12456383
ENSRNOG00000002919	Gfap	142.1220364	0.093541934	0.613095548
ENSRNOG00000012873	Gfm1	23.99244485	-0.055896327	0.274715378
ENSRNOG00000025285	Gfm2	76.7416319	-0.020949427	0.149987585
ENSRNOG00000017953	Gfod2	80.77114051	0.041035642	0.133483265
ENSRNOG00000018507	Gfpt1	159.3587823	0.093276553	0.131813218
ENSRNOG00000002810	Gfpt2	15.19706003	-0.176508723	0.314231811
ENSRNOG00000021241	Gfra4	15.50850045	0.078159316	0.412486526
ENSRNOG00000018599	Gga2	94.51770389	-0.14733097	0.245218743
ENSRNOG00000027057	Gga3	18.08148977	0.150194781	0.297123632
ENSRNOG00000034084	Ggct	107.1805772	-0.085417958	0.133175684
ENSRNOG00000012975	Ggcx	10.25167975	-0.146828227	0.425195571
ENSRNOG00000016767	Ggps1	82.39847311	0.02049737	0.154604726
ENSRNOG00000010349	Ghrl	42.72312202	-0.032118209	0.233203053
ENSRNOG00000001410	Gigyf1	32.00592635	0.00966465	0.240574421
ENSRNOG00000023577	Gigyf2	179.0080562	0.011415015	0.114330895
ENSRNOG00000008416	Gimap5	9.94132127	-0.524664024	0.411758617
ENSRNOG00000033338	Gimap6	14.65464103	0.136881514	0.359978006
ENSRNOG00000020169	Gimap8	7.905397156	0.270987706	0.451522622
ENSRNOG00000011962	Gin1	49.12011811	0.046548356	0.211256729
ENSRNOG00000015239	Ginm1	8.088219877	0.06166834	0.506406035
ENSRNOG00000017575	Gins2	172.6298087	0.088709484	0.162749099
ENSRNOG00000011863	Gins3	15.01142915	-0.488800958	0.321403649

ENSRNOG00000018040	Gins4	9.762671341	-0.256647879	0.514739326
ENSRNOG00000003864	Gipc1	14.45327845	-0.110750252	0.454548415
ENSRNOG00000042152	Gipc2	17.17416547	0.150483766	0.306838202
ENSRNOG00000015860	Gipr	29.89804704	-0.306336403	0.231459879
ENSRNOG00000061270	Git1	91.71009819	-0.03711738	0.149186184
ENSRNOG00000014357	Gja4	31.20368745	0.34192032	0.220479169
ENSRNOG00000008855	Gjb2	73.16933204	0.472315093	0.38015717
ENSRNOG00000048838	Gjc1	20.04922465	0.474880344	0.289744859
ENSRNOG00000038328	Gjc2	71.64531621	0.043083277	0.204580958
ENSRNOG00000051691	Gjd3	11.22945566	0.213071469	0.505914367
ENSRNOG00000034116	Gk	44.84652401	0.162850666	0.203144139
ENSRNOG00000011513	Gla	11.53357786	0.511724881	0.430444901
ENSRNOG00000010196	Glb1	106.1475037	0.072774935	0.139732421
ENSRNOG00000008524	Glcci1	161.7128106	-0.035867272	0.135480085
ENSRNOG00000025372	Glce	178.3252542	0.011437591	0.108390187
ENSRNOG00000011599	Gldc	89.79279644	0.093284623	0.153578374
ENSRNOG00000024082	Gldn	20.17005028	-0.943454731	0.333170266
ENSRNOG00000015237	Gle1	23.16736799	-0.056231634	0.283717725
ENSRNOG00000014395	Gli3	19.35452584	0.437520359	0.366560762
ENSRNOG00000021765	Gli4	35.21207002	-0.241014499	0.208149958
ENSRNOG00000026644	Glipr1	35.80562231	0.168993314	0.208873622
ENSRNOG00000002054	Glmn	26.84363286	-0.031916856	0.269743579
ENSRNOG00000007788	Glod4	31.9332844	-0.105064837	0.218570293
ENSRNOG00000039571	Glod5	9.88864265	0.43810984	0.459856517
ENSRNOG00000003313	Glra2	71.64977385	-0.373612586	0.198810209
ENSRNOG00000056246	Gls	143.3219948	0.052624599	0.114987114
ENSRNOG00000031612	Gls2	24.43387901	0.36373431	0.295060819
ENSRNOG00000000961	Glt1d1	81.89719765	0.028064045	0.143751639
ENSRNOG00000018179	Glt8d1	14.22637048	0.443718727	0.392648172
ENSRNOG00000036622	Glyatl1	8.39862886	0.992124904	0.565579078
ENSRNOG00000003065	Glyr1	20.40834402	0.1279476	0.360192767
ENSRNOG00000028116	Gm11437	29.0743019	0.078462919	0.260537596
ENSRNOG00000055177	Gm23011	32.53423282	0.11808054	0.24927182
ENSRNOG00000059581	Gm23377	7.788414858	0.393634102	0.569512006
ENSRNOG00000058981	Gm23639	11.27276157	-0.472599372	0.476367805
ENSRNOG00000061433	Gm24564	41.45879328	0.476836526	0.287944895
ENSRNOG00000043487	Gm715	9.864289222	0.285982198	0.428215506
ENSRNOG00000017605	Gmds	96.28873078	0.109834343	0.136868956
ENSRNOG00000010910	Gmeb1	103.6877521	0.208951417	0.153866528
ENSRNOG00000013339	Gmeb2	39.76365983	0.042840625	0.214696106
ENSRNOG00000019838	Gmfg	13.82195794	0.006247864	0.334095515
ENSRNOG00000027257	Gmip	12.48869225	-0.710024173	0.422598099
ENSRNOG00000018782	Gmnn	21.9563306	-0.074666056	0.345924843
ENSRNOG00000019946	Gmppa	100.0749776	-0.028641184	0.154382467
ENSRNOG00000020216	Gmpr2	16.35425294	0.84849386	0.41591657
ENSRNOG00000051151	Gmps	88.63962419	-0.387794674	0.141784774
ENSRNOG00000005446	Gna11	151.1369479	-0.525857608	0.153300303
ENSRNOG00000001235	Gna12	102.2789279	-0.151039026	0.171297045
ENSRNOG00000014840	Gna14	48.60888486	0.028570186	0.182334482
ENSRNOG00000019465	Gnai3	181.2297797	-0.010683791	0.126294461

ENSRNOG00000010440	Gnal	136.5648068	-0.005168583	0.110774842
ENSRNOG00000014183	Gnaq	17.67971035	-0.015130352	0.301711638
ENSRNOG00000025889	Gnas	25.96124119	-0.236800845	0.248276982
ENSRNOG00000001313	Gnaz	75.28009207	-0.119582372	0.145489458
ENSRNOG00000001891	Gnb1l	32.43158442	0.098122606	0.224451152
ENSRNOG00000001409	Gnb2	115.5252269	0.361552905	0.141740188
ENSRNOG00000011070	Gnb4	13.75253569	-0.356745789	0.408922833
ENSRNOG00000047799	Gnb5	11.50981313	-0.525278199	0.390835176
ENSRNOG00000014365	Gne	37.83131187	-0.009524798	0.223767185
ENSRNOG00000050469	Gng11	53.97047839	0.182002228	0.20720653
ENSRNOG00000050231	Gng12	43.94894136	0.113716568	0.197073393
ENSRNOG00000016701	Gng8	29.94690973	-0.001470339	0.291980086
ENSRNOG00000047213	Gnpda1	166.6290125	0.037061737	0.111324014
ENSRNOG00000002177	Gnpda2	14.83657608	-0.178148828	0.320918132
ENSRNOG00000008641	Gnpnat1	42.67292107	-0.148398667	0.211308497
ENSRNOG00000005228	Gnptab	124.8478338	0.18745755	0.160963227
ENSRNOG00000024455	Gnptg	69.95001138	-0.011989519	0.176571698
ENSRNOG00000014493	Golga1	189.30006	0.058544849	0.123009414
ENSRNOG00000011884	Golga2	99.31346056	-0.001012055	0.149250077
ENSRNOG00000037437	Golga3	67.5403381	0.011111113	0.167580748
ENSRNOG00000007699	Golga5	108.4795698	-0.160165144	0.122505721
ENSRNOG00000030314	Golgb1	60.87311249	-0.209544609	0.214575634
ENSRNOG00000020297	Gon4l	17.95897007	-0.070156578	0.360775818
ENSRNOG00000046085	Gon7	78.33533498	-0.224108303	0.167733542
ENSRNOG00000000408	Gopc	190.32492	0.064568098	0.127336808
ENSRNOG00000003861	Gorab	70.01445327	-0.227127642	0.154065373
ENSRNOG00000023386	Gorasp2	76.89847874	0.236003213	0.150983906
ENSRNOG00000003971	Gosr1	66.75399498	0.149204127	0.178515793
ENSRNOG00000001037	Gpalpp1	38.80727896	-0.408658164	0.246260411
ENSRNOG00000015124	Gpam	9.997538823	0.302245509	0.396597525
ENSRNOG00000002159	Gpat3	14.30038239	0.055671578	0.350420655
ENSRNOG00000011584	Gpatch1	42.39569621	0.201363154	0.256227521
ENSRNOG00000025317	Gpatch11	66.98839583	-0.299761961	0.160946103
ENSRNOG00000010227	Gpatch2l	17.59039669	0.393767976	0.319380147
ENSRNOG00000018969	Gpatch4	16.23626285	0.018515723	0.334328506
ENSRNOG00000021044	Gpatch8	151.2915054	0.012095564	0.130469282
ENSRNOG00000037595	Gpbp1l1	38.83338509	-0.030435555	0.238023614
ENSRNOG00000046675	Gpbp1l2	86.66946571	0.067150102	0.165909424
ENSRNOG00000060179	Gpc3	83.50235096	0.042665139	0.211781647
ENSRNOG00000002413	Gpc4	110.8318103	-0.031564783	0.149994364
ENSRNOG00000053201	Gpcpd1	144.7908635	0.10895103	0.127960148
ENSRNOG00000056457	Gpd1	22.5862841	0.278462807	0.350089387
ENSRNOG00000022939	Gpkow	153.6979244	-0.069686223	0.103570809
ENSRNOG00000017702	Gpld1	68.63388561	0.202748295	0.175639492
ENSRNOG00000004613	Gpm6b	133.0138702	0.340256729	0.185673723
ENSRNOG00000004941	Gpn1	66.64922201	-0.036843843	0.173871262
ENSRNOG00000007083	Gpn2	72.28950328	-0.202435202	0.157723961
ENSRNOG00000008816	Gpnmb	34.84147051	0.127973146	0.31281842
ENSRNOG00000023589	Gpr107	159.8937863	0.03261571	0.100097194
ENSRNOG00000046128	Gpr108	181.8200569	-0.041724357	0.100801847

ENSRNOG00000039832	Gpr12	64.19818051	0.241290643	0.188028503
ENSRNOG00000004499	Gpr135	16.63130369	-0.117836956	0.324522619
ENSRNOG00000001288	Gpr146	18.42003474	0.899044121	0.346741417
ENSRNOG00000014793	Gpr149	8.551702456	0.000357258	0.498377346
ENSRNOG00000026944	Gpr150	62.33495174	-0.003739237	0.190469323
ENSRNOG00000018485	Gpr155	8.291277004	0.215782079	0.428672897
ENSRNOG00000009487	Gpr160	88.1073539	0.353057169	0.144707836
ENSRNOG00000005971	Gpr176	70.99552405	-0.443348368	0.154098427
ENSRNOG00000007126	Gpr19	88.36374359	-0.150830043	0.158552529
ENSRNOG00000042269	Gpr21	8.610822767	0.394040336	0.456535771
ENSRNOG00000009540	Gpr3	23.35436648	-0.241680089	0.355754597
ENSRNOG00000039759	Gpr34	30.25126736	0.165189413	0.237292064
ENSRNOG00000002524	Gpr37	166.0210705	0.026800511	0.121784339
ENSRNOG00000006237	Gpr37l1	52.26757825	-0.008555535	0.195872133
ENSRNOG00000016362	Gpr4	29.7618642	0.089323992	0.272607109
ENSRNOG00000016446	Gpr45	30.55797765	-0.084653064	0.231206122
ENSRNOG00000055673	Gpr52	43.03214506	0.119943991	0.221664307
ENSRNOG00000049580	Gpr6	10.24172755	1.755832536	0.510187392
ENSRNOG00000019738	Gpr61	10.43401066	-0.604619416	0.488315987
ENSRNOG00000022176	Gpr75	25.66809097	0.255621725	0.24672749
ENSRNOG00000030318	Gpr83	74.95145129	0.025495958	0.162035924
ENSRNOG00000036834	Gpr84	23.78742902	-0.136647275	0.308717341
ENSRNOG00000024636	Gpr85	190.1393971	-0.051214556	0.103815206
ENSRNOG00000000095	Gpr89b	25.09393076	0.437354657	0.30429846
ENSRNOG00000016013	Gprc5b	104.8162829	0.028254074	0.137671687
ENSRNOG00000003144	Gprc5c	13.56667041	0.109990909	0.337396948
ENSRNOG00000017974	Gprin1	11.47391527	0.149375052	0.381490479
ENSRNOG00000046698	Gps1	21.82389782	-0.440505202	0.282639646
ENSRNOG00000018666	Gpsm1	12.65082012	0.603911568	0.41870056
ENSRNOG00000009751	Gpx7	56.77681743	0.347914922	0.176761662
ENSRNOG00000010461	Gpx8	74.1112827	-0.135180723	0.196311107
ENSRNOG00000021106	Gramd1a	40.73289165	-0.259154026	0.213087444
ENSRNOG00000053577	Gramd1b	30.23516618	0.220570463	0.228069835
ENSRNOG00000015225	Gramd2b	185.279822	-0.26397322	0.137505507
ENSRNOG00000016968	Gramd4	10.83408088	0.25162203	0.388484615
ENSRNOG00000004290	Grb10	125.8063421	0.045564228	0.123583941
ENSRNOG00000052498	Grb14	155.6704326	-0.372159076	0.141930502
ENSRNOG00000024651	Greb1	11.88319308	-0.18071037	0.412065991
ENSRNOG00000023492	Greb1l	8.919353445	0.146911611	0.44526017
ENSRNOG00000026053	Grem1	13.33727935	-0.164090391	0.358214437
ENSRNOG00000003616	Grem2	146.8278564	-0.131901815	0.120157642
ENSRNOG00000045816	Gria1	140.7318542	-0.003756853	0.138423384
ENSRNOG00000055499	Grid1	186.2220569	-0.363516555	0.131227239
ENSRNOG00000006174	Grid2	29.74232554	0.120294976	0.230384686
ENSRNOG00000030927	Grid2ip	14.57821518	-0.010705105	0.361536026
ENSRNOG00000001575	Grik1	45.00137031	-0.327615395	0.220507062
ENSRNOG00000000368	Grik2	115.2875411	0.11095349	0.120754673
ENSRNOG00000030910	Grik4	77.52566737	-0.009158592	0.165726601
ENSRNOG00000020310	Grik5	64.90208279	0.34540923	0.198041281
ENSRNOG00000011726	Grin1	121.9618146	0.26100087	0.153339351

ENSRNOG00000033942	Grin2a	11.55496053	-0.156036576	0.427903878
ENSRNOG00000008766	Grin2b	139.60692	0.091642878	0.116176805
ENSRNOG00000005723	Grin3a	9.131185546	0.385452836	0.459524804
ENSRNOG00000012562	Grin3b	20.8245528	-0.185590059	0.319715255
ENSRNOG00000004013	Grip1	22.25827299	0.467192563	0.308762097
ENSRNOG00000009071	Gripap1	77.01884475	-0.355803989	0.189273754
ENSRNOG00000018985	Grk2	21.29421278	0.136643602	0.293623193
ENSRNOG00000059456	Grk3	19.53233753	-0.089565021	0.295848098
ENSRNOG00000011847	Grk4	49.55438637	0.513908693	0.230136883
ENSRNOG00000011439	Grk5	20.23841202	0.178657337	0.321693116
ENSRNOG00000014290	Grm1	78.8119581	-0.018175624	0.163867349
ENSRNOG00000000487	Grm4	50.79159883	0.095642173	0.193229217
ENSRNOG00000016429	Grm5	26.63201967	0.482365477	0.286517222
ENSRNOG00000005662	Grm7	12.32483167	0.448814432	0.407699181
ENSRNOG00000021468	Grm8	68.30474853	0.072777127	0.181959788
ENSRNOG00000006593	Grpel1	60.35138645	-0.246054432	0.583964253
ENSRNOG00000003392	Grsf1	14.73229509	2.017911688	0.764402409
ENSRNOG00000021058	Grwd1	29.41961125	0.099300071	0.247985231
ENSRNOG00000038891	Grxcr1	31.39861354	-0.202352219	0.224087717
ENSRNOG00000007728	Gsdmd	21.74937264	0.122725411	0.305048964
ENSRNOG00000009970	Gsdme	34.36305799	-0.089731236	0.208436743
ENSRNOG00000017489	Gse1	9.98623872	-0.191578901	0.471472734
ENSRNOG00000020417	Gsk3a	54.1583322	0.06000839	0.179740002
ENSRNOG00000002833	Gsk3b	119.3914063	0.038920614	0.1360141
ENSRNOG00000042427	Gskip	8.617064719	-0.421934802	0.475886626
ENSRNOG00000048817	Gspt2	97.34549543	-0.302695417	0.14484732
ENSRNOG00000014915	Gsr	119.590637	0.05152554	0.138790923
ENSRNOG00000056847	Gsta3	13.45563043	0.003638195	0.348826321
ENSRNOG00000030449	Gsta4	172.6277405	-0.219771487	0.15971856
ENSRNOG00000033402	Gsta6	59.29809748	-0.051590493	0.207578882
ENSRNOG00000011669	Gstcd	15.97362924	0.202221915	0.32191146
ENSRNOG00000016484	Gstk1	135.4459175	0.00265852	0.12477338
ENSRNOG00000019221	Gstm4	114.2311465	0.255502822	0.146183384
ENSRNOG00000028746	Gsto1	126.4789248	0.046284852	0.132799033
ENSRNOG00000049771	Gstt1	4.890510584	0.402994134	0.668721018
ENSRNOG00000052415	Gstt2	43.62894717	0.184734102	0.207438816
ENSRNOG00000038300	Gstt4	46.34794204	4.825093906	2.523771914
ENSRNOG00000004300	Gtf2a1	115.2227154	0.015695719	0.123957956
ENSRNOG00000011135	Gtf2b	96.85930427	-0.026839658	0.145012834
ENSRNOG00000026008	Gtf2e1	35.34151491	0.312515376	0.235580713
ENSRNOG00000014422	Gtf2e2	119.9504757	-0.459093695	0.142628254
ENSRNOG00000047134	Gtf2f1	24.8268442	0.003866304	0.264898839
ENSRNOG00000029316	Gtf2f2	66.91432893	0.073353009	0.180146787
ENSRNOG00000018230	Gtf2h2	62.05299803	-0.215820148	0.182016347
ENSRNOG00000001035	Gtf2h3	12.62987765	0.240561426	0.406987933
ENSRNOG00000001478	Gtf2ird1	93.26934236	0.04662749	0.136938667
ENSRNOG00000001482	Gtf2ird2	34.24043613	0.057702285	0.220619606
ENSRNOG00000050016	Gtf3a	9.537417833	0.730036858	0.466148555
ENSRNOG00000056904	Gtf3c2	28.68313448	0.323131268	0.308478502
ENSRNOG00000002091	Gtf3c3	9.891560861	0.256861672	0.409734602

ENSRNOG00000054725	Gtf3c4	93.01302743	0.135235423	0.155714164
ENSRNOG00000010981	Gtf3c5	8.907101026	0.160419392	0.446036846
ENSRNOG00000007098	Gtpbp10	51.6672632	0.161127158	0.182284914
ENSRNOG00000019332	Gtpbp2	17.07489753	-0.004797754	0.317902255
ENSRNOG00000023403	Gtpbp3	8.908521887	0.631753316	0.487113206
ENSRNOG00000016217	Gtpbp4	68.72036137	-0.180673457	0.181866931
ENSRNOG00000002044	Gtpbp8	13.94079675	0.010336136	0.368605369
ENSRNOG00000036831	Gtsf1	22.92681762	-0.230126415	0.305714426
ENSRNOG00000029876	Gucy1a2	9.637464254	0.428078165	0.425926717
ENSRNOG00000002207	Guf1	43.52307549	0.146763152	0.202942102
ENSRNOG00000003242	Gulp1	25.43639613	-0.229876859	0.316273295
ENSRNOG00000005234	Gxylt1	110.8073591	-0.14252585	0.135632077
ENSRNOG00000005621	Gxylt2	8.053510284	0.613936208	0.504730727
ENSRNOG00000011146	Gyg1	14.55083553	-0.77409894	0.34932852
ENSRNOG00000029939	Gypc	51.71935676	0.301362982	0.180471025
ENSRNOG00000030530	Gzmm	128.1076208	-0.266019864	0.169975877
ENSRNOG00000027722	H1f10	118.0411434	0.066555575	0.128240067
ENSRNOG00000061863	H1f6	18.31561216	0.220747826	0.300483859
ENSRNOG00000025251	H1f8	13.62066161	0.715361305	0.574041377
ENSRNOG00000052275	H2az2	36.87074855	-0.225456694	0.254716468
ENSRNOG00000017523	H6pd	13.97386757	-0.373069396	0.411863082
ENSRNOG00000019027	Habp4	13.77442204	0.22041492	0.353993956
ENSRNOG00000043192	Hacd1	48.55538136	0.451322469	0.206313468
ENSRNOG00000038761	Hacd2	119.418969	-0.006062898	0.124308261
ENSRNOG00000030232	Hacd3	25.99554123	0.195820556	0.255499122
ENSRNOG00000019630	Hacl1	34.30914452	-0.021131048	0.249549685
ENSRNOG00000010697	Hadh	125.2004193	0.186715567	0.123225153
ENSRNOG00000024629	Hadha	124.5509795	0.059731312	0.135238837
ENSRNOG00000032002	Hapln1	151.985067	-0.004668443	0.122162735
ENSRNOG00000018870	Hapln2	42.7381146	0.283184207	0.219320816
ENSRNOG00000028105	Hars1	28.37947597	-0.489112354	0.52785501
ENSRNOG00000010994	Has1	32.51766601	0.155652536	0.252426221
ENSRNOG00000059362	Has3	9.875243269	0.121433309	0.474023144
ENSRNOG00000001524	Hat1	66.94606154	0.070899717	0.156719394
ENSRNOG00000017067	Haus1	12.26097715	-0.338027194	0.384498337
ENSRNOG00000039284	Haus4	28.04309067	-0.013629823	0.32024237
ENSRNOG00000046028	Haus6	16.59535843	0.427983427	0.322046775
ENSRNOG00000055648	Haus7	18.36438332	0.220516505	0.355466329
ENSRNOG00000052038	Haus8	16.24147413	-0.023986808	0.325395524
ENSRNOG00000045647	Hax1	31.25066997	-0.266590611	0.246387291
ENSRNOG00000047098	Hbb-b1	113.4020049	-2.206489856	0.730318874
ENSRNOG00000018646	Hbegf	21.28107214	-0.106737425	0.294092134
ENSRNOG00000008927	Hbp1	70.7620343	0.003873109	0.166573789
ENSRNOG00000014531	Hbs1l	174.8027623	-0.019509744	0.132126299
ENSRNOG00000025910	Hccs	98.40133079	-0.104077442	0.152024784
ENSRNOG00000053510	Hcfc2	87.97552855	-0.10820851	0.206273749
ENSRNOG00000009331	Hck	14.30556725	-0.467785574	0.333975873
ENSRNOG00000055382	Hcn1	32.05095491	0.117512545	0.258659434
ENSRNOG00000008831	Hcn2	63.53675038	-0.342694437	0.234011753
ENSRNOG00000009450	Hcn4	46.05322268	0.206705775	0.227431243

ENSRNOG00000013838	Hcrtr1	8.168591525	0.184726734	0.488504337
ENSRNOG00000011251	Hcrtr2	8.142114543	0.107225109	0.450879112
ENSRNOG00000009568	Hdac1	18.05124185	-0.670503795	0.656359711
ENSRNOG00000031915	Hdac10	23.54013685	-0.341420922	0.258405684
ENSRNOG00000019618	Hdac3	21.60216951	-0.221523423	0.285853318
ENSRNOG00000020372	Hdac4	88.5566758	0.139279146	0.154059265
ENSRNOG00000020905	Hdac5	106.3871691	-0.687026404	0.216252981
ENSRNOG00000003122	Hdac8	38.37783917	0.195456608	0.249860824
ENSRNOG00000004158	Hdac9	26.36028998	0.109088213	0.345323479
ENSRNOG00000021442	Hddc2	26.54239726	-0.423100352	0.241594026
ENSRNOG00000042261	Hdgf	18.50257374	0.16392737	0.328389174
ENSRNOG00000049142	Hdgfl2	30.23483332	-0.01969632	0.269593308
ENSRNOG00000019740	Hdgfl3	31.62260013	0.121312991	0.232691888
ENSRNOG00000011338	Hdhd5	69.54133603	0.267767552	0.171811366
ENSRNOG00000031479	Hdlbp	122.1386729	0.07343331	0.13008426
ENSRNOG00000047594	Heatr1	161.6742866	-0.01561571	0.128430711
ENSRNOG00000015459	Heatr3	82.45946045	0.007198132	0.146554547
ENSRNOG00000038183	Heatr4	21.98262523	0.619293777	0.305411707
ENSRNOG00000006483	Heatr5a	30.60251337	0.00090273	0.259063166
ENSRNOG00000025926	Heatr5b	170.6067604	0.081771134	0.13280242
ENSRNOG00000002542	Heatr6	23.45807984	0.02848658	0.292407232
ENSRNOG00000053735	Hebp2	15.01511465	-0.225614752	0.370164701
ENSRNOG00000060972	Heca	124.4173392	-0.113873353	0.124029265
ENSRNOG00000006905	Hectd1	173.8694159	0.029454059	0.117453436
ENSRNOG00000056753	Hectd2	115.7599818	0.171114887	0.145963363
ENSRNOG00000018363	Hectd3	8.53332297	0.54905572	0.46542706
ENSRNOG00000001352	Hectd4	80.79176727	0.075890102	0.184952741
ENSRNOG00000013257	Hecw2	20.42677666	0.368731011	0.305142681
ENSRNOG00000001793	Heg1	37.59861051	0.015228694	0.230761504
ENSRNOG00000004189	Helb	22.22769192	0.698772272	0.281644015
ENSRNOG00000047692	Hells	8.201751499	-0.242521406	0.611801053
ENSRNOG00000002181	Helq	45.82177797	0.084748483	0.206297297
ENSRNOG00000003213	Helz	117.1687508	0.103697464	0.148497567
ENSRNOG00000013267	Helz2	18.24127975	0.146253346	0.332365871
ENSRNOG00000015458	Hemk1	37.91689071	-0.127476122	0.235834688
ENSRNOG00000009219	Hepacam	37.14700718	0.243715189	0.23736245
ENSRNOG00000012294	Heph	8.39777408	0.553997099	0.503865311
ENSRNOG00000051671	Herc1	190.354488	0.131882544	0.142122282
ENSRNOG00000061040	Herc4	72.43612353	0.179575619	0.151116986
ENSRNOG00000023969	Herc6	14.20746944	0.254697708	0.404263735
ENSRNOG00000001720	Hes1	114.1778441	-0.185809344	0.214688756
ENSRNOG00000010893	Hes3	82.98375809	0.050733648	0.161325472
ENSRNOG00000020194	Hes6	155.7421559	-0.081962269	0.113881102
ENSRNOG00000025274	Hexb	71.30804274	0.176755623	0.165797692
ENSRNOG00000036667	Hexd	9.848971187	0.203265388	0.44949963
ENSRNOG00000003203	Hexim1	148.8803037	0.113618967	0.125864914
ENSRNOG00000021287	Hexim2	49.98633614	-0.396125622	0.206587172
ENSRNOG00000013364	Hey2	59.15007162	0.061791162	0.168417316
ENSRNOG00000015318	Heyl	146.6457604	-0.134810527	0.115077993
ENSRNOG00000023299	Hfm1	13.80226776	0.825474605	0.353426926

ENSRNOG00000007027	Hgf	30.84974453	0.157230612	0.237202426
ENSRNOG00000036696	Hgs	20.52922167	-0.096932916	0.274859129
ENSRNOG00000019404	Hhatl	13.09517702	0.457905659	0.412203238
ENSRNOG00000016595	Hhex	22.89158547	-0.043212175	0.269839197
ENSRNOG00000015502	Hiatl3	27.15278326	-0.138615302	0.255838448
ENSRNOG00000028557	Hibch	128.0360124	-0.118938845	0.134192964
ENSRNOG00000051458	Hic2	21.033761	0.284128561	0.280202349
ENSRNOG00000003464	Hid1	94.1399832	-0.3787975	0.345077594
ENSRNOG00000009499	Hinfp	19.91556628	-0.32299331	0.304066326
ENSRNOG00000015866	Hint2	17.98076538	0.558278334	0.40991983
ENSRNOG00000014190	Hint3	124.3377916	0.14252214	0.131318497
ENSRNOG00000001091	Hip1r	36.73718556	-0.658182886	0.286506798
ENSRNOG00000019333	Hipk1	18.03370193	0.354916194	0.698527629
ENSRNOG00000020835	Hipk4	150.149599	0.275964202	0.119718385
ENSRNOG00000045770	Hira	121.4775925	0.222548238	0.178493395
ENSRNOG00000014460	Hivep1	19.14996168	0.183390842	0.304794085
ENSRNOG00000046891	Hk1	71.15682289	-0.160949797	0.159389356
ENSRNOG00000006116	Hk2	23.87194649	0.127593012	0.265837671
ENSRNOG00000001686	Hlcs	74.59104189	0.18479066	0.175591286
ENSRNOG00000000082	Hltf	12.08687576	0.127835009	0.403340926
ENSRNOG00000013326	Hmbox1	14.16529674	0.050448425	0.3976226
ENSRNOG00000010390	Hmbs	16.23193156	0.603342815	0.496121395
ENSRNOG00000028627	Hmcn1	9.40006982	0.089669465	0.451892912
ENSRNOG00000016905	Hmg20a	27.65478693	0.040745578	0.237009925
ENSRNOG00000020601	Hmg20b	22.98479215	0.486634855	0.288276974
ENSRNOG00000000488	Hmga1	18.71144175	-0.575848759	0.303529947
ENSRNOG00000013167	Hmgb2l1	27.61510877	0.420764091	0.269507252
ENSRNOG00000033321	Hmgb2l1	10.05184734	0.045691881	0.411220807
ENSRNOG00000009422	Hmgcl	10.8318616	0.093884131	0.383528141
ENSRNOG00000011193	Hmgcll1	90.53672768	-0.287098088	0.129848451
ENSRNOG00000016552	Hmgcs1	47.94753912	0.107525916	0.438965408
ENSRNOG00000019120	Hmgcs2	16.98354243	0.06397239	0.351433787
ENSRNOG00000031032	Hmgn3	189.6722145	0.001177182	0.101057347
ENSRNOG00000018132	Hmgxb3	27.3616637	0.332856019	0.25575046
ENSRNOG00000013878	Hmgxb4	15.88213608	0.001820142	0.330884901
ENSRNOG00000014117	Hmox1	30.0368024	0.332869184	0.256124582
ENSRNOG00000003773	Hmox2	14.43145436	0.166371289	0.352830189
ENSRNOG00000008895	Hnf4a	9.802765333	0.296454615	0.45710415
ENSRNOG00000005223	Hnmt	49.09813585	-0.114235966	0.20365153
ENSRNOG00000039876	Hnrnpa0	15.00422035	0.186923749	0.331304772
ENSRNOG00000036839	Hnrnpa1	43.28305378	-0.158104277	0.260278393
ENSRNOG00000011175	Hnrnpa2b1	121.9985436	-0.06098728	0.155372918
ENSRNOG00000052968	Hnrnpa3	49.32323086	0.090877793	0.182386933
ENSRNOG00000003645	Hnrnpab	9.788045837	-0.171567845	0.507411977
ENSRNOG00000002270	Hnrnpdl	90.1094437	0.015993968	0.158858639
ENSRNOG00000003399	Hnrnph1	15.44748612	-0.008479697	0.335220822
ENSRNOG00000011661	Hnrnph2	41.99068166	0.041712255	0.195477471
ENSRNOG00000020235	Hnrnpl	30.66552939	0.199946301	0.246751041
ENSRNOG00000006929	Hnrnpll	45.42683705	0.2474385	0.20660816
ENSRNOG00000007921	Hnrnpm	107.1337002	-0.097074712	0.134030527

ENSRNOG00000020683	Hnrnpul1	22.78865462	0.302353403	0.348424914
ENSRNOG00000019507	Hnrnpul2	49.14372061	0.069318702	0.202215346
ENSRNOG00000029501	Hoga1	16.67316038	0.169061621	0.349226173
ENSRNOG00000047014	Homer1	85.45160833	-0.48027042	0.172163673
ENSRNOG00000061450	Homer2	84.90058907	0.304895863	0.229450464
ENSRNOG00000020229	Homer3	35.17552826	0.102383471	0.227328133
ENSRNOG00000014887	Homez	12.64931118	-0.222853174	0.394825314
ENSRNOG00000026226	Hook1	92.69413354	-0.12226595	0.131047906
ENSRNOG00000003682	Hook2	18.16872658	0.180795821	0.354306415
ENSRNOG00000006979	Hpca	19.75637246	0.140625031	0.31435319
ENSRNOG00000001338	Hpd	113.1338681	-0.084899554	0.128117598
ENSRNOG00000031406	Hps3	14.44394592	0.060587678	0.421062614
ENSRNOG00000000661	Hps4	116.1235088	0.051419728	0.123133447
ENSRNOG00000055531	Hps5	30.04931716	0.14959779	0.237371917
ENSRNOG00000018433	Hps6	73.68960157	-0.230944416	0.154593745
ENSRNOG00000006062	Hs1bp3	75.13326109	0.031247562	0.155291722
ENSRNOG00000000605	Hs3st5	46.61631181	0.056302829	0.235465411
ENSRNOG00000014672	Hs3st6	9.481144138	-0.667357949	0.431083237
ENSRNOG00000037508	Hscb	108.3026249	-0.015970711	0.136653376
ENSRNOG00000005861	Hsd11b1	136.7813914	-0.087157386	0.142915099
ENSRNOG00000002210	Hsd17b11	88.7560645	0.171439704	0.145609469
ENSRNOG00000002212	Hsd17b13	17.31175402	0.424633582	0.312796574
ENSRNOG00000015840	Hsd17b4	183.5921597	0.170143649	0.097864587
ENSRNOG00000000466	Hsd17b8	19.69505427	-1.013061604	1.232755247
ENSRNOG00000016692	Hsdl2	27.26395824	0.00692631	0.237995255
ENSRNOG00000019834	Hsp90ab1	139.9027599	0.233582843	0.329090457
ENSRNOG00000026963	Hsp90b1	100.9406119	-0.41540656	0.383680495
ENSRNOG00000018019	Hspa12a	19.02503432	0.318702722	0.329491766
ENSRNOG00000021244	Hspa12b	20.61757498	-0.380560616	0.266873503
ENSRNOG00000060979	Hspa13	169.449064	0.165536166	0.108007282
ENSRNOG00000015212	Hspa14	102.8313599	0.084907861	0.145735597
ENSRNOG00000006472	Hspa2	51.28134576	0.116861105	0.204886301
ENSRNOG00000018294	Hspa5	97.06618815	-0.047014539	0.130047193
ENSRNOG00000023546	Hspb1	34.23852263	0.083659528	0.226253815
ENSRNOG00000010241	Hspb11	82.26912662	0.06407133	0.173360243
ENSRNOG00000022392	Hspb8	142.4015342	-0.002133445	0.130192266
ENSRNOG00000002241	Hspbap1	43.59660829	0.22568648	0.20515916
ENSRNOG00000017795	Hspbp1	77.51538472	-0.006594726	0.161369478
ENSRNOG00000051624	Hspe1	161.269747	0.019037487	0.117217562
ENSRNOG00000010063	Htr2a	152.2072844	0.19399139	0.12923566
ENSRNOG00000030877	Htr2c	135.8271885	-0.066538931	0.162662849
ENSRNOG00000006595	Htr3a	50.95628749	0.189187974	0.207391784
ENSRNOG00000049761	Htr6	15.24746268	0.042815445	0.354396049
ENSRNOG00000008182	Htra3	54.18186004	0.285642573	0.192250435
ENSRNOG00000061160	Htra4	36.18543196	-0.356852926	0.264045415
ENSRNOG00000011073	Htt	141.1610077	0.247112686	0.134218405
ENSRNOG00000002092	Hunk	9.817002893	0.614184163	0.472338521
ENSRNOG00000005141	Hus1	67.5137427	0.137518395	0.175235538
ENSRNOG00000061262	Huwe1	25.76827106	0.305953025	0.294483801
ENSRNOG00000017178	Hydin	16.34401834	0.670321687	0.364743679

ENSRNOG00000013419	Hykk	98.49354409	0.086932705	0.165656715
ENSRNOG00000002158	Ibsp	15.06288118	0.478493417	0.361581093
ENSRNOG00000027728	Ibtk	11.59122709	-0.114560117	0.40292246
ENSRNOG00000008628	Ica1	173.6429354	-0.036310225	0.115789322
ENSRNOG00000017258	Ica1l	33.35030872	0.376806119	0.231891167
ENSRNOG00000023053	Ice1	38.05205956	0.085315404	0.221462145
ENSRNOG00000010160	Ice2	19.23222861	0.070691234	0.331717792
ENSRNOG00000010953	Icmt	22.49617449	-0.438258405	0.28565482
ENSRNOG00000023109	Icoslg	107.7714559	0.090413661	0.185190182
ENSRNOG00000021750	Id1	131.0408579	0.022840929	0.149437153
ENSRNOG00000026124	Id3	134.7171728	-0.080756685	0.152560527
ENSRNOG00000016099	Id4	126.8125115	0.017668732	0.156660078
ENSRNOG00000016833	Ide	118.7997865	0.013873081	0.139938701
ENSRNOG00000015020	Idh1	190.4366955	0.165891095	0.128993819
ENSRNOG00000013949	Idh2	94.38506525	-0.192589341	0.129661148
ENSRNOG00000010277	Idh3a	99.59127106	-0.096809829	0.147240285
ENSRNOG00000007316	Idh3b	11.80503263	-0.013106451	0.421809544
ENSRNOG00000055572	Idh3g	21.18585128	-0.291782915	0.314437973
ENSRNOG00000016690	Idi1	180.7405975	-0.041997038	0.138102227
ENSRNOG00000019519	Idnk	31.91663489	-0.217996434	0.233119491
ENSRNOG00000001465	Ids	12.94994135	0.45956353	0.385986655
ENSRNOG00000000043	Idua	27.82619388	0.358762929	0.281220788
ENSRNOG00000024846	Ier5l	126.7271523	-0.261053589	0.194294152
ENSRNOG00000009263	Ifi27	64.44330843	0.289398751	0.480484949
ENSRNOG00000022218	Ifi44	25.62523135	0.312074029	0.277427141
ENSRNOG00000002470	Ifi47	11.83387346	-0.141536885	0.351228017
ENSRNOG00000036604	Ifit2	14.65797101	-0.309586859	0.341802986
ENSRNOG00000004273	Ifitm1	15.69032403	-0.443002127	0.349002254
ENSRNOG00000020164	Ifitm10	46.86062878	0.295604138	0.244450939
ENSRNOG00000014936	Ifitm2	32.31439092	-0.116681175	0.383520388
ENSRNOG00000015078	Ifitm3	54.49773306	0.148270647	0.174641744
ENSRNOG00000014923	Ifitm5	16.34323921	0.179145406	0.340977519
ENSRNOG00000036645	Ifitm6	67.89414855	0.081307243	0.168475369
ENSRNOG00000028594	Ifnar1	188.5082219	-0.033861344	0.101422622
ENSRNOG00000045668	Ifnar2	61.53885168	-0.308738796	0.200394761
ENSRNOG00000033984	Ifnlr1	10.45831036	-0.832537174	0.441686184
ENSRNOG00000050997	Ifrd1	158.0094625	0.22170712	0.125176965
ENSRNOG00000016150	Ifrd2	77.93963008	0.049173712	0.170792963
ENSRNOG00000010952	Ift122	136.08653	0.238875801	0.111587878
ENSRNOG00000016545	Ift140	74.32007801	0.242806701	0.15610192
ENSRNOG00000031346	Ift22	138.3617184	-0.174989757	0.146538463
ENSRNOG00000006440	Ift27	103.875782	0.175411309	0.135530324
ENSRNOG00000010194	Ift43	34.06016773	-0.037118522	0.251750086
ENSRNOG00000013919	Ift46	27.29279687	0.25193073	0.296851263
ENSRNOG00000007692	Ift52	143.2367593	0.17148528	0.124731344
ENSRNOG00000008075	Ift74	95.92851043	0.211875361	0.151123675
ENSRNOG00000009808	Ift80	60.74558835	-0.009640711	0.175747311
ENSRNOG00000001294	Ift81	180.0112242	0.061430677	0.113027766
ENSRNOG00000037567	Iftap	118.3521587	0.189588729	0.143294507
ENSRNOG00000026267	Igbp1	175.0328831	-0.054128869	0.09738866

ENSRNOG00000033496	Igdcc4	27.84099375	0.049820644	0.25157389
ENSRNOG00000004517	Igf1	32.41747133	-0.489814522	0.216436419
ENSRNOG00000014187	Igf1r	31.75104844	0.150138442	0.28938447
ENSRNOG00000009052	Igf2bp3	22.19107487	0.460551327	0.313786426
ENSRNOG00000061910	Igfbp3	46.60622417	-0.199726125	0.185309931
ENSRNOG00000017206	Igfbp5	173.3084042	0.157038008	0.13277724
ENSRNOG00000002050	Igfbp7	122.7384312	0.038453567	0.129292022
ENSRNOG00000013456	Ighmbp2	123.9111638	-0.001180026	0.128541687
ENSRNOG00000017918	Iglon5	120.2658145	-0.003403656	0.12260181
ENSRNOG00000013917	Igsf10	49.64258981	0.141972025	0.201034594
ENSRNOG00000023851	Igsf3	88.34614414	0.02283319	0.168719577
ENSRNOG00000027008	Igtp	19.48024192	-0.156919792	0.313670836
ENSRNOG00000008247	Ikbip	74.58214083	-0.046371416	0.15913428
ENSRNOG00000019073	Ikbkb	76.50631086	0.467380789	0.174678561
ENSRNOG00000025100	Ikbke	9.117648023	-0.439155875	0.407441376
ENSRNOG00000027430	Ikzf2	8.878150327	-0.241873444	0.444639684
ENSRNOG00000005535	Ikzf4	7.859444632	0.411913784	0.536921978
ENSRNOG00000016308	Il10ra	22.27325014	-0.019519261	0.311266296
ENSRNOG00000028638	Il10rb	95.15215031	0.140602977	0.153206877
ENSRNOG00000009468	Il12a	19.22782531	-0.134649594	0.294038165
ENSRNOG00000007270	Il12rb2	54.56851863	0.257038327	0.176591391
ENSRNOG00000001713	Il13ra1	24.96729722	0.15340212	0.285442603
ENSRNOG00000011680	Il16	12.70638323	0.680963391	0.405651648
ENSRNOG00000011153	Il17ra	90.42288963	-0.141889039	0.147199074
ENSRNOG00000015498	Il17rb	13.78995769	0.050055685	0.349331133
ENSRNOG00000009848	Il18	15.20461631	0.165408402	0.352226369
ENSRNOG00000001928	Il1rap	134.2600389	0.155673748	0.133324114
ENSRNOG00000062221	Il1rapl2	84.79781483	-0.115219128	0.145352513
ENSRNOG00000004470	Il24	125.8240649	-2.554085618	1.512607454
ENSRNOG00000005747	Il27ra	8.087965787	0.07024923	0.537571719
ENSRNOG00000016456	Il33	98.44211545	-0.029943595	0.160221252
ENSRNOG00000001325	Il3ra	11.5021764	0.411382193	0.382514224
ENSRNOG00000020148	Il4i1	18.29264459	-0.271156512	0.379133491
ENSRNOG00000015441	Il4r	25.84972597	-0.511830378	0.251610557
ENSRNOG00000020811	Il6r	37.26016508	-0.016812459	0.219232293
ENSRNOG00000013963	Il6st	13.76530464	0.12212178	0.385332254
ENSRNOG00000025151	Ildr2	22.84168559	0.008850618	0.275550474
ENSRNOG00000014154	Ilf2	165.0400796	0.007545046	0.106099906
ENSRNOG00000020115	Ilkap	81.81430311	0.199257145	0.15750593
ENSRNOG00000038883	Ilrun	34.50726964	-3.019055184	1.227057277
ENSRNOG00000028512	Ilvbl	32.20827879	-0.014657696	0.262600285
ENSRNOG00000004829	Immp1l	21.06369024	-0.052823389	0.279498117
ENSRNOG00000017460	Imp3	149.0010673	-0.093866297	0.133729459
ENSRNOG00000013285	Imp4	151.0872133	-0.074696303	0.114039743
ENSRNOG00000018516	Impa2	21.16242097	0.07170607	0.291551247
ENSRNOG00000032929	Incenp	49.04283418	-0.27998639	0.184890756
ENSRNOG00000028650	Inf2	8.942779057	0.401502526	0.48658855
ENSRNOG00000013480	Ing2	187.616977	-0.00588869	0.103720812
ENSRNOG00000005496	Ing3	52.37034176	-0.181609656	0.17136003
ENSRNOG00000023363	Ing4	17.72781408	-0.243418543	0.368933587

ENSRNOG00000018988	Ing5	10.54342333	0.054794073	0.41610555
ENSRNOG00000020097	Inha	36.07815946	-0.403041497	0.200992199
ENSRNOG00000014320	Inhba	62.92701373	0.062289024	0.176878525
ENSRNOG00000060237	Inhbb	34.37969619	-0.145768147	0.24472896
ENSRNOG00000014483	Ino80	39.06534674	0.072432477	0.199571599
ENSRNOG00000024272	Ino80d	10.08947465	0.163595566	0.443110673
ENSRNOG00000012375	Inpp1	80.88866921	-0.095383061	0.161078996
ENSRNOG00000017660	Inpp4a	10.70664064	-0.075696504	0.396520318
ENSRNOG00000018382	Inpp4b	18.72451621	-0.043287548	0.30632131
ENSRNOG00000017635	Inpp5a	11.48804487	0.2233927	0.435070954
ENSRNOG00000017020	Inpp5d	98.94378777	-0.406072939	0.219125272
ENSRNOG00000019039	Inpp5e	114.144735	-0.045867707	0.1172689
ENSRNOG00000020388	Inpp5f	57.14412026	-0.174115622	0.19431026
ENSRNOG00000019361	Inpp5j	15.81809933	0.179263412	0.394176508
ENSRNOG00000024712	Insc	9.169758035	0.881459081	0.4997068
ENSRNOG00000002478	Insig2	13.20476965	0.622285686	0.381712622
ENSRNOG00000029986	Insr	25.42339632	0.083232698	0.252095252
ENSRNOG00000033881	Insyn2a	39.33241611	0.016551045	0.233402701
ENSRNOG00000019712	Ints11	15.53463255	-0.240469107	0.322200419
ENSRNOG00000011844	Ints12	12.36247328	0.027997509	0.399079627
ENSRNOG00000001808	Ints13	52.41304214	0.173040661	0.196832765
ENSRNOG00000012469	Ints14	18.37290719	0.682851922	0.405605129
ENSRNOG00000015153	Ints3	28.33370236	0.291758033	0.277155174
ENSRNOG00000012552	Ints4	127.8937064	0.099676325	0.127731881
ENSRNOG00000009873	Ints6	17.26001002	0.426332542	0.364999502
ENSRNOG00000004263	Ints7	83.4778225	-0.38388146	0.158204185
ENSRNOG00000008124	Ints8	30.4155947	0.202187073	0.234807242
ENSRNOG00000013459	Ints9	45.99922529	-0.270057141	0.194936836
ENSRNOG00000008632	Invs	8.521682254	0.657035351	0.483006707
ENSRNOG00000020361	Ip6k2	28.41240354	0.007526239	0.252094283
ENSRNOG00000000609	Ipmk	98.48862051	-0.044981981	0.138870296
ENSRNOG00000019758	Ipo13	32.28754934	-0.116309925	0.251138168
ENSRNOG00000010989	Ipo5	177.1910692	-0.257521286	0.508685691
ENSRNOG00000007418	Ipo9	161.0566045	0.058219223	0.106302566
ENSRNOG00000016183	Ipp	17.64213062	-0.278192063	0.322794827
ENSRNOG00000015631	Ippk	10.58363981	-0.262715462	0.477883772
ENSRNOG00000019592	Iqca1	13.73384746	0.505971896	0.436519783
ENSRNOG00000038868	Iqcb1	140.4706484	-0.064347814	0.128157167
ENSRNOG00000048553	Iqcc	8.506921501	0.685793732	0.529504242
ENSRNOG00000001241	Iqce	73.69681939	0.091690031	0.148811038
ENSRNOG00000026420	Iqcg	59.15404735	-0.031831238	0.171788261
ENSRNOG00000036758	Iqck	88.55649841	0.057395262	0.141890478
ENSRNOG00000012002	Iqgap1	17.05817613	-0.169701123	0.344643532
ENSRNOG00000025406	Iqgap2	14.42983377	0.554358726	0.389064788
ENSRNOG00000060350	Iqsec1	127.7835594	0.104947307	0.114451585
ENSRNOG00000017767	Irag1	8.906276303	1.052466887	0.505239677
ENSRNOG00000029308	Irag2	7.92794671	0.292912968	0.476393391
ENSRNOG00000021817	Irak2	12.79625727	0.006965138	0.44340514
ENSRNOG00000005965	Irak4	33.72114499	-0.022063643	0.285325848
ENSRNOG00000013271	Ireb2	13.88883072	-0.065316503	0.355857868

ENSRNOG00000008144	Irf1	39.63824276	0.266434766	0.231662425
ENSRNOG00000009824	Irf2	60.28126593	-0.034047333	0.197175473
ENSRNOG00000011026	Irf2bpl	86.08003229	0.087615632	0.14950373
ENSRNOG00000017869	Irf8	31.57620878	0.152769772	0.244181037
ENSRNOG00000019478	Irf9	23.46770161	-0.145232604	0.25038828
ENSRNOG00000031138	Irgm	26.7959446	0.068207954	0.247245734
ENSRNOG00000023509	Irs2	24.6441054	0.318138167	0.32258469
ENSRNOG00000012085	Isca2	127.0071103	0.007485894	0.135990528
ENSRNOG00000005077	Isca2-ps1	24.93895393	-0.064105202	0.271818183
ENSRNOG00000054561	Isg20	7.601401864	-0.037253045	0.548008109
ENSRNOG00000011402	Isg20l2	17.63269566	-0.146551776	0.298560631
ENSRNOG00000050312	Ism2	88.06926034	0.02973836	0.15761874
ENSRNOG00000050348	Isoc2a	140.2888203	0.034326495	0.118907715
ENSRNOG00000016829	Isoc2b	10.89457027	-0.541965587	0.409482165
ENSRNOG00000015144	Ist1	10.95838095	-0.265454306	0.416411693
ENSRNOG00000010021	Isy1	91.40929356	0.077381878	0.165170812
ENSRNOG00000059043	Itch	19.87790127	0.194601988	0.322294088
ENSRNOG00000006264	Itfg2	84.98727261	-0.065906469	0.141381693
ENSRNOG00000006723	Itga11	30.67097715	-0.296090238	0.267073682
ENSRNOG00000004276	Itga3	11.55626077	-0.138149863	0.465209901
ENSRNOG00000001518	Itga6	107.0290685	0.303735464	0.128782758
ENSRNOG00000016538	Itga8	17.61974933	-0.219715516	0.350269583
ENSRNOG00000046639	Itgae	28.15958207	0.217835327	0.25310646
ENSRNOG00000004912	Itgav	21.56912146	0.050584905	0.287600894
ENSRNOG00000001224	Itgb2	18.58229029	-0.056296378	0.385582319
ENSRNOG00000009116	Itgb3bp	36.73036527	-0.227179827	0.215180481
ENSRNOG00000001795	Itgb5	8.497851832	0.135546629	0.464076533
ENSRNOG00000012208	Itgb7	8.763258814	0.192318701	0.497941363
ENSRNOG00000033386	Itih1	32.58823248	-0.409440638	0.360649574
ENSRNOG00000021233	Itpa	32.95897175	0.016248096	0.291311575
ENSRNOG00000008051	Itpk1	29.25570271	0.32644075	0.248929869
ENSRNOG00000002969	Itpkb	23.63178107	-0.223697295	0.26971437
ENSRNOG00000013945	Itpkc	44.92257444	-0.066389922	0.181025052
ENSRNOG00000052795	Itpr3	15.34598781	0.198523838	0.319204342
ENSRNOG00000005865	Itprid2	116.3152213	0.15931017	0.153288137
ENSRNOG00000002001	Itsn1	104.2177323	0.45631338	0.16030072
ENSRNOG00000046741	Itsn2	60.48278821	-0.212977695	0.255078217
ENSRNOG00000014630	Iws1	49.14102022	0.333727129	0.224957418
ENSRNOG00000014066	Jade1	142.252852	0.104007284	0.123285995
ENSRNOG00000004956	Jade2	18.79990685	-0.098369352	0.287663715
ENSRNOG00000007443	Jag1	38.40240794	-0.057861977	0.220376447
ENSRNOG00000013927	Jag2	129.2786175	0.142875251	0.143304144
ENSRNOG00000009175	Jagn1	169.2169752	-0.14071746	0.127378531
ENSRNOG00000011157	Jak1	123.5508665	-0.055668249	0.125263084
ENSRNOG00000059968	Jak2	146.8100755	0.018734749	0.120035809
ENSRNOG00000019159	Jakmip2	28.94604449	-0.091005852	0.225209248
ENSRNOG00000027590	Jakmip3	13.57742247	1.028143156	0.428170428
ENSRNOG00000042848	Jam2	32.20608678	0.254307623	0.249638323
ENSRNOG00000009149	Jam3	27.4385465	-0.121102777	0.242640024
ENSRNOG00000045918	Jarid2	37.8769238	0.0456661	0.246629533

ENSRNOG00000027026	Jazf1	9.061131878	-0.579997768	0.418050056
ENSRNOG00000004751	Jkamp	154.9279504	-0.035596355	0.119923434
ENSRNOG00000022438	Jmjd4	15.77871674	-0.009914542	0.326342503
ENSRNOG00000000250	Jmjd6	67.81007	0.243297622	0.150322314
ENSRNOG00000050343	Jmy	8.902363203	1.105375019	0.499777477
ENSRNOG00000019442	Josd2	130.8001407	0.000871781	0.146176767
ENSRNOG00000006110	Jph1	60.28062065	-0.2476258	0.170247329
ENSRNOG00000024661	Jpt2	105.2087976	0.220103017	0.134551178
ENSRNOG00000027238	Jrk	14.64253977	-0.173358084	0.379179548
ENSRNOG00000047133	Jrkl	18.05758789	-0.261030119	0.317671208
ENSRNOG00000016023	Kank1	163.3400269	-0.146840935	0.497829638
ENSRNOG00000010643	Kank2	43.92441428	0.27930627	0.200189073
ENSRNOG00000007779	Kank4	12.87276391	-0.13382733	0.337550704
ENSRNOG00000053680	Kansl1	29.86301208	0.173382519	0.272484028
ENSRNOG00000028149	Kansl1l	29.62885907	0.0787126	0.270323696
ENSRNOG00000019456	Kars	41.12689179	-0.012947178	0.189591344
ENSRNOG00000007160	Kat14	28.84794567	0.335806743	0.248859985
ENSRNOG00000061012	Kat5	43.405852	-0.046114333	0.230162408
ENSRNOG00000025174	Kat6a	20.73429013	0.027207294	0.287950875
ENSRNOG00000012850	Kat6b	70.07664703	-0.187634338	0.206161681
ENSRNOG00000019485	Kat8	20.80398056	0.065628593	0.310673164
ENSRNOG00000014996	Katna1	67.76651982	-0.095417464	0.184682212
ENSRNOG00000047618	Katnal1	39.72461882	0.012493349	0.272850706
ENSRNOG00000014626	Katnb1	16.34726286	0.596981894	0.308652457
ENSRNOG00000016058	Kazald1	11.8946481	-0.327482901	0.440251271
ENSRNOG00000014322	Kazn	17.53006224	0.227857856	0.303702695
ENSRNOG00000012333	Kbtbd11	34.80252692	0.278141689	0.227801737
ENSRNOG00000013390	Kbtbd8	23.58806628	-0.257608186	0.31027497
ENSRNOG00000062002	Kcna3	20.54835884	0.009435145	0.281343746
ENSRNOG00000004918	Kcna4	65.72046504	0.130657559	0.167590866
ENSRNOG00000056697	Kcnab1	10.78184596	0.643553305	0.418166345
ENSRNOG00000008480	Kcnab3	9.116216885	0.455388593	0.442046066
ENSRNOG00000046949	Kcnb1	12.98746537	0.027833517	0.33870263
ENSRNOG00000019959	Kcnc3	38.01191247	0.214820048	0.220932475
ENSRNOG00000060988	Kcnc4	52.92741711	-0.049352252	0.191065585
ENSRNOG00000014686	Kcnd3	49.78096742	-0.084950912	0.232547873
ENSRNOG00000024310	Kcnf1	39.45720027	-0.172606387	0.21566251
ENSRNOG00000054314	Kcng1	76.50613832	-0.158435909	0.153458342
ENSRNOG00000015746	Kcng4	14.93674632	0.203004568	0.398892377
ENSRNOG00000003841	Kcnh1	44.00556141	0.242907431	0.268126175
ENSRNOG00000009872	Kcnh2	94.7917263	-0.05690212	0.144151017
ENSRNOG00000057315	Kcnh3	53.33912022	0.080547318	0.250895189
ENSRNOG00000007528	Kcnh7	22.48068519	0.042733682	0.262801467
ENSRNOG00000005365	Kcnip1	163.0961365	-0.0214831	0.123126683
ENSRNOG00000014152	Kcnip3	38.91159819	-0.351216499	0.222080217
ENSRNOG00000032350	Kcnip4	54.89376557	-0.760442993	0.261891168
ENSRNOG00000021128	Kcnj11	147.3869653	0.123856605	0.11020461
ENSRNOG00000002303	Kcnj12	58.03072971	-0.185628053	0.169862562
ENSRNOG00000021056	Kcnj14	33.55876184	-0.008063524	0.224239186
ENSRNOG00000005369	Kcnj3	10.00579047	-0.298082405	0.497504965

ENSRNOG00000001658	Kcnj6	20.34697696	0.032066003	0.271926561
ENSRNOG00000013463	Kcnj8	20.88706656	0.515017977	0.312205146
ENSRNOG00000007645	Kcnj9	14.57815952	0.019998294	0.336255051
ENSRNOG00000016110	Kcnk12	57.29477754	0.035682237	0.272563553
ENSRNOG00000047363	Kcnk13	17.4282045	-0.154851674	0.314564059
ENSRNOG00000002653	Kcnk2	145.9412124	-0.05527601	0.118479448
ENSRNOG00000009790	Kcnk3	137.5789665	-0.031668639	0.132825889
ENSRNOG00000021140	Kcnk4	11.8537663	0.176685479	0.397029973
ENSRNOG00000020598	Kcnk6	12.02982095	0.119052664	0.423518625
ENSRNOG00000020784	Kcnk7	140.0774316	0.205292657	0.147887532
ENSRNOG00000010094	Kcnmb2	7.954200995	0.00838765	0.545253304
ENSRNOG00000029264	Kcnn1	21.27349628	-0.441441531	0.284233621
ENSRNOG00000016675	Kcnn2	95.01261907	0.188610497	0.127919529
ENSRNOG00000020706	Kcnn3	17.34878792	0.509647768	0.358819081
ENSRNOG00000005206	Kcnq3	21.34345125	0.095992637	0.368738679
ENSRNOG00000013681	Kcns1	51.1864733	0.353491246	0.179663126
ENSRNOG00000004899	Kcns3	71.38553985	-0.010064344	0.16701686
ENSRNOG00000013312	Kcnt2	87.36169313	0.05017449	0.152040563
ENSRNOG00000020010	Kctd13	33.64312383	-0.45974372	0.345690638
ENSRNOG00000012494	Kctd14	17.65856241	-0.279603542	0.352800525
ENSRNOG00000021137	Kctd15	98.18631924	0.004931017	0.154544142
ENSRNOG00000000231	Kctd17	31.92300373	0.127017806	0.227676743
ENSRNOG00000027091	Kctd18	59.28445239	-0.004764357	0.185862832
ENSRNOG00000000517	Kctd20	10.18788786	-0.032017815	0.417269577
ENSRNOG00000024793	Kctd21	63.84473862	-0.058848882	0.196797249
ENSRNOG00000050996	Kctd4	140.7518175	0.265044689	0.123267162
ENSRNOG00000007817	Kctd6	15.60377911	1.062658616	0.443141197
ENSRNOG00000042384	Kctd7	10.51457868	0.419721082	0.472748011
ENSRNOG00000021082	Kdelr1	16.53278245	0.181833838	0.315101665
ENSRNOG00000001083	Kdelr2	117.8800258	-0.180632122	0.121667501
ENSRNOG00000022372	Kdm1a	20.2765322	0.70327505	0.343781023
ENSRNOG00000016519	Kdm1b	15.51115901	0.615625897	0.382770412
ENSRNOG00000019145	Kdm2a	102.9477588	0.186112075	0.145571135
ENSRNOG00000025702	Kdm2b	20.9708162	0.067310939	0.311542797
ENSRNOG00000007814	Kdm3a	126.6679225	-0.214262055	0.127210135
ENSRNOG00000019956	Kdm4a	8.211282237	0.402874529	0.456751166
ENSRNOG00000006644	Kdm4c	56.36262663	0.036034716	0.182090759
ENSRNOG00000010591	Kdm5a	154.836323	0.016785327	0.136770527
ENSRNOG00000060544	Kdm5b	174.3944701	0.171370233	0.11518704
ENSRNOG00000057706	Kdm5c	90.36767801	0.204148945	0.135709432
ENSRNOG00000060496	Kdm5d	43.94848835	-0.064765286	0.195436359
ENSRNOG00000052721	Kdm6a	41.39919706	0.05977011	0.260593634
ENSRNOG00000037613	Kdm6b	14.11381387	-0.31165463	0.444548145
ENSRNOG00000052445	Kdm7a	15.03470678	-0.184215327	0.312007739
ENSRNOG00000015180	Kdm8	21.28156315	0.182110218	0.286895923
ENSRNOG00000046829	Kdr	31.22097314	0.033781623	0.324661543
ENSRNOG00000002781	Kdsr	12.97734186	-0.178959457	0.341609051
ENSRNOG00000020139	Khdc4	111.5001501	-0.047834663	0.131082467
ENSRNOG00000008047	Khk	13.75814839	-0.228282932	0.360983712
ENSRNOG00000028782	Kics2	33.09618512	-0.157756276	0.211626861

ENSRNOG00000001455	Kif13a	54.52455161	-0.068341498	0.186120119
ENSRNOG00000013089	Kif13b	101.5564463	0.015237783	0.14916045
ENSRNOG00000004951	Kif16b	31.20757236	-0.165634806	0.228573095
ENSRNOG00000014970	Kif17	59.82001229	0.096605429	0.184171069
ENSRNOG00000031364	Kif1c	48.39171599	0.194827273	0.221813328
ENSRNOG00000014844	Kif21a	131.5377679	0.027439474	0.175882155
ENSRNOG00000008471	Kif21b	31.73278698	-0.051443537	0.228824606
ENSRNOG00000028624	Kif26b	7.896311955	-0.065613435	0.426586971
ENSRNOG00000019257	Kif27	19.14384849	0.276517788	0.305344441
ENSRNOG00000014000	Kif2a	104.8100608	-0.131482668	0.156024948
ENSRNOG00000010361	Kif3b	146.3222644	0.010313816	0.14202748
ENSRNOG00000020891	Kif9	15.88269521	0.076066114	0.319436416
ENSRNOG00000060123	Kifc2	9.564490383	1.497671294	0.528010679
ENSRNOG00000014087	Kifc3	163.5285479	-0.077281932	0.118034522
ENSRNOG00000009772	Kirrel3	158.9856628	0.042581361	0.117209403
ENSRNOG00000002227	Kit	85.85543505	0.04121572	0.184235971
ENSRNOG00000005386	Kitlg	77.82563806	-0.013433466	0.160804374
ENSRNOG00000025141	Kiz	25.5248239	0.270632046	0.287956152
ENSRNOG00000020299	Klc2	87.3961144	-0.107915116	0.150541033
ENSRNOG00000018168	Klc4	96.00845775	-0.016810186	0.139926888
ENSRNOG00000054259	Klf11	15.02926592	0.20730814	0.328665412
ENSRNOG00000017808	Klf15	132.6353443	-0.13128356	0.135865474
ENSRNOG00000002163	Klf3	122.0950827	0.063476342	0.149148219
ENSRNOG00000016299	Klf4	103.6565569	0.477505874	0.482133805
ENSRNOG00000008785	Klf5	66.22739503	-0.362475622	0.181775492
ENSRNOG00000016885	Klf6	136.2298622	-0.056892821	0.14558926
ENSRNOG00000046242	Klf7	88.09723522	0.003347539	0.174409918
ENSRNOG00000004425	Klhdc1	55.70557586	0.006366303	0.204118764
ENSRNOG00000017495	Klhdc3	8.324669544	0.167034545	0.444201302
ENSRNOG00000000036	Klhdc8a	130.6700919	0.144308042	0.166713708
ENSRNOG00000016921	Klhl11	15.9984489	-0.102198731	0.327050315
ENSRNOG00000004193	Klhl12	64.38632747	0.023161683	0.155546648
ENSRNOG00000014029	Klhl13	143.1855628	0.077772703	0.127542299
ENSRNOG00000020880	Klhl18	17.05158516	-0.010098464	0.315924835
ENSRNOG00000002875	Klhl20	30.66399178	0.220001271	0.315920763
ENSRNOG00000001878	Klhl22	9.79260782	0.299229255	0.416547251
ENSRNOG00000033372	Klhl24	61.19353692	-0.07245222	0.193652894
ENSRNOG00000010959	Klhl25	41.09077575	0.10110846	0.211768007
ENSRNOG00000005371	Klhl29	82.82493243	0.031233618	0.141393737
ENSRNOG00000019533	Klhl3	15.03376065	-0.271210853	0.317383656
ENSRNOG00000007441	Klhl32	66.21475481	0.160366119	0.186079899
ENSRNOG00000024479	Klhl34	55.46463665	-0.095762278	0.190550068
ENSRNOG00000017365	Klhl35	67.3969245	-0.019508547	0.181718777
ENSRNOG00000016422	Klhl36	42.34732911	0.014700497	0.208639585
ENSRNOG00000005215	Klhl4	32.83746309	-0.083206358	0.248306735
ENSRNOG00000001844	Klhl42	29.74969581	-0.613478769	0.253573209
ENSRNOG00000008421	Klhl5	34.14833611	0.077754392	0.209973594
ENSRNOG00000010453	Klhl7	23.95541546	-0.609904194	0.252178622
ENSRNOG00000031927	Klk6	56.42341304	-0.133921733	0.211577779
ENSRNOG00000015133	Kmt2a	59.44851374	0.217198087	0.177977182

ENSRNOG00000061080	Kmt2c	68.45439554	-0.214929693	0.175962261
ENSRNOG00000016790	Kmt5b	118.9026358	0.054673086	0.124148448
ENSRNOG00000027240	Kndc1	13.10574582	0.255016562	0.371880726
ENSRNOG00000060100	Knl1	9.328410809	-0.045270321	0.462207448
ENSRNOG00000033658	Kntc1	10.5602047	0.275261891	0.435388805
ENSRNOG00000015329	Kpna2	117.5092946	0.133854568	0.134729131
ENSRNOG00000014945	Kpna3	16.55920314	-0.449500942	0.337710155
ENSRNOG00000010768	Kpna4	12.11550189	0.312714195	0.368468917
ENSRNOG00000030109	Kpna5	11.1381891	-0.010745925	0.389339235
ENSRNOG00000007405	Krba1	69.58990268	0.369733214	0.177293793
ENSRNOG00000007124	Krcc1	113.9850927	-0.122266008	0.123768365
ENSRNOG00000051487	Kremen1	37.21620662	-0.435199522	0.244196214
ENSRNOG00000057447	Kri1	10.50521418	0.202142739	0.384653544
ENSRNOG00000007937	Krit1	149.5685714	0.213719657	0.123260706
ENSRNOG00000028996	Krt1	17.7109418	0.002735871	0.306656165
ENSRNOG00000030170	Krt10	35.06819273	0.230321333	0.227620323
ENSRNOG00000010839	Krt222	111.2737457	-0.08116282	0.202045603
ENSRNOG00000033617	Krt26	61.73985757	0.107505971	0.177717044
ENSRNOG00000049495	Krt71	8.043757035	0.025055436	0.44396943
ENSRNOG00000036871	Krt81	27.45844672	-0.337651905	0.246853317
ENSRNOG00000012818	Ksr1	64.60511723	0.092911504	0.172757077
ENSRNOG00000028630	Ksr2	182.2836128	-0.25505414	0.522783013
ENSRNOG00000019971	Kxd1	22.18203719	-0.324119103	0.279006643
ENSRNOG00000043426	Kyat3	37.10957835	0.227943982	0.229509032
ENSRNOG00000004857	L2hgdh	29.42253272	-0.064196135	0.243481475
ENSRNOG00000004503	L3hypdh	40.1403866	-0.296834954	0.20053343
ENSRNOG00000007044	L3mbtl1	38.77984278	0.525163175	0.210273097
ENSRNOG00000024743	L3mbtl2	57.68716728	0.14233094	0.167513464
ENSRNOG00000011720	L3mbtl3	16.69749807	0.099958454	0.312938068
ENSRNOG00000018081	Lactb	11.61767189	0.003297323	0.366960208
ENSRNOG00000007829	Lactb2	84.94407636	-0.141670531	0.149587128
ENSRNOG00000017237	Lama1	12.82139049	0.405924788	0.361884216
ENSRNOG00000011134	Lama2	13.33815616	0.149353178	0.376284025
ENSRNOG00000000599	Lama4	20.34599587	0.289991354	0.295554352
ENSRNOG00000053691	Lama5	8.594690739	0.086102951	0.543104293
ENSRNOG00000005678	Lamb1	29.04139209	0.103422651	0.246833997
ENSRNOG00000002680	Lamc1	26.31008749	0.051796968	0.252138233
ENSRNOG00000019629	Lamp1	100.9492526	0.062208817	0.145758806
ENSRNOG00000020016	Lamtor1	13.37032039	-0.494541182	0.368917715
ENSRNOG00000019908	Lamtor2	12.44993431	-0.696181096	0.374563091
ENSRNOG00000010552	Lamtor3	151.34594	-0.030437221	0.112512836
ENSRNOG00000018186	Lamtor5	158.2557651	-0.093806838	0.105473518
ENSRNOG00000006810	Lancl2	52.59983462	-0.203387561	0.181465812
ENSRNOG00000003289	Lap3	124.6706003	0.19829621	0.126954059
ENSRNOG00000011054	Laptm5	154.1481093	0.125059855	0.137284646
ENSRNOG00000006353	Large2	57.29557596	0.128281051	0.178163248
ENSRNOG00000031544	Larp1	90.41950269	0.064584617	0.165383341
ENSRNOG00000038366	Larp1b	54.82582826	0.268026583	0.198594572
ENSRNOG00000015888	Larp4b	12.16612849	-0.138010591	0.41757183
ENSRNOG00000048989	Larp7	33.7199181	-0.042272266	0.2183689

ENSRNOG00000018304	Lars1	170.8521137	0.161014504	0.105758283
ENSRNOG00000014916	Lats1	77.18132361	0.336392897	0.157936178
ENSRNOG00000056343	Lats2	57.13982939	0.04224717	0.173943856
ENSRNOG00000011228	Layn	28.95244159	-0.521350537	0.241217221
ENSRNOG00000039902	Lbh	18.3940547	0.071390044	0.350212724
ENSRNOG00000043137	Lbhd2	33.10572708	-0.235332133	0.241016118
ENSRNOG00000009580	Lca5	30.59458764	0.234231876	0.238796052
ENSRNOG00000028248	Lca5l	10.84364942	-0.375577789	0.376371302
ENSRNOG00000009705	Lck	20.06805357	-0.414484357	0.279193235
ENSRNOG00000042679	Lcor	28.7770186	0.209071789	0.27526393
ENSRNOG00000010319	Lcp1	48.00941017	-0.265839572	0.182124749
ENSRNOG00000005620	Lcp2	19.48246234	0.369471672	0.32767447
ENSRNOG00000009193	Lctl	15.68633269	0.009165902	0.351969586
ENSRNOG00000021475	Ldah	145.5870898	-0.077334076	0.104928559
ENSRNOG00000059166	Ldb3	13.85884092	0.194969723	0.338180011
ENSRNOG00000009946	Ldlr	99.79920564	-0.02416394	0.166423269
ENSRNOG00000033503	Ldlrad2	18.89315897	0.09195823	0.34324272
ENSRNOG00000058157	Ldlrad3	48.39310655	-0.002884776	0.20684413
ENSRNOG00000016879	Ldlrad4	14.18841288	0.559354908	0.350240302
ENSRNOG00000010121	Lef1	58.4454286	0.27377053	0.175449314
ENSRNOG00000031247	Lekr1	8.760323866	-0.207968017	0.477945929
ENSRNOG00000025864	Lemd2	190.1022252	0.112460486	0.115625969
ENSRNOG00000018642	Leng8	45.65622703	0.050987715	0.327567373
ENSRNOG00000010116	Leo1	135.1849477	-0.009467234	0.112070028
ENSRNOG00000016427	Letm1	93.42572065	-0.193318939	0.136916871
ENSRNOG00000029855	Letmd1	8.163934226	0.320962621	0.502036232
ENSRNOG00000009884	Lgals1	12.45332595	0.66358454	0.394672301
ENSRNOG00000003217	Lgals3bp	63.54296994	0.178875089	0.161555288
ENSRNOG00000020380	Lgals7	8.685857042	0.594092106	0.464126447
ENSRNOG00000018046	Lgals8	25.56442751	0.203291558	0.250934965
ENSRNOG00000012681	Lgals9	10.19376768	0.45723502	0.448112657
ENSRNOG00000021087	Lgi4	10.04029695	-0.228072479	0.396280678
ENSRNOG00000007089	Lgmn	23.203279	-0.479899398	0.265616354
ENSRNOG00000004221	Lgr5	8.319503112	0.098264075	0.45173868
ENSRNOG00000005093	Lgr6	80.59266271	-0.142621973	0.175056432
ENSRNOG00000011032	Lhfpl2	55.57163521	-0.101789691	0.202712815
ENSRNOG00000022283	Lhfpl5	132.0326188	0.123096961	0.123584489
ENSRNOG00000002759	Lias	54.91113219	-0.09219618	0.173702266
ENSRNOG00000011696	Lifr	10.14611285	0.423276194	0.42897106
ENSRNOG00000014193	Lig1	50.77039933	0.145440492	0.181742488
ENSRNOG00000059801	Lima1	9.666244953	-0.145637657	0.446510408
ENSRNOG00000002318	Limch1	53.70275002	0.221768135	0.19260952
ENSRNOG00000004837	Limd1	16.33215939	1.069238531	0.353980961
ENSRNOG00000025448	Limd2	18.49944822	-1.149898953	0.871331047
ENSRNOG00000049336	Lime1	102.4274306	0.256086742	0.135365148
ENSRNOG00000001470	Limk1	184.3387734	-0.154815589	0.157741456
ENSRNOG00000019000	Limk2	34.32421218	0.767624387	0.343258325
ENSRNOG00000037765	Lims1	13.89616298	0.04738772	0.369793853
ENSRNOG00000016021	Lims2	167.3133505	0.137622895	0.102820444
ENSRNOG00000002203	Lin54	50.82558232	0.036259775	0.184304003

ENSRNOG00000004527	Lin7a	34.37031785	-0.092132633	0.275844212
ENSRNOG00000005634	Lingo2	126.8151691	-0.22320438	0.1372359
ENSRNOG00000032569	Lingo3	11.38541665	-1.659212969	0.448320992
ENSRNOG00000042776	Lins1	17.33702575	-0.113787341	0.287331244
ENSRNOG00000019077	Lipa	184.4998094	-0.200571195	0.097860797
ENSRNOG00000020546	Lipe	35.89771263	0.017124678	0.238256898
ENSRNOG00000016906	Lipt2	66.1723548	-0.031947291	0.171783437
ENSRNOG00000002520	Litaf	127.1472322	-0.036775588	0.124005046
ENSRNOG00000021213	Lix1l	89.28959627	0.045184672	0.171248599
ENSRNOG00000004316	Llph	20.30262067	0.011966892	0.309452476
ENSRNOG00000024470	Lman1	25.4720316	0.546290361	0.264163978
ENSRNOG00000015699	Lman2l	26.11785075	0.296477885	0.258474106
ENSRNOG00000059589	Lmbr1	92.38691324	-0.118720364	0.134960285
ENSRNOG00000061607	Lmbr1l	32.36727721	0.04890697	0.218727723
ENSRNOG00000054751	Lmbrd2	8.326367154	0.108551666	0.465561288
ENSRNOG00000005690	Lmcd1	76.75074282	-0.018786439	0.171141296
ENSRNOG00000000230	Lmf1	121.7256633	0.052733623	0.139041005
ENSRNOG00000030633	Lmf2	123.1171265	0.365065573	0.134413891
ENSRNOG00000025742	Lmnb2	38.75804445	-0.280967173	0.342816931
ENSRNOG00000014629	Lmo1	141.022387	-0.022541011	0.118968537
ENSRNOG00000009401	Lmo2	77.71470202	0.340180211	0.148775588
ENSRNOG00000060775	Lmo7	50.27721535	-0.031729409	0.207236699
ENSRNOG00000025155	Lmtk2	46.76263209	0.510067525	0.225522023
ENSRNOG00000055229	Lnpep	14.53190728	1.034995699	0.378348329
ENSRNOG00000001593	Lnpk	28.27871078	0.136430856	0.228481884
ENSRNOG00000002272	Lnx1	25.83752568	0.113590988	0.257001635
ENSRNOG00000000955	Lnx2	34.77679743	0.131270841	0.222951665
ENSRNOG00000046502	Lonp1	9.150809699	0.49033274	0.713282712
ENSRNOG00000059604	Loricrin	13.16552637	-0.24628474	0.337105816
ENSRNOG00000008680	Loxl1	29.76712819	-0.019158815	0.251614359
ENSRNOG00000016758	Loxl2	32.27969011	-0.982804237	0.659802569
ENSRNOG00000015727	Loxl4	18.2599363	-0.050926442	0.309032036
ENSRNOG00000013656	Lpar1	153.9166094	0.121244156	0.15995736
ENSRNOG00000002428	Lpar4	17.73538678	0.145650817	0.316915765
ENSRNOG00000021401	Lpar5	2.587148842	-0.908707032	0.730566484
ENSRNOG00000015577	Lpar6	17.03403201	0.088957584	0.311999341
ENSRNOG00000017930	Lpcat1	170.2875751	0.071245638	0.10241974
ENSRNOG00000016643	Lpcat2	59.41812811	-0.03880895	0.169922419
ENSRNOG00000012269	Lpcat3	56.94045041	0.256537408	0.206192614
ENSRNOG00000004402	Lpgat1	172.6489527	0.040978913	0.167978801
ENSRNOG00000004377	Lpin1	22.29969408	0.148031356	0.257319785
ENSRNOG00000014876	Lpin2	92.60704787	0.111890839	0.218380242
ENSRNOG00000012181	Lpl	77.98531283	-0.15248818	0.190013355
ENSRNOG00000012611	Lpxn	29.09713834	-0.092200409	0.227484486
ENSRNOG00000023453	Lrba	61.34158268	0.205092071	0.170924929
ENSRNOG00000009412	Lrch1	11.41025529	0.348469336	0.399727693
ENSRNOG00000029582	Lrch2	24.42491628	-0.257530334	0.239630061
ENSRNOG00000001774	Lrch3	43.09078784	0.088741098	0.276711473
ENSRNOG00000001402	Lrch4	35.39838072	-0.429842126	0.500551571
ENSRNOG00000019869	Lrfn1	27.64572516	0.007413353	0.279844999

ENSRNOG00000011803	Lrfn2	183.5412293	-0.026136752	0.132747048
ENSRNOG00000020839	Lrfn3	155.2159314	-0.290575505	0.115520675
ENSRNOG00000019356	Lrfn4	32.77855162	-0.188412321	0.918936075
ENSRNOG00000008775	Lrguk	75.88193863	-0.170648963	0.156028818
ENSRNOG00000017784	Lrif1	83.87018707	-0.026694312	0.155145304
ENSRNOG00000012952	Lrig1	46.43812685	0.079632989	0.18265602
ENSRNOG00000019957	Lrig2	35.764662	-0.166822199	0.220965008
ENSRNOG00000007654	Lrig3	10.69950452	-0.442214194	0.406770726
ENSRNOG00000025053	Lrp1	41.86163196	-0.056182752	0.20919347
ENSRNOG00000004152	Lrp12	167.6643413	-0.025474408	0.11395809
ENSRNOG00000011178	Lrp2bp	10.99213916	1.009527229	0.435920371
ENSRNOG00000015285	Lrp4	106.7384263	0.31396249	0.142807725
ENSRNOG00000015911	Lrp5	18.11653128	0.024852056	0.289725843
ENSRNOG00000006338	Lrp6	23.92927628	-0.17211633	0.308744873
ENSRNOG00000013064	Lrp8	74.08823745	0.002367768	0.159608797
ENSRNOG00000009313	Lrpap1	49.65940207	-0.044271905	0.205397391
ENSRNOG00000005877	Lrpprc	98.25733951	0.322627379	0.139189285
ENSRNOG00000005970	Lrrc1	19.53215541	-0.130502927	0.301468585
ENSRNOG00000005555	Lrrc10	174.4772795	0.166849055	0.108594189
ENSRNOG00000028357	Lrrc14b	164.8184444	-0.021556033	0.117225715
ENSRNOG00000000560	Lrrc20	34.16700972	-0.183902021	0.220018941
ENSRNOG00000026309	Lrrc23	51.98044141	0.170680747	0.206513028
ENSRNOG00000017538	Lrrc27	25.23652115	-0.227206833	0.273613487
ENSRNOG00000023274	Lrrc28	91.76280335	-0.019334978	0.145352892
ENSRNOG00000027935	Lrrc34	10.76605759	0.290920353	0.397850317
ENSRNOG00000015264	Lrrc38	10.93620522	-0.155598155	0.400281514
ENSRNOG00000015117	Lrrc39	12.32243066	0.308624513	0.405376657
ENSRNOG00000005857	Lrrc3b	46.99518829	0.324454939	0.21180036
ENSRNOG00000013642	Lrrc41	11.82898599	0.181800062	0.379319125
ENSRNOG00000008470	Lrrc43	14.9833514	-0.246484396	0.318170602
ENSRNOG00000045926	Lrrc45	19.13868257	0.268296542	0.344160751
ENSRNOG00000024796	Lrrc47	44.34164941	-0.042351662	0.214650548
ENSRNOG00000019418	Lrrc4b	12.58758857	0.113028061	0.407335614
ENSRNOG00000020124	Lrrc51	50.64332785	0.033373243	0.209193765
ENSRNOG00000028266	Lrrc55	64.20372806	-0.181492364	0.172238021
ENSRNOG00000009143	Lrrc57	145.0277988	0.095103948	0.1195463
ENSRNOG00000003524	Lrrc59	11.27202187	-0.386679711	0.475649351
ENSRNOG00000011980	Lrrc7	98.82289809	0.266042679	0.190467721
ENSRNOG00000014937	Lrrc71	79.13070593	0.238862026	0.163337721
ENSRNOG00000025049	Lrrc72	8.703443614	0.205121617	0.43682404
ENSRNOG00000039856	Lrrc73	6.793550203	1.717635598	1.241236223
ENSRNOG00000046428	Lrrc75b	34.05802751	0.045357464	0.223964473
ENSRNOG00000025296	Lrrc8a	64.88128082	-0.14190505	0.257468361
ENSRNOG00000002129	Lrrc8b	58.8604912	-0.0732019	0.202713154
ENSRNOG00000002121	Lrrc8d	116.6775848	0.091406228	0.120510668
ENSRNOG00000010891	Lrrcc1	63.54995671	-0.096361296	0.183458093
ENSRNOG00000019892	Lrrfip1	27.71063978	-0.251758735	0.249945508
ENSRNOG00000021047	Lrrfip2	30.54421467	-0.127592053	0.21541397
ENSRNOG00000012730	Lrrk1	16.13922501	0.191618103	0.367687819
ENSRNOG00000004048	Lrrk2	94.8239785	0.174830847	0.135346955

ENSRNOG00000006802	Lrrn1	177.2443957	-0.098680193	0.11223467
ENSRNOG00000006368	Lrrn3	150.935506	-0.080733604	0.117050679
ENSRNOG00000047085	Lrrtm2	143.7805614	-0.034309187	0.127069816
ENSRNOG00000021938	Lrrtm4	51.90564563	0.088384039	0.210114235
ENSRNOG00000022312	Lrsam1	26.80301012	-0.202586605	0.29607083
ENSRNOG00000007508	Lrtm2	54.17246961	-0.022734087	0.202520101
ENSRNOG00000023434	Lrtomt	45.88627264	-0.176159058	0.17427971
ENSRNOG00000001727	Lsg1	74.80041017	-0.125515617	0.14840569
ENSRNOG00000015375	Lsm1	119.2749729	0.037579857	0.136257553
ENSRNOG00000054557	Lsm14b	67.17716683	-0.076740484	0.191035771
ENSRNOG00000048725	Lsm2	157.9316911	0.166570859	0.119872632
ENSRNOG00000008733	Lsm3	29.32369134	-0.229651382	0.284925557
ENSRNOG00000042126	Lsm5	162.4553832	-0.06895712	0.107553026
ENSRNOG00000027305	Lsm5	26.2249461	0.0059956	0.239612798
ENSRNOG00000030374	Lsm6	9.72155335	-0.273361732	0.372803038
ENSRNOG00000019552	Lsm7	110.770707	0.006864352	0.149245096
ENSRNOG00000052634	Lsmem2	23.17968445	-0.06842534	0.303232309
ENSRNOG00000021053	Lsr	17.10323308	-0.067459317	0.326407307
ENSRNOG00000054549	Lss	137.2648753	-0.075054585	0.155158621
ENSRNOG00000004494	Lta4h	36.4214742	0.098261548	0.226822214
ENSRNOG00000033090	Ltbp1	11.47142588	-0.403538862	0.438540111
ENSRNOG00000020813	Ltbp3	9.111875397	-0.321311971	0.438550892
ENSRNOG00000020871	Ltbp4	106.4313519	0.155042251	0.138648391
ENSRNOG00000019264	Ltbr	130.707501	-0.047894426	0.136704923
ENSRNOG00000003244	Ltc4s	9.025595435	-0.325236382	0.414084744
ENSRNOG00000001602	Ltn1	106.4382358	-0.036159468	0.138349769
ENSRNOG00000020903	Lto1	102.3479855	-0.072002495	0.146633706
ENSRNOG00000020488	Luc7l	67.90623237	-0.032904054	0.174389244
ENSRNOG00000004610	Lum	16.44728358	0.758717627	0.34384051
ENSRNOG00000033740	Lurap1l	70.46077779	-0.049984159	0.155473481
ENSRNOG00000022402	Luzp1	50.43967728	-0.180691151	0.175650002
ENSRNOG00000021507	Luzp2	18.31287935	-0.238927624	0.328450475
ENSRNOG00000007012	Ly75	10.09563043	0.405844218	0.415023936
ENSRNOG00000000137	Ly86	25.35608405	-0.346932501	0.244849971
ENSRNOG00000005374	Lyar	55.3476307	0.151268847	0.178540495
ENSRNOG00000034139	Lyc2	163.3821991	-0.036735744	0.146139271
ENSRNOG00000002950	Lyl1	37.69037739	-0.155335035	0.204366916
ENSRNOG00000008180	Lyn	48.57835815	0.044306137	0.198339271
ENSRNOG00000006086	Lynx1	30.8468238	-0.045143593	0.288546262
ENSRNOG00000019432	Lypd5	15.5255617	-0.034200994	0.312826325
ENSRNOG00000004614	Lypd6b	30.41872971	-0.210639965	0.227030103
ENSRNOG00000008320	Lypla1	169.036345	0.025613629	0.109747632
ENSRNOG00000010067	Lypla2	22.14320842	-0.235284824	0.637258137
ENSRNOG00000023130	Lyplal1	107.0626338	0.129840655	0.154169718
ENSRNOG00000043105	Lyrm2	11.87275983	0.177528045	0.371169159
ENSRNOG00000047970	Lyrm4	141.2109019	-0.149736204	0.109575752
ENSRNOG00000045961	Lyrm7	105.6577851	-0.17336392	0.129789256
ENSRNOG00000037204	Lyrm9	108.9877564	-0.045555371	0.127137982
ENSRNOG00000016298	Lysmd3	9.972434589	0.156995501	0.404482175
ENSRNOG00000019350	Lyzl4	13.67199979	0.608728048	0.406331417

ENSRNOG00000006244	Lztfl1	110.7712614	-0.162010188	0.145541408
ENSRNOG00000011826	Lzts1	178.4113395	-0.066966362	0.107968051
ENSRNOG00000021231	Lzts3	73.20635954	0.10572854	0.173630711
ENSRNOG00000014992	M6pr	16.62939352	0.637507622	0.342644501
ENSRNOG00000032941	Mab21l1	9.981582941	0.604305878	0.52338367
ENSRNOG00000056156	Maco1	144.3564059	-0.141649906	0.125357299
ENSRNOG00000021174	Macrod1	15.11850399	-0.015985367	0.355006681
ENSRNOG00000024751	Macroh2a2	27.01583684	0.015182248	0.254406841
ENSRNOG00000001265	Mad1l1	74.04362294	-0.401318965	0.175093801
ENSRNOG00000005376	Mad2l1	11.98710329	-0.215002371	0.388874338
ENSRNOG00000008217	Madcam1	18.61436196	-0.336811962	0.291576492
ENSRNOG00000012568	Madd	37.14516287	-0.226707825	0.196837913
ENSRNOG00000012428	Maf	44.81200624	0.054008653	0.212317723
ENSRNOG00000016037	Mafb	28.17573588	-0.195070386	0.249241035
ENSRNOG00000021400	Magee2	14.44502034	0.276958506	0.375810867
ENSRNOG00000022060	Magi1	121.0292191	0.263428323	0.144234636
ENSRNOG00000013962	Magi2	32.7427057	0.586476802	0.245809734
ENSRNOG00000019885	Magi3	73.48273812	0.00665417	0.164029662
ENSRNOG00000012778	Magoh	17.95376682	-0.266402208	0.360243444
ENSRNOG00000010316	Magohb	22.08919333	0.439984408	0.271996244
ENSRNOG00000051408	Magt1	76.36040687	0.068351867	0.187996537
ENSRNOG00000015983	Maip1	110.8804021	-0.141834435	0.126165816
ENSRNOG00000009035	Malsu1	32.45295914	-0.045638522	0.245436349
ENSRNOG00000017181	Malt1	22.0170917	-0.001727906	0.299737063
ENSRNOG00000003268	Maml1	26.55110272	-0.231155036	0.269656338
ENSRNOG00000000800	Man1a1	84.48273852	0.204233799	0.191916565
ENSRNOG00000015226	Man1a2	103.8034909	-0.134865123	0.153782244
ENSRNOG00000012559	Man1b1	19.65316631	-0.242427055	0.275136538
ENSRNOG00000015439	Man2a1	62.57766655	-0.071976947	0.183245027
ENSRNOG00000012055	Man2a2	63.7826331	0.056213044	0.169388476
ENSRNOG00000023910	Man2b1	92.52393513	0.047328253	0.150875774
ENSRNOG00000030654	Man2c1	40.53986663	0.018930693	0.200149104
ENSRNOG00000052247	Manba	44.94036738	0.344766846	0.198296712
ENSRNOG00000031453	Manbal	19.42259409	0.509202292	0.299443444
ENSRNOG00000008626	Manea	44.36061137	0.271498326	0.207965319
ENSRNOG00000025349	Maneal	16.73016403	0.137593736	0.367649886
ENSRNOG00000036925	Mansc4	11.76475726	-0.108761473	0.360242808
ENSRNOG00000002848	Maoa	99.03764124	-0.032514847	0.133859665
ENSRNOG00000003834	Map2k4	10.631223	0.390539359	0.449016855
ENSRNOG00000007926	Map2k5	190.1285145	0.00959427	0.10821705
ENSRNOG00000004437	Map2k6	147.4406806	0.115857574	0.113978285
ENSRNOG00000013177	Map3k1	14.15473122	0.018016256	0.345580574
ENSRNOG00000023521	Map3k10	139.1504904	0.180484619	0.133023286
ENSRNOG00000025423	Map3k13	47.24087054	-0.003726143	0.205397851
ENSRNOG00000014089	Map3k2	21.6549457	-0.445819268	0.262220254
ENSRNOG00000061424	Map3k3	61.10479495	-0.075371258	0.171857974
ENSRNOG00000017419	Map3k4	149.3674747	0.148860355	0.119351895
ENSRNOG00000031700	Map3k5	27.99042571	0.099069534	0.248763504
ENSRNOG00000005724	Map3k7	139.3999615	0.140287271	0.112639965
ENSRNOG00000001584	Map3k7cl	31.45749461	0.153781816	0.283303593

ENSRNOG00000007271	Map3k9	48.94859063	0.06834623	0.187558711
ENSRNOG00000020748	Map4	82.22963068	-0.042541731	0.218426796
ENSRNOG00000021061	Map4k2	70.38225734	-0.108972434	0.160813248
ENSRNOG00000004923	Map4k5	33.96477174	0.228760338	0.233977355
ENSRNOG00000012701	Map7	143.7326779	0.026703421	0.131003638
ENSRNOG00000001849	Mapk1	173.5895842	0.052845424	0.113699606
ENSRNOG00000006984	Mapk11	61.88943727	-0.080143745	0.189125474
ENSRNOG00000016935	Mapk1ip1	129.7724235	-0.022355369	0.124087178
ENSRNOG00000011611	Mapk1ip1l	143.0208035	-0.187607922	0.121929076
ENSRNOG00000053583	Mapk3	22.08863694	-0.42192291	0.304826713
ENSRNOG00000020155	Mapk8	34.35521948	0.192952027	0.260216834
ENSRNOG00000058478	Mapk8ip1	9.6951657	-0.047005169	0.525270598
ENSRNOG00000033568	Mapk8ip3	54.45886485	0.072629685	0.181148602
ENSRNOG00000002823	Mapk9	162.7077913	0.059628073	0.123583131
ENSRNOG00000014832	Mapkapk3	70.48453853	0.25827436	0.209884578
ENSRNOG00000001345	Mapkapk5	25.53223079	-0.217285119	0.256841528
ENSRNOG00000007018	Mapkbp1	41.84452339	0.267226382	0.254298814
ENSRNOG00000008961	Mapre3	33.13161665	-0.120894388	0.266174569
ENSRNOG00000026310	Marchf1	11.66368977	0.005183599	0.427738097
ENSRNOG00000007084	Marchf10	39.15306875	-0.005110109	0.271052805
ENSRNOG00000024461	Marchf11	57.80326329	0.449853373	0.182104865
ENSRNOG00000023013	Marchf3	14.46761051	0.235385651	0.325916679
ENSRNOG00000017396	Marchf5	129.5079046	0.073584838	0.119802504
ENSRNOG00000011066	Marchf6	77.83911237	-0.112462588	0.152451842
ENSRNOG00000006241	Marchf7	15.47938021	0.309475792	0.349448676
ENSRNOG00000021184	Mark2	37.9077625	0.232649867	0.228400454
ENSRNOG00000017069	Mark4	29.1704982	-0.583160237	0.604777869
ENSRNOG00000025459	Mars1	24.12800777	-0.0315208	0.294651319
ENSRNOG00000042620	Marveld1	28.2694792	0.377926998	0.257082565
ENSRNOG00000018297	Marveld2	163.7986569	-0.054149249	0.106681558
ENSRNOG00000016291	Marveld3	105.5752007	0.094496494	0.168270975
ENSRNOG00000014971	Mas1	94.29923232	-0.191978617	0.169488683
ENSRNOG00000011258	Masp2	132.9310343	0.183516329	0.121946146
ENSRNOG00000003469	MAST1	37.69439989	0.03491988	0.221866676
ENSRNOG00000022753	Mast3	61.71351939	0.34435247	0.168541561
ENSRNOG00000027002	Mast4	10.09600144	0.159229599	0.419124101
ENSRNOG00000054474	Mastl	25.25386161	0.124362755	0.275547392
ENSRNOG00000013520	Mat2a	11.97207434	0.173523874	0.398862971
ENSRNOG00000006060	Matn2	22.25258557	-0.084434002	0.275841246
ENSRNOG00000014021	Matn4	9.823067122	0.304603781	0.437674843
ENSRNOG00000025295	Mavs	72.45842599	0.077244923	0.154388806
ENSRNOG00000024040	Mb21d2	126.4197107	0.104954924	0.120812853
ENSRNOG00000024104	Mbd1	12.05490385	0.046890943	0.374878213
ENSRNOG00000027596	Mbd5	100.926803	-0.06347579	0.145016064
ENSRNOG00000008610	Mbip	48.76645096	-0.023678947	0.195059069
ENSRNOG00000016252	Mblac2	127.7535105	0.014462119	0.128760526
ENSRNOG00000014076	Mbnl1	9.518732257	-0.298470381	0.472307475
ENSRNOG00000052600	Mboat7	54.08390561	0.52923308	0.216494969
ENSRNOG00000049988	Mbtd1	28.77969481	0.09983306	0.302190454
ENSRNOG00000007644	Mbtps2	51.58987798	0.142098601	0.18537804

ENSRNOG00000038193	Mc1r	7.970410527	0.440825528	0.487833752
ENSRNOG00000018692	Mc4r	28.10111727	-0.781274619	0.257318111
ENSRNOG00000010539	Mcat	16.3677841	0.483126439	0.315273833
ENSRNOG00000011271	Mcc	115.9713889	-0.066929701	0.147630817
ENSRNOG00000013293	Mccc1	61.19702039	-0.027827654	0.164418626
ENSRNOG00000017752	Mccc2	150.7025849	-0.046285953	0.117280539
ENSRNOG00000016327	Mcee	110.6162961	-0.172533188	0.125448333
ENSRNOG00000028426	Mcf2l	98.21670681	0.298057993	0.135546448
ENSRNOG00000018895	Mchr1	128.628693	0.087057364	0.151716559
ENSRNOG00000016316	Mcm2	15.77659398	0.259908356	0.341874466
ENSRNOG00000001272	Mcm3ap	139.5859401	0.07106984	0.111222148
ENSRNOG00000001833	Mcm4	48.70095202	-0.039636478	0.180516331
ENSRNOG00000020394	Mcmbp	17.42204916	-0.013083524	0.338826289
ENSRNOG00000000975	Mcoln1	30.47640953	-0.189707513	0.233152416
ENSRNOG00000036691	Mcrip1	40.43193315	0.083896834	0.198833638
ENSRNOG00000013282	Mctp1	33.97214606	0.144991245	0.245277945
ENSRNOG00000009932	Mctp2	12.9421936	0.122956096	0.359267255
ENSRNOG00000045920	Mcu	9.361538228	-0.302514194	0.407105734
ENSRNOG00000017988	Mcur1	14.34397058	-0.479811697	0.392049951
ENSRNOG00000053787	Mdfic	11.27580978	0.03134585	0.388187769
ENSRNOG00000000536	Mdga1	15.88488353	0.42978066	0.355118608
ENSRNOG00000000618	Mdga2	87.46456395	-0.111389323	0.186722444
ENSRNOG00000012891	Mdh1b	13.9439454	0.294363561	0.385428805
ENSRNOG00000007286	Mdm1	27.76835114	0.173121306	0.24555501
ENSRNOG00000006304	Mdm2	32.70069595	-0.055454077	0.223708301
ENSRNOG00000009696	Mdm4	30.89113822	0.10235466	0.294711194
ENSRNOG00000047513	Mdn1	44.68905795	0.47610713	0.210598862
ENSRNOG00000019840	Mdp1	118.2537497	-0.064310459	0.120210555
ENSRNOG00000015582	Me2	82.11922965	0.173375928	0.156369997
ENSRNOG00000017144	Mea1	52.28670569	-0.914297027	1.239319566
ENSRNOG00000016224	Meak7	21.04579078	0.077548366	0.313440591
ENSRNOG00000012645	Mecom	11.83041804	0.437516552	0.37180257
ENSRNOG00000056659	Mecp2	10.18614069	0.177434917	0.466088823
ENSRNOG00000028047	Mecr	39.62359424	-0.026725725	0.209750404
ENSRNOG00000005606	Med1	47.72467841	0.323098077	0.208050101
ENSRNOG00000010680	Med12l	19.4197508	0.522368657	0.333923938
ENSRNOG00000003679	Med13	66.538035	0.380807282	0.179199486
ENSRNOG00000003792	Med14	19.43955645	-0.083105097	0.276326767
ENSRNOG00000001877	Med15	35.60046246	0.63131062	0.224638521
ENSRNOG00000013852	Med20	8.14931783	0.116184095	0.484709948
ENSRNOG00000001820	Med21	110.8444904	-0.210932425	0.130590956
ENSRNOG00000005059	Med22	134.9781396	0.203031194	0.128087688
ENSRNOG00000013422	Med23	16.78207653	0.376904023	0.324647868
ENSRNOG00000012270	Med26	43.56265095	-0.247264273	0.206724992
ENSRNOG00000013933	Med27	153.5241669	0.041942041	0.121003356
ENSRNOG00000003592	Med28	21.15728666	0.175432163	0.288245883
ENSRNOG00000019702	Med29	156.1996377	-0.074882061	0.13562914
ENSRNOG00000004844	Med30	132.0316356	0.205670901	0.118209394
ENSRNOG00000017170	Med4	29.68023079	-0.025440354	0.252915578
ENSRNOG00000006976	Med6	48.21296724	-0.271972235	0.184637371

ENSRNOG00000007053	Med7	72.66543487	0.076404817	0.173570231
ENSRNOG00000028477	Med8	40.20411254	-0.090575851	0.204886614
ENSRNOG00000000906	Medag	132.0199892	0.048905711	0.142514872
ENSRNOG00000047756	Mef2a	175.5377644	-0.0879701	0.122264891
ENSRNOG00000020400	Mef2b	35.21167968	-0.178342786	0.311955679
ENSRNOG00000031778	Mef2d	33.0750501	-0.023305403	0.207751978
ENSRNOG00000013674	Megf10	27.78294446	0.023556781	0.23827598
ENSRNOG00000010634	Megf11	166.7166233	0.050344293	0.105280393
ENSRNOG00000052687	Megf8	55.91251073	0.333074046	0.214741473
ENSRNOG00000005932	Megf9	31.02563535	0.088483099	0.231200922
ENSRNOG00000023794	Mei4	8.913813949	0.009398633	0.433429322
ENSRNOG00000014711	Meiob	32.8304897	-0.007329529	0.313995813
ENSRNOG00000004606	Meis1	10.83569543	0.412928196	0.372385688
ENSRNOG00000021390	Meis3	182.2829818	-0.108490802	0.112000204
ENSRNOG00000001739	Meltf	64.58942212	-0.261745522	0.171915969
ENSRNOG00000006340	Memo1	176.2310712	0.011164462	0.123970558
ENSRNOG00000002154	Mepe	20.25718527	-0.488977407	0.285834818
ENSRNOG00000017319	Mertk	115.3393243	-0.083357043	0.135634616
ENSRNOG00000012947	Metap1	63.26828713	-0.027291781	0.161610312
ENSRNOG00000061587	Metap1d	46.84781876	0.063449595	0.179059377
ENSRNOG00000021881	Metap2	51.27941517	0.400279467	0.176755766
ENSRNOG00000046202	Metrnl	31.44163961	-0.227273248	0.282094775
ENSRNOG00000015250	Mettl14	21.15470935	-0.062292412	0.275209582
ENSRNOG00000002764	Mettl16	51.03196501	-0.075703594	0.189919168
ENSRNOG00000025189	Mettl18	14.00759787	-0.237521729	0.364445343
ENSRNOG00000014212	Mettl21a	40.60993118	-0.058522159	0.207604951
ENSRNOG00000002714	Mettl22	140.9267031	-0.12524729	0.110873084
ENSRNOG00000027451	Mettl25	25.2735123	0.264324085	0.298968798
ENSRNOG00000013111	Mettl3	63.14187786	0.234522032	0.168678974
ENSRNOG00000008469	Mettl5	89.79067337	-0.089274128	0.158247893
ENSRNOG00000052391	Mettl6	10.25948802	0.511874399	0.430810687
ENSRNOG00000025142	Mex3b	109.2538825	-0.009227434	0.133072501
ENSRNOG00000015777	Mex3c	123.8777265	0.272552931	0.146396242
ENSRNOG00000030830	Mex3d	71.47338448	-0.196874066	0.17788307
ENSRNOG00000008257	Mfap2	37.85479345	0.067086995	0.232341851
ENSRNOG00000002443	Mfap3	42.28824495	-0.654822514	0.305532749
ENSRNOG00000011775	Mfap3l	30.02688127	0.15156211	0.236234851
ENSRNOG00000011431	Mfhas1	20.77517514	-0.631685208	0.350427859
ENSRNOG00000046424	Mfn2	85.03657591	0.283674339	0.16130227
ENSRNOG00000008133	Mfng	46.99790739	0.226555537	0.194645625
ENSRNOG00000013790	Mfsd1	53.27695161	0.07795298	0.209601469
ENSRNOG00000000247	Mfsd11	127.4949792	0.038046379	0.119011518
ENSRNOG00000019674	Mfsd13a	17.39255847	-0.113350779	0.341848898
ENSRNOG00000018227	Mfsd14b	75.19425209	-0.039258434	0.157993302
ENSRNOG00000014008	Mfsd2a	83.96731114	-0.073152225	0.178676618
ENSRNOG00000023508	Mfsd2b	116.9765857	0.055049106	0.123668475
ENSRNOG00000024657	Mfsd4a	46.46962397	-0.247774105	0.188641826
ENSRNOG00000024543	Mfsd4b	28.85539696	-0.233899117	0.240386152
ENSRNOG00000012729	Mfsd8	9.503914161	0.424636154	0.451657493
ENSRNOG00000023129	Mfsd9	42.26490834	-0.050942756	0.203927466

ENSRNOG00000006378	Mga	33.4037763	0.293960667	0.221380094
ENSRNOG00000026177	Mgam	26.91069669	0.252584192	0.263291351
ENSRNOG00000011923	Mgarp	10.50807258	-0.336364506	0.410650201
ENSRNOG00000004234	Mgat2	31.59322044	-0.485847249	0.236827467
ENSRNOG00000017434	Mgat3	11.8039992	-0.055330212	0.385038711
ENSRNOG00000018276	Mgat4a	17.85277906	-0.250301646	0.303532923
ENSRNOG00000003235	Mgat4b	12.21638618	0.088037693	0.351166925
ENSRNOG00000004149	Mgat4c	21.28787777	0.05347764	0.279792842
ENSRNOG00000003614	Mgat5	48.08021692	0.114405401	0.18669049
ENSRNOG00000019542	MGC108823	9.493453223	0.451915307	0.483130823
ENSRNOG00000033341	MGC116197	29.57907372	-0.141628356	0.250409653
ENSRNOG00000011243	MGC94891	143.5464889	0.007446667	0.13852151
ENSRNOG00000000720	MGC95208	38.98123402	0.056673971	0.240049118
ENSRNOG00000024780	MGC95210	21.46187153	0.056337339	0.282858556
ENSRNOG00000028196	Mgme1	11.81902913	0.181032949	0.372036531
ENSRNOG00000016038	Mgmt	13.99367029	-0.340333405	0.35082121
ENSRNOG00000005695	Mgp	106.4423682	0.088302591	0.163656825
ENSRNOG00000003234	Mgrn1	39.97131885	-0.070080713	0.225894758
ENSRNOG00000007743	Mgst1	20.80613348	0.332507466	0.292337921
ENSRNOG00000061857	Mgst2	14.55639131	-0.079985633	0.324261799
ENSRNOG00000005101	Mia2	43.79759032	0.275468104	0.22729961
ENSRNOG00000059202	Mia3	33.35853066	-0.158411205	0.213315102
ENSRNOG00000013281	Mib1	93.49051612	-0.04848006	0.161526399
ENSRNOG00000017564	Mib2	29.25969738	-0.159191026	0.236551218
ENSRNOG00000053203	Mical3	22.38342062	0.348412232	0.306122315
ENSRNOG00000026212	Micall1	171.6924836	-0.084577534	0.096850965
ENSRNOG00000022533	Micall2	8.640850432	-0.247483749	0.439763943
ENSRNOG00000046002	Micos13	22.46331537	-0.345376187	0.311010811
ENSRNOG00000011168	Micu2	162.3415461	0.160334845	0.111885853
ENSRNOG00000012767	Micu3	102.430771	-0.116900637	0.152093522
ENSRNOG00000003613	Mid1	25.47744808	0.378458218	0.266883403
ENSRNOG00000060542	Mid2	30.66662782	0.254266393	0.257747337
ENSRNOG00000010653	Mideas	8.636783648	-0.448812302	0.434118364
ENSRNOG00000015434	Midn	37.94847023	-0.215845219	0.227313786
ENSRNOG00000017553	Mief1	25.87076315	0.078924742	0.243898956
ENSRNOG00000007227	Mien1	61.22297652	-0.14353954	0.997706024
ENSRNOG00000007175	Mier1	150.6433748	-0.008649741	0.122755635
ENSRNOG00000013121	Mier3	75.45268848	0.016809877	0.155856048
ENSRNOG00000056332	Miga1	21.78428771	0.252875265	0.316098986
ENSRNOG00000017513	Miga2	25.27362696	0.184006736	0.273186263
ENSRNOG00000061337	Mindy2	177.4971786	0.009620378	0.155520131
ENSRNOG00000011530	Mindy4	14.03862772	0.552559745	0.344795471
ENSRNOG00000033508	Mink1	69.25389716	0.175046071	0.198828629
ENSRNOG00000007924	Mios	43.40658471	0.159707425	0.193852773
ENSRNOG00000013876	Mipep	70.07464162	-0.130680799	0.156720849
ENSRNOG00000035499	Mir124-2	15.30604992	0.539040087	0.364334261
ENSRNOG00000035593	Mir124-3	22.19151696	-0.135024731	0.263644256
ENSRNOG00000035641	Mir138-1	29.7378819	-0.088083302	0.305823388
ENSRNOG00000035480	Mir21	7.958070745	0.476036517	0.541875709
ENSRNOG00000035627	Mir325	28.36832337	0.558489244	0.306731288

ENSRNOG00000035580	Mir3597-3	37.11208665	-0.090713903	0.234316937
ENSRNOG00000035615	Mir370	8.37029755	-0.318931279	0.489393547
ENSRNOG00000035555	Mir384	16.61269477	0.204575282	0.323359171
ENSRNOG00000047361	Mir6329	115.5037035	-0.002837797	0.126669997
ENSRNOG00000023093	Mis18bp1	11.3763271	0.467708891	0.470464504
ENSRNOG00000018467	Mitd1	9.06826488	-0.330560854	0.431224919
ENSRNOG00000008658	Mitf	26.91354028	0.491010568	0.26552803
ENSRNOG00000031653	Mix23	26.29893515	-0.344142887	0.232317896
ENSRNOG00000028137	Mki67	42.21535487	0.226870017	0.244505824
ENSRNOG00000054514	Mkln1	74.00446911	0.060469528	0.165618716
ENSRNOG00000010381	Mknk1	50.7534094	-0.206480664	0.205720648
ENSRNOG00000032871	Mlc1	14.23041409	-0.525227136	0.326665379
ENSRNOG00000021725	Mlec	41.22225655	-0.172690409	0.213191074
ENSRNOG00000016589	Mlf2	70.01140523	0.273304593	0.158512514
ENSRNOG00000033809	Mlh1	67.38645694	-0.126882214	0.160642603
ENSRNOG00000006699	Mlh3	75.31633517	0.021756516	0.17761323
ENSRNOG00000005934	Mlip	64.15167144	0.145126713	0.165538236
ENSRNOG00000022249	Mllt10	62.62227737	0.094035721	0.173824701
ENSRNOG00000011280	Mllt3	97.15997146	0.23526081	0.139519602
ENSRNOG00000053285	Mllt6	26.98277154	0.107747584	0.368623703
ENSRNOG00000012789	Mlnr	9.98627361	-0.161209978	0.390085646
ENSRNOG00000014522	Mlycd	9.950270821	0.055972441	0.427120339
ENSRNOG00000011618	Mmaa	52.85102946	0.034845903	0.18233823
ENSRNOG00000049426	Mmab	35.38997701	0.09738608	0.224567836
ENSRNOG00000004740	Mmadhc	107.556018	0.020747838	0.13584389
ENSRNOG00000012593	Mmel1	9.587463897	0.103563576	0.391269177
ENSRNOG00000028344	Mmp11	10.59284771	0.445258786	0.45902511
ENSRNOG00000010947	Mmp14	18.88286882	1.208842824	0.489594945
ENSRNOG00000012622	Mmp15	165.5170977	0.117569086	0.112130372
ENSRNOG00000005708	Mmp16	53.3339118	-0.080003684	0.197681314
ENSRNOG00000016695	Mmp2	12.57845154	-0.37267536	0.373571993
ENSRNOG00000017756	Mmp21	11.81575985	0.179666558	0.427962008
ENSRNOG00000047940	Mmp28	31.41151398	-0.106512502	0.28679483
ENSRNOG00000051977	Mmrn2	36.78258924	0.116334973	0.295027813
ENSRNOG00000046769	Mms19	15.00983621	-0.182316631	0.342425659
ENSRNOG00000007267	Mnat1	25.59294201	-0.448856326	0.300034054
ENSRNOG00000024733	Mnd1	9.747907732	0.179238283	0.425928426
ENSRNOG00000002894	Mnt	35.54378649	0.195686003	0.221075792
ENSRNOG00000059474	Mob1a	51.78948164	0.057744526	0.229260501
ENSRNOG00000018971	Mob3a	45.00406901	-0.083782001	0.218700275
ENSRNOG00000009453	Mob3b	20.1236284	0.282371511	0.280257813
ENSRNOG00000011784	Mocs1	98.05902316	0.085381233	0.148407556
ENSRNOG00000025067	Mocs3	38.03732555	-0.229425199	0.210510118
ENSRNOG00000000775	Mog	44.98370678	0.145513393	0.210404088
ENSRNOG00000014692	Mogat1	18.54072106	0.178630537	0.362142049
ENSRNOG00000027228	Mogat2	14.46875821	0.385549369	0.387275681
ENSRNOG00000007850	Mok	18.09053068	-0.106584455	0.309321922
ENSRNOG00000018502	Mon1a	26.79373622	-0.006675609	0.266469674
ENSRNOG00000004185	Mon2	72.9428924	-0.114368333	0.149884176
ENSRNOG00000032101	Morc1	92.39914749	-0.087061517	0.147849169

ENSRNOG00000019624	Morc2	72.68241856	0.172308231	0.178374218
ENSRNOG00000026236	Morc3	98.47174642	0.157920351	0.15993579
ENSRNOG00000014642	Morn1	47.35680759	0.288588437	0.216861408
ENSRNOG00000045574	Morn3	8.139080923	-0.043723494	0.48399345
ENSRNOG00000027156	Morn4	51.34363572	0.134499626	0.450596949
ENSRNOG00000026111	Morn5	63.18269451	0.038699496	0.164806998
ENSRNOG00000058316	Mospd1	54.38245473	0.038755886	0.182923352
ENSRNOG00000003350	Mospd2	39.22234579	0.256978721	0.197763074
ENSRNOG00000001396	Mospd3	143.4971136	-0.212495219	0.126430971
ENSRNOG00000012946	Mov10	34.23893094	0.237337864	0.221770979
ENSRNOG00000015321	Moxd1	90.4418998	-0.371684842	0.151720841
ENSRNOG00000007894	Mpdz	152.7763035	0.153433692	0.118962809
ENSRNOG00000020571	Mpg	26.66010479	0.035494652	0.260429123
ENSRNOG00000016266	Mphosph10	18.2157275	-0.269013723	0.312111412
ENSRNOG00000050087	Mphosph6	126.7281194	0.163280452	0.154035125
ENSRNOG00000001074	Mphosph9	42.85258001	-0.047126607	0.199924353
ENSRNOG00000013806	Mplkip	63.89860856	-0.039946252	0.188937666
ENSRNOG00000048282	Mpnd	15.88489931	-0.584009801	0.680522859
ENSRNOG00000059683	Mpp2	35.4850555	0.074577027	0.227028349
ENSRNOG00000010486	Mpp4	16.10194848	0.509348513	0.330285954
ENSRNOG00000008788	Mpp5	43.1691525	0.004548067	0.198393048
ENSRNOG00000000185	Mpst	131.9053891	-0.087506984	0.124474754
ENSRNOG00000049430	Mpv17	71.47149189	0.14318775	0.168814814
ENSRNOG00000003285	Mpv17l	16.401917	-0.582056554	0.340854014
ENSRNOG00000018251	Mrc1	14.80446784	-0.122941513	0.365726668
ENSRNOG00000006548	Mrc2	16.75681243	-0.055682154	0.330626045
ENSRNOG00000009506	Mre11	44.32458443	0.308780166	0.229355962
ENSRNOG00000015774	Mreg	24.86724287	-0.313313436	0.321057887
ENSRNOG00000013426	Mrgprf	9.248610661	0.236241631	0.515332662
ENSRNOG00000026211	Mri1	11.17246277	-0.766743847	0.352980841
ENSRNOG00000027901	Mrm1	49.26411346	0.218171376	0.197412861
ENSRNOG00000043267	Mrm2	30.38230917	0.113295951	0.233207033
ENSRNOG00000037238	Mrm3	66.25255714	-0.145618657	0.152142471
ENSRNOG00000003068	Mrnip	12.39889891	1.492460873	0.567531708
ENSRNOG00000030355	Mroh1	30.98994668	0.707613171	0.269878466
ENSRNOG00000007341	Mroh8	20.19671772	0.089773656	0.305247671
ENSRNOG00000002070	Mrpl1	41.30638762	-0.14093311	0.214884043
ENSRNOG00000019970	mrpl11	97.62724569	-0.017882024	0.167865672
ENSRNOG00000036695	Mrpl12	26.11842817	0.098444618	0.242515632
ENSRNOG00000004401	Mrpl13	155.2604752	-0.428200302	0.100327666
ENSRNOG00000019734	Mrpl14	163.7698493	0.091494048	0.107927331
ENSRNOG00000021005	Mrpl16	79.34947341	0.088981354	0.175196531
ENSRNOG00000006968	Mrpl19	14.49773705	-0.055172526	0.343802041
ENSRNOG00000018647	Mrpl20	105.0620684	-0.012360468	0.146871003
ENSRNOG00000013845	Mrpl21	86.90217242	-0.285193292	0.14271933
ENSRNOG00000027039	Mrpl22	10.39892413	0.348535414	0.445426156
ENSRNOG00000003724	Mrpl27	39.66374967	0.016994038	0.214427438
ENSRNOG00000012650	Mrpl3	38.95756298	0.183255753	0.221345243
ENSRNOG00000015989	Mrpl32	122.521909	-0.272078547	0.14208992
ENSRNOG00000008546	Mrpl35	100.0820304	0.037723606	0.15421106

ENSRNOG00000008256	Mrpl38	31.09176949	-0.294684278	0.392342678
ENSRNOG00000047563	Mrpl39	83.82909443	-0.324684263	0.158839163
ENSRNOG00000049686	Mrpl40	169.4740697	-0.023298719	0.099999967
ENSRNOG00000042740	Mrpl42	180.2054077	-0.252689787	0.141663923
ENSRNOG00000018547	Mrpl46	75.2685444	-0.262980622	0.150396228
ENSRNOG00000019165	Mrpl51	59.50157349	-25.32681113	3.14474057
ENSRNOG00000039297	Mrpl52	17.24057261	0.302749988	0.3093529
ENSRNOG00000002943	Mrpl55	27.74006648	-0.079965037	0.238098611
ENSRNOG00000032780	Mrpl58	66.12380024	0.145576437	0.164809317
ENSRNOG00000020869	mrpl9	54.00721116	-0.055893742	0.173887236
ENSRNOG00000022609	Mrps10	68.99507239	-0.292330846	0.206290033
ENSRNOG00000018531	Mrps11	183.1989187	-0.219171234	0.129564637
ENSRNOG00000019949	Mrps12	45.45415282	-0.043207416	0.877498657
ENSRNOG00000002569	Mrps14	33.14891376	-0.216229736	0.212442103
ENSRNOG00000008279	Mrps15	95.11584673	0.03982346	0.128547028
ENSRNOG00000000926	Mrps17	27.25342942	0.086237824	0.250312067
ENSRNOG00000019511	Mrps18a	17.91327506	0.001269221	0.286809511
ENSRNOG00000002178	Mrps18c	14.76583999	0.534107399	0.483201344
ENSRNOG00000024845	Mrps21	46.82320249	0.011047686	0.192318794
ENSRNOG00000010363	Mrps23	121.0799394	-0.155728161	0.140652832
ENSRNOG00000017272	Mrps27	174.9150346	-0.153290761	0.109414122
ENSRNOG00000032630	Mrps28	136.7917821	-0.233608345	0.123235524
ENSRNOG00000011839	Mrps31	80.60775535	0.242859521	0.160038722
ENSRNOG00000001842	Mrps35	117.7619032	-0.023859755	0.139376356
ENSRNOG00000016201	Mrps9	177.9565463	-0.110364254	0.097706434
ENSRNOG00000025997	Mrrf	81.77753076	-0.048965993	0.138433464
ENSRNOG00000018803	Mrtfa	25.03838081	0.259616845	0.290508786
ENSRNOG00000008159	Msantd3	84.56889936	0.109353078	0.13797903
ENSRNOG00000022245	Msantd4	167.7358277	-0.034896259	0.109445191
ENSRNOG00000015796	Msh2	21.69154419	0.509791421	0.328978101
ENSRNOG00000025338	Msi2	33.57991679	-0.324880956	0.208912061
ENSRNOG00000023021	Msl2	55.22835914	0.061874521	0.221151389
ENSRNOG00000004016	Msl3	132.8704964	-0.13678485	0.116670072
ENSRNOG00000000801	Msl3l2	26.77871445	-0.038083588	0.303496375
ENSRNOG00000030118	Msn	77.43512369	0.122941841	0.171122419
ENSRNOG00000012440	Msra	9.169986677	-0.013130701	0.494267359
ENSRNOG00000055314	Msrb1	136.7206533	-0.273283623	0.130491962
ENSRNOG00000016873	Msrb2	99.24652081	0.018122555	0.154000272
ENSRNOG00000042668	Msrb3	19.78124272	-0.254470452	0.289950677
ENSRNOG00000028841	Mt1m	92.96569355	-0.372486219	0.153057368
ENSRNOG00000004711	Mta1	23.15788302	0.134978496	0.27159352
ENSRNOG00000004685	Mta3	88.16917185	-0.039611285	0.159623172
ENSRNOG00000006615	Mtap	20.65059808	-0.060010274	0.284844187
ENSRNOG00000037850	Mtarc1	100.7903181	-0.055041985	0.170667716
ENSRNOG00000025527	Mtcl1	131.5900489	0.117403281	0.153890744
ENSRNOG00000007899	Mterf1	39.5526629	-0.203667242	0.20386926
ENSRNOG00000004492	Mterf3	69.41287965	0.234643002	0.159808297
ENSRNOG00000037376	Mterf4	28.82554453	0.05895619	0.252768174
ENSRNOG00000025724	Mtf1	80.6973846	0.092011104	0.1528135
ENSRNOG00000000075	Mtf2	63.00218521	0.003690509	0.170804957

ENSRNOG00000014602	Mtfmt	78.55277548	0.10701647	0.15520428
ENSRNOG00000004640	Mtfp1	127.9509468	0.054908869	0.117172289
ENSRNOG00000021359	Mtfr1	9.578286153	0.922996414	0.430098331
ENSRNOG00000005602	Mthfd1	90.60499472	0.095822234	0.14909847
ENSRNOG00000019582	Mthfd1l	77.43236599	0.181437062	0.209790742
ENSRNOG00000021347	Mthfd2l	11.5495966	0.346738777	0.395798453
ENSRNOG00000008553	Mthfr	7.693162971	-0.012792614	0.49031806
ENSRNOG00000013229	Mthfs	33.91083979	-0.02873637	0.230122779
ENSRNOG00000004161	Mtif2	176.502781	-0.111293065	0.105439051
ENSRNOG00000002516	Mtm1	68.73907009	0.156628058	0.153027261
ENSRNOG00000016090	Mtmr10	52.11517415	0.217184229	0.188107081
ENSRNOG00000021176	Mtmr11	29.9417916	0.153373765	0.258911299
ENSRNOG00000022929	Mtmr12	91.8300491	0.25743559	0.178639183
ENSRNOG00000007496	Mtmr4	21.80593209	0.372072253	0.301734034
ENSRNOG00000011420	Mtmr7	143.877469	0.165692321	0.114853573
ENSRNOG00000009615	Mtor	25.35469827	0.342081491	0.28370825
ENSRNOG00000016578	Mtpap	117.2967206	-0.01840055	0.132319213
ENSRNOG00000011857	Mtpn	32.3735007	0.190711915	0.214801198
ENSRNOG00000017593	Mtr	58.50553007	-0.070430117	0.176353519
ENSRNOG00000047118	Mtres1	115.8092497	-0.140695985	0.14833714
ENSRNOG00000010125	Mtrex	63.63006013	0.097674718	0.18355832
ENSRNOG00000047106	Mtrf1	39.66932223	-0.077796762	0.22576477
ENSRNOG00000018773	Mtrf1l	165.9437476	-0.085657862	0.11065167
ENSRNOG00000001073	Mtrfr	37.78936152	0.087126018	0.243822765
ENSRNOG00000017826	Mtrr	39.71573352	0.13827638	0.219979162
ENSRNOG00000009001	Mtss1	23.44449123	0.164055122	0.314829781
ENSRNOG00000010205	Mturn	32.32764698	-0.229202013	0.248014206
ENSRNOG00000032410	Mtus2	46.776759	-0.148118307	0.180891491
ENSRNOG00000004703	Muc15	26.09130411	-0.470878662	1.017595091
ENSRNOG00000016010	Mul1	118.7247868	-0.022834736	0.127955505
ENSRNOG00000017369	Mustn1	122.5674513	-0.175481603	0.128173973
ENSRNOG00000017949	Mvb12a	123.6120286	-0.117091373	0.115003334
ENSRNOG00000049604	Mvk	24.54602995	-0.209014635	0.273506407
ENSRNOG00000020182	Mvp	153.5352364	0.358057847	0.185545634
ENSRNOG00000001959	Mx1	29.36866099	0.102599829	0.230827656
ENSRNOG00000017720	Mxd1	123.2029328	0.024467963	0.13269183
ENSRNOG00000015033	Mxd4	146.9022628	-0.030037959	0.149736722
ENSRNOG00000042915	Mxra7	106.2581628	0.274786248	0.127242151
ENSRNOG00000053450	Myadm	36.92352913	-0.155595516	0.228738578
ENSRNOG00000021669	Mybl1	11.80171256	0.29794661	0.387804468
ENSRNOG00000056493	Mybpc1	12.85600179	-0.56250919	0.354351007
ENSRNOG00000019627	Mybpc2	11.19358912	-0.186972417	0.380967032
ENSRNOG00000014259	Mycl	85.19015247	0.101088922	0.155348468
ENSRNOG00000051372	Mycn	40.27661372	0.137746822	0.222665842
ENSRNOG00000013634	Myd88	28.07725138	-0.209270488	0.243911455
ENSRNOG00000005258	Myef2	30.38084467	0.491096757	0.244235294
ENSRNOG00000013343	Myg1	13.19019641	0.344308121	0.38751675
ENSRNOG00000057880	Myh11	14.18891396	0.098730114	0.399943881
ENSRNOG00000046276	Myh3	9.189453357	-0.27779065	0.419845273
ENSRNOG00000049695	Myh4	20.05719579	-0.421994892	0.336307812

ENSRNOG00000025757	Myh6	14.23092965	0.233175554	0.347474298
ENSRNOG00000049236	Myh9	23.79951411	0.279800492	0.346965847
ENSRNOG00000015278	Myl12a	20.53303476	-0.090841797	0.337916202
ENSRNOG00000050675	Myl4	17.82560169	-0.456591606	0.340922063
ENSRNOG00000017579	Mylip	79.53806932	0.030590634	0.150950034
ENSRNOG00000002215	Mylk	53.588491	0.177585802	0.181474283
ENSRNOG00000017645	Mylpf	8.431706367	0.210816923	0.461270437
ENSRNOG00000027923	Mynn	26.76978823	-0.094167693	0.268519222
ENSRNOG00000010161	Myo10	72.26481227	-0.165396308	0.190474258
ENSRNOG00000016483	Myo16	77.46574005	-0.263789009	0.146733454
ENSRNOG00000033101	Myo18a	75.80817386	0.288046752	0.154963245
ENSRNOG00000002852	Myo19	162.7175716	-0.100716528	0.118666475
ENSRNOG00000048152	Myo1b	26.67795894	0.298968953	0.262995712
ENSRNOG00000004072	Myo1c	8.653230309	0.53248353	0.527099629
ENSRNOG00000008409	Myo1f	19.1867874	0.413505871	0.36799415
ENSRNOG00000059140	Myo1g	14.69002128	0.713164743	0.38074487
ENSRNOG00000058866	Myo5a	187.6904921	-0.054057086	0.17095349
ENSRNOG00000008356	Myo5c	9.258913886	0.771960639	0.466034946
ENSRNOG00000011852	Myo6	47.22977945	0.292628872	0.20344547
ENSRNOG00000011619	Myo9a	74.14547769	-0.029239872	0.176243251
ENSRNOG00000016256	Myo9b	75.46488159	0.30523163	0.151972896
ENSRNOG00000016117	Myof	25.69118003	0.532052519	0.312275283
ENSRNOG00000014132	Mypop	10.36384944	0.103547987	0.404403622
ENSRNOG00000028274	Myrf	33.97692935	-0.212147857	0.262962822
ENSRNOG00000026299	Mysm1	8.419041471	-0.078702366	0.407688059
ENSRNOG00000017346	Myt1	9.917289537	0.229533011	0.394962646
ENSRNOG00000004269	Myt1l	175.0942194	0.237574401	0.100592881
ENSRNOG00000022009	Mzb1	9.518322958	0.467781479	0.441238945
ENSRNOG00000027550	Mzf1	47.09825177	0.015915883	0.201268943
ENSRNOG00000015121	N4bp1	64.8177574	-0.05578327	0.156418115
ENSRNOG00000059322	N4bp2l1	15.46883599	0.07817383	0.38376249
ENSRNOG00000001108	N4bp2l2	57.44550819	-0.038628715	0.172319917
ENSRNOG00000021731	N4bp3	98.91541073	-0.117519693	0.185549445
ENSRNOG00000001603	N6amt1	37.46049678	-0.072489739	0.238689312
ENSRNOG00000048230	NA	18.19609272	1.375896002	0.389011254
ENSRNOG00000053173	NA	15.71147571	4.719244791	1.414573882
ENSRNOG00000016193	NA	69.54469069	0.541582336	0.175973165
ENSRNOG00000061345	NA	12.24948461	1.216281552	0.404678484
ENSRNOG00000059601	NA	19.5310889	-0.844685624	0.284079268
ENSRNOG00000061552	NA	8.457656919	-1.343993625	0.457121572
ENSRNOG00000019778	NA	148.835031	0.393282712	0.13398988
ENSRNOG00000050384	NA	80.22580123	-0.40541659	0.140205129
ENSRNOG00000029278	NA	172.5866701	-0.350952085	0.122342669
ENSRNOG00000005209	NA	151.9004672	1.252292977	0.440129174
ENSRNOG00000019215	NA	123.8469736	-0.388526046	0.143987208
ENSRNOG00000060412	NA	10.80011164	-1.085513789	0.404318132
ENSRNOG00000051559	NA	129.7349047	0.316965331	0.122954561
ENSRNOG00000060657	NA	124.2510614	-0.408489256	0.159152498
ENSRNOG00000058166	NA	19.15685019	-0.762966878	0.298210019
ENSRNOG00000045545	NA	73.91095228	0.42864854	0.16939799

ENSRNOG00000004262	NA	29.68588403	-0.901437273	0.356635028
ENSRNOG00000049519	NA	21.1957959	1.15603536	0.469824127
ENSRNOG00000047992	NA	21.06740023	-1.722777218	0.70142917
ENSRNOG00000051617	NA	10.79967664	1.004282376	0.411993748
ENSRNOG00000018102	NA	85.86889412	-0.912597102	0.375522087
ENSRNOG00000046094	NA	133.8303863	-0.356162226	0.148485839
ENSRNOG00000054488	NA	34.1571059	0.527939735	0.22110099
ENSRNOG00000057097	NA	9.299659771	-0.961621534	0.403894158
ENSRNOG00000046308	NA	78.23289655	-0.334070121	0.140573782
ENSRNOG00000031951	NA	22.66257818	0.666007697	0.285844356
ENSRNOG00000057698	NA	11.5802271	-0.996683898	0.434536435
ENSRNOG00000048922	NA	44.08353239	-0.416470585	0.182193707
ENSRNOG00000004553	NA	54.84430386	0.514033149	0.224876375
ENSRNOG00000048561	NA	118.6796928	-0.285642282	0.12508221
ENSRNOG00000047821	NA	163.525108	0.240943563	0.106116026
ENSRNOG00000049740	NA	55.99906745	-0.531146924	0.235963862
ENSRNOG00000048545	NA	51.4795733	-0.530181558	0.235696836
ENSRNOG00000047387	NA	14.23853251	0.834870233	0.371162536
ENSRNOG00000014092	NA	43.27437345	0.453456148	0.202399517
ENSRNOG00000054990	NA	39.11667269	0.589965411	0.26545694
ENSRNOG00000028690	NA	132.8058872	-0.280953669	0.127007696
ENSRNOG00000049433	NA	42.76105111	0.580801671	0.262834609
ENSRNOG00000024149	NA	25.83793456	-0.623191633	0.282019465
ENSRNOG00000001484	NA	55.73966062	-0.590764023	0.267973832
ENSRNOG00000055201	NA	12.9185271	-1.802237467	0.825682389
ENSRNOG00000043100	NA	66.99556058	-0.377555547	0.173200027
ENSRNOG00000047911	NA	20.20780334	0.655875055	0.304797937
ENSRNOG00000057244	NA	8.202939548	1.121712438	0.525278193
ENSRNOG00000048894	NA	16.29829179	-0.892074741	0.421251567
ENSRNOG00000014205	NA	15.76998409	1.001748389	0.474784457
ENSRNOG00000001262	NA	8.192392885	1.137023947	0.539255077
ENSRNOG00000001756	NA	10.77897344	0.857877419	0.407405769
ENSRNOG00000025558	NA	69.59632085	0.43182441	0.205099692
ENSRNOG00000046937	NA	15.79180995	-0.888912679	0.424158984
ENSRNOG00000060927	NA	46.85832244	-0.412323424	0.197066503
ENSRNOG00000061236	NA	42.35292554	-0.430866596	0.207570655
ENSRNOG00000053541	NA	78.95860067	-0.294733047	0.142922142
ENSRNOG00000048357	NA	144.0650604	-0.258556302	0.12546172
ENSRNOG00000059220	NA	173.3124846	0.221587378	0.10805961
ENSRNOG00000039666	NA	11.86609035	0.850163617	0.416863285
ENSRNOG00000013228	NA	143.641741	-0.268879297	0.13203906
ENSRNOG00000045814	NA	51.32807723	-0.398249866	0.195852545
ENSRNOG00000059262	NA	10.51692459	0.939332487	0.462235185
ENSRNOG00000009137	NA	37.97187381	-0.436951132	0.215819681
ENSRNOG00000053808	NA	34.34909757	0.457635441	0.226081606
ENSRNOG00000046386	NA	17.10579591	-0.604277134	0.298860341
ENSRNOG00000047080	NA	156.740699	0.233985555	0.116342355
ENSRNOG00000047854	NA	56.0744646	-0.357502641	0.17983748
ENSRNOG00000047864	NA	55.89948545	-0.353406218	0.178539971
ENSRNOG00000060310	NA	40.72905001	-0.391852405	0.198615965

ENSRNOG00000036386	NA	12.90214113	0.884583397	0.452122135
ENSRNOG00000061018	NA	52.12971418	-0.324365429	0.167840845
ENSRNOG00000058744	NA	60.96570849	-0.318366807	0.164744716
ENSRNOG00000015267	NA	147.5920616	0.234399298	0.122792009
ENSRNOG00000059093	NA	10.35248136	0.872064485	0.458183479
ENSRNOG00000059149	NA	13.88994491	0.804845479	0.424688956
ENSRNOG00000042838	NA	99.99417151	-0.398717739	0.210436873
ENSRNOG00000048608	NA	40.85051378	-0.393058615	0.207820833
ENSRNOG00000050067	NA	11.31142018	-0.676370563	0.358595911
ENSRNOG00000045929	NA	8.332064434	0.999111451	0.532673133
ENSRNOG00000033244	NA	180.0364533	-0.200282523	0.106819857
ENSRNOG00000059467	NA	31.55750887	-0.430296363	0.23087757
ENSRNOG00000061562	NA	49.00777048	1.080628043	0.580015332
ENSRNOG00000037522	NA	32.61205042	0.491542002	0.264067343
ENSRNOG00000057672	NA	20.13630542	0.61188136	0.329333277
ENSRNOG00000010657	NA	16.92417706	-0.641575022	0.346596838
ENSRNOG00000053126	NA	92.5147878	0.278612313	0.151420657
ENSRNOG00000007392	NA	34.66594389	-0.45503224	0.248231871
ENSRNOG00000051753	NA	11.82998873	0.712179307	0.38860523
ENSRNOG00000061102	NA	21.37746719	0.565808513	0.309820784
ENSRNOG00000045785	NA	64.07215743	-0.316821421	0.173625351
ENSRNOG00000049751	NA	121.6421385	-0.246328503	0.135008699
ENSRNOG00000060047	NA	149.0539982	0.227338193	0.124880429
ENSRNOG00000050646	NA	132.7318624	0.223571401	0.123116263
ENSRNOG00000060813	NA	33.02598286	-0.451315462	0.249060268
ENSRNOG00000060827	NA	64.42965504	-0.303168998	0.169494818
ENSRNOG00000056265	NA	17.79875049	0.544538735	0.304883216
ENSRNOG00000054961	NA	18.13962197	-2.027109028	1.139790637
ENSRNOG00000000881	NA	124.4133097	0.235127198	0.132241787
ENSRNOG00000048834	NA	8.342060748	0.868920653	0.494213836
ENSRNOG00000046391	NA	76.64735863	0.26062375	0.148462161
ENSRNOG00000014552	NA	27.00849553	-0.464556243	0.264711234
ENSRNOG00000058634	NA	34.92786825	-0.399866634	0.229120726
ENSRNOG00000030522	NA	42.58239689	-0.470724241	0.270086939
ENSRNOG00000050485	NA	9.758068709	-0.925180773	0.531912979
ENSRNOG00000048831	NA	26.04117897	0.505956972	0.293905098
ENSRNOG00000051384	NA	11.85695662	0.647887129	0.377368671
ENSRNOG00000032542	NA	16.3468277	-0.533553507	0.311162172
ENSRNOG00000051731	NA	10.81931302	0.797257051	0.465698064
ENSRNOG00000050978	NA	8.528320692	0.859912723	0.502653022
ENSRNOG00000059734	NA	7.893638238	-0.90015754	0.526992884
ENSRNOG00000048471	NA	17.73847638	0.596103526	0.348999566
ENSRNOG00000058320	NA	35.72369701	1.942676165	1.137844987
ENSRNOG00000057085	NA	16.73492884	0.625713603	0.366925634
ENSRNOG00000052237	NA	10.20386909	-0.818780418	0.485692131
ENSRNOG00000059653	NA	98.08198767	-0.231051875	0.137402698
ENSRNOG00000059886	NA	47.90893501	-0.350208042	0.208272921
ENSRNOG00000046666	NA	19.88913171	-0.47873172	0.286672027
ENSRNOG00000051938	NA	12.96779729	-0.627424265	0.377267413
ENSRNOG00000011827	NA	172.0544382	-0.205163353	0.123733866

ENSRNOG00000039469	NA	26.07777403	0.419905628	0.253418899
ENSRNOG00000061059	NA	12.53856502	-0.587133016	0.357524735
ENSRNOG00000006939	NA	123.9687373	-0.206278336	0.126251582
ENSRNOG00000021084	NA	120.0741982	0.222697186	0.136399746
ENSRNOG00000029130	NA	26.92643378	-0.373173008	0.229627104
ENSRNOG00000059191	NA	8.266327655	-0.816273687	0.503682717
ENSRNOG00000046617	NA	58.52109594	0.320856049	0.198084531
ENSRNOG00000046712	NA	12.69644928	0.621030322	0.385148948
ENSRNOG00000041539	NA	8.12572892	0.915723863	0.570706679
ENSRNOG00000022718	NA	53.94970147	0.292198513	0.183014486
ENSRNOG00000062125	NA	9.290971746	-0.656098996	0.411466322
ENSRNOG00000010732	NA	189.6255192	0.229299117	0.144599263
ENSRNOG00000017306	NA	51.43869444	-0.2751484	0.173987927
ENSRNOG00000011304	NA	10.72736314	0.651195591	0.412282869
ENSRNOG00000019082	NA	176.6184477	-0.187125223	0.118664401
ENSRNOG00000047471	NA	21.0338634	-0.427539823	0.273493415
ENSRNOG00000037661	NA	70.22198323	-0.253845137	0.162391721
ENSRNOG00000061869	NA	19.44492377	-0.444890082	0.2849475
ENSRNOG00000025716	NA	37.74053321	-0.365362353	0.234278243
ENSRNOG00000050854	NA	24.68692523	0.403235564	0.258671937
ENSRNOG00000037997	NA	60.94065197	-0.263980209	0.171722406
ENSRNOG00000020641	NA	180.7005429	-0.189319285	0.123443275
ENSRNOG00000047281	NA	15.02223228	0.504116918	0.333072265
ENSRNOG00000028430	NA	13.47127133	0.603248344	0.39949726
ENSRNOG00000053609	NA	74.39978848	0.289747102	0.193511626
ENSRNOG00000057352	NA	76.36800517	-0.2206445	0.147825923
ENSRNOG00000058861	NA	8.621122333	0.712252128	0.478606005
ENSRNOG00000000887	NA	23.08571851	-0.423564894	0.28531602
ENSRNOG00000054459	NA	56.67686523	0.362643894	0.2452039
ENSRNOG00000022082	NA	37.32222851	-0.304061728	0.206597857
ENSRNOG00000000478	NA	61.86386193	-0.234344954	0.159601736
ENSRNOG00000013269	NA	12.58679669	0.537981831	0.367364654
ENSRNOG00000027040	NA	61.93387955	-0.271671689	0.185761197
ENSRNOG00000057851	NA	51.23701419	-0.249690755	0.17127978
ENSRNOG00000005408	NA	73.69906989	0.212754798	0.146093324
ENSRNOG00000014072	NA	33.13550992	0.346503859	0.23853109
ENSRNOG00000052436	NA	150.4502338	-0.153366517	0.105994916
ENSRNOG00000062184	NA	28.28410455	-0.365991267	0.25302392
ENSRNOG00000056515	NA	44.25773091	0.315867638	0.218412877
ENSRNOG00000005665	NA	8.853614341	0.654895057	0.45416361
ENSRNOG00000031474	NA	53.24299101	-0.247800566	0.172301408
ENSRNOG00000045680	NA	18.17889557	0.425542883	0.296631948
ENSRNOG00000057803	NA	10.37519979	0.642470346	0.448772432
ENSRNOG00000008360	NA	68.05020418	-0.245213033	0.171292399
ENSRNOG00000016952	NA	94.17249186	-0.18889368	0.132382823
ENSRNOG00000049939	NA	15.71356149	0.454958101	0.320790951
ENSRNOG00000051310	NA	10.4550513	0.639522494	0.452174983
ENSRNOG00000048085	NA	13.11895651	-0.473814551	0.335083853
ENSRNOG00000021986	NA	9.071443269	0.595664349	0.424190741
ENSRNOG00000058611	NA	11.49122778	0.597639179	0.425892324

ENSRNOG00000051364	NA	8.21960829	0.638660074	0.457674015
ENSRNOG00000055160	NA	7.784525147	0.796251299	0.572064587
ENSRNOG00000059138	NA	46.65697776	-0.835996357	0.606725032
ENSRNOG00000009596	NA	9.360204506	0.57598755	0.418919514
ENSRNOG00000034130	NA	132.1312294	-0.158291985	0.115262182
ENSRNOG00000051494	NA	24.17959579	0.369210961	0.269320224
ENSRNOG00000031939	NA	10.65801969	0.684125285	0.500804076
ENSRNOG00000046393	NA	18.75824219	-0.455234745	0.333524715
ENSRNOG00000039505	NA	16.97162215	0.429735945	0.315748325
ENSRNOG00000061155	NA	58.03813445	0.27951866	0.206314271
ENSRNOG00000031434	NA	8.05356733	0.71489282	0.528476094
ENSRNOG00000017740	NA	26.65608956	-0.321611926	0.238186208
ENSRNOG00000059428	NA	20.27128437	0.40986556	0.303814617
ENSRNOG00000059110	NA	11.3859748	-0.511559836	0.379844117
ENSRNOG00000051880	NA	52.60351466	0.252230744	0.18731737
ENSRNOG00000056731	NA	13.79268703	0.505192029	0.375346752
ENSRNOG00000061832	NA	18.54637451	-0.446238914	0.331753342
ENSRNOG00000017800	NA	38.98339375	0.288715113	0.214873023
ENSRNOG00000061432	NA	9.435033753	0.651210542	0.485465356
ENSRNOG00000058750	NA	37.78729339	0.293974181	0.221121606
ENSRNOG00000048963	NA	88.06098588	0.195328861	0.146968484
ENSRNOG00000047657	NA	78.0098495	0.208641913	0.157049359
ENSRNOG00000016071	NA	13.60898824	0.743631085	0.561095385
ENSRNOG00000016146	NA	9.157326462	0.577222636	0.436319888
ENSRNOG00000049255	NA	119.2210829	0.226165012	0.170963169
ENSRNOG00000048876	NA	108.6617667	-0.155794474	0.117824416
ENSRNOG00000008224	NA	97.38835552	-0.240088141	0.181696811
ENSRNOG00000057696	NA	170.6005186	-0.158759858	0.12024732
ENSRNOG00000053818	NA	52.11439557	0.292136184	0.221518284
ENSRNOG00000027694	NA	22.64737502	-0.339517521	0.257969214
ENSRNOG00000050497	NA	8.893073371	0.59600909	0.452855942
ENSRNOG00000010119	NA	8.404661694	-0.5257783	0.399976187
ENSRNOG00000058821	NA	26.9742967	0.337801608	0.25762587
ENSRNOG00000055496	NA	13.46143376	0.603132424	0.460038328
ENSRNOG00000054737	NA	9.12870915	0.556679468	0.425030543
ENSRNOG00000019351	NA	72.66915227	0.26782977	0.20464365
ENSRNOG00000051252	NA	58.73268071	0.226408292	0.173026378
ENSRNOG00000053206	NA	19.11039784	-0.396032387	0.305139839
ENSRNOG00000053665	NA	11.35104945	0.576340979	0.446586628
ENSRNOG00000012475	NA	84.61197117	-0.189808872	0.147706815
ENSRNOG00000058289	NA	90.3607666	-0.206975616	0.161421506
ENSRNOG00000034287	NA	16.69343465	-0.587363998	0.460637956
ENSRNOG00000047206	NA	34.59082538	0.279512037	0.219511157
ENSRNOG00000010468	NA	10.52193695	0.519620632	0.408318238
ENSRNOG00000002635	NA	12.13232535	0.500520477	0.393940121
ENSRNOG00000047683	NA	73.58532301	0.196643202	0.154969045
ENSRNOG00000038106	NA	37.55048355	-0.253556318	0.199826131
ENSRNOG00000014214	NA	172.3117296	-0.13144102	0.103893048
ENSRNOG00000057060	NA	10.6758819	0.501806357	0.396817378
ENSRNOG00000009835	NA	179.2717453	-0.122574981	0.096940598

ENSRNOG00000009917	NA	91.89110527	-0.179987232	0.142392546
ENSRNOG00000005718	NA	54.65059262	0.260647162	0.206232698
ENSRNOG00000044267	NA	10.38940638	-0.526958317	0.417953632
ENSRNOG00000062218	NA	100.3262822	0.175243456	0.139041371
ENSRNOG00000050741	NA	118.2067808	0.162576463	0.12911165
ENSRNOG00000047350	NA	59.03568242	-0.225145458	0.178993957
ENSRNOG00000001289	NA	13.16848534	0.46040427	0.366042682
ENSRNOG00000014700	NA	84.59463091	0.233201851	0.188552323
ENSRNOG00000051404	NA	34.201141	0.296274511	0.240079731
ENSRNOG00000015763	NA	67.83525644	-0.245077096	0.199080867
ENSRNOG00000047112	NA	10.42637393	0.507684758	0.412480603
ENSRNOG00000055247	NA	82.54273726	-0.230922464	0.18806109
ENSRNOG00000027286	NA	58.79332723	-0.228886758	0.186492829
ENSRNOG00000025385	NA	46.14324898	-0.260277963	0.212122926
ENSRNOG00000032840	NA	158.0744169	0.186362381	0.152797866
ENSRNOG00000050669	NA	75.00167139	0.532482117	0.438617017
ENSRNOG00000026302	NA	36.54241304	0.285353795	0.236462497
ENSRNOG00000025630	NA	58.4099312	-0.228442483	0.189630368
ENSRNOG00000058276	NA	9.503929533	0.555951167	0.462065618
ENSRNOG00000040715	NA	24.30611063	0.748232294	0.623434376
ENSRNOG00000055738	NA	21.25100515	0.368716018	0.307239093
ENSRNOG00000061122	NA	12.38116035	-0.494381991	0.412388787
ENSRNOG00000059244	NA	19.1198713	0.399926938	0.333684017
ENSRNOG00000049757	NA	17.70775277	0.389073529	0.325233998
ENSRNOG00000059834	NA	33.22740173	-0.288459425	0.241812205
ENSRNOG00000048130	NA	16.38216515	-0.372517948	0.312591723
ENSRNOG00000023151	NA	45.02429571	-0.246198213	0.207767891
ENSRNOG00000045919	NA	57.627426	-0.206074154	0.173985356
ENSRNOG00000057207	NA	34.88655243	0.28170098	0.238675357
ENSRNOG00000031660	NA	41.6458295	0.253316789	0.21511139
ENSRNOG00000016565	NA	69.19975155	0.195558345	0.166137845
ENSRNOG00000023261	NA	84.75226497	-0.178263087	0.151827056
ENSRNOG00000019736	NA	28.6250034	0.297738539	0.253692616
ENSRNOG00000027574	NA	112.9740165	0.152676519	0.130371718
ENSRNOG00000053067	NA	22.35758927	0.456362807	0.392037676
ENSRNOG00000011119	NA	42.31930476	0.284551315	0.244536103
ENSRNOG00000006591	NA	9.115228677	-0.530863674	0.456248271
ENSRNOG00000001834	NA	57.94341651	-0.206458565	0.178673424
ENSRNOG00000059013	NA	37.6342259	0.280695531	0.24372607
ENSRNOG00000029078	NA	43.59832857	0.223409488	0.194118855
ENSRNOG00000021960	NA	141.7329388	-0.144797955	0.125862981
ENSRNOG00000047689	NA	75.23894262	-0.205955364	0.179121285
ENSRNOG00000037426	NA	16.807276	-0.365093301	0.318037291
ENSRNOG00000043400	NA	65.69274489	-0.287979869	0.250994145
ENSRNOG00000053079	NA	34.19124693	-0.275767701	0.240817772
ENSRNOG00000048094	NA	18.60211759	0.390614675	0.341110169
ENSRNOG00000043366	NA	17.86562253	0.354075655	0.309325908
ENSRNOG00000023814	NA	9.041283095	-0.503340443	0.440634608
ENSRNOG00000051196	NA	20.27471744	0.422458801	0.370104524
ENSRNOG00000061031	NA	19.10062648	0.369731882	0.325324153

ENSRNOG00000062063	NA	24.45103691	-0.317433251	0.279777875
ENSRNOG00000051406	NA	9.116502882	-0.487508766	0.432750656
ENSRNOG00000046845	NA	139.5013796	0.139000884	0.124312232
ENSRNOG00000047790	NA	44.95084042	0.241544085	0.216786241
ENSRNOG00000049962	NA	46.80575773	0.300585152	0.269816205
ENSRNOG00000046740	NA	105.3455351	0.174128985	0.15723981
ENSRNOG00000053744	NA	51.48320901	0.213893135	0.19405392
ENSRNOG00000039587	NA	14.35357697	0.373976517	0.33934493
ENSRNOG00000061254	NA	98.1820936	-0.158305144	0.144117543
ENSRNOG00000047165	NA	178.1519291	0.130049792	0.118768026
ENSRNOG00000009371	NA	12.94693945	-0.411675222	0.3769521
ENSRNOG00000052964	NA	8.416708408	-0.47107926	0.432549165
ENSRNOG00000051080	NA	15.92789431	-0.388420785	0.358231129
ENSRNOG00000051851	NA	13.237802	0.405440024	0.376632539
ENSRNOG00000000639	NA	54.6161251	-0.189098573	0.176733927
ENSRNOG00000048121	NA	35.17234141	-0.238444154	0.223572043
ENSRNOG00000015999	NA	128.2480872	-0.194900358	0.183500494
ENSRNOG00000049380	NA	36.61255343	-0.242897158	0.229011658
ENSRNOG00000008548	NA	94.69829237	0.155178291	0.146455062
ENSRNOG00000056233	NA	20.12355014	0.326641431	0.308293364
ENSRNOG00000006341	NA	26.68130445	-0.261308609	0.246999351
ENSRNOG00000011226	NA	48.18396385	-0.216954337	0.205348344
ENSRNOG00000047934	NA	23.45962986	-0.293630307	0.278195372
ENSRNOG00000012801	NA	97.9400745	0.153518276	0.145529595
ENSRNOG00000037744	NA	11.60811638	0.410416729	0.389798535
ENSRNOG00000061051	NA	19.96296094	-0.360010844	0.342432742
ENSRNOG00000048933	NA	14.43600611	0.445274748	0.423621131
ENSRNOG00000048753	NA	27.23656915	-0.242882983	0.231333212
ENSRNOG00000022502	NA	68.55640158	-0.171081323	0.163066311
ENSRNOG00000031790	NA	105.2124812	-0.146429398	0.139763611
ENSRNOG00000029443	NA	138.791595	-0.142350723	0.136098898
ENSRNOG00000018061	NA	13.02005818	0.411589149	0.395585935
ENSRNOG00000061939	NA	8.322900771	0.716515586	0.688686888
ENSRNOG00000052357	NA	30.66975631	-0.293657058	0.282311706
ENSRNOG00000004791	NA	142.5262997	-0.138634152	0.133706975
ENSRNOG00000050181	NA	82.62146502	0.172288134	0.166897335
ENSRNOG00000038980	NA	124.8669725	-0.125152434	0.121569773
ENSRNOG00000000787	NA	11.99681625	0.39921365	0.388889595
ENSRNOG00000047250	NA	8.021754256	0.484144988	0.472134403
ENSRNOG00000060874	NA	15.47218719	0.377405094	0.369114691
ENSRNOG00000058319	NA	23.29356974	-0.315221872	0.308637514
ENSRNOG00000008605	NA	15.15181396	0.323116015	0.316677901
ENSRNOG00000003883	NA	64.52075001	-0.240820191	0.236177691
ENSRNOG00000046272	NA	16.24554553	0.375193586	0.369378476
ENSRNOG00000012677	NA	13.52621163	0.417024199	0.412292106
ENSRNOG00000028730	NA	27.61046484	0.25169439	0.24919221
ENSRNOG00000026208	NA	27.08324649	-0.255296104	0.254186917
ENSRNOG00000020693	NA	42.33818602	0.199934163	0.199335
ENSRNOG00000057476	NA	71.89649251	0.180613646	0.180161849
ENSRNOG00000034241	NA	58.27338731	-0.179373825	0.179525332

ENSRNOG00000030704	NA	182.8312155	-0.108090807	0.108286372
ENSRNOG00000032195	NA	9.889524702	0.410221591	0.41129862
ENSRNOG00000053948	NA	147.7028806	0.120930656	0.121294908
ENSRNOG00000047664	NA	31.27756989	-0.252585299	0.253496451
ENSRNOG00000060994	NA	9.948710948	0.415792351	0.417460689
ENSRNOG00000021364	NA	14.73330126	0.328150917	0.330143823
ENSRNOG00000055307	NA	8.794753232	-0.454414611	0.458942053
ENSRNOG00000054872	NA	15.14632043	-0.308805308	0.311940502
ENSRNOG00000045666	NA	78.6345048	-0.145984188	0.147904383
ENSRNOG00000053679	NA	12.23373282	0.386758409	0.392015057
ENSRNOG00000019932	NA	18.03619975	0.343658607	0.349177674
ENSRNOG00000060288	NA	37.29333609	-0.21246089	0.217319032
ENSRNOG00000015195	NA	11.83089858	0.470867318	0.482129057
ENSRNOG00000038678	NA	29.01695308	-0.234605725	0.240473412
ENSRNOG00000007115	NA	9.395307017	0.401714643	0.411827046
ENSRNOG00000060463	NA	10.74815748	0.430325791	0.441338685
ENSRNOG00000062290	NA	29.59032531	-0.215420561	0.221850907
ENSRNOG00000058779	NA	9.457523766	0.399859754	0.41221704
ENSRNOG00000049192	NA	41.92523796	0.187004675	0.193187207
ENSRNOG00000041744	NA	52.9548472	0.237092816	0.245026706
ENSRNOG00000002556	NA	84.71895603	-0.143322743	0.148262512
ENSRNOG00000057161	NA	11.24169748	0.430052367	0.444984408
ENSRNOG00000060574	NA	28.4664895	-0.230635757	0.239050473
ENSRNOG00000050396	NA	21.58304042	0.314325667	0.325974844
ENSRNOG00000049964	NA	14.83469577	0.325166458	0.337397897
ENSRNOG00000019585	NA	45.22050989	-0.1854424	0.192568596
ENSRNOG00000012088	NA	48.6398551	-0.179132997	0.186074138
ENSRNOG00000017900	NA	25.16477611	0.239915534	0.249360809
ENSRNOG00000010598	NA	161.1334736	0.126265855	0.131635933
ENSRNOG00000039576	NA	62.31608008	-0.167720402	0.175266281
ENSRNOG00000046527	NA	73.49826559	0.192958432	0.202270738
ENSRNOG00000054471	NA	14.28089676	0.348655843	0.366372628
ENSRNOG00000016848	NA	30.36484871	0.231252554	0.243157814
ENSRNOG00000045698	NA	25.79799769	-0.252182508	0.265210657
ENSRNOG00000055393	NA	64.67941793	0.170570223	0.179546463
ENSRNOG00000024505	NA	102.3399747	-0.120562117	0.127681457
ENSRNOG00000020368	NA	22.11837913	0.248576583	0.263976093
ENSRNOG00000019799	NA	102.674008	-0.124315302	0.132498368
ENSRNOG00000030330	NA	35.05322873	0.243536262	0.260122434
ENSRNOG00000013618	NA	53.61745116	-0.189522572	0.202771657
ENSRNOG00000057941	NA	19.38964349	-0.306437587	0.32794161
ENSRNOG00000004693	NA	80.73043372	-0.146853192	0.157191012
ENSRNOG00000052493	NA	9.757918473	-0.433450928	0.464395872
ENSRNOG00000051813	NA	21.7171826	-0.267234564	0.28908309
ENSRNOG00000026982	NA	10.74389619	-0.368988878	0.399347626
ENSRNOG00000059187	NA	68.25018974	0.195736747	0.212520104
ENSRNOG00000014538	NA	9.385574233	-0.36978941	0.402405813
ENSRNOG00000047605	NA	80.95987897	0.149762647	0.163244077
ENSRNOG00000015936	NA	23.92685462	-0.243610411	0.266056703
ENSRNOG00000057641	NA	11.28364122	0.341533128	0.373587736

ENSRNOG00000045790	NA	150.1153519	-0.112558415	0.123166723
ENSRNOG00000054147	NA	48.44566101	0.18393878	0.201435516
ENSRNOG00000047113	NA	34.35605688	-0.245367467	0.269647444
ENSRNOG00000061993	NA	14.2771645	-0.319779817	0.351628374
ENSRNOG00000055899	NA	8.450038424	0.435355138	0.480187406
ENSRNOG00000025909	NA	35.56987005	-0.190892487	0.210660659
ENSRNOG00000055568	NA	55.63994741	-0.174672665	0.193272351
ENSRNOG00000053420	NA	7.80828378	0.483474334	0.535636118
ENSRNOG00000031222	NA	69.68500873	-0.138585567	0.153673728
ENSRNOG00000060130	NA	12.65768455	0.374377148	0.416450893
ENSRNOG00000024071	NA	22.29244921	0.268296776	0.298529463
ENSRNOG00000027818	NA	12.76165868	-0.336832342	0.374895852
ENSRNOG00000016200	NA	75.31802317	0.169036911	0.188557922
ENSRNOG00000015161	NA	77.1488684	-0.183117174	0.204659796
ENSRNOG00000060445	NA	9.209550143	-0.398984663	0.447351495
ENSRNOG00000031710	NA	4.936391186	-0.492780948	0.552736264
ENSRNOG00000006326	NA	12.54061877	0.727424492	0.820125626
ENSRNOG00000058158	NA	16.24565751	0.327794711	0.370643607
ENSRNOG00000052459	NA	22.74453086	0.256662058	0.292184023
ENSRNOG00000009993	NA	75.33347508	-0.143696563	0.163647383
ENSRNOG00000057228	NA	14.8259971	0.279189097	0.318191544
ENSRNOG00000016686	NA	16.03773994	-0.303101667	0.345852809
ENSRNOG00000014055	NA	13.93041872	0.313693582	0.358100512
ENSRNOG00000021802	NA	12.08743404	-0.322732192	0.369468567
ENSRNOG00000016242	NA	34.74011127	0.194689674	0.223010672
ENSRNOG00000057372	NA	88.79889907	-0.122936725	0.141631562
ENSRNOG00000053805	NA	14.8422988	0.299437189	0.345269491
ENSRNOG00000055554	NA	42.11017247	0.498841342	0.577509048
ENSRNOG00000048656	NA	120.963986	-0.104315688	0.120844484
ENSRNOG00000039717	NA	23.16398706	-0.247730426	0.287388932
ENSRNOG00000020616	NA	10.31051774	0.352865856	0.409509232
ENSRNOG00000060833	NA	8.852757587	0.439230425	0.510667911
ENSRNOG00000052421	NA	8.074214225	-0.364992684	0.424486632
ENSRNOG00000047065	NA	4.790488471	-0.475911834	0.553554516
ENSRNOG00000057886	NA	13.37360153	-0.351219888	0.408616075
ENSRNOG00000052514	NA	9.921927273	-0.413063412	0.481056735
ENSRNOG00000010918	NA	32.86454332	-0.212218106	0.248849093
ENSRNOG00000061934	NA	18.71894493	-0.261031114	0.30613078
ENSRNOG00000060063	NA	77.42093947	-0.127027758	0.149460477
ENSRNOG00000023459	NA	57.07571634	-0.21550885	0.25483394
ENSRNOG00000059911	NA	190.52873	-0.084984306	0.100505685
ENSRNOG00000042308	NA	12.2592504	0.318165197	0.376529755
ENSRNOG00000049116	NA	24.22098485	0.258562151	0.306717217
ENSRNOG00000050193	NA	53.06832105	0.18096042	0.214709846
ENSRNOG00000048390	NA	9.317275744	-0.393969477	0.467613942
ENSRNOG00000059373	NA	8.189237166	0.397182874	0.471435945
ENSRNOG00000036912	NA	12.5262179	-0.445872128	0.530223899
ENSRNOG00000030152	NA	14.98385057	-0.251830207	0.29979195
ENSRNOG00000001858	NA	17.69794994	-0.256112912	0.306252816
ENSRNOG00000045772	NA	16.64013842	0.258810945	0.31065108

ENSRNOG00000003837	NA	22.13469174	-0.250586215	0.301280279
ENSRNOG00000053888	NA	16.26009422	0.27744616	0.333673278
ENSRNOG00000015491	NA	114.6488017	0.118680989	0.142774177
ENSRNOG00000059859	NA	14.60147968	-0.283617563	0.341457754
ENSRNOG00000053643	NA	19.16802373	-0.252420256	0.30481476
ENSRNOG00000006876	NA	27.0678577	0.234982647	0.283790405
ENSRNOG00000004387	NA	42.7220043	0.189791364	0.229760793
ENSRNOG00000061084	NA	9.342271606	0.343030894	0.416023762
ENSRNOG00000047335	NA	9.53165305	0.375223456	0.455821547
ENSRNOG00000018714	NA	43.97237118	0.188226532	0.229251204
ENSRNOG00000001474	NA	23.59318806	-0.244446853	0.299255535
ENSRNOG00000054367	NA	36.17197543	-0.193396067	0.237018145
ENSRNOG00000061425	NA	79.8683899	0.132239782	0.162223258
ENSRNOG00000046570	NA	78.88281451	-0.119430134	0.146931245
ENSRNOG00000050210	NA	37.01427487	-0.230856031	0.284650727
ENSRNOG00000045837	NA	44.26654907	-0.161889274	0.199889077
ENSRNOG00000017095	NA	22.39074124	0.221256717	0.273202577
ENSRNOG00000029785	NA	96.09357173	0.12475369	0.154044553
ENSRNOG00000059040	NA	56.20451089	0.175941271	0.218525099
ENSRNOG00000015122	NA	141.4150329	-0.116359251	0.144557047
ENSRNOG00000030216	NA	21.06393923	0.308885508	0.384072772
ENSRNOG00000003984	NA	184.4991241	0.108344417	0.134896088
ENSRNOG00000059440	NA	13.61869913	-0.296140286	0.368757988
ENSRNOG00000031935	NA	17.08068125	-0.251417375	0.313278686
ENSRNOG00000049324	NA	19.27297052	0.22385107	0.280784073
ENSRNOG00000016016	NA	37.80907035	-0.191704113	0.243122344
ENSRNOG00000046062	NA	76.72679274	0.11833379	0.15112186
ENSRNOG00000000866	NA	70.98172119	0.140021991	0.179365599
ENSRNOG00000052336	NA	60.70203492	-0.13620273	0.174474663
ENSRNOG00000050101	NA	10.81134839	0.322847348	0.415700857
ENSRNOG00000050431	NA	8.943138394	0.352804325	0.455958341
ENSRNOG00000049995	NA	9.334624551	0.339321822	0.439445843
ENSRNOG00000003279	NA	83.91475701	-0.129435919	0.167763317
ENSRNOG00000062172	NA	14.680708	0.249222084	0.325261101
ENSRNOG00000057036	NA	18.44086331	0.242788508	0.317102819
ENSRNOG00000021705	NA	10.02349735	0.340735921	0.44538642
ENSRNOG00000062158	NA	124.325007	-0.434323454	0.56796715
ENSRNOG00000011527	NA	158.8161971	0.095380619	0.125029179
ENSRNOG00000051943	NA	123.744647	0.103254457	0.136151062
ENSRNOG00000046447	NA	24.92890707	0.214364644	0.283028332
ENSRNOG00000004860	NA	23.60507297	0.261838658	0.346308857
ENSRNOG00000042653	NA	146.3846785	-0.083479173	0.110717023
ENSRNOG00000050278	NA	54.19142752	-0.16173446	0.215249324
ENSRNOG00000050036	NA	20.19682795	0.238197319	0.317864036
ENSRNOG00000029091	NA	55.19938119	-0.146735728	0.196022296
ENSRNOG00000053968	NA	33.4061513	-0.159003394	0.212949625
ENSRNOG00000001342	NA	162.5645617	0.092821386	0.124927047
ENSRNOG00000047890	NA	51.65285642	0.155437837	0.209574694
ENSRNOG00000046536	NA	91.69931441	-0.130660659	0.176194547
ENSRNOG00000042971	NA	25.43190913	-0.306121321	0.415945822

ENSRNOG00000058912	NA	28.02880866	-0.194956492	0.265806584
ENSRNOG00000037695	NA	15.0655437	0.246467545	0.336899555
ENSRNOG00000026902	NA	28.28915241	-0.174353134	0.238369819
ENSRNOG00000030273	NA	33.6925763	0.175151995	0.239604798
ENSRNOG00000046147	NA	66.8337767	0.120921116	0.166167872
ENSRNOG00000003746	NA	36.73077877	0.166285662	0.229646154
ENSRNOG00000048563	NA	125.2057967	0.086276464	0.11929183
ENSRNOG00000046156	NA	185.9881886	0.084494356	0.117384039
ENSRNOG00000050955	NA	8.119473808	0.360914752	0.501432782
ENSRNOG00000009373	NA	127.5164536	0.108939515	0.151408206
ENSRNOG00000039528	NA	33.42983499	-0.392378542	0.545769395
ENSRNOG00000016444	NA	187.4367312	0.103780604	0.144603302
ENSRNOG00000056017	NA	14.62982895	0.256288117	0.358069596
ENSRNOG00000028087	NA	57.62058671	-0.125727793	0.175820185
ENSRNOG00000013570	NA	24.86645624	-0.174261631	0.245351191
ENSRNOG00000047244	NA	13.68237175	-0.251416534	0.356329622
ENSRNOG00000058416	NA	10.08532188	-0.313676896	0.444686582
ENSRNOG00000047651	NA	9.506192869	-0.309791992	0.440174199
ENSRNOG00000058249	NA	62.25503113	0.122087158	0.173590504
ENSRNOG00000055892	NA	10.30015345	-0.307703425	0.438409957
ENSRNOG00000060735	NA	23.03428209	0.19807918	0.282534886
ENSRNOG00000052044	NA	9.308308419	0.317640587	0.454890043
ENSRNOG00000006987	NA	14.15737989	-0.245915083	0.355980069
ENSRNOG00000033894	NA	9.157256943	0.284839844	0.412503714
ENSRNOG00000061842	NA	18.98234831	0.214551745	0.310993397
ENSRNOG00000001107	NA	101.048385	-0.09633884	0.139679363
ENSRNOG00000060873	NA	65.45172591	-0.118029702	0.171353165
ENSRNOG00000051396	NA	15.54206672	0.250493802	0.364413062
ENSRNOG00000057852	NA	134.9702463	0.077399435	0.113401069
ENSRNOG00000045691	NA	63.95163835	0.128356866	0.18827569
ENSRNOG00000034040	NA	36.07262771	0.144210457	0.212716375
ENSRNOG00000054795	NA	17.7178098	0.256776848	0.379431341
ENSRNOG00000019416	NA	166.5104289	0.078196506	0.115753682
ENSRNOG00000037919	NA	110.9750708	-0.088245745	0.130757608
ENSRNOG00000043107	NA	45.78256329	-0.141736298	0.210030469
ENSRNOG00000057017	NA	13.79634919	0.241884444	0.358538128
ENSRNOG00000061272	NA	74.92153019	-0.115726447	0.17163303
ENSRNOG00000059938	NA	10.23750987	-0.271497837	0.403634189
ENSRNOG00000048426	NA	186.6696601	-0.080618473	0.120225137
ENSRNOG00000014551	NA	31.92734154	0.167432886	0.249697217
ENSRNOG00000062275	NA	18.24746549	-0.219210441	0.327392696
ENSRNOG00000049308	NA	21.55392815	0.304526942	0.455110296
ENSRNOG00000043197	NA	9.195233804	0.286713576	0.429860809
ENSRNOG00000004101	NA	41.10074221	0.163026604	0.24520374
ENSRNOG00000006612	NA	98.70914027	0.099401437	0.150332964
ENSRNOG00000061902	NA	39.29235083	-0.539421314	0.816352931
ENSRNOG00000028895	NA	29.19719059	-0.192913322	0.292510706
ENSRNOG00000057139	NA	132.5728798	-0.078940875	0.120105869
ENSRNOG00000056631	NA	28.00459563	0.171823024	0.261877915
ENSRNOG00000056011	NA	18.5819884	-0.207935743	0.317117261

ENSRNOG00000054637	NA	9.554132243	0.278488668	0.424797842
ENSRNOG00000058777	NA	23.95779457	0.223683372	0.341889887
ENSRNOG00000047719	NA	56.58220118	-0.11424002	0.174868224
ENSRNOG00000050735	NA	180.5762643	0.071712464	0.11021014
ENSRNOG00000061770	NA	17.42399466	-0.221733067	0.341812746
ENSRNOG00000039025	NA	77.87654381	0.103379323	0.159510411
ENSRNOG00000019070	NA	51.50747321	-0.14472961	0.224372177
ENSRNOG00000062001	NA	9.597964334	0.304925143	0.473229209
ENSRNOG00000061201	NA	49.95300831	-0.156862988	0.24386662
ENSRNOG00000048861	NA	119.8346278	-0.102355991	0.159750974
ENSRNOG00000028747	NA	149.3306283	-0.070281149	0.109693112
ENSRNOG00000049598	NA	21.84616981	0.213923372	0.334391404
ENSRNOG00000059412	NA	35.73618513	0.13631313	0.21420355
ENSRNOG00000030870	NA	39.24896723	-0.134591165	0.211563306
ENSRNOG00000048168	NA	12.90183843	-0.230258784	0.362860991
ENSRNOG00000048049	NA	23.58348008	0.171452153	0.27042316
ENSRNOG00000047976	NA	53.86385157	0.111295529	0.175545922
ENSRNOG00000058486	NA	75.17939034	-0.090939084	0.143615908
ENSRNOG00000046944	NA	24.01064794	0.165671788	0.261758867
ENSRNOG00000054579	NA	18.61270364	-0.195273211	0.308748643
ENSRNOG00000046029	NA	8.116781568	0.301325622	0.476909413
ENSRNOG00000055634	NA	35.96794027	0.134437748	0.212796653
ENSRNOG00000050441	NA	17.3550571	-0.216697693	0.343017062
ENSRNOG00000030321	NA	10.50203912	-0.240715215	0.383213971
ENSRNOG00000025003	NA	17.03742107	-0.201387157	0.321112359
ENSRNOG00000002536	NA	162.9400045	-0.076139622	0.121724325
ENSRNOG00000038432	NA	59.97299448	-0.112438729	0.179867277
ENSRNOG00000009140	NA	161.4994801	0.077425255	0.124490806
ENSRNOG00000056002	NA	8.988647928	0.250882172	0.406292169
ENSRNOG00000050795	NA	27.34822965	-0.148858736	0.242039793
ENSRNOG00000055930	NA	15.65555033	-0.217810184	0.354221635
ENSRNOG00000033776	NA	8.505823977	-0.292318425	0.476921508
ENSRNOG00000058725	NA	17.96836626	-0.197328132	0.321995158
ENSRNOG00000062181	NA	95.78071827	0.08973373	0.146690082
ENSRNOG00000037566	NA	14.76085205	-0.244605838	0.4001566
ENSRNOG00000046658	NA	9.627130721	0.26913825	0.445925627
ENSRNOG00000015415	NA	56.90041433	0.118240362	0.196753988
ENSRNOG00000010594	NA	20.67812713	0.199809975	0.333790061
ENSRNOG00000051702	NA	9.328334606	0.249917872	0.417737644
ENSRNOG00000057241	NA	11.00096173	-0.254998979	0.426256595
ENSRNOG00000052134	NA	68.04886146	-0.110340168	0.18462104
ENSRNOG00000049908	NA	21.0419276	0.17630524	0.297499472
ENSRNOG00000022725	NA	101.9492796	-0.345763827	0.591927631
ENSRNOG00000062287	NA	12.91442301	0.20141541	0.345965481
ENSRNOG00000045965	NA	102.7723784	-0.111077924	0.190973571
ENSRNOG00000043439	NA	63.21674046	0.096104497	0.166514997
ENSRNOG00000045646	NA	95.38642172	-0.088785309	0.154187348
ENSRNOG00000002871	NA	173.8656516	-0.061425881	0.106818075
ENSRNOG00000051383	NA	146.1007844	0.066560686	0.115772211
ENSRNOG00000048973	NA	31.87779391	0.143914698	0.251369878

ENSRNOG00000021526	NA	12.78968414	-0.210805671	0.368922473
ENSRNOG00000047914	NA	49.82059687	0.121887211	0.213326733
ENSRNOG00000032130	NA	56.36321732	0.106045388	0.185704903
ENSRNOG00000051356	NA	117.0307566	0.069872308	0.122443465
ENSRNOG00000056685	NA	34.59561565	0.144910152	0.254014429
ENSRNOG00000051039	NA	40.19712093	0.117263757	0.205558061
ENSRNOG00000020232	NA	104.4101328	0.073586968	0.129232114
ENSRNOG00000059041	NA	24.29613095	0.182412217	0.321974817
ENSRNOG00000048409	NA	11.72009821	0.232047192	0.409628398
ENSRNOG00000055143	NA	12.90552484	-0.198500609	0.35080107
ENSRNOG00000008962	NA	8.358733453	-0.264989644	0.468462981
ENSRNOG00000051002	NA	50.65000249	-0.115716876	0.204899058
ENSRNOG00000050315	NA	191.3293535	-0.155810781	0.27606627
ENSRNOG00000011895	NA	36.7882705	-0.134099052	0.2379989
ENSRNOG00000001161	NA	11.03936179	0.219643825	0.391357196
ENSRNOG00000049921	NA	11.93370333	0.226467973	0.403827559
ENSRNOG00000052654	NA	13.20144701	0.246370337	0.440051092
ENSRNOG00000051681	NA	117.1044138	0.082015492	0.14732004
ENSRNOG00000003675	NA	52.50038806	-0.102147689	0.183940915
ENSRNOG00000019657	NA	38.12224969	0.12699459	0.229279537
ENSRNOG00000054866	NA	10.89954837	0.256427708	0.463074441
ENSRNOG00000055292	NA	72.64846451	-0.084906508	0.153343729
ENSRNOG00000046379	NA	11.31638994	-0.218541191	0.395541768
ENSRNOG00000011279	NA	19.53037492	-0.194588215	0.352748065
ENSRNOG00000021302	NA	37.41434105	-0.135303655	0.245767374
ENSRNOG00000026035	NA	8.018641814	-0.254057377	0.461906798
ENSRNOG00000030666	NA	9.277353993	-1.091852014	1.985339135
ENSRNOG00000031045	NA	73.86129048	0.094276591	0.171801187
ENSRNOG00000001011	NA	12.15809914	-0.187714278	0.342788964
ENSRNOG00000055526	NA	143.8144816	-0.079401138	0.145112314
ENSRNOG00000055317	NA	16.31838214	-0.18592578	0.341901532
ENSRNOG00000051156	NA	8.324231103	-0.266655552	0.493404773
ENSRNOG00000048016	NA	81.65745734	0.082157825	0.152064933
ENSRNOG00000052837	NA	110.548214	0.068326151	0.127567775
ENSRNOG00000055822	NA	56.50323533	0.104925059	0.196390618
ENSRNOG00000036745	NA	137.1334954	0.071173687	0.133635392
ENSRNOG00000000097	NA	13.11047157	0.206939378	0.388793688
ENSRNOG00000059884	NA	47.1845547	0.120128315	0.226412757
ENSRNOG00000016221	NA	67.12698943	-0.105856772	0.199531478
ENSRNOG00000050289	NA	161.6145269	-0.0601695	0.114028221
ENSRNOG00000054905	NA	83.46999788	0.27401732	0.520863107
ENSRNOG00000051446	NA	64.55144162	0.090153853	0.172160196
ENSRNOG00000049342	NA	9.022764418	0.268038902	0.513721034
ENSRNOG00000033449	NA	80.79041409	-0.078078762	0.149714025
ENSRNOG00000013673	NA	52.14880763	0.094152524	0.18094406
ENSRNOG00000042939	NA	19.90611007	-0.151591569	0.29159837
ENSRNOG00000052918	NA	11.19221689	0.207685228	0.399589212
ENSRNOG00000060518	NA	22.60896956	-0.182837514	0.352487928
ENSRNOG00000050874	NA	8.453831942	0.226776303	0.440010919
ENSRNOG00000060837	NA	16.2550091	-0.17471529	0.339736083

ENSRNOG00000059210	NA	10.46745908	0.622942662	1.214519928
ENSRNOG00000026564	NA	11.60975042	-0.196339476	0.382878767
ENSRNOG00000045943	NA	24.27272554	-0.145876697	0.284703038
ENSRNOG00000059898	NA	16.26391604	0.177218658	0.347135553
ENSRNOG00000014861	NA	10.28167288	-0.209965198	0.411347095
ENSRNOG00000049900	NA	26.6005628	-0.134612479	0.264955826
ENSRNOG00000047507	NA	79.91794717	-0.086383362	0.170729701
ENSRNOG00000048268	NA	19.18324694	-0.184742226	0.365317506
ENSRNOG00000004180	NA	76.40591979	-0.097475485	0.192985031
ENSRNOG00000061174	NA	144.3792011	-0.063494399	0.12576951
ENSRNOG00000059319	NA	20.2165899	-0.135643731	0.269383136
ENSRNOG00000052899	NA	18.08430149	-0.150310131	0.298951656
ENSRNOG00000009617	NA	54.94688603	0.093928929	0.188337289
ENSRNOG00000029046	NA	15.49211633	0.168886862	0.339828198
ENSRNOG00000061785	NA	103.5192612	-0.066396279	0.133724983
ENSRNOG00000000723	NA	15.16070735	-0.154382147	0.31223861
ENSRNOG00000053109	NA	118.1923811	-0.061607546	0.124650392
ENSRNOG00000048864	NA	10.70836769	0.199027525	0.404220128
ENSRNOG00000001493	NA	13.95289824	0.160697556	0.327108975
ENSRNOG00000057505	NA	18.62261092	0.151503085	0.311511629
ENSRNOG00000028592	NA	25.50336035	0.126440032	0.260409929
ENSRNOG00000052041	NA	71.18192491	-0.076952957	0.158588719
ENSRNOG00000046183	NA	16.89405814	0.161724421	0.334691577
ENSRNOG00000032944	NA	138.6968521	-0.054814627	0.114398026
ENSRNOG00000009635	NA	25.43693732	0.124634354	0.260170578
ENSRNOG00000050575	NA	8.167418636	-0.536643802	1.121429554
ENSRNOG00000055330	NA	21.54104072	-0.14019321	0.293275832
ENSRNOG00000058558	NA	107.4609073	0.075626055	0.158821273
ENSRNOG00000027006	NA	15.95315998	0.184284192	0.388080862
ENSRNOG00000048571	NA	9.537052075	0.222082311	0.46881785
ENSRNOG00000042502	NA	8.900880744	-0.209365042	0.44229341
ENSRNOG00000061925	NA	9.859498537	0.261686357	0.55284776
ENSRNOG00000059030	NA	9.80294383	-0.210874956	0.447836908
ENSRNOG00000062120	NA	20.20910294	-0.142337379	0.302924912
ENSRNOG00000054408	NA	19.52812634	0.135962289	0.290089747
ENSRNOG00000046068	NA	23.4442055	-0.142402549	0.303875134
ENSRNOG00000012830	NA	9.965579305	0.204874464	0.437725865
ENSRNOG00000027702	NA	9.509369781	0.249789011	0.534821819
ENSRNOG00000050855	NA	33.56156541	-0.104022987	0.223024102
ENSRNOG00000045527	NA	11.6090254	0.177187712	0.380636396
ENSRNOG00000052667	NA	78.61486198	0.065656522	0.141155944
ENSRNOG00000003983	NA	19.07350507	0.139457816	0.299946274
ENSRNOG00000057968	NA	80.15237233	-0.079682222	0.171589237
ENSRNOG00000007727	NA	11.37653672	-0.172633178	0.372059822
ENSRNOG00000061264	NA	11.74995141	0.168867516	0.364942532
ENSRNOG00000056941	NA	20.39514778	0.13140609	0.285425659
ENSRNOG00000011225	NA	34.86958262	0.104214326	0.227295052
ENSRNOG00000015967	NA	27.94701741	0.539380471	1.180328545
ENSRNOG00000057987	NA	27.10706474	0.115163131	0.252621932
ENSRNOG00000045684	NA	9.22594296	-0.19285126	0.423229268

ENSRNOG00000004419	NA	48.17699984	0.093728849	0.205789748
ENSRNOG00000061210	NA	11.84072496	-0.209054206	0.459363134
ENSRNOG00000001339	NA	34.10679687	0.104504165	0.230078883
ENSRNOG00000046594	NA	64.7070705	-0.083873037	0.184896192
ENSRNOG00000003670	NA	123.8112755	-0.055207493	0.121851208
ENSRNOG00000052003	NA	17.75335583	0.166815357	0.369389907
ENSRNOG00000054724	NA	34.47652925	0.103040055	0.22985184
ENSRNOG00000053896	NA	12.95946218	0.155337414	0.346517649
ENSRNOG00000031599	NA	9.007074229	-0.223868966	0.501963787
ENSRNOG00000046882	NA	12.08690157	0.163564223	0.367768066
ENSRNOG00000038205	NA	47.33802938	0.097997565	0.221156032
ENSRNOG00000060404	NA	100.1611904	0.066659817	0.150585076
ENSRNOG00000024250	NA	77.89073178	-0.073018413	0.165604105
ENSRNOG00000025156	NA	47.36992284	-0.084840344	0.19257042
ENSRNOG00000051304	NA	43.33092971	-0.101561043	0.23131304
ENSRNOG00000057710	NA	141.9471525	-0.064912384	0.148010032
ENSRNOG00000051349	NA	9.601027956	-0.172060028	0.392515928
ENSRNOG00000031216	NA	10.36030167	0.173830492	0.398277319
ENSRNOG00000048190	NA	12.63040378	-0.174545708	0.401065371
ENSRNOG00000051264	NA	22.86914225	-0.134782564	0.310429141
ENSRNOG00000012086	NA	14.47087643	0.15107866	0.350823019
ENSRNOG00000061418	NA	12.90540981	0.155234117	0.360764942
ENSRNOG00000050201	NA	8.235515131	0.235566798	0.549692925
ENSRNOG00000052817	NA	121.3743657	0.053218939	0.124699878
ENSRNOG00000046382	NA	18.69883433	-0.169321625	0.39767398
ENSRNOG00000001341	NA	15.53640385	0.14293516	0.336375302
ENSRNOG00000053260	NA	9.526668765	0.193262661	0.45792315
ENSRNOG00000060803	NA	8.98033929	0.185910729	0.442675509
ENSRNOG00000060069	NA	22.79375553	-0.116454762	0.278394717
ENSRNOG00000025810	NA	101.4119166	-0.375271284	0.897552486
ENSRNOG00000054614	NA	72.22245032	0.066602844	0.160117641
ENSRNOG00000049802	NA	16.58008474	0.136827183	0.329235555
ENSRNOG00000025987	NA	188.4891634	0.041892031	0.10122715
ENSRNOG00000058688	NA	9.243651962	0.191589089	0.464116397
ENSRNOG00000055373	NA	92.28633073	-0.060447084	0.14665512
ENSRNOG00000038911	NA	42.23827786	-0.08172946	0.199456979
ENSRNOG00000030554	NA	17.16738993	0.139437475	0.343343786
ENSRNOG00000046414	NA	15.39877471	0.137719984	0.339929896
ENSRNOG00000053342	NA	10.27107373	0.187342865	0.46619749
ENSRNOG00000049975	NA	140.9847083	0.055149399	0.137787617
ENSRNOG00000047551	NA	21.64824926	0.103327313	0.258216885
ENSRNOG00000061532	NA	12.92636683	-0.173039145	0.438727399
ENSRNOG00000046275	NA	12.94351625	0.139011191	0.354609391
ENSRNOG00000057389	NA	14.6821531	-0.128352511	0.327740506
ENSRNOG00000013200	NA	17.31846186	-0.124440658	0.318380858
ENSRNOG00000048613	NA	46.36347741	-0.073540774	0.188743363
ENSRNOG00000032095	NA	171.4316552	0.051725641	0.133494148
ENSRNOG00000019206	NA	9.56181948	0.181247268	0.468332383
ENSRNOG00000055179	NA	29.48129071	0.098952219	0.25588399
ENSRNOG00000015851	NA	113.5178122	-0.050485886	0.131207837

ENSRNOG00000045924	NA	85.27631547	0.086883521	0.226746289
ENSRNOG00000054485	NA	8.714891809	-0.159534747	0.419738507
ENSRNOG00000003214	NA	49.22632616	-0.07307023	0.192863864
ENSRNOG00000054392	NA	8.723686539	0.155800802	0.412660063
ENSRNOG00000059425	NA	27.03126299	0.09217397	0.24432551
ENSRNOG00000019831	NA	96.50348163	0.117368769	0.315595023
ENSRNOG00000051631	NA	23.77712216	0.099307569	0.269015689
ENSRNOG00000030463	NA	66.51913635	0.070482996	0.191667907
ENSRNOG00000053812	NA	49.67896073	0.073900272	0.200976777
ENSRNOG00000020467	NA	8.968073252	-0.154316584	0.419774801
ENSRNOG00000031912	NA	8.852381919	-0.172954219	0.47110513
ENSRNOG00000060019	NA	21.18143858	0.117498891	0.323194094
ENSRNOG00000056964	NA	52.54451235	-0.068303434	0.18816017
ENSRNOG00000052685	NA	53.35829971	-0.076256357	0.210726941
ENSRNOG00000051771	NA	165.7236068	0.043193719	0.119846219
ENSRNOG00000060990	NA	147.7031714	0.039734084	0.11058026
ENSRNOG00000051824	NA	51.46499992	-0.079201024	0.220890235
ENSRNOG00000059709	NA	18.1178572	0.112229441	0.313082654
ENSRNOG00000048597	NA	28.20099849	0.098813878	0.276894134
ENSRNOG00000037087	NA	10.02782341	0.142496422	0.401696803
ENSRNOG00000016723	NA	22.21641604	0.101584672	0.28647015
ENSRNOG00000027359	NA	10.3292197	0.15240252	0.432169977
ENSRNOG00000057009	NA	13.64060629	-0.11931561	0.342494935
ENSRNOG00000057419	NA	16.99704872	0.119009733	0.343241929
ENSRNOG00000037709	NA	79.32722428	-0.055248531	0.159760389
ENSRNOG00000008475	NA	9.292023979	0.143098322	0.41418161
ENSRNOG00000018936	NA	11.54431369	-0.137279806	0.397790061
ENSRNOG00000034134	NA	23.6252023	0.086677908	0.251180614
ENSRNOG00000043289	NA	10.41419649	0.145413763	0.423261949
ENSRNOG00000047003	NA	42.14900458	0.078907707	0.229995303
ENSRNOG00000009519	NA	17.60277682	-0.112598219	0.330032929
ENSRNOG00000003281	NA	72.4966156	0.053666838	0.157355122
ENSRNOG00000058096	NA	18.16275447	0.105910468	0.313982224
ENSRNOG00000022857	NA	32.37625354	-0.071847834	0.214569062
ENSRNOG00000060414	NA	17.40053321	0.132797113	0.397948296
ENSRNOG00000001687	NA	60.93017534	-0.070645544	0.211896124
ENSRNOG00000030579	NA	8.881288364	0.159664354	0.479411719
ENSRNOG00000056150	NA	29.65043971	-0.323991548	0.975739307
ENSRNOG00000022252	NA	32.64651418	-0.080988215	0.244454199
ENSRNOG00000029292	NA	34.81617614	-0.072418279	0.218739814
ENSRNOG00000060228	NA	162.2366298	-0.039528054	0.120300334
ENSRNOG00000004247	NA	17.06521596	-0.107271315	0.328919807
ENSRNOG00000030058	NA	88.33842927	0.049305915	0.152215836
ENSRNOG00000048800	NA	16.51563567	0.095739161	0.295710765
ENSRNOG00000045569	NA	33.01364098	-0.080232279	0.249295054
ENSRNOG00000001357	NA	36.92295542	0.070562001	0.22005954
ENSRNOG00000010986	NA	45.18157701	0.059875148	0.186901325
ENSRNOG00000032328	NA	33.62804202	0.076236917	0.238159917
ENSRNOG00000022919	NA	113.7900881	-0.052494023	0.164045591
ENSRNOG00000057821	NA	13.31230695	-0.11217043	0.352037128

ENSRNOG00000047587	NA	18.52726162	-0.088543513	0.280722486
ENSRNOG00000050051	NA	64.56122451	-0.059607684	0.189663994
ENSRNOG00000052951	NA	21.61037973	-0.084165574	0.267895533
ENSRNOG00000048237	NA	127.3260911	-0.04099308	0.13209207
ENSRNOG00000032660	NA	165.9053221	-0.038210314	0.123257411
ENSRNOG00000037960	NA	44.6096521	-0.064624673	0.208968591
ENSRNOG00000057223	NA	21.35095615	-0.0819634	0.266504076
ENSRNOG00000061118	NA	32.92998858	-0.086720743	0.282255822
ENSRNOG00000012490	NA	54.4388214	0.210711644	0.688300945
ENSRNOG00000042851	NA	9.215424675	-0.129808296	0.426829186
ENSRNOG00000045322	NA	51.64349414	0.052263679	0.172394977
ENSRNOG00000033654	NA	116.2887609	-0.043759743	0.144506584
ENSRNOG00000061488	NA	12.30345711	0.116446622	0.385973351
ENSRNOG00000054855	NA	11.46406495	-0.128396019	0.428338602
ENSRNOG00000038471	NA	17.30621529	0.09387909	0.31359723
ENSRNOG00000049132	NA	101.6598696	0.04370701	0.146309756
ENSRNOG00000056849	NA	26.32561403	-0.074193506	0.2484239
ENSRNOG00000011040	NA	47.66287051	0.054830125	0.184228454
ENSRNOG00000036397	NA	109.8264568	-0.043268005	0.145877519
ENSRNOG00000033729	NA	13.79730159	0.118743971	0.401056539
ENSRNOG00000048689	NA	14.50205029	-0.095925493	0.326276282
ENSRNOG00000047329	NA	10.05686682	-0.125773789	0.429475532
ENSRNOG00000057494	NA	49.33410165	0.05781494	0.197567376
ENSRNOG00000046920	NA	94.18555461	-0.042433573	0.145308506
ENSRNOG00000060952	NA	11.7718789	0.107061215	0.367523565
ENSRNOG00000002574	NA	186.1148474	0.028458619	0.098161422
ENSRNOG00000058246	NA	67.51370736	-0.043626862	0.152132831
ENSRNOG00000051346	NA	52.9559495	0.061690415	0.215640268
ENSRNOG00000050781	NA	58.18763438	0.054245808	0.189722479
ENSRNOG00000029470	NA	45.85569345	-0.058890325	0.2059936
ENSRNOG00000052004	NA	145.9991981	0.032683594	0.114533518
ENSRNOG00000017167	NA	44.41226386	-0.057858042	0.202874191
ENSRNOG00000049661	NA	163.6192081	-0.030601312	0.107536852
ENSRNOG00000061623	NA	10.41566336	-0.109259021	0.385225421
ENSRNOG00000032426	NA	65.53124306	0.046227784	0.168310451
ENSRNOG00000057137	NA	12.20407199	-0.121001709	0.441163956
ENSRNOG00000044494	NA	8.958981361	-0.332407875	1.220101161
ENSRNOG00000056970	NA	23.97035236	-0.085418303	0.313804436
ENSRNOG00000019773	NA	86.38252456	0.037706767	0.139606419
ENSRNOG00000047379	NA	8.746513558	0.116671951	0.433321534
ENSRNOG00000001062	NA	20.47195426	-0.073694599	0.276648443
ENSRNOG00000052311	NA	114.8189626	0.123856698	0.466721048
ENSRNOG00000056566	NA	88.25512652	-0.041054973	0.1547826
ENSRNOG00000038987	NA	44.86609081	0.088940117	0.335433485
ENSRNOG00000050146	NA	12.70242399	0.105839824	0.401405705
ENSRNOG00000043204	NA	20.67344734	-0.077008447	0.294806471
ENSRNOG00000004673	NA	8.931001431	-0.108220009	0.415161039
ENSRNOG00000054945	NA	20.77611038	-0.09595083	0.370925469
ENSRNOG00000019310	NA	74.70193456	-0.040635188	0.157966914
ENSRNOG00000047971	NA	56.74927462	0.049369876	0.192735771

ENSRNOG00000047076	NA	33.48366412	0.216042193	0.858629973
ENSRNOG00000051989	NA	84.40448158	-0.035248758	0.142574646
ENSRNOG00000047000	NA	102.0785871	0.032550036	0.133053056
ENSRNOG00000050534	NA	10.73910586	-0.09191653	0.376873785
ENSRNOG00000010867	NA	14.83863606	0.076497666	0.314130517
ENSRNOG00000006908	NA	62.52261275	0.045035215	0.188269492
ENSRNOG00000055871	NA	16.55422388	0.080109279	0.341971065
ENSRNOG00000048489	NA	28.71867239	0.155895868	0.671405306
ENSRNOG00000029686	NA	70.6497533	-0.036301839	0.157701319
ENSRNOG00000057566	NA	9.058637281	0.10627842	0.46186171
ENSRNOG00000038437	NA	23.2331042	0.058925499	0.257820403
ENSRNOG00000027547	NA	39.41877011	0.048416883	0.211895443
ENSRNOG00000009338	NA	119.3038249	0.04997505	0.219270298
ENSRNOG00000025970	NA	25.82718384	-0.065333773	0.287310979
ENSRNOG00000020442	NA	171.7508512	0.025705966	0.113145544
ENSRNOG00000006263	NA	52.09252487	-0.044151223	0.195513889
ENSRNOG00000007917	NA	28.09118862	-0.056708974	0.25284593
ENSRNOG00000047115	NA	54.64806342	0.044749468	0.200877562
ENSRNOG00000039204	NA	65.34609815	0.035724839	0.160631096
ENSRNOG00000027469	NA	18.70892468	0.062906682	0.283665577
ENSRNOG00000001531	NA	44.69161586	-0.04641362	0.210958744
ENSRNOG00000001378	NA	12.48775007	-0.089056225	0.407128515
ENSRNOG00000052701	NA	14.73363267	0.079856718	0.365289737
ENSRNOG00000055834	NA	17.23385355	0.067832636	0.311590377
ENSRNOG00000018927	NA	12.79268808	-0.075618121	0.351932007
ENSRNOG00000045989	NA	87.34283225	-0.036385111	0.169341196
ENSRNOG00000013892	NA	10.35707241	0.10176055	0.476042241
ENSRNOG00000049706	NA	11.51854148	-0.084974951	0.401215548
ENSRNOG00000030807	NA	48.9664253	0.043813199	0.209886904
ENSRNOG00000055308	NA	15.88153812	-0.080346241	0.385107848
ENSRNOG00000058990	NA	11.17057197	-0.080120296	0.393609113
ENSRNOG00000062283	NA	82.38265201	-0.029621483	0.145666414
ENSRNOG00000046646	NA	9.554706327	0.100534437	0.495264406
ENSRNOG00000062155	NA	20.08855882	-0.061792706	0.304532288
ENSRNOG00000010149	NA	90.62984111	-0.032118303	0.158390998
ENSRNOG00000018450	NA	48.55891752	0.085479452	0.421548367
ENSRNOG00000002122	NA	98.83507784	0.03531883	0.174355077
ENSRNOG00000047261	NA	26.70845755	0.051544644	0.255256487
ENSRNOG00000005153	NA	34.64309237	0.042642007	0.211695896
ENSRNOG00000038965	NA	22.76874143	0.059690896	0.296723821
ENSRNOG00000006324	NA	77.52026124	-0.037081735	0.192123578
ENSRNOG00000008067	NA	21.99679032	0.054348638	0.282660969
ENSRNOG00000030319	NA	13.87660139	-0.063183345	0.3320686
ENSRNOG00000047123	NA	18.97742207	0.069486761	0.373431277
ENSRNOG00000049007	NA	18.8497792	0.053946262	0.291141797
ENSRNOG00000011382	NA	78.02094369	0.028675104	0.155475552
ENSRNOG00000050287	NA	133.521412	-0.0223498	0.122344223
ENSRNOG00000005840	NA	15.18553802	0.060607181	0.332604174
ENSRNOG00000048988	NA	11.46244268	0.076830333	0.424232536
ENSRNOG00000000916	NA	35.0953602	-0.048792207	0.273681651

ENSRNOG00000061960	NA	17.11731799	0.171615597	0.963087483
ENSRNOG00000046990	NA	20.60513227	0.053022351	0.298011744
ENSRNOG00000054907	NA	37.63482893	0.041289742	0.233310688
ENSRNOG00000057683	NA	46.3483298	0.039265981	0.222592405
ENSRNOG00000043210	NA	16.58130999	0.054963736	0.313220905
ENSRNOG00000004282	NA	122.0260923	0.025670757	0.147173273
ENSRNOG00000024794	NA	8.32347422	0.08037962	0.465091961
ENSRNOG00000055809	NA	33.32013545	-0.048004067	0.278671567
ENSRNOG00000049970	NA	10.63902277	0.066177932	0.387740187
ENSRNOG00000027263	NA	17.45157331	-0.053338447	0.313679533
ENSRNOG00000051207	NA	121.6124829	-0.021707045	0.127935674
ENSRNOG00000017100	NA	17.41384277	-0.05338023	0.317603402
ENSRNOG00000059443	NA	86.04827612	-0.026371919	0.15721091
ENSRNOG00000046305	NA	33.74161655	-0.037471174	0.224518685
ENSRNOG00000050864	NA	18.3815438	-0.050522605	0.306665472
ENSRNOG00000062291	NA	65.2030459	-0.027409786	0.166774525
ENSRNOG00000054776	NA	9.280232315	0.068490858	0.419120088
ENSRNOG00000060635	NA	8.25389333	0.092675224	0.569642125
ENSRNOG00000016740	NA	102.0529142	0.019796134	0.12215325
ENSRNOG00000048060	NA	8.397035369	0.070696307	0.438566521
ENSRNOG00000006645	NA	39.53419787	-0.045212963	0.280599664
ENSRNOG00000011019	NA	17.04857871	-0.047383455	0.297145921
ENSRNOG00000009330	NA	149.6868538	0.018087272	0.113436161
ENSRNOG00000015188	NA	21.55677569	0.045092221	0.28359494
ENSRNOG00000001426	NA	91.47622354	0.023621315	0.148736826
ENSRNOG00000022572	NA	43.23540873	-0.03445522	0.221151839
ENSRNOG00000017166	NA	94.7224235	-0.021479527	0.139065496
ENSRNOG00000017864	NA	65.94712179	-0.02915246	0.189083542
ENSRNOG00000020821	NA	56.32364252	0.026955481	0.17667968
ENSRNOG00000053615	NA	31.93066253	0.034615688	0.228411075
ENSRNOG00000004926	NA	8.908679985	0.072782671	0.482806071
ENSRNOG00000032018	NA	33.20803874	-0.031062631	0.206151904
ENSRNOG00000029837	NA	54.41262729	0.030967039	0.206812847
ENSRNOG00000049104	NA	40.25402144	-0.03387493	0.22797709
ENSRNOG00000055608	NA	56.69782642	-0.02702447	0.183003046
ENSRNOG00000051243	NA	10.30804428	-0.067225626	0.458572467
ENSRNOG00000007336	NA	73.45877241	-0.021095687	0.145361173
ENSRNOG00000058956	NA	77.87795698	0.020521443	0.143331141
ENSRNOG00000062146	NA	10.8795413	0.060660225	0.427387131
ENSRNOG00000033199	NA	144.757181	0.018417239	0.131872408
ENSRNOG00000039890	NA	15.03464639	-0.055207171	0.395826784
ENSRNOG00000031736	NA	119.466383	0.018369511	0.133790179
ENSRNOG00000062057	NA	112.6406229	0.019138722	0.14095946
ENSRNOG00000043180	NA	16.19997882	0.044443106	0.331007012
ENSRNOG00000045570	NA	14.41294483	-0.053661899	0.409938449
ENSRNOG00000009008	NA	100.0087283	-0.017768747	0.136660371
ENSRNOG00000046993	NA	13.10022671	-0.048268711	0.373459064
ENSRNOG00000062216	NA	26.66355208	-0.037728431	0.292569374
ENSRNOG00000003789	NA	8.345945368	0.056165848	0.43690653
ENSRNOG00000049622	NA	14.61562344	-0.044946625	0.349708192

ENSRNOG00000015703	NA	8.833631448	-0.057891591	0.450632739
ENSRNOG00000052316	NA	111.8379964	0.133205791	1.0415463
ENSRNOG00000055119	NA	16.86679117	-0.039529787	0.316346851
ENSRNOG00000059489	NA	9.598745736	-0.051738304	0.439933793
ENSRNOG00000048176	NA	9.477110658	-0.044922241	0.396948252
ENSRNOG00000054344	NA	89.45824624	0.026093437	0.23317698
ENSRNOG00000042667	NA	9.426499482	0.042595013	0.414035985
ENSRNOG00000038960	NA	12.0141008	0.044697192	0.448344232
ENSRNOG00000049370	NA	139.4633163	-0.010914518	0.111001621
ENSRNOG00000025589	NA	48.61701798	0.019703438	0.203276185
ENSRNOG00000050424	NA	74.71325257	0.015210912	0.159239049
ENSRNOG00000024838	NA	30.556492	0.028030077	0.297949779
ENSRNOG00000019346	NA	145.4833988	-0.009921192	0.105681161
ENSRNOG00000058655	NA	53.95930973	0.016800044	0.179935974
ENSRNOG00000062031	NA	34.7296819	-0.025681657	0.276283609
ENSRNOG00000050700	NA	25.45546239	0.026080123	0.281764899
ENSRNOG00000055096	NA	59.40297256	-0.015726079	0.173435507
ENSRNOG00000050374	NA	57.13953994	-0.015763051	0.174633717
ENSRNOG00000047516	NA	44.92744534	0.01613169	0.181292849
ENSRNOG00000057636	NA	11.94811609	-0.030816343	0.348915757
ENSRNOG00000056936	NA	8.521357996	-0.046170439	0.528489622
ENSRNOG00000054376	NA	10.45205438	0.030555266	0.363051392
ENSRNOG00000032812	NA	12.56844702	-0.028795857	0.345373856
ENSRNOG00000051336	NA	37.98195228	-0.020125891	0.241654875
ENSRNOG00000059679	NA	15.04210547	0.030842448	0.370577812
ENSRNOG00000061630	NA	58.43517343	0.0139969	0.168458829
ENSRNOG00000003054	NA	13.19323303	0.029182314	0.355846114
ENSRNOG00000022882	NA	88.46804914	0.010510979	0.134253423
ENSRNOG00000048985	NA	12.31133722	-0.026429924	0.34020005
ENSRNOG00000060667	NA	19.31374189	0.023685654	0.311450377
ENSRNOG00000059100	NA	13.64198627	-0.02653376	0.351316326
ENSRNOG00000055297	NA	23.26558164	0.020736247	0.275602151
ENSRNOG00000058727	NA	90.42056617	0.114026092	1.51950036
ENSRNOG00000033346	NA	100.537246	-0.011363956	0.154925296
ENSRNOG00000049123	NA	28.21867041	-0.01814862	0.252932046
ENSRNOG00000031167	NA	11.70660316	-0.032190138	0.450486539
ENSRNOG00000051941	NA	29.87174329	0.019206034	0.277303573
ENSRNOG00000054165	NA	11.42347566	0.027006968	0.391114637
ENSRNOG00000007374	NA	188.0382954	-0.007633537	0.110931604
ENSRNOG00000039744	NA	152.7717523	0.017847551	0.270327427
ENSRNOG00000043288	NA	30.36612049	0.015611811	0.239668137
ENSRNOG00000054513	NA	35.68423448	0.013053114	0.206458165
ENSRNOG00000019857	NA	67.00293703	0.013593577	0.215824634
ENSRNOG00000018471	NA	8.113955096	-0.02816428	0.466377487
ENSRNOG00000054811	NA	39.27937205	0.014990236	0.259256175
ENSRNOG00000049194	NA	10.40192125	0.023455119	0.406622125
ENSRNOG00000048415	NA	12.45063174	0.021786385	0.387804813
ENSRNOG00000046383	NA	36.98215179	0.012595674	0.227479199
ENSRNOG00000009906	NA	158.7190262	0.007081026	0.135309403
ENSRNOG00000061918	NA	11.30682551	-0.01914222	0.375704985

ENSRNOG00000048270	NA	33.32308947	-0.0119651	0.236957834
ENSRNOG00000058327	NA	137.3009816	-0.006471689	0.138229928
ENSRNOG00000051701	NA	11.7790814	-0.016370679	0.375724401
ENSRNOG00000051838	NA	8.994313348	0.01841079	0.425658313
ENSRNOG00000029567	NA	11.14926305	0.016176315	0.378347472
ENSRNOG00000018736	NA	16.80616348	-0.012276048	0.288271791
ENSRNOG00000050827	NA	24.72897254	-0.012085014	0.28409892
ENSRNOG00000010720	NA	9.950186132	0.017640513	0.414824016
ENSRNOG00000059841	NA	88.88344583	0.005534436	0.132981294
ENSRNOG00000053952	NA	9.177574032	-0.016916644	0.413576481
ENSRNOG00000049575	NA	38.20737461	0.007796213	0.211383707
ENSRNOG00000059466	NA	11.90394071	-0.013527083	0.373454632
ENSRNOG00000004036	NA	182.1906763	-0.003926612	0.114350302
ENSRNOG00000022601	NA	19.54798499	0.009656369	0.282469818
ENSRNOG00000055278	NA	72.73175461	-0.005901044	0.181245186
ENSRNOG00000006471	NA	165.1406073	0.003715927	0.114569397
ENSRNOG00000046049	NA	23.6703004	-0.008947813	0.277249765
ENSRNOG00000061632	NA	12.42463475	-0.01209295	0.391822128
ENSRNOG00000052830	NA	181.1992029	-0.003800298	0.124187969
ENSRNOG00000003269	NA	144.8641054	-0.003841985	0.126862689
ENSRNOG00000025388	NA	23.74220755	0.00772174	0.257620082
ENSRNOG00000057482	NA	87.72614081	-0.004173906	0.141890798
ENSRNOG00000048986	NA	11.36451423	-0.01205458	0.429345905
ENSRNOG00000040198	NA	50.49934399	0.00430636	0.178776296
ENSRNOG00000048248	NA	32.48655273	0.005612437	0.254196576
ENSRNOG00000053628	NA	54.51479892	0.00336075	0.174571006
ENSRNOG00000004564	NA	9.381996244	0.007199055	0.39565731
ENSRNOG00000043076	NA	12.77076639	0.005352694	0.336152784
ENSRNOG00000049965	NA	8.65653375	-0.006050419	0.451651946
ENSRNOG00000005114	NA	28.34148438	-0.003361439	0.266005387
ENSRNOG00000060009	NA	9.73831296	-0.004900766	0.423610251
ENSRNOG00000060486	NA	153.3362607	-0.00150336	0.139135192
ENSRNOG00000042682	NA	65.31382981	0.001649875	0.168941714
ENSRNOG00000023233	NA	13.07679761	-0.003044496	0.384769379
ENSRNOG00000049895	NA	57.51034961	0.000992434	0.174236153
ENSRNOG00000049298	NA	35.12476293	-0.001320091	0.278430702
ENSRNOG00000050407	NA	13.8669242	0.001491888	0.359814656
ENSRNOG00000058555	NA	24.59482068	-0.000829861	0.264103124
ENSRNOG00000061524	NA	9.962104297	-0.001375043	0.485670118
ENSRNOG00000060892	NA	119.4972747	0.000293077	0.128760048
ENSRNOG00000055548	NA	111.9587163	-0.000191644	0.141442747
ENSRNOG00000062179	NA	11.10665895	-6.828390847	3.146975897
ENSRNOG00000018710	NA	21.75575316	-0.593621631	1.04947915
ENSRNOG00000017758	NA	41.59447585	-0.788937678	0.708510896
ENSRNOG00000012385	Naa15	160.8085907	0.029954323	0.116236184
ENSRNOG00000045863	Naa16	50.92891233	0.198710862	0.189998257
ENSRNOG00000010523	Naa20	72.66430699	0.013553552	0.147856198
ENSRNOG00000001350	Naa25	173.521218	0.014598615	0.159918568
ENSRNOG00000014192	Naa30	179.7519261	-0.039050194	0.123279085
ENSRNOG00000018417	Naa35	186.4556605	0.132287364	0.107425947

ENSRNOG00000002273	Naaa	7.927853339	-0.242195441	0.507220833
ENSRNOG00000021000	Naaladl1	23.91623324	-0.2624002	0.270013233
ENSRNOG00000012959	Nab1	177.6091757	-0.221234122	0.110404564
ENSRNOG00000008415	Nab2	8.189353618	-0.382474575	0.445164008
ENSRNOG00000015416	Nabp1	58.18102545	-0.277600963	0.186020012
ENSRNOG00000018231	Nacc2	175.935434	0.041431034	0.097164257
ENSRNOG00000054157	Nadk2	9.262225298	0.510965072	0.433484487
ENSRNOG00000020736	Nadsyn1	22.15024886	0.264273973	0.275931307
ENSRNOG00000033133	Nae1	16.04633538	0.232643942	0.330383709
ENSRNOG00000026403	Naf1	108.3708528	-0.007236917	0.139275853
ENSRNOG00000008064	Naga	104.508612	-0.00518335	0.145110171
ENSRNOG00000002895	Nagpa	38.97361372	0.09081447	0.211064633
ENSRNOG00000050204	Naif1	12.37024131	0.156896338	0.413720994
ENSRNOG00000004752	Nalcn	21.20476304	0.307154096	0.292041209
ENSRNOG00000009754	Nampt	108.8011756	0.073896819	0.126010476
ENSRNOG00000025060	Nanos1	12.44030842	-0.447733468	0.392153371
ENSRNOG00000008307	Nanp	35.9172724	-0.056203127	0.22060176
ENSRNOG00000008945	Nans	29.16930184	0.413863222	0.266436959
ENSRNOG00000001494	Napa	53.37686847	0.142741205	0.186786385
ENSRNOG00000004753	Napb	47.52352871	0.320500397	0.206503198
ENSRNOG00000011476	Nars2	10.66268825	-0.231764163	0.367866046
ENSRNOG00000016454	Nasp	22.077404	-0.053021016	0.339697891
ENSRNOG00000056962	Nat8b	125.5269003	-0.11121452	0.128765362
ENSRNOG00000015799	Nat8f1	42.54089245	0.351238093	0.226414908
ENSRNOG00000015768	Nat8f5	55.8801953	-0.163083817	0.191236282
ENSRNOG00000049351	Nat8l	182.5932372	-0.578995815	0.345553996
ENSRNOG00000003264	Nat9	158.0655915	0.172061598	0.129250918
ENSRNOG00000008425	Nav1	22.39707124	-0.401219437	0.306237603
ENSRNOG00000014530	Nav2	85.03677231	0.261728634	0.182119934
ENSRNOG00000052157	Nav3	110.5318615	0.220041577	0.145579336
ENSRNOG00000015021	Naxd	9.589431069	-0.012008082	0.422961619
ENSRNOG00000019201	Naxe	47.48150128	-0.273672217	0.443526067
ENSRNOG00000024503	Nbas	48.09369573	0.059663982	0.19175777
ENSRNOG00000021525	Nbeal1	33.53610787	-0.15058761	0.227609665
ENSRNOG00000008580	Nbn	111.6506972	-0.053637713	0.133619181
ENSRNOG00000020730	Nbr1	25.90848385	0.387728948	0.2486254
ENSRNOG00000042978	Ncald	61.69903887	-0.158128863	0.174705414
ENSRNOG00000031890	Ncam1	114.8385876	0.057092698	0.129883941
ENSRNOG00000002126	Ncam2	116.8647815	-0.204446241	0.166329449
ENSRNOG00000055300	Ncapd2	14.44703564	0.042951317	0.324745041
ENSRNOG00000008932	Ncapd3	31.75380885	0.388014462	0.240297647
ENSRNOG00000038572	Ncapg	48.71068981	0.133195607	0.192767594
ENSRNOG00000004968	Ncapg2	13.87224594	0.210026148	0.379472011
ENSRNOG00000012051	Ncaph	16.82721528	-0.30152672	0.343251949
ENSRNOG00000009598	Ncaph2	8.14919411	0.021656808	0.485322262
ENSRNOG00000018550	Ncbp3	35.17703465	0.305322272	0.24048931
ENSRNOG00000001480	Ncf1	42.70970816	0.096588981	0.232758901
ENSRNOG00000014917	Nck1	136.1167624	0.003405448	0.124303568
ENSRNOG00000007518	Nckap1	139.1470538	0.09132091	0.13541937
ENSRNOG00000036829	Nckap1l	16.45195321	0.17935826	0.348156817

ENSRNOG00000021553	Nckap5	12.07951134	0.548318087	0.433616192
ENSRNOG00000056678	Nckap5l	25.70437533	0.212936523	0.250143654
ENSRNOG00000004793	Ncln	7.797667698	0.242133461	0.456822317
ENSRNOG00000007975	Ncoa2	17.17485744	1.010325189	0.415171612
ENSRNOG00000005616	Ncoa3	20.67193264	0.28558204	0.290133384
ENSRNOG00000019768	Ncoa4	73.76797922	1.125918697	0.243517842
ENSRNOG00000018288	Ncoa6	61.80894093	-0.063164992	0.203468759
ENSRNOG00000014004	Ncoa7	91.32741446	-0.192114347	0.14874473
ENSRNOG00000010620	Ndc1	30.6398496	0.030180152	0.223500079
ENSRNOG00000002960	Ndp	117.8644434	-0.033387345	0.125414854
ENSRNOG00000036813	Ndrg3	22.67241355	0.570586689	0.388948724
ENSRNOG00000012482	Ndrg4	40.80101505	0.40893418	0.2316936
ENSRNOG00000019014	Ndst1	17.15595063	0.118597231	0.31868117
ENSRNOG00000015781	Ndst3	8.469752287	-0.062325532	0.461813927
ENSRNOG00000062245	Ndufa10l1	58.70546353	-1.099605008	0.404436635
ENSRNOG00000020602	Ndufa13	31.15124944	-0.210857236	0.229523001
ENSRNOG00000017571	Ndufa2	129.3544997	-0.450466223	0.336652963
ENSRNOG00000060293	Ndufa3	24.14687073	-0.32927266	0.263428513
ENSRNOG00000031851	Ndufa4l2	9.173282085	1.081821809	0.468321316
ENSRNOG00000005006	Ndufaf1	41.42578039	-0.50038855	0.228774128
ENSRNOG00000055079	Ndufaf2	53.40526286	0.136717088	0.232519317
ENSRNOG00000007506	Ndufaf4	66.13555385	0.012731229	0.16344268
ENSRNOG00000040040	Ndufaf6	93.78271876	-0.12279351	0.14946638
ENSRNOG00000005279	Ndufaf7	59.85344987	0.052334878	0.170477186
ENSRNOG00000010005	Ndufb2	62.25328105	0.154390044	0.197687365
ENSRNOG00000028717	Ndufb7	160.8726704	-0.038665637	0.121069351
ENSRNOG00000038372	Ndufs2	20.1258454	-0.004404646	0.301537667
ENSRNOG00000018068	Ndufs6	14.41288514	-0.486049179	0.328943525
ENSRNOG00000024568	Ndufs7	44.84937928	-0.102111187	0.253271621
ENSRNOG00000018117	Ndufv1	31.22168215	-0.474529267	0.263374256
ENSRNOG00000049452	Nebl	36.50953004	0.156701352	0.21580931
ENSRNOG00000009236	Necap1	104.5196538	-0.165613691	0.54204539
ENSRNOG00000008427	Necap2	148.1544687	0.114745976	0.123911694
ENSRNOG00000018730	Nectin2	53.28559169	-0.212633104	0.183816854
ENSRNOG00000002176	Nectin3	68.34343307	-0.227114306	0.173635114
ENSRNOG00000004011	Nedd1	10.64973329	0.086203709	0.39412419
ENSRNOG00000019895	Nedd8	28.05057108	0.06956079	0.246672904
ENSRNOG00000014548	Nedd9	60.29697018	0.07898431	0.192654667
ENSRNOG00000008716	Nefh	74.19264185	0.006033528	0.205367264
ENSRNOG00000021410	Negr1	182.2349103	5.21351E-05	0.104957181
ENSRNOG00000010418	Nek1	58.77157624	-0.140082195	0.189892411
ENSRNOG00000012757	Nek3	14.47672682	0.073649325	0.376296021
ENSRNOG00000017997	Nek4	40.6059538	-0.105712848	0.205932053
ENSRNOG00000048769	Nek5	29.86773901	0.08179767	0.266593944
ENSRNOG00000010897	Nek6	11.05986014	0.469991415	0.461862523
ENSRNOG00000058572	Nek9	50.85627934	-0.538592792	0.190316481
ENSRNOG00000015474	Nelfa	28.31133579	0.03478936	0.248513131
ENSRNOG00000009377	Nelfb	31.47623804	0.396112638	0.343799819
ENSRNOG00000006490	Neo1	57.94672565	-0.020520307	0.187594547
ENSRNOG00000018681	Nes	14.29067348	-0.035314626	0.362383948

ENSRNOG00000017765	Net1	95.02308194	0.06709657	0.14836172
ENSRNOG00000014006	Neto1	79.15034347	-0.101051043	0.148003509
ENSRNOG00000016245	Neto2	19.32924117	0.519155642	0.312796765
ENSRNOG00000032942	Neu1	125.1458849	-0.18568362	0.122749218
ENSRNOG00000016962	Neu2	38.47641562	-0.23882538	0.233431374
ENSRNOG00000019031	Neu4	37.43528905	0.103011157	0.210380801
ENSRNOG00000005609	Neurod1	48.237591	0.237035713	0.207886411
ENSRNOG00000029792	NEWGENE_1308171	55.63730545	0.307966437	0.235546059
ENSRNOG00000003176	NEWGENE_1310011	14.20400666	-0.433644753	0.351496348
ENSRNOG00000024889	NEWGENE_1359268	94.476861	-0.038774332	0.147144359
ENSRNOG00000045695	NEWGENE_1559832	11.21094827	-0.264575265	0.417178753
ENSRNOG00000001160	NEWGENE_1586233	123.8377943	-0.109050776	0.114704632
ENSRNOG00000018426	NEWGENE_2134	79.37166187	-0.368799627	0.177166032
ENSRNOG00000038540	NEWGENE_2724	87.91124534	-0.077293842	0.136481901
ENSRNOG00000003872	NEWGENE_620180	40.90495816	0.133235827	0.209443517
ENSRNOG00000004461	NEWGENE_6497122	27.70428777	-0.150357995	0.291168374
ENSRNOG00000046473	NEWGENE_735020	47.76582586	0.039421866	0.206849091
ENSRNOG00000021589	Nexmif	60.78345365	0.131531054	0.245689891
ENSRNOG00000012512	Nexn	47.5584586	0.323418072	0.215683644
ENSRNOG00000013780	Nf1	150.736763	0.35046314	0.154258597
ENSRNOG00000022975	Nfam1	137.823636	-0.12128313	0.149059425
ENSRNOG00000017146	Nfatc1	19.52739539	-0.228094707	0.278993748
ENSRNOG00000054264	Nfatc3	10.27105898	0.305360715	0.422308733
ENSRNOG00000001548	Nfe2l2	106.5326211	-0.167966624	0.157375741
ENSRNOG00000009795	Nfib	68.50254492	0.120457408	0.181806058
ENSRNOG00000004505	Nfic	10.04277618	0.672488779	0.435422876
ENSRNOG00000023258	Nfkb1	11.5436729	0.41059889	0.433609972
ENSRNOG00000020063	Nfkbib	12.73991573	-0.770886357	0.784234448
ENSRNOG00000025111	Nfkbid	10.95529503	0.113173954	0.418483785
ENSRNOG00000000839	Nfkbil1	76.04871887	0.045840825	0.168051853
ENSRNOG00000031163	Nfkbiz	8.928489628	-0.014094319	0.449282959
ENSRNOG00000008278	Nfrkb	12.99482124	-0.073413354	0.367838543
ENSRNOG00000018410	Nfu1	121.5856331	-0.126847381	0.146854616
ENSRNOG00000058588	Nfxl1	26.6237052	0.17603598	0.270264733
ENSRNOG00000012702	Nfya	18.34299999	0.440412756	0.34918233
ENSRNOG00000011719	Ngb	33.54351241	-0.057304318	0.240393727
ENSRNOG00000018069	Ngdn	78.13063956	-0.338862937	0.149409218
ENSRNOG00000016653	Ngef	115.8452247	0.401761099	0.397756959
ENSRNOG00000016571	Ngf	13.12891805	0.437442664	0.401672554
ENSRNOG00000024330	Ngp	82.68892409	0.040189196	0.137541497
ENSRNOG00000013553	Ngrn	115.0534115	0.045757147	0.129421533
ENSRNOG00000016948	Nhlrc2	39.08125516	0.062355576	0.219610976
ENSRNOG00000060603	Nhsl1	22.80220963	-0.348153788	0.306534764
ENSRNOG00000015845	Niban2	155.1440886	-0.032945438	0.145028611
ENSRNOG00000039357	Niban3	14.45408034	0.086975503	0.74888477
ENSRNOG00000049083	Nicn1	11.76297494	0.058473595	0.404569935
ENSRNOG00000002461	Nid1	30.06993425	0.51126643	0.247421651
ENSRNOG00000013446	Nif3l1	10.29835624	-0.211268925	0.494574215
ENSRNOG00000025701	Nifk	124.225925	-0.254412529	0.142607448
ENSRNOG00000016353	Nim1k	10.3492613	-1.204210065	0.452048331

ENSRNOG00000005540	Nin	83.75226698	0.109459752	0.169040076
ENSRNOG00000042702	Nipa1	43.66469438	-0.197314812	0.227933208
ENSRNOG00000012690	Nipa2	28.00597369	0.026906563	0.251058774
ENSRNOG00000005190	Nipal2	54.74646379	0.139234501	0.216677227
ENSRNOG00000056907	Nipbl	160.7044533	0.1635917	0.109189898
ENSRNOG00000010332	Nipsnap3b	14.13497655	-0.059291793	0.4196253
ENSRNOG00000011445	Nkain1	9.441832033	-0.21397576	0.383539526
ENSRNOG00000029608	Nkain2	9.61560008	0.563047143	0.480116402
ENSRNOG00000031834	Nkain4	53.88216865	-0.059945869	0.177854672
ENSRNOG00000053813	Nkap	30.28191045	0.190339496	0.245670143
ENSRNOG00000009958	Nkapd1	32.35892748	-0.117421408	0.225291301
ENSRNOG00000014293	Nkd1	87.39751727	0.163668431	0.145700509
ENSRNOG00000043378	Nkpd1	21.45827777	0.107519204	0.325929883
ENSRNOG00000049128	Nktr	59.98353961	-0.07063424	0.178151524
ENSRNOG00000018147	Nkx6-3	56.77582402	-0.149547215	0.185099485
ENSRNOG00000032576	Nlgn1	175.1602516	-0.185887645	0.107983049
ENSRNOG00000015430	Nlgn2	58.58191512	-0.293141486	0.222396637
ENSRNOG00000011561	Nln	124.9184684	-0.039799883	0.14628698
ENSRNOG00000024210	Nlrc3	50.09671008	-0.068142822	0.197096173
ENSRNOG00000023847	Nlrp14	72.49549617	-0.367888066	0.154427209
ENSRNOG00000052386	Nlrx1	32.51875009	-0.389501349	0.651932648
ENSRNOG00000009310	Nmd3	64.84125591	-0.141064926	0.180555864
ENSRNOG00000020118	Nme5	33.05182144	0.055522291	0.217864562
ENSRNOG00000002898	Nme7	28.64002831	-0.240820757	0.242246681
ENSRNOG00000015962	Nmnat1	61.26335101	0.170969959	0.172567935
ENSRNOG00000027697	Nmnat2	56.61730081	0.059286326	0.18544352
ENSRNOG00000013585	Nmnat3	21.46652916	0.386789562	0.329022172
ENSRNOG00000012665	Nmrk1	46.6772285	-0.084041959	0.185199585
ENSRNOG00000042897	Nmrk2	106.4746625	0.023937196	0.134769549
ENSRNOG00000002989	Nmt1	71.29393716	-0.130630046	0.153137696
ENSRNOG00000026248	Nmt2	99.21713216	0.224721153	0.144326218
ENSRNOG00000005930	Nnmt	10.92689436	0.615305893	0.423419908
ENSRNOG00000026842	Nnt	94.5479397	-0.117782898	0.150482029
ENSRNOG00000002055	Noa1	129.8003858	-0.082060161	0.119679025
ENSRNOG00000021392	Noc2l	36.77434266	-0.011806217	0.304679003
ENSRNOG00000037478	Noc4l	155.8154291	-0.037275487	0.113590145
ENSRNOG00000010629	Nod1	23.24025427	-0.110960367	0.252143008
ENSRNOG00000000556	Nodal	146.6220025	0.128152305	0.123631556
ENSRNOG00000023683	Nog	140.0757424	0.119412451	0.114002081
ENSRNOG00000005344	Nol10	59.31525639	-0.238801904	0.175170153
ENSRNOG00000052934	Nol11	23.98143207	-0.18703518	0.265554602
ENSRNOG00000010209	Nol12	34.74321911	-0.21008144	0.220016064
ENSRNOG00000015588	Nol3	53.05392326	-0.217939452	0.181317077
ENSRNOG00000010417	Nol4l	13.43050409	-0.140249517	0.340943793
ENSRNOG00000046968	Nol8	60.35067045	-0.121751496	0.167254927
ENSRNOG00000048969	Nom1	28.32380011	-0.188010905	0.278514352
ENSRNOG00000003689	Nono	10.99472738	-0.239175581	0.527266004
ENSRNOG00000005184	Nop10	31.39958558	-0.150110438	0.251000588
ENSRNOG00000012147	Nop14	28.87296005	-0.05037237	0.23870084
ENSRNOG00000013023	Nop53	170.1239448	-0.248796075	0.105100928

ENSRNOG00000016486	Nop58	120.8586562	-0.015930426	0.119756005
ENSRNOG00000008696	Nopchap1	24.4241782	-0.546243115	0.239252006
ENSRNOG00000001130	Nos1	17.74224236	-0.045847217	1.002850989
ENSRNOG00000042929	Nos1ap	70.75898684	0.260516632	0.21397856
ENSRNOG00000057443	Nos2	45.99606013	0.506166496	0.222096319
ENSRNOG00000020543	Nosip	46.40913411	0.359157134	0.193273848
ENSRNOG00000019322	Notch1	124.7969261	0.526406635	0.125638771
ENSRNOG00000018835	Notch2	53.37463936	0.190491275	0.174735833
ENSRNOG00000036680	Notum	22.96321114	0.145933031	0.282753199
ENSRNOG00000031440	Nova1	123.5911232	-0.148872991	0.116049147
ENSRNOG00000013847	Nova2	44.4927976	0.028992965	0.223667857
ENSRNOG00000015376	Npas1	120.2604157	0.099545921	0.126843837
ENSRNOG00000013408	Npas2	44.89361489	0.479310463	0.226734449
ENSRNOG00000024934	Npat	99.72870156	0.113844898	0.149911747
ENSRNOG00000036686	Npb	44.42772175	0.102421575	0.194270011
ENSRNOG00000028384	Npepl1	72.55350894	-0.11662066	0.150696902
ENSRNOG00000015756	Nphp1	164.5836383	-0.066931595	0.141108982
ENSRNOG00000048978	Nphp3	12.150488	-0.225001523	0.476753695
ENSRNOG00000011967	Nphp4	59.56677612	0.107277629	0.191736869
ENSRNOG00000002775	Npl	33.98784257	0.046424099	0.235888586
ENSRNOG00000036698	Nploc4	43.92698375	0.543366501	0.235828131
ENSRNOG00000018854	Nppc	130.8712784	-0.106210056	0.147164407
ENSRNOG00000014684	Npr1	50.40940575	0.164587166	0.192409931
ENSRNOG00000020541	Nprl3	15.61817203	-0.453642359	0.339059537
ENSRNOG00000009029	Nptn	24.16700752	0.392373836	0.298691976
ENSRNOG00000014149	Npy1r	151.1971495	-0.166488782	0.112050712
ENSRNOG00000014172	Npy5r	31.66416363	-0.170701114	0.25957935
ENSRNOG00000012772	Nqo1	82.78913425	0.290042996	0.14814473
ENSRNOG00000017820	Nqo2	26.33139816	0.325140496	0.294761434
ENSRNOG00000009329	Nr1d1	45.37928104	0.119957056	0.291040541
ENSRNOG00000019812	Nr1h2	12.67747737	-0.024800275	0.468712811
ENSRNOG00000006983	Nr2c1	52.98207762	0.024513311	0.181454572
ENSRNOG00000010536	Nr2c2	28.93712652	-0.025124299	0.283188095
ENSRNOG00000050550	Nr2e1	11.0554481	-0.046491304	0.4028758
ENSRNOG00000010308	Nr2f2	47.38090828	-0.09327141	0.218662805
ENSRNOG00000016892	Nr2f6	16.65958992	-0.139789143	0.307695291
ENSRNOG00000005600	Nr4a2	102.7705964	0.100959764	0.203231905
ENSRNOG00000005964	Nr4a3	55.8166242	0.102048617	0.270336366
ENSRNOG00000009354	Nrarp	70.0909554	-0.057314149	0.181046073
ENSRNOG00000023079	Nras	18.55555463	-0.008322857	0.298906337
ENSRNOG00000000641	Nrbf2	176.4774263	0.118334094	0.110734209
ENSRNOG00000050570	Nrbp1	22.98247103	-0.368075121	0.344655132
ENSRNOG00000029535	Nrbp2	44.38840977	0.350884652	0.194657782
ENSRNOG00000007111	Nrdc	109.210156	-0.0978815	0.167110717
ENSRNOG00000008752	Nrf1	63.82096775	-0.3233804	0.164216214
ENSRNOG00000010392	Nrg1	30.32655448	-0.149055017	0.233657041
ENSRNOG00000001585	Nrip1	67.07575203	0.370251909	0.163037919
ENSRNOG00000010744	Nrp1	102.4724768	0.242410686	0.164293649
ENSRNOG00000031232	Nrp2	24.39571795	0.26185042	0.278747786
ENSRNOG00000001752	Nrros	23.25120235	-0.269028545	0.288181078

ENSRNOG00000048651	Nrtn	36.22054026	-0.199865493	0.213316685
ENSRNOG00000021103	Nrxn2	79.36868624	0.077546046	0.15258136
ENSRNOG00000055506	Nscme3l	23.4081048	0.022874447	0.908357235
ENSRNOG00000038140	Nsd2	125.8407614	-0.014655055	0.133233412
ENSRNOG00000015621	Nsd3	176.7602117	-0.041802347	0.251207966
ENSRNOG00000057814	Nsdhl	111.1845667	-0.039896463	0.137409734
ENSRNOG00000003905	Nsf	121.7411399	-0.039831743	0.135301181
ENSRNOG00000005700	Nsg1	80.65412276	-0.117692698	0.144438976
ENSRNOG00000010234	Nsmaf	24.74134626	-0.361342975	0.239650652
ENSRNOG00000015218	Nsmce1	9.109881131	0.041879074	0.457037938
ENSRNOG00000003964	Nsmce2	21.57178814	0.371160972	0.279648464
ENSRNOG00000017254	Nsun2	100.023197	-0.01555125	0.14281995
ENSRNOG00000046192	Nsun4	24.59773709	-0.146126548	0.253539315
ENSRNOG00000012020	Nsun4	49.9786244	0.020387537	0.192622691
ENSRNOG00000018520	Nsun6	159.9004225	0.030614978	0.111951866
ENSRNOG00000025494	Nsun7	179.9556362	-0.012435719	0.10909867
ENSRNOG00000020179	Nt5c2	118.5453558	-0.037438213	0.125956395
ENSRNOG00000005981	Nt5c3a	20.43868384	-0.188727039	0.287599478
ENSRNOG00000016475	Nt5c3b	58.14224144	0.394911079	0.386592571
ENSRNOG00000018358	Nt5dc2	103.0946884	0.303565714	0.86770543
ENSRNOG00000026793	Nt5dc3	14.89043256	-0.165065824	0.345570955
ENSRNOG00000011071	Nt5e	14.30834973	1.038046552	0.416773805
ENSRNOG00000003332	Nt5m	111.1918777	-0.01768224	0.139415274
ENSRNOG00000019716	Ntf3	25.187827	0.348240992	0.296014297
ENSRNOG00000006970	Ntn3	16.45097422	-0.329216519	0.320275598
ENSRNOG00000021016	Ntn5	9.920848878	0.042170596	0.440113798
ENSRNOG00000031136	Ntng1	38.36139742	0.06943145	0.223875179
ENSRNOG00000019922	Ntpcr	178.8113796	-0.111677758	0.123311154
ENSRNOG00000028708	Ntsr1	15.44605594	0.303784054	0.381737653
ENSRNOG00000000034	Nuak2	12.74361776	0.405541961	0.368874056
ENSRNOG00000009282	Nub1	24.97748816	-0.154575459	0.310412075
ENSRNOG00000027444	Nubpl	55.63403914	0.036108367	0.170206378
ENSRNOG00000020889	Nucb1	30.24653392	0.054378598	0.232294556
ENSRNOG00000020456	Nucb2	106.5497277	-0.005556976	0.147768508
ENSRNOG00000051720	Nudc	58.01849546	0.184197201	0.403523384
ENSRNOG00000024929	Nudcd1	43.62518273	0.064955777	0.189305767
ENSRNOG00000060914	Nudcd2	75.56201021	0.00012884	0.147346422
ENSRNOG00000022576	Nudt12	26.29551687	0.092384508	0.269096435
ENSRNOG00000014362	Nudt14	105.8181523	-0.070163597	0.138552183
ENSRNOG00000059386	Nudt19	158.0291859	0.017250691	0.115855819
ENSRNOG00000017420	Nudt6	85.60367451	0.121430818	0.155612299
ENSRNOG00000011976	Nudt7	24.19322416	0.259948037	0.281140355
ENSRNOG00000002186	Nudt9	47.98321461	-0.235190394	0.192108437
ENSRNOG00000001033	Nufip1	149.1497819	0.064568484	0.117882221
ENSRNOG00000024964	Nufip2	19.62621803	-0.447023903	0.277883165
ENSRNOG00000000417	Numa1	18.01391836	-0.214193842	0.395980787
ENSRNOG00000009653	Numb	16.7318511	-0.296889419	0.362023199
ENSRNOG00000006541	Nup107	11.12666468	0.235899067	0.372871901
ENSRNOG00000017919	Nup133	57.40353931	-0.055282649	0.170791513
ENSRNOG00000013411	Nup155	26.09772326	-0.02472227	0.247520707

ENSRNOG00000025185	Nup188	61.91544843	-0.490995432	0.829944802
ENSRNOG00000010852	Nup205	44.5911561	0.248164615	0.208796029
ENSRNOG00000005390	Nup210	70.68968825	-0.002157143	0.173738293
ENSRNOG00000023393	Nup214	109.826328	-0.033407951	0.17033972
ENSRNOG00000009290	Nup35	56.99542323	0.025803627	0.166000052
ENSRNOG00000004727	Nup37	46.72181223	-0.017174141	0.187819462
ENSRNOG00000049505	Nup43	16.36130092	0.165010759	0.348263781
ENSRNOG00000013423	Nup50	173.6457668	0.061670437	0.122413531
ENSRNOG00000012644	Nup58	121.7711917	0.079202909	0.130720257
ENSRNOG00000003673	Nup85	9.461180261	0.21248096	0.445539145
ENSRNOG00000006126	Nup88	99.80765591	-0.168011057	0.155085989
ENSRNOG00000018564	Nup93	48.51716949	-0.090971525	0.207429043
ENSRNOG00000020347	Nup98	40.89566919	0.089278051	0.200186841
ENSRNOG00000010435	Nupl2	78.51289913	0.115101802	0.176785322
ENSRNOG00000050922	Nupr1l1	9.53467471	0.113063694	0.467172172
ENSRNOG00000000917	Nupr2	62.47723486	0.053266508	0.174716264
ENSRNOG00000004921	Nusap1	61.18169833	-0.522888281	0.169037345
ENSRNOG00000018945	Nutf2	77.72594531	-0.491259799	0.155951709
ENSRNOG00000003629	Nvl	56.41898983	0.035821277	0.169265496
ENSRNOG00000052129	Nwd1	14.09017894	0.443802254	0.379378561
ENSRNOG00000051837	Nwd2	105.7874925	0.064377676	0.138632489
ENSRNOG00000008178	Nxn	43.74539649	0.204967829	0.20759745
ENSRNOG00000001608	Nxpe3	50.48580024	0.263437714	0.184465807
ENSRNOG00000004700	Nxt1	54.28976956	0.189276337	0.176007267
ENSRNOG00000019168	Nxt2	14.17251362	0.095216159	0.363729197
ENSRNOG00000024641	Nyap1	185.3065027	-0.090768543	0.102492639
ENSRNOG00000033220	Oas1k	25.36773577	0.121221436	0.262123521
ENSRNOG00000028814	Oasl2	81.33427119	0.048954488	0.179983604
ENSRNOG00000015953	Oaz2	25.56980666	0.190095082	0.283671944
ENSRNOG00000009690	Obi1	43.47496616	-0.035687317	0.213588415
ENSRNOG00000025516	Oc90	8.686514039	-0.2329994	0.470317565
ENSRNOG00000001031	Ocm	14.57904047	0.150879918	0.359464757
ENSRNOG00000003875	Ocrl	9.018321406	0.32681288	0.43264711
ENSRNOG00000014584	Odf2	115.3034481	-0.165343775	0.119058473
ENSRNOG00000014059	Odf2l	15.27127093	-0.223657119	0.332326555
ENSRNOG00000012858	Odf3	8.70746492	0.173297923	0.511716371
ENSRNOG00000008199	Odf3l2	67.0465958	0.235350555	0.181902617
ENSRNOG00000002473	Odr4	32.58560597	0.311634355	0.241992966
ENSRNOG00000017822	Oga	103.7430986	-0.235809845	0.164629726
ENSRNOG00000005130	Ogdh	115.9417658	0.318342488	0.125924143
ENSRNOG00000001081	Ogfod2	19.45097276	0.397327427	0.313375369
ENSRNOG00000036668	Ogfod3	58.96008938	-0.392935911	0.157269599
ENSRNOG00000009355	Ogfr	178.0146052	0.013061907	0.10703024
ENSRNOG00000014142	Ogfrl1	80.09443158	-0.203208364	0.140067842
ENSRNOG00000003359	Ogt	136.5121977	0.267371528	0.131633143
ENSRNOG00000020519	Olfm2	39.71738321	0.527825872	0.204603257
ENSRNOG00000017969	Olfm3	157.8929054	0.103778978	0.163119128
ENSRNOG00000014034	Olfml2a	51.65862698	0.3468557	0.189407463
ENSRNOG00000003018	Olfml2b	48.38759928	0.339962919	0.177108275
ENSRNOG00000019211	Olfml3	25.33866293	-0.18438562	0.287830941

ENSRNOG00000021988	Olfr225	53.78562535	0.160441591	0.181223514
ENSRNOG00000049480	Olfr692	76.21711082	-0.10359007	0.154147134
ENSRNOG00000028658	Olig2	162.4430608	-0.07677715	0.19367015
ENSRNOG00000018606	Olr59	56.32313202	0.513247157	0.198092565
ENSRNOG00000007214	Oma1	23.60786441	-0.086340887	0.26996468
ENSRNOG00000039560	Omd	9.715234877	0.223625727	0.444916318
ENSRNOG00000001717	Opa1	53.58119543	0.208020015	0.181003361
ENSRNOG00000025890	Opa3	44.48771092	0.016622678	0.286594363
ENSRNOG00000023809	Opcml	96.87793657	0.281966526	0.141560712
ENSRNOG00000026573	Ophn1	9.258436028	0.945153072	0.446736233
ENSRNOG00000011781	Oplah	14.48299823	-0.025369517	0.343587082
ENSRNOG00000016768	Oprl1	67.71913663	0.026100559	0.163840049
ENSRNOG00000003059	Optc	9.350541304	-0.499416177	0.50764361
ENSRNOG00000001336	Orai1	92.34308307	0.227731783	0.155984453
ENSRNOG00000001427	Orai2	43.99433188	0.133041095	0.219599363
ENSRNOG00000039730	Orai3	96.68927748	0.029854801	0.179904034
ENSRNOG00000008841	Orc1	22.12563466	0.478925923	0.310074251
ENSRNOG00000012558	Orc2	17.29454773	-0.107096459	0.320619531
ENSRNOG00000008314	Orc3	49.56741592	0.029976285	0.17951483
ENSRNOG00000005021	Orc4	115.3911366	-0.125175852	0.122428322
ENSRNOG00000011519	Orc5	96.81247837	0.321129073	0.164185286
ENSRNOG00000024043	Orc6	45.19738405	-0.067123823	0.185976543
ENSRNOG00000025570	Os9	32.19836153	0.163649675	0.250074036
ENSRNOG00000011435	Osbpl10	34.23685273	0.008450598	0.238423785
ENSRNOG00000001782	Osbpl11	75.75659517	0.096811746	0.141014442
ENSRNOG00000010011	Osbpl3	44.96675138	0.194220121	0.232028836
ENSRNOG00000020713	Osbpl5	82.50205886	0.109023184	0.160790554
ENSRNOG00000009473	Osbpl7	75.89877415	-0.233152285	0.143366246
ENSRNOG00000026962	Osbpl8	102.845605	0.293596755	0.141243059
ENSRNOG00000033593	Osbpl9	70.97752942	-0.046299112	0.154697238
ENSRNOG00000008297	Oser1	77.88081063	-0.007176168	0.141496882
ENSRNOG00000009333	Osgep	43.42737354	0.093646664	0.25437769
ENSRNOG00000004001	Osgepl1	15.35357616	0.048619011	0.325688043
ENSRNOG00000009358	Osgin2	17.82118417	0.156994576	0.354082926
ENSRNOG00000033192	Osmr	13.43964736	0.293255613	0.394242478
ENSRNOG00000004210	Osr1	16.62551726	-0.358381928	0.309662444
ENSRNOG00000009967	Otof	25.14937594	0.236986126	0.264309623
ENSRNOG00000010983	Otog	11.95547927	-0.467359994	0.48687542
ENSRNOG00000017037	Otud3	23.46032302	0.206172967	0.291618064
ENSRNOG00000018477	Otud4	14.37528803	0.281036653	0.325556357
ENSRNOG00000008764	Otud5	16.07535899	0.208111549	0.383858409
ENSRNOG00000006636	Otud6b	140.7590958	0.276068678	0.109547899
ENSRNOG00000015503	Otud7a	52.69140151	-0.186431296	0.18370179
ENSRNOG00000042068	Otud7b	15.80269451	0.122795907	0.321919691
ENSRNOG00000020669	Ovol1	25.97608814	-0.02852281	0.264654719
ENSRNOG00000006850	Ovol2	80.19567324	-0.120962012	0.161397654
ENSRNOG00000009713	Oxa1l	23.55420138	-0.14445004	0.267730268
ENSRNOG00000047517	Oxld1	43.58898416	-0.042113784	0.195767562
ENSRNOG00000019760	Oxnad1	48.46286561	0.174684442	0.215061241
ENSRNOG00000005993	Oxsm	25.66616053	-0.238701201	0.250253209

ENSRNOG00000013136	Oxsr1	125.7827418	-0.145081118	0.121520299
ENSRNOG00000019208	P2rx5	9.090072915	0.66213561	0.458176083
ENSRNOG00000001873	P2rx6	67.64153972	-0.209565102	0.165384295
ENSRNOG00000001296	P2rx7	30.21232568	0.234750057	0.294131849
ENSRNOG00000013902	P2ry12	10.78266977	-0.235414824	0.406641702
ENSRNOG00000013872	P2ry14	10.99158858	0.283629248	0.439819113
ENSRNOG00000019270	P2ry6	28.21170901	0.43306873	0.245061639
ENSRNOG00000053991	P3h1	26.83395657	0.084664429	0.23635079
ENSRNOG00000016143	P3h3	79.09285762	0.701038106	0.389480524
ENSRNOG00000015787	P3h4	85.52884755	-0.269836013	0.14397887
ENSRNOG00000050655	P4ha1	60.35068066	-0.012793563	0.203139596
ENSRNOG00000033663	P4ha2	150.7117692	-0.045029982	0.119546672
ENSRNOG00000017118	P4ha3	19.14870005	0.172491662	0.305743751
ENSRNOG00000020249	P4htm	8.805187196	0.497200106	0.443475382
ENSRNOG00000051051	Pabir1	108.019573	-0.015434247	0.129118119
ENSRNOG00000012750	Pabpc1l	69.56645196	-0.250440378	0.180050486
ENSRNOG00000029558	Pabpn1l	101.4834625	-0.093228195	0.152547816
ENSRNOG00000003915	Pacc1	19.0207813	0.014512021	0.301373984
ENSRNOG00000020350	Pacs1	24.25861769	-0.233598747	0.257173073
ENSRNOG00000014744	Pacs2	8.714793184	-0.036764892	0.495561271
ENSRNOG00000007574	Padi2	80.04061027	0.124983439	0.171954233
ENSRNOG00000019746	Paf1	27.01449208	1.175007749	0.80510547
ENSRNOG00000002755	Pafah1b1	188.4753475	0.090025018	0.102137117
ENSRNOG00000022288	Pafah2	17.22276368	0.341465395	0.34128127
ENSRNOG00000004302	Pah	11.043202	0.019081066	0.425372307
ENSRNOG00000002101	Paics	21.66791139	-0.377904366	0.25961284
ENSRNOG00000019883	Pak4	40.90198094	0.094846262	0.206960378
ENSRNOG00000005509	Pak5	162.2083877	-0.201681121	0.156709195
ENSRNOG00000007925	Pak6	169.7002486	-0.065463301	0.137750484
ENSRNOG00000000561	Pald1	43.17938458	0.031373413	0.240691256
ENSRNOG00000010107	Palldl1	67.13639485	-0.084395283	0.17444162
ENSRNOG00000009760	Palm	183.8391841	-0.432550109	0.463262477
ENSRNOG00000058609	Palmd	48.4292728	-0.069092538	0.187175002
ENSRNOG00000004608	Pam16	144.0680893	-0.074191887	0.132420675
ENSRNOG00000005348	Pamr1	183.3420308	0.05751358	0.110823961
ENSRNOG00000032441	Pan2	14.74836363	0.254881471	0.336262262
ENSRNOG00000000942	Pan3	48.87452909	0.218469173	0.266999217
ENSRNOG00000018944	Pank1	49.83226686	-0.209174704	0.278870174
ENSRNOG00000018838	Paox	39.60306347	-0.187656628	0.215562527
ENSRNOG00000009448	Papln	109.2597528	-0.008470579	0.153438941
ENSRNOG00000004827	Papola	166.1707556	-0.009851576	0.111002481
ENSRNOG00000006261	Papolg	60.007502	0.223413511	0.175481015
ENSRNOG00000042860	Pappa2	9.901243916	0.417717091	0.484176698
ENSRNOG00000011068	Papss2	39.46294458	0.12170914	0.242563337
ENSRNOG00000002035	Paqr3	171.4295793	0.057623366	0.114550029
ENSRNOG00000014164	Paqr5	20.38887667	-0.245590957	0.295846349
ENSRNOG00000022054	Paqr7	59.51060432	-0.421004739	0.190882625
ENSRNOG00000032437	Pard3	38.17423854	0.16523776	0.222309398
ENSRNOG00000010883	Pard6b	30.97774097	0.132220361	0.231781066
ENSRNOG00000055157	Pard6g	15.58106894	0.744767809	0.444903912

ENSRNOG00000019978	Parg	122.2684095	0.169825735	0.129904617
ENSRNOG00000023271	Parl	23.68931712	-0.103648636	0.263083692
ENSRNOG00000002912	Parn	97.42529645	-0.079122692	0.150188471
ENSRNOG00000003084	Parp1	172.8801958	-0.01952578	0.131469506
ENSRNOG00000060689	Parp11	35.7582773	0.003635113	0.257731786
ENSRNOG00000008196	Parp12	25.79395457	-0.13034056	0.262264984
ENSRNOG00000023334	Parp14	12.47410141	0.662997498	0.382858579
ENSRNOG00000029127	Parp16	8.341258107	0.129720822	0.410196714
ENSRNOG00000012865	Parp3	14.68895906	0.350687294	0.341878768
ENSRNOG00000010824	Parp8	73.98861472	0.237317609	0.18793681
ENSRNOG00000023463	Parp9	35.2773691	0.528436979	0.249603215
ENSRNOG00000007327	Pars2	42.84680422	0.06795502	0.202123827
ENSRNOG00000015713	Parva	65.37076868	0.111361058	0.156381925
ENSRNOG00000016888	Pask	30.98558613	-0.175259308	0.222173288
ENSRNOG00000007551	Patj	16.52643827	0.081897666	0.32740767
ENSRNOG00000021052	Patl1	98.06266672	0.114375577	0.156476281
ENSRNOG00000018709	Patz1	141.949606	0.193261923	0.11944245
ENSRNOG00000005917	Pawr	13.42779051	-0.047681538	0.375104033
ENSRNOG00000002036	Paxbp1	20.4339903	0.035414166	0.354518124
ENSRNOG00000007131	Paxip1	166.1241306	-0.040135291	0.119956068
ENSRNOG00000002662	Pbdc1	163.7349513	0.055718649	0.121323727
ENSRNOG00000000386	Pbld1	36.921857	-0.138003702	0.22257211
ENSRNOG00000028227	Pbrm1	149.1197742	-0.746003039	0.697624466
ENSRNOG00000022162	Pbx3	40.10677326	0.275718055	0.251522289
ENSRNOG00000010736	Pbx4	49.88110278	-0.133720116	0.195804485
ENSRNOG00000020673	Pbxip1	169.7346747	0.001327589	0.11381793
ENSRNOG00000000566	Pcbd1	8.310241059	0.092988024	0.417580212
ENSRNOG00000036852	Pcbp2	56.88378895	0.261299887	0.17944406
ENSRNOG00000012406	Pcbp4	82.92829312	0.040087288	0.158969629
ENSRNOG00000057042	Pcca	29.00653172	-0.525918189	0.280400639
ENSRNOG00000015869	Pccb	102.2865892	0.049458834	0.135666278
ENSRNOG00000060410	Pcdh1	45.86304339	-0.171228232	0.218249863
ENSRNOG00000000606	Pcdh15	11.97689065	0.539671014	0.42070196
ENSRNOG00000009949	Pcdh18	17.67036942	0.62699817	0.335967945
ENSRNOG00000003929	Pcdh19	183.9157694	0.100664482	0.125998257
ENSRNOG00000013306	Pcdh20	124.1819409	0.086834551	0.160462111
ENSRNOG00000012367	Pcdh7	95.09295126	0.076855112	0.195610753
ENSRNOG00000013101	Pcdh8	69.21169421	0.033612594	0.178573113
ENSRNOG00000038068	Pcdh9	64.94642964	0.028310542	0.18729957
ENSRNOG00000029244	Pcdhb10	10.51882305	-0.186237298	0.458240525
ENSRNOG00000039471	Pcdhb16l	8.210367941	0.01434375	0.463766414
ENSRNOG00000020062	Pcdhb6l	13.36850586	-0.190767545	0.359619274
ENSRNOG00000020072	Pcdhb9	23.52091328	0.245166129	0.294907237
ENSRNOG00000021221	Pced1a	145.2548722	-0.054154122	0.107117028
ENSRNOG00000022807	Pced1b	50.38236234	0.110740208	0.177257257
ENSRNOG00000009891	Pcf11	75.88873086	0.028562343	0.188247073
ENSRNOG00000051433	Pcgf1	62.41452971	-0.275274372	0.174283192
ENSRNOG00000000062	Pcgf3	46.92778857	0.31724647	0.198715578
ENSRNOG00000018532	Pcgf5	90.354023	0.072648504	0.138935008
ENSRNOG00000020250	Pcgf6	54.92781723	0.221522726	0.17790722

ENSRNOG00000016870	Pcif1	58.00228067	0.037737977	0.184802213
ENSRNOG00000018536	Pck2	158.1734016	-0.350229425	0.484409443
ENSRNOG00000010155	Pcm1	81.25387457	-0.010111815	0.181387823
ENSRNOG00000014330	Pcmt1	166.8515803	0.12890679	0.107985853
ENSRNOG00000017404	Pcmtd2	52.94693861	-0.055677899	0.227746886
ENSRNOG00000028411	Pcnp	91.35097515	0.151815688	0.147975353
ENSRNOG00000001276	Pcnt	55.71441325	0.175593122	0.209204686
ENSRNOG00000007459	Pcnx1	145.7526031	0.084373037	0.124044913
ENSRNOG00000028580	Pcnx2	26.35843856	0.038122531	0.273475617
ENSRNOG00000020742	Pcnx3	10.62349177	0.653489315	0.445705868
ENSRNOG00000025001	Pcolce	42.8442622	0.074068564	0.21935745
ENSRNOG00000046848	PCOLCE2	13.77507287	0.451037533	0.450890098
ENSRNOG00000011107	Pcsk1	127.6774706	0.100339626	0.125984413
ENSRNOG00000012036	Pcsk5	127.245661	0.064492734	0.12235901
ENSRNOG00000011526	Pcsk6	148.4830856	-0.234034829	0.394950588
ENSRNOG00000017478	Pcsk7	52.93085401	0.273111056	0.21281659
ENSRNOG00000016704	Pcyox1	33.13461175	0.286340186	0.702577034
ENSRNOG00000001762	Pcyt1a	11.16147034	0.896929346	0.403599305
ENSRNOG00000036684	Pcyt2	16.12083674	-0.600639279	0.31769538
ENSRNOG00000000990	Pdap1	31.34635922	-0.280102164	0.262034568
ENSRNOG00000010147	Pdcd10	130.4797272	-0.247241198	0.129445268
ENSRNOG00000020304	Pdcd11	11.82619228	-0.013130003	0.399896837
ENSRNOG00000001490	Pdcd2	55.24271622	0.131220704	0.172756203
ENSRNOG00000021119	Pdcd2l	54.16294027	-0.144203615	0.215574308
ENSRNOG00000014779	Pdcd4	125.5901351	-0.150974744	0.119080045
ENSRNOG00000008484	Pdcl	87.1675039	-0.18311857	0.148429888
ENSRNOG00000013286	Pdcl3	105.5612789	-0.108817896	0.128077462
ENSRNOG00000011310	Pde10a	63.60262433	-0.105061903	0.170779204
ENSRNOG00000059442	Pde12	82.574003	-0.390418591	0.158247016
ENSRNOG00000054212	Pde1a	61.73191564	-0.227540945	0.172157677
ENSRNOG00000036828	Pde1b	13.58079662	-0.553105068	0.410055418
ENSRNOG00000011417	Pde3b	18.78619355	0.060601058	0.356675541
ENSRNOG00000042536	Pde4d	13.03806772	-0.065360366	0.4860059
ENSRNOG00000017816	Pde6a	34.69412382	0.301188406	0.218867373
ENSRNOG00000018610	Pde6d	158.6852042	-0.255103465	0.14478662
ENSRNOG00000013048	Pde7a	24.74031758	0.03336651	0.285198516
ENSRNOG00000013436	Pde7b	15.50601418	-0.044571741	0.336862674
ENSRNOG00000019005	Pde8a	8.621773935	0.227705829	0.473657739
ENSRNOG00000010280	Pde8b	46.65930295	-0.050261476	0.195556686
ENSRNOG00000001174	Pde9a	94.30386681	0.124662914	0.146523498
ENSRNOG00000017197	Pdgfb	105.1386578	-0.047181502	0.162026763
ENSRNOG00000010695	Pdgfc	55.4196368	0.075715882	0.200137679
ENSRNOG00000029148	Pdgfd	14.95732374	-0.013634425	0.313852025
ENSRNOG00000002244	Pdgfra	116.0445638	0.18635227	0.129474987
ENSRNOG00000018461	Pdgfrb	73.04715389	0.364378312	0.195744691
ENSRNOG00000010832	Pdgfrl	31.30007033	0.006273016	0.228314447
ENSRNOG00000014745	Pdha1l1	11.04574811	0.209419029	0.393950303
ENSRNOG00000006947	Pdhx	100.0929864	0.099067027	0.15448797
ENSRNOG00000015018	Pdia3	44.71202249	0.059693204	0.220280327
ENSRNOG00000032327	Pdia5	14.69992727	0.14828615	0.343204387

ENSRNOG00000050197	Pdia6	10.14491388	-0.305121912	0.43962929
ENSRNOG00000016536	Pdik1l	60.00640504	0.072252129	0.157381449
ENSRNOG00000004172	Pdk2	18.44557973	-2.74133E-05	0.283371006
ENSRNOG00000012513	Pdk3	123.6949215	-0.137993302	0.12258562
ENSRNOG00000016166	Pdlim1	22.25353445	-0.18945063	0.298168784
ENSRNOG00000008543	Pdlim2	111.9053187	-0.060405393	0.130303193
ENSRNOG00000012658	Pdlim3	27.74537699	0.178822696	0.261629281
ENSRNOG00000016419	Pdlim5	26.25060542	-0.297776976	0.28550102
ENSRNOG00000012343	Pdp2	24.31557029	0.115868474	0.241663238
ENSRNOG00000006136	Pdpk1	168.0294946	-0.023515676	0.162649577
ENSRNOG00000014961	Pdpn	87.4759487	-0.074484478	0.179279043
ENSRNOG00000022593	Pdpr	39.22977695	0.185150152	0.210279169
ENSRNOG00000008845	Pdrg1	11.09978121	0.722907729	0.508885165
ENSRNOG00000002541	Pds5a	19.6661377	-0.112873972	0.406957763
ENSRNOG00000048849	Pdss1	92.1416421	-0.058182404	0.14465261
ENSRNOG00000042962	Pdss2	78.8658933	-0.206141731	0.160815509
ENSRNOG00000002407	Pdxdc1	20.83649401	-0.407186682	0.271433511
ENSRNOG00000049937	Pdxk	51.41018786	-0.294131677	0.205291484
ENSRNOG00000026036	Pdyn	99.21988245	-0.267918991	0.168203688
ENSRNOG00000013140	Pdzd2	23.90229912	-0.194679629	0.282621908
ENSRNOG00000008526	Pdzd3	50.34954364	0.064192279	0.197609214
ENSRNOG00000056174	Pdzd4	30.46190689	0.047288279	0.232895503
ENSRNOG00000009460	Pdzd8	19.09976413	-0.204854893	0.405748635
ENSRNOG00000016292	Pdzd9	24.99434231	-0.313727675	0.247288827
ENSRNOG00000000096	Pdzk1	68.68114612	0.581597222	0.176133531
ENSRNOG00000057556	Pdzrn3	166.3084162	0.147478801	0.124366836
ENSRNOG00000022844	Pdzrn4	86.71090396	-0.227923191	0.152393633
ENSRNOG00000042519	Peak1	14.39860765	0.365998193	0.352887443
ENSRNOG00000055295	Pecr	51.23088276	-0.256707219	0.184487878
ENSRNOG00000006329	Peli1	145.3407219	-0.058029258	0.123451147
ENSRNOG00000012363	Peli2	16.06685303	-0.38222032	0.338100601
ENSRNOG00000019950	Peli3	33.26500456	0.122877494	0.248685719
ENSRNOG00000061128	Pelo	107.9502445	-0.004946507	0.142173552
ENSRNOG00000054423	Pemt	17.93757928	0.127595175	0.314719303
ENSRNOG00000007387	Per1	26.30163911	-0.169402594	0.298278161
ENSRNOG00000020254	Per2	49.49741158	-0.325205057	0.220815473
ENSRNOG00000018413	Per3	101.6300641	0.23387131	0.156811667
ENSRNOG00000011994	Perp	32.97649504	-0.012649479	0.2318871
ENSRNOG00000004515	Pes1	11.98807512	0.398569666	0.379338933
ENSRNOG00000024012	Pex10	91.9494002	-0.097808457	0.157961576
ENSRNOG00000054896	Pex13	132.2309156	-0.091919365	0.136395562
ENSRNOG00000006539	Pex16	17.42909043	0.028532427	0.344734605
ENSRNOG00000008748	Pex2	127.7082543	-0.117555715	0.112574578
ENSRNOG00000049982	Pex26	100.4780889	-0.193257759	0.132266299
ENSRNOG00000015660	Pex3	115.6176715	-0.085335724	0.134890998
ENSRNOG00000016655	Pex6	10.99506615	0.300617306	0.375299175
ENSRNOG00000012322	Pex7	59.76387961	0.149471761	0.182354784
ENSRNOG00000005193	Pfas	98.99348352	0.156564914	0.146270879
ENSRNOG00000050932	Pfdn4	47.19479925	-0.12772242	0.179361185
ENSRNOG00000004162	Pfkfb2	98.51294043	0.035773808	0.129598754

ENSRNOG00000018911	Pfkfb3	105.0516206	-0.032059676	0.15000141
ENSRNOG00000020656	Pfkfb4	15.95769903	-0.366623822	0.305351605
ENSRNOG00000001214	Pfkl	39.0532045	0.084660379	0.217254269
ENSRNOG00000057988	Pfkm	80.89456319	-0.64823636	0.190806191
ENSRNOG00000017163	Pfkp	93.94661	0.019552966	0.142624554
ENSRNOG00000017427	Pfn2	18.34943448	0.29809273	0.321710317
ENSRNOG00000050386	Pfn4	26.32542703	0.480542036	0.807919637
ENSRNOG00000013532	Pgam2	9.040315097	0.058644351	0.438548448
ENSRNOG00000013388	Pgap1	24.13686497	0.742594162	0.298424883
ENSRNOG00000020371	Pgap2	40.82312082	-0.018213356	0.201525923
ENSRNOG00000020374	Pgap6	13.10970772	0.2516285	0.343496939
ENSRNOG00000026791	Pgbd5	37.80212566	0.068089498	0.223625513
ENSRNOG00000014492	Pgc	10.41464346	-0.738648724	0.488495875
ENSRNOG00000005650	Pgf	12.27138954	0.260543664	0.400237977
ENSRNOG00000003541	Pggt1b	24.89073893	0.121295149	0.264593893
ENSRNOG00000018326	Pgls	42.62411431	-0.003042941	0.239729772
ENSRNOG00000002185	Pgm2	88.1860848	0.245931361	0.143564808
ENSRNOG00000009515	Pgm3	12.503661	0.065916089	0.349158111
ENSRNOG00000009536	Pgp	25.51398638	0.114611557	0.251899087
ENSRNOG00000019639	Pgpep1	22.98137185	-0.393470552	0.251915021
ENSRNOG00000006831	Pgr	31.95241182	0.11768159	0.242620886
ENSRNOG00000012786	Pgrmc1	12.38911904	0.065079265	0.385514999
ENSRNOG00000002949	Pgs1	20.79033585	0.29121262	0.278184422
ENSRNOG00000014459	Phax	58.16347269	-0.43317264	0.178161047
ENSRNOG00000006004	Phc2	15.51688276	0.478757963	0.343056812
ENSRNOG00000009491	Phc3	31.10021297	0.571688443	0.24082669
ENSRNOG00000028206	Pheta1	24.35386689	0.283281407	0.377717225
ENSRNOG00000061312	Phf10	20.03339125	0.096666489	0.296960029
ENSRNOG00000009566	Phf12	133.8939325	0.025126997	0.128335873
ENSRNOG00000016816	Phf2	79.72226117	-0.051288775	0.157209773
ENSRNOG00000019948	Phf20	162.2401645	0.166685163	0.10775963
ENSRNOG00000005486	Phf20l1	57.64764747	0.044337115	0.169720174
ENSRNOG00000006063	Phf21a	19.27973613	-0.07863184	0.314791386
ENSRNOG00000013067	Phf21b	11.10007584	0.405063636	0.406612488
ENSRNOG00000017657	Phf23	91.68113084	0.012526835	0.141345888
ENSRNOG00000000129	Phf24	40.46491009	0.192050072	0.201431163
ENSRNOG00000011756	Phf3	171.1862029	-0.049248774	0.120731178
ENSRNOG00000024170	Phf5a	162.4966914	-0.00561744	0.120411893
ENSRNOG00000002276	Phf6	92.84776844	0.271885744	0.16527695
ENSRNOG00000018996	Phf7	89.25769448	0.180527684	0.14567551
ENSRNOG00000002708	Phf8	65.30643622	0.107477237	0.193719448
ENSRNOG00000008652	Phip	155.6589565	0.042760732	0.113924698
ENSRNOG00000003063	Phka1	55.79938873	-0.214257144	0.213853052
ENSRNOG00000024101	Phkb	20.18317208	-0.039374385	0.281061053
ENSRNOG00000018725	Phkg2	10.53303935	1.067400946	0.424955234
ENSRNOG00000009068	Phlda3	95.33445938	0.097805713	0.141913327
ENSRNOG00000026985	Phldb1	19.62801825	0.11678711	0.349136262
ENSRNOG00000016108	Phlpp2	168.3412573	0.197862878	0.113861212
ENSRNOG00000005569	Phospho1	13.85789103	0.544441931	0.373199508
ENSRNOG00000007979	Phospho2	141.5693252	-0.07977206	0.131596348

ENSRNOG00000019706	Phox2a	64.61275275	0.123410768	0.175216239
ENSRNOG00000017299	Phrf1	21.91049934	-0.14083616	0.282095624
ENSRNOG00000019785	Phtf1	187.6920957	-0.070194188	0.12719228
ENSRNOG00000013517	Phtf2	27.62780255	0.314050235	0.291601302
ENSRNOG00000016794	Phyhd1	156.1353261	-0.198629113	0.138998542
ENSRNOG00000010555	Phyhip	141.3532383	-0.099161686	0.108902473
ENSRNOG00000000274	Phyhipl	170.6569075	-0.013509866	0.112841051
ENSRNOG00000000525	Pi16	24.09640162	-0.205559385	0.286673116
ENSRNOG00000014675	Pi4k2a	38.97333598	0.14692431	0.220152176
ENSRNOG00000060045	Pi4ka	63.77307695	-0.105775218	0.206246214
ENSRNOG00000021024	Pi4kb	106.3927954	0.211143325	0.164663942
ENSRNOG00000034272	Pias1	158.2058311	0.235631782	0.118832554
ENSRNOG00000017493	Pias2	94.12233994	0.025484083	0.137293465
ENSRNOG00000009208	Pibf1	31.58314119	-0.198987185	0.255467036
ENSRNOG00000049179	Pidd1	20.25214459	-0.758680547	1.16311164
ENSRNOG00000056786	Piezo1	12.57679444	0.373226083	0.930235975
ENSRNOG00000038784	Piezo2	7.931878971	0.194414972	0.484548185
ENSRNOG00000015325	Pigf	19.3670572	-0.09306348	0.301191987
ENSRNOG00000010914	Pigh	39.95504879	-0.020257173	0.258376402
ENSRNOG00000003070	Pigl	39.81228098	0.306921382	0.221148923
ENSRNOG00000014866	Pign	10.5289357	0.144969724	0.440105185
ENSRNOG00000011366	Pigs	8.93635749	-0.267921958	0.417451068
ENSRNOG00000025181	Pigu	32.43700791	-0.130690481	0.208814075
ENSRNOG00000000121	Pigv	42.39513142	-0.134528883	0.206868083
ENSRNOG00000033623	Pigx	163.478697	-0.136649037	0.118755289
ENSRNOG00000020634	Pih1d1	14.31494337	-0.601469324	0.326996036
ENSRNOG00000013309	Pik3ap1	9.58966488	0.345141005	0.407937991
ENSRNOG00000020479	Pik3c2a	33.78503304	0.153697604	0.240387652
ENSRNOG00000029938	Pik3c2b	64.19986546	0.012299551	0.197504776
ENSRNOG00000017840	Pik3c3	113.8826367	0.015815598	0.139947197
ENSRNOG00000016384	Pik3cb	9.096601764	-0.161533201	0.477167769
ENSRNOG00000009385	Pik3cg	13.45302144	0.184584792	0.386168443
ENSRNOG00000018859	Pik3ip1	10.9713063	0.334939745	0.370665179
ENSRNOG00000019228	Pik3r2	36.106657	0.338894674	0.219827928
ENSRNOG00000000145	Pik3r3	30.96725442	0.498240314	0.269292517
ENSRNOG00000013669	Pik3r4	12.16848816	-0.095572983	0.356380984
ENSRNOG00000023428	Pik3r5	9.611130984	0.595296949	0.428030479
ENSRNOG00000015158	Pikfyve	115.6609849	0.14267698	0.117824115
ENSRNOG00000000529	Pim1	18.70014788	-0.03694996	0.401169698
ENSRNOG00000008177	Pim2	74.4108015	0.166504363	0.198316927
ENSRNOG00000029698	Pim3	171.1430998	0.055083531	0.118720379
ENSRNOG00000020474	Pin1	163.5545268	-0.072877099	0.122493144
ENSRNOG00000012012	Pinx1	58.03476448	0.1111324	0.190931797
ENSRNOG00000013030	Pip4k2b	16.58298157	0.635248318	0.365019539
ENSRNOG00000005138	Pip4k2c	10.32501453	0.523771558	0.405066431
ENSRNOG00000009948	Pip4p1	19.02353852	0.044353758	0.356659367
ENSRNOG00000021068	Pip5k1a	138.0206288	-0.128074588	0.117600166
ENSRNOG00000015232	Pip5k1b	149.1525506	-0.0533137	0.128264946
ENSRNOG00000020577	Pip5k1c	162.3922028	0.216756095	0.434844587
ENSRNOG00000010873	Pithd1	81.27796731	1.533379579	0.623423954

ENSRNOG00000003846	Pitpna	125.7130888	0.053536894	0.118275163
ENSRNOG00000000665	Pitpnb	17.10673393	-0.372317882	0.298080406
ENSRNOG00000018553	Pitpnm1	10.318861	-0.081684646	0.382171966
ENSRNOG00000029260	Pitpnm2	60.57042329	0.005510244	0.156242253
ENSRNOG00000008323	Pitpnm3	50.38157454	0.049490104	0.189824028
ENSRNOG00000009871	Piwil2	13.85882949	0.556757417	0.371812728
ENSRNOG00000010771	Pkd1	37.08961487	0.207889249	0.208499911
ENSRNOG00000052310	Pkd1l3	21.29164036	0.004579823	0.279985293
ENSRNOG00000002146	Pkd2	52.94232867	0.12544396	0.19516816
ENSRNOG00000004205	Pkdcc	56.48801536	0.159112378	0.174561453
ENSRNOG00000012095	Pkia	17.42566111	0.115745255	0.301100232
ENSRNOG00000000811	Pkib	18.23503986	0.039867584	0.305690516
ENSRNOG00000010235	Pkig	55.91474032	0.285408919	0.220471278
ENSRNOG00000004131	Pkn1	48.70562664	0.016078122	0.198736914
ENSRNOG00000011317	Pkn2	107.6189344	-0.092262032	0.126539427
ENSRNOG00000025892	Pkn3	145.9071724	-0.07606263	0.118625953
ENSRNOG00000001184	Pknox1	9.049966024	-0.063436702	0.388325143
ENSRNOG00000001825	Pkp2	26.40226968	0.154133652	0.274582694
ENSRNOG00000019859	Pla2g15	124.8734254	0.060525914	0.1534077
ENSRNOG00000016647	Pla2g2c	22.76971254	-0.207613483	0.274381088
ENSRNOG00000002657	Pla2g4a	26.48033926	0.133974353	0.34190241
ENSRNOG00000007447	Pla2g4b	25.61959695	0.061025283	0.297775705
ENSRNOG00000024904	Pla2g4e	34.52769686	0.117504983	0.23340828
ENSRNOG00000008135	Pla2g4f	46.02857026	0.174722455	0.201938631
ENSRNOG00000012295	Pla2g6	23.12660497	-0.163926677	0.331070908
ENSRNOG00000025691	Pla2g7	17.38037383	-0.091142423	0.382921783
ENSRNOG00000007753	Plaa	120.2285829	0.091462127	0.125766931
ENSRNOG00000008933	Plbd1	95.46275878	0.057511658	0.150064533
ENSRNOG00000001385	Plbd2	14.71439638	0.088225482	0.376300106
ENSRNOG00000058337	Plcb2	12.33270317	-0.390526171	0.387733748
ENSRNOG00000033119	Plcb4	149.9676786	-0.066728782	0.116921108
ENSRNOG00000016361	Plcd4	24.19783798	0.169900769	0.271575556
ENSRNOG00000014276	Plce1	49.53752797	-0.016186556	0.175302588
ENSRNOG00000009955	Plch1	22.87349966	0.287095643	0.277120171
ENSRNOG00000014226	Plch2	40.74945653	0.008102541	0.201633201
ENSRNOG00000032659	Plcl1	151.043525	-0.074486622	0.149145331
ENSRNOG00000014550	Plcxd3	80.54306066	-0.181819843	0.14721159
ENSRNOG00000028156	Pld1	41.3673716	0.413768002	0.210057318
ENSRNOG00000019604	Pld2	21.026916	-0.136849521	0.33800182
ENSRNOG00000018390	Pld3	140.8567993	-0.001295156	0.119169089
ENSRNOG00000028566	Pld4	56.75073975	-0.055168368	0.190507283
ENSRNOG00000021657	Pld6	110.116947	-0.093604625	0.143848434
ENSRNOG00000005214	Plek	18.10336233	-0.270560217	0.315803597
ENSRNOG00000010098	Plek2	65.91981018	0.23129529	0.175391868
ENSRNOG00000011830	Plekha3	89.55787379	-0.064219531	0.187621376
ENSRNOG00000002907	Plekha6	146.2723084	0.356600916	0.141924109
ENSRNOG00000024602	Plekha7	28.376241	-0.036982332	0.247041117
ENSRNOG00000009971	Plekha8	40.8946676	-0.012572921	0.224585439
ENSRNOG00000018627	Plekhb1	34.36477089	-0.379274748	0.232013745
ENSRNOG00000038297	Plekhd1	74.41704042	-0.052555743	0.158616306

ENSRNOG00000027724	Plekhf1	17.16452689	-0.237086496	0.298915016
ENSRNOG00000026662	Plekhf2	75.55803644	0.201454316	0.162373897
ENSRNOG00000016011	Plekhg1	9.66759619	0.267829351	0.432952096
ENSRNOG00000030266	Plekhg2	28.93913472	-0.036495859	0.283738631
ENSRNOG00000006570	Plekhg3	187.8773863	0.256065365	0.101770944
ENSRNOG00000022694	Plekhg5	54.18564015	-0.178888375	0.694470606
ENSRNOG00000005124	Plekhh2	58.31537942	0.142068728	0.200629012
ENSRNOG00000028521	Plekhm1	12.96936339	0.31045514	0.391108682
ENSRNOG00000012163	Plekhm2	17.35572177	0.469579571	0.34205941
ENSRNOG00000020295	Plekhn1	55.89893661	0.294285898	0.194773109
ENSRNOG00000017223	Plg	61.52757406	0.045929738	0.19515456
ENSRNOG00000015932	Plgrkt	79.49227503	-0.046514185	0.162463008
ENSRNOG00000011654	Plk4	138.9283417	-0.080316252	0.131507219
ENSRNOG00000034102	Plk5	34.63961498	-0.002221203	0.247513965
ENSRNOG00000016558	Pllp	31.0841282	0.161816247	0.234819267
ENSRNOG00000007763	Plod1	62.34142916	-0.16265765	0.161843346
ENSRNOG00000030183	Plod2	11.04719897	-0.133337546	0.370877161
ENSRNOG00000001417	Plod3	93.06113003	0.215759988	0.153106375
ENSRNOG00000009980	Plpp1	49.45908322	0.351502845	0.204234579
ENSRNOG00000000177	Plpp2	10.40007805	0.005862243	0.38918556
ENSRNOG00000020424	Plpp4	80.01714165	0.123100267	0.153644174
ENSRNOG00000007575	Plppr1	68.68495957	0.001602062	0.155426327
ENSRNOG00000016878	Plppr5	97.51414751	0.142274044	0.152766156
ENSRNOG00000008048	Plscr2	15.48952714	0.30858003	0.342124329
ENSRNOG00000008151	Plscr4	28.15356458	0.019191399	0.272206035
ENSRNOG00000016488	Pltp	102.6828349	0.023967172	0.130438095
ENSRNOG00000021536	Plxdc1	47.90515351	-0.178051453	0.432154691
ENSRNOG00000000142	Plxdc2	75.15890496	0.11032195	0.141417788
ENSRNOG00000060464	Plxna3	93.3995176	0.101796428	0.173729824
ENSRNOG00000013072	Plxna4	20.08168402	0.577766198	0.360203105
ENSRNOG00000029510	Plxnb1	12.15137849	0.375698025	0.375400022
ENSRNOG00000007133	Plxnb2	155.0914545	0.013080027	0.107537769
ENSRNOG00000007970	Plxnc1	11.86680137	0.639436926	0.41396569
ENSRNOG00000025209	Plxnd1	20.54345905	-0.066633962	0.283733049
ENSRNOG00000018770	Pmaip1	60.34811486	0.159702323	0.162951474
ENSRNOG00000014505	Pmfbp1	8.725723449	-0.401281187	0.453884613
ENSRNOG00000005358	Pmm1	72.12110239	-0.170270706	0.607884516
ENSRNOG00000002615	Pmm2	19.15950559	-0.045130622	0.316672209
ENSRNOG00000003338	Pmp22	153.2942333	0.088703156	0.11317506
ENSRNOG00000026775	Pmpca	8.360673402	0.899284305	0.484574571
ENSRNOG00000012693	Pmpcb	11.64872568	-0.303303573	0.36040984
ENSRNOG00000004076	Pms1	26.96617574	-0.484702378	0.248144226
ENSRNOG00000001040	Pms2	17.60291529	0.090268028	0.334999359
ENSRNOG00000017725	Pnlip	20.16778509	0.087985275	0.297281684
ENSRNOG00000009815	Pnma2	115.3952397	-0.051858655	0.165236297
ENSRNOG00000052022	Pnma3	25.2097254	0.410033039	0.262182312
ENSRNOG00000027944	Pnma8a	29.90157715	-0.099838502	0.259021214
ENSRNOG00000004061	Pnn	88.47230938	0.064605126	0.152239437
ENSRNOG00000005524	Pno1	24.22848643	0.161211978	0.262130696
ENSRNOG00000014231	Pnoc	13.37383714	-0.399670648	0.380743251

ENSRNOG00000009982	Pnp	156.8987641	0.216040892	0.107244354
ENSRNOG00000028622	Pnpla1	22.52579858	0.213854251	0.381382741
ENSRNOG00000026157	Pnpla4	12.34193737	-0.254434741	0.352347857
ENSRNOG00000000977	Pnpla6	54.8465408	-0.058530507	0.189365349
ENSRNOG00000046493	Pnpo	20.05121839	0.28210417	0.295775542
ENSRNOG00000003600	Pnpt1	94.38250715	0.001424534	0.147596014
ENSRNOG00000024450	Poc1b	41.49038422	-0.054989796	0.205824484
ENSRNOG00000018193	Poc5	55.78368707	0.438972632	0.186516106
ENSRNOG00000012495	Podxl	132.3899918	0.0597455	0.121828643
ENSRNOG00000016255	Podxl2	31.84756065	0.463776441	0.251590335
ENSRNOG00000010104	Pofut1	44.96530181	-0.645391071	0.261905127
ENSRNOG00000001228	Pofut2	152.4555381	-0.008603806	0.108117164
ENSRNOG00000032058	Pogk	8.196850814	-0.263490671	0.429313216
ENSRNOG00000007177	Poglut3	12.4551369	0.767657385	0.445286624
ENSRNOG00000053599	Pogz	35.21286615	0.029274894	0.302425902
ENSRNOG00000013322	Pola1	9.82096475	-0.256063109	0.433151977
ENSRNOG00000020906	Pola2	13.28953045	0.162125247	0.355909289
ENSRNOG00000019150	Polb	10.84664557	-0.525716422	0.367864834
ENSRNOG00000018411	Pold3	56.57148644	-0.061798004	0.201051155
ENSRNOG00000018765	Pold4	42.67292593	0.057792736	0.246295725
ENSRNOG00000009252	Poldip2	38.59352855	0.132378371	0.238013
ENSRNOG00000004242	Pole2	45.16230965	0.118053531	0.20225096
ENSRNOG00000006102	Pole4	161.7845977	0.182694212	0.111513863
ENSRNOG00000032293	Polg	10.03416882	0.111159663	0.425398147
ENSRNOG00000019195	Polh	15.57425677	0.056862511	0.499959952
ENSRNOG00000060626	Polk	17.24300778	0.23102185	0.316376775
ENSRNOG00000016748	Poll	21.45850433	-0.204918217	0.345462219
ENSRNOG00000013647	Polm	21.0892644	0.127459796	0.331560437
ENSRNOG00000009545	Polr1a	47.2965431	0.209220176	0.190623518
ENSRNOG00000012664	Polr1e	110.1782149	-0.174595372	0.156000832
ENSRNOG00000010750	Polr1f	53.16195221	0.091566999	0.173287619
ENSRNOG00000000779	Polr1h	36.59040551	0.127025785	0.235301923
ENSRNOG00000028834	Polr2a	151.5491536	0.037630559	0.138932538
ENSRNOG00000024779	Polr2b	26.83033066	0.584787717	0.2571838
ENSRNOG00000015720	Polr2c	90.87610251	0.126353438	0.163813326
ENSRNOG00000016231	Polr2d	76.43841298	0.038096321	0.177015468
ENSRNOG00000013545	Polr2e	29.41702765	-0.468432537	0.25359227
ENSRNOG00000011214	Polr2f	61.7827395	-0.111985127	0.174048688
ENSRNOG00000019439	Polr2g	124.9798412	-0.026586961	0.136139919
ENSRNOG00000001748	Polr2h	32.95482124	0.035367179	0.276654649
ENSRNOG00000001430	Polr2j	11.81440896	-0.077811751	0.524102753
ENSRNOG00000010008	Polr3a	70.43153188	0.31107192	0.184213981
ENSRNOG00000007432	Polr3b	42.5463396	-0.11080214	0.1828987
ENSRNOG00000033560	Polr3c	8.390566033	-0.074392134	0.498878408
ENSRNOG00000010028	Polr3d	109.0681352	0.100722877	0.137802553
ENSRNOG00000016960	Polr3e	143.8878563	-0.111262417	0.115940554
ENSRNOG00000007548	Polr3f	46.55679844	-0.110439394	0.182441347
ENSRNOG00000016260	Polr3g	19.64424474	-0.263621608	0.307239807
ENSRNOG00000021209	Polr3gl	57.73633515	0.117537131	0.193647228
ENSRNOG00000004471	Polr3h	9.54962656	0.482277373	0.488614497

ENSRNOG00000001449	Pom121	74.24987493	-0.040595939	0.163149592
ENSRNOG00000023455	Pomgnt1	14.96741931	-0.238142895	0.357763361
ENSRNOG00000019492	Pomgnt2	11.24866818	-0.064920806	0.374778634
ENSRNOG00000014628	Pomk	162.4889464	0.050995389	0.111272619
ENSRNOG00000012146	Pomt2	35.30168836	-0.266217494	0.238007479
ENSRNOG00000027646	Pop4	119.9716389	-0.138621734	0.13915739
ENSRNOG00000001199	Pop5	12.9309544	-0.259205914	0.921219313
ENSRNOG00000025310	Pop7	173.6850362	-0.088852768	0.10047074
ENSRNOG00000058752	Popdc2	29.78700453	0.230212418	0.303773108
ENSRNOG00000001442	Por	18.97205966	0.581631302	0.669179787
ENSRNOG00000004819	Porcn	40.05580052	-0.28648553	0.198988914
ENSRNOG00000019986	Pot1	32.05583367	0.088201055	0.227453361
ENSRNOG00000047439	Pot1b	16.61516856	0.129488623	0.348611762
ENSRNOG00000012218	Poteg	46.45868084	0.09490461	0.206860844
ENSRNOG00000003581	Pou2f1	26.01433031	0.348245987	0.27205263
ENSRNOG00000055650	Pou2f2	87.31651482	0.271254236	0.161428398
ENSRNOG00000004595	Pou6f1	21.3199485	0.218410596	0.328387467
ENSRNOG00000021463	Ppara	12.37660589	-0.10704285	0.439355428
ENSRNOG00000000503	Ppard	15.77613795	0.920371856	0.384626413
ENSRNOG00000004473	Ppargc1a	14.43921375	-0.270615604	0.361388171
ENSRNOG00000017503	Ppargc1b	17.0801008	0.018156097	0.308125477
ENSRNOG00000002128	Ppat	94.07318503	0.182934452	0.143054612
ENSRNOG00000018711	Ppcdc	45.71701102	0.064441576	0.191594671
ENSRNOG00000053404	Ppcdc	10.6466699	0.137286573	0.419678295
ENSRNOG00000020857	Ppfia1	128.7699378	0.144303749	0.141213578
ENSRNOG00000004353	Ppfia2	48.05709731	0.12646253	0.203255394
ENSRNOG00000020731	Ppfia3	128.4520286	-0.628954876	0.447178274
ENSRNOG00000003494	Ppfia4	98.31567617	-0.13275185	0.126618038
ENSRNOG00000031709	Ppfibp1	19.97857802	-0.017737387	0.305107081
ENSRNOG00000019667	Ppfibp2	55.18549402	-0.178342221	0.180880516
ENSRNOG00000022778	Pphln1	83.27198636	-0.123102331	0.142998657
ENSRNOG00000027864	Ppial4d	85.38346013	0.160505802	0.204298733
ENSRNOG00000016781	Ppib	43.47950313	-0.330224529	0.213698568
ENSRNOG00000017416	Ppic	19.63937214	-0.180381855	0.330797842
ENSRNOG00000027408	Ppid	25.33970519	-0.155764295	0.265782314
ENSRNOG00000037230	Ppidl1	49.93518366	-0.12150126	0.204237326
ENSRNOG00000014762	Ppie	53.00107579	-0.244019247	0.200805016
ENSRNOG00000010558	Ppif	100.7390682	-0.160246606	0.144101765
ENSRNOG00000008489	Ppih	40.48746042	-0.021762952	0.206235994
ENSRNOG00000000523	Ppil1	150.6003481	0.008751172	0.13912526
ENSRNOG00000026900	Ppil2	78.36657413	-0.035052736	0.156732409
ENSRNOG00000013636	Ppil3	42.46634332	-0.165092964	0.231327998
ENSRNOG00000015552	Ppil4	122.9291534	-0.037375995	0.123653839
ENSRNOG00000014436	Ppip5k1	86.6373302	-0.099809312	0.147659645
ENSRNOG00000011613	Ppip5k2	55.97276072	0.120316461	0.203418883
ENSRNOG00000005916	Ppm1a	104.4912547	-0.079727253	0.139795225
ENSRNOG00000003329	Ppm1d	139.4136444	-0.077232373	0.134214441
ENSRNOG00000024730	Ppm1e	53.78422377	0.010038349	0.194086234
ENSRNOG00000037909	Ppm1f	146.8370847	0.173003425	0.118040004
ENSRNOG00000026905	Ppm1g	14.19178531	0.556614981	0.39357524

ENSRNOG00000004314	Ppm1h	53.82422065	-0.009157113	0.182079704
ENSRNOG00000006893	Ppm1k	32.72922088	-0.254867922	0.228085576
ENSRNOG00000018708	Ppp1ca	4.320747549	0.198987527	0.601794993
ENSRNOG00000059268	Ppp1r10	60.61240091	0.064490924	0.191483187
ENSRNOG00000051440	Ppp1r12b	47.19467177	0.239174795	0.196657061
ENSRNOG00000053828	Ppp1r12c	11.76324533	0.046293747	0.404968989
ENSRNOG00000012653	Ppp1r13b	22.95159551	0.223259117	0.310546119
ENSRNOG00000020676	Ppp1r14a	43.23517703	0.101867209	0.202797224
ENSRNOG00000021151	Ppp1r14b	18.52373447	0.286887462	0.706930722
ENSRNOG00000016368	Ppp1r14c	117.356746	0.128142401	0.127306382
ENSRNOG00000028493	Ppp1r15b	187.7542528	-0.110046825	0.135061206
ENSRNOG00000015614	Ppp1r16b	8.009671171	-0.165514444	0.427290037
ENSRNOG00000028404	Ppp1r1b	28.16309639	-0.046902999	0.234379542
ENSRNOG00000020620	Ppp1r32	20.32611889	0.367636508	0.330745752
ENSRNOG00000038480	Ppp1r36	45.51371099	0.154267905	0.219788971
ENSRNOG00000016222	Ppp1r3g	11.94862704	0.238536534	0.357759538
ENSRNOG00000016974	Ppp1r7	97.82980703	0.106665779	0.142876422
ENSRNOG00000059550	Ppp1r8	76.21241429	0.128559825	0.162163303
ENSRNOG00000008869	Ppp1r9a	94.73038665	-0.247993068	0.169560949
ENSRNOG00000005389	Ppp2ca	62.49183473	0.113817002	0.171077139
ENSRNOG00000015182	Ppp2cb	57.1965576	0.642557518	0.556722627
ENSRNOG00000010922	Ppp2r1b	82.44114426	0.115015435	0.172257697
ENSRNOG00000058202	Ppp2r2c	27.25759501	0.388208732	0.2749254
ENSRNOG00000016940	Ppp2r2d	190.9758483	0.004541133	0.100672966
ENSRNOG00000022999	Ppp2r3a	161.8518791	-0.028414061	0.106580114
ENSRNOG00000049270	Ppp2r3b	31.42320585	-0.145958444	0.243573022
ENSRNOG00000023591	Ppp2r3c	167.9065212	0.035932597	0.109534928
ENSRNOG00000000068	Ppp2r5a	46.64092535	-0.266330054	0.200266011
ENSRNOG00000021025	Ppp2r5b	22.42780922	0.376010683	0.294807777
ENSRNOG00000016849	Ppp2r5d	8.973191672	-0.288547004	0.442710253
ENSRNOG00000054782	Ppp3cb	85.69135092	-0.031494366	0.14094396
ENSRNOG00000009745	Ppp3cc	76.51709174	-0.006845266	0.148618663
ENSRNOG00000019813	Ppp4c	63.70276494	-0.247912936	0.158614679
ENSRNOG00000013733	Ppp4r1	71.96096711	-0.138566365	0.178213284
ENSRNOG00000027773	Ppp4r3a	124.6546865	0.042698048	0.134365823
ENSRNOG00000003712	Ppp4r3b	38.58172505	0.165268602	0.237489504
ENSRNOG00000015145	Ppp6c	92.11956709	0.190583301	0.141104847
ENSRNOG00000018090	Ppp6r1	12.92097572	0.137568685	0.356301894
ENSRNOG00000026425	Ppp6r2	45.15742676	0.085090569	0.221221141
ENSRNOG00000015540	Ppp6r3	137.1760009	-0.016747251	0.112495428
ENSRNOG00000018561	Pprc1	8.58395903	0.221423322	0.413794043
ENSRNOG00000012616	Ppt1	20.29255699	0.040541462	0.300402769
ENSRNOG00000000435	Ppt2	13.25262835	-0.344009648	0.366378509
ENSRNOG00000012505	Ppwd1	74.19681265	0.112237471	0.147974112
ENSRNOG00000050483	Pradc1	16.00728418	0.135802698	0.398868919
ENSRNOG00000027278	Pramef8	21.00446663	0.291705714	0.329465281
ENSRNOG00000013057	Prc1	29.1179868	-0.027584103	0.243247766
ENSRNOG00000010630	Prcp	35.93300929	-0.099882322	0.206099617
ENSRNOG00000007853	Prdm10	8.174686943	0.215646155	0.434115432
ENSRNOG00000001620	Prdm15	72.0791038	0.007104885	0.171728765

ENSRNOG00000045913	Prdm16	38.05277871	0.008195942	0.215477608
ENSRNOG00000033522	Prdm2	83.52047411	-0.02972998	0.182431086
ENSRNOG00000004962	Prdm4	23.76509596	0.405215484	0.299327641
ENSRNOG00000002004	Prdm8	17.6117434	0.024596945	0.331860395
ENSRNOG00000003520	Prdx2	161.8275195	-0.078970287	0.111920234
ENSRNOG00000003763	Prdx4	133.2344564	-0.109972589	0.135114807
ENSRNOG00000017954	Prelid3a	26.01741767	0.120534509	0.256885709
ENSRNOG00000003120	Prelp	38.47835778	-0.144974563	0.246235913
ENSRNOG00000051061	Prep	153.3995794	-0.007006844	0.111476641
ENSRNOG00000007326	Prepl	100.3468979	-0.784757933	0.185104928
ENSRNOG00000006952	Prex1	12.49264109	0.119510918	0.354131783
ENSRNOG00000005391	Prex2	46.74242758	0.101052556	0.183181158
ENSRNOG00000002385	Prg4	10.11077858	0.11830042	0.411160064
ENSRNOG00000031993	Prim1	100.1864546	0.079704132	0.220543831
ENSRNOG00000012486	Prim2	22.47860936	0.104381855	0.268635125
ENSRNOG00000008915	Prima1	23.39911068	0.237723804	0.288853531
ENSRNOG00000007706	Prkaa2	12.39019695	0.019040244	0.384372604
ENSRNOG00000018166	Prkab2	54.1296016	0.101680115	0.205575318
ENSRNOG00000004978	Prkacb	22.22763934	-0.189878126	0.27661656
ENSRNOG00000028733	Prkar1b	99.51673902	-0.171373739	0.145676256
ENSRNOG00000020284	Prkar2a	33.64854166	0.312567326	0.240015698
ENSRNOG00000003491	Prkca	25.70126758	0.1855148	0.260476593
ENSRNOG00000016346	Prkcd	50.36126959	-0.137440616	0.188352859
ENSRNOG00000009652	Prkci	169.0381784	0.059882458	0.128865369
ENSRNOG00000019057	Prkcq	34.94800482	0.01446444	0.220203349
ENSRNOG00000015480	Prkcz	117.3540244	-0.329935875	0.173158026
ENSRNOG00000004165	Prkd1	17.4198408	0.169311235	0.303201515
ENSRNOG00000016434	Prkd2	16.68418631	-0.262692582	0.415861939
ENSRNOG00000005289	Prkd3	12.78664404	0.523909445	0.41467354
ENSRNOG00000025028	Prkdc	30.12602029	0.032126134	0.25453861
ENSRNOG00000002361	Prkg2	73.76163516	-0.060302353	0.155996769
ENSRNOG00000011195	Prkra	188.3689629	-0.035359051	0.103141792
ENSRNOG00000060168	Prkx	28.75875595	-0.021596368	0.276266847
ENSRNOG00000026109	Prmt1	14.08921512	-0.288013769	0.33161437
ENSRNOG00000001297	Prmt2	30.37234033	-0.207700398	0.24157459
ENSRNOG00000014829	Prmt3	123.9303497	0.080611937	0.123659608
ENSRNOG00000017187	Prmt6	56.23048445	0.074329226	0.193230444
ENSRNOG00000000258	Prmt7	8.002956111	-0.360358341	0.444824661
ENSRNOG00000012928	Prmt9	92.14548326	-0.190448866	0.137110416
ENSRNOG00000023381	Proca1	44.79015458	-0.049954774	0.21640709
ENSRNOG00000000281	Prodh1	179.7002476	0.034970076	0.128348931
ENSRNOG00000003098	Prom1	56.28887071	0.082993524	0.171879693
ENSRNOG00000007043	Prorp	55.81256974	0.140477956	0.186103563
ENSRNOG00000004145	Prorsd1	125.7795459	0.052228414	0.121748569
ENSRNOG00000048723	Pros1	33.35323441	0.303071868	0.240176652
ENSRNOG00000010980	Proser1	58.53871416	0.130697639	0.189131411
ENSRNOG00000045553	Proser2	9.445137859	0.237408053	0.449297378
ENSRNOG00000024625	Proser3	24.62365472	-0.012233218	0.28460279
ENSRNOG00000042833	Prp2l1	31.49975975	0.669877866	0.238546163
ENSRNOG00000018396	Prpf18	136.622417	0.166674807	0.11809018

ENSRNOG00000025629	Prpf3	79.00072282	-0.050442611	0.139320971
ENSRNOG00000061039	Prpf31	10.38435607	0.653501657	0.529890051
ENSRNOG00000009451	Prpf38a	61.69989941	-0.179456341	0.16840269
ENSRNOG00000020438	Prpf38b	142.8960594	0.093296725	0.138739264
ENSRNOG00000004864	Prpf40a	165.0368364	0.092884342	0.09960329
ENSRNOG00000052539	Prpf40b	84.50793922	0.422132014	0.16435695
ENSRNOG00000016705	Prpf4b	175.2189183	-0.188952015	0.102856585
ENSRNOG00000051498	Prpf6	33.81935741	0.368786393	0.349812605
ENSRNOG00000003495	Prpf8	11.9668902	0.181335997	0.356769887
ENSRNOG00000052880	Prph	22.6849668	0.238831156	0.27464107
ENSRNOG00000004160	Prps2	141.1617082	0.164992594	0.13023891
ENSRNOG00000010083	Prpsap1	15.38667625	0.087099582	0.336940385
ENSRNOG00000059408	Prr12	22.53417415	0.837851347	0.30761044
ENSRNOG00000042094	Prr13	166.4556837	-0.023647813	0.107007938
ENSRNOG00000048891	Prr14l	89.60266277	0.196677253	0.149954482
ENSRNOG00000009564	Prr15	49.87751408	-0.296730287	0.256901022
ENSRNOG00000025806	Prr3	10.78737029	0.323214365	0.427909968
ENSRNOG00000028892	Prr36	30.99535983	0.298234691	0.260515563
ENSRNOG00000004666	Prr5l	9.177623592	0.276195395	0.414862864
ENSRNOG00000016433	Prrc1	8.393901315	0.189869334	0.428376421
ENSRNOG00000000852	Prrc2a	15.52075264	0.804185709	0.343698061
ENSRNOG00000010217	Prrc2b	41.3432928	0.168203042	0.229334066
ENSRNOG00000026044	Prrg1	36.36532956	0.180743786	0.223881682
ENSRNOG00000009863	Prrt3	34.83001447	0.130491852	0.324418035
ENSRNOG00000003720	Prrx1	12.72942042	-0.390495091	0.353459468
ENSRNOG00000015353	Prss12	90.86014801	-0.167387469	0.166008917
ENSRNOG00000039698	Prss22	10.39707763	-0.051296359	0.397989517
ENSRNOG00000017307	Prss23	66.44114411	-0.106341325	0.175643146
ENSRNOG00000025184	Prss35	12.00951805	-0.154993865	0.434186807
ENSRNOG00000037175	Prss50	34.9618913	0.161339834	0.230471919
ENSRNOG00000047637	Prss51	10.94093911	0.147139633	0.379539407
ENSRNOG00000026040	Prss53	17.21277567	0.041670651	0.372985685
ENSRNOG00000024874	Prtfdc1	124.4565955	0.448421788	0.134603198
ENSRNOG00000021120	Prune1	14.81358627	-0.855613553	0.945341848
ENSRNOG00000015088	Prune2	35.53483335	-0.230554594	0.217772467
ENSRNOG00000000571	Psap	71.85198055	-0.00049732	0.165437489
ENSRNOG00000019177	Psd2	31.97594316	0.173466332	0.23134937
ENSRNOG00000013884	Psd3	14.11703063	0.256835408	0.340780449
ENSRNOG00000009110	Psen1	121.6736068	-0.024895537	0.13577495
ENSRNOG00000002879	Psen2	122.6695588	0.084171418	0.129443433
ENSRNOG00000011745	Psma1	110.0953671	-0.240663588	0.139511346
ENSRNOG00000049920	Psma2	74.97280294	-0.03060781	0.166650038
ENSRNOG00000056853	Psma7	142.2140163	-0.00684086	0.135893562
ENSRNOG00000020979	Psmb4	83.34294615	-0.260762169	0.20697077
ENSRNOG00000011732	Psmb7	111.5861486	-0.0617972	0.130382891
ENSRNOG00000000459	Psmb9	20.25219467	0.044912549	0.315486722
ENSRNOG00000011414	Psmc3	59.63236584	0.00830807	0.2290723
ENSRNOG00000007203	Psmc6	79.06013688	0.132177797	0.1679911
ENSRNOG00000014109	Psmd13	14.0801816	0.385295612	0.342672603
ENSRNOG00000061129	Psmd14	68.88714564	-0.281425955	0.162742491

ENSRNOG00000018809	Psmd5	105.1888285	-0.014195907	0.149298994
ENSRNOG00000037580	Psmd8	18.33452879	0.704981173	0.343089174
ENSRNOG00000061359	Psme1-ps1	11.27827879	-0.007276528	0.606719903
ENSRNOG00000019246	Psme2	11.9618301	-0.164981312	0.387031594
ENSRNOG00000053448	Psme3	10.25634173	-0.259586719	0.400878858
ENSRNOG00000017841	Psme3ip1	96.19376774	0.108392202	0.152296932
ENSRNOG00000060340	Psme4	103.7373862	0.065022767	0.129962587
ENSRNOG00000001643	Psmg1	123.1820618	0.089024643	0.120945742
ENSRNOG00000017729	Psmg2	109.1027283	-0.198104646	0.144136081
ENSRNOG00000039265	Psmg4	18.01680395	-0.166052323	0.298898778
ENSRNOG00000020782	Pspc1	115.6290132	-0.087932507	0.125808548
ENSRNOG00000020605	Pstk	114.3090759	0.081032907	0.134528244
ENSRNOG00000016413	Pstpip1	23.75954637	0.399816287	0.29742966
ENSRNOG00000016987	Pstpip2	31.27996871	-0.232330232	0.262184473
ENSRNOG00000013231	Ptafr	28.73653961	0.118799711	0.25128396
ENSRNOG00000014891	Ptar1	10.07370054	-0.571107588	0.422760045
ENSRNOG00000010827	Ptbp2	26.46030808	0.256875922	0.335776785
ENSRNOG00000016334	Ptbp3	93.73129145	-0.062647836	0.146340047
ENSRNOG00000028403	Ptcd2	70.98228381	-0.097517333	0.171972746
ENSRNOG00000009484	Ptcd3	11.01948086	0.928825555	0.426522384
ENSRNOG00000019354	Ptch1	132.5054611	0.236836187	0.135483324
ENSRNOG00000057616	Ptch2	11.14455047	0.411480789	0.37186908
ENSRNOG00000052289	Ptdss1	184.2086648	0.015854157	0.106675218
ENSRNOG00000036631	Ptgdr2	52.91396374	0.038864995	0.230041265
ENSRNOG00000010325	Ptger3	18.57355618	0.569414819	0.323126404
ENSRNOG00000013240	Ptger4	10.06650253	0.551021375	0.433542575
ENSRNOG00000038166	Ptgr2	18.88332787	0.44816191	0.302403268
ENSRNOG00000007415	Ptgs1	33.53012363	0.268198978	0.259047056
ENSRNOG00000002525	Ptgs2	148.3992026	-0.297973661	0.184752292
ENSRNOG00000020948	Pth1r	58.87383399	0.276027989	0.210760785
ENSRNOG00000007916	Ptk2	93.69697205	0.111763347	0.147640394
ENSRNOG00000027839	Ptk2b	77.00009993	0.597187613	0.259445789
ENSRNOG00000020358	Ptov1	136.9413346	0.433976653	0.482270583
ENSRNOG00000009723	Ptpmt1	21.68849661	-0.076829699	0.417876134
ENSRNOG00000010574	Ptpn1	21.832078	0.300492251	0.280767705
ENSRNOG00000013135	Ptpn12	121.9692107	0.074208359	0.118499407
ENSRNOG00000002061	Ptpn13	23.75956798	0.026513292	0.292636054
ENSRNOG00000013415	Ptpn18	16.87871134	0.443894188	0.359492379
ENSRNOG00000017453	Ptpn2	85.25582689	0.034694489	0.175209419
ENSRNOG00000020862	Ptpn23	10.31885862	0.152729197	0.476239336
ENSRNOG00000011425	Ptpn3	121.9123221	0.125928541	0.12898456
ENSRNOG00000002625	Ptpn4	160.1918351	0.022537062	0.104068422
ENSRNOG00000014294	Ptpn6	76.06833359	-0.096428818	0.203273639
ENSRNOG00000005807	Ptpn7	20.51137257	0.048330917	0.276597291
ENSRNOG00000017600	Ptpn9	168.5838271	0.148289714	0.124092457
ENSRNOG00000055293	Ptprb	48.11760175	0.17439196	0.220000002
ENSRNOG00000000655	Ptprc	11.60614826	0.21283309	0.408822066
ENSRNOG00000015717	Ptpre	121.4696825	-0.005527441	0.145508595
ENSRNOG00000009419	Ptprg	19.86461034	0.913981933	0.349143636
ENSRNOG00000011700	Ptprm	123.8535596	-0.006840345	0.133629139

ENSRNOG00000019587	Ptprn	105.8080005	-0.158072345	0.142333049
ENSRNOG00000006231	Ptpro	67.08396977	0.063454186	0.162155418
ENSRNOG00000047247	Ptprs	84.02562134	0.122190738	0.159895764
ENSRNOG00000032656	Ptprt	12.87767099	0.412982729	0.37931866
ENSRNOG00000013515	Ptpru	144.0374892	0.082530415	0.129343407
ENSRNOG00000004288	Ptrh2	25.9421229	-0.056260079	0.25262002
ENSRNOG00000004059	Ptrhd1	52.25928687	0.247469604	0.205286009
ENSRNOG00000009250	Pts	60.21942759	-0.03684267	0.183408643
ENSRNOG00000009960	Puf60	170.6123047	0.166029664	0.150166782
ENSRNOG00000012574	Pum3	73.34978677	-0.060114849	0.169858331
ENSRNOG00000037500	Pus1	9.249198505	1.166531464	0.54641841
ENSRNOG00000054151	Pus10	52.03853526	0.315062985	0.216258669
ENSRNOG00000012994	Pus3	28.38757193	-0.52016237	0.25474049
ENSRNOG00000010572	Pus7	29.34349878	-0.00868419	0.237601241
ENSRNOG00000022570	Pus7l	18.85012826	0.33969583	0.352333584
ENSRNOG00000019202	Pvr	12.2972926	-0.27362932	0.393044154
ENSRNOG00000005350	Pwp1	111.2694505	-0.035247994	0.135504038
ENSRNOG00000003936	Pwwp2a	73.00843097	0.091397649	0.156949736
ENSRNOG00000036649	Pwwp2b	62.14369603	0.248000051	0.175289834
ENSRNOG00000024549	Pwwp3a	139.9885243	0.070682882	0.12168416
ENSRNOG00000017093	Pxdc1	34.7974298	-0.338723954	0.269149696
ENSRNOG00000008024	Pxk	20.63429656	0.277488542	0.60728033
ENSRNOG00000037446	Pxmp2	31.77201881	0.321728072	0.245998148
ENSRNOG00000016975	Pxmp4	64.92348784	-0.366799909	0.258688437
ENSRNOG00000012480	Pxylp1	69.25790888	0.028863286	0.171050541
ENSRNOG00000019675	Pycard	32.21866626	-0.034168513	0.277773386
ENSRNOG00000036682	Pycr1	48.61852446	0.405583422	0.210236254
ENSRNOG00000003267	Pycr2	94.3061351	-0.045438705	0.148116943
ENSRNOG00000021638	Pycr3	19.8586875	-0.715717812	0.352185512
ENSRNOG00000007583	Pygb	10.64864326	0.476542888	0.388536815
ENSRNOG00000006388	Pygl	27.5495042	-0.376193214	0.341222675
ENSRNOG00000021090	Pygm	20.30281146	-0.092747821	0.307566873
ENSRNOG00000058130	Pym1	73.51993204	-0.202825028	0.171800622
ENSRNOG00000015807	Pyroxd2	52.01092851	-0.105507387	0.179219319
ENSRNOG00000006709	Pzp	8.300259303	0.952720196	0.528129204
ENSRNOG00000060912	Qars1	26.58814251	-0.805956114	0.316844392
ENSRNOG00000005705	Qpct	48.42107181	-0.061584796	0.228024918
ENSRNOG00000015413	Qpctl	20.25239204	0.249799342	0.302096066
ENSRNOG00000016980	Qprt	11.95641618	0.210593526	0.412867252
ENSRNOG00000025071	Qrich1	49.56806982	0.0373721	0.200709117
ENSRNOG00000026049	Qrsl1	51.6827104	-0.160822	0.188661652
ENSRNOG00000037514	Qser1	40.48589925	0.51608029	0.249856159
ENSRNOG00000003649	Qsox1	93.12909889	-0.043469133	0.138175321
ENSRNOG00000007158	Qtrt1	8.308731037	-0.260734673	0.449863989
ENSRNOG00000060729	Qtrt2	21.88864196	-0.183085984	0.270649331
ENSRNOG00000016777	R3hcc1	50.47075323	0.098022395	0.204372086
ENSRNOG00000007801	R3hdm2	65.72510896	0.263682205	0.198138031
ENSRNOG00000032152	Rab11fip3	65.12277436	0.132459415	0.192399595
ENSRNOG00000014135	Rab11fip4	61.7881459	0.218081199	0.21719329
ENSRNOG00000016733	Rab13	59.89123825	-0.34416652	0.15964214

ENSRNOG00000018901	Rab14	160.3808632	-0.215170012	0.155594327
ENSRNOG00000007364	Rab15	88.65146393	0.181863237	0.193629266
ENSRNOG00000050510	Rab1b	28.08834762	0.130796693	0.267673008
ENSRNOG00000023991	Rab20	133.4932386	0.230893679	0.13995656
ENSRNOG00000003923	Rab21	183.6487608	0.015599444	0.112122124
ENSRNOG00000005762	Rab22a	164.4777959	-0.006212427	0.116366936
ENSRNOG00000012629	Rab23	40.98133778	-0.002149866	0.339311549
ENSRNOG00000016539	Rab24	81.20173426	0.058944205	0.152274719
ENSRNOG00000042086	Rab26	10.49716933	0.099118161	0.434129233
ENSRNOG00000052499	Rab27a	35.62148743	0.030690531	0.207038615
ENSRNOG00000049641	Rab29	190.661496	-0.001131424	0.097110904
ENSRNOG00000012488	Rab2b	27.07503698	0.389591747	0.29853146
ENSRNOG00000010224	Rab30	33.05292659	0.056551054	0.247400556
ENSRNOG00000014258	Rab32	21.15969681	-0.949966738	0.605441803
ENSRNOG00000006698	Rab33a	138.415576	0.174684371	0.123181289
ENSRNOG00000022014	Rab35	29.21619906	-0.962498334	0.79763638
ENSRNOG00000001311	Rab36	169.6348327	-0.033474472	0.16133266
ENSRNOG00000019433	Rab3a	16.27210866	0.4078986	0.33239019
ENSRNOG00000011623	Rab3c	53.40785792	-0.201461853	0.186208298
ENSRNOG00000011582	Rab3d	122.3468076	0.060479996	0.123994638
ENSRNOG00000002353	Rab3gap2	177.5568337	-0.122056587	0.12238893
ENSRNOG00000005362	Rab3ip	88.23398186	0.130612627	0.146079556
ENSRNOG00000036661	Rab40b	12.18182592	0.439352348	0.371680187
ENSRNOG00000020067	Rab40c	8.462064884	0.187333715	0.436382169
ENSRNOG00000001500	Rab4b	48.3968424	-0.122369545	0.190705626
ENSRNOG00000006130	Rab5b	37.55675303	0.309926518	0.270126741
ENSRNOG00000018568	Rab5c	35.24390016	-0.748823829	0.468495877
ENSRNOG00000020317	Rab5if	19.08760459	-0.06014477	0.319368559
ENSRNOG00000018176	Rab6a	93.33267208	0.129948667	0.18336835
ENSRNOG00000039754	Rab7b	32.02614908	0.081317307	0.251756436
ENSRNOG00000018009	Rab8b	129.0962008	0.140474416	0.135804138
ENSRNOG00000047960	Rab9b	142.4983347	-0.257171818	0.113958656
ENSRNOG00000018462	Rabep2	9.939075537	-0.013414155	0.4408154
ENSRNOG00000018591	Rabepk	32.93626669	-0.081958919	0.225874609
ENSRNOG00000002736	Rabgap1l	27.87430901	0.091640378	0.2361162
ENSRNOG00000030483	Rabggta	8.504468975	-0.536086892	0.467052989
ENSRNOG00000004424	Rabif	27.70216182	-0.093211496	0.266532204
ENSRNOG00000013947	Rabl2	112.6384038	0.176716425	0.134367642
ENSRNOG00000026085	Rabl3	10.18898603	-0.485188058	0.401061263
ENSRNOG00000001068	Rac1	21.7560515	0.002707977	0.269086685
ENSRNOG00000007350	Rac2	72.80538205	0.105538028	0.251673018
ENSRNOG00000049033	Racgap1	12.01060335	0.086951436	0.365619092
ENSRNOG00000018063	Rad1	32.39672657	-0.214285198	0.243828728
ENSRNOG00000018353	Rad17	14.74194935	0.53016183	0.409554818
ENSRNOG00000005907	Rad18	35.42394849	-0.060520943	0.232389597
ENSRNOG00000003026	Rad23a	32.23954878	0.028077763	0.212194929
ENSRNOG00000033065	Rad50	67.80697788	-0.089580891	0.163139403
ENSRNOG00000049489	Rad54l2	7.96830929	0.189022532	0.466992936
ENSRNOG00000021480	Rad9b	84.98233356	-0.021947903	0.154634624
ENSRNOG00000024456	Radil	64.48694207	0.111363054	0.184291029

ENSRNOG00000010153	Raf1	10.09655082	0.195016638	0.4295556
ENSRNOG00000060615	Rai1	12.5060203	-0.325019215	0.37599287
ENSRNOG00000028872	Rai14	24.45624277	0.131155197	0.314922447
ENSRNOG00000006670	Rai2	37.35751367	0.069049739	0.294097348
ENSRNOG00000002440	Ralb	158.5341612	0.091291422	0.125064727
ENSRNOG00000046256	Ralgapa1	31.04884342	0.092621247	0.261017079
ENSRNOG00000036964	Ralgapa2	10.46773584	0.155356114	0.465032527
ENSRNOG00000014836	Ralgapb	39.76322367	-0.118721679	0.210120452
ENSRNOG00000010219	Ralgds	15.76687436	-0.330063483	0.30650931
ENSRNOG00000016910	Ralgps1	119.9086988	0.043829755	0.141797082
ENSRNOG00000004736	Ralgps2	31.11850766	0.05369935	0.239055694
ENSRNOG00000017405	Raly	156.6987392	-0.0488114	0.130168746
ENSRNOG00000019426	Ramac	121.9325056	-0.193098636	0.122167133
ENSRNOG00000001884	Ranbp1	14.54167231	-0.115528784	0.32725805
ENSRNOG00000018000	Ranbp10	141.6671598	-0.118709214	0.124162414
ENSRNOG00000005042	Ranbp17	9.488394015	0.354601691	0.492747527
ENSRNOG00000000796	Ranbp2	34.47895042	0.253754262	0.253425775
ENSRNOG00000049785	Ranbp3	99.36857988	-0.040578652	0.146009658
ENSRNOG00000017951	Ranbp9	52.04601147	-0.052665089	0.192318294
ENSRNOG00000051564	Rap2a	128.9783758	-0.295075991	0.158004866
ENSRNOG00000002529	Rap2c	149.0765599	-0.076667098	0.11311236
ENSRNOG00000014316	Rapgef1	31.74265944	0.380889751	0.284395294
ENSRNOG00000059961	Rapgef3	110.4643962	0.247636817	0.140533786
ENSRNOG00000001516	Rapgef4	24.46285501	0.033953938	0.281530967
ENSRNOG00000009995	Rapgef6	57.33098521	0.232351634	0.183774687
ENSRNOG00000014722	Raph1	36.87453123	0.141267778	0.222402264
ENSRNOG00000009972	Rara	93.64579912	0.035168152	0.170206371
ENSRNOG00000037853	Rarres1	23.32003673	-0.033370391	0.270750914
ENSRNOG00000024705	Rarres2	14.54497667	-0.354163273	0.319676386
ENSRNOG00000029185	Rasa1	65.66248863	0.104746913	0.160481958
ENSRNOG00000011909	Rasa2	124.0561381	0.136748541	0.149638388
ENSRNOG00000017671	Rasa3	92.11175779	0.037008328	0.145889797
ENSRNOG00000004917	Rasal2	145.864066	0.198894132	0.113583654
ENSRNOG00000031671	Rasgef1a	54.25845691	0.056067403	0.191285887
ENSRNOG00000002345	Rasgef1b	65.92802325	-0.093608781	0.236962938
ENSRNOG00000003005	Rasgef1c	147.966067	0.249973508	0.143152251
ENSRNOG00000005404	Rasgrp1	33.69116786	-0.167170038	0.219490847
ENSRNOG00000021098	Rasgrp2	33.89935789	0.367373588	0.255158979
ENSRNOG00000032703	Rasgrp3	50.2015764	0.573386875	0.193410451
ENSRNOG00000033744	Rasgrp4	10.41849283	-0.824511681	0.443187991
ENSRNOG00000008951	Rasl10a	53.81730028	-0.621642321	0.292432932
ENSRNOG00000010302	Rasl10b	29.7594266	-0.069890932	0.250087918
ENSRNOG00000021548	Rassf1	9.583604082	-0.199058513	0.396313322
ENSRNOG00000021261	Rassf2	26.18675716	0.372618305	0.274932219
ENSRNOG00000005388	Rassf3	57.52180773	0.223491847	0.178166985
ENSRNOG00000013526	Rassf4	26.12967718	-0.238072572	0.233806724
ENSRNOG00000005342	Rassf5	15.61009873	0.292350037	0.33617375
ENSRNOG00000015986	Rassf8	46.63550779	0.05200032	0.186989382
ENSRNOG00000020710	Raver1	11.55121729	-0.002750085	0.429656682
ENSRNOG00000016029	Rb1	13.89455196	0.381539952	0.361897448

ENSRNOG00000001099	Rbak	36.66127903	-0.248794743	0.21753786
ENSRNOG00000021289	Rbbp5	12.47671189	0.476505995	0.404708738
ENSRNOG00000005157	Rbbp7	60.78852579	-0.037766124	0.18638082
ENSRNOG00000012899	Rbbp8	73.05580577	-0.186683581	0.146104671
ENSRNOG00000057245	Rbbp8nl	9.320729169	0.128815377	0.473405916
ENSRNOG00000007972	Rbbp9	11.60171366	0.236988658	0.394023831
ENSRNOG00000055647	Rbfa	181.1286526	-0.148549476	0.104830839
ENSRNOG00000004688	Rbfox2	115.0792523	-0.17607578	0.122678665
ENSRNOG00000038902	Rbis	40.21851407	0.191523081	0.194402277
ENSRNOG00000004710	Rbks	70.31024933	-0.276173027	0.182187901
ENSRNOG00000029147	Rbm11	86.4002844	-0.109416173	0.187665
ENSRNOG00000045568	Rbm14	100.5089176	0.131231112	0.140469876
ENSRNOG00000047499	Rbm15	82.60690662	-0.451986801	0.148921035
ENSRNOG00000001397	Rbm19	26.93814558	-0.165530397	0.228018254
ENSRNOG00000046547	Rbm24	98.88397303	-0.272402518	0.187614491
ENSRNOG00000002874	Rbm25	38.28707818	0.369043252	0.251296446
ENSRNOG00000027250	Rbm27	70.19392875	-0.131346439	0.167427262
ENSRNOG00000005468	Rbm28	116.7843509	0.099215417	0.120434748
ENSRNOG00000006718	Rbm33	19.51614191	0.671446597	0.341332193
ENSRNOG00000020004	Rbm34	9.49806073	-0.118437733	0.449366381
ENSRNOG00000006420	Rbm38	26.14278549	-0.338298396	0.259045528
ENSRNOG00000057622	Rbm41	16.48947302	-0.379415476	0.334452152
ENSRNOG00000024278	Rbm42	178.6907942	-0.021398957	0.107189103
ENSRNOG00000010595	Rbm45	158.1828177	-0.115158994	0.132998882
ENSRNOG00000018333	Rbm6	68.36142036	0.171396866	0.176010646
ENSRNOG00000005710	Rbm7	145.593304	0.050819176	0.126822615
ENSRNOG00000021215	Rbm8a	86.0895278	0.014199405	0.15597232
ENSRNOG00000008482	Rbms1	103.750272	0.346480079	0.16973594
ENSRNOG00000003076	Rbms2	130.7808058	0.00939197	0.111895079
ENSRNOG00000025881	Rbms3	9.999844875	-0.027184498	0.472738111
ENSRNOG00000007371	Rbmx2	16.68330409	0.220611252	0.319437983
ENSRNOG00000059347	Rbmxl1b	78.25786755	0.05694881	0.210675173
ENSRNOG00000013794	Rbp1	58.74758993	-0.358070697	0.193707453
ENSRNOG00000015518	Rbp4	13.36974937	-0.079767427	0.34799819
ENSRNOG00000046327	Rbpj	24.00576747	7.83133E-05	0.264824722
ENSRNOG00000013328	Rbpms	18.31609026	0.254895536	0.332239464
ENSRNOG00000015896	Rbpms2	46.57366114	0.053976542	0.243704523
ENSRNOG00000011391	Rbsn	26.76437269	0.149385926	0.32660296
ENSRNOG00000002750	Rc3h1	17.76053912	-0.25600602	0.336009536
ENSRNOG00000009196	Rc3h2	78.3702902	0.031882131	0.175069704
ENSRNOG00000021712	Rcbtb1	102.5805571	0.099225832	0.142252279
ENSRNOG00000015054	Rcbtb2	38.21529527	0.023927263	0.220791876
ENSRNOG00000050106	Rcc1	35.1655109	-0.157344311	0.234455402
ENSRNOG00000013452	Rcn1	11.85891579	-0.207953551	0.500239226
ENSRNOG00000049783	Rcor1	72.50658129	0.081746291	0.160086624
ENSRNOG00000046445	Rcor3	47.0935779	-0.019417699	0.185196411
ENSRNOG00000003283	Rcsd1	23.89165886	0.050680276	0.26915446
ENSRNOG00000006681	Rdh10	48.0399309	-0.095243536	0.213182828
ENSRNOG00000054770	Rdh11	11.54461204	-0.151442297	0.378221363
ENSRNOG00000056553	Rdh12	15.73327225	0.501446192	0.376643089

ENSRNOG00000027919	Rdh13	39.49655916	-0.023925458	0.208304587
ENSRNOG00000012237	Rdx	108.7484453	-0.340638027	0.184087301
ENSRNOG00000009332	Rec114	8.64770118	-0.224577835	0.470890328
ENSRNOG00000012602	Recql	22.98961448	-0.02417941	0.311949234
ENSRNOG00000005107	Recql5	10.34605533	0.182461859	0.406936948
ENSRNOG00000008481	Reep1	49.24277593	-0.300982376	0.175745421
ENSRNOG00000000645	Reep3	123.6673548	0.138817712	0.129170265
ENSRNOG00000033262	Reep6	131.223501	0.075824952	0.116120643
ENSRNOG00000033235	Relb	20.18114834	-0.505426312	0.327753946
ENSRNOG00000002192	Rell1	37.16001079	-0.268415243	0.249401529
ENSRNOG00000021441	Reln	55.32603426	0.301413944	0.211420866
ENSRNOG00000007567	Rem1	16.855967	0.08608861	0.347206611
ENSRNOG00000011646	Rem2	35.03031349	-0.091948754	0.248007444
ENSRNOG00000054765	Renbp	10.14371561	-0.398724058	0.372747906
ENSRNOG00000008239	Repin1	97.90929016	-0.858457961	0.310305957
ENSRNOG00000059224	Reps1	78.4029012	0.065740502	0.145976804
ENSRNOG00000014270	Rer1	89.31558247	-0.246716712	0.16008428
ENSRNOG00000027592	Rerg	46.84210184	-0.138508877	0.21367177
ENSRNOG00000036913	Resf1	33.44887416	-0.36885253	0.215800789
ENSRNOG00000014090	Retsat	20.56220813	0.333418599	0.348759684
ENSRNOG00000018623	Rev1	108.6367366	-0.037922367	0.13385527
ENSRNOG00000000593	Rev3l	95.05117971	-0.130521856	0.136026202
ENSRNOG00000017916	Rexo1	43.19684396	-0.114234549	0.218992996
ENSRNOG00000027867	Rexo4	12.33250906	0.164491729	0.38993668
ENSRNOG00000014513	Rexo5	65.65605513	-0.087034406	0.183236862
ENSRNOG00000002855	Rfc1	135.0180515	-0.101477539	0.146640525
ENSRNOG00000001457	Rfc2	32.8673127	0.016766595	0.259707636
ENSRNOG00000001088	Rfc3	34.02924809	-0.291485945	0.244035169
ENSRNOG00000006674	Rflnb	30.39334768	0.22443442	0.275518488
ENSRNOG00000021805	Rft1	36.36799643	0.120109874	0.207123425
ENSRNOG00000011321	Rftn1	49.29048793	0.150696509	0.232398597
ENSRNOG00000015594	Rftn2	30.57778048	0.514624097	0.24759607
ENSRNOG00000051461	Rfwd3	26.37803852	0.08240689	0.240049418
ENSRNOG00000006049	Rfx1	41.85662013	0.14290124	0.215100458
ENSRNOG00000014486	Rfx3	12.66552816	-0.268924938	0.440903938
ENSRNOG00000051536	Rfx4	8.658806126	-0.209379931	0.46412895
ENSRNOG00000021012	Rfx5	106.4566327	0.040633077	0.142283551
ENSRNOG00000060367	Rfx7	69.0854892	0.186921835	0.158590608
ENSRNOG00000025034	RGD1304567	23.84148961	0.368423575	0.274222237
ENSRNOG00000000040	RGD1304622	12.36057638	-0.188842253	0.404745887
ENSRNOG00000006986	RGD1304624	26.49148538	0.310804898	0.270667441
ENSRNOG00000017431	RGD1304884	15.83643853	-0.053467499	0.334417655
ENSRNOG00000020597	RGD1305014	17.12487671	-0.165491928	0.310857943
ENSRNOG00000054669	RGD1305110	50.86424246	0.30663468	0.174948906
ENSRNOG00000007968	RGD1305350	158.0706676	0.01664488	0.10811089
ENSRNOG00000018689	RGD1305464	19.45490858	-0.463726796	0.279610285
ENSRNOG00000013729	RGD1306271	76.14490663	0.045621011	0.176262757
ENSRNOG00000022745	RGD1306502	154.407614	-0.115079857	0.115742571
ENSRNOG00000017380	RGD1306746	93.80128721	0.235149425	0.137198066
ENSRNOG00000018141	RGD1307443	14.00535847	-0.062863391	0.370376663

ENSRNOG00000015718	RGD1307461	26.91150223	0.280184849	0.270738918
ENSRNOG00000018408	RGD1307554	134.0464276	0.024161907	0.124173026
ENSRNOG00000023230	RGD1307621	14.64670197	-0.050547079	0.368107787
ENSRNOG00000011887	RGD1307929	14.63585903	0.090748754	0.341138839
ENSRNOG00000015523	RGD1308147	165.2758682	-0.24625097	0.106406776
ENSRNOG00000047406	RGD1308544	24.59277664	0.04641278	0.272999458
ENSRNOG00000017215	RGD1308601	96.68414043	0.39109376	0.141423505
ENSRNOG00000017090	RGD1308706	184.7095829	-0.036793768	0.122384379
ENSRNOG00000004112	RGD1308775	24.99184022	-0.035018463	0.258263612
ENSRNOG00000048412	RGD1309028	14.52589089	-0.07445663	0.348895521
ENSRNOG00000020307	RGD1309139	14.18250313	-0.43286297	0.400270175
ENSRNOG00000024623	RGD1309170	20.54713297	0.698316554	0.652329029
ENSRNOG00000010887	RGD1309534	9.406725801	0.412408185	0.471192633
ENSRNOG00000014798	RGD1309540	24.68422559	0.028395501	0.258909713
ENSRNOG00000017646	RGD1309651	20.33135397	0.177882977	0.286844788
ENSRNOG00000047782	RGD1309808	13.73178967	0.524917394	0.422745345
ENSRNOG00000009434	RGD1310507	10.0246261	0.527959515	0.475689764
ENSRNOG00000016447	RGD1310553	73.07887613	-0.285277	0.161782123
ENSRNOG00000002322	RGD1310587	48.39983545	-0.491090021	0.182302915
ENSRNOG00000006370	RGD1310852	27.68351863	0.116056973	0.252964057
ENSRNOG00000051418	RGD1310935	128.3872559	-0.219817973	0.134017465
ENSRNOG00000016780	RGD1310951	33.5822688	0.372771167	0.232549081
ENSRNOG00000018486	RGD1311084	116.8281457	-0.092820192	0.117853733
ENSRNOG00000055036	RGD1311164	46.68909331	0.087572664	0.193454671
ENSRNOG00000019232	RGD1311345	34.4145311	-0.303118023	0.207091676
ENSRNOG00000020199	RGD1311517	30.0354271	0.337266475	0.26744211
ENSRNOG00000021245	RGD1311739	58.87638099	-0.115701386	0.182590332
ENSRNOG00000005960	RGD1311744	43.39828716	0.435333127	0.528766444
ENSRNOG00000025245	RGD1311946	96.52865999	0.116780849	0.125243059
ENSRNOG00000027161	RGD1359158	45.06548704	-0.275841157	0.19140519
ENSRNOG00000038330	RGD1359508	16.15626321	0.13105275	0.357918107
ENSRNOG00000026462	RGD1559962	15.65028947	-0.064484426	0.427132289
ENSRNOG00000014857	RGD1560065	32.62756384	0.173080598	0.243974048
ENSRNOG00000022155	RGD1560289	10.25339703	0.551537186	0.468690965
ENSRNOG00000013622	RGD1560813	116.1836794	-0.179075828	0.162749974
ENSRNOG00000045639	RGD1560883	17.44069921	0.639496112	0.318909047
ENSRNOG00000034128	RGD1560917	25.20536319	0.18685688	0.269329043
ENSRNOG00000051868	RGD1561277	54.62007798	0.046186702	0.199382579
ENSRNOG00000028997	RGD1561671	44.3086186	-0.031905211	0.277666954
ENSRNOG00000022723	RGD1562029	27.68186676	-0.451301582	0.267194077
ENSRNOG00000019169	RGD1562218	98.86825658	0.139119359	0.129104514
ENSRNOG00000028670	RGD1562726	12.97336188	0.343519339	0.400276288
ENSRNOG00000021609	RGD1563072	179.8338241	-0.212640173	0.128375445
ENSRNOG00000014424	RGD1563354	22.99259023	-0.214659601	0.250621393
ENSRNOG00000031706	RGD1563601	16.55829518	0.086310883	0.312087656
ENSRNOG00000029979	RGD1563815	18.13386841	-0.224722005	0.357296939
ENSRNOG00000029958	RGD1563861	62.00387026	-0.311880067	0.201781707
ENSRNOG00000005375	RGD1564053	72.10014033	0.121732455	0.178412912
ENSRNOG00000028386	RGD1564162	28.89241312	-0.206464348	0.242150761
ENSRNOG00000059641	RGD1564480	26.69441864	-0.060847428	0.269405845

ENSRNOG00000027455	RGD1564804	72.33133906	-0.08563542	0.166855682
ENSRNOG00000021324	RGD1565059	18.84401861	0.200837453	0.350589481
ENSRNOG00000025212	RGD1565131	70.23978188	0.330648992	0.154719021
ENSRNOG00000021796	RGD1565166	20.754294	0.647558432	0.296804618
ENSRNOG00000013317	RGD1565222	11.7688935	0.422259216	0.365517874
ENSRNOG00000026919	RGD1565367	12.31575019	0.419589956	0.374966264
ENSRNOG00000023187	RGD1565685	59.43058206	0.16830205	0.19831026
ENSRNOG00000043374	RGD1566099	31.47509382	-0.332835277	0.23350104
ENSRNOG00000002323	RGD1566265	37.33663174	0.102377652	0.266501204
ENSRNOG00000006010	RGD1566368	16.92786466	0.155352479	0.317867801
ENSRNOG00000028651	RGD1566386	33.57167739	-0.175724973	0.224834691
ENSRNOG00000014668	RGD621098	50.18420295	-0.362771682	0.211116193
ENSRNOG00000002347	Rgl1	152.1367027	-0.051742737	0.109453198
ENSRNOG00000000474	Rgl2	63.78893858	-0.474112809	0.193366079
ENSRNOG00000013027	Rgl3	27.34729781	0.514538281	0.276006655
ENSRNOG00000003888	Rgs13	14.86779384	0.001352646	0.315209069
ENSRNOG00000027024	Rgs16	63.04176604	0.228766932	0.224938369
ENSRNOG00000018690	Rgs17	123.3008748	-0.115668562	0.120374232
ENSRNOG00000016547	Rgs19	89.45297418	0.025793259	0.155900634
ENSRNOG00000003687	Rgs2	26.22606011	-0.0109387	0.281552955
ENSRNOG00000008100	Rgs20	131.2841438	0.053512475	0.126529054
ENSRNOG00000025551	Rgs22	8.608009483	0.270831491	0.503533969
ENSRNOG00000024501	Rgs3	64.81940549	0.149935682	0.168614464
ENSRNOG00000002730	Rgs5	21.56186328	0.07167679	0.287627585
ENSRNOG00000008082	Rgs6	164.3091213	0.068235857	0.114030229
ENSRNOG00000002369	Rgs8	77.85590437	-0.070198689	0.160398891
ENSRNOG00000003800	Rgs9	10.36589069	0.303045572	0.412778478
ENSRNOG00000026952	Rgsl1	12.37374313	0.084768835	0.359293851
ENSRNOG00000001443	Rhbdd2	10.82482798	-0.25871229	0.35871002
ENSRNOG00000005515	Rhbdl3	181.8314229	-0.118323079	0.110699383
ENSRNOG00000054385	Rhebl1	102.2933564	0.06859172	0.149684858
ENSRNOG00000028517	Rhno1	41.78127425	0.038571196	0.194304696
ENSRNOG00000050519	Rhoa	19.77760596	0.236196922	0.280880694
ENSRNOG00000000633	Rhobtb1	24.38445093	-0.0243814	0.265579349
ENSRNOG00000017373	Rhobtb2	28.90872483	-0.210836995	0.259930348
ENSRNOG00000012414	Rhobtb3	27.3845553	0.588435801	0.263197271
ENSRNOG00000042607	Rhof	30.76164834	-0.176932476	0.278176395
ENSRNOG00000020393	Rhog	83.08124904	-0.136120265	0.159218924
ENSRNOG00000021919	Rhoj	24.21627794	0.58147157	0.319833931
ENSRNOG00000019930	Rhot2	30.38360814	0.285304501	0.265683468
ENSRNOG00000013380	Rhov	9.097864652	0.840198451	0.514164464
ENSRNOG00000007597	Rhpn1	51.79343547	-0.040040372	0.173211947
ENSRNOG00000011885	Rhpn2	8.481215645	-0.211998511	0.451967088
ENSRNOG00000016172	Ric1	53.21673305	-0.036905586	0.240881282
ENSRNOG00000007323	Ric8b	12.83757532	-0.114793555	0.361798424
ENSRNOG00000011341	Rictor	12.32491532	0.071755734	0.367750763
ENSRNOG00000005437	Rida	111.8563452	-0.088626165	0.134968987
ENSRNOG00000054901	Rif1	26.77546651	-0.56992591	0.250887336
ENSRNOG00000037310	Riiad1	23.15270526	0.257034789	0.299972041
ENSRNOG00000001055	Rilpl1	44.46516375	-0.102367182	0.212165456

ENSRNOG00000022893	Rimbp2	146.4090715	0.09239086	0.155175875
ENSRNOG00000030452	Rimbp3	11.97333772	-0.279894612	0.387547027
ENSRNOG00000008625	Rimkla	58.81992165	-1.938590099	0.568355287
ENSRNOG00000011000	Rims1	144.4146418	0.008710461	0.183114163
ENSRNOG00000011171	Rims3	27.11260002	-0.011824406	0.288970448
ENSRNOG00000010131	Rin2	78.63235027	-0.001178231	0.145724071
ENSRNOG00000022182	Rint1	39.04939833	0.246558526	0.213708731
ENSRNOG00000014049	Riok1	148.1044299	-0.085521513	0.154289361
ENSRNOG00000012692	Riok2	68.88684852	-0.19946089	0.160821055
ENSRNOG00000001680	Riox2	8.584732108	0.341238105	0.449291011
ENSRNOG00000017787	Ripk1	11.47104469	0.121944154	0.384484688
ENSRNOG00000009389	Ripk2	50.08474048	0.159457686	0.204173057
ENSRNOG00000018804	Ripor2	51.25920657	0.111185454	0.207999009
ENSRNOG00000010791	Ripor3	141.8010556	-0.113606435	0.126593323
ENSRNOG00000016897	Rlbp1	177.6147175	-0.012556632	0.120654504
ENSRNOG00000027921	Rlf	59.91237804	-0.084519648	0.173177568
ENSRNOG00000060867	Rln1	22.05642055	0.4723135	0.29180377
ENSRNOG00000012597	Rmc1	9.694926072	-0.026521171	0.417323096
ENSRNOG00000025145	Rmdn1	105.2598767	-0.191998586	0.151194753
ENSRNOG00000006082	Rmdn2	14.61970735	0.158402403	0.372819029
ENSRNOG00000011690	Rmdn3	11.22494093	0.103923409	0.415797182
ENSRNOG00000019108	Rmi1	47.89389354	0.13831692	0.185589201
ENSRNOG00000019501	Rmnd1	149.0105285	0.018497735	0.134259715
ENSRNOG00000047396	Rmnd5b	56.78506857	-0.109827429	0.17491468
ENSRNOG00000008584	Rnaseh1	33.26879585	0.056192829	0.230462882
ENSRNOG00000003504	Rnaseh2a	24.08727364	0.354545263	0.26117637
ENSRNOG00000009192	Rnaseh2b	68.75572703	-0.07799247	0.18103894
ENSRNOG00000027017	Rnasel	50.60000837	-0.227293538	0.183881863
ENSRNOG00000013190	Rnaset2	129.9914771	-0.209066132	0.118289363
ENSRNOG00000004624	Rnd3	62.26205495	0.38198128	0.203508226
ENSRNOG00000001172	Rnf10	105.1642712	-0.119857059	0.160374612
ENSRNOG00000007272	Rnf103	176.7493129	-0.140973863	0.109425865
ENSRNOG00000060750	Rnf111	169.2009782	-0.105384527	0.109366845
ENSRNOG00000057589	Rnf11l1	34.4063932	-0.230374476	0.203414081
ENSRNOG00000023473	Rnf122	20.80977036	0.400710014	0.277269871
ENSRNOG00000033378	Rnf123	70.15961091	0.249910835	0.161684652
ENSRNOG00000009028	Rnf126	45.71818051	0.085155245	0.225148614
ENSRNOG00000055721	Rnf128	59.28881688	0.303892897	0.168023603
ENSRNOG00000004120	Rnf138rt1	12.7511154	-0.663867201	0.33967305
ENSRNOG00000007370	Rnf144a	148.6078765	0.109142164	0.105882074
ENSRNOG00000016123	Rnf144b	111.6769389	-0.004302452	0.135506518
ENSRNOG00000011588	Rnf146	14.7639674	0.049683243	0.365995559
ENSRNOG00000013946	Rnf149	11.26069586	0.070015312	0.41077867
ENSRNOG00000003479	Rnf150	80.3855045	-0.238013122	0.153580032
ENSRNOG00000014127	Rnf151	10.57670786	-0.183300057	0.408347469
ENSRNOG00000014859	Rnf152	7.991827782	0.049139206	0.472139096
ENSRNOG00000049862	Rnf157	23.33202158	0.01124975	0.279999691
ENSRNOG00000013777	Rnf166	131.3546775	-0.03725939	0.128009584
ENSRNOG00000043345	Rnf168	91.24131042	0.016522537	0.131957249
ENSRNOG00000012035	Rnf181	60.48328104	0.099516865	0.161300988

ENSRNOG00000018070	Rnf182	27.41364713	0.502936543	0.266070853
ENSRNOG00000019325	Rnf185	163.7627719	-0.00925181	0.120289514
ENSRNOG00000009658	Rnf19a	9.335254451	-0.518711315	0.444567915
ENSRNOG00000002454	Rnf2	57.92188241	-0.220374309	0.189514798
ENSRNOG00000006087	Rnf20	114.8503275	-0.197384454	0.118448513
ENSRNOG00000029658	Rnf213	62.91378164	0.426542747	0.523572571
ENSRNOG00000004940	Rnf215	50.48155277	0.104208099	0.187575056
ENSRNOG00000001101	Rnf216	175.8346816	0.004433844	0.109651735
ENSRNOG00000013323	Rnf217	9.654821996	0.098733302	0.41516252
ENSRNOG00000019236	Rnf220	184.3281896	-0.584333321	0.458786275
ENSRNOG00000021250	Rnf24	61.61100717	-0.11220625	0.171612506
ENSRNOG00000016886	Rnf25	7.919352382	0.482897852	0.448909049
ENSRNOG00000019438	Rnf31	11.90958005	-0.165956903	0.367158015
ENSRNOG00000005691	Rnf32	58.54543657	0.091288598	0.162496483
ENSRNOG00000001331	Rnf34	20.86356365	0.494852863	0.35905685
ENSRNOG00000013956	Rnf38	27.52248244	-0.292741455	0.256914329
ENSRNOG00000000781	Rnf39	10.91344589	-0.606585188	0.388650992
ENSRNOG00000013930	Rnf4	13.71728842	0.08091251	0.36290868
ENSRNOG00000018840	Rnf40	89.12273823	-0.38934468	0.159239843
ENSRNOG00000007606	Rnf43	9.551419691	0.622222538	0.444493458
ENSRNOG00000000968	Rnf6	46.92200701	0.20383428	0.193426533
ENSRNOG00000047171	Rnf8	16.29397484	-0.119836842	0.373315619
ENSRNOG00000003805	Rnft1	75.60367056	0.113173628	0.159600422
ENSRNOG00000007867	Rngtt	115.1840516	0.089168081	0.14956779
ENSRNOG00000016416	Rnh1	35.3162352	0.725297301	0.524401899
ENSRNOG00000020705	Rnls	22.09338113	0.26108182	0.306976231
ENSRNOG00000016698	Rnmt	187.4313249	-0.003295783	0.109185513
ENSRNOG00000017310	Rnpc3	62.25265814	0.285795717	0.19728035
ENSRNOG00000006720	Rnpep	102.6816268	0.627288579	0.408519437
ENSRNOG00000045775	Rnpepl1	154.120422	0.141036541	0.109062804
ENSRNOG00000008703	Rnps1	22.834215	-0.566644767	0.298436664
ENSRNOG00000003434	Ro60	8.596531688	0.251306119	0.429630947
ENSRNOG00000029614	Robo1	37.15970672	-0.115067605	0.22213252
ENSRNOG00000030016	Robo3	17.40210682	0.030546918	0.363664527
ENSRNOG00000053232	Ror2	9.570812268	-0.069736941	0.416214201
ENSRNOG00000027145	Rora	20.42952052	0.188203493	0.313359039
ENSRNOG00000013413	Rorb	17.31244753	-0.017776479	0.347192866
ENSRNOG00000000406	Ros1	12.5448326	0.065392558	0.410448216
ENSRNOG00000025428	Rp2	19.9006087	0.236968774	0.294260664
ENSRNOG00000029456	Rp9	61.89584908	-0.09496835	0.175429965
ENSRNOG00000003123	Rpa1	125.0741065	0.000977863	0.122119236
ENSRNOG00000013005	Rpa2	16.50426903	-0.343611699	0.488990469
ENSRNOG00000008309	Rpa3	39.94058236	-0.144952945	0.220525971
ENSRNOG00000005483	Rpap1	80.54182997	-0.080030967	0.175854884
ENSRNOG00000023484	Rpap2	105.8327645	-0.06300406	0.147194232
ENSRNOG00000038085	Rpe	71.85487081	0.099412467	0.148508493
ENSRNOG00000015969	Rpf1	14.32393456	0.459332458	0.380281158
ENSRNOG00000000587	Rpf2	130.5858499	-0.235101782	0.133793938
ENSRNOG00000003013	Rpgr	21.10742926	-0.519570532	0.328287507
ENSRNOG00000011809	Rpgrip1	174.1855972	0.199002113	0.105947194

ENSRNOG00000011829	Rpgrip1l	66.71162825	0.05071646	0.162674749
ENSRNOG00000001368	Rph3a	53.95761304	0.047839791	0.174995525
ENSRNOG00000061429	Rph3al	16.25238432	0.273918833	0.335252176
ENSRNOG00000005576	Rpia	75.87189965	-0.045975497	0.156191015
ENSRNOG00000018795	Rpl18a	120.4230196	-0.014650108	0.154866561
ENSRNOG00000001611	Rpl24	152.4233365	-0.136444255	0.135471835
ENSRNOG00000011138	Rpl29	101.3333334	-1.029518983	0.384641319
ENSRNOG00000005975	Rpl30	25.62650216	0.044238306	0.310997488
ENSRNOG00000010746	Rpl32	4.689138025	-2.248394371	1.458024091
ENSRNOG00000032605	Rpl32-ps3	81.60771617	0.363179946	0.75393586
ENSRNOG00000011494	Rpl36a	151.5636866	0.086841733	0.108148201
ENSRNOG00000033803	Rpl37	139.1376039	-0.299834389	0.171617546
ENSRNOG00000029377	Rpl38	104.5177398	-0.344970287	0.155570247
ENSRNOG00000033686	Rpl38	88.4393706	-0.324394078	0.147337504
ENSRNOG00000048701	Rpl38	143.1875037	-0.260665681	0.134920151
ENSRNOG00000049047	Rpl38	125.916716	-0.263388014	0.137215386
ENSRNOG00000023529	Rpl5	9.711612116	-0.744420936	0.461806769
ENSRNOG00000032635	Rpl8	10.14637279	-2.573391584	1.854481871
ENSRNOG00000030476	Rpl9	8.453231641	-0.423914994	0.409101065
ENSRNOG00000001148	Rplp0	10.85850069	0.612177612	0.498579746
ENSRNOG00000039829	Rpp14	129.1082511	-0.078984285	0.134372674
ENSRNOG00000000786	Rpp21	79.50317097	0.044587332	0.154515912
ENSRNOG00000018812	Rpp25	72.93968079	0.014648973	0.167899519
ENSRNOG00000018718	Rpp30	77.77747579	-0.121484704	0.149902376
ENSRNOG00000016226	Rpp40	21.39381963	-0.698946246	0.254904324
ENSRNOG00000012923	Rprd1b	86.67105092	-0.034007615	0.174626954
ENSRNOG00000045885	Rps17	134.4087971	-0.338383421	0.130183058
ENSRNOG00000029627	Rps20	174.7101606	-0.057003109	0.108129275
ENSRNOG00000027503	Rps25	0.186192237	-0.528147103	3.0280184
ENSRNOG00000016961	Rps27	77.12502246	-0.223300052	0.172322574
ENSRNOG00000020492	Rps27a-ps28	118.5891238	-0.23246361	0.11973168
ENSRNOG00000050473	Rps27l	27.26985177	0.108288322	0.238627109
ENSRNOG00000019453	Rps5	9.281572787	0.219147204	0.429569781
ENSRNOG00000042411	Rps6ka1	54.40550042	-0.097117924	0.20528909
ENSRNOG00000013194	Rps6ka2	187.5916693	-0.072928424	0.116038424
ENSRNOG00000006632	Rps6ka3	52.25847236	0.040953359	0.204480908
ENSRNOG00000004362	Rps6ka5	95.76495001	-0.019940317	0.183389827
ENSRNOG00000002592	Rps6ka6	17.95586605	0.192831249	0.357451154
ENSRNOG00000003919	Rps6kb1	65.28256186	-0.127180113	0.154166321
ENSRNOG00000021689	Rps6kb2	28.35721753	0.087243335	0.273172608
ENSRNOG00000005530	Rps6kl1	168.762301	-0.073817147	0.115716774
ENSRNOG00000003821	Rptor	177.3245368	-0.077907695	0.099435597
ENSRNOG00000023639	Rpusd1	170.4831399	0.02002784	0.109386853
ENSRNOG00000010528	Rpusd2	39.54332345	0.084088588	0.213711888
ENSRNOG00000009084	Rpusd3	13.46296982	-0.266751872	0.40840184
ENSRNOG00000011567	Rpusd4	79.34484429	-0.268266049	0.166510477
ENSRNOG00000007051	Rraga	182.8718703	-0.108564149	0.110154472
ENSRNOG00000007331	Rragd	47.04778849	0.092570183	0.212253689
ENSRNOG00000037247	Rras	78.83328348	-0.245341032	0.168186744
ENSRNOG00000012258	Rras2	161.4016613	0.071313932	0.103821257

ENSRNOG00000005958	Rrbp1	61.43538678	-0.144832831	0.219516553
ENSRNOG00000015701	Rreb1	11.75149786	-0.500358966	0.370228145
ENSRNOG00000045752	Rrm1	72.55844075	0.008571948	0.153801264
ENSRNOG00000025454	Rrm2b	15.89566925	0.12146464	0.319956734
ENSRNOG00000003326	Rrn3	13.32279901	-0.341472202	0.451469844
ENSRNOG00000011645	Rrnad1	22.99485498	0.261465183	0.312223981
ENSRNOG00000048495	Rrp12	74.23484703	-0.365971443	0.17818707
ENSRNOG00000002450	Rrp15	75.13855217	-0.377674638	0.144683336
ENSRNOG00000001194	Rrp1b	30.63127464	0.168277891	0.223406826
ENSRNOG00000017836	Rrp36	22.54156339	-0.013897003	0.295819344
ENSRNOG00000022896	Rrp7a	117.7506342	0.240426148	0.891159349
ENSRNOG00000018766	Rrp8	46.53800574	-0.148230508	0.206113512
ENSRNOG00000012927	Rrp9	24.44770136	0.07192044	0.281505993
ENSRNOG00000030434	Rs1	25.59693561	0.146303148	0.270062498
ENSRNOG00000019671	Rsbn1	35.33495733	0.010678089	0.215920192
ENSRNOG00000013431	Rsbn1l	89.53752413	-0.187573571	0.157312595
ENSRNOG00000024194	Rsf1	76.00152245	-0.116328996	0.179858146
ENSRNOG00000057862	Rsph1	20.77761353	0.021898568	0.288145324
ENSRNOG00000001036	Rsph10b	38.62541263	0.013057631	0.26920616
ENSRNOG00000018762	Rsph3	12.72112653	0.168573689	0.391423383
ENSRNOG00000061526	Rsph6a	15.6542874	0.285288033	0.342460141
ENSRNOG00000019474	Rsph9	45.47697758	-0.09304262	0.225959458
ENSRNOG00000009656	Rspo1	10.33632813	0.202556326	0.392497299
ENSRNOG00000037687	Rspo2	125.5331699	0.019382111	0.147537445
ENSRNOG00000011568	Rspo3	132.3962087	-0.125522169	0.141542808
ENSRNOG00000060931	Rspry1	29.20175831	-0.202732426	0.241466393
ENSRNOG00000017595	Rsu1	24.27995267	0.4000846	0.288307543
ENSRNOG00000030712	RT1-CE1	107.9721917	0.328755181	0.238346066
ENSRNOG00000038999	RT1-CE11	60.81737069	-0.447574924	0.287515124
ENSRNOG00000033215	RT1-Db1	13.66065184	1.012318132	0.379972533
ENSRNOG00000030431	RT1-Db2	105.1126165	-0.042448083	0.159505478
ENSRNOG00000049491	RT1-DMb	51.42113142	-0.086145815	0.200253052
ENSRNOG00000000763	RT1-M3-1	12.58443601	0.598441286	0.399149585
ENSRNOG00000029386	RT1-N2	93.75027631	0.274807983	0.190697189
ENSRNOG00000000777	RT1-S3	71.32825882	0.132113516	0.238204616
ENSRNOG00000032596	RT1-T24-1	11.83522143	-0.039015935	0.38356207
ENSRNOG00000042905	RT1-T24-4	12.33144196	0.133250595	0.501355532
ENSRNOG00000043215	Rtbdn	19.89038678	0.331103984	0.324106683
ENSRNOG00000005083	Rtfdc1	9.591276594	0.363435736	0.426680849
ENSRNOG00000009022	Rtkn	21.27029306	0.019894577	0.339660954
ENSRNOG00000021202	Rtn3	131.1491343	-0.162289172	0.208917826
ENSRNOG00000021513	Rtn4rl2	26.99398171	0.16325474	0.254077348
ENSRNOG00000000340	Rtraf	107.8042556	0.020715997	0.121156596
ENSRNOG00000038200	Rttn	10.15363793	-0.161152697	0.380724699
ENSRNOG00000059880	Rubcn	16.73770813	0.099195828	0.337844828
ENSRNOG00000003536	Rufy1	25.80223767	-0.161602592	0.276432479
ENSRNOG00000023768	Rundc1	10.08279694	-0.421923533	0.409261326
ENSRNOG00000020970	Rundc3a	66.62502629	-0.225946963	0.359199826
ENSRNOG00000008463	Rundc3b	159.0829444	-0.197423707	0.110951156
ENSRNOG00000005673	Runx1t1	176.5645069	-0.030825264	0.151333967

ENSRNOG00000020193	Runx2	13.57835746	0.304145913	0.383899154
ENSRNOG00000045843	Rusc2	16.85990337	-0.299725634	0.30593871
ENSRNOG00000020793	Ruvbl2	8.605355692	0.037726302	0.418569146
ENSRNOG00000009599	Rwdd2a	26.28452396	0.009028147	0.270611861
ENSRNOG00000001599	Rwdd2b	76.45681201	0.028658721	0.150045775
ENSRNOG00000029893	Rwdd3	21.85241585	-0.060131948	0.280785859
ENSRNOG00000022500	Rwdd4	133.9173122	0.052612638	0.121302124
ENSRNOG00000024120	Rxfp1	74.71033834	0.087542341	0.185292093
ENSRNOG00000009446	Rxra	11.0132335	0.23934357	0.387961988
ENSRNOG00000004537	Rxrg	79.83786904	-0.120029919	0.179571558
ENSRNOG00000004421	Rxylt1	122.6571543	0.052065279	0.130935037
ENSRNOG00000005353	Rybp	182.846209	0.006164302	0.099935584
ENSRNOG00000008593	Ryk	52.51017022	-0.176613668	0.182250374
ENSRNOG00000020557	Ryr1	28.98865836	0.316616891	0.267364464
ENSRNOG00000017060	Ryr2	102.9058055	0.224753577	0.189522243
ENSRNOG00000010105	S100a11	17.51332528	0.332696784	0.311139375
ENSRNOG00000011647	S100a6	16.11673724	0.203618256	0.370687078
ENSRNOG00000020653	S1pr2	21.71733261	0.44261181	0.300236491
ENSRNOG00000020901	S1pr5	45.44061105	-0.103214935	0.206830039
ENSRNOG00000005149	Sacm1l	25.79389644	0.304082084	0.371332986
ENSRNOG00000014509	Sacs	171.1038815	0.142353134	0.139368565
ENSRNOG00000050543	Safb	61.81075191	0.001453814	0.19223854
ENSRNOG00000049511	Safb2	60.66906163	-0.18405087	0.178881067
ENSRNOG00000013907	Sall1	55.49028341	0.149819426	0.207547325
ENSRNOG00000013287	Sall2	85.08291752	-0.054906433	0.148929145
ENSRNOG00000026116	Sall3	18.81807356	-0.110141908	0.359415184
ENSRNOG00000020342	Samd11	80.34800522	0.009254113	0.175213867
ENSRNOG00000043390	Samd12	30.75457279	-0.038943281	0.233511439
ENSRNOG00000050413	Samd15	9.450916382	0.301523895	0.441665044
ENSRNOG00000010489	Samd4a	54.24849643	0.068648403	0.195828303
ENSRNOG00000023549	Samd5	11.22275076	-0.038994872	0.379783095
ENSRNOG00000052444	Samd9	115.3732411	0.080387159	0.150967467
ENSRNOG00000016773	Sap130	121.1556489	-0.140383379	0.123203842
ENSRNOG00000013218	Sap30	16.54329155	-0.015066966	0.311837453
ENSRNOG00000013069	Sapcd2	9.008362562	0.247516438	0.410812417
ENSRNOG00000006916	Sardh	40.95928628	-0.298335084	0.232328334
ENSRNOG00000010244	Sarm1	32.89240848	0.380873107	0.272396645
ENSRNOG00000000702	Sart3	10.9827375	-0.158560709	0.426454488
ENSRNOG00000013160	Sash1	137.3420942	0.149471196	0.137918068
ENSRNOG00000004409	Sash3	10.31905837	-0.084027767	0.420753315
ENSRNOG00000011714	Sat2	118.0082234	-0.199460394	0.133014313
ENSRNOG00000005264	Sav1	176.196903	-0.126954239	0.104071646
ENSRNOG00000000888	Sbds	66.6816203	-0.5453505	0.173157088
ENSRNOG00000009812	Sbf2	76.20446568	0.134590517	0.223337372
ENSRNOG00000021015	Sbsn	36.4462262	-0.1657286	0.233524643
ENSRNOG00000007258	Sbspon	9.434452888	-0.12335636	0.47235864
ENSRNOG00000056946	Scaf1	52.5480046	0.418087846	0.46285692
ENSRNOG00000005263	Scaf11	101.0467699	0.027692399	0.128339827
ENSRNOG00000002104	Scaf4	38.27682268	0.168301007	0.271529211
ENSRNOG00000016919	Scaf8	88.44619473	-0.096672603	0.145544902

ENSRNOG00000025278	Scai	171.1844112	0.019051848	0.118282128
ENSRNOG00000054008	Scamp5	34.70996084	0.178532222	0.225543579
ENSRNOG00000020853	Scap	27.13965383	-0.025337475	0.246726853
ENSRNOG00000006864	Scaper	109.7980525	0.090456617	0.127403816
ENSRNOG00000002225	Scarb2	84.08667264	-0.081267354	0.194935114
ENSRNOG00000059618	Scarf1	33.93170739	-0.203557573	0.232976961
ENSRNOG00000000288	Scarf2	46.4476306	0.002960345	0.211470101
ENSRNOG00000013552	Scd	55.27935223	0.024505913	0.174281228
ENSRNOG00000031203	Scfd1	160.3199562	-0.073849723	0.113130173
ENSRNOG00000026828	Scfd2	77.05396519	-0.105669707	0.143897069
ENSRNOG00000016628	Scgn	11.42702197	0.132663989	0.366373114
ENSRNOG00000014139	Sclt1	58.60916474	0.259520348	0.18805894
ENSRNOG00000020083	Scly	8.442697233	0.396702754	0.446452401
ENSRNOG00000032183	Scmh1	21.32944686	0.273920865	0.310996979
ENSRNOG00000026110	Scml4	26.03362406	-0.443190913	0.358057928
ENSRNOG00000026679	Scn4b	47.66719653	0.194422634	0.226875519
ENSRNOG00000005309	Scn8a	70.32224706	-0.015224079	0.172108003
ENSRNOG00000006639	Scn9a	29.87041059	0.349000646	0.270563429
ENSRNOG00000028699	Sco1	59.22600757	-0.13105803	0.188831968
ENSRNOG00000011413	Scp2	116.7464824	-0.053702989	0.171211164
ENSRNOG00000032574	Scrib	147.0715122	0.021623333	0.109068772
ENSRNOG00000009636	Scrn1	120.3542649	0.071226227	0.13657256
ENSRNOG00000018657	Scrn3	11.37786498	0.255010524	0.417132327
ENSRNOG00000025594	Scrt1	16.71517797	0.376229736	0.35053606
ENSRNOG00000005148	Scrt2	10.42257388	0.128220894	0.3876574
ENSRNOG00000010617	Scube1	36.78131629	0.299066153	0.223088034
ENSRNOG00000013123	Scube2	14.12741938	0.64175202	0.36322883
ENSRNOG00000000500	Scube3	24.11244952	0.111155848	0.264439872
ENSRNOG00000021812	Scx	26.11327571	-0.329313832	0.26105094
ENSRNOG00000023668	Scyl1	8.252501729	-0.388442742	0.443306798
ENSRNOG00000024955	Scyl2	115.5642624	0.031142196	0.136731117
ENSRNOG00000025318	Scyl3	8.778124955	0.08530491	0.421213382
ENSRNOG00000022229	Sdad1	17.34934669	-0.22095839	0.341973342
ENSRNOG00000009683	Sdcbp	47.56343113	0.043203099	0.189214021
ENSRNOG00000003247	Sde2	94.18270222	-0.149522791	0.134027889
ENSRNOG00000001859	Sdf2l1	35.9435136	0.030088949	0.208539958
ENSRNOG00000019981	Sdf4	28.00157259	0.294549224	0.292883278
ENSRNOG00000011283	Sdhaf3	17.86022313	0.052849314	0.350520425
ENSRNOG00000003163	Sdhc	75.44377633	-0.976893419	0.247234795
ENSRNOG00000001103	Sdk1	25.50533867	0.047200435	0.257270507
ENSRNOG00000024711	Sdk2	82.65839533	0.013133594	0.203676955
ENSRNOG00000020544	Sdr39u1	19.82483095	0.636586991	0.29516379
ENSRNOG00000051819	Sdr42e1	23.21844115	-0.407285135	0.257728195
ENSRNOG00000001388	Sds	28.08614924	-0.100340902	0.239782323
ENSRNOG00000001391	Sdsl	31.86568891	-0.304086557	0.249007659
ENSRNOG00000004672	Sec14l2	28.2533567	0.01827713	0.258898246
ENSRNOG00000019122	Sec16a	13.73566789	-0.367177101	0.368843718
ENSRNOG00000005229	Sec16b	9.461771235	0.861164775	0.51212015
ENSRNOG00000043069	Sec22a	26.17543457	1.592553274	0.399320761
ENSRNOG00000047873	Sec22c	11.36747682	0.263033812	0.379675815

ENSRNOG00000004657	Sec23a	18.0819898	0.290881014	0.331155038
ENSRNOG00000020411	Sec23ip	138.0208923	0.074431052	0.133503436
ENSRNOG00000004563	Sec24a	44.88027069	0.078376378	0.226007853
ENSRNOG00000023373	Sec24b	166.5824169	0.091013552	0.112514709
ENSRNOG00000009042	Sec24c	24.22813716	-0.271037626	0.304668216
ENSRNOG00000002251	Sec31a	187.0579629	0.089033318	0.093309625
ENSRNOG00000013773	Secisbp2	42.0832413	0.108481444	0.206222667
ENSRNOG00000004932	Sel1l3	49.5774289	0.142085988	0.189743486
ENSRNOG00000047158	Selenbp1	46.46113108	0.048696485	0.193628542
ENSRNOG00000054563	Selenoh	20.81375588	-1.778814397	1.125644774
ENSRNOG00000059295	Selenoi	32.64021442	-0.139976719	0.24048485
ENSRNOG00000014624	Selenok	48.43558734	0.187266506	0.193478921
ENSRNOG00000061231	Selenom	54.29640956	0.129649022	0.271685601
ENSRNOG00000017078	Selenon	9.413991135	0.122203704	0.408560731
ENSRNOG00000060120	Selenoo	40.67457219	0.038613613	0.20781694
ENSRNOG00000012576	Selenos	142.2480521	-0.102827631	0.114373528
ENSRNOG00000013507	Selenot	35.69452437	0.056508464	0.229022935
ENSRNOG00000051208	Selenov	78.86863671	-0.101632458	0.163517772
ENSRNOG00000000699	Selplg	13.68092769	0.005918722	0.337253924
ENSRNOG00000006526	Sema3c	176.1643473	0.052273251	0.122443069
ENSRNOG00000007202	Sema3d	8.943850822	0.006568687	0.398232643
ENSRNOG00000006631	Sema3e	42.58314724	-0.180467517	0.27324787
ENSRNOG00000025167	Sema4b	11.95829783	0.230633244	0.377048397
ENSRNOG00000013679	Sema4d	71.0302829	0.059360539	0.200587733
ENSRNOG00000004033	Sema6a	10.60948639	0.387236269	0.421280845
ENSRNOG00000045998	Sema6b	14.37296417	0.023648878	0.43294284
ENSRNOG00000021101	Sema6c	14.28221162	0.208388498	0.356869428
ENSRNOG00000004812	Sema6d	123.9554871	0.137176639	0.131483201
ENSRNOG00000007687	Sema7a	28.51475995	0.628985292	0.279289708
ENSRNOG00000001773	Senp2	15.27068351	0.29170181	0.326282827
ENSRNOG00000013746	Senp3	12.79670852	-0.768583453	0.49940285
ENSRNOG00000001616	Senp7	10.78870869	0.325052759	0.437897488
ENSRNOG00000011595	Senp8	23.07890949	0.465062478	0.330553235
ENSRNOG00000018125	Sephs1	103.0756246	-0.116875716	0.133490719
ENSRNOG00000048397	Sephs2	180.1119137	-0.162938518	0.098121698
ENSRNOG00000049244	Sepsecs	47.09412995	0.124090481	0.186597321
ENSRNOG00000049507	Septin10	153.9700359	-0.009321711	0.118361561
ENSRNOG00000017952	Septin2	11.62956621	0.450800169	0.387407147
ENSRNOG00000007686	Septin3	104.2794988	0.016538715	0.144797733
ENSRNOG00000007367	Septin4	87.72217106	0.320214002	0.160931728
ENSRNOG00000029912	Septin5	87.04558898	-0.102466635	0.153021482
ENSRNOG00000017889	Serac1	27.13765345	-0.062716811	0.267936507
ENSRNOG00000017945	Serf1	182.0966914	0.089382459	0.109804161
ENSRNOG00000029360	Serinc1	31.72964437	-0.239643016	0.229351165
ENSRNOG00000012989	Serinc2	89.29726639	0.063637786	0.153265417
ENSRNOG00000024039	Serinc5	80.48162277	0.411617758	0.16438157
ENSRNOG00000050761	Serpina10	12.24026519	0.063456106	0.423298251
ENSRNOG00000011141	Serpina11	11.31985812	-0.122068298	0.427611741
ENSRNOG00000016581	Serpinb1a	15.58878456	-0.013239796	0.330062586
ENSRNOG00000017962	Serpinb6a	120.2132565	0.061194591	0.137469918

ENSRNOG00000016420	Serpinb6b	9.34489129	0.197910269	0.399435928
ENSRNOG00000033772	Serpinb9	34.21044756	-0.022136834	0.273399328
ENSRNOG00000002783	Serpinc1	14.72342738	-0.053462724	0.342937748
ENSRNOG00000001865	Serpind1	18.66554912	-0.570977332	0.340068084
ENSRNOG00000009814	Serpine3	11.38298035	-0.443408947	0.36355653
ENSRNOG00000003172	Serpinf1	8.368638644	0.183648911	0.482439918
ENSRNOG00000024363	Sertad1	134.3922901	-0.095711099	0.165983505
ENSRNOG00000005393	Sertad2	18.1379701	-0.048998132	0.333073198
ENSRNOG00000037690	Sertad3	110.0698337	-0.014731521	0.149484593
ENSRNOG00000060773	Sertad4	65.35897218	-0.022410804	0.207264606
ENSRNOG00000014046	Sertm1	76.61003158	-0.03150281	0.165755894
ENSRNOG00000000302	Sesn1	187.9165299	-0.037437913	0.109684308
ENSRNOG00000042785	Sesn2	120.495089	-0.111581925	0.121952285
ENSRNOG00000008173	Sesn3	63.23975521	-0.265922621	0.175063744
ENSRNOG00000012603	Sestd1	155.0535502	-0.019072412	0.167945659
ENSRNOG00000055028	Setd1a	9.137395943	0.104063431	0.397270225
ENSRNOG00000001337	Setd1b	14.32435852	0.088565798	0.324852672
ENSRNOG00000020915	Setd2	34.11418943	-0.006517389	0.227622708
ENSRNOG00000006587	Setd3	97.62123417	0.171218693	0.136167586
ENSRNOG00000001699	Setd4	50.41825661	0.259656819	0.188708766
ENSRNOG00000007472	Setd5	179.1107661	0.001155165	0.125401255
ENSRNOG00000013045	Setd7	42.62848924	0.030196434	0.231298627
ENSRNOG00000021143	Setdb1	26.36702513	0.247533917	0.338570336
ENSRNOG00000021680	Setdb2	9.511311811	-0.069000284	0.394667853
ENSRNOG00000006806	Setmar	16.05369388	-0.669925327	0.320603301
ENSRNOG00000013491	Setx	53.24025334	0.446560083	0.201958555
ENSRNOG00000025612	Sez6l	58.30218496	0.199545419	0.223398618
ENSRNOG00000027098	Sez6l2	45.81756217	0.062444691	0.2085765
ENSRNOG00000017724	Sf3b3	98.37178503	-0.068129809	0.12744877
ENSRNOG00000046172	Sf3b6	38.45639754	-0.044796026	0.235712915
ENSRNOG00000016645	Sfmbt1	91.97355819	0.182602613	0.133623076
ENSRNOG00000033153	Sfn	188.8650922	-0.008214254	0.104288343
ENSRNOG00000017783	Sfrp1	37.68651388	0.220839463	0.316174972
ENSRNOG00000009465	Sfrp2	38.63548055	-0.252216743	0.235565101
ENSRNOG00000054957	Sfrp4	31.76932554	0.031586364	0.240381912
ENSRNOG00000000931	Sfswap	181.9151633	0.093462882	0.138388625
ENSRNOG00000024140	Sft2d1	187.3971539	0.119325762	0.121749223
ENSRNOG00000003038	Sft2d2	13.62346282	0.555200018	0.476725913
ENSRNOG00000010761	Sftpb	14.85574935	0.060541721	0.346286427
ENSRNOG00000003998	Sgca	189.9440208	0.002940996	0.291611479
ENSRNOG00000046905	Sgce	109.9050259	-0.016487162	0.138199155
ENSRNOG00000006357	Sgip1	43.54824984	0.185215113	0.194090977
ENSRNOG00000049052	Sgk3	15.01929975	-0.138130777	0.338272063
ENSRNOG00000012536	Sgms1	38.03493423	0.054439873	0.22834158
ENSRNOG00000000565	Sgpl1	42.58073056	0.253633934	0.207231987
ENSRNOG00000005175	Sgpp1	155.0496475	0.162110389	0.107329032
ENSRNOG00000027194	Sgsm2	56.8065476	0.333389062	0.214699967
ENSRNOG00000011937	Sgtb	34.84555314	0.295422887	0.231139265
ENSRNOG00000054560	Sh2d3c	12.76517859	-0.108116465	0.382240894
ENSRNOG00000014909	Sh2d5	31.09253612	-0.228256489	0.243209812

ENSRNOG00000028238	Sh3bgr	27.92762173	-0.028543153	0.262884823
ENSRNOG00000013747	Sh3bp2	16.38148984	0.063261294	0.315430441
ENSRNOG00000019316	Sh3bp4	151.4577694	0.159623022	0.130421679
ENSRNOG00000054400	Sh3bp5l	126.0755865	-0.008755606	0.116545368
ENSRNOG00000011752	Sh3d19	29.53585239	0.387356524	0.267584567
ENSRNOG00000024566	Sh3d21	165.1398983	0.086391521	0.107735842
ENSRNOG00000049683	Sh3gl1	10.09513951	0.510952487	0.456084146
ENSRNOG00000006761	Sh3gl2	24.69773805	-0.186082403	0.304511962
ENSRNOG00000017295	Sh3glb2	29.90040764	-0.144984352	0.267812507
ENSRNOG00000020353	Sh3pxd2a	15.82723898	0.052347996	0.342391737
ENSRNOG00000004063	Sh3pxd2b	17.77137365	-0.05629459	0.340218086
ENSRNOG00000046791	Sh3rf3	15.41407964	0.096292746	0.337498122
ENSRNOG00000007993	Sh3tc1	10.68299162	0.117273785	0.476148216
ENSRNOG00000005522	Sh3yl1	34.87059602	0.193270722	0.256417164
ENSRNOG00000052296	Shank3	124.2172336	0.117981584	0.121269899
ENSRNOG00000012812	Sharpin	13.28431935	0.248773369	0.449050503
ENSRNOG00000011503	Shb	10.89733104	0.044313847	0.403319326
ENSRNOG00000011357	Shbg	31.45930736	-0.03915396	0.252319188
ENSRNOG00000008030	Shc2	28.93412272	0.244337707	0.287176705
ENSRNOG00000014366	Shc3	19.23705559	0.202986127	0.309033138
ENSRNOG00000037134	Shc4	11.66756202	-0.245603803	0.439884749
ENSRNOG00000028685	Shf	52.70109776	0.169942578	0.195365299
ENSRNOG00000012521	Shisa2	12.32365877	-0.015625815	0.402655044
ENSRNOG00000025670	Shisa3	12.76251874	0.308984992	0.416269616
ENSRNOG00000007369	Shisa4	133.9023756	0.120598602	0.131742288
ENSRNOG00000020667	Shisa5	37.62570901	0.063110549	0.210071222
ENSRNOG00000013372	Shisal2b	7.893415211	-0.402948701	0.495814376
ENSRNOG00000020882	Shkbp1	10.20761205	0.462998142	0.411095022
ENSRNOG00000059057	Shld2	34.13826577	-0.103147907	0.20253328
ENSRNOG00000005275	Shmt1	149.2448127	0.088416742	0.11009134
ENSRNOG00000008106	Shmt2	21.73727096	-0.085458709	0.288129609
ENSRNOG00000015339	Shoc2	9.981801978	-0.317042312	0.400952393
ENSRNOG00000019458	Shpk	26.94646826	-0.64172428	0.239048547
ENSRNOG00000014450	Shprh	83.47817155	0.250332073	0.173999117
ENSRNOG00000005433	Shq1	9.305167298	-0.629427915	0.411132853
ENSRNOG00000002208	Shroom3	35.56511976	0.525907405	0.257316536
ENSRNOG00000018350	Shtn1	17.89786343	-0.26236495	0.296803925
ENSRNOG00000031266	Siae	13.55900512	0.150912003	0.360238682
ENSRNOG00000013703	Siah2	124.5918518	0.026223178	0.129920154
ENSRNOG00000033588	Siglec15	42.03499229	0.036526894	0.21282239
ENSRNOG00000021474	Siglec5	16.07776441	0.215541753	0.422184385
ENSRNOG00000021499	Siglech	31.52391233	0.055177678	0.225772331
ENSRNOG00000001189	Sik1	67.0035783	0.242138113	0.258973944
ENSRNOG00000043498	Sik2	25.99214473	0.584355351	0.263743482
ENSRNOG00000019826	Sil1	102.5991894	-0.085657707	0.13159101
ENSRNOG00000054203	Sim2	36.19226266	0.295302604	0.218604828
ENSRNOG00000016932	Simc1	14.12658615	-0.235993663	0.335901313
ENSRNOG00000032254	Sin3a	16.00419098	0.369513583	0.325803827
ENSRNOG00000048622	Sin3b	73.00532547	0.071790954	0.165447079
ENSRNOG00000049943	Sinhcaf	8.542817441	0.005611946	0.429742564

ENSRNOG00000020726	Sipa1	182.7714573	0.040558804	0.101571015
ENSRNOG00000019791	Sipa1l2	110.5361644	0.317777361	0.139865832
ENSRNOG00000020703	Sipa1l3	18.32605216	-0.001925504	0.297110962
ENSRNOG00000051592	Sirt1	16.49348482	-0.073980521	0.324357177
ENSRNOG00000020102	Sirt2	32.80724418	0.248755011	0.238097452
ENSRNOG00000013828	Sirt3	22.94607324	-0.413509111	0.273627151
ENSRNOG00000001151	Sirt4	12.13384496	0.241311602	0.403932228
ENSRNOG00000017866	Sirt5	173.5796407	0.077288667	0.102554527
ENSRNOG00000006393	Sirt6	166.217885	0.022164782	0.126738761
ENSRNOG00000015275	Ska1	19.05845747	0.09785782	0.32362742
ENSRNOG00000025981	Ska2	18.2373598	0.315936277	0.339530208
ENSRNOG00000021847	Ska3	123.4026124	-0.01005693	0.12996203
ENSRNOG00000012228	Skap2	33.45487201	-0.169557531	0.216401749
ENSRNOG00000055340	Ski	58.67711751	-0.380223653	0.474394521
ENSRNOG00000056714	Sla	16.13328401	-0.174712746	0.332143058
ENSRNOG00000020337	Sla2	98.46750689	0.20070883	0.14347015
ENSRNOG00000024125	Slain1	190.963456	0.11424778	0.125279782
ENSRNOG00000002271	Slain2	116.0739141	-0.019400964	0.13577632
ENSRNOG00000037162	Slbp	159.4016038	-0.041146351	0.10775305
ENSRNOG00000011991	Slc10a7	20.92163895	-0.092508172	0.302230973
ENSRNOG00000014956	Slc11a1	9.002183953	-0.118256561	0.420509029
ENSRNOG00000019550	Slc11a2	155.9701102	-0.15480451	0.113430472
ENSRNOG00000015971	Slc12a2	123.1884744	0.065021488	0.158813228
ENSRNOG00000057072	Slc12a3	80.17541403	0.077415253	0.153463301
ENSRNOG00000018111	Slc12a5	46.11948529	-0.046236138	0.204202787
ENSRNOG00000005196	Slc12a6	28.24455795	-0.171912883	0.257989125
ENSRNOG00000011184	Slc13a4	18.36601505	0.031929737	0.295446945
ENSRNOG00000014870	Slc13a5	48.6053091	-0.159980899	0.204533698
ENSRNOG00000016753	Slc14a1	84.91095995	0.058891359	0.161185193
ENSRNOG00000000962	Slc15a4	121.4603582	-0.024346186	0.122617609
ENSRNOG00000019996	Slc16a1	10.10555633	0.278466493	0.444797141
ENSRNOG00000018748	Slc16a11	10.05782439	-0.095196114	0.404127479
ENSRNOG00000017072	Slc16a14	15.61797998	0.447247286	0.386605641
ENSRNOG00000002832	Slc16a2	12.75765815	-0.041762175	0.352945905
ENSRNOG00000018131	Slc16a4	33.72679412	0.36035753	0.224172227
ENSRNOG00000000245	Slc16a6	23.36559373	-0.06776938	0.267801372
ENSRNOG00000007839	Slc16a7	46.47381129	0.190185601	0.213706865
ENSRNOG00000012090	Slc16a8	36.9880479	-0.140791025	0.208080717
ENSRNOG00000016147	Slc17a6	32.97761029	0.251607485	0.223352753
ENSRNOG00000020650	Slc17a7	125.5456784	-0.155102802	0.137607356
ENSRNOG00000010275	Slc17a9	74.19782326	0.269710608	0.165796441
ENSRNOG00000011992	Slc18a1	23.04914718	0.599618472	0.383419208
ENSRNOG00000008890	Slc18a2	51.26213785	0.027056213	0.239316809
ENSRNOG00000016371	Slc18b1	83.88422749	-0.025720712	0.151699193
ENSRNOG00000002839	Slc19a2	51.80790402	0.113866939	0.214235398
ENSRNOG00000005479	Slc1a2	167.7257063	-0.009780434	0.127930899
ENSRNOG00000005248	Slc1a4	183.9640355	0.083692724	0.100013204
ENSRNOG00000015948	Slc1a5	50.55164524	-0.195464109	0.18414222
ENSRNOG00000007509	Slc1a6	19.10775626	0.149183111	0.284229058
ENSRNOG00000011644	Slc1a7	36.9481206	0.315901708	0.228640213

ENSRNOG00000019490	Slc20a2	161.7668925	0.146573928	0.106680111
ENSRNOG00000021108	Slc22a12	38.84026803	-0.159624086	0.224457174
ENSRNOG00000033051	Slc22a15	26.5823734	0.098879196	0.235513557
ENSRNOG00000008432	Slc22a5	60.88126428	-0.133791611	0.197901033
ENSRNOG00000018215	Slc22a6	58.17642207	0.187277831	0.402613867
ENSRNOG00000018086	Slc22a8	78.12326524	0.142057347	0.235169749
ENSRNOG00000008169	Slc24a2	33.58022127	0.151356914	0.257750885
ENSRNOG00000060687	Slc24a3	12.69570246	-0.07603675	0.383447453
ENSRNOG00000006729	Slc24a4	36.9492794	-0.109194849	0.211069481
ENSRNOG00000036693	Slc25a10	143.7730227	0.060968631	0.122232366
ENSRNOG00000011881	Slc25a15	29.21288118	0.270513634	0.288577743
ENSRNOG00000042731	Slc25a18	29.63054158	0.305500872	0.288814212
ENSRNOG00000003918	Slc25a19	16.28165054	0.202117947	0.341875199
ENSRNOG00000020288	Slc25a20	22.48854429	0.232794414	0.280919505
ENSRNOG00000049944	Slc25a22	24.02274691	-0.02522539	0.279997277
ENSRNOG00000047781	Slc25a23	106.6553027	-0.028014224	0.155604617
ENSRNOG00000020470	Slc25a24	17.09061732	-0.371160816	0.350063536
ENSRNOG00000012831	Slc25a26	74.03333508	0.359246217	0.16121005
ENSRNOG00000010592	Slc25a27	134.7572552	-0.166735878	0.117045239
ENSRNOG00000001052	Slc25a30	15.35972658	0.145336217	0.361346599
ENSRNOG00000004403	Slc25a32	62.30942432	0.257258115	0.202382136
ENSRNOG00000013802	Slc25a36	11.9966624	0.36083755	0.377175888
ENSRNOG00000018552	Slc25a38	10.41768299	-0.503689493	0.532375055
ENSRNOG00000022837	Slc25a40	14.79907792	0.269231662	0.321268823
ENSRNOG00000020345	Slc25a42	18.10218127	0.178229767	0.297523577
ENSRNOG00000025269	Slc25a44	20.61838346	-0.140467369	0.360105892
ENSRNOG00000030616	Slc25a45	21.11004297	-0.047563499	0.27784227
ENSRNOG00000033251	Slc25a52	28.26083085	-0.082324379	0.250980182
ENSRNOG00000030537	Slc26a11	8.812411086	0.085637152	0.415960767
ENSRNOG00000058692	Slc26a4	18.37235992	-0.406779035	0.273275576
ENSRNOG00000000512	Slc26a8	96.60059955	0.245307278	0.141316928
ENSRNOG00000018170	Slc27a1	25.46537877	-0.091576247	0.288778273
ENSRNOG00000015421	Slc27a3	50.05278864	-0.079857711	0.182972099
ENSRNOG00000019626	Slc27a5	14.42459489	-0.253404159	0.3453293
ENSRNOG00000028668	Slc28a2	10.17248183	0.643136476	0.509350157
ENSRNOG00000019752	Slc29a1	30.73763648	-0.042509043	0.269749147
ENSRNOG00000020025	Slc29a2	28.24732697	0.828719906	0.414526164
ENSRNOG00000000568	Slc29a3	90.02221194	0.138134224	0.139887228
ENSRNOG00000001115	Slc29a4	143.2281431	0.393215197	0.140542768
ENSRNOG00000025384	Slc2a10	13.43174239	0.621048251	0.412995865
ENSRNOG00000011161	Slc2a12	118.6347898	0.020068378	0.12651219
ENSRNOG00000017226	Slc2a4	9.437553127	0.770989669	0.462471496
ENSRNOG00000017693	Slc2a5	49.7970331	0.025897442	0.252968753
ENSRNOG00000005900	Slc2a6	6.241551848	-5.973434277	3.149166047
ENSRNOG00000004749	Slc30a1	63.87758738	-0.122113284	0.191191607
ENSRNOG00000002397	Slc30a10	91.78255693	0.070969212	0.141527993
ENSRNOG00000005856	Slc30a6	13.36256309	0.469661465	0.379378707
ENSRNOG00000013912	Slc30a7	15.54613381	0.041092323	0.327177336
ENSRNOG00000002246	Slc30a9	59.12572876	0.13556313	0.176763222
ENSRNOG00000014475	Slc31a1	40.40392359	0.133583435	0.202891021

ENSRNOG00000013631	Slc31a2	57.49609898	0.088964146	0.168335458
ENSRNOG00000010023	Slc33a1	103.4322203	0.235769796	0.135600748
ENSRNOG00000008908	Slc35a1	29.52085651	0.148946014	0.23637395
ENSRNOG00000002069	Slc35a5	179.7250442	0.108122494	0.116123534
ENSRNOG00000016174	Slc35b3	13.93620574	-0.119827744	0.369600455
ENSRNOG00000007732	Slc35c1	131.8095892	-0.046455998	0.12829734
ENSRNOG00000018649	Slc35c2	164.368228	-0.244911029	0.164456251
ENSRNOG00000022967	Slc35d1	47.15515361	-0.050311326	0.206440145
ENSRNOG00000027229	Slc35d2	11.82964939	0.012609279	0.40551957
ENSRNOG00000012311	Slc35d3	11.83322017	-0.322247189	0.380178508
ENSRNOG00000017075	Slc35e2b	20.21323494	0.883270919	0.377540519
ENSRNOG00000027622	Slc35e3	112.3494204	-0.455041623	0.128761568
ENSRNOG00000009014	Slc35f2	11.16698365	-0.777415026	0.36426423
ENSRNOG00000049456	Slc35f3	15.2483358	0.172991588	0.33824402
ENSRNOG00000014982	Slc35f4	66.11660971	-0.131813225	0.167361067
ENSRNOG00000003403	Slc35f5	83.46739415	0.117104538	0.16992582
ENSRNOG00000015370	Slc35g2	114.0211351	-0.003451226	0.136136901
ENSRNOG00000012356	Slc36a1	44.34262731	0.353026136	0.561282418
ENSRNOG00000011455	Slc36a4	31.35421369	-0.017324964	0.237215674
ENSRNOG00000001169	Slc37a1	48.55393766	0.071113486	0.183929889
ENSRNOG00000039390	Slc37a2	18.1230954	0.272840731	0.302610573
ENSRNOG00000005291	Slc38a1	56.72039908	-0.057510218	0.17719692
ENSRNOG00000004604	Slc38a10	16.40485321	0.464785553	0.322373087
ENSRNOG00000006653	Slc38a4	9.418192061	-0.241157289	0.421928053
ENSRNOG00000022622	Slc38a6	15.26348693	0.28481315	0.34897003
ENSRNOG00000012007	Slc38a7	90.48797085	0.029464711	0.147511515
ENSRNOG00000009851	Slc38a9	20.37266961	-0.256673364	0.279279734
ENSRNOG00000059463	Slc39a1	94.24734323	-0.371608216	0.149719509
ENSRNOG00000031213	Slc39a11	63.95131396	-0.133536256	0.20311706
ENSRNOG00000011981	Slc39a13	23.69063443	-0.257714718	0.328223444
ENSRNOG00000009832	Slc39a14	53.74037526	0.046508593	0.183787445
ENSRNOG00000045524	Slc39a3	111.0171739	-0.041477273	0.142296619
ENSRNOG00000033361	Slc39a5	185.0031774	0.019365351	0.118602687
ENSRNOG00000028703	Slc39a6	89.98856992	0.020293423	0.145465797
ENSRNOG00000000465	Slc39a7	65.43223398	1.332300258	0.844570808
ENSRNOG00000012508	Slc39a8	13.18021129	0.132229777	0.375719146
ENSRNOG00000005052	Slc39a9	170.5421289	0.156872498	0.099383238
ENSRNOG00000008713	Slc41a2	54.07147698	-0.213826086	0.17953053
ENSRNOG00000045821	Slc41a3	41.26724042	-0.052703878	0.23607343
ENSRNOG00000003835	Slc43a2	174.2422381	-0.052203438	0.114192309
ENSRNOG00000061768	Slc43a3	12.94601455	0.852819692	0.361912907
ENSRNOG00000031824	Slc44a2	147.5608861	0.044726244	0.117107692
ENSRNOG00000018759	Slc45a2	13.51877514	-0.364798134	0.360574763
ENSRNOG00000010291	Slc46a1	45.82659377	0.127661163	0.195156976
ENSRNOG00000017324	Slc46a2	83.42446061	-0.048938608	0.155191118
ENSRNOG00000000937	Slc46a3	26.21075121	0.410508923	0.274229169
ENSRNOG00000038055	Slc47a2	48.69786154	0.104068059	0.176888786
ENSRNOG00000053196	Slc48a1	88.55666056	0.049240105	0.170579428
ENSRNOG00000005307	Slc4a10	141.014367	-0.066091084	0.120988608
ENSRNOG00000004901	Slc4a1ap	150.4101363	-0.013551167	0.137864961

ENSRNOG00000003134	Slc4a4	46.65056842	-0.034583595	0.187471452
ENSRNOG00000005957	Slc4a7	13.20536204	0.346307173	0.369229085
ENSRNOG00000028879	Slc4a8	22.28040156	-0.330864833	0.26028841
ENSRNOG00000001765	Slc51a	53.11395466	-0.121613809	0.175005887
ENSRNOG00000032561	Slc52a2	51.41549442	0.007774454	0.189769367
ENSRNOG00000005132	Slc52a3	20.09432518	-0.108520577	0.311153331
ENSRNOG00000013407	Slc5a11	9.597997881	0.378104159	0.429619619
ENSRNOG00000018822	Slc5a5	19.68959046	0.377783705	0.328476281
ENSRNOG00000057628	Slc5a6	146.1619544	0.045139134	0.112821261
ENSRNOG00000010597	Slc5a7	8.947318751	-0.296126729	0.486491427
ENSRNOG00000017706	Slc66a1	16.13589527	-0.090888436	0.346837348
ENSRNOG00000059215	Slc66a2	100.8141923	0.05454551	0.15922388
ENSRNOG00000005126	Slc66a3	13.3565491	0.204869129	0.351031858
ENSRNOG00000005697	Slc6a11	18.34520982	-0.481932561	0.313532069
ENSRNOG00000012876	Slc6a13	21.3015101	0.572272838	0.278787062
ENSRNOG00000009019	Slc6a6	41.31397524	0.204745469	0.197936193
ENSRNOG00000057620	Slc6a8	37.41088509	0.119990615	0.231847771
ENSRNOG00000019484	Slc6a9	12.02688867	-0.219947343	0.413309008
ENSRNOG00000000924	Slc7a1	16.25040406	-0.007771219	0.387925386
ENSRNOG00000010938	Slc7a10	75.20711862	-0.113640138	0.151317675
ENSRNOG00000010210	Slc7a11	167.7288346	0.304523258	0.108945476
ENSRNOG00000028097	Slc7a14	148.447791	0.161059031	0.116628625
ENSRNOG00000004133	Slc7a3	25.00274418	-0.038772729	0.282743426
ENSRNOG00000001874	Slc7a4	8.106338128	-0.69859452	0.482473357
ENSRNOG00000019943	Slc7a6	13.90867789	0.677511142	0.34620098
ENSRNOG00000020049	Slc7a6os	57.10471327	0.300688301	0.22139791
ENSRNOG00000010296	Slc7a7	9.99407845	-0.171303724	0.440201129
ENSRNOG00000008479	Slc8a1	9.332115212	-0.259207566	0.442842662
ENSRNOG00000029871	Slc8a3	17.69828094	0.126872065	0.289146857
ENSRNOG00000007982	Slc9a1	156.121337	0.283103093	0.109093697
ENSRNOG00000015567	Slc9a2	10.39269138	-0.347656116	0.445805755
ENSRNOG00000015159	Slc9a3	56.46338874	-0.060727896	0.196478656
ENSRNOG00000002997	Slc9a3r2	14.06490143	-0.20202692	0.434133369
ENSRNOG00000028844	Slc9a5	57.43180667	-0.050492024	0.158922587
ENSRNOG00000004150	Slc9a7	11.18159034	0.459540353	0.38718622
ENSRNOG00000008354	Slc9a8	24.88313939	-0.165391969	0.279037958
ENSRNOG00000008554	Slc9a9	39.96522809	0.152112711	0.202961083
ENSRNOG00000023404	Slc9b2	24.49594186	-0.209950399	0.269923752
ENSRNOG00000047493	Slco1a4	138.3301267	0.518940956	0.156222292
ENSRNOG00000017976	Slco2b1	106.3687805	0.089834075	0.159631924
ENSRNOG00000032798	Slco3a1	93.90162768	-0.024421834	0.134182026
ENSRNOG00000022711	Slco4c1	13.03034945	0.007109193	0.364911058
ENSRNOG00000040279	Slf1	54.45884215	-0.190459563	0.199388848
ENSRNOG00000014482	Slf2	140.1578539	0.275824908	0.134737169
ENSRNOG00000021719	Slfn5	46.98404963	-0.13619384	0.25056972
ENSRNOG00000026065	Slit1	179.4884087	0.105620247	0.137910166
ENSRNOG00000003840	Slit2	110.6386836	-0.062584531	0.153214607
ENSRNOG00000007377	Slit3	123.0259083	0.017273595	0.141014127
ENSRNOG00000009821	Slitrk3	64.48059135	0.166490107	0.203178501
ENSRNOG00000011339	Slk	87.73925993	-0.225743545	0.17307656

ENSRNOG00000009047	Sln	9.212418848	0.558252486	0.544906657
ENSRNOG00000003822	Slu7	103.5695333	-0.302440018	0.131415195
ENSRNOG00000019369	Slx1b	158.5428601	-0.082876128	0.114440425
ENSRNOG00000007430	Slx4ip	17.16019976	-0.256553442	0.298530148
ENSRNOG00000018483	Smad1	120.3434432	-0.062412548	0.124842941
ENSRNOG00000018140	Smad2	138.1702367	0.073266215	0.116819387
ENSRNOG00000008620	Smad3	138.9516037	-0.020869346	0.131415476
ENSRNOG00000051965	Smad4	19.12379208	-0.169653307	0.365769772
ENSRNOG00000022870	Smad5	33.7906039	-0.254060035	0.224677085
ENSRNOG00000018359	Smad7	100.1095082	0.080106235	0.131849888
ENSRNOG00000011931	Smarca2	188.0343107	-0.162216063	0.148653901
ENSRNOG00000018149	Smarca5	63.82919628	-0.217359363	0.170296648
ENSRNOG00000006391	Smarcad1	17.69843252	0.381365704	0.323999591
ENSRNOG00000016503	Smarcal1	45.73855856	-0.149529075	0.195222862
ENSRNOG00000028302	Smarcb1	110.703868	0.057555925	0.135067269
ENSRNOG00000020804	Smarcc1	103.292452	0.054586021	0.144924873
ENSRNOG00000031135	Smarcc2	95.63711353	-0.099965138	0.143816919
ENSRNOG00000061572	Smarcd1	29.86485542	0.516853738	0.305018036
ENSRNOG00000010077	Smarcd3	77.08800547	0.105869425	0.165013446
ENSRNOG00000022325	Smc2	67.46158677	-0.06491464	0.16079798
ENSRNOG00000014173	Smc3	174.1493866	0.050415131	0.121331566
ENSRNOG00000010274	Smc4	10.70364439	-0.400630738	0.387582295
ENSRNOG00000030572	Smc5	94.85824203	0.414013085	0.149114068
ENSRNOG00000004908	Smc6	167.8613723	0.136175056	0.143124941
ENSRNOG00000014319	Smchd1	30.0131421	0.436213933	0.229405037
ENSRNOG00000011162	Smco4	32.52409225	-0.016963383	0.24770643
ENSRNOG00000005165	Smcr8	8.803221776	-0.211114066	0.458457869
ENSRNOG00000047386	Smg1	33.88342599	0.270459921	0.234159959
ENSRNOG00000019590	Smg5	21.14667478	0.309251844	0.301235987
ENSRNOG00000003041	Smg6	17.04737848	0.308504388	0.366043408
ENSRNOG00000027962	Smg7	19.52910634	-0.21547872	0.283622499
ENSRNOG00000019596	Smg9	152.1974452	-0.023207807	0.107433406
ENSRNOG00000039877	Smim11a	37.58636717	0.115955955	0.227031756
ENSRNOG00000049689	Smim18	52.47553944	-0.142005195	0.237254812
ENSRNOG00000036971	Smim26	20.45379922	0.159211677	0.29203194
ENSRNOG00000019536	Smim3	116.6383296	-0.094679429	0.142889712
ENSRNOG00000023035	Smim8	62.81372232	-0.176018836	0.180561224
ENSRNOG00000018067	Smn1	163.4409295	-0.025474308	0.103092518
ENSRNOG00000014833	Smndc1	126.1639598	0.069142775	0.125412011
ENSRNOG00000008332	Smo	38.7386229	0.163924375	0.20332637
ENSRNOG00000021255	Smox	181.5775395	-0.019152414	0.104313507
ENSRNOG00000017977	Smpd1	15.74139912	0.151737057	0.406934202
ENSRNOG00000000257	Smpd3	79.98418383	0.04819554	0.175432565
ENSRNOG00000000815	Smpdl3a	104.4001596	-0.233409901	0.151795369
ENSRNOG00000042326	Smpdl3b	8.927726543	-0.006235055	0.459862653
ENSRNOG00000007516	Smtnl1	48.54579956	-0.194046312	0.46484313
ENSRNOG00000036842	Smug1	54.54765736	0.008123294	0.197106133
ENSRNOG00000000999	Smurf1	138.3022406	0.048202832	0.179133775
ENSRNOG00000014623	Smurf2	16.06710919	-0.163133213	0.300344727
ENSRNOG00000026783	Smyd4	9.242046557	0.79320138	0.427558463

ENSRNOG00000015589	Smyd5	32.64545119	-0.059089687	0.823597455
ENSRNOG00000009594	Snai1	15.21718862	0.454691605	0.334385313
ENSRNOG00000001867	Snap29	146.7888002	-0.005398846	0.113052101
ENSRNOG00000009296	Snapc1	47.47955921	0.213007708	0.188616877
ENSRNOG00000010825	Snapc3	76.16559395	0.015179565	0.158086375
ENSRNOG00000018845	Snapc4	23.530737	-0.082934406	0.260819846
ENSRNOG00000010156	Snapc5	45.79445862	-0.038286781	0.201716826
ENSRNOG00000013356	Snapin	65.53738323	-0.477764038	0.270860692
ENSRNOG00000018254	Sncaip	20.32834883	0.188244552	0.299003725
ENSRNOG00000058006	Sncg	61.07106312	-1.338424826	0.586994422
ENSRNOG00000006500	Snf8	130.3344638	0.271704049	0.134561601
ENSRNOG00000058739	Snn	14.62136635	0.444566749	0.340292259
ENSRNOG00000059077	SNORA70	8.357221118	-0.491386078	0.401978215
ENSRNOG00000053071	SNORA70	34.31520927	0.06401027	0.223735716
ENSRNOG00000060059	SNORA70	10.32027117	0.065193313	0.375077573
ENSRNOG00000058113	SNORA71	10.2000145	0.623990817	0.452641123
ENSRNOG00000053202	SNORD116	63.18560123	0.236205991	0.166720536
ENSRNOG00000054081	Snord12	19.19728928	-0.185056339	0.352573355
ENSRNOG00000061186	Snord123	12.85167385	0.21437322	0.395266534
ENSRNOG00000057762	SNORD14	33.65613922	0.106630853	0.232223063
ENSRNOG00000009588	Snph	27.47785262	0.053320016	0.253576751
ENSRNOG00000012157	Snrnp200	55.52081092	0.233942251	0.235048665
ENSRNOG00000001060	Snrnp35	13.26319397	0.052900112	0.411090644
ENSRNOG00000012219	Snrnp40	104.5267519	0.093554946	0.139425699
ENSRNOG00000013756	Snrnp48	64.7398962	0.073728383	0.180079595
ENSRNOG00000001501	Snrpa	82.88781209	-0.11373683	0.145472006
ENSRNOG00000011932	Snrpa1	129.4502932	0.052381868	0.139319873
ENSRNOG00000004967	Snrpb2	184.7267256	-0.005304726	0.098406168
ENSRNOG00000000493	Snrpc	29.77580468	0.019195263	0.223782767
ENSRNOG00000048758	Snrpd2l	24.76121791	-0.020873434	0.288423068
ENSRNOG00000050410	Snrpd3	159.5866019	-0.121926481	0.108051448
ENSRNOG00000031127	Snrpel1	114.7757833	-0.191031928	0.126233266
ENSRNOG00000032232	Snrpg	162.451605	0.012047349	0.119681079
ENSRNOG00000016062	Snta1	19.83464832	0.000409618	0.289366931
ENSRNOG00000004821	Sntb1	23.78744157	-0.60993367	0.276575176
ENSRNOG00000020344	Sntb2	21.80056317	-0.17956405	0.292220884
ENSRNOG00000007294	Sntg1	13.11820995	-0.018969122	0.347012638
ENSRNOG00000004928	Sntg2	15.1416845	-0.097529435	0.351038418
ENSRNOG00000006762	Snu13	9.029975992	-0.514965773	0.421070971
ENSRNOG00000025154	Snu13	28.65125158	-0.04450227	0.236080906
ENSRNOG00000005426	Snupn	66.69858083	-0.012118161	0.17796597
ENSRNOG00000037998	Snw1	23.91982059	0.354646384	0.322077415
ENSRNOG00000011944	Snx10	67.31733682	-0.031457396	0.1788779
ENSRNOG00000008642	Snx11	186.2550407	-0.213567943	0.18692512
ENSRNOG00000004186	Snx13	16.9835876	0.634459804	0.333493222
ENSRNOG00000011348	Snx14	53.91735555	-0.181025391	0.195834696
ENSRNOG00000021007	Snx15	15.80955698	0.334709546	0.336613806
ENSRNOG00000009953	Snx16	161.6961756	-0.064607807	0.121523165
ENSRNOG00000026884	Snx17	38.06044107	-0.249404608	0.594580766
ENSRNOG00000010971	Snx18	85.98523418	-0.025491924	0.144908943

ENSRNOG00000014719	Snx19	140.1685417	-0.09798846	0.113139524
ENSRNOG00000014202	Snx20	26.32944799	0.053131399	0.246046038
ENSRNOG00000017488	Snx24	175.4352931	-0.031639539	0.102399565
ENSRNOG00000011116	Snx25	75.24510766	-0.134435936	0.152018968
ENSRNOG00000017132	Snx30	9.20182954	-0.720971574	0.383709735
ENSRNOG00000026527	Snx32	146.1065217	-0.024141981	0.116676867
ENSRNOG00000017382	Snx33	35.34033439	-0.333884639	0.203146996
ENSRNOG00000001786	Snx4	174.016077	0.108119259	0.122306216
ENSRNOG00000005249	Snx6	45.41708373	0.059514472	0.192785116
ENSRNOG00000017077	Snx7	121.0595598	-0.204061872	0.137681707
ENSRNOG00000001258	Snx8	11.93833505	-0.103390898	0.440757956
ENSRNOG00000046874	Snx9	8.925048932	-0.180247311	0.397102343
ENSRNOG00000000316	Sobp	126.6788404	0.074190184	0.137318229
ENSRNOG00000002568	Socs1	12.19941976	0.089121897	0.39627978
ENSRNOG00000008965	Socs2	107.2992566	-0.143589312	0.155676726
ENSRNOG00000038212	Socs6	30.56764203	-0.042013544	0.268706431
ENSRNOG00000002115	Sod1	81.82554483	-0.016715812	0.152018343
ENSRNOG00000003869	Sod3	148.25606	0.116760642	0.118768058
ENSRNOG00000053240	Soga1	9.946503041	0.091595704	0.427417946
ENSRNOG00000012324	Soga3	58.86669176	-0.270566716	0.194141806
ENSRNOG00000002021	Son	146.8500385	-0.073164296	0.124746945
ENSRNOG00000015658	Sorbs1	45.94098977	0.196177802	0.216595297
ENSRNOG00000008829	Sorbs3	35.08633856	0.168735153	0.233376043
ENSRNOG00000011313	Sorcs1	40.30849682	0.148245986	0.204582196
ENSRNOG00000028832	Sorcs3	188.5891777	-0.086419312	0.138569692
ENSRNOG00000017291	Sord	152.0861015	-0.108133625	0.120887511
ENSRNOG00000007106	Sos1	124.0410595	0.06783825	0.11811325
ENSRNOG00000004826	Sos2	142.4511598	0.020082312	0.112349904
ENSRNOG00000060061	Sowaha	17.70803685	0.672776331	0.357051934
ENSRNOG00000011305	Sox10	68.44166566	0.051283071	0.182888202
ENSRNOG00000027357	Sox17	34.71195007	0.170426761	0.238198911
ENSRNOG00000016248	Sox18	23.81979125	-0.295848411	0.270614081
ENSRNOG00000027869	Sox5	58.40623795	0.109244252	0.181573682
ENSRNOG00000012049	Sox7	10.49537939	-0.310079308	0.3855481
ENSRNOG00000014084	Sp1	59.4696671	0.120508726	0.174474987
ENSRNOG00000033747	Sp110	17.6192245	0.156678563	0.338728351
ENSRNOG00000010492	Sp2	92.15465109	-0.105409936	0.144332641
ENSRNOG00000060479	Sp3	62.70643792	-0.2923238	0.160778157
ENSRNOG00000010934	Spa17	9.881300781	-0.470504779	0.386589994
ENSRNOG00000039076	Spaca6	44.56751506	-0.115499433	0.213249576
ENSRNOG00000012697	Spaca9	132.5962131	0.094877384	0.116948446
ENSRNOG00000010078	Spag1	12.16834199	-0.537966953	0.362813062
ENSRNOG00000004246	Spag7	38.6514844	0.296679698	0.259678758
ENSRNOG00000014155	Spart	7.904775107	-0.004530047	0.564209528
ENSRNOG00000027136	Spast	148.3885604	-0.045326761	0.106057381
ENSRNOG00000002120	Spata18	126.0831671	-0.184126722	0.132313651
ENSRNOG00000009207	Spata2	20.09412088	0.006538937	0.276032711
ENSRNOG00000016167	Spata2L	24.55318086	0.095998187	0.385443351
ENSRNOG00000038239	Spata33	41.49931984	0.124915441	0.213212254
ENSRNOG00000017462	Spata5	37.62560616	0.118995411	0.204298745

ENSRNOG00000000169	Spata5l1	18.83628684	0.783362982	0.36768729
ENSRNOG00000007568	Spata6	28.92822982	-0.06131221	0.236893153
ENSRNOG00000015137	Spata6l	24.17987589	0.299110908	0.293006934
ENSRNOG00000003955	Spata7	41.84725582	-0.024394517	0.198250647
ENSRNOG00000012770	Spata9	46.77170113	0.167653741	0.217297975
ENSRNOG00000019963	Spats1	22.53257427	0.233209958	0.26480247
ENSRNOG00000016012	Spats2l	86.53852962	0.124450115	0.158363568
ENSRNOG00000029862	Spc24	14.20937181	0.253469546	0.338573696
ENSRNOG00000002903	Specc1	136.6845286	0.068720759	0.115427909
ENSRNOG00000001303	Specc1l	45.807006	0.498520075	0.193805173
ENSRNOG00000036648	Spef1l	17.6066816	0.555871263	0.335214177
ENSRNOG00000058275	Spef2	10.98071781	0.504709307	0.403631035
ENSRNOG00000033556	Spen	60.64261492	-0.149978949	0.211738553
ENSRNOG00000021954	Spg11	151.301709	0.030158463	0.121832549
ENSRNOG00000015150	Spg7	67.26917278	0.087031883	0.167490764
ENSRNOG00000021032	Sphk2	11.96882336	-0.376751001	0.51903893
ENSRNOG00000012172	Spi1	16.52946193	-0.88078958	0.363219893
ENSRNOG00000039024	Spice1	22.60893291	0.280682549	0.276407231
ENSRNOG00000037851	Spidr	10.33065687	-0.101807256	0.445501789
ENSRNOG00000031286	Spin2a	12.81419459	0.235327426	0.39091133
ENSRNOG00000025061	Spindoc	67.37408827	0.01475949	0.158366833
ENSRNOG00000012811	Spint1	21.30089429	0.095671691	0.334131626
ENSRNOG00000020636	Spint2	151.6409945	-0.363277488	0.124584502
ENSRNOG00000016920	Spire2	18.64269104	-0.465457264	0.324212035
ENSRNOG00000017621	Spns1	164.212743	-0.504997844	0.491253728
ENSRNOG00000057040	Spns2	12.82859986	0.425549332	0.362526125
ENSRNOG00000061544	Spock2	89.49536323	0.198348096	0.138563325
ENSRNOG00000029903	Spock3	60.59015102	-0.020327846	0.162383991
ENSRNOG00000006033	Spon2	9.167847399	0.223276578	0.408921997
ENSRNOG00000004686	Spop	24.89400139	0.046883145	0.248483176
ENSRNOG00000025711	Spout1	68.17908805	-0.183967301	0.234015034
ENSRNOG00000011652	Sppl2a	113.574055	-0.088649898	0.120875897
ENSRNOG00000057881	Sppl2b	161.0085522	0.231559138	0.115009308
ENSRNOG00000015455	Spr	104.2408292	-0.149720855	0.12767775
ENSRNOG00000004888	Spred2	38.10637965	0.23778353	0.315667873
ENSRNOG00000051915	Spred3	18.9553491	-0.249832321	0.292271541
ENSRNOG00000021330	Sprtn	29.59109758	0.278003013	0.25298718
ENSRNOG00000025371	Spry1	32.71345542	-0.280734093	0.217509618
ENSRNOG00000060153	Spry3	36.67133845	0.188157978	0.204370353
ENSRNOG00000013851	Spry4	28.51983121	0.299630566	0.311478356
ENSRNOG00000010793	Spryd3	151.2029018	-0.136334056	0.116920966
ENSRNOG00000017212	Spsb1	139.0857478	-0.073907821	0.137480557
ENSRNOG00000015300	Spsb3	12.56696033	0.022169766	0.361037612
ENSRNOG00000012862	Spsb4	15.6274558	0.294086897	0.371963194
ENSRNOG00000015396	Sptan1	159.9114679	0.250559995	0.13799286
ENSRNOG00000006911	Sptb	60.58085506	0.136038756	0.178375506
ENSRNOG00000005434	Sptbn1	161.1801744	-0.043000113	0.147852907
ENSRNOG00000058842	Sptbn2	99.97212189	0.147948758	0.153739387
ENSRNOG00000055371	Sptbn4	54.31273561	-0.375952478	0.198894632
ENSRNOG00000012210	Sptlc2	41.56062196	0.167133972	0.208224307

ENSRNOG00000004292	Sptssa	180.1646608	0.070299731	0.103542202
ENSRNOG00000009388	Sptssb	34.94099551	0.075182173	0.276079442
ENSRNOG00000000172	Sqor	15.76090428	0.062872989	0.331602505
ENSRNOG00000014720	Srbd1	18.07102082	0.295159216	0.33338441
ENSRNOG00000050282	Srcap	89.13912739	0.304832596	0.150458529
ENSRNOG00000011475	Srcin1	91.97792037	0.094512716	0.150638377
ENSRNOG00000017601	Srd5a1	91.06566194	-0.079059839	0.152375757
ENSRNOG00000002216	Srd5a3	112.2476634	-0.049270239	0.127213184
ENSRNOG00000003463	Srebf1	8.21134003	0.329537068	0.472610617
ENSRNOG00000032735	Srek1	57.26026008	0.058473525	0.210982428
ENSRNOG00000047506	Srek1ip1	109.1941973	-0.145459308	0.131524971
ENSRNOG00000014808	Srfbp1	84.24013517	-0.084261792	0.140876104
ENSRNOG00000004603	Srgap1	27.14371184	0.24170238	0.256295241
ENSRNOG00000006733	Srgap2	79.07061647	0.109782766	0.149307049
ENSRNOG00000006509	Srgap3	45.46180934	0.126102719	0.241886816
ENSRNOG00000000394	Srgn	79.64105145	0.000242057	0.185344726
ENSRNOG00000032776	Srp54a	93.76129799	0.118695459	0.132954663
ENSRNOG00000002100	Srp72	171.2220196	-0.176457003	0.104456343
ENSRNOG00000000511	Srpk1	128.5265405	0.121013355	0.119976259
ENSRNOG00000010543	Srpra	40.97493573	-0.074838756	0.210325738
ENSRNOG00000002991	Srr	9.375591846	-0.243793536	0.42767548
ENSRNOG00000001141	Srrm4	70.31450822	-0.086553444	0.190210658
ENSRNOG00000007992	Srsf10	49.01905795	-0.046579402	0.221871726
ENSRNOG00000000248	Srsf2	60.25495704	-0.442041135	0.523506322
ENSRNOG00000005513	Srsf5	18.30251068	0.051558872	0.302613259
ENSRNOG00000027360	Srsf7	106.4624083	0.288702166	0.155843712
ENSRNOG00000001163	Srsf9	8.88805724	0.401621201	0.571006086
ENSRNOG00000016800	Ss18	126.323194	0.066712454	0.124451595
ENSRNOG00000060010	Ss18l1	12.34959391	0.192590473	0.351027869
ENSRNOG00000012100	Ssbp1	39.61884916	-0.269248446	0.238948017
ENSRNOG00000016213	Ssbp2	111.1422652	-0.077309952	0.162581639
ENSRNOG00000007920	Ssbp3	60.92299594	0.034684201	0.179445912
ENSRNOG00000019698	Ssbp4	15.12209586	0.033154314	0.311635015
ENSRNOG00000016687	Ssc5d	10.40100128	0.452542764	0.443647504
ENSRNOG00000000695	Ssh1	43.97877851	0.783935362	0.222151918
ENSRNOG00000014285	Ssh2	30.36892928	0.309712374	0.310375474
ENSRNOG00000018878	Ssh3	104.4846891	0.249660355	0.150191499
ENSRNOG00000001807	Sspn	53.76565861	0.012594833	0.185354809
ENSRNOG00000011148	Ssr3	109.0102959	-0.4798554	0.160810817
ENSRNOG00000053172	Ssr4	10.04738122	0.044154248	0.43328076
ENSRNOG00000008825	Ssrp1	52.38646285	-0.178984326	0.226984548
ENSRNOG00000048145	Sstr1	125.5692056	0.137046849	0.146769216
ENSRNOG00000002793	Sstr2	131.0287992	0.04767059	0.285885112
ENSRNOG00000007332	Sstr3	69.66139582	0.405629232	0.170791755
ENSRNOG00000004641	Sstr4	22.83955088	0.006223835	0.290272184
ENSRNOG00000017829	Ssu72	13.37514146	0.297659228	0.377006738
ENSRNOG00000027850	Ssx1	12.23435583	0.31498847	0.39283393
ENSRNOG00000006200	St18	8.931141648	-0.340193311	0.486770598
ENSRNOG00000008209	St3gal1	80.65778056	0.111033654	0.177245107
ENSRNOG00000019843	St3gal3	10.34608901	0.291242607	0.431970708

ENSRNOG00000001653	St3gal6	99.60492534	0.052887151	0.141264965
ENSRNOG00000001823	St6gal1	70.32905668	0.344982006	0.16912054
ENSRNOG00000046515	St6gal2	8.397208817	0.151015143	0.495230698
ENSRNOG00000012083	St6galnac2	39.08584013	-0.651967056	0.196180656
ENSRNOG00000056894	St6galnac3	35.29842088	0.080606182	0.227382415
ENSRNOG00000048870	St6galnac4	114.072717	0.286191485	0.161781162
ENSRNOG00000049676	St6galnac5	160.1072303	-0.168724562	0.124839558
ENSRNOG00000056243	ST7	108.1904916	-0.025184858	0.129860312
ENSRNOG00000014002	St7l	100.8899112	0.122526696	0.133305535
ENSRNOG00000018305	St8sia3	31.19084605	0.473039867	0.322527232
ENSRNOG00000022691	St8sia5	132.6500769	0.198047198	0.125032829
ENSRNOG00000015389	Stag1	42.79428344	0.024903994	0.208502257
ENSRNOG00000029885	Stag2	93.12478885	-0.135073214	0.139661736
ENSRNOG00000027447	Stam2	20.15647554	0.075565373	0.309301977
ENSRNOG00000012227	Stambp	186.2720467	-0.185422125	0.115584904
ENSRNOG00000050224	Stambpl1	15.36298901	-0.119591879	0.34861237
ENSRNOG00000015052	Star	50.37770043	-0.106812072	0.19055572
ENSRNOG00000001090	Stard13	16.11945131	0.185104939	0.350308686
ENSRNOG00000042044	Stard3	9.011855223	0.681585096	0.446169702
ENSRNOG00000020468	Stard4	19.33985944	0.655172886	0.325094089
ENSRNOG00000026324	Stard6	18.60853003	0.309663707	0.310901906
ENSRNOG00000033883	Stard8	71.98545628	-0.01442708	0.145901644
ENSRNOG00000010050	Stard9	43.93336083	0.174086179	0.251979718
ENSRNOG00000031081	Stat2	11.92556512	-0.30919344	0.406382416
ENSRNOG00000019742	Stat3	16.37442501	0.276695783	0.316170401
ENSRNOG00000014079	Stat4	28.78557189	-0.112762468	0.267547209
ENSRNOG00000019075	Stat5b	31.90442344	-0.549881171	0.255083862
ENSRNOG00000007781	Stau1	23.57913978	-0.486315459	0.295500287
ENSRNOG00000015075	Stc1	16.82702799	0.118140053	0.339107219
ENSRNOG00000006076	Steap2	35.57463878	-0.352632767	0.221805066
ENSRNOG00000049471	Steap3	71.28293854	0.081700393	0.184134953
ENSRNOG00000012564	Steep1	101.8494598	-0.207786985	0.144209732
ENSRNOG00000002956	Stim2	163.0292567	-0.034196884	0.115070664
ENSRNOG00000017051	Stimate	33.44763708	0.212694788	0.259077965
ENSRNOG00000021164	Stip1	53.01922549	0.292969493	0.234830952
ENSRNOG00000020107	Stk11ip	95.21052457	0.018200036	0.138420459
ENSRNOG00000012502	Stk17b	42.63096188	0.262551113	0.234267598
ENSRNOG00000011511	Stk24	16.31260278	-0.091362638	0.351055739
ENSRNOG00000018181	Stk25	16.5771754	-0.190758586	0.333344436
ENSRNOG00000011278	Stk3	29.42718664	-0.33088398	0.297669742
ENSRNOG00000027066	Stk32a	123.5655997	-0.102211991	0.115911986
ENSRNOG00000006002	Stk35	35.50332106	0.04531558	0.241054863
ENSRNOG00000000519	Stk38	97.72190039	0.15424874	0.139372539
ENSRNOG00000001828	Stk38l	63.43079035	0.129449086	0.163171268
ENSRNOG00000013529	Stk4	64.24351475	-0.374794974	0.219318232
ENSRNOG00000020376	Stn1	56.64687969	-0.003067495	0.172917948
ENSRNOG00000019147	Stom	23.79244489	0.656963132	0.325680699
ENSRNOG00000004458	Ston2	69.24271011	-0.267546011	0.184613524
ENSRNOG00000009590	Stox2	30.84323891	-0.01610874	0.226140645
ENSRNOG00000008637	Strada	24.27629306	-0.104412171	0.244724958

ENSRNOG00000010728	Stradb	10.15780864	0.558770158	0.425985938
ENSRNOG00000007134	Strap	51.73413598	-0.014578189	0.175064642
ENSRNOG00000004806	Strn	27.476555	-0.012938093	0.236363828
ENSRNOG00000060335	Strn3	31.02911337	-0.01441505	0.23474704
ENSRNOG00000016145	Strn4	30.1485807	0.570006549	0.289181228
ENSRNOG00000032487	Sts	51.12700044	-0.083666581	0.188577973
ENSRNOG00000031896	Stt3a	173.1222682	-0.128437118	0.141172064
ENSRNOG00000011922	Stt3b	155.0734091	0.004499769	0.118342148
ENSRNOG00000005281	Stx16	167.6043468	0.010407936	0.114795848
ENSRNOG00000006139	Stx18	37.32403655	-0.016977373	0.23493126
ENSRNOG00000029165	Stx1a	99.97105661	0.19932075	0.163696415
ENSRNOG00000000936	Stx2	63.54832463	0.202356599	0.168772521
ENSRNOG00000021013	Stx3	38.2943911	0.157021197	0.257348985
ENSRNOG00000019302	Stx4	119.8986686	0.085587148	0.12437265
ENSRNOG00000003849	Stx8	130.356182	-0.280007414	0.119776492
ENSRNOG00000020392	Stxbp3	138.9406752	0.104310474	0.126926517
ENSRNOG00000032768	Stxbp4	61.13407367	0.074982024	0.17836556
ENSRNOG00000002496	Stxbp5l	28.42650921	-0.17834904	0.377911782
ENSRNOG00000004198	Stxbp6	13.10459155	0.06063598	0.34134995
ENSRNOG00000007956	Styx	24.34264898	0.074619783	0.254714631
ENSRNOG00000050563	Sub1	106.9367125	-0.148214863	0.134732652
ENSRNOG00000017481	Sucla2	83.36398393	0.10097802	0.170091056
ENSRNOG00000026542	Suco	14.92397893	-0.549429057	0.339740205
ENSRNOG00000019807	Sufu	11.27694028	-0.172662794	0.39219534
ENSRNOG00000052111	Sugp1	59.29254067	-0.100028706	0.211436804
ENSRNOG00000009037	Sulf1	52.32578293	-0.046105255	0.240932883
ENSRNOG00000006052	Sulf2	118.8339435	0.248865207	0.139821573
ENSRNOG00000001960	Sult1d1	14.74117811	0.573258915	0.366015479
ENSRNOG00000047986	Sult2a2	28.70330754	0.358964024	0.261100659
ENSRNOG00000021046	Sult2b1	15.28400433	0.310891037	0.331642252
ENSRNOG00000006813	Sumf1	168.3061625	-0.053307582	0.122561591
ENSRNOG00000000922	Sumf2	15.70412001	0.227583115	0.318546134
ENSRNOG00000001299	Sun1	85.2212562	0.16514417	0.185841311
ENSRNOG00000015177	Sun2	134.6109	-0.190515448	0.127699345
ENSRNOG00000005987	Suox	112.3049147	-0.097254249	0.140475339
ENSRNOG00000011953	Supt16h	26.59841775	-0.131597527	0.244886249
ENSRNOG00000059480	Supt20h	16.38610544	-0.060035634	0.35497331
ENSRNOG00000020153	Supt3h	18.96716242	0.089316242	0.276101892
ENSRNOG00000007845	Supt4h1	171.0156042	0.015123219	0.118914742
ENSRNOG00000032034	Supt5h	88.39814333	0.296135501	0.136952086
ENSRNOG00000012240	Supt6h	40.52917755	0.30582565	0.259112474
ENSRNOG00000004927	Supt7l	63.59392514	-0.044283931	0.164284123
ENSRNOG00000000392	Supv3l1	47.93456952	0.168137445	0.237794231
ENSRNOG00000060005	Surf4	117.7707949	0.147592036	0.168895782
ENSRNOG00000005031	Surf6	132.9803442	0.048644268	0.130506433
ENSRNOG00000033389	Susd2	33.85986571	0.17673596	0.244481567
ENSRNOG00000016525	Susd3	27.85429446	0.026086971	0.280198502
ENSRNOG00000045941	Susd6	18.38675165	0.28412776	0.312722424
ENSRNOG00000015585	Suv39h2	18.84780671	-0.058026274	0.330918539
ENSRNOG00000058663	Suz12	14.69894504	-0.144463791	0.368563437

ENSRNOG00000021182	Sv2a	180.3938939	-0.254991942	0.152958572
ENSRNOG00000007404	Svbp	10.43875847	0.653802316	0.423713018
ENSRNOG00000018110	Svil	24.70011858	0.096954352	0.253529567
ENSRNOG00000037137	Svip	71.68805511	-0.113139111	0.166826699
ENSRNOG00000000693	Svop	163.9440378	-0.01939589	0.158007979
ENSRNOG00000009910	Swap70	57.35371908	0.027466163	0.162429112
ENSRNOG00000032258	Swt1	40.47775133	-0.113953471	0.202197551
ENSRNOG00000004304	Syap1	148.7308118	-0.207488161	0.122041834
ENSRNOG00000031097	Syce1l	151.1310056	0.011395846	0.139098123
ENSRNOG00000005270	Sycp3	69.64023184	0.335091612	0.160834452
ENSRNOG00000060597	Syf2	190.4040161	-0.073195782	0.107058061
ENSRNOG00000012160	Syk	11.43488078	0.359914227	0.382644746
ENSRNOG00000010365	Syn1	65.97479806	-0.061710872	0.174637649
ENSRNOG00000008118	Sync	39.72156997	-0.175170407	0.198303435
ENSRNOG00000000204	Syncrip	65.92913139	-0.121139193	0.192745273
ENSRNOG00000027645	Syndig1l	10.39314329	0.302888499	0.419714007
ENSRNOG00000005323	Syne2	80.50735904	0.104902448	0.149547439
ENSRNOG00000000483	Syngap1	34.44258285	-0.037414323	0.285115852
ENSRNOG00000012620	Syngr3	145.9616271	0.034636849	0.568439659
ENSRNOG00000002051	Synj1	114.4560043	0.334312134	0.153454655
ENSRNOG00000017114	Synj2	149.1712731	0.342082131	0.120437196
ENSRNOG00000006399	Synj2bp	149.1832889	0.322213225	0.13825874
ENSRNOG00000008203	Synpr	33.87727001	-0.00683742	0.22129798
ENSRNOG00000059720	Syp	145.5651293	-0.018689987	0.11240852
ENSRNOG00000009856	Sypl1	8.455647806	0.058946675	0.451932359
ENSRNOG00000014296	Syt10	12.24968323	-0.097307116	0.386199761
ENSRNOG00000046139	Syt14	7.932994911	-0.03509714	0.532477092
ENSRNOG00000004756	Syt2	9.268872221	0.192221079	0.3964545
ENSRNOG00000019163	Syt6	108.3974731	-0.320051513	0.189555755
ENSRNOG00000026432	Syt7	67.66874487	0.023667081	0.182396289
ENSRNOG00000018321	Sytl3	77.20910459	-0.073078712	0.153758218
ENSRNOG00000009183	Szrd1	14.75132318	0.169866184	0.355105986
ENSRNOG00000028659	Szt2	9.01556575	0.38467537	0.446838657
ENSRNOG00000017285	Tab1	113.1864153	0.216207426	0.159080514
ENSRNOG00000016054	Tab2	58.12289256	0.299260047	0.172425738
ENSRNOG00000004229	Tac3	123.7649833	-0.029634332	0.13114363
ENSRNOG00000017259	Tacc3	9.943390594	-0.041391545	0.387952222
ENSRNOG00000008405	Taco1	30.57655681	-0.324312249	0.249115539
ENSRNOG00000050658	Tacr2	11.70014261	-0.002852925	0.382918428
ENSRNOG00000009372	Tacr3	35.85430678	0.311739636	0.260780527
ENSRNOG00000002757	Tada2a	66.46336233	0.22508801	0.156205058
ENSRNOG00000027326	Tada2b	34.92327482	0.308012306	0.249016054
ENSRNOG00000002565	Taf1	18.60154102	0.055608556	0.298627857
ENSRNOG00000024601	Taf11	80.64380933	0.046657245	0.161726658
ENSRNOG00000048288	Taf12	59.60043249	-0.142211618	0.169531699
ENSRNOG00000020315	Taf13	97.62948269	0.045832991	0.139596287
ENSRNOG00000058393	Taf15	43.81754322	0.440274967	0.206786345
ENSRNOG00000061415	Taf1b	72.66965249	0.041303432	0.172290157
ENSRNOG00000010921	Taf1d	8.508145121	0.313010009	0.494143477
ENSRNOG00000034022	Taf2	163.4898596	-0.028442123	0.10480334

ENSRNOG00000054497	Taf4	9.968565905	-0.257086858	0.425344416
ENSRNOG00000026023	Taf4b	27.20374378	-0.119895444	0.289940536
ENSRNOG00000020274	Taf5	126.6098489	-0.304117251	0.516187375
ENSRNOG00000018027	Taf5l	123.7354528	0.062618783	0.13514949
ENSRNOG00000001355	Taf6	142.3800062	0.072709089	0.114191933
ENSRNOG00000019419	Taf6l	12.16607377	0.198739015	0.401362399
ENSRNOG00000026315	Taf7l	22.3196468	0.31164804	0.295959946
ENSRNOG00000015249	Taf8	41.69546004	0.522917229	0.203272252
ENSRNOG00000039848	Taf9	177.1739575	0.064804401	0.101521961
ENSRNOG00000025051	Tal1	9.600637257	0.693433988	0.417621801
ENSRNOG00000007874	Tamm41	121.3533692	0.162315563	0.144422585
ENSRNOG00000025394	Tanc1	30.46969108	0.103580263	0.269101384
ENSRNOG00000052840	Tanc2	46.89390329	0.008684758	0.266631681
ENSRNOG00000022524	Tango6	19.7126921	0.205310699	0.296118401
ENSRNOG00000008859	Tank	87.94401375	0.02186488	0.140088481
ENSRNOG00000019964	Taok2	45.32150207	-0.024866307	0.191776988
ENSRNOG00000001138	Taok3	139.7941604	-0.034043833	0.1389401
ENSRNOG00000003174	Tapt1	123.3269449	-0.06783251	0.135071225
ENSRNOG00000053574	Tarbp1	87.91923151	0.174641065	0.144983994
ENSRNOG00000057194	Tars2	31.94892924	-0.027268141	0.249524533
ENSRNOG00000009708	Tas1r1	23.64807223	-0.070345951	0.34050139
ENSRNOG00000014750	Tasor	75.66040935	0.06089747	0.156765909
ENSRNOG00000018011	Tasor2	58.28269425	-0.147620954	0.184688069
ENSRNOG00000004493	Tasp1	91.32109913	0.066932324	0.164603897
ENSRNOG00000047198	Tatdn1	27.91931018	0.479289363	0.250813924
ENSRNOG00000042482	Tatdn2	92.37662757	0.072090281	0.144126831
ENSRNOG00000053023	Taz	180.5152697	-0.114716819	0.109125455
ENSRNOG00000002180	Tbc1d1	80.09999103	0.265850349	0.150860186
ENSRNOG00000017349	Tbc1d10b	110.3882512	-0.163152096	0.681949274
ENSRNOG00000036598	Tbc1d12	19.74466607	0.44138232	0.334901821
ENSRNOG00000015970	Tbc1d13	11.05504882	-0.115577177	0.408028768
ENSRNOG00000006400	Tbc1d14	161.41668	-0.062622076	0.132462187
ENSRNOG00000003889	Tbc1d15	105.9911335	0.14872595	0.126480779
ENSRNOG00000003003	Tbc1d19	50.28278168	-0.189140569	0.186974212
ENSRNOG00000023348	Tbc1d2	18.54290181	0.226094855	0.310429839
ENSRNOG00000005766	Tbc1d20	65.54372357	0.066136445	0.179230362
ENSRNOG00000017057	Tbc1d22a	10.05925997	0.063231613	0.38010568
ENSRNOG00000059458	Tbc1d22b	16.77243054	-0.359444252	0.305694509
ENSRNOG00000001642	Tbc1d23	67.06266869	-0.076689989	0.170566802
ENSRNOG00000052204	Tbc1d24	10.40170077	0.090661013	0.398733672
ENSRNOG00000005303	Tbc1d25	95.04153111	0.055415154	0.157145447
ENSRNOG00000014543	Tbc1d2b	12.79073627	0.467225447	0.364160089
ENSRNOG00000023951	Tbc1d30	24.60706899	0.119206845	0.304399361
ENSRNOG00000006103	Tbc1d31	24.31256201	0.152703409	0.292623682
ENSRNOG00000000802	Tbc1d32	14.45312304	0.136882012	0.370465185
ENSRNOG00000010637	Tbc1d5	20.72533799	-0.515258354	0.318632196
ENSRNOG00000014019	Tbc1d7	36.59851891	0.098974977	0.274698236
ENSRNOG00000013583	Tbc1d8	26.26576903	-0.239075641	0.246211145
ENSRNOG00000054011	Tbc1d8b	13.56131339	-0.153378078	0.374169202
ENSRNOG00000048291	Tbcc	114.0185162	-0.14580782	0.151906949

ENSRNOG00000036658	Tbcd	78.01765026	-0.247528581	0.141936233
ENSRNOG00000029667	Tbce	37.53566094	-0.039032331	0.214152707
ENSRNOG00000011454	Tbck	46.58126962	0.250076944	0.192213537
ENSRNOG00000046252	Tbl2	48.61594531	0.206195444	0.227934885
ENSRNOG00000001489	Tbp	35.03056778	0.269522402	0.241511415
ENSRNOG00000011114	Tbpl1	21.4734658	-0.224873943	0.291335572
ENSRNOG00000005049	Tbr1	11.32160902	-0.626037757	0.401193347
ENSRNOG00000023850	Tbrg1	9.093847467	0.635202581	0.478055307
ENSRNOG00000052477	Tbrg4	63.46711556	-0.235899291	0.158028683
ENSRNOG00000001892	Tbx1	10.11262581	-0.162373414	0.410056592
ENSRNOG00000010685	Tbx18	13.01600068	0.390064242	0.372236918
ENSRNOG00000007918	Tbxas1	22.33389075	-0.062068429	0.269983838
ENSRNOG00000017911	Tcaf1	99.01502337	-0.064893098	0.151916914
ENSRNOG00000004085	Tcaim	43.11458372	0.114526435	0.225900217
ENSRNOG00000016288	Tcea2	82.12342083	-0.200247515	0.161776542
ENSRNOG00000011794	Tcea3	19.54661971	0.192341058	0.28639371
ENSRNOG00000037645	Tceal7	13.37179546	0.155690311	0.359980592
ENSRNOG00000009398	Tceanc2	38.66629535	-0.094344069	0.236229638
ENSRNOG00000018849	Tcerg1	169.5776385	-0.190955866	0.132484415
ENSRNOG00000016737	Tcerg1l	36.65844814	0.011433395	0.269838089
ENSRNOG00000057754	Tcf12	16.1425127	0.384312523	0.337687493
ENSRNOG00000000012	Tcf15	37.24028266	-0.081400435	0.223199728
ENSRNOG00000009585	Tcf20	38.02437392	0.337766412	0.229869455
ENSRNOG00000051499	Tcf3	17.29796515	0.521184162	0.363864479
ENSRNOG00000014753	Tcf7l1	36.023202	-0.018029464	0.205904861
ENSRNOG00000049232	Tcf7l2	32.37520258	0.112725195	0.287762231
ENSRNOG00000061199	Tcfl5	28.7407853	0.156831735	0.280575238
ENSRNOG00000001191	Tchp	95.07056435	0.011270803	0.139728507
ENSRNOG00000047977	Tcim	16.09165513	-0.023396539	0.36904326
ENSRNOG00000004280	Tcn2	22.22737806	-0.133566572	0.328180292
ENSRNOG00000026108	Tcof1	12.13753662	0.579199928	0.394795678
ENSRNOG00000042576	Tcp11l1	31.00779925	0.138333906	0.287916039
ENSRNOG00000028523	Tctn1	24.88133604	-0.364276437	0.285911614
ENSRNOG00000052704	Tctn2	180.6738094	-0.017728753	0.099284699
ENSRNOG00000027124	Tdg	89.11588808	0.101740528	0.211425037
ENSRNOG00000003831	Tdp1	36.67328417	-0.496483718	0.234412966
ENSRNOG00000009034	Tdrd3	30.97340223	0.180560532	0.252130333
ENSRNOG00000055779	Tdrd7	126.8199116	0.090698476	0.124984666
ENSRNOG00000020860	Tdrkh	15.44394696	0.401864333	0.335853759
ENSRNOG00000027245	Tdrp	85.3622883	0.099821661	0.144663221
ENSRNOG00000001010	Tecpr1	28.76991162	0.01665381	0.26040824
ENSRNOG00000021904	Tecpr2	65.98404281	-0.107101971	0.162402759
ENSRNOG00000031126	Tecta	10.55769124	-0.5441901	0.419183355
ENSRNOG00000007436	Tedc2	54.63880319	0.04109762	0.175046562
ENSRNOG00000004000	Tefm	8.23341922	-0.141429326	0.488968996
ENSRNOG00000008587	Tek	44.24253506	0.144827503	0.192489761
ENSRNOG00000002571	Tekt5	16.03421896	-0.340120888	0.329951158
ENSRNOG00000016774	Telo2	108.0655697	-0.03100921	0.129652301
ENSRNOG00000060307	Ten1	110.7502444	0.077797174	0.133040771
ENSRNOG00000012802	Tenm3	31.46094179	-0.098593515	0.24533731

ENSRNOG00000011151	Tenm4	110.6389213	0.0680348	0.172814994
ENSRNOG00000012099	Tent2	14.51646555	-0.065267642	0.334531308
ENSRNOG00000017613	Tent4a	142.6696732	0.102417061	0.127855002
ENSRNOG00000024212	Tent4b	166.2263143	0.147769424	0.099265816
ENSRNOG00000010240	Tent5a	17.51509551	0.141443921	0.337188572
ENSRNOG00000013465	Tepp	140.2253412	0.265969591	0.12830002
ENSRNOG00000028161	Tepsin	11.68487843	0.224004014	0.450646026
ENSRNOG00000055097	Terb1	13.28402381	0.000946509	0.363903498
ENSRNOG00000007291	Terf1	53.37789278	0.029174588	0.169083072
ENSRNOG00000020435	Terf2	10.07775343	-0.056042591	0.482125846
ENSRNOG00000051952	Tes	109.1708895	0.069776067	0.14726922
ENSRNOG00000001128	Tesc	156.1555899	-0.130749921	0.105893202
ENSRNOG00000000277	Tet1	9.394563054	-0.561001176	0.396370918
ENSRNOG00000023579	Tet2	46.28323685	0.443881556	0.205972865
ENSRNOG00000011387	Tet3	25.34856398	0.062740896	0.275229195
ENSRNOG00000008618	Tex10	28.97038685	0.277809536	0.283037587
ENSRNOG00000007748	Tex15	112.4303269	-0.291248553	0.117167109
ENSRNOG00000013659	Tex2	25.35305343	-0.247631112	0.281826842
ENSRNOG00000000905	Tex26	13.39243649	-0.032753767	0.352929026
ENSRNOG00000013201	Tex264	89.61464288	-0.098402883	0.140789254
ENSRNOG00000011658	Tex30	30.57533413	-0.131218508	0.250116108
ENSRNOG00000057264	Tex49	13.35696018	-0.18573626	0.425925681
ENSRNOG00000059702	Tex9	28.65160899	-0.003690342	0.252391832
ENSRNOG00000000613	Tfam	138.422588	-0.019742535	0.135097471
ENSRNOG00000011823	Tfap2b	8.692090661	0.098725071	0.499534122
ENSRNOG00000005227	Tfap4	17.17908253	-0.314817845	0.311172038
ENSRNOG00000056223	Tfb1m	138.6410813	-0.15245465	0.107776423
ENSRNOG00000002695	Tfb2m	88.78387407	-0.045553829	0.144143299
ENSRNOG00000032395	Tfcp2	105.637219	0.101924265	0.160196721
ENSRNOG00000011241	Tfdp2	45.30483934	0.162355065	0.206643184
ENSRNOG00000014666	Tfeb	44.20984652	0.271982153	0.215980859
ENSRNOG00000001633	Tfg	16.09952826	0.037105045	0.344470934
ENSRNOG00000000663	Tfip11	77.47667448	-0.233698277	0.162013764
ENSRNOG00000005039	Tfpi	121.1981216	-0.021446553	0.129961425
ENSRNOG00000056098	Tfpt	34.7963981	0.026888513	0.260530227
ENSRNOG00000001766	Tfrc	112.0748399	0.012964405	0.14714769
ENSRNOG00000009661	Tgds	69.86257909	0.272504926	0.177206036
ENSRNOG00000016182	Tgfa	42.0295515	-0.007483048	0.253316534
ENSRNOG00000020652	Tgfb1	81.58391323	0.0249548	0.143005992
ENSRNOG00000002418	Tgfb2	114.3659119	0.042901991	0.130222001
ENSRNOG00000009867	Tgfb3	59.34869375	-0.139118829	0.177101048
ENSRNOG00000007036	Tgfbr1	58.6036117	-0.158084526	0.160100495
ENSRNOG00000002093	Tgfbr3	33.32077174	0.260428979	0.231281448
ENSRNOG00000050573	Tgfbr3l	164.7596926	-0.026966725	0.0971506
ENSRNOG00000024562	Tgfbrap1	110.4810956	0.120451777	0.141576433
ENSRNOG00000015906	Tgif1	39.68228375	0.309832846	0.21773928
ENSRNOG00000042374	Tgif2	10.3399023	0.266128033	0.420640599
ENSRNOG00000020136	Tgm1	26.5881316	0.095871574	0.253474184
ENSRNOG00000012956	Tgm2	52.98981896	-0.00658579	0.220783268
ENSRNOG00000004320	Tgm4	29.52048054	-0.286872671	0.24559195

ENSRNOG00000014617	Tgoln2	72.37168117	-0.234501489	0.163898263
ENSRNOG00000008156	Tgs1	107.994752	0.002960515	0.123607254
ENSRNOG00000020410	Th	12.35523889	0.05441692	0.350514119
ENSRNOG00000025899	Thada	10.27758718	0.197270223	0.421858525
ENSRNOG00000056956	Thap1	13.39725267	-0.099094869	0.332656491
ENSRNOG00000053923	Thap12	191.4589484	-0.138778843	0.117556678
ENSRNOG00000018351	Thap4	163.2505414	0.015239739	0.104851254
ENSRNOG00000037967	Thap7	139.5029284	0.054790124	0.109910645
ENSRNOG00000004687	Thbd	87.24967405	0.342940634	0.202477407
ENSRNOG00000007960	Theg	8.832830136	0.365994715	0.547634002
ENSRNOG00000020829	Them4	98.77399445	-0.064207684	0.139592921
ENSRNOG00000043445	Them5	45.22300493	0.223694771	0.213504251
ENSRNOG00000005929	Them6	9.780298809	0.176041282	0.453507724
ENSRNOG00000005539	Thg1l	10.27723682	0.2365281	0.385639022
ENSRNOG00000033076	Thnsl1	25.70418633	-0.387506366	0.280889302
ENSRNOG00000006508	Thnsl2	25.8902121	-0.257143423	0.249797163
ENSRNOG00000007315	Thoc2	42.07336175	-0.032887897	0.200908275
ENSRNOG00000042340	Thoc2l	53.59357159	0.219330536	0.181556563
ENSRNOG00000000104	Thoc3	158.1967318	-0.162567292	0.133642724
ENSRNOG00000007701	Thoc7	25.13455499	0.313116149	0.331434744
ENSRNOG00000046850	Thpol1	51.46488938	0.268725912	0.196919977
ENSRNOG00000009066	Thra	116.4869893	-0.546776892	0.448454866
ENSRNOG00000009977	Thrap3	42.10744137	-0.083113613	0.209077128
ENSRNOG00000012404	Thrsp	131.2881401	-0.028323783	0.140217389
ENSRNOG00000012108	Thsd1	48.50999008	0.126972473	0.194463399
ENSRNOG00000030151	Thsd7a	80.69164628	-0.00072057	0.170085521
ENSRNOG00000008291	Thumpd2	41.81226635	0.139079597	0.198279629
ENSRNOG00000006941	Thumpd3	123.856186	0.189281319	0.117782968
ENSRNOG00000047053	Thyn1	84.97838271	0.20200046	0.182088192
ENSRNOG00000016813	Tia1	104.7125785	-0.478929118	0.506006035
ENSRNOG00000020271	Tial1	95.60121148	0.132532311	0.143281554
ENSRNOG00000021569	Tiam1	18.93385331	0.293145867	0.356048725
ENSRNOG00000016728	Tiam2	47.25985995	0.177470555	0.206422367
ENSRNOG00000042070	Ticam2	8.925931329	0.292486291	0.435792404
ENSRNOG00000015520	Ticrr	17.71939625	0.516838707	0.355327033
ENSRNOG00000010941	Tifa	90.2488836	0.133166761	0.136910031
ENSRNOG00000011947	Tifab	33.03879026	0.404693189	0.249790238
ENSRNOG00000051816	Tigar	74.31589516	-0.093097534	0.16182102
ENSRNOG00000038449	Tigd2	17.88227221	-0.221896315	0.307383411
ENSRNOG00000007883	Timm10	142.2009188	-0.125070966	0.117881488
ENSRNOG00000050846	Timm10b	118.7618092	-0.139777248	0.132944934
ENSRNOG00000015142	Timm21	54.78278031	-0.060575659	0.200466904
ENSRNOG00000019811	Timm23	55.2635475	-0.054818443	0.173152412
ENSRNOG00000001058	Timm44	24.02738205	1.24199048	0.578930068
ENSRNOG00000037638	Timm50	118.0103406	-0.06311982	0.124460974
ENSRNOG00000010208	Timp1	20.12870301	-0.057680987	0.281513772
ENSRNOG00000004303	Timp3	9.373598208	-0.03596958	0.439660988
ENSRNOG00000013179	Tinagl1	39.58113027	-0.140204211	0.217350938
ENSRNOG00000043068	Tipin	11.00974501	0.046751696	0.427412461
ENSRNOG00000037221	Tipinl1	37.40659735	-0.141361416	0.266875635

ENSRNOG00000011077	Tjp1	161.001763	0.197228271	0.121888529
ENSRNOG00000015030	Tjp2	59.14199606	-0.143295998	0.16088177
ENSRNOG00000047314	Tk1	67.2971062	0.249569078	0.155592148
ENSRNOG00000012853	Tk2	93.51147243	-0.094977414	0.156261549
ENSRNOG00000007722	Tlcd3a	10.61060994	-0.139052246	0.366079847
ENSRNOG00000019914	Tlcd3b	11.96733015	0.552445372	0.398274076
ENSRNOG00000025160	Tlcd4	10.45149579	0.174989963	0.402856383
ENSRNOG00000006395	Tldc2	20.50077511	0.375171577	0.297485738
ENSRNOG00000005882	Tle1	109.1889259	0.09211614	0.148197034
ENSRNOG00000013013	Tle3	172.9555035	0.193052812	0.149522787
ENSRNOG00000006285	Tlk2	159.499112	-0.104186571	0.110163491
ENSRNOG00000013519	Tll2	156.1313249	0.033893957	0.117243197
ENSRNOG00000016630	Tln1	161.2787267	0.163579072	0.112591976
ENSRNOG00000018373	Tln2	69.22785234	-0.082726436	0.184501755
ENSRNOG00000009822	Tlr2	21.01493651	0.705243302	0.278625546
ENSRNOG00000021726	Tlr3	9.966218345	-0.071821706	0.419989563
ENSRNOG00000022067	Tlr5	14.47210131	0.155948133	0.337718485
ENSRNOG00000002161	Tlr6	9.334580411	-0.012762398	0.397436742
ENSRNOG00000004249	Tlr7	12.58218699	0.160646379	0.39921866
ENSRNOG00000016559	Tm2d2	37.36436369	0.660400786	0.697804567
ENSRNOG00000011290	Tm2d3	64.61060059	0.001823594	0.166246033
ENSRNOG00000015812	Tm4sf1	51.61706612	0.285485858	0.252844331
ENSRNOG00000019662	Tm6sf1	50.61092742	0.160377583	0.226849468
ENSRNOG00000042237	Tm6sf2	14.68658024	-0.69896653	0.317925928
ENSRNOG00000020989	Tm7sf2	13.80818635	-0.065880943	0.828580197
ENSRNOG00000013443	Tm9sf3	45.0824755	0.433185855	0.230815554
ENSRNOG00000009406	Tm9sf4	136.2398775	0.02439911	0.150713825
ENSRNOG00000014188	Tma16	115.7291939	-0.130191309	0.13046216
ENSRNOG00000004312	Tmbim4	45.55323482	0.014911203	0.186442016
ENSRNOG00000016679	Tmc7	11.29705752	0.696015198	0.397487688
ENSRNOG00000011614	Tmcc1	10.02060628	-0.370160641	0.379984905
ENSRNOG00000007713	Tmcc3	58.05943245	-0.25498649	0.198753014
ENSRNOG00000046973	Tmco3	149.699498	0.007420942	0.105174138
ENSRNOG00000017401	Tmco4	36.02376365	-0.037404339	0.217787369
ENSRNOG00000005100	Tmco5a	20.55910561	0.010610248	0.406261958
ENSRNOG00000008037	Tmed1	18.0180765	-0.106041204	0.292805503
ENSRNOG00000001053	Tmed2	17.37210639	0.479102055	0.320681161
ENSRNOG00000013889	Tmed3	185.3222223	0.001491082	0.120002273
ENSRNOG00000005016	Tmed4	10.84345331	-1.747806626	0.519472521
ENSRNOG00000000073	Tmed5	11.26833728	0.519393464	0.370877794
ENSRNOG00000020406	Tmed6	157.5385775	-0.061231172	0.106474328
ENSRNOG00000021882	Tmed9	11.89530813	-0.598489661	0.352577273
ENSRNOG00000008034	Tmeff1	9.146138794	0.026208821	0.421551836
ENSRNOG00000002434	Tmem100	61.21430676	0.058354958	0.239012358
ENSRNOG00000053269	Tmem106c	19.53912851	0.052327414	0.286165274
ENSRNOG00000028017	Tmem109	20.36770072	0.439549946	0.436745761
ENSRNOG00000021899	Tmem115	148.310307	0.290836503	0.113710121
ENSRNOG00000006068	Tmem117	47.95064564	0.277007879	0.20377587
ENSRNOG00000000700	Tmem119	77.69297777	-0.418710941	0.233410113
ENSRNOG00000001441	Tmem120a	14.15214841	0.562704528	0.367725142

ENSRNOG00000005174	Tmem121	39.71520724	-0.153503539	0.230372909
ENSRNOG00000046851	Tmem121b	23.2399235	0.196861079	0.261718187
ENSRNOG00000010584	Tmem123	28.5434327	0.290348562	0.243305571
ENSRNOG00000006924	Tmem125	45.69821914	0.163734977	0.212166925
ENSRNOG00000022732	Tmem126b	141.1863175	-0.295044656	0.117757063
ENSRNOG00000017265	Tmem131	33.95136364	-0.039587052	0.212735487
ENSRNOG00000009837	Tmem131l	26.38069155	0.265032212	0.257759512
ENSRNOG00000021338	Tmem132a	22.03946425	0.008611155	0.288218953
ENSRNOG00000008447	Tmem132d	9.931769704	-0.369401298	0.368377642
ENSRNOG00000007455	Tmem132e	30.80759909	-0.020254509	0.241849505
ENSRNOG00000022153	Tmem134	19.52472347	-0.056995716	0.28956374
ENSRNOG00000016815	Tmem135	189.3184482	0.139465459	0.107229342
ENSRNOG00000021096	Tmem143	12.10782669	0.106407434	0.353645883
ENSRNOG00000010081	Tmem144	52.7198778	0.145896635	0.219419079
ENSRNOG00000015437	Tmem14c	38.29944591	-0.438536317	0.19679323
ENSRNOG00000061100	Tmem150a	43.15396144	0.125954759	0.2234755
ENSRNOG00000004757	Tmem158	16.05104498	0.199191453	0.349332226
ENSRNOG00000048250	Tmem159	20.77567327	0.076751957	0.281773296
ENSRNOG00000015304	Tmem160	52.50269921	0.12889275	0.169476745
ENSRNOG00000012787	Tmem164	125.0116695	-0.20562095	0.128426882
ENSRNOG00000000376	Tmem167b	113.7621149	-0.022994032	0.141938531
ENSRNOG00000016091	Tmem169	32.68732266	0.062769853	0.238523963
ENSRNOG00000009220	Tmem17	112.7698387	-0.153179654	0.130061332
ENSRNOG00000049019	Tmem170a	20.44648701	0.173653377	0.319814247
ENSRNOG00000031495	Tmem170b	23.53571374	0.144807671	0.353067433
ENSRNOG00000000044	Tmem175	38.8463838	0.282644049	0.224925709
ENSRNOG00000025484	Tmem177	37.33154456	-0.180075528	0.282081838
ENSRNOG00000013128	Tmem179	48.46531808	-0.121634691	0.237008943
ENSRNOG00000005144	Tmem18	93.66352274	0.063404461	0.146736827
ENSRNOG00000018158	Tmem181	30.35162071	0.382264103	0.253068538
ENSRNOG00000003594	Tmem183a	78.4463487	-0.2459346	0.230703765
ENSRNOG00000022802	Tmem184b	14.61486841	0.196521978	0.346095738
ENSRNOG00000011047	Tmem185a	77.96812537	0.02234951	0.142666979
ENSRNOG00000027087	Tmem186	66.23598401	0.055988954	0.15696381
ENSRNOG00000003985	Tmem19	93.83704652	0.111302224	0.139590484
ENSRNOG00000042528	Tmem190	17.25463729	-0.413184011	0.329922036
ENSRNOG00000001864	Tmem191c	163.2596568	0.152950845	0.111764115
ENSRNOG00000030199	Tmem192	189.9086475	0.067635815	0.104526655
ENSRNOG00000037435	Tmem196	177.1219401	0.050117077	0.111184918
ENSRNOG00000020061	Tmem198	13.02422049	-0.161680622	0.364409124
ENSRNOG00000047783	Tmem200a	33.84768701	-0.124236949	0.210563269
ENSRNOG00000049880	Tmem200c	180.4503712	-0.071689103	0.12091834
ENSRNOG00000023152	Tmem201	190.8772991	-0.093184463	0.105625779
ENSRNOG00000026112	Tmem202	23.69041239	0.010463644	0.263560549
ENSRNOG00000011747	Tmem205	51.01957571	0.203147012	0.249842293
ENSRNOG00000028219	Tmem209	40.63215094	-0.317592597	0.197459466
ENSRNOG00000051192	Tmem211	10.68369208	-0.368976553	0.406296313
ENSRNOG00000008812	Tmem214	162.9161322	-0.184755605	0.107024446
ENSRNOG00000042246	Tmem215	19.65212801	0.300887896	0.31314029
ENSRNOG00000020692	Tmem216	150.4828106	0.091173759	0.124496748

ENSRNOG00000008757	Tmem218	62.01145242	0.099068492	0.164220419
ENSRNOG00000020000	Tmem219	39.86673365	-0.310631578	0.237387859
ENSRNOG00000003367	Tmem220	8.853726093	-0.951675169	0.395227537
ENSRNOG00000008564	Tmem222	135.6730557	-1.090242292	0.555960525
ENSRNOG00000049326	Tmem234	11.83034943	0.256048832	0.395740641
ENSRNOG00000048231	Tmem235	15.93743348	-0.089457163	0.39929611
ENSRNOG00000024085	Tmem237	116.6554319	0.316628044	0.143766186
ENSRNOG00000023412	Tmem241	9.87116748	-0.088584108	0.399086675
ENSRNOG00000016973	Tmem242	27.38802951	0.279501735	0.267670762
ENSRNOG00000000893	Tmem248	119.8397777	-0.066065842	0.121982346
ENSRNOG00000008101	Tmem251	9.546399838	0.559370874	0.434965384
ENSRNOG00000025476	Tmem252	26.76789307	0.351306047	0.296092557
ENSRNOG00000028085	Tmem255a	131.9914979	0.251194081	0.152085887
ENSRNOG00000026336	Tmem255b	14.79931692	0.024271873	0.320923083
ENSRNOG00000013353	Tmem260	92.14905444	-0.005630451	0.143976939
ENSRNOG00000015201	Tmem266	50.34742931	0.015834702	0.196020071
ENSRNOG00000008712	Tmem268	19.66160741	-0.159920129	0.296238506
ENSRNOG00000028063	Tmem38b	93.62825927	-0.072907407	0.160603419
ENSRNOG00000003075	Tmem39a	31.14604351	0.35464465	0.25132235
ENSRNOG00000023746	Tmem39b	16.37060036	0.369675395	0.307916664
ENSRNOG00000046283	Tmem41a	43.48876123	-0.172149308	0.201837827
ENSRNOG00000001726	Tmem44	100.0813677	0.171373166	0.16932464
ENSRNOG00000017279	Tmem50a	135.7597839	0.337928857	0.275161827
ENSRNOG00000014197	Tmem51	27.68454437	0.318849582	0.255526896
ENSRNOG00000058653	Tmem52b	14.31734607	0.394267848	0.357178181
ENSRNOG00000013510	Tmem60	182.8442622	0.032123455	0.129390329
ENSRNOG00000023874	Tmem62	29.78021415	0.125763695	0.227929056
ENSRNOG00000003310	Tmem63a	15.04290886	0.575204656	0.430317366
ENSRNOG00000019743	Tmem63b	52.78418763	0.219014816	0.194711333
ENSRNOG00000008934	Tmem65	20.99854239	0.142105256	0.302271848
ENSRNOG00000016187	Tmem67	58.85291373	0.029168198	0.20116024
ENSRNOG00000008872	Tmem68	128.4592555	0.04119999	0.121602856
ENSRNOG00000029152	Tmem69	183.6013678	0.033280969	0.102759558
ENSRNOG00000006608	Tmem70	178.587061	-0.050253987	0.121924783
ENSRNOG00000019414	Tmem79	54.17193453	-0.023993407	0.193231828
ENSRNOG00000028752	Tmem81	71.45968707	0.206970041	0.252496051
ENSRNOG00000011970	Tmem82	51.98384791	-0.422896421	0.213530569
ENSRNOG00000013663	Tmem86a	45.92502428	0.094968807	0.1931489
ENSRNOG00000008455	Tmem87a	11.86286875	-0.322023372	0.374991087
ENSRNOG00000017443	Tmem87b	176.2293371	-0.036954607	0.105246194
ENSRNOG00000036869	Tmem88b	12.69487102	-0.020958123	0.391941952
ENSRNOG00000004032	Tmem94	8.88927758	-0.212631499	1.000641679
ENSRNOG00000021316	Tmem98	28.82447568	0.105346592	0.25095888
ENSRNOG00000056462	Tmf1	65.97520068	0.080975094	0.161527054
ENSRNOG00000027799	Tmie	14.7239019	-0.184896414	0.357626469
ENSRNOG00000043375	Tmigd3	10.2270634	0.219332213	0.381746951
ENSRNOG00000000729	Tmlhe	18.18668251	0.07593932	0.328454357
ENSRNOG00000009761	Tmod1	56.08667695	-0.088946547	0.195313112
ENSRNOG00000010447	Tmod2	55.63990425	0.143549278	0.194660748
ENSRNOG00000032436	Tmod3	42.24950971	0.076325352	0.201092589

ENSRNOG00000021088	Tmod4	10.0941223	-0.489346768	0.391000787
ENSRNOG00000008797	Tmpo	98.51826204	0.075362606	0.168468341
ENSRNOG00000000184	Tmprss6	12.72636918	0.220029813	0.420115895
ENSRNOG00000032429	Tmprss9	53.58522035	0.473706467	0.523349845
ENSRNOG00000036921	Tmsb10	177.9510504	-0.015854638	0.109442687
ENSRNOG00000001854	Tmtc1	17.2866669	-0.413275732	0.30959627
ENSRNOG00000006749	Tmtc3	42.73855088	-0.269399886	0.207772311
ENSRNOG00000014310	Tmtc4	9.071386247	-0.446295209	0.446890218
ENSRNOG00000013383	Tmub1	14.10649925	-0.076406623	0.323123104
ENSRNOG00000057934	Tmx1	72.47556561	0.107638882	0.152278617
ENSRNOG00000045740	Tmx3	8.722465975	0.053541256	0.462832234
ENSRNOG00000058645	Tnc	19.95463843	0.35280449	0.328706565
ENSRNOG00000050792	Tnfaip6	18.5742051	0.255115885	0.330358939
ENSRNOG00000026136	Tnfaip8	35.00430618	0.021644431	0.215946098
ENSRNOG00000024230	Tnfaip8l3	71.19058811	0.046266844	0.173148754
ENSRNOG00000015615	Tnfrsf11a	39.58290942	-0.07365732	0.234360767
ENSRNOG00000008336	Tnfrsf11b	106.9532735	0.13936616	0.194152328
ENSRNOG00000014265	Tnfrsf19	156.426979	-0.176578863	0.123045385
ENSRNOG00000031312	Tnfrsf1a	15.08609195	-0.302775755	0.322511378
ENSRNOG00000020106	Tnfrsf4	176.7385759	-0.109594671	0.130839423
ENSRNOG00000008930	Tnfsf15	22.60318657	0.25060186	0.315525277
ENSRNOG00000045595	Tnfsf9	15.40935685	-0.293942189	0.363681395
ENSRNOG00000012422	Tnik	48.00743165	-0.036862481	0.228988437
ENSRNOG00000010370	Tnip1	48.34173998	0.045578497	0.205324212
ENSRNOG00000015578	Tnk1	12.51909201	0.844796085	0.413541434
ENSRNOG00000011625	Tnks	33.25717691	-0.029313243	0.21488726
ENSRNOG00000009195	Tnks1bp1	172.9382582	-0.043199185	0.117531954
ENSRNOG00000018943	Tnnc1	14.86441726	0.206221946	0.350629812
ENSRNOG00000028225	Tnni3k	9.690074708	0.013585183	0.465273812
ENSRNOG00000033734	Tnnt2	26.97982981	-0.063644647	0.36058152
ENSRNOG00000014999	Tnpo1	99.14241637	0.124306193	0.165896536
ENSRNOG00000003970	Tnpo2	41.05311057	-0.16369313	0.327777687
ENSRNOG00000021758	Tnpo3	45.6811804	0.17270589	0.213507077
ENSRNOG00000002468	Tnr	138.9431008	0.309759494	0.147318637
ENSRNOG00000024482	Tnrc18	43.92503334	-0.009074933	0.229453044
ENSRNOG00000022395	Tnrc6c	53.65721893	0.242835844	0.203975188
ENSRNOG00000010588	Tns2	11.15812638	0.113253596	0.481230628
ENSRNOG00000050500	Tob2	58.72657889	0.061945714	0.165614615
ENSRNOG00000004415	Togaram1	129.9261984	0.045115279	0.134528444
ENSRNOG00000002474	Tom1l1	72.06862017	-0.044940757	0.156503156
ENSRNOG00000003590	Tom1l2	92.05881115	0.159773732	0.174869234
ENSRNOG00000029799	Tomm34	11.49306996	0.461137925	0.437862816
ENSRNOG00000018556	Tomm40	13.90069709	0.40849392	0.342181336
ENSRNOG00000001640	Tomm70	44.77925303	-0.051214746	0.199965719
ENSRNOG00000047611	Top1	189.0939225	0.151692694	0.113324727
ENSRNOG00000007500	Top1mt	87.43156778	-0.186347219	0.138411188
ENSRNOG00000048795	Top2b	17.23851366	0.108917076	0.301906329
ENSRNOG00000001845	Top3b	16.22697988	0.314329946	0.401634588
ENSRNOG00000009789	Topbp1	10.92701489	0.873787081	0.398643789
ENSRNOG00000006485	Topors	78.15389244	0.298382129	0.187240361

ENSRNOG00000006894	Tor1a	15.54853045	-0.36486908	0.336709156
ENSRNOG00000003946	Tor1aip1	109.0963661	-0.059020202	0.122188306
ENSRNOG00000048267	Tor1aip2	147.7961493	-0.161364225	0.117189118
ENSRNOG00000024849	Tor1aip2	50.90979348	0.166925398	0.187767181
ENSRNOG00000022514	Tor2a	26.83732777	0.203821626	0.260018941
ENSRNOG00000010777	Tox	167.6823297	-0.041182977	0.126980141
ENSRNOG00000028649	Tox3	157.5440734	0.073519562	0.134340948
ENSRNOG00000012844	Tox4	15.37469033	-0.405992869	0.328587687
ENSRNOG00000010756	Tp53	70.05710719	-0.107474618	0.143295624
ENSRNOG00000013837	Tp53bp1	83.38875839	0.075183833	0.18141411
ENSRNOG00000003237	Tp53bp2	78.86366237	-0.138841463	0.167622204
ENSRNOG00000008738	Tp53i11	16.43755633	0.236497976	0.311969975
ENSRNOG00000005177	Tp53i3	8.255963294	-0.104194167	0.442955121
ENSRNOG00000007964	Tp53inp1	14.26279307	1.131743788	0.374900879
ENSRNOG00000018225	Tp53inp2	25.35616681	0.256743339	0.330440487
ENSRNOG00000007285	Tp53rk	54.71065655	0.198141349	0.217292763
ENSRNOG00000010694	Tpbg	123.0680154	-0.053522768	0.158826756
ENSRNOG00000059344	Tpcn1	13.55098381	0.276760851	0.428267174
ENSRNOG00000013387	Tpcn2	11.04986714	0.033554316	0.392298474
ENSRNOG00000011441	Tpd52	182.490079	-0.027883314	0.09705936
ENSRNOG00000021478	Tpd52l1	44.86823141	0.082266327	0.20156687
ENSRNOG00000042441	Tpd52l3	10.72736314	0.651195591	0.412282869
ENSRNOG00000015290	Tpi1	27.5840259	0.738486733	0.641137078
ENSRNOG00000029922	Tpk1	16.15855871	0.088727012	0.36570776
ENSRNOG00000016731	Tpm2	25.21138107	0.19041321	0.295803754
ENSRNOG00000017441	Tpm3	96.78300556	-0.081375019	0.144970035
ENSRNOG00000016468	Tpmt	45.65489053	-0.142555552	0.260399565
ENSRNOG00000028261	Tppp	13.39491966	-0.449559057	0.336673736
ENSRNOG00000002394	Tpr	132.6086816	-0.130843366	0.160390561
ENSRNOG00000016665	Tpra1	148.4696912	-0.022109141	0.107650651
ENSRNOG00000046227	Tprg1l	15.14957763	0.139469475	0.329321174
ENSRNOG00000010896	Tprn	37.40156275	-0.054871116	0.231401203
ENSRNOG00000000664	Tpst2	142.6289195	0.059717478	0.120641167
ENSRNOG00000024837	Tpte2	7.859610461	0.073521606	0.492698738
ENSRNOG00000008165	Tpx2	45.54353805	-0.145961226	0.213078851
ENSRNOG00000006238	Traf2	37.89537455	-0.001611926	0.208842079
ENSRNOG00000008145	Traf3	101.5191945	0.342664767	0.170546832
ENSRNOG00000024468	Traf3ip1	24.69060205	0.119286812	0.268643193
ENSRNOG00000000595	Traf3ip2	14.70458582	0.708480188	0.371568831
ENSRNOG00000005565	Traf3ip3	16.54108489	-0.372189315	0.331866794
ENSRNOG00000013169	Traf4	9.026937024	0.124675047	0.454458774
ENSRNOG00000047957	Traf5	9.549207364	0.218746866	0.509547663
ENSRNOG00000004639	Traf6	13.2713129	0.171782634	0.431022877
ENSRNOG00000003131	Traf7	8.630443381	0.472238533	0.451866331
ENSRNOG00000021091	Trank1	83.99545324	-0.094330843	0.175634217
ENSRNOG00000005418	Trap1	27.76892434	0.11827624	0.27401578
ENSRNOG00000023210	Trappc10	37.99887576	0.152980353	0.20258846
ENSRNOG00000009039	Trappc12	55.8503288	0.041425342	0.169866247
ENSRNOG00000042276	Trappc2	15.70689297	-0.399655152	0.320379099
ENSRNOG00000049062	Trappc2b	107.4728123	0.025233882	0.131658413

ENSRNOG00000014581	Trappc2l	31.27870313	0.118829405	0.233742429
ENSRNOG00000022202	Trappc8	102.0615197	0.007394699	0.133572497
ENSRNOG00000013578	Trem2	71.79331781	0.084722992	0.200370285
ENSRNOG00000022598	Trerf1	44.07442325	0.130124709	0.188353242
ENSRNOG00000005278	Trhde	42.53763505	0.466248085	0.218383901
ENSRNOG00000004100	Trib1	13.20376894	-0.075701551	0.424296728
ENSRNOG00000004110	Trib2	141.7841408	0.211501528	0.171478139
ENSRNOG00000002915	Trim11	32.27060349	-0.096683427	0.225703578
ENSRNOG00000008922	Trim14	87.92955033	0.062509341	0.17728942
ENSRNOG00000031689	Trim15	12.08637792	0.429294858	0.371159787
ENSRNOG00000022983	Trim17	45.81637933	0.140887018	0.195100492
ENSRNOG00000012354	Trim23	18.69147144	-0.093489028	0.280174665
ENSRNOG00000013251	Trim24	121.6497748	0.100271283	0.133184482
ENSRNOG00000027487	Trim28	16.47554633	-0.22892525	0.311133987
ENSRNOG00000018946	Trim33	81.1652958	-0.346567485	0.197730438
ENSRNOG00000006248	Trim37	112.9230164	0.057419972	0.17321837
ENSRNOG00000000785	Trim39	9.986940358	0.487801347	0.514328491
ENSRNOG00000002388	Trim41	16.47903795	0.472846934	0.335766875
ENSRNOG00000005191	Trim44	34.43476278	-0.264743933	0.359219544
ENSRNOG00000015347	Trim45	46.13603436	-0.311839955	0.184028171
ENSRNOG00000055433	Trim46	57.25282996	-0.317978637	0.387217304
ENSRNOG00000008215	Trim47	53.88718595	0.545290322	0.178504154
ENSRNOG00000024145	Trim65	24.37994068	0.451251825	0.331678378
ENSRNOG00000007031	Trim9	116.3052209	0.10438639	0.125423076
ENSRNOG00000059015	Triobp	48.96181798	0.117238199	0.183331827
ENSRNOG00000055524	Trip10	14.86356107	0.33436254	0.325614428
ENSRNOG00000005292	Trip11	68.9909149	0.099091523	0.243946497
ENSRNOG00000016121	Trip4	52.28022727	-0.098214129	0.172349326
ENSRNOG00000048580	Trip6	21.14271947	0.302641201	0.286413061
ENSRNOG00000039924	Triqk	120.318234	0.095693537	0.144272547
ENSRNOG00000014274	Trit1	110.8002219	0.14875471	0.133304895
ENSRNOG00000009492	Trmo	10.42692204	-0.513749014	0.390694913
ENSRNOG00000011025	Trmt10a	59.86765017	-0.17344473	0.184774603
ENSRNOG00000012454	Trmt10b	40.65460301	0.284871671	0.203910499
ENSRNOG00000039567	Trmt10c	101.9762844	-0.174504441	0.148186875
ENSRNOG00000008978	Trmt12	29.74721641	0.354507884	0.232665527
ENSRNOG00000015140	Trmt13	20.65524571	0.775555439	0.338578107
ENSRNOG00000002580	Trmt1l	94.5044479	0.169023521	0.150749634
ENSRNOG00000008898	Trmt44	23.78854256	-0.039726079	0.280874233
ENSRNOG00000007785	Trmt5	96.59722964	0.035530234	0.157317186
ENSRNOG00000021270	Trmt6	72.08236818	0.10989035	0.169128737
ENSRNOG00000011253	Trmt9b	26.31296104	0.179246795	0.241256417
ENSRNOG00000016465	Trmu	35.81108939	0.349601718	0.253501194
ENSRNOG00000055344	Trnau1ap	41.25721429	0.082260335	0.20828848
ENSRNOG00000055936	Trnp1	11.10689241	0.441149911	0.449223833
ENSRNOG00000006432	Trnt1	96.01809473	-0.006610673	0.136183274
ENSRNOG00000054902	Trpc1	120.0596497	-0.201798778	0.146852912
ENSRNOG00000020188	Trpc2	17.85407391	0.471824786	0.334763299
ENSRNOG00000016070	Trpc3	74.7319605	-0.073812273	0.18739763
ENSRNOG00000011133	Trpc4	34.59911392	0.015316751	0.229680772

ENSRNOG00000019152	Trpc4ap	34.88223804	-0.080588065	0.257172975
ENSRNOG00000012727	Trpc7	10.57300731	0.468586581	0.406746861
ENSRNOG00000001216	Trpm2	9.070080565	-0.082953087	0.416910213
ENSRNOG00000027770	Trpm3	17.10523517	-0.202204958	0.367938928
ENSRNOG00000020714	Trpm4	24.89593851	-0.11791868	0.265484401
ENSRNOG00000057806	Trpm7	55.83779757	0.497606243	0.193634745
ENSRNOG00000003104	Trpv2	59.03670191	0.123435781	0.185745146
ENSRNOG00000001195	Trpv4	10.41930183	-0.002707703	0.443053774
ENSRNOG00000017321	Trub1	86.87316641	-0.041513972	0.155746191
ENSRNOG00000043189	Trub2	130.8347884	0.284480418	0.154388329
ENSRNOG00000011470	Tsc1	14.05839156	0.291924421	0.34401946
ENSRNOG00000011375	Tsc2	23.53531675	0.226403188	0.377487487
ENSRNOG00000038044	Tsc22d2	63.71917638	-0.07165935	0.168418821
ENSRNOG00000050805	Tsc22d4	13.30664867	0.244274468	0.353635026
ENSRNOG00000002352	Tsen15	106.089805	0.03740373	0.137940845
ENSRNOG00000060175	Tsen2	12.39854861	0.123056787	0.416607463
ENSRNOG00000059572	Tsen34	31.11226775	0.098309905	0.242262147
ENSRNOG00000048843	Tsfm	8.854539408	0.585731674	0.42792571
ENSRNOG00000058681	Tsga10	13.34983055	0.482130606	0.379398588
ENSRNOG00000027564	Tsga10ip	137.2505797	-0.090336928	0.337651499
ENSRNOG00000003972	Tshr	11.7990531	0.0912605	0.352751124
ENSRNOG00000027784	Tsku	8.167457219	-0.28920812	0.52794531
ENSRNOG00000046076	Tspan10	12.49828374	-0.721104328	0.999897515
ENSRNOG00000059016	Tspan12	117.8874468	0.093448157	0.14364622
ENSRNOG00000046204	Tspan15	8.725693323	0.313054718	0.479075691
ENSRNOG00000025592	Tspan31	63.69836768	0.193516658	0.164477436
ENSRNOG00000029810	Tspan4	20.5001056	0.261823828	0.270865624
ENSRNOG00000004411	Tspan8	17.04715911	0.16592575	0.305728042
ENSRNOG00000051620	Tspan9	63.01733456	0.004967571	0.172124006
ENSRNOG00000010549	Tspo	69.14172509	0.043243446	0.173246011
ENSRNOG00000007957	Tspoap1	40.10397366	0.09913856	0.20505119
ENSRNOG00000026770	Tspy26	104.2773218	-0.056795037	0.163949835
ENSRNOG00000006278	Tspyl5	7.809428302	-0.068221719	0.472836138
ENSRNOG00000002980	Tsr1	15.20015561	0.18960546	0.338360206
ENSRNOG00000009724	Tstd2	31.37126845	0.2222123	0.236501196
ENSRNOG00000018416	Ttbk1	157.9785137	0.269786865	0.133676965
ENSRNOG00000011059	Ttbk2	13.02075036	-0.471981397	0.381838712
ENSRNOG00000008595	Ttc12	62.27868553	-0.241910694	0.510776771
ENSRNOG00000018884	Ttc13	110.9843627	0.248269183	0.136582906
ENSRNOG00000011261	Ttc14	16.02294994	0.456389012	0.368844209
ENSRNOG00000050650	Ttc16	22.68852197	0.265676695	0.284642332
ENSRNOG00000010495	Ttc17	119.4393272	0.122672296	0.129882417
ENSRNOG00000002977	Ttc19	119.1337805	0.150179641	0.131799471
ENSRNOG00000034089	Ttc21a	23.30210741	-0.229675373	1.084437741
ENSRNOG00000005868	Ttc21b	12.76112106	0.514124834	0.375802433
ENSRNOG00000007189	Ttc22	50.56323139	-0.118084431	0.20159513
ENSRNOG00000027617	Ttc26	24.19590945	0.135732707	0.295190883
ENSRNOG00000042932	Ttc27	98.17289679	-0.038950276	0.129109913
ENSRNOG00000032590	Ttc28	63.48202896	0.415518328	0.214816792
ENSRNOG00000052519	Ttc30a1	18.23744061	-0.025214431	0.316481775

ENSRNOG00000059460	Ttc30b	107.1278588	0.095436732	0.148028481
ENSRNOG00000049088	Ttc32	38.72697631	-0.093024584	0.215046041
ENSRNOG00000013217	Ttc33	66.69606799	-0.217478863	0.158792294
ENSRNOG00000040297	Ttc37	21.3286129	-0.174210415	0.334715127
ENSRNOG00000047573	Ttc39a	24.0241709	-0.419876623	0.296488364
ENSRNOG00000050949	Ttc39c	126.0463398	-0.183509829	0.361066023
ENSRNOG00000042467	Ttc4	88.20515094	-0.057242801	0.158405334
ENSRNOG00000026807	Ttc41	8.088368361	0.254128063	0.510226016
ENSRNOG00000008850	Ttc5	178.6913131	-0.130641722	0.096486119
ENSRNOG00000014879	Ttc7a	47.28063377	0.036095345	0.226523006
ENSRNOG00000004542	Ttc8	69.0639348	0.28794225	0.167053846
ENSRNOG00000018850	Ttc9b	42.21979062	-0.163951889	0.208664863
ENSRNOG00000019464	Ttc9c	23.58267845	-0.208256967	0.26014865
ENSRNOG00000039777	Ttf1	37.10805915	-0.199480323	0.205567786
ENSRNOG00000012880	Tti1	157.1353469	-0.317855448	0.473634801
ENSRNOG00000029055	Ttk	25.66667396	0.302042016	0.316979494
ENSRNOG00000018205	Ttl	40.88260219	0.008927089	0.222881618
ENSRNOG00000019339	Ttll11	148.8829481	-0.061799847	0.120346542
ENSRNOG00000056362	Ttll13	31.28204395	-0.129647366	0.234679715
ENSRNOG00000009032	Ttll3	74.26845571	-0.086833063	0.163866134
ENSRNOG00000017129	Ttll4	12.20393182	0.188933944	0.403606513
ENSRNOG00000009318	Ttll5	19.5318882	0.289192042	0.317199537
ENSRNOG00000008596	Ttll9	14.54620184	-0.117808482	0.353363338
ENSRNOG00000007139	Ttpa	8.541361707	-0.139874213	0.512386636
ENSRNOG00000016275	Ttr	11.89510392	-1.086886604	0.420871575
ENSRNOG00000046566	Tub	83.74970981	0.266324104	0.146716075
ENSRNOG00000003597	Tuba4a	36.15962099	0.132695323	0.209935085
ENSRNOG00000048169	Tuba8	74.96521448	0.039234157	0.155689108
ENSRNOG00000017209	Tubb3	34.03474493	-0.051151717	0.251450209
ENSRNOG00000010170	Tubb4b	27.51877777	0.128816756	0.261271459
ENSRNOG00000061216	Tubb5	32.05323628	-0.073798816	0.230219152
ENSRNOG00000006494	Tubg2	9.828737902	-0.169111869	0.417785931
ENSRNOG00000018137	Tubgcp2	11.4740001	-0.08415366	0.381022341
ENSRNOG00000017085	Tubgcp3	91.69492447	0.053199128	0.147945253
ENSRNOG00000012798	Tubgcp4	52.95470357	0.471967187	0.214032391
ENSRNOG00000011480	Tubgcp5	73.13999132	-0.162739324	0.160819887
ENSRNOG00000018604	Tufm	12.76497774	0.207022554	0.426181729
ENSRNOG00000005767	Tulp3	7.921998664	1.015622764	0.528659548
ENSRNOG00000018012	Tulp4	120.9455524	-0.093579715	0.180961082
ENSRNOG00000052062	Tut4	35.02488621	0.37846002	0.21289995
ENSRNOG00000016629	Tut7	38.23633697	-0.049340913	0.243602331
ENSRNOG00000050649	Tvp23b	106.9125531	0.005396704	0.173739512
ENSRNOG00000011101	Twist1	9.314718612	-0.368526106	0.454772753
ENSRNOG00000014935	Twnk	135.7153379	0.006677229	0.135489177
ENSRNOG00000013340	Twsg1	165.0523201	0.188527968	0.127491287
ENSRNOG00000004971	Txlng	81.3009428	0.014981802	0.162375446
ENSRNOG00000002452	Txndc11	12.85138794	0.731188353	0.397797423
ENSRNOG00000000133	Txndc15	26.89503967	0.347511084	0.241256108
ENSRNOG00000006334	Txndc16	8.70684456	-0.402249801	0.443155172
ENSRNOG00000013469	Txndc5	87.81838128	0.241192434	0.149625639

ENSRNOG00000021201	Txnip	82.81808198	-0.161912879	0.193534814
ENSRNOG00000001890	Txnrd2	112.3424929	0.073251627	0.160066743
ENSRNOG00000059810	Txnrd3	46.12020718	0.21765223	0.200286927
ENSRNOG00000032948	Tyk2	47.71964899	0.298381695	0.203255872
ENSRNOG00000024839	Tysnd1	38.54091759	0.457086769	0.208315321
ENSRNOG00000024352	Tyw1	68.87040562	-0.200926195	0.160085679
ENSRNOG00000028328	Tyw3	61.89381116	-0.304331524	0.163934871
ENSRNOG00000010100	Tyw5	37.56976346	0.057362206	0.208656412
ENSRNOG00000057317	U1	113.1960827	-0.137024339	0.144917537
ENSRNOG00000061673	U1	36.44757626	0.153940525	0.274085627
ENSRNOG00000061336	U1	145.2848153	0.036512712	0.124347908
ENSRNOG00000054047	U2	47.92321336	0.257589661	0.238964559
ENSRNOG00000053870	U2	31.07865032	-0.053871355	0.221790742
ENSRNOG00000061734	U2	26.82989247	0.054085854	0.254637413
ENSRNOG00000015914	U2af2	175.1409344	-0.125183001	0.122971128
ENSRNOG00000008607	U2surp	78.25760664	0.046500735	0.150150542
ENSRNOG00000054676	U3	20.08470872	0.230410436	0.299647091
ENSRNOG00000059105	U4	33.66899209	0.17419187	0.227655892
ENSRNOG00000051990	U4	36.30803106	-0.086241182	0.23126468
ENSRNOG00000059917	U6	59.27553829	-0.29901024	0.205672991
ENSRNOG00000060282	U6	13.13019648	-0.621858002	0.431381173
ENSRNOG00000054670	U6	7.96413711	-0.546695764	0.47664351
ENSRNOG00000058201	U6	40.14073273	-0.089422778	0.210111273
ENSRNOG00000057697	U6	9.416904145	0.149927905	0.478758179
ENSRNOG00000057055	U6	56.16619503	-0.039499454	0.178073197
ENSRNOG00000059108	U7	17.47443286	-0.093244661	0.292922663
ENSRNOG00000012868	Uaca	38.82419292	0.071254054	0.220044661
ENSRNOG00000012960	Uap1l1	63.150409	-0.027374818	0.187213013
ENSRNOG00000021113	Uba2	44.30537213	-0.439965426	0.286245958
ENSRNOG00000006221	Uba3	16.15791389	0.034970242	0.312329886
ENSRNOG00000011027	Uba5	101.383661	-0.02863755	0.178086458
ENSRNOG00000021931	Uba6	77.32291234	0.046352309	0.147372583
ENSRNOG00000012004	Ubap1	14.9071315	0.150564308	0.366561735
ENSRNOG00000017990	Ubap2l	113.1663956	0.176563198	0.130599451
ENSRNOG00000008187	Ubash3b	59.28492867	-0.038603383	0.175760606
ENSRNOG00000032690	Ube2e2	179.7876647	0.207638078	0.104765484
ENSRNOG00000019953	Ube2f	40.73789675	0.211261385	0.207308876
ENSRNOG00000017907	Ube2i	56.72433128	-0.420134564	0.188022117
ENSRNOG00000007434	Ube2j1	69.56801518	0.057264277	0.168773667
ENSRNOG00000019749	Ube2j2	38.05940281	0.444794879	0.206125927
ENSRNOG00000001862	Ube2l3	40.11436364	0.317148019	0.205378485
ENSRNOG00000027514	Ube2m	24.32916928	-0.072681202	0.273568421
ENSRNOG00000058053	Ube2n	94.59404677	-0.244665721	0.359876077
ENSRNOG00000011007	Ube2o	37.56561708	-0.073495256	0.228226938
ENSRNOG00000014529	Ube2q2	28.04839828	0.345294353	0.262243875
ENSRNOG00000006868	Ube2z	28.94467727	-0.082669865	0.242474145
ENSRNOG00000047219	Ube3b	16.31483261	0.197362335	0.347783697
ENSRNOG00000010802	Ube3d	27.87639936	0.431138947	0.308705014
ENSRNOG00000026833	Ube4a	76.37066312	-0.029249752	0.189578827
ENSRNOG00000009575	Ubiad1	51.78263351	0.104285179	0.193847783

ENSRNOG00000003019	Ubn1	92.7332236	0.107665671	0.142467839
ENSRNOG00000005564	Ubn2	155.4359249	0.006059847	0.146614799
ENSRNOG00000037207	Ubr1	30.11159002	0.208265063	0.279801453
ENSRNOG00000015813	Ubr2	76.24859897	-0.052634989	0.169013732
ENSRNOG00000008616	Ubr3	41.58232363	0.385093135	0.246010405
ENSRNOG00000006816	Ubr5	80.04961594	0.350948913	0.173528207
ENSRNOG00000004137	Ubtd2	73.73582669	0.070043669	0.164301876
ENSRNOG00000019666	Ubxn1	121.6007745	0.007870014	0.122691684
ENSRNOG00000015476	Ubxn11	28.61332189	0.058040258	0.265513956
ENSRNOG00000048509	Ubxn6	21.21562847	0.07724541	0.302726753
ENSRNOG00000015109	Ubxn8	22.76158753	-0.319060409	0.266572734
ENSRNOG00000003545	Uchl5	109.667545	-0.047107269	0.122199945
ENSRNOG00000003917	Uck2	103.5724985	-0.3384929	0.16340954
ENSRNOG00000050277	Uckl1	172.7567282	0.093836223	0.12129889
ENSRNOG00000017987	Ucma	47.28814625	-0.304710076	0.287097861
ENSRNOG00000017854	Ucp2	9.700872496	-0.236441152	0.394271892
ENSRNOG00000017716	Ucp3	48.99580567	0.083020728	0.186398652
ENSRNOG00000003706	Ufc1	17.23036936	-0.331262619	0.293479671
ENSRNOG00000047394	Ufd1	43.23888141	-0.387982314	0.208430003
ENSRNOG00000007831	Ufl1	95.73628326	-0.041155627	0.144674485
ENSRNOG00000038176	Ufm1	172.0479241	0.034330946	0.12265626
ENSRNOG00000012087	Ufsp2	42.11670856	0.174887809	0.223934695
ENSRNOG00000002643	Ugdh	22.07496463	0.253257895	0.305399416
ENSRNOG00000014901	Uggt1	149.4613679	0.145219678	0.123777474
ENSRNOG00000008079	Ugp2	42.28960553	-0.156665297	0.197292277
ENSRNOG00000018740	Ugt1a3	61.24131461	-0.119910284	0.187971275
ENSRNOG00000009345	Ugt8	177.7511901	0.124994348	0.180418866
ENSRNOG00000002941	Uhmk1	71.72994794	-0.02428295	0.163912924
ENSRNOG00000000495	Uhrf1bp1	8.950291324	-0.275085091	0.469191768
ENSRNOG00000011308	Uhrf2	153.8851965	0.164345299	0.122995148
ENSRNOG00000016891	Uimc1	132.6314304	-0.004637109	0.115512702
ENSRNOG00000001797	Umps	147.8539766	-0.080002413	0.104945052
ENSRNOG00000018452	Unc13a	57.84422291	-0.025726531	0.189061673
ENSRNOG00000008237	Unc13b	32.10877854	0.1289882	0.338769873
ENSRNOG00000056612	Unc13c	11.2612149	-0.133867173	0.409173082
ENSRNOG00000012357	Unc45a	12.64294049	-0.029109911	0.34121377
ENSRNOG00000018128	Unc50	9.319501672	-0.442783277	0.449767147
ENSRNOG00000059840	Unc5a	30.04861294	-0.280769204	0.237332811
ENSRNOG00000000567	Unc5b	69.09161013	0.154071127	0.211284077
ENSRNOG00000029071	Unc5c	47.76235465	0.263099659	0.186156932
ENSRNOG00000008728	Unc79	79.55093716	0.189245542	0.150119409
ENSRNOG00000028362	Unc80	38.1012352	0.016233389	0.229822702
ENSRNOG00000000692	Ung	26.72961512	0.019947691	0.274217109
ENSRNOG00000054605	Unkl	115.5015857	-0.014108087	0.141092121
ENSRNOG00000020134	Upf1	73.56233246	-0.582104572	0.166712366
ENSRNOG00000023593	Upf2	72.9023818	0.169489438	0.156499908
ENSRNOG00000039994	Upf3b	123.642411	-0.044285397	0.122058582
ENSRNOG00000043324	Upk2	28.0987191	-0.31313411	0.237885606
ENSRNOG00000004972	Upp1	30.89444595	-0.140077167	0.238386097
ENSRNOG00000048309	Uqcc1	116.6528607	-0.139149636	0.129871336

ENSRNOG00000036742	Uqcrc2	85.28866909	0.002440434	0.148671893
ENSRNOG00000002080	Urb1	17.24722138	0.209364624	0.36306005
ENSRNOG00000026853	Urb2	24.46920639	-0.253799123	0.2664648
ENSRNOG00000014463	Uri1	145.8196362	0.023585784	0.126497822
ENSRNOG00000026636	Urm1	74.02678601	0.033561377	0.160800416
ENSRNOG00000018211	Urod	43.36168463	0.111458812	0.20808269
ENSRNOG00000017810	Uros	24.98644991	-0.117441742	0.266201167
ENSRNOG00000016619	Use1	13.35685084	-0.381328332	0.363578212
ENSRNOG00000053725	Usf2	23.83520997	-0.023274724	0.273602074
ENSRNOG00000027756	Usf3	65.42188212	-0.004862441	0.170792687
ENSRNOG00000007890	Usp1	13.66370344	-0.116875561	0.420770872
ENSRNOG00000016509	Usp10	185.1272091	0.142042086	0.106184366
ENSRNOG00000033411	Usp12	43.69354862	0.16454812	0.188519652
ENSRNOG00000030639	Usp13	185.9028725	-0.025716145	0.128942223
ENSRNOG00000014981	Usp14	80.52348458	0.193888675	0.163932113
ENSRNOG00000023202	Usp15	59.26565657	0.367818769	0.187108529
ENSRNOG00000001598	Usp16	48.84455168	-0.016050986	0.189480674
ENSRNOG00000037198	Usp18	17.67640612	0.28435115	0.358678131
ENSRNOG00000049531	Usp19	152.1113465	0.074629627	0.109128804
ENSRNOG00000038347	Usp21	10.52631641	-0.118518248	0.407020185
ENSRNOG00000032492	Usp22	57.39971718	0.465259258	0.179758444
ENSRNOG00000005802	Usp24	29.31973084	0.583580522	0.271226929
ENSRNOG00000001573	Usp25	116.4012759	0.208035716	0.143418039
ENSRNOG00000002844	Usp27x	10.03324139	-0.416948136	0.447421794
ENSRNOG00000007325	Usp28	62.51553777	-0.087518797	0.186718495
ENSRNOG00000015956	Usp29	136.0791773	0.110862217	0.118653171
ENSRNOG00000028556	Usp30	10.62698463	0.442112924	0.401124072
ENSRNOG00000025793	Usp31	70.9169015	0.031671714	0.161461193
ENSRNOG00000051889	Usp34	109.1073241	-0.070795729	0.135726731
ENSRNOG00000024961	Usp35	71.69720916	-0.020766722	0.16983688
ENSRNOG00000028255	Usp36	166.5152087	0.379591574	0.264988972
ENSRNOG00000022720	Usp37	9.149667233	0.474264809	0.469153386
ENSRNOG00000026880	Usp38	64.70247718	0.047893958	0.156400753
ENSRNOG00000010930	Usp39	126.5186544	-0.013589431	0.131738934
ENSRNOG00000054863	Usp4	45.94365579	0.094509917	0.199818512
ENSRNOG00000001059	Usp42	15.32636617	-0.034346893	0.329077855
ENSRNOG00000003785	Usp43	19.22848358	0.2226672	0.320876406
ENSRNOG00000005637	Usp44	49.59969658	-0.157076245	0.195163465
ENSRNOG00000008688	Usp45	133.2429656	0.205157729	0.142915273
ENSRNOG00000026754	Usp47	116.5904888	-0.11149982	0.125244057
ENSRNOG00000015409	Usp5	61.35150173	0.828647976	0.257970102
ENSRNOG00000021434	Usp51	9.67758792	-0.600141155	0.393226882
ENSRNOG00000027012	Usp54	113.1656644	-0.023592435	0.123693884
ENSRNOG00000017644	Usp6nl	63.32241136	0.03130051	0.170405222
ENSRNOG00000025496	Usp7	103.0007878	-0.211624903	0.340241371
ENSRNOG00000010729	Usp8	17.80013225	0.054640699	0.321199421
ENSRNOG00000000909	Uspl1	93.39035747	0.151411741	0.131929935
ENSRNOG00000016381	Ust	15.42721457	-0.094098832	0.320423101
ENSRNOG00000007174	Utp11	178.4590998	-0.079890879	0.111322789
ENSRNOG00000005012	Utp14a	44.60352711	0.005094911	0.224943849

ENSRNOG00000016591	Utp15	17.17889232	0.363120506	0.320852876
ENSRNOG00000002644	Utp18	47.21735701	0.277290546	0.189249079
ENSRNOG00000004789	Utp25	55.98534771	0.512303269	0.195426876
ENSRNOG00000003599	Utp3	145.7095186	-0.01274619	0.138851473
ENSRNOG00000020333	Utp4	112.5773721	-0.146818169	0.141874799
ENSRNOG00000011058	Utrn	171.066672	0.188177126	0.11723641
ENSRNOG00000018393	Uts2	71.25457291	-0.046269854	0.162396409
ENSRNOG00000060617	Uty	47.60050641	-0.257312053	0.215089385
ENSRNOG00000016206	Uvrag	135.7783157	0.000204295	0.120695614
ENSRNOG00000009893	Uxt	34.15907615	0.040274072	0.232710506
ENSRNOG00000017219	Vac14	15.83328131	-0.104707972	0.324833222
ENSRNOG00000019219	Vamp1	22.56032414	0.168494072	0.283913232
ENSRNOG00000008372	Vamp7	34.34558441	-0.210999233	0.251974424
ENSRNOG00000012748	Vamp8	13.36754703	0.240510518	0.357862176
ENSRNOG00000016477	Vangl1	15.81179698	0.362159606	0.31682179
ENSRNOG00000004889	Vangl2	39.40051138	-0.154499401	0.25106345
ENSRNOG00000005331	Vapb	11.26475222	-0.037515297	0.38468496
ENSRNOG00000000833	Vars2	53.18253273	-0.024881777	0.171260036
ENSRNOG00000010457	Vash1	15.670634	0.131947801	0.352566204
ENSRNOG00000003832	Vash2	164.8977156	0.190427891	0.120635938
ENSRNOG00000016367	Vasp	69.32352183	0.095332128	0.210862994
ENSRNOG00000020684	Vat1	10.0778242	-0.232915507	0.380300982
ENSRNOG00000011989	Vat1l	52.24270272	-0.189702286	0.232845698
ENSRNOG00000052373	Vault	75.38107948	-0.229934296	0.161940686
ENSRNOG00000007422	Vav2	28.94273049	0.510473446	0.273276761
ENSRNOG00000020485	Vav3	16.22076199	-0.460061346	0.305522924
ENSRNOG00000014333	Vcam1	150.0180019	-0.124221346	0.12202892
ENSRNOG00000029212	Vcan	44.39418837	-0.111833705	0.205804499
ENSRNOG00000010765	Vcl	26.35253393	-0.271196767	0.263495988
ENSRNOG00000006980	Vcpip1	18.24900432	-0.080367366	0.280863924
ENSRNOG00000021156	Vegfb	48.09484628	-0.245274077	0.187531422
ENSRNOG00000011416	Vegfc	27.73683691	0.317206441	0.273222355
ENSRNOG00000009819	Vezf1	104.7710492	-0.138188368	0.165054967
ENSRNOG00000006514	Vezt	189.0502808	0.075840879	0.120263164
ENSRNOG00000001416	Vgf	119.6483795	-0.232659399	0.222232441
ENSRNOG00000007822	Vgll4	82.49422248	-0.109437293	0.15938373
ENSRNOG00000018087	Vim	141.2846184	-0.017305755	0.167649614
ENSRNOG00000049223	Vipas39	38.59462725	-0.154141699	0.337631606
ENSRNOG00000004317	Vipr2	14.30655582	-0.348542536	0.358008121
ENSRNOG00000014664	Virma	47.03326697	0.073060479	0.246890753
ENSRNOG00000027491	Vldlr	103.7095041	0.062314481	0.134547375
ENSRNOG00000003967	Vmp1	10.06672312	0.099305708	0.38976151
ENSRNOG00000040210	Vom2r69	126.8362632	-0.243255128	0.126060096
ENSRNOG00000010733	Vps11	23.47739382	0.549750999	0.312367787
ENSRNOG00000025539	Vps13a	16.46560625	-0.103125515	0.327083635
ENSRNOG00000030213	Vps13c	101.0540722	0.074757914	0.161810142
ENSRNOG00000016443	Vps13d	146.5513652	0.217354224	0.131884946
ENSRNOG00000021222	Vps16	138.255719	0.206727448	0.139583975
ENSRNOG00000013689	Vps18	125.6918474	0.020142496	0.115065973
ENSRNOG00000030683	Vps26a	29.19746133	-0.223339746	0.248575545

ENSRNOG00000047235	Vps26b	68.33007362	-0.583617086	0.275562102
ENSRNOG00000001681	Vps26c	82.20883601	0.027546736	0.185268755
ENSRNOG00000014633	Vps28	111.7063348	0.250879256	0.167405147
ENSRNOG00000001274	Vps29	18.57665257	-1.739325476	0.390728879
ENSRNOG00000056889	Vps33a	158.9628977	0.040680929	0.10488086
ENSRNOG00000013149	Vps33b	16.37989711	0.411390193	0.310564513
ENSRNOG00000016063	Vps35l	76.65395841	0.188064429	0.146652486
ENSRNOG00000012654	Vps36	65.95334046	-0.04048843	0.162347007
ENSRNOG00000012001	Vps37a	75.93552424	0.150106824	0.17162792
ENSRNOG00000001086	Vps37b	26.8063347	0.189810065	0.264771813
ENSRNOG00000008316	Vps39	101.6366868	0.222884818	0.139857016
ENSRNOG00000012940	Vps41	27.15306209	0.079015399	0.237169652
ENSRNOG00000021173	Vps45	92.58427512	0.351242139	0.171347252
ENSRNOG00000020351	Vps4a	93.76830765	-0.126474625	0.133350426
ENSRNOG00000002705	Vps4b	112.5394513	-0.103789996	0.136743804
ENSRNOG00000000470	Vps52	87.49459329	-0.095350763	0.159348121
ENSRNOG00000007125	Vps54	33.28633212	0.454790776	0.263644678
ENSRNOG00000021081	Vps72	187.670822	-0.091324027	0.105548541
ENSRNOG00000001764	Vps8	143.4511718	0.07425907	0.117857436
ENSRNOG00000028904	Vps9d1	126.2989402	0.083998784	0.165822987
ENSRNOG00000005274	Vrk1	32.74296484	0.134645533	0.249180553
ENSRNOG00000026517	Vrk3	181.4457774	0.013908922	0.109879298
ENSRNOG00000022698	Vsig10	14.01697583	0.196852925	0.406609022
ENSRNOG00000023411	Vsig10l	106.155051	0.272977122	0.140510471
ENSRNOG00000055680	Vsig10l2	32.82052603	0.120607139	0.228218025
ENSRNOG00000000569	Vsir	107.1687063	0.030479695	0.139673081
ENSRNOG00000005180	Vstm2a	45.4596593	0.183495751	0.185677912
ENSRNOG00000034031	Vstm2l	141.2372004	0.114568006	0.136551783
ENSRNOG00000020078	Vstm4	18.28902756	0.100335429	0.28958994
ENSRNOG00000042786	Vti1a	21.97407702	0.099346904	0.303488362
ENSRNOG00000060436	Vti1b	69.94855687	0.116805258	0.154741757
ENSRNOG00000018338	Vwa1	138.329026	0.103905847	0.13502037
ENSRNOG00000025110	Vwa3a	119.7480265	-0.210779765	0.282416521
ENSRNOG00000000195	Vwa5a	21.87482619	0.007101313	0.284275106
ENSRNOG00000016553	Vwa5b1	30.02627292	-0.102570795	0.24339804
ENSRNOG00000001707	Vwa5b2	31.72284226	0.234964826	0.225468801
ENSRNOG00000049076	Vwc2	41.47937733	0.06031715	0.203553099
ENSRNOG00000019689	Vwf	15.52594752	0.670894094	0.358790382
ENSRNOG00000018698	Wac	40.62014542	-0.264207707	0.216847932
ENSRNOG00000052513	Wapl	61.83298272	0.225765055	0.179081804
ENSRNOG00000004359	Wars1	35.91913353	-0.071306376	0.24119722
ENSRNOG00000009805	Wasf2	14.04502014	-0.140767177	0.334328461
ENSRNOG00000048898	Wasf3	18.31246964	0.069575799	0.299122715
ENSRNOG00000053306	Washc1	155.6518014	0.010004802	0.122908452
ENSRNOG00000011653	Washc2c	19.00161924	0.050997328	0.291318836
ENSRNOG00000005102	Washc3	131.5239088	-0.130848721	0.145881441
ENSRNOG00000008331	Washc4	20.09444244	0.138883018	0.282727913
ENSRNOG00000009739	Washc5	25.78586652	0.186808503	0.258717183
ENSRNOG00000008720	Wbp1	41.84201303	-0.400714294	0.799727792
ENSRNOG00000007971	Wbp2	81.38378941	0.034806755	0.14729263

ENSRNOG00000010042	Wdfy2	23.44519664	0.342287958	0.302867256
ENSRNOG00000061121	Wdfy3	144.8474301	-0.337031426	0.147433954
ENSRNOG00000054331	Wdpcp	18.48807197	-0.183080713	0.328491194
ENSRNOG00000020430	Wdr11	157.1964248	0.060635951	0.121342639
ENSRNOG00000017340	Wdr12	124.4880533	-0.077784874	0.129930776
ENSRNOG00000048847	Wdr17	65.41120623	0.222460894	0.214236002
ENSRNOG00000002932	Wdr19	13.56785216	-0.181590503	0.369465375
ENSRNOG00000007333	Wdr20	64.44358856	-0.118827682	0.158682536
ENSRNOG00000015609	Wdr27	20.92886719	-0.435490524	0.271543312
ENSRNOG00000019670	Wdr3	31.07119416	0.396948153	0.236195291
ENSRNOG00000014869	Wdr31	74.70975602	-0.209923692	0.141930574
ENSRNOG00000028783	Wdr35	8.580189692	0.098579225	0.493513672
ENSRNOG00000027355	Wdr36	58.41946254	0.327985836	0.184642202
ENSRNOG00000001181	Wdr4	34.96739658	0.454716664	0.225668069
ENSRNOG00000026316	Wdr43	76.64206615	-0.014908935	0.164149417
ENSRNOG00000029068	Wdr44	59.48682783	-0.152711322	0.15841132
ENSRNOG00000009749	Wdr45	97.51956063	-0.084637163	0.211393832
ENSRNOG00000036662	Wdr45b	186.4804488	-0.00783267	0.122770963
ENSRNOG00000031171	Wdr46	10.18291726	-0.220961022	0.473323012
ENSRNOG00000016942	Wdr48	13.79222919	0.708709425	0.365783349
ENSRNOG00000008212	Wdr5	11.04192459	-0.277538552	0.39987817
ENSRNOG00000001754	Wdr53	51.43367833	0.222139475	0.20581037
ENSRNOG00000009324	Wdr54	38.51147956	-0.643429389	0.721298481
ENSRNOG00000018967	Wdr59	9.6271275	-0.071911782	0.399824982
ENSRNOG00000002253	Wdr5b	9.518000465	0.231496371	0.491689966
ENSRNOG00000013336	Wdr70	96.91452989	0.087550128	0.139880267
ENSRNOG00000010664	Wdr73	92.08798484	-0.097053928	0.150150621
ENSRNOG00000042878	Wdr74	24.77781824	0.002195772	0.295978306
ENSRNOG00000003825	Wdr75	66.84705242	0.119413361	0.176366178
ENSRNOG00000022491	Wdr76	69.3406437	0.218811368	0.175280367
ENSRNOG00000048441	Wdr82	9.31554402	-0.53931389	0.41331044
ENSRNOG00000021628	Wdr89	8.849917064	-0.15522151	0.524211718
ENSRNOG00000020006	Wdr90	9.564111905	0.160302799	0.448530108
ENSRNOG00000010421	Wdr91	20.1634421	0.68262513	0.377879681
ENSRNOG00000000904	Wdr95	10.2990406	0.286416175	0.392399796
ENSRNOG00000005990	Wdsub1	78.95158929	-0.099742806	0.163429766
ENSRNOG00000010017	Wee1	141.0217285	-0.044189572	0.120420119
ENSRNOG00000015904	Wfdc1	123.3163499	0.076196146	0.189396898
ENSRNOG00000042162	Wfdc3	78.51296574	-0.036124239	0.164412948
ENSRNOG00000028113	Whamm	83.2966605	0.181352465	0.149163104
ENSRNOG00000001700	Whrn	182.7488061	0.088865393	0.09989824
ENSRNOG00000004476	Wif1	28.3400806	0.244290274	0.235051402
ENSRNOG00000018406	Wipf1	51.79224958	0.342546962	0.24778647
ENSRNOG00000027849	Wipf2	25.0176332	0.245938988	0.283703308
ENSRNOG00000003827	Wipi1	12.81845711	0.467361589	0.401290079
ENSRNOG00000001114	Wipi2	140.2750606	0.109750833	0.153943582
ENSRNOG00000006217	Wiz	38.85898577	-0.234192892	0.197848932
ENSRNOG00000009956	Wnk1	125.6490622	0.020833621	0.178292517
ENSRNOG00000052510	Wnt10a	37.76633414	0.356614763	0.239957608
ENSRNOG00000005781	Wnt16	12.91778033	0.660633086	0.415374832

ENSRNOG00000051905	Wnt2	12.27099405	-0.051354445	0.359789868
ENSRNOG00000003845	Wnt3	12.27124596	0.138459473	0.347040623
ENSRNOG00000013166	Wnt4	134.5399651	0.134933737	0.142569451
ENSRNOG00000015618	Wnt5a	11.68636499	0.452731832	0.415546205
ENSRNOG00000008168	Wnt5b	17.00469396	0.04526591	0.402569882
ENSRNOG00000017409	Wnt6	13.49295919	-0.139911759	0.493240234
ENSRNOG00000048782	Wnt7a	172.514764	0.120250477	0.109438109
ENSRNOG00000003807	Wnt9b	8.702132804	-1.661243109	1.185799835
ENSRNOG00000015440	Wrn	8.648322378	-0.154907339	0.506826324
ENSRNOG00000012929	Wsb1	10.12613588	-0.017912909	0.429378871
ENSRNOG00000001135	Wsb2	30.16620653	-0.448645514	0.317528262
ENSRNOG00000053045	Wscd2	9.166955397	1.048267959	0.44900682
ENSRNOG00000023296	Wtip	10.3174749	0.286153446	0.434017759
ENSRNOG00000006328	Wwp1	50.9981127	0.045219011	0.20877715
ENSRNOG00000012937	Wwp2	42.16047014	0.058412261	0.191567168
ENSRNOG00000010298	Xbp1	69.10308562	-0.044582503	0.164624666
ENSRNOG00000007081	Xdh	22.20834926	0.081724882	0.292985479
ENSRNOG00000003749	Xk	113.5503212	-0.029497935	0.187738207
ENSRNOG00000027276	Xkr4	16.2059773	0.056434425	0.301063763
ENSRNOG00000011634	Xkr6	23.71052421	-0.001065251	0.338710411
ENSRNOG00000024312	Xkr8	105.2393274	0.151124556	0.135787488
ENSRNOG00000009576	Xpa	25.13590001	-0.414177956	0.242165413
ENSRNOG00000008274	Xpc	12.96427961	-0.161336046	0.427658866
ENSRNOG00000019196	Xpnpep3	32.70855443	-0.090442966	0.234803046
ENSRNOG00000009935	Xpo1	123.4135985	0.056115108	0.118257555
ENSRNOG00000010137	Xpo4	41.78227523	0.00042575	0.199492498
ENSRNOG00000019085	Xpo5	17.50106655	0.648259385	0.526377791
ENSRNOG00000007088	Xpot	20.17982301	-0.043563544	0.30358795
ENSRNOG00000000042	Xpr1	10.99535554	-0.044423333	0.395458135
ENSRNOG00000007493	Xrcc2	8.896492575	1.205110061	0.513213323
ENSRNOG00000029966	Xrcc4	43.60093492	-0.00626784	0.197915117
ENSRNOG00000016105	Xrcc5	72.58525735	0.004447916	0.163260365
ENSRNOG00000042951	Xrn1	45.09094409	0.181883016	0.28428933
ENSRNOG00000026503	Xrra1	53.97857506	0.143604711	0.206912566
ENSRNOG00000001729	Xxylt1	58.80694965	-0.007285587	0.186690178
ENSRNOG00000014168	Xylb	47.80733755	0.362797642	0.188972636
ENSRNOG00000056658	Xylt1	7.826333186	0.136408335	0.561538934
ENSRNOG00000003728	Xylt2	34.41196626	0.09028257	0.23704229
ENSRNOG00000013438	Yae1	111.3469927	-0.023498429	0.133252275
ENSRNOG00000007213	Yars1	12.63914366	0.117102349	0.362306102
ENSRNOG00000032902	Ybx1-ps3	114.551145	0.005490502	0.154785114
ENSRNOG00000001861	Ydjc	11.03700575	-0.797283237	0.985933125
ENSRNOG00000005689	Yeats4	51.34161549	-0.859270821	0.579224638
ENSRNOG00000037227	Yes1	44.29780803	0.131306393	0.209112404
ENSRNOG00000055286	Yif1b	10.9352157	0.414004728	0.40164712
ENSRNOG00000018998	Yipf3	23.00564688	0.833041776	0.400372401
ENSRNOG00000006642	Yipf6	55.24554172	-0.073908623	0.178275325
ENSRNOG00000014785	Ykt6	102.3803034	0.024063632	0.134928195
ENSRNOG00000055012	Yme1l1	58.33051912	0.040913099	0.183041535
ENSRNOG00000005447	Ypel2	15.12941902	0.235894656	0.328133504

ENSRNOG00000019721	Ypel3	27.42034007	0.028971752	2.770004779
ENSRNOG00000025424	Yrdc	144.7863359	0.119903105	0.110240473
ENSRNOG00000001996	Ythdc1	156.9998257	-0.058990692	0.122533519
ENSRNOG00000039241	Ythdc2	9.916474227	0.345116407	0.466110651
ENSRNOG00000010084	Ythdf3	45.06622969	-0.089101532	0.2198285
ENSRNOG00000004339	Yy1	54.16286801	0.12987245	0.190772654
ENSRNOG00000016239	Zadh2	118.5263767	-0.074043003	0.126858331
ENSRNOG00000016995	Zap70	106.1878505	0.113936536	0.12506011
ENSRNOG00000026150	Zar1	42.89342422	0.218348225	0.196452734
ENSRNOG00000028941	Zbed3	41.07692015	0.240272021	0.206292382
ENSRNOG00000004588	Zbed4	43.76139834	0.428196364	0.234435771
ENSRNOG00000058593	Zbtb1	19.94036746	0.118129776	0.328774486
ENSRNOG00000001613	Zbtb11	158.7547728	0.058892641	0.134831102
ENSRNOG00000029980	Zbtb16	53.91105425	0.065664227	0.196939534
ENSRNOG00000010436	Zbtb17	9.129434789	-0.347219426	0.417663707
ENSRNOG00000004423	Zbtb18	56.45699908	-0.297433092	0.250534516
ENSRNOG00000019544	Zbtb2	40.99247108	-0.06923273	0.239354283
ENSRNOG00000056716	Zbtb20	128.125651	0.261902984	0.134043868
ENSRNOG00000001623	Zbtb21	49.67802483	0.17196732	0.205628847
ENSRNOG00000000476	Zbtb22	10.22808131	-2.472210321	1.205964303
ENSRNOG00000000308	Zbtb24	45.10212361	0.044816824	0.206032857
ENSRNOG00000019461	Zbtb3	133.5341765	-0.171825232	0.123167438
ENSRNOG00000028899	Zbtb33	185.3079797	0.082845938	0.116572217
ENSRNOG00000032700	Zbtb34	57.57665396	0.265120558	0.186765532
ENSRNOG00000012386	Zbtb38	15.80620731	-0.605164677	0.319014036
ENSRNOG00000014689	Zbtb4	8.306929319	0.297398667	0.496587455
ENSRNOG00000022039	Zbtb40	11.76621553	0.683977189	0.422747191
ENSRNOG00000005578	Zbtb44	16.47728216	-0.301445145	0.322872799
ENSRNOG00000027459	Zbtb45	175.6914726	0.060009583	0.118360297
ENSRNOG00000014592	Zbtb46	48.73512218	0.449294008	0.20350102
ENSRNOG00000019382	Zbtb47	27.14979332	0.476553699	0.268266552
ENSRNOG00000005633	Zbtb49	24.26362581	0.187804915	0.261377648
ENSRNOG00000009340	Zbtb6	22.52223221	-0.41559407	0.279894887
ENSRNOG00000020161	Zbtb7a	75.32137158	-0.489299992	0.174445615
ENSRNOG00000047924	Zbtb7c	8.940503667	7.82908E-05	0.446263775
ENSRNOG00000008300	Zbtb8a	13.02404242	0.373001009	0.371973563
ENSRNOG00000026898	Zbtb8b	7.826747231	0.585041626	0.558080872
ENSRNOG00000008226	Zbtb8os	59.67612701	-0.054164289	0.190981533
ENSRNOG00000022356	Zc2hc1a	163.6818133	-0.057203153	0.113602462
ENSRNOG00000027115	Zc2hc1c	97.29022322	0.050208055	0.152205832
ENSRNOG00000040281	Zc3h10	84.18722832	-0.020034644	0.148786816
ENSRNOG00000053210	Zc3h11a	26.39155783	0.175281647	0.262478661
ENSRNOG00000009131	Zc3h12a	72.81650307	0.139700227	0.173332668
ENSRNOG00000004083	Zc3h14	63.39183091	0.039974461	0.178423184
ENSRNOG00000028501	Zc3h18	91.59993966	-0.132693878	0.161956646
ENSRNOG00000007684	Zc3h3	99.36888473	-0.064936202	0.12975858
ENSRNOG00000015229	Zc3h4	140.5520493	0.104643751	0.121125678
ENSRNOG00000026277	Zc3h6	60.1521484	0.215034187	0.189084499
ENSRNOG00000002420	Zc3h7a	33.68993565	0.085659744	0.219712134
ENSRNOG00000017647	Zc3h8	104.0439764	0.165225145	0.137787975

ENSRNOG00000013948	Zc3hav1	21.07673759	0.620960079	0.285561712
ENSRNOG00000010154	Zc3hc1	91.08669456	-0.167223628	0.137688465
ENSRNOG00000056735	Zc4h2	127.467885	0.035452931	0.133233444
ENSRNOG00000018226	Zcchc14	17.88284018	0.28333065	0.307781776
ENSRNOG00000012266	Zcchc17	34.70292829	-0.05818996	0.27232768
ENSRNOG00000055939	Zcchc18	181.6219326	-0.154248112	0.112674698
ENSRNOG00000002882	Zcchc2	17.02963323	0.685277202	0.350512298
ENSRNOG00000029064	Zcchc4	19.22917247	0.203370604	0.298001296
ENSRNOG00000013078	Zcchc7	64.95460506	0.301797845	0.201409561
ENSRNOG00000001243	Zcchc8	45.73866629	0.17718916	0.210954678
ENSRNOG00000051682	Zcchc9	125.7168053	0.031945859	0.13494749
ENSRNOG00000024114	Zdbf2	74.710831	0.087947374	0.213771581
ENSRNOG00000014277	Zdhhc13	19.93943414	0.078631396	0.287601781
ENSRNOG00000046530	Zdhhc16	7.777100212	-0.533707641	0.435771658
ENSRNOG00000003803	Zdhhc17	11.56244129	-0.459556743	0.381914096
ENSRNOG00000058156	Zdhhc18	36.63710516	-0.168078923	0.227754453
ENSRNOG00000011024	Zdhhc20	79.98498815	-0.070631076	0.215045288
ENSRNOG00000010484	Zdhhc21	10.28989044	-0.201892336	0.396202915
ENSRNOG00000011285	Zdhhc22	47.31693375	0.09092575	0.178793868
ENSRNOG00000019825	Zdhhc24	61.37167332	-0.134887459	0.219998446
ENSRNOG00000004344	Zdhhc3	182.6988852	-0.091116631	0.130502901
ENSRNOG00000051699	Zdhhc4	26.97183621	0.116216931	0.26207165
ENSRNOG00000006832	Zdhhc5	21.6440865	0.400749552	0.309954571
ENSRNOG00000036576	Zdhhc6	14.8716353	0.410459815	0.336253134
ENSRNOG00000021891	Zdhhc8	14.123063	0.144635104	0.345553192
ENSRNOG00000017863	Zeb1	58.44580679	0.223561689	0.17221872
ENSRNOG00000004677	Zeb2	42.20032081	0.180896823	0.239158288
ENSRNOG00000025832	Zer1	58.49239705	0.159896884	0.175583346
ENSRNOG00000032917	Zfand2a	111.8200411	0.115806191	0.121189852
ENSRNOG00000025140	Zfat	32.13107582	-0.443152943	0.243990575
ENSRNOG00000053060	Zfc3h1	43.79179495	0.233421356	0.22949371
ENSRNOG00000025634	Zfhx2	120.7330067	0.146280545	0.151076546
ENSRNOG00000014452	Zfhx3	12.6821763	0.066887829	0.415592449
ENSRNOG00000008765	Zfhx4	54.3769313	-0.042069051	0.207279164
ENSRNOG00000019059	Zfp1	25.14175381	0.23931714	0.248300621
ENSRNOG00000052583	Zfp106	175.0945345	0.028485067	0.155756112
ENSRNOG00000051442	Zfp11	36.79719377	0.09643766	0.218408157
ENSRNOG00000031328	Zfp110	24.29553425	0.436585347	0.309337966
ENSRNOG00000024376	Zfp111	20.14830317	0.537660767	0.307097772
ENSRNOG00000029022	Zfp112	21.6249981	-0.367598285	0.267948319
ENSRNOG00000031459	Zfp128	66.4543334	0.353972913	0.16470744
ENSRNOG00000015925	Zfp131	117.4504775	0.158848232	0.151268665
ENSRNOG00000030932	Zfp14	64.10235704	0.269511563	0.196073768
ENSRNOG00000022414	Zfp142	20.00779073	0.056106629	0.289380956
ENSRNOG00000010087	Zfp143	45.31075093	0.030268188	0.205093966
ENSRNOG00000001323	Zfp157	61.97143933	-0.132725629	0.196926783
ENSRNOG00000059492	Zfp160	34.53852761	-0.275644401	0.202439843
ENSRNOG00000016719	Zfp161	83.46536444	0.170085121	0.147426838
ENSRNOG00000017092	Zfp169	91.1070875	0.0246065	0.134274261
ENSRNOG00000007655	Zfp174	18.28937786	0.035957426	0.360744731

ENSRNOG00000029336	Zfp180	22.51803327	-0.128268254	0.269823497
ENSRNOG00000061612	Zfp184	22.02605781	0.095653952	0.278362188
ENSRNOG00000006972	Zfp189	25.95415752	-0.006451949	0.254317936
ENSRNOG00000030750	Zfp2	11.56657415	0.522534251	0.381585059
ENSRNOG00000058932	Zfp202	20.70606385	0.076510804	0.289981432
ENSRNOG00000000236	Zfp207	103.5852635	-0.076276072	0.134114591
ENSRNOG00000006711	Zfp212	14.67508275	0.214388397	0.472917043
ENSRNOG00000021872	Zfp213	171.1877291	-0.05515412	0.104618048
ENSRNOG00000016303	Zfp236	16.95165248	0.001473537	0.320681998
ENSRNOG00000016562	Zfp24	30.1547541	0.084190833	0.240462177
ENSRNOG00000037251	Zfp248	19.09302491	0.005754069	0.313753097
ENSRNOG00000030308	Zfp251	123.742987	0.019884598	0.114698255
ENSRNOG00000025937	Zfp26	11.74916929	0.076580234	0.383032102
ENSRNOG00000034037	Zfp266	82.21857211	-0.029227725	0.147578096
ENSRNOG00000042283	Zfp267	24.77173589	-0.44235592	0.321864618
ENSRNOG00000061073	Zfp275	36.54400703	0.178485158	0.221994696
ENSRNOG00000016631	Zfp276	9.621214516	-0.184860965	0.423154657
ENSRNOG00000045854	Zfp277	85.97202261	-0.226943184	0.162485963
ENSRNOG00000028441	Zfp280b	23.54358388	0.437653947	0.293505563
ENSRNOG00000006797	Zfp280c	32.99954094	-0.232201579	0.239715212
ENSRNOG00000055631	Zfp280d	144.3087725	0.207813256	0.133278393
ENSRNOG00000058643	Zfp281	140.6267299	0.035474988	0.14264016
ENSRNOG00000026981	Zfp282	94.81760634	0.26843623	0.151458522
ENSRNOG00000031031	Zfp292	101.5104847	0.116878851	0.131511799
ENSRNOG00000005009	Zfp3	28.16317301	0.571546056	0.334998648
ENSRNOG00000006684	Zfp317	15.96735698	-0.101239343	0.397062299
ENSRNOG00000018741	Zfp318	61.90326871	0.071646995	0.169482521
ENSRNOG00000017318	Zfp322a	26.02812701	0.091772382	0.281599792
ENSRNOG00000047761	Zfp326	71.71790008	-0.118842947	0.218037779
ENSRNOG00000003461	Zfp330	181.9296666	-0.025287336	0.112302538
ENSRNOG00000017290	Zfp335	29.3738362	-0.087958341	0.245895627
ENSRNOG00000016867	Zfp346	126.9071311	0.118647761	0.137582955
ENSRNOG00000049137	Zfp35	34.81728421	0.289802345	0.267944175
ENSRNOG00000003634	Zfp354a	32.19514475	-0.072308257	0.247035917
ENSRNOG00000029205	Zfp354c	8.600920881	1.133158027	0.504943986
ENSRNOG00000043364	Zfp362	13.99687704	0.094628592	0.356094541
ENSRNOG00000058646	Zfp36l1	172.7136866	-0.000463673	0.116537339
ENSRNOG00000020774	Zfp383	99.5551474	0.223406889	0.162972158
ENSRNOG00000017066	Zfp384	18.96462868	-1.015882647	0.473132754
ENSRNOG00000036833	Zfp385a	18.01533917	0.224150774	0.311523105
ENSRNOG00000019065	Zfp385b	131.405878	-0.028827888	0.143448517
ENSRNOG00000025370	Zfp385c	48.64758688	-0.22037043	0.179514642
ENSRNOG00000014661	Zfp385d	27.63657961	0.074002456	0.275448943
ENSRNOG00000004268	Zfp386	36.50864089	0.069529638	0.211043731
ENSRNOG00000014712	Zfp39	24.63216079	-0.325272951	0.273118972
ENSRNOG00000048101	Zfp397	25.35510509	0.064354022	0.294908795
ENSRNOG00000006703	Zfp398	41.9709644	-0.26058545	0.248833503
ENSRNOG00000011675	Zfp40	32.40368529	0.059521661	0.215696887
ENSRNOG00000043357	Zfp407	41.1719634	-0.31551172	0.207941416
ENSRNOG00000010985	Zfp410	54.60761979	0.258139836	0.205573132

ENSRNOG00000008378	Zfp414	89.88680289	-0.187287113	0.152650799
ENSRNOG00000037556	Zfp420	89.3495224	0.539606638	0.471060956
ENSRNOG00000013379	Zfp422	111.5044359	-0.180707872	0.133422449
ENSRNOG00000014658	Zfp423	119.8150694	0.033541238	0.149290961
ENSRNOG00000033624	Zfp426	43.204918	-0.072539802	0.198213088
ENSRNOG00000028329	Zfp438	14.33853747	-0.066084959	0.403659743
ENSRNOG00000012718	Zfp451	70.72449226	0.093652726	0.152550123
ENSRNOG00000031576	Zfp455	12.82070632	-0.436309032	0.340229891
ENSRNOG00000032048	Zfp462	31.31465336	-0.009251533	0.242947351
ENSRNOG00000007707	Zfp467	55.84404986	0.22961111	0.183053447
ENSRNOG00000004572	Zfp472	11.6153083	-0.60107741	0.43567435
ENSRNOG00000014975	Zfp483	9.973163289	-0.080246659	0.478932128
ENSRNOG00000003129	Zfp496	54.09901141	0.032779002	0.16920155
ENSRNOG00000014237	Zfp503	16.8434805	-0.014750184	0.387022555
ENSRNOG00000013565	Zfp507	48.93615961	-0.221530196	0.177428897
ENSRNOG00000056036	Zfp51	13.49961758	0.084263155	0.336052561
ENSRNOG00000018272	Zfp511	125.7877191	-0.010573037	0.121780694
ENSRNOG00000039815	Zfp512	13.86570604	1.048631639	0.404973347
ENSRNOG00000036592	Zfp518a	17.34797542	-0.217636157	0.306981102
ENSRNOG00000016874	Zfp521	17.24244893	0.018776339	0.323795484
ENSRNOG00000000501	Zfp523	16.84658509	-0.004133214	0.296555838
ENSRNOG00000017116	Zfp532	127.8791235	0.158375781	0.122298826
ENSRNOG00000014163	Zfp536	20.16620786	-0.461525726	0.28963943
ENSRNOG00000048394	Zfp560	56.77645453	0.1492495	0.177280477
ENSRNOG00000055761	Zfp574	172.3963166	0.087196105	0.113043581
ENSRNOG00000016608	Zfp579	68.35547027	-0.054376652	0.185489652
ENSRNOG00000034184	Zfp583	33.49561097	0.034974338	0.251670046
ENSRNOG00000060206	Zfp592	102.7116013	0.060654102	0.136099492
ENSRNOG00000016392	Zfp593	99.84599434	0.120711918	0.153289906
ENSRNOG00000007576	Zfp597	8.787130387	0.250554131	0.53104174
ENSRNOG00000012434	Zfp598	9.964416082	0.51282615	0.440706495
ENSRNOG00000018885	Zfp60	125.1977221	-0.003751338	0.1673074
ENSRNOG00000037428	Zfp605	26.30998814	0.370309555	0.27519289
ENSRNOG00000023197	Zfp608	32.36494778	0.095164918	0.226151108
ENSRNOG00000016971	Zfp612	61.05264526	0.05498164	0.18278084
ENSRNOG00000038622	Zfp628	8.750501694	-0.081190566	0.542489784
ENSRNOG00000018877	Zfp629	132.0177714	-0.255212228	0.130339188
ENSRNOG00000014501	Zfp638	135.8427235	-0.122460072	0.144502321
ENSRNOG00000043053	Zfp639	43.10342875	-0.181973671	0.227709819
ENSRNOG00000012762	Zfp64	62.69050554	0.159495623	0.167128305
ENSRNOG00000002112	Zfp644	167.8703211	0.045396163	0.104477243
ENSRNOG00000026115	Zfp646	30.83267414	-0.069946797	0.228373028
ENSRNOG00000017001	Zfp652	23.78637212	-0.052571738	0.287303992
ENSRNOG00000014065	Zfp653	37.69460619	0.061393804	0.224733161
ENSRNOG00000060129	Zfp655	118.0442935	-0.150189349	0.133917157
ENSRNOG00000033906	Zfp667	43.58170735	0.146407664	0.255754336
ENSRNOG00000016683	Zfp668	94.87126739	-0.117729713	0.141575925
ENSRNOG00000002713	Zfp672	64.11306056	0.203939388	0.168638919
ENSRNOG00000001317	Zfp68	136.8421863	0.085543234	0.119658629
ENSRNOG00000018379	Zfp688	60.68454838	0.15706319	0.199082772

ENSRNOG00000007398	Zfp691	45.9150659	-0.431724661	0.185137638
ENSRNOG00000002682	Zfp692	10.40903068	0.113037715	0.424500119
ENSRNOG00000050695	Zfp7	19.70207014	0.165329294	0.294998305
ENSRNOG00000014199	Zfp703	164.0501806	0.03685029	0.119511589
ENSRNOG00000057901	Zfp707	56.75228988	-0.045775777	0.177641077
ENSRNOG00000016186	Zfp709	10.71731401	0.350129109	0.43394563
ENSRNOG00000014278	Zfp710	18.65011249	-0.08516551	0.321577382
ENSRNOG00000007064	Zfp746	127.7017418	-0.090607483	0.118729566
ENSRNOG00000049259	Zfp748	11.60423693	-0.180799789	0.397464467
ENSRNOG00000043225	Zfp771	28.04468264	-0.009222126	0.266271753
ENSRNOG00000008362	Zfp775	173.4371117	0.089608806	0.126367199
ENSRNOG00000033639	Zfp777	149.530624	0.098779655	0.115107545
ENSRNOG00000042777	Zfp78	12.02178908	-0.123424617	0.365317429
ENSRNOG00000006925	Zfp786	12.10056526	0.217901458	0.370213967
ENSRNOG00000015456	Zfp787	78.12053779	-0.086003831	0.167575484
ENSRNOG00000023920	Zfp791	10.32460326	0.280236027	0.40416094
ENSRNOG00000007662	Zfp800	43.4206822	0.063919145	0.18491166
ENSRNOG00000038004	Zfp804a	18.87438613	-0.329762112	0.30104913
ENSRNOG00000007578	Zfp830	31.76666108	0.007121817	0.238946141
ENSRNOG00000024693	Zfp831	7.932568755	0.048517533	0.590095253
ENSRNOG00000031661	Zfp839	14.72459826	-0.095876011	0.336643645
ENSRNOG00000046652	Zfp84	8.629902596	0.28866469	0.48325993
ENSRNOG00000029043	Zfp84	114.1960386	0.034912255	0.127810225
ENSRNOG00000020356	Zfp846	22.33095771	0.138407544	0.299643894
ENSRNOG00000016279	Zfp865	21.18955311	0.101188051	0.268490134
ENSRNOG00000022431	Zfp867	26.41892635	-0.002323695	0.251415028
ENSRNOG00000028919	Zfp868	10.84514747	0.263825495	0.470912956
ENSRNOG00000004947	Zfp871	19.46370188	-0.68677514	0.350549027
ENSRNOG00000020087	Zfp90	42.81921155	0.01985306	0.214146042
ENSRNOG00000051836	Zfp92	46.86265287	-0.153054826	0.189018316
ENSRNOG00000033583	Zfp938	15.44379734	0.286065494	0.326603095
ENSRNOG00000043223	Zfp949	8.546540793	-0.093554895	0.426364845
ENSRNOG00000022742	Zfp954	34.82541161	-0.016485032	0.214604363
ENSRNOG00000048577	Zfp955a	121.0542418	-0.04334174	0.139556401
ENSRNOG00000026874	Zfp956	46.1149344	0.072377575	0.202682063
ENSRNOG00000030410	Zfp958	32.30898929	0.170828828	0.241117441
ENSRNOG00000000891	Zfp958l1	8.338405434	0.013499135	0.437935576
ENSRNOG00000043077	Zfpm1	89.45537572	0.062080872	0.153624529
ENSRNOG00000004109	Zfpm2	87.25416192	-0.238132563	0.170072041
ENSRNOG00000005624	Zfx	36.14374045	-0.332851774	0.239313746
ENSRNOG00000008614	Zfyve1	16.95043691	-0.299281788	0.320079413
ENSRNOG00000012528	Zfyve19	11.27554694	0.650833927	0.414459377
ENSRNOG00000059875	Zfyve26	14.36608216	0.537619075	0.339130703
ENSRNOG00000014874	Zfyve28	133.568318	0.252556979	0.115491468
ENSRNOG00000027183	Zfyve9	61.03526173	0.024075159	0.169116055
ENSRNOG00000036980	Zgrf1	9.008027092	-0.91031521	1.651202703
ENSRNOG00000006412	Zhx1	135.9464264	0.034835342	0.130513645
ENSRNOG00000005417	Zhx2	10.86322683	-0.13714827	0.371708162
ENSRNOG00000027988	Zhx3	27.28558747	0.347426591	0.295940555
ENSRNOG00000054879	Zic2	9.332771497	-0.303325508	0.445592031

ENSRNOG00000000861	Zic3	35.03093941	-0.162206512	0.23868746
ENSRNOG00000014871	Zic4	26.4956793	0.119693513	0.275101346
ENSRNOG00000014358	Zic5	9.502245341	0.333298859	0.405536133
ENSRNOG00000024245	Zkscan2	23.05625201	-0.01016446	0.288964112
ENSRNOG00000055000	Zkscan3	87.22213781	-0.01870364	0.135857348
ENSRNOG00000059552	Zkscan5	69.24200301	-0.172364081	0.175988358
ENSRNOG00000004104	Zkscan7	18.4511263	0.033489276	0.313837704
ENSRNOG00000053635	Zkscan8	13.30868622	0.141778378	0.375216904
ENSRNOG00000024135	Zmat1	69.16813497	-0.235743996	0.153405765
ENSRNOG00000042690	Zmat4	84.92253377	-0.347038312	0.171093808
ENSRNOG00000012054	Zmpste24	9.105966826	-0.50195239	0.386134288
ENSRNOG00000013855	Zmym1	42.92644797	0.103122712	0.206570534
ENSRNOG00000008734	Zmym2	45.23758445	-0.138888076	0.208597935
ENSRNOG00000003707	Zmym3	145.1380698	-0.059323274	0.10938321
ENSRNOG00000012397	Zmym4	29.59464139	0.177562196	0.303968963
ENSRNOG00000029220	Zmym5	97.69645164	-0.030008542	0.144034797
ENSRNOG00000013865	Zmym6	14.18280652	-0.115764604	0.450961278
ENSRNOG00000022199	Zmynd12	24.75670304	0.237605799	0.349845428
ENSRNOG00000029678	Znf454	16.62198317	-0.288351346	0.355912447
ENSRNOG00000017837	Znf48	11.9000369	-0.070682524	0.354189479
ENSRNOG00000029490	Znf768	9.670917658	0.565525309	0.44717967
ENSRNOG00000027857	Znhit3	26.91055186	-0.073903413	0.254893808
ENSRNOG00000030049	Znhit6	92.27487668	0.014420099	0.160791741
ENSRNOG00000012736	Znrd2	9.826481694	-0.210939471	0.416594239
ENSRNOG00000019028	Znrf1	93.15959135	0.00150316	0.1425487
ENSRNOG00000049057	Znrf2	117.6671756	-0.196028548	0.1207496
ENSRNOG00000027506	Zp4	11.47727357	0.747072086	0.507519067
ENSRNOG00000018481	Zpr1	21.7936835	0.284892974	0.275696208
ENSRNOG00000017294	Zranb1	112.0386743	-0.130256338	0.128680743
ENSRNOG00000003979	Zranb3	48.63519207	0.092828896	0.200200756
ENSRNOG00000009639	Zrsr1	98.25212557	-0.145161377	0.141543874
ENSRNOG00000052959	Zscan12	48.35754012	0.128833704	0.21561542
ENSRNOG00000022164	Zscan2	14.89728406	-0.307500392	0.335341001
ENSRNOG00000005886	Zscan20	10.92096121	0.462216836	0.447076075
ENSRNOG00000039234	Zscan21	68.97151091	-0.063522853	0.156007025
ENSRNOG00000012419	Zscan29	34.14067348	0.036649327	0.22399167
ENSRNOG00000030131	Zscan30	22.62823946	0.242539519	0.252361338
ENSRNOG00000007582	Zswim4	40.06121156	0.478663188	0.230645871
ENSRNOG00000056617	Zswim8	11.34448278	0.042161127	0.395798134
ENSRNOG00000000398	Zup1	29.3665262	-0.45450518	0.231950922
ENSRNOG00000054479	Zw10	39.23782308	0.405493387	0.234633688
ENSRNOG00000025818	Zxdc	33.51121315	0.282810341	0.235102386
ENSRNOG00000010885	Zyg11a	26.36836622	0.031123534	0.410653375
ENSRNOG00000010859	Zyg11b	28.24386375	0.044271174	0.274161175
ENSRNOG00000024535	Zzef1	30.80181479	0.035637461	0.221645807
ENSRNOG00000050321	Zzz3	32.38870284	0.134532969	0.238196796

stat	pvalue	padj
-7.29172312	3,06015572916524e-13	0.000000000986900223
-6.569570328	5,04606577494861e-11	0.000000081367810621
-5.044343367	4,55081442772431e-07	0,00048921255098036З
-4.791840304	1,65258423297811e-06	0,00106591683027088
-4.793732161	1,63706807971346e-06	0,00106591683027088
-4.420285709	9,85704903505482e-06	0,00529816385634196
-4.347202141	1,37885132414547e-05	0,00555849440046142
4.373237224	1,22417575002209e-05	0,00555849440046142
-4.188648678	2,80620436387441e-05	0,00973203035015603
-4.161727281	3,15849422739001e-05	0,00973203035015603
-4.149845562	3,32699671216837e-05	0,00973203035015603
-4.130406687	3,62122059540689e-05	0,00973203035015603
-3.933904199	8,35771057980473e-05	0,0192525832999073
-3.88402123	0,000102742930710407	0,021721221978385
-3.82816033	0,000129104648894594	0,0244919113344156
-3.761867592	0,000168649327896748	0,0300576938799299
-3.715871057	0,000202504840565181	0,0310989576582243
-3.722992042	0,000196875729280435	0,0310989576582243
3.695077419	0,000219819797775793	0,0322235839921333
-3.608252746	0,000308266106607979	0,0395538119091877
-3.553454346	0,000380207184243355	0,0395538119091877
-3.609152928	0,000307198479945265	0,0395538119091877
3.5624824	0,000367364543391132	0,0395538119091877
-3.553658656	0,000379911964486675	0,0395538119091877
-3.558853818	0,000372476762650959	0,0395538119091877
-3.553724859	0,000379816350053113	0,0395538119091877
-3.564235903	0,000364917656816213	0,0395538119091877
-3.590733802	0,000329748285236504	0,0395538119091877
-3.543577578	0,000394737156643074	0,0397821040679348
-3.48709877	0,000488290989993031	0,0460330716452918
-3.469068755	0,000522265778638758	0,0460330716452918
3.466067147	0,000528131364612651	0,0460330716452918
-3.477652692	0,000505824860565191	0,0460330716452918
-3.47382015	0,000513104833401961	0,0460330716452918
-3.454972519	0,000550348914296122	0,0467072433843419
-3.446591384	0,000567706527349989	0,0469449628385568
-3.398318929	0,000678013287302654	0,0546648212887765
-3.374208554	0,000740282593730078	0,0572693712208766
-3.372151657	0,000745833671713742	0,0572693712208766
3.362299941	0,00077296113339054	0,0579720850042905
-3.354039623	0,000796409577792927	0,0583732020086861
-3.323054788	0,000890374249833671	0,0638101545714131
-3.315463442	0,000914913070355188	0,0641433619977279
-3.307854534	0,000940136292471565	0,0645093519834212
-3.299888615	0,000967232092277802	0,0649859061999148
3.271223698	0,00107083170340251	0,0682655096205894
-3.258030673	0,00112188279376473	0,0682655096205894
-3.278727509	0,00104276272120739	0,0682655096205894
3.264275869	0,00109744253230868	0,0682655096205894

3.26234838	0,00110493262207515	0,0682655096205894
-3.232187376	0,00122846473589072	0,0733666439490293
-3.203589761	0,00135725723293944	0,0781634745755301
3.193209454	0,00140700849620201	0,0783288196560542
-3.110201017	0,00186960061925796	0,0922311901951016
3.122610589	0,00179254747485208	0,0922311901951016
-3.093948797	0,00197511570874379	0,0922311901951016
-3.127990423	0,00176005916227567	0,0922311901951016
-3.089947719	0,00200191730656034	0,0922311901951016
-3.114515328	0,00184247383657303	0,0922311901951016
-3.114399353	0,00184319828693004	0,0922311901951016
-3.103441705	0,00191283914668225	0,0922311901951016
-3.101324082	0,00192657308455403	0,0922311901951016
-3.105370703	0,00190040685738833	0,0922311901951016
-3.11979018	0,00180979903517973	0,0922311901951016
-3.09092383	0,00199534812917376	0,0922311901951016
-3.064095149	0,00218329458548343	0,0969992025439615
-3.045644618	0,00232182088966681	0,0969992025439615
-3.054687602	0,00225295060757951	0,0969992025439615
-3.042524495	0,00234602722431907	0,0969992025439615
-3.055081923	0,00224999054174188	0,0969992025439615
3.050206209	0,00228684294320027	0,0969992025439615
-3.050269604	0,0022863602543209	0,0969992025439615
-3.068938196	0,00214821027752005	0,0969992025439615
3.026671739	0,00247262316128223	0,100500117856283
-3.024186602	0,00249302617938067	0,100500117856283
-3.016945112	0,0025533604074363	0,101661571777556
-3.008031418	0,00262945952383703	0,103414719077737
-2.992520176	0,00276684378050283	0,107506881832791
-2.985856815	0,00282785017006728	0,108569247600797
-2.974045859	0,00293901147200648	0,110212930200243
-2.976699937	0,00291369028858834	0,110212930200243
-2.966998387	0,00300722536467515	0,111474733345717
-2.95341492	0,00314279227027578	0,115176193995902
-2.948301815	0,00319524942025943	0,115782914385805
-2.93924211	0,00329015950732596	0,117897382345847
-2.922615159	0,00347105237547692	0,123012570449594
-2.910675999	0,00360647785952879	0,125363515257037
2.909926819	0,00361513392834247	0,125363515257037
-2.894907826	0,00379269882236889	0,128913632585362
-2.894514323	0,00379745584359981	0,128913632585362
-2.888800752	0,00386714024648438	0,129911742655335
2.875456046	0,00403444312673444	0,132766113099169
-2.877695984	0,00400590991338822	0,132766113099169
-2.86719672	0,00414125509397626	0,134904522000742
-2.859626302	0,00424140484918643	0,136048339735579
-2.858183537	0,00426073870179643	0,136048339735579
2.854324814	0,00431284108381971	0,136361887209006
2.846598537	0,00441890510651519	0,138358922024383
-2.842270859	0,00447934177664978	0,138902665670149

-2.82267667	0,00476245744628822	0,146275478707424
-2.799940077	0,00511120936810318	0,155506134076724
-2.777929376	0,00547065072925016	0,16335970927622
-2.778898526	0,00545435608717226	0,16335970927622
-2.765427148	0,00568483015765178	0,166668884167518
-2.766360834	0,00566857737588637	0,166668884167518
-2.758005146	0,00581552824220611	0,168054474492076
-2.756838865	0,00583631043197288	0,168054474492076
-2.752670594	0,00591113401492936	0,168702718567674
-2.743506253	0,00607868905089826	0,171962913939885
-2.728756003	0,00635737233901877	0,175235263190902
2.732357526	0,00628828619885879	0,175235263190902
-2.729879168	0,00633575431098022	0,175235263190902
-2.72432954	0,00644321811090815	0,176096427183718
-2.716216406	0,00660327402173175	0,178954274958697
-2.713143711	0,0066648195689095	0,179117025914443
-2.704661052	0,00683741164432074	0,182236797958135
-2.692311033	0,00709587345908785	0,187575343488183
-2.689300336	0,00716019665569955	0,187736863533586
-2.68624148	0,00722608429996475	0,18793646667247
-2.679918477	0,0073640092102677	0,188260203842132
2.680309032	0,00735542202424156	0,188260203842132
2.676197922	0,00744626572486749	0,188260203842132
2.675040025	0,00747203289667997	0,188260203842132
-2.667260162	0,00764724491617509	0,188550669651913
-2.669853802	0,00758842796924621	0,188550669651913
2.664188834	0,00771742275784573	0,188550669651913
-2.664500146	0,00771028329049689	0,188550669651913
-2.656475143	0,00789622719630502	0,190725845785163
-2.655259889	0,00792473281711995	0,190725845785163
-2.646804768	0,00812562492500376	0,192684855758361
-2.649022739	0,0080724899872973	0,192684855758361
2.638983558	0,00831550139253348	0,194329652108119
-2.641240451	0,00826030714106904	0,194329652108119
2.63335394	0,00845461910788399	0,194758190163756
2.634698695	0,00842119996345633	0,194758190163756
2.628802238	0,00856861749372621	0,195984336292674
-2.626241602	0,00863335142279053	0,196074354496475
-2.621230604	0,00876129759894692	0,196108686754718
-2.619058219	0,0088172897920726	0,196108686754718
-2.622370617	0,0087320415985276	0,196108686754718
-2.612269358	0,00899433583657097	0,198676253924256
-2.608480325	0,00909452397032304	0,199522719757087
2.592402356	0,00953082323440246	0,200681579922242
2.596865531	0,009407874606192	0,200681579922242
-2.589440194	0,00961321196952552	0,200681579922242
-2.593397169	0,00950329533796821	0,200681579922242
2.590761873	0,00957637302511575	0,200681579922242
-2.588297596	0,0096451612055651	0,200681579922242
-2.596122874	0,00942823420797715	0,200681579922242

-2.603728238	0,00922158449194746	0,200681579922242
-2.585104795	0,0097349403932003	0,201251171590199
2.580165594	0,00987529484506753	0,202226169752712
-2.572536464	0,010095632040368	0,202226169752712
-2.572895076	0,0100851777047976	0,202226169752712
2.576734418	0,00997385506027161	0,202226169752712
2.575262623	0,0100164000622287	0,202226169752712
-2.567822558	0,0102339527086912	0,20316361658362
-2.560325584	0,0104574140629088	0,20316361658362
-2.561454349	0,0104234939126462	0,20316361658362
-2.561386051	0,0104255435107638	0,20316361658362
2.56081135	0,0104428044578436	0,20316361658362
-2.554527044	0,0106332169113735	0,20534206310886
-2.541174876	0,0110480645079956	0,212083381180272
-2.533339238	0,011298155177452	0,215600890220608
2.515633661	0,011881861431278	0,225405900681597
-2.511547535	0,0120203095950369	0,226698821309907
-2.509443012	0,0120921725264095	0,226728234870177
-2.493856347	0,0126363673628684	0,228945419917138
-2.495080097	0,0125928700738234	0,228945419917138
2.498715304	0,012464440122144	0,228945419917138
-2.499892926	0,0124230847877404	0,228945419917138
-2.497607414	0,0125034578408931	0,228945419917138
2.496771698	0,0125329616689905	0,228945419917138
2.484627638	0,0129687007584469	0,233653966178723
2.477332618	0,0132368479780458	0,235975083704138
-2.477143265	0,0132438729148679	0,235975083704138
2.474117826	0,0133565638331869	0,236675375615537
2.4697815	0,0135195601858846	0,236959682605858
-2.470744588	0,0134832079974534	0,236959682605858
2.466393041	0,0136481482675944	0,237920422502659
-2.46150995	0,0138353560027652	0,239367244963359
2.456544726	0,0140280338908759	0,239367244963359
-2.457977556	0,0139721905717608	0,239367244963359
2.456813404	0,0140175474082583	0,239367244963359
-2.444043401	0,0145236792440545	0,246520345063556
-2.439403546	0,0147115294048929	0,247718515846869
-2.438511412	0,0147478930364648	0,247718515846869
2.434809045	0,0148996503305188	0,248697843053123
-2.429620022	0,0151146596088099	0,248697843053123
-2.43004248	0,015097053249471	0,248697843053123
-2.430832693	0,0150641688765927	0,248697843053123
-2.424914781	0,0153119805929199	0,249399683899832
-2.426665771	0,0152382869640594	0,249399683899832
2.421996497	0,0154354993311718	0,250148167552909
-2.408763309	0,0160066751369553	0,254950267962137
-2.410065199	0,0159496699284132	0,254950267962137
2.404649136	0,0161879995713099	0,254950267962137
-2.41172158	0,0158774008788942	0,254950267962137
2.407443636	0,0160646417762218	0,254950267962137

2.402460451	0,0162851954109148	0,254950267962137
-2.402570071	0,0162803152121191	0,254950267962137
-2.397261508	0,016518130108656	0,25734767922906
-2.386520769	0,0170086466264051	0,258765983196695
2.380882329	0,0172712280039589	0,258765983196695
2.381660717	0,0172347683060187	0,258765983196695
-2.389130585	0,016888298406375	0,258765983196695
-2.386050417	0,0170304161370487	0,258765983196695
2.385311181	0,0170646799472555	0,258765983196695
2.382266636	0,0172064338345128	0,258765983196695
-2.381104064	0,017260835034077	0,258765983196695
2.379602907	0,0173313030606158	0,258765983196695
-2.37578233	0,0175117897828672	0,2602558619804
-2.367863955	0,0178911133220075	0,262267456652156
2.36798754	0,0178851382061431	0,262267456652156
-2.368074553	0,0178809323533992	0,262267456652156
-2.365690989	0,0179964582806707	0,262617999797117
-2.363562028	0,0181001963925017	0,262942042188369
-2.35890012	0,0183291883237817	0,265074584503121
-2.355543863	0,0184956133143335	0,265103790838781
-2.356476648	0,0184492275807064	0,265103790838781
-2.351530248	0,0186963694364617	0,265422552868802
-2.350171614	0,0187647572260734	0,265422552868802
-2.350261554	0,0187602232670714	0,265422552868802
2.346386145	0,0189564568259188	0,266938750026611
-2.344796007	0,0190374922499599	0,266938750026611
-2.34159706	0,0192014325590533	0,268071948064706
2.339388658	0,0193153279832086	0,268499710111413
-2.334306816	0,0195796620804268	0,271006052400757
2.32603805	0,0200165208777146	0,275868717224913
-2.317482445	0,0204774658656028	0,280826274667651
2.314551937	0,0206374657662739	0,280826274667651
2.315990426	0,0205587914450317	0,280826274667651
-2.308318687	0,0209814178500901	0,284307027590507
-2.295946261	0,0216789542180359	0,286535358004777
2.297064074	0,0216151148297464	0,286535358004777
-2.298942081	0,0215082284116558	0,286535358004777
-2.303533994	0,0212488167606675	0,286535358004777
-2.296546015	0,0216446813198746	0,286535358004777
-2.299215264	0,0214927186592916	0,286535358004777
2.288311334	0,0221193969609832	0,287007113505473
2.290713667	0,0219799796560226	0,287007113505473
-2.28712365	0,0221886068413999	0,287007113505473
2.286096504	0,0222486134500367	0,287007113505473
-2.290521497	0,0219911038959066	0,287007113505473
-2.29322438	0,021835090229472	0,287007113505473
-2.276696167	0,0228043755897019	0,293004427397564
-2.273853962	0,0229747684963513	0,294022334923543
-2.270895542	0,0231533021895406	0,294280172680172
2.270497494	0,0231774151506244	0,294280172680172

-2.268856353	0,0232770627117678	0,29438638135471
2.262837189	0,0236457274997901	0,297192429933725
-2.262229271	0,0236832417032456	0,297192429933725
-2.254530923	0,0241627875362692	0,302034844203364
-2.228828591	0,0258253118344378	0,305079233209018
-2.24879126	0,0245257796936248	0,305079233209018
-2.229649598	0,025770714432862	0,305079233209018
-2.230358239	0,0257236696862294	0,305079233209018
-2.231506673	0,0256475860238953	0,305079233209018
2.231731223	0,0256327323330941	0,305079233209018
2.247347852	0,0246178049397943	0,305079233209018
-2.245437193	0,0247400799986089	0,305079233209018
-2.229371426	0,0257892018184563	0,305079233209018
-2.231223385	0,0256663356704112	0,305079233209018
-2.229405173	0,0257869583831978	0,305079233209018
-2.233202152	0,025535616499273	0,305079233209018
-2.229208594	0,0258000289963552	0,305079233209018
-2.237215041	0,0252722887986046	0,305079233209018
2.228990991	0,0258145042081114	0,305079233209018
-2.22728259	0,0259283933601668	0,30517908243262
-2.225780768	0,0260288696457219	0,305247653118011
2.224271192	0,0261302037350401	0,305325750164871
-2.219799414	0,0264323867518417	0,306077773695699
-2.219109345	0,0264792864685581	0,306077773695699
-2.221244869	0,0263343806733491	0,306077773695699
2.212881886	0,0269057898429811	0,309877869524133
2.211514755	0,027000211267064	0,309877869524133
2.202404801	0,027636727024895	0,315068794975836
-2.202246552	0,0276478973575695	0,315068794975836
2.200631396	0,027762129120424	0,315256571878055
-2.197337231	0,0279963705588268	0,315886131864384
-2.197097721	0,0280134678180508	0,315886131864384
-2.195718843	0,0281120735934953	0,315893509892064
-2.191798703	0,0283940448208883	0,317954147733906
-2.188162802	0,0286577455899962	0,31979664196449
2.17860202	0,0293612460216203	0,32428088499906
-2.179495871	0,029294851103654	0,32428088499906
-2.178680213	0,0293554327094628	0,32428088499906
2.169125356	0,0300731681592717	0,325403894030127
-2.173515907	0,0297415099039426	0,325403894030127
-2.167755059	0,0301773278315639	0,325403894030127
2.16346355	0,0305055442915245	0,325403894030127
2.175563004	0,0295879522401223	0,325403894030127
2.162588663	0,0305728309739933	0,325403894030127
-2.163191276	0,0305264709774306	0,325403894030127
-2.170416871	0,0299752798636604	0,325403894030127
-2.164327606	0,0304392154959326	0,325403894030127
-2.167758976	0,0301770296472879	0,325403894030127
2.167029053	0,0302326388833541	0,325403894030127
-2.159430286	0,0308168003920834	0,32692164889628

-2.151248814	0,0314565660360958	0,331926781156741
-2.150768861	0,0314944480725466	0,331926781156741
-2.148172425	0,0317000604543599	0,333005521059644
2.141357395	0,0322452278974315	0,337632662237717
2.134423088	0,0328081633496802	0,339527986741414
-2.13552613	0,0327180581392151	0,339527986741414
2.137376664	0,0325673678842803	0,339527986741414
-2.133944078	0,0328473587173089	0,339527986741414
-2.130248596	0,033151094940713	0,341518573552179
-2.129030575	0,0332517308822897	0,341518573552179
-2.117448552	0,0342217972990247	0,35036601966205
2.109996083	0,0348586933089947	0,355757233928823
2.107918607	0,0350380304183991	0,356459457726615
2.106112805	0,0351945547685848	0,356925909209704
-2.104467278	0,0353377060383539	0,357254238162042
-2.10284366	0,0354794381294989	0,357566212398857
-2.100704219	0,0356669387364944	0,358336066745154
-2.097269842	0,0359696949659623	0,358337009783071
-2.091942415	0,0364436672714959	0,358337009783071
2.091929549	0,0364448183593325	0,358337009783071
-2.098708742	0,0358425836181194	0,358337009783071
2.093516655	0,0363030589207633	0,358337009783071
-2.092033149	0,0364355504643034	0,358337009783071
-2.094206985	0,036241545807179	0,358337009783071
-2.090144505	0,0366048208195807	0,358816252714735
-2.085618282	0,0370132139760208	0,361720045674749
-2.08220188	0,0373240336576898	0,363655614942748
-2.080717204	0,0374597984362221	0,363879066134989
-2.074610307	0,03802266610306	0,368237532079185
-2.07073329	0,0383837273825968	0,369608020353469
-2.070629948	0,0383933912615231	0,369608020353469
2.056692053	0,0397158571112612	0,371701444223491
2.064825309	0,0389395322164613	0,371701444223491
2.062296796	0,0391794878678066	0,371701444223491
2.057780701	0,0396111894891092	0,371701444223491
-2.056252056	0,0397582269702659	0,371701444223491
-2.056198256	0,0397634103122804	0,371701444223491
-2.056693629	0,0397157054431999	0,371701444223491
-2.066850793	0,0387482156660548	0,371701444223491
2.061330341	0,0392715354566225	0,371701444223491
-2.060935259	0,0393092169425749	0,371701444223491
-2.051888724	0,0401804779555254	0,37343527782873
-2.052398317	0,0401309680077728	0,37343527782873
-2.046851192	0,0406726976111218	0,376923706309965
-2.043703238	0,0409828731335018	0,376957306866768
-2.045120825	0,040842947672462	0,376957306866768
2.042528391	0,0410991461283017	0,376957306866768
-2.042077269	0,0411438672921247	0,376957306866768
2.039095022	0,041440544896907	0,378165309194942
2.038080567	0,0415418762098522	0,378165309194942

2.037225005	0,0416274991516913	0,378165309194942
-2.035517711	0,041798808307945	0,378408158536257
2.034622187	0,0418889031309903	0,378408158536257
-2.031152356	0,0422395417989279	0,38037540906012
-2.030137498	0,0423425649155296	0,38037540906012
-2.028114415	0,0425485720351796	0,381164291148484
-2.019829503	0,043401075738727	0,385500429599843
-2.0193115	0,0434548533345165	0,385500429599843
-2.018773696	0,043510746162587	0,385500429599843
2.020812658	0,0432991620056711	0,385500429599843
-2.009304066	0,0445048969795959	0,392153805352996
-2.009406138	0,0444940798632247	0,392153805352996
-2.004157867	0,0450531522388015	0,395686363010877
-2.000961167	0,0453965749810929	0,395686363010877
2.001861667	0,0452996113866625	0,395686363010877
-2.000971652	0,0453954449345175	0,395686363010877
-1.993212304	0,0462382044105603	0,401108152076353
-1.992946014	0,0462673589371793	0,401108152076353
-1.987361695	0,0468823293668845	0,405349898681508
-1.986011215	0,0470320785948607	0,405557362215042
-1.980900611	0,0476024205904524	0,407209035554931
1.98255235	0,0474174543493415	0,407209035554931
-1.98131704	0,0475557306425387	0,407209035554931
1.971183182	0,0487029268393756	0,41048845645778
-1.972740998	0,0485250784398365	0,41048845645778
1.97120892	0,0486999840596323	0,41048845645778
-1.971832857	0,0486286901497377	0,41048845645778
-1.971106398	0,0487117070425705	0,41048845645778
-1.970776186	0,0487494818056836	0,41048845645778
1.967275847	0,0491514180202777	0,411722917182845
-1.96733976	0,0491440541913486	0,411722917182845
-1.963828329	0,0495500036177712	0,413986429189928
-1.960320698	0,0499583182505999	0,416319318754999
-1.954615736	0,0506284425534635	0,42024492447949
1.954094792	0,0506900079449679	0,42024492447949
-1.950752048	0,0510865485524203	0,422446459183475
1.94846043	0,0513598951214599	0,423620618329177
-1.945561617	0,0517074214616932	0,424316626498628
-1.946207255	0,0516298488325404	0,424316626498628
1.942032251	0,0521331961008603	0,426724765038768
-1.936463834	0,0528109179271192	0,429005567543978
-1.937061324	0,0527378476170083	0,429005567543978
1.936533127	0,0528024393892355	0,429005567543978
-1.931271536	0,0534494814398719	0,433101953878359
-1.927634891	0,0539005558598638	0,435662387589125
-1.924133169	0,0543378934089862	0,438099265609951
-1.91992316	0,0548676059847556	0,440683420676873
-1.91454602	0,0555504248570782	0,440683420676873
-1.917153223	0,0552184686776862	0,440683420676873
1.914970441	0,0554962734144068	0,440683420676873

-1.916788915	0,0552647537812545	0,440683420676873
-1.914040893	0,055614930919531	0,440683420676873
1.919398084	0,0549339732180284	0,440683420676873
1.908811522	0,0562864100181579	0,442906102966797
1.907585755	0,056444777773443	0,442906102966797
-1.907981904	0,0563935551917719	0,442906102966797
-1.909809694	0,0561577207339552	0,442906102966797
-1.905519852	0,0567125293445337	0,443926959068255
-1.904087013	0,0568988528271144	0,444307022681462
1.897763071	0,0577272998786899	0,448603715924759
1.898769969	0,0575947272957925	0,448603715924759
-1.894322559	0,0581822084029463	0,450102714564745
-1.893142704	0,0583388944769189	0,450102714564745
1.893235234	0,0583265937975535	0,450102714564745
-1.888950678	0,0588984378585527	0,451583937197744
-1.890498556	0,0586913135172097	0,451583937197744
1.888558909	0,0589509573830233	0,451583937197744
-1.885491833	0,0593634660079569	0,452946861290704
-1.885148628	0,0594097743646411	0,452946861290704
-1.881097942	0,0599585968799374	0,456053006928769
1.872787794	0,0610976941067933	0,460739114937851
-1.871404634	0,0612890171498723	0,460739114937851
-1.871824302	0,0612309150233577	0,460739114937851
-1.871752171	0,0612408981153481	0,460739114937851
-1.874707749	0,0608329403218085	0,460739114937851
1.866923889	0,0619122172814501	0,463349676420018
-1.866842173	0,0619236311742722	0,463349676420018
-1.858597702	0,0630841830984735	0,468770715420684
-1.859393164	0,062971430066192	0,468770715420684
1.859468698	0,0629607321854861	0,468770715420684
1.856469998	0,0633865955534728	0,469935104965402
-1.852016071	0,0640235151710101	0,473568432170889
1.850279889	0,0642732196695833	0,474327536463172
-1.849189123	0,0644305087513249	0,474402718545714
-1.847470145	0,0646790311722222	0,475147780251518
-1.843409847	0,0652691934206493	0,477560119749456
1.843174273	0,065303569863414	0,477560119749456
-1.840845869	0,0656441487870913	0,478964660267805
1.836937584	0,0662191100759478	0,481214777273861
-1.834879388	0,0665235624226884	0,481214777273861
-1.833736027	0,066693188010277	0,481214777273861
-1.834616898	0,0665624730764752	0,481214777273861
1.833699544	0,0666986063384235	0,481214777273861
-1.831368017	0,0670456317644428	0,482638755447161
-1.826980775	0,067702659948736	0,486042767438801
-1.826164274	0,0678255209024756	0,486042767438801
1.825201471	0,0679706319736122	0,486042767438801
-1.819655689	0,0688114552496145	0,486741517795016
1.818686028	0,068959344515006	0,486741517795016
1.823413473	0,0682407913103197	0,486741517795016

-1.818590591	0,068973914304596	0,486741517795016
1.820563818	0,0686731870718277	0,486741517795016
-1.821254647	0,0685681571370571	0,486741517795016
-1.802110296	0,0715280541442816	0,486982465840131
1.812742505	0,069871545352154	0,486982465840131
-1.812414326	0,0699222008754039	0,486982465840131
1.803440836	0,0713190091708447	0,486982465840131
-1.806282548	0,070874216468742	0,486982465840131
1.803464241	0,0713153364106967	0,486982465840131
1.79042343	0,0733858664554498	0,486982465840131
1.797585033	0,0722427919529876	0,486982465840131
-1.793377414	0,0729125965297192	0,486982465840131
1.81110186	0,070125085722499	0,486982465840131
1.805963164	0,0709240935631276	0,486982465840131
-1.790706734	0,0733403685642314	0,486982465840131
-1.80462622	0,0711331921910676	0,486982465840131
-1.805884452	0,0709363902239222	0,486982465840131
-1.804607019	0,0711361989156812	0,486982465840131
-1.796375137	0,0724348752592666	0,486982465840131
1.794329111	0,0727606542061641	0,486982465840131
-1.799977423	0,0718642031348954	0,486982465840131
-1.81016356	0,0702704267435027	0,486982465840131
1.801562977	0,0716141906519342	0,486982465840131
-1.796091337	0,0724799920142559	0,486982465840131
-1.790415595	0,0733871250847453	0,486982465840131
1.80753307	0,0706792032825033	0,486982465840131
-1.80308471	0,0713749120271761	0,486982465840131
1.804710788	0,0711199508642426	0,486982465840131
-1.795058621	0,0726443606502485	0,486982465840131
-1.813527705	0,0697504695476333	0,486982465840131
1.797159072	0,0723103699003014	0,486982465840131
-1.792154648	0,0731081970984676	0,486982465840131
-1.788767867	0,0736522078987525	0,48773792705026
1.782470123	0,0746725991848343	0,48847694192919
-1.783159504	0,0745603423942668	0,48847694192919
-1.784859509	0,0742841079010398	0,48847694192919
-1.784231991	0,0743859759331792	0,48847694192919
-1.785782446	0,0741344900018551	0,48847694192919
1.785366838	0,0742018339200736	0,48847694192919
-1.777359695	0,0755090778114252	0,492948939153535
-1.776159627	0,0757066104942888	0,493240038068851
-1.772881856	0,0762482857434629	0,493778351446911
1.774448408	0,0759890087425274	0,493778351446911
-1.772880177	0,0762485640373835	0,493778351446911
1.77016381	0,0766998561762554	0,495705483303454
1.76702498	0,0772240449251258	0,496150813651277
-1.767070457	0,0772164294853077	0,496150813651277
1.766987647	0,0772302971947104	0,496150813651277
-1.765234034	0,0775244408937932	0,497050341714678
-1.760111311	0,0783889353032323	0,499613273424751

1.760708268	0,0782877926172148	0,499613273424751
1.761734519	0,0781141627927705	0,499613273424751
-1.757302254	0,0788663038223723	0,500796377986804
1.753778642	0,0794684441793024	0,500796377986804
-1.754277146	0,0793830297580318	0,500796377986804
1.753519694	0,0795128420029926	0,500796377986804
-1.754303783	0,0793784679946081	0,500796377986804
-1.752653143	0,079661563382087	0,500796377986804
1.755350127	0,0791994371219733	0,500796377986804
1.751446138	0,0798690915631877	0,501124163990817
-1.748473003	0,0803821564446527	0,503363989386417
-1.746552779	0,0807149443804991	0,504468402378119
1.744205231	0,0811233094412689	0,506039986359946
-1.743234556	0,081292651682632	0,506117377753838
-1.741476452	0,0816000973256047	0,507052627890318
-1.735841115	0,0825919320916612	0,510298156173142
-1.736136816	0,0825396461116764	0,510298156173142
-1.735532399	0,0826465480671675	0,510298156173142
-1.734918009	0,0827553288925747	0,510298156173142
-1.731061641	0,0834407710393347	0,513542913362318
-1.726720957	0,084217790078072	0,515026002410941
-1.721100189	0,085232644995097	0,515026002410941
-1.720846267	0,0852787241201372	0,515026002410941
1.724724669	0,0845771030852528	0,515026002410941
1.72430239	0,0846532680402048	0,515026002410941
-1.724196483	0,0846723788516998	0,515026002410941
1.72611119	0,0843274111558841	0,515026002410941
-1.721054539	0,0852409276277401	0,515026002410941
1.723375588	0,0848206264874352	0,515026002410941
1.725187453	0,0844936961035843	0,515026002410941
1.718721382	0,0856651167294335	0,516392526079295
-1.714282264	0,0864768989429862	0,51894177399239
1.715215258	0,0863057683649318	0,51894177399239
1.713771216	0,0865707517543894	0,51894177399239
-1.712248224	0,0868509337956749	0,519655401653157
-1.704920529	0,0882092471292389	0,522886414440466
-1.70060704	0,0890168013655134	0,522886414440466
-1.701131485	0,08891829983605	0,522886414440466
-1.700362329	0,0890627930093219	0,522886414440466
-1.70430492	0,0883241363751705	0,522886414440466
1.708078182	0,0876218365843292	0,522886414440466
-1.702420188	0,0886766283243562	0,522886414440466
-1.707066886	0,0878096210388712	0,522886414440466
1.700074386	0,0891169345147069	0,522886414440466
1.699768774	0,0891744272689167	0,522886414440466
1.700336421	0,0890676634802381	0,522886414440466
-1.697932915	0,0895204240043157	0,523013346764345
1.697951384	0,0895169379854181	0,523013346764345
-1.695670432	0,0899483112034815	0,524562936042003
-1.6940036	0,0902645988956126	0,525457276964532

-1.692379742	0,0905735922829346	0,526306009211647
1.687690798	0,0914705990663433	0,53056237767798
-1.683601868	0,092258637565218	0,534172542455706
1.682483973	0,092475030142113	0,534465900014901
-1.67884917	0,0931814430706469	0,536428636390309
-1.678559986	0,0932378305506907	0,536428636390309
-1.678171455	0,0933136325627794	0,536428636390309
-1.67640336	0,093659211195729	0,537457217270865
-1.67462233	0,0940083552783482	0,538502567979881
-1.670990275	0,0947235987968808	0,541637599503441
-1.668154135	0,0952851330861979	0,543884166730953
1.656129169	0,0976956635065293	0,544159783779891
-1.663908045	0,0961308070507901	0,544159783779891
-1.666209453	0,0956717037789893	0,544159783779891
1.656470022	0,0976266710155655	0,544159783779891
-1.662728964	0,0963667017699292	0,544159783779891
-1.657825585	0,0973526755512355	0,544159783779891
-1.660185632	0,0968771142125144	0,544159783779891
-1.664379243	0,0960366653742972	0,544159783779891
-1.660747049	0,0967642598949214	0,544159783779891
-1.666300503	0,0956535766452033	0,544159783779891
-1.663579998	0,0961963917444336	0,544159783779891
1.65720056	0,0974789334411298	0,544159783779891
-1.660249953	0,0968641791579547	0,544159783779891
1.657788399	0,0973601836003574	0,544159783779891
-1.652142208	0,0985055642720676	0,54722491285856
-1.651600186	0,0986160820501019	0,54722491285856
1.650742232	0,0987912203227269	0,54722491285856
-1.650089218	0,098924689673346	0,54722491285856
1.644114484	0,100152556596682	0,552123068417606
-1.644710174	0,100029593641525	0,552123068417606
-1.640603992	0,10087964577245	0,554321553696687
-1.640529515	0,100895116905412	0,554321553696687
-1.638545547	0,101307940867318	0,554484441025926
-1.637909546	0,101440564404743	0,554484441025926
1.638333308	0,101352183030101	0,554484441025926
-1.63562667	0,101917745094417	0,556150131860399
-1.630984867	0,102893512681436	0,55720099451882
-1.632876347	0,102495005621222	0,55720099451882
-1.633504418	0,102362952290523	0,55720099451882
1.63186842	0,102707207984098	0,55720099451882
-1.630602604	0,102974199297121	0,55720099451882
1.627173467	0,10370026121343	0,559509458539676
-1.62694961	0,103747800374179	0,559509458539676
-1.625350157	0,104087969855085	0,560406849386728
1.624345824	0,10430202256063	0,560623371263387
-1.619934126	0,105246428280165	0,560770849844436
-1.620131188	0,105204099095144	0,560770849844436
-1.618538311	0,105546637474596	0,560770849844436
1.618619818	0,105529088368177	0,560770849844436

1.619436366	0,105353407554495	0,560770849844436
-1.623041093	0,10458062076007	0,560770849844436
-1.621899487	0,104824871408726	0,560770849844436
-1.616235836	0,106043333529946	0,562173738640116
-1.613286527	0,106682272262869	0,562173738640116
-1.613839385	0,106562269156536	0,562173738640116
-1.615686917	0,10616202094477	0,562173738640116
-1.613913128	0,106546270722957	0,562173738640116
1.611563293	0,107057003713751	0,563228119048691
-1.610063897	0,107383907718761	0,563381806779982
-1.609827132	0,107435600362694	0,563381806779982
-1.607883656	0,107860662121692	0,564692589841652
-1.60693465	0,108068704445632	0,564864784176925
1.604095851	0,108692926217499	0,566291901537052
1.604891909	0,108517594471011	0,566291901537052
-1.600416725	0,109506167177058	0,568691447900178
1.600968726	0,109383846002306	0,568691447900178
1.598496061	0,109932621373649	0,56998826998395
-1.597052684	0,110253963998731	0,570736812031955
-1.595635203	0,110570263192958	0,571456889098219
-1.593983234	0,11093979015621	0,57153486142776
-1.593984339	0,110939542727425	0,57153486142776
-1.59313157	0,111130678455741	0,571605164305845
1.583992473	0,113195431957938	0,573026319654312
-1.57897009	0,114342917604078	0,573026319654312
1.565574835	0,117448185206667	0,573026319654312
-1.579078139	0,114318135245502	0,573026319654312
1.569904217	0,116437396474779	0,573026319654312
1.582247025	0,113593189092501	0,573026319654312
1.567042168	0,117104835407462	0,573026319654312
-1.578577426	0,114433015957834	0,573026319654312
-1.569463476	0,116539983457629	0,573026319654312
-1.566364726	0,117263255883313	0,573026319654312
1.566544675	0,117221158302815	0,573026319654312
-1.576510679	0,114908159656962	0,573026319654312
-1.576703341	0,11486380129096	0,573026319654312
1.590500454	0,111722043365591	0,573026319654312
-1.585538774	0,112843974629131	0,573026319654312
-1.584675094	0,113040173468086	0,573026319654312
-1.580998362	0,113878412202091	0,573026319654312
-1.574230028	0,115434279551114	0,573026319654312
-1.567617553	0,116970413114412	0,573026319654312
-1.566248567	0,117290436779103	0,573026319654312
-1.566085239	0,117328663400474	0,573026319654312
-1.577992936	0,114567232555739	0,573026319654312
-1.570506873	0,11629723666207	0,573026319654312
-1.571355049	0,116100200983535	0,573026319654312
1.580384492	0,114018840961544	0,573026319654312
1.579357759	0,114254020412123	0,573026319654312
-1.575339751	0,115178043260204	0,573026319654312

-1.583818068	0,113235126325861	0,573026319654312
1.572888179	0,115744712756095	0,573026319654312
-1.581698958	0,113718310662447	0,573026319654312
-1.568405952	0,116786422874558	0,573026319654312
-1.565937917	0,117363152087854	0,573026319654312
1.589997307	0,111835412021733	0,573026319654312
-1.575122334	0,115228209747273	0,573026319654312
-1.562791172	0,118101721296331	0,574449561341426
-1.56280274	0,118098999548211	0,574449561341426
-1.562058253	0,118274266273087	0,574449561341426
1.558659364	0,119077024440553	0,576611717448625
-1.558809622	0,1190414462661	0,576611717448625
1.556982473	0,119474646708201	0,577539523149924
-1.556341992	0,119626791151674	0,577539523149924
-1.555542171	0,119816999287835	0,577593158001894
-1.553197552	0,120375945923693	0,579421530752107
-1.552236349	0,12060568157109	0,57966218042737
-1.550469493	0,121028870131811	0,580830515141502
1.548529563	0,121494849632459	0,581336632143442
1.549003042	0,12138098891572	0,581336632143442
-1.54773817	0,121685348282117	0,581385552903447
1.546765176	0,121919880240436	0,581644399076046
-1.545353631	0,122260749591263	0,582409036088367
-1.54237734	0,122981924844086	0,582492921342988
1.542260622	0,123010273871748	0,582492921342988
-1.54189723	0,12309856913506	0,582492921342988
-1.543468665	0,12271710461019	0,582492921342988
-1.541556312	0,123181448792533	0,582492921342988
1.538568146	0,12390976009748	0,583370768342152
1.539669641	0,123640900660249	0,583370768342152
-1.539210318	0,123752959515735	0,583370768342152
1.537109384	0,124266525486214	0,584197587016094
-1.535556377	0,124647220020993	0,585134329792872
-1.531580056	0,125626097149172	0,585479702022339
-1.530795685	0,125819896359516	0,585479702022339
1.532128667	0,125490686898287	0,585479702022339
-1.530101524	0,125991600993334	0,585479702022339
-1.532160749	0,125482771781153	0,585479702022339
-1.53059823	0,125868719508693	0,585479702022339
1.53296126	0,125285400334933	0,585479702022339
-1.527340362	0,126676397764297	0,585755400575546
-1.528171563	0,126469947256589	0,585755400575546
-1.526933904	0,126777447938521	0,585755400575546
-1.52898826	0,126267354385794	0,585755400575546
1.524799462	0,127309124156123	0,587370422608724
1.521395614	0,1281605888403	0,587826756111636
-1.521146929	0,128222970065409	0,587826756111636
-1.521677374	0,128089939666659	0,587826756111636
-1.520762727	0,128319391101579	0,587826756111636
1.521638098	0,128099785941585	0,587826756111636

-1.517610988	0,129112495325812	0,588118357945964
-1.518566895	0,128871549358319	0,588118357945964
-1.517740075	0,129079937093785	0,588118357945964
-1.518409141	0,128911288586483	0,588118357945964
-1.515686529	0,129598635396513	0,589500139850146
-1.513178146	0,130234413879126	0,590725716962282
1.513609274	0,130124967706813	0,590725716962282
-1.511337456	0,13070249548806	0,592016219029485
-1.507290271	0,131736270275963	0,593434835970784
1.507222083	0,131753741716903	0,593434835970784
1.50778441	0,131609713542408	0,593434835970784
1.508840193	0,131339626497917	0,593434835970784
1.506512172	0,131935744927458	0,593434835970784
-1.503370004	0,132743657257812	0,595111038452289
-1.502195875	0,13304652983693	0,595111038452289
1.502223089	0,133039503705699	0,595111038452289
1.503152871	0,132799627402594	0,595111038452289
1.494024556	0,13516920112945	0,5954250539541
-1.491254079	0,135894800197907	0,5954250539541
-1.493032917	0,135428570769552	0,5954250539541
-1.495589211	0,13476073636442	0,5954250539541
-1.492989655	0,135439894969927	0,5954250539541
-1.498480227	0,134008526114332	0,5954250539541
-1.498549908	0,133990436054985	0,5954250539541
-1.492636337	0,135532406146082	0,5954250539541
-1.490709652	0,136037740907921	0,5954250539541
1.491347467	0,135870292775777	0,5954250539541
-1.490583878	0,136070779771836	0,5954250539541
-1.496547448	0,134511052951311	0,5954250539541
1.498613011	0,133974055286078	0,5954250539541
1.494795279	0,134967878769733	0,5954250539541
1.495707133	0,134729990729067	0,5954250539541
1.491512964	0,135826870186014	0,5954250539541
-1.489574485	0,136336154927032	0,59577791279089
-1.48817944	0,136703577880292	0,596575153807769
1.486912865	0,137037826079516	0,597225660954649
-1.485572275	0,137392293143661	0,597962409430911
-1.483927991	0,137828025180813	0,598072880405533
-1.48365152	0,137901393790212	0,598072880405533
-1.483377949	0,137974022642393	0,598072880405533
-1.481701871	0,138419638965606	0,599199108273933
-1.479914256	0,138896130689224	0,60045579285891
-1.476819137	0,139724125317806	0,600862180899925
-1.476082654	0,139921704761502	0,600862180899925
1.476745725	0,139743810261755	0,600862180899925
-1.476475361	0,139816324959365	0,600862180899925
-1.476998766	0,139675968001082	0,600862180899925
1.474488633	0,140350075862515	0,601100922518739
-1.475170497	0,140166711198276	0,601100922518739
-1.473632226	0,140580639618273	0,601223428664624

-1.472998162	0,140751531361796	0,601223428664624
-1.47228539	0,140943826895225	0,601248467906218
-1.470504495	0,141425169070538	0,601968540085928
1.470279987	0,14148593903415	0,601968540085928
-1.46812184	0,142071130147848	0,602472668063477
-1.467776774	0,14216486833994	0,602472668063477
1.468455494	0,141980537061388	0,602472668063477
-1.465427473	0,142804326425242	0,603828774536525
1.465225756	0,142859334874843	0,603828774536525
1.457429305	0,144997912803229	0,604110321730627
1.460228972	0,144227155433472	0,604110321730627
-1.455294112	0,145587856066949	0,604110321730627
1.462530448	0,143595907008072	0,604110321730627
1.460950564	0,144029008811116	0,604110321730627
-1.45948384	0,144431985142634	0,604110321730627
-1.455924265	0,14541355690966	0,604110321730627
1.455319006	0,145580967279288	0,604110321730627
1.454759834	0,145735761335327	0,604110321730627
1.460995683	0,144016626213968	0,604110321730627
-1.464047462	0,143180980820631	0,604110321730627
-1.45624074	0,145326081067156	0,604110321730627
1.455555818	0,145515449196432	0,604110321730627
-1.45925818	0,144494061162642	0,604110321730627
1.460536088	0,144142796765694	0,604110321730627
-1.453943045	0,145962097460989	0,604136728093352
-1.453385356	0,146116790050485	0,604136728093352
-1.452083349	0,146478431279743	0,604856518408668
1.448741142	0,147409886321012	0,604904086564741
-1.449040121	0,147326378527231	0,604904086564741
-1.449812066	0,147110933739894	0,604904086564741
-1.448445823	0,147492407336254	0,604904086564741
1.450923854	0,146801064851445	0,604904086564741
-1.448006067	0,147615353837659	0,604904086564741
-1.445888951	0,148208351312215	0,605793932068398
-1.4445516	0,148583876051505	0,605793932068398
-1.444782533	0,14851897884233	0,605793932068398
-1.44637687	0,148071525652482	0,605793932068398
1.438114	0,150401710896326	0,608589106199061
-1.439570821	0,149988859754	0,608589106199061
1.440957284	0,14959675089506	0,608589106199061
-1.438632986	0,150254535406707	0,608589106199061
-1.438578049	0,150270109377752	0,608589106199061
1.438592103	0,150266125139393	0,608589106199061
-1.433466813	0,151724476576654	0,612989991766669
-1.432959337	0,151869458425292	0,612989991766669
-1.432100111	0,152115172825595	0,613214290453181
-1.43077002	0,152496137919099	0,613965558123843
1.430121022	0,152682287632658	0,613965558123843
-1.428344695	0,153192669099391	0,613967888539457
-1.428131543	0,153254000085043	0,613967888539457

-1.428324364	0,153198518402761	0,613967888539457
-1.424608264	0,154270469873637	0,616167123396738
-1.423040886	0,154724303273819	0,616167123396738
-1.422923772	0,154758254248483	0,616167123396738
-1.423150425	0,154692553242201	0,616167123396738
-1.423918178	0,154470159050331	0,616167123396738
-1.421484352	0,155175999506497	0,61706855537417
-1.414314996	0,157269435916008	0,623037280766222
1.413043544	0,157642921272942	0,623037280766222
-1.415457864	0,156934294375502	0,623037280766222
1.414616324	0,157181019953532	0,623037280766222
1.413456229	0,157521622519504	0,623037280766222
-1.41054514	0,158378777364668	0,624558150389996
1.410422181	0,15841505954078	0,624558150389996
1.409388163	0,158720422322395	0,624636587857127
1.404311775	0,160226029326249	0,624636587857127
-1.405624563	0,159835638100412	0,624636587857127
-1.402642437	0,160723490472436	0,624636587857127
-1.408517935	0,158977759989425	0,624636587857127
1.402596581	0,160737171959345	0,624636587857127
-1.408181923	0,159077207677269	0,624636587857127
-1.405361799	0,159913720261911	0,624636587857127
1.404848998	0,160066185466287	0,624636587857127
-1.404715986	0,160105750085768	0,624636587857127
1.40646855	0,159585037039429	0,624636587857127
-1.40252281	0,160759183851602	0,624636587857127
-1.401168253	0,16116376549093	0,624967927172884
1.400939934	0,161232035785377	0,624967927172884
-1.399225586	0,161745346304512	0,624973295601003
-1.39899397	0,161814791264136	0,624973295601003
-1.399540017	0,161651106896058	0,624973295601003
-1.396590173	0,162536846977811	0,62540396675406
-1.397278101	0,162329958545336	0,62540396675406
-1.391638458	0,164031906794785	0,62540396675406
1.394474476	0,163174371903868	0,62540396675406
-1.391707716	0,164010924923237	0,62540396675406
-1.392786137	0,163684471906044	0,62540396675406
-1.395056376	0,162998839747365	0,62540396675406
1.394080627	0,163293258704269	0,62540396675406
-1.392708522	0,163707950613018	0,62540396675406
-1.391547523	0,164059459185716	0,62540396675406
1.395341857	0,162912775576316	0,62540396675406
-1.390675298	0,164323911620997	0,625672508828471
-1.388845783	0,164879650109684	0,626102300020602
-1.387608358	0,165256334892327	0,626102300020602
1.387825663	0,165190138173969	0,626102300020602
1.3871124	0,16540749135428	0,626102300020602
1.388902725	0,164862331735225	0,626102300020602
-1.383299528	0,166573042610112	0,626630720078734
-1.383767249	0,166429734494768	0,626630720078734

1.384223654	0,166289982689277	0,626630720078734
-1.383073123	0,166642445823644	0,626630720078734
-1.385665462	0,165849079169142	0,626630720078734
-1.382843306	0,166712917155831	0,626630720078734
-1.381377042	0,167163060997881	0,626861478742055
-1.381600965	0,167094257527171	0,626861478742055
-1.37763166	0,168317040920044	0,627101574699327
1.378798248	0,167956967104543	0,627101574699327
-1.376754258	0,168588237291261	0,627101574699327
-1.379614132	0,167705483919285	0,627101574699327
-1.376885346	0,168547698393706	0,627101574699327
-1.378709429	0,167984361089855	0,627101574699327
-1.376878414	0,168549842160027	0,627101574699327
-1.375357052	0,169020777063582	0,627778145447079
-1.374909691	0,169159444463104	0,627778145447079
-1.372505611	0,169906093408114	0,629824311771458
-1.367733775	0,171395423801197	0,630273935870993
-1.371236252	0,170301320588272	0,630273935870993
-1.370421049	0,170555505087038	0,630273935870993
-1.368682169	0,171098646333061	0,630273935870993
1.368588534	0,171127930213421	0,630273935870993
-1.368041039	0,171299230622246	0,630273935870993
1.369824597	0,170741661909614	0,630273935870993
-1.365944008	0,171956538429002	0,630733661863263
-1.361113071	0,173477959456057	0,630733661863263
1.361809021	0,173258163940065	0,630733661863263
1.363159367	0,172832289897337	0,630733661863263
-1.363046378	0,172867894615484	0,630733661863263
-1.35988172	0,173867356712075	0,630733661863263
1.365073463	0,172229961789489	0,630733661863263
-1.360112105	0,173794450871355	0,630733661863263
1.364154696	0,172518883239836	0,630733661863263
-1.362939364	0,172901621507761	0,630733661863263
-1.3629969	0,172883487479288	0,630733661863263
-1.359968713	0,173839824970719	0,630733661863263
-1.358045234	0,174449331232995	0,632133812613944
-1.357022515	0,174774056507833	0,632599699481214
-1.353804877	0,175798637450292	0,633856970040881
-1.354872134	0,17545829933217	0,633856970040881
-1.353997051	0,175737318518605	0,633856970040881
-1.353463531	0,175907593236152	0,633856970040881
1.351760874	0,176451824174767	0,63506439283825
-1.35056932	0,176833434036821	0,63506439283825
1.350872518	0,176736272851493	0,63506439283825
-1.349689356	0,177115648125963	0,635370372865662
1.34890359	0,177367935128087	0,635568434208979
1.347263905	0,177895252608261	0,636750487970747
-1.346625761	0,178100792939323	0,636779442604563
-1.345634638	0,17842037510243	0,637215625365823
-1.343862162	0,178992963614525	0,637847853764468

1.344014192	0,178943797707802	0,637847853764468
-1.341160378	0,179868387506791	0,640259988641722
-1.339405727	0,180438625945696	0,641030655409552
1.339271112	0,180482429492054	0,641030655409552
1.338531833	0,18072313051608	0,641179423448139
1.336176524	0,181491582406009	0,641786534253021
1.336650641	0,181336700536014	0,641786534253021
-1.336863936	0,181267054714909	0,641786534253021
1.335568764	0,181690265355972	0,641786534253021
-1.334438006	0,182060351313927	0,64239018926413
1.331679503	0,182965527297352	0,644734041642805
1.331196206	0,183124459579786	0,644734041642805
-1.330410118	0,183383182574038	0,644940854745118
1.326079595	0,184813334101723	0,649262529932525
1.325159321	0,185118314434843	0,649626293854591
-1.323063355	0,18581430989314	0,64998195641637
-1.323033316	0,185824298857641	0,64998195641637
1.323779746	0,185576204483369	0,64998195641637
-1.322108581	0,186131996755949	0,650352859737742
1.320725502	0,186592907506579	0,650553650495912
-1.320726353	0,186592623806847	0,650553650495912
1.319859932	0,18688178752141	0,650857197361282
-1.318639118	0,187289789506372	0,651574510418607
-1.317119307	0,187798636471746	0,652640735583383
-1.314272367	0,188754562908178	0,654552113310619
-1.314622626	0,188636762029282	0,654552113310619
-1.3112697	0,18976666138271	0,655243557772204
-1.311530855	0,189678476383128	0,655243557772204
-1.311827284	0,189578416920115	0,655243557772204
-1.312636668	0,189305407621008	0,655243557772204
-1.309668794	0,190307904664965	0,65623299088502
-1.309218995	0,190460179680117	0,65623299088502
-1.307423399	0,19106895471021	0,657627939103977
-1.305956283	0,191567424849099	0,658352465769314
1.305602584	0,191687741196089	0,658352465769314
-1.303375219	0,192446692633169	0,660255940151031
-1.300412434	0,193459650595925	0,660282734844523
-1.300582716	0,19340132641927	0,660282734844523
-1.300160318	0,193546027887593	0,660282734844523
1.301157771	0,193204456711645	0,660282734844523
-1.299760885	0,193682935554393	0,660282734844523
-1.30024639	0,193516535670577	0,660282734844523
1.298230169	0,194208253985043	0,661374465788557
1.283799203	0,199212222534238	0,661574832000868
-1.297071809	0,194606480155948	0,661574832000868
-1.290928783	0,196728373509582	0,661574832000868
1.283275488	0,19939557727282	0,661574832000868
1.285470389	0,198627957623656	0,661574832000868
1.285935286	0,198465647758252	0,661574832000868
1.293758619	0,195748810277309	0,661574832000868

-1.284211923	0,199067814500192	0,661574832000868
-1.296087219	0,194945437937041	0,661574832000868
-1.288148579	0,197694247664053	0,661574832000868
-1.293130532	0,195965917028514	0,661574832000868
-1.283572735	0,199291494789777	0,661574832000868
-1.295760496	0,19505801208832	0,661574832000868
1.283504787	0,199315283698734	0,661574832000868
1.293982886	0,195671332306377	0,661574832000868
-1.284572868	0,198941585092998	0,661574832000868
-1.284965881	0,19880420695327	0,661574832000868
-1.287403681	0,197953622024448	0,661574832000868
-1.294812908	0,195384778006544	0,661574832000868
-1.287538434	0,197906682418134	0,661574832000868
-1.29056039	0,196856158399249	0,661574832000868
-1.289945926	0,197069433439216	0,661574832000868
-1.28425856	0,199051501334821	0,661574832000868
-1.290302388	0,19694568826646	0,661574832000868
1.294863536	0,195367309256543	0,661574832000868
1.28168307	0,19995384624869	0,662745276620786
-1.281048603	0,200176595228169	0,662752783157829
-1.280506328	0,200367120489576	0,662752783157829
1.27948798	0,200725269729499	0,663257166882821
1.276719748	0,201701206785747	0,6635410371862
-1.276564347	0,20175609589265	0,6635410371862
-1.277930659	0,201273876629756	0,6635410371862
1.278062304	0,201227458893994	0,6635410371862
1.27632707	0,201839924799895	0,6635410371862
-1.274107133	0,202625450975165	0,665445091033509
-1.272868505	0,203064707121229	0,666209237503524
-1.271072468	0,203702868908469	0,667623731940866
1.270432917	0,203930464315099	0,667691114128117
-1.269537761	0,204249331413506	0,668056890272369
1.265439802	0,205713714830506	0,669451796496852
-1.266003601	0,205511793065526	0,669451796496852
-1.266829871	0,205216128802954	0,669451796496852
-1.265458282	0,20570709393777	0,669451796496852
-1.266502929	0,205333081331966	0,669451796496852
-1.264760487	0,205957199593672	0,669568516824184
-1.263113605	0,206548355264234	0,670814144740336
-1.262228671	0,206866514766205	0,671171539357155
-1.25850258	0,208210054594899	0,674174122558785
1.259024983	0,208021308347463	0,674174122558785
1.255639719	0,209246623839537	0,676850914626386
-1.254928492	0,209504719906595	0,677006735169109
-1.252118645	0,210526634596149	0,677593210152274
-1.252961221	0,21021981983746	0,677593210152274
-1.25293632	0,210228882540433	0,677593210152274
-1.252133712	0,210521145510796	0,677593210152274
-1.251536339	0,21073886428025	0,67760003719223
1.250720779	0,211036366753174	0,677880759740026

1.249623452	0,21143713185281	0,678492288781406
1.248357503	0,211900164521003	0,679302217276575
1.246987591	0,212402047772016	0,680234959349308
1.242464096	0,214065379469537	0,684404062798529
-1.242293538	0,21412827887247	0,684404062798529
-1.241655876	0,214363556209344	0,684477691856568
-1.23962597	0,215113770448229	0,685399865630876
-1.238023152	0,215707476219521	0,685399865630876
-1.237429901	0,215927523559991	0,685399865630876
-1.237596863	0,215865578202902	0,685399865630876
1.237695554	0,215828968421771	0,685399865630876
-1.240174724	0,214910775062778	0,685399865630876
1.236121324	0,216413469754178	0,686266902612807
-1.234824965	0,216895654078769	0,686879675381363
-1.23445695	0,217032678825925	0,686879675381363
-1.232437591	0,217785665900984	0,688365704166124
1.232053469	0,217929111303446	0,688365704166124
1.230274137	0,218594465672842	0,689791733654517
-1.227083789	0,219791100057343	0,691553324927104
1.22618873	0,220127661286677	0,691553324927104
-1.226767304	0,219910062993263	0,691553324927104
1.225930345	0,220224888279112	0,691553324927104
-1.227812335	0,219517424030295	0,691553324927104
1.222970356	0,221340893195065	0,694381693145997
1.222168241	0,221644011220402	0,694656886477938
1.220640866	0,222222026275699	0,695321781735504
-1.219331035	0,222718573808613	0,695321781735504
-1.219410114	0,222688573204996	0,695321781735504
1.219383697	0,222698594616255	0,695321781735504
1.215073969	0,224337880842071	0,69739184008641
-1.208807401	0,226736847456283	0,69739184008641
-1.211327513	0,225769908377627	0,69739184008641
1.212374255	0,225369151092651	0,69739184008641
-1.207760078	0,227139561566225	0,69739184008641
-1.212834418	0,225193133467199	0,69739184008641
-1.206331011	0,227689884948797	0,69739184008641
-1.208949323	0,226682315331583	0,69739184008641
-1.21145683	0,225720370141416	0,69739184008641
1.209724388	0,226384668253454	0,69739184008641
-1.213410929	0,224972750009993	0,69739184008641
-1.213752364	0,224842301923402	0,69739184008641
1.212970688	0,225141027466076	0,69739184008641
1.207740741	0,227147001844857	0,69739184008641
-1.205251387	0,228106270308885	0,69739184008641
-1.213174885	0,225062964202375	0,69739184008641
-1.216085667	0,223952289885525	0,69739184008641
-1.215267207	0,224264194780189	0,69739184008641
1.206643904	0,227569310979389	0,69739184008641
-1.20516649	0,228139036059275	0,69739184008641
-1.205527929	0,227999563042549	0,69739184008641

-1.209534346	0,226457624009928	0,69739184008641
-1.203242968	0,228882311711163	0,69900137809517
-1.200067309	0,230113200596821	0,699117334796461
-1.202035765	0,229349671761292	0,699117334796461
1.199789858	0,230220964202742	0,699117334796461
1.200047875	0,230120747808398	0,699117334796461
-1.200526879	0,229934780127824	0,699117334796461
-1.200062073	0,230115233776723	0,699117334796461
-1.191346156	0,233517721398165	0,699358778196289
-1.191255485	0,233553303602296	0,699358778196289
-1.196756993	0,231401285710686	0,699358778196289
-1.193451185	0,232692719229873	0,699358778196289
-1.195336004	0,231955778209262	0,699358778196289
-1.193756708	0,232573150762916	0,699358778196289
-1.196955623	0,231323852229195	0,699358778196289
1.19191269	0,23329548171159	0,699358778196289
-1.193691808	0,23259854598216	0,699358778196289
1.198903533	0,23056545769494	0,699358778196289
1.198453484	0,230740521400875	0,699358778196289
-1.19280602	0,232945351703855	0,699358778196289
-1.195566614	0,231865726606991	0,699358778196289
-1.192767171	0,232960570715626	0,699358778196289
1.195089565	0,232052038507461	0,699358778196289
1.190060226	0,234022721577478	0,700114357223902
1.189044455	0,23442217387205	0,70020441115633
1.188879575	0,234487058619794	0,70020441115633
1.187305208	0,235107254385154	0,701406933757745
-1.185358686	0,235875659031746	0,703048983712921
1.182342599	0,23706978928298	0,703090704440998
1.183525637	0,236600892169289	0,703090704440998
1.18371576	0,236525598287101	0,703090704440998
-1.184153503	0,23635230417345	0,703090704440998
-1.182020083	0,237197732226917	0,703090704440998
-1.182687009	0,236933214496653	0,703090704440998
1.181426405	0,237433373543276	0,703142910630915
1.180232113	0,237907909923713	0,703901843581628
1.176897509	0,239236416608267	0,707183724621136
-1.176016257	0,239588379872665	0,70757557242614
-1.16916086	0,242338827291368	0,710088180999458
-1.168447354	0,242626363221073	0,710088180999458
1.172457041	0,241013609780732	0,710088180999458
1.168183883	0,242732600053943	0,710088180999458
-1.169053465	0,242382091140598	0,710088180999458
-1.173107815	0,240752572939776	0,710088180999458
-1.171357531	0,241455095547572	0,710088180999458
-1.167957226	0,24282401891941	0,710088180999458
1.167442893	0,243031557537257	0,710088180999458
1.169443256	0,242225090564879	0,710088180999458
1.170001897	0,242000205271683	0,710088180999458
1.167319543	0,243081349402605	0,710088180999458

-1.166342436	0,243476022324799	0,710597440721697
-1.165194188	0,243940397534126	0,711309025359455
-1.163172115	0,244759677792086	0,711655880653073
-1.163440076	0,244650997711272	0,711655880653073
1.164108154	0,244380184535353	0,711655880653073
1.161635979	0,245383361018982	0,711655880653073
1.161903167	0,245274800522338	0,711655880653073
1.161995805	0,245237168950279	0,711655880653073
-1.160395401	0,245887858163111	0,712478295216561
1.159211002	0,246370188022274	0,713235059579742
1.156399051	0,247517969689685	0,714739548615451
-1.156164711	0,247613791249228	0,714739548615451
1.155767239	0,247776376853356	0,714739548615451
1.156387657	0,247522627886785	0,714739548615451
-1.154606987	0,248251404014448	0,715288311819075
1.154122696	0,248449869660551	0,715288311819075
-1.153678506	0,248631999240057	0,715288311819075
-1.150760585	0,24983074411084	0,715306199690896
1.152649152	0,249054420048614	0,715306199690896
1.14988602	0,250190819612816	0,715306199690896
1.150404907	0,249977140091061	0,715306199690896
-1.152008278	0,249317671611425	0,715306199690896
-1.14994949	0,250164675370097	0,715306199690896
1.151902012	0,249361341567183	0,715306199690896
1.148767505	0,250651861862584	0,715989596551667
-1.146658437	0,251522814564951	0,716573389551207
-1.147537539	0,251159527684586	0,716573389551207
-1.146832113	0,251451014385485	0,716573389551207
1.146010887	0,25179064748444	0,716703299326847
-1.142724653	0,253152930605867	0,718170120695359
-1.142619221	0,253196721621899	0,718170120695359
-1.143016543	0,253031722647461	0,718170120695359
-1.143780971	0,252714483279583	0,718170120695359
-1.141523384	0,253652186663858	0,718762205463773
1.138304142	0,254993505022405	0,718762205463773
-1.140229782	0,254190582876929	0,718762205463773
-1.139046374	0,254683812552795	0,718762205463773
1.137837263	0,255188441939851	0,718762205463773
1.139101131	0,25466097581692	0,718762205463773
-1.138669923	0,254840851974553	0,718762205463773
-1.139197027	0,254620985278013	0,718762205463773
1.136828093	0,255610157045739	0,719321777026621
1.13576122	0,256056511185862	0,719949650021277
-1.134442242	0,256609088200798	0,720081167384326
-1.134544135	0,256566371513966	0,720081167384326
1.134051063	0,256773129454876	0,720081167384326
-1.132555637	0,257400907711144	0,721214532900469
-1.13160055	0,257802408587756	0,721517125165822
1.130171877	0,258403807617527	0,721517125165822
-1.130177648	0,258401376121714	0,721517125165822

-1.131201543	0,257970272637623	0,721517125165822
1.128022645	0,259310355630775	0,72175312997669
-1.129018135	0,258890183600053	0,72175312997669
-1.128535044	0,259094025474249	0,72175312997669
1.127849397	0,259383527951313	0,72175312997669
1.126223384	0,260070978038413	0,721797680011947
-1.126281673	0,260046312838128	0,721797680011947
-1.1269598	0,259759477757502	0,721797680011947
1.125678275	0,260301722514852	0,721816900352878
-1.118590039	0,263315075104556	0,722036204171255
1.123732917	0,261126347412064	0,722036204171255
1.120143242	0,262652725955101	0,722036204171255
-1.124928914	0,260619158364491	0,722036204171255
-1.123275429	0,261320535975564	0,722036204171255
-1.123374566	0,261278447247977	0,722036204171255
1.119433818	0,262955110460706	0,722036204171255
-1.122040496	0,261845222071874	0,722036204171255
-1.120114533	0,262664958381582	0,722036204171255
1.118071055	0,26353664755834	0,722036204171255
-1.120350705	0,262564342700741	0,722036204171255
1.121208793	0,262198997786857	0,722036204171255
-1.11857483	0,263321566501417	0,722036204171255
1.117597082	0,263739115818214	0,722036204171255
-1.119207659	0,263051558891574	0,722036204171255
1.1154709	0,264648681995237	0,722776227545922
-1.116051006	0,26440030221173	0,722776227545922
1.115393637	0,264681775110615	0,722776227545922
1.114219554	0,265185008850304	0,723537777954509
-1.112808169	0,265790826335743	0,724194623082889
-1.112612561	0,265874863172137	0,724194623082889
-1.109890025	0,267046419281802	0,726771900577057
1.107653865	0,268011331098964	0,728168949278987
1.108007083	0,267858756819055	0,728168949278987
-1.105032435	0,269145534835058	0,730634974615371
-1.103478148	0,269819575983277	0,731848723756154
1.102766454	0,270128599470968	0,732071204448633
-1.097976486	0,272214756997213	0,733410686145375
1.098436759	0,272013818409895	0,733410686145375
1.098319522	0,27206499043303	0,733410686145375
1.099755034	0,271438869153857	0,733410686145375
-1.09972301	0,27145282623997	0,733410686145375
1.100977898	0,270906276595973	0,733410686145375
1.098797827	0,271856260401431	0,733410686145375
1.097301961	0,272509413867656	0,733591702607004
1.096012617	0,273073252970114	0,734031815181447
-1.095367368	0,273355724041215	0,734031815181447
-1.095857658	0,273141071204022	0,734031815181447
-1.091776704	0,274931261169974	0,736057288421705
-1.091050814	0,275250524169662	0,736057288421705
-1.091316054	0,275133835930949	0,736057288421705

1.092685457	0,274531927196854	0,736057288421705
-1.091049299	0,275251190647001	0,736057288421705
1.090283384	0,275588332248965	0,736348277964301
1.089510256	0,275928934721193	0,736648025228351
-1.087659941	0,276745258478086	0,738216260208294
-1.086575258	0,277224564678277	0,738883653791275
1.085124838	0,277866368040302	0,739982689454973
-1.081048384	0,27967559164161	0,740269054042723
1.080230014	0,280039766180503	0,740269054042723
-1.081048808	0,279675403256814	0,740269054042723
-1.083084323	0,278770996569018	0,740269054042723
-1.080998653	0,279697712936678	0,740269054042723
1.082762188	0,278913992787768	0,740269054042723
-1.080654798	0,279850697182758	0,740269054042723
-1.08193003	0,279283620085957	0,740269054042723
1.083786128	0,278459637103601	0,740269054042723
-1.070269399	0,284498064359093	0,740414152643027
1.051514722	0,293022251457405	0,740414152643027
1.058060504	0,290027859245139	0,740414152643027
-1.051359562	0,29309348084556	0,740414152643027
1.053029497	0,292327473533448	0,740414152643027
1.074880187	0,282428373756378	0,740414152643027
-1.062357417	0,288073457740682	0,740414152643027
1.052901648	0,292386070760326	0,740414152643027
-1.068410438	0,285335410676862	0,740414152643027
-1.076443481	0,281728965343751	0,740414152643027
1.054572654	0,291620815956534	0,740414152643027
-1.078073779	0,281000832058584	0,740414152643027
-1.060961702	0,288707306477863	0,740414152643027
1.066023822	0,286412873266917	0,740414152643027
1.054876539	0,291481793399781	0,740414152643027
-1.070728996	0,284291301292392	0,740414152643027
-1.075939886	0,281954142282745	0,740414152643027
-1.052807662	0,292429152800751	0,740414152643027
1.061906186	0,288278276579098	0,740414152643027
-1.067130079	0,28591310075131	0,740414152643027
-1.059555203	0,289347002555638	0,740414152643027
-1.056113994	0,290916137752848	0,740414152643027
-1.063308132	0,287642238597938	0,740414152643027
-1.056839375	0,290584900699916	0,740414152643027
-1.055176708	0,291344514535927	0,740414152643027
-1.053078855	0,292304853212545	0,740414152643027
1.064985177	0,286882637711925	0,740414152643027
1.051168853	0,293181045868262	0,740414152643027
1.078249167	0,280922575712379	0,740414152643027
1.077557388	0,281231327479023	0,740414152643027
1.075483274	0,282158416780895	0,740414152643027
-1.066705346	0,286104912372191	0,740414152643027
-1.064993112	0,286879046935177	0,740414152643027
-1.064124809	0,287272163589394	0,740414152643027

-1.060157664	0,289072877686727	0,740414152643027
-1.062608871	0,287959362603093	0,740414152643027
-1.068461241	0,285312504862657	0,740414152643027
-1.070557229	0,284368563558232	0,740414152643027
1.077243474	0,281371507783094	0,740414152643027
-1.055323991	0,291277172249505	0,740414152643027
-1.075119994	0,282321008874277	0,740414152643027
-1.061313304	0,288547541494122	0,740414152643027
-1.07149218	0,283948184970396	0,740414152643027
1.052071683	0,292766662887697	0,740414152643027
1.070781743	0,284267577626974	0,740414152643027
-1.061103921	0,288642676047323	0,740414152643027
1.062047633	0,288214061598974	0,740414152643027
-1.064278598	0,287202510141897	0,740414152643027
-1.058804668	0,289688747452673	0,740414152643027
-1.054645717	0,291587386877188	0,740414152643027
1.062619601	0,287954494486985	0,740414152643027
-1.065870511	0,2864821811809	0,740414152643027
-1.057000541	0,29051134044042	0,740414152643027
1.070996512	0,284170997761052	0,740414152643027
-1.073081344	0,283234620539221	0,740414152643027
-1.077653706	0,28118832532392	0,740414152643027
1.061137182	0,288627562166839	0,740414152643027
-1.046537734	0,295312832777973	0,741415101582906
1.046479297	0,295339798498834	0,741415101582906
-1.048403762	0,294452620857341	0,741415101582906
-1.044457485	0,296273779482941	0,741415101582906
-1.046024235	0,295549843140272	0,741415101582906
-1.047001907	0,295098698784877	0,741415101582906
-1.048471161	0,294421582483365	0,741415101582906
-1.045973571	0,295573234719459	0,741415101582906
1.045659289	0,295718365193504	0,741415101582906
-1.045307364	0,295880935294245	0,741415101582906
-1.044741184	0,296142604861462	0,741415101582906
1.044322634	0,296336144477633	0,741415101582906
-1.042730084	0,29707332051171	0,742683301279275
1.041136469	0,29781221607164	0,742809612740963
-1.041237176	0,297765485885367	0,742809612740963
-1.041130834	0,297814830782656	0,742809612740963
-1.038763652	0,298914669994554	0,743493653434405
1.03807816	0,299233668494745	0,743493653434405
1.039536338	0,298555367262051	0,743493653434405
1.039816665	0,298425085321275	0,743493653434405
-1.038060721	0,299241786715615	0,743493653434405
1.036794922	0,299831442518673	0,744385221033657
-1.035156735	0,300595720197327	0,744563131825177
-1.03558562	0,30039550335862	0,744563131825177
-1.03542696	0,300469560343658	0,744563131825177
-1.031569515	0,302273827376929	0,747571390560274
-1.031925242	0,302107139992769	0,747571390560274

-1.031074337	0,30250596130083	0,747572203214695
-1.028298007	0,303809668300351	0,747928381884453
-1.030007921	0,303006287217896	0,747928381884453
1.029377402	0,303302363734823	0,747928381884453
1.028957305	0,303499738374381	0,747928381884453
-1.028643493	0,303647232195216	0,747928381884453
1.026317053	0,304742161621646	0,749651770579563
-1.025225017	0,305257026536277	0,749774493967627
1.02562018	0,305070651527684	0,749774493967627
-1.024585364	0,305558873162928	0,749944722945542
1.023590125	0,306028911324126	0,749956868556464
-1.02393318	0,305866837215213	0,749956868556464
1.023094207	0,306263305519807	0,749961397343491
-1.02070968	0,307392007173389	0,751099936589058
-1.020635949	0,30742695078994	0,751099936589058
-1.021619052	0,306961236873701	0,751099936589058
-1.019682663	0,307878986409218	0,751211612451306
-1.019557168	0,307938527646706	0,751211612451306
-1.017634591	0,308851642263138	0,752571131558239
-1.017401359	0,308962535870731	0,752571131558239
-1.016651478	0,309319256023734	0,752871396737013
-1.015165812	0,310026794466782	0,753509653224585
1.014060516	0,310553876982653	0,753509653224585
-1.012478994	0,311309085427661	0,753509653224585
1.01299627	0,311061942411086	0,753509653224585
-1.011096559	0,311970217122199	0,753509653224585
-1.013331764	0,310901720447337	0,753509653224585
-1.01123087	0,311905943897965	0,753509653224585
-1.011908348	0,311581877261795	0,753509653224585
-1.010717631	0,312151595878464	0,753509653224585
1.010836529	0,312094676349207	0,753509653224585
1.013604463	0,310771527182616	0,753509653224585
-1.008178049	0,313368993977911	0,754268380010016
1.006163427	0,314336962087895	0,754268380010016
1.007340492	0,313771177701631	0,754268380010016
-1.009201627	0,312877946748468	0,754268380010016
1.006361701	0,314241610050816	0,754268380010016
-1.006379893	0,314232861987019	0,754268380010016
-1.006242974	0,314298704653336	0,754268380010016
-1.007670796	0,313612529339701	0,754268380010016
-1.005654254	0,314581915896611	0,754294928450981
-1.003759982	0,315494317525959	0,755920634488276
1.000757214	0,31694419960206	0,756957576688927
-1.001245182	0,316708287849598	0,756957576688927
1.00043368	0,317100677862555	0,756957576688927
-1.000843286	0,31690257882917	0,756957576688927
-1.001390576	0,316638018543521	0,756957576688927
-0.999039378	0,317775615936127	0,757098389982834
-0.998857051	0,317863944197444	0,757098389982834
0.999095741	0,317748314248448	0,757098389982834

-0.997687649	0,318430843960759	0,757888909057896
-0.997092693	0,318719519549206	0,758016556449993
-0.994768012	0,319849107210338	0,760142498712852
0.983566172	0,325328864527317	0,761803714797369
-0.990551065	0,321904842480721	0,761803714797369
-0.984022857	0,325104274486834	0,761803714797369
0.985931948	0,324166508204599	0,761803714797369
-0.983474216	0,325374099447744	0,761803714797369
0.987198554	0,323545310577328	0,761803714797369
0.987393667	0,323449687896201	0,761803714797369
-0.992108467	0,321144616700117	0,761803714797369
0.99088961	0,321739486320151	0,761803714797369
0.982720728	0,325744906265293	0,761803714797369
-0.985864181	0,324199765751097	0,761803714797369
-0.982841429	0,325685488467631	0,761803714797369
-0.98364589	0,325289653472042	0,761803714797369
-0.990241382	0,322056150307144	0,761803714797369
-0.98769373	0,32330266624796	0,761803714797369
-0.991030346	0,321670762876913	0,761803714797369
0.984243446	0,324995828968294	0,761803714797369
-0.987836627	0,32323266630186	0,761803714797369
-0.987183689	0,323552596588616	0,761803714797369
0.99117006	0,321602547666413	0,761803714797369
0.982989878	0,325612420438175	0,761803714797369
-0.986329271	0,323971560461027	0,761803714797369
0.980655658	0,326762577564304	0,76362993669919
0.979620281	0,327273590018788	0,763717313900573
-0.979715907	0,327226371694246	0,763717313900573
0.978793428	0,327682057600581	0,764117596357103
-0.97716167	0,328489120856215	0,765446108931572
-0.976127383	0,329001343264161	0,765478187126404
0.976431316	0,328850768923487	0,765478187126404
-0.975696353	0,329214959858705	0,765478187126404
-0.975094086	0,329513592041462	0,765620557877317
0.970000498	0,332046243767741	0,77040093392796
0.969995421	0,332048774623214	0,77040093392796
0.96867995	0,332704899389008	0,771368296570489
0.967917819	0,333085414600767	0,771695734258243
0.963895041	0,335098550363814	0,773438550583462
0.963951808	0,33507008801854	0,773438550583462
-0.961983717	0,336057783499031	0,773438550583462
-0.962579592	0,335758542961914	0,773438550583462
-0.961629153	0,336235921835043	0,773438550583462
-0.962721764	0,335687171600702	0,773438550583462
-0.96569701	0,33419581634249	0,773438550583462
-0.963233533	0,335430340317233	0,773438550583462
0.962420951	0,335838193889979	0,773438550583462
-0.964321603	0,334884713297039	0,773438550583462
0.960656049	0,336725136723062	0,773460517045496
0.960967694	0,33656841195787	0,773460517045496

-0.95650299	0,338818174231855	0,773638561716474
-0.945352269	0,34447906189918	0,773638561716474
-0.951473251	0,34136419103764	0,773638561716474
0.957654317	0,338237099684346	0,773638561716474
0.957483773	0,3383231330829	0,773638561716474
0.947952015	0,343153883100916	0,773638561716474
-0.946486841	0,34390033070113	0,773638561716474
-0.953348369	0,340413592062005	0,773638561716474
-0.946435569	0,343926470643102	0,773638561716474
0.945499961	0,344403690487562	0,773638561716474
-0.957923734	0,338101217317921	0,773638561716474
-0.954257002	0,33995356753343	0,773638561716474
-0.949682035	0,34227384070095	0,773638561716474
0.945984324	0,344156582027347	0,773638561716474
-0.952228721	0,340980997877421	0,773638561716474
-0.947873432	0,343193891851755	0,773638561716474
-0.95744569	0,338342346436053	0,773638561716474
0.955197646	0,339477756221915	0,773638561716474
-0.946520331	0,343883257602407	0,773638561716474
-0.945454922	0,344426674299992	0,773638561716474
0.946716567	0,343783226127121	0,773638561716474
0.946648003	0,343818174398794	0,773638561716474
0.949599117	0,342315987397378	0,773638561716474
-0.95074882	0,341731898758761	0,773638561716474
0.948073622	0,343091975993234	0,773638561716474
-0.952918843	0,340631192301733	0,773638561716474
0.948690988	0,342777798804553	0,773638561716474
-0.95870066	0,337709565847978	0,773638561716474
0.95328257	0,340446920224825	0,773638561716474
-0.947961058	0,343149279138186	0,773638561716474
-0.947958127	0,34315077154939	0,773638561716474
-0.951981218	0,34110650752005	0,773638561716474
0.943958998	0,345190601093176	0,774158336944013
-0.943976047	0,345181888745699	0,774158336944013
-0.9428165	0,345774770364217	0,774929558321472
0.941440159	0,346479341847117	0,775969359345105
-0.940953169	0,346728858766521	0,775989291826531
0.938362483	0,348058159286127	0,777345958239445
-0.939030011	0,347715336106902	0,777345958239445
-0.938451954	0,348012196996657	0,777345958239445
-0.937577839	0,348461404509678	0,777707978922983
-0.935484801	0,3495385126813	0,779572408988376
-0.934653524	0,349966885837446	0,779988394489124
-0.933932746	0,350338586201208	0,78027758321747
0.932265775	0,351199190390577	0,780577111653763
-0.932431885	0,35111337285062	0,780577111653763
-0.932882802	0,350880482870183	0,780577111653763
0.930225074	0,352254562241485	0,780623900842817
-0.931447147	0,351622312545012	0,780623900842817
-0.930770376	0,351972356499591	0,780623900842817

-0.929885229	0,352430511512292	0,780623900842817
-0.930021856	0,352359768668032	0,780623900842817
0.929004827	0,35288658501101	0,781097622965346
-0.927874719	0,353472559510498	0,781322141481396
0.92823286	0,353286792766928	0,781322141481396
-0.923184812	0,355910902312212	0,782099802194511
-0.926191882	0,354346270657218	0,782099802194511
0.926434596	0,354220171857315	0,782099802194511
0.92214055	0,356455268193964	0,782099802194511
-0.923529386	0,355731393511533	0,782099802194511
-0.925328509	0,354795053783039	0,782099802194511
-0.923102865	0,355953601746162	0,782099802194511
0.922428706	0,356305002076703	0,782099802194511
-0.924600253	0,355173881369754	0,782099802194511
-0.922070117	0,356492002860754	0,782099802194511
-0.925312921	0,354803159373694	0,782099802194511
-0.920688419	0,357213124147672	0,782172612660544
0.920518696	0,357301767549191	0,782172612660544
-0.920148184	0,357495327460974	0,782172612660544
-0.920596632	0,357261061199023	0,782172612660544
0.918834233	0,358182284043926	0,783144315960449
-0.918324231	0,358449145183218	0,783196811121869
-0.902876029	0,366591693959194	0,784479343824101
-0.906238748	0,364809497744196	0,784479343824101
-0.915007836	0,360187516636174	0,784479343824101
0.902536502	0,366771940407922	0,784479343824101
-0.912152253	0,36168857465117	0,784479343824101
-0.901987782	0,367063358086998	0,784479343824101
-0.908454762	0,36363800401288	0,784479343824101
-0.907281911	0,364257737438038	0,784479343824101
-0.904531509	0,365713634171116	0,784479343824101
-0.903292377	0,366370741275135	0,784479343824101
-0.904599072	0,365677827376263	0,784479343824101
-0.910823044	0,362388618674061	0,784479343824101
0.911246822	0,362165338792107	0,784479343824101
-0.914931315	0,360227689353965	0,784479343824101
0.905401683	0,365252624344575	0,784479343824101
0.904289491	0,365841917598465	0,784479343824101
0.909352893	0,363163878940845	0,784479343824101
0.907338954	0,364227580548148	0,784479343824101
0.916271214	0,359524662159769	0,784479343824101
-0.913698275	0,360875410706202	0,784479343824101
-0.903497783	0,366261764475363	0,784479343824101
0.902311347	0,366891499836923	0,784479343824101
-0.907207787	0,364296926807976	0,784479343824101
0.906701543	0,364564647274069	0,784479343824101
-0.903577161	0,366219656115464	0,784479343824101
-0.908819432	0,363445447654999	0,784479343824101
0.912971951	0,361257293737265	0,784479343824101
0.915301103	0,36003358026327	0,784479343824101

0.906909333	0,364454745413813	0,784479343824101
-0.906416802	0,364715283135349	0,784479343824101
-0.907498611	0,364143183888587	0,784479343824101
-0.903483668	0,366269252594725	0,784479343824101
-0.909824814	0,362914906239555	0,784479343824101
0.901070743	0,367550707695822	0,785000683654983
0.900303958	0,367958515113811	0,785152190672691
0.899860717	0,368194377850429	0,785152190672691
-0.899564568	0,368352019996211	0,785152190672691
-0.899095521	0,368601783873722	0,785165622848581
0.898020856	0,369174430749958	0,785258342002043
0.897643229	0,369375784439411	0,785258342002043
-0.898317346	0,369016387391823	0,785258342002043
-0.896536982	0,369966038144888	0,785366530394378
0.896178852	0,37015724843394	0,785366530394378
0.896508137	0,369981436552367	0,785366530394378
-0.89339873	0,371643682336264	0,786262022204935
-0.891804785	0,372497576712477	0,786262022204935
-0.89295915	0,37187904912815	0,786262022204935
0.890381386	0,373261133642095	0,786262022204935
0.893498735	0,371590149210666	0,786262022204935
0.892283813	0,372240828044798	0,786262022204935
-0.890809424	0,373031418529089	0,786262022204935
-0.891216666	0,37281294541025	0,786262022204935
0.892971263	0,371872562169299	0,786262022204935
0.892154478	0,37231013792561	0,786262022204935
0.891042366	0,372906442149284	0,786262022204935
-0.888861953	0,374077275486658	0,787466849506836
0.887151712	0,374997226043031	0,788888489229469
-0.886383727	0,37541078534773	0,789243665414883
0.885569795	0,375849393945066	0,789651006822695
-0.884698369	0,376319335819642	0,790123605480694
-0.883890028	0,376755580820009	0,790524884934632
-0.883252484	0,377099870193827	0,790732822740631
-0.88083927	0,378404821491123	0,792953573300112
-0.879593271	0,379079685245943	0,79368914008835
-0.878835323	0,379490570920916	0,79368914008835
0.878828711	0,379494156284104	0,79368914008835
-0.87674014	0,38062779886046	0,795241890896294
-0.876552477	0,380729761098877	0,795241890896294
0.875315273	0,381402386033004	0,795702444233961
0.874332559	0,381937173232301	0,795702444233961
0.875079383	0,381530713883199	0,795702444233961
-0.874437412	0,381880090650296	0,795702444233961
0.872062368	0,383174354299429	0,797764553012046
0.866634353	0,386142384818483	0,79814171218438
0.866760204	0,386073410997514	0,79814171218438
0.869381083	0,384638726733775	0,79814171218438
-0.866359289	0,386293162194278	0,79814171218438
-0.870158086	0,384214017655976	0,79814171218438

0.867280876	0,385788132346936	0,79814171218438
-0.867286212	0,38578520946039	0,79814171218438
0.8663006	0,386325337277463	0,79814171218438
0.870042726	0,384277055102684	0,79814171218438
0.868189618	0,385290536559325	0,79814171218438
0.867608329	0,385608785184372	0,79814171218438
0.8668045	0,386049136201794	0,79814171218438
-0.864013777	0,38758032216762	0,798687884339024
-0.86474359	0,387179538807997	0,798687884339024
0.864320223	0,387412004098514	0,798687884339024
-0.865260159	0,38689601257238	0,798687884339024
-0.861914796	0,388734406319805	0,799668750602917
-0.862200836	0,388577009745175	0,799668750602917
-0.861795273	0,388800186339651	0,799668750602917
0.860344814	0,389598996748036	0,800290932810456
-0.860447672	0,389542316837359	0,800290932810456
0.858173134	0,390796868621678	0,802240548252648
-0.856766755	0,391573802047215	0,803324116795337
0.850279079	0,395169945301067	0,80644051679658
-0.850470854	0,39506335785037	0,80644051679658
0.853251794	0,393519686345876	0,80644051679658
0.851423766	0,394533994423519	0,80644051679658
-0.853007075	0,393655380773887	0,80644051679658
0.850228629	0,395197987847161	0,80644051679658
-0.849967065	0,395343397536556	0,80644051679658
0.851607114	0,394432189672701	0,80644051679658
-0.850083788	0,395278504291871	0,80644051679658
-0.849142185	0,395802179366258	0,806866010402137
-0.847974574	0,396452131099169	0,80768043132964
-0.847383867	0,396781193870295	0,807840498883649
-0.846851195	0,397078068617132	0,807934871476499
-0.844022325	0,398656930227675	0,810125141767014
-0.844258891	0,398524752419773	0,810125141767014
-0.842793519	0,399343933476133	0,811010192355497
-0.842175214	0,39968988520521	0,81120193819182
0.841236651	0,400215371793468	0,811247004584701
-0.841323838	0,400166539888333	0,811247004584701
0.840787951	0,400466738387239	0,811247004584701
0.840177889	0,400808653691011	0,811250098923589
-0.839825948	0,401005982403881	0,811250098923589
-0.839439159	0,401222917142054	0,811250098923589
-0.837878235	0,402099093496495	0,811787011794663
-0.837505951	0,402308233521426	0,811787011794663
-0.837371529	0,402383764353174	0,811787011794663
0.837173007	0,402495327708424	0,811787011794663
0.836277288	0,402998927331397	0,811787345811215
-0.836501848	0,402872637802544	0,811787345811215
-0.835036513	0,40369715044183	0,812398903932547
-0.83483567	0,403810239238467	0,812398903932547
0.833604656	0,404503800799939	0,812398903932547

-0.833054549	0,404813965463443	0,812398903932547
-0.833323844	0,404662112129207	0,812398903932547
-0.833268731	0,404693187149156	0,812398903932547
-0.832498113	0,405127842881846	0,812523192346985
0.831921197	0,405453426529578	0,812670789656862
-0.831414039	0,405739770596919	0,812739602593208
-0.828973119	0,407119618161682	0,813098720206446
-0.828867002	0,407179669188655	0,813098720206446
0.829241153	0,406967962466236	0,813098720206446
-0.829501706	0,406820571803897	0,813098720206446
0.830402142	0,406311455194161	0,813098720206446
0.826124849	0,408733272778287	0,815351230105766
-0.824647438	0,409571780704292	0,815351230105766
-0.825028239	0,409355558630469	0,815351230105766
0.825922848	0,408847859014859	0,815351230105766
-0.824824432	0,409471273706959	0,815351230105766
-0.823977895	0,409952118068005	0,815604923361701
-0.823492004	0,410228262317036	0,815651138084118
0.823040823	0,410484778998031	0,815658294681856
0.821479426	0,411373238185166	0,816920377553671
0.820276267	0,412058632936586	0,817777902289531
-0.819144343	0,412704065145027	0,818555110758126
-0.815639382	0,414706418030704	0,820005026455561
0.816183463	0,41439521383124	0,820005026455561
0.815914765	0,414548886678465	0,820005026455561
-0.816916155	0,413976344880659	0,820005026455561
0.816479789	0,414225778351587	0,820005026455561
0.814364312	0,415436277221863	0,820786332758097
0.81406002	0,4156105678741	0,820786332758097
-0.813320444	0,416034358726706	0,821120444855342
0.812329476	0,416602601218773	0,821739075798498
-0.811560931	0,417043616763469	0,821809560238523
-0.811379123	0,417147984530376	0,821809560238523
-0.810836711	0,417459449475961	0,821921077264942
0.810299243	0,417768210413431	0,822027137634726
0.809299585	0,418342845122172	0,822249959856378
-0.809215197	0,418391374922269	0,822249959856378
-0.807728201	0,419247065458762	0,82303965297616
0.807629998	0,419303612353436	0,82303965297616
-0.806539667	0,419931745592994	0,823771216263629
-0.803718339	0,421559660507253	0,824742409811957
-0.804016982	0,421387167443016	0,824742409811957
0.8034659	0,421705498846486	0,824742409811957
-0.803661185	0,421592676718666	0,824742409811957
-0.804196958	0,421283235591419	0,824742409811957
-0.801003094	0,423129854898862	0,825130761085469
0.801766029	0,422688312944642	0,825130761085469
0.800653623	0,423332197922954	0,825130761085469
-0.801189859	0,423021741408286	0,825130761085469
-0.800468863	0,423439196774093	0,825130761085469

-0.802158647	0,422461193759301	0,825130761085469
-0.799233896	0,424154800844609	0,82602610671731
-0.79791103	0,424922122276587	0,826092634172401
0.798555215	0,424548365123159	0,826092634172401
-0.797447526	0,425191166867831	0,826092634172401
0.797408933	0,425213572938352	0,826092634172401
0.795936219	0,426069107877495	0,827185677568787
-0.795557672	0,426289177091263	0,827185677568787
-0.794204535	0,42707636904233	0,827676846454063
0.794236289	0,427057886334586	0,827676846454063
0.793799382	0,427312232355353	0,827676846454063
0.791387409	0,428717955390178	0,827726452452224
-0.79193961	0,428395889296988	0,827726452452224
0.791179134	0,428839466019942	0,827726452452224
-0.792997949	0,4277790177546	0,827726452452224
0.790772719	0,429076632137346	0,827726452452224
-0.791800347	0,428477100113117	0,827726452452224
-0.790673646	0,429134458449649	0,827726452452224
-0.788999348	0,43011238763833	0,828752091408618
-0.788883406	0,430180155354427	0,828752091408618
-0.78830199	0,430520084116945	0,828911803747551
-0.787131476	0,431204905111701	0,829734975528183
-0.786543362	0,431549225848309	0,829902357400594
0.785601892	0,432100755538627	0,830467781056062
-0.784368629	0,432823840737201	0,830973544575743
0.782938977	0,433662947526226	0,830973544575743
0.781641587	0,434425239117737	0,830973544575743
0.781823579	0,43431826210426	0,830973544575743
-0.782935364	0,4336650695018	0,830973544575743
-0.781899139	0,434273851016506	0,830973544575743
-0.781739837	0,434367484580821	0,830973544575743
-0.783417089	0,433382224405807	0,830973544575743
0.769496375	0,441598695494073	0,832100080587139
0.771647	0,440323529105098	0,832100080587139
-0.769287619	0,44172258541556	0,832100080587139
0.779689374	0,435573735131507	0,832100080587139
-0.776808634	0,437271682213128	0,832100080587139
0.771800407	0,440232650475358	0,832100080587139
-0.77072464	0,440870163551578	0,832100080587139
-0.771598164	0,440352462314636	0,832100080587139
0.771093665	0,440651415392557	0,832100080587139
0.773187928	0,439411169272597	0,832100080587139
-0.775349062	0,438133426052882	0,832100080587139
-0.770244386	0,441154938892078	0,832100080587139
0.769985527	0,441308477970618	0,832100080587139
0.778101142	0,436509389801676	0,832100080587139
0.777294844	0,436984836330421	0,832100080587139
0.779662051	0,435589821481662	0,832100080587139
0.778736597	0,436134893133707	0,832100080587139
-0.770281453	0,441132955948157	0,832100080587139

-0.779159841	0,435885563309715	0,832100080587139
0.77169389	0,440295750625594	0,832100080587139
-0.777023879	0,437144682410477	0,832100080587139
-0.776050743	0,437719025303351	0,832100080587139
0.769633936	0,441517068332503	0,832100080587139
-0.771854777	0,440200444418763	0,832100080587139
0.769776094	0,441432722533424	0,832100080587139
0.770050264	0,441270077363049	0,832100080587139
-0.768109028	0,442422413533676	0,832931864358497
-0.767119527	0,443010453017719	0,833530828696154
-0.766704076	0,443257479446172	0,833530828696154
0.765123744	0,444197862124505	0,834812415706019
0.764394087	0,444632431458621	0,835105288181222
0.763992701	0,444871592277625	0,835105288181222
0.7611449	0,44657052484341	0,837009797057627
-0.760987116	0,44666476301897	0,837009797057627
-0.761806452	0,446175528759196	0,837009797057627
-0.757272606	0,448886559148846	0,837455138504275
-0.758267984	0,44829056972516	0,837455138504275
-0.75999844	0,447255517376029	0,837455138504275
-0.757116909	0,448979824643067	0,837455138504275
0.757292479	0,448874656046001	0,837455138504275
-0.757338035	0,448847369160009	0,837455138504275
-0.75968843	0,447440846224065	0,837455138504275
0.757441382	0,448785471830084	0,837455138504275
0.756475536	0,449364134672243	0,837687476484383
-0.754798641	0,450369809619323	0,838410029089858
0.753705469	0,451026096979232	0,838410029089858
-0.754625709	0,450473593675555	0,838410029089858
-0.753663012	0,451051597045241	0,838410029089858
-0.754308127	0,450664223299361	0,838410029089858
-0.751835602	0,452149929120861	0,839483892581909
0.751932359	0,45209173723445	0,839483892581909
-0.750913464	0,452704736789023	0,840030366021059
0.749911371	0,453308085361751	0,840183089248072
0.750296657	0,453076055688142	0,840183089248072
-0.749421131	0,45360341878518	0,840247573568182
0.74768659	0,454649221937418	0,841701343713074
-0.746007919	0,455662631282463	0,842610083650196
-0.746399138	0,455426339978552	0,842610083650196
-0.745095965	0,456213708490134	0,843145679014718
0.743459778	0,457203364542534	0,84346835496143
-0.743077987	0,457434465993036	0,84346835496143
-0.744237794	0,456732627040172	0,84346835496143
0.743552464	0,457147270873669	0,84346835496143
0.742060939	0,458050414497939	0,844121478146201
-0.740558062	0,458961444266634	0,845317337384292
-0.739114943	0,459837203996835	0,845964051848142
0.739415868	0,459654509541904	0,845964051848142
0.737749327	0,460666790925798	0,847007069974742

0.73731264	0,460932247138327	0,847012249014875
0.736566809	0,461385825283282	0,847362919441108
-0.735696697	0,461915300009803	0,847852500017993
0.735066335	0,462299095797907	0,848074279834044
-0.733125471	0,46348190652215	0,849277925303372
-0.733388571	0,463321467652746	0,849277925303372
0.732572096	0,463819455283839	0,849413823560693
-0.731974482	0,464184143206366	0,849599240545136
-0.731481541	0,464485075279453	0,849667820633145
-0.730146393	0,465300705732332	0,849861682361065
0.730411506	0,465138687112153	0,849861682361065
-0.730014008	0,465381622030896	0,849861682361065
0.729515979	0,465686097236877	0,84993642534744
-0.727763304	0,466758491287765	0,851411840725703
-0.726615064	0,467461797251309	0,852212716865727
-0.725163048	0,468352009270736	0,852871388988212
-0.725424695	0,468191527551191	0,852871388988212
0.722979977	0,469692185838601	0,853049181684638
-0.722729642	0,469846000440672	0,853049181684638
-0.721005223	0,470906301328407	0,853049181684638
-0.721603037	0,470538571157371	0,853049181684638
-0.718551486	0,472417314260082	0,853049181684638
-0.721262883	0,470747789000614	0,853049181684638
-0.722074934	0,47024840815043	0,853049181684638
-0.718973456	0,472157274916365	0,853049181684638
-0.722500052	0,469987093205387	0,853049181684638
0.719836838	0,471625460881422	0,853049181684638
-0.718682422	0,472336616487319	0,853049181684638
-0.723181236	0,469568545560415	0,853049181684638
-0.721744157	0,470451788089601	0,853049181684638
-0.718589724	0,472393746545578	0,853049181684638
0.719566047	0,471792223107374	0,853049181684638
-0.717116632	0,473302134168398	0,853213741024642
-0.717245713	0,473222497759102	0,853213741024642
-0.71717621	0,473265376362001	0,853213741024642
0.715149779	0,474516497672479	0,854447629812253
-0.715487035	0,474308149255117	0,854447629812253
-0.714525725	0,474902154605667	0,854664870872363
-0.71172329	0,476636142597576	0,857307060723471
-0.707671463	0,479149302559439	0,857545670888814
0.707741087	0,479106057133535	0,857545670888814
-0.70868651	0,478519037997946	0,857545670888814
0.706135737	0,480103729345586	0,857545670888814
0.706219413	0,48005169938867	0,857545670888814
-0.708059706	0,478908180926644	0,857545670888814
-0.705084926	0,480757386966504	0,857545670888814
0.706525792	0,479861218792547	0,857545670888814
0.710220186	0,477567604937942	0,857545670888814
-0.705159933	0,480710712383207	0,857545670888814
-0.708252644	0,478788379264298	0,857545670888814

0.705728949	0,48035671461503	0,857545670888814
0.706317673	0,479990605601921	0,857545670888814
0.706806391	0,479686802431195	0,857545670888814
-0.708113425	0,478874823548969	0,857545670888814
-0.703318398	0,481857347346602	0,858721711426346
0.70317092	0,481949239591221	0,858721711426346
-0.702685112	0,48225200787549	0,858786706459666
-0.701693239	0,482870490507016	0,859063278709516
0.701581697	0,482940069550497	0,859063278709516
-0.700718232	0,48347887574424	0,859219970721464
-0.700586659	0,483561006778126	0,859219970721464
-0.69898237	0,484563048946644	0,860526339676721
-0.697473451	0,485506548751211	0,861253366184078
0.697573677	0,48544384817323	0,861253366184078
-0.696278694	0,48625431324514	0,861255970063688
0.693273696	0,488137807054006	0,861255970063688
0.693494862	0,487999049322447	0,861255970063688
0.695386994	0,486812808819831	0,861255970063688
-0.693308433	0,488116012123568	0,861255970063688
-0.692783173	0,488445633874879	0,861255970063688
-0.694026997	0,487665278518767	0,861255970063688
0.693174581	0,488199998060095	0,861255970063688
0.693594716	0,487936408373442	0,861255970063688
-0.696377214	0,486192629252814	0,861255970063688
0.694149065	0,487588731200321	0,861255970063688
0.692259145	0,488774601957097	0,861365077219474
0.690473837	0,489896257356865	0,861372092587865
0.689278178	0,490648227622917	0,861372092587865
-0.691322701	0,489362768604905	0,861372092587865
0.689564261	0,490468248714789	0,861372092587865
-0.690118082	0,490119932740087	0,861372092587865
-0.691166724	0,48946077255609	0,861372092587865
-0.690129916	0,490112491590975	0,861372092587865
-0.685903501	0,492773962240901	0,863224349933137
-0.686354397	0,492489654289559	0,863224349933137
0.686723629	0,492256903619667	0,863224349933137
-0.686308457	0,49251861712027	0,863224349933137
-0.684936369	0,493384074712113	0,863326624832018
-0.684859388	0,493432655491189	0,863326624832018
-0.684538098	0,493635440679145	0,863326624832018
0.682681994	0,494807809683847	0,863971405647215
-0.683069208	0,494563111386208	0,863971405647215
0.683488235	0,494298381809098	0,863971405647215
0.677922511	0,497820826376053	0,864110368507252
-0.680038404	0,496480144199788	0,864110368507252
-0.677957926	0,497798370464806	0,864110368507252
-0.678610903	0,497384432915798	0,864110368507252
0.681572938	0,495509031517339	0,864110368507252
0.677900554	0,497834748739992	0,864110368507252
-0.679027781	0,497120259826266	0,864110368507252

-0.677904398	0,497832311073713	0,864110368507252
-0.679635933	0,496735011355824	0,864110368507252
0.678136269	0,497685296395174	0,864110368507252
-0.682014564	0,49522974162662	0,864110368507252
0.677088525	0,498349788920182	0,864539036722747
0.676444515	0,498758462613273	0,864562752028483
0.676222133	0,498899622178297	0,864562752028483
-0.672969042	0,500966986337011	0,865864084865809
0.673474785	0,500645284891343	0,865864084865809
0.673387577	0,500700749867905	0,865864084865809
-0.672928175	0,50099298677817	0,865864084865809
-0.673233793	0,500798565319848	0,865864084865809
-0.672278811	0,501406216571533	0,866114112717297
0.670470523	0,502557890894877	0,866247567149107
0.670496071	0,502541610319387	0,866247567149107
0.670555952	0,502503451152215	0,866247567149107
-0.671551758	0,501869098775752	0,866247567149107
0.66978963	0,50299190535988	0,866532529265818
-0.667891582	0,504202801499677	0,866857278712826
-0.668278939	0,503955554802794	0,866857278712826
0.668703769	0,503684462945065	0,866857278712826
0.667652991	0,504355123468241	0,866857278712826
0.666967068	0,504793168813236	0,866857278712826
-0.667104345	0,504705484738171	0,866857278712826
-0.664778532	0,506192155526661	0,868797073748527
0.663604779	0,506943297855119	0,869161156609654
-0.663631142	0,506926420653032	0,869161156609654
0.662405311	0,507711500656008	0,869553685403943
-0.662738499	0,507498048593666	0,869553685403943
0.661662613	0,508187470137745	0,869906895538337
0.655010781	0,512460812822181	0,870001059327008
-0.654439815	0,512828490060892	0,870001059327008
0.657999429	0,51053849819837	0,870001059327008
0.658066573	0,510495353852974	0,870001059327008
0.656788259	0,511317074367266	0,870001059327008
-0.656736359	0,511350451211184	0,870001059327008
0.657570644	0,510814063119159	0,870001059327008
-0.654118703	0,513035332734534	0,870001059327008
-0.659796051	0,509384717701828	0,870001059327008
0.654588682	0,512732612797534	0,870001059327008
0.655112688	0,512395203689219	0,870001059327008
0.657162555	0,511076400026256	0,870001059327008
0.658552714	0,510183036235002	0,870001059327008
-0.654020964	0,513098299175184	0,870001059327008
-0.655217181	0,51232793417343	0,870001059327008
0.659744822	0,509417597824334	0,870001059327008
-0.655638905	0,512056487223667	0,870001059327008
-0.657482956	0,510870426926342	0,870001059327008
0.653450439	0,513465930592212	0,870166908123953
0.651361809	0,514812957746057	0,87056340941853

-0.651658009	0,514621816803672	0,87056340941853
-0.650613135	0,515296249545312	0,87056340941853
-0.652091112	0,514342397283123	0,87056340941853
-0.649755365	0,515850255092631	0,87056340941853
-0.652663964	0,513972939274154	0,87056340941853
0.649741157	0,515859434232189	0,87056340941853
-0.650558269	0,515331676051513	0,87056340941853
-0.64761208	0,517235882390836	0,871518140392082
-0.647912772	0,517041369257305	0,871518140392082
-0.64817567	0,5168713360688	0,871518140392082
0.64679342	0,51776565182351	0,871811913618829
-0.646507268	0,517950891936024	0,871811913618829
0.64445899	0,519277842422032	0,872527427567614
-0.644503927	0,519248711882984	0,872527427567614
0.643879722	0,519653432330409	0,872527427567614
0.645128514	0,518843906997307	0,872527427567614
-0.643763601	0,519728740575934	0,872527427567614
-0.638597618	0,523084731104677	0,873149503632037
-0.637179718	0,524007786998242	0,873149503632037
0.640474454	0,521864192324198	0,873149503632037
-0.63461581	0,525679014467403	0,873149503632037
0.635839294	0,524881173311076	0,873149503632037
0.636558592	0,524412404544347	0,873149503632037
-0.637215287	0,523984621412837	0,873149503632037
0.636987679	0,524132869292391	0,873149503632037
0.638695931	0,523020760263718	0,873149503632037
0.636004059	0,524773776502169	0,873149503632037
-0.634054023	0,526045566320804	0,873149503632037
-0.641484959	0,52120765023311	0,873149503632037
-0.635169427	0,525317920463628	0,873149503632037
-0.637176623	0,524009802686835	0,873149503632037
0.633623748	0,526326398468428	0,873149503632037
0.636601092	0,524384713956626	0,873149503632037
0.640834906	0,521629951269488	0,873149503632037
-0.640260306	0,522003382072243	0,873149503632037
-0.639974423	0,522189227775316	0,873149503632037
-0.635341413	0,525205769720156	0,873149503632037
0.635900069	0,524841557559048	0,873149503632037
-0.634024305	0,526064960342876	0,873149503632037
-0.63620395	0,524643498878434	0,873149503632037
-0.632323317	0,527175630242833	0,874073431606183
0.631941055	0,527425394699421	0,874073431606183
-0.628629691	0,529591517314272	0,876489234690887
-0.628766703	0,52950180156538	0,876489234690887
-0.628466393	0,529698455321718	0,876489234690887
0.626915874	0,530714380954662	0,877486094783914
0.626301557	0,531117164966884	0,877486094783914
-0.626704506	0,530852949370114	0,877486094783914
-0.624849765	0,532069665085908	0,878609662008221
-0.617783317	0,536718181909375	0,881074869436761

-0.620158044	0,535153741230361	0,881074869436761
0.619412437	0,535644689953374	0,881074869436761
-0.618467995	0,536266888622644	0,881074869436761
-0.617253985	0,537067212592778	0,881074869436761
0.617242861	0,53707454863457	0,881074869436761
-0.617182786	0,537114168469046	0,881074869436761
-0.620689056	0,534804231175229	0,881074869436761
0.618099652	0,536509651952658	0,881074869436761
-0.619426445	0,53563546403481	0,881074869436761
-0.619430021	0,535633108963477	0,881074869436761
-0.620248197	0,535094394793321	0,881074869436761
-0.619648407	0,535489289743417	0,881074869436761
-0.61659882	0,537499373174888	0,88115673361892
-0.616278801	0,53771052767815	0,88115673361892
-0.614115511	0,539138999262239	0,88277506771959
0.613954272	0,539245545242665	0,88277506771959
-0.613037206	0,539851738791103	0,882871124544274
0.613317743	0,539666263508562	0,882871124544274
-0.609017461	0,542512864994886	0,883922674711391
0.609362871	0,542283942737417	0,883922674711391
0.610948777	0,541233491804417	0,883922674711391
0.6084238	0,542906430663077	0,883922674711391
0.610755344	0,541361560849203	0,883922674711391
-0.609269881	0,542345567174265	0,883922674711391
-0.608340758	0,542961494140548	0,883922674711391
0.611549878	0,540835608141703	0,883922674711391
0.610260247	0,541689426061705	0,883922674711391
0.607727287	0,543368362357907	0,884138732898208
-0.605737893	0,544688817507696	0,884502233868238
0.60632796	0,544296995506594	0,884502233868238
0.606035538	0,544491154745291	0,884502233868238
-0.606685586	0,544059589448526	0,884502233868238
0.605235276	0,545022679853821	0,884598964533755
0.594924125	0,551894190499732	0,886093683171482
0.597882208	0,54991853250911	0,886093683171482
-0.598095723	0,549776063782246	0,886093683171482
0.591943401	0,553888489916974	0,886093683171482
0.593216376	0,553036354720724	0,886093683171482
-0.601266864	0,547662256309363	0,886093683171482
-0.59692752	0,550555772868066	0,886093683171482
-0.600339804	0,548279796550366	0,886093683171482
-0.598650731	0,549405818361376	0,886093683171482
0.594522159	0,552162925883282	0,886093683171482
-0.596473626	0,550858867795176	0,886093683171482
0.592572496	0,553467290600228	0,886093683171482
-0.591991006	0,553856611087856	0,886093683171482
-0.598884124	0,549250159331639	0,886093683171482
-0.601875551	0,547256980052473	0,886093683171482
-0.599183421	0,549050577220513	0,886093683171482
-0.593360751	0,552939750017283	0,886093683171482

0.591088535	0,554461101593814	0,886093683171482
0.591878664	0,553931841810225	0,886093683171482
-0.598813523	0,549297243433074	0,886093683171482
-0.599493693	0,548843714481151	0,886093683171482
0.591557667	0,554146828469555	0,886093683171482
-0.59700265	0,550505611390173	0,886093683171482
0.597815317	0,549963169321118	0,886093683171482
0.593584177	0,552790266870208	0,886093683171482
0.594018984	0,552499415845629	0,886093683171482
-0.602821073	0,546627726706257	0,886093683171482
0.601537171	0,547482262024827	0,886093683171482
-0.591272876	0,554337600226807	0,886093683171482
-0.597985696	0,549849477483455	0,886093683171482
0.59218984	0,553723472252046	0,886093683171482
-0.589206438	0,555722799074412	0,887209767529638
-0.588405881	0,556259891455792	0,887209767529638
0.58870703	0,556057821317886	0,887209767529638
0.588669771	0,556082819968697	0,887209767529638
-0.586723514	0,557389412582255	0,887578931591027
0.584610669	0,558809531975111	0,887578931591027
0.586916	0,557260123223243	0,887578931591027
0.585177731	0,558428216720104	0,887578931591027
-0.583965488	0,559243531470688	0,887578931591027
0.586941007	0,557243328086102	0,887578931591027
0.585887727	0,557950965181264	0,887578931591027
0.58573745	0,558051963476307	0,887578931591027
-0.584333497	0,558995959964353	0,887578931591027
0.584788029	0,558690254647903	0,887578931591027
-0.582372998	0,56031546507997	0,888405789027976
0.582378976	0,560311439290811	0,888405789027976
-0.580992975	0,56124518787954	0,889442619612539
-0.580565645	0,561533232161431	0,889462020491461
-0.579806959	0,562044804515611	0,889835294336203
-0.57859615	0,562861703449894	0,890691360954812
0.577140287	0,563844691079653	0,890787591750711
0.576984987	0,563949597163897	0,890787591750711
0.575479811	0,564966840840641	0,890787591750711
-0.576854652	0,564037646806906	0,890787591750711
0.576141898	0,564519273718232	0,890787591750711
0.575020215	0,565277625475688	0,890787591750711
-0.577699859	0,56346677458016	0,890787591750711
-0.575233623	0,565133306157649	0,890787591750711
0.574826808	0,5654084342058	0,890787591750711
0.574411933	0,565689079926584	0,890794571661735
-0.572415421	0,567040571256979	0,891537523383004
-0.572081641	0,567266666040907	0,891537523383004
0.572246394	0,567155061089275	0,891537523383004
-0.572378564	0,56706553466561	0,891537523383004
-0.571033974	0,567976613216199	0,892109746687849
-0.570728043	0,568184006107548	0,892109746687849

-0.569765111	0,568837022231774	0,892700436349134
-0.567619365	0,570293455007857	0,894140474364677
0.56759628	0,570309133571516	0,894140474364677
0.567166775	0,570600879079723	0,894163185146796
0.565041745	0,572045370440932	0,894254153985461
0.565284327	0,571880387044488	0,894254153985461
0.565673783	0,571615560569604	0,894254153985461
-0.565940049	0,571434535207674	0,894254153985461
-0.565924133	0,571445355637161	0,894254153985461
-0.564106202	0,57268185721102	0,894382077242393
0.564119104	0,572673077505793	0,894382077242393
-0.558364681	0,576595385318329	0,894431819238565
-0.557721144	0,577034817364675	0,894431819238565
0.55714474	0,577428541908432	0,894431819238565
0.557800539	0,576980594642988	0,894431819238565
-0.557214724	0,577380730817521	0,894431819238565
0.559867946	0,575569514159107	0,894431819238565
-0.560760224	0,574961005417228	0,894431819238565
-0.560073994	0,575428968086491	0,894431819238565
0.563320787	0,573216465559564	0,894431819238565
0.557402143	0,577252701525215	0,894431819238565
-0.559523691	0,575804367711327	0,894431819238565
-0.56131086	0,574585639353399	0,894431819238565
0.559504786	0,575817265602342	0,894431819238565
0.561425955	0,574507194310306	0,894431819238565
-0.557312362	0,577314031001872	0,894431819238565
0.562941876	0,573474463699524	0,894431819238565
0.562766085	0,573594177562124	0,894431819238565
0.552698117	0,580470142771272	0,897625086930728
-0.552103382	0,580877524772939	0,897625086930728
0.551502349	0,581289356716179	0,897625086930728
-0.551662808	0,581179396131003	0,897625086930728
-0.551716617	0,581142523175537	0,897625086930728
0.551284898	0,581438389642881	0,897625086930728
-0.551519849	0,581277363693197	0,897625086930728
0.550748251	0,581806263069981	0,897763252823296
-0.54983129	0,582435094858023	0,898303768970408
-0.54871475	0,583201222307119	0,898636432262679
0.548704761	0,583208078364585	0,898636432262679
-0.547349222	0,584138833805876	0,899300330209638
-0.547265054	0,584196648616804	0,899300330209638
-0.537856863	0,590675870986743	0,89947444141212
-0.531628716	0,59498317216405	0,89947444141212
-0.537359236	0,591019495591756	0,89947444141212
-0.533993247	0,593346206216906	0,89947444141212
-0.542679058	0,587350795889142	0,89947444141212
0.532501192	0,594378916262665	0,89947444141212
-0.531997695	0,594727592031208	0,89947444141212
0.543708537	0,58664205652604	0,89947444141212
-0.532096691	0,594659029145513	0,89947444141212

0.538689708	0,590100976277811	0,89947444141212
-0.53051823	0,595752672725899	0,89947444141212
-0.532520982	0,594365213484579	0,89947444141212
-0.540228471	0,589039480022643	0,89947444141212
0.534106292	0,593267996826617	0,89947444141212
0.540199263	0,589059620940216	0,89947444141212
0.535743828	0,592135608577624	0,89947444141212
0.545965507	0,585089647856279	0,89947444141212
0.544857068	0,585851825245727	0,89947444141212
-0.539317235	0,58966797864209	0,89947444141212
-0.531992905	0,594730909844364	0,89947444141212
-0.530127059	0,596023839162077	0,89947444141212
-0.532700326	0,594241040382463	0,89947444141212
-0.532943958	0,594072375137041	0,89947444141212
0.541143348	0,58840878225668	0,89947444141212
-0.539396979	0,589612965001185	0,89947444141212
-0.540314956	0,588979846026543	0,89947444141212
0.538299049	0,590370607674948	0,89947444141212
0.54115194	0,588402860479003	0,89947444141212
0.546502409	0,58472063200087	0,89947444141212
0.53120983	0,59527338179261	0,89947444141212
0.533323624	0,593809577007866	0,89947444141212
-0.530926037	0,595470033977478	0,89947444141212
-0.53373144	0,593527353562051	0,89947444141212
-0.543481058	0,586798629542156	0,89947444141212
-0.54104043	0,588479715775973	0,89947444141212
-0.541884925	0,587897782440555	0,89947444141212
-0.537219318	0,59111612865243	0,89947444141212
0.53131211	0,595202515050518	0,89947444141212
0.537877571	0,590661574127036	0,89947444141212
-0.535829057	0,592076698240137	0,89947444141212
-0.544693943	0,585964031605474	0,89947444141212
-0.535403996	0,592370527487589	0,89947444141212
0.528819084	0,596930957490234	0,900001092990185
-0.528997567	0,59680713781516	0,900001092990185
-0.526774625	0,598350106187032	0,900400652203339
0.524917188	0,59964076003957	0,900400652203339
0.527500796	0,597845863948181	0,900400652203339
0.526991283	0,598199641697144	0,900400652203339
-0.524819444	0,599708713467527	0,900400652203339
-0.526560469	0,598498850089147	0,900400652203339
-0.526291634	0,598685595063593	0,900400652203339
-0.525247533	0,59941112539211	0,900400652203339
0.525314984	0,599364242690883	0,900400652203339
-0.523558165	0,600585885736916	0,90095441912688
-0.522447871	0,60135853264098	0,90095441912688
-0.522767683	0,601135931232477	0,90095441912688
-0.522413473	0,60138247747751	0,90095441912688
-0.521761584	0,601836340549561	0,90095441912688
-0.521942617	0,601710284339703	0,90095441912688

0.521154741	0,602258979578357	0,90095441912688
0.521077946	0,602312473686063	0,90095441912688
0.519646415	0,603310041365655	0,902028225963949
0.518519555	0,604095820092483	0,902784531880564
0.51394459	0,607290731243545	0,903530679119129
-0.514120956	0,607167427032777	0,903530679119129
0.513968238	0,607274196923063	0,903530679119129
0.515929425	0,605903701600394	0,903530679119129
-0.516289361	0,60565232559654	0,903530679119129
-0.513792964	0,607396748009386	0,903530679119129
-0.516163181	0,605740443576734	0,903530679119129
0.515104334	0,60648011421349	0,903530679119129
0.514474347	0,606920390880213	0,903530679119129
0.51390132	0,607320984353184	0,903530679119129
0.513316046	0,607730261338755	0,903610001298979
-0.512655979	0,608191988035224	0,903879797886451
-0.509034075	0,610728339862388	0,903900365330979
-0.509793489	0,610196147946105	0,903900365330979
0.511111138	0,609273237116306	0,903900365330979
0.509035329	0,610727460962763	0,903900365330979
-0.510375854	0,609788170153088	0,903900365330979
0.509551095	0,610365993490402	0,903900365330979
0.511746778	0,608828243227464	0,903900365330979
0.510662309	0,609587537308793	0,903900365330979
-0.511914056	0,608711160177956	0,903900365330979
-0.508560473	0,611060340729023	0,903976880206926
0.506418653	0,612562782618701	0,905784032070293
-0.504180328	0,614134663042327	0,906031238934815
-0.504333009	0,614027385312448	0,906031238934815
-0.504270113	0,614071576355867	0,906031238934815
-0.505699931	0,6130673169385	0,906031238934815
-0.5048909	0,613635465664943	0,906031238934815
-0.503449398	0,614648348504896	0,90611157752984
0.503303297	0,614751048569082	0,90611157752984
0.500846881	0,616478889186596	0,906926484256798
-0.500840519	0,616483366408908	0,906926484256798
-0.500518534	0,616710009294622	0,906926484256798
0.501054902	0,61633248485909	0,906926484256798
0.501957761	0,615697233492142	0,906926484256798
-0.499813989	0,617206059293589	0,907242270383693
-0.497919936	0,618540475100978	0,907549150227777
0.497934659	0,618530097761608	0,907549150227777
0.499080772	0,617722482760497	0,907549150227777
0.498656454	0,618021427221655	0,907549150227777
0.496958088	0,619218607650649	0,907972132220522
0.496712449	0,61939184213493	0,907972132220522
-0.495201878	0,620457624759502	0,909121235733482
-0.494586563	0,620891989417776	0,909344534910231
0.494146597	0,621202651726089	0,909386541904964
0.493173189	0,62189022047525	0,909567329266522

-0.493248382	0,621837095973652	0,909567329266522
0.492236102	0,622552445467401	0,909619344170357
-0.492481346	0,622379105746315	0,909619344170357
-0.491925598	0,622771941683147	0,909619344170357
0.49098226	0,623438994437783	0,910117309336749
-0.490645375	0,623677287948594	0,910117309336749
-0.487538702	0,625876623217046	0,912237880470401
0.487137847	0,626160647254514	0,912237880470401
-0.486927128	0,626309973813505	0,912237880470401
0.487222413	0,626100723719146	0,912237880470401
0.486595833	0,626544776819206	0,912237880470401
-0.486164797	0,626850328379471	0,912270897573915
-0.485062144	0,627632264452699	0,912554309482258
-0.48515358	0,627567407034168	0,912554309482258
0.4846849	0,627899879592965	0,912554309482258
0.484294442	0,628176920015694	0,912554309482258
-0.483159931	0,628982183759725	0,913312716175197
-0.482005749	0,629801862594266	0,913352726328677
-0.481127634	0,630425788777561	0,913352726328677
-0.482117902	0,629722193593765	0,913352726328677
-0.481197143	0,63037639140447	0,913352726328677
-0.481502565	0,630159357214764	0,913352726328677
0.480605938	0,630796594217336	0,913479576268931
0.478916311	0,631998166697912	0,91398838009003
-0.479374021	0,631672571946301	0,91398838009003
-0.479212647	0,631787358086607	0,91398838009003
0.477685754	0,632873885414566	0,914025203968642
0.47786206	0,63274838627547	0,914025203968642
0.478144365	0,632547457072135	0,914025203968642
0.477235865	0,633194174756479	0,914078430434039
0.47606304	0,634029467388576	0,914874734822442
0.47108505	0,637580001021638	0,914970466554201
-0.469346334	0,638822101169407	0,914970466554201
0.468949696	0,639105593304924	0,914970466554201
-0.470967245	0,637664125985203	0,914970466554201
-0.471857617	0,637028421850449	0,914970466554201
0.470605651	0,637922371196755	0,914970466554201
-0.474006124	0,635495539987346	0,914970466554201
-0.473886797	0,635580634572183	0,914970466554201
-0.473413683	0,635918069621552	0,914970466554201
0.468417109	0,639486335383928	0,914970466554201
-0.469750483	0,638533295308166	0,914970466554201
-0.471779424	0,637084239074968	0,914970466554201
0.469086372	0,639007900080369	0,914970466554201
-0.474228257	0,635337145409312	0,914970466554201
0.468502611	0,639425204529396	0,914970466554201
-0.470731262	0,637832656732318	0,914970466554201
-0.472333388	0,636688842517006	0,914970466554201
0.469086671	0,639007686183925	0,914970466554201
0.470102981	0,638281444190413	0,914970466554201

-0.467213065	0,640347447120522	0,915390300072555
0.467491669	0,640148151271122	0,915390300072555
-0.461994627	0,644085173508329	0,915623381294811
-0.461907801	0,644147439715314	0,915623381294811
0.462990735	0,643371008505172	0,915623381294811
-0.466492991	0,640862663441053	0,915623381294811
0.464085887	0,642586212839045	0,915623381294811
0.460638576	0,645057930256432	0,915623381294811
0.463545675	0,642973283515109	0,915623381294811
-0.461435538	0,644486159871867	0,915623381294811
-0.460882327	0,644883031747452	0,915623381294811
-0.460249649	0,645337037421118	0,915623381294811
0.461379364	0,644526453803321	0,915623381294811
0.461055806	0,644758567779558	0,915623381294811
0.462529415	0,643701713553059	0,915623381294811
-0.465328038	0,641696559359381	0,915623381294811
-0.462448533	0,643759702323863	0,915623381294811
-0.462427515	0,643774771427617	0,915623381294811
0.465023057	0,641914945163655	0,915623381294811
-0.443741793	0,6572292757644	0,916242042683998
-0.44207989	0,658431395954542	0,916242042683998
0.448812931	0,6535666099235	0,916242042683998
0.442927463	0,657818202222797	0,916242042683998
0.446274457	0,655399004827036	0,916242042683998
0.448579298	0,653735170487076	0,916242042683998
0.452703051	0,65076258250547	0,916242042683998
-0.444336143	0,656799574116921	0,916242042683998
0.451345327	0,651740680982849	0,916242042683998
0.452002126	0,651267450701987	0,916242042683998
-0.454940454	0,649152078583477	0,916242042683998
0.458167094	0,646832398535208	0,916242042683998
0.44141655	0,658911462915924	0,916242042683998
-0.439636444	0,660200440804338	0,916242042683998
0.446740766	0,655062244135735	0,916242042683998
0.447458866	0,654543781240122	0,916242042683998
-0.456844112	0,647783096582813	0,916242042683998
-0.440052015	0,659899434539615	0,916242042683998
-0.444175504	0,656915701468231	0,916242042683998
0.440811915	0,659349166863151	0,916242042683998
-0.44998365	0,652722230002451	0,916242042683998
-0.454063918	0,649782822610388	0,916242042683998
0.448229858	0,653987316483378	0,916242042683998
0.44730003	0,654658445445054	0,916242042683998
0.439871252	0,660030358163692	0,916242042683998
0.449256828	0,653246397284234	0,916242042683998
0.442880539	0,657852144396714	0,916242042683998
0.450694147	0,652210001505996	0,916242042683998
-0.458925681	0,646287535981054	0,916242042683998
-0.455056326	0,649068716720371	0,916242042683998
0.454689276	0,649332796972534	0,916242042683998

-0.444704315	0,656533450780844	0,916242042683998
0.439188098	0,660525248216826	0,916242042683998
-0.447626875	0,6544225040747	0,916242042683998
-0.457763059	0,647122678037038	0,916242042683998
-0.458196228	0,646811469343159	0,916242042683998
-0.441366258	0,658947865972733	0,916242042683998
0.442971982	0,657786000897909	0,916242042683998
0.444801278	0,656463370672355	0,916242042683998
-0.439158644	0,660546588911719	0,916242042683998
-0.440649791	0,659466550438719	0,916242042683998
0.441891532	0,658567698481327	0,916242042683998
-0.446188314	0,655461223275569	0,916242042683998
0.449877791	0,652798562104285	0,916242042683998
0.448991013	0,65343813972902	0,916242042683998
-0.444643486	0,656577416126642	0,916242042683998
0.456258577	0,64820404755403	0,916242042683998
-0.447813067	0,654288112080896	0,916242042683998
0.455677619	0,648621818358023	0,916242042683998
-0.443553766	0,657365238848534	0,916242042683998
-0.456284555	0,648185368942827	0,916242042683998
-0.444651216	0,656571828750662	0,916242042683998
0.438586926	0,660960872097528	0,916422533325248
0.437913988	0,661448635168961	0,916704704950537
-0.437248549	0,661931104040692	0,916979300056371
0.435238806	0,663389097888145	0,918604482906513
-0.434607456	0,663847381992984	0,918694371645948
0.434364537	0,664023745831536	0,918694371645948
-0.424875119	0,670927713581022	0,920670661989761
-0.425273528	0,670637288989138	0,920670661989761
-0.424870186	0,670931309418513	0,920670661989761
-0.428471586	0,668307816220571	0,920670661989761
0.425543963	0,670440180354035	0,920670661989761
0.42689303	0,669457242107659	0,920670661989761
0.426349925	0,669852883867513	0,920670661989761
-0.428847522	0,668034192356768	0,920670661989761
0.426915556	0,66944083440582	0,920670661989761
-0.424900363	0,670909309843101	0,920670661989761
-0.426265214	0,669914602615948	0,920670661989761
0.427256209	0,669192724687895	0,920670661989761
-0.425499723	0,670472423851313	0,920670661989761
-0.426701159	0,669597005979542	0,920670661989761
-0.431960365	0,665770219963728	0,920670661989761
0.428141646	0,668547997980515	0,920670661989761
-0.430101306	0,667121950187996	0,920670661989761
0.429730522	0,667391678047148	0,920670661989761
0.428931615	0,667972992117561	0,920670661989761
-0.424554078	0,67116177560866	0,920670661989761
0.424057487	0,671523889185029	0,920775740910594
-0.423032137	0,672271814158151	0,921409520042514
-0.422157125	0,672910334251074	0,921892875089088

0.421621624	0,673301220051409	0,922036702618172
-0.420253555	0,674300235313263	0,92259952366675
-0.420125331	0,674393898696786	0,92259952366675
-0.419883664	0,674570442420526	0,92259952366675
0.41945768	0,674881677287791	0,922633916597341
-0.416148995	0,677300979722061	0,922677352869808
-0.417318231	0,67644565520192	0,922677352869808
0.407631891	0,683543947488863	0,922677352869808
0.408911232	0,682604806026735	0,922677352869808
-0.41117819	0,680941879450371	0,922677352869808
-0.40910508	0,682462548401611	0,922677352869808
-0.409100577	0,682465852982549	0,922677352869808
0.417731746	0,676143259095685	0,922677352869808
-0.414302728	0,678652416446423	0,922677352869808
0.411369572	0,680801562448299	0,922677352869808
-0.408127824	0,683179833321947	0,922677352869808
-0.411390196	0,680786441892953	0,922677352869808
-0.407858689	0,683377423227056	0,922677352869808
0.407788385	0,683429041704358	0,922677352869808
-0.415808779	0,67754993453787	0,922677352869808
0.411865354	0,680438116257678	0,922677352869808
0.410430333	0,681490299189774	0,922677352869808
-0.415131432	0,678045691043732	0,922677352869808
-0.415387736	0,677858083436297	0,922677352869808
0.418247622	0,675766080983813	0,922677352869808
0.415600041	0,677702696972474	0,922677352869808
0.417453209	0,676346942424824	0,922677352869808
-0.413455746	0,679272739143342	0,922677352869808
-0.407306899	0,683782596390339	0,922677352869808
-0.40831012	0,6830460107312	0,922677352869808
0.410440929	0,681482527352975	0,922677352869808
0.412391864	0,680052225637052	0,922677352869808
-0.413790639	0,679027440399186	0,922677352869808
-0.415532534	0,677752103481518	0,922677352869808
-0.409787738	0,681961661764798	0,922677352869808
-0.407656581	0,683525818389407	0,922677352869808
-0.406669365	0,68425084356874	0,922889926279119
0.406313229	0,684512466251055	0,922889926279119
-0.404390915	0,685925279616535	0,924408285316894
-0.40104482	0,68838712595304	0,924489995422733
0.402725317	0,687150307162006	0,924489995422733
0.402516928	0,687303632498037	0,924489995422733
0.400802033	0,688565881862141	0,924489995422733
-0.403299685	0,686727773460506	0,924489995422733
-0.401292048	0,688205117636069	0,924489995422733
0.401900302	0,68775740038128	0,924489995422733
-0.40227631	0,687480687370388	0,924489995422733
-0.402418499	0,68737605801801	0,924489995422733
-0.400389611	0,688869575084865	0,924512850457216
0.399777248	0,689320590077313	0,924733320715197

-0.399035679	0,689866914564652	0,925081413501457
0.395145257	0,692735690772143	0,925219276941285
-0.396623725	0,691644954893044	0,925219276941285
-0.396265125	0,691909452287311	0,925219276941285
0.395623709	0,692382643619549	0,925219276941285
-0.398212653	0,690473440129784	0,925219276941285
-0.397061478	0,691322125961145	0,925219276941285
0.395795761	0,692255703918334	0,925219276941285
0.397166218	0,691244892137899	0,925219276941285
0.396208017	0,69195157767367	0,925219276941285
0.395005781	0,692838621337427	0,925219276941285
0.391934197	0,695106834509688	0,927864048548734
0.389453315	0,696940838897616	0,927912488781173
0.388382968	0,697732646602897	0,927912488781173
0.389679671	0,696773430580257	0,927912488781173
-0.390377777	0,696257218588451	0,927912488781173
-0.388750941	0,69746039531435	0,927912488781173
-0.389654957	0,696791707978221	0,927912488781173
0.38963019	0,696810024601207	0,927912488781173
-0.388883021	0,697362682654477	0,927912488781173
-0.39144304	0,695469783968264	0,927912488781173
-0.387158497	0,698638873225409	0,928352025608546
0.387485044	0,698397155083433	0,928352025608546
-0.386708546	0,698971988937164	0,928412135223375
-0.385675573	0,699736955445234	0,928858996805925
-0.385476447	0,699884453407255	0,928858996805925
0.382896548	0,701796471244721	0,931013418249373
0.378535062	0,705033152458016	0,931418748134436
-0.38107662	0,703146395274395	0,931418748134436
-0.377525286	0,705783276383775	0,931418748134436
-0.378002709	0,705428581219511	0,931418748134436
0.372378418	0,709611120671723	0,931418748134436
0.37578011	0,707080375860839	0,931418748134436
-0.377515269	0,7057907193996	0,931418748134436
-0.379629815	0,704220225691962	0,931418748134436
-0.379527663	0,704296065719473	0,931418748134436
-0.376256336	0,706726337261236	0,931418748134436
0.378427355	0,705113150344378	0,931418748134436
0.373867863	0,708502627291307	0,931418748134436
-0.37463672	0,707930660050136	0,931418748134436
0.378139505	0,705326962282479	0,931418748134436
0.375337757	0,707409289325004	0,931418748134436
-0.373000595	0,709148000946132	0,931418748134436
-0.378935616	0,704735675237884	0,931418748134436
0.376996674	0,706176076417643	0,931418748134436
0.37261283	0,709436622281905	0,931418748134436
-0.373439743	0,708821184720055	0,931418748134436
-0.373134338	0,709048463064893	0,931418748134436
-0.373108452	0,709067728185203	0,931418748134436
-0.372450078	0,709557774250705	0,931418748134436

0.375177709	0,707528307241039	0,931418748134436
0.374677265	0,707900502693171	0,931418748134436
-0.378475375	0,70507748385004	0,931418748134436
0.371391336	0,710346077986527	0,931857582030609
0.371153309	0,710523347042874	0,931857582030609
0.37076079	0,710815708176683	0,931862056451139
-0.370106635	0,711303038217363	0,931902498282001
0.369943645	0,711424480859003	0,931902498282001
0.36911358	0,712043067397745	0,931955719301026
0.369168624	0,712002041251797	0,931955719301026
-0.367632572	0,713147224022459	0,93302223021194
-0.366163208	0,714243294219963	0,933698672014342
-0.366431111	0,714043408157196	0,933698672014342
0.360951844	0,718135446344945	0,934526274323425
-0.358174817	0,720212492033936	0,934526274323425
0.361197142	0,717952078192664	0,934526274323425
-0.359050241	0,719557504513706	0,934526274323425
0.35757583	0,720660768676839	0,934526274323425
-0.358993869	0,719599675480791	0,934526274323425
0.363595237	0,716160283922941	0,934526274323425
0.36059051	0,718405584929825	0,934526274323425
0.359652741	0,719106837310019	0,934526274323425
-0.361279374	0,717890611246494	0,934526274323425
0.360885443	0,718185086144403	0,934526274323425
0.364002615	0,715856056969281	0,934526274323425
0.358696795	0,719821925936841	0,934526274323425
-0.362725611	0,716809866075612	0,934526274323425
0.363171095	0,716477078688256	0,934526274323425
-0.359579439	0,719161662104719	0,934526274323425
-0.359640563	0,719115945359963	0,934526274323425
0.360094675	0,718776334889002	0,934526274323425
-0.357560972	0,720671889687553	0,934526274323425
0.364490434	0,715491816124397	0,934526274323425
-0.357075226	0,721035490535562	0,934621968238419
-0.356572364	0,721411969522138	0,934734271478061
-0.356036332	0,721813356445683	0,934878744794107
-0.355617178	0,722127277355653	0,93490986329666
-0.347382183	0,728304216916899	0,935023679909554
-0.352964128	0,724115333738075	0,935023679909554
-0.347911835	0,727906397056166	0,935023679909554
-0.345656314	0,729601018781948	0,935023679909554
-0.346367872	0,729066266760762	0,935023679909554
0.350138222	0,726234966867459	0,935023679909554
0.346038131	0,729314058177281	0,935023679909554
0.347970664	0,727862214993677	0,935023679909554
0.352531263	0,724439877114853	0,935023679909554
0.351529819	0,72519090570785	0,935023679909554
0.34539882	0,729794564386166	0,935023679909554
0.355041121	0,722558784931261	0,935023679909554
0.346811678	0,728732803111879	0,935023679909554

-0.351172576	0,72545888307534	0,935023679909554
-0.352265263	0,724639336477112	0,935023679909554
-0.34950741	0,72670840867315	0,935023679909554
-0.346369195	0,729065272918272	0,935023679909554
-0.348492471	0,727470367389318	0,935023679909554
0.345474386	0,729737763572819	0,935023679909554
0.344296715	0,730623154533977	0,935023679909554
0.345961258	0,729371830283744	0,935023679909554
0.344539254	0,730440780797257	0,935023679909554
-0.354107195	0,72325855038195	0,935023679909554
0.352857652	0,724195160346313	0,935023679909554
0.353152689	0,723973974156434	0,935023679909554
0.351216466	0,725425957853229	0,935023679909554
0.351192029	0,725444289326529	0,935023679909554
-0.349119483	0,726999610492408	0,935023679909554
-0.344938466	0,730140631472043	0,935023679909554
0.343604098	0,731144042585508	0,93520457161945
0.343337631	0,731344474302094	0,93520457161945
-0.341092646	0,733033836948752	0,935449260639196
0.339012767	0,734600112778275	0,935449260639196
0.341641189	0,732620936010048	0,935449260639196
0.337690161	0,73559668991659	0,935449260639196
0.338056762	0,735320413271583	0,935449260639196
0.338071863	0,735309033273187	0,935449260639196
-0.340501507	0,733478888125957	0,935449260639196
-0.338050811	0,735324897738495	0,935449260639196
0.33861804	0,734897490649678	0,935449260639196
-0.339060461	0,734564183444604	0,935449260639196
-0.341149105	0,732991335317263	0,935449260639196
-0.338894016	0,734689572809709	0,935449260639196
-0.342260443	0,73215490198207	0,935449260639196
0.34137878	0,732818447623303	0,935449260639196
-0.336928517	0,736170787353756	0,93578912845575
-0.336565746	0,736444281556804	0,93578912845575
-0.335210717	0,737466138474862	0,936718509878468
-0.3332906	0,738914933920017	0,937117107514791
-0.328187	0,742770276244879	0,937117107514791
-0.332721563	0,739344471015104	0,937117107514791
-0.327869454	0,743010370206301	0,937117107514791
0.332447929	0,739551052121629	0,937117107514791
-0.328265844	0,742710667448624	0,937117107514791
-0.329562757	0,741730367696201	0,937117107514791
0.333016552	0,739121789242825	0,937117107514791
-0.33178397	0,74005238904745	0,937117107514791
-0.33058233	0,740959994887049	0,937117107514791
-0.332259979	0,73969295706582	0,937117107514791
-0.328243019	0,742727923607787	0,937117107514791
-0.33255137	0,739472956583006	0,937117107514791
0.330310674	0,741165228627374	0,937117107514791
0.329974751	0,741419040096226	0,937117107514791

-0.32902651	0,74213565029539	0,937117107514791
-0.331637071	0,740163323969537	0,937117107514791
-0.330198969	0,741249626043038	0,937117107514791
0.326340148	0,744167015525437	0,938019120096419
-0.326154764	0,744307264597438	0,938019120096419
0.324706451	0,745403251782865	0,939033393359273
0.324126984	0,745841898408725	0,939219102838008
-0.322113888	0,747366417376664	0,940285362627887
-0.321853993	0,747563308458264	0,940285362627887
0.322060563	0,747406814038175	0,940285362627887
-0.320597265	0,748515610224265	0,940385044937233
-0.320594988	0,748517336543838	0,940385044937233
0.320857428	0,748318436654153	0,940385044937233
-0.318390254	0,750188930020946	0,940461160063868
-0.31955573	0,749305138611552	0,940461160063868
0.319477252	0,749364638859359	0,940461160063868
0.318937945	0,749773569873548	0,940461160063868
-0.318207404	0,750327617005995	0,940461160063868
-0.319086518	0,749660906641051	0,940461160063868
-0.315902388	0,752076599624414	0,940825071291209
-0.316365984	0,751724732903468	0,940825071291209
0.316254083	0,751809660400762	0,940825071291209
0.317030167	0,751220712200777	0,940825071291209
0.316452215	0,751659289529321	0,940825071291209
0.314983128	0,75277446453492	0,941332938396711
0.313439395	0,75394685800292	0,94243357250365
0.31152643	0,755400453439606	0,943130442174386
0.301375811	0,763127937112777	0,943130442174386
0.309511305	0,756932613820247	0,943130442174386
0.302853427	0,762001565453431	0,943130442174386
-0.312108919	0,754957748168129	0,943130442174386
-0.301435236	0,763082628265294	0,943130442174386
0.307454283	0,758497615808354	0,943130442174386
0.302545066	0,762236584433148	0,943130442174386
0.304923034	0,76042477221875	0,943130442174386
-0.305522482	0,759968249493728	0,943130442174386
-0.310629876	0,756082012549192	0,943130442174386
-0.302786598	0,762052497882316	0,943130442174386
-0.301179449	0,76327766017834	0,943130442174386
0.304649505	0,760633111805048	0,943130442174386
0.310633113	0,756079550951458	0,943130442174386
0.306725564	0,759052269732388	0,943130442174386
0.303431498	0,761561044697637	0,943130442174386
-0.302853662	0,762001386076173	0,943130442174386
0.303603478	0,761430001694887	0,943130442174386
-0.306177421	0,759469562838428	0,943130442174386
-0.303043078	0,761857032843742	0,943130442174386
-0.303180297	0,761752463568211	0,943130442174386
-0.306509878	0,759216460056339	0,943130442174386
-0.30792055	0,758142788653121	0,943130442174386

0.303429922	0,761562245629462	0,943130442174386
0.301486322	0,763043678413165	0,943130442174386
0.309332557	0,757068567574047	0,943130442174386
-0.301558917	0,762988330050258	0,943130442174386
0.304027049	0,761107284015565	0,943130442174386
0.302569764	0,762217759820757	0,943130442174386
-0.300496416	0,763798529846926	0,943412584740075
-0.299315131	0,764699610563512	0,943805025986501
-0.299312575	0,764701560589993	0,943805025986501
-0.297719038	0,765917611579011	0,944944260651228
0.296756328	0,766652549419323	0,945431644976034
-0.296433596	0,766898971552653	0,945431644976034
-0.294893055	0,768075577197889	0,945436540634806
0.295440431	0,767657451407418	0,945436540634806
0.295766423	0,767408467246567	0,945436540634806
-0.295148375	0,767880536752717	0,945436540634806
-0.285724605	0,775089058028288	0,945749621253379
-0.293095799	0,769448927876031	0,945749621253379
-0.286094054	0,77480608538208	0,945749621253379
-0.291536829	0,770640780826606	0,945749621253379
0.287926751	0,773402809125524	0,945749621253379
-0.287092387	0,774041581381659	0,945749621253379
-0.288715674	0,772798967413462	0,945749621253379
-0.28673844	0,774312602328651	0,945749621253379
-0.291519151	0,770654298988656	0,945749621253379
-0.293165128	0,769395937570676	0,945749621253379
0.285568447	0,775208673434158	0,945749621253379
0.285516666	0,775248338005116	0,945749621253379
-0.291315647	0,770809921962333	0,945749621253379
-0.292759934	0,769705654958326	0,945749621253379
0.285360376	0,775368061579515	0,945749621253379
-0.288949542	0,772619991084574	0,945749621253379
0.290481364	0,771448005909088	0,945749621253379
-0.285387295	0,775347439869794	0,945749621253379
0.291484707	0,770680638169592	0,945749621253379
-0.287686893	0,773586424283912	0,945749621253379
-0.286024533	0,77485933165738	0,945749621253379
0.287886472	0,773433642200255	0,945749621253379
0.289019169	0,772566708675746	0,945749621253379
-0.293181568	0,76938337192995	0,945749621253379
0.283835907	0,776536135723281	0,946458442066357
0.283942206	0,776454671618539	0,946458442066357
0.282656061	0,777440501436317	0,947202726532725
-0.280132709	0,779375690454113	0,948090290905581
-0.280810711	0,77885558783308	0,948090290905581
-0.280281285	0,779261707701505	0,948090290905581
0.279789641	0,779638899684217	0,948090290905581
0.280746821	0,77890459399517	0,948090290905581
0.279384471	0,779949786947458	0,948110841653054
0.277192516	0,781632286315205	0,949574641935512

0.276980746	0,781794890447835	0,949574641935512
-0.276665082	0,78203728650565	0,949574641935512
-0.275310369	0,783077798873389	0,950480203751103
0.274848695	0,783432484651102	0,950552958239204
0.274297782	0,783855788742296	0,950708882547539
-0.270433981	0,786826400583597	0,951528783962207
0.272070048	0,785568159705696	0,951528783962207
0.271646892	0,785893539897943	0,951528783962207
-0.270348475	0,786892175760374	0,951528783962207
-0.271469333	0,786030082950455	0,951528783962207
-0.271025886	0,786371123245354	0,951528783962207
-0.270846437	0,786509142345484	0,951528783962207
-0.270675274	0,786640795690705	0,951528783962207
-0.269793097	0,787319433431648	0,951616741720178
0.269291703	0,787705216446545	0,951616741720178
0.269103367	0,787850139656705	0,951616741720178
0.268411017	0,788382959941835	0,951903798507083
-0.267928561	0,788754308702381	0,95199575058577
-0.265591509	0,790553825320338	0,953180871476549
-0.266039603	0,790208709078604	0,953180871476549
-0.265284846	0,790790037240651	0,953180871476549
0.26473448	0,791214013315573	0,953180871476549
-0.264821479	0,791146989455713	0,953180871476549
0.263262547	0,792348225164931	0,954190823807656
0.261780199	0,7934909060384	0,954912844857452
-0.260927258	0,794148603644981	0,954912844857452
-0.260564632	0,794428267520169	0,954912844857452
-0.26073399	0,794297651858526	0,954912844857452
0.260715252	0,794312103363976	0,954912844857452
-0.260114404	0,794775527515985	0,954974320506353
0.25710807	0,797095348164655	0,955332463528153
0.25576714	0,798130648719446	0,955332463528153
0.259129323	0,795535460669246	0,955332463528153
-0.258702893	0,795864487406813	0,955332463528153
0.255746948	0,79814624085897	0,955332463528153
0.255580387	0,79827486412111	0,955332463528153
0.257444881	0,796835360464204	0,955332463528153
0.256090829	0,79788070315649	0,955332463528153
0.257744881	0,79660380591617	0,955332463528153
0.256791343	0,797339853096737	0,955332463528153
0.255506285	0,798332089677015	0,955332463528153
-0.254527728	0,799087885677551	0,955715287649321
0.254324736	0,799244691717898	0,955715287649321
0.253754855	0,799684952649993	0,955887313675399
0.253275769	0,800055119632858	0,955975457879203
0.2524224	0,800714587764082	0,956409090940431
-0.251819827	0,801180331634983	0,956611095713743
-0.250933017	0,80186589716839	0,95707532137974
-0.2494248	0,803032203940629	0,957799144577079
0.249380689	0,803066321530673	0,957799144577079

-0.246733335	0,805114606485131	0,959887100153252
-0.244346767	0,806962266357775	0,959960645150802
-0.244443858	0,806887078258013	0,959960645150802
-0.245131284	0,806354780292883	0,959960645150802
0.24578125	0,805851572144567	0,959960645150802
-0.244426329	0,806900652613178	0,959960645150802
0.245161348	0,806331502734001	0,959960645150802
-0.243561304	0,807570600520146	0,960330083583138
0.24317097	0,807872954294474	0,960335524364054
0.241738573	0,808982738938728	0,961021357300284
0.241657083	0,809045886843495	0,961021357300284
-0.240434514	0,809993424757706	0,961347735917818
0.239549791	0,810679292123522	0,961347735917818
-0.239456404	0,810751697746564	0,961347735917818
0.23996973	0,810353723220221	0,961347735917818
-0.239379772	0,81081111370433	0,961347735917818
0.238234807	0,811698982452562	0,961373397654908
0.237944035	0,81192450147201	0,961373397654908
0.238696696	0,811340779174568	0,961373397654908
-0.23781426	0,812025158205262	0,961373397654908
0.236774686	0,812831591073036	0,961420751939621
-0.23660982	0,812959500942433	0,961420751939621
-0.236986395	0,812667345090258	0,961420751939621
0.235551375	0,813780809103386	0,961686738497039
-0.235741745	0,813633074852897	0,961686738497039
-0.233340709	0,815496849375942	0,963323214225617
0.232156042	0,816416818352628	0,963323214225617
-0.232858496	0,815871287627558	0,963323214225617
-0.231844076	0,816659121765221	0,963323214225617
-0.232144604	0,816425701898597	0,963323214225617
0.231155976	0,817193629540061	0,963601263351626
0.229717001	0,818311683399763	0,963905931269257
-0.229993963	0,818096461196126	0,963905931269257
0.229669414	0,818348663508597	0,963905931269257
-0.229144482	0,818756621167443	0,964034356796277
0.223643143	0,823034990875435	0,96449412993215
0.224645082	0,822255390723873	0,96449412993215
0.224551139	0,822328479838829	0,96449412993215
0.224604112	0,822287265969067	0,96449412993215
0.225948351	0,821241591099107	0,96449412993215
0.22436926	0,822469988087243	0,96449412993215
-0.224212411	0,822592026611889	0,96449412993215
-0.227955409	0,819680906311466	0,96449412993215
-0.224920303	0,822041274726887	0,96449412993215
0.223829585	0,822889908678542	0,96449412993215
0.224155931	0,822635973139588	0,96449412993215
0.223920941	0,822818820853473	0,96449412993215
-0.223794799	0,822916977483478	0,96449412993215
-0.223058889	0,823489674186908	0,964676425445978
0.220888753	0,825179055282729	0,96530832826888

0.220827981	0,825226375515443	0,96530832826888
0.220967285	0,825117905901586	0,96530832826888
-0.221422843	0,824763207545722	0,96530832826888
0.219450286	0,826299301884201	0,965516008560491
0.219446729	0,826302072442467	0,965516008560491
0.220048758	0,825833181970323	0,965516008560491
-0.218512833	0,827029560639931	0,966016056886555
-0.217925144	0,827487435756401	0,966200934219549
0.216854533	0,828321712024244	0,966825016749253
-0.21646783	0,828623098923261	0,966826879170593
0.214518503	0,830142739666136	0,966896060567965
-0.211971931	0,832128932661337	0,966896060567965
-0.212228509	0,83192876650044	0,966896060567965
0.21260123	0,831638011619714	0,966896060567965
0.213318152	0,831078816172878	0,966896060567965
0.212446585	0,831758645901428	0,966896060567965
-0.215506337	0,829372571909475	0,966896060567965
0.211778114	0,832280143918348	0,966896060567965
-0.211857989	0,832217826239025	0,966896060567965
0.212057126	0,832062467565904	0,966896060567965
-0.214837052	0,82989436411504	0,966896060567965
0.215294903	0,829537403308669	0,966896060567965
-0.208317638	0,834980960770406	0,967383410124056
-0.208080314	0,835166257551114	0,967383410124056
-0.20864979	0,83472163911163	0,967383410124056
-0.207781835	0,835399316959843	0,967383410124056
0.209107206	0,834364549688847	0,967383410124056
0.209721754	0,833884845985986	0,967383410124056
-0.208278501	0,835011517001622	0,967383410124056
-0.20793113	0,835282741917725	0,967383410124056
-0.208806518	0,834599282787986	0,967383410124056
0.202726328	0,839348943823645	0,967431774805664
-0.201501485	0,840306468555463	0,967431774805664
0.202770872	0,839314125420834	0,967431774805664
0.206909632	0,836080434046508	0,967431774805664
0.200447001	0,841131003484058	0,967431774805664
-0.204354433	0,838076535733255	0,967431774805664
-0.202856465	0,839247222833203	0,967431774805664
-0.202279506	0,839698220761084	0,967431774805664
-0.201455382	0,840342514251825	0,967431774805664
-0.200051045	0,841440659947252	0,967431774805664
-0.200143108	0,841368659815695	0,967431774805664
0.201237691	0,840512721469538	0,967431774805664
0.205923172	0,836850924936726	0,967431774805664
0.202242526	0,839727128924155	0,967431774805664
-0.204741498	0,837774096400228	0,967431774805664
-0.203485301	0,838755733727556	0,967431774805664
0.206464334	0,836428222106962	0,967431774805664
0.205792524	0,83695298096996	0,967431774805664
0.200339087	0,841215395228891	0,967431774805664

0.206143621	0,836678725469059	0,967431774805664
-0.199548273	0,841833885980111	0,967538945932237
0.198729004	0,842474734545088	0,967930537551803
0.197020563	0,843811446023312	0,968776046075181
0.197264281	0,843620729752304	0,968776046075181
0.196350095	0,844336154377859	0,969033486785976
-0.19586624	0,844714861886654	0,969123240691732
0.19501137	0,845384046334189	0,969546070209019
0.193060309	0,846911736719433	0,969619375152358
-0.193009856	0,846951249551687	0,969619375152358
0.193249723	0,846763399193618	0,969619375152358
-0.194117961	0,846083518385158	0,969619375152358
0.194048084	0,846138231862835	0,969619375152358
-0.192194458	0,847589886676994	0,969662073264741
-0.192573812	0,847292756178033	0,969662073264741
0.191766158	0,847925381239167	0,969701898757558
0.191144741	0,848412195536619	0,969914686496135
0.190068275	0,8492556300407	0,970534871325746
0.187855297	0,850990086471122	0,971483111417504
-0.187472329	0,85129031716771	0,971483111417504
0.18812326	0,850780028135626	0,971483111417504
0.188581265	0,850421017757583	0,971483111417504
-0.1867211	0,851879311689645	0,971574942832179
-0.186247726	0,852250498721949	0,971574942832179
-0.185785288	0,852613142316384	0,971574942832179
0.185718887	0,852665216030557	0,971574942832179
-0.185064598	0,853178368403328	0,971574942832179
-0.185197979	0,85307375409516	0,971574942832179
-0.184130151	0,853911353532986	0,972066401392121
0.182938258	0,854846463891914	0,972787525071074
-0.182049542	0,855543846453126	0,972894536252233
-0.18212949	0,855481106051079	0,972894536252233
0.181535538	0,855947241388018	0,973010170418173
-0.178174574	0,858585877174078	0,973297062708487
-0.178131284	0,858619873755436	0,973297062708487
-0.177886022	0,858812488367076	0,973297062708487
-0.178752757	0,858131841853003	0,973297062708487
0.177569253	0,859061273303658	0,973297062708487
-0.179726728	0,857367108990174	0,973297062708487
0.180774037	0,856544942967413	0,973297062708487
0.177370226	0,859217593032888	0,973297062708487
0.179708649	0,857381302973707	0,973297062708487
0.178177703	0,858583419622362	0,973297062708487
-0.175218864	0,860907662674998	0,973680755313652
-0.175634654	0,860580975254143	0,973680755313652
0.175017483	0,861065895861871	0,973680755313652
-0.175683402	0,860542675910244	0,973680755313652
0.175438029	0,860735461480557	0,973680755313652
-0.174516376	0,861459661676775	0,973784580759762
0.173980336	0,861880915787402	0,9738701395874

-0.173651568	0,862139301867295	0,9738701395874
-0.173132932	0,862546939928608	0,973989454226107
-0.17181083	0,863586251044827	0,974594615354001
-0.171682354	0,863687259126119	0,974594615354001
-0.17037761	0,864713179012964	0,974613872147115
-0.170195797	0,864856157182478	0,974613872147115
0.168970601	0,865819765488832	0,974613872147115
-0.169522299	0,865385834067457	0,974613872147115
0.169121305	0,865701227573373	0,974613872147115
0.169280594	0,865575939148767	0,974613872147115
0.171192833	0,864072143096997	0,974613872147115
0.16815287	0,866463017370027	0,974680213337959
0.168127273	0,866483153996877	0,974680213337959
-0.167243076	0,867178791618537	0,974914297155241
-0.167014965	0,867358274189621	0,974914297155241
-0.166710111	0,867598149716447	0,974914297155241
0.165920099	0,868219829821451	0,974933478821093
-0.166035666	0,868128882590747	0,974933478821093
0.163942151	0,869776683985843	0,976326462764236
-0.163574888	0,870065815188965	0,976326462764236
-0.162593401	0,870838583071497	0,976626241803173
-0.162288986	0,871078287868434	0,976626241803173
0.162081777	0,871241456641156	0,976626241803173
0.161348361	0,871819035357288	0,976934117104675
0.159725784	0,87309708929324	0,977686844781493
0.159893117	0,872965270901305	0,977686844781493
-0.159255597	0,873467502912927	0,977762130126411
-0.157553674	0,874808510521892	0,978244606946291
0.157799159	0,874615061293702	0,978244606946291
-0.15794669	0,874498806312448	0,978244606946291
-0.156703992	0,875478140938404	0,978555514914834
0.156423492	0,875699221197742	0,978555514914834
-0.156045916	0,875996828390426	0,978555514914834
0.155419455	0,876490647483005	0,978768122622123
-0.154411682	0,877285142927451	0,978977365377519
-0.154619502	0,877121294575885	0,978977365377519
0.153847393	0,87773006447426	0,979135059816495
-0.152155054	0,879064643174369	0,97963811863332
-0.152273514	0,878971214309135	0,97963811863332
0.152119969	0,879092314829404	0,97963811863332
-0.148829121	0,881688471341268	0,979865432359261
0.148600045	0,881869237525641	0,979865432359261
-0.149024169	0,881534561505573	0,979865432359261
0.147146465	0,883016414866445	0,979865432359261
0.148502112	0,881946519508352	0,979865432359261
-0.146914512	0,883199497101828	0,979865432359261
-0.150653789	0,880248827775874	0,979865432359261
0.150174918	0,88062661412664	0,979865432359261
-0.150434601	0,880421743895695	0,979865432359261
-0.147370283	0,88283975988959	0,979865432359261

-0.146855416	0,883246143214999	0,979865432359261
0.14836549	0,882054333471014	0,979865432359261
0.147553805	0,882694914124522	0,979865432359261
0.144273569	0,885284449083814	0,979904612483815
-0.143883137	0,885592751979902	0,979904612483815
-0.140301672	0,888421643562788	0,979904612483815
0.144080769	0,885436690544268	0,979904612483815
0.145655726	0,884193174302949	0,979904612483815
-0.14551993	0,884300381756359	0,979904612483815
-0.142379587	0,886780185444593	0,979904612483815
-0.141234065	0,887685035909119	0,979904612483815
-0.144481358	0,885120376186121	0,979904612483815
-0.142897913	0,886370806607328	0,979904612483815
-0.142514336	0,886673756527135	0,979904612483815
0.13999155	0,888666667056748	0,979904612483815
0.145056153	0,884666537224383	0,979904612483815
0.141345253	0,88759720188856	0,979904612483815
0.139900338	0,888738734017807	0,979904612483815
-0.14178342	0,887251080731191	0,979904612483815
0.141449398	0,887514932393586	0,979904612483815
-0.1398852	0,88875069504346	0,979904612483815
-0.139337828	0,889183198683817	0,980046416867844
0.138374316	0,889944594728392	0,98055050153709
-0.137043199	0,890996649010121	0,98062727687928
0.136747279	0,891230557684703	0,98062727687928
-0.137843344	0,890364228054508	0,98062727687928
-0.13691204	0,891100322076245	0,98062727687928
-0.135496801	0,892219093382256	0,981380141936485
-0.133383077	0,89389042820729	0,981812646766438
0.129682792	0,896817397615361	0,981812646766438
-0.130217993	0,896393959222849	0,981812646766438
-0.132420317	0,894651845167146	0,981812646766438
0.129462318	0,8969918397759	0,981812646766438
-0.129843111	0,896690554202813	0,981812646766438
0.13007069	0,896510499171336	0,981812646766438
0.134057928	0,89335676765945	0,981812646766438
-0.132910338	0,894264291016074	0,981812646766438
-0.129669052	0,896828269121754	0,981812646766438
-0.129225936	0,897178874425021	0,981812646766438
0.133362295	0,893906863487074	0,981812646766438
-0.132048083	0,894946260024619	0,981812646766438
0.134264725	0,89319324605481	0,981812646766438
-0.131437393	0,895429309787398	0,981812646766438
-0.126599684	0,899257247194028	0,982117389219205
-0.127955595	0,898184113974969	0,982117389219205
0.127552243	0,898503326898944	0,982117389219205
0.126266598	0,899520895828123	0,982117389219205
0.126956906	0,898974506381885	0,982117389219205
-0.126180464	0,899589075272412	0,982117389219205
0.126311191	0,89948559781906	0,982117389219205

-0.125590456	0,900056118211756	0,98229474830217
-0.125112016	0,900434870479879	0,982375662143982
0.121389987	0,903382147710366	0,984937014195608
0.12137579	0,903393391624995	0,984937014195608
0.119959784	0,90451500594626	0,985826594855252
-0.118796478	0,905436598882847	0,986497645742291
-0.109564112	0,912755073672987	0,986546189874773
-0.104332533	0,916905461016228	0,986546189874773
-0.100594632	0,919872258530794	0,986546189874773
0.112244325	0,910629684909431	0,986546189874773
0.114392031	0,908927030701404	0,986546189874773
0.106578172	0,915123636496508	0,986546189874773
-0.105112118	0,916286843857059	0,986546189874773
0.094683033	0,924566595468	0,986546189874773
-0.103507399	0,917560277845461	0,986546189874773
0.094759477	0,924505874646862	0,986546189874773
-0.108926655	0,913260664717236	0,986546189874773
-0.093723475	0,925328822122316	0,986546189874773
-0.113298793	0,909793674439077	0,986546189874773
-0.109847597	0,912530242266624	0,986546189874773
-0.100657145	0,919822632132629	0,986546189874773
-0.100773969	0,919729891493414	0,986546189874773
-0.109903195	0,912486148373603	0,986546189874773
-0.092870613	0,92600635264482	0,986546189874773
-0.097653901	0,922207122012596	0,986546189874773
-0.117046622	0,906823108608042	0,986546189874773
-0.104190402	0,917018250607396	0,986546189874773
-0.094493108	0,924717457303259	0,986546189874773
0.10069298	0,919794184474684	0,986546189874773
-0.111601732	0,911139198888366	0,986546189874773
0.110333449	0,912144930877613	0,986546189874773
-0.09358519	0,925438674678142	0,986546189874773
-0.108318001	0,913743443699102	0,986546189874773
-0.11406585	0,909185593132317	0,986546189874773
-0.11614835	0,90753497064052	0,986546189874773
-0.094163788	0,924979049803629	0,986546189874773
-0.108232796	0,913811030497561	0,986546189874773
-0.098343096	0,921659858333943	0,986546189874773
-0.09488736	0,924404296608664	0,986546189874773
0.102140165	0,918645420437604	0,986546189874773
0.117754584	0,906262116186298	0,986546189874773
0.112681634	0,910282962331308	0,986546189874773
-0.107571021	0,914335985812869	0,986546189874773
-0.105960788	0,915613463339176	0,986546189874773
-0.103404975	0,917641564191989	0,986546189874773
-0.09711566	0,922634544555713	0,986546189874773
-0.092522711	0,926282748198702	0,986546189874773
0.112231176	0,910640110530261	0,986546189874773
0.105938715	0,915630976726772	0,986546189874773
-0.095889479	0,923608350869422	0,986546189874773

-0.112792978	0,910194685928857	0,986546189874773
0.099061212	0,921089668946688	0,986546189874773
-0.093116952	0,925810650288437	0,986546189874773
0.116743615	0,907063226606522	0,986546189874773
0.105796832	0,915743550301689	0,986546189874773
-0.110698796	0,911855200500942	0,986546189874773
-0.109847045	0,912530680002302	0,986546189874773
0.099416753	0,920807381560868	0,986546189874773
0.094257451	0,924904648879986	0,986546189874773
-0.103938345	0,917218277112822	0,986546189874773
-0.105134885	0,916268778711227	0,986546189874773
-0.110470463	0,912036273502636	0,986546189874773
0.101847904	0,918877401717999	0,986546189874773
0.096448526	0,923164353472105	0,986546189874773
0.117949053	0,906108026079101	0,986546189874773
0.094145803	0,924993336435853	0,986546189874773
0.098626204	0,921435063793928	0,986546189874773
0.093319359	0,925649852692952	0,986546189874773
-0.101015934	0,919537811712497	0,986546189874773
-0.115874766	0,907751795080281	0,986546189874773
0.103971754	0,917191764458178	0,986546189874773
0.095226803	0,924134681013507	0,986546189874773
-0.114472928	0,908862905795195	0,986546189874773
-0.110784193	0,911787480437227	0,986546189874773
-0.091267424	0,927280101446015	0,987066958516887
0.090640467	0,927778275852372	0,987066958516887
-0.090664448	0,927759220996673	0,987066958516887
0.090366503	0,927995974642853	0,987066958516887
-0.08998018	0,928302964638279	0,98706793965
0.081975813	0,934665946948828	0,987154079980907
0.082468779	0,934273944110419	0,987154079980907
-0.080143469	0,936123150954483	0,987154079980907
0.081065405	0,935389937894628	0,987154079980907
0.08511179	0,932172517487204	0,987154079980907
-0.082701315	0,934089038989568	0,987154079980907
-0.084783479	0,932433528476931	0,987154079980907
-0.088018444	0,92986201653088	0,987154079980907
-0.082184411	0,934500070083687	0,987154079980907
-0.086449846	0,931108823769115	0,987154079980907
0.079345407	0,936757890920041	0,987154079980907
-0.087136409	0,930563084919688	0,987154079980907
-0.080330198	0,935974641654519	0,987154079980907
-0.079731594	0,936450731028644	0,987154079980907
0.087820154	0,930019618908661	0,987154079980907
0.083687443	0,9333049408876	0,987154079980907
0.080278877	0,936015457686927	0,987154079980907
0.084787359	0,932430443787095	0,987154079980907
-0.079098072	0,936954616688854	0,987154079980907
-0.083009933	0,933843641597306	0,987154079980907
-0.082693099	0,934095571996988	0,987154079980907

0.082485024	0,934261026865948	0,987154079980907
-0.079997874	0,936238947238082	0,987154079980907
0.079349432	0,936754689280187	0,987154079980907
-0.083314413	0,933601539984564	0,987154079980907
-0.085763322	0,931654564143382	0,987154079980907
0.08314844	0,93373350993756	0,987154079980907
0.089033737	0,929055098157636	0,987154079980907
0.060659922	0,951630050089055	0,987361820121015
0.076574959	0,938961679904393	0,987361820121015
-0.068324116	0,9455276268439	0,987361820121015
-0.05279879	0,957892225293511	0,987361820121015
0.057676846	0,954006036843197	0,987361820121015
-0.054194938	0,956779853492053	0,987361820121015
0.070823481	0,943538243386161	0,987361820121015
0.059001946	0,952950558522522	0,987361820121015
-0.056100128	0,955262042126721	0,987361820121015
-0.073898003	0,941091544609288	0,987361820121015
0.077257779	0,938418477672198	0,987361820121015
0.070682819	0,943650195428034	0,987361820121015
-0.070675726	0,943655840382881	0,987361820121015
-0.067623996	0,946084953430669	0,987361820121015
-0.066819891	0,94672508821711	0,987361820121015
-0.06289309	0,949851637659197	0,987361820121015
-0.049557403	0,960475092686006	0,987361820121015
0.063731899	0,949183704450089	0,987361820121015
0.054632639	0,956431135578719	0,987361820121015
-0.069324663	0,944731194536261	0,987361820121015
-0.073672879	0,941270679813153	0,987361820121015
0.049250914	0,960719336713105	0,987361820121015
0.060351039	0,951876053076327	0,987361820121015
0.048090034	0,961644488992282	0,987361820121015
-0.061797701	0,950723933939595	0,987361820121015
0.059829566	0,952291377094961	0,987361820121015
-0.051422457	0,958988890398331	0,987361820121015
-0.069258425	0,944783918411596	0,987361820121015
-0.069858878	0,944305983752259	0,987361820121015
0.051252887	0,959124009611205	0,987361820121015
0.064915084	0,948241610623383	0,987361820121015
-0.061808799	0,950715096246791	0,987361820121015
-0.048799676	0,961078939995952	0,987361820121015
0.056195097	0,955186386764035	0,987361820121015
-0.061939476	0,95061103073095	0,987361820121015
-0.067383118	0,946276708456124	0,987361820121015
-0.050479646	0,95974016861191	0,987361820121015
-0.070056323	0,944148830463484	0,987361820121015
-0.053201325	0,957571499847515	0,987361820121015
-0.068597252	0,945310206334051	0,987361820121015
0.051245429	0,959129952251971	0,987361820121015
-0.075121464	0,940118070386436	0,987361820121015
-0.04895655	0,960953922640986	0,987361820121015

-0.06684093	0,946708338901948	0,987361820121015
0.073649298	0,941289443111563	0,987361820121015
-0.064992895	0,948179657594577	0,987361820121015
-0.062406887	0,950238810516843	0,987361820121015
0.05769163	0,953994260709184	0,987361820121015
0.052797053	0,957893609209774	0,987361820121015
0.071922494	0,94266358883701	0,987361820121015
0.048170146	0,96158064294223	0,987361820121015
-0.069420603	0,944654829961747	0,987361820121015
0.062228879	0,950380565185882	0,987361820121015
-0.0655409	0,947743342696723	0,987361820121015
-0.053334619	0,957465297312837	0,987361820121015
-0.050825911	0,959464243933021	0,987361820121015
0.069888483	0,944282419581991	0,987361820121015
-0.060686566	0,951608830597977	0,987361820121015
0.048385517	0,9614090014506	0,987361820121015
0.070993756	0,943402725104358	0,987361820121015
0.056982992	0,954558743347285	0,987361820121015
-0.051005837	0,959320869677043	0,987361820121015
-0.068595224	0,945311820268332	0,987361820121015
0.05632322	0,955084321362107	0,987361820121015
-0.067599121	0,946104754958965	0,987361820121015
0.077807523	0,93798116201253	0,987361820121015
-0.070727916	0,943614302395484	0,987361820121015
-0.063882007	0,949064179267028	0,987361820121015
-0.0594289	0,9526104952063	0,987361820121015
0.053204116	0,957569275936445	0,987361820121015
0.068833736	0,945121964652459	0,987361820121015
0.053752744	0,957132160083217	0,987361820121015
-0.058824282	0,953092067476065	0,987361820121015
-0.069529849	0,9445678743231	0,987361820121015
0.053930078	0,956990872798507	0,987361820121015
-0.056421924	0,955005691741543	0,987361820121015
-0.066328824	0,947116035283576	0,987361820121015
0.067171656	0,946445049198817	0,987361820121015
0.050891273	0,959412159354084	0,987361820121015
0.054658163	0,956410800292098	0,987361820121015
-0.047489418	0,962123164365582	0,987538894041694
0.047064155	0,962462096001954	0,987572465671747
-0.042115698	0,966406466282125	0,988384205769095
0.041457837	0,966930905178758	0,988384205769095
-0.043867229	0,965010237797434	0,988384205769095
0.043184386	0,965554551796143	0,988384205769095
0.041713452	0,966727130000763	0,988384205769095
-0.041566831	0,966844015279477	0,988384205769095
-0.041741593	0,9667046966405	0,988384205769095
-0.041892847	0,966584118799202	0,988384205769095
0.042453203	0,966137416675638	0,988384205769095
0.045567001	0,963655371552417	0,988384205769095
0.045272903	0,963889785537847	0,988384205769095

0.042065463	0,966446512057333	0,988384205769095
0.037237679	0,970295495605757	0,988990136969422
-0.038252444	0,969486407151256	0,988990136969422
0.036935245	0,970536637144512	0,988990136969422
0.034574352	0,972419153464334	0,988990136969422
0.035217788	0,971906078125051	0,988990136969422
-0.039747057	0,968294784891923	0,988990136969422
-0.03744692	0,970128661924091	0,988990136969422
0.035252213	0,971878628450971	0,988990136969422
-0.034193875	0,972722550418651	0,988990136969422
0.035091154	0,972007054997793	0,988990136969422
-0.034883432	0,972172692268475	0,988990136969422
-0.035613043	0,971590907761722	0,988990136969422
-0.033406667	0,973350292942929	0,988990136969422
-0.037105038	0,970401254835504	0,988990136969422
-0.033723827	0,97309737886128	0,988990136969422
0.037505818	0,970081701187773	0,988990136969422
0.034109672	0,972789696114053	0,988990136969422
-0.034301306	0,97263688319516	0,988990136969422
-0.039086098	0,968821744780288	0,988990136969422
0.03291734	0,973740504597239	0,989075000732628
-0.031877204	0,974569978245754	0,989361121232605
0.031792292	0,974637693720207	0,989361121232605
-0.031410052	0,974942525047199	0,989361121232605
0.029373377	0,976566805880137	0,990633965177718
-0.029066986	0,97681116566361	0,990633965177718
-0.028206885	0,977497146031614	0,991018011930826
0.027165668	0,978327598864873	0,991236728350366
-0.027273548	0,978241554729569	0,991236728350366
-0.025820679	0,979400367676685	0,991389261066324
-0.026574874	0,978798813902712	0,991389261066324
-0.026113076	0,979167147843122	0,991389261066324
-0.025407329	0,979730065134824	0,991411816774336
0.024304237	0,980609933095486	0,991679847674175
-0.024481855	0,980468256884656	0,991679847674175
-0.021402829	0,982924316501306	0,993100846283391
0.021384567	0,982938884135375	0,993100846283391
0.021729528	0,98266370973962	0,993100846283391
-0.020893899	0,983330293068019	0,993185153505907
0.019351565	0,984560648541823	0,993955790746036
0.019164734	0,984709690367003	0,993955790746036
-0.015562549	0,987583383679757	0,993978539241065
0.015648913	0,987514483432922	0,993978539241065
0.017033927	0,986409549484636	0,993978539241065
-0.01558912	0,987562185947836	0,993978539241065
0.016554808	0,986791777443245	0,993978539241065
-0.016045724	0,987197913715715	0,993978539241065
0.015273065	0,987814331245772	0,993978539241065
-0.016448034	0,986876959637273	0,993978539241065
0.017600685	0,985957410064082	0,993978539241065

-0.015554438	0,987589854666382	0,993978539241065
0.013857175	0,988943928062938	0,994494595573113
-0.014014543	0,988818378353405	0,994494595573113
0.01346361	0,989257917638725	0,994500244509005
0.012789892	0,98979542048144	0,994730517623137
0.011740608	0,990632565661989	0,994951735988762
0.011865425	0,990532982476451	0,994951735988762
-0.011225432	0,991043589507701	0,995054662566106
0.010683543	0,991475928471946	0,99517891980144
-0.01014767	0,991903469672322	0,995298285529944
-0.008241775	0,993424089185995	0,996514055248782
0.007263906	0,9942042929045	0,99667666913802
0.007344284	0,994140161597292	0,99667666913802
0.004032995	0,996782144400808	0,998395676848084
-0.003950736	0,9968477765739	0,998395676848084
0.004153118	0,996686301052569	0,998395676848084
0.003475297	0,997227119946665	0,998465526801613
0.002641109	0,997892702143511	0,99866462139892
-0.002449862	0,998045294501928	0,99866462139892
0.000646248	0,999484368695365	0,999794382457367
-0.000147021	0,99988269460499	0,99988269460499
2.156880107	0,0310150072946225	NA
0.961641322	0,336229806789422	NA
0.55616939	0,578095062341115	NA
-1.164036129	0,244409370445977	NA
-0.772945624	0,439554562017047	NA
-1.718794242	0,0856518443954319	NA
0.104298444	0,916932512929446	NA
-1.171602544	0,241356666159038	NA
-0.040554636	0,96765094992468	NA
0.465139405	0,641831629102339	NA
0.928731834	0,353028078500766	NA
-0.143070442	0,886234548621118	NA
-0.639482637	0,522509005262764	NA
0.669012148	0,503487728672212	NA
2.380928417	0,0172690673570679	NA
1.72054202	0,0853339624323665	NA
-0.179773887	0,85733008475169	NA
1.370982939	0,170380274631987	NA
0.314942906	0,752805003482535	NA
1.367569675	0,171446814199518	NA
-0.925886921	0,35450474884182	NA
-0.626031273	0,531294429411534	NA
-1.397641068	0,162220879322653	NA
0.766127023	0,443600725368768	NA
0.174058618	0,861819394552181	NA
0.370031588	0,711358954475782	NA
-1.907155219	0,0565004904350787	NA
0.490916107	0,623485784723102	NA
-2.447195745	0,014397262388982	NA

-0.396045402	0,692071534592142	NA
1.208651338	0,22679682423192	NA
-1.906876661	NA	NA
-0.538090032	0,590514893918525	NA
-0.700883733	0,483375577195044	NA
1.024336689	0,305676274317879	NA
-1.924413623	0,0543027582366421	NA
-0.700267384	0,483760336785094	NA
0.434553183	0,663886783499591	NA
0.226425451	0,82087053490099	NA
-0.142083643	0,887013938391013	NA
-0.807685991	0,419271370388017	NA
-1.796091912	0,0724799004932608	NA
1.123596323	0,261184316815694	NA
-0.727740556	0,466772419249631	NA
1.547875001	0,121652394496608	NA
1.225753229	0,220291552590411	NA
-1.031652414	0,302234977202948	NA
-0.497859859	0,618582821518815	NA
-0.702545706	0,482338908588293	NA
-1.225004599	0,220573487756751	NA
-0.293672757	0,769007973234682	NA
0.124931894	0,900577468660605	NA
-0.375653385	0,707174597003314	NA
-1.679782009	0,0929997368524558	NA
-1.13090062	0,258096922221544	NA
-0.484541155	0,628001864352292	NA
-0.689841613	0,490293796278356	NA
-0.02999472	0,976071263911598	NA
-0.588892534	0,555933366550906	NA
1.298747722	0,194030520729081	NA
-0.555379128	0,578635366134383	NA
1.486592338	0,137122512666656	NA
1.485820138	0,137326702057208	NA
1.230015677	0,218691234238588	NA
1.915247253	0,0554609788568648	NA
0.800465463	0,423441165780069	NA
0.568316716	0,569819930543193	NA
0.016411927	0,98690576460769	NA
0.236041172	0,813400722249512	NA
-0.077725322	0,938046550634864	NA
-0.991254009	0,321561564065865	NA
0.558824492	0,576281505599321	NA
-0.644408571	0,519310527827183	NA
-0.554579956	0,579182002204377	NA
0.298101764	0,765625495015557	NA
-1.089955599	0,275732703126644	NA
0.728684084	0,466194931028464	NA
-1.293551536	0,195820371855959	NA
-1.394146546	0,16327335584796	NA

0.041511075	0,966888464005178	NA
-1.036043303	0,300181940829043	NA
-0.485494079	0,627325912050978	NA
-0.324625952	0,745464183113627	NA
-1.51179315	0,130586492521486	NA
-2.364690324	0,0180451527133782	NA
-0.150231728	0,880581794752608	NA
0.135028571	0,892589284486792	NA
1.42376862	0,154513462228421	NA
0.264121846	0,791686030372631	NA
0.212406244	0,831790114869675	NA
1.829186715	0,0673716419928051	NA
-1.98954001	0,0466416301974616	NA
-0.068820267	0,945132685808986	NA
-2.528347276	0,0114600933642722	NA
1.241507238	0,214418426006759	NA
1.103893392	0,269639385861539	NA
-1.850305981	0,0642694611554635	NA
-2.408878784	0,0160016116668868	NA
0.542565548	0,587428964996769	NA
-0.600222126	0,548358209550253	NA
0.482185522	0,629674160511496	NA
0.181409565	0,856046111621147	NA
1.387887564	0,165171285210829	NA
-0.007363156	0,994125104988452	NA
-1.025077049	0,30532683379336	NA
-0.60623266	0,544360268276142	NA
0.462805758	0,643503603871726	NA
-0.090061064	0,928238689851523	NA
0.488273074	0,625356430849879	NA
-0.294414003	0,768441567966785	NA
-1.111933487	0,266166749217664	NA
-0.824433034	0,40969355113866	NA
-0.303931838	0,761179821073663	NA
-0.847184612	0,396892229146758	NA
-0.74783811	0,454557811858703	NA
-1.632553703	0,10256289497896	NA
-0.671576954	0,501853053730034	NA
0.323393331	0,746397379356696	NA
-1.967070621	0,0491750697290159	NA
-0.662236071	0,507819940126752	NA
-1.076992934	0,281483422392647	NA
0.210716081	0,833108825763953	NA
0.6060262	0,544497355641694	NA
-0.334190268	0,738235987291512	NA
1.734406663	0,0828459537557513	NA
0.400337156	0,688908204348479	NA
0.213529845	0,830913712604631	NA
-3.657709147	0,000254479586386595	NA
1.367226681	0,171554264764756	NA

-0.488000481	0,625549500582276	NA
0.621422012	0,53432199301654	NA
1.087693275	0,276730537517252	NA
-0.829665976	0,40672766306778	NA
1.412800548	0,157714377211972	NA
0.879913255	0,378906303745121	NA
-0.941597171	0,346398918910965	NA
0.495954367	0,619926607215715	NA
-0.120813055	0,903839109284289	NA
0.144686132	0,884958689091009	NA
-0.944105239	0,345115872235767	NA
-1.080606011	0,279872407598843	NA
-0.745016588	0,456261691716941	NA
0.328808506	0,742300433465016	NA
0.025943923	0,979302066714758	NA
-0.734157911	0,462852502160667	NA
0.692326	0,488732625559817	NA
-0.328477989	0,742550283776857	NA
1.12120139	0,262202148122923	NA
-0.686459494	0,492423398588193	NA
-0.277062882	0,78173182207649	NA
0.097280743	0,922503448605319	NA
1.189585658	0,234209285469551	NA
-0.360948367	0,718138045722174	NA
1.409280289	0,158752304871362	NA
-0.563228963	0,573278983359158	NA
1.636404692	0,101754917952604	NA
-1.236812281	0,216156781738888	NA
-1.057527119	0,290271084808323	NA
0.540386127	0,588930773305623	NA
-1.086318326	0,277338182100069	NA
0.297416716	0,766148383001472	NA
0.109665493	0,912674667927714	NA
1.432242681	0,152074380679296	NA
-0.092173519	0,926560176885866	NA
-1.013098893	0,311012926762538	NA
0.649975983	0,515707735230394	NA
-1.869839058	0,0615061707082346	NA
1.581632607	0,113733465810322	NA
0.73106417	0,464739958019014	NA
0.31248501	0,754671953500603	NA
0.809395041	0,418287953576575	NA
0.029935165	0,976118761122698	NA
-1.169926582	0,242030515349359	NA
-0.256465202	0,797591647104949	NA
1.153076924	0,24887881246621	NA
0.577738251	0,563440850386531	NA
1.257984617	0,208397319517116	NA
-0.399539174	0,689495965294945	NA
0.700944954	0,483337368723674	NA

-2.023999062	0,0429702482978067	NA
0.74684077	0,455159682535826	NA
1.278873909	0,200941461843517	NA
2.400309737	0,0163812041846816	NA
-0.076747695	0,938824261203897	NA
0.702466302	0,48238840995265	NA
-0.834255976	0,404136754229845	NA
-0.353179588	0,723953809360567	NA
0.153034365	0,878371175504764	NA
-0.591513924	0,55417612869689	NA
-0.645261144	0,518757968510845	NA
-0.485611662	0,627242526548842	NA
1.539389234	0,123709300663918	NA
0.172490626	0,863051829961642	NA
1.346004167	0,178301172601855	NA
0.436595268	0,662404894807894	NA
0.917314835	0,358977685702576	NA
0.103861647	0,917279143472725	NA
-0.590203425	0,555054276217299	NA
-1.14824668	0,250866743780098	NA
0.228513941	0,819246719182714	NA
-0.615479423	0,538238154312891	NA
-1.497641012	0,134226545158253	NA
-2.388470933	0,0169186466205664	NA
0.24591432	0,805748558507228	NA
-0.981090174	0,326548275483349	NA
-2.39951336	0,0164168807517931	NA
-1.116385711	0,264257067007468	NA
-1.038004563	0,299267930656116	NA
1.1161297	0,264366620809818	NA
0.748335266	0,454257957646476	NA
0.718174731	0,47264955666755	NA
-0.088294419	0,929642674441076	NA
0.800607912	0,423358668664036	NA
0.583539788	0,559529980461514	NA
-0.758929499	0,447894731565985	NA
0.164341066	0,869462655059514	NA
1.297222631	NA	NA
-2.148046185	0,0317100866553524	NA
0.982545797	0,325831032825385	NA
-0.003733937	0,997020756093177	NA
-0.909703823	0,36297872732125	NA
-0.016052049	0,987192867553454	NA
1.832789459	0,066833887321474	NA
0.939457323	0,347495993787637	NA
0.481444913	0,630200322655693	NA
0.765241757	0,44412759912628	NA
1.970875468	0,0487381217948973	NA
-0.395649807	0,692363388348261	NA
0.374293618	0,708185880019654	NA

-1.087867123	0,276653771855534	NA
1.173341262	0,240658981680628	NA
-0.283326307	0,776926713223212	NA
-0.011105566	0,991139222614512	NA
0.449871343	0,652803212253154	NA
0.497053127	0,619151587570775	NA
-0.02376577	0,981039444033519	NA
1.314347236	0,18872937819088	NA
-0.32485677	0,745289476542373	NA
0.438308471	0,661162685857385	NA
-1.68829558	0,0913545033231533	NA
1.113245602	0,265602962238773	NA
1.381615837	0,167089688677256	NA
0.590793036	0,554659101945721	NA
1.788179907	0,0737469869322021	NA
0.965264746	0,334412224889261	NA
0.72578855	0,467968407282315	NA
1.940782987	0,0522846054672384	NA
1.904865942	0,0567974994684051	NA
-0.867111646	0,385880840322478	NA
2.363349766	0,0181105679513272	NA
0.061766102	0,950749098335824	NA
-0.465575865	0,641519122064919	NA
-0.211561406	0,832449221334953	NA
2.275775624	0,0228594423984811	NA
-0.191034563	0,848498514195578	NA
-0.40995385	0,681839801867469	NA
-0.550763648	0,581795707078984	NA
-1.053656866	0,292040043967871	NA
-0.497694828	0,618699153805359	NA
-0.559016302	0,57615059455614	NA
0.087060949	0,930623065953707	NA
1.701362526	0,088874933599116	NA
0.363745776	0,716047857396679	NA
1.584828519	0,113005300750965	NA
1.756783404	0,078954734917252	NA
0.133900242	0,893481458701306	NA
-0.320296804	0,748743345462865	NA
-0.739978379	0,459313113842148	NA
1.369126011	0,170959888781316	NA
-1.81362784	0,0697350412954701	NA
-0.064643052	0,948458206214746	NA
-0.647793157	0,51711874176637	NA
0.205066685	0,83752002480351	NA
-0.781922671	0,434260020662377	NA
0.966796143	0,333645953124098	NA
-0.04871543	0,961146078837892	NA
-0.68311368	0,494535011912482	NA
1.241173702	0,214541587546594	NA
-3.428096996	0,000607828247341698	NA

2.543969997	0,0109600498216201	NA
0.676771257	0,498551097697066	NA
-0.31991942	0,749029415538591	NA
-1.159201447	0,246374081963737	NA
-1.096947746	0,272664235209289	NA
0.399975233	0,689174758523658	NA
-1.080716731	0,279823138433297	NA
-1.020157717	0,307653667341627	NA
-0.414870541	0,678236676208686	NA
-1.219808537	0,222537464356453	NA
-0.727142309	0,467138783105818	NA
-0.122584558	0,902436083222384	NA
-1.674502856	0,0940318135784169	NA
-0.865008705	0,387034010686949	NA
-1.668241578	0,0952677801131718	NA
-0.623886103	0,532702387770103	NA
2.105196121	0,0352742397837105	NA
1.673532158	0,0942225814305459	NA
0.873633574	0,382317836171246	NA
-1.007294519	0,313793262746687	NA
-0.648858962	0,516429542266101	NA
-0.424143881	0,671460885135662	NA
-0.380694414	0,703430014536786	NA
-0.547336687	0,584147443895378	NA
-1.109608049	0,267167961246138	NA
-0.934347579	0,350124628999495	NA
0.215600374	0,829299264535624	NA
0.841288098	0,400186556641313	NA
1.200154673	0,230079275487247	NA
0.609949113	0,541895517140879	NA
-1.309418715	0,190392555359728	NA
-0.163395386	0,870207135653348	NA
-0.465038201	0,641904100368683	NA
-0.258470345	0,796043933057824	NA
-0.062200241	0,950403370207732	NA
0.722801156	0,469802056807938	NA
-1.911446095	0,0559472778168949	NA
0.593832067	0,552624439236969	NA
0.696523892	0,486100800322564	NA
-0.784789872	0,432576779304661	NA
0.867716202	0,385549713831553	NA
1.005412925	0,314698058758209	NA
1.589872955	0,111863444998006	NA
0.258881842	0,795726408841561	NA
1.087181374	0,276956660630225	NA
0.513587788	0,607540219198015	NA
2.047579123	0,0406012566628234	NA
1.372148456	0,170017227613002	NA
1.620618022	0,105099584710118	NA
1.72725187	0,0841224389612982	NA

1.515584109	0,12962454775142	NA
-0.282876709	0,777271349057716	NA
-1.983200244	0,0473450666934986	NA
-0.340726022	0,733309847196858	NA
0.296509869	0,766840731456317	NA
-0.488102633	0,625477145916584	NA
1.095179391	0,273438052765462	NA
-0.937379515	0,348563374184387	NA
1.111876374	0,266191308138781	NA
-0.56469232	0,572283058920084	NA
-0.354344369	0,723080820257867	NA
0.172994256	0,862655942499606	NA
0.080132056	0,936132227710989	NA
0.492486311	0,622375597008321	NA
-0.387147845	0,698646759076867	NA
-0.893985859	0,371329457007033	NA
1.527299057	0,126686663776216	NA
-0.305616234	0,759896858955422	NA
0.777376246	0,436936822651447	NA
-0.225371934	0,821689943126501	NA
0.492876507	0,622099847699894	NA
-1.388920307	0,164856984703676	NA
-0.651677787	0,514609055336158	NA
0.393346208	0,694063793913631	NA
0.306826826	0,758975188474941	NA
-0.563163015	0,57332388521691	NA
-1.27209632	0,203338898459696	NA
0.209938798	0,833715440470223	NA
-0.138443065	0,889890263410438	NA
0.94369236	0,345326879164603	NA
-1.135446144	0,25618843489832	NA
1.49414029	0,135138955144482	NA
0.443521402	0,65738864243985	NA
0.253980823	0,799510373523742	NA
-0.51471181	0,606754419699897	NA
-0.883114389	0,377174470421168	NA
2.096973244	0,0359959439348225	NA
-0.632210866	0,527249097896075	NA
1.775669062	0,0757874796063011	NA
-0.622699828	0,533481795638992	NA
0.459391559	0,645953008719626	NA
0.585482101	0,558223598719996	NA
-0.556244567	0,57804367613144	NA
0.125874668	0,899831135128904	NA
0.183028518	0,854775642612783	NA
0.028615403	0,977171327169217	NA
-0.025826784	0,979395498785527	NA
-1.239169919	0,215282577869036	NA
1.497059823	0,13437769255209	NA
-0.023365141	0,981359010974215	NA

1.967892321	0,0490804285700627	NA
0.227018218	0,820409575674509	NA
0.93881978	0,347823281492079	NA
1.472221235	0,140961144850927	NA
1.146454751	0,251607039520409	NA
1.607155384	0,108020286556259	NA
0.804824122	0,420921179572504	NA
-0.474429285	0,635193813720514	NA
-1.471692478	0,141103939752447	NA
1.391034191	0,16421505891805	NA
0.252820105	0,800407230481713	NA
-0.434240467	0,664113830106241	NA
-0.441135482	0,659114917770159	NA
2.117342728	0,0342307710871155	NA
-1.538880411	0,12383349421937	NA
1.211875322	0,225560110073807	NA
2.474021691	0,013360158495278	NA
-0.396628739	0,691641256217398	NA
-0.436790487	0,662263298391087	NA
-0.29418758	0,768614571011598	NA
1.412005307	0,157948398627808	NA
-0.25323371	0,800087618194162	NA
-0.387503683	0,698383358720357	NA
-0.975971249	0,32907871223369	NA
-0.300076845	0,764118540724757	NA
0.013700207	0,989069158504745	NA
0.703430225	0,4817876762137	NA
-0.1566362	0,87553157087528	NA
-0.478386343	0,632375252167815	NA
0.890368048	0,373268293482164	NA
-0.516134482	0,605760486082465	NA
-0.029025895	0,976843937818995	NA
0.123065733	0,902055046108493	NA
0.733080278	0,463509467912136	NA
0.307290237	0,758622466200497	NA
-0.524492369	0,599936126346514	NA
1.275630177	0,202086281300926	NA
1.628182347	0,103486226847997	NA
0.460405181	0,645225416492471	NA
1.790588086	0,0733594202870249	NA
-0.277890572	0,781096361730051	NA
-0.372100686	0,709817885540442	NA
-0.449988701	0,65271858780164	NA
0.446199463	0,655453170986592	NA
0.224199889	0,822601769526221	NA
-0.128723383	0,897576532263741	NA
0.091207998	0,927327320275134	NA
1.841569606	0,0655381302416016	NA
-1.524843632	0,127298104132063	NA
-0.1854728	0,852858212553999	NA

1.836021252	0,0663545136577683	NA
1.715145758	0,0863185067466146	NA
1.009758248	0,312611128150457	NA
-0.440180748	0,659806202516641	NA
0.189083874	0,850027080373929	NA
0.797900273	0,424928365246001	NA
1.094830022	0,273591110942017	NA
1.064677753	0,287021780950762	NA
-0.829156448	0,407015885407696	NA
-1.713898609	0,0865473484914618	NA
-0.62264549	0,533517510677935	NA
1.340250242	0,180164002290349	NA
-1.681420746	0,092681218593192	NA
0.268559053	0,788269025894489	NA
1.955694531	0,0505011493971639	NA
0.954609271	0,339775326835115	NA
0.242897548	0,808084765023665	NA
-0.266399471	0,7899315724227	NA
0.374116014	0,708318005266934	NA
-1.579627382	0,114192224632059	NA
-1.319352182	0,18705139996887	NA
0.232279247	0,816321130367142	NA
0.15315721	0,878274300739187	NA
1.295810753	0,195040692380606	NA
0.096318828	0,923267357746143	NA
0.136778143	0,891206160531045	NA
0.431530579	0,666082623370599	NA
0.239944899	0,810372973258904	NA
0.991756229	0,321316454618739	NA
-0.500141607	0,616975370918834	NA
0.948489259	0,342880438243337	NA
0.226109657	0,821116134203424	NA
-0.2187256	0,826863805560854	NA
0.257368941	0,796893976940363	NA
0.888156374	0,374456642008554	NA
-0.051404177	0,959003456634767	NA
-0.937831183	0,348331172969795	NA
0.438031696	0,661363307155539	NA
0.375242611	0,707480042482228	NA
0.332269855	0,739685500490419	NA
-1.198939446	0,230551492123084	NA
1.500709676	0,133430669188469	NA
1.108243135	0,267756826466437	NA
1.20834876	0,226913140248134	NA
-1.025767297	0,305001284497449	NA
1.104070944	0,269562364055735	NA
-0.96892091	0,33258465183353	NA
1.090154551	0,27564506969821	NA
-2.504006479	0,0122795785552528	NA
-0.278823486	0,780380289972494	NA

-0.16229744	0,871071630328385	NA
2.021259534	0,0432529057192267	NA
0.266641595	0,789745126528997	NA
0.004402802	0,996487083875531	NA
1.019241947	0,308088117108968	NA
-0.11483061	0,908579386260345	NA
1.660887402	0,0967360628765446	NA
2.07940562	0,037580084388956	NA
2.549152083	0,0107985197883542	NA
-0.880417561	0,378633146474041	NA
0.982949267	0,325632408629313	NA
-0.237295896	0,812427246018203	NA
1.211552447	0,225683746857488	NA
1.411858218	0,157991712275227	NA
1.182368534	0,23705950305861	NA
-2.065675405	0,0388591391240294	NA
-1.020036915	0,307710953438542	NA
-1.093106354	0,274347106625824	NA
0.225345472	0,821710527548635	NA
0.353597997	0,723640175652606	NA
-1.112287915	0,266014378172639	NA
0.821485016	0,411370055804804	NA
2.037467144	0,0416032512621089	NA
-0.204872565	0,837671690723696	NA
0.088225391	0,929697537109467	NA
-0.785240938	0,43231231710394	NA
0.514787472	0,606701540898063	NA
0.266765488	0,789649728486397	NA
0.678645281	0,497362644809791	NA
-0.219968148	0,825895961391149	NA
1.130807959	0,258135929366501	NA
-0.261881845	0,793412536972306	NA
1.491204835	0,135907724691063	NA
-0.346243113	0,729160016354625	NA
0.583396727	0,559626260906508	NA
1.318902749	0,187201626938296	NA
-1.623479179	0,104487010799541	NA
0.404163562	0,686092446592637	NA
-0.59739978	0,550240500917428	NA
0.251001438	0,801812997234075	NA
0.197353396	0,843550996801161	NA
0.165963143	0,868185955408438	NA
1.685346605	0,0919217189668246	NA
-0.172048688	0,863399252318551	NA
0.038447001	0,969331287637565	NA
-0.131672309	0,895243489197873	NA
-0.768616148	0,442121216516905	NA
-0.753892079	0,450914027130215	NA
0.487446167	0,625942183587834	NA
-0.775537196	0,438022295306359	NA

2.137340612	0,0325702979458869	NA
-0.327315639	0,743429165301332	NA
0.645191706	0,518802960270126	NA
-0.461710322	0,644289068106215	NA
0.746933395	0,455103766426611	NA
-1.87135075	0,0612964805687419	NA
0.742440383	0,457820560028068	NA
-1.231434123	0,218160541197228	NA
-0.655812537	0,51194474906672	NA
0.691397706	0,489315645284663	NA
0.641168988	0,521412896190525	NA
1.849266254	0,0644193760120507	NA
0.951211266	0,341497140773527	NA
-0.204187719	0,838206808208382	NA
0.943130192	0,345614314189918	NA
-0.175618582	0,860593602816986	NA
1.423846286	0,154490973637816	NA
-0.343676422	0,731089644880504	NA
0.749205401	0,453733414422768	NA
-0.505641452	0,613108376472989	NA
-1.306862943	0,191259263419148	NA
0.133019829	0,89417769847986	NA
0.669739602	0,503023801806684	NA
-0.674910724	0,499732486808583	NA
-0.188625857	0,850386065928925	NA
-0.266030487	0,790215729325186	NA
0.585851088	0,557975588669983	NA
0.321058581	0,748165997818249	NA
-0.435342176	0,663314075493132	NA
-0.7730842	0,439472551313688	NA
-0.238691125	0,8113450991214	NA
-0.562986237	0,573444255973068	NA
1.921450509	0,0546749360409157	NA
-1.24730444	0,212285889982231	NA
1.307268633	0,191121493341685	NA
-0.400551256	0,68875053898329	NA
-0.490735306	0,623613671791567	NA
-0.948779981	0,342732525607042	NA
0.239787102	0,810495306065746	NA
0.844279946	0,398512989606039	NA
-0.023303239	0,981408388400479	NA
1.009899525	0,312543430080707	NA
0.377069717	0,706121795592659	NA
-1.481261908	0,138536794912164	NA
-0.448807746	0,65357035019988	NA
-1.099077845	0,271734112699088	NA
-0.39270594	0,694536682804064	NA
0.157920385	0,87451953478145	NA
0.011452275	0,990862606375963	NA
0.766936957	0,443118999227558	NA

-1.614720403	0,106371257020675	NA
1.431561719	0,152269291823187	NA
0.456102612	0,648316192238241	NA
0.657354401	0,510953064119069	NA
0.605313982	0,544970392608408	NA
0.351909511	0,724906125780294	NA
0.855005403	0,392548156459786	NA
-0.267703203	0,788927784368869	NA
2.628595783	0,00857382064534272	NA
-0.488780289	0,624997253987245	NA
-0.460826023	0,644923429617822	NA
1.46659611	0,142485958756207	NA
-0.014184521	0,988682769395487	NA
-0.319219939	0,74955973812636	NA
1.163731006	0,244533040093373	NA
-0.150843339	0,880099297504634	NA
0.333204265	0,738980099217844	NA
-0.251792206	0,801201681908716	NA
-1.231222303	0,218239732003312	NA
-0.137821389	0,890381579831548	NA
-0.847930262	0,396476809931935	NA
-0.327164379	0,743543560796462	NA
-2.05046436	0,0403191382410778	NA
0.91270207	0,361399254979512	NA
0.417968806	0,67596992437247	NA
0.911992071	0,361772891673366	NA
-0.648823953	0,516452173409161	NA
-0.191744848	0,847942074189051	NA
0.424676203	0,671072733421836	NA
0.239366404	0,810821478532773	NA
1.128365405	0,25916563169598	NA
1.010837216	0,312094347830101	NA
1.168477545	0,242614191788326	NA
0.495062089	0,620556293568549	NA
-0.952201145	0,34099498033627	NA
0.455107999	0,649031543791889	NA
-0.365025277	0,71509253805573	NA
0.741911284	0,458141088534463	NA
0.179093262	0,857864472660807	NA
-0.04887762	0,961016823987377	NA
0.403782783	0,686372458151724	NA
0.385423993	0,699923309140852	NA
-0.364225564	0,715689578651928	NA
1.620961756	0,105025840819631	NA
0.064877898	0,948271218701479	NA
-0.499340209	0,617539733035272	NA
1.850542406	0,064235412018375	NA
2.303614729	0,0212442802908057	NA
0.858863901	0,39041560742515	NA
-0.471131959	0,637546503629802	NA

-0.643440987	0,519937994991248	NA
-1.001241659	0,316709990885711	NA
0.122928059	0,902164066110456	NA
0.623150968	0,533185319202853	NA
-0.708430414	0,478678011015293	NA
0.663660139	0,506907857363087	NA
0.071991516	0,942608659383348	NA
-0.426451633	0,669778784580367	NA
0.288371582	0,773062318362396	NA
-0.185301194	0,852992801507051	NA
-0.592166535	0,553739076319274	NA
-1.474936934	0,140229499475102	NA
-1.100187298	0,271250523669794	NA
0.290050837	0,771777345852922	NA
-0.136793959	0,891193658437272	NA
-0.034121484	0,972780276458267	NA
1.057761399	0,290164235276966	NA
0.710950441	0,477114947165803	NA
0.321867587	0,747553009346658	NA
0.43179635	0,665889432520987	NA
-0.34444356	0,730512734709455	NA
0.953495822	0,340338912024251	NA
-0.775209832	0,438215679378682	NA
1.079592628	0,28032362560935	NA
1.758287983	0,0786985213917669	NA
-0.587576057	0,556816885677627	NA
0.176986106	0,859519303157545	NA
-2.042500617	0,0411018982966989	NA
1.249935339	0,21132316888982	NA
1.102587366	0,270206399233926	NA
-0.159342251	0,873399234622259	NA
-1.201164415	0,229687429939404	NA
0.830022598	0,406526007212201	NA
1.644229573	0,10012879048363	NA
0.320580716	0,748528153031856	NA
0.78853051	0,430386459699534	NA
-0.816997284	0,41392998016877	NA
0.220711336	0,825317204174771	NA
1.529556725	0,126126487971359	NA
0.081833812	0,934778868261489	NA
0.997880841	0,31833714339384	NA
-0.34877894	0,727255275425552	NA
0.021422393	0,982908710781302	NA
-0.777126969	0,437083863804599	NA
-0.492041957	0,622689683031455	NA
1.800909003	0,0717172237896355	NA
1.727245723	0,084123542429612	NA
0.261145842	0,793980040703797	NA
0.144400953	0,885183863949398	NA
-0.475673049	0,634307324339828	NA

-0.108330925	0,913733192440246	NA
0.505265959	0,613372048479462	NA
0.15375814	0,877800441029346	NA
-1.198455688	0,230739664173166	NA
1.009966075	0,312511543815438	NA
-2.054358374	0,039941018802775	NA
1.228066488	0,219422009777781	NA
0.292859072	0,769629873842455	NA
-1.387237099	0,165369476263343	NA
0.24339251	0,807701345288205	NA
1.496679319	0,134476720115965	NA
0.582892586	0,559965612033363	NA
0.772596664	0,43976111947237	NA
-2.272709018	0,0230437208638648	NA
0.342409423	0,732042797969901	NA
0.312040416	0,755009808016116	NA
0.532237935	0,594561212169225	NA
-0.029215459	0,976692752293765	NA
0.384674166	0,700478837549471	NA
0.600982577	0,547851590828483	NA
0.134186068	0,893255442243871	NA
0.348294744	0,727618840655934	NA
0.855668394	0,392181227006042	NA
1.227865419	0,219497492825816	NA
-0.372532095	0,709496720469498	NA
-0.883237304	0,377108070251703	NA
-0.697362047	0,485576246819957	NA
0.261910306	0,793390593954481	NA
-0.178310212	0,858479359460576	NA
-0.951464454	0,341368654530078	NA
0.447926116	0,654206519733871	NA
-1.009770802	0,31260511183474	NA
1.115543927	0,264617405719661	NA
-1.968324914	0,0490306651371431	NA
0.8793185	0,379228607794443	NA
1.091870005	0,274890243355812	NA
0.037847	0,969809670944125	NA
0.600727502	0,548021498039655	NA
1.673092062	0,0943091739557298	NA
-1.745952974	0,0808191236055242	NA
-1.691653035	0,090712147986264	NA
-0.711483057	0,476784946208673	NA
0.293699116	0,768987829645441	NA
0.004152757	0,996686589015301	NA
-0.936089021	0,349227355072742	NA
0.873051293	0,382635120233116	NA
1.192001202	0,233260773776683	NA
-0.69776088	0,485326748065523	NA
-1.5728577	0,115751771547421	NA
-0.137427542	0,89069286232625	NA

0.833558635	0,404529743324996	NA
-0.451463651	0,651655417076124	NA
-0.295867252	0,767331461170011	NA
0.401003533	0,688417522524111	NA
0.536131002	0,591868015341939	NA
1.087348448	0,276882845133623	NA
-0.325916297	0,744487684833097	NA
-0.128652925	0,897632286310059	NA
0.196378584	0,844313857504865	NA
0.079511355	0,936625900613495	NA
0.982287486	0,325958237562728	NA
0.117499267	0,906464425160061	NA
-0.936316815	0,349110093089339	NA
-0.328157578	0,742792521543716	NA
1.155359676	0,247943167680539	NA
-0.131831939	0,895117223414872	NA
1.28755596	0,197900577993196	NA
1.47979994	0,138926644647403	NA
-1.394406132	0,163194997556087	NA
-0.823217336	0,410384412190233	NA
-0.319639375	0,749241721823114	NA
-1.628881895	0,103338023610706	NA
-1.159102192	0,246414532558746	NA
-0.752000335	0,452050857533861	NA
-0.8611068	0,389179224610695	NA
0.272342662	0,785358555726333	NA
-1.612435192	0,10686727189352	NA
0.429201411	0,667776656280244	NA
1.70348604	0,0884771482223732	NA
-0.121891039	0,902985312872777	NA
-0.236836436	0,812783684088206	NA
-0.831254076	0,405830112214321	NA
1.433158157	0,151812644600808	NA
1.497687368	0,134214495394869	NA
0.310794144	0,75595712142397	NA
0.603216179	0,546364886007479	NA
0.912030571	0,361752624506254	NA
0.206166759	0,836660652224321	NA
-0.266580946	0,789791827594641	NA
-0.033355498	0,973391097456837	NA
0.077928212	0,937885157568298	NA
-0.567033815	0,570691207473781	NA
1.693032149	0,0904493479522149	NA
-0.522201	0,601530389841084	NA
0.421794149	0,673175276633909	NA
0.163712444	0,8699575205076	NA
0.048985037	0,96093122025439	NA
0.313704919	0,753745164176084	NA
1.862896341	0,0624768517586528	NA
1.180040098	0,2379842671908	NA

-0.19205848	0,847696397611194	NA
-0.225410723	0,821659770533604	NA
1.551157765	0,120863880841579	NA
-1.177261785	0,239091034978873	NA
0.423658892	0,671814598962654	NA
0.506721113	0,612350514102135	NA
-0.381711076	0,702675683788488	NA
0.284617529	0,77593718002208	NA
-0.115050657	0,908404969860912	NA
1.615316171	0,106242243446989	NA
-0.00121301	0,999032157993876	NA
-0.313210033	0,754121096545972	NA
1.320206898	0,186765949424711	NA
0.429407923	0,667626389065656	NA
0.400867333	0,688517802145712	NA
-0.358990222	0,719602404224038	NA
0.922689162	0,356169215512438	NA
0.207399885	0,835697572140193	NA
0.789402892	0,42987656620989	NA
-0.483962993	0,628412133022696	NA
-1.561807605	0,118333319730732	NA
-0.854990662	0,392556317577883	NA
1.811959973	0,0699923815118291	NA
-0.175331942	0,860818814031141	NA
-0.012570058	0,989970809244554	NA
1.59943158	0,109724740003762	NA
0.693649709	0,487901911699985	NA
-0.685880257	0,492788621087008	NA
0.715581375	0,474249877661613	NA
0.334733526	0,737826108796973	NA
1.31363992	0,188967408038515	NA
0.740222214	0,459165170523663	NA
-0.780870809	0,434878482115141	NA
0.417501655	0,676311513969243	NA
2.007038252	0,0447455901303421	NA
0.653940033	0,513150441005203	NA
-0.20900294	0,834445943733809	NA
0.267122656	0,789374724163995	NA
2.043971364	0,040956376164166	NA
0.557385238	0,577264249387069	NA
0.633997915	0,526082182669053	NA
-0.839548138	0,401161787860009	NA
-1.374475336	0,16929416199747	NA
-1.634929586	0,102063809465237	NA
1.60568167	0,108343870699642	NA
0.845774533	0,397678535046993	NA
-0.897945445	0,369214634726787	NA
1.772984946	0,076231201417807	NA
-1.352421072	0,17624065214855	NA
1.408034013	0,159120998668609	NA

0.240650636	0,809825901052446	NA
-0.18300717	0,854792392827314	NA
-0.210876659	0,832983518077361	NA
-0.099242036	0,920946099734519	NA
-0.160331643	0,872619833907617	NA
-0.638339442	0,523252742104441	NA
-0.762898935	0,445523671514572	NA
-1.367676997	0,171413203259266	NA
-0.110277863	0,912189012883545	NA
0.84992537	0,395366579568041	NA
-1.095753462	0,273186679579432	NA
-0.991756423	0,321316359711299	NA
0.213937723	0,830595622775365	NA
-0.377991851	0,705436647644128	NA
-0.295420192	0,76767291064912	NA
1.059982201	0,289152697092446	NA
-1.127536214	0,259515837959188	NA
-0.405330403	0,685234660978532	NA
-1.145411034	0,252038929770933	NA
1.521431751	0,128151526001365	NA
-0.697146918	0,485710854413598	NA
-1.990534761	0,0465320587093242	NA
0.951525525	0,341337667592902	NA
-1.253180781	0,210139922815984	NA
-1.326865769	0,184553089691824	NA
2.697984852	0,00697606148593727	NA
-0.319309252	0,749492017724724	NA
0.555861188	0,578305752551986	NA
0.581351877	0,561003323627212	NA
-0.332239316	0,739708558028731	NA
1.052960694	0,292359006834587	NA
-1.94327989	0,0519823498825954	NA
-0.784031621	0,433021556014021	NA
0.534286647	0,593143229166671	NA
-0.327001836	0,743666496379675	NA
1.30308076	0,192547191530854	NA
0.20600518	0,83678686493678	NA
1.00176508	0,316457066122068	NA
0.906842131	0,364490286955803	NA
2.09708891	0,0359857054983334	NA
1.063735899	0,287448357140443	NA
1.360502268	0,173671035664908	NA
1.108416941	0,267681792053099	NA
2.446421535	0,0144282199090727	NA
0.304090313	0,761059086976055	NA
0.212510234	0,831708994802823	NA
-1.513513562	0,130149258832791	NA
1.064613704	0,287050775597967	NA
-0.896514822	0,369977867434914	NA
-0.154738739	0,877027288576021	NA

0.014419358	0,988495415719389	NA
0.771502628	0,440409065069653	NA
0.318146363	0,75037391678146	NA
1.362269778	0,173112761833157	NA
0.121380171	0,903389921773897	NA
1.843420931	0,0652675764449917	NA
1.358999474	0,174146755491323	NA
-0.812273799	0,416634541535303	NA
-0.85611769	0,391932684737337	NA
0.042748915	0,965901686675953	NA
0.702281086	0,482503886277687	NA
0.886510313	0,375342599362674	NA
-1.074282311	0,282696170494641	NA
-0.424638846	0,671099970522658	NA
1.980577578	0,0476386654781793	NA
0.447621898	0,654426096817797	NA
-0.022344588	0,982173081814282	NA
0.422299788	0,672806212801132	NA
0.21391214	0,830615573061645	NA
1.539101618	0,123779490113077	NA
0.643806196	0,519701115482075	NA
1.041320369	0,297726886415861	NA
1.531935865	0,125538262114643	NA
-0.535912746	0,592018854959038	NA
-1.125701865	0,260291733939635	NA
-1.201551504	0,229537340238275	NA
-0.418418986	0,675640807258984	NA
1.065391164	0,28669895372686	NA
0.136941752	0,891076836098412	NA
-0.460566329	0,645109773628201	NA
-0.03378867	0,973045671095666	NA
-1.193584172	0,232640668515792	NA
0.705603346	0,480434843101233	NA
-1.859455066	0,0629626626931993	NA
1.423411521	0,154616894732951	NA
-1.616969886	0,105884781490102	NA
0.745437071	0,456007540133343	NA
1.936932344	0,0527536141105075	NA
0.423357032	0,672034789536393	NA
0.490734608	0,623614165426184	NA
-0.23455419	0,814554769058755	NA
-0.793331893	0,427584479709426	NA
-1.963566997	0,0495803278252679	NA
-1.063393833	0,287603388239012	NA
0.464984002	0,641942913326792	NA
1.025293843	0,305224560126054	NA
-0.79654327	0,425716335777397	NA
-0.749247337	0,453708142711314	NA
-0.127717954	0,898372181125243	NA
0.214769174	0,829947287554313	NA

-1.377460022	0,16837006658606	NA
1.550435781	0,121036956101284	NA
-0.532982043	0,59404601100557	NA
0.939843789	0,347297693583322	NA
-0.023858629	0,980965374514369	NA
0.959551092	0,337281193944533	NA
1.042355764	0,297246767684296	NA
1.108404302	0,267687247699721	NA
-0.765413801	0,444025177220003	NA
-0.967308156	0,33339000770822	NA
-0.556355017	0,577968183350275	NA
0.767577679	0,442738127546657	NA
-1.032624706	0,301779561573027	NA
-0.29221129	0,770125080376207	NA
-1.684185006	0,0921459200983885	NA
1.956426901	0,0504148858201247	NA
0.785059073	0,43241893402669	NA
0.635206767	0,52529357029451	NA
-0.302188271	0,762508544728777	NA
-1.567885315	0,116907899444387	NA
-0.975542456	0,329291252489546	NA
0.711819616	0,476576483885508	NA
1.836634155	0,0662639216618504	NA
0.640485792	0,521856823015256	NA
1.231948941	0,217968157958999	NA
-1.178282644	0,238683944846076	NA
0.886950802	0,375105388875404	NA
0.700183229	0,483812884006252	NA
0.88585317	0,375696654208349	NA
-0.420335206	0,674240594496913	NA
-0.451152069	0,651879951837092	NA
-0.518781704	0,603912977819935	NA
-0.61958349	0,535532039160813	NA
-0.506425264	0,612558142716836	NA
-0.223070451	0,823480675730183	NA
-0.417304832	0,676455454286411	NA
-0.381549054	0,702795879108208	NA
1.355921796	0,175124051920209	NA
0.240187929	0,810184571053269	NA
0.362868635	0,716703018081516	NA
1.224908567	0,22060967203456	NA
0.647669192	0,517198934472772	NA
0.156156685	0,875909518171588	NA
-0.509958588	0,610080474848078	NA
0.636220141	0,524632947360352	NA
1.153235406	0,248813774844079	NA
-1.069567207	0,284814162609157	NA
0.712124315	0,47638779882443	NA
0.367625	0,713152870633631	NA
1.635589411	0,101925548070795	NA

1.204170706	0,228523605998463	NA
1.168866421	0,242457454149401	NA
-0.42896991	0,667945122684365	NA
0.510069629	0,610002681406913	NA
-0.975360491	0,329381474468882	NA
2.741020471	0,00612486914600794	NA
0.405306296	0,685252379414702	NA
1.497732564	0,13420274779156	NA
0.373454868	0,708809929930444	NA
-0.96017714	0,336966070007623	NA
2.481313954	0,0130899023252896	NA
-0.439101181	0,660588223087847	NA
-0.675936517	0,499080951400125	NA
-0.033267534	0,973461243192727	NA
-1.08991209	0,275751870204316	NA
-0.265831756	0,790368785044742	NA
1.673714167	0,094186788218827	NA
-0.062262047	0,950354152084355	NA
1.185065505	0,235991548391092	NA
1.773426587	0,0761580466655514	NA
0.325649135	0,744689832530497	NA
-0.437457976	0,661779246212158	NA
0.11985515	0,904597893449653	NA
0.908887719	0,363409396967122	NA
-0.513035077	0,607926784405474	NA
-0.373105459	0,709069956217194	NA
-0.05310973	0,957644479355065	NA
0.634193625	0,525954467414303	NA
-0.25535254	0,798450823307748	NA
-1.588301647	0,112218145235931	NA
-0.79495688	0,426638585254595	NA
-0.653985311	0,51312126928870 2	NA
0.916077645	0,359626171900935	NA
0.859607376	0,390005507540842	NA
1.166277133	0,243502415663661	NA
0.289498606	0,772199846799679	NA
0.092786421	0,926073238561612	NA
1.102173892	0,270386079863786	NA
-0.496220318	0,619738977285517	NA
-0.645978562	0,518293239456316	NA
0.221267542	0,824884121683641	NA
0.059219234	0,952777490183663	NA
0.765122508	0,444198598349218	NA
-1.172554552	0,240974483746612	NA
-0.536751806	0,59143906595891	NA
-0.954984581	0,339585494522885	NA
0.864766887	0,38716674915722	NA
0.202827154	0,839270133199442	NA
3.755034491	0,000173317609160483	NA
-0.125531231	0,900103001428674	NA

0.30935303	0,757052995746162	NA
0.631717034	0,527571795256947	NA
-0.589886788	0,555266552202013	NA
1.111858211	0,266199118811466	NA
-0.211899776	0,832185225210887	NA
1.299337605	0,193828094253187	NA
-1.253060883	0,21018355048554	NA
-1.584174791	0,113153948191497	NA
-0.962017027	0,336041050828586	NA
-1.093553521	0,27415084333576	NA
-0.45406435	0,649782511784098	NA
0.780628277	0,435021155908747	NA
0.439613574	0,660217007919479	NA
0.360431875	0,718524193718927	NA
-1.802621672	0,071447651055224	NA
-0.804219666	0,421270122914004	NA
0.180289939	0,856924953643412	NA
1.41514722	0,15702533579608	NA
-0.077824938	0,937967308686475	NA
0.859820252	0,389888133822243	NA
0.44078195	0,65937086176194	NA
-0.111495486	0,911223444897194	NA
-2.702617745	0,00687958132571543	NA
-0.993252166	0,320587082185542	NA
0.841569589	0,400028917448532	NA
1.121846686	0,261927632195578	NA
0.020765604	0,983432636071181	NA
-1.016534525	0,309374915322094	NA
0.731654616	0,464379403646102	NA
0.508511481	0,611094689537073	NA
-0.678422031	0,497504144539275	NA
1.611247412	0,107125807690271	NA
1.505577082	0,132175775366893	NA
1.359178119	0,174090153300873	NA
0.86735241	0,385748948884649	NA
-0.104509516	0,916765017208384	NA
-0.387922847	0,698073129737659	NA
-0.174962036	0,861109464090363	NA
0.811425783	0,41712119738024	NA
1.379657406	0,167692153352816	NA
-0.510027799	0,610031986730329	NA
1.377772613	0,168273504242837	NA
0.503267705	0,614776068936231	NA
1.06139337	0,288511168567192	NA
-0.389817949	0,696671170050979	NA
1.095595317	0,273255912058137	NA
-0.001752186	0,998601958413595	NA
-0.145997083	0,88392369149292	NA
0.195583536	0,844936147555894	NA
-0.428632683	0,66819055701918	NA

-1.295368256	0,195193223399029	NA
-0.956406955	0,338866672088277	NA
0.266010529	0,790231100009162	NA
0.724831155	0,468555620080323	NA
-0.280735707	0,778913119045924	NA
-0.071075964	0,943337297603556	NA
-0.027523425	0,978042256159274	NA
2.235337544	0,025395197061476	NA
-0.614487812	0,538893024902779	NA
0.945509163	0,344398994987259	NA
-0.221436361	0,824752682927086	NA
0.463111251	0,643284626024682	NA
-0.080030234	0,936213209766413	NA
-0.273434239	0,784519435982864	NA
0.318732227	0,749929574533193	NA
1.192426521	0,233094046309514	NA
0.374582746	0,707970807129853	NA
-1.073847069	0,282891229837622	NA
0.082333925	0,934381177911923	NA
-0.533934966	0,593386529589607	NA
1.327789041	0,18424780817475	NA
-0.134214137	0,893233247505131	NA
0.561877875	0,574199228715838	NA
2.66554945	0,00768626266962237	NA
0.303114162	0,761802861647057	NA
-0.599034009	0,549150205931328	NA
1.330572141	0,183329834337851	NA
0.761799416	0,446179728771068	NA
1.429713504	0,152799262938498	NA
0.828179629	0,407568778458919	NA
1.330210949	0,183448777598793	NA
-1.582141326	0,113617311328034	NA
0.558663649	0,576391292082225	NA
-0.916125243	0,359601209528982	NA
0.317314607	0,751004895177768	NA
-0.927958098	0,353429305745961	NA
0.182465404	0,855217501964678	NA
-0.121915531	0,902965915210738	NA
1.821336621	0,0685557029571289	NA
0.177816292	0,858867252467143	NA
0.226452028	0,820849866037576	NA
0.220115744	0,825781013538433	NA
-2.328313719	0,0198954513361854	NA
1.462306829	0,143657148055747	NA
1.256874782	0,20879898132631	NA
-2.160887282	0,0307040475757803	NA
0.156546324	0,875602408312367	NA
1.079043519	0,280568327842275	NA
0.149870369	0,880866889360991	NA
-0.540976425	0,588523832014423	NA

-0.320652601	0,74847367080534	NA
-0.074502371	0,940610654809934	NA
-0.181860057	0,855692551678391	NA
-0.587466424	0,556890494171647	NA
-1.109582086	0,267179154187845	NA
2.709280813	0,00674292422207046	NA
1.812785857	0,0698648560679695	NA
0.372119718	0,709803715696901	NA
0.1199869	0,904493524904122	NA
0.277117722	0,781689714086248	NA
-0.134518224	0,892992801069636	NA
0.241180715	0,8094150567652	NA
-0.266069447	0,790185724562972	NA
-1.575317793	0,115183109037388	NA
-1.11199802	0,266139001516955	NA
0.665975658	0,505426659459909	NA
-1.50518977	0,13227529445297	NA
-0.14149898	0,887475765994632	NA
1.568329545	0,116804244132134	NA
1.391401535	0,164103699563505	NA
1.059571607	0,289339536213392	NA
-1.050375279	0,293545606881628	NA
0.885526199	0,375872896018763	NA
-2.155098482	0,0311541285068199	NA
0.308969964	0,757344374648027	NA
-3.090873111	0,00199568897391529	NA
-0.762853004	0,44555106617982	NA
-1.71387785	0,0865511617537639	NA
-0.232285045	0,816316627269895	NA
-0.403745787	0,686399666321194	NA
0.029706331	0,976301263018986	NA
1.894489834	0,0581600224593456	NA
-0.518700452	0,603969646097916	NA
-0.543546949	0,586753274941493	NA
-1.515654638	0,129606703547911	NA
-1.322886836	0,185873013653658	NA
0.407869376	0,68336957656485	NA
-1.431346225	0,152331012074797	NA
0.596943191	0,550545309849369	NA
-0.650831486	0,515155272785292	NA
0.608911687	0,542582977150781	NA
0.524499549	0,599931133520896	NA
1.938684261	0,0525397963136356	NA
1.063280997	0,287654540400984	NA
0.643884196	0,519650530641464	NA
-0.98399139	0,32511974642784	NA
-0.872531971	0,382918234355426	NA
-0.055273431	0,955920628523214	NA
0.755409051	0,45000358398221	NA
-0.50167229	0,615898059292174	NA

-0.655137537	0,512379206231255	NA
-0.486337729	0,626727733365864	NA
-0.326969756	0,743690760514412	NA
-0.653828599	0,513222238775334	NA
-0.028529413	0,977239908994222	NA
0.055801662	0,955499810543647	NA
1.342626326	0,179393001517219	NA
-0.729998383	0,46539117329995	NA
-0.724914858	0,468504265193583	NA
-0.833315601	0,404666760085022	NA
1.444911317	0,148482796971034	NA
-0.901419006	0,367365579580922	NA
0.241735653	0,808985001606673	NA
0.600212514	0,548364614873741	NA
-0.892492263	0,372129138152004	NA
0.250635969	0,802095570239205	NA
-0.57181306	0,567448628528457	NA
-0.400244532	0,688976417563458	NA
3.47683529	0,000507369402778081	NA
0.705681948	0,480385949295536	NA
1.008592685	0,313170016873275	NA
-1.214868532	0,224416237337373	NA
2.457768234	0,0139803364517574	NA
-2.391797648	0,016766083150211	NA
0.252475911	0,800673231348808	NA
-1.125258843	0,260479364491297	NA
-0.203974061	0,838373768828524	NA
0.412538391	0,679944847937995	NA
0.210852761	0,833002166594553	NA
-1.710181179	0,0872323750100295	NA
0.164120731	0,869636102037443	NA
0.041298639	0,967057817668978	NA
1.209335627	0,226533928708516	NA
0.18842902	0,850540352576698	NA
-0.21957888	0,826199141230904	NA
0.820476949	0,411944264499317	NA
-0.273325276	0,784603187135017	NA
0.931020654	0,351842880608663	NA
0.193199793	0,846802501085595	NA
0.375897302	0,706993246599398	NA
1.120223032	0,262618731410422	NA
-1.519470413	0,128644129912563	NA
0.946509577	0,343888739760427	NA
0.1318129	0,895132283024235	NA
-1.253555961	0,210003446813185	NA
1.25615344	0,209060343916056	NA
0.16509149	0,868871970986598	NA
1.468391141	0,141998006492802	NA
-0.489685958	0,624356140040396	NA
1.324128909	0,185460236031162	NA

0.213494053	0,830941627368637	NA
-0.120698761	0,903929640227894	NA
1.435130914	0,151249795622426	NA
1.236036514	0,216444991329041	NA
1.985700577	0,0470665809038297	NA
-0.057205824	0,954381238802488	NA
0.386059955	0,699452266441961	NA
1.239820557	0,215041772801314	NA
-0.72712889	0,467147002322857	NA
2.290035095	0,0220192822733449	NA
0.221675801	0,824566269340834	NA
0.26315778	0,792428971114473	NA
-1.593478712	0,111052840263115	NA
-1.019906179	0,307772958662567	NA
0.641433941	0,521240786887355	NA
-0.308966593	0,75734693892646	NA
-0.207007982	0,836003624948982	NA
0.672486301	0,501274157972906	NA
-1.175898487	0,239635443620274	NA
-1.257175447	0,208690111909682	NA
0.293899479	0,768834716385709	NA
0.613954445	0,539245430784907	NA
0.111910658	0,910894245492229	NA
-0.019883569	0,984136252634614	NA
0.72335133	0,469464064613356	NA
0.028617356	0,977169769360723	NA
0.604847117	0,545280583860853	NA
1.423205112	0,154676704082883	NA
0.280315407	0,77923553111692	NA
0.22116911	0,824960759991332	NA
0.03615491	0,971158839085452	NA
0.845643799	0,397751483988916	NA
-0.740573373	0,458952157786881	NA
0.941462229	0,34646803688406	NA
-2.10925381	0,0349226794155514	NA
-0.629946435	0,528729630300407	NA
0.48132413	0,630286150116951	NA
-0.242699668	0,808238064307396	NA
2.322450438	0,0202086941932664	NA
0.021756896	0,982641877948449	NA
2.135877027	0,0326894385650452	NA
-1.689714247	0,0910826361915026	NA
1.25663967	0,208884142967236	NA
1.274596235	0,202452190693281	NA
0.317242259	0,751059786605433	NA
0.471954081	0,636959565176426	NA
1.694853936	0,0901031327047084	NA
-0.29133227	0,770797209058456	NA
2.298898925	0,0215106794486456	NA
0.800447909	0,423451332635431	NA

-1.291804666	0,19642479920196	NA
-0.142125911	0,886980551594493	NA
-0.4099014	0,681878278671514	NA
0.136043784	0,891786668328761	NA
-0.084039989	0,93302463781123	NA
-0.959355958	0,337379454280832	NA
1.033863423	0,301200016659397	NA
0.141959929	0,887111657195617	NA
0.728951597	0,466031271060102	NA
1.277656057	0,201370725847517	NA
-0.75446861	0,450567887376003	NA
-0.206412717	0,836468538082805	NA
-0.981432668	0,326379422817458	NA
1.42006418	0,155588997239353	NA
2.350648173	0,0187407444174539	NA
-1.473231046	0,140688746323841	NA
1.545052488	0,122333567910285	NA
0.848899327	0,395937313248637	NA
0.307899746	0,758158619298503	NA
-0.409694211	0,68203027744454	NA
-0.294506147	0,768371167052	NA
-0.126730442	0,899153750755229	NA
1.397170637	0,162362264294192	NA
0.764515138	0,444560318800746	NA
-0.786786086	0,431407100157338	NA
1.64308194	0,10036598103925	NA
1.021699675	0,306923064796397	NA
-1.211208313	0,225815577395791	NA
-0.602002642	0,547172378899045	NA
0.565735019	0,571573925854872	NA
0.544161599	0,586330274845946	NA
-0.000560866	0,999552493481108	NA
-0.703420895	0,481793488611753	NA
-0.120407832	0,90416008774479	NA
0.113211835	0,909862613192533	NA
-0.979666671	0,327250682906848	NA
0.392578093	0,69463112272095	NA
-1.442159291	0,149257441812364	NA
-0.202884279	0,839225482118791	NA
1.055537416	0,291179606403991	NA
-0.134480812	0,893022383221714	NA
-0.161372015	0,87180040634871	NA
-1.473413311	0,140639623219019	NA
0.781312209	0,434618891022347	NA
0.684208351	0,493843609661819	NA
0.908316979	0,363710774497185	NA
-0.799484165	0,424009724897273	NA
0.081200444	0,935282546034911	NA
0.910047394	0,362797515821887	NA
-1.185240723	0,23592228296546	NA

2.641109096	0,00826351053957357	NA
-0.131951221	0,895022874074167	NA
0.604049663	0,545810624575739	NA
-0.485188404	0,627542706640321	NA
0.323634776	0,746214556577277	NA
1.154662532	0,248228648700591	NA
0.476934923	0,633408463055681	NA
0.814000631	0,415644589508537	NA
0.774872227	0,43841516502356 9	NA
0.526575841	0,598488172217496	NA
0.290056772	0,771772805447403	NA
0.073892662	0,941095794843875	NA
0.353177836	0,723955122832438	NA
0.033698715	0,973117403721775	NA
-1.641088036	0,100779141783946	NA
0.931382643	0,351655665987857	NA
0.624408497	0,532359346268886	NA
0.230897604	0,817394352395051	NA
-0.15875966	0,873858232034907	NA
1.190308989	0,233924969145058	NA
-0.212990355	0,831334485854677	NA
0.671579765	0,501851263554243	NA
0.651722408	0,514580264299331	NA
-1.918553426	0,0550408746226085	NA
-1.269838204	0,204142268929733	NA
0.070645354	0,943680013361017	NA
-0.352470872	0,724485159393377	NA
0.641564895	0,521155732754566	NA
-0.209825569	0,833803815941643	NA
0.112075495	0,910763547399525	NA
0.894152848	0,371240116548162	NA
-0.620886023	0,534674618157647	NA
-1.664668031	0,095979004500817	NA
2.815426548	0,00487125309601438	NA
0.502568135	0,615267937277899	NA
1.6652225	0,0958683736960834	NA
0.968934911	0,332577665570779	NA
-0.536713536	0,591465504637591	NA
0.408254303	0,683086984902719	NA
1.028454785	0,303735949381397	NA
0.085004975	0,932257435782832	NA
0.6133034	0,53967574543662	NA
-0.184515952	0,853608714457507	NA
-1.438370865	0,150328854725992	NA
-0.919313931	0,357931393437549	NA
2.158383775	0,0308980061783502	NA
0.871757892	0,383340470715253	NA
0.042438682	0,966148992072409	NA
1.813798335	0,0697087789558806	NA
-1.108138791	0,267801880473782	NA

0.985575957	0,324341241260163	NA
-0.050233735	0,95993612862179	NA
1.872255296	0,0611712922614386	NA
0.311277133	0,755589949260051	NA
0.247391156	0,804605517869187	NA
0.957932788	0,338096651795592	NA
-0.493969735	0,62132755410095	NA
0.063375758	0,949467290883799	NA
-0.131244165	0,895582159099122	NA
-0.07644785	0,939062802251175	NA
0.525929094	0,598937473460605	NA
1.380392347	0,167465875280665	NA
-0.977554671	0,328294625516841	NA
0.042893295	0,965786593437384	NA
1.693312134	0,0903960696562907	NA
-0.100048547	0,920305783502825	NA
0.73590063	0,461791174103423	NA
0.33390961	0,738447768010648	NA
0.617427438	0,53695282848267	NA
0.618849835	0,536015288573133	NA
-1.441684897	0,14939128605531	NA
2.008220639	0,0446198507290212	NA
0.183259729	0,854594231176056	NA
0.509243886	0,610581285133082	NA
-0.687028	0,49206508405038	NA
0.246214768	0,805515983835475	NA
-2.045053492	0,0408495846823204	NA
0.068404015	0,945464025564145	NA
-0.510625335	0,609613432477892	NA
0.164844671	0,86906624298546	NA
-0.074127496	0,940908936493767	NA
-0.732581202	0,463813899476634	NA
0.598914416	0,549229957375577	NA
-2.07608119	0,0378864425660273	NA
0.532336557	0,594492916909217	NA
2.114204814	0,0344977779082666	NA
-1.868396948	0,0617067621081654	NA
2.13911138	0,0324266489739373	NA
0.373887138	0,70848828603894	NA
1.724242876	0,0846640068685534	NA
2.475743703	0,0132958985959919	NA
-1.345975349	0,178310466763546	NA
0.57386589	0,566058556942359	NA
-0.722244881	0,470143934343725	NA
-0.336345409	0,736610411231783	NA
-1.023582557	0,306032487750058	NA
2.421489218	0,015457059565747	NA
0.287620458	0,773637283202226	NA
-0.92333625	0,355832002396917	NA
-1.014893976	0,31015637023431	NA

-0.441419255	0,658909504981246	NA
-0.146105745	0,883837911378644	NA
1.845466592	0,0649696938684479	NA
-1.184680435	0,236143820931797	NA
0.12486971	0,900626698766602	NA
1.806254155	0,0708786493277827	NA
0.041768726	0,96668306667242	NA
-1.145924322	0,251826466739237	NA
0.222151978	0,824195575768419	NA
1.123135363	0,261380009360783	NA
-0.148849468	0,881672415372585	NA
1.043147408	0,296880026555481	NA
-1.267372245	0,205022219366112	NA
-0.386663241	0,699005533198529	NA
-0.948147918	0,343054156936077	NA
0.197119387	0,843734112705168	NA
0.730786301	0,464909692100524	NA
0.213307289	0,83108728885108	NA
-0.324489588	0,745567403427274	NA
2.271031021	0,0231451001235236	NA
0.57221761	0,567174558866759	NA
-0.947592471	0,343336960607592	NA
0.05617899	0,955199218274046	NA
1.067225201	0,285870155109326	NA
-1.266379628	0,205377201002182	NA
-0.755524126	0,449934562045382	NA
-0.80645254	0,419981963153093	NA
-1.431768866	0,152209980128138	NA
1.163980161	0,244432051832914	NA
1.466748848	0,142444389223249	NA
-0.272471965	0,785259144840376	NA
0.179127873	0,857837296675947	NA
-0.8159577	0,414524329290336	NA
0.021953694	0,982484893224264	NA
0.137008424	0,891024136685409	NA
2.839928076	0,00451237065770805	NA
-0.926984538	0,35393456218115	NA
2.068510951	0,0385920018838488	NA
-0.162550982	0,870871984264153	NA
-1.193489556	0,232677699824301	NA
-1.928170629	0,0538339062349824	NA
0.820219682	0,412090883593086	NA
0.509529756	0,61038094648685	NA
-0.146227568	0,8837417436458	NA
1.611656239	0,107036765366418	NA
-0.185756942	0,852635372378193	NA
-0.444344922	0,656793227453734	NA
-1.659627502	0,0969894121317718	NA
0.896426695	0,370024915452525	NA
0.81800956	0,413351728433543	NA

-1.905593378	0,056702981954692	NA
0.056334374	0,955075435577393	NA
-0.118107154	0,905982755028784	NA
0.81651147	0,414207666189339	NA
1.158595749	0,246621002400267	NA
1.00956372	0,312704358682135	NA
1.265630095	0,205645546412576	NA
-1.06404791	0,2873069964203	NA
0.684101686	0,493910957037782	NA
1.143256165	0,252932249033011	NA
-2.405927465	0,0161314672773679	NA
-0.840381232	0,400694668484517	NA
1.103237374	0,269924094978601	NA
0.881594137	0,377996326926428	NA
0.556865794	0,577619126859876	NA
-0.749588868	0,453502357558383	NA
0.397084553	0,691305110761555	NA
0.845105003	0,398052215293058	NA
0.784640206	0,432664549886363	NA
0.635730879	0,524951846018424	NA
-0.207952937	0,835265714455462	NA
-2.070166641	0,0384367420190172	NA
-0.557211496	0,577382936030841	NA
0.420448782	0,674157637738757	NA
1.823400743	0,0682427179124959	NA
-0.677665674	0,497983695555388	NA
-0.306197716	0,759454111262512	NA
0.228209704	0,819483218075678	NA
0.452352859	0,65101480174968	NA
-0.169653617	0,865282553150673	NA
-0.676739095	0,498571507163037	NA
-0.361989396	0,71735995463155	NA
1.132036528	0,257619077601647	NA
-1.072535092	0,283479760701287	NA
0.315383002	0,752470871408012	NA
-0.471150031	0,637533599060907	NA
-0.328868502	0,742255083321102	NA
-0.243974611	0,807250483331964	NA
0.05668058	0,954799643714963	NA
-0.489228345	0,624680044262724	NA
0.14246212	0,886714997667134	NA
1.538090981	0,124026371003925	NA
-0.879721962	0,379009948770338	NA
-0.176502403	0,859899261306309	NA
-0.76237238	0,445837786152653	NA
-0.639029128	0,522803983040506	NA
-1.114462437	0,265080850549705	NA
1.215429553	0,224202302190051	NA
-0.700844238	0,483400227080135	NA
-0.128757175	0,897549792666351	NA

0.210913645	0,832954656187648	NA
0.030933775	0,975322354345077	NA
0.388749236	0,697461656627881	NA
0.387439372	0,698430960615247	NA
0.309475319	0,756959983518207	NA
1.742235517	0,0814672414496855	NA
0.028574662	0,977203820252661	NA
-0.272448753	0,7852769905813	NA
0.166353093	0,867879085715351	NA
0.23956857	0,810664733037707	NA
-0.050484212	0,959736530486342	NA
-0.632436305	0,527101817272313	NA
2.282032858	0,0224874004004165	NA
-0.27877907	0,780414377809443	NA
0.666668978	0,504983598478037	NA
-0.793596376	0,427430443146552	NA
1.161439098	0,245463376586039	NA
1.402147901	0,160871085441085	NA
0.021099229	0,983166499798756	NA
0.225928999	0,821256643187674	NA
0.935987419	0,349279665304256	NA
1.948922529	0,0513046771774155	NA
0.700372588	0,483694650439417	NA
-0.148844593	0,881676262714136	NA
0.427975457	0,668668989076101	NA
-0.237486807	0,812279152874783	NA
0.825700789	0,408973844366442	NA
-0.782147836	0,434127696441083	NA
1.350776901	0,176766909487186	NA
0.004164335	0,996677350752462	NA
1.004552335	0,315112458786654	NA
0.180006802	0,857147228193793	NA
-2.106118271	0,0351940801296963	NA
0.215271024	0,829556019293131	NA
1.178140291	0,238740681750796	NA
0.010483874	0,991635232129083	NA
1.537227385	0,124237636383034	NA
-1.380466154	0,167443163899914	NA
-1.15726108	0,247165709440986	NA
-1.57784525	0,114601165516019	NA
0.815705064	0,414668841836275	NA
1.660927295	0,0967280495669718	NA
0.381042815	0,70317147926269	NA
0.448403885	0,653861738822502	NA
2.087315491	0,0368596254400252	NA
-0.166389929	0,867850099295711	NA
1.001410504	0,316628388018501	NA
0.843059267	0,399195298368316	NA
1.046973921	0,295111606288496	NA
0.77908788	0,435927949236302	NA

-0.935601042	0,349478637949955	NA
-0.581935386	0,560610204175434	NA
1.340122651	0,180205473074422	NA
-0.862970653	0,388153602311228	NA
0.527684427	0,597718383512211	NA
1.094290311	0,273827673450973	NA
-0.741408678	0,458445684261708	NA
1.336709331	0,181317535076084	NA
0.314614487	0,753054378612213	NA
0.490927956	0,623477403914233	NA
0.10064399	0,919833075105738	NA
1.434571122	0,151409348474847	NA
1.021478491	0,307027794363683	NA
1.946089542	0,0516439846077984	NA
1.887137529	0,0591418301537972	NA
-0.703799229	0,481557814023461	NA
-0.029049695	0,976824956574976	NA
0.414342391	0,678623372922143	NA
0.137896689	0,890322067411058	NA
1.574430922	0,115387859713013	NA
1.115233756	0,264750264079463	NA
-0.163153567	0,87039752438553	NA
0.66751163	0,504445383343231	NA
-1.229488027	0,218888884348622	NA
-0.867153699	0,38585780110747	NA
1.400748813	0,161289200356572	NA
0.572652867	0,566879756711693	NA
-0.052079882	0,958465043189331	NA
-0.663343077	0,507110853289903	NA
-0.930960788	0,351873848312362	NA
1.406909604	0,159454195307442	NA
-0.252557837	0,800609914976679	NA
-0.968726454	0,332681690131682	NA
0.673424612	0,500677195066344	NA
-1.546256635	0,122042600604433	NA
1.00858031	0,313175954128927	NA
0.555991279	0,5782168167483	NA
0.415009475	0,678134967052714	NA
1.739379475	0,0819680360535793	NA
-0.524383288	0,600011977890081	NA
-0.311564114	0,755371810541431	NA
-0.824887052	0,409435718193914	NA
0.11659103	0,907184146208851	NA
1.312214499	0,189447771113249	NA
0.691795717	0,489065626483347	NA
0.194484959	0,845796171794289	NA
-1.789605166	0,0735174074455524	NA
-0.075461203	0,939847765176161	NA
-0.892992825	0,371861014778694	NA
0.332188398	0,739747004163456	NA

0.936991288	0,348763039379831	NA
2.820916669	0,00478866403633114	NA
1.181985889	0,237211299868393	NA
-0.442389449	0,658207412852177	NA
-0.628607464	0,529606072833987	NA
-0.772228844	0,4399789008681	NA
-0.180576159	0,856700270256759	NA
-0.182470435	0,855213554446999	NA
-0.074485029	0,940624453312755	NA
0.022587293	0,981979480163765	NA
0.54329019	0,586930017572127	NA
-1.076695319	0,281616404081429	NA
-1.330125257	0,183477005196967	NA
-0.307145935	0,758732295429253	NA
0.138597503	0,889768216445666	NA
0.524025367	0,600260896332975	NA
0.991123161	0,321625445033951	NA
-0.070337894	0,943924722097059	NA
1.969491969	0,0488966238870489	NA
-0.270289725	0,786937369551382	NA
0.452005013	0,651265371331041	NA
-0.444810545	0,656456673189556	NA
-0.427007624	0,66937377440906	NA
2.00211435	0,0452724344852002	NA
-1.129462026	0,258702980093563	NA
-0.00528311	0,995784707613665	NA
0.692822705	0,488420821907822	NA
1.265098391	0,205836059493994	NA
-0.299868588	0,764277395534952	NA
-0.287765954	0,773525900459376	NA
0.456075412	0,648335750705825	NA
0.864738077	0,387182565195754	NA
-0.735525224	0,462019683557616	NA
1.832586614	0,0668640702979548	NA
-0.4132332	0,679435766075965	NA
-0.213192378	0,831176912940645	NA
-0.58657243	0,55749090359625	NA
0.327168019	0,743540808367704	NA
-0.723602417	0,469309857409387	NA
0.559338112	0,575930989490171	NA
-1.444711825	0,148538847126315	NA
-0.047151831	0,962392218077985	NA
0.967455348	0,333316452964174	NA
2.383501858	0,017148797905831	NA
0.148241352	0,882152298536574	NA
-0.548114489	0,583613292171691	NA
-0.20638787	0,836487945498362	NA
1.074967122	0,28238944847264	NA
0.203596321	0,838668968189004	NA
1.395043574	0,163002699958558	NA

-0.444472911	0,656700710028619	NA
-1.266721983	0,205254716886689	NA
-1.300239283	0,193518970824906	NA
0.766273509	0,443513577171524	NA
1.140213299	0,254197448240581	NA
1.394158761	0,163269667921074	NA
1.930079923	0,053596935655321	NA
1.305583855	0,191694113805863	NA
1.670177543	0,0948842417156654	NA
1.185695277	0,235742660284375	NA
0.06364712	0,949251211621867	NA
-1.583287557	0,113355937714871	NA
0.456577108	0,647975036060363	NA
-1.03441401	0,30094265745327	NA
1.062667844	0,287932608097368	NA
0.236673665	0,81290996718547	NA
2.535827413	0,0112182006207722	NA
0.19190927	0,847813276249535	NA
-0.009345681	0,992543333900073	NA
-0.337506812	0,73573487778216	NA
0.175836302	0,860422548910783	NA
-0.033294145	0,973440022551384	NA
0.561968508	0,574137475469961	NA
-1.831617329	0,0670084531463551	NA
0.663091653	0,507271856172575	NA
0.864894039	0,387096949884511	NA
1.13803299	0,255106707118565	NA
1.903663593	0,0569540108834573	NA
0.461958213	0,644111287055222	NA
1.205842379	0,227878271513732	NA
1.591994149	0,111386019227342	NA
-2.256019993	0,0240693790422335	NA
0.746971301	0,455080884363051	NA
0.210860078	0,832996456829648	NA
0.687960018	0,491477959893288	NA
1.011693556	0,311684597540288	NA
-0.077881078	0,93792265123159	NA
0.354907589	0,722658822773599	NA
-0.254677976	0,798971828336426	NA
-0.357923272	0,720400734332875	NA
0.005953915	0,995249491519464	NA
1.522808506	0,127806621212539	NA
-1.095146452	0,273452480936071	NA
1.919454171	0,0549268809137443	NA
-0.975385771	0,329368938898028	NA
1.260911901	0,207340589789044	NA
-1.042259508	0,297291380057767	NA
0.283882032	0,7765007868538	NA
-0.124887311	0,900612763815212	NA
-3.245706829	0,00117159424279426	NA

1.62851611	0,103415496005157	NA
0.762503572	0,445759511846241	NA
0.076680125	0,938878016138974	NA
0.783629653	0,433257451107154	NA
2.523329307	0,0116249481895721	NA
-0.650289693	0,515505113725068	NA
-0.10590045	0,915661336803782	NA
-0.096631149	0,923019318544915	NA
0.674184019	0,500194328581482	NA
0.6947134	0,487234928458889	NA
-2.490862557	0,0127433405536647	NA
-0.290123086	0,771722075204112	NA
1.301314112	0,193150958745779	NA
-1.267220293	0,205076531501418	NA
1.701500724	0,0888490020066799	NA
1.835093488	0,0664918386008763	NA
-0.557239414	0,577363864017299	NA
2.377595405	0,0174259344158388	NA
0.145622556	0,884219360935723	NA
0.151928918	0,879242999978783	NA
-0.314346865	0,753257607492297	NA
-1.284152084	0,199088747029212	NA
1.365343548	0,172145097925197	NA
2.013681239	0,0440430109363317	NA
0.629087563	0,529291732296589	NA
-1.016463332	0,309408800203509	NA
0.419572025	0,674798128208907	NA
-0.886776929	0,375199011364871	NA
0.027422712	0,97812258388367	NA
0.379169069	0,704562319120034	NA
1.09718148	0,272562067318732	NA
-1.498732822	0,133942958220318	NA
-0.50221797	0,615514204059134	NA
1.091192596	0,275188145039112	NA
0.240420755	0,810004089664883	NA
0.769168198	0,441793466849503	NA
-0.182893439	0,85488163076495	NA
0.487036441	0,626232506999856	NA
0.962352191	0,335872720298467	NA
1.639835023	0,101039473963222	NA
-1.889912242	0,0587696980574253	NA
-0.158319928	0,874204706122565	NA
0.232027073	0,816516986696256	NA
-0.331494211	0,740271212706622	NA
0.418558001	0,675539189170807	NA
-1.643853873	0,100206390152212	NA
-0.483918643	0,628443608600621	NA
-0.463586142	0,642944284612232	NA
1.123014946	0,261431146813247	NA
0.409039466	0,68251069887756	NA

1.786173679	0,0740711414208862	NA
-0.660549017	0,508901573905485	NA
-0.253219827	0,800098345613009	NA
-0.131176192	0,895635929383537	NA
-0.085284984	0,932034829342163	NA
-1.3425594	0,179414684361076	NA
-1.276708738	0,201705095375093	NA
1.648994099	0,0991488436649736	NA
0.424107781	0,671487211117041	NA
1.401196532	0,161155311132433	NA
-1.351264455	0,176610734045823	NA
-1.665625125	0,0957881036689653	NA
-1.314080002	0,188819283078387	NA
-0.670337494	0,502642670474395	NA
-0.063168498	0,949632329292045	NA
1.080932172	0,279727286206721	NA
-0.539487799	0,589550313626033	NA
0.931893185	0,351391729732077	NA
-1.397665903	0,162213417981483	NA
0.45855983	0,646550288428729	NA
0.621455752	0,53429979953995	NA
1.186813088	0,235301355125361	NA
-0.908922029	0,363391284521367	NA
-0.120236256	0,904295997642391	NA
0.869059623	0,384814520166818	NA
0.554334716	0,57934979564649	NA
-0.942259804	0,346059642194614	NA
0.077852571	0,937945327401881	NA
-2.080652227	0,0374657497365804	NA
1.945564608	0,0517070618372089	NA
0.318661545	0,749983178496626	NA
-0.464853628	0,642036280150426	NA
1.409981115	0,158545259343189	NA
0.935653461	0,349451639580322	NA
0.772312405	0,439929419952198	NA
0.053188709	0,957581551939146	NA
0.387009219	0,698749382728731	NA
0.060454527	0,95179363211829	NA
-1.591000932	0,111609366096378	NA
0.211442647	0,83254188120976	NA
-1.345667137	0,178409889127036	NA
0.486307915	0,62674886835475	NA
1.149478431	0,250358755584434	NA
0.060648776	0,951638927730124	NA
0.500128217	0,616984799081003	NA
-1.61002526	0,107392341854159	NA
0.742081075	0,458038215566037	NA
1.379989181	0,167589975999096	NA
0.148761901	0,881741514941431	NA
-0.225073176	0,821922347826334	NA

-1.723848132	0,0847352628292431	NA
-0.027482396	0,978074980388563	NA
-0.594426992	0,552226559285923	NA
-0.174027832	0,861843589461095	NA
0.34651146	0,728958373099278	NA
-0.870772432	0,383878420776291	NA
1.573336353	0,115640956042062	NA
-0.522699737	0,601183221479352	NA
-0.189206036	0,849931337359047	NA
2.00723905	0,0447242154513029	NA
-0.216197425	0,828833860686335	NA
-1.420110672	0,155575463748255	NA
-0.457859434	0,647053432853665	NA
0.346053391	0,729302590030047	NA
-0.958687508	0,337716193372303	NA
0.367833507	0,712997383139354	NA
0.677776227	0,497913586472113	NA
1.166897343	0,243251828963562	NA
-0.193628954	0,84646642493454	NA
-1.344597357	0,178755297371442	NA
-0.300024844	0,764158204997874	NA
-0.655129152	0,512384604563762	NA
-1.646583635	0,0996436564290807	NA
-2.010768471	0,0443499178450738	NA
-0.81311666	0,41615117542121	NA
0.183022767	0,854780155062184	NA
-0.102526121	0,918339080228457	NA
1.22788631	0,219489649213676	NA
2.441154842	0,0146403759402235	NA
0.417225212	0,67651368577507	NA
-0.309830306	0,756690004074227	NA
-0.117173198	0,90672280556695	NA
-0.516989774	0,6051632972893	NA
0.029833908	0,976199515795027	NA
-0.36301248	0,716595561950462	NA
-2.549570469	0,0107855711263696	NA
1.034168011	0,301057626023455	NA
1.333054209	0,182514014813279	NA
-0.957408905	0,338360905399153	NA
1.009281523	0,312839638949695	NA
1.117215591	0,263902156016727	NA
0.490743122	0,623608143110196	NA
0.054402628	0,956614385217136	NA
0.774051654	0,438900245666296	NA
-0.009456895	0,992454602241948	NA
1.334362468	0,182085094153566	NA
0.844103118	0,398611785401998	NA
-1.183844976	0,236474434700481	NA
-0.354983257	0,722602134723122	NA
-0.221324036	0,824840136010657	NA

2.678417951	0,00739708526354117	NA
0.485990934	0,626973594166745	NA
1.760350525	0,0783483924323035	NA
-1.152974269	0,248920945850463	NA
0.099647455	0,920624217341932	NA
-0.163760264	0,86991987387561	NA
3.11674269	0,0018286109993115	NA
0.100416369	0,92001377496722	NA
0.129822575	0,896706801587581	NA
-0.009449087	0,99246083148252	NA
-0.357689815	0,720575455907725	NA
-0.574726592	0,565476220378555	NA
-0.14586339	0,884029232730149	NA
0.150694732	0,880216529303292	NA
-0.979835186	0,327167480779685	NA
-0.411904388	0,680409504159763	NA
0.199079073	0,842200891275014	NA
1.193480417	0,232681277139685	NA
-0.39626323	0,69191084971373	NA
-0.990888439	0,321740058165136	NA
-0.884090531	0,376647344384445	NA
-0.242984122	0,808017698038372	NA
-0.483098774	0,629025604431478	NA
-0.560658509	0,575030356839772	NA
1.858707946	0,0630685465360818	NA
-0.024824048	0,980195309567808	NA
2.860829549	0,00422534156201787	NA
-0.366403752	0,714063820184494	NA
0.189655838	0,849578828621206	NA
-0.879217226	0,379283506242355	NA
0.63008164	0,52864117106001	NA
-0.36805348	0,712833357423691	NA
-0.025469769	0,979680261483945	NA
0.976125243	0,329002403526047	NA
-0.408238456	0,683098617860971	NA
-0.417384547	0,676397155701524	NA
0.777605037	0,436801890414743	NA
0.138221359	0,890065475114954	NA
1.196192941	0,231621274875441	NA
1.083107009	0,278760928216339	NA
0.096427707	0,923180887361493	NA
-0.040289792	0,967862091794492	NA
-0.723257985	0,469521400151723	NA
-2.193728673	0,0282549212537999	NA
0.161587937	0,871630357342804	NA
2.050533807	0,0403123682918383	NA
-0.541003631	0,588505079966745	NA
0.700636796	0,483529709068448	NA
0.860380628	0,389579260820767	NA
-0.650860475	0,515136557591502	NA

-0.563851655	0,572855093877947	NA
1.918751418	0,0550158008386459	NA
-0.163547137	0,870087662633544	NA
1.190630633	0,233798620414165	NA
2.385080439	0,0170753872754927	NA
0.155873784	0,87613250973853	NA
0.780346759	0,435186797124209	NA
-1.62320447	0,104545702689204	NA
0.425352145	0,670579985889146	NA
-0.936047514	0,349248724498657	NA
0.805199556	0,420704532628259	NA
-1.377565621	0,1683374415371	NA
-0.727551699	0,466888057097922	NA
-0.060219405	0,951980890643111	NA
1.125919167	0,26019973540749	NA
0.572368512	0,56707234329183	NA
-0.162887492	0,870607019588796	NA
1.940060751	0,0523723072334391	NA
2.500549912	0,0124000658501614	NA
2.134093363	0,0328351390378456	NA
2.15929226	0,0308275002801551	NA
0.439744489	0,66012217676161	NA
-0.559292543	0,575962083714898	NA
-1.245515007	0,212942502489505	NA
-0.201116451	0,840607519005612	NA
-1.075260472	0,282258128214988	NA
1.378094576	0,168174090101496	NA
0.77670212	0,437334535903952	NA
-1.294089311	0,195634573295507	NA
0.637233688	0,523972637185439	NA
1.040491771	0,29811148504318	NA
1.813077206	0,0698199142816348	NA
0.714481401	0,474929552975847	NA
0.241452553	0,809204385531714	NA
-0.373571961	0,708722797599495	NA
-0.636458366	0,524477709118852	NA
0.909941934	0,362853133329351	NA
0.21294524	0,831369674955984	NA
-1.150320665	0,250011822717163	NA
-0.519957382	0,603093279689846	NA
-0.565301787	0,571868513518252	NA
0.525901505	0,598956642895295	NA
-1.609805794	0,107440259953197	NA
-0.332421783	0,739570792192358	NA
-1.266860201	0,20520528178727	NA
0.735868646	0,461810640298063	NA
2.229650266	0,0257706700478926	NA
1.452354018	0,146403194681292	NA
1.220377223	0,22232190785984	NA
0.798565715	0,424542274387965	NA

-1.741149851	0,0816573150801805	NA
0.242414131	0,808459285705126	NA
-1.086313501	0,277340316010344	NA
-0.466869428	0,640593299354804	NA
1.757205281	0,078882825500088	NA
1.537166748	0,12425248094903 6	NA
1.812585487	0,0698957778598825	NA
-0.006483185	0,994827203006492	NA
-0.989236742	0,322547325879931	NA
-0.598409955	0,549566424977646	NA
1.146643825	0,251528856149462	NA
0.638344504	0,523249447552691	NA
1.525541168	0,127124173557653	NA
-1.612932427	0,106759189167625	NA
1.504648675	0,132414425162004	NA
-0.675765555	0,499189507494973	NA
0.615678228	0,538106909215812	NA
-0.79312321	0,427706041572714	NA
-0.075258348	0,940009161216312	NA
-1.554582568	0,120045518029049	NA
-1.139306301	0,254575421358083	NA
0.092185272	0,926550839333428	NA
-1.315215365	0,188437532580293	NA
1.181444798	0,237426070586928	NA
0.214467599	0,830182431496366	NA
0.987160034	0,323564190804989	NA
0.961393458	0,336354372288873	NA
-1.504352082	0,132490735317247	NA
0.055601689	0,955659118683933	NA
0.970023209	0,332034923552179	NA
1.372646694	0,169862208161374	NA
-1.309564925	0,190343060599459	NA
-1.418041778	0,156178567802467	NA
-1.150383165	0,249986090828483	NA
1.613974875	0,106532876069171	NA
1.239270554	0,215245319596738	NA
-0.842052009	0,399758842099495	NA
0.453531551	0,650166029309233	NA
0.444206845	0,656893043940136	NA
-1.240892348	0,214645520024578	NA
0.883914318	0,376742467625675	NA
-0.034735079	0,972290989028377	NA
-0.117957804	0,906101091820667	NA
-0.676173323	0,4989306077145	NA
-0.003735471	0,997019531982622	NA
1.679074079	0,0931376073558624	NA
-1.179743605	0,238102205228536	NA
-0.305505209	0,759981402992263	NA
-1.120519933	0,262492263354585	NA
-1.451313452	0,146692597277137	NA

0.646801062	0,517760704795901	NA
1.657898138	0,0973380280871779	NA
0.586668567	0,557426322212424	NA
-1.023281296	0,306174864732277	NA
-0.649981509	0,51570416615251	NA
0.613743411	0,539384897237371	NA
-0.637335933	0,523906049855487	NA
-0.67855756	0,497418241228834	NA
-0.99524932	0,319615019802075	NA
0.477612219	0,632926232567918	NA
0.007941626	0,993663566028807	NA
0.44742625	0,654567326492868	NA
1.4676759	0,142192280216237	NA
0.130230811	0,896383818662756	NA
1.027765641	0,304060082086779	NA
-0.766647335	0,443291223930111	NA
0.51423887	0,607084995083386	NA
1.223626014	0,221093341379747	NA
0.163694702	0,869971488283345	NA
-0.954902853	0,339626827050541	NA
-0.583005123	0,559889851691681	NA
0.476062449	0,634029888375903	NA
-1.020928915	0,307288118638116	NA
-0.867765321	0,385522818125867	NA
1.102421085	0,270278648902268	NA
0.323605691	0,74623657882582	NA
-1.256301828	0,209006559372998	NA
0.005085426	0,995942434828066	NA
-0.770212164	0,441174049284021	NA
-0.689142144	0,490733821439491	NA
1.015140027	0,31003908394802	NA
-1.125158954	0,26052168301557	NA
-0.431791953	0,66589262860051	NA
-1.506877069	0,131842170108146	NA
-0.755216357	0,450119175534135	NA
0.090161201	0,928159115607389	NA
1.534673265	0,124864106018147	NA
0.69823288	0,485031567089583	NA
1.584310086	0,113123171757635	NA
0.224040319	0,822725930740113	NA
0.292858305	0,769630459654025	NA
-0.149811084	0,880913663938074	NA
-0.274387289	0,783787010071201	NA
-0.588520169	0,556183200243747	NA
1.345590518	0,178434611317097	NA
-1.109711085	0,267123544471681	NA
-2.584906131	0,00974055120760132	NA
0.658666839	0,510109731932461	NA
0.06400237	0,948968340054993	NA
0.654081172	0,513059510995172	NA

0.277041007	0,781748619132184	NA
0.335254443	0,737433156197398	NA
-0.039757456	0,968286494773401	NA
2.923672573	0,00345928411831945	NA
1.670386808	0,0948428579615705	NA
-2.323981724	0,0201264736012511	NA
-0.948594172	0,342827056440627	NA
1.294646054	0,195442357334755	NA
0.794010006	0,427189606041747	NA
1.112044832	0,266118874950002	NA
-0.617182286	0,537114498509947	NA
0.710293412	0,477522204144607	NA
0.137542898	0,890601687398792	NA
1.63593358	0,101853489078954	NA
-0.055808644	0,955494248743703	NA
0.281403185	0,77840117542237	NA
0.082752958	0,93404797514011	NA
0.341734251	0,732550893395931	NA
0.293920356	0,768818762753476	NA
-1.500024746	0,13360799262544	NA
-1.629688299	0,103167391709942	NA
-1.669253012	0,0950672481878108	NA
-0.045244262	0,963912614736721	NA
-1.218320224	0,223102307522016	NA
0.179366918	0,857649605773666	NA
0.370200134	0,711233376583752	NA
0.80561978	0,420462116919954	NA
0.982935218	0,325639323318785	NA
-1.049628163	0,29388910368678	NA
0.088827078	0,929219337336513	NA
-2.642753001	0,00822350013737349	NA
0.21016509	0,833538824867079	NA
-0.537570274	0,590873757048928	NA
-0.809591665	0,418174900011195	NA
1.716174459	0,0861301165090623	NA
-1.381139158	0,167236177545568	NA
0.39557795	0,692416406408256	NA
-0.724065771	0,469025357300897	NA
-0.320442555	0,748632870452204	NA
-0.191999827	0,847742340899757	NA
-1.044079068	0,296448809219671	NA
-0.420266995	0,674290417757861	NA
0.888222396	0,374421134157139	NA
1.713890259	0,0865488824639867	NA
-0.328478323	0,742550031798832	NA
-0.460290569	0,645307669966689	NA
-0.664839263	0,506153307141561	NA
0.651329189	0,514834010039366	NA
-0.572802476	0,566778442226557	NA
-1.246146534	0,212710603673976	NA

0.448004401	0,654150020218895	NA
0.967104038	0,333492026904532	NA
-1.902685372	0,0570816118545786	NA
-0.321137957	0,748105846678345	NA
0.029786431	0,976237380388666	NA
2.27214819	0,0230775613043539	NA
0.5482591	0,583514006810809	NA
-0.669139929	0,503406221488825	NA
-1.299260554	0,193854526561514	NA
0.843710152	0,398831393231863	NA
-1.203692817	0,228708329530844	NA
0.468827722	0,6391927827507	NA
-0.331561219	0,740220607036557	NA
-0.05459037	0,956464811106628	NA
1.403785372	0,160382771310832	NA
-0.926307575	0,354286160171292	NA
1.372999237	0,169752583824826	NA
0.603133706	0,546419745085777	NA
1.32038017	0,186708120911186	NA
-1.749643551	0,0801798399154095	NA
0.267131511	0,789367906506856	NA
0.811518589	0,417067922189847	NA
0.938866255	0,347799416780787	NA
0.594990858	0,55184958174441	NA
-0.814059917	0,41561062668145	NA
0.373723933	0,708609717394835	NA
0.827086838	0,408187843881514	NA
-0.908131297	0,363808856877745	NA
1.343278126	0,179181932385115	NA
-0.915118687	0,360129325626108	NA
-0.281466984	0,778352248060147	NA
0.433138815	0,664913925631805	NA
-0.091861486	0,926808091608807	NA
1.11410687	0,265233341858096	NA
-0.567516212	0,570363515150104	NA
-1.102439988	0,270270435073717	NA
0.416467845	0,677067691870501	NA
0.309979306	0,756576693578052	NA
-0.980910576	0,326636841510029	NA
-0.284792242	0,775803316486659	NA
-1.437017026	0,150713155686458	NA
1.332054901	0,182842148406846	NA
-0.545006961	0,585748730329158	NA
-0.204422197	0,838023585989779	NA
1.45226523	0,14642787156545	NA
-0.569198875	0,569221183949378	NA
1.039470944	0,298585764510218	NA
0.716215039	0,473858578424452	NA
-0.700155921	0,483829935709959	NA
-0.26896028	0,787960248906893	NA

2.912042714	0,00359073529412581	NA
-1.356992469	0,174783603512803	NA
-0.537746382	0,590752152962148	NA
2.172533147	0,0298154722731778	NA
0.267475434	0,789103125908198	NA
1.663555168	0,0962013573402146	NA
0.036987475	0,970494992790217	NA
-1.590876052	0,111637473116782	NA
0.361278067	0,71789158825719	NA
-0.953772716	0,340198702973448	NA
0.348597505	0,727391501434603	NA
0.324061925	0,74589115236859	NA
1.437759496	0,150502305329182	NA
-0.059277516	0,952731068980313	NA
0.116905532	0,906934914072108	NA
0.831555463	0,405659909936385	NA
0.906372517	0,364738714122409	NA
0.375595758	0,707217444678276	NA
-0.012029969	0,990401705110607	NA
0.225269106	0,821769931961694	NA
-1.096363399	0,272919774770036	NA
0.496862591	0,619285953659733	NA
-0.324901076	0,745255942865674	NA
0.33905388	0,734569141456559	NA
-1.330192302	0,183454919789442	NA
-0.736278058	0,461561496928194	NA
0.054225241	0,95675571091795	NA
0.468727705	0,639264280802956	NA
-1.263547485	0,206392492687983	NA
-0.260664272	0,794351420418626	NA
-0.202729178	0,839346715916979	NA
0.301327056	0,763165111056011	NA
0.164221511	0,869556767749328	NA
0.261498056	0,793708448661749	NA
-0.389120323	0,697187140491629	NA
-1.356992076	0,174783728173854	NA
1.707130163	0,0877978618282757	NA
0.899841196	0,368204767765373	NA
1.742694362	0,0813870170719795	NA
0.689558205	0,490472058408337	NA
-0.68336147	0,494378460562173	NA
-0.897661507	0,369366036869107	NA
1.180882528	0,237649392924508	NA
0.483111779	0,629016370943628	NA
-0.050005684	0,960117858810944	NA
0.516758278	0,605324908119165	NA
0.423721164	0,671769178505366	NA
0.931316693	0,351689769385753	NA
0.678683467	0,497338443676594	NA
1.028090932	0,30390705588418	NA

-1.043162868	0,296872867838477	NA
1.002693556	0,316008739995452	NA
2.55192656	0,0107129097548207	NA
0.963365416	0,335364175271377	NA
1.6242078	0,104331466694419	NA
-1.039049117	0,298781893745202	NA
0.564131086	0,572664923244393	NA
-0.272576524	0,785178760310459	NA
0.940058422	0,347187594338406	NA
-0.275024549	0,783297378275904	NA
1.395749569	0,162789921797284	NA
0.114893747	0,908529341596074	NA
1.661732929	0,096566335654229	NA
0.441565146	0,658803910256228	NA
-0.522681415	0,601195973722439	NA
0.605557382	0,544808709556838	NA
0.647542817	0,517280692564767	NA
-1.756671376	0,0789738392278555	NA
1.397536381	0,162252334296098	NA
0.350008185	0,726332555106666	NA
-0.487095903	0,626190369486184	NA
-0.755783631	0,449778932720322	NA
-0.593541186	0,552819028178526	NA
-0.67958543	0,496766997874895	NA
0.485774024	0,627127393135144	NA
-0.439094203	0,660593279058139	NA
1.076143506	0,281863081040438	NA
1.828957688	0,0674059470623025	NA
2.290673426	0,0219823086992475	NA
1.841928197	0,065485653426666	NA
1.586066099	0,112724316108792	NA
0.115219384	0,908271234404384	NA
0.477600192	0,632934794002013	NA
-0.512356252	0,608401702647377	NA
1.200499365	0,229945459195369	NA
-0.479244481	0,631764713682506	NA
1.53705062	0,124280914005444	NA
-0.239960332	0,810361008630203	NA
-0.75444086	0,450584544271162	NA
-1.092328639	0,274688676691281	NA
0.056774736	0,954724639468338	NA
-1.288948211	0,197416091737155	NA
-0.379968718	0,703968635804669	NA
-0.023770299	0,981035831220231	NA
0.981031302	0,326577305428599	NA
-0.161455885	0,87173435382451	NA
2.436000941	0,0148506459109106	NA
0.458376943	0,646681653603736	NA
0.032605379	0,973989280569701	NA
0.619857102	0,53535187048637	NA

0.955827311	0,339159487972715	NA
-0.903801174	0,366100838906932	NA
1.344793933	0,178691789908584	NA
1.960558103	0,0499305938773154	NA
-0.182185941	0,855436805571469	NA
-0.902000169	0,367056777954405	NA
-0.326317097	0,744184453906551	NA
0.120581096	0,904022843106978	NA
0.274788171	0,783478986749051	NA
1.806611822	0,0708228248884792	NA
0.268961321	0,787959447620626	NA
1.349176554	0,177280263873173	NA
0.742884842	0,457551403481454	NA
1.902604212	0,0570922090786578	NA
1.289123084	0,197355299603512	NA
-0.33620873	0,736713469842527	NA
0.383104042	0,701642622401804	NA
0.927813561	0,353504288612454	NA
-1.497808796	0,134182934872389	NA
0.653151827	0,513658403188429	NA
1.042633976	0,297117847052683	NA
1.086596535	0,27721515716879	NA
-0.948959586	0,342641167317591	NA
-0.602767272	0,546663521846949	NA
-0.53627905	0,59176570725262	NA
0.9061409	0,364861279418388	NA
-0.084897663	0,93234274979673	NA
1.480052899	0,13885912999081	NA
0.560846369	0,574902273451399	NA
-0.586441245	0,557579034593267	NA
1.312091028	0,18948942277414	NA
0.634793288	0,525563241214465	NA
0.84320232	0,399115301280832	NA
-0.208612768	0,834750542554734	NA
1.217416236	0,223445888724061	NA
0.402924831	0,687003524036295	NA
0.09912824	0,921036450212609	NA
0.802218887	0,422426353089873	NA
1.763287224	0,0778520590170816	NA
2.052256413	0,0401447495483767	NA
-0.293186556	0,769379559515155	NA
-0.447417308	0,654573781177372	NA
0.28340795	0,776864135016904	NA
1.112411303	0,265961347021029	NA
-0.578814474	0,562714363556942	NA
-0.781953097	0,434242138801445	NA
-0.200321652	0,841229029511386	NA
-0.217784216	0,827597243413089	NA
0.890050796	0,37343861129527	NA
-2.286852461	0,022204436225236	NA

-0.345671693	0,72958946000633	NA
-0.311885156	0,755127804293126	NA
0.509893656	0,610125966523122	NA
-1.501721222	0,133169120839137	NA
0.293062668	0,769474251539194	NA
-0.066704841	0,94681667999178	NA
0.38840546	0,697716004190569	NA
-0.72508655	0,468398934939538	NA
1.692070553	0,0906325222114682	NA
0.713132738	0,475763622978292	NA
-0.487943129	0,625590124094943	NA
-0.561749085	0,574286986137886	NA
0.776730477	0,437317801743922	NA
0.533213826	0,593885571952203	NA
-0.528189142	0,597368065110326	NA
1.23896344	0,215359037878934	NA
-1.041652978	0,297572597259538	NA
-1.859353675	0,0629770235367396	NA
1.377099192	0,168481582608648	NA
0.171463903	0,863859011130802	NA
-0.836780152	0,402716156142624	NA
-0.128217616	0,897976759700219	NA
-0.062198205	0,950404991961299	NA
-0.292771312	0,769696957788741	NA
-0.52625878	0,59870841859098	NA
-1.848197535	0,0645737719020012	NA
0.031748934	0,97467227092365	NA
-1.089478728	0,275942830448913	NA
0.068694309	0,945232948288421	NA
2.136608591	0,0326298402635712	NA
-2.078574368	0,0376564889659194	NA
0.56431674	0,572538590831708	NA
-1.819611056	0,0688182567451552	NA
1.772406008	0,0763271852088913	NA
0.12569652	0,899972156611863	NA
0.386750145	0,698941188818184	NA
-0.353371055	0,723810282429422	NA
1.932674293	0,0532763339156263	NA
-0.647002248	0,517630488264816	NA
-1.560420463	0,118660552327715	NA
0.438341093	0,661139041909555	NA
0.334195769	0,738231836819597	NA
1.027451627	0,304207852141055	NA
-0.413638598	0,67913880151965	NA
-0.55268674	0,580477935017341	NA
0.620627368	0,534844828182117	NA
0.500066465	0,617028278389394	NA
0.449925772	0,652763963896658	NA
0.042625468	0,966000093230855	NA
-2.063472766	0,03906773271244	NA

0.413515283	0,679229127232405	NA
0.182295108	0,855351137926652	NA
-0.08169727	0,934887449689378	NA
-1.085122395	0,277867449828612	NA
-3.021918192	0,00251178420671462	NA
0.493101092	0,621941159091333	NA
0.318912083	0,749793181638115	NA
-0.754010626	0,450842841289546	NA
0.633492359	0,526412169357686	NA
0.143231304	0,886107507767571	NA
-1.003679772	0,315532989930268	NA
0.418466945	0,675605749511045	NA
0.213570768	0,830881797034978	NA
0.377690865	0,705660254881785	NA
-0.335396237	0,737326206079398	NA
1.097168489	0,272567745246015	NA
0.085083325	0,932195146739163	NA
-1.355117314	0,175380183325781	NA
1.149465684	0,250364009031501	NA
1.020466674	0,307507186896337	NA
-1.18824793	0,234735745618168	NA
0.199774064	0,841657286438386	NA
0.259685131	0,795106663227413	NA
1.34295985	0,179284974836564	NA
0.606321092	0,544301555121775	NA
-0.2010424	0,840665420494405	NA
-0.513843887	0,607361141367141	NA
0.65318425	0,513637502740579	NA
0.047699963	0,961955363338803	NA
-0.562547587	0,573742990981405	NA
-0.627517784	0,530319882724758	NA
0.318761358	0,749907482785003	NA
-0.31176747	0,755217248042811	NA
1.837838306	0,0660862351379404	NA
0.862089098	0,388638489984429	NA
0.093596869	0,925429397045952	NA
1.237025102	0,216077763758281	NA
-0.289580796	0,772136960283348	NA
-0.238017503	0,811867518455769	NA
-0.379211117	0,704531096912659	NA
0.306814227	0,75898477848946	NA
0.828978961	0,407116312559305	NA
-1.516628045	0,129360620626513	NA
0.634861967	0,525518443814438	NA
1.632022173	0,10267481519885	NA
0.06657395	0,946920884433791	NA
0.890047623	0,373440315211583	NA
1.259199403	0,207958316974329	NA
1.181857673	0,237262179082272	NA
1.373667975	0,169544783330694	NA

0.010788958	0,991391823615717	NA
0.603817285	0,545965125983688	NA
1.018057389	0,30865068397537	NA
-0.806889575	0,419730105703208	NA
-0.378495964	0,70506219107813	NA
-0.28887244	0,772678994894366	NA
2.685491772	0,00724231579725654	NA
-0.758803483	0,447970122092014	NA
-0.136922635	0,891091947555433	NA
1.541822087	0,123116833224759	NA
-1.564794439	0,117631116055094	NA
1.077950702	0,281055756741719	NA
0.945850005	0,344225096196358	NA
0.008004846	0,993613124853356	NA
1.78287591	0,0746065053848436	NA
-0.063581991	0,949303071876369	NA
2.098002516	0,0359049230809927	NA
-0.372812708	0,709287844301034	NA
-2.524532269	0,0115852368088697	NA
-0.4696394	0,638612670128917	NA
-0.118720322	0,905496936134034	NA
0.721678625	0,470492086623393	NA
-0.725524343	0,468130416289982	NA
1.56460477	0,117675609785378	NA
-0.305102059	0,760288422706972	NA
0.334538927	0,737972921815809	NA
-0.685379082	0,493104740133495	NA
0.020128595	0,983940789391347	NA
0.540478252	0,588867254920719	NA
-0.355261767	0,722393495165309	NA
0.042324967	0,966239642657153	NA
0.354211934	0,723180060518866	NA
0.945442169	0,34443318239364	NA
0.001098019	0,999123907549944	NA
-0.960707953	0,336699031247055	NA
-0.753631645	0,451070436852024	NA
-0.817219524	0,413802987259716	NA
-1.093208674	0,274302189386407	NA
-1.448465302	0,147486963225031	NA
-0.749469248	0,453574427012042	NA
0.298201962	0,765549024497856	NA
1.196983697	0,231312909158287	NA
-0.875143639	0,381495755450125	NA
-0.128285512	0,897923030409798	NA
1.092552209	0,274590455633733	NA
-0.094767982	0,924499118952845	NA
-0.61429564	0,539019982837385	NA
-0.141570884	0,887418966213108	NA
0.899006501	0,368649197899167	NA
0.116124014	0,907554257117096	NA

0.196017426	0,844596526339095	NA
0.519891125	0,603139461249772	NA
0.28233955	0,777683162239615	NA
1.397864198	0,162153851502527	NA
-0.418573046	0,675528191558217	NA
-0.021195103	0,98309002083607	NA
0.17564319	0,860574268984748	NA
0.999347299	0,317626480034964	NA
-0.96327684	0,335408612642539	NA
0.247768468	0,804313553301411	NA
0.482308236	0,629586997114193	NA
0.090684459	0,927743319416166	NA
1.364444696	0,172427648982822	NA
0.371824345	0,710023636753428	NA
-0.290405565	0,771505986943795	NA
0.120578483	0,904024912317848	NA
1.370480905	0,170536831976187	NA
0.578397841	0,562995551413654	NA
2.401685542	0,0163197305346346	NA
0.513341123	0,607712722957127	NA
1.606213174	0,108227079750515	NA
0.174932092	0,861132993257538	NA
-1.621374547	0,104937336189171	NA
-1.923422327	0,0544270325622676	NA
0.209628695	0,833957481741049	NA
0.783729568	0,433198808711034	NA
-0.619758408	0,535416855131461	NA
2.871540526	0,00408476402789402	NA
0.731169948	0,464675353466593	NA
-0.613523412	0,53953030710021	NA
-1.111833011	NA	NA
1.713046846	0,0867039216472778	NA
-1.366180534	0,171882305661424	NA
0.585656058	0,558106668805371	NA
-0.46311141	0,643284511921916	NA
-0.563701443	0,572957334869233	NA
-0.29663616	0,766744301136487	NA
0.531979624	0,594740108222407	NA
1.144013851	0,252617892724515	NA
1.180567946	0,237774403697175	NA
-0.504208496	0,614114871073341	NA
-1.698999887	0,0893192049240089	NA
-3.148840485	0,00163919644181395	NA
-0.769412291	0,441648594501874	NA
1.092035451	0,2748175192225	NA
-0.307931826	0,758134207757825	NA
-0.099662883	0,920611968464966	NA
0.408437982	0,682952153443358	NA
1.443009145	0,149017895030175	NA
3.035309964	0,00240288597427304	NA

-1.625666272	0,104020668672049	NA
0.480155906	0,63111653821511	NA
1.940720085	0,052292238819627	NA
1.091733225	0,274950376759932	NA
-0.686744325	0,492243859889093	NA
-0.400548073	0,68875288354046	NA
-0.619424007	0,535637069908201	NA
-0.966536264	0,333775909809113	NA
1.34245731	0,179447763116258	NA
0.117229289	0,906678357583265	NA
-0.327726734	0,743118287823431	NA
-0.205506165	0,837176682195788	NA
-0.499309389	0,617561442036571	NA
-0.037246082	0,970288796339796	NA
0.711531265	0,47675508377276	NA
-0.543360177	0,586881838526491	NA
1.892540817	0,0584189606671645	NA
1.21120084	0,225818440932387	NA
-0.205517218	0,837168047322147	NA
1.757636484	0,078809381429186	NA
-1.260559552	0,207467579200014	NA
-1.04683085	0,295177599259696	NA
0.042359564	0,966212062773054	NA
1.427042211	0,153567728167834	NA
-0.054840385	0,956265626416423	NA
-0.034803366	0,972236536625373	NA
0.466330848	0,640978701415451	NA
2.386369172	0,0170156603949821	NA
0.415628866	0,6776816008293	NA
-0.260582738	0,794414303054966	NA
0.013214037	0,989457030810752	NA
0.497048039	0,619155175465936	NA
0.659618701	0,509498550450159	NA
-0.051269094	0,959111094882698	NA
-2.269579165	0,0232331289502035	NA
0.333252621	0,738943600273534	NA
-0.055576387	0,955679275975722	NA
-0.690962946	0,489588827386325	NA
-1.04284215	0,297021406064043	NA
2.003561643	0,0451170381161301	NA
0.197055164	0,84378436920459	NA
-0.467748601	0,63996438066073	NA
-0.120208563	0,904317934940071	NA
-1.604202498	0,108669424389926	NA
-0.920126587	0,357506612228229	NA
-0.471414532	0,637344740441076	NA
2.223460577	0,0261847588927717	NA
-0.945794164	0,344253582653903	NA
-0.413138272	0,679505310628703	NA
-1.166880513	0,243258626412392	NA

0.191134396	0,848420300213954	NA
0.075715354	0,939645560016972	NA
-0.618996974	0,535918352232058	NA
0.488606608	0,625120233530821	NA
-1.111948452	0,266160314636368	NA
-0.628205502	0,52986932674748	NA
0.714125713	0,475149447671043	NA
1.561914103	0,118308225768514	NA
0.583663909	0,559446453497346	NA
-0.775412693	0,438095836955901	NA
2.549045708	0,0108018141797461	NA
0.202513082	0,839515632341979	NA
0.406464424	0,684401391633406	NA
0.590388007	0,554930549088818	NA
-1.378587184	0,168022070259317	NA
-1.145323663	0,252075107199939	NA
-0.005960203	0,995244474401237	NA
0.761897677	0,446121076523	NA
2.439763372	0,0146968852236273	NA
0.240376062	0,810038733837228	NA
-1.399010229	0,161809915658815	NA
0.004297102	0,996571419064293	NA
0.945290693	0,344510488709448	NA
-0.387284871	0,698545324413286	NA
-0.739751111	0,459451028919488	NA
0.499191829	0,617644250608015	NA
-1.617458908	0,105779258792861	NA
0.744940653	0,456307597976444	NA
2.312487449	0,0207508361906406	NA
1.530367668	0,125925747298981	NA
2.004524652	0,0450138888978927	NA
-0.064160732	0,948842243872882	NA
2.622910755	0,00871821060609907	NA
-0.811391113	0,417141100915366	NA
0.398559962	0,690217467751762	NA
0.374152529	0,708290839559131	NA
0.884827933	0,376249441530534	NA
0.146592401	0,883453751776077	NA
-0.403530109	0,686558289437629	NA
1.764197494	0,0776987345534128	NA
-1.228383379	0,21930308413461	NA
0.919844325	0,357654116577326	NA
1.047148865	0,295030924945989	NA
-1.670736472	0,0947737415377589	NA
-0.396687574	0,691597864168955	NA
0.750768606	0,452791926397246	NA
0.658483962	0,510227199316528	NA
-1.259856437	0,207721156007211	NA
-0.846816108	0,397097628384799	NA
1.7313384	0,0833914263404671	NA

0.925922671	0,354486168512608	NA
-0.695981919	0,486440153309598	NA
0.274051741	0,784044859198304	NA
-0.421674222	0,673262822875704	NA
-0.640669002	0,521737758116131	NA
0.007984811	0,993629110329329	NA
-0.657781331	0,510678652554612	NA
1.290845244	0,196757345593539	NA
3.215521526	0,00130207728936837	NA
-1.013812098	0,310672421204832	NA
0.143871512	0,885601931492245	NA
-0.433822256	0,664417518296597	NA
-0.104749541	0,916574550444189	NA
-0.569802176	0,568811879356822	NA
0.057312855	0,954295980362018	NA
-0.091415566	0,927162393673428	NA
0.463970386	0,64266896298779	NA
0.242531055	0,808368696802691	NA
0.717558509	0,473029548387455	NA
0.331502517	0,740264940347731	NA
-1.377773336	0,168273280986989	NA
0.459120775	0,646147438901037	NA
0.459095601	0,646165515209136	NA
-0.035539369	0,971649653870319	NA
-2.517707488	0,0118121373841662	NA
1.811583759	0,0700505363395772	NA
0.464735937	0,64212056964696	NA
-0.388382913	0,697732687179785	NA
1.37184171	0,170112719554545	NA
-1.609777907	0,107446349961963	NA
-1.898828631	0,0575870114616013	NA
0.380856488	0,703309740753045	NA
1.105394116	0,268988852255297	NA
-0.250833702	0,801942683923886	NA
0.047260515	0,962305597492461	NA
0.938114387	0,348185629064183	NA
0.463496894	0,643008240537442	NA
-0.596510822	0,55083402625866	NA
-0.231285158	0,817093276376786	NA
-1.129815723	0,258553881443603	NA
0.51801942	0,604444719719586	NA
0.439817423	0,660069347864741	NA
-0.436111184	0,662756063184696	NA
-0.58102294	0,561224992831227	NA
-0.414147589	0,678766022628207	NA
-0.432772164	0,665180298051526	NA
0.175546291	0,860650400558568	NA
-0.77472848	0,438500118417288	NA
1.246803586	0,212469525285489	NA
0.800315794	0,423527854300009	NA

-0.155896549	0,876114565923255	NA
0.737531167	0,460799396767813	NA
0.301032839	0,763389453245469	NA
-1.176916773	0,239228726584422	NA
-0.217736199	0,827634657493489	NA
-0.24806653	0,804082931181955	NA
0.087580204	0,93021033648926	NA
-0.932669613	0,350990578867124	NA
0.076890509	0,938710647533324	NA
-0.398203752	0,690480000827604	NA
0.415035602	0,678115841029977	NA
1.199706127	0,230253492807756	NA
-0.822082906	0,41102971360066	NA
1.024999581	0,305363385594577	NA
1.037979766	0,299279475448336	NA
-0.902773971	0,366645868183877	NA
0.110515627	0,912000457012016	NA
-0.894806353	0,370890613418073	NA
0.83817478	0,401932549217812	NA
-0.818781912	0,412910852769478	NA
0.37474063	0,707853372370726	NA
-0.125567044	0,90007465126369	NA
-0.930660187	0,352029370054186	NA
0.928287053	0,353258688132517	NA
-0.202524133	0,839506993743541	NA
0.893119781	0,37179303114959	NA
-0.44531284	0,656093689777349	NA
0.674151298	0,500215128596663	NA
0.260332706	0,794607146257015	NA
-0.365060249	0,715066432838229	NA
0.17295741	0,8626849046455	NA
-1.522329173	0,12792662175591	NA
1.053165241	0,292265265856947	NA
-1.189334284	0,234308149358758	NA
0.884973659	0,376170838105601	NA
0.271024169	0,786372443246123	NA
-0.275645716	0,782820193441604	NA
1.667240078	0,0954666765045079	NA
-2.29045208	0,0219951234138486	NA
0.488382363	0,625279031867565	NA
0.592933079	0,553225939018935	NA
-0.107507174	0,914386633949918	NA
1.242184167	0,214168620028388	NA
1.19103349	0,23364043792064	NA
-0.011427178	0,990882629650157	NA
-0.62599974	0,531315111631154	NA
-3.5617995	0,000368321625421103	NA
0.263054743	0,792508385958191	NA
0.57258811	0,566923611940497	NA
0.565423664	0,571785632674487	NA

0.986454111	0,323910322990841	NA
1.466129892	0,142612903647031	NA
-0.088688201	0,929329710021528	NA
-0.446296842	0,655382837426755	NA
0.570451461	0,568371534495731	NA
-2.058978496	0,0394962985814468	NA
0.573079707	0,566590726588884	NA
0.178934551	0,857989092923226	NA
-0.071590354	0,942927916602966	NA
1.030814425	0,302627852587085	NA
0.645274445	0,518749350534693	NA
0.418093728	0,675878590302859	NA
0.680193073	0,496382217798949	NA
0.54212939	0,587729373580875	NA
-0.926461262	0,354206319810036	NA
0.823774798	0,410067530150452	NA
-0.29144768	0,770708953098684	NA
-0.251912383	0,80110878788095	NA
-1.302616385	0,192705761111203	NA
0.176425701	0,85995951449841	NA
1.331946905	0,1828776364125	NA
-0.24257688	0,808333193320778	NA
1.376865054	0,16855397333838	NA
-0.33724902	0,735929186754611	NA
1.846598428	0,0648053622581205	NA
1.315350818	0,1883920265756	NA
0.24689418	0,804990120383907	NA
2.782571901	0,00539299141535771	NA
0.152573175	0,878734880426555	NA
-0.203469962	0,838767721841148	NA
-0.13967441	0,888917246040843	NA
0.307421628	0,758522468171038	NA
0.707641878	0,479167679324869	NA
-0.561715004	0,574310210135272	NA
0.189483318	0,849714028245882	NA
-0.600814474	0,547963562365764	NA
0.505495912	0,613210568987277	NA
-0.641393046	0,521267349688848	NA
-0.345319277	0,729854356264299	NA
0.132579191	0,894526190139166	NA
-0.137726364	0,890456682759724	NA
0.040173225	0,967955023444045	NA
0.099841914	0,920469831826609	NA
-1.274202899	0,202591518026293	NA
0.380249658	0,70376010090317	NA
0.600164185	0,548396819850699	NA
0.22034023	0,825606191119536	NA
0.121776472	0,903076048199994	NA
0.545068973	0,585706080798347	NA
-1.520832012	0,12830199897255	NA

-0.498597769	0,618062777058368	NA
-0.243648968	0,807502700236655	NA
0.490433607	0,623827100152963	NA
-1.323496773	0,185670228791416	NA
-0.248799041	0,803516232928126	NA
1.550805551	0,120948289858011	NA
1.242420584	0,214081424965119	NA
1.638960378	0,101221511870514	NA
0.210592804	0,833205028260304	NA
0.421161134	0,673637422865836	NA
0.801650822	0,422754970554105	NA
1.188827837	0,234507421340305	NA
0.520816395	0,602494681044085	NA
-0.264742023	0,79120820233199	NA
0.105522383	0,915961309679671	NA
0.607407281	0,543580657374105	NA
-2.831749489	0,00462941022851973	NA
-0.198195703	0,842891948955942	NA
1.193582086	0,232641485042169	NA
-1.157888772	0,246909430172123	NA
0.809069679	0,418475067729444	NA
-0.11832295	0,905811773274644	NA
-0.480691296	0,630735918414948	NA
0.952709864	0,340737094277568	NA
-1.879242461	0,0602113942686961	NA
0.457656489	0,647199252975738	NA
1.232743512	0,217671472373895	NA
0.195225915	0,845216092337451	NA
1.130066961	0,258448009926956	NA
1.754175398	0,0794004573446688	NA
0.355219794	0,72242493728908	NA
0.301157762	0,763294196216732	NA
0.473701921	0,635712483043305	NA
0.691177882	0,489453761946308	NA
-0.992089236	0,321153996718474	NA
1.655999233	0,0977219741046697	NA
0.667846433	0,504231623741155	NA
0.802478128	0,422276435339204	NA
1.358004372	0,1744622967694	NA
0.199540765	0,84183975874714	NA
0.018700832	0,985079764756319	NA
-1.680140478	0,0929299868304417	NA
-0.215844735	0,829108776650153	NA
-0.185520963	0,852820439397195	NA
2.040057842	0,0413445650356997	NA
-2.735093928	0,00623624782296278	NA
-3.430245068	0,000603036334769077	NA
-0.881737487	0,377918783800857	NA
0.156691075	0,875488320996274	NA
-0.084594299	0,932583931762136	NA

-0.046658454	0,96278544277729	NA
-0.050148386	0,96000414176614	NA
-0.953776881	0,340196594491542	NA
-0.821931519	0,411115872899207	NA
0.43716686	0,661990341401878	NA
2.550814341	0,0107471559554542	NA
-0.872403692	0,382988187366432	NA
-1.343989055	0,178951926158465	NA
-0.042565662	0,966047768425426	NA
0.878361451	0,379747597170663	NA
0.57702649	0,563921560620856	NA
-0.005035751	0,995982069354542	NA
0.332917725	0,739196389129227	NA
-0.555122351	0,578810975704345	NA
-0.702284432	0,482501800242361	NA
1.164598608	0,24418150745178	NA
-0.067901705	0,945863881175474	NA
0.475937956	0,634118580369379	NA
-0.006780938	0,994589635837869	NA
0.06630304	0,947136562981187	NA
-1.307409497	0,191073673757202	NA
-0.976553605	0,328790197150529	NA
-0.194460312	0,845815468826939	NA
-1.336097124	0,181517529771807	NA
0.507065466	0,612108884608127	NA
-1.474229005	0,140419942945434	NA
1.563101789	0,118028654877493	NA
0.835803514	0,403265449355864	NA
-1.659455378	0,0970240652323097	NA
0.762096307	0,446002526610268	NA
0.15887071	0,873770737867164	NA
0.785876367	0,431939920982376	NA
-1.862499032	0,0625327817105104	NA
1.232913127	0,217608177405449	NA
0.055381826	0,955834274614384	NA
0.092708137	0,926135432469558	NA
-0.127867797	0,898253596060372	NA
0.404739527	0,685668984013091	NA
0.201458151	0,840340349596642	NA
-0.21043979	0,833324439750706	NA
0.85144501	0,394522197585678	NA
0.795404883	0,426378020668577	NA
-0.67283652	0,501051301091709	NA
1.154343437	0,248359394744056	NA
1.832551874	0,0668692407517866	NA
-0.211903008	0,832182704046201	NA
-1.283477798	0,199324733033766	NA
0.409097219	0,682468317056587	NA
0.325840405	0,744545106510616	NA
-0.413924533	0,678929376694495	NA

1.283266305	0,1993987932551	NA
-0.363108608	0,716523754608995	NA
2.592837419	0,0095187756729032	NA
0.000716842	0,999428043176741	NA
-0.0196317	0,984337175974619	NA
0.50337234	0,614702514750573	NA
2.439793023	0,0146956790409158	NA
-2.877046683	0,0040141620497656	NA
-0.951293832	0,341455236990591	NA
0.863109445	0,38807729566095	NA
-0.679344951	0,496919320978109	NA
0.696143856	0,486338743579579	NA
0.220065329	0,825820276290525	NA
-0.043679186	0,965160130337758	NA
0.327665672	0,743164461545994	NA
-0.366136774	0,714263017865034	NA
0.541106471	0,588434197997172	NA
3.441544349	0,000578403647775239	NA
-1.238172479	0,215652113961555	NA
1.036048818	0,30017936828894	NA
0.157347566	0,874970934501739	NA
-0.442629089	0,658034040617574	NA
-0.493324226	0,621783513029413	NA
1.437255571	0,150645387966542	NA
0.205227923	0,837394054413475	NA
0.325998531	0,744425466272351	NA
0.391556435	0,695385982637511	NA
-1.558540026	0,119105287344514	NA
1.442347171	0,14920445913124	NA
1.968271393	0,0490368195842807	NA
-0.688604557	0,491072152357414	NA
-1.21618628	0,223913968862603	NA
0.96711809	0,333485003388792	NA
-1.919728354	0,0548922208303046	NA
0.647701402	0,517178097115679	NA
0.368690523	0,712358413624299	NA
-2.622121879	0,00873841747328736	NA
-0.43854716	0,660989691351427	NA
0.329945548	0,741441106485997	NA
-0.458078664	0,646895927175621	NA
-1.097739709	0,272318164854889	NA
-0.027140305	0,978347827733623	NA
-2.77013032	0,00560338681661277	NA
0.522148316	0,601567068103262	NA
-0.029610063	0,976378039378546	NA
-1.485736517	0,137348827484446	NA
0.918833929	0,358182442739827	NA
-0.055263256	0,955928735211432	NA
1.744127432	0,0811368716515741	NA
1.702112781	0,0887342283903163	NA

-0.364653334	0,715370197038188	NA
0.788822499	0,43021575767871	NA
0.838807466	0,401577360539728	NA
-0.580485467	0,561587284521686	NA
1.513115008	0,130250447936399	NA
-1.879837973	0,0601301635182703	NA
0.465370602	0,641666083249234	NA
-0.302739891	0,762088095038197	NA
2.233056632	0,0255452100200834	NA
0.555365747	0,578644516801567	NA
-0.110916692	0,911682408963792	NA
0.494967451	0,620623096831294	NA
1.683547933	0,0922690684634723	NA
1.100847027	0,270963240566858	NA
0.399962695	0,689183993908873	NA
-0.421351874	0,67349815619113	NA
2.639855219	0,00829414517362841	NA
0.400427357	0,688841777984893	NA
-0.90300451	0,366523501495733	NA
0.402313809	0,687453093474852	NA
-0.43049625	0,666834694194262	NA
-0.406341422	0,684491753795437	NA
0.333862187	0,738483554684995	NA
0.286151314	0,774762230883537	NA
-0.88662883	0,375278766767337	NA
-2.089755036	0,036639810323837	NA
0.371245749	0,710454501481614	NA
0.010429817	0,991678361071767	NA
-1.37599216	0,168824060389136	NA
-0.2485344	0,803720957212831	NA
0.628191102	0,529878758861156	NA
0.021306791	0,983000926370706	NA
1.747823969	0,0804945135899716	NA
0.348533053	0,727439895578511	NA
0.602634167	0,546752085322126	NA
0.890547419	0,373172018388919	NA
1.911858151	0,055894390641529	NA
0.126621313	0,899240127194683	NA
-0.185084706	0,85316259750107	NA
1.326574538	0,184649463207686	NA
-3.218813107	0,0012872235263729	NA
0.014595398	0,988354970765034	NA
0.407184667	0,683872362594661	NA
-1.185718491	0,23573348979822	NA
0.591077539	0,554468468726881	NA
0.340499077	0,733480717991105	NA
0.261546498	0,793671096604551	NA
1.566103445	0,117324401718155	NA
1.047500122	0,294868976147452	NA
0.62689768	0,530726308150391	NA

0.868485044	0,385128856546189	NA
0.359655024	0,71910513028671	NA
0.883930294	0,376733842526966	NA
-0.015091916	0,987958850386458	NA
1.296933255	0,194654153028662	NA
-0.993437655	0,320496718739448	NA
0.028041197	0,977629293510995	NA
-0.752749609	0,451600392763862	NA
1.005051217	0,314872188432881	NA
0.723177449	0,469570871461195	NA
-0.726829809	0,467330219795773	NA
-1.05082701	0,293338048208496	NA
1.216363845	0,223846350392784	NA
-2.215962615	0,0266940625986807	NA
1.669869067	0,0949452716663686	NA
-1.565044809	0,117572403063695	NA
0.51899205	0,603766285116608	NA
0.734641212	0,462558032003479	NA
1.246184218	0,212696771719139	NA
-0.885128063	0,376087565212568	NA
-0.905809267	0,365036815419937	NA
0.622651648	0,533513463038033	NA
2.187556991	0,0287018875644987	NA
-0.048773088	0,961100129006963	NA
0.766423596	0,443424297123888	NA
-0.084676715	0,932518408479273	NA
1.515239077	0,129711870347923	NA
0.441672769	0,658726017291715	NA
-0.03821495	0,96951630116079	NA
1.291187096	0,196638808667277	NA
-0.926603603	0,354132383670541	NA
0.616625862	0,537481531936089	NA
0.256175907	0,797815011777733	NA
0.452399127	0,65098147594097	NA
-0.879138248	0,37932632170354	NA
-0.042560961	0,966051515916556	NA
1.328948029	0,183865116591805	NA
0.62035835	0,53502188711314	NA
-0.073715851	0,941236485889129	NA
-1.081998222	0,279253317885088	NA
-3.02126911	0,00251717533161125	NA
-0.362938729	0,716650655096033	NA
0.02325161	0,981449571062495	NA
-0.147659807	0,882611253183435	NA
-0.68460839	0,493591071382144	NA
-0.524586915	0,599870384658771	NA
-1.400656788	0,161316730419543	NA
0.454313778	0,649603000570529	NA
-1.464432592	0,143075788343192	NA
0.9088715	0,363417959200346	NA

0.378147581	0,70532096322278	NA
0.237813431	0,812025800880525	NA
-1.021549288	0,306994269823279	NA
-1.321259333	0,186414908640744	NA
-0.774954877	0,438366323103111	NA
0.904062123	0,365962461497118	NA
-3.176956923	0,00148829140432707	NA
0.782261921	0,434060660486922	NA
0.315901524	0,752077255243982	NA
-1.751286487	0,0798965741798465	NA
0.499186278	0,61764816076996	NA
-0.073059378	0,941758866526273	NA
0.521346025	0,60212574433345	NA
1.558497311	0,119115405030646	NA
0.564505729	0,572410002460708	NA
-0.1215886	0,903224842077375	NA
0.04911572	0,96082707594522	NA
2.027734245	0,0425873785890804	NA
0.003484595	0,997219700861207	NA
0.615735266	0,538069257999211	NA
0.097420915	0,922392136081508	NA
-0.60949829	0,542194206043562	NA
-0.918116808	0,358557716287692	NA
0.250772219	0,801990221769614	NA
1.172313948	0,241071033525303	NA
1.179681561	0,238126890441366	NA
0.410321588	0,681570057714997	NA
1.208388844	0,226897729071433	NA
0.065993218	0,94738322477334	NA
2.481047829	0,0130996794347813	NA
-0.396405669	0,691805784767019	NA
0.410807533	0,681213669112998	NA
0.698310865	0,484982805835551	NA
0.440037197	0,659910167020623	NA
-0.540531688	0,588830413887569	NA
1.026763877	0,304531662640658	NA
1.099494422	0,271552465761195	NA
0.92795121	0,353432878774265	NA
1.18832187	0,234706624611542	NA
0.630028581	0,528675884620689	NA
-0.865482419	0,386774061176729	NA
0.314480081	0,753156442555114	NA
-0.719717911	0,471698696134911	NA
1.066092185	0,286381971997379	NA
0.452203682	0,651122255882794	NA
0.902705635	0,366682145224391	NA
-1.917474438	0,0551776854161714	NA
0.366893168	0,713698707719331	NA
-1.17147096	0,241409523938273	NA
2.335630212	0,0195105225582147	NA

0.66285415	0,507423969204182	NA	
-0.352663987	0,724340361207203	NA	
1.110873512	0,266622798441065	NA	
-0.160140469	0,872770422989316	NA	
-0.541807937	0,587950822756617	NA	
-0.886326985	0,375441351952348	NA	
1.01401206	0,310576997742931	NA	
-1.09771688	0,272328136434271	NA	
-1.062246233	0,288123916052275	NA	
1.36192078	0,173222887466239	NA	
1.085316562	0,277781472850665	NA	
-2.297267953	0,0216034887920954	NA	
0.508091848	0,611388931612349	NA	
2.305111309	0,0211603402805262	NA	
1.246814978	0,212465347218758	NA	
0.601687668	0,547382060615387	NA	
-1.009790123	0,31259585301218	NA	
0.47996588	0,631251654640976	NA	
1.052390832	0,292620273078461	NA	
0.316940336	0,751288874623453	NA	
0.126875144	0,899039219838286	NA	
1.216119323	0,223939470789876	NA	
0.198430784	0,842708034147335	NA	
0.17191507	0,863504299074542	NA	
1.688080905	0,0913956993335075	NA	
-1.897172797	0,0578051358707027	NA	
1.561234766	0,118468368799625	NA	
0.111112764	0,911526926966063	NA	
0.244790724	0,806618476508536	NA	
-2.211024358	0,0270341503211855	NA	
0.24495305	0,806492784596687	NA	
0.182032555	0,855557177308691	NA	
0.011648655	0,990705927942758	NA	
1.30148747	0,193091650781226	NA	
0.005500832	0,995610993114918	NA	
1.299637784	0,193725143329029	NA	
0.471533599	0,637259731662294	NA	
0.648549385	0,51662968020594	NA	
-0.560938412	0,57483952329413	NA	
0.564204817	0,57261474961049	NA	
-0.607442956	0,543556988705249	NA	
-0.392521943	0,694672601771083	NA	
0.498269209	0,618294307376913	NA	
-0.338123365	0,735270223803637	NA	
0.100680508	0,919804085076658	NA	
-0.025295854	0,979818980801621	NA	
0.213386509	0,831025502363979	NA	
0.810315938	0,41775861743662	NA	
1.197622105	0,231064166429302	NA	
-0.724273149	0,46889805816131	NA	

0.867772056	0,385519130694876	NA
0.342796447	0,731751598836906	NA
0.484103524	0,628312400123912	NA
-2.789615321	0,00527706994751532	NA
1.328809096	0,183910960479794	NA
0.450377477	0,652438282390242	NA
-0.564434868	0,572458214442177	NA
-0.932986671	0,350826850339649	NA
0.510280971	0,609854632384638	NA
0.447347238	0,654624365101436	NA
-0.662668947	0,507542602010388	NA
0.143892323	0,885585497669604	NA
0.417593509	0,676244343081462	NA
0.630225311	0,528547181442977	NA
-1.49387943	0,135207135798065	NA
0.201218465	0,840527754150715	NA
0.239112948	0,811018001824692	NA
-1.548095335	0,121599345141965	NA
-0.116870226	0,906962892784891	NA
-0.609854293	0,541958332546199	NA
1.177393927	0,239038312679718	NA
1.357539107	0,174609976256127	NA
1.738561966	0,0821118414304444	NA
-0.821786487	0,41119842568045	NA
0.029102725	0,976782662391906	NA
0.709783095	0,477838653939904	NA
-1.082688762	0,278946593821296	NA
0.967255498	0,333416324705741	NA
-1.425996254	0,153869423247431	NA
1.532638945	0,125364840224938	NA
0.582615793	0,560151971485801	NA
0.570370515	0,568426423576145	NA
-0.361519063	0,717711457015209	NA
0.369759635	0,711561593177275	NA
0.369584911	0,711691795248105	NA
-0.016386883	0,986925744311574	NA
1.100055587	0,271307902966368	NA
-0.040867248	0,967401727850653	NA
0.162411559	0,870981768605378	NA
1.501067049	0,133338220392621	NA
-0.409060407	0,682495331391924	NA
0.91222502	0,361650275715452	NA
0.12081242	0,903839612070927	NA
1.485783754	0,137336328757389	NA
-1.351483136	0,176540718527046	NA
1.841123693	0,0656034342906247	NA
1.300305047	0,193496438943896	NA
0.784763164	0,432592441131706	NA
1.038946873	0,298829444969622	NA
1.837788359	0,0660935976388418	NA

0.524776223	0,599738762642519	NA
1.323336389	0,185723535458652	NA
-0.284322985	0,776162873223613	NA
-0.31358872	0,753833428113753	NA
1.624926576	0,104178204615193	NA
0.385236963	0,700061860478814	NA
0.213106548	0,831243857043569	NA
-1.534223735	0,124974620539617	NA
0.488220594	0,6253935992438	NA
0.152846391	0,878519413184721	NA
-0.529341938	0,596568267850274	NA
0.112783881	0,910201897982782	NA
0.099136841	0,921029621371677	NA
1.286039101	0,198429415938974	NA
-1.485328053	0,137456944042643	NA
-0.65749553	0,510862344278642	NA
-0.031071965	0,975212147818998	NA
-0.927950629	0,353433180217271	NA
-0.30410109	0,76105087644548	NA
-0.935127445	0,349722624247524	NA
1.190620256	0,233802695995793	NA
1.275734028	0,202049555334585	NA
-1.343599825	0,179077826094068	NA
0.602305549	0,546970767560289	NA
1.124886443	0,260637157512313	NA
-0.402977207	0,686964992007041	NA
-0.905746501	0,365070043799901	NA
-1.26933887	0,204320228620711	NA
1.209257527	0,226563923006827	NA
-0.304237217	0,760947173080733	NA
0.848998892	0,39588190857185	NA
0.525387734	0,599313678342765	NA
0.482606116	0,629375435495547	NA
-0.333863825	0,738482318426656	NA
-1.540653029	0,123401254370294	NA
-1.884906533	0,0594424579540538	NA
1.771149512	0,0765358424353374	NA
0.287914154	0,773412451893287	NA
2.140696683	0,0322985061863268	NA
1.555436989	0,119842030571478	NA
-1.194155814	0,232417023601515	NA
1.294258763	0,195576055060655	NA
-0.147441943	0,882783201917089	NA
0.848676631	0,396061253417994	NA
1.3748371	0,16918195327634	NA
1.401999793	0,160915308521339	NA
-0.054855522	0,95625356653576	NA
0.543500766	0,586785063297489	NA
1.32307046	0,185811947210922	NA
-0.555802584	0,57834581876456	NA

0.19803583	0,84301703078748	NA
-2.263087351	0,0236303051486378	NA
0.518819969	0,603886290987341	NA
1.467722278	0,142179676879828	NA
-1.077795046	0,28112523107104	NA
1.182717825	0,236920997245004	NA
0.297416394	0,766148629031902	NA
-0.009180104	0,992675439513053	NA
-0.027761873	0,97785207507411	NA
0.706206939	0,480059455802333	NA
0.135332193	0,892349232967289	NA
-0.500268991	0,616885685628186	NA
-0.29139803	0,770746920686351	NA
2.675661999	0,00745818191829497	NA
-1.077838191	0,281105972797516	NA
-0.543976286	0,586457791850993	NA
0.767176239	0,442976738186052	NA
-0.062709202	0,949998069544954	NA
0.917732809	0,358758766548857	NA
-0.45793237	0,647001030417393	NA
1.455554119	0,145515919181979	NA
0.537418445	0,590978605304839	NA
1.678690062	0,0932124638059393	NA
-0.963926166	0,335082944265111	NA
0.143290078	0,886061091357519	NA
0.469608231	0,638634942830077	NA
1.167633849	0,242954490456038	NA
-0.792687557	0,427959881766342	NA
-1.688531688	0,0913092114111981	NA
0.130678805	0,896029399617926	NA
-0.186889034	0,851747637415678	NA
1.075469007	0,282164800825213	NA
-0.715200631	0,474485079184235	NA
-2.034216622	0,0419297593494466	NA
-0.076687876	0,938871849320355	NA
0.316925922	0,751299812586553	NA
0.032119771	0,974376536594444	NA
0.071112657	0,943308095159483	NA
1.265077097	0,205843692217581	NA
-2.460402819	0,0138781151631723	NA
-0.055817518	0,95548717953135	NA
-0.18899372	0,850097738613442	NA
-0.701911884	0,482734117371258	NA
-0.655002385	0,512466218199955	NA
0.24594671	0,805723484679873	NA
-1.51433417	0,129941106197377	NA
0.825137636	0,409293454741913	NA
-0.056774427	0,954724885737248	NA
-1.059813403	0,28922949792182	NA
-0.659789599	0,509388859060051	NA

0.13168311	0,895234945144293	NA
-2.00525941	0,0449353217396027	NA
0.352157073	0,724720467869465	NA
-0.595630964	0,551421786464794	NA
0.362939802	0,716649853396902	NA
0.36919798	0,711980161355979	NA
-0.592150827	0,553749593875575	NA
-0.190901956	0,848602407977045	NA
-0.141314191	0,887621739338082	NA
0.513462684	0,607627707402375	NA
-1.853154293	0,0638602467476871	NA
-0.391132745	0,695699117707782	NA
-0.896070137	0,370215305084171	NA
0.454779543	0,649267848653028	NA
1.763952543	0,0777399694198374	NA
1.630246551	0,103049398877317	NA
0.330163686	0,741276283957169	NA
0.07091197	0,943467816477086	NA
-0.746333936	0,455465716507632	NA
0.070155195	0,944070135927455	NA
0.879125284	0,379333349864598	NA
1.683538676	0,0922708587460322	NA
1.052688388	0,2924838324037	NA
0.780355886	0,435181426024433	NA
1.168035956	0,242792261611981	NA
-2.426493711	0,0152455145525325	NA
0.861076816	0,389195736900253	NA
-1.385357158	0,16594328424122	NA
1.360302749	0,173734138753549	NA
0.029854857	0,976182808345692	NA
-0.323913625	0,746003428254535	NA
-0.463131215	0,643270316448868	NA
-0.506248339	0,612682324612242	NA
0.54767467	0,583915307663806	NA
-0.861823179	0,388784827972497	NA
-0.549747611	0,582492496158416	NA
1.159104157	0,246413731447315	NA
-0.502100786	0,615596627521815	NA
1.295161922	0,195264377583556	NA
0.616150741	0,537795035482029	NA
-0.185293442	0,852998881173774	NA
0.404503972	0,68584215794347	NA
-0.492396078	0,622439371108593	NA
1.424849389	0,154200742274734	NA
0.916958091	0,35916460125709	NA
2.083104185	0,0372417279889911	NA
0.614855231	0,538650330579503	NA
0.015708294	0,987467110102904	NA
-0.077327879	0,938362712921674	NA
-0.183546604	0,854369155453264	NA

1.150099186	0,250103021493716	NA
-0.172675295	0,862906663656847	NA
0.586043116	0,557846540773684	NA
0.625641426	0,531550160698484	NA
-0.580029562	0,561894682479056	NA
0.275871107	0,782647066053267	NA
0.986215953	0,324027152605928	NA
0.055047171	0,956100883814925	NA
-0.235838533	0,813557966148875	NA
-0.067979003	0,945802348618043	NA
-0.490861021	0,623524747872931	NA
0.188672746	0,850349313327161	NA
0.288681813	0,772824881617701	NA
-1.323538933	0,185656217911759	NA
-0.637480431	0,523811952703419	NA
0.468496082	0,639429871852269	NA
0.603802536	0,545974933353661	NA
-0.466159851	0,641101085164059	NA
-1.108646259	0,26758281384629	NA
-0.29696243	0,766495192418885	NA
2.358510328	0,0183484491993858	NA
-0.627275466	0,53047868261023	NA
0.860647045	0,389432467005712	NA
0.17588577	0,860383684622434	NA
-0.146003525	0,883918605439133	NA
-1.055764099	0,291076004035453	NA
0.292078179	0,770226849913356	NA
0.386227606	0,699328110145664	NA
-1.135286628	0,256255243314116	NA
0.055775662	0,95552052327196	NA
1.311376381	0,189730634574697	NA
-0.829385897	0,40688607908661	NA
-0.366744391	0,713809690736444	NA
0.62193339	0,533985668240688	NA
1.039285711	0,298671878338708	NA
2.846608433	0,0044187677536558	NA
-0.834950542	0,403745555721029	NA
1.483512448	0,137938311784836	NA
0.843626108	0,398878370111137	NA
-0.341959543	0,732381338809996	NA
-0.262559715	0,792889953438039	NA
0.997007115	0,31876105618313	NA
-1.104718818	0,269281446940891	NA
-0.44441065	0,656745714956684	NA
0.15607633	0,875972855448018	NA
-0.404094648	0,686143120525415	NA
2.40124747	0,016339282420027	NA
1.018704261	0,308343389267658	NA
-0.499104704	0,617705624068536	NA
0.185160712	0,853102983781623	NA

-1.387388328	0,165323382098265	NA
-0.296825684	0,76659959553632	NA
-0.030380788	0,975763366781223	NA
1.618506363	0,105553516610725	NA
2.211734361	0,0269850248158044	NA
0.005963881	0,995241540029374	NA
-1.454506224	0,14580600904719	NA
1.63583121	0,101874918069249	NA
-0.457192602	0,647532615007369	NA
-0.821699015	0,411248219765717	NA
-0.294962221	0,768022739885427	NA
1.395212363	0,162951809923408	NA
-0.396465859	0,691761389033001	NA
0.636303422	0,524578674963234	NA
0.291179061	0,770914376044471	NA
-0.068289922	0,945554846443978	NA
1.349380225	0,17721486915341	NA
-0.200355624	0,841202462742106	NA
0.094485889	0,924723191617462	NA
-0.910131972	0,362752915089363	NA
0.211246418	0,832694991302462	NA
-0.516657574	0,60539521708901	NA
0.045788224	0,963479045045133	NA
1.934155652	0,0530939931465609	NA
-0.743854526	0,456964488621317	NA
0.750266165	0,453094416448229	NA
1.220981422	0,222093054030493	NA
-0.83025771	0,406393092717011	NA
0.033536004	0,973247155215365	NA
0.779622197	0,435613286668716	NA
1.538988761	0,123807040168689	NA
1.030183567	0,302923842418756	NA
0.082176857	0,934506076947129	NA
0.972333508	0,330884659256083	NA
-0.258300061	0,796175338866826	NA
-0.365305048	0,714883710859797	NA
-0.800354162	0,4235056310212	NA
-1.032435946	0,30186793972218	NA
0.508920651	0,610807844116368	NA
0.905944489	0,364965234676812	NA
-0.394739633	0,693035050409884	NA
0.321039708	0,748180299819659	NA
0.162608232	0,870826905373371	NA
-0.174479648	0,861488523996417	NA
-1.58148485	0,113767220182754	NA
-2.903660319	0,00368828082459331	NA
1.123878618	0,261064523158313	NA
-1.092813219	0,274475816248055	NA
-0.035959476	0,971314671732238	NA
-0.599154633	0,549069772387	NA

0.117921555	0,906129813652537	NA
1.649613991	0,0990219115037386	NA
0.059473587	0,952574902516288	NA
0.130913458	0,895843768616559	NA
-0.492273893	0,622525733689639	NA
-0.466545138	0,640825346353301	NA
-0.238422187	0,811553660706814	NA
0.445017985	0,656306756718499	NA
0.281103727	0,778630842504163	NA
1.388167441	0,165086064342231	NA
0.015383033	0,987726599447907	NA
-1.553093998	0,120400679752627	NA
1.474446462	0,140361422596819	NA
1.420347455	0,155506552037833	NA
0.260327008	0,794611540985764	NA
1.967522521	0,0491230021765301	NA
-0.060259447	0,951949000059066	NA
0.330007263	0,741394474506235	NA
-1.329928177	0,183541936505009	NA
-0.792525868	0,428054114607015	NA
0.031906855	0,974546332198144	NA
0.557886608	0,576921816626398	NA
-0.025633728	0,979549483859602	NA
-0.076731725	0,938836965804812	NA
-0.299033051	0,764914827397107	NA
2.150164605	0,0315421968257916	NA
2.398013597	0,0164842533337452	NA
0.887832571	0,37463081954872	NA
0.577064034	0,563896199488291	NA
-1.484637405	0,137639901300906	NA
2.045706435	0,0407852622143061	NA
1.60834244	0,107760200653509	NA
1.278495729	0,201074689614737	NA
0.216056797	0,82894347545014	NA
-1.684315912	0,0921206317588971	NA
0.882043789	0,377753127642509	NA
0.197894262	0,843127793289986	NA
0.122726202	0,902323914232626	NA
1.487756191	0,136815202815652	NA
1.504309189	0,132501774048043	NA
-0.33138811	0,74035134499075	NA
0.22936136	0,8185880656894	NA
-0.701059341	0,483265982879799	NA
-0.590419095	0,554909712224964	NA
0.634760563	0,525584587982843	NA
0.104051816	0,91712822836788	NA
-0.523872116	0,600367489975442	NA
-0.364920376	0,715170844080992	NA
-0.632391225	0,527131266136737	NA
-0.745445428	0,45600249000206	NA

-0.367190277	0,713477091167505	NA
0.102156993	0,918632063744401	NA
-0.724646991	0,468668622797946	NA
0.524541827	0,59990173575279	NA
0.878338894	0,379759834529406	NA
0.156010561	0,876024696484219	NA
-0.224816456	0,82212206409867	NA
-0.153810218	0,877759376803549	NA
0.905592998	0,365151316026376	NA
-0.842702847	0,399394654659668	NA
0.072618454	0,942109741093768	NA
0.238125994	0,811783374002031	NA
2.836441295	0,00456193640601206	NA
-0.654740077	0,512635117210692	NA
0.36329304	0,716385991641776	NA
0.223711581	0,82298173374596	NA
-0.083539185	0,933422821408389	NA
0.939837694	0,347300820645953	NA
-0.716848512	0,473467574280964	NA
-0.120135494	0,904375816056577	NA
0.630757397	0,528199163610093	NA
-0.966276102	0,333906041048651	NA
0.425592355	0,670404911657528	NA
0.990401147	0,321978085188741	NA
-1.994084115	0,046142862930104	NA
-0.390776222	0,695962652357027	NA
0.019193515	0,984686730571892	NA
0.031189211	0,975118644062626	NA
0.376033443	0,706892033723966	NA
0.865603938	0,386707395163513	NA
-0.312486264	0,754671000218152	NA
0.148905062	0,881628546477846	NA
0.60977969	0,542007757308978	NA
-0.031964767	0,974500148732447	NA
0.696381173	0,486190150236972	NA
0.718356091	0,472537753117258	NA
-0.374134452	0,708304287866337	NA
0.477449176	0,633042303863754	NA
0.220898171	0,825171721866228	NA
-0.854520538	0,392816634860994	NA
0.861813232	0,388790302478564	NA
-0.502557039	0,615275740631937	NA
-0.107355702	0,914506795843251	NA
0.070458809	0,943828484499186	NA
-0.335095053	0,737553384768909	NA
-2.419335904	0,0155488744677433	NA
0.370305571	0,711154823228797	NA
-2.418540436	0,0155829134939216	NA
-0.63296691	0,526755252089941	NA
1.220370416	0,222324486993817	NA

-1.22145335	0,221914418056932	NA
0.440384773	0,6596584511624	NA
0.685748262	0,492871867228466	NA
-0.097943728	0,921976976747962	NA
0.632220876	0,52724255795602	NA
0.993508733	0,320462096812606	NA
-1.331025837	0,183180509589028	NA
0.848685412	0,396056365949014	NA
-0.027600417	0,977980848912579	NA
2.085426732	0,0370305824180307	NA
-0.987861539	0,32322046404652	NA
-1.782171456	0,0747212761029268	NA
0.525518983	0,59922246053458	NA
1.733883124	0,0829388227149839	NA
0.008921626	0,99288166710437	NA
1.011866298	0,311601985455726	NA
-0.401705452	0,687900811513039	NA
0.604927746	0,545227006827052	NA
0.056435048	0,954995237353355	NA
-1.367824056	0,171367155987043	NA
0.537815906	0,590704149661617	NA
-0.487877636	0,625636516380427	NA
-1.161689554	0,245361590335376	NA
0.993180876	0,320621816105423	NA
-0.263659218	0,792042523350842	NA
-1.480226787	0,138812733921783	NA
2.497529079	0,0125062207504006	NA
0.849668655	0,395509329787311	NA
0.319419285	0,749408589132852	NA
0.008985505	0,992830700859429	NA
-0.947077016	0,343599535713603	NA
1.121697962	0,261990883372042	NA
0.396916073	0,691429350915954	NA
0.981177749	0,326505094691627	NA
0.158538521	0,874032468985529	NA
0.418996786	0,675218481083139	NA
0.205430837	0,837235529709416	NA
0.426792855	0,669530211388866	NA
-1.340518936	0,180076692303129	NA
-1.858648416	0,0630769895947167	NA
-0.270522208	0,786758534226332	NA
-0.889387597	0,37379480818889	NA
-1.120803744	0,262371410135041	NA
1.56195251	0,118299176941383	NA
0.910952966	0,362320156218824	NA
0.720019714	0,471512857901324	NA
0.390514645	0,696156028409135	NA
1.349616863	0,177138912335177	NA
-0.330507428	0,741016581277389	NA
-0.193581899	0,846503272814105	NA

1.52247654	0,127889719359028	NA
2.129929318	0,0331774492449925	NA
0.242878199	0,808099754482625	NA
-2.161332161	0,0306696902708808	NA
0.20384766	0,838472547699501	NA
-0.976416579	0,32885806892288	NA
0.980886722	0,326648605968071	NA
-0.997891674	0,318331889657513	NA
-1.484578797	0,137655435523948	NA
0.759532091	0,447534324733764	NA
-1.459656494	0,14438450436543	NA
1.12755709	0,259507017180948	NA
0.026041744	0,979224042831859	NA
-0.737016466	0,461112335760265	NA
0.576526456	0,564259393206352	NA
-0.145195683	0,884556374217472	NA
0.267910212	0,788768432751771	NA
-0.013946618	0,988872569363614	NA
1.597060374	0,110252250083243	NA
1.560396694	0,118666165537026	NA
-0.435128756	0,663468971748198	NA
0.972623081	0,330740667047064	NA
0.155655659	0,876304449842009	NA
-0.084476054	0,932677940692736	NA
-1.411943887	0,157966484038368	NA
0.639321627	0,522613722103143	NA
1.681355742	0,0926938367042962	NA
1.107206648	0,268204593752293	NA
1.280022094	0,200537365846701	NA
0.810136436	0,417861765376318	NA
1.020357298	0,307559038271822	NA
-0.575532675	0,564931098863861	NA
-1.806738891	0,0708030007334714	NA
0.217524155	0,827799884284797	NA
-0.81471998	0,415232613848618	NA
-0.502137423	0,61557085746008	NA
0.996055104	0,319223372302813	NA
-0.530771313	0,595577261633673	NA
0.986724359	0,323777785277281	NA
0.800255862	0,423562570238432	NA
-0.326168561	0,744296826391246	NA
1.151387196	0,249572979185277	NA
3.020610284	0,00252265821069258	NA
-1.319703867	0,186933908281916	NA
1.891821821	0,0585147249425964	NA
-0.981451504	0,326370138270554	NA
2.23628775	0,0253329283309919	NA
0.128146316	0,898033183280801	NA
1.33847793	0,180740689919343	NA
0.196738946	0,844031831277305	NA

-0.334002413	0,738377737948468	NA
-1.626428507	0,103858530576045	NA
-3.700948647	0,000214794946866872	NA
-0.396014505	0,692094327528214	NA
-2.04955612	0,0404077661350802	NA
0.071874844	0,942701509493973	NA
-0.185974221	0,852464977672735	NA
-0.296565252	0,766798442674223	NA
0.263854259	0,791892223270955	NA
0.03867118	0,969152551026336	NA
2.067997178	0,0386402884949696	NA
1.147031279	0,251368693541241	NA
-0.879668893	0,379038705677068	NA
0.223597492	0,823070515064896	NA
0.233162999	0,815634836461773	NA
-0.109771514	0,912590582961274	NA
0.379266698	0,704489827035411	NA
2.715981002	0,00660797098177718	NA
-0.819583714	0,412453460803762	NA
-0.189470352	0,849724189512319	NA
2.286532462	0,0222231272398887	NA
-0.153107093	0,878313822362716	NA
2.261719359	0,0237147478662633	NA
2.735563023	0,00622736602345	NA
0.597118918	0,55042798836717	NA
0.441985468	0,658499721574637	NA
0.5887861	0,556004771715345	NA
0.687431129	0,491811087159717	NA
-0.730585852	0,465032156358954	NA
-0.07614357	0,939304877015084	NA
-1.489542908	0,136344463132825	NA
-0.164793408	0,869106593126317	NA
0.757977977	0,448464166862889	NA
0.459588424	0,645811669987577	NA
-1.243838927	0,213558850275363	NA
0.285121065	0,775551391907888	NA
0.695624091	0,486664274772594	NA
-0.228392172	0,819341374795065	NA
1.24416391	0,213439243552605	NA
0.243952883	0,807267311257143	NA
0.57528167	0,565100816252799	NA
0.512367043	0,608394152135242	NA
-0.802512961	0,422256294314587	NA
-0.405304162	0,685253947535708	NA
1.199895607	0,230179886413233	NA
0.871766809	0,383335604959745	NA
-1.074699615	0,282509236149546	NA
0.32069902	0,748438490110366	NA
-0.858736943	0,390485663614706	NA
0.026490925	0,978865771573723	NA

-0.196891398	0,843912525290237	NA
-2.515354988	0,0118912584461995	NA
-0.20503311	0,837546256621006	NA
-1.093701569	0,274085886060794	NA
-0.172060073	0,863390301883783	NA
0.435972431	0,66285673227551	NA
-0.754971115	0,450266313709605	NA
-1.087133773	0,276977694048817	NA
-0.268568381	0,788261846674397	NA
-0.223541902	0,82311377532658	NA
2.31585238	0,0205663301628667	NA
2.198497938	0,0279136413274567	NA
0.085777836	0,931643026061408	NA
-0.557471054	0,577205630866295	NA
0.01483482	0,988163959969579	NA
-0.215542686	0,829344235776696	NA
2.317903846	0,0204545473840644	NA
-0.432890634	0,665094224884692	NA
1.536445522	0,124429149338635	NA
-0.183884846	0,854103793569054	NA
-0.835846315	0,403241367131488	NA
0.826489001	0,408526754715008	NA
-0.830393397	0,406316397729443	NA
-0.133020938	0,894176821252417	NA
0.731230668	0,464638270762705	NA
-0.388721812	0,697481945560702	NA
0.761327785	0,446461308788144	NA
1.5318904	0,125549482825376	NA
0.479279977	0,631739464315911	NA
-0.774692552	0,438521353238	NA
0.779567518	0,435645481289081	NA
-0.197305167	0,843588736518882	NA
0.277481412	0,781410476609717	NA
0.159532685	0,873249209315855	NA
-1.053729966	0,292006565609806	NA
0.795540707	0,426299041306072	NA
-0.812951202	0,416246036768169	NA
1.396786173	0,16247788155755	NA
1.46238128	0,143636756254327	NA
0.469574926	0,638658742045568	NA
1.383810403	0,166416516749017	NA
0.202520801	0,839509598233302	NA
-0.551155294	0,581527223640881	NA
-0.361110756	0,718016652953135	NA
0.758490758	0,448157243612627	NA
-0.525249634	0,599409665077646	NA
-1.007254488	0,313812494614762	NA
-0.592310949	0,55364238540672	NA
0.521143462	0,602266836292145	NA
1.291723529	0,196452906077483	NA

-0.87923093	0,379276077172931	NA
-0.68973204	0,490362713438308	NA
-0.270002647	0,787158217137214	NA
0.420647552	0,674012464847955	NA
-0.684250473	0,493817015432637	NA
-0.112255952	0,910620466218439	NA
-1.010783511	0,312120056699552	NA
-0.845760136	0,397686568130242	NA
0.275800177	0,782701547985323	NA
-0.401707405	0,687899373986929	NA
1.38956538	0,164660894763116	NA
-0.806004855	0,420240049906899	NA
-0.641145333	0,521428263054897	NA
0.025022035	0,98003738811207	NA
-0.733260474	0,463399577754361	NA
0.04599382	0,963315175715813	NA
-0.225653198	0,821471161786835	NA
-0.206672201	0,836265868835994	NA
-0.483728102	0,628578846467931	NA
0.433209542	0,664862547160999	NA
-0.920186891	0,357475103664362	NA
-0.732667466	0,463761271585852	NA
1.118240533	0,263464277055217	NA
-0.350348945	0,726076837485318	NA
-0.785434352	0,432198946243765	NA
-0.261362689	0,793812826994585	NA
-0.491036452	0,62340066637376	NA
-0.188681673	0,850342316231905	NA
2.206597549	0,0273421883799443	NA
-0.321496364	0,747834267391417	NA
-1.028699961	0,303620688180623	NA
-0.727438816	0,46695718312843	NA
0.977881472	0,3281329499028	NA
-1.416918696	0,15650669969327	NA
0.847252311	0,396854501639299	NA
-0.251374897	0,801524273928187	NA
-0.760079169	0,447207263503413	NA
0.223385599	0,82323541120732	NA
-0.156451837	0,875676880263997	NA
-0.109329015	0,912941534027879	NA
-0.927806325	0,353508042534888	NA
0.23338662	0,815461202074011	NA
-0.369214311	0,711967989979784	NA
0.842192985	0,399679939904978	NA
0.478294171	0,632440844785006	NA
-1.366508577	0,171779390735366	NA
-1.335733981	0,181636238393464	NA
-0.358314413	0,720108033258051	NA
0.38813948	0,697912817246265	NA
1.498107265	0,134105383933404	NA

-1.113155286	0,26564174273282	NA
-0.620242394	0,53509821460982	NA
0.608927646	0,542572398790998	NA
1.860551155	0,0628075880634799	NA
1.154613552	0,248248714604782	NA
-1.12996936	0,258489135493488	NA
-0.045028355	0,964084707659102	NA
0.059677045	0,952412854957751	NA
-2.292022692	0,0219043338115867	NA
-0.552883927	0,580342894160563	NA
-1.155141005	0,248032688857528	NA
-1.151748778	0,249424321829771	NA
0.254376564	0,79920465501295	NA
-0.78265758	0,4338282191283	NA
0.736962472	0,461145171420455	NA
1.826387411	0,0677919267496037	NA
2.385897385	0,0170375042390984	NA
0.040566872	0,967641195024116	NA
-0.739506055	0,45959976460636	NA
1.61761207	0,105746226288067	NA
0.363580458	0,716171321519198	NA
-1.124190685	0,260932140436376	NA
-0.185948505	0,852485144171684	NA
-0.005764741	0,995400427994489	NA
-0.857220854	0,391322839419196	NA
1.064180151	0,28724709711096	NA
-0.876987608	0,380493368972753	NA
-0.881115451	0,378255335108604	NA
-0.392790727	0,694474054352405	NA
0.331858727	0,739995936872788	NA
0.313690209	0,753756338119708	NA
0.094582954	0,92464609007026	NA
1.738641269	0,0820978826283747	NA
1.700495707	0,0890377232608823	NA
1.305498084	0,191723299221355	NA
0.374252084	0,708216777251291	NA
-0.301911573	0,762719473264525	NA
-0.242902498	0,808080930183562	NA
0.869765476	0,384428581967437	NA
0.088657651	0,929353989374834	NA
1.016488135	0,309396994988462	NA
0.052133299	0,95842247996303	NA
1.356789663	0,174848052934876	NA
-0.018141099	0,985526290904385	NA
-1.70017099	0,0890987673121299	NA
-0.438567129	0,660975219447217	NA
1.247239137	0,21230982661248	NA
0.398247866	0,690447485910935	NA
1.245448463	0,212966948553091	NA
0.542816327	0,587256270409703	NA

0.364399121	0,715559991802311	NA
-0.194764251	0,845577510151141	NA
-0.677633439	0,498004139005315	NA
0.977702897	0,328221288430555	NA
0.203837245	0,838480686639074	NA
0.464781064	0,642088249758153	NA
-0.423759651	0,671741107467438	NA
-0.180158573	0,857028079833276	NA
-1.538664347	0,12388626064481	NA
-1.384140206	0,166315527787345	NA
0.741504783	0,458387432761649	NA
-0.089487531	0,928694462257159	NA
0.400939805	0,688464443095939	NA
0.482493624	0,629455326658356	NA
1.230554253	0,218489623894352	NA
-0.845989046	0,397558854876763	NA
1.050836134	0,293333856965723	NA
-0.454192105	0,649690565171535	NA
0.012118628	0,990330970424525	NA
-0.018852816	0,984958519894853	NA
2.470298489	0,0135000354424459	NA
0.722226465	0,470155254489295	NA
0.614217864	0,539071370385481	NA
-0.737692547	0,460701301987603	NA
0.885611574	0,375826872439752	NA
1.0186051	0,308390482104422	NA
-0.964255254	0,334917968732519	NA
-0.106976613	0,914807533583329	NA
1.470060787	0,14154529137266	NA
-0.507578349	0,611749077321711	NA
0.561573344	0,574406745773096	NA
-1.132692836	0,257343267315675	NA
1.504896507	0,132350686547462	NA
0.15739128	0,874936485048341	NA
2.043130888	0,041039482981255	NA
0.379910387	0,704011936924892	NA
0.451329821	0,651751855216022	NA
0.435046336	0,663528794858364	NA
-0.306096361	0,759531278345237	NA
0.695959079	0,486454456931476	NA
0.50032723	0,616844683808857	NA
0.868739718	0,384989512011825	NA
0.125083138	0,900457732146001	NA
-0.437715576	0,661592476945189	NA
-0.121393724	0,903379187759604	NA
0.112317956	0,910571304996834	NA
-0.631940838	0,527425536897632	NA
2.444551397	0,0145032414022641	NA
0.330364702	0,741124409063514	NA
0.766534164	0,443358531966341	NA

0.903638845	0,36618693652744	NA
-3.036220866	0,00239563809129919	NA
1.532402591	0,125423118530793	NA
-0.453358602	0,650290541159422	NA
-0.169248791	0,865600954003233	NA
-0.394660127	0,693093733030176	NA
-1.375332648	0,169028339095253	NA
2.198936216	0,0278824580243043	NA
0.573815836	0,566092431643231	NA
0.760245009	0,447108145938799	NA
0.638989994	0,522829441410975	NA
-0.21957281	0,826203869362985	NA
-0.038614253	0,96919793827852	NA
-0.81366308	0,415837990124448	NA
0.421944871	0,67306525719913	NA
0.59113038	0,554433065967421	NA
0.34224131	0,732169298936678	NA
-0.743085074	0,457430175801864	NA
-1.223853481	0,22100750498932	NA
0.080749194	0,935641413112515	NA
1.210245393	0,226184744442637	NA
-0.596550262	0,550807687166629	NA
0.76373005	0,445028129200599	NA
0.705020459	0,480797504297858	NA
-0.247885646	0,804222886032345	NA
0.347304963	0,728362222341568	NA
2.260729826	0,023775992333311	NA
-0.534981801	0,592662441467924	NA
1.108754438	0,267536130404369	NA
-0.737741138	0,46067176868813	NA
0.247410095	0,804590862001443	NA
1.17674429	0,2392975844299	NA
0.380689061	0,703433986770684	NA
-0.127416798	0,898610521949614	NA
1.552982077	0,12042741654608	NA
1.564334263	0,117739089682413	NA
2.125046734	0,033582713514183	NA
-0.300749357	0,763605628590248	NA
2.810339993	0,00494891898164147	NA
0.239698185	0,810564241787728	NA
-1.615214647	0,106264219742243	NA
1.585095318	0,112944679170473	NA
1.160962569	0,245657121867225	NA
-1.196102465	0,231656575715182	NA
0.346618827	0,728877700017527	NA
0.60861984	0,542776450863113	NA
-0.552108942	0,580873715913349	NA
1.739886265	0,081878990852685	NA
-0.100588322	0,919877266988603	NA
-1.473007518	0,140749008704624	NA

0.440195397	0,659795593470058	NA
-0.442077935	0,65843281068727	NA
0.34316216	0,731476470196145	NA
-0.719504178	0,471830328664641	NA
-0.571692703	0,567530178889677	NA
-0.112178969	0,910681504059762	NA
0.09886343	0,921246705362185	NA
0.478192489	0,632513208167508	NA
1.551046663	0,120890501857961	NA
0.382710841	0,701934175293576	NA
0.021684351	0,982699747278702	NA
-0.023342759	0,981376864175191	NA
1.108872359	0,267485249689986	NA
-0.968666111	0,332711806157704	NA
-1.522520124	0,127878807028196	NA
0.090057366	0,928241628200044	NA
-1.710699244	0,0871366473890818	NA
-0.614570567	0,538838357164196	NA
-0.168874005	0,865895746607967	NA
0.354349471	0,723076997176536	NA
2.264590714	0,0235378072739761	NA
-0.805662735	0,420437342125046	NA
-0.22634536	0,82093282127181	NA
-0.398609549	0,690180924163313	NA
-0.651734845	0,514572239773598	NA
-0.281891923	0,778026382871515	NA
-1.129645589	0,258625593146096	NA
0.884119301	0,376631814892525	NA
1.390345378	0,164424024570054	NA
-0.564141017	0,572658165317876	NA
1.188165508	0,234768209776474	NA
-0.069341406	0,944717868042802	NA
1.861748131	0,0626386008343063	NA
-1.106761123	0,2683972210836	NA
0.288742621	0,772778344103634	NA
-2.143215471	0,0320958005392427	NA
0.641573879	0,521149897909737	NA
-1.80261127	0,0714492859040406	NA
1.758650635	0,0786368669372013	NA
1.1639385	0,244448936029667	NA
0.371910466	0,709959512761414	NA
0.319686528	0,749205973528695	NA
-0.331581524	0,740205272920899	NA
-0.248481636	0,80376177703234	NA
-0.409411292	0,682237854515226	NA
0.445134514	0,656222547946964	NA
-1.313463247	0,189026897432953	NA
-0.973014107	0,330546290986557	NA
0.940172943	0,347128858293932	NA
-0.249808215	0,802735666097879	NA

1.327855014	0,184226008705162	NA	
0.959333421	0,337390803877464	NA	
-0.819102257	0,412728074064574	NA	
-2.051481846	0,0402200458069834	NA	
-0.143700389	0,885737064351279	NA	
-0.824627666	0,40958300944315	NA	
0.250700414	0,802045740878204	NA	
0.19113298	0,84842140946458	NA	
0.612807871	0,540003386091437	NA	
0.935389103	0,349587810682022	NA	
-0.565586647	0,571674807310791	NA	
0.053758201	0,957127811908437	NA	
0.236093228	0,813360328387794	NA	
0.199171415	0,842128658854273	NA	
0.486599927	0,626541875649855	NA	
-0.970104984	0,331994165012665	NA	
0.539559475	0,589500870539563	NA	
-0.310236121	0,756381403793708	NA	
1.137407916	0,255367799614785	NA	
-0.246669924	0,805163684963579	NA	
1.211916307	0,225544419114415	NA	
-0.742615985	0,457714207767056	NA	
-0.30013707	0,764072604338961	NA	
-0.672966416	0,500968656878945	NA	
1.138147121	0,255059054728577	NA	
-0.873275083	0,382513158625346	NA	
-0.562764987	0,573594925413067	NA	
-1.11049576	0,266785454199534	NA	
1.433021603	0,151851663617033	NA	
-0.768610231	0,442124730004498	NA	
1.418065769	0,156171564024992	NA	
0.986494746	0,323890392325155	NA	
-1.03384777	0,301207335487759	NA	
-0.94954742	0,342342266277931	NA	
0.323596064	0,746243868348926	NA	
-0.143869574	0,885603462513772	NA	
-0.070463087	0,943825080130152	NA	
0.107855146	0,91411059747988	NA	
0.799987586	0,423717989506889	NA	
0.673557792	0,500592495286838	NA	
0.061859371	0,950674822424123	NA	
1.602572516	0,109029065628011	NA	
0.880386653	0,378649884350448	NA	
0.823859378	0,410019464487586	NA	
-0.833844376	0,404368685145957	NA	
1.479520716	0,139001198858685	NA	
-0.51214744	0,608547824970617	NA	
-0.288020164	0,773331303858833	NA	
0.878497613	0,379673732634625	NA	
1.8207769	0,0686407770272904	NA	

-0.387141894	0,698651164299732	NA
-0.651686728	0,514603286264552	NA
0.632656502	0,526957981195036	NA
-0.022403073	0,982126429270358	NA
0.994142783	0,32015335905304	NA
-0.766562505	0,443341675480169	NA
1.84918544	0,0644310404660839	NA
-1.481344713	0,1385147393734	NA
0.927871628	0,353474163203136	NA
0.365112887	0,715027141443422	NA
-1.003694406	0,315525934071508	NA
-1.607844507	0,107869237988938	NA
-0.81002645	0,417924973975711	NA
1.724183068	0,0846747997957168	NA
-0.789841621	0,42962026839525	NA
0.122493782	0,902507970292692	NA
0.876696024	0,38065176672186	NA
0.540980195	0,588521233964001	NA
1.686220471	0,0917533424438365	NA
0.292296949	0,770059591310986	NA
-0.413268161	0,679410154011605	NA
0.131046068	0,895738864857966	NA
0.191105854	0,848442661378108	NA
0.433659964	0,664535383388894	NA
0.152732948	0,878608876649553	NA
0.264686262	0,791251161659044	NA
0.970009649	0,33204168269717	NA
2.469067208	0,0135465773485468	NA
1.048503486	0,294406697005057	NA
-0.404710403	0,685690394680932	NA
-0.997599839	0,318473439368386	NA
0.419818942	0,674617726039368	NA
-0.3713892	0,710347668510746	NA
0.394318662	0,693345786734081	NA
-0.532426896	0,59443036091027	NA
-1.496017934	0,13464898181791	NA
0.420817845	0,673888099648351	NA
0.885153464	0,376073867401997	NA
0.251872982	0,801139243540959	NA
-0.383090516	0,70165265124604	NA
1.007541977	0,313674396095343	NA
0.575315941	0,56507764265992	NA
-1.089853552	0,275777659933383	NA
0.691590138	0,489194756915462	NA
0.493260966	0,621828205038348	NA
0.995542422	0,319472522674619	NA
-0.344574528	0,730414258135213	NA
-0.025052041	0,980013454168862	NA
-0.763044746	0,445436710734262	NA
-0.588853612	0,555959478140688	NA

0.965992916	0,334047725592694	NA
0.987398446	0,323447345972921	NA
1.330750546	0,183271105271724	NA
-0.090339021	0,928017812876122	NA
0.298492091	0,765327611369444	NA
0.234817066	0,814350721188966	NA
0.211869539	0,832208815724538	NA
1.299427219	0,193797355635908	NA
-1.680721247	0,0928170717000197	NA
1.070193516	0,284532212312159	NA
-2.449794236	0,0142937871716891	NA
1.289761837	0,197133362139972	NA
0.136292945	0,891589700912016	NA
-0.861915689	0,38873391512402	NA
1.060020896	0,289135093030466	NA
-0.235722194	0,813648246465391	NA
-0.211425559	0,832555214390018	NA
-0.858178081	0,390794137408777	NA
0.328492134	0,742539590878153	NA
1.542144033	0,123038596720894	NA
0.02292453	0,981710473561008	NA
-0.703009897	0,482049581808207	NA
0.84819422	0,396329815852841	NA
-1.707641779	0,0877028312669413	NA
-0.336156818	0,736752614305939	NA
-0.168414301	0,866257357926785	NA
1.346292301	0,178208267829948	NA
-0.975878336	0,329124759061517	NA
0.458425496	0,64664677744477	NA
-2.172196785	0,0298408231253377	NA
1.105152799	0,269093385501156	NA
0.485817042	0,627096890065199	NA
-0.957120366	0,338506505177889	NA
2.629740067	0,00854501758029912	NA
2.621969736	0,00874231939162341	NA
0.294101035	0,768680700095155	NA
-0.655856562	0,511916419292843	NA
-0.106525797	0,915165189170563	NA
0.40593102	0,6847932849774	NA
-4.26801818	1,97217258781081e-05	NA
0.847737521	0,396584165547479	NA
0.507894498	0,61152733320124	NA
-0.160477598	0,872504867439934	NA
-0.084158668	0,932930279557962	NA
-1.998280771	0,0456862289584156	NA
0.782476307	0,433934704300824	NA
0.079253093	0,936831315526923	NA
0.82791819	0,407716832659906	NA
-1.914833559	0,0555137331940761	NA
0.244623223	0,806748181064226	NA

-0.751089022	0,452599082021409	NA
-2.044107113	0,0409429666625152	NA
-0.232987265	0,815771294497584	NA
-1.783727155	0,0744680113367088	NA
-1.748585224	0,0803627422824082	NA
-8.053704451	NA	NA
0.97865573	0,327750113001544	NA
-0.335848398	0,736985191564874	NA
0.883302231	0,377072998696054	NA
-0.321436717	0,747879461746327	NA
-1.417086625	0,156457602532857	NA
-1.691597638	0,0907227171275383	NA
-0.049239296	0,960728595810217	NA
-1.017829015	0,308759220419927	NA
0.309796815	0,756715473954915	NA
0.344521243	0,730454323242824	NA
0.004425309	0,996469125791068	NA
1.105351643	0,269007248509632	NA
0.057444653	0,954190993988829	NA
-1.107181128	0,268215625019091	NA
-1.401014403	0,161209766124614	NA
-1.895625044	0,0580096432890745	NA
1.517504751	0,129139294970219	NA
-0.171189401	0,864074841626758	NA
-1.129549501	0,258666099956065	NA
-0.353715002	0,723552478522098	NA
0.893662625	0,371502428036442	NA
0.792534043	0,428049350063361	NA
-0.318846888	0,749842621302593	NA
1.549621144	0,121232474861662	NA
-1.555108666	0,119920191501433	NA
0.279783553	0,779643571158354	NA
-1.17240735	0,241033550083271	NA
-0.125482843	0,900141306791367	NA
0.718443802	0,472483687212661	NA
-0.026565989	0,978805900156044	NA
-2.094256374	0,0362371483276836	NA
0.11767872	0,906322228528292	NA
-0.877633583	0,380142598429064	NA
-2.433637958	0,0149479379168006	NA
0.496987172	0,619198097456501	NA
-0.248154979	0,804014498515708	NA
-0.210677544	0,833138898712239	NA
-0.322509648	0,747066630238884	NA
0.762900213	0,445522909024253	NA
-0.99900908	0,317790292685428	NA
1.46827797	0,142028732456201	NA
0.233242141	0,815573383918996	NA
0.602113716	0,547098445405421	NA
0.021606568	0,982761794516976	NA

0.689520096	0,490496031476345	NA
0.468616512	0,639343772284245	NA
2.146012545	0,0318719778568766	NA
0.642677517	0,520433372282467	NA
0.864847804	0,387122329107718	NA
0.876048844	0,381003480970541	NA
-0.02609044	0,979185201859843	NA
-0.124874077	0,900623241393729	NA
-1.055520352	0,291187406460795	NA
1.023530429	0,306057120112636	NA
1.154577635	0,24826342961341	NA
0.592379572	0,55359644246824	NA
1.441092519	0,149558546714208	NA
1.233113311	0,217533491988321	NA
1.442639674	0,149122001119256	NA
1.205750947	0,227913534505491	NA
-0.139061814	0,889401302660912	NA
0.887853129	0,374619760028852	NA
-0.399368937	0,689621379040821	NA
-0.948487916	0,342881121928358	NA
0.532118175	0,594644149865899	NA
-0.344592124	0,73040102748166	NA
-0.774121728	0,438858809786205	NA
0.3573334	0,720842229053569	NA
0.628588538	0,529618466250605	NA
0.521091497	0,602303033903628	NA
-0.924148729	0,355408885381783	NA
-0.818824074	0,412886793497381	NA
-0.462736766	0,64355306117813	NA
-0.178458409	0,858362981404078	NA
-1.369089208	0,170971391300736	NA
-1.01815633	0,308603668821637	NA
-0.764203797	0,444745804435663	NA
1.929756248	0,0536370469201902	NA
0.444486728	0,656690722583821	NA
0.18439691	0,853702093959048	NA
-0.200605158	0,841007324229297	NA
2.159553616	0,030807242468438	NA
-0.680232941	0,496356977913313	NA
0.768290814	0,442314430992747	NA
-1.587434998	0,112414157993915	NA
-0.490783719	0,62357942643414	NA
0.65072365	0,51522489341574	NA
0.618625742	0,536162939839307	NA
-0.857977282	0,390905008172877	NA
2.010752623	0,0443515926161937	NA
0.888498679	0,374272566150291	NA
0.246859919	0,805016636359708	NA
-0.661650058	0,508195518176138	NA
-1.25478766	0,209555853453613	NA

0.671058422	0,50218331286667	NA
0.806420846	0,420000230894912	NA
-0.268829362	0,788060997290597	NA
-1.339284415	0,180478100384053	NA
0.202654037	0,839405451100218	NA
0.978572827	0,32779109073481	NA
0.457035409	0,647645594598982	NA
-0.350692558	0,725819009378065	NA
-0.868339426	0,385208544800354	NA
-1.797742792	0,0722177767568265	NA
1.858806915	0,0630545119719095	NA
-0.848736161	0,396028120086444	NA
1.136782613	0,255629173469237	NA
1.010214199	0,312392678009255	NA
1.123675119	0,261150875245493	NA
1.873077747	0,0610576497302251	NA
-0.316209315	0,751843637905043	NA
1.656443677	0,0976320020963231	NA
1.438365137	0,150330479207729	NA
-0.165906337	0,868230660675646	NA
2.008460976	0,044594328935224	NA
1.703793251	0,0884197192350993	NA
0.256051087	0,797911389907002	NA
-0.806759888	0,419804833050415	NA
-0.19304555	0,846923294832661	NA
0.581151188	0,561138561788849	NA
2.361741681	0,0181893115594113	NA
1.060154559	0,289074290199692	NA
0.079077687	0,93697083088534	NA
-0.356629221	0,721369399058614	NA
0.203703676	0,838585069519675	NA
-0.224168603	0,822626113323683	NA
-0.633360518	0,526498241631259	NA
-0.30369914	0,761357113073009	NA
3.53690539	0,000404844662017725	NA
3.336159992	0,000849442631585867	NA
3.077641618	0,0020864567270726	NA
3.005550332	0,00265100717421599	NA
-2.97341524	0,00294505733840602	NA
-2.940122947	0,00328082036279602	NA
2.935167279	0,00333367916605452	NA
-2.891596006	0,00383290465497924	NA
-2.868599214	0,00412293868623379	NA
2.845285088	0,00443716903299249	NA
-2.698337234	0,00696868065410576	NA
-2.684801157	0,0072572968368476	NA
2.577906252	0,0099400961356888	NA
-2.566653124	0,0102685276380716	NA
-2.558488411	0,0105128324199554	NA
2.530422814	0,0113925148012732	NA

-2.527618442	0,0114839081391289	NA
2.460570445	0,0138716336984509	NA
-2.45609577	0,0140455720545483	NA
2.437615572	0,0147844873717725	NA
-2.430208859	0,0150901242111702	NA
-2.398627554	0,0164566437597212	NA
2.387776439	0,0169506494395216	NA
-2.380875079	0,0172715679057022	NA
-2.376475293	0,0174789319264114	NA
2.329966235	0,0198079357430687	NA
-2.293671641	0,021809366868497	NA
-2.285867018	0,0222620395214361	NA
2.285847718	0,0222631689269703	NA
-2.283636344	0,0223929120000573	NA
2.27056715	0,023173193944626	NA
-2.250967239	0,0243876124186251	NA
-2.249421617	0,0244856845779231	NA
2.24933864	0,0244909592435388	NA
2.240401335	0,0250648797011268	NA
2.222452387	0,026252748330464	NA
-2.212099563	0,0269597862943123	NA
2.209761009	0,0271217531071717	NA
-2.209746875	0,0271227345566375	NA
-2.204558622	0,0274850820521661	NA
-2.182724848	0,0290560789038549	NA
-2.179881571	0,0292662413499587	NA
2.151835609	0,0314103041560588	NA
2.135463557	0,0327231638860469	NA
-2.117676969	0,0342024346834245	NA
2.109901396	0,0348668499645203	NA
2.108508562	0,0349870229626376	NA
2.105707587	0,035229760386868	NA
2.105436657	0,0352533157392232	NA
-2.095706358	0,0361082474981729	NA
-2.09230598	0,0364111531145788	NA
-2.075758719	0,0379162721033095	NA
-2.062193047	0,039189360441096	NA
-2.060838172	0,0393184814497724	NA
2.050603159	0,0403056084900894	NA
2.039430304	0,0414071005148032	NA
-2.036361799	0,0417140384491718	NA
-2.033416858	0,0420104256083445	NA
2.032152718	0,0421381978605486	NA
-2.024612076	0,0429072133005656	NA
2.024204658	0,0429490985236666	NA
-2.021938181	0,0431827387200098	NA
2.01118117	0,0443063239632881	NA
-1.987920652	0,0468204662704131	NA
-1.979423515	0,0477683431461563	NA
-1.97291494	0,0485052541895494	NA

1.956514243	0,0504046063292736	NA
-1.932577432	0,0532882748161212	NA
-1.93248569	0,0532995866261798	NA
1.90891329	0,0562732783213234	NA
1.903308445	0,0570003096030589	NA
1.895141062	0,0580737159476259	NA
-1.894714237	0,0581302705972725	NA
-1.891334035	0,0585797681471488	NA
-1.886163626	0,059272908508787	NA
1.875655801	0,0607025587851302	NA
-1.874955926	0,0607987871745299	NA
-1.863742601	0,062357859794133	NA
1.863102547	0,0624478399323679	NA
1.861426695	0,0626839436671739	NA
1.857939673	0,0631775817248277	NA
-1.851070034	0,0641594784421246	NA
1.839988799	0,0657698818248285	NA
-1.833093543	0,066788661244652	NA
1.832654971	0,0668538976407524	NA
1.826244535	0,0678134357740493	NA
-1.824741715	0,0680400151216932	NA
-1.82453801	0,0680707754244255	NA
1.82044693	0,0686909711152625	NA
1.8159372	0,0693800060735195	NA
-1.812073304	0,0699748706727373	NA
-1.788662342	0,0736692112163793	NA
1.786056782	0,0740900647211855	NA
-1.778492438	0,075323012877833	NA
1.778009834	0,0754022398584896	NA
1.758187629	0,0787155894851365	NA
1.755489403	0,0791756317084881	NA
-1.754954774	0,0792670439395576	NA
-1.745222451	0,0809461547342216	NA
-1.742861918	0,0813577374778485	NA
-1.739346113	0,0819739007763378	NA
1.721497773	0,0851605359511953	NA
1.716854571	0,0860057471147211	NA
-1.714711986	0,0863980449163701	NA
1.711961275	0,0869038052647545	NA
1.710748141	0,0871276165043041	NA
-1.708101888	0,0876174387478737	NA
1.708035149	0,0876298206647844	NA
1.707329371	0,0877608499399057	NA
1.705287242	0,0881408658155842	NA
-1.685801282	0,0918340807999454	NA
-1.681567233	0,0926527888907952	NA
-1.6814862	0,0926685147089052	NA
-1.669963144	0,0949266557672691	NA
-1.663075693	0,0962972847244921	NA
-1.658101855	0,0972969095992696	NA

1.656962565	0,0975270437719322	NA
-1.642216495	0,100545145538735	NA
-1.633867335	0,102286709893572	NA
1.632680363	0,102536239446737	NA
-1.625126136	0,104135685013378	NA
-1.620610873	0,105101118839043	NA
1.619793569	0,105276628158637	NA
1.612441952	0,106865801974507	NA
1.6045438	0,108594237998029	NA
1.596586806	0,110357842002885	NA
-1.594538752	0,110815418305597	NA
1.58575578	0,112794720387504	NA
-1.581422365	0,113781497165381	NA
1.579487388	0,114224307131105	NA
-1.576928055	0,114812080427427	NA
-1.563254541	0,117992736218178	NA
-1.563165507	0,118013670999161	NA
-1.561305439	0,118451700871444	NA
-1.559523189	0,118872600388711	NA
1.558868613	0,119027480442962	NA
-1.537249654	0,124232185044087	NA
-1.533654105	0,125114770103524	NA
1.513536164	0,13014352235801	NA
1.510018727	0,131038645690459	NA
1.497311082	0,134312332708151	NA
-1.4925968	0,135542761405253	NA
1.488180508	0,136703296171492	NA
-1.484546483	0,137664001200765	NA
1.478948312	0,139154129647915	NA
-1.471756448	0,141086658148823	NA
-1.468310805	0,142019817280321	NA
1.464435473	0,143075001844031	NA
-1.462478131	0,143610232874762	NA
-1.457794699	0,144897139746444	NA
1.456293771	0,145311426768037	NA
1.452657007	0,14631900963272	NA
-1.446923331	0,147918397944412	NA
-1.446469041	0,148045689222212	NA
1.446195124	0,148122480912169	NA
1.441980471	0,14930788292812	NA
-1.43818074	0,150382778583349	NA
1.434582102	0,151406217707749	NA
1.431617231	0,15225339564362	NA
-1.431546497	0,152273650951784	NA
-1.42687454	0,153616060545746	NA
1.418238575	0,156121123205679	NA
1.414325244	0,157266428187801	NA
-1.414017858	0,157356659253462	NA
1.404237037	0,160248276406691	NA
1.40326356	0,160538260240734	NA

1.39544753	0,162880927023037	NA
1.391890561	0,163955540662799	NA
-1.377883411	0,168239287673908	NA
1.374936071	0,169151265095511	NA
-1.373321079	0,169652552394584	NA
1.370899502	0,1704062865872	NA
1.366053747	0,171922094197968	NA
-1.364920575	0,172278014649765	NA
1.361007837	0,17351121273299	NA
1.354819803	0,175474975532049	NA
1.352743914	0,176137456123039	NA
-1.350254193	0,176934460081342	NA
1.349064649	0,177316201719251	NA
-1.346762562	0,178056715791521	NA
1.346542201	0,178127720049784	NA
1.345934196	0,178323739361598	NA
-1.345092443	0,178595383207986	NA
1.343654538	0,179060124795511	NA
1.341415066	0,179785728643157	NA
1.329468371	0,18369349394203	NA
1.329052702	0,183830583093413	NA
1.328511713	0,184009116816165	NA
1.325320266	0,185064949951424	NA
1.322934506	0,185857158928959	NA
1.322887341	0,185872845568877	NA
-1.322259668	0,186081698080962	NA
-1.321366838	0,186379077689759	NA
-1.320277727	0,186742309223309	NA
1.31879039	0,187239197755427	NA
-1.316116429	0,188134967641989	NA
1.316111891	0,188136490349887	NA
-1.314524007	0,188669924644566	NA
1.311209965	0,189786836640146	NA
1.311048207	0,18984147752815	NA
1.309739916	0,190283835332787	NA
1.308761695	0,190615085924384	NA
1.308518935	0,190697356618815	NA
-1.297871784	0,194331397222215	NA
1.290546878	0,196860846694259	NA
-1.28503801	0,198779001879022	NA
-1.282205954	0,199770408242628	NA
-1.275109855	0,202270361806569	NA
1.273338637	0,202897902052591	NA
1.272587368	0,203164503481548	NA
1.270549636	0,203888913837582	NA
1.268919234	0,204469871420837	NA
-1.268884692	0,20448219284742	NA
-1.265157035	0,205815040625773	NA
1.264577576	0,206022795793393	NA
-1.264433921	0,206074324247713	NA

-1.264021452	0,20622232739388	NA
1.263849835	0,206283930273563	NA
-1.260805688	0,207378863888499	NA
1.260369157	0,207536222652774	NA
1.259192827	0,207960691664824	NA
-1.25783832	0,208450233927967	NA
1.257788486	0,208468260763598	NA
1.236801792	0,216160676600977	NA
1.234067158	0,217177880369701	NA
-1.231042942	0,218306804090666	NA
1.230808805	0,218394381557052	NA
-1.22791197	0,219480015533667	NA
-1.227322029	0,21970157891744	NA
-1.227014765	0,219817041344029	NA
1.219666126	0,222591467766921	NA
1.214002411	0,224746803522738	NA
1.206761318	0,227524077040023	NA
-1.204672466	0,228329769562932	NA
1.203186614	0,228904113583619	NA
1.200178115	0,23007017297309	NA
1.200094733	0,230102550860926	NA
-1.198825009	0,230595995991548	NA
1.19851991	0,230714676503388	NA
1.196288001	0,231584189631395	NA
-1.192906806	0,232905873437125	NA
-1.191707652	0,233375896499143	NA
-1.18496757	0,236030269351243	NA
-1.184433904	0,236241346006929	NA
1.180268393	0,237893484740285	NA
1.177607514	0,238953113023124	NA
1.177084879	0,239161629933589	NA
-1.174119368	0,240347216703044	NA
1.173619252	0,240547566456198	NA
1.171086196	0,241564131896413	NA
1.164078951	0,244392018001686	NA
1.163637236	0,244571054451875	NA
-1.163541229	0,244609980584527	NA
-1.15550797	0,247882470812522	NA
1.151684476	0,249450753880278	NA
1.150890197	0,249777411435932	NA
-1.150441167	0,249962212716766	NA
-1.149809548	0,250222321780461	NA
-1.147957525	0,250986098621842	NA
-1.147356918	0,251234138831838	NA
-1.14513019	0,252155231194043	NA
1.145127619	0,252156296197874	NA
1.144668603	0,252346462129387	NA
-1.142308012	0,253326012283741	NA
1.141458086	0,253679344568724	NA
1.13650302	0,255746102283609	NA

-1.134590259	0,25654703622959	NA
-1.126535014	0,259939128550094	NA
1.118159343	0,263498945189454	NA
1.114203944	0,265191703726569	NA
1.11403669	0,265263446660535	NA
1.107410298	0,268116575751683	NA
1.10223558	0,270359267269485	NA
1.102054232	0,270438095061459	NA
-1.09844465	0,272010374517595	NA
1.094989929	0,273521048683089	NA
-1.092115478	0,274782347126665	NA
-1.0890768	0,276120019801809	NA
-1.084274239	0,278243223877567	NA
1.07648698	0,281709520930246	NA
-1.069961925	0,284636447303392	NA
-1.066520442	0,28618844279265	NA
-1.062124431	0,288179200586123	NA
-1.060632287	0,288857043533267	NA
1.059562501	0,289343681029163	NA
1.059514958	0,289365320527704	NA
-1.057932373	0,290086274898761	NA
-1.056518564	0,290731364222009	NA
-1.055482357	0,291204774536602	NA
1.05489386	0,291473870794088	NA
1.052894488	0,292389352471727	NA
-1.051333006	0,293105672947843	NA
1.051115525	0,293205534755312	NA
-1.049926989	0,293751681783052	NA
-1.049151856	0,294108232928129	NA
-1.047693293	0,294779938654037	NA
-1.045935897	0,295590629475687	NA
1.040454458	0,298128812229152	NA
1.040408345	0,29815022644158	NA
-1.040187322	0,298252880661973	NA
-1.036850558	0,299805509244648	NA
1.032300088	0,301931559830972	NA
-1.029469999	0,303258870320373	NA
1.026547523	0,304633575363598	NA
1.025438912	0,305156135517829	NA
1.022460234	0,306563124303282	NA
-1.021333628	0,307096399303685	NA
1.020330164	0,30757190203379	NA
-1.019656808	0,307891252677281	NA
1.015742959	0,309751805151389	NA
1.011477524	0,311787933164655	NA
1.010041167	0,312475567458133	NA
-1.004363669	0,315203355197044	NA
1.003005807	0,315858059521413	NA
1.002507734	0,316098433272698	NA
-0.999156066	0,317719094786429	NA

-0.998194001	0,318185294949702	NA
0.997381394	0,318579419206852	NA
0.996996976	0,318765977811886	NA
-0.996405661	0,319053083443434	NA
0.996003605	0,319248393522467	NA
0.993963522	0,320240626726581	NA
-0.990135047	0,322108114894111	NA
-0.989949383	0,322198860410296	NA
-0.987017321	0,323634147430335	NA
0.9865907	0,323843331266516	NA
0.984194102	0,325020085094345	NA
-0.977645113	0,328249876935317	NA
0.976641651	0,328746591491502	NA
-0.975599435	0,329263004664463	NA
0.975445024	0,329339559317912	NA
0.975046614	0,329537138213857	NA
-0.971015014	0,331540802160332	NA
0.970022379	0,332035337226696	NA
0.967997195	0,333045770346652	NA
0.967620304	0,333234032945807	NA
-0.96668228	0,333702887849825	NA
0.966443677	0,333822217486475	NA
-0.964799417	0,334645288149706	NA
0.964263568	0,334913801090957	NA
0.963747733	0,3351724168509	NA
-0.962993987	0,335550540768648	NA
-0.962696909	0,335699648391402	NA
0.962122054	0,335988297081238	NA
0.959205075	0,337455444199014	NA
-0.956946204	0,33859440853363	NA
0.953961182	0,340103292080139	NA
0.951642715	0,341278210731774	NA
0.951038957	0,341584600398351	NA
-0.950876222	0,341667213529874	NA
0.950006033	0,342109187217346	NA
-0.94424139	0,345046308640608	NA
0.941663242	0,346365080194584	NA
-0.938240255	0,348120955280133	NA
0.936237059	0,349151146268619	NA
-0.934660078	0,349963507145366	NA
-0.934427279	0,350083531804246	NA
-0.934234023	0,350183188920824	NA
-0.933365163	0,350631461125566	NA
-0.924421293	0,355267012580012	NA
-0.923979145	0,355497173871908	NA
0.921026969	0,357036346686041	NA
-0.918946492	0,358123560401586	NA
0.917415504	0,358924951520861	NA
-0.915633427	0,359859192853963	NA
0.914197911	0,360612861852482	NA

-0.913870343	0,360784978842739	NA
0.913139768	0,361169037332634	NA
-0.909956581	0,362845408405482	NA
-0.909425519	0,36312555672346	NA
0.906635893	0,36459937447293	NA
-0.906161064	0,364850608078547	NA
-0.903764375	0,366120355891101	NA
0.902617127	0,366729133358772	NA
-0.901816913	0,367154133749666	NA
0.898970692	0,368668271666155	NA
0.898727962	0,368797579503799	NA
-0.898469108	0,368935507430679	NA
0.896472075	0,370000688260049	NA
-0.894739352	0,370926437068569	NA
-0.891881815	0,372456282961237	NA
-0.891529974	0,372644918534747	NA
0.886967153	0,37509658519463	NA
0.884393267	0,376483956292086	NA
0.878426053	0,379712551130834	NA
-0.878086529	0,379896762786916	NA
0.877424626	0,380256042030227	NA
-0.8763892	0,380818487648898	NA
0.875993112	0,381033778286089	NA
-0.873503785	0,382388543933436	NA
0.873006087	0,382659759713476	NA
-0.868003734	0,385392288676191	NA
0.867256437	0,385801519560379	NA
0.863780999	0,387708207859469	NA
-0.863222589	0,388015095991994	NA
-0.862004059	0,388685284120891	NA
0.861679855	0,388863714037679	NA
0.860109702	0,38972857361338	NA
-0.859844943	0,38987452144772	NA
-0.859737966	0,38993350160984	NA
-0.859535172	0,390045323805782	NA
-0.85865841	0,390529002266998	NA
-0.852798391	0,393771116465685	NA
-0.852678431	0,393837655120365	NA
-0.849908687	0,395375855659852	NA
-0.845683468	0,397729348123444	NA
-0.845567153	0,397794256018215	NA
0.844993506	0,398114465037689	NA
0.84299849	0,399229288127734	NA
0.842813795	0,399332591353252	NA
-0.842510117	0,399502479281406	NA
0.842495949	0,399510406231974	NA
-0.840912922	0,400396718760044	NA
-0.840016575	0,400899093180757	NA
-0.836279372	0,402997754799465	NA
0.83312424	0,404774664187901	NA

-0.831737861	0,405556925010368	NA
0.831490498	0,405696594089008	NA
0.831249676	0,405832597008167	NA
-0.83060806	0,406195079814572	NA
-0.828110346	0,407608010591852	NA
0.82801477	0,407662135041154	NA
0.826038948	0,408781998519206	NA
0.824546397	0,409629164261311	NA
0.823180604	0,410405297151463	NA
0.821049265	0,411618206967301	NA
-0.816849897	0,414014213578942	NA
-0.815954691	0,414526050106857	NA
0.815171529	0,414974132211255	NA
-0.812830068	0,416315493598927	NA
-0.811015076	0,417357013330528	NA
-0.809895551	0,418000209069037	NA
0.809863212	0,418018797304071	NA
0.809854606	0,418023744370574	NA
0.805130723	0,420744248422655	NA
-0.804936551	0,420856294579513	NA
0.804236931	0,421260153716492	NA
0.803169451	0,421876800069224	NA
-0.803074904	0,421931442067471	NA
-0.802535845	0,422243062162882	NA
0.797235641	0,4253141904249	NA
-0.78850882	0,430399141506452	NA
0.783035561	0,433606229982053	NA
0.780651317	0,435007600836793	NA
-0.780644755	0,435011461334567	NA
0.776633827	0,437374837948596	NA
0.773764384	0,439070137748443	NA
0.772158451	0,440020586301418	NA
-0.771538862	0,440387596647388	NA
0.766221608	0,443544453089878	NA
0.765646009	0,443886960499748	NA
0.765034374	0,444251076041478	NA
-0.764698193	0,444451282209474	NA
0.762866876	0,44554279231247	NA
0.758381574	0,448222585328507	NA
0.757396415	0,448812403211271	NA
0.756084208	0,449598709851715	NA
-0.753986791	0,450857153072715	NA
-0.751381963	0,452422814460792	NA
0.749368575	0,453635086489774	NA
-0.748566522	0,454118516788703	NA
-0.746671398	0,455261938542531	NA
0.743004719	0,457478823461178	NA
0.741682277	0,458279860422813	NA
-0.741570392	0,458347668020335	NA
-0.735964409	0,461752358020961	NA

-0.733452456	0,46328251526248	NA
0.731575751	0,464427553301789	NA
-0.731439637	0,464510662151591	NA
0.731003705	0,464776889913122	NA
0.727704546	0,466794466792971	NA
0.72409513	0,469007334466089	NA
0.723238663	0,46953326943987	NA
0.719811287	0,471641194246049	NA
0.719766966	0,471668487131386	NA
0.719508658	0,471827569789925	NA
-0.718945667	0,472174397502439	NA
0.717691797	0,472947342188953	NA
0.715749452	0,47414606940708З	NA
-0.715093052	0,474551547215663	NA
-0.710253864	0,477546723886955	NA
-0.705572928	0,480453764728846	NA
-0.705388714	0,48056836578883	NA
-0.703794073	0,481561025608589	NA
0.703305508	0,481865378951428	NA
-0.701862309	0,482765035989867	NA
0.701078662	0,48325392616707	NA
0.698279929	0,485002148475464	NA
-0.690811382	0,489684082108491	NA
0.690514616	0,489870621631342	NA
0.689891641	0,490262332748262	NA
-0.689714198	0,490373935486356	NA
-0.688809586	0,490943102277267	NA
0.687389745	0,491837157959129	NA
0.682528263	0,49490497785563	NA
0.681749544	0,495397333561506	NA
0.677947136	0,497805211941947	NA
0.676741271	0,498570126153329	NA
0.675542276	0,499331301399487	NA
-0.674880388	0,499751761314055	NA
-0.674836837	0,499779433972729	NA
0.674640783	0,499904015230756	NA
-0.674266764	0,500141730254116	NA
-0.6726334	0,501180547402033	NA
-0.670562537	0,502499254811231	NA
0.670543662	0,50251128281074	NA
-0.669564238	0,503135617608183	NA
0.66912778	0,50341397017283	NA
0.666991662	0,504777459227182	NA
0.664861815	0,50613888092172	NA
0.661208524	0,508478595569036	NA
-0.66076974	0,508759991212414	NA
-0.659508585	0,509569234910243	NA
-0.657260759	0,511013263604069	NA
0.656118804	0,511747686566172	NA
-0.655706165	0,512013201131298	NA

0.655579289	0,512094854829647	NA	
0.654255597	0,512947148102442	NA	
-0.653292048	0,513568017926724	NA	
0.650688436	0,515247629382473	NA	
-0.64869748	0,516533933698943	NA	
0.648103923	0,516917736846417	NA	
-0.645042588	0,518899587261487	NA	
0.644349794	0,51934863327186	NA	
-0.643232713	0,520073109338612	NA	
-0.640722168	0,521703208816502	NA	
-0.640707037	0,52171304128943	NA	
0.639739447	0,522342005326021	NA	
0.636371947	0,52453402091997	NA	
-0.636174426	0,524662739908569	NA	
-0.634564723	0,525712342061868	NA	
0.634014312	0,526071482151271	NA	
0.633996664	0,52608299921004	NA	
-0.633210377	0,526596270026984	NA	
0.632917577	0,526787468821065	NA	
-0.632466621	0,527082013305408	NA	
0.631829889	0,527498040603418	NA	
0.631766273	0,52753961535321	NA	
-0.631740276	0,527556605504947	NA	
-0.628148328	0,529906776543298	NA	
-0.627154799	0,530557768920257	NA	
-0.625508684	0,53163725094343	NA	
-0.625120538	0,5318919493026	NA	
0.621935527	0,533984263251691	NA	
0.61749202	0,536910243014445	NA	
-0.615017616	0,538543087091271	NA	
-0.614898024	0,53862206772586	NA	
-0.612927747	0,539924115503336	NA	
-0.612829502	0,539989081668628	NA	
0.611723224	0,540720893409273	NA	
-0.611275281	0,54101735266427	NA	
0.603549636	0,546143105892728	NA	
0.600955353	0,547869723503796	NA	
0.598609721	0,549433172130386	NA	
0.598265144	0,549663029958803	NA	
-0.598228817	0,549687265713258	NA	
-0.597657599	0,550068422867858	NA	
0.592623706	0,553433010229801	NA	
-0.584131925	0,559131557606998	NA	
0.582183545	0,560443055719354	NA	
-0.581640293	0,5608089963561	NA	
0.577152201	0,563836643209246	NA	
-0.57582746	0,564731810693548	NA	
-0.575051371	0,565256555121345	NA	
0.574928004	0,565339989765063	NA	
0.572521652	0,566968621582101	NA	

-0.571409133	0,56772233938325	NA	
0.571363977	0,567752942661592	NA	
0.571042479	0,567970848231067	NA	
0.570649547	0,56823722511074	NA	
0.570480002	0,568352181890428	NA	
0.57046538	0,568362096599723	NA	
0.569417041	0,569073155070156	NA	
0.566541876	0,571025474313756	NA	
0.566482189	0,57106603709648	NA	
-0.565849496	0,571496096211449	NA	
-0.565657597	0,571626565998352	NA	
-0.564750648	0,572243379392819	NA	
-0.564396298	0,572484457930065	NA	
-0.563444	0,573132583048602	NA	
0.561236198	0,574636529637913	NA	
0.56080366	0,574931391223131	NA	
0.559867574	0,575569768025131	NA	
0.556716464	0,577721166278968	NA	
-0.555328806	0,578669779182631	NA	
0.55388541	0,579657271074612	NA	
0.55375051	0,579749602398991	NA	
-0.553700556	0,57978379441764	NA	
-0.552511035	0,580598276116575	NA	
-0.551635102	0,581198381501548	NA	
-0.550535463	0,581952159868982	NA	
-0.550018701	0,582306546756019	NA	
-0.549957433	0,58234857027198	NA	
0.548754013	0,583174273202709	NA	
-0.547608872	0,583960496511715	NA	
-0.54717023	0,584261786945808	NA	
-0.543799201	0,58657965898126	NA	
-0.54043975	0,588893800797755	NA	
0.540281205	0,589003118338643	NA	
0.535606672	0,592230416285707	NA	
0.534267165	0,593156706152945	NA	
0.532596086	0,59431321239407	NA	
0.532260127	0,594545844294114	NA	
0.5305722	0,595715264292714	NA	
-0.530526678	0,595746816980833	NA	
-0.52767201	0,597727003620213	NA	
0.526083182	0,598830413566262	NA	
0.523662585	0,600513243515376	NA	
0.521759641	0,601837693484851	NA	
-0.521519353	0,602005027734645	NA	
0.520340509	0,602826266249315	NA	
-0.519864253	0,603158192065581	NA	
0.519746835	0,603240039242103	NA	
-0.518705747	0,60396595334873	NA	
0.515387899	0,606281986823003	NA	
-0.514267688	0,607064849520654	NA	

0.512912672	0,608012408822825	NA
-0.512798025	0,608092611263641	NA
-0.512381947	0,608383722929961	NA
0.510517163	0,609689193340224	NA
-0.510433161	0,609748029847332	NA
-0.508056308	0,611413854994891	NA
-0.505965639	0,612880771567368	NA
-0.505703185	0,613065032384705	NA
-0.5050935	0,613493166639537	NA
-0.504847314	0,613666081419612	NA
-0.503534606	0,614588456027663	NA
-0.502790761	0,615111390173611	NA
0.498727203	0,617971577789744	NA
0.496977187	0,619205138906257	NA
-0.496513649	0,61953205959489	NA
-0.494436443	0,620997982209708	NA
-0.494242696	0,621134789366448	NA
0.492374109	0,622454899280446	NA
0.491266116	0,623238242005766	NA
0.486348087	0,626720390551897	NA
0.485542286	0,627291724377195	NA
-0.485236011	0,627508940274866	NA
0.483204334	0,628950658303037	NA
-0.479157105	0,631826867623645	NA
0.479048612	0,631904046191226	NA
-0.478535455	0,632269145534846	NA
-0.478025103	0,632632338396987	NA
0.476170814	0,633952690503105	NA
0.474860292	0,634886555326697	NA
0.473707028	0,635708840644947	NA
-0.473362336	0,635954696318167	NA
0.473342529	0,635968824624897	NA
-0.470874446	0,637730397524276	NA
-0.46987677	0,638443061578043	NA
0.468690432	0,639290927216522	NA
-0.468621922	0,639339904834944	NA
0.468042855	0,639753943312623	NA
0.467050899	0,640463462697235	NA
-0.46642038	0,640914626234363	NA
0.465503863	0,641570671376078	NA
0.465134662	0,641835024834709	NA
0.464942651	0,641972525916167	NA
-0.464377737	0,642377137898542	NA
-0.463993066	0,642652713730529	NA
0.462723583	0,643562511919185	NA
0.460386394	0,645238898770591	NA
0.458497996	0,646594701747404	NA
0.45697486	0,647689115599523	NA
0.455871467	0,648482408250058	NA
-0.455666171	0,648630051630587	NA

0.45545927	0,648778863262166	NA
-0.455095742	0,649040361104735	NA
0.454210156	0,649677573921584	NA
-0.453622306	0,650100695573718	NA
-0.453073009	0,650496170510589	NA
0.451596956	0,651559363535217	NA
0.448289017	0,653944625884874	NA
0.448281392	0,653950128649023	NA
-0.445986289	0,655607150065681	NA
0.444748303	0,656501657708604	NA
0.443115043	0,657682524990074	NA
0.442672137	0,65800289898329	NA
-0.440921515	0,659269817113386	NA
-0.44056789	0,65952585330782	NA
-0.439063198	0,660615744499813	NA
-0.438567462	0,660974978061412	NA
-0.438351711	0,661131346033313	NA
0.436455916	0,66250597711867	NA
-0.435205133	0,663413537191781	NA
-0.434181417	0,664156706449	NA
0.430640671	0,666729664219808	NA
0.430291579	0,666983551903501	NA
0.428542532	0,668256175267035	NA
0.426776194	0,669542347752911	NA
-0.425779995	0,67026816552613	NA
0.424927631	0,670889431464822	NA
0.422041692	0,672994586447701	NA
0.419970668	0,674506881501179	NA
-0.418308091	0,675721875079405	NA
-0.418105114	0,675870266346513	NA
0.415961936	0,67743785638185	NA
0.415590542	0,67770964901285	NA
0.413841843	0,67898993742159	NA
0.412803966	0,679750245719797	NA
-0.412171656	0,680213610523629	NA
-0.409759843	0,681982126457021	NA
0.406116202	0,684657221334851	NA
0.405142313	0,685372905122507	NA
0.401853011	0,687792206142894	NA
0.400249312	0,688972897899136	NA
0.400157076	0,689040827893038	NA
-0.394411531	0,693277231528748	NA
0.392012154	0,695049233270699	NA
-0.391628464	0,695332753478926	NA
-0.390854711	0,695904631511005	NA
-0.389633696	0,696807431924162	NA
0.387474972	0,698404610235256	NA
0.387005628	0,698752041265605	NA
0.38670735	0,698972874556425	NA
-0.384777976	0,700401917522318	NA

0.383175054	0,701589972920857	NA
-0.380081274	0,703885085678356	NA
-0.378869469	0,704784797311249	NA
0.377552413	0,705763121693855	NA
0.377258886	0,705981222798733	NA
0.371896768	0,709969711258853	NA
0.369151589	0,712014737733112	NA
0.367734989	0,713070848407649	NA
0.367705528	0,713092818489036	NA
-0.36761755	0,713158426719781	NA
-0.367124465	0,713526178755117	NA
0.363555193	0,716190190845817	NA
-0.363006866	0,716599755159595	NA
-0.361872841	0,717447056342132	NA
0.360409526	0,718540904023537	NA
0.359323483	0,719353108935934	NA
-0.35855376	0,719928943799811	NA
0.358465855	0,719994716126225	NA
0.35686519	0,721192730194475	NA
0.354736261	0,722787183172315	NA
0.354608226	0,722883112662013	NA
0.352644858	0,72435470385358	NA
-0.348371898	0,727560904701343	NA
0.346722596	0,728799733281937	NA
-0.34582121	0,72947708438999	NA
0.34549656	0,729721096028144	NA
-0.345106174	0,73001455198863	NA
0.345081997	0,730032727534793	NA
0.343555009	0,731180965128298	NA
0.343083992	0,731535273495037	NA
-0.34117268	0,732973589276983	NA
0.34105555	0,733061762959125	NA
0.337313578	0,735880524833528	NA
-0.334847126	0,73774040905348	NA
0.333704441	0,738602598081856	NA
-0.333397055	0,738834586256113	NA
0.333042242	0,739102396833243	NA
-0.332047244	0,739853585317842	NA
-0.331302206	0,740416225476436	NA
-0.331070407	0,740591304322229	NA
-0.328578089	0,742474611762117	NA
-0.326132123	0,74432439355259	NA
0.323921063	0,745997796895017	NA
0.323759472	0,746120141737616	NA
-0.321836627	0,747576464674711	NA
0.320649587	0,74847595521182	NA
0.320357002	0,748697716378542	NA
0.320108093	0,748886390506695	NA
-0.31999655	0,748970946157845	NA
-0.318632385	0,750005292450836	NA

-0.315412971	0,752448119518343	NA	
-0.314280442	0,753308050874398	NA	
-0.314173113	0,753389562109576	NA	
-0.310337176	0,756304563434658	NA	
-0.310004192	0,756557768391904	NA	
-0.309255437	0,757127226915351	NA	
-0.307550266	0,758424568743089	NA	
-0.307241644	0,758659449789792	NA	
0.306133016	0,759503370566108	NA	
-0.304122352	0,761034678073126	NA	
0.303162423	0,761766084311963	NA	
-0.302821796	0,762025672206467	NA	
0.301696015	0,762883806696716	NA	
-0.299753555	0,764365144646722	NA	
0.299361988	0,764663862468934	NA	
0.298729297	0,765146601673168	NA	
-0.298656877	0,765201863379033	NA	
0.297620284	0,765992991691085	NA	
-0.296605026	0,766768073647578	NA	
0.296077882	0,767170604808858	NA	
-0.294000817	0,768757279223904	NA	
-0.292854377	0,769633462543098	NA	
0.292634045	0,769801888059292	NA	
-0.292024013	0,77026826398545	NA	
0.291304354	0,770818557775737	NA	
0.289916537	0,771880089700342	NA	
-0.286768223	0,774289796117405	NA	
0.28608022	0,77481668086885	NA	
0.285921881	0,774937954773267	NA	
-0.285884248	0,77496677868452	NA	
0.285362698	0,77536628251145	NA	
-0.285191734	0,775497252573993	NA	
-0.284565812	0,775976806970804	NA	
-0.283623601	0,776698848273246	NA	
0.274657832	0,783579130647015	NA	
-0.274278321	0,783870743370278	NA	
-0.272442881	0,785281505276805	NA	
-0.272202344	0,785466439934804	NA	
0.270093359	0,78708843093868	NA	
0.269250296	0,787737078202791	NA	
-0.266383569	0,789943818252233	NA	
0.265376284	0,790719603922678	NA	
-0.265242819	0,79082241137925	NA	
0.265149787	0,790894074857616	NA	
0.263672945	0,792031944847116	NA	
-0.261216951	0,793925207113591	NA	
-0.260669954	0,794347038689057	NA	
-0.258679541	0,795882506259622	NA	
-0.2572386	0,796994587457331	NA	
0.256153154	0,797832580068083	NA	

0.251612685	0,801340452589469	NA
-0.247230201	0,804730073457533	NA
0.24463952	0,806735561465747	NA
-0.243892076	0,807314406296122	NA
0.243521917	0,807601108965297	NA
0.239206124	0,810945754495264	NA
0.234257476	0,814785096408335	NA
0.23219338	0,816387819063303	NA
-0.230193632	0,817941310238261	NA
0.23010875	0,818007266173786	NA
0.228552507	0,819216741202703	NA
0.228494216	0,819262052399881	NA
0.227915275	0,819712107302407	NA
-0.227397412	0,820114732143205	NA
0.227193795	0,820273052258723	NA
-0.225821412	0,821340322504273	NA
-0.224282725	0,822537316984165	NA
0.222769868	0,82371462055014	NA
0.222403008	0,824000170720219	NA
0.221763538	0,824497965265059	NA
-0.220012781	0,825861200564238	NA
-0.218742294	0,826850801229624	NA
0.218611993	0,826952309424506	NA
0.217698109	0,827664337128999	NA
-0.214865712	0,829872018297744	NA
-0.214862725	0,829874347421761	NA
0.213763698	0,830731335110917	NA
-0.211793764	0,832267933974173	NA
0.2087467	0,834645981602996	NA
-0.208633091	0,834734676227752	NA
-0.203552949	0,838702864562537	NA
-0.203351493	0,838860310782551	NA
0.202991444	0,839141719352154	NA
-0.202910194	0,839205226078465	NA
-0.202778591	0,839308092371452	NA
0.202774956	0,839310933388516	NA
0.202568407	0,839472385589916	NA
0.201932749	0,839969299410737	NA
0.201430484	0,840361980953712	NA
0.201166513	0,840568375083592	NA
-0.19300981	0,84695128533168	NA
0.192275	0,847526800082988	NA
-0.190271965	0,849096020940769	NA
0.186076434	0,852384821834211	NA
0.185292057	0,852999968036466	NA
0.184434811	0,853672363304673	NA
-0.182679652	0,855049382737172	NA
0.182220147	0,855409963029698	NA
0.181104292	0,856285716135438	NA
-0.178280884	0,85850239031874	NA

0.178193155	0,858571285057673	NA
0.17792034	0,858785536913716	NA
0.176973213	0,859529430403882	NA
0.176403058	0,859977302282985	NA
0.175479144	0,860703157059833	NA
0.174425398	0,861531155886412	NA
0.172825218	0,862788814297106	NA
-0.172260371	0,863232838113598	NA
0.170675968	0,864478560246069	NA
-0.170041209	0,86497772826984	NA
-0.169671559	0,865268442040276	NA
-0.168071971	0,866526659247714	NA
-0.167748657	0,866781016032763	NA
-0.16689557	0,867452219258227	NA
-0.164748266	0,869142125523418	NA
-0.164352357	0,869453766883654	NA
0.163415833	0,870191037161289	NA
0.162690257	0,870762318652024	NA
0.16205982	0,871258746880721	NA
0.161198594	0,871936988205034	NA
-0.161129784	0,871991182480649	NA
-0.159461907	0,873304967905174	NA
0.159448906	0,873315210576499	NA
0.159002205	0,873667136805102	NA
0.158812821	0,873816347433095	NA
-0.155798931	0,876191512893522	NA
-0.154456191	0,877250051412635	NA
-0.154177668	0,877469649622105	NA
0.152566956	0,878739784844646	NA
0.151549956	0,879541906547765	NA
0.150749287	0,880173491777605	NA
-0.15067836	0,880229444594981	NA
0.1497346	0,880974008822363	NA
-0.148589185	0,881877807433925	NA
-0.147672241	0,882601440114726	NA
-0.146597606	0,883449643674569	NA
-0.145126009	0,884611383150947	NA
0.143175051	0,886151932961433	NA
0.141932735	0,887133136881133	NA
0.139659533	0,888929001073479	NA
-0.139473055	0,889076346616242	NA
0.137300889	0,890792967676946	NA
0.135774657	0,891999426103484	NA
0.134266357	0,893191955269316	NA
-0.130902331	0,895852570594727	NA
-0.130021211	0,896549645063383	NA
-0.129247664	0,897161682291728	NA
-0.128955503	0,89739285796146	NA
0.128553464	0,897710991145743	NA
-0.128526086	0,897732656118468	NA

-0.128467343	0,897779140850736	NA
0.127892338	0,898234174944155	NA
-0.124957106	0,900557508705511	NA
-0.11760475	0,906380840760193	NA
-0.11316901	0,909896564429479	NA
0.111904002	0,910899523380924	NA
0.102877563	0,918060145650808	NA
0.099693915	0,920587331934668	NA
-0.098327552	0,921672200888062	NA
0.096929396	0,922782463937591	NA
0.095522501	0,92389981886061	NA
0.094076517	0,925048374740459	NA
-0.093878532	0,925205647237271	NA
0.093366788	0,925612174256763	NA
-0.092953966	0,925940132454751	NA
0.092559873	0,926253223707359	NA
-0.09067393	0,927751686340219	NA
-0.090263503	0,928077822292708	NA
0.088981389	0,929096700779213	NA
-0.088320295	0,929622109043675	NA
-0.087363001	0,930382977924501	NA
0.084162371	0,932927335844252	NA
-0.083375903	0,933552648086409	NA
-0.083283615	0,93362602875418	NA
0.083227994	0,933670254339492	NA
0.083087958	0,933781600832991	NA
0.08200824	0,934640160888161	NA
0.078292073	0,937595722968179	NA
-0.077689359	0,938075158960843	NA
0.076049526	0,93937969592217	NA
-0.075526693	0,939795660025202	NA
0.075239785	0,940023930304493	NA
0.075041833	0,940181427850623	NA
-0.073351195	0,941526653275456	NA
-0.071752947	0,942798518732801	NA
-0.071456382	0,94303453779553	NA
0.069259959	0,944782696987819	NA
0.069051284	0,944948798240205	NA
-0.068813007	0,945138464176402	NA
0.066021978	0,947360327713742	NA
0.065139285	0,948063102282201	NA
0.063224017	0,949588120576571	NA
0.062984364	0,949778955151734	NA
-0.06038945	0,951845460766435	NA
0.05782017	0,953891871626918	NA
0.057682841	0,954001262131257	NA
0.056178739	0,95519941847217	NA
0.055370663	0,955843167807074	NA
0.052332105	0,95826407210409	NA
-0.050950136	0,959365254230134	NA

-0.050494638	0,959728222524477	NA
-0.046818289	0,962658052312673	NA
-0.043570976	0,96524638733453	NA
0.043252509	0,965500248369647	NA
0.042755183	0,965896690312753	NA
-0.042584978	0,966032370515094	NA
-0.042538048	0,966069781188158	NA
0.042525294	0,966079947996682	NA
0.041618155	0,966803100125402	NA
-0.040903303	0,967372984637562	NA
0.036881808	0,970579244682589	NA
-0.03622149	0,971105750508476	NA
-0.034338452	0,972607262766821	NA
0.034185491	0,972729236674502	NA
-0.03255835	0,974026784374669	NA
0.032433849	0,974126068888679	NA
-0.032273475	0,974253962218192	NA
-0.030863366	0,975378505755515	NA
-0.030601177	0,975587603768626	NA
-0.030284596	0,975840081938836	NA
0.029973361	0,976088298223731	NA
-0.029416327	0,97653255108608	NA
-0.028076616	0,97760104452923	NA
0.024087983	0,980782428941979	NA
0.022079123	0,982384839813005	NA
0.019251481	0,984640488913686	NA
0.018195177	0,98548315048947	NA
0.015923396	0,987295504895042	NA
-0.013396198	0,989311700381304	NA
-0.012636734	0,989917613190586	NA
-0.011569044	0,990769444203879	NA
-0.010805033	0,991378999117355	NA
0.009765943	0,992208028505251	NA
-0.00791252	0,993686788117107	NA
0.005695914	0,995455342737377	NA
-0.004741185	0,996217096233937	NA
0.004146267	0,996691766811901	NA
-0.003142185	0,997492902869474	NA
-0.002831228	0,997741009887612	NA
0.002276149	0,998183897181713	NA
-0.001354926	0,998918925872739	NA
-2.16982623	NA	NA
-0.565634516	NA	NA
-1.113515238	NA	NA
0.25770222	0,796636732833255	NA
1.045856233	0,295627414156035	NA
0.091667124	0,92696251890597	NA
0.091287807	0,927263905980247	NA
-0.316762522	0,75142380359846	NA
1.231428417	0,218162673973646	NA

-0.477495057	0,633009639570932	NA
-0.971804966	0,331147585009941	NA
-2.003849414	0,0450861937600924	NA
-0.85917677	0,390242997429043	NA
-1.492317731	0,135615870534789	NA
0.426402002	0,669814943068793	NA
1.178739001	0,23850212033912	NA
0.95775277	0,338187440379461	NA
0.704162872	0,481331349556174	NA
-0.05196103	0,958559745050809	NA
-0.035720101	0,971505542281505	NA
0.430268534	0,667000313294821	NA
0.379232236	0,704515415093265	NA
1.051749158	0,292914650780576	NA
0.586433936	0,557583945026931	NA
-1.14173051	0,253566054757979	NA
-0.254771886	0,798899290481638	NA
1.553325123	0,120345481260985	NA
0.764194913	0,444751097606582	NA
1.552035999	0,120653610105814	NA
-0.630023253	0,5286793704909	NA
-0.156082852	0,875967715011301	NA
-0.863699047	0,387753237093098	NA
1.55130285	0,120829124210574	NA
-0.852787012	0,393777427805979	NA
-1.675558152	0,093824771578091	NA
1.331221471	0,183116148410347	NA
-1.31015732	0,190142620734838	NA
1.437122388	0,150683220924372	NA
1.511489086	0,130663887135745	NA
-0.028390477	0,977350719463542	NA
-0.617037504	0,537209988788357	NA
0.311142447	0,755692333210498	NA
-0.661604639	0,508224633835313	NA
-0.401422254	0,688109268025493	NA
1.559490495	0,118880332497276	NA
-0.905117131	0,365403337208486	NA
0.43956703	0,660250724064252	NA
-1.229164421	0,219010165756594	NA
0.132261655	0,894777335790832	NA
1.61472435	0,106370401942783	NA
0.690964724	0,489587709626238	NA
0.553469407	0,579942023897806	NA
-0.878441392	0,379704230105233	NA
0.044623561	0,964407362042481	NA
1.26958771	0,204231529503678	NA
0.414974381	0,678160657818102	NA
0.027396223	0,978143710525629	NA
0.674356335	0,500084796354173	NA
0.515165155	0,606437615858071	NA

1.264524014	0,20604200699798	NA
0.851256948	0,394626634784995	NA
0.530038599	0,596085168831446	NA
2.433512214	0,014953130923792	NA
0.984312924	0,324961676727253	NA
4.623557305	3,7721446818326e-06	NA
-0.310440739	0,756225818203751	NA
-1.291570784	0,196505827389816	NA
0.135034189	0,892584843332361	NA
-0.266215233	0,790073451703317	NA
1.466997201	0,142376816747089	NA
1.764978315	0,0775674103394647	NA
0.372150105	0,70978109232945	NA
-0.134958104	0,892644999579722	NA
-2.718856092	0,00655081017540023	NA
-0.91867584	0,358265150243038	NA
-1.338072949	0,180872657922382	NA
-1.249950722	0,211317549195544	NA
2.309999078	0,0208882051736846	NA
-2.187260221	0,0287235328685573	NA
0.587981635	0,556544619883021	NA
0.077894151	0,937912252198037	NA
-0.821546023	0,411335320259041	NA
0.306990511	0,758850595028315	NA
0.780980836	0,4348137658475	NA
-0.319367673	0,74944772118546	NA
-0.014607284	0,988345488054817	NA
-1.477606768	0,13951306176473	NA
-0.403168686	0,686824133919431	NA
-1.80172988	0,071587914631776	NA
0.726110252	0,46777118448814	NA
-0.305534728	0,759958924887337	NA
0.926030237	0,354430266601826	NA
-1.156766092	0,247367938942808	NA
-1.307997559	0,190874137302021	NA
0.218722197	0,826866456896204	NA
0.28199607	0,777946523775628	NA
0.409978702	0,681821571103192	NA
0.029379212	0,976562152103477	NA
0.000496727	0,999603669042959	NA
-0.737692434	0,460701370773601	NA
0.195721774	0,844827940742555	NA
-0.513338483	0,607714568949595	NA
0.306824937	0,758976626388626	NA
1.017600242	0,308867972068728	NA
-2.829985026	0,00465501826776613	NA
0.139990027	0,888667870357865	NA
1.152160692	0,249255046951967	NA
-0.109386478	0,912895958121201	NA
-0.097450856	0,922368359294033	NA

0.452249884	0,651088975398395	NA
-0.68276113	0,494757795035201	NA
1.659721906	0,0969704103927655	NA
-1.512707154	0,130354061489865	NA
-1.023107462	0,306257039031978	NA
0.489641433	0,624387651937843	NA
1.140217447	0,254195720646455	NA
1.307457396	0,191057415346783	NA
-1.23371055	0,217310781184215	NA
-0.263512188	0,792155830559977	NA
-0.6342012	0,525949524635926	NA
-0.950709434	0,341751897687228	NA
-2.081661042	0,0373734414366039	NA
-0.56633034	0,571169239003778	NA
0.636142043	0,524683844907505	NA
-0.516395353	0,605578310941438	NA
0.190582736	0,848852516704111	NA
0.535353953	0,592405124505095	NA
1.499502073	0,133743431267978	NA
2.271919666	0,0230913628488264	NA
-0.813656231	0,415841914656107	NA
-0.817562072	0,413607291893001	NA
0.723074594	0,469634057081124	NA
-1.067296795	0,28583783491036	NA
0.662559922	0,507612445504197	NA
1.54444981	0,122479401089966	NA
0.946931382	0,343673745378294	NA
-0.982979463	0,325617546395159	NA
0.270438087	0,786823241834974	NA
0.272777863	0,785023976892455	NA
-0.031370695	0,974973911743449	NA
-0.199580374	0,841808778239403	NA
-0.86376162	0,387718855526318	NA
0.651346471	0,514822856257231	NA
1.261268736	0,207212041103013	NA
-0.238376929	0,811588759798937	NA
-2.268018946	0,0233280519517633	NA
1.010066799	0,31246328735202	NA
1.089052909	0,276130554437476	NA
0.292196876	0,770136099739306	NA
0.35355127	0,723675199118063	NA
0.283936518	0,776459030269727	NA
-1.135772605	0,256051744935996	NA
-0.227165096	0,820295367229317	NA
0.116140034	0,907541560870764	NA
0.144532726	0,88507981625632	NA
2.066377088	0,0387928876264421	NA
-0.427173352	0,669253069360798	NA
-1.784005911	0,074422704645013	NA
-2.66389672	0,0077241273449376	NA

0.647537283	0,517284272755268	NA
-0.865669435	0,386671466121529	NA
0.107172365	0,914652238600573	NA
0.642589452	0,520490529073528	NA
1.498231092	0,134073220221121	NA
-0.141296994	0,88763532437303	NA
-0.557897546	0,576914347202064	NA
1.172730489	0,24090390099374	NA
-0.337049728	0,736079413753563	NA
0.774776671	0,43847163647252	NA
-0.521198146	0,60222874539887	NA
1.123320935	0,261301215787985	NA
0.329884462	0,741487264471545	NA
-0.396484063	0,691747962343923	NA
-0.807928858	0,419131537742919	NA
-1.721452088	0,0851688191779819	NA
-1.318102153	0,187469454033549	NA
-0.27206716	0,785570379931251	NA
-0.345733869	0,729542728598765	NA
-2.382274916	0,0172060469385838	NA
-0.597456424	0,550202691997212	NA
-0.781281333	0,434637046471068	NA
0.254847741	0,798840700000263	NA
-0.994113755	0,320167489687651	NA
0.990740016	0,321812545830576	NA
0.319700178	0,749195625070235	NA
1.175572942	0,239765573510638	NA
-0.453791294	0,649979050462723	NA
0.177615761	0,859024745713618	NA
-0.853023454	0,393646298030912	NA
1.557036247	0,119461879929744	NA
1.453181301	0,146173422578934	NA
-0.782704079	0,433800906138246	NA
-0.685668701	0,492922048039161	NA
-0.038749689	0,969089956983279	NA
-0.328157756	0,742792386320409	NA
-0.440069182	0,659887001985133	NA
1.036566305	0,299938023357166	NA
1.047458519	0,294888154246213	NA
-1.363256809	0,172801588846716	NA
-0.704319105	0,481234070856511	NA
-0.954845917	0,339655623092262	NA
-1.201979737	0,229371378942658	NA
-0.411356711	0,680810991138661	NA
-0.727939666	0,466650520454371	NA
-0.675049251	0,499644474386323	NA
-0.453614643	0,650106211971592	NA
-0.598048154	0,549807802588205	NA
-0.211027199	0,832866047678782	NA
-2.367211023	0,0179227103879258	NA

-0.133024024	0,894174380592544	NA
-2.283128669	0,0224227902758559	NA
-0.045716879	0,963535910627595	NA
1.217489413	0,22341806203142	NA
2.279040449	0,0226646609402095	NA
1.858281075	0,0631291099100789	NA
4.189842291	2,79148401071603e-05	NA
1.090167205	0,275639496866505	NA
0.516114519	0,605774427796704	NA
-1.282844334	0,199546618437897	NA
0.129625085	0,896863055943921	NA
0.784791148	0,432576031129812	NA
2.113972821	0,0345175887910787	NA
0.759412791	0,447605664563696	NA
0.527212481	0,598046042278288	NA
-0.773875633	0,439004340421734	NA
-0.474325546	0,635267777227156	NA
-0.471945002	0,636966045649683	NA
0.559504438	0,575817503090863	NA
0.196805194	0,843979986558584	NA
2.304078389	0,0212182436066066	NA
-0.721710219	0,470472658174875	NA
0.855398495	0,392330576449081	NA
-1.337943075	0,180914993922007	NA
1.313640364	0,188967258462665	NA
-1.485834215	0,137322977701321	NA
-0.657606677	0,510790902844467	NA
1.957835392	0,0502493309898315	NA
1.103063218	0,269999712555817	NA
0.412166138	0,680217654531478	NA
-0.052911451	0,957802460512162	NA
0.13509338	0,892538044068143	NA
-0.088719475	0,929304854577124	NA
-0.115398602	0,908129186825953	NA
-0.426553614	0,669704489405067	NA
-0.45431031	0,649605496231044	NA
0.496771232	0,619350383926744	NA
0.377487568	0,705811301530372	NA
-0.316572177	0,751568250195616	NA
-0.027844365	0,977786281770974	NA
1.068631768	0,285235628028521	NA
-1.067951951	0,285542186325433	NA
1.802571917	0,0714554707280367	NA
-0.58572844	0,558058018824473	NA
-1.969235515	0,0489260523824618	NA
-0.637922213	0,523524317523568	NA
2.270955804	0,0231496535405323	NA
1.475472047	0,140085678008202	NA
0.939381166	0,347535079087804	NA
-0.933539936	0,350541260843811	NA

-0.936942617	0,348788075811061	NA
0.50822752	0,611293793516102	NA
0.025182215	0,97990962287861	NA
-0.109995347	0,912413065047538	NA
-0.166405338	0,867837973300324	NA
-0.2903467	0,771551015300026	NA
-0.294393169	0,768457486404528	NA
-0.814826449	0,415171659012787	NA
-1.50779049	0,131608156947599	NA
0.091631505	0,926990819636943	NA
1.327241231	0,18442889722382	NA
-0.108887101	0,913292038037922	NA
-0.576346704	0,564380860412953	NA
0.105841826	0,915707850814255	NA
0.273465548	0,78449537174026	NA
-0.113985981	0,90924890631007	NA
-0.297231536	0,766289746785213	NA
-0.656214817	0,511685916350591	NA
1.021517506	0,307009319602137	NA
0.349848812	0,726452164587052	NA
-0.477661163	0,632891390365863	NA
2.490671294	0,0127502018744137	NA
-0.126831439	0,899073811930333	NA
1.176433014	0,239421885770392	NA
-1.027916334	0,30398918522082	NA
0.095817483	0,923665531669146	NA
0.31013465	0,75645856384799	NA
-0.905658202	0,365116792410388	NA
0.795792743	0,426152510543742	NA
1.099404946	0,271591474352078	NA
-0.497968575	0,618506191542298	NA
0.212144619	0,831994211010592	NA
0.234093295	0,814912551466843	NA
-0.037605953	0,970001861644156	NA
0.456472184	0,64805046853651	NA
0.343126246	0,731503486758795	NA
0.000874402	0,999302328289855	NA
0.343313757	0,731362432773434	NA
-0.50640557	0,612571965092578	NA
0.148897923	0,881634180152015	NA
0.780342039	0,435189574649005	NA
0.92462015	0,355163528098452	NA
-1.224258533	0,220854714540376	NA
0.547737256	0,583872326881847	NA
-1.60867573	0,107687265313414	NA
-0.540919784	0,5885628738054	NA
-0.820083961	0,412168244897598	NA
0.632654449	0,526959321770044	NA
-0.323684991	0,746176535263026	NA
-0.099879602	0,920439910941615	NA

-0.591600105	0,554118403318694	NA
1.188550458	0,234616612897816	NA
-0.012416047	0,99009368195357	NA
-0.196125433	0,844511989912083	NA
0.155443486	0,876471704128954	NA
-0.091439626	0,927143276677226	NA
0.473809704	0,635635613796585	NA
0.503787745	0,614410540350985	NA
0.605896215	0,544583673001991	NA
0.4769075	0,633427990893461	NA
-1.083341298	0,278656961074518	NA
-0.438566962	0,660975340270989	NA
0.445973625	0,655616297883768	NA
0.651082346	0,514993331751233	NA
0.242017185	0,808766847304186	NA
0.304874353	0,76046185007781	NA
-3.093329951	0,00197923946063583	NA
-3.150076395	0,00163227774397457	NA
0.211627759	0,83239745149007	NA
1.16981374	0,242075932941758	NA
0.464376542	0,642377993429149	NA
0.987333076	0,32347938071765	NA
1.428111358	0,153259808727375	NA
1.075389327	0,282200458006898	NA
0.261777607	0,793492904604047	NA
-0.885610364	0,375827524710522	NA
0.462459207	0,643752049404607	NA
0.271994155	0,7856265133667	NA
0.670122957	0,502779411270788	NA
-0.167084517	0,867303548514693	NA
-0.495408671	0,620311675057079	NA
0.419734938	0,674679098660504	NA
0.755379781	0,450021141325505	NA
-1.388761089	0,16490541110427	NA
-0.673004054	0,50094471171022	NA
0.338660111	0,734865793793374	NA
1.293827207	0,195725112586683	NA
1.287782703	0,197821615175993	NA
-1.432364924	0,152039411530133	NA
2.52804968	0,0114698120729043	NA
1.267896159	0,204835036034395	NA
-2.498486131	0,0124725022965019	NA
0.122039406	0,902867810327967	NA
-1.450785282	0,146839659192478	NA
2.03118699	0,0422360295967145	NA
2.579753024	0,00988709979240269	NA
0.636215869	0,524635731381211	NA
1.831267338	0,0670606502220432	NA
1.919520246	0,0549185264912861	NA
-0.640603888	0,521780073088385	NA

0.885324354	0,375981718703063	NA
-0.672020733	0,501570497534496	NA
-0.396432544	0,691785961573892	NA
2.590946096	0,00957124822694289	NA
-0.3198229	0,749102586914927	NA
0.502624241	0,61522848310851	NA
1.149260509	0,250448577028577	NA
0.058000715	0,953748059143443	NA
1.991841684	0,0463884310717213	NA
2.115684833	0,0343716213350855	NA
-0.073837226	0,941139905398033	NA
0.159305122	0,873428485498401	NA
-0.983792895	0,325217353391469	NA
1.459964617	0,144299798726168	NA
0.605835524	0,54462397765996	NA
0.165948481	0,86819749430694	NA
1.544552382	0,122454571777486	NA
-0.33402974	0,738357116641222	NA
0.166985007	0,867381846153925	NA
-1.022441949	0,306571774919883	NA
1.955894354	0,0504776005678964	NA
-0.360926288	0,718154551180406	NA
0.654404901	0,512850977430517	NA
0.035443605	0,9717260149455	NA
0.686537812	0,49237402890678	NA
0.837051654	0,402563533781846	NA
0.678044707	0,497743347051178	NA
-1.626270422	0,103892140950516	NA
2.078663244	0,0376483136286246	NA
-0.29928855	0,764719890088664	NA
-0.050716083	0,959551760745718	NA
0.368140241	0,712768666378813	NA
0.149280922	0,881331968827774	NA
0.443383639	0,657488268224318	NA
0.743845806	0,456969765016506	NA
-1.157330941	0,247137177005797	NA
0.896623145	0,369920043264419	NA
-0.959917004	0,337096987308269	NA
0.706996557	0,479568617539787	NA
0.863250394	0,387999811895643	NA
0.542157067	0,587710308288825	NA
2.520072783	0,0117330570475058	NA
-1.014858353	0,31017335296321	NA
0.381448884	0,702870193381616	NA
-0.10777367	0,91417522972177	NA
-0.749465737	0,45357654223808	NA
-0.539535708	0,589517264713637	NA
-0.215121357	0,829672703488697	NA
0.812254413	0,416645662995848	NA
-0.953838724	0,340165284446636	NA

-1.193883814	0,232523419694884	NA
1.445155334	0,148414259267657	NA
-1.267140278	0,20510513566325	NA
0.798111655	0,424805698416352	NA
-0.578924451	0,56264015105412	NA
0.644877041	0,519006871264157	NA
1.767182867	0,077197608099028	NA
0.358215131	0,720182324863916	NA
1.799931097	0,0718715186468829	NA
-1.87413621	0,0609116536393893	NA
-0.062979167	0,94978309331249	NA
-0.376672815	0,706416767474706	NA
0.564170686	0,572637975844719	NA
1.121144771	0,262226243927943	NA
-0.119535875	0,904850820023994	NA
-1.390945306	0,16424201288197	NA
-0.611140802	0,541106369746534	NA
0.048152866	0,961594414162749	NA
-0.908332836	0,363702398989892	NA
-0.074188389	0,940860484527323	NA
0.726841307	0,467323175228541	NA
1.45944574	0,144442464689355	NA
0.881413346	0,378094136707737	NA
1.000539509	0,317049487468105	NA
0.04485733	0,964221028693209	NA
-1.455645903	0,145490531609001	NA
0.458282222	0,646749694887054	NA
-1.286976947	0,19810232353681	NA
-0.475231001	0,634622332775872	NA
0.130347125	0,896291797456147	NA
-0.483802446	0,628526079318056	NA
-0.933704174	0,350456511365401	NA
-0.369133362	0,71202832296628	NA
-0.560274197	0,575292424836016	NA
0.518963405	0,603786260918898	NA
0.75798417	0,448460459414579	NA
0.818239003	0,413220728105631	NA
-0.750079152	0,453207034816702	NA
-0.87054383	0,384003277517383	NA
-0.055204883	0,955975238937543	NA
-0.08875095	0,92927984025616	NA
1.273149185	0,202965108307105	NA
0.862736873	0,388282154069402	NA
0.501762311	0,615834727590168	NA
0.503041046	0,614935414322302	NA
-0.830130093	0,406465234254785	NA
-2.205568676	0,0274142143596833	NA
0.743278339	0,457313182708595	NA
0.570453675	0,56837003339602	NA
1.673996988	0,094131191242275	NA

1.307311005	0,191107108063487	NA
-0.393975904	0,693598829297368	NA
-0.526822676	0,598316734049998	NA
-0.148519459	0,881932829919215	NA
0.014104249	0,988746810628343	NA
-0.496980414	0,619202863342074	NA
1.731703386	0,0833263875097251	NA
0.316240521	0,751819953333695	NA
1.025765056	0,305002340822897	NA
1.262752141	0,206678269059081	NA
2.117108059	0,0342506778939489	NA
0.336204893	0,736716363207398	NA
0.712109524	0,476396957009518	NA
-0.788840592	0,430205181174195	NA
0.250139731	0,802479291583902	NA
0.730945138	0,464812663722978	NA
1.61803382	0,105655309330102	NA
-0.127115505	0,898848980109941	NA
0.099893809	0,920428631798608	NA
-0.334583251	0,737939481383687	NA
0.459256	0,646050340377746	NA
-0.620040407	0,535231184588729	NA
-1.069347586	0,28491307594337	NA
1.096197305	0,27299243881091	NA
-0.682926726	0,494653144988575	NA
0.011664143	0,990693571077184	NA
0.222683022	0,823782216105861	NA
1.456163481	0,145347432256248	NA
0.252169477	0,800910069454995	NA
-1.875595546	0,0607108384205989	NA
0.364562477	0,715438029008158	NA
-0.784551384	0,432716643513801	NA
1.282787019	0,199566703133072	NA
1.866244025	0,0620072321876009	NA
0.798935507	0,424327808768701	NA
0.541152985	0,588402140292871	NA
0.392898197	0,694394673621081	NA
0.18822875	0,850697336192675	NA
0.151151134	0,879856496493824	NA
-0.406418218	0,684435336285626	NA
0.030928824	0,97532630230614	NA
-0.530470857	0,595785509439851	NA
0.831333036	0,405785517072036	NA
-0.505560351	0,613165321713625	NA
0.624742874	0,532139828849367	NA
0.151727954	0,87940150814276	NA
-1.579465977	0,114229214383503	NA
1.596485157	0,110380517089255	NA
0.522895596	0,601046909782295	NA
1.24515872	0,213073413234912	NA

0.204207387	0,838191438729027	NA
-0.723002884	NA	NA
-0.055746935	0,955543408544334	NA
1.193737756	0,232580566323924	NA
-0.244472714	0,806864731847895	NA
1.02595253	0,304913959947324	NA
0.839336463	0,401280527167142	NA
0.680181356	0,496389636001507	NA
0.139400108	0,889133986868134	NA
1.466189616	0,142596636753733	NA
0.337661491	0,735618297618443	NA
1.000326987	0,317152291184609	NA
0.796444768	0,425773566317554	NA
0.527077933	0,598139470253717	NA
-0.592567364	0,553470725984387	NA
1.283316566	0,199381191298925	NA
0.407556997	0,683598941186958	NA
2.222326288	0,0262612628337948	NA
-1.890613832	0,0586759125121279	NA
-1.068951194	0,285091661835869	NA
-1.910005685	0,0561324812955976	NA
-0.032833476	0,973807382419446	NA
0.759571591	0,44751070609395	NA
-0.668927651	0,503541630292021	NA
-1.267842509	0,204854198394602	NA
-1.233704161	0,217313162772554	NA
-0.849625644	0,39553325004688	NA
-0.615191435	0,538428303343251	NA
-2.467146621	0,0136194577392401	NA
-1.321700829	0,186267792393352	NA
-1.348854431	0,177383727312917	NA
0.169905281	0,865084628105735	NA
-0.134484717	0,89301929516622	NA
1.376122904	0,16878358571554	NA
-1.761927074	0,0780816196116618	NA
0.116993982	0,906864822189456	NA
-0.132314277	0,894735715453824	NA
0.480739171	0,630701887456577	NA
-0.257017424	0,797165322434928	NA
0.85080493	0,394877722445864	NA
-0.29119573	0,770901627856254	NA
0.378318975	0,705193651068154	NA
-0.043442208	0,965349031944307	NA
1.439291669	0,150067902222699	NA
1.861497803	0,0626739106333522	NA
0.027475334	0,978080612940789	NA
0.531587429	0,59501177324357	NA
0.64126046	0,521353474297428	NA
0.270987449	0,786400685805452	NA
0.432063677	0,665695133263432	NA

-0.694043639	0,48765484223586	NA
0.459089238	0,646170084587039	NA
-9.67399E-05	0,999922812757336	NA
-1.125689149	0,260297118344955	NA
-0.635380495	0,525180286157662	NA
-0.463575697	0,64295176954696	NA
0.683496494	0,494293165038675	NA
-1.042997944	0,296949245086706	NA
0.479462559	0,631609597763037	NA
-0.144578769	0,885043461037486	NA
-0.415466733	0,677800263715162	NA
0.880496879	0,378590195019473	NA
1.420571433	0,155441388147192	NA
-0.27736041	0,781503377622122	NA
-0.4022216	0,687520946771295	NA
-1.281852304	0,199894462251043	NA
-1.500134164	0,133579652966533	NA
-1.432751481	0,151928871479575	NA
-1.592824714	0,111199519362892	NA
-0.688834174	0,490927627308045	NA
0.324844566	0,745298713097478	NA
0.203045048	0,839099822089095	NA
-0.504881288	0,613642217629692	NA
-1.268669025	0,204559134563159	NA
3.302024422	0,000959897121260178	NA
1.185837046	0,235686658485962	NA
-1.495621484	0,13475232136406	NA
1.037152781	0,299664660382932	NA
-1.39145846	0,164086447913478	NA
-0.470058477	0,638313239080607	NA
-1.130492875	0,258268598916676	NA
0.49410756	0,621230219005396	NA
-0.03479203	0,972245575655668	NA
0.405425322	0,685164900729858	NA
-0.567934954	0,570079136226145	NA
-1.472745783	0,140819596526713	NA
1.491415243	0,135852508574196	NA
-0.054550163	0,95649684391948	NA
1.050695385	0,293398516153527	NA
-0.619191447	0,535790245391376	NA
-0.673917569	0,500363721680583	NA
0.082766356	0,934037321335001	NA
-1.044247439	0,296370923957588	NA
-1.461126235	0,143980801735339	NA
-0.632627273	0,52697707260386	NA
0.801007104	0,423127533197823	NA
0.819675568	0,412401081718854	NA
1.070376512	0,284449867148452	NA
-0.712096212	0,476405199781536	NA
0.27603512	0,782521091732777	NA

-0.213729163	0,830758267626109	NA
-1.200661192	0,229882653604481	NA
0.389683383	0,696770685599817	NA
-3.397354956	0,000680406470548928	NA
0.137093968	0,890956520253386	NA
0.926587411	0,354140793796527	NA
0.5947894	0,551984253790555	NA
0.133723769	0,893621008963137	NA
2.488378836	0,01283269556959	NA
-0.090377238	0,927987444181067	NA
0.732549469	0,463833260539253	NA
0.304480008	0,760762221405245	NA
-1.512087945	0,130511491228543	NA
0.650971868	0,515064646863862	NA
0.458420064	0,646650679939103	NA
-0.012693214	0,989872552175213	NA
1.713033747	0,0867063313523963	NA
0.188785787	0,850260712084316	NA
0.454989966	0,649116457294208	NA
-1.561917783	0,118307358593235	NA
0.485043112	0,627645764084342	NA
0.16881124	0,865945116580951	NA
1.046832956	0,295176627736473	NA
-2.431354364	0,0150424943214333	NA
1.395564662	0,162845630209758	NA
2.373858325	0,0176033034155698	NA
0.74998276	0,453265088059548	NA
0.325520204	0,744787394277043	NA
0.195790906	0,844773828492708	NA
-0.326244191	0,744239609538575	NA
1.54682383	0,12190573232473	NA
0.261236564	0,793910082696344	NA
-0.249790317	0,802749508462203	NA
0.99619084	0,319157429082345	NA
0.088625393	0,929379626910441	NA
0.953427805	0,340373359259875	NA
-0.407920925	0,683331729511137	NA
-0.046651868	0,962790691935549	NA
1.645031233	0,0999633704253129	NA
1.239245252	0,215254687026291	NA
0.55480871	0,579025508650122	NA
0.375342074	0,70740607942252	NA
-1.001889577	0,316396927373252	NA
-0.140091928	0,888587358462883	NA
2.511796206	0,0120118433079797	NA
0.689193291	0,49070163826657	NA
0.334502951	0,738000064418517	NA
1.737754884	0,0822540132989416	NA
1.45884954	0,144606524047884	NA
-0.606187491	0,544390258215022	NA

0.704334075	0,481224750440747	NA
-0.499249715	0,617603475286928	NA
-0.551874594	0,581034275796699	NA
1.076985023	0,281486956608862	NA
-1.429001415	0,153003826668661	NA
-0.910554954	0,362529914325901	NA
-0.119724746	0,904701196919084	NA
-0.717051489	0,473342326696369	NA
0.667376141	0,504531901854485	NA
-0.512858957	0,608049985497694	NA
1.282268127	0,199748604873054	NA
1.982889154	0,0473798124230314	NA
0.185617593	0,852744655924319	NA
-0.778915309	0,436029605089094	NA
-0.652285233	0,514217184687326	NA
0.401216566	0,688260685212855	NA
0.401229389	0,6882512453062	NA
-0.308983917	0,757333760718514	NA
-0.078401791	0,937508448862212	NA
1.387849317	0,165182933992132	NA
0.3293979	0,741854954634953	NA
-0.641804462	0,521000151991238	NA
-0.625870074	0,531400164433511	NA
-0.650312415	0,515490438980927	NA
-1.150677481	0,249864944043365	NA
-1.839378031	0,0658596031657693	NA
0.846062423	0,397517922031575	NA
0.639373954	0,522579688969869	NA
0.0622747	0,95034407577212	NA
0.113011181	0,91002169080391	NA
-0.338524963	0,734967618780574	NA
0.477990356	0,632657068974724	NA
0.903618048	0,366197968045599	NA
1.541636117	0,123162043885965	NA
1.850182546	0,0642872437469223	NA
-0.268176439	0,788563510268773	NA
1.390781681	0,164291639762878	NA
1.210931908	0,225921502292171	NA
-0.09210556	0,926614170830479	NA
0.839587248	0,401139851489855	NA
0.463977052	0,642664186850937	NA
-0.594948394	0,551877966976955	NA
0.582052868	0,560531070729453	NA
1.740313188	0,0818040396207658	NA
1.293051012	0,195993416673457	NA
0.124358876	0,901031132486231	NA
-1.089068092	0,276123859492092	NA
-0.415652928	0,677663990469322	NA
0.498467962	0,618154245312607	NA
2.459609659	0,0139088199200292	NA

0.45264697	0,650802971008691	NA
-1.249051848	0,211646110842	NA
-0.213737933	0,830751428534594	NA
0.035267313	0,971866587349896	NA
0.260715697	0,794311759777895	NA
1.497413553	0,13428568396696	NA
0.997071166	0,318729967821434	NA
0.01635737	0,986949288871363	NA
0.642748079	0,520387578302208	NA
0.911497787	0,362033150992293	NA
0.384407726	0,700676275384671	NA
0.130418126	0,896235626918943	NA
1.294540141	0,195478913309567	NA
0.080901536	0,935520258127629	NA
-0.729116878	0,465930171229674	NA
-0.641197205	0,521394565089379	NA
-0.163359759	0,870235184879099	NA
0.561337824	0,574567261299289	NA
0.394542867	0,693180284861539	NA
-0.756661053	0,44925295371955	NA
0.39184969	0,695169277606497	NA
0.204937078	0,837621285980499	NA
0.503431083	0,614661222316603	NA
0.865225511	0,386915025979506	NA
-0.495140686	0,620500815591416	NA
-0.238018381	0,811866838222755	NA
0.727235104	0,467081945385306	NA
0.38324617	0,701537248075208	NA
0.234455107	0,814631681250491	NA
-1.007201909	0,313837755993138	NA
-0.570716296	0,568191970649877	NA
0.625611418	0,531569847931881	NA
-0.092334954	0,926431917522217	NA
1.035996917	0,300203580649713	NA
0.040184557	0,967945989049262	NA
-0.49942309	0,617481356054585	NA
-1.235091906	0,216796301361685	NA
1.969786179	0,0488628811917996	NA
-0.40487806	0,685567146517681	NA
-0.010868218	0,991328586981269	NA
-0.289586664	0,772132470790397	NA
-0.650717023	0,515229172202797	NA
-0.856735704	0,391590966680744	NA
1.318734401	0,187257921702243	NA
-0.342282594	0,732138233456145	NA
2.5126169	0,0119839393745226	NA
-0.149701122	0,881000422834875	NA
-0.055982797	0,955355511688341	NA
-1.634708098	0,102110254015969	NA
-0.331338841	0,740388555848413	NA

-0.793156862	0,427686437163753	NA
1.240681663	0,214723371027469	NA
0.618611975	0,536172011121458	NA
-0.128624921	0,897654446036097	NA
2.516095002	0,0118663192170988	NA
-0.257589557	0,796723690417422	NA
0.708116572	0,478872869384689	NA
0.793782276	0,427322192642164	NA
1.372801206	0,169814155845804	NA
1.510916464	0,13080973567909	NA
0.235350576	0,813936643077344	NA
-0.286306315	0,774643521512372	NA
-0.610736449	0,541374071789081	NA
-0.008974053	0,992839837913458	NA
0.689109752	0,490754203551903	NA
-1.005031434	0,314881714155181	NA
-0.35951943	0,719206544850385	NA
1.409216221	0,158771242861081	NA
1.721074104	0,0852373777241968	NA
0.015062847	0,987982041447622	NA
0.801203611	0,423013781325284	NA
0.010307531	0,991775925491468	NA
0.931319134	0,351688507099104	NA
0.901952897	0,367081889701312	NA
0.07050321	0,943793145635277	NA
0.183743652	0,854214562889163	NA
-0.412008608	0,680333113937239	NA
0.780113674	0,435323968385091	NA
0.585946766	0,557911289048946	NA
1.60400116	0,108713796939097	NA
1.000793827	0,31692649471656	NA
0.121631939	0,903190517861181	NA
1.544661651	0,122428125404114	NA
-0.234847377	0,814327194177425	NA
0.980060619	0,327056197046118	NA
-0.884103967	0,376640092083238	NA
-0.280103706	0,779397941407889	NA
-0.14251526	0,886673026605681	NA
0.783769456	0,433175399465639	NA
1.855822321	0,0634788878860866	NA
-0.841551865	0,400038842067872	NA
-1.953309115	0,0507829780771742	NA
0.269457316	0,787577784819938	NA
0.295966013	0,767256036563774	NA
-0.313845417	0,753638448268303	NA
1.563923349	0,117835570540873	NA
-0.38544527	0,699907548229958	NA
0.424365242	0,671299465530685	NA
0.615006103	0,538550690006033	NA
-1.049711707	0,293850679512345	NA

2.014473346	0,0439598605360458	NA
0.56073395	0,574978919604697	NA
-0.722112353	0,470225403863508	NA
-0.309087734	0,757254789605779	NA
0.953777879	0,340196088814505	NA
0.009651572	0,992299279074313	NA
-0.26716839	0,789339513184304	NA
2.353537404	0,0185957368746839	NA
0.490406025	0,623846613961182	NA
1.843379399	0,065273635835604	NA
-2.464217017	0,0137312944058895	NA
-0.079578538	0,936572465128465	NA
-0.613749266	0,5393810275398	NA
1.723962372	0,084714636156025	NA
0.096800222	0,922885047642575	NA
-0.591162277	0,554411695569312	NA
0.455524069	0,64873225596804	NA
-1.429102143	0,152974877799899	NA
-0.307374529	0,758558313551316	NA
0.234647743	0,814482150234603	NA
0.556181265	0,578086945440189	NA
0.583698247	0,559423347115603	NA
1.638309421	0,101357163507368	NA
0.261307352	0,793855497422175	NA
0.113734131	0,90944855538987	NA
0.730211153	0,465261126220564	NA
-0.593171135	0,553066627936291	NA
0.38442402	0,700664200360883	NA
1.097557	0,272397978193859	NA
-1.119195136	0,263056900089232	NA
0.528410509	0,597214444941771	NA
0.539841676	0,58930622320781	NA
0.270854905	0,786502629252922	NA
2.273812412	0,0229772676478154	NA
0.771325763	0,440513865637694	NA
0.215214646	0,829599972919179	NA
-1.847187758	0,0647199329719087	NA
-0.643412647	0,519956379378711	NA
-0.195291443	0,845164795655131	NA
0.127838729	0,898276600214196	NA
-0.148466595	0,881974547362041	NA
1.688644466	0,0912875838642995	NA
-0.605811524	0,544639916616805	NA
-0.149118768	0,881459916458575	NA
0.730921709	0,464826974967939	NA
-0.959650559	0,337231114119436	NA
-0.605341912	0,544951838404339	NA
-0.858032074	0,390874752875799	NA
0.606965212	0,543873999260873	NA
0.987030421	0,323627725257984	NA

-0.248826483	0,803495004687049	NA
-0.665643608	0,505638925959697	NA
-0.173224406	0,862475040071298	NA
0.458292342	0,646742425340121	NA
-1.118525748	0,263342516020931	NA
-0.996150723	0,319176917830635	NA
-0.281372644	0,778424597803761	NA
-0.884364619	0,376499415698483	NA
0.757843312	0,448544789867325	NA
0.869170459	0,384753902854029	NA
-1.439705981	0,149950600573967	NA
0.387776441	0,698181481575282	NA
0.371440776	0,710309259762731	NA
0.458784795	0,646388714931239	NA
1.280068442	0,200521066302008	NA
1.680337784	0,0928916136496493	NA
0.665100281	0,505986354944645	NA
-0.243636116	0,807512654428441	NA
2.392898213	0,0167158777264001	NA
-0.748822531	0,453964177918532	NA
0.058924363	0,953012353218996	NA
1.278773538	0,200976815061964	NA
0.336343257	0,736612033665458	NA
0.327123358	0,743574585682299	NA
1.021882958	0,306836299198416	NA
0.622185357	0,533819993311307	NA
-1.406496945	0,159576611186625	NA
-1.048443431	0,294434352372352	NA
-0.058134958	0,95364112889577	NA
-0.985967003	0,324149305440974	NA
-0.860863541	0,389313203775819	NA
0.785642669	0,432076859430125	NA
-1.545281894	0,122278092976618	NA
-0.545293327	0,585551793109878	NA
-0.58605967	0,557835416666695	NA
-0.594902326	0,551908762252932	NA
-1.215204939	0,224287937189815	NA
-1.112037772	0,266121910284998	NA
-0.105524509	0,915959623124053	NA
0.062901389	0,949845028478963	NA
-0.223647017	0,823031975711968	NA
-0.713674805	0,475428289341777	NA
-0.302263118	0,762451492080316	NA
-0.675941707	0,499077656060176	NA
0.59147145	0,554204579338844	NA
-0.570314564	0,56846436480248	NA
-0.575440114	0,564993681127101	NA
0.051721079	0,958750940933511	NA
1.465633853	0,142748063540376	NA
1.414253042	0,157287619032332	NA

-0.050291786	0,959889869605294	NA
-1.117422358	0,263813779763676	NA
0.330656667	0,740903837356547	NA
0.336796799	0,736270086050447	NA
1.216202428	0,223907818916804	NA
0.114314302	0,908988645244437	NA
0.71892419	0,472187631017017	NA
0.502310667	0,615449007151611	NA
0.405821183	0,684873992873363	NA
1.006566984	0,314142907277187	NA
-0.814792261	0,415191231326797	NA
-0.387358538	0,698490794653296	NA
-0.200115585	0,841390185204907	NA
1.111538111	0,266336795074344	NA
0.701891021	0,482747128789166	NA
0.666751012	0,504931188699489	NA
0.746559701	0,455329381733981	NA
0.792780013	0,427906003587704	NA
-1.462560042	0,14358780359761	NA
0.665296384	0,505860943228799	NA
1.154178916	0,248426824678969	NA
0.667694022	0,504328926470333	NA
1.412051167	0,157934895937705	NA
0.045107774	0,964021405023611	NA
-0.266598144	0,789778584712994	NA
-0.59923896	0,549013545818602	NA
0.328046929	0,742876179813384	NA
-1.329881451	0,183557333783861	NA
1.275443567	0,20215228637356	NA
-0.651773934	0,514547019101302	NA
-0.223453108	0,823182875103074	NA
-0.04605926	0,963263017156446	NA
-1.562988604	0,118055275384409	NA
-0.777531066	0,436845513026825	NA
0.317774614	0,750655908553908	NA
0.695898552	0,48649236404124	NA
1.350650285	0,176807484656312	NA
0.386101471	0,699421520308068	NA
0.384640311	0,700503923702815	NA
-0.148870506	0,881655814611934	NA
0.535105147	0,592577150699008	NA
0.134957019	0,892645856969968	NA
-0.938946035	0,347758452224153	NA
0.758493962	0,448155326193473	NA
0.340469491	0,733502994259459	NA
0.885391363	0,375945589028277	NA
-0.113399202	0,909714072186787	NA
-0.484631285	0,627937917749976	NA
0.496748421	0,619366472248681	NA
0.041372711	0,966998767749237	NA

0.038036167	0,96965884553897	NA
-0.162965536	0,870545570281435	NA
1.353752305	0,175815414809502	NA
0.07411835	0,940916214589827	NA
-0.705594376	0,480440422704399	NA
-0.813919596	0,41569101400528	NA
0.469214536	0,638916296582482	NA
-0.588762872	0,556020355135229	NA
-0.062854822	0,949882110292693	NA
-4.239530209	2,23988051298883e-05	NA
0.337475831	0,735758228702223	NA
0.551653656	0,581185667366499	NA
0.287723518	0,773558386027976	NA
0.361398149	0,717801831876041	NA
0.388563689	0,697598931912715	NA
0.822990819	0,410513214402696	NA
0.049535903	0,96049222549191	NA
0.494612468	0,620873699340963	NA
-0.686430799	0,492441488254939	NA
-1.176401318	0,239434545476967	NA
1.302278679	0,192821137273317	NA
0.712212939	0,476332926021939	NA
-0.729697534	0,465575088988429	NA
0.46469007	0,642153420152386	NA
0.065686738	0,947627231282719	NA
-1.905403304	0,0567276660089532	NA
0.558411573	0,576563371601674	NA
-0.631682193	0,527594566067735	NA
1.263426271	0,206436027650778	NA
0.126213206	0,899563157817523	NA
-0.38656155	0,699080827445692	NA
-0.342819824	0,731734011190055	NA
-0.078172131	0,937691130569478	NA
-0.86852017	0,385109635067571	NA
-0.859777504	0,389911702238065	NA
0.651885754	0,514474875938983	NA
0.384666228	0,70048471974268	NA
-0.810113225	0,417875104164901	NA
-1.389018219	0,164827210013512	NA
-0.23083705	0,81744139649372	NA
0.272460984	0,78526758717161	NA
0.482858226	0,62919640485257	NA
0.754837543	0,450346464722187	NA
0.428985856	0,667933518178113	NA
1.261870649	0,206995335020977	NA
0.691041422	0,48953951075309	NA
0.528398484	0,597222789263303	NA
-0.042983478	0,965714704021281	NA
2.808168695	0,00498241194174419	NA
1.411419701	0,158120897384248	NA

-0.362060432	0,717306870877824	NA
1.233277838	0,217472123400128	NA
-1.065638207	0,286587220756307	NA
0.67246086	0,501290349358283	NA
0.932542918	0,35105601759042	NA
2.568385545	0,0102173446873271	NA
-1.837043444	0,0662034822552925	NA
1.054239863	0,291773113488062	NA
0.508271587	0,61126289332937	NA
0.869611946	0,384512507136085	NA
1.266845634	0,205210491122134	NA
0.258501461	0,79601992154591	NA
2.723741583	0,00645469892647074	NA
-0.220991207	0,825099279691037	NA
1.311579695	0,189661987949097	NA
-1.155037395	0,248075113389966	NA
0.75533264	0,450049419177762	NA
1.144786468	0,252297622348756	NA
0.665751069	0,505570225011266	NA
0.443230124	0,657599291897674	NA
2.339802871	0,019293920616492	NA
0.733441151	0,463289408241342	NA
0.807318334	0,419483103630966	NA
0.402233655	0,687512075953772	NA
-1.104780398	0,269254756249006	NA
-1.008304083	0,313308503498633	NA
-0.128888718	0,89744570369119	NA
-0.605439653	0,544886910678631	NA
-0.356975067	0,721110471069187	NA
0.700041181	0,483901586885164	NA
0.387679461	0,698253256977465	NA
0.111721851	0,911043953046738	NA
3.331434874	0,000863995138826491	NA
-0.905083758	0,365421015762456	NA
-1.05869487	0,289738764815849	NA
-0.003006091	0,997601490036605	NA
0.749802485	0,453373671785596	NA
0.753668261	0,451048444887106	NA
-0.183358837	0,854516471660214	NA
0.650256382	0,515526626517205	NA
-1.725046708	0,0845190553467495	NA
-0.183665183	0,854276124213846	NA
-0.050339838	0,959851577753557	NA
-1.259898528	0,207705969683971	NA
-0.473967097	0,635523370521586	NA
0.142359554	0,886796008135042	NA
0.036268332	0,971068400661419	NA
0.786814285	0,431390589804221	NA
1.124384057	0,260850132494815	NA
-1.729271518	0,0837605116773871	NA

-0.095083742	0,924248311481039	NA
2.054804481	0,0398978932046339	NA
-0.011993224	0,990431021287257	NA
-0.426273499	0,669908566407629	NA
-0.647544048	0,517279895970514	NA
0.711716257	0,476640498914828	NA
0.500319117	0,616850395272644	NA
0.736070908	0,461687546121036	NA
-1.374427862	0,169308891289399	NA
-0.555547011	0,578520563978843	NA
-0.698939048	0,484590123501027	NA
0.602348655	0,546942079855271	NA
1.34423812	0,178871398439706	NA
-0.886132691	0,37554602877724	NA
0.472770771	0,636376729551151	NA
-1.350902467	0,17672667796025	NA
0.765019897	0,444259696449127	NA
-0.42809769	0,668579998701564	NA
-0.567051089	0,570679471861452	NA
2.177671301	0,0294305169764653	NA
1.748083675	0,08044953936108	NA
1.106520576	0,268501262983327	NA
0.148620811	0,881852850683634	NA
0.168947927	0,86583760074571	NA
1.762204552	0,0780347433262223	NA
1.270974078	0,203737870824247	NA
1.482000884	0,13834005962629	NA
1.035329187	0,300515203729561	NA
-1.612827954	0,1067818911291	NA
1.309674324	0,190306033163842	NA
0.756997076	0,449051614133169	NA
2.301781871	0,0213474764251988	NA
0.899861339	0,368194046514818	NA
-0.18385759	0,854125176192184	NA
1.07025219	0,284505808095036	NA
0.626234015	0,531161459727126	NA
0.090601591	0,927809167433369	NA
1.234780523	0,216912198003813	NA
0.198017258	0,843031560941993	NA
0.320698409	0,748438953267613	NA
0.976307095	0,32891230500094	NA
0.216560041	0,828551229171171	NA
-0.474379356	0,635229411638264	NA
0.174733877	0,861288747357721	NA
1.194993779	0,232089460706048	NA
0.79269072	0,427958038173212	NA
0.520600789	0,602644899291771	NA
-0.037987038	0,969698016816032	NA
2.617782019	0,00885033213149821	NA
-0.051189021	0,959174900221426	NA

-1.110580756	0,266748849810105	NA
0.391317088	0,695562869442853	NA
0.764189963	0,444754046729285	NA
1.088748782	0,276264683399585	NA
0.638072069	0,52342676766465	NA
-0.222706338	0,823764068567344	NA
1.205486946	0,228015374455479	NA
-0.200877499	0,840794362050735	NA
1.105635088	0,268884496266668	NA
-0.353911691	0,723405065125022	NA
2.134868522	0,0327717512077242	NA
1.45688026	0,145149436039081	NA
-2.041930475	0,0411584283163448	NA
-0.036549431	0,970844264412983	NA
0.964131282	0,334980111195143	NA
-0.696179596	0,486316364122212	NA
-0.260125048	0,794767317217164	NA
0.582337067	0,560339662711764	NA
1.414799964	0,157127154794078	NA
0.580871673	0,561326945442217	NA
-1.258496514	0,208212247137999	NA
0.45693649	0,647716695515736	NA
1.307847539	0,190925026157995	NA
-1.417921551	0,156213669704626	NA
0.168741273	0,866000153238925	NA
-0.12300859	0,902100295481506	NA
1.929179263	0,0537086116833948	NA
-0.306775881	0,759013968110346	NA
-2.032218216	0,0421315696243438	NA
1.226506395	0,220008170304979	NA
-1.102485974	0,270250452747755	NA
-0.301553351	0,762992573855733	NA
-1.180583784	0,237768108617108	NA
-0.588705432	0,556058893046307	NA
1.803952875	0,071238694814192	NA
-2.543696947	0,0109686202679446	NA
-0.270079238	0,787099294368137	NA
0.826887107	0,408301051848084	NA
0.510075636	0,609998473236288	NA
0.186200312	0,852287679689083	NA
-0.852436089	0,393972097058749	NA
2.065509577	0,0388748103557938	NA
-0.314594047	0,753069899447277	NA
-0.579585563	0,562194131391611	NA
-0.676469376	0,498742682839834	NA
0.47962712	0,631492558745897	NA
1.330800577	0,183254637976826	NA
0.688459951	0,49116318195128	NA
1.004088105	0,315336148537669	NA
-2.1558626	0,0310943955864188	NA

-1.382891114	0,166698255255377	NA
0.939234246	0,347610489896202	NA
0.488643565	0,625094064815337	NA
1.649752462	0,0989935754033945	NA
0.139129048	0,889348174703931	NA
-0.053386531	0,957423936236484	NA
-0.006335964	0,994944666133827	NA
0.387091206	0,698688687922169	NA
0.228314874	0,819401462026574	NA
0.148235782	0,882156693611454	NA
-0.011650842	0,990704183365138	NA
1.305027438	0,191883505009596	NA
0.228580951	0,819194631730488	NA
-1.569047155	0,11663695173547	NA
1.418108	0,15615923603299	NA
-1.206688107	0,227552281098944	NA
-0.20748726	0,835629341138873	NA
1.227167984	0,219759459932093	NA
-1.081916622	0,279289578445004	NA
0.487762999	0,625717722862933	NA
-0.997284533	0,318626419189062	NA
0.894119828	0,371257781330393	NA
1.182070939	0,237177554153825	NA
0.429288199	0,667713503935242	NA
-0.641667198	0,521089291828739	NA
1.147337417	0,251242195251437	NA
-1.598357352	0,10996347030482	NA
-0.188324016	0,850622660140837	NA
0.708675552	0,478525839599354	NA
0.322999913	0,746695307718313	NA
1.034389805	0,300953968458429	NA
-2.256711567	0,0240261036257865	NA
-0.030430324	0,975723861235478	NA
-0.362851404	0,716715890163237	NA
0.388115587	0,697930497879826	NA
-1.147807432	0,251048068592332	NA
-0.34971945	0,726549255405127	NA
1.315170989	0,188452442829368	NA
-1.20976046	0,226370822468799	NA
0.010063586	0,991970555249665	NA
0.419345819	0,67496341477313	NA
0.237819736	0,81202091100315	NA
-0.878834909	0,37949079544112	NA
1.294483198	0,195498569299295	NA
-0.260428796	0,794533032759519	NA
0.13232061	0,894730705912786	NA
-0.549106406	0,582932429522079	NA
0.404765293	0,685650042646274	NA
-0.141933951	0,887132176865637	NA
0.604278215	0,545658687339341	NA

0.453996264	0,649831516015335	NA
-0.864429199	0,387352158750504	NA
0.41646824	0,677067402791287	NA
0.234785318	0,81437536367296	NA
0.729953393	0,465418673819609	NA
0.354847459	0,72270387187003	NA
0.334075801	0,738322360329707	NA
-0.565017246	0,572062033804884	NA
-1.076846515	0,281548840703171	NA
0.309101956	0,757243971057379	NA
0.224631129	0,822266246249562	NA
-0.374985561	0,707671205104481	NA
-1.580610355	0,113967156621431	NA
-0.353020448	0,724073111105425	NA
-0.95608011	0,339031762587991	NA
0.719641747	0,471745601490487	NA
1.001296187	0,31668363567049	NA
-0.277917586	0,781075623669921	NA
-0.273843367	0,784204995182366	NA
-1.867512044	0,0618301165400415	NA
-0.677795934	0,497901089289333	NA
1.339296955	0,180474019787053	NA
1.762115894	0,0780497183484586	NA
0.120604628	0,904004202978821	NA
1.264328819	0,206112029814023	NA
0.635190379	0,525304257279157	NA
0.206620658	0,836306125870975	NA
-0.123251258	0,901908136554744	NA
-1.107880622	0,267913375555491	NA
0.652702114	0,513948339689639	NA
0.913860024	0,360790401846606	NA
0.253673175	0,799748059433473	NA
1.751080587	0,0799320296893543	NA
0.293107893	0,769439683906184	NA
-0.395035535	0,6928166633097	NA
1.746207325	0,0807749325122661	NA
-0.761626467	0,446282973608827	NA
1.439783112	0,149928770553647	NA
2.964611642	0,00303065254046695	NA
-1.860410703	0,0628274416234035	NA
-2.125760316	0,0335232218418749	NA
-0.279465449	0,779887649696079	NA
-0.502275603	0,615473668373199	NA
1.355309704	0,175318904485628	NA
1.254395404	0,209698322106924	NA
-1.018245189	0,308561449069274	NA
0.869639693	0,38449733884816	NA
0.278092366	0,78094145602751	NA
-0.006400658	0,994893049062354	NA
1.054276436	0,291756373981264	NA

-1.143684797	0,252754380384178	NA
1.17740476	0,23903399101354	NA
-0.202628812	0,839425168461475	NA
-1.277738617	0,201341604192375	NA
0.272103436	0,785542487475688	NA
0.601457675	0,547535193890275	NA
-1.417039841	0,156471279539482	NA
-1.435259989	0,151213024897783	NA
0.985189493	0,324531000915392	NA
-1.515869192	0,129552431739642	NA
-0.583039849	0,559866474884527	NA
0.934229574	0,350185483369493	NA
-3.035076964	0,00240474312523125	NA
-0.725952391	0,467867957053203	NA
-1.451926857	0,146521943949787	NA
1.468557388	0,14195287974663	NA
-0.784498521	0,432747649156708	NA
0.823810557	0,410047208268627	NA
1.967135275	0,0491676174423596	NA
-0.263566075	0,792114302504252	NA
-1.305941849	0,191572333705739	NA
-1.134438733	0,256610559425225	NA
-0.199637433	0,841764149899647	NA
-0.865864372	0,386564543236711	NA
0.973786926	0,330162345857114	NA
0.400710678	0,688633148368669	NA
0.091037981	0,927462412249299	NA
2.041288841	0,0412221256296612	NA
0.083935502	0,933107712432454	NA
-0.057504351	0,954143440281274	NA
0.690623105	0,489802423808905	NA
0.270315713	0,786917378026308	NA
-1.848512755	0,0645282008842682	NA
-0.229217938	0,81869953131751	NA
0.000295718	0,99976405153846	NA
0.767204272	0,442960073515981	NA
0.221483547	0,824715945814573	NA
0.457393056	0,647388554447137	NA
-0.761901056	0,446119059854399	NA
0.182111071	0,855495560882232	NA
0.697534218	0,485468532994061	NA
0.108370215	0,91370202708523	NA
-0.671105505	0,502153320319585	NA
-0.415708206	0,677623535498641	NA
0.510637862	0,609604658567674	NA
-0.104849219	0,91649545426672	NA
0.188294395	0,850645878662993	NA
-0.446769266	0,655041664410936	NA
-0.400406512	0,688857128439568	NA
1.331356412	0,183071764304914	NA

-0.114858047	0,908557638904585	NA
-1.850415671	0,0642536620778513	NA
-0.476921742	0,633417849304557	NA
-0.077510722	0,93821726224436	NA
0.448378699	0,653879911974355	NA
-1.712604374	0,0867853480400437	NA
1.074687835	0,282514511873132	NA
0.652984254	0,513766429542004	NA
-1.542090701	0,123051554420132	NA
-1.076237358	0,281821116166262	NA
1.425658448	0,153966956226357	NA
0.247946346	0,804175920498707	NA
-0.370749976	0,710823763063341	NA
-1.069688257	0,284759653818169	NA
-2.766488819	0,0056663527994186	NA
0.450348961	0,652458840625517	NA
-1.541167636	0,123275992035182	NA
-0.648231992	0,516834913215263	NA
-1.709226976	0,0874089138207216	NA
0.956012449	0,339065945049361	NA
-0.283308733	0,776940183366501	NA
-0.959534661	0,337289466996439	NA
-0.521635626	0,601924053414118	NA
0.421842155	0,673140233715536	NA
-0.474983065	0,63479904371806	NA
-0.692015656	0,48892749690162	NA
0.0645595	0,948524732043782	NA
-1.194442367	0,232304972550557	NA
0.814589329	0,41530742043788	NA
0.579895169	0,561985314058962	NA
0.648439839	0,516700510144063	NA
2.078482496	0,0376649414824105	NA
0.343291355	0,731379283821341	NA
0.664346517	0,506468555151655	NA
-0.609939977	0,541901569422856	NA
-0.45112448	0,651899835443454	NA
0.285578176	0,775201220984876	NA
1.178643787	0,23854004821373	NA
1.343521877	0,17910304705323	NA
-0.466569911	0,64080761863318	NA
1.148290672	0,250848588574226	NA
-0.159882405	0,872973708754178	NA
-0.532371558	0,594468680061667	NA
1.752709899	0,0796518155953732	NA
0.153961178	0,877640345816858	NA
-1.658475461	0,0972215365648993	NA
0.25882388	0,795771131905388	NA
-0.994274235	0,320089375521295	NA
1.713941247	0,0865395167442501	NA
-0.169728272	0,865223838711052	NA

1.034889448	0,300720543043474	NA
0.194582571	0,845719748597025	NA
-0.137315974	0,890781044336416	NA
0.266017068	0,790226064306876	NA
-2.314241426	0,0206544827840024	NA
0.17001052	0,865001863279527	NA
2.765408486	0,00568515544509868	NA
-0.300641049	0,763688226324434	NA
-0.135591935	0,892143881752247	NA
-0.213406667	0,83100978047331	NA
-1.081426988	0,279507222075101	NA
1.070497436	0,284395462351894	NA
0.875243277	0,381441550246175	NA
0.109673371	0,912668420166941	NA
0.620136688	0,535167799549954	NA
1.24168699	0,214352072075942	NA
1.109882018	0,267049870297917	NA
-1.763340683	0,077843047685322	NA
-2.693813318	0,00706397174882624	NA
0.458788392	0,646386131170182	NA
-1.640218857	0,100959669914639	NA
1.602978456	0,108939410730698	NA
-0.787588047	0,430937708622725	NA
0.452677951	0,6507806586005	NA
-1.463690036	0,143278659226034	NA
1.261082165	0,207279245477909	NA
-0.633666554	0,526298456820612	NA
0.823299459	0,410337721480181	NA
0.932433701	0,351112434965874	NA
-1.441137288	0,149545900976423	NA
0.366152892	0,714250991667231	NA
-0.150970618	0,879998893338593	NA
0.709422167	0,478062535497656	NA
1.176760865	0,239290966883155	NA
-1.10031248	0,271195996609092	NA
2.005261746	0,0449350721271152	NA
0.693786596	0,487816049650516	NA
0.231648634	0,816810930205363	NA
-0.114904604	0,908520735926649	NA
-1.68904037	0,0912116938431036	NA
1.077571605	0,281224979668075	NA
0.858205567	0,390778962685137	NA
-1.656392884	0,0976422813177774	NA
-0.856509487	0,391716027581339	NA
0.276559747	0,782118177012422	NA
-0.628950267	0,529381615879976	NA
-1.545631029	0,122193702920223	NA
0.682307427	0,495044577731291	NA
-0.852627297	0,393866019796351	NA
-0.225857861	0,821311972850018	NA

-0.513230468	0,607790115997514	NA
0.572856472	0,56674187890304	NA
2.137093352	0,0325903995438089	NA
2.181766698	0,0291267555194024	NA
1.155235476	0,247994010759194	NA
1.119007218	0,263137059843297	NA
0.848680498	0,396059100887512	NA
-1.425412395	0,154038027122615	NA
0.384154557	0,7008638969482	NA
0.488732987	0,625030747011615	NA
-0.781574107	0,434464908671677	NA
-1.718350819	0,0857326451404657	NA
-0.472738464	0,636399780965751	NA
-2.451892348	0,0142107169325141	NA
1.864224181	0,062290228959325	NA
0.004291265	0,99657607664418	NA
1.017020498	0,309143678211475	NA
-0.960907994	0,336598430820018	NA
0.165446789	0,868592328560411	NA
-0.038851304	0,969008940798698	NA
0.422926382	0,672348973837433	NA
0.537861411	0,59067273104268	NA
0.889221942	0,373883812779568	NA
0.249199985	0,803206090930636	NA
0.598401476	0,549572081376469	NA
-0.437650711	0,661639504284144	NA
0.7341603	0,462851046105491	NA
0.235931774	0,81348561231718	NA
-0.721229617	0,470768252659661	NA
-1.068868451	0,285128949719376	NA
0.458240871	0,646779399742674	NA
0.198508822	0,842646983409988	NA
0.840915474	0,400395288962093	NA
-0.091804577	0,926853307848147	NA
-0.811128815	0,417291699496255	NA
2.23572151	0,0253700191574874	NA
-0.636044175	0,524747629509481	NA
-0.854925166	0,392592577792786	NA
1.818042157	0,0690576897122788	NA
1.073851162	0,282889395174073	NA
1.634104476	0,102236915188733	NA
-0.231164031	0,817187372127993	NA
-0.469057408	0,639028602246727	NA
-0.153210685	0,878232131395376	NA
-0.317285947	0,751026639630903	NA
0.195120558	0,84529856914506	NA
-0.656640955	0,511411808098229	NA
-2.271640805	0,0231082141955536	NA
0.856862489	0,391520885434106	NA
-0.482487509	0,629459669911811	NA

0.595394485	0,551579810892104	NA
-0.722221027	0,47015859772217	NA
-3.410877216	0,000647542444149796	NA
0.047568473	0,962060158701801	NA
-0.040919084	0,967360403555717	NA
-0.008085355	0,993548890727925	NA
1.153712931	0,248617880696769	NA
-0.554293004	0,579378337697073	NA
-1.240266022	0,214877015593684	NA
0.759503522	0,447551408216112	NA
0.317162575	0,751120245476583	NA
0.780992793	0,434806733495543	NA
0.534547998	0,592962450804537	NA
-0.897412532	0,369498827879844	NA
-0.104070974	0,917113025294282	NA
-0.488051944	0,625513048994474	NA
1.618599721	0,105533415250466	NA
-0.063550691	0,949327994870581	NA
-1.269875988	0,204128807769419	NA
0.424877463	0,670926004184135	NA
0.249937742	0,802635495228609	NA
0.745285393	0,456099209857551	NA
0.137775762	0,89041764101781	NA
-0.627891433	0,53007506331421	NA
0.24382594	0,807365629002173	NA
1.357493648	0,174624410532784	NA
-0.430804943	0,666610205294803	NA
-1.236084597	0,216427119806332	NA
-1.767412783	0,0771591237353537	NA
1.876982011	0,0605205593665834	NA
-0.747356814	0,454848206489378	NA
-1.288304762	0,197639896023305	NA
-0.963587522	0,335252765156766	NA
-1.132539476	0,257407697821994	NA
1.445198543	0,148402125597833	NA
1.545668255	0,122184707822193	NA
0.378217939	0,705268700219454	NA
1.808632196	0,0705081632265896	NA
-1.954430007	0,0506503848965879	NA
1.030789824	0,302639391274543	NA
-0.031750887	0,974670713309996	NA
0.135748214	0,892020331595427	NA
0.170445345	0,864659913787296	NA
-1.549766059	0,121197676247049	NA
-0.44888256	0,653516377383228	NA
0.104077816	0,917107595627018	NA
0.040177724	0,967951436732422	NA
-0.291067195	0,77099992892303	NA
0.125211287	0,90035628190241	NA
0.616963767	0,537258625085456	NA

1.890235388	0,0587264857511604	NA
-0.076912852	0,938692873536155	NA
-1.166776318	0,243300713298115	NA
-1.162834312	0,244896732892217	NA
-1.666415638	0,0956306581998072	NA
0.814677413	0,41525698551532	NA
0.555554141	0,578515688551245	NA
0.040435697	0,967745771114961	NA
0.237818439	0,812021916788307	NA
-1.273650396	0,202787343479912	NA
-0.653834928	0,513218160788623	NA
1.075714229	0,282055081991493	NA
-0.452004032	0,651266078052139	NA
0.561788146	0,574260369501877	NA
1.378201985	0,168140934688998	NA
-1.139451644	0,254514826057126	NA
-1.560745248	0,118583870529413	NA
0.222955564	0,823570090568071	NA
-2.445020488	0,0144843913667452	NA
1.399846335	0,161559339129563	NA
1.053807235	0,291971180591969	NA
-0.321006772	0,748205258662163	NA
0.709106067	0,478258658271685	NA
0.596171684	0,551060540089362	NA
1.383094345	0,166635939399207	NA
0.85049523	0,395049811231651	NA
-0.030185168	0,975919377030081	NA
1.448678068	0,147427508089164	NA
1.535517097	0,12465686081104	NA
1.293168123	0,195952917943759	NA
-1.8987103	0,0576025765393068	NA
0.584934863	0,558591515743828	NA
-0.518013324	0,604448972930022	NA
0.083997519	0,933058404525523	NA
-0.167550604	0,8669368338549	NA
0.600600172	0,548106323392968	NA
-0.051200589	0,959165682152257	NA
0.159319873	0,873416864210768	NA
0.805302247	0,420645285179437	NA
-0.541346231	0,588268960706038	NA
0.008007443	0,993611053465824	NA
-0.702696106	0,482245154845259	NA
-0.657305553	0,510984466646218	NA
-0.455096643	0,649039712891129	NA
-0.428033485	0,66862674139801	NA
0.669405936	0,503236566097905	NA
1.207875932	0,227094988751635	NA
-1.757193082	0,0788849040673732	NA
-1.582669217	0,113496878024147	NA
1.878314143	0,0603382029321417	NA

0.311766024	0,755218346655372	NA
0.273377226	0,784563257347993	NA
0.817053109	0,413898078443367	NA
-0.294354299	0,76848718507205	NA
-0.094598263	0,924633929768954	NA
-1.007178021	0,313849232951969	NA
-2.676568872	0,00743802768397731	NA
0.142246506	0,886885298621784	NA
-1.542083141	NA	NA
0.481711993	0,630010555315704	NA
0.802988235	0,421981534472146	NA
-1.747108005	0,0806186051286304	NA
-2.217456699	0,0265918988727118	NA
-2.201707433	0,0276859813668537	NA
-1.931999615	0,0533595533901531	NA
-1.919522443	0,0549182488306642	NA
-1.611974936	0,106967393996405	NA
-1.387660685	NA	NA
-1.036210928	0,300103749756174	NA
1.22784292	0,219505940217611	NA
-0.587800203	0,55666640797913	NA
0.288561426	0,772917018043108	NA
0.087248449	0,930474029076773	NA
-0.810425478	0,417695679566783	NA
-2.741994472	0,00610673694194107	NA
-0.194744366	0,84559307760874	NA
-2.59928924	0,00934170234650728	NA
-0.527175541	0,598071692019854	NA
-0.174420044	0,861535362922084	NA
-1.295825883	0,195035478843336	NA
-1.941538032	0,0521930510411071	NA
0.453797234	0,649974774280821	NA
0.510155075	0,609942823129108	NA
-0.473078834	0,636156936491073	NA
-0.628485129	0,52968618558887	NA
0.200279623	0,84126189816134	NA
-0.108731857	0,913415173235007	NA
0.539461818	0,589568235861882	NA
-0.824953937	0,409397742925735	NA
0.319370729	0,749445404155499	NA
-0.637912248	0,523530804359484	NA
-0.78349904	0,433334117255317	NA
0.183091843	0,85472595574946	NA
0.393467059	0,693974548996876	NA
-0.65316031	0,513652934411812	NA
-1.611106128	0,107156592979352	NA
-0.985562787	0,324347706404228	NA
0.436129913	0,662742475419071	NA
-1.458741785	0,144636190752813	NA
0.68689144	0,492151141773117	NA

-0.659780909	0,509394436235311	NA
-1.351488193	0,176539099597027	NA
0.055733923	0,955553774774905	NA
0.379628327	0,704221330711255	NA
-0.756356613	0,449435413637506	NA
0.837428255	0,402351889060453	NA
-2.053860831	0,0399891633272895	NA
-2.610353393	0,00904487317604161	NA
0.753235229	0,451308573117765	NA
-0.046978006	0,962530757001179	NA
0.2697903	0,78732158600604	NA
-0.719169288	0,472036620069615	NA
0.255484579	0,798348852461738	NA
0.541738112	0,587998930853983	NA
0.049453868	0,960557600238954	NA
-1.192362062	0,233119309199604	NA
-0.646781914	0,517773098994072	NA
0.075998345	0,939420414537066	NA
0.048504207	0,961314411687897	NA
0.430668417	0,666709486114068	NA
0.833054709	0,404813875440741	NA
-0.411766878	0,680510300994421	NA
0.516070623	0,605805084941079	NA
0.131370791	0,895481993526766	NA
-0.886814179	0,375178952520933	NA
-0.83958858	0,401139104429723	NA
1.387700775	0,165228179911871	NA
1.379318676	0,167796520638219	NA
-1.556700454	0,119541620808982	NA
2.664187654	0,00771744982529544	NA
-0.266123043	0,790144448745734	NA
-0.430184779	0,667061233245701	NA
1.499290763	0,133798217610065	NA
1.441069917	0,149564931218117	NA
0.554621981	0,57915325116987	NA
-0.101720004	0,918978923590279	NA
0.265780642	0,790408152515041	NA
1.021589499	0,306975229632283	NA
0.851774193	0,394339432418216	NA
0.058571869	0,953293117180142	NA
-0.77680864	0,437271678806953	NA
0.64253953	0,52052293150286	NA
0.170985296	0,864235327161638	NA
-0.42327881	0,672091853010779	NA
0.293613574	0,769053201886337	NA
-0.584600595	0,5588163077933	NA
-1.030939174	0,302569344912042	NA
-0.629028596	0,529330334974723	NA
-1.779374941	0,0751783119799477	NA
-0.203690318	0,838595508909602	NA

0.792254711	0,428212173211009	NA
-0.979691761	0,327238294087089	NA
0.090131587	0,928182648259558	NA
0.033361979	0,973385928819173	NA
0.190999856	0,848525706256386	NA
-0.214155898	0,830425486124316	NA
0.433732213	0,664482911611883	NA
0.472455891	0,636601418736774	NA
0.61692531	0,537283991388412	NA
-0.668423892	0,503863048708708	NA
0.397642065	0,690894048346138	NA
0.06168275	0,950815477627523	NA
-0.969071632	0,33250945048905	NA
1.184214558	0,236328140616604	NA
1.185895505	0,235663568430645	NA
1.069285379	0,284941096687619	NA
0.549299579	0,582799876489433	NA
1.474210575	0,14042490348474	NA
-0.499032612	0,61775640936915	NA
0.818893271	0,412847309198766	NA
1.021414938	0,307057891061092	NA
0.00756255	0,993966015572588	NA
-1.028900784	0,303526299936517	NA
0.721856697	0,470382587168151	NA
-0.368674868	0,712370083403271	NA
-0.306447565	0,759263897304162	NA
0.052816099	0,957878434132947	NA
-0.166772478	0,867549074968572	NA
0.68269812	0,49479761783434	NA
0.350554043	0,725922939746002	NA
-0.102676692	0,918219573249818	NA
0.532480015	0,594393579681952	NA
-1.139439949	0,254519701623521	NA
-0.048316731	0,961463820436206	NA
0.602504763	0,54683819334105	NA
-1.284109773	0,199103548956051	NA
1.398229801	0,162044070379371	NA
-0.371811561	0,710033155018639	NA
1.083768051	0,27846765412759	NA
-0.199707915	0,8417090229517	NA
-1.499540838	0,133733382634216	NA
-1.219873469	0,222512844853043	NA
-3.149455242	0,00163575162723552	NA
0.602633207	0,546752724487476	NA
-0.709683561	0,477900388440239	NA
-0.261149791	0,793976996156395	NA
0.903276644	0,366379089092116	NA
0.215774012	0,82916390635596	NA
0.619826524	0,535372003820045	NA
-0.664211533	0,50655493289656	NA

0.161071234	0,872037296042449	NA
0.7915642	0,428614828484143	NA
-0.102694437	0,918205489303701	NA
0.709999274	0,477704586081677	NA
-0.41689438	0,676755664225582	NA
-0.873724047	0,382268552195538	NA
0.013998881	0,988830874162801	NA
0.140611315	0,88817700873566	NA
-0.652785381	0,513894649781719	NA
-0.734342315	0,462740134224665	NA
0.362100777	0,71727672289223	NA
1.379994739	0,16758826474432	NA
0.888566739	0,374235973392014	NA
0.880783039	0,378435261618741	NA
-1.237763162	0,215803891505897	NA
0.856957308	0,391468479205104	NA
-0.088456546	0,929513820524194	NA
1.289903252	0,197084251549818	NA
-0.69404578	0,487653499298028	NA
-0.313665231	0,753775310888615	NA
0.198254112	0,842846252072588	NA
0.521526627	0,601999961255412	NA
0.611342033	0,540973169737456	NA
1.073298241	0,283137324127457	NA
0.330758277	0,740827078612711	NA
1.340574605	0,180058606940859	NA
1.766798135	0,0772620411217446	NA
0.420344509	0,674233799162328	NA
-1.261492611	0,207131420570917	NA
-0.876239084	0,380900073312245	NA
0.227762316	0,819831024457522	NA
0.202521843	0,839508784141597	NA
-0.646127528	0,518196769008839	NA
0.22832927	0,819390271665088	NA
-1.115609539	0,264589307341175	NA
0.144283855	0,885276327128172	NA
1.00568809	0,314565634047548	NA
0.150773851	0,880154114650144	NA
-3.951278045	7,77349467755641e-05	NA
0.183466171	0,854432259564295	NA
0.064482473	0,948586062411024	NA
2.156724544	0,0310271333896094	NA
-1.580289399	0,114040606424715	NA
-0.418466636	0,675605975654747	NA
-1.221193589	0,222012730867195	NA
0.070595804	0,943719450125512	NA
-0.99548151	0,319502132627469	NA
1.681567841	0,0926526710669351	NA
3.98815546	6,6589023934158e-05	NA
0.692785271	0,488444317056409	NA

0.878383176	0,379735811393878	NA
0.557521622	0,577171090555123	NA
0.346786084	0,728752032489326	NA
0.808903587	0,418570605726376	NA
-0.889615693	0,373672277116966	NA
0.95417078	0,339997202626182	NA
0.526040348	0,598860173446782	NA
0.748831969	0,453958488368517	NA
0.251494354	0,801431926939853	NA
-1.580262654	0,114046728764898	NA
-0.582060444	0,560525967884151	NA
0.967891001	0,333098809128043	NA
0.477202403	0,63321800019341	NA
0.299107807	0,764857789582739	NA
0.185805896	0,852596980745973	NA
-0.89905097	0,368625511884227	NA
0.246737139	0,805111662399565	NA
-0.621537685	0,534245907032921	NA
0.017549749	0,985998045003954	NA
0.426918827	0,669438451935574	NA
0.016494596	0,986839812918208	NA
-0.660453516	0,50896283899701	NA
0.611680744	0,540749004451837	NA
0.295933049	0,767281211377855	NA
0.919187931	0,357997282698555	NA
0.054623558	0,956438370425298	NA
0.583934857	0,559264140690048	NA
1.043301638	0,296808612551717	NA
2.252089047	0,0243166453396054	NA
0.894015208	0,371313754193878	NA
-1.53900494	0,123803090334165	NA
0.742303319	0,457903581117951	NA
1.406921575	0,159450645082079	NA
-0.875534401	0,381283200559637	NA
-1.660575803	0,0967986720519501	NA
0.665017482	0,506039311514766	NA
-0.078756239	0,937226512392189	NA
1.163634104	0,244572324272114	NA
0.114219432	0,909063848246117	NA
1.9897506	0,0466184156708953	NA
-0.66962255	0,503098435226497	NA
-0.234073406	0,814927991399197	NA
0.814017049	0,415635184379894	NA
-1.044873766	0,296081316052837	NA
0.415212949	0,677986020320764	NA
2.504038369	0,0122784718037801	NA
0.149908738	0,880836617985444	NA
-0.285465264	0,77528771335087	NA
-0.040112988	0,96800304750513	NA
0.445148962	0,656212107118668	NA

0.495474379	0,620265302876444	NA
-0.080968866	0,935466712429816	NA
-0.155896294	0,876114766351285	NA
-1.679008879	0,0931503133894621	NA
-1.219642366	0,222600478536275	NA
0.380666905	0,703450429207744	NA
-0.576630188	0,564189301834795	NA
-0.1471092	0,883045828336146	NA
-0.098548759	0,921496556422238	NA
-0.108126535	0,913895319418864	NA
-0.190055444	0,849265684228294	NA
-0.341324244	0,732859498909509	NA
-0.914963795	0,360210637270126	NA
-1.519004533	0,128761354944109	NA
-0.113562992	0,90958422545762	NA
0.261946214	0,793362909348438	NA
0.272633738	0,785134774782943	NA
-0.028632419	0,977157755754067	NA
1.257411529	0,208604656575516	NA
1.375965854	0,168832204966165	NA
0.009211753	0,992650188600135	NA
0.130551722	0,896129935957905	NA
0.731115195	0,46470879356615	NA
-0.174831275	0,861212212941044	NA
-2.089577134	0,0366558023856878	NA
2.211147148	0,0270256488961807	NA
0.893225843	0,371736241534766	NA
0.299385073	0,764646249872539	NA
-0.534566231	0,592949839562061	NA
-0.190044852	0,849273983996961	NA
1.366549983	0,171766403734151	NA
-0.078764833	0,937219676639991	NA
0.698472308	0,484881869847695	NA
-1.070688071	0,284309708432707	NA
0.131400751	0,895458294893978	NA
0.675365351	0,499443673317535	NA
0.980094646	0,327039401530314	NA
1.16461053	0,244176679650455	NA
0.174831341	0,861212160794966	NA
0.010085323	0,991953213225899	NA
-0.119300018	0,905037669614902	NA
0.95426957	0,339947207369041	NA
-0.408342254	0,683022421907738	NA
0.23841419	0,811559862582671	NA
1.223913054	0,220985028418426	NA
1.510405758	0,130939920165552	NA
1.552813753	0,120467636187335	NA
1.278116405	0,201208385424881	NA
-0.282849027	0,77729257046391	NA
-0.938516777	0,347978899240918	NA

-0.108576647	0,913538283828224	NA
0.200555449	0,841046196473904	NA
1.223899457	0,22099015863695	NA
-0.07512616	0,940114333818391	NA
1.447604131	0,147727795058412	NA
0.80188282	0,422620744861505	NA
1.12030311	0,262584617075007	NA
-0.611084051	0,541143937926394	NA
-0.541365126	0,588255939611761	NA
0.152889191	0,878485660083217	NA
-0.165466189	0,868577059881118	NA
0.285313426	0,775404027405915	NA
0.246296807	0,805452481361633	NA
0.753735513	0,451008052840345	NA
0.972884328	0,330610794810016	NA
0.553998643	0,579579774401964	NA
0.109872857	0,912510209200392	NA
-0.155176308	0,876682325223571	NA
0.850827042	0,394865437747913	NA
0.656842593	0,511282133926335	NA
-0.558336709	0,576614482726244	NA
0.869870847	0,384370989022381	NA
-0.038806953	0,969044301118524	NA
0.742271308	0,457922972143787	NA
0.91541299	0,359974861676234	NA
0.300424534	0,763853351802771	NA
-0.812700721	0,416389667707309	NA
1.126255773	0,260057272107506	NA
-0.509288684	0,610549888932713	NA
0.80312168	0,421904407837172	NA
-0.29659815	0,766773323792465	NA
-0.790723082	0,429105603820887	NA
-2.684493546	0,00726397856980009	NA
1.438697374	0,150236283616812	NA
-1.530959909	0,125779301345845	NA
2.043815036	0,0409718231860413	NA
-0.883967252	0,37671389093911	NA
0.41892226	0,675272948149699	NA
0.201840723	0,840041244097661	NA
0.171630878	0,863727730775591	NA
0.510539378	0,609673634518234	NA
0.244395216	0,806924746307429	NA
0.934990248	0,349793325156093	NA
2.215620062	0,0267175337271265	NA
-0.650938901	0,515085928320701	NA
1.350851241	0,176743090102896	NA
-0.702568445	0,482324733479293	NA
1.134159739	0,256727548815568	NA
0.433920952	0,664345844708511	NA
0.013058856	0,989580836683029	NA

0.399314748	0,689661301361577	NA
2.272015661	0,0230855644167601	NA
-0.006480757	0,99482914018998	NA
-0.228083502	0,81958132570684	NA
1.04476133	0,296133291287948	NA
-1.511213745	0,130734001507947	NA
0.597406161	0,550236241265827	NA
0.753634868	0,451068501121351	NA
0.174885584	0,861169538228198	NA
0.302378025	0,762363904358566	NA
0.930510069	0,352107053262571	NA
-0.077383602	0,938318385425472	NA
-0.783531243	0,433315214151588	NA
-0.801492505	0,42284658149052	NA
-0.52601655	0,598876708062094	NA
1.398958807	0,161825336252539	NA
0.911941078	0,361799735699124	NA
-0.142889155	0,88637772402492	NA
-0.381857879	0,702566784741997	NA
-0.306084355	0,759540419896184	NA
-0.281222406	0,778539820329539	NA
-1.364752415	0,172330879354965	NA
0.409421108	0,682230651867125	NA
0.5044545	0,613942027825418	NA
-0.22642266	0,82087270546392	NA
-0.666357092	0,505182881312908	NA
0.108072659	0,913938055732424	NA
-0.782173797	0,434112441346478	NA
0.365364572	0,714839283724667	NA
-0.198553747	0,842611837838791	NA
0.626052796	0,531280312601336	NA
-0.235559617	0,81377441237196	NA
1.156856596	0,247330954235495	NA
-0.118324576	0,905810485362079	NA
1.60750301	0,107944069734482	NA
-0.253058374	0,800223105029871	NA
0.889936786	0,373499830296219	NA
-0.676617356	0,498648764155371	NA
1.126502726	0,259952787190723	NA
-1.127140341	0,259683149438698	NA
1.626757519	0,103788607226952	NA
1.563871761	0,117847687481526	NA
0.11305605	0,909986118618886	NA
-0.169550748	0,865363459075126	NA
0.53150385	0,595069674093354	NA
-0.076450912	0,939060366388401	NA
0.836816746	0,402695582997598	NA
-1.061484482	0,288469781508802	NA
0.524869316	0,599674041127549	NA
1.381654188	0,167077907033907	NA

1.373957403	0,169454907183286	NA
-0.711156088	0,476987516556638	NA
0.419845031	0,674598666477643	NA
-0.676053124	0,499006916987752	NA
0.465154945	0,641820501103319	NA
0.604063011	0,545801750197349	NA
0.587221705	0,557054816870982	NA
-0.198297705	0,842812147405389	NA
-0.517340775	0,60491829517408	NA
0.498792855	0,617925321483441	NA
0.937402971	0,348551313453227	NA
1.05777645	0,290157371761476	NA
0.591203889	0,554383817172489	NA
0.828687254	0,407281399841756	NA
-0.090091554	0,92821446090073	NA
-0.180034659	0,857125358798148	NA
-1.060267001	0,289023147172156	NA
2.22843561	0,0258514806787753	NA
-1.424542162	0,154289589158202	NA
0.402207236	0,68753151699769	NA
1.271150314	0,203675178737371	NA
0.956682444	0,338727561959146	NA
-0.946117758	0,344088527092706	NA
0.838026109	0,402016039909483	NA
0.599044181	0,549143423196648	NA
-0.390072399	0,696483011658395	NA
-0.171188849	0,864075275001332	NA
-0.328011471	0,74290298914984	NA
0.205877954	0,836886246969573	NA
-1.48853052	0,136611039606381	NA
1.735866189	0,0825874973146205	NA
-0.317116124	0,751155490734367	NA
-0.436447478	0,662512098111155	NA
-0.733804399	0,463067959042607	NA
1.262660799	0,206711107820961	NA
-0.157587312	0,874782002148724	NA
1.999198068	0,0455869275439969	NA
0.987468451	0,323413041813158	NA
2.797833019	0,00514467018014819	NA
1.503763848	0,132642182726505	NA
0.158628022	0,873961950091512	NA
1.667107433	0,0954930443937578	NA
0.102374077	0,918459759451646	NA
-1.896830522	NA	NA
-0.638695838	0,523020821003773	NA
0.501449997	0,616054460668781	NA
1.237975292	0,215725222605774	NA
0.125596485	0,900051345612873	NA
0.766919318	0,443129487251084	NA
0.658399935	0,510281177305082	NA

0.528493206	0,597157061458954	NA
1.73870572	0,0820865393198667	NA
0.630128719	0,528610371013697	NA
0.931098893	0,351802411052083	NA
-0.324208866	0,745779910319032	NA
-0.362096343	0,717280036480428	NA
-1.489216903	0,136430261210921	NA
-0.243709024	0,807456183888076	NA
0.031094132	0,975194469500534	NA
-0.847620741	0,396649219515317	NA
2.339539398	0,0193075350691043	NA
-3.533986351	0,000409342216651139	NA
-2.134206332	0,032825894618981	NA
0.511440198	0,6090428532674	NA
-0.787597902	0,430931942228964	NA
0.689150935	0,490728289598132	NA
-0.025351145	0,979774878997334	NA
0.628963468	0,529372972896343	NA
-0.07303465	0,941778544305444	NA
0.386633661	0,699027434525147	NA
0.901623259	0,367257031341547	NA
-0.324555401	0,745517585977878	NA
1.441762887	0,149369275870515	NA
-0.571560216	0,567619954840549	NA
0.81615361	0,414412285544011	NA
0.199745158	0,841679894396118	NA
-0.919054741	0,35806694051787	NA
-2.482029354	0,0130636513240705	NA
-0.657434958	0,510901279749762	NA
-0.785180715	0,432347620659779	NA
0.253056421	0,800224613771432	NA
-0.2914846	0,770680720118341	NA
0.163279194	0,870298615216368	NA
0.139506494	0,889049924720076	NA
1.577487932	0,114683296674931	NA
0.351937819	0,724884895616223	NA
1.578460313	0,114459898676748	NA
-1.191029104	0,233642159809251	NA
-0.223252052	0,823339342063852	NA
-0.457153709	0,647560568008851	NA
2.356422432	0,0184519208586913	NA
0.381924053	0,702517698801475	NA
-1.011712887	0,311675351927694	NA
0.654146041	0,513017721618118	NA
-0.31534413	0,752500381944107	NA
1.496955716	0,134404781179975	NA
0.588324798	0,556314303710801	NA
0.288663795	0,77283867098987	NA
-0.546258738	0,584888094958653	NA
-0.098293049	0,921699597334699	NA

-0.184473926	0,853641680123051	NA
0.937919538	0,348285761681944	NA
-1.271147006	0,203676355349203	NA
-0.694912674	0,487110029574976	NA
0.040967906	0,967321481952919	NA
-0.34876881	0,727262880910864	NA
0.880090532	0,37881026794617	NA
1.150109542	0,250098756881888	NA
0.400094219	0,689087122274722	NA
-0.608698761	0,542724128620718	NA
-0.262049161	0,793283540833638	NA
0.342571165	0,731921097462426	NA
0.58361976	0,559476162505529	NA
-1.537107715	0,124266934125932	NA
2.052723803	0,0400993721763494	NA
1.034401368	0,300948565047871	NA
0.517540517	0,604778893118057	NA
-0.532161988	0,594613807865107	NA
-0.020032768	0,984017232964052	NA
-0.75100373	0,452650410920634	NA
2.795189582	0,00518692879848784	NA
1.380956268	0,167292407327899	NA
-0.137130435	0,89092769575025	NA
-1.447944244	0,147632644579587	NA
1.956987938	0,0503488864748798	NA
1.358135229	0,174420778586485	NA
-0.389148762	0,697166104155647	NA
-0.585326548	0,558328167632842	NA
0.438780717	0,660820434197597	NA
2.595045358	0,0094578436772809	NA
-0.779837657	0,435486437858299	NA
-0.309081392	0,757259613404688	NA
-0.465356809	0,641675958472125	NA
-0.317714585	0,750701446928453	NA
1.186871663	0,235278246145612	NA
-0.592722118	0,55336713637841	NA
0.749467378	0,4535755533662	NA
-0.777813726	0,436678834615075	NA
3.321811186	0,000894351920634098	NA
0.562757581	0,573599968862709	NA
-0.182005258	0,855578599528093	NA
0.01948199	0,984456604606513	NA
-0.955216729	0,339468107556743	NA
2.047133019	0,0406450257834624	NA
-0.543536704	0,586760326716983	NA
0.765862658	0,443758026745169	NA
-0.40847627	0,682924048668668	NA
0.122495495	0,90250661351254	NA
0.819427777	0,412542392287569	NA
-1.304298774	0,192131733332621	NA

1.024491955	0,305602968874896	NA
-2.301408284	0,0213685640937978	NA
-0.724185776	0,468951689681525	NA
-0.859388721	0,390126090367358	NA
-0.499928529	0,617125403350058	NA
0.627175139	0,530544437542579	NA
-0.158804329	0,873823038206662	NA
-0.46382539	0,642772851046238	NA
-1.130778576	0,258148299389301	NA
0.607556336	0,543481768121207	NA
-1.091233138	0,275170309915854	NA
-1.276357259	0,201829257923454	NA
1.177056128	0,239173104271969	NA
-0.76594039	0,443711772032977	NA
0.426127851	0,670014687032152	NA
0.376650463	0,706433380732715	NA
-0.695086073	0,487001362071685	NA
1.694502219	0,0901698904107557	NA
0.641580597	0,521145534236716	NA
-0.403703082	0,686431072705119	NA
0.415515375	0,677764662387216	NA
-1.033666252	0,301292215480124	NA
2.776485757	0,00549500425356622	NA
0.95144183	0,34138013425912	NA
1.901501108	0,0572364075060008	NA
-0.068481803	0,945402105148791	NA
-0.460487387	0,645166422951776	NA
1.155022074	0,248081387250795	NA
1.02660989	0,304604195143386	NA
0.842808206	0,399335717972281	NA
-0.759737751	0,44741135825272	NA
-0.216020394	0,828971850817506	NA
0.510747735	0,609527710862453	NA
-0.59853452	0,54948333266908	NA
0.545185833	0,585625714064123	NA
-0.662604936	0,507583607805245	NA
-0.974842947	0,32963817033347	NA
-0.247101418	0,80482973604892	NA
0.551324987	0,581410912353969	NA
0.806213057	0,420120011458134	NA
-0.183604352	0,85432384868092	NA
0.372878603	0,709238797717714	NA
0.274724025	0,783528271954632	NA
-1.53766154	0,124131392045913	NA
-0.013558516	0,98918220124013	NA
-0.417444724	0,67635314764769	NA
0.041212793	0,967126254795568	NA
0.269088465	0,787861607079484	NA
-0.543153247	0,587024293929272	NA
1.855188119	0,063569367730077	NA

-0.071745834	0,942804179543356	NA
1.359783422	0,173898469971174	NA
-0.047755378	0,961911199293955	NA
1.129314147	0,258765335270318	NA
0.096020704	0,923504128955868	NA
-0.317975827	0,750503272953943	NA
-0.189804597	0,84946225328351	NA
-1.763873651	0,077753253999371	NA
0.629572596	0,528974257927951	NA
-2.280132104	0,0225998546725455	NA
2.019179666	0,0434685490064727	NA
1.306426274	0,191407635744159	NA
-1.222419673	0,221548963493199	NA
0.286097682	0,774803306719158	NA
0.173812878	0,8620125230365	NA
1.378555294	0,168031908507791	NA
1.416778018	0,156547838437876	NA
-0.524873297	0,599671273307802	NA
0.542351049	0,58757669435469	NA
0.45917426	0,646109032830349	NA
0.210271705	0,833455617482865	NA
0.995292829	0,319593864442047	NA
0.128682354	0,897608999047074	NA
0.671002169	0,502219147833668	NA
0.409421084	0,682230669553698	NA
-0.781846849	0,434304584603586	NA
0.375982742	0,706929726107272	NA
-0.053906436	0,957009709025247	NA
0.085776325	0,931644226772852	NA
-0.072370891	0,94230674976108	NA
-1.128411364	0,25914623077886	NA
-1.513324769	0,130197184015018	NA
0.100662099	0,919818699294588	NA
0.001415564	0,998870543456503	NA
-2.205308798	0,0274324328606852	NA
-0.614480551	0,538897821527001	NA
-0.054664067	0,956406097049705	NA
-0.27783123	0,781141916527808	NA
-1.222990441	0,221333306722919	NA
-0.188504317	0,850481331870271	NA
-0.068092576	0,945711940394572	NA
1.101121555	0,270843756706615	NA
-0.175859599	0,860404246098274	NA
-1.142532061	0,25323292733224	NA
1.902466866	0,057110146614907	NA
-0.924378542	0,355289262688604	NA
0.994342894	0,320055959883273	NA
-0.531650132	0,594968336787081	NA
-0.419462961	0,674877818687593	NA
-0.175916845	0,86035927126609	NA

-0.866085131	0,386443478405087	NA	
0.215940884	0,829033828089364	NA	
-0.308981187	0,757335837945064	NA	
-0.884336592	0,376514540973106	NA	
-1.878950437	0,0602512610994829	NA	
-0.206913179	0,836077664038591	NA	
-1.643561783	0,100266753621129	NA	
0.884004609	0,376693724901933	NA	
0.308708851	0,75754301060777	NA	
-1.482127702	0,138306319073508	NA	
-0.234575227	0,814538439008601	NA	
-0.453906441	0,649896167102392	NA	
0.540279208	0,589004495178827	NA	
0.224896404	0,822059866887992	NA	
-0.922355679	0,356343079893691	NA	
-0.156354813	0,875753352696682	NA	
-0.109959177	0,912441750862959	NA	
0.983098012	0,325559202666062	NA	
0.214300091	0,830313047370732	NA	
-1.393655091	0,163421783757675	NA	
-0.586501704	0,557538416431969	NA	
0.905734359	0,365076471897615	NA	
0.723018313	0,469668632992925	NA	
0.724627993	0,468680281167321	NA	
-0.623652333	0,532855933571487	NA	
-0.894497902	0,371055551715487	NA	
0.574349194	0,565731525887105	NA	
0.178747919	0,858135640816208	NA	
1.88425343	0,0595307033772481	NA	
0.280406664	0,779165524560834	NA	
0.715480857	0,474311965254709	NA	
-1.093248402	0,274284750832319	NA	
0.601652459	0,547405502025899	NA	
-0.804255832	0,421249240007001	NA	
0.690693422	0,489758223898836	NA	
0.462549304	0,643687454056648	NA	
-0.730326388	0,465190702368919	NA	
-1.818181059	0,0690364639701766	NA	
-1.217064036	0,22357985268491	NA	
-0.541616235	0,588082905575068	NA	
0.811275285	0,417207599832431	NA	
-1.482766221	0,138136533489252	NA	
1.142487355	0,253251499161107	NA	
-0.008029016	0,993593840960609	NA	
-0.427379601	0,669102862978025	NA	
-1.391592788	0,16404574388537	NA	
0.023688992	0,981100686764247	NA	
0.249059133	0,803315040558673	NA	
0.585873647	0,557960427535564	NA	
0.582457865	0,560258314504528	NA	

2.13051417	0,0331291871858733	NA
-0.25881799	0,795775676888541	NA
1.020832185	0,307333953029373	NA
-0.123048865	0,902068402937497	NA
0.771538441	0,440387846206464	NA
0.880694045	0,378483440857166	NA
0.785850666	0,431954979755905	NA
0.748639216	0,454074689133424	NA
0.595356528	0,551605177741807	NA
2.572274354	0,0101032792444917	NA
1.658257022	0,0972655998589571	NA
1.250422448	0,211145268759641	NA
-0.708321401	0,478745689811473	NA
0.247540279	0,804490122875385	NA
0.519621981	0,603327074749689	NA
-0.725862704	0,467922942506799	NA
-2.424948624	0,0153105532967356	NA
1.015467462	0,309883049336213	NA
-0.228522665	0,819239937852816	NA
0.601996944	0,547176171814335	NA
0.093198112	0,925746174585088	NA
0.286329349	0,774625881195913	NA
-2.91591235	0,00354650117696695	NA
-1.435656956	0,151099979042944	NA
-1.027977631	0,303960350056232	NA
1.173844595	0,240457277924651	NA
1.431461726	0,152297928781551	NA
-0.125183808	0,900378036000265	NA
0.546012638	0,585057249893041	NA
0.18867734	0,850345712344196	NA
-0.786134543	0,43178866924057	NA
-0.733395984	0,463316948044468	NA
2.01339476	0,0440731163834349	NA
-1.172646414	0,240937628318524	NA
0.75327124	0,451286937277113	NA
-0.854795235	0,392664516843524	NA
1.098881821	0,271819616815575	NA
-1.290674389	0,196816609185097	NA
0.920671591	0,35722191267837	NA
0.96196272	0,336068331037597	NA
-1.166036004	0,24359988896188	NA
-0.537587444	0,590861900449641	NA
0.061405697	0,951036115411293	NA
0.790634402	0,42915736540631	NA
1.815746081	0,0694093318247831	NA
0.762653787	0,445669897896647	NA
-0.290830356	0,771181067959545	NA
0.962334768	0,335881469538841	NA
-1.89020927	0,058729977420274	NA
0.802663121	0,422169474308755	NA

0.678947617	0,49717105318634	NA
0.272320797	0,785375366963909	NA
0.189603479	0,849619860711946	NA
0.885341987	0,375972210842322	NA
2.026024031	0,0427623221260421	NA
0.627414595	0,530387503112163	NA
-0.518847885	0,603866822754026	NA
-0.387304504	0,69853079135694	NA
0.697269711	0,485634019481047	NA
0.277148792	0,781665858048722	NA
-1.105944424	0,268750575270351	NA
-0.598126932	0,549755241490886	NA
0.943062301	0,345649037245781	NA
0.735281865	0,462167850257592	NA
0.521329442	0,60213729437108	NA
0.001305983	0,99895797622805	NA
0.892751386	0,371990325376984	NA
-1.689289492	0,0911639660267895	NA
1.008644182	0,313145310228134	NA
-0.355823098	0,721973049823659	NA
-0.570043287	0,568648339404009	NA
-0.455039926	0,649080515021664	NA
-0.209938432	0,833715726086679	NA
-0.844385475	0,39845403621407	NA
0.170378762	0,86471227347112	NA
1.852510838	0,0639525027588022	NA
0.703357128	0,481833216901405	NA
0.536051416	0,591923016400699	NA
0.548647245	0,583247556677075	NA
-1.12680762	0,25982382815006	NA
-0.475514658	0,634420188335441	NA
0.193284991	0,84673578034	NA
0.106388281	0,91527429041476	NA
1.020050288	0,307704611725493	NA
3.528825534	0,000417408215417777	NA
0.997863554	0,318345527136342	NA
1.662280204	0,096456605167819	NA
0.06794986	0,94582554779848	NA
-2.983974642	0,00284530351016092	NA
0.101906783	0,918830666180858	NA
-0.788530883	0,430386241669288	NA
0.933757448	0,350429023922481	NA
0.166747368	0,867568833001947	NA
2.37499306	0,0175492801524524	NA
0.021441375	0,982893568522127	NA
0.789532912	0,42980060148951	NA
0.801836213	0,42264770815065	NA
-0.6988781	0,48462821455505	NA
0.626441295	0,531025530943028	NA
0.674218416	0,500172463113541	NA

0.374382642	0,708119655410663	NA	
2.039858702	0,0413644011091241	NA	
0.304938978	0,760412628718008	NA	
-3.323299395	0,000889593804583164	NA	
0.35449611	0,72296711900097	NA	
1.76900377	0,076893245418125	NA	
-1.351531234	0,17652532155571	NA	
-0.19393807	0,846224374086562	NA	
0.919141853	0,358021380105687	NA	
1.466666438	0,142466816929229	NA	
1.583961583	0,11320246178377	NA	
0.119442322	0,904924933432208	NA	
-0.967145464	0,333471320485349	NA	
0.244309375	0,806991223298403	NA	
-1.604207121	0,10866840565146	NA	
-0.343051163	0,731559970033065	NA	
-0.560529341	0,575118432076235	NA	
0.528405221	0,597218114426539	NA	
1.52763644	0,126602829127964	NA	
2.015333126	0,0438697562394774	NA	
0.996017396	0,319241692711765	NA	
-0.098882232	0,92123177671473	NA	
0.690873774	0,48964486864836	NA	
-0.76084355	0,446750518949679	NA	
0.875147647	0,381493574832022	NA	
-0.421467554	0,673413699052613	NA	
-2.155687806	0,031108050942393	NA	
-1.645735994	0,0998181249776535	NA	
0.348385543	0,727550658261741	NA	
-1.589831885	0,111872704670217	NA	
0.443698448	0,65726061801927	NA	
-1.44086659	0,149622376475	NA	
-0.297181597	0,766327870978536	NA	
0.82096827	0,411664341953907	NA	
1.247576144	0,212186319357309	NA	
0.131483711	0,895392671199253	NA	
1.120731653	0,262402104406292	NA	
-0.260251087	0,794670099558917	NA	
-0.572256697	0,567148081637787	NA	
-1.111580831	0,266318418250028	NA	
-0.88180692	0,377881229094404	NA	
0.187988656	0,850885543463327	NA	
1.106736962	0,268407669734763	NA	
0.7933326	0,427584068355718	NA	
-1.708909337	0,087467744761941	NA	
-0.017739601	0,98584658835913	NA	
2.017200083	0,0436746374269225	NA	
-1.449222166	0,147275549206719	NA	
-0.071233282	0,943212093869693	NA	
-0.426651095	0,669633475855398	NA	

1.311710336	0,189617887901179	NA
-0.083273177	0,933634328238988	NA
-0.054738041	0,956347162646105	NA
-0.061406739	0,951035285280064	NA
1.971104944	0,048711873360351	NA
-0.443671015	0,657280454429994	NA
-0.909791316	0,362932575281073	NA
0.038023381	0,969669040340815	NA
0.090664746	0,927758983508176	NA
-0.072265281	0,94239079429289	NA
1.217624408	0,223366734741491	NA
1.198990202	0,230531755445865	NA
0.610148889	0,541763183896141	NA
0.688150875	0,49135777595892	NA
-2.337749336	0,0194002552310789	NA
0.821817827	0,411180585996774	NA
0.42038398	0,674204969135313	NA
-0.471932996	0,636974615238071	NA
0.177635825	0,859008987744613	NA
0.292954444	0,769556973372253	NA
-1.100066399	0,271303192510939	NA
0.593670367	0,552732606225593	NA
-1.617203525	0,10583435571527	NA
-0.440246929	0,659758273578104	NA
-0.473090324	0,636148739507842	NA
-0.191361403	0,84824245687914	NA
1.779877034	0,0750960870044909	NA
1.566214951	0,117298303755167	NA
1.374810872	0,169190086712276	NA
0.937428917	0,348537972285561	NA
-0.434945251	0,663602167656896	NA
0.714443187	0,474953175208619	NA
0.888630033	0,374201945062923	NA
-1.49190622	0,135723731855549	NA
-0.69232258	0,488734773169848	NA
-0.537382266	0,591003590845252	NA
-0.169127178	0,865696608196168	NA
0.323490147	0,746324067964707	NA
0.127176994	0,89880031400206	NA
2.162329251	0,0305928065774176	NA
1.180281464	0,237888287873801	NA
-0.269556971	0,787501107436679	NA
0.707071171	0,479522250108494	NA
0.873864545	0,382192025032853	NA
0.372734639	0,709345953350506	NA
0.722900962	0,469740732384293	NA
0.093101751	0,925822726109883	NA
0.908562156	0,363581290211594	NA
-0.175349119	0,860805317909729	NA
-0.39196452	0,695084428998575	NA

-1.667065394	0,0955014024038988	NA
1.543030986	0,122823257961862	NA
0.382418322	0,702151102626126	NA
-0.678183481	0,497655365268193	NA
-0.122752601	0,902303009172602	NA
0.169096309	0,865720888092732	NA
-0.563574931	0,573043451640948	NA
-1.700139652	0,0891046603137954	NA
0.08192667	0,934705026035384	NA
2.083456668	0,0372096175697189	NA
-0.683701735	0,494163527873603	NA
0.940596285	0,346911786443049	NA
-0.353365223	0,72381465427163	NA
-0.883345302	0,377049734228861	NA
-0.628493717	0,529680561209183	NA
0.721654493	0,470506926745819	NA
0.701466032	0,483012226004874	NA
-0.131224982	0,895597334128425	NA
0.060933203	0,95141240652895	NA
2.178572782	0,029363419938273	NA
2.840336241	0,00450660046860637	NA
2.330508905	0,0197792699375273	NA
-0.030896894	0,975351767120422	NA
-0.166268417	0,867945719701982	NA
0.130432516	0,896224241955295	NA
-0.251960581	0,801071532709135	NA
-0.065912957	0,94744712495046	NA
0.48485029	0,62778254661703	NA
-1.688429418	0,0913288272949477	NA
0.129756371	0,896759182016883	NA
-0.475283293	0,634585065750612	NA
0.478353478	0,632398639622443	NA
0.860882029	0,38930302024925	NA
1.359106907	0,174112714397457	NA
1.735588032	0,0826367036086722	NA
-0.225968518	0,821225905872449	NA
-0.106692378	0,915033029923453	NA
-1.301854756	0,192966041355848	NA
-0.007450477	0,994055434642599	NA
1.195409945	0,231926901826447	NA
1.440977728	0,149590975096227	NA
1.236917466	0,216117724892288	NA
0.186213559	0,852277291094055	NA
0.288494458	0,772968272235007	NA
-0.838849718	0,401553646970891	NA
0.32832529	0,742665724662325	NA
2.129129784	0,0332435242331803	NA
0.239731817	0,810538167802401	NA
0.633439524	0,526446661288548	NA
-0.271385658	0,786094431867837	NA

-0.604420437	0,545564151529283	NA
-0.413517356	0,679227609006975	NA
-0.589160575	0,555753561256614	NA
0.463329773	0,64312800833332	NA
0.636727016	0,524302672843826	NA
0.495161019	0,620486464066256	NA
1.05300749	0,292337559077043	NA
2.572496855	0,0100967873421461	NA
0.638328893	0,523259607584742	NA
1.660435318	0,0968269100704194	NA
1.123893213	0,261058330780211	NA
0.38491167	0,70030285942025	NA
0.032572116	0,974015806237787	NA
0.693339887	0,488096277415707	NA
0.156079071	0,87597069542157	NA
-0.129662622	0,896833356015754	NA
-0.24502525	0,806436880546075	NA
-0.50219808	0,615528193467786	NA
1.204554103	0,228375483995841	NA
-0.1092804	0,912980091751102	NA
-0.206595195	0,8363260129476	NA
0.388461182	0,697674775547531	NA
-0.79929881	0,424117168743096	NA
0.406626603	0,684282255160199	NA
1.910936024	0,0560128029202687	NA
0.500186399	0,616943834327249	NA
-1.051237949	0,293149317788847	NA
1.762230018	0,0780304423084058	NA
-0.239243742	0,810916585793065	NA
1.317945419	0,187521919850736	NA
-0.283257423	0,776979513094481	NA
-0.472754358	0,636388440117294	NA
1.175877878	0,239643680246928	NA
-1.011586396	0,311735853040258	NA
0.728328359	0,466412606174508	NA
0.369002463	0,71212588899033	NA
0.166352718	0,867879380705533	NA
-1.175828292	0,239663498469419	NA
-0.449618492	0,652985551916155	NA
0.227372353	0,820134216790226	NA
0.352636077	0,724361287451689	NA
1.283022111	0,19948432986225	NA
0.391613321	0,695343944159677	NA
0.521842278	0,601780150162427	NA
0.369486844	0,711764877769884	NA
-1.617094442	0,105857896384726	NA
0.360304376	0,718619527037611	NA
-0.971018758	0,331538937569189	NA
-0.409916361	0,681867303268944	NA
-0.959849571	0,337130929381715	NA

-1.743942158	0,0811691764574482	NA
-0.18226401	0,855375541319393	NA
1.301037106	0,193245753942813	NA
0.904624334	0,365664439382512	NA
1.115982039	0,264429822811097	NA
-0.771872591	0,440189892159494	NA
-1.560439027	0,118656168276906	NA
1.328721953	0,183939719342066	NA
-1.492762498	0,13549936703744	NA
-0.395978061	0,692121213262816	NA
1.047892412	0,294688178785487	NA
-0.229896831	0,818171939252292	NA
-0.427161767	0,669261506659227	NA
0.506977977	0,612170271258103	NA
-1.237803162	0,215789055848666	NA
0.671596656	0,501840507694494	NA
0.432496403	0,665380665697559	NA
-0.399374398	0,6896173558276	NA
-1.441345881	0,14948699148723	NA
0.042371318	0,96620269310077	NA
1.138071533	0,25509061366309	NA
-0.364697733	0,715337051067994	NA
1.469383618	0,141728768262512	NA
1.432357902	0,152041420109191	NA
-0.087562108	0,930224720154574	NA
0.391730334	0,695257474098542	NA
0.558964989	0,576185614703653	NA
0.080662158	0,935710632312854	NA
-0.063397823	0,949449720389969	NA
-0.406991446	0,68401427071239	NA
1.467087812	0,142352169248565	NA
0.480466132	0,630895979601487	NA
-1.274087582	0,202632378990206	NA
-0.178564807	0,858279430490193	NA
0.481213246	0,63036494739954	NA
-2.117987442	0,03417613127246	NA
0.716139665	0,473905113558569	NA
0.725676826	0,468036911717068	NA
1.196545587	0,231483719747791	NA
0.690027913	0,490176633778055	NA
0.063952702	0,94900788831818	NA
-0.65948369	0,509585216125933	NA
-1.298214956	0,194213480093236	NA
0.234781075	0,814378657129872	NA
-0.289239864	0,772397827568732	NA
0.752390684	0,451816147037755	NA
-1.030821925	0,302624334789976	NA
-0.239172073	0,810972157186376	NA
0.584761902	0,55870782489568	NA
-0.401869225	0,68778027279454	NA

0.393685748	0,693813064737739	NA
-0.195101745	0,845313296496901	NA
0.801040703	0,423108082582104	NA
1.488623466	0,136586548692425	NA
0.419480174	0,674865241533775	NA
2.073028437	0,0381696331425066	NA
0.497073094	0,619137507194971	NA
0.002600989	0,997924713076922	NA
0.172545882	0,86300839370388	NA
-0.116240586	0,90746187138361	NA
0.47379939	0,635642969701325	NA
-1.234733853	0,216929572550125	NA
-1.415343938	0,156967678376809	NA
2.155048707	0,0311580229183172	NA
0.227958722	0,819678330747374	NA
0.981528775	0,326332051387574	NA
-2.485753506	0,0129277474752097	NA
-0.878664042	0,379583460851588	NA
-0.092805535	0,926058053541169	NA
-0.698937454	0,484591119532788	NA
-0.524630376	0,599840166359809	NA
-0.436076687	0,662781090687392	NA
-0.014621478	0,988334163961019	NA
-0.146135489	0,883814431306911	NA
0.197634289	0,843331204967251	NA
-1.01171637	0,311673685958259	NA
-1.414545461	0,157201809303313	NA
-0.316031544	0,751978565687319	NA
0.636244389	0,524617145361284	NA
0.785678298	0,432055980570103	NA
1.259288228	0,207926243665738	NA
0.10771604	0,914220945251766	NA
-1.442459405	0,149172815791784	NA
-0.165022455	0,868926307641496	NA
0.103206886	0,917798775086822	NA
0.088104713	0,929793450841417	NA
1.537785807	0,124100995008719	NA
-0.029540305	0,976433674485894	NA
0.174501782	0,861471130418028	NA
0.329452708	0,74181353401762	NA
-0.78553363	0,432140760915233	NA
-0.987408103	0,323442613827551	NA
1.12602624	0,260154412595584	NA
-0.277576517	0,781337460573974	NA
0.850789746	0,394886158475272	NA
1.42295339	0,154749667496981	NA
0.632673199	0,52694707494294	NA
0.378230131	0,705259644169749	NA
-0.02982921	0,976203262899669	NA
-1.16808662	0,242771826864081	NA

-1.430774708	0,152494793989998	NA
0.023950979	0,980891710842263	NA
0.155248867	0,876625124704552	NA
0.467621753	0,640055106359209	NA
-0.29788948	0,765787517555377	NA
-1.180527088	0,237790643358131	NA
0.145346277	0,884437478763616	NA
0.498496974	0,618133801731913	NA
1.693722968	0,0903179379550141	NA
0.668319926	0,503929396885215	NA
-0.459963755	0,645542236408415	NA
1.047729821	0,294763103843279	NA
0.38817703	0,697885030077251	NA
0.61334068	0,539651100505977	NA
-1.379569687	0,167719176164733	NA
-1.029408899	0,303287569197069	NA
-0.163696078	0,869970405102411	NA
1.208056226	0,22702563585882	NA
-1.21643205	0,223820381248543	NA
0.944729406	0,344797040282484	NA
1.364645252	0,172364574831309	NA
-1.219246202	0,222750760979609	NA
-0.397526088	0,690979552670971	NA
-0.201999074	0,839917448307864	NA
0.652937642	0,513796480252146	NA
-0.004236518	0,996619757504287	NA
0.701431599	0,483033707625563	NA
1.607034714	0,108046753369256	NA
1.109355077	0,267277033659204	NA
-0.946488944	0,343899258791913	NA
0.924978181	0,354977256869908	NA
0.82333076	0,410319925795754	NA
0.859744793	0,389929737212579	NA
0.671159682	0,502118810129189	NA
1.454543727	0,145795619640869	NA
0.972658906	0,330722855551917	NA
1.620132128	0,105203897161287	NA
-0.57531175	0,565080476328246	NA
-0.721887737	0,470363501512651	NA
-1.060989026	0,288694888513159	NA
-1.051392057	0,293078562472677	NA
-0.302172868	0,762520286127087	NA
-0.316590701	0,751554192850292	NA
2.145320393	0,031927239108958	NA
-0.507145476	0,612052748486374	NA
-0.20489579	0,837653544839424	NA
-0.081812081	0,93479614905913	NA
-0.645059147	0,518888856583035	NA
0.109383091	0,91289864467626	NA
-0.529690226	0,596326725686354	NA

1.618103625	0,105640267537087	NA
-0.890691334	0,373094785230905	NA
1.603995321	0,10871508395374	NA
-0.607810525	0,543313147854269	NA
-0.379841303	0,704063220968534	NA
1.387098496	0,165411730583532	NA
0.434373069	0,664017551349822	NA
1.26114139	0,207257910607285	NA
0.621578833	0,534218843197865	NA
1.291126367	0,196659862618675	NA
-0.945745	0,344278664244341	NA
0.289091032	0,772511716381058	NA
1.452848394	0,146265851905745	NA
-0.4483775	0,653880777223398	NA
2.531150901	0,0113688925818496	NA
-0.171008313	0,864217229420096	NA
0.46176961	0,644246546592684	NA
-0.032111772	0,974382915182553	NA
0.402401979	0,687388213944097	NA
0.946397913	0,343945669626622	NA
0.010969246	0,991247983515608	NA
1.129097326	0,258856779298425	NA
0.706977999	0,479580150141181	NA
-2.198520061	0,0279120665954102	NA
-0.079510641	0,936626467913178	NA
1.876761975	0,0605507242318321	NA
0.161890325	0,871392221621945	NA
-0.997923916	0,318316253961506	NA
0.079977696	0,936254995165949	NA
1.751035867	0,0799397320325049	NA
-0.974145647	0,329984227625433	NA
-1.282931432	0,199516099528027	NA
0.070558623	0,943749042111565	NA
-0.171747053	0,863636392165195	NA
0.026116766	0,979164204364053	NA
-0.362155776	0,717235624873215	NA
1.49401372	0,135172033403995	NA
0.012425449	0,990086181259755	NA
-3.364579559	0,000766603791795488	NA
1.400443682	0,16138049691828	NA
-0.575079204	0,565237731793003	NA
-1.697470904	0,0896076675205588	NA
0.062172239	0,950425670101498	NA
0.24415038	0,807114355455136	NA
0.182857315	0,854909975764373	NA
1.006420634	0,314213272156812	NA
2.557701104	0,010536661411948	NA
1.359375274	0,174027702078611	NA
-1.793885175	0,0728314981210375	NA
1.530231316	0,125959482336106	NA

-0.666326348	0,505202527972429	NA
0.752187234	0,45193846936555	NA
1.19334942	0,232732555308428	NA
0.771727151	0,440276046098924	NA
-2.505536816	0,0122265668739679	NA
-0.186085795	0,852377480982477	NA
1.028215062	0,303848674684068	NA
0.029877131	0,976165044832039	NA
-1.002778821	0,315967589463697	NA
-0.083748399	0,933256475652094	NA
-0.196833055	0,843958182867356	NA
1.30062776	0,193385900147792	NA
0.300886957	0,76350069646592	NA
0.664922281	0,506100203784689	NA
-2.228411604	0,0258530800002831	NA
0.563617752	0,573014302826563	NA
0.570206349	0,568537750255672	NA
0.272389038	0,785322900322133	NA
0.760533547	0,446935724590658	NA
-1.601074061	0,109360516627195	NA
-0.16199993	0,871305909035162	NA
0.263159525	0,792427625594202	NA
-1.17774938	0,238896534319622	NA
0.54298199	0,587142202485288	NA
0.410141683	0,68170201726756	NA
1.256610685	0,208894643707079	NA
-0.638380441	0,523226060007283	NA
-0.513207177	0,607806405936906	NA
0.432096443	0,665671320076578	NA
1.510516105	0,130911783200679	NA
-1.066019011	0,286415047988507	NA
0.567825478	0,570153477701553	NA
0.156655101	0,875516673956316	NA
0.356699765	0,721316581173437	NA
0.797348221	0,425248821878934	NA
-1.252368636	0,210435569313637	NA
1.368514797	0,171150993678624	NA
0.647067634	0,5175881718148	NA
0.450754278	0,652166657517491	NA
-0.443678852	0,657274787084409	NA
-0.590021939	0,555175941093629	NA
-0.592872044	0,553266788278416	NA
-0.882213258	0,377661493524997	NA
0.039701103	0,968331422374325	NA
0.813100977	0,416160166241336	NA
-1.608393883	0,10774894060689	NA
-0.908146448	0,363800853147158	NA
-1.726293493	0,084294625371137	NA
0.960872508	0,336616275293637	NA
0.732338478	0,463961999212078	NA

0.603265369	0,5463321674801	NA
-1.30854029	0,190690118284709	NA
-2.407917164	0,0160438208667495	NA
-1.96100666	0,0498782459969972	NA
0.647011719	0,517624359088444	NA
-0.224037152	0,822728394902595	NA
2.202381885	0,0276383443606042	NA
-0.221967091	0,824339501553063	NA
1.044199718	0,296392997429267	NA
-0.541601666	0,588092943846452	NA
1.286012392	0,198438737065171	NA
1.186473751	0,235435261517216	NA
1.651659375	0,0986040086418926	NA
0.075631435	0,939712326767056	NA
-0.03910662	0,968805382875065	NA
0.080781024	0,935616099547872	NA
-0.539835726	0,589310326789212	NA
-0.453959245	0,649858160626229	NA
1.411114649	0,158210811134515	NA
1.200569627	0,229918188869812	NA
-0.852909042	0,393709747028493	NA
1.012098221	0,311491093073207	NA
1.228109511	0,219405861257695	NA
1.247812215	0,212099834416315	NA
1.103840796	0,26966220451686	NA
0.248267819	0,803927196515652	NA
0.551766841	0,581108108167328	NA
1.336698683	0,181321012003614	NA
1.12481802	0,260666156781015	NA
0.47012402	0,638266413841912	NA
0.144999815	0,884711017670482	NA
0.338807746	0,734754565331103	NA
0.323872249	0,746034754834897	NA
-0.412172041	0,680213328754927	NA
-0.124169022	0,901181449178883	NA
0.819696152	0,412389344339727	NA
-1.980495922	0,0476478311262345	NA
0.491687019	0,622940616199423	NA
-0.858749404	0,390478787162827	NA
-0.351125351	0,725494309780847	NA
-0.053472517	0,957355427190987	NA
-0.212495145	0,831720764827728	NA
0.419776309	0,6746488735992	NA
0.501309796	0,616153111912066	NA
-0.51700987	0,605149268984356	NA
0.574548695	0,565596558568501	NA
0.231202049	0,817157838069874	NA
-0.455404895	0,648817974123108	NA
0.737433095	0,460859015937863	NA
0.379553282	0,704277045441176	NA

-1.25152374	0,21074345772267	NA
0.447339871	0,654629683048526	NA
0.523735988	0,600462181444563	NA
0.905143035	0,365389616042059	NA
-0.14486704	0,884815849925402	NA
-1.334886018	0,181913654562291	NA
-1.296611107	0,194765028997661	NA
-0.998668558	0,317955276796738	NA
-0.236462888	0,813073501530751	NA
0.706854867	0,479656673708276	NA
0.115681779	0,907904747829768	NA
1.073311357	0,283131441227378	NA
0.772238479	0,439973195105589	NA
0.100230713	0,920161163504543	NA
0.267208643	0,789308521911246	NA
-0.314290318	0,753300550792232	NA
0.717818641	0,472869117717867	NA
-1.435070997	0,151266867209605	NA
-0.938806427	0,347830138414629	NA
-0.837627287	0,402240062782197	NA
0.794237033	0,427057453184224	NA
-0.808240931	0,418951899284685	NA
-0.160979662	0,872109419135966	NA
0.22198306	0,824327070813901	NA
2.042832993	0,0410689733087114	NA
-0.136412194	0,891495434591856	NA
-0.367552682	0,713206802803279	NA
0.588147211	0,556433486309676	NA
0.029198253	0,976706474464982	NA
-0.176505569	0,85989677356968	NA
0.749299509	0,453676703586312	NA
-0.499402907	0,617495571831326	NA
0.80890007	0,418572628729983	NA
2.102649742	0,0354963983483135	NA
-0.039550285	0,968451663525515	NA
1.190516582	0,23384341665912	NA
0.23534162	0,813943593788949	NA
0.374035313	0,708378043726626	NA
0.335358665	0,737354544889193	NA
-0.287155596	0,773993184371628	NA
0.913675484	0,360887389911594	NA
1.053156167	0,292269424177619	NA
1.19379369	0,232558680648399	NA
-0.256117631	0,79786000852219	NA
1.338566596	0,180711806792955	NA
-1.346330604	0,178195920138191	NA
0.360764467	0,718275527518894	NA
0.782626684	0,43384636681165	NA
2.191899398	0,0283867715368539	NA
1.593578049	0,111030574181078	NA

-1.083632785	0,278527648649349	NA
-0.483026595	0,629076852698592	NA
-1.376955704	0,168525943415453	NA
0.889001992	0,374002009308399	NA
0.783872225	0,433115089117603	NA
-0.324326124	0,745691143365619	NA
0.547261003	0,584199431400973	NA
-1.235569331	0,2166186904822	NA
-0.750020241	0,453242514163915	NA
0.414432116	0,678557672419736	NA
-0.828299946	0,407500653305784	NA
0.758079284	0,448403520674236	NA
-0.235225109	0,814034018808265	NA
3.01878137	0,00253793608901096	NA
0.776973008	0,437174695697405	NA
0.911863544	0,361840554856475	NA
-0.336988361	0,736125674396986	NA
0.6462341	0,518127758856435	NA
0.085532619	0,931837964612913	NA
-0.287281047	0,773897133703042	NA
0.408134172	0,683175173128098	NA
1.579487388	0,114224307131105	NA
1.151839067	0,249387211062665	NA
0.242617252	0,808301915446312	NA
0.64371465	0,519760488102624	NA
-0.561323032	0,574577343360047	NA
-0.547449271	0,5840701128836	NA
-1.335295891	0,181779523088537	NA
-0.815779712	0,414626138755105	NA
-0.205378612	0,837276329643022	NA
0.423505945	0,671926162276029	NA
-0.237125456	0,812559464116868	NA
0.495000833	0,620599532259686	NA
0.149222233	0,88137827676392	NA
-0.685010386	0,493337367107628	NA
-0.007718397	0,993841671684726	NA
2.009212155	0,0445146392378587	NA
0.444034375	0,657017730966451	NA
1.906726639	0,0565559955666067	NA
-1.121502126	0,262074187022902	NA
0.27433742	0,783825330517141	NA
0.429296181	0,667707695947051	NA
0.398546441	0,690227432488814	NA
1.045084575	0,295983882500831	NA
-0.537086932	0,59120756740289	NA
0.431640251	0,666002899117935	NA
0.755128663	0,450171786552205	NA
0.243870355	0,807331229040004	NA
-1.247444522	0,212234550343079	NA
0.191661753	0,848007166906065	NA

0.508377557	0,611188589219226	NA
0.055360942	0,955850912192647	NA
0.422832119	0,672417751475342	NA
0.690854631	0,489656899860629	NA
2.13499293	0,0327615876078709	NA
-0.178416533	0,858395866297531	NA
1.233402289	0,21742571156571	NA
-0.42836462	0,668385679043294	NA
0.352583593	0,724400639762343	NA
1.156630845	0,24742321460977	NA
0.722125385	0,470217392234614	NA
-0.333681235	0,73862011145599	NA
0.752875122	0,451524958856781	NA
-0.735777061	0,461866383720704	NA
-1.75272704	0,0796488720210872	NA
0.331488928	0,740275202982617	NA
0.948423733	0,342913782125361	NA
1.408259626	0,159054206221053	NA
-0.736997576	0,461123823601125	NA
-1.694522923	0,09016595955464	NA
-0.821189121	0,411538552084422	NA
3.05477666	0,0022522817607705	NA
1.360510226	0,173668519064905	NA
0.832274192	0,405254194747755	NA
0.63948634	0,522506596987575	NA
1.026866472	0,304483343582088	NA
0.40620187	0,68459428004633	NA
-0.569855024	0,56877603183535	NA
1.056659918	0,290666824080384	NA
0.663283065	0,507149280437654	NA
1.115898332	0,264465655794205	NA
-1.314962126	0,18852263136176	NA
-0.938682737	0,347893659474224	NA
1.397042683	0,162400736023998	NA
-1.177597148	0,23895724754053	NA
1.523680316	0,127588589033029	NA
2.290624888	0,0219851182388163	NA
1.121220112	0,262194180838112	NA
-0.141437249	0,88752452918606	NA
0.225850937	0,821317358758493	NA
0.649743811	0,515857719539718	NA
0.742972134	0,45749855181688	NA
1.379092984	0,167866086900062	NA
0.394934634	0,692891129377801	NA
0.982026951	0,32608657094727	NA
-0.04854247	0,961283917809043	NA
-1.374155787	0,16939332351092	NA
1.409428056	0,158708632993305	NA
-0.393880502	0,693669266567117	NA
0.066687129	0,946830781397007	NA

-0.313361329	0,754006161013107	NA
1.152034902	0,249306731319778	NA
-0.198971108	0,842285344874018	NA
-0.5495612	0,582620377698366	NA
-0.444164251	0,656923836174973	NA
2.569818982	0,0101751667755063	NA
0.664543778	0,50634233927159	NA
-0.006111455	0,995123794941027	NA
-0.266548874	0,789816524388403	NA
1.842629036	0,0653831915255076	NA
0.848569498	0,396120885658301	NA
0.599763426	0,548663911417811	NA
-0.425483027	0,670484592171996	NA
0.690753035	0,48972075462617	NA
0.271157758	0,786269700357798	NA
0.295378259	0,767704939708995	NA
0.405799695	0,684889783099741	NA
1.368769533	0,171071327404635	NA
1.270775962	0,203808362837359	NA
-0.267544875	0,789049667858344	NA
0.258710727	0,795858442027345	NA
-0.547799393	0,583829655560354	NA
-0.721178238	0,470799859238972	NA
0.650543796	0,515341021284577	NA
0.653455652	0,513462570725721	NA
1.176554438	0,239373392097965	NA
0.966618884	0,333734590962778	NA
0.542723358	0,587320289073103	NA
0.028860419	0,976975913743006	NA
0.249607164	0,802891157972809	NA
0.483482003	0,62875353561007	NA
-0.346417167	0,729029225372743	NA
-0.144281949	0,885277832085715	NA
0.560365719	0,575230009152871	NA
0.939582141	0,347431940576816	NA
2.018200103	0,0435704254071112	NA
-1.23607529	0,216430578721004	NA
-0.473613343	0,635775658667736	NA
1.81771782	0,0691072726025059	NA
1.237348995	0,215957545608767	NA
0.933370286	0,350628816526175	NA
0.944487314	0,344920681557903	NA
1.139455569	0,254513190020257	NA
-0.211792124	0,832269212903869	NA
1.368072127	0,171289500343586	NA
-0.585750415	0,558043249704165	NA
0.459813344	0,645650204579235	NA
-0.301683078	0,762893669728308	NA
1.934291651	0,0530772791724885	NA
-0.079671035	0,936498896639415	NA

0.644718714	0,519109486755777	NA
-0.432579851	0,665320030197208	NA
-1.369580712	0,170817823824935	NA
-0.520473682	0,602733466198224	NA
-1.416165605	0,156727023721839	NA
-0.508244523	0,611281870285038	NA
-0.361369151	0,717823505713341	NA
0.498069592	0,618434992175462	NA
-1.353994987	0,175737977122413	NA
0.159345163	0,873396940200569	NA
1.723649334	0,0847711667660588	NA
-0.785718719	0,432032294113367	NA
-0.800530645	0,423403415530107	NA
-0.970387077	0,331853587572833	NA
-0.671098169	0,502157993257947	NA
0.95287557	0,340653119545848	NA
0.040053051	0,96805083186688	NA
-0.513515767	0,607590584485646	NA
-0.552443855	0,58064429127635	NA
-0.529902435	0,596179577869203	NA
0.46811421	0,639702918237981	NA
0.911703857	0,361924632889833	NA
-0.333391922	0,738838460210747	NA
-0.272985678	0,784864225230965	NA
-2.582466172	0,00980969786750463	NA
1.815234653	0,0694878564931576	NA
0.632077877	0,52733599023126	NA
0.252003224	0,801038571683141	NA
-0.203426824	0,838801435506305	NA
0.493037993	0,62198574258311	NA
-0.320558977	0,748544629504307	NA
-0.404781148	0,685638387305886	NA
-0.220862796	0,825199266186743	NA
0.359586587	0,719156315885863	NA
2.20512038	0,0274456483105557	NA
-1.011935321	0,311568979541935	NA
0.485761213	0,627136477197666	NA
1.921128198	0,0547155474128493	NA
-0.517126195	0,605068068983863	NA
1.777642597	0,0754625730481566	NA
-0.202546966	0,839489145439434	NA
0.03106204	0,975220062443468	NA
-0.810352211	0,417737775385153	NA
0.049282382	0,960694259832947	NA
1.478751782	0,139206667125556	NA
0.092266423	0,926486364922417	NA
1.838092231	0,0660488157822322	NA
1.440423985	0,14974748199718	NA
-0.907695152	0,364039306020751	NA
1.611972622	0,106967897575754	NA

-0.836608542	0,402812642363229	NA
0.457631773	0,647217012710179	NA
1.086702129	0,277168472906393	NA
1.468010204	0,14210145105612	NA
2.194206201	0,0282205890102903	NA
-1.255116611	0,209436431302876	NA
-1.856417266	0,0633941055690343	NA
0.274912262	0,783383646230725	NA
-0.945533181	0,344386739276362	NA
0.561651212	0,574353680737158	NA
0.293633507	0,769037968915131	NA
1.077940852	0,281060152900977	NA
-0.242892712	0,808088511898899	NA
0.212403406	0,831792328585203	NA
-1.017986932	0,308684166535312	NA
0.309694085	0,756793601418645	NA
0.768939337	0,441929323490105	NA
0.765154234	0,444179708456377	NA
-0.372911166	0,709214561066278	NA
-1.453813832	0,145997927610019	NA
-1.441551092	0,149429054006536	NA
-1.146969912	0,25139405652777	NA
-0.425597239	0,670401352541852	NA
0.313159986	0,75415911687799	NA
-0.22181583	0,824457255498667	NA
-0.318325185	0,750238282523607	NA
0.323816326	0,746077095129022	NA
-0.14622284	0,883745475586352	NA
-1.537018829	0,124288698537344	NA
0.111965723	0,91085058444929	NA
-0.160807006	0,872245406862966	NA
0.314524642	0,753122603764212	NA
0.410747476	0,681257710527926	NA
1.351944406	0,176393100616334	NA
-0.219636152	0,826154533899639	NA
1.981932114	0,047486839070871	NA
1.019065797	0,308171730776998	NA
-2.234495444	0,0254504920112474	NA
0.339296277	0,734386547322823	NA
2.157879333	0,0309372147148319	NA
1.544212475	0,122536867789736	NA
-0.265678332	0,790486952417985	NA
-0.679861031	0,49659245818452	NA
-0.322027088	0,747432173246197	NA
1.316691773	0,187941962277962	NA
-0.34094301	0,733146485416651	NA
0.567485873	0,57038412171326	NA
1.396604938	0,16253240457088	NA
-0.154288075	0,877382599567017	NA
0.537974579	0,59059459883406	NA

0.755719132	0,449817611069642	NA
0.041331759	0,967031415063753	NA
0.744331601	0,456675888074146	NA
-0.311424336	0,755478055321825	NA
1.565353039	0,11750015325201	NA
2.022431507	0,0431317928835064	NA
0.42631083	0,669881367721868	NA
0.064144644	0,948855054230761	NA
0.218595885	0,826964858390235	NA
0.255165457	0,798595309575809	NA
-1.196898139	0,231346259781613	NA
-0.385493371	0,699871917157276	NA
-2.071439039	0,038317785818092	NA
0.773595066	0,439170290625273	NA
-1.061345682	0,288532832201095	NA
-0.599690611	0,548712446598903	NA
0.445393394	0,656035485359256	NA
-1.128741278	0,259006990719119	NA
-1.861451368	0,0626804623184898	NA
-0.284470525	0,776049818708421	NA
0.279895587	0,779557613182974	NA
0.78097683	0,4348161222225	NA
0.829267776	0,406952900959032	NA
1.173231877	0,24070283213831	NA
-0.794077192	0,427150494400386	NA
-0.63791813	0,5235269755629	NA
0.692800874	0,488434523705094	NA
-0.148145429	0,882227997582048	NA
-0.586295646	0,557676856298803	NA
1.336193355	0,181486082365695	NA
-0.040143714	0,967978551028671	NA
-0.762326676	0,445865056534143	NA
-0.136074811	0,891762140176839	NA
0.380754637	0,703385322603712	NA
-0.32716515	0,743542977707985	NA
-0.085312827	0,932012693943572	NA
-0.984472254	0,324883367249181	NA
-1.183018916	0,236801650732813	NA
0.729213148	0,465871289701672	NA
1.413321848	0,157561112973816	NA
1.260633405	0,207440957339412	NA
0.0706344	0,94368873150078	NA
0.072744151	0,942009713942672	NA
-0.099992026	0,920350656154436	NA
-3.491670023	0,000480010854008823	NA
1.083000236	0,278808318114114	NA
-0.362820836	0,716738725917935	NA
-1.316322221	0,188065915697784	NA
-0.587606276	0,556796597613276	NA
-1.071442241	0,283970628339475	NA

0.016414897	0,986903395458571	NA
0.576666655	0,564164662368587	NA
-0.952467731	0,340859823468607	NA
0.186452095	0,852090242574321	NA
0.208714493	0,83467112573877	NA
0.535646729	0,592202726905575	NA
-0.44117666	0,659085108956043	NA
-1.048820637	0,294260677554354	NA
-0.08506779	0,932207497279378	NA
-0.028469843	0,97728741971924	NA
-0.277765332	0,78119250522195	NA
1.337693017	0,180996528165485	NA
0.872843327	0,38274847994924	NA
-0.199439286	0,841919132116126	NA
1.182737604	0,236913156019912	NA
1.965804395	0,0493212099467687	NA
-0.084710412	0,932491618242507	NA
0.792775262	0,427908771620205	NA
0.683867355	0,494058929496159	NA
-0.291185186	0,770909692060457	NA
2.588247027	0,00964657738753747	NA
2.15163193	0,0314263552053186	NA
1.450554739	0,146903886233557	NA
-0.931890536	0,351393099334917	NA
-0.468720558	0,639269390439137	NA
0.934338422	0,350129350895645	NA
1.102184973	0,270381263438314	NA
0.196156821	0,844487423232474	NA
-0.521604906	0,601945446863322	NA
-0.122274516	0,90268161406084	NA
1.432480647	0,152006312911231	NA
1.010894993	0,312066690620497	NA
0.306225877	0,759432671558638	NA
-0.103154251	0,917840548464283	NA
0.472978788	0,636228313057574	NA
-0.104373153	0,916873226951294	NA
0.693934473	0,4877233035326	NA
-0.804844522	0,420909406092055	NA
1.435519972	0,151138981130814	NA
-0.890260367	0,37332609680363	NA
3.212186094	0,00131729016662487	NA
-1.526195645	0,126961147817594	NA
-0.190732427	0,848735232290874	NA
0.183682812	0,85426229358023 7	NA
-0.621984628	0,533951976039744	NA
0.170114563	0,864920040448611	NA
1.147667817	0,251105721990084	NA
-0.293670561	0,769009651543193	NA
-0.717650721	0,472972675130947	NA
0.022649699	0,981929699615977	NA

1.131735237	0,257745762424464	NA
1.46521477	0,142862331124111	NA
2.621457594	0,00875546540741057	NA
-0.091797296	0,926859092832885	NA
-1.034843183	0,300742152167843	NA
1.605108219	0,108469990737812	NA
-0.284919195	0,775706049938135	NA
-1.196302887	0,23157838241622	NA
0.00169265	0,998649461256373	NA
0.17306512	0,862600241659614	NA
-0.32234379	0,747192262152582	NA
0.593470304	0,552866450953413	NA
-0.83738353	0,402377020365644	NA
0.672075828	0,501535424064053	NA
1.143101951	0,25299626452127	NA
-0.615379903	0,538303860204085	NA
-0.097522131	0,922311759977144	NA
-0.145286536	0,88448464481322	NA
0.374249715	0,708218539401783	NA
1.578533682	0,114443056651235	NA
0.452104593	0,651193635304548	NA
-0.612450449	0,540239772177428	NA
-0.814712438	0,415236932077887	NA
-1.419867374	0,155646295914699	NA
1.867972396	0,061765918674294	NA
-1.505816127	0,13211438224558	NA
-1.017966453	0,308693899131857	NA
-0.543397765	0,586855964129475	NA
-1.029225411	0,303373764200618	NA
-0.286143426	0,774768272256199	NA
-1.307909228	0,190904099083531	NA
1.160982752	0,245648913733807	NA
-0.837226352	0,402465347737746	NA
0.630624345	0,528286176676631	NA
-1.046919153	0,295136867538559	NA
-0.68662776	0,492317330587587	NA
-0.103225736	0,917783814736426	NA
-0.456538121	0,64800306437276	NA
-0.973560418	0,330274847583183	NA
0.2959223	0,76728942080389	NA
0.46314156	0,643262901723555	NA
0.254785825	0,798888523767524	NA
-1.92967588	0,0536470103896902	NA
1.759947798	0,078416657986632	NA
-0.315287907	0,75254306585974	NA
0.462010061	0,644074105178335	NA
1.6480594	0,0993404824719822	NA
1.481025655	0,138599737151804	NA
0.175051715	0,861038998379828	NA
-0.89847835	0,368930582407634	NA

-2.11791492	0,0341822738256845	NA
0.148685274	0,881801982028473	NA
1.498635256	0,133968281138945	NA
-4.451489429	8,52767314917714e-06	NA
0.387877527	0,69810666969616	NA
1.324652936	0,185286289869794	NA
1.282381453	0,199708867124449	NA
-0.249394373	0,803055737358792	NA
0.874606093	0,381788271220806	NA
0.71688169	0,473447100894471	NA
1.593662046	0,111011749361874	NA
0.33315982	0,739013645873413	NA
2.049884872	0,0403756668216048	NA
-0.9484381	0,342906471004492	NA
-0.759010594	0,447846219576264	NA
-0.598380218	0,549586262521422	NA
1.725014056	0,0845249394388174	NA
-0.865232488	0,386911197245455	NA
0.630075391	0,528645259422232	NA
0.506556935	0,612465731356863	NA
0.540353296	0,588953410213888	NA
0.126583642	0,899269944298868	NA
0.484133194	0,62829134474473	NA
1.942752888	0,0520460226286721	NA
0.528473326	0,597170855630965	NA
0.218221685	0,827256389871725	NA
0.988247599	0,323031401769582	NA
0.839007767	0,401464951260729	NA
0.346474153	0,728986405711561	NA
0.327349964	0,743403206292847	NA
0.754839936	0,450345028369401	NA
0.769556828	0,441562822690055	NA
-0.746343608	0,455459875275907	NA
0.024980425	0,980070577701164	NA
-0.421411754	0,673454437427102	NA
1.042116804	0,297357528879624	NA
0.296321455	0,766984602549589	NA
1.869877586	0,0615008190223962	NA
-1.21840086	0,223071678592417	NA
1.260681146	0,2074237494244	NA
-0.295635148	0,767508728911836	NA
-0.421044551	0,673722549960087	NA
0.232599517	0,816072402270047	NA
0.081400442	0,935123497861783	NA
0.175056748	0,861035043383403	NA
-0.89695248	0,369744274427611	NA
0.491224997	0,623267320794872	NA
0.722056808	0,470259552044807	NA
-0.501063359	0,616326533036796	NA
0.23631023	0,813191949505967	NA

1.130158347	0,258409507695575	NA
-2.285982412	0,02225528751259	NA
-0.557338269	0,577296333333046	NA
0.499708526	0,617280328494169	NA
-0.598663968	0,549396989935586	NA
1.038391737	0,299087715057191	NA
-0.49149532	0,623076161488892	NA
-0.748839067	0,453954209894284	NA
-1.603760815	0,108766785059769	NA
1.680593	0,0928419964934568	NA
-1.479058997	0,139124547518539	NA
0.199749734	0,841676315756609	NA
1.776331911	0,0756782264463083	NA
2.014980077	0,0439067366172876	NA
-0.090825393	0,927631332792927	NA
-0.964017735	0,335037034327855	NA
-0.400376688	0,688879091765927	NA
-0.063799046	0,949130238163447	NA
-0.466829241	0,640622053654361	NA
1.937511447	0,0526828554729417	NA
-0.694057772	0,487645979612386	NA
1.079340539	0,280435947256328	NA
-0.892043178	0,372369789442628	NA
-0.179858152	0,857263929532356	NA
0.470817765	0,637770876859393	NA
0.625893344	0,531384900672925	NA
-0.646377131	0,518035147389738	NA
0.007418691	0,994080795195404	NA
0.677076311	0,498357538356686	NA
1.248350697	0,211902656034984	NA
-1.304863942	0,191939181425629	NA
-0.296104618	0,76715018770026	NA
0.357395848	0,720795484295421	NA
1.806461591	0,0708462684872612	NA
0.729909081	0,465445761126641	NA
-0.610309914	0,541656530639621	NA
-0.3669617	0,713647587307735	NA
0.402309367	0,687456361591986	NA
-0.219716506	0,826091949078213	NA
1.215799749	0,224061214272771	NA
0.889559147	0,373702650795034	NA
1.039305756	0,298662558900289	NA
1.382428031	0,166840314084571	NA
0.866887982	0,386003389031372	NA
1.164647753	0,244161605903736	NA
0.712928931	0,475889735594728	NA
-1.18369551	0,236533616823659	NA
0.116850785	0,906978298486245	NA
1.486157349	0,137237506138651	NA
1.590450444	0,111733307528304	NA

-0.142734551	0,886499828332235	NA
0.398971947	0,689913874724243	NA
0.946442141	0,343923119940154	NA
1.089486142	0,27593956269703	NA
0.112442365	0,910472665583386	NA
-0.283658446	0,776672142091825	NA
1.098798926	0,271855781000159	NA
-1.400947326	0,161229824972471	NA
-0.305641856	0,759877347899812	NA
-0.041718189	0,966723353464973	NA
-1.412930967	0,157676022874695	NA
2.334637055	0,0195623890821703	NA
0.659312759	0,509694950959484	NA
0.216589847	0,828527998484256	NA
0.304917912	0,760428672944999	NA
-0.270813019	0,786534845845338	NA
0.278938337	0,78029214665359	NA
-0.157122703	0,875148145216823	NA
0.187450076	0,851307763139245	NA
-0.003145018	0,997490642446478	NA
1.112949052	0,265730311193992	NA
-1.710310115	0,0872085422551552	NA
-0.377254067	0,705984803621538	NA
-0.385186511	0,700099236225505	NA
0.474516053	0,635131953291053	NA
0.002134165	0,998297183677155	NA
1.231547752	0,218118068366039	NA
-0.14349563	0,88589876195332	NA
-0.112333846	0,910558706334698	NA
2.348165972	0,0188661124764634	NA
-0.031669332	0,974735752080671	NA
0.027244311	0,978264873876383	NA
0.639781365	0,522314749432426	NA
0.694035715	0,487659811261155	NA
-0.039025012	0,968870446778034	NA
1.919842207	0,0548778337438792	NA
0.242918749	0,808068340905751	NA
0.380871152	0,703298859015469	NA
-0.176345424	0,860022577943637	NA
0.323213847	0,746533295003714	NA
0.03547177	0,971703556496298	NA
-0.808658535	0,418711585328844	NA
-1.483484583	0,137945709658338	NA
0.627922544	0,530054681715314	NA
1.030767325	0,302649944321466	NA
2.080667332	0,0374643661778512	NA
-0.414575731	0,678452517148332	NA
0.178343982	0,858452839967744	NA
0.223518112	0,823132288019073	NA
0.718898415	0,472203513418569	NA

0.010459098	0,991654999051635	NA
1.087650496	0,276749429611382	NA
-0.481424944	0,630214511646074	NA
0.740417337	0,459046802111361	NA
-0.40532293	0,685240153408641	NA
0.68077079	0,496016535299779	NA
-0.583666859	0,559444468616083	NA
0.911054177	0,362266828773031	NA
1.111454246	0,266372873725489	NA
1.164715918	0,24413400332043	NA
1.826497558	0,0677753487559251	NA
0.359303355	0,719368164483275	NA
0.436788252	0,662264920055502	NA
0.33342329	0,738814785219784	NA
-0.831337318	0,405783098590858	NA
-1.187194071	0,235151079074049	NA
-0.289247926	0,772391658523465	NA
1.95386024	0,0507177477758562	NA
0.836299587	0,402986385266343	NA
-2.049986319	NA	NA
0.217522701	0,827801017568607	NA
-1.395054038	0,162999544893405	NA
0.710683387	0,477280456764196	NA
1.419536868	0,155742556309328	NA
-1.896984486	0,0578299857449051	NA
0.598884776	0,549249724140695	NA
1.617934321	0,10567675291367	NA
-0.933634378	0,350492525527825	NA
0.507007709	0,612149409257136	NA
2.207821894	0,0272566912453186	NA
1.776418619	0,0756639445452213	NA
0.718519415	0,472437080977356	NA
-1.484821943	0,137590997665463	NA
-2.804885587	0,00503344432726567	NA
0.000175436	0,999860022235448	NA
1.002762148	0,315975635857286	NA
1.048309762	0,294495913641683	NA
-0.283610088	0,776709205655435	NA
-0.503537968	0,614586093218746	NA
0.329869454	0,74149860507893	NA
-0.134653355	0,892885954415335	NA
0.667793893	0,504265165519166	NA
0.805965939	0,420262489088958	NA
0.224042975	0,822723863937555	NA
-0.819317279	0,412605416989307	NA
-0.500438595	0,616766283410308	NA
0.863927058	0,387627961630984	NA
1.13723858	0,255438562840056	NA
0.389872613	0,696630746546433	NA
1.199126013	0,230478950690856	NA

2.174521492	0,0296659931126732	NA
-1.214507171	0,224554113309926	NA
0.266096332	0,790165019799214	NA
0.920556943	0,357281790600702	NA
-0.213676258	0,830799526925535	NA
-1.368968501	0,171009121210063	NA
1.955073205	0,0505744303478172	NA
0.682448724	0,494955255116324	NA
1.498428595	0,134021931776173	NA
0.839939466	0,400942327785727	NA
0.236728073	0,812867754859928	NA
0.411408163	0,680773269388906	NA
0.273403716	0,78454289649624	NA
-1.224741515	0,220672626795308	NA
-1.20329872	0,22886074422675	NA
-0.737983035	0,460524758672089	NA
-0.32844745	0,742573371282154	NA
-0.509568024	0,610354130806871	NA
0.508550716	0,611067181312704	NA
-0.613129147	0,539790949187462	NA
-0.698196208	0,485054497607778	NA
0.4434548	0,657436805624808	NA
1.292929963	0,196035283465637	NA
1.22068696	0,222204567056231	NA
0.418561043	0,675536965964461	NA
1.298126529	0,194243859438189	NA
0.756389523	0,449415687588941	NA
0.910660877	0,362474083620079	NA
0.955576637	0,339286169630003	NA
-1.816270742	0,0693288509915053	NA
1.017114392	0,309099014557573	NA
0.968254498	0,33291728348497	NA
0.160945727	0,872136146926676	NA
-0.202958418	0,839167533200271	NA
0.963820143	0,335136105835135	NA
0.1828825	0,854890214352326	NA
0.441547888	0,658816400778861	NA
1.411353907	0,158140286701472	NA
1.750780422	0,0799837401726456	NA
-1.371899949	0,170094586290394	NA
2.149100938	0,031626399454845	NA
1.050106659	0,293669077406042	NA
1.374541667	0,169273583936984	NA
0.193885008	0,846265922902013	NA
0.14758205	0,882672621877102	NA
-0.673984651	0,500321072375643	NA
-1.361611414	0,173320551087908	NA
1.153691715	0,248626581939871	NA
0.18325552	0,85459753327229	NA
0.099675541	0,920601919146496	NA

-0.475378369	0,634517309197938	NA
0.343631269	0,731123606170411	NA
-0.02536962	0,979760142850108	NA
1.369378172	0,170881093708491	NA
0.263847251	0,791897623017093	NA
-0.568738055	0,56953391845469	NA
0.453331932	0,650309742354526	NA
-0.527195076	0,598058127671306	NA
0.004595009	0,996333726380312	NA
0.350120895	0,726247969854361	NA
0.018339482	0,985368030572648	NA
0.173364436	0,862364977711266	NA
0.199931633	0,841534050622662	NA
-0.198049206	0,843006565257204	NA
-1.374353983	0,169331814877397	NA
0.80400641	0,421393272970387	NA
-0.436863833	0,662210102541753	NA
-1.396694094	0,162505580975402	NA
1.491126582	0,135928264650503	NA
-0.968656004	0,332716850545035	NA
1.559241911	0,118939134661593	NA
0.248702665	0,803590787254008	NA
1.772341538	0,076337879991602	NA
0.888732809	0,374146694208338	NA
1.706114516	0,0879867603674905	NA
-0.254970927	0,79874555386217	NA
0.422739728	0,672485166483058	NA
0.325896482	0,744502677489542	NA
-0.545056674	0,585714539493043	NA
-0.225171548	0,821845822900597	NA
-0.357706	0,720563342155852	NA
0.862372534	0,388482549857814	NA
1.081577328	0,279440383324913	NA
-0.292703415	0,769748859070727	NA
2.2441262	0,0248242826972817	NA
0.26574008	0,790439393222071	NA
-0.003978753	0,996825422615547	NA
1.370828564	0,170428404516569	NA
-2.147140813	0,0317820729737944	NA
0.719531781	0,471813327780285	NA
-0.200963304	0,840727267506225	NA
-1.227590277	0,219600813711329	NA
0.268661245	0,788190377805131	NA
0.329456068	0,741810994583865	NA
-1.190956996	0,233670467631551	NA
0.218216694	0,82726027830898	NA
-1.04722815	0,294994364959754	NA
0.275950487	0,782586095899173	NA
-1.517310624	0,129188277052661	NA
1.255708043	0,209221842018168	NA

-1.226899003	0,21986055341642	NA
1.1455134	0,251996547609478	NA
-1.354403799	0,175607586130975	NA
0.224670254	0,822235806799547	NA
-0.365968783	0,714388370006193	NA
-0.163714516	0,869955889994429	NA
0.613914454	0,539271858472291	NA
-1.282394769	0,199704198315136	NA
-0.038080404	0,969623575693858	NA
1.254339176	0,209718750143181	NA
-1.379648377	0,167694934499039	NA
-0.167553301	0,86693471246023	NA
0.193727549	0,846389219356352	NA
-0.038111948	0,969598424868597	NA
-1.248557589	0,211826932459732	NA
0.250743975	0,80201205898547	NA
-0.086820305	0,930814347360983	NA
2.589384329	0,00961477185998596	NA
-0.708956205	0,478351654999544	NA
0.057988266	0,95375797516233	NA
-0.013937388	0,988879933671645	NA
1.294990198	0,195323610884911	NA
-1.593449232	0,111059448847804	NA
0.841883455	0,399853192407275	NA
0.771349463	0,440499821287827	NA
-0.293151944	0,769406014235089	NA
0.138969012	0,889474636543901	NA
0.445660019	0,65584284882675	NA
0.787474671	0,431004050057589	NA
0.471816267	0,637057938915632	NA
1.163645548	0,244567684821638	NA
-0.022421827	0,98211146933968	NA
1.345636347	0,178419823843939	NA
0.420802353	0,673899413474551	NA
0.300806366	0,763562153469673	NA
-0.149662847	0,881030621809462	NA
-1.958062132	0,0502227225704742	NA
-0.847460931	0,396738254491716	NA
-0.799147232	0,424205045305904	NA
0.954330401	0,33991642393962	NA
0.434507666	0,663919829544406	NA
-0.30628309	0,759389113181136	NA
-0.182982972	0,854811379362036	NA
0.273185334	0,784710753443085	NA
-1.121509389	0,262071097259814	NA
0.572454279	0,567014252517928	NA
-0.831565913	0,40565400924557	NA
1.209325752	0,226537720913678	NA
0.714893986	0,47467455370692	NA
0.788934112	0,43015051729177	NA

-2.331911906	0,0197053260256367	NA
0.266284295	0,790020267183161	NA
0.560441504	0,575178329026627	NA
0.308340727	0,757823079295894	NA
-0.257686893	0,796648562203333	NA
0.806850177	0,419752806470524	NA
-0.264836753	0,791135222867355	NA
-0.763141699	0,445378893960326	NA
-0.454882899	0,649193486739753	NA
-0.034634265	0,972371378399535	NA
0.709114446	0,478253459322904	NA
0.858150994	0,390809092155459	NA
-0.337855812	0,73547184850938	NA
0.588582487	0,556141384710278	NA
-0.513224426	0,607794341349766	NA
0.693377314	0,488072795458155	NA
0.345673957	0,729587758582658	NA
-1.095376399	0,273351769156882	NA
0.029805116	0,97622247853602	NA
0.082219833	0,934471902744137	NA
-0.284799706	0,775797597488196	NA
0.597328006	0,550288409846729	NA
0.273156976	0,784732550778014	NA
0.461906771	0,644148178241364	NA
0.376878096	0,706264199773841	NA
-0.009242468	0,992625682527332	NA
0.560242591	0,575313979387804	NA
-1.959141483	0,0500962198633121	NA
0.092708041	0,926135508500858	NA
-0.809735418	0,418092257079803	NA
0.875881149	0,381094648323752	NA
-0.219424504	0,826319383622168	NA
-0.076815923	0,93876998314043	NA
-0.310567909	0,756129126333029	NA
0.357099063	0,721017646284942	NA
0.708488063	0,478642222536378	NA
0.030824478	0,97540951932631	NA
0.404107811	0,686133441531325	NA
-1.400186422	0,161457500653262	NA
-1.390859402	0,16426806571348	NA
-0.935023547	0,349776164467426	NA
1.570320188	0,116340639841242	NA
1.585285763	0,112901422345995	NA
2.186802042	0,0287569783517665	NA
0.142358803	0,886796601267251	NA
-0.551304336	0,581425066614355	NA
0.266909576	0,789538783646467	NA
-0.368967606	0,712151870824464	NA
1.173974249	0,240405340351625	NA
-0.680724715	0,496045694819428	NA

-0.679577016	0,496772327154654	NA
0.435088796	0,66349797588253	NA
0.821872162	0,411149658220771	NA
-0.035175509	0,971939790748593	NA
-0.13767117	0,890500305426312	NA
-0.979406157	0,327379336040794	NA
0.106708901	0,915019921122113	NA
0.377857119	0,705536739132123	NA
-1.536734926	0,124358234575369	NA
-2.028351086	0,0425244285759059	NA
-1.299942548	0,193620660800861	NA
0.499213078	0,617629282246414	NA
-0.665817119	0,505528001179393	NA
-0.542343507	0,587581889382934	NA
0.58414581	0,559122216398745	NA
-0.208342307	0,834961700278466	NA
-0.256706305	0,797405503714989	NA
0.679173658	0,497027835971229	NA
-0.810174941	0,417839637818453	NA
-0.19956133	0,841823673261292	NA
1.264648968	0,205997191044103	NA
-0.289938047	0,771863633625151	NA
0.089681838	0,928540048658768	NA
-0.506342746	0,612616059959818	NA
0.010544888	0,991586552863211	NA
-1.623430198	0,104497473604517	NA
1.47200792	0,141018738995021	NA
1.033358332	0,301436238503143	NA
-1.012244217	0,311421299340711	NA
0.463679047	0,642877711077433	NA
-1.02555747	0,305100222985656	NA
0.59751619	0,550162801055121	NA
-0.916978213	0,35915405675692	NA
1.033866186	0,301198724613465	NA
-0.40717944	0,683876200785668	NA
0.16361915	0,870030968021218	NA
0.961080333	0,336511777386533	NA
2.075316528	0,0379572086113202	NA
0.106521794	0,915168364299194	NA
-1.959488567	0,0500555975720956	NA
1.72819764	0,0839527966485033	NA
1.202924167	0,229005667453671	NA
0.075790279	0,939585949845153	NA
0.161478642	0,871716432006295	NA
0.160785632	0,872262242369086	NA
0.564797558	0,572211468271365	NA
