# Supplementary material for: Transcriptomic analysis reveals mitochondrial pathways associated with distinct adolescent behavioral phenotypes and stress response
Source: Transl Psychiatry. 2023 Nov 17;13:351. doi: 10.1038/s41398-023-02648-3 (PMC10656500; doi:10.1038/s41398-023-02648-3)
Supplement: Supplementary file 1 — Supplemental Figures [file 41398_2023_2648_MOESM1_ESM.pdf]

Supplementary material

Transcriptomic analysis reveals mitochondrial pathways associated with distinct adolescent behavioral phenotypes and stress response

Thamyris Santos-Silva, Doğukan Hazar Ülgen, Caio Fábio Baeta Lopes, Francisco Silveira Guimarães,Luciane Carla Alberici, Carmen Sandi, Felipe Villela Gomes

RESULTS

Revealing individual behavioral variability

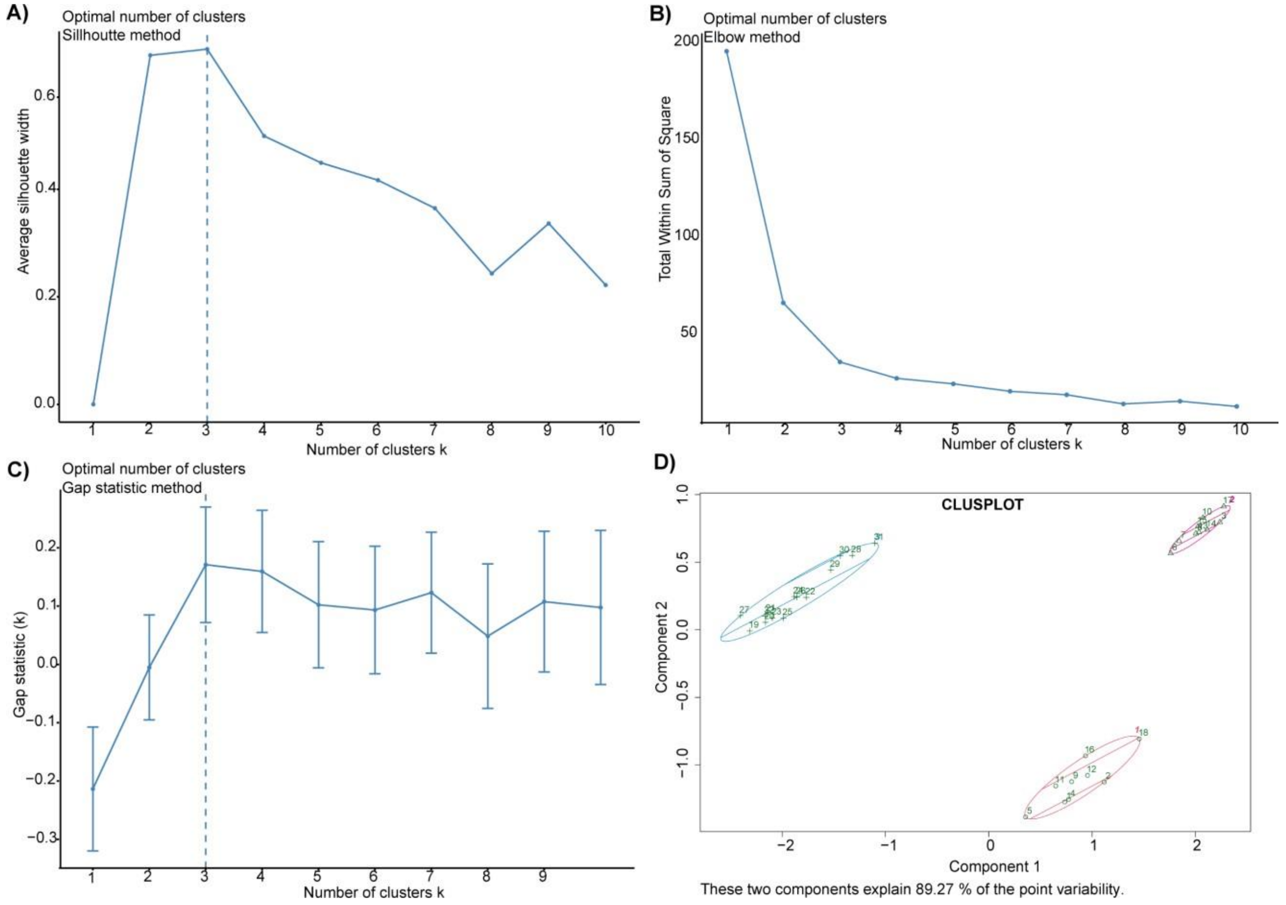

Supplementary Figure 1. Optimal number of clusters. The optimal number of clusters (3) was determined by Silhouette (A), Elbow (B) and Gap statistic methods (B) before applying the k-means clustering algorithm in the z-normalized behavioral data set (D).

RESULTS

Transcriptomic analysis of the PFC reveals changes in mitochondrial pathways

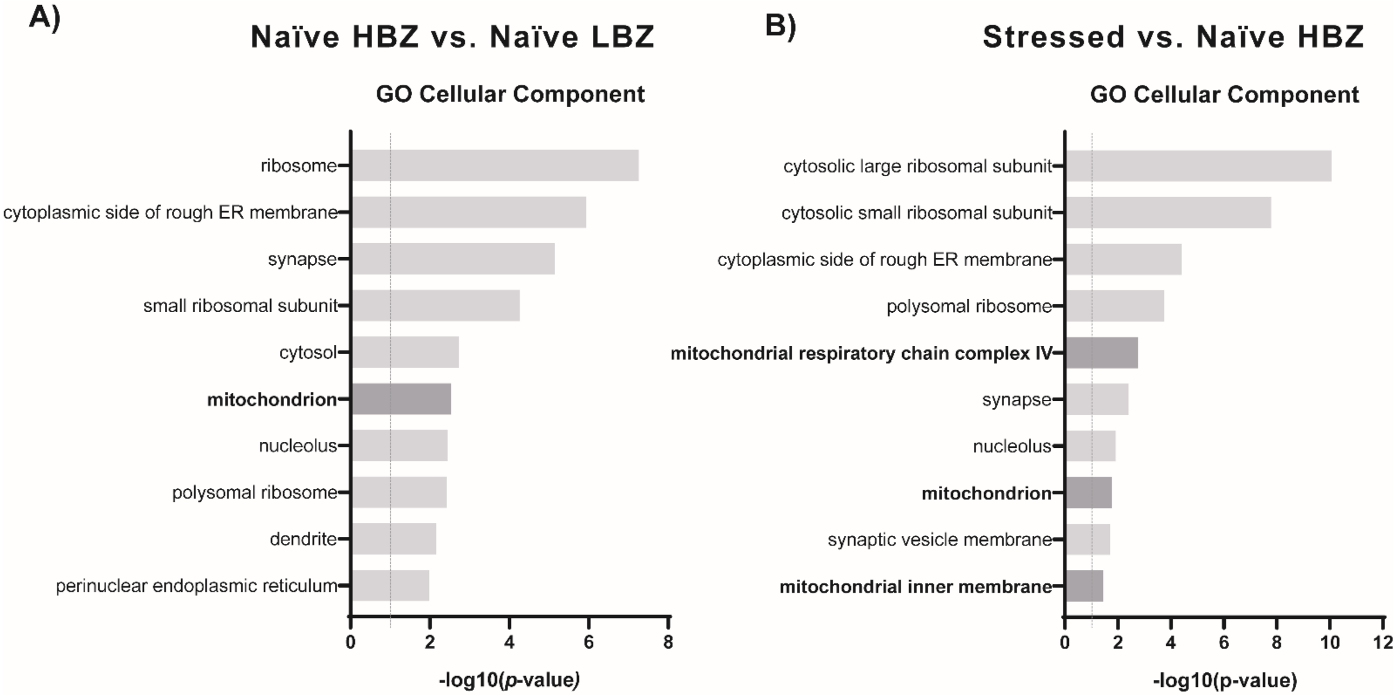

Supplementary Figure 2. Gene ontology – cellular component classes. Gene set enrichment analysis of DEGs in the PFC of naïve LBZ and **(A)** stressed animals **(B)** compared to naïve HBZ. Mitochondrial pathways are highlighted in black font.

RESULTS

Expression profiles of mitochondria-associated genes in the PFC relate to behavioral phenotypes

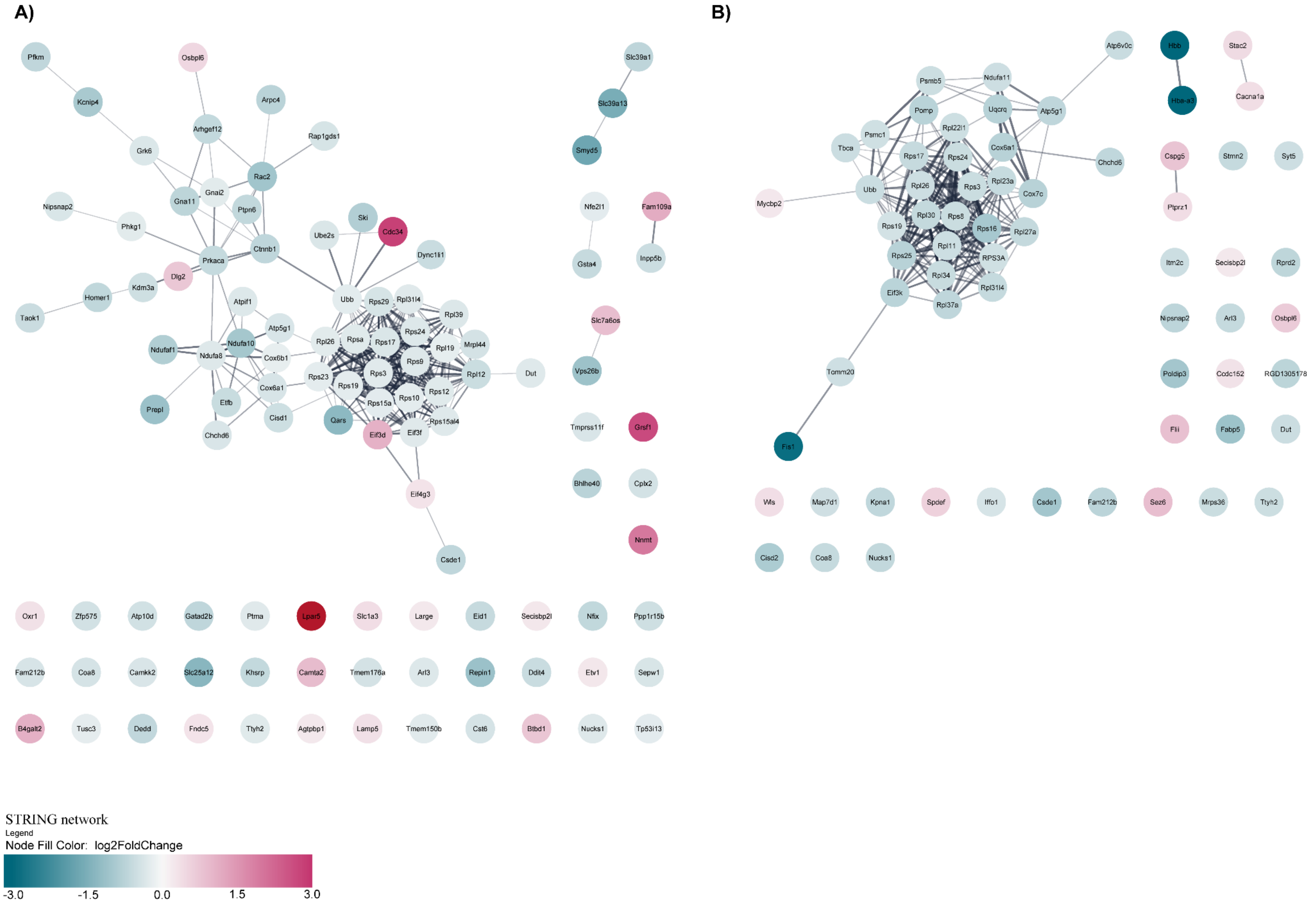

Supplementary Figure 3. Gene-gene interactive networks and non-connected DEGs. DEGs were mapped onto interaction networks using Cytoscape software for Naïve LBZ **(A)** and stressed animals **(B).** Nodes corresponding to DEGs, and edges equivalent to predicted functional and/or physical interactions with a 0.4 confidence score cut-off. The nodes colors represent the corresponding genes' relative expression (log2(Fold Change)).
